# Supplementary material for: LinkPred: a high performance library for link prediction in complex networks
Source: PeerJ Comput Sci. 2021 May 21;7:e521. doi: 10.7717/peerj-cs.521 (PMC8157017; doi:10.7717/peerj-cs.521)
Supplement: Supplemental Information 1 [file peerj-cs-07-521-s001.zip › LinkPred/UserGuide.pdf]

LinkPred

A High Performance Library for Link Prediction in Complex Networks

Said Kerrache

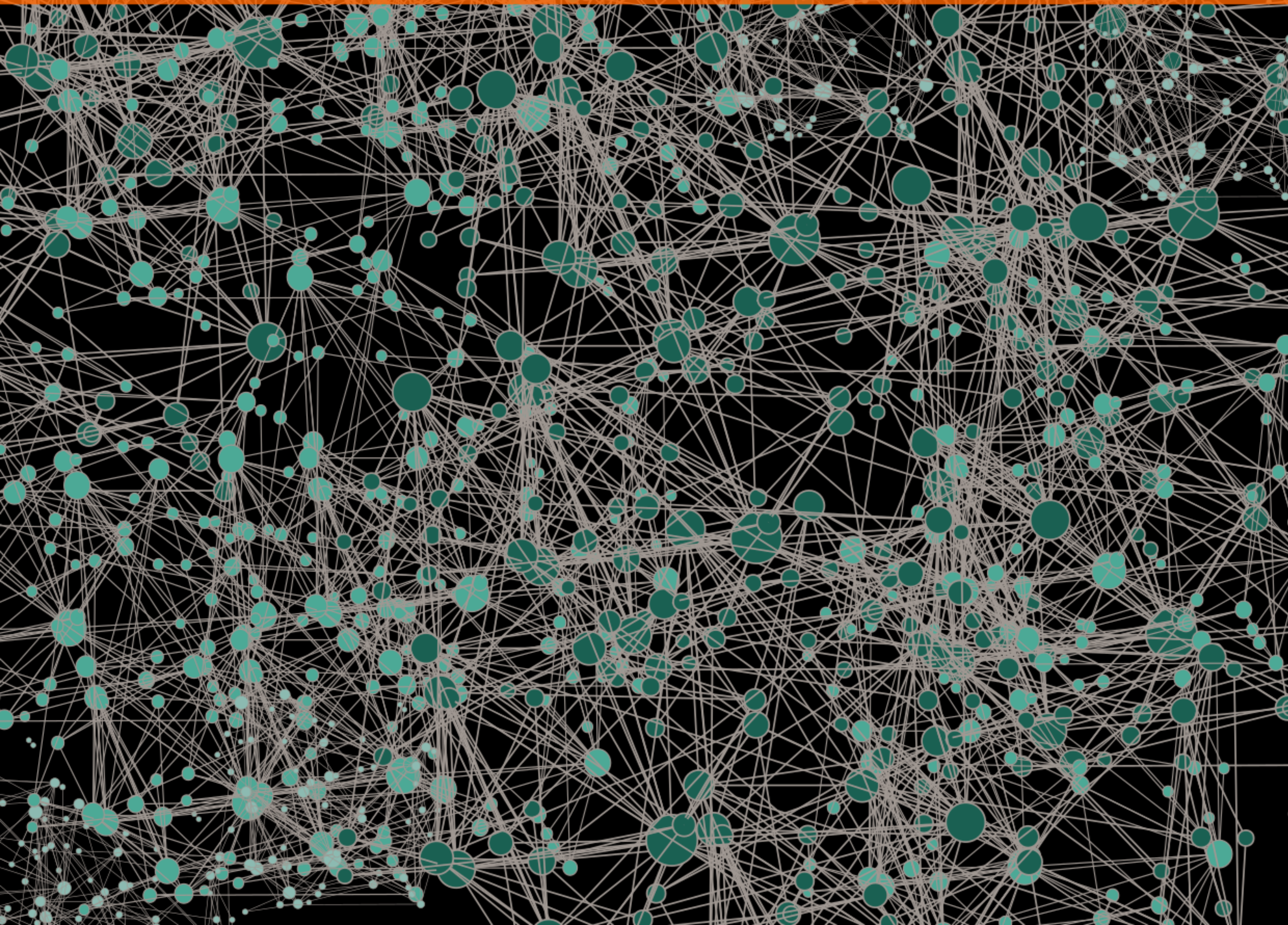

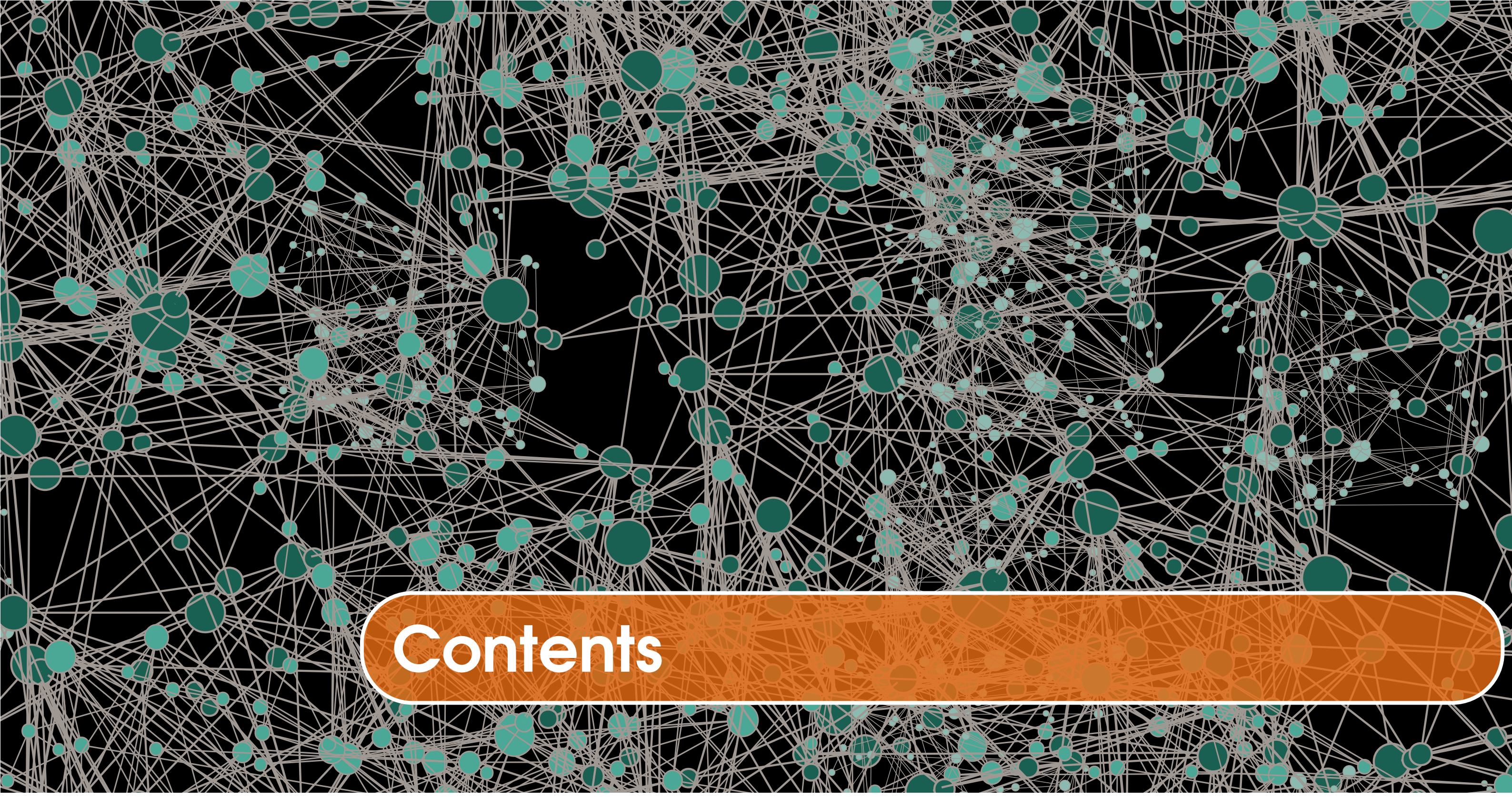

Contents

1 Introduction 7

1.1 Design principles 7

1.2 Functionalities 8

1.3 Requirements 8

1.4 Installation 9

1.5 Sample Programs 9

1.6 Third-party software 11

1.7 Documentation 12

1.8 Data 12

1.9 Citation 13

2 Quick Start 15

2.1 C++ 15

2.1.1 Predicting links using the class `Simp::Predictor` 16

2.1.2 Evaluating performance using the class `Simp::Evaluator` 18

2.2 Java Bindings 22

2.2.1 Predicting links using the class `Predictor` 22

2.2.2 Evaluating performance using the class `Evaluator` 25

2.3 Python Bindings 29

2.3.1 Predicting links using the class `Predictor` 30

2.3.2 Evaluating performance using the class `Evaluator` 31

3 Core Components 37

3.1 The undirected network data structure **37**

3.1.1 Building the network 38

3.1.2 Accessing nodes 41

3.1.3 Accessing edges 42

3.2 The directed network data structure **44**

3.3 Maps **46**

3.3.1 Sparse maps 48

4 Graph Algorithms 51

4.1 Graph traversal **51**

4.2 Shortest paths **54**

4.2.1 Memory management 56

4.2.2 Approximate shortest path distances 58

4.3 Graph embedding **60**

4.3.1 The Encoder interface 61

4.3.2 Examples 61

5 Machine Learning Algorithms 65

6 Predictors 71

6.1 The predictor interface **71**

6.2 Link predictors for undirected networks **72**

6.2.1 Topological-ranking methods 73

6.2.2 Global predictors 77

6.2.3 Network embedding methods 81

6.2.4 Utility predictors 83

6.3 Link predictors for directed networks **85**

6.4 Implementing a new link prediction algorithm **87**

7 Performance Evaluation 89

7.1 Data setup **89**

7.1.1 Creating test data by removing edges 90

7.1.2 Creating test data by adding edges 91

7.1.3 Creating test data by adding and removing edges 92

7.1.4 Loading test data from file 94

7.1.5 Creating test data from two snapshots of an evolving network 101

7.2 Prediction results **109**

7.3 Performance measures **109**

7.3.1 Receiver operating characteristic curve (ROC) 110
7.3.2 Precision-recall curve 112
7.3.3 General performance curves 113
7.3.4 Top precision 116

7.4 Performance evaluation classes **117**

7.4.1 The class `PerfEvalExp` 117
7.4.2 The class `PerfEvaluator` 121

8 Parallelism, Templates and Library Extension 125

8.1 Parallelism **125**

8.1.1 Shared memory parallelism 125
8.1.2 Distributed parallelism 127

8.2 Templates **129**

8.3 Extending LinkPred **130**

Bibliography 133

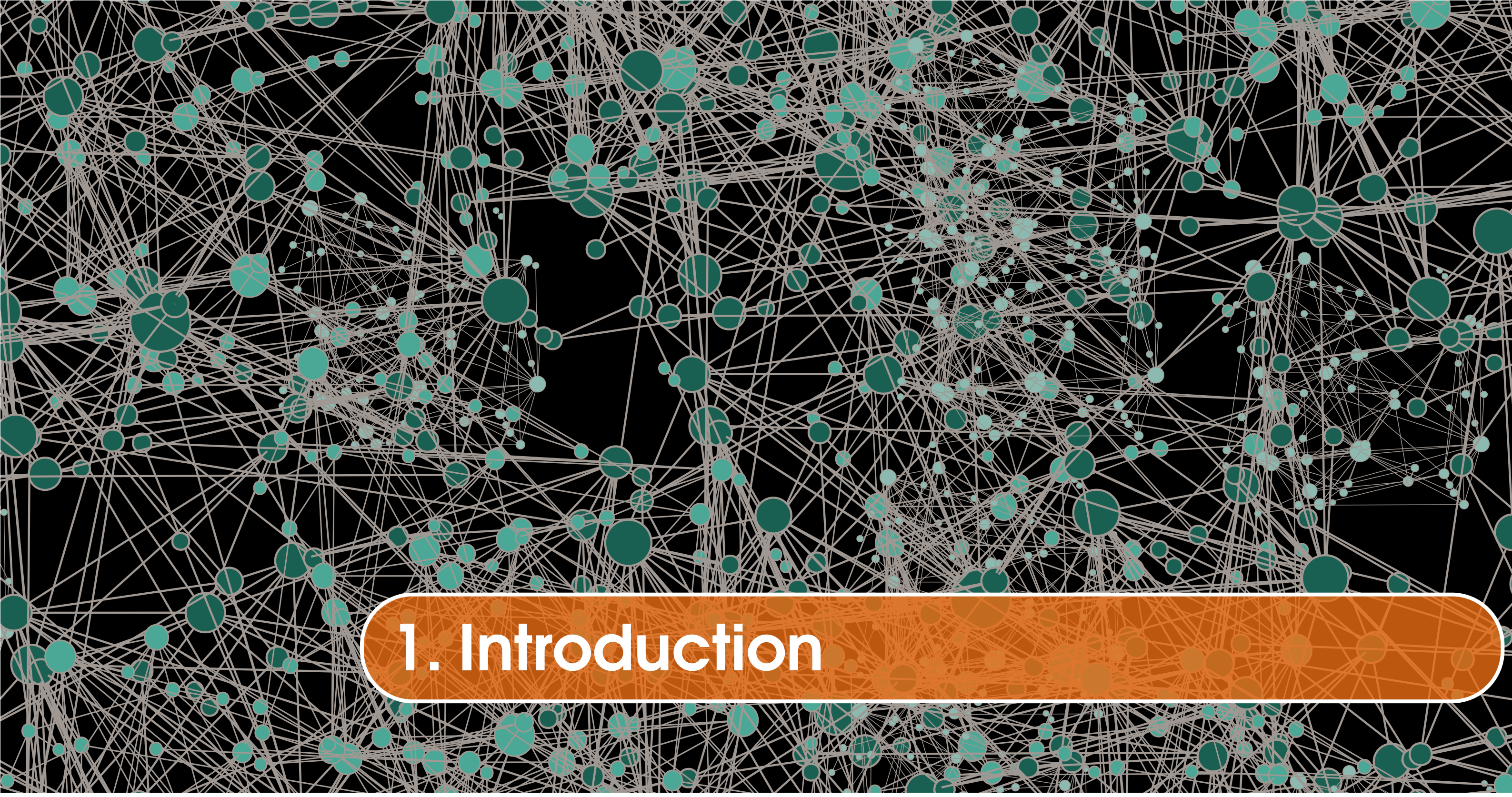

1. Introduction

The problem of determining the likelihood of existence of a link between two nodes in a network is called *link prediction*. Such prediction is made possible thanks to the existence of a topological structure in most real life networks. In other words, the topologies of networked systems such as the World Wide Web, the Internet, metabolic networks and human society are far from random, which implies that partial observations of these networks can be used to infer information about undiscovered interactions.

Significant research efforts have been invested into the development of link prediction algorithms, and some researchers have made the implementation of their methods available to the research community. However, these implementations are often written in different languages and use different modalities of interaction with the user, which hinders their effective use. LinkPred is a high performance parallel and distributed link prediction library that includes the implementation of the major link prediction algorithms available in the literature by development from scratch and wrapping or translating existing implementations. The library offers a unified interface that facilitates the use and comparison of link prediction algorithms by researchers as well as practitioners.

1.1 Design principles

LinkPred is designed with the following guiding principles:

- Ease of use: LinkPred borrows heavily from the STL design and aims at offering an elegant and powerful interface. C++ users with minimum experience using STL will find the interface of LinkPred to be very familiar. Furthermore, the use of templates allows for greater flexibility when using LinkPred and allows for its integration within a variety of contexts.
- Extensibility: LinkPred was not only designed for practitioners of link prediction, but also fo researchers in the field. The library is designed in a way that allows developers of new link prediction algorithms to easily integrate their code into the library and take advantage of the existing functionalities such network data structures and performance evaluation algorithms.

- Efficiency: the data structures used and implemented in LinkPred are all chosen and designed to achieve the best possible performance. Additionally, most code in LinkPred is parallelized using OpenMp, which allows to take advantage of shared memory architectures. Furthermore, a significant portion of the predictors support distributed processing using MPI allowing the library to handle very large networks (hundreds of thousands to millions of nodes).

1.2 Functionalities

LinkPred provides the following functionalities:

- Basic data structures to efficiently store and access network data.
- Basic graph algorithms such graph traversal, shortest path algorithms, and graph embedding methods.
- Implementation of several topological similarity index predictors, for example: common neighbors, Adamic-Adard index and Jackard index among other predictors (a full list is available in the library documentation).
- Implementation of several state-of-the-art link predictors, such as SBM, HRG, FBM and KAB (a full list is available in the library documentation).
- Implementation of several link prediction algorithms based on graph embedding techniques.
- Test data generation from ground truth networks.
- Performance evaluation functionalities.

1.3 Requirements

The following softwares are used by LinkPred:

- A C++14 compliant compiler (required). Note that strict compliance with the standard C++14 is enforced during compilation and that C+11 compliance is not enough to build the library.
- The GNU Scientific Library (GSL) (required). LinkPred was tested with the version 2.1, but earlier versions might work as well.
- OpenMP (optional, default on): LinkPred works with OpenMP 3.0 or higher as it uses loop parallelization for STL iterators.

> R Unfortunately, Visual C++ only supports OpenMp 2.0. LinkPred can still be compiled by disabling OpenMP, bu parallelism cannot be used when compiling with Visual C++.

- MPI (optional, default on): To take advantage of distributed architectures, several predictors as well as performance evaluation routines can run distributively using MPI. Although optional, it is strongly recommended.
- mlpack (optional, default on): contains machine learning related classes used in graph embedding prediction methods.
- Intel Math Kernel Library (MKL) (optional, default off): LinkPred was tested with the version 2016, but earlier versions might work as well. The MKL library is used by some prediction algorithms that incorporate linear algebra calculations. This library is nonetheless optional, since LinkPred offers replacements of the

required methods. The replacement code is, however, a naive one and may result in significant loss of performance in the said algorithms.

1.4 Installation

LinkPred is distributed as source code that can be used to build the library using CMake. In the default setting, the building process is as follows:

1. Create a build directory in the root of the LinkPred directory:

```
$ mkdir build
```

2. Configure the library:

```
$ cd build
$ cmake ../
```

> **R** Build options can be set by editing the file `CMakeLists.txt` or through the user interface if GUI CMake is used.

> **R** Building the Python and Java bindings requires a recent version of CMake. If you do not need these bindings and intend to only work with C++, you may ignore the warning messages generated by CMake. If you intend to use Python and Java bindings, you should upgrade CMake to at least 3.12.

3. Build the library:

```
$ make
```

4. Build documentation (optional): this step requires Doxygen and generates documentation in HTML and Latex:

```
$ make doc
```

5. If you want to install the library:

```
$ make install
```

To install the library system-wide, you may need root privilege:

```
$ sudo make install
```

If you prefer a local install instead (which is usually the case when working on institution-wide HPC clusters/supercomputers), you need to set the install directory in the configuration step (Step 2 above):

```
$ cmake -DCMAKE_INSTALL_PREFIX=YOUR_PATH ../
```

The `examples` directory contains sample code that can be used as a start point for using the library.

1.5 Sample Programs

The following example shows how to use the Common Neighbors link predictor to predict links in a network that is loaded from file. The network file must have the following format (one edge per line):

```
1       2
2       4
2       8
2       14
3       2
3       4
3       8
3       9
```

Here, we consider two scenarios: In the first one, we would like to compute the score for all non-existing links, whereas in the second we want to find out the *k* top links (those more likely to be missing).

To compute the score of all non-existing links, proceed as follows. In a file named `cne.cpp`, type the following code[1]:

Listing 1.1: code/introduction/cne.cpp

```cpp
#include <linkpred.hpp>
#include <iostream>
using namespace LinkPred;

int main() {
  auto net = UNetwork<>::read("Infectious.edges");
  UCNEPredictor<> predictor(net);
  predictor.init();
  predictor.learn();
  std::cout << "#Start\tEnd\tScore\n";
  for (auto it=net->nonEdgesBegin();it!=net->nonEdgesEnd();++it){
    auto i = net->getLabel(net->start(*it));
    auto j = net->getLabel(net->end(*it));
    double sc = predictor.score(*it);
    std::cout << i << "\t" << j << "\t" << sc << std::endl;
  }
  return 0;
}
```

R In this code, the predictor is instantiated with default template parameters. You may use non-default parameters if needed.

Compile your code. For example, if you compiled LinkPred with MPI an OpenMP enabled:

```
$ mpiCC cne.cpp -o cne -fopenmp -lLinkPred
```

R If you face any dialect-related complaints from the compiler, you may need to add the option: `-std=c++14`. Also, depending on the LinkPred functionalities used in your code, you may need to additionally link against the MKL library (using `-lmkl_rt`) and/or gsl (using `-lgsl -lgslcblas`).

If you built LinkPred without MPI and OpenMP, compile as follows:

[1]This code is available in the examples directory.

```
$ g++ cne.cpp -o cne -lLinkPred
```

Run your code:

```
$ ./cne
```

R Make sure that the library is located in the load path. Under Linux, you may need to set the environment variable `LD_LIBRARY_PATH`. In the case of a default system-wide install, LinkPred will be installed to the default directory, which is already in the load path. You may, however, need to refresh the ld cache by running:

```
$ sudo ldconfig
```

To get the top k links, type the following code[2] in a file named `cnetop.cpp`:

Listing 1.2: code/introduction/cnetop.cpp

```
#include <linkpred.hpp>
#include <iostream>
using namespace LinkPred;

int main() {
  std::size_t k = 10;
  auto net = UNetwork<>::read("Infectious.edges");
  UCNEPredictor<> predictor(net);
  predictor.init();
  predictor.learn();
  std::vector<typename UNetwork<>::Edge> edges(k);
  std::vector<double> scores(k);
  k = predictor.top(k, edges.begin(), scores.begin());
  std::cout << "#Start\tEnd\tScore\n";
  for (std::size_t l = 0; l < k; l++) {
    auto i = net->getLabel(net->start(edges[l]));
    auto j = net->getLabel(net->end(edges[l]));
    std::cout << i << "\t" << j << "\t" << scores[l] <<std::endl;
  }
  return 0;
}
```

Compile the code and run it as previously shown.

1.6 Third-party software

LinkPred includes modified and/or translated versions of the following software sources:

- HRG code [7]: we used the implementation available at `http://tuvalu.santafe.edu/~aaronc/hierarchy/hrg_20120527_predictHRG_v1.0.4.zip`.
- SBM code [12]: we used the C code provided by the authors at `http://seeslab.info/media/filer_public/eb/ae/ebaee03f-a53a-430f-a4a1-6b713d36e91e/rgraph-2.0.1.tar.gz`.
- FBM code [20]: we translated the Matlab code provided by the authors into C++.

[2]This code is available in the examples directory.

- HyperMap (HYP) code [23, 24]: we used the code provided by the authors at `http://www.cut.ac.cy/eecei/staff/f.papadopoulos/?languageId=2`.
- CG_DESCENT: a conjugate gradient method with guaranteed descent[13].
- *plfit*: a C++ implementation of Clauset, Shalizi and Newman [8] method for fitting power law distributions written by Tamas Nepusz. The code is available at `http://tuvalu.santafe.edu/~aaronc/powerlaws/`.
- Implementation of DeepWalk graph embedding algorithm [25] available at `https://github.com/xgfs/deepwalk-c`. An adapted version of the code is included in LinkPred.
- Implementation of LINE (Large Information Networks Embedding) graph embedding algorithm [29] available at `https://github.com/tangjianpku/LINE`. An adapted version of the code is included in LinkPred.
- Implementation of LargeVis graph embedding algorithm [30] available at `https://github.com/lferry007/LargeVis`. An adapted version of the code is included in LinkPred.
- Implementation of Node2Vec graph embedding algorithm [11] available at `https://github.com/xgfs/node2vec-c`. An adapted version of the code is included in LinkPred.

1.7 Documentation

You may learn about LinkPred through:

- This user guide, which contains detailed description of the library components, code snippets and full working examples.
- The tutorials which are available in the directory `tutorials`. These contain fully working examples along with comments and compilation instructions.
- The library reference manual, available in html and PDF format in the directory `doc`.

1.8 Data

Two small networks are included with the library and can be used with the example programs:

- Zakaray's Karate Club[34] (file: `Zakarays_Karate_Club.edges`): A social network that represents friendships between members of a karate club at an American university. The data was collected in the 1970s by Wayne Zachary and is available at `http://konect.cc/networks/ucidata-zachary`.
- Infectious[14] (file: `Infectious.edges`): Face-to-face interaction between visitors of the exhibition INFECTIOUS: STAY AWAY in 2009 at the Science Gallery in Dublin. A link indicates that a face-to-face interaction took place for more than 20 seconds. The dataset is available at `http://konect.cc/networks/sociopatterns-infectious`.

More data can be found in the following public data repositories [3, 18, 19, 26, 28, 35, 36].

1.9 Citation

If you use LinkPred in your research, kindly cite the references of the algorithms you used and cite LinkPred as: Said Kerrache. “LinkPred: A High Performance Library for Link Prediction in Complex Networks”. In: Submitted (2019).

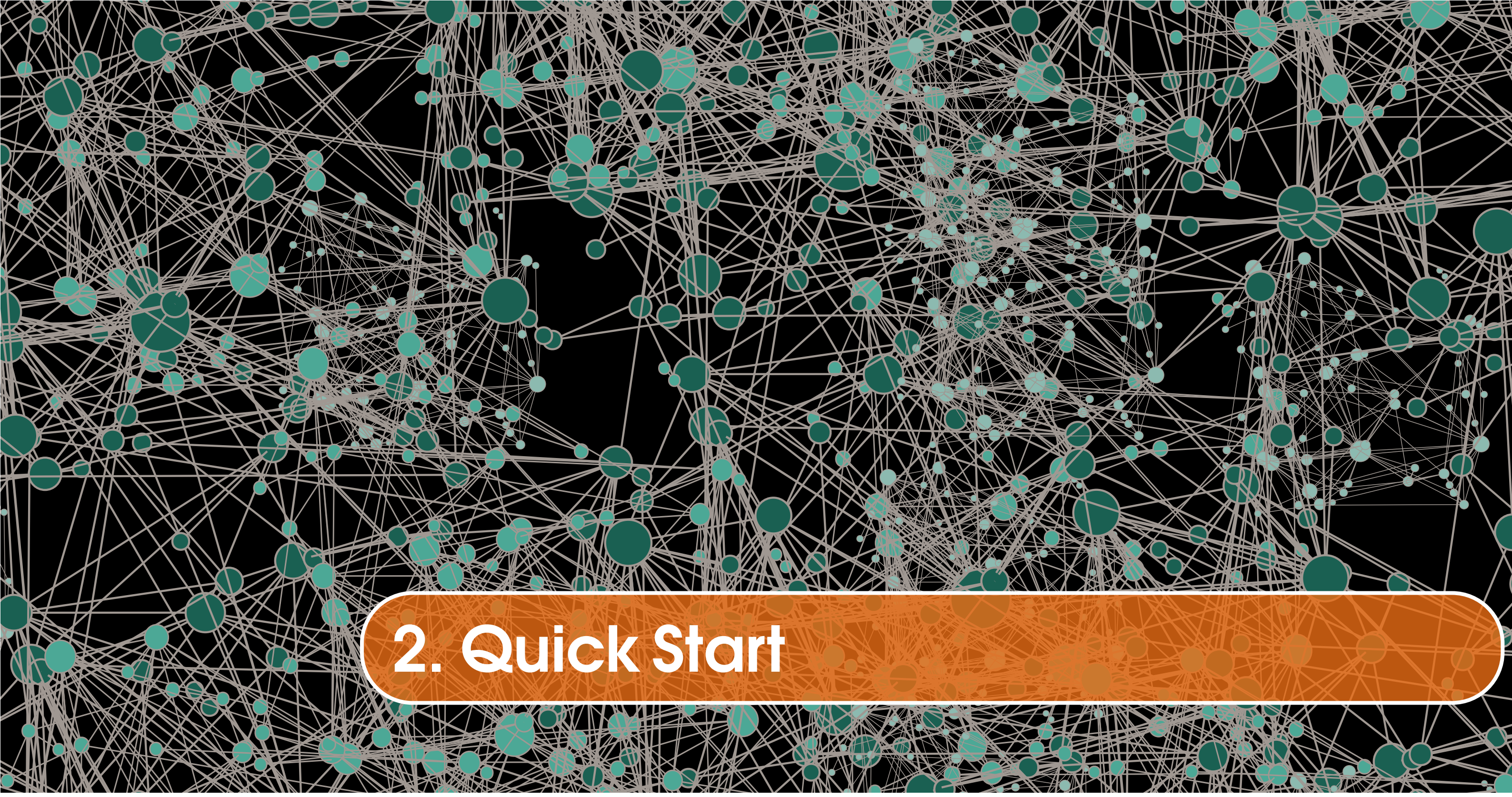

2. Quick Start

The easiest and fastest way to start using `linkPred` is by using the classes available under the namespace `LinkPred::Simp` (Simp here stands for "simple"). These classes are very intuitive and can be used with a minimum learning effort. They are ideal for initial use of the library and exploring its main functionalities. Java and Python bindings for these classes are also available, facilitating the use of the library by users who are more comfortable using these languages than C++. This chapter gives several examples of using this simplified interface in C++, Java, and Python.

> The simplified interface presented in this chapter is a good starting point to learn about `LinkPred`. To take full advantage of its performance and capabilities, however, users should use the programming interface presented in subsequent chapters.

2.1 C++

The namespace `LinkPred::Simp` contains the following classes and structures:

- The class `Predictor` allows computing the scores for an input network using all link prediction algorithms available in the library.
- The class `Evaluator` allows for the performance evaluation of link prediction algorithms.
- The structure `EdgeScore` is a simple structure used by the class `Predictor` to store the score of an edge.
- The structure `PerfRes` is a simple structure used by the class `Evaluator` to store the performance result of link prediction algorithms.

> Classes in the namespace `LinkPred::Simp` can be imported using:
>
> ```
> using namespace LinkPred::Simp;
> ```
>
> In the examples included in this chapter, we assume that this namespace is imported and drop the prefix `LinkPred::Simp::` from all classes for convenience.

2.1.1 Predicting links using the class `Simp::Predictor`

The following code shows how to compute the score of all non-existing links of a network using Adamic Adar index and print the result:

Listing 2.1: code/simp/predictor1.cpp

```
#include <linkpred.hpp>
#include <iostream>
using namespace LinkPred::Simp;
int main() {
  // Create a prtedictor object
  Predictor p;
  // Load network from file
  p.loadnet("Zakarays_Karate_Club.edges");
  // Predict the score of all non-existing edges using Adamic
     Adar index
  std::vector<EdgeScore> esv = p.predAllADA();
  // Print the scores
  for (auto it = esv.begin(); it != esv.end(); ++it) {
    std::cout << it->i << "\t" << it->j << "\t" << it->score <<
        std::endl;
  }
  return 0;
}
```

The class `Predictor` returns the results in an object of type `std::vector<EdgeScore>`, where each entry stores the score for a single edge as follows:

Listing 2.2: code/simp/edgescore.hpp

```
struct EdgeScore {
  std::string i; /**< The label of the start node. */
  std::string j; /**< The label of the end node. */
  double score; /**< The score. */
};
```

It is also possible to limit the prediction to specific edges which are passed as parameter as shown in the next code:

Listing 2.3: code/simp/predictor4.cpp

```
#include <linkpred.hpp>
#include <iostream>
using namespace LinkPred::Simp;
int main() {
  // Create a prtedictor object
  Predictor p;
  // Load network from file
  p.loadnet("Zakarays_Karate_Club.edges");
  // Compute the score for the two edges (1, 34) and (26,34)
  std::vector<EdgeScore> esv = {{"1","34"},{"26","34"}};
  p.predADA(esv);
  // Print the scores
  for (auto it = esv.begin(); it != esv.end(); ++it) {
    std::cout << it->i << "\t" << it->j << "\t" << it->score <<
        std::endl;
```

```
  }
  return 0;
}
```

The following program shows how to obtain the top *k* ranked edges:

Listing 2.4: code/simp/predictor2.cpp

```
#include <linkpred.hpp>
#include <iostream>
using namespace LinkPred::Simp;
int main() {
  int k = 10;
  // Create a prtedictor object
  Predictor p;
  // Load network from file
  p.loadnet("Zakarays_Karate_Club.edges");
  // Predict the top k edges using Adamic Adar index
  std::vector<EdgeScore> esv = p.predTopADA(k);
  // Print the scores
  for (auto it = esv.begin(); it != esv.end(); ++it) {
    std::cout << it->i << "\t" << it->j << "\t" << it->score <<
        std::endl;
  }
  return 0;
}
```

As you might have guessed from the examples above, the class `Predictor` provides three methods for each link prediction algorithm (in what follows, `???` stands for the name of the predictor):

- The method `predAll???()` : This method returns the scores of all non-existing links. Note that for large networks, this method can be memory and CPU-intensive.
- The method `pred???(std::vector<EdgeScore> es)` : This method computes the scores of the edges passed as parameter.
- The method `predTop???(int k)` : Returns the top-*k*-ranked edges along with their scores.

R For most topological similarity methods, computing the top-scored edges using the method `predTop???` is much faster and consumes less memory than calculating the scores of all non-existing link then selecting the links with the highest score.

The parameters of the prediction algorithm -if any- are passed to the methods above but are all given reasonable default values. For example, the following calls:

```
auto es = p.predAllSBM();
p.predSBM(es);
auto es = p.predTopSBM(k);
```

all use `SBM` with the default parameters: the maximum number of iteration is set to 1000 and the seed of the random number generator is set to 0. These can be changed to, respectively, 10000 and 777 as follows:

```
auto es = p.predAllSBM(10000, 777);
p.predSBM(es, 10000, 777);
auto es = p.predTopSBM(k, 10000, 777);
```

2.1.2 Evaluating performance using the class `Simp::Evaluator`

The following program shows how to compare the evaluate the performance of multiple link prediction algorithms using the class `Evaluator` :

Listing 2.5: code/simp/evaluator1.cpp

```
#include <linkpred.hpp>
#include <iostream>
using namespace LinkPred::Simp;
int main() {
  int nbRuns = 10;
  double edgeRemRatio = 0.1;
  // Create an evaluator object
  Evaluator eval;
  // Add predictors to be evaluated
  eval.addCNE();
  eval.addADA();
  eval.addKAB();
  // Add performance measures
  eval.addROC();
  eval.addTPR();
  // Run experiment on the specified network
  eval.run("Zakarays_Karate_Club.edges", nbRuns, edgeRemRatio);
  return 0;
}
```

The output of this program is as follows:

```
#ratio  ROCADA  ROCCNE  ROCKAB  TPRADA  TPRCNE  TPRKAB
0.10    0.7737  0.7149  0.8280  0.1250  0.1932  0.1250
0.10    0.6593  0.6333  0.7030  0.1250  0.0000  0.1250
0.10    0.5967  0.5762  0.6095  0.1875  0.1818  0.2500
0.10    0.8464  0.7913  0.9343  0.1875  0.1290  0.3750
0.10    0.8324  0.7785  0.8967  0.1250  0.1750  0.1250
0.10    0.7240  0.6953  0.7547  0.0000  0.2222  0.0000
0.10    0.6753  0.6610  0.7262  0.0000  0.1591  0.1250
0.10    0.6048  0.5792  0.6672  0.0000  0.0000  0.0000
0.10    0.7627  0.7547  0.7808  0.2917  0.3194  0.3750
0.10    0.6442  0.5835  0.6727  0.1250  0.1250  0.1250
```

Predictors can be added to the evaluation process using the methods `add???` . Similar to the class `Predictor` , the algorithm parameters are passed to these methods if non-default values are needed. Performance measures are added in the same way. Three measures are supported by this class: ROC (area under the ROC curve), PR (area under the precision-recall curve), and TPR (top precision). More information about these performance measures can be found in Chapter 7.

In the example above, the performance evaluation is conducted on the network located in the file `"Zakarays_Karate_Club.edges"` . This is the ground-truth network containing all edges. The library will automatically generate test data by randomly removing the specified ratio of edges passed through the argument `edgeRemRatio` (in this case 10%). The removed edges will be used as a test set. This process is repeated `nbRunTimes` (in this example, 10 times).

The output above is printed from within the method `run` . It is also possible to access the results of each iteration as follows (after calling `run`):

Listing 2.6: code/simp/evaluator2.cpp

```
// Print the header row
auto res = eval.getPerfRes(0);
for (auto it =  res.begin(); it != res.end(); ++it) {
  std::cout << it->name << "\t" ;
}
std::cout << "\n";
// Print the results of each iteration
for(int i = 0; i < nbRuns; i++) {
  auto res = eval.getPerfRes(i);
  for (auto it =  res.begin(); it != res.end(); ++it) {
    std::cout << it->res << "\t";
  }
  std::cout << "\n";
}
```

> **R** Each call to the method `run` overrides the performance results. Therefore, only the results from the latest call are available.

The performance results of each iteration are returned as an `std::vector<PerfRes>`, where `PerfRes` is a simple structure containing two fields: The name of the result, which a concatenation of the name of the performance measure followed by the name of the prediction algorithm, and a second field containing the numerical value of the result:

Listing 2.7: code/simp/perfres.hpp

```
struct PerfRes {
  std::string name; /**< Concatenation of the name of the
     performance mneasure and that of the predictor. */
  double res; /**< The result. */
};
```

Instead of automatically generating the test data, it is possible to pass a pre-split network to the method `run`. This is useful when comparing with algorithms implemented elsewhere.

Listing 2.8: code/simp/evaluator3.cpp

```
#include <linkpred.hpp>
#include <iostream>
using namespace LinkPred::Simp;
int main() {
  // Create an evaluator object
  Evaluator eval;
  // Add predictors to be evaluated
  eval.addADA();
  eval.addRAL();
  // Add performance measures
  eval.addPR();
  eval.addTPR();
  // Run experiment on the specified network
```

```
  eval.run("Zakarays_Karate_Club_Train.edges", "
     Zakarays_Karate_Club_Test.edges");
  return 0;
}
```

The output of this program is as follows:

```
PRADA   PRRAL   TPRADA  TPRRAL
0.1561  0.1568  0.1250  0.1250
```

Note that in this setting, only one test run is conducted. To get the results, it also possible to proceed as follows:

Listing 2.9: code/simp/evaluator4.cpp

```
auto res = eval.getPerfRes(0);
for (auto it =  res.begin(); it != res.end(); ++it) {
  std::cout << it->name << "\t" ;
}
std::cout << "\n";
for (auto it =  res.begin(); it != res.end(); ++it) {
  std::cout << it->res << "\t" ;
}
std::cout << "\n";
```

The class `Simp::Evaluator` simplifies further the process of comparing the performance of new link prediction algorithms to those implemented in LinkPred by providing a method to generate test data and one that allows to include pre-calculated prediction results into the evaluation process.

To create test data, the class `Simp::Evaluator` provides the method:

```
void genTestData(std::string const & fullNetFileName, std::string
     const &obsEdgesFileName, std::string const &remEdgesFileName,
     double remRatio = 0.1, bool keepConnected = false, long int
    seed = 0);
```

where

- `fullNetFileName` is the file containing the ground truth network.
- `obsEdgesFileName` is the file where the remaining (non-removed) edges are written. This file will contain the observed network (the training set).
- `remEdgesFileName` is the file where the removed edges are written. This file will contain the set of positive examples of the test set.
- `remRatio` is the ratio of edges that will be removed.
- `keepConnected` indicates whether to keep the graph connected when removing edges. Note that keeping the graph connected may be impossible for high edge removed ratios or if the network is initially disconnected.
- `seed` is used to initialize the random number generator.

The training set (the observed network composed of the edges stored in `obsEdgesFileName`) can be used to train the user's link predictor. The results of all non-existing links in the observed network and stored in a text file, which is then added to the evaluation process using the method:

```
void addPST(std::string const & name = "PST", std::string
    fileName = "pst.txt");
```

This method creates a new link prediction algorithm that plays a proxy role on behalf of the user's algorithm and uses the pre-stored data to predict links. The parameters `name` is the name given to this link predictor, and `fileName` is where the scores of all non-existing links are stored. The format of this file is as follows (the first is just a comment and can be omitted):

```
#Start   End     Score
1        31      1.20225
1        10      0.45512
1        28      0.45512
1        29      1.07645
1        33      1.17647
1        17      1.4427
1        34      2.15291
1        26      0.621335
1        25      0.621335
...
```

The two following programs show how to use these two methods:

Listing 2.10: code/simp/evaluator5.cpp

```
#include <linkpred.hpp>
#include <iostream>
using namespace LinkPred::Simp;
int main() {
  double edgeRemRatio = 0.1;
  bool keepConnected = false;
  long int seed = 0;
  // Create an evaluator object
  Evaluator eval;
  // Generate test data
  eval.genTestData("Zakarays_Karate_Club.edges", "Zakarays_Train.
     edges", "Zakarays_Test.edges", edgeRemRatio, keepConnected,
     seed);
  return 0;
}
```

After running this code, two files will be generated, `Zakarays_Train.edges`, which contains the observed network and `Zakarays_Test.edges`, which contains the removed edges. Use the edges in `Zakarays_Train.edges` to train your algorithm, then compute the scores of all edges that are not observed (not only the removed edges!) and store them in a file named `pst.txt`. Now, you can use these predictions to compare the performance of your algorithm against `ADA` and `RAL` for example.

Listing 2.11: code/simp/evaluator6.cpp

```
#include <linkpred.hpp>
#include <iostream>
using namespace LinkPred::Simp;
int main() {
  Evaluator eval;
  eval.addADA();
  // Load scores from pst.txt
  eval.addPST("PST", "pst.txt");
```

```
  eval.addRAL();
  eval.addPR();
  eval.addTPR();
  eval.run("Zakarays_Train.edges", "Zakarays_Test.edges");
  return 0;
}
```

The output of this code is as follows:

```
PRADA   PRPST   PRRAL   TPRADA  TPRPST  TPRRAL
0.0510  0.0918  0.0391  0.1250  0.2500  0.1250
```

2.2 Java Bindings

The Java bindings to LinkPred are generated using SWIG (ww.swig.org), which wraps C/C++ code using Java proxy classes. Building the Java bindings requires a Java compiler and uses JNI to interface with the C++ code. Upon successful building, the library `LinkPredJava` will be generated (named `libLinkPredJava.so` in Linux). This library will be loaded when running your program, and for that, it must be accessible to the Java virtual machine.

R In Linux, you can make the library accessible to the JVM by including its path in the environment variable `LD_LIBRARY_PATH`. If LinkPred is installed in the default location, this can be accomplished using the following command:

```
$ export LD_LIBRARY_PATH=$LD_LIBRARY_PATH:/usr/
  local/lib
```

The Java proxy classes needed to interface with the library can be found in source form and JAR form (`LinkPredJava.jar`) in the source directory of LinkPred in `/bindings/Java`. These classes (either in source or as JAR) must be included in the class path during compilation and at run-time. Assuming that your code is in the class `Example` and that the file `LinkPredJava.jar` is in the same directory as `Example.java`, you can compile and run your code using:

```
$ javac -cp .:./LinkPredJava.jar Example.java
$ java -cp .:./LinkPredJava.jar Example
```

2.2.1 Predicting links using the class `Predictor`

The following code shows how to compute the score of all non-existing links of a network using Adamic Adar index and print the result:

Listing 2.12: code/simp/Predictor1.java

```
public class Predictor1 {
  static {
    // Load the library
    System.loadLibrary("LinkPredJava");
  }
  public static void main(String[] args) {
```

```
    // Create a prtedictor object
    Predictor p = new Predictor();
    // Load network from file
    p.loadnet("Zakarays_Karate_Club.edges");
    // Predict the score of all non-exisitng edges using Adamic
       Adar index
    EdgeScoreVec esv = p.predAllADA();
    // Print the scores
    for (int i = 0; i < esv.size(); i++) {
      EdgeScore es = esv.get(i);
      System.out.println(es.getI() + "\t" + es.getJ() + "\t" + es
         .getScore());
    }
  }
}
```

The class `Predictor` returns the results in an object of type `EdgeScoreVec` (a SWIG proxy for `std::vector<EdgeScore>`), where each entry stores the score of an edge in the class `EdgeScore` which has the following member accessors:

Listing 2.13: code/simp/EdgeScore.java

```
public class EdgeScore {
  ...
  public String getI() // Get the label of the start node
  public String getJ() // Get the label of the end node
  public double getScore() // Get the score
  public String setI(String i) // Set the label of the start node
  public String setJ(String j) // Set the label of the end node
  public double setScore(double score) // Set the score
}
```

It is also possible to limit the prediction to specific edges which are passed as parameter as shown in the next code:

Listing 2.14: code/simp/Predictor4.java

```
public class Predictor4 {
  static {
    // Load the library
    System.loadLibrary("LinkPredJava");
  }
  public static void main(String[] args) {
    // Create a predictor object
    Predictor p = new Predictor();
    // Load network from file
    p.loadnet("Zakarays_Karate_Club.edges");
    // Compute the score for the two edges (1, 34) and (26,34)
    EdgeScoreVec esv = new EdgeScoreVec();
    EdgeScore es;
    es = new EdgeScore();
    es.setI("1");
    es.setJ("34");
    esv.add(es);
    es = new EdgeScore();
    es.setI("26");
```

```
    es.setJ("34");
    esv.add(es);
    p.predKAB(esv);
    // Print the scores
    for (int i = 0; i < esv.size(); i++) {
      es = esv.get(i);
      System.out.println(es.getI() + "\t" + es.getJ() + "\t" + es
         .getScore());
    }
  }
}
```

The following program shows how to obtain the top k ranked edges:

Listing 2.15: code/simp/Predictor2.java

```
public class Predictor2 {
  static {
    // Load the library
    System.loadLibrary("LinkPredJava");
  }
  public static void main(String[] args) {
    int k = 10;
    // Create a prtedictor object
    Predictor p = new Predictor();
    // Load network from file
    p.loadnet("Zakarays_Karate_Club.edges");
    // Predict the top k edges using Adamic Adar index
    EdgeScoreVec esv = p.predTopADA(k);
    // Print the scores
    for (int i = 0; i < esv.size(); i++) {
      EdgeScore es = esv.get(i);
      System.out.println(es.getI() + "\t" + es.getJ() + "\t" + es
         .getScore());
    }
  }
}
```

As you might have guessed from the examples above, the class `Predictor` provides three methods for each link prediction algorithm (in what follows, `???` stands for the name of the predictor):

- The method `predAll???()` : This method returns the scores of all non-existing links. Note that for large networks, this method can be memory and CPU-intensive.
- The method `pred???(EdgeScoreVec esv)` : This method computes the scores of the edges passed as parameter.
- The method `predTop???(int k)` : Returns the top-k-ranked edges along with their scores.

> **R** For most topological similarity methods, computing the top-scored edges using the method `predTop???` is much faster and consumes less memory than calculating the scores of all non-existing link then selecting the links with the highest score.

The parameters of the prediction algorithm -if any- are passed to the methods above but are all given reasonable default values. For example, the following calls:

```
EdgeScoreVec esv = p.predAllSBM();
p.predSBM(esv);
EdgeScoreVec esv = p.predTopSBM(k);
```

all use `SBM` with the default parameters: the maximum number of iteration is set to 1000 and the seed of the random number generator is set to 0. These can be changed to, respectively, 10000 and 777 as follows:

```
EdgeScoreVec esv = p.predAllSBM(10000, 777);
p.predSBM(esv, 10000, 777);
EdgeScoreVec esv = p.predTopSBM(k, 10000, 777);
```

2.2.2 Evaluating performance using the class `Evaluator`

The following program shows how to compare the evaluate the performance of multiple link prediction algorithms using the class `Evaluator`:

Listing 2.16: code/simp/Evaluator1.java

```
public class Evaluator1 {
  static {
    // Load the library
    System.loadLibrary("LinkPredJava");
  }
  public static void main(String[] args) {
    int nbRuns = 10;
    double edgeRemRatio = 0.1;
    // Create an evaluator object
    Evaluator eval = new Evaluator();
    // Add predictors to be evaluated
    eval.addCNE();
    eval.addADA();
    eval.addKAB();
    // Add performance measures
    eval.addROC();
    eval.addTPR();
    // Run experiment on the specified network
    eval.run("Zakarays_Karate_Club.edges", nbRuns, edgeRemRatio);
  }
}
```

The output of this program is as follows:

```
#ratio  ROCADA  ROCCNE  ROCKAB  TPRADA  TPRCNE  TPRKAB
0.10    0.7737  0.7149  0.8280  0.1250  0.1932  0.1250
0.10    0.6593  0.6333  0.7030  0.1250  0.0000  0.1250
0.10    0.5967  0.5762  0.6095  0.1875  0.1818  0.2500
0.10    0.8464  0.7913  0.9343  0.1875  0.1290  0.3750
0.10    0.8324  0.7785  0.8967  0.1250  0.1750  0.1250
0.10    0.7240  0.6953  0.7547  0.0000  0.2222  0.0000
0.10    0.6753  0.6610  0.7262  0.0000  0.1591  0.1250
0.10    0.6048  0.5792  0.6672  0.0000  0.0000  0.0000
0.10    0.7627  0.7547  0.7808  0.2917  0.3194  0.3750
0.10    0.6442  0.5835  0.6727  0.1250  0.1250  0.1250
```

Predictors can be added to the evaluation process using the methods `add???`. Similar to the class `Predictor`, the algorithm parameters are passed to these methods if non-default values are needed. Performance measures are added in the same way. Three measures are supported by this class: ROC (area under the ROC curve), PR (area under the precision-recall curve), and TPR (top precision). More information about these performance measures can be found in Chapter 7.

In the example above, the performance evaluation is conducted on the network located in the file `"Zakarays_Karate_Club.edges"`. This is the ground-truth network containing all edges. The library will automatically generate test data by randomly removing the specified ratio of edges passed through the argument `edgeRemRatio` (in this case 10%). The removed edges will be used as a test set. This process is repeated `nbRunTimes` (in this example, 10 times).

The output above is printed from within the method `run`. It is also possible to access the results of each iteration as follows (after calling `run`):

Listing 2.17: code/simp/Evaluator2.java

```
// Print the header row
PerfResVec res = eval.getPerfRes(0);
for (int j = 0; j < res.size(); j++) {
  System.out.print(res.get(j).getName() + "\t") ;
}
System.out.println();
// Print the results of each iteration
for(int i = 0; i < nbRuns; i++) {
  res = eval.getPerfRes(i);
  for (int j = 0; j < res.size(); j++) {
    System.out.printf("%.4f\t", res.get(j).getRes()) ;
  }
  System.out.println();
}
```

R Each call to the method `run` overrides the performance results. Therefore, only the results from the latest call are available.

The performance results of each iteration are returned as an object of type `PerfResVec` (a SWIG proxy for `std::vector<PerfRes>`), where `PerfRes` is a simple class containing two fields: The name of the result, which a concatenation of the name of the performance measure followed by the name of the prediction algorithm, and a second field containing the numerical value of the result:

Listing 2.18: code/simp/PerfRes.java

```
public class PerfRes {
  ...
  public String getName() // Get the name of the performance
     result
  public double getRes() // Get the value of the performance
     result
  public void setName(String value) // Set the name of the
     performance result
```

```
  public void setRes(double value) // Set the value of the
      performance result
}
```

Instead of automatically generating the test data, it is possible to pass a pre-split network to the method `run`. This is useful when comparing with algorithms implemented elsewhere.

Listing 2.19: code/simp/Evaluator3.java

```
public class Evaluator3 {
  static {
    // Load the library
    System.loadLibrary("LinkPredJava");
  }
  public static void main(String[] args) {
    // Create an evaluator object
    Evaluator eval = new Evaluator();
    // Add predictors to be evaluated
    eval.addCNE();
    eval.addADA();
    eval.addKAB();
    // Add performance measures
    eval.addROC();
    eval.addTPR();
    // Run experiment on the specified network
    eval.run("Zakarays_Karate_Club_Train.edges", "
        Zakarays_Karate_Club_Test.edges");
  }
}
```

The output of this program is as follows:

```
PRADA   PRRAL   TPRADA  TPRRAL
0.1561  0.1568  0.1250  0.1250
```

Note that in this setting, only one test run is conducted. To get the results, it also possible to proceed as follows:

Listing 2.20: code/simp/Evaluator4.java

```
PerfResVec res = eval.getPerfRes(0);
for (int j = 0; j < res.size(); j++) {
  System.out.print(res.get(j).getName() + "\t") ;
}
System.out.println();
for (int j = 0; j < res.size(); j++) {
  System.out.printf("%.4f\t", res.get(j).getRes()) ;
}
System.out.println();
```

The class `Evaluator` simplifies further the process of comparing the performance of new link prediction algorithms to those implemented in LinkPred by providing a method to generate test data and one that allows to include pre-calculated prediction results into the evaluation process.

To create test data, the class `Simp::Evaluator` provides the method:

```
void genTestData(String fullNetFileName, String obsEdgesFileName,
    String remEdgesFileName, double remRatio, boolean
   keepConnected, int seed);
```

where

- `fullNetFileName` is the file containing the ground truth network.
- `obsEdgesFileName` is the file where the remaining (non-removed) edges are written. This file will contain the observed network (the training set).
- `remEdgesFileName` is the file where the removed edges are written. This file will contain the set of positive examples of the test set.
- `remRatio` is the ratio of edges that will be removed.
- `keepConnected` indicates whether to keep the graph connected when removing edges. Note that keeping the graph connected may be impossible for high edge removed ratios or if the network is initially disconnected.
- `seed` is used to initialize the random number generator.

The training set (the observed network composed of the edges stored in `obsEdgesFileName`) can be used to train the user's link predictor. The results of all non-existing links in the observed network and stored in a text file, which is then added to the evaluation process using the method:

```
void addPST(String name, String fileName);
```

This method creates a new link prediction algorithm that plays a proxy role on behalf of the user's algorithm and uses the pre-stored data to predict links. The parameters `name` is the name given to this link predictor, and `fileName` is where the scores of all non-existing links are stored. The format of this file is as follows (the first is just a comment and can be omitted):

```
#Start  End     Score
1       31      1.20225
1       10      0.45512
1       28      0.45512
1       29      1.07645
1       33      1.17647
1       17      1.4427
1       34      2.15291
1       26      0.621335
1       25      0.621335
...
```

The two following programs show how to use these two methods:

Listing 2.21: code/simp/Evaluator5.java

```
public class Evaluator5 {
  static {
    System.loadLibrary("LinkPredJava"); // Load the library
  }
  public static void main(String[] args) {
    double edgeRemRatio = 0.1;
    boolean keepConnected = false;
    int seed = 0;
    Evaluator eval = new Evaluator();
```

```
    eval.genTestData("Zakarays_Karate_Club.edges", "
        Zakarays_Train.edges", "Zakarays_Test.edges", edgeRemRatio
        , keepConnected, seed);
  }
}
```

After running this code, two files will be generated, `Zakarays_Train.edges`, which contains the observed network and `Zakarays_Test.edges`, which contains the removed edges. Use the edges in `Zakarays_Train.edges` to train your algorithm, then compute the scores of all edges that are not observed (not only the removed edges!) and store them in a file named `pst.txt`. Now, you can use these predictions to compare the performance of your algorithm against `ADA` and `RAL` for example.

Listing 2.22: code/simp/Evaluator6.java

```java
public class Evaluator6 {
  static {
    System.loadLibrary("LinkPredJava"); // Load the library
  }
  public static void main(String[] args) {
    Evaluator eval = new Evaluator();
    eval.addADA();
    // Load scores from pst.txt
    eval.addPST("PST", "pst.txt");
    eval.addRAL();
    eval.addPR();
    eval.addTPR();
    eval.run("Zakarays_Train.edges", "Zakarays_Test.edges");
  }
}
```

The output of this code is as follows:

```
PRADA   PRPST   PRRAL   TPRADA  TPRPST  TPRRAL
0.0510  0.0918  0.0391  0.1250  0.2500  0.1250
```

2.3 Python Bindings

The Python bindings to LinkPred are generated using SWIG (ww.swig.org), which wraps C/C++ code using Python proxy classes. Upon successful building, the library `_LinkPredPython` will be generated (named `_LinkPredPython.so` in Linux). This library will be loaded when running your program, and for that, it must be accessible to Python.

R In Linux, you can make the library accessible to the Python by including its path in the environment variable `PYTHONPATH`. If LinkPred is installed in the default location, this can be accomplished using the following command:

```
$ export PYTHONPATH=$PYTHONPATH:/usr/local/lib
```

The Python module `LinkPredPython` containing the proxy classes needed to interface with `LinkPred` is located in `/bindings/Python`. Python programs that use `LinkPred` must import this module, which must therefore be in the Python module search path (for instance, in the same directory as your code).

2.3.1 Predicting links using the class `Predictor`

The following code shows how to compute the score of all non-existing links of a network using Adamic Adar index and print the result:

Listing 2.23: code/simp/predictor1.py

```
# Import the module
import LinkPredPython as lpp
# Create a predictor object
p = lpp.Predictor();
# Load network from file
p.loadnet("Zakarays_Karate_Club.edges");
# Predict the score of all non-exisitng edges using Adamic Adar
    index
esv = p.predAllADA();
# Print the scores
for es in esv:
  print(es.i + "\t" + es.j + "\t" + "{:.4f}".format(es.score));
```

The class `Predictor` returns the results in an object of type `EdgeScoreVec` (a SWIG proxy for `std::vector<EdgeScore>`), where each entry stores the score of an edge in the class `EdgeScore`:

Listing 2.24: code/simp/edgescore.py

```
class EdgeScore:
  i = ""; # The label of the start node.
  j = ""; # The label of the end node.
  score = 0; # The score.
```

It is also possible to limit the prediction to specific edges which are passed as parameter as shown in the next code:

Listing 2.25: code/simp/predictor4.py

```
# Import the module
import LinkPredPython as lpp
# Create a prtedictor object
p = lpp.Predictor();
# Load network from file
p.loadnet("Zakarays_Karate_Club.edges");
# Compute the score for the two edges (1, 34) and (26,34)
esv = lpp.EdgeScoreVec();
es = lpp.EdgeScore();
es.i = "1";
es.j = "34";
esv.push_back(es);
es.i = "26";
es.j = "34";
esv.push_back(es);
p.predKAB(esv);
# Print the scores
for es in esv:
  print(es.i + "\t" + es.j + "\t" + "{:.4f}".format(es.score));
```

The following program shows how to obtain the top k ranked edges:

Listing 2.26: code/simp/predictor2.py

```
# Import the module
import LinkPredPython as lpp
k = 10;
# Create a predictor object
p = lpp.Predictor();
# Load network from file
p.loadnet("Zakarays_Karate_Club.edges");
# Predict the top k edges using Adamic Adar index
esv = p.predTopADA(k);
# Print the scores
for es in esv:
  print(es.i + "\t" + es.j + "\t" + "{:.4f}".format(es.score));
```

As you might have guessed from the examples above, the class `Predictor` provides three methods for each link prediction algorithm (in what follows, `???` stands for the name of the predictor):

- The method `predAll???()` : This method returns the scores of all non-existing links. Note that for large networks, this method can be memory and CPU-intensive.
- The method `pred???(EdgeScoreVec esv)` : This method computes the scores of the edges passed as parameter.
- The method `predTop???(int k)` : Returns the top-k-ranked edges along with their scores.

> **R** For most topological similarity methods, computing the top-scored edges using the method `predTop???` is much faster and consumes less memory than calculating the scores of all non-existing link then selecting the links with the highest score.

The parameters of the prediction algorithm -if any- are passed to the methods above but are all given reasonable default values. For example, the following calls:

```
esv = p.predAllSBM();
p.predSBM(esv);
esv = p.predTopSBM(k);
```

all use `SBM` with the default parameters: the maximum number of iteration is set to 1000 and the seed of the random number generator is set to 0. These can be changed to, respectively, 10000 and 777 as follows:

```
esv = p.predAllSBM(10000, 777);
p.predSBM(esv, 10000, 777);
esv = p.predTopSBM(k, 10000, 777);
```

2.3.2 Evaluating performance using the class `Evaluator`

The following program shows how to compare the evaluate the performance of multiple link prediction algorithms using the class `Evaluator` :

Listing 2.27: code/simp/evaluator1.py

```
# Import the module
import LinkPredPython as lpp
```

```
nbRuns = 10;
edgeRemRatio = 0.1;
# Create an evaluator object
ev = lpp.Evaluator();
# Add predictors to be evaluated
ev.addCNE();
ev.addADA();
ev.addKAB();
# Add performance measures
ev.addROC();
ev.addTPR();
# Run experiment on the specified network
ev.run("Zakarays_Karate_Club.edges", nbRuns, edgeRemRatio);
```

The output of this program is as follows:

```
#ratio  ROCADA  ROCCNE  ROCKAB  TPRADA  TPRCNE  TPRKAB
0.10    0.7737  0.7149  0.8280  0.1250  0.1932  0.1250
0.10    0.6593  0.6333  0.7030  0.1250  0.0000  0.1250
0.10    0.5967  0.5762  0.6095  0.1875  0.1818  0.2500
0.10    0.8464  0.7913  0.9343  0.1875  0.1290  0.3750
0.10    0.8324  0.7785  0.8967  0.1250  0.1750  0.1250
0.10    0.7240  0.6953  0.7547  0.0000  0.2222  0.0000
0.10    0.6753  0.6610  0.7262  0.0000  0.1591  0.1250
0.10    0.6048  0.5792  0.6672  0.0000  0.0000  0.0000
0.10    0.7627  0.7547  0.7808  0.2917  0.3194  0.3750
0.10    0.6442  0.5835  0.6727  0.1250  0.1250  0.1250
```

Predictors can be added to the evaluation process using the methods `add???`. Similar to the class `Predictor`, the algorithm parameters are passed to these methods if non-default values are needed. Performance measures are added in the same way. Three measures are supported by this class: ROC (area under the ROC curve), PR (area under the precision-recall curve), and TPR (top precision). More information about these performance measures can be found in Chapter 7.

In the example above, the performance evaluation is conducted on the network located in the file `"Zakarays_Karate_Club.edges"`. This is the ground-truth network containing all edges. The library will automatically generate test data by randomly removing the specified ratio of edges passed through the argument `edgeRemRatio` (in this case 10%). The removed edges will be used as a test set. This process is repeated `nbRunTimes` (in this example, 10 times).

The output above is printed from within the method `run`. It is also possible to access the results of each iteration as follows (after calling `run`):

Listing 2.28: code/simp/evaluator2.py

```
import sys # For printing
# Print the header row
res = ev.getPerfRes(0);
for r in res:
  sys.stdout.write(r.name + "\t");
sys.stdout.write("\n");
# Print the results of each iteration
for i in range(nbRuns):
  res = ev.getPerfRes(i);
```

```
  for r in res:
    sys.stdout.write("{:.4f}".format(r.res) + "\t");
  sys.stdout.write("\n");
```

Ⓡ Each call to the method `run` overrides the performance results. Therefore, only the results from the latest call are available.

The performance results of each iteration are returned as an object of type `PerfResVec` (a SWIG proxy for `std::vector<PerfRes>`), where `PerfRes` is a simple class containing two fields: The name of the result, which a concatenation of the name of the performance measure followed by the name of the prediction algorithm, and a second field containing the numerical value of the result:

Listing 2.29: code/simp/perfres.py

```
class PerfRes:
  name = ""; # Concatenation of the name of the performance
      mneasure and that of the predictor.
  res = 0; # The result.
```

Instead of automatically generating the test data, it is possible to pass a pre-split network to the method `run`. This is useful when comparing with algorithms implemented elsewhere.

Listing 2.30: code/simp/evaluator3.py

```
# Import the module
import LinkPredPython as lpp
# Create an evaluator object
ev = lpp.Evaluator();
# Add predictors to be evaluated
ev.addCNE();
ev.addADA();
ev.addKAB();
# Add performance measures
ev.addROC();
ev.addTPR();
ev.run("Zakarays_Karate_Club_Train.edges", "
    Zakarays_Karate_Club_Test.edges");
```

The output of this program is as follows:

```
PRADA   PRRAL   TPRADA  TPRRAL
0.1561  0.1568  0.1250  0.1250
```

Note that in this setting, only one test run is conducted. To get the results, it also possible to proceed as follows:

Listing 2.31: code/simp/evaluator4.py

```
import sys # For printing
res = ev.getPerfRes(0);
for r in res:
  sys.stdout.write(r.name + "\t");
```

```
sys.stdout.write("\n");
for r in res:
  sys.stdout.write("{:.4f}".format(r.res) + "\t");
sys.stdout.write("\n");
```

The class `Evaluator` simplifies further the process of comparing the performance of new link prediction algorithms to those implemented in LinkPred by providing a method to generate test data and one that allows to include pre-calculated prediction results into the evaluation process.

To create test data, the class `Simp::Evaluator` provides the method:

```
genTestData(self, fullNetFileName, obsEdgesFileName,
    remEdgesFileName, remRatio=0.1, keepConnected=False, seed=0)
```

where

- `fullNetFileName` is the file containing the ground truth network.
- `obsEdgesFileName` is the file where the remaining (non-removed) edges are written. This file will contain the observed network (the training set).
- `remEdgesFileName` is the file where the removed edges are written. This file will contain the set of positive examples of the test set.
- `remRatio` is the ratio of edges that will be removed.
- `keepConnected` indicates whether to keep the graph connected when removing edges. Note that keeping the graph connected may be impossible for high edge removed ratios or if the network is initially disconnected.
- `seed` is used to initialize the random number generator.

The training set (the observed network composed of the edges stored in `obsEdgesFileName`) can be used to train the user's link predictor. The results of all non-existing links in the observed network and stored in a text file, which is then added to the evaluation process using the method:

```
addPST(name, fileName);
```

This method creates a new link prediction algorithm that plays a proxy role on behalf of the user's algorithm and uses the pre-stored data to predict links. The parameters `name` is the name given to this link predictor, and `fileName` is where the scores of all non-existing links are stored. The format of this file is as follows (the first is just a comment and can be omitted):

```
#Start   End      Score
1        31       1.20225
1        10       0.45512
1        28       0.45512
1        29       1.07645
1        33       1.17647
1        17       1.4427
1        34       2.15291
1        26       0.621335
1        25       0.621335
...
```

The two following programs show how to use these two methods:

Listing 2.32: code/simp/evaluator5.py

```
# Import the library
```

```python
import LinkPredPython as lpp
edgeRemRatio = 0.1;
keepConnected = False;
seed = 0;
# Create an evaluator object
ev = lpp.Evaluator();
ev.genTestData("Zakarays_Karate_Club.edges", "Zakarays_Train.
    edges", "Zakarays_Test.edges", edgeRemRatio, keepConnected,
    seed);
```

After running this code, two files will be generated, `Zakarays_Train.edges`, which contains the observed network and `Zakarays_Test.edges`, which contains the removed edges. Use the edges in `Zakarays_Train.edges` to train your algorithm, then compute the scores of all edges that are not observed (not only the removed edges!) and store them in a file named `pst.txt`. Now, you can use these predictions to compare the performance of your algorithm against `ADA` and `RAL` for example.

Listing 2.33: code/simp/evaluator6.py

```python
# Import the library
import LinkPredPython as lpp
# Create an evuator object
ev = lpp.Evaluator();
ev.addADA();
# Load scores from pst.txt
ev.addPST("PST", "pst.txt");
ev.addRAL();
ev.addPR();
ev.addTPR();
ev.run("Zakarays_Train.edges", "Zakarays_Test.edges");
```

The output of this code is as follows:

```
PRADA   PRPST   PRRAL   TPRADA  TPRPST  TPRRAL
0.0510  0.0918  0.0391  0.1250  0.2500  0.1250
```

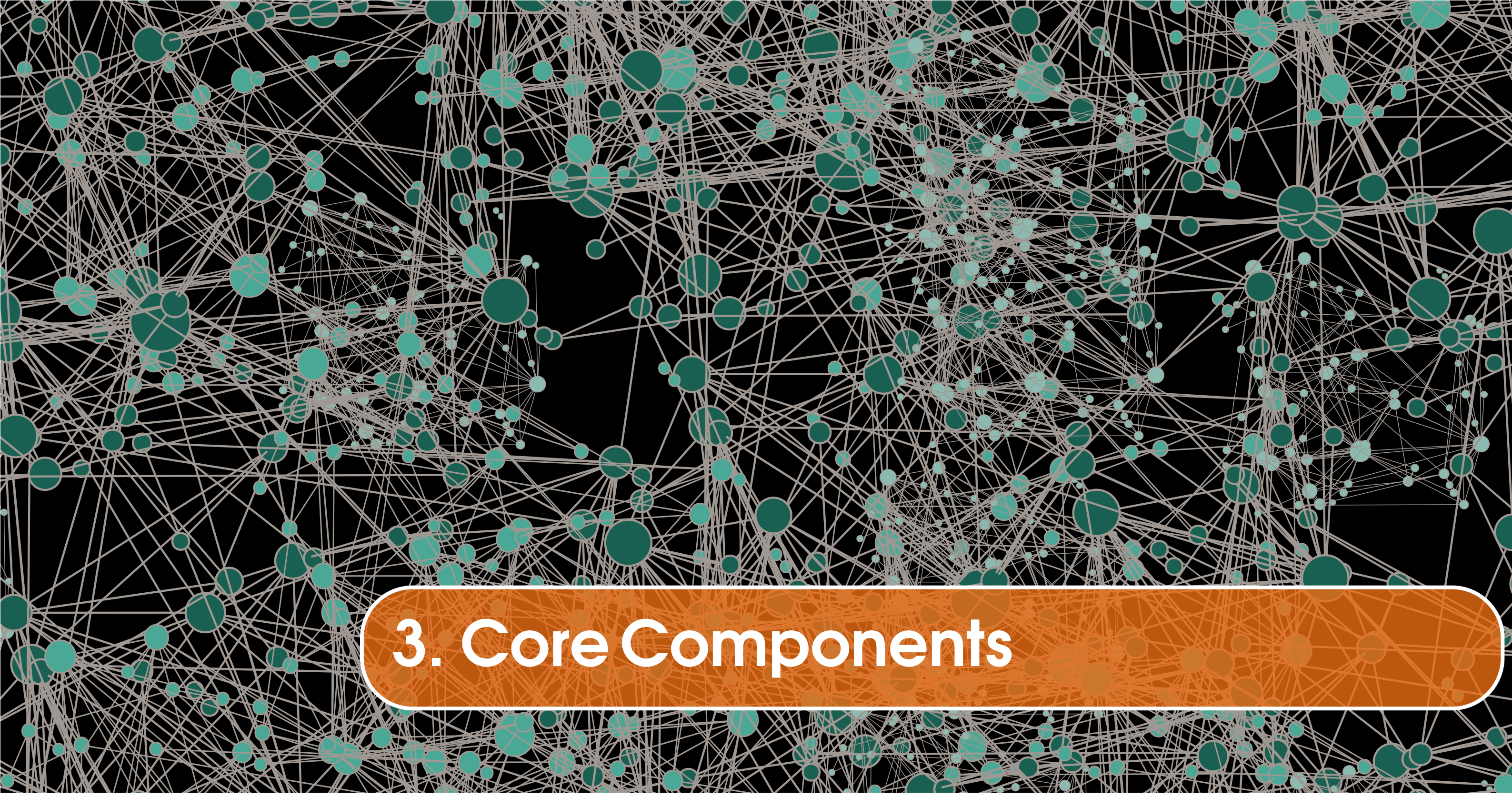

3. Core Components

This chapter is concerned with the basic building blocks of LinkPred. Some of these components, for instance the network data structures, are essential for an optimal use of the library. Other components can be very useful for building new efficient link prediction algorithms. For a first reading, we invite the reader to study Section 3.1 and come back for the remaining sections at a later time or when necessary.

> R Core classes are grouped under the namespace `Core`, and can be imported using:
>
> ```
> using namespace LinkPred;
> ```
>
> In the examples included in this chapter, we assume that this namespace is imported and drop the prefix `LinkPred::` from all classes for convenience.

3.1 The undirected network data structure

At the heart of LinkPred lies the class `UNetwork`, which represents an undirected network. This is a data structure designed to efficiently represent immutable graphs (graphs that once created are not modified). It offers efficient access to nodes, edges and non-existing edges as well.

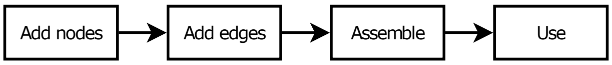

Figure 3.1: Example network.

The life cycle of a network has two distinct phases:

- **Pre-assembly**: In this phase, it is possible to add nodes and edges to the network. It is also possible to access nodes and translate external labels to internal IDs and vice versa. However, most functionalities related to accessing edges are not yet available. As a result, the network at this stage is practically unusable. To be able to use the network, it is first necessary to assemble it.

- **Post-assembly**: Once assembled, no new nodes or edges can be added (or removed) to the network. The network is now fully functional and can be passed as argument to any method that requires so.

R Attempting to add new nodes or edges after assembling the network produces an exception. On the other hand, due to performance considerations, no such checks are made in methods that prerequire assembly. Therefore, using a network before assembling it may result in unspecified behavior.

3.1.1 Building the network

To build a network, we first create an empty network, named for instance `net`, by calling the default constructor:

```
UNetwork<> net;
```

Most classes in LinkPred manipulate networks through smart pointers for efficient memory management. To create a shared pointer to a `UNetwork` object:

```
auto net = std::make_shared<UNetwork<>>();
```

Notice that the class `UNetwork` is a class template, which is here instantiated with the default template arguments. In this default setting, the labels are of type `std::string`, whereas internal IDs are of type **`unsigned int`**, but `UNetwork` can be instantiated with a number of other data types if wanted. For instance, the labels can be of type **`unsigned int`**, which may reduce storage size in some situations.

Adding nodes is achieved by calling the method `addNode`, which takes as parameter the node label and returns an `std::pair` containing, respectively, the node ID and a Boolean which is set to true if the node is newly inserted, false if the node already exists. The nodes IDs are guaranteed to be contiguous in $0,\ldots,n-1$, where n is the number of nodes. Inserting a node that already exists has no effect.

```
auto res = net.addNode(label);
auto id  = res.first; // This the node ID
bool inserted = res.second; // Was the node inserted or did it
   already exist?
```

The method `addEdge` is used to create an edge between two nodes specified by their IDs (**not their labels**):

```
net.addEdge(i, j);
```

A possible and shorter way to create edges without the need for adding nodes beforehand or storing externally their IDs is as follows[1]:

```
net.addEdge(net.addNode(labelI).first, net.addNode(labelJ).first)
   ;
```

[1]Notice that the ID assigned to a node depends on the order in which this node is added to the network. Therefore, depending on the order in which function arguments are processed (which is implementation-dependent), the nodes may be assigned different internal IDs when using this code. This, however, has no effect whatsoever on the results.

Loops are not allowed, and attempting to add one results in an exception. Adding the same edge more than one time, including the case where both an edge (i, j) and its inverse (j, i) are inserted, has no effect.

The last step in building the network is to assemble it:

```
net.assemble();
```

The method `assemble` initializes the internal data structures and makes the network ready to be used.

The class `UNetwork` offers also a static method that reads the network data from file:

```
std::string fileName = "Infectious.edges";
auto net = UNetwork<>::read(fileName);
```

The file must be in text format with each line specifying an edge. No comments are allowed in the file. An example input file is the following:

```
1 2
1 3
2 4
3 5
2 6
```

■ **Example 3.1** Consider the network shown in Figure 3.2.

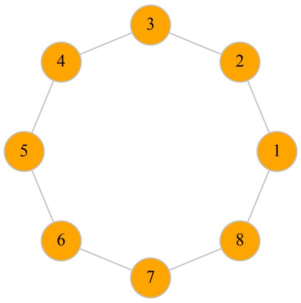

Figure 3.2: Example network.

The code below shows how to build this network:

Listing 3.1: code/core/NetworkBuild1.cpp

```
#include <linkpred.hpp>
#include <iostream>
using namespace LinkPred;
int main() {
  int n = 8;
  UNetwork<unsigned int> net; // Labels are of type unsigned int
  std::cout << "Label\tID\tNew?" << std::endl;
  for (int i = 1; i <= n; i++) {
    auto res = net.addNode(i);
    std::cout << i << "\t" << res.first << "\t" << res.second <<
        std::endl;
  }
  for (int i = 1; i <= n; i++) {
    net.addEdge(net.getID(i), net.getID(i % n + 1));
```

```
    net.addEdge(net.getID(i), net.getID((i + 1) % n + 1));
  }
  net.assemble();
  std::cout << "Printing␣network:" << std::endl;
  net.print();
  return 0;
}
```

This is the output of this code:

```
Label   ID      New?
1       0       1
2       1       1
3       2       1
4       3       1
5       4       1
6       5       1
7       6       1
8       7       1
Printing network:
1       2
1       3
1       7
1       8
2       3
2       4
2       8
3       4
3       5
4       5
4       6
5       6
5       7
6       7
6       8
7       8
```

The following is another version of the code that builds the same network:

Listing 3.2: code/core/NetworkBuild2.cpp

```
#include <linkpred.hpp>
#include <iostream>
using namespace LinkPred;
int main() {
  int n = 8;
  UNetwork<unsigned int> net;
  for (int i = 1; i <= n; i++) {
    net.addEdge(net.addNode(i).first, net.addNode(i % n + 1).
        first);
    net.addEdge(net.addNode(i).first, net.addNode((i + 1) % n +
        1).first);
  }
  net.assemble();
  std::cout << "Printing␣network:" << std::endl;
  net.print();
  return 0;
```

```
}
```

■

3.1.2 Accessing nodes

Nodes can be accessed through iterators provided by `nodesBegin()` and `nodesEnd()`. The order of iteration is that of internal IDs, which is also the order of insertion. For convenience, the iterator points to a pair, the first element of which is the internal ID, whereas the second is the external label.

```
std::cout << "ID\tLabel" << std::endl;
for (auto it = net.nodesBegin(); it != net.nodesEnd(); ++it) {
std::cout << it->first << "\t" << it->second << std::endl;
}
```

Alternatively, one can iterate over labels (in increasing order) in a similar way using the iterators `labelsBegin()` and `labelsEnd()`:

```
std::cout << "Label\tID" << std::endl;
for (auto it = net.labelsBegin(); it != net.labelsEnd(); ++it) {
std::cout << it->first << "\t" << it->second << std::endl;
}
```

It is also possible to translate labels to IDs and vice versa using `getID(label)` and `getLabel(id)` respectively.

Oftentimes, one would want to iterate over a random sample of nodes instead of the whole set. This can be easily done using the two methods:

```
RndNodeIt rndNodesBegin(double ratio, long int seed) const
RndNodeIt rndNodesEnd() const
```

The method `rndNodesBegin` takes two parameters: the ratio of nodes contained in the sample (must be in $[0,1]$) and a seed for the random number generator. For example, the following for loop iterates over about half the nodes and skips the other half. The nodes are accessed in increasing order of their IDs:

```
double ratio = 0.5;
long int seed = 777;
std::cout << "ID\tLabel" << std::endl;
for (auto it = net.rndNodesBegin(ratio, seed); it != net.
    rndNodesEnd(); ++it) {
std::cout << it->first << "\t" << it->second << std::endl;
}
```

R Notice that `ratio` specifies the probability that a node gets selected. Because of the random nature of the selection process, the actual number of nodes selected may be different from `ratio` $\times n$.

The methods above can be used to access nodes data even before the networks is assembled. After assembling the network, more functionalities become available. For instance, it is possible to access nodes degrees:

```
std::cout << "ID\tDegree" << std::endl;
for (auto it = net.nodesDegBegin(); it != net.nodesDegEnd(); ++it
   ) {
std::cout << it->first << "\t" << it->second << std::endl;
}
```

The iterator returned by `nodesDegBegin()` points to a pair where the first element is the node ID and the second element is its degree. It is also possible to obtain the degree of a given node using `getDeg(id)` :

```
std::cout << "ID\tDegree" << std::endl;
for (auto it = net.nodesBegin(); it != net.nodesEnd(); ++it) {
std::cout << it->first << "\t" << net.getDeg(it->first) << std::
   endl;
}
```

3.1.3 Accessing edges

Information on edges can only be accessed after assembling the network. One way to access edges is to iterate over all edges in the network. This can be done using the method `edgesBegin()` and `edgesEnd()` . Obtaining the start and end nodes of an edge is accomplished by means of the two **static** methods `start` and `end` :

```
std::cout << "Start\tEnd" << std::endl;
for (auto it = net.edgesBegin(); it != net.edgesEnd(); ++it) {
std::cout << net.start(*it) << "\t" << net.end(*it) << std::endl;
}
```

As it is the case with nodes, it is possible to access a random sample of edges:

```
double ratio = 0.5;
long int seed = 777;
std::cout << "Start\tEnd" << std::endl;
for (auto it = net.rndEdgesBegin(ratio, seed); it != net.
   rndEdgesEnd(); ++it) {
std::cout << net.start(*it) << "\t" << net.end(*it) << std::endl;
}
```

LinkPred offers the possibility to iterate over negative links in the same way one iterates over positive edges. This can be done using the method `nonEdgesBegin()` and `nonEdgesEnd()` :

```
std::cout << "Start\tEnd" << std::endl;
for (auto it = net.nonEdgesBegin(); it != net.nonEdgesEnd(); ++it
   ) {
std::cout << net.start(*it) << "\t" << net.end(*it) << std::endl;
}
```

It is also possible to iterate over a randomly selected sample of negative links:

```
double ratio = 0.5;
long int seed = 777;
std::cout << "Start\tEnd" << std::endl;
for (auto it = net.rndNonEdgesBegin(ratio, seed); it != net.
   rndNonEdgesEnd(); ++it) {
std::cout << net.start(*it) << "\t" << net.end(*it) << std::endl;
}
```

Negative edges are not stored in memory for obvious performance reasons. As a result, instead of $O(1)$ in the case of positive edges iterators, the incrementation operator (`++`) for negative links iterators has a higher running time, which depends on the network density.

The neighbors of a given node can be accessed by means of the two methods `neighbBegin(id)` and `net.neighbEnd(id)`:

```
unsigned int i = 0;
std::cout << "Start\tEnd" << std::endl;
for (auto it = net.neighbBegin(i); it != net.neighbEnd(i); ++it)
    {
std::cout << net.start(*it) << "\t" << net.end(*it) << std::endl;
}
```

Notice that the iterator points to the edges adjacent to the node and not directly to its neighbors. The neighbors are always located at the end of these edges, whereas the node passed to `neighbBegin` is stored as the starting node.

■ **Example 3.2** Consider the network shown in Figure 3.3.

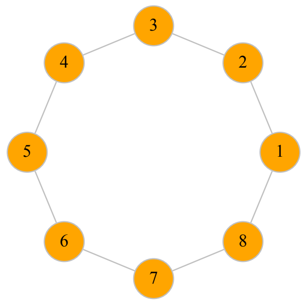

Figure 3.3: Example network.

The following code iterates over the neighbors of all nodes and a random sample of negative links:

Listing 3.3: code/core/EdgeItExample.cpp

```
#include <linkpred.hpp>
#include <iostream>
using namespace LinkPred;
int main() {
  int n = 8;
  UNetwork<unsigned int> net;
  for (int i = 1; i <= n; i++) {
    net.addEdge(net.addNode(i).first, net.addNode(i % n + 1).
       first);
  }
  net.assemble();

  std::cout << "Positive␣links:" << std::endl;
  std::cout << "Start\tEnd" << std::endl;
  for (auto it = net.nodesDegBegin(); it != net.nodesDegEnd(); ++
     it) {
```

```
    for (auto nit = net.neighbBegin(it->first); nit != net.
        neighbEnd(it->first); ++nit) {
      std::cout << net.getLabel(net.start(*nit)) << "\t" << net.
          getLabel(net.end(*nit)) << std::endl;
    }
  }

  std::cout << "Random negative links:" << std::endl;
  double ratio = 0.2;
  long int seed = 777;
  std::cout << "Start\tEnd" << std::endl;
  for (auto it = net.rndNonEdgesBegin(ratio, seed); it != net.
      rndNonEdgesEnd(); ++it) {
    std::cout << net.getLabel(net.start(*it)) << "\t" << net.
        getLabel(net.end(*it)) << std::endl;
  }
  return 0;
}
```

The following is the output of this code:

```
Positive links:
Start   End
2       1
2       3
1       2
1       8
3       2
3       4
4       3
4       5
5       4
5       6
6       5
6       7
7       6
7       8
8       1
8       7
Random negative links:
Start   End
2       4
1       3
3       7
3       8
4       8
```

■

3.2 The directed network data structure

To represent directed networks, LinkPred offers the class `DNetwork`, which offers a very similar interface to `UNetwork`.

■ **Example 3.3** For example, the code below shows how to create the directed network shown in Figure 3.4 and iterate over the neighbors of all nodes as well as a random

sample of negative links:

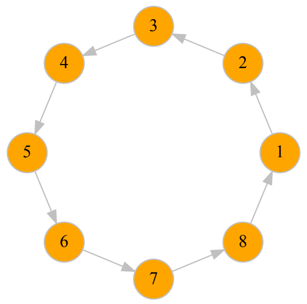

Figure 3.4: Example of a directed network.

Listing 3.4: code/core/DEdgeItExample.cpp

```
#include <linkpred.hpp>
#include <iostream>
using namespace LinkPred;
int main() {
  int n = 8;
  DNetwork<unsigned int> net;
  for (int i = 1; i <= n; i++) {
    net.addEdge(net.addNode(i).first, net.addNode(i % n + 1).
        first);
  }
  net.assemble();

  std::cout << "Positive␣links:" << std::endl;
  std::cout << "Start\tEnd" << std::endl;
  for (auto it = net.nodesDegBegin(); it != net.nodesDegEnd(); ++
      it) {
    for (auto nit = net.neighbBegin(it->first); nit != net.
        neighbEnd(it->first); ++nit) {
      std::cout << net.getLabel(net.start(*nit)) << "\t" << net.
          getLabel(net.end(*nit)) << std::endl;
    }
  }

  std::cout << "Random␣negative␣links:" << std::endl;
  double ratio = 0.2;
  long int seed = 777;
  std::cout << "Start\tEnd" << std::endl;
  for (auto it = net.rndNonEdgesBegin(ratio, seed); it != net.
      rndNonEdgesEnd(); ++it) {
    std::cout << net.getLabel(net.start(*it)) << "\t" << net.
        getLabel(net.end(*it)) << std::endl;
  }
  return 0;
}
```

The following is the output of this code:

```
Positive links:
```

```
Start   End
2       3
1       2
3       4
4       5
5       6
6       7
7       8
8       1
Random negative links:
Start   End
2       1
2       8
3       2
3       1
3       7
5       2
5       8
7       6
8       2
```

■

3.3 Maps

Maps are a useful way to associate data to nodes or edges. Two types of maps are available in LinkPred: *node maps* (class `NodeMap`) and *edge maps* (class `EdgeMap`), both member of `UNetwork` and `DNetwork`. The first assigns data to the nodes of the network, whereas the latter maps data to edges.

Creating a node map is achieved by calling the method `createNodeMap` on the network object. This is a template method with the mapped data type as the only template argument. For example, to create a node map with data type `double` over the network `net`:

```
auto nodeMap = net.template createNodeMap<double>();
```

To obtain a smart pointer (`std::shared_ptr`) to a node map, the method `createNodeMapSP` must be called instead:

```
auto nodeMapSP = net.template createNodeMapSP<double>();
```

Creating an edge map can be done in a similar way:

```
auto edgeMap = net.template createEdgeMap<double>();
auto edgeMapSP = net.template createEdgeMapSP<double>();
```

Both `NodeMap` and `EdgeMap` offer the same interface, which in fact is similar to `std::map`. This includes the operator `[]`, the methods `at`, `begin`, `end`, `cbegin` and `cend`. From the performance point of view, `NodeMap` offers constant time access to mapped values, whereas `EdgeMap` requires logarithmic time access ($O(\log m)$, m being the number of edges).

■ **Example 3.4** The following code shows how to create and use node and edge maps:

Listing 3.5: code/core/MapExample.cpp

```
#include <linkpred.hpp>
#include <iostream>
using namespace LinkPred;
int main() {
  int n = 8;
  UNetwork<unsigned int> net;
  for (int i = 1; i <= n; i++) {
    net.addEdge(net.addNode(i).first, net.addNode(i % n + 1).
        first);
    net.addEdge(net.addNode(i).first, net.addNode((i + 1) % n +
        1).first);
  }
  net.assemble();

  int i = 0;
  auto nodeMap = net.template createNodeMap<double>();
  for (auto it = net.nodesBegin(); it != net.nodesEnd(); ++it) {
    nodeMap[it->first] = i++ / 2.0;
  }

  std::cout << "ID\tValue" << std::endl;
  for (auto it = net.nodesBegin(); it != net.nodesEnd(); ++it) {
    std::cout << it->second << "\t" << nodeMap.at(it->first) <<
        std::endl;
  }

  i = 0;
  auto edgeMap = net.template createEdgeMap<double>();
  for (auto it = net.edgesBegin(); it != net.edgesEnd(); ++it) {
    edgeMap[*it] = i++ / 2.0;
  }

  std::cout << "Start\tEnd\tValue" << std::endl;
  for (auto it = net.edgesBegin(); it != net.edgesEnd(); ++it) {
    std::cout << net.getLabel(net.start(*it)) << "\t" << net.
        getLabel(net.end(*it)) << "\t" << edgeMap.at(*it) << std::
        endl;
  }
  return 0;
}
```

The following is the output of this code:

```
ID      Value
2       0
1       0.5
3       1
4       1.5
5       2
6       2.5
7       3
8       3.5
Start   End     Value
2       1       0
2       3       0.5
```

```
2       4       1
2       8       1.5
1       3       2
1       7       2.5
1       8       3
3       4       3.5
3       5       4
4       5       4.5
4       6       5
5       6       5.5
5       7       6
6       7       6.5
6       8       7
7       8       7.5
```

■

3.3.1 Sparse maps

If a node map is sparse, that is, has non-default values only on a small subset of the elements, it is better to use sparse node and edge maps. To create a sparse node map:

```
auto nodeSMap = net.template createNodeSMap<double>(0.0);
```

Notice that the methods takes as input one parameter that specifies the default value of the map (in this case, it is 0.0). Hence, in this example any node which is not explicitly assigned a value is assumed to have the default value 0.0. To obtain a smart pointer (`std::shared_ptr`) to a sparse node map, the method `createNodeSMapSP` must be called instead:

```
auto nodeSMapSP = net.template createNodeSMapSP<double>(0.0);
```

Sparse maps use $O(\log(k))$ space and time to sore and access data, where k is the number of elements explicitly assigned elements (having non-default value).

> **R** Any element that is explicitly assigned, even with the default value, is stored in memory. Hence, you should avoid explicit assignment with the default value as it unnecessarily increases the size of the map.

■ **Example 3.5** The following code shows how to create and use a sparse node map:

Listing 3.6: code/core/SMapExample.cpp

```
#include <linkpred.hpp>
#include <iostream>
using namespace LinkPred;
int main() {
  int n = 8;
  UNetwork<unsigned int> net;
  for (int i = 1; i <= n; i++) {
    net.addEdge(net.addNode(i).first, net.addNode(i % n + 1).
        first);
    net.addEdge(net.addNode(i).first, net.addNode((i + 1) % n +
        1).first);
  }
```

```
  net.assemble();

  auto nodeSMap = net.template createNodeSMap<double>(-1.0);
  nodeSMap[2] = 2.0;
  nodeSMap[3] = 3.0;

  std::cout << "ID\tValue" << std::endl;
  for (auto it = net.nodesBegin(); it != net.nodesEnd(); ++it) {
    std::cout << it->second << "\t" << nodeSMap.at(it->first) <<
        std::endl;
  }
  return 0;
}
```

The following is the output of this code:

```
ID      Value
2       -1
1       -1
3       2
4       3
5       -1
6       -1
7       -1
8       -1
```

■

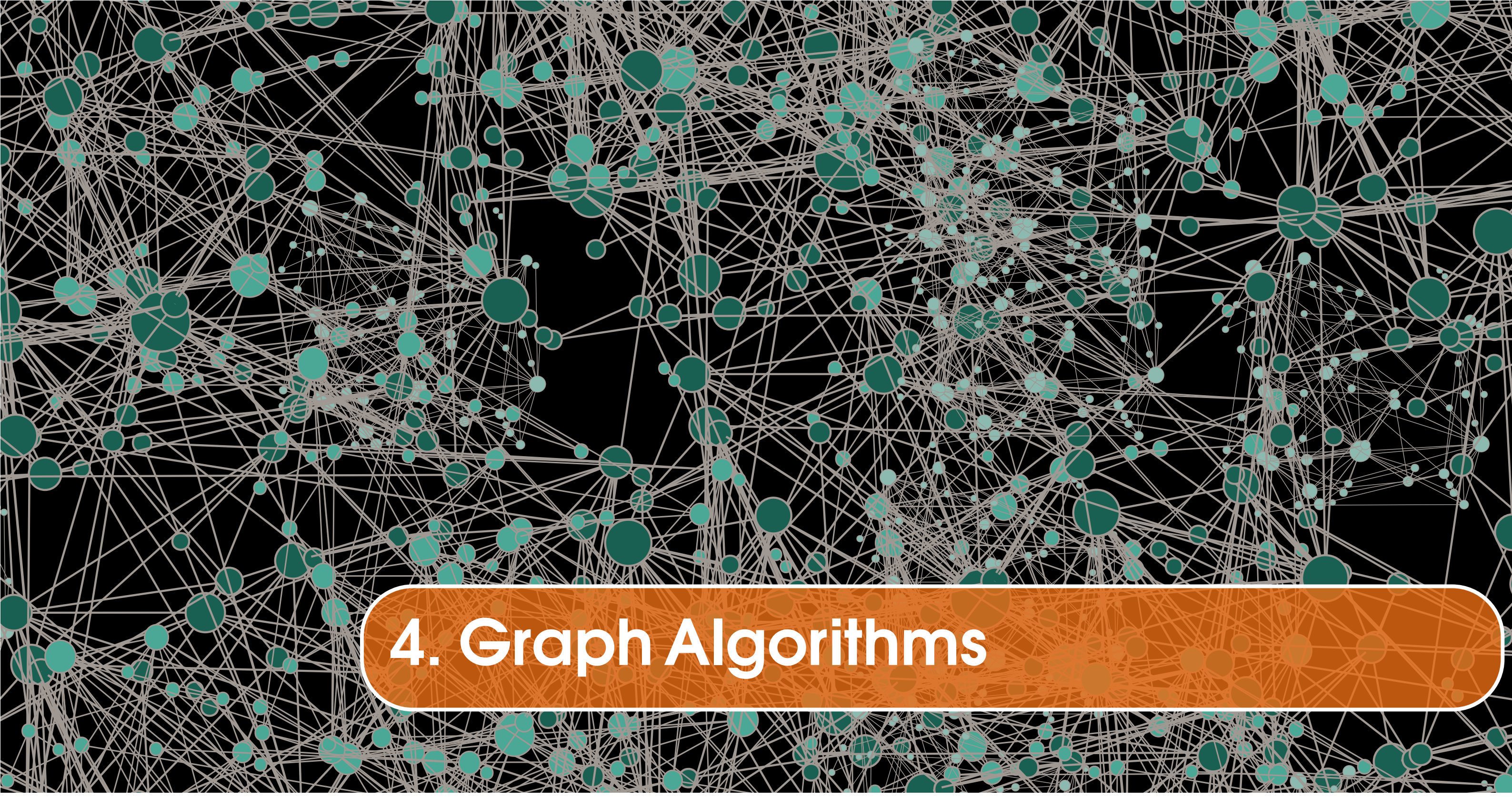

4. Graph Algorithms

4.1 Graph traversal

LinkPred provides two classes for graph traversal: `BFS`, for Breadth First traversal, and `DFS` for Depth First traversal. They both inherit from the abstract class `GraphTraversal`, which declares one virtual method `traverse`. It takes as parameter the source node, from where the traversal starts, and a reference to a `NodeProcessor` object which is in charge of processing nodes sequentially as they are visited.

Listing 4.1: code/graphalg/GraphTraversal.hpp

```
/**
 * Abstract graph traversal.
 */
template<typename Network = UNetwork<>, typename NodeProcessor =
    Collector<Network>> class GraphTraversal {
protected:
  std::shared_ptr<Network const> net;
public:
  GraphTraversal(std::shared_ptr<Network const> net) : net(net) {
  }
...
  /**
   * Traverse the graph.
   * @param srcNode The source node.
   * @param processor The node processor.
   */
  virtual void traverse(typename Network::NodeID srcNode,
      NodeProcessor & processor) = 0;
};
```

The class `NodeProcessor` is a template argument of `GraphTraversal` and is required to implement the method `bool process(typename Network::NodeID const & i)`, which processes node *i* and returns true if the traversal must continue, false otherwise. Notice, however, that independently of the return value of `process`, only the nodes in the same

connected component as the source node are visited by `BFS` and `DFS`.

The library offers two useful implementations of `NodeProcessor`: `Counter`, which simply counts the visited nodes, and `Collector`, which collects the visited nodes' IDs into a queue in the order of their visit. `Collector` is the default value for the template argument `NodeProcessor`. The two classes `Counter` and `Collector` are shown below.

Listing 4.2: code/graphalg/Counter.hpp

```
/**
 * A class that counts nodes during traversal.
 */
template<typename Network = Core::UNetwork<>> class Counter {
protected:
  std::size_t count = 0;
public:
...
  /**
   * Node processing.
   */
  bool process(typename Network::NodeIdType const & i) {
    count++;
    return true;
  }

  /**
   * @return The nodes count.
   */
  std::size_t getCount() const {
    return count;
  }

  /**
   * Reset the nodes count to 0.
   */
  void resetCount() {
    count = 0;
  }
};
```

Listing 4.3: code/graphalg/Collector.hpp

```
/**
 * A class that collects nodes during traversal.
 */
template<typename Network = Core::UNetwork<>> class Collector {
protected:
  std::queue<typename Network::NodeIdType> visited;
public:
...
  /**
   * Node processing.
   */
  bool process(typename Network::NodeIdType const & i) {
    visited.push(i);
```

```
    return true;
  }

  /**
   * @return The visited nodes.
   */
  const std::queue<typename Network::NodeIdType>& getVisited()
      const {
    return visited;
  }
};
```

■ **Example 4.1** Consider the network shown in Figure 4.1.

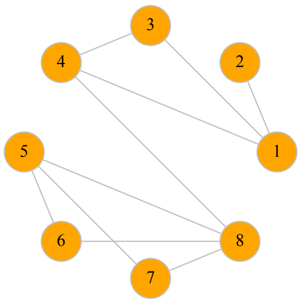

Figure 4.1: Example network.

The code below shows how to traverse this graph using BFS and DFS classes. For BFS, we collect the nodes, whereas for DFS we only count them.

Listing 4.4: code/graphalg/TraversalExample.cpp

```
#include <linkpred.hpp>
#include <iostream>
using namespace LinkPred;

int main() {
  auto net = UNetwork<>::read("net-traversal.edges");
  // BFS
  BFS<> bfs(net);
  Collector<> col;
  bfs.traverse(net->getID("1"), col);
  auto visited = col.getVisited();
  std::cout << "BFS:" << std::endl;
  while (!visited.empty()) {
    auto i = visited.front();
    visited.pop();
    std::cout << net->getLabel(i) << std::endl;
  }
  // DFS
  DFS<UNetwork<>, Counter<>> dfs(net);
  Counter<> counter;
  dfs.traverse(net->getID("1"), counter);
  std::cout << "DFS␣visited␣" << counter.getCount() << "␣nodes"
      << std::endl;
```

```
  return 0;
}
```

Here is the output of this code:

```
BFS:
1
2
3
4
8
5
6
7
DFS visited 8 nodes
```

■

4.2 Shortest paths

The LinkPred library contains an implementation of Dijkstra's algorithm for solving the shortest path problem[1]. To use it, it is first necessary to define a length (or weight) map that specifies the length associated with every edge in the graph. A length map is simply a map over the set of edges which can take integer as well as double values. It can therefore be created using the template methods `createEdgeMap()` and `createEdgeMapSP()` defined in the class `UNetwork` (see Section 3.3). The method `createEdgeMap` returns an `EdgeMap` object, whereas `createEdgeMapSP` returns a smart pointer (`std::shared_ptr`) to an `EdgeMap` object. For example, in order to create a length map taking double values, one can proceed as follows:

Listing 4.5: code/graphalg/CreateLengthMap.cpp

```
auto length = net->template getEdgeMapSP<double>();
for (auto it = net->edgesBegin(); it != net->edgesEnd(); ++it) {
  (*length)[*it] = 1;
}
```

The next step is to create a `Dijkstra` object and register the length map:

Listing 4.6: code/graphalg/RegisterLengthMap.cpp

```
Dijkstra<> dijkstra(net);
auto lengthMapId = dijkstra.registerLengthMap(length);
```

The identifier `lengthMapId` is used to uniquely identify the registered length map. It is possible to register multiple length maps with the same `Dijkstra` object, and once there is no more need for a given length map, it can be unregistered as follows:

Listing 4.7: code/graphalg/UnregisterLengthMap.cpp

```
dijkstra.unregisterLengthMap(lengthMapId);
```

[1]The current implementation uses a binary heap instead of a Fibonacci heap. Consequently, the performance of the implementation is $O(nm+n^2\log^2 m)$ instead of $O(nm+n^2\log m)$.

The class `Dijkstra` offers two methods for computing distances:

1. `getShortestPath`: This method computes and returns the shortest path between two nodes and its length:

Listing 4.8: code/graphalg/ShortestPathExample.cpp

```
auto res = dijkstra.getShortestPath(i, j, lengthMapId);
auto path = res.first; // This is the shortest path
auto dist = res.second; // This is its length
```

2. `getDist`: Computes the distance between a source node and all other nodes. The returned value is a node map, where each node is mapped to a pair containing the distance from the source node and the number of edges in the corresponding shortest path:

Listing 4.9: code/graphalg/DistExample.cpp

```
auto distMap = dijkstra.getDist(i, lengthMapId);
auto res = distMap->at(j);
double dist = res.first; // Distance between i and j
std::size_t nbHops =  res.second; // Number of hops along the
    shortest path
```

Both methods run Dijkstra's algorithm, except that `getShortestPath` stops once the destination node is reached, whereas `getDist` continues until all reachable nodes are visited. The distance and number of hops assigned to disconnected couples are passed as the last two arguments of these two methods. By default, they are assigned the values `std::numeric_limits<double>::infinity()` and `std::numeric_limits<std::size_t>::max()` respectively.

■ **Example 4.2** Consider the network shown in Figure 4.2.

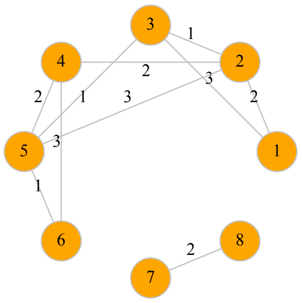

Figure 4.2: Example network with an associated length map.

In the following code, we first compute the shortest path from 1 to 6, then compute the shortest path distances from 1 to all other nodes.

Listing 4.10: code/graphalg/ShortestPathFullExample.cpp

```
#include <linkpred.hpp>
#include <iostream>
using namespace LinkPred;
```

```
int main() {
  auto net = UNetwork<>::read("net-sp.edges");
  auto length = net->template createEdgeMapSP<double>();
  int i = 1;
  for (auto it = net->edgesBegin(); it != net->edgesEnd(); ++it,
     i++) {
    (*length)[*it] = (13 * i) % 3 + 1;
  }
  Dijkstra<> dijkstra(net);
  auto lengthMapId = dijkstra.registerLengthMap(length);
  {
    auto res = dijkstra.getShortestPath(net->getID("1"), net->
       getID("6"), lengthMapId);
    auto path = res.first;
    auto dist = res.second;
    std::cout << "Path: ";
    for (auto it = path->begin(); it != path->end(); ++it) {
      std::cout << net->getLabel(*it) << " ";
    }
    std::cout << "\ndist: " << dist << std::endl;
  }
  {
    auto distMap = dijkstra.getDist(net->getID("1"), lengthMapId)
       ;
    std::cout << "dist: " << std::endl;
    for (auto it = net->nodesBegin(); it != net->nodesEnd(); ++it
       ) {
      auto res = distMap->at(it->first);
      std::cout << it->second << " : " << res.first << ", " <<
         res.second << std::endl;
    }
  }
  return 0;
}
```

Here is the output of this code:

```
Path: 1 2 4 6
dist: 5
dist:
1 : 0, 0
2 : 2, 1
3 : 3, 1
4 : 4, 2
5 : 4, 2
6 : 5, 3
7 : inf, 18446744073709551615
8 : inf, 18446744073709551615
```

■

4.2.1 Memory management

Computing shortest-path distances in large networks require not only considerable time but also significant space resources. Consequently, efficient management of memory is necessary to render the task feasible in such situations. The abstract class `NetDistCalculator` provides an interface for an additional layer over the class `Dijkstra`

which facilitates its use and can serve to manage memory usage. A `NetDistCalculator` object is associated with a single length map and provides two methods for computing distances:

- `getDist(i, j)` : Computes and returns the distance between the two nodes *i* and *j*. The return value is an `std::pair`, with the first element being the distance, whereas the second is the number of hops in the shortest path joining the two nodes.
- `getDist(i)` : Computes and returns a node map containing the distances from node *i* to all other nodes in the network.

LinkPred includes two implementations of `NetDistCalculator` : `ESPDistCalculator` (exact shortest path distance calculator) and `ASPDistCalculator` (approximate shortest path distance calculator). In what follows, a description of the former is given, whereas the latter implementation is presented in the next section (4.2.2).

The class `ESPDistCalculator` implements the interface `NetDistCalculator` and returned the exact shortest path distances as computed by `Dijkstra`. Additionally, it caches the computed results for better performance. The constructor of `ESPDistCalculator` takes three parameters: a `Dijkstra` object, a length map and a third parameter of type `CacheLevel`, which is an enumeration of the available caching strategies:

- `NoCache` : The results computed by `Dijkstra` are discarded immediately after the call to the method has ended. This minimizes memory consumption, but is very inefficient from the time perspective. This strategy should only be used when memory is scarce and the couples for which the distance is to be computed are few in number and have a few or no nodes in common.
- `NodeCache` : In this strategy, the distances from a single node to all other nodes (a distance node map) are kept in cache and replaced in case of a cache miss. Moderate memory use is incurred from using this scheme, and if the couples between which the distances to be computed are grouped according to their starting or ending node, time requirements are optimal in the sens that no results are wasted. Therefore, this strategy should be used with large network with the precaution of ordering couples as explained.
- `NetworkCache` : In this scheme, any computed distance map is kept in cache, which results in maximum memory consumption and minimal computation time. This should be with small to average-size networks.

■ **Example 4.3** In the following code, we compute the distance between all couples in the network of Figure 4.2.

Listing 4.11: code/graphalg/NetDistCalculatorExample.cpp

```
#include <linkpred.hpp>
#include <iostream>
using namespace LinkPred;

int main() {
  auto net = UNetwork<>::read("net-sp.edges");
  auto length = net->template createEdgeMapSP<double>();
  int i = 1;
  for (auto it = net->edgesBegin(); it != net->edgesEnd(); ++it,
      i++) {
```

```
    (*length)[*it] = (13 * i) % 3 + 1;
  }
  Dijkstra<> dijkstra(net);
  ESPDistCalculator<> calc(dijkstra, length, NetworkCache);
  std::cout << "Src\tDst\tDist" << std::endl;
  for (auto sit = net->nodesBegin(); sit != net->nodesEnd(); ++
     sit) {
    for (auto dit = sit + 1; dit != net->nodesEnd(); ++dit) {
      std::cout << sit->second << "\t" << dit->second << "\t" <<
         calc.getDist(sit->first, dit->first).first << std::endl;
    }
  }
  return 0;
}
```

The output of this code is as follows:

```
Src     Dst     Dist
1       2       2
1       3       3
1       4       4
1       5       4
1       6       5
1       7       inf
1       8       inf
2       3       1
2       4       2
2       5       2
2       6       3
2       7       inf
2       8       inf
3       4       3
3       5       1
3       6       2
3       7       inf
3       8       inf
4       5       2
4       6       3
4       7       inf
4       8       inf
5       6       1
5       7       inf
5       8       inf
6       7       inf
6       8       inf
7       8       2
```

■

4.2.2 Approximate shortest path distances

Computing exact distances in very large networks can be time consuming, and resorting to approximations may be necessary. `ASPDistCalculator` is an implementation of `NetDistCalculator` that computes approximate shortest path distances of exact ones. The approximation works as follows. A set $\mathscr{L}$ of nodes called *landmarks* is selected, and the distance from each landmark to all other nodes is pre-computed and stored in

memory. The distance between any two nodes i, j is then approximated by:

$$d_{ij} \simeq \min_{k \in \mathscr{L}} [d_{ik} + d_{kj}]. \quad (4.1)$$

The landmarks are passed to `ASPDistCalculator` object using the method `setLandmarks`. Of course, by increasing the number of landmarks, more precision can be obtained, be it though at a higher computational and memory cost. The choice of the landmarks is left to the user.

■ **Example 4.4** In the following code, we compute the approximate distances between all couples in the network of Figure 4.2 using 30% of nodes as landmarks.

Listing 4.12: code/graphalg/ASPDistCalculatorExample.cpp

```
#include <linkpred.hpp>
#include <iostream>
using namespace LinkPred;

int main() {
  auto net = UNetwork<>::read("net-sp.edges");
  auto length = net->template createEdgeMapSP<double>();
  int i = 1;
  for (auto it = net->edgesBegin(); it != net->edgesEnd(); ++it,
     i++) {
    (*length)[*it] = (13 * i) % 3 + 1;
  }
  Dijkstra<> dijkstra(net);
  ASPDistCalculator<> calc(dijkstra, length);
  double landmarkRatio = 0.3;
  long int seed = 777;
  std::vector<typename UNetwork<>::NodeID> landmarks;
  std::cout << "Landmarks:" << std::endl;
  for (auto it = net->rndNodesBegin(landmarkRatio, seed); it !=
     net->rndNodesEnd(); ++it) {
    landmarks.push_back(it->first);
    std::cout << it->second << std::endl;
  }
  calc.setLandmarks(landmarks.begin(), landmarks.end());
  std::cout << "Src\tDst\tDist" << std::endl;
  for (auto sit = net->nodesBegin(); sit != net->nodesEnd(); ++
     sit) {
    for (auto dit = sit + 1; dit != net->nodesEnd(); ++dit) {
      std::cout << sit->second << "\t" << dit->second << "\t" <<
          calc.getDist(sit->first, dit->first).first << std::endl;
    }
  }
  return 0;
}
```

The output of this code is as follows:

```
Landmarks:
1
4
Src     Dst     Dist
1       2       2
```

```
1       3       3
1       4       4
1       5       4
1       6       5
1       7       inf
1       8       inf
2       3       5
2       4       2
2       5       4
2       6       5
2       7       inf
2       8       inf
3       4       3
3       5       5
3       6       6
3       7       inf
3       8       inf
4       5       2
4       6       3
4       7       inf
4       8       inf
5       6       5
5       7       inf
5       8       inf
6       7       inf
6       8       inf
7       8       inf
```

■

4.3 Graph embedding

Graph embedding consists in transforming the graph's nodes and edges into elements of a low-dimensional vector space while preserving, as much as possible, its structural properties [10]. It is a problem with important applications in various fields, including link prediction [2, 10, 15], product recommendation [17], data visualization [22, 30], and node classification [5, 31]. Several approaches for graph embedding have been proposed in the literature. These include methods based on matrix decomposition, such as locally linear embedding [27], Laplacian eigenmaps [4], and matrix factorization [17] (also referred to as graph factorization in [1, 10]); methods based on random walks, such as DeepWalk [25], LINE [29] and Node2Vec [11]; and deep learning-based methods [6, 32].

LinkPred contains the implementation of the following graph embedding techniques:

1. **DeepWalk** [25]: This algorithm is implemented in the class `DeepWlak` based on the code available at `https://github.com/xgfs/deepwalk-c`.
2. **Hidden Metric Space Model (HMSM)** [2]: This algorithm is implemented in the class `HMSM`.
3. **LargeVis** [30]: This algorithm is implemented in the class `LargeVis` based on the code available at `https://github.com/lferry007/LargeVis`.
4. **Laplacian Eigenmaps (LEM)** [4]: This algorithm is implemented in the class `LEM` (this encoder requires compilation with Armadillo library, that the option

`LINKPRED_WITH_ARMADILLO` must be on).

5. **Large Information Networks Embedding (LINE)** [29]: This algorithm is implemented in the class `LINE` based on the code available at https://github.com/tangjianpku/LINE.
6. **Locally Linear Embedding (LLE)** [27]: This algorithm is implemented in the class `LLE` (this encoder requires compilation with Armadillo library, that the option `LINKPRED_WITH_ARMADILLO` must be on).
7. **Matrix Factorization** [17]: This algorithm is implemented in the class `MatFact`.
8. **Node2Vec** [11]: This algorithm is implemented in the class `LargeVis` based on the code available at https://github.com/xgfs/node2vec-c.

4.3.1 The Encoder interface

To provide a uniform interface, all encoders inherits from the abstract class `Encoder`:

Listing 4.13: code/graphalg/encoder.hpp

```
template<...> class Encoder {
public:
  // Return the dimension of the embedding.
  int getDim() const;
  // Set the dimension of the embedding.
  void setDim(int dim);
  // Initialize encoder.
  virtual void init() = 0;
  // Encode the network.
  virtual void encode() = 0;
  // Return the code of given node.
  Vec getNodeCode(NodeID const &i);
  // Return the code of an edge.
  Vec getEdgeCode(Edge const &e);
  // Return the dimension of the edge emebedding.
  virtual int getEdgeCodeDim() const;
  ...
};
```

First, the method `init` is called to initialize the internal data structures of the encoder. Once the encoder is initialized, the method `encode` can be called to perform the embedding. This step typically involves solving an optimization problem, which can be computationally intensive both in terms of memory and CPU usage, especially for very large networks. The dimension of the embedding space can be queried and set using `getDim` and `setDim` respectively.

The node embedding or the node code, which is the vector of coordinates assigned to the node, can be obtained by calling the method `getNodeCode`. The edge code is by default the concatenation of the two nodes' codes and can be obtained using `getEdgeCode`. Hence, in the default case, the edge code dimension is double that of a node. Classes that implement the `Encoder` may change this default behavior if necessary. The user can query the dimension of the edge code using the method `getEdgeCodeDim`.

4.3.2 Examples

This is an example of using `Node2Vec` to embed a network:

Listing 4.14: code/graphalg/node2vec.cpp

```
#include <linkpred.hpp>
#include <iostream>
using namespace LinkPred;

int main() {
  long int seed = 777;
  // Read the network
  auto net = UNetwork<>::read("Zakarays_Karate_Club.edges");
  // Create a Node2Vec encoder
  Node2Vec<> encoder(net, seed);
  // Set the dimension to 5
  encoder.setDim(5);
  // Initialize the encoder
  encoder.init();
  // Embed the network
  encoder.encode();
  // Print the code of every node
  std::cout << std::fixed << std::setprecision(3);
  for (std::size_t i = 0; i < net->getNbNodes(); i++) {
    auto v = encoder.getNodeCode(i);
    std::cout << net->getLabel(i) << " :\t";
    for (int j = 0; j < v.size(); j++) {
      std::cout << v[j] << "\t";
    }
    std::cout << std::endl;
  }
  return 0;
}
```

The following is a partial listing of the output of this program:.

```
1 :     -0.424  -0.168  0.691   -1.523  -0.522
2 :     0.397   0.280   1.195   -1.148  0.161
3 :     0.191   -0.240  0.158   -1.099  0.835
4 :     0.118   -0.307  1.007   -1.339  0.243
5 :     -0.602  0.699   0.568   -1.668  -0.789
6 :     -0.847  1.162   0.490   -1.547  -1.016
7 :     -0.875  1.185   0.562   -1.605  -0.881
...
```

This is an example of using `HMSM` to embed a network:

Listing 4.15: code/graphalg/hmsm.cpp

```
#include <linkpred.hpp>
#include <iostream>
using namespace LinkPred;

int main() {
  long int seed = 777;
  // Read the network
  auto net = UNetwork<>::read("Zakarays_Karate_Club.edges");
  // Create a HMSM encoder
  HMSM<> encoder(net, seed);
  // Set the dimension to 3
```

```
  encoder.setDim(3);
  // Initialize the encoder
  encoder.init();
  // Embed the network
  encoder.encode();
  // Print the code of every node
  std::cout << std::fixed << std::setprecision(3);
  for (std::size_t i = 0; i < net->getNbNodes(); i++) {
    auto v = encoder.getNodeCode(i);
    std::cout << net->getLabel(i) << " :\t";
    for (int j = 0; j < v.size(); j++) {
      std::cout << v[j] << "\t";
    }
    std::cout << std::endl;
  }
  return 0;
}
```

The following is a partial listing of the output of this program:.

```
1 :     16.000  9.550    -8.157
2 :     9.000   19.015   -7.989
3 :     10.000  20.929   -7.757
4 :     6.000   19.985   -8.358
5 :     3.000   0.388    -13.094
6 :     4.000   -3.115   -15.756
7 :     4.000   -3.144   -16.056
...
```

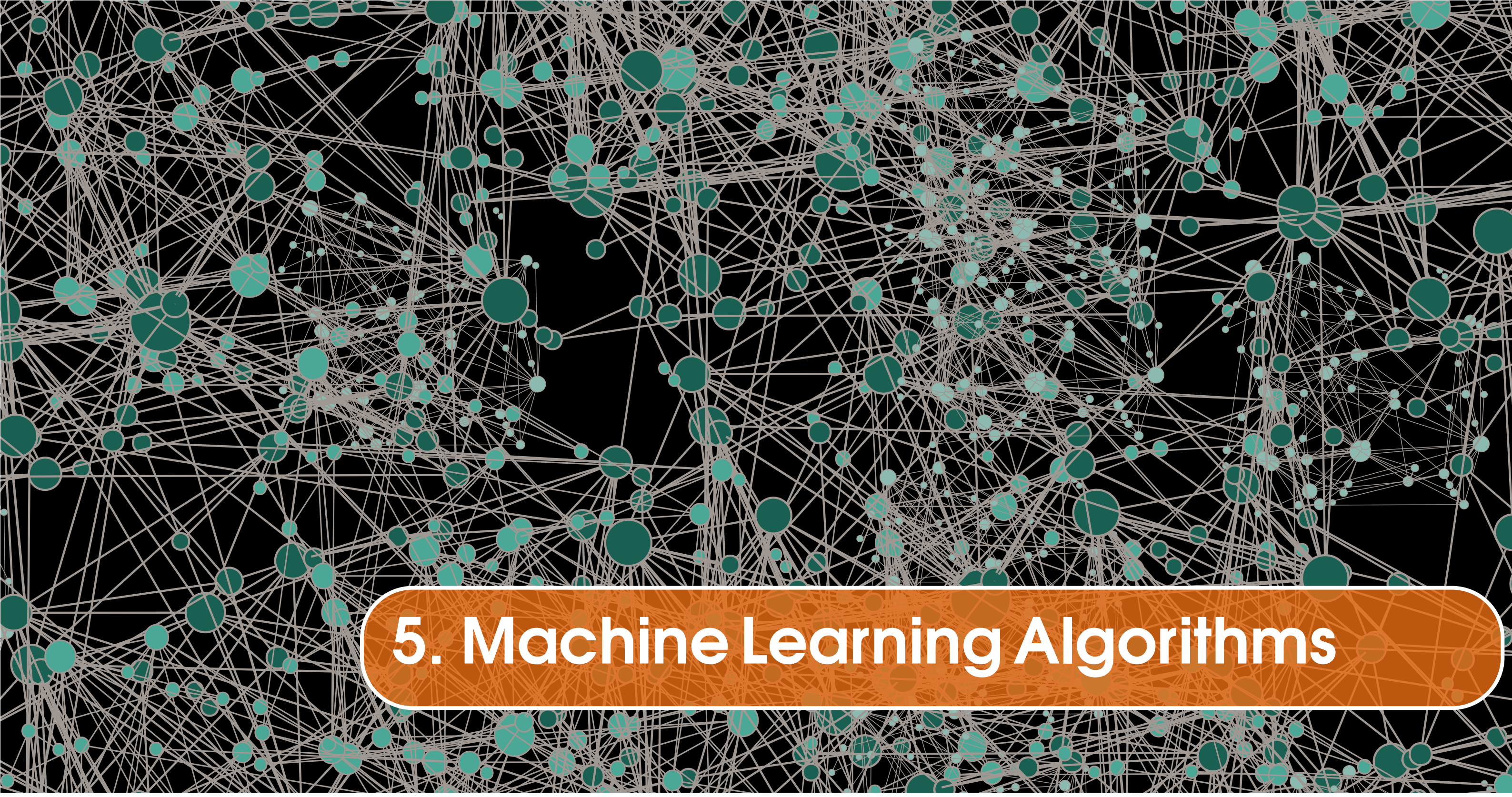

5. Machine Learning Algorithms

Classifiers

All binary classifiers in LinkPred implements the interface `Classifier`, which provides two important methods: the method `learn` which trains the classifier on a training set, and the method `predict` which predicts the output for a given input:

Listing 5.1: code/ml/classifier.hpp

```
template<...> class Classifier {
  /**
   * Learn from data.
   * @param trInBegin Iterator to the first example features (
     input).
   * @param trInEnd Iterator to one-past-the-last example
     features (input).
   * @param trOutBegin Iterator to the first example class (
     output).
   * @param trOutEnd Iterator to one-past-the-last example class
     (output).
   */
  virtual void learn(InRndIt trInBegin, InRndIt trInEnd, OutRndIt
     trOutBegin, OutRndIt trOutEnd) = 0;

  /**
   * Predict.
   * @param inBegin Iterator to the first instance features (
     input).
   * @param inEnd Iterator to one-past-the-last instance features
      (input).
   * @param scoresBegin Iterator to the first location where to
     store prediction scores. Memory must be pre-allocated.
   */
  virtual void predict(InRndIt inBegin, InRndIt inEnd, ScoreRndIt
     scoresBegin) = 0;

  /**
```

```
   * @return The name of the classifier.
   */
  const std::string& getName() const {
    return name;
  }

  /**
   * Set the name of the classifier.
   * @param name The new name of the classifier.
   */
  void setName(const std::string &name) {
    this->name = name;
  }
};
```

The following code shows an example of using the logistic regression classifier included with LinkPred.

> **R** The class `LogisticRegresser` is the only classifier "native" to LinkPred. The other classifiers inherit from mlpack classes and therefore require that LinkPred is compiled with mlpack (that is, the option `LINKPRED_WITH_MLPACK` is set to true, which is the default setting).

Listing 5.2: code/ml/logisticregresser.cpp

```
#include <linkpred.hpp>
#include <iostream>
using namespace LinkPred;
int main(int argc, char *argv[]) {
  // Training data
  std::vector<Vec> trnIn;
  trnIn.push_back({ 0.912145, 0.709983, 0.226475 });
  trnIn.push_back({ 0.934958, 0.123857, 0.802411 });
  trnIn.push_back({ 0.039990, 0.781305, 0.560989 });
  trnIn.push_back({ 0.322438, 0.241671, 0.637029 });
  trnIn.push_back({ 0.895175, 0.726442, 0.406118 });
  trnIn.push_back({ 0.140349, 0.068158, 0.488275 });
  trnIn.push_back({ 0.474313, 0.968052, 0.370530 });
  trnIn.push_back({ 0.437717, 0.953002, 0.371601 });
  trnIn.push_back({ 0.655664, 0.527321, 0.712499 });
  trnIn.push_back({ 0.123821, 0.552098, 0.846477 });
  std::vector<bool> trnOut = { 0, 1, 0, 1, 0, 1, 0, 0, 1, 0 };
  // Create a logistic regression classifier with rergularization
      coefficient lambda = 0.001 and seed = 777
  LogisticRegresser<> classifier(0.001, 777);
  // Train the classifier
  classifier.learn(trnIn.begin(), trnIn.end(), trnOut.begin(),
     trnOut.end());
  // Test data
  std::vector<Vec> tstIn;
  tstIn.push_back({ 0.85568, 0.36109, 0.86532 });
  tstIn.push_back({ 0.13094, 0.61792, 0.80714 });
  tstIn.push_back({ 0.61693, 0.47719, 0.67608 });
  tstIn.push_back({ 0.47321, 0.57101, 0.10932 });
```

```
  tstIn.push_back({ 0.73278, 0.19042, 0.70569 });
  std::vector<bool> tstOut = { 1, 0, 1, 0, 1 };
  // Predict output for test set
  std::vector<double> pred(tstIn.size());
  classifier.predict(tstIn.begin(), tstIn.end(), pred.begin());
  // Print results
  std::cout << "Predicted\tActual" << std::endl;
  for (int j = 0; j < 5; j++) {
    std::cout << pred[j] << "\t" << tstOut[j] << std::endl;
  }
  return 0;
}
```

This is the output of for this code:

```
Predicted       Actual
0.997813        1
0.058436        0
0.821603        1
0.00838614      0
0.998843        1
```

The general pattern for using a classifier is as follows:

```
std::vector<Vec> trnIn; // Training input
std::vector<bool> trnOut; // Training output
std::vector<Vec> tstIn; // Test input
// Fill or load data
...
// Train the classifier
classifier.learn(trnIn.begin(), trnIn.end(), trnOut.begin(),
   trnOut.end());
// Predict output for test set
std::vector<double> pred(tstIn.size());
classifier.predict(tstIn.begin(), tstIn.end(), pred.begin());
```

LinkPred contains the implementation of the following classifiers:

- **Logistic regression**: This classifier is implemented by the class `LogisticRegresser` and has two parameters, the regularization coefficient `lambda` and a seed for the random number generator. These two parameters are passed to the constructor of the class.

```
  // Create a logistic regression classifier with
     rergularization coefficient lambda = 0.001 and seed =
     777
  LogisticRegresser<> classifier(0.001, 777);
```

- **Feed-forward neural network (requires mlpack)**: This classifier is implemented in the class `FFN`, which inherits from `mlpack::ann::FFN<>`. The methods of the latter can be used to design the architecture of the network (see mlpack documentation). Alternatively, LinkPred provides the method `setAutoArch(int dim)`, which allows to create a default architecture with an input layer of size `dim`. The default architectures consists of a pipeline of blocks, each containing a linear layer (`mlpack::ann::Linear<>`) followed by a sigmoid layer (`mlpack::ann::SigmoidLayer<>`). The last block consists of a linear layer followed by a log softmax layer (mlpack::ann::LogSoftMax<>). The layers' dimension is divided by two at each block until it reaches two at the output layer.

```
// Create a feed-forward network and set its architecture
    autoimatically
FFN<> classifier;
classifier.setAutoArch(dim);
```

- **Linear SVM (requires mlpack)**: This classifier is implemented in the class `LinearSVM`, which inherits from the class `mlpack::svm::LinearSVM<>`. All parameters of this classifier can be set using the methods of the latter class (see mlpack documentation).

```
// Create a linear SVM architecture
LinearSVM<> classifier;
```

- **Naive Bayes classifier (requires mlpack)**: This classifier is implemented in the class `NaiveBayes`, which inherits from the mlpack class `mlpack::naive_bayes::NaiveBayesClassifier`[illegible]

```
// Create a naive Bayes classifier
NaiveBayes<> classifier;
```

- **Random classifier**: This classifier is implemented in the class `RndClassifier` and mainly serves for debugging purposes.

```
// Create a random classifier with seed = 777
RndClassifier<> classifier(777);
```

Similarity measures

All similarity measures in LinkPred inherits from the abstract class `SimMeasure`, which defines the following interface:

Listing 5.3: code/ml/simmeasure.hpp

```
class SimMeasure {
protected:
  std::string name; /**< The name of the similarity measure. */
public:
  /**
   * Compute the similarity between two vectors.
   * @param x First vector.
   * @param y Second vector. Must be of the same dimension as x.
   * @return The similarity between x and y.
   */
  virtual double sim(Vec const & x, Vec const & y) = 0;
  const std::string& getName() const {
    return name;
  }
  void setName(const std::string &name) {
    this->name = name;
  }
};
```

The library contains the most commonly used similarity measures:

- **Cosine similarity**: is implemented in the class `CosineSim` and returns the cosine of the degree between the two input vectors x and y, that is:

$$\frac{\sum_{i=1}^{d} x_i y_i}{\sqrt{\sum_{i=1}^{d} x_i^2}\sqrt{\sum_{i=1}^{d} y_i^2}}. \tag{5.1}$$

- **Dot product similarity**: is implemented in the class `DotProd` and simply returns the dot product of the two input vectors x and y, that is:

$$\sum_{i=1}^{d} x_i y_i. \tag{5.2}$$

- L_2 **similarity**: is implemented in the class `L2Sim` and returns the negative of the L_2 (Euclidean) distance between x and y, that is:

$$-\sqrt{\sum_{i=1}^{d} (x_i - y_i)^2}. \tag{5.3}$$

- L_1 **similarity**: is implemented in the class `L1Sim` and returns the negative of the L_1 (Manhattan) distance between x and y, that is:

$$-\sum_{i=1}^{d} |x_i - y_i|. \tag{5.4}$$

- L_p **similarity**: is implemented in the class `LPSim` and returns the negative of the L_p (p is passed as parameter to the constructor) distance between x and y, that is:

$$-\left(\sum_{i=1}^{d} |x_i - y_i|^p\right)^{\frac{1}{p}}. \tag{5.5}$$

- **Pearson similarity**: is implemented in the class `Pearson` and returns the Pearson correlation coefficient between the two input vectors x and y, that is:

$$\frac{\sum_{i=1}^{d} (x_i - \bar{x})(y_i - \bar{y})}{\sqrt{\sum_{i=1}^{d} (x_i - \bar{x})^2 \sum_{i=1}^{d} (y_i - \bar{y})^2}}. \tag{5.6}$$

The following code shows how to use these classes to compute the similarity between two vectors:

Listing 5.4: code/ml/simmeasures.cpp

```
#include <linkpred.hpp>
#include <iostream>
using namespace LinkPred;

int main(int argc, char *argv[]) {

  Vec x = {1, 0, 1};
  Vec y = {-1, 1, 2};

  CosineSim csm;
  std::cout << "CosineSim: " << csm.sim(x, y) << std::endl;

  DotProd dp;
```

```
  std::cout << "DotProd: " << dp.sim(x, y) << std::endl;

  L1Sim l1;
  std::cout << "L1Sim: " << l1.sim(x, y) << std::endl;

  L2Sim l2;
  std::cout << "L2Sim: " << l2.sim(x, y) << std::endl;

  LPSim l3(3);
  std::cout << "L3Sim: " << l3.sim(x, y) << std::endl;

  Pearson prs;
  std::cout << "Pearson: " << prs.sim(x, y) << std::endl;

  return 0;
}
```

This out the output of the code above:

```
CosineSim: 0.288675
DotProd: 1
L1Sim: -4
L2Sim: -2.44949
L3Sim: -1.81712
Pearson: -0.188982
```

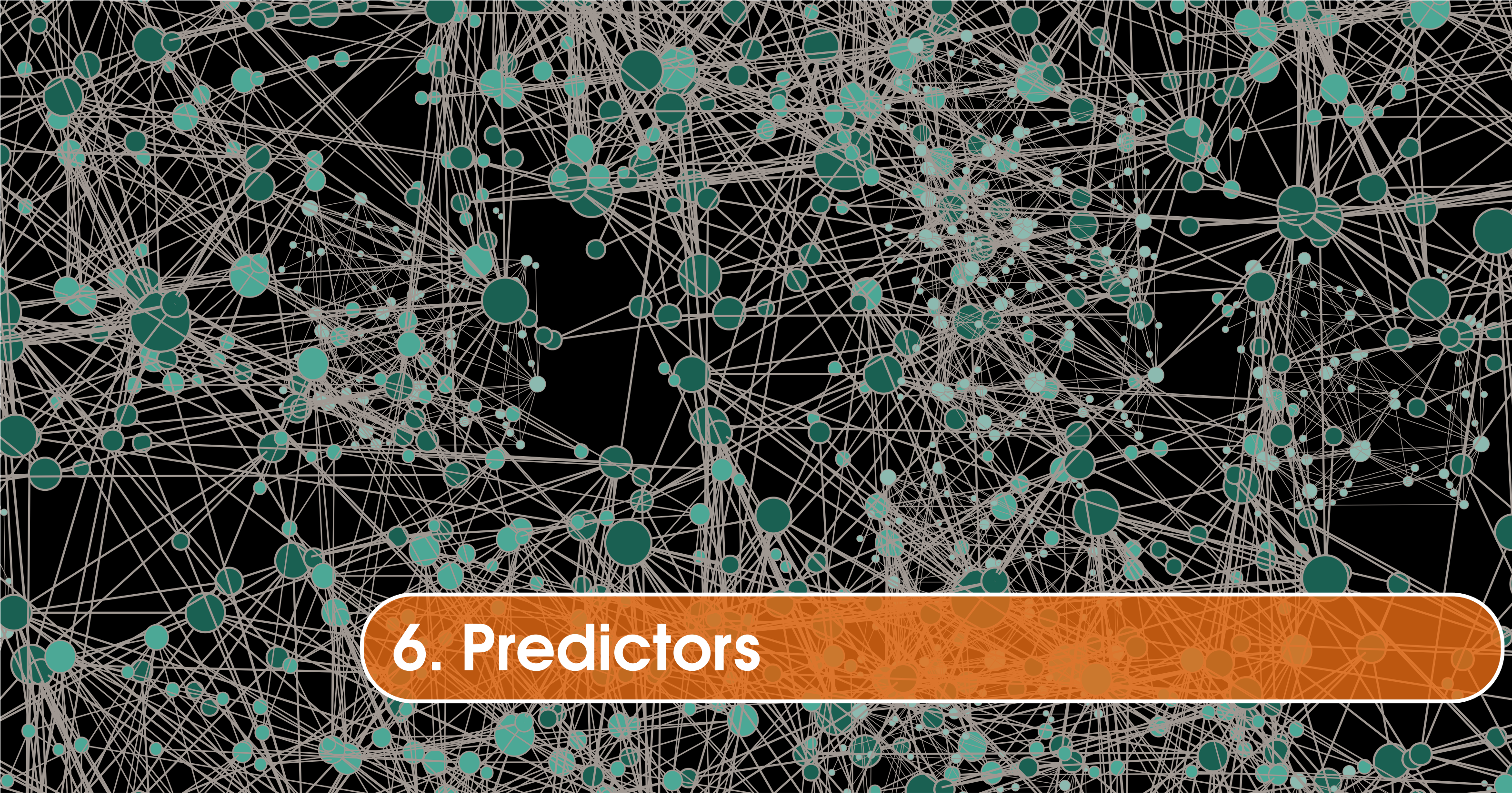

6. Predictors

In this chapter, we cover the link prediction algorithms available in LinkPred. The library offers a unified interface for all link prediction algorithms which simplifies the use and comparison of different prediction methods. This interface is presented first in this chapter. The two subsequent sections present the available prediction algorithms for undirected networks and directed networks respectively. We end the chapter with an explanation on how to implement your own link prediction algorithm so that it can be used with LinkPred classes.

> **R** Predictor classes are grouped under the namespace `Predictors`, and can be imported using:
>
> In the examples included in this chapter, we assume that this namespace is imported and drop the prefix `LinkPred::` from all classes for convenience.

6.1 The predictor interface

All link predictors for undirected networks must inherit from the abstract class `ULPredictor` shown below. It declares three important virtual methods that must be implemented by the derivative classes:

- The method `void init()`: This method is used to initialize the state of the predictor, including any internal data structures. Depending on the predictor, this method may be left empty if no such initialization is required.
- The method `void learn()`: In algorithms that require learning, it is in this method that the model is built. The learning is separated from prediction, because usually the model is independent from the set of edges to be predicted. Notice that even if the algorithm does not require any learning, this method must still be implemented (it can be left empty).
- The method `double score(Edge const & e)`: returns the score for the edge `e` (usually a negative edge).

In addition to these three basic methods, `ULPredictor` declares the following three methods:

- The method `void predict(EdgeRndIt begin, EdgeRndIt end, ScoreRndIt scores)`: In this method, the edges to be predicted are passed to the predictor in the form of a range (`begin`, `end`) in addition to a third parameter (`scores`) to which the scores are written. All iterators must allow random access, and the memory for storing the scores must already be allocated.
- The method `std::pair<NonEdgeIt, NonEdgeIt> predictNeg(ScoreRndIt scores)` predicts the score for all negative (non-existing) links in the network. The scores are written into the random output iterator `scores`. The method returns a pair of iterators begin and end to the range of non-existing links predicted by the method.
- The method `std::size_t top(std::size_t k, EdgeRndOutIt eit, ScoreRndIt sit)` finds the k negative edges with the top score. The edges are written to the output iterator `eit`, whereas the scores are written to `sit`. The scores are written in the same order as the edges. The method returns the number of negative edges inserted. It is the minimum between k and the number of negative edges in the network. Ties are broken randomly.

The class `ULPredictor` offers default implementations for the methods `top`, `predict` and `predictNeg`. Sub-classes may use these implementations or redefine them to achieve better performance.

Listing 6.1: code/predictors/ulpredictor.hpp

```
template<...> class ULPredictor {
  virtual void init() = 0;
  virtual void learn() = 0;
  virtual double score(EdgeType const & e);
  virtual void predict(EdgesRandomIteratorT begin,
     EdgesRandomIteratorT end, ScoresRandomIteratorT scores);
  virtual std::pair<typename Network::NonEdgeIterator, typename
     Network::NonEdgeIterator> predictNeg(ScoresRandomIteratorT
     scores);
  virtual std::size_t top(std::size_t k,
     EdgesRandomOutputIteratorT eit, ScoresRandomIteratorT sit);
};
```

The abstract class `DLPredictor` plays the same role as `ULPredictor` but for link predictors in directed networks. It offers the same interface as the latter but with different default template arguments and methods implementation.

6.2 Link predictors for undirected networks

Most link predictors proposed in the literature apply to undirected networks. In this section, we present the main prediction algorithms implemented in LinkPred. We divide these into topological ranking methods and global methods. Topological ranking methods (or local methods) are fast and can scale to very large networks. On the other hand, global methods, which although produce good prediction results, are usually limited to small to medium sized networks because of their high computational requirements.

6.2.1 Topological-ranking methods

Topological-ranking methods use local topological information to assign scores to edges [21, 33]. Since they do not require learning, they are in general computationally efficient, and depending on the type of network, may produce highly precise predictions. A large number of topological measures have been proposed by researchers, and implementing all of them can be an arduous task. Nevertheless, LinkPred contains the implementation of the most important measures found in the literature.

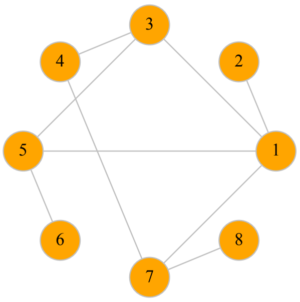

Figure 6.1: Example network.

1. **Adamic-Adar index (ADA)**: In this method, a couple (i, j) is assigned the score:

$$s_{ij} = \sum_{k \in \Gamma_{ij}} \frac{1}{\log(\kappa_k)}, \tag{6.1}$$

where Γ_{ij} is the set of nodes adjacent to both i and j (set of common neighbors of i and j), and κ_k is the degree of node k. If i and j have no common neighbors, their score is set to 0. Eq. (6.24) is well defined, because $\kappa_k \neq 1$ (since k is a common neighbor of i and j, its degree must be at least 2).

■ **Example 6.1** Consider the network of Figure 6.1. The Adamic-Adar index score of $(3,7)$ is:

$$\frac{1}{\log(\kappa_1)} + \frac{1}{\log(\kappa_4)} = \frac{1}{\log(4)} + \frac{1}{\log(2)} \approx 2.1640.$$

The score of $(5,8)$ is zero, since the two nodes have no common neighbors. ■

The Adamic-Adar index method is implemented in the class `ADAPredictor`.

2. **Common neighbors (CNE)**: In this approach, the score of a couple (i, j) is simply the number of common neighbors of i and j:

$$s_{ij} = |\Gamma_{ij}|. \tag{6.2}$$

■ **Example 6.2** For the network shown in Figure 6.1, the score of $(3,7)$ is 2, whereas the score of $(5,8)$ is zero, since thy have no common neighbors. ■

The common neighbors index method is implemented in the class `CNEPredictor`.

3. **Cannistraci resource allocation** index (CRA): The score of a couple (i,j) is given by:

$$s_{ij} = \sum_{k \in \Gamma_{ij}} \frac{|\Gamma_k \cap \Gamma_{ij}|}{\kappa_k},$$

where Γ_k is the set of nodes adjacent to node k.

■ **Example 6.3** For the network shown in Figure 6.2, the score given by CRA to $(3,7)$ is $\frac{1}{5} + \frac{1}{3} = 0.53$. ■

The predictor CRA is implemented by the class `CRAPredictor`.

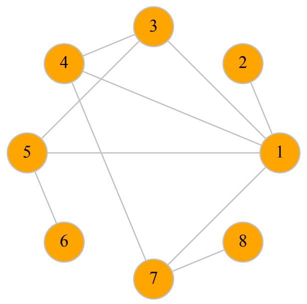

Figure 6.2: Example network.

4. **Hub depromoted index (HDI)**: In this approach, the score of a couple (i,j) is given by:

$$s_{ij} = \frac{|\Gamma_{ij}|}{\max(\kappa_i, \kappa_j)}. \quad (6.3)$$

■ **Example 6.4** For the network shown in Figure 6.1. The score of $(1,4)$ is 0.5, and so is the score of $(4,8)$. ■

The hub depromoted index method is implemented in the class `UHDIPredictor`.

5. **Hub promoted index (HPI)**: In this approach, the score of a couple (i,j) is given by:

$$s_{ij} = \frac{|\Gamma_{ij}|}{\min(\kappa_i, \kappa_j)}. \quad (6.4)$$

■ **Example 6.5** For the network shown in Figure 6.1, the score of $(1,4)$ is 1, and so is the score of $(4,8)$. ■

The hub promoted index method is implemented in the class `UHPIPredictor`.

6. **Jackard index (JID)**: In this approach, the score of a couple (i,j) is given by:

$$s_{ij} = \frac{|\Gamma_{ij}|}{\kappa_i + \kappa_j - |\Gamma_{ij}|}. \quad (6.5)$$

If i and j have no common neighbors, the score 0 is assigned.

Example 6.6 For the network shown in Figure 6.1. The score of $(1,4)$ is 0.5, and so is the score of $(4,8)$. ■

The Jackard index method is implemented in the class `UJIDPredictor`.

7. **Local path index (LCP)**: This method can be thought of as higher order correction to common neighbors. Instead of considering only paths of length two between the two nodes i and j (which is equal to the number of common neighbors), the number of paths of length three, Π^3_{ij}, is also considered:

$$s_{ij} = |\Gamma_{ij}| + \varepsilon \Pi^3_{ij}, \tag{6.6}$$

where ε is an algorithm parameter, which usually takes small values (1e-3 for instance). It is worth mentioning that the computation of paths of length three increases the computational complexity of LCP compared to the other topological-ranking methods.

Example 6.7 For the network shown in Figure 6.1, and taking $\varepsilon = 0.001$. The score of $(3,7)$ is $2+0.001\times 1 = 2.001$, and that of $(5,8)$ is $0+0.001\times 1 = 0.001$. ■

The local path index method is implemented in the class `ULCPPredictor`. The default value of ε is 1e-3. To read the value ε use the method `double getEpsilon()const`, and to modify it use `void setEpsilon(double epsilon)`.

8. **Leicht-Holme-Newman index (LHN)**: In this approach, the score of a couple (i,j) is given by:

$$s_{ij} = \frac{|\Gamma_{ij}|}{\kappa_i \kappa_j}. \tag{6.7}$$

Example 6.8 For the network shown in Figure 6.1. The score of $(1,4)$ is 0.25, whereas the score of $(4,8)$ is 0.5. ■

The Leicht-Holme-Newman index method is implemented in the class `ULHNPredictor`.

9. **Preferential attachment index (PAT)**: In this approach, the score of a couple (i,j) is simply given by:

$$s_{ij} = \kappa_i \kappa_j. \tag{6.8}$$

Example 6.9 For the network shown in Figure 6.1. The score of $(1,4)$ is 8, whereas the score of $(4,8)$ is 2. ■

The preferential attachment index method is implemented in the class `UPATPredictor`.

10. **Resource allocation index (RAL)**: In this approach, the score of a couple (i,j) is given by:

$$s_{ij} = \sum_{k\in\Gamma_{ij}} \frac{1}{\kappa_k}. \tag{6.9}$$

Example 6.10 For the network shown in Figure 6.1. The score of $(1,4)$ is 0.67, whereas the score of $(4,8)$ is 0.33. ■

The resource allocation index method is implemented in the class `URALPredictor`.

11. **Salton index (SAI)**: In this approach, the score of a couple (i,j) is given by:

$$s_{ij} = \frac{|\Gamma_{ij}|}{\sqrt{\kappa_i \kappa_j}}. \quad (6.10)$$

■ **Example 6.11** For the network shown in Figure 6.1. The score of $(1,4)$ is $1/\sqrt{2} = 0.707$, and so is the score of $(4,8)$. ■

The Salton index method is implemented in the class `USAIPredictor`.

12. **Sorensen index (SOI)**: In this approach, the score of a couple (i,j) is given by:

$$s_{ij} = \frac{|\Gamma_{ij}|}{\kappa_i + \kappa_j}. \quad (6.11)$$

■ **Example 6.12** For the network shown in Figure 6.1. The score of $(1,4)$ is 0.33, and so is the score of $(4,8)$. ■

The Sorensen index method is implemented in the class `USOIPredictor`.

13. **Sum of degrees index (SUM)**: A popularity index where the score for couple (i,j) is simply given by:

$$s_{ij} = \kappa_i + \kappa_j. \quad (6.12)$$

■ **Example 6.13** For the network shown in Figure 6.1. The score of $(1,4)$ is 6, whereas the score of $(4,8)$ is 3. ■

The sum of degrees index method is implemented in the class `USUMPredictor`.

■ **Example 6.14** The following code shows how to compute the scores of all non-existing links using ADA:

Listing 6.2: code/predictors/ada.cpp

```
#include <linkpred.hpp>
#include <iostream>
using namespace LinkPred;
int main() {
  auto net = UNetwork<>::read("Zakarays_Karate_Club.edges");
  UADAPredictor<> predictor(net);
  predictor.init();
  predictor.learn();
  std::cout << "#Start\tEnd\tScore\n";
  for (auto it = net->nonEdgesBegin(); it != net->nonEdgesEnd();
     ++it) {
    auto i = net->getLabel(net->start(*it));
    auto j = net->getLabel(net->end(*it));
    double sc = predictor.score(*it);
    std::cout << i << "\t" << j << "\t" << sc << std::endl;
  }
  return 0;
}
```

■

■ **Example 6.15** The following code shows how to compute the top k scores using RAL:

Listing 6.3: code/predictors/raltop.cpp

```
#include <linkpred.hpp>
#include <iostream>
using namespace LinkPred;
int main() {
  int k = 10;
  auto net = UNetwork<>::read("Zakarays_Karate_Club.edges");
  URALPredictor<> predictor(net);
  predictor.init();
  predictor.learn();
  std::vector<typename UNetwork<>::Edge> edges(k);
  std::vector<double> scores(k);
  k = predictor.top(k, edges.begin(), scores.begin());
  std::cout << "#Start\tEnd\tScore\n";
  for (int i = 0; i < k; i++) {
    std::cout << net->getLabel(net->start(edges[i])) << "\t" <<
       net->getLabel(net->end(edges[i])) << "\t" << scores[i] <<
       std::endl;
  }
  return 0;
}
```

■

6.2.2 Global predictors

1. **Similarity-popularity algorithm introduced in [16] (KAB)**:This method assumes that the likelihood of the existence of a link depends on popularity, similarity and local attraction. The algorithm pre-weights the graph with a specific weight map that factors out non-similarity factors and uses it to find similarity between non-connected nodes. The result is then used to assign scores to non-existing edges.
More precisely, the likelihood of a link between two nodes i, j is assumed proportional to:

$$\Psi(i,j) = \left(\pi_{ij} + \eta_{ij}\right) s_{ij}, \tag{6.13}$$

where s_{ij} is the similarity between i and j, π_{ij} is a measure of the popularity of the two nodes, and η_{ij} represents the local attraction between them. Given ϕ:

$$\phi(x) = \log(x+1). \tag{6.14}$$

The popularity term π_{ij} is defined as:

$$\pi_{ij} = \frac{\phi(\kappa_i) + \phi(\kappa_j)}{2\phi(\kappa_{\max})}, \tag{6.15}$$

where κ_i and κ_j are the degrees of i and j receptively and κ_{max} the maximum degree in the network. The local attraction term η_{ij} depends on the local topology near the two nodes:

$$\eta_{ij} = 1 - \prod_{k \in \Gamma_{ij}} \frac{\phi(\kappa_k)}{\phi(\kappa_{\max})}, \tag{6.16}$$

where Γ_{ij} is the set of common neighbors of i and j, and κ_k is the degree of node k. The similarity term s_{ij} is defined as follows:

$$s_{ij} = \frac{1}{1+d_{ij}}. \tag{6.17}$$

Every edge $(i,j) \in E$ is assigned the length $\omega(i,j)$ given by:

$$\omega(i,j) = \frac{2\pi_{ij}}{1+\eta_{ij}}, \tag{6.18}$$

Using this weight map, shortest path distance is used to compute the dissimilarity between non-adjacent. The latter can be used to assign a score $\psi_{ij} = \Psi(i,j)$ to any negative link (i,j).
This predictor is implemented in the class `UKABPredictor` and has two parameters. The first parameter is the horizon limit, an integer that limits computation when computing shortest paths: any two nodes separated by more nodes than the horizon limit are considered disconnected. The horizon limit can be set and get using `setHorizLim(int h)` and `int getHorizLim()const` respectively. The second parameter is the cache strategy used to store the shortest path distances. This can be read set using the method `CacheLevel getCacheLevel()const` and `void setCacheLevel(CacheLevel cacheLevel)` respectively (see Section 4.2.2 for more details).

2. **Hierarchical Random Graph (HRG)** [7] is a probabilistic model where a hierarchical structure consisting of a binary tree is used to predict connection probabilities. The leaves of the binary tree represent the nodes of the network, whereas internal nodes correspond to nested clusters. Each cluster is assigned the probability of a link existing between its children. The probability of two nodes being connected is then determined by finding their lowest common ancestor. This algorithm is implemented by the class `UHRGPredictor` (which is actually a C++ wrapper around the code provided by the authors). This algorithms requires three parameters: a seed passed to the constructor and used to initialize the internal random generator, the number of bins which can be accessed and modified by `int getNbBeans()const` and `void setNbBeans(int nbBeans)` , and the number of samples which can be read and set using `int getNbSamples()const` and `void setNbSamples(int nbSamples)` . The default value for `nbBeans` is 25 and that of `nbSamples` is 10000.
3. **Stochastic block model (SBM)** [12] is a probabilistic model, where the nodes are divided into non-overlapping partitions. A matrix Q specifies the partition-to-partition connection probability, which is the probability that a node from one partition connects to a node from the other one. SBM can be used to detect both missing and spurious links. It produces excellent results in general, but its high computational cost limits its use to small networks. This algorithm is implemented by the class `USBMPredictor` (which is actually a C++ wrapper around the C code provided by the authors). This algorithms requires two parameters: a seed passed to the constructor and used to initialize the internal random generator, and the maximum number of iterations. The latter can be read and modified

by `std::size_t getMaxIter()const` and `void setMaxIter(std::size_t maxIter)` respectively. The default value of `maxIter` is 10000.

4. **Fast blocking model (FBM)** [20] uses as greedy search strategy to efficiently partition the network into communities, reducing hence the computation complexity of the graph partitioning task. The link densities within and between communities are used to estimate the connection probability between nodes. Despite producing results that are in general slightly lower than those of SBM, its low computational requirements make FBM a good choice for average-size networks. FBM is implemented in the class `UFBMPredictor` (a C++ translation of the Matlab code provided by the authors). This class requires a single parameter, which is the maximum number of iterations. It can be read and set using `std::size_t getMaxIter()const` and `void setMaxIter(std::size_t maxIter)` respectively. The default value of `maxIter` is 50.
5. **HyperMap (HYP)** [23, 24]: The *Popularity×Similarity Optimization* (PSO) and its variant E-PSO are complex network models that assume the existence of a hidden hyperbolic space that controls the topology of real networks. The likelihood of connection between nodes is a trade-off between the similarity of the nodes and their popularity, and it is the behavior of the connection probability with respect to nodes popularity that gives the hidden metric space its hyperbolic geometry. In these models, every is assigned a radial coordinate r_i and an angular coordinate θ_i. The probability that two nodes i, j connect is then given by:

$$p_{ij} = \frac{1}{1+e^{(d_{ij}-R)/T}}, \tag{6.19}$$

where R and T are model parameters and d_{ij} is the hyperbolic distance between i and j given by:

$$d_{ij} \approx r_i + r_j + \frac{2}{\zeta}\ln(\theta_{ij}/2), \tag{6.20}$$

where θ_{ij} is angular distance between i and j given by $\theta_{ij} = \pi - |\pi - |\theta_i - \theta_j||$. The parameter $\zeta = \sqrt{-K}$ with K representing the curvature of the hyperbolic plane.
The HyperMap algorithm embeds the network according to the E-PSO model by assuming that r_i are equal degree and using the *Metropolis-Hastings* algorithm to find the coordinates θ_i that maximize the local likelihood L_i,

$$L_i = \prod_{1\leq j\leq i} (p_{ij})^{a_{ij}}[1-(p_{ij})]^{1-a_{ij}} \tag{6.21}$$

HyperMap is implemented in the class `UHYPPredictor` (a wrapper around the code provided by the authors with the additional feature of fitting power law distribution to estimate the exponent γ as explained below). It receives a random number generator seed in its constructor. Additionally, it has five parameters required by the E-PSO model:
   (a) m: represents the average number of nodes with which new nodes connect. It is set to the minimum degree in the network. After calling `init`, it is possible to get and set m using `double getM() const` and `void setM(double m)` respectively.

(b) L: represents the average number of nodes with which old nodes connect and is set to $L = (\langle k \rangle - 2m)/2$, where $\langle k \rangle$ is the average node degree. After calling `init`, it is possible to get and set L using `double getL() const` and `void setL(double L)` respectively.

(c) γ: is the exponent of the power-law degree distribution. In our implementation, it is calculated from the degree distribution of the training set network using *plfit*, a C++ implementation of Clauset, Shalizi and Newman [8] method for fitting power law distributions written by Tamas Nepusz (`http://tuvalu.santafe.edu/~aaronc/powerlaws/`). After calling `init`, it is possible to get and set γ using `double getGamma() const` and `void setGamma(double gamma)` respectively.

(d) T: controls the average clustering. It can be read and modified using `double getT() const` and `void setT(double T)` respectively. The default value is 0.8.

(e) $\zeta = \sqrt{-K}$ where K is the curvature of the hyperbolic plane. It is set by default to 1 and can be read and modified using `double getZeta() const` and `void setZeta(double zeta)`.

HyperMap gives good results in Internet networks and networks with similar properties, but it has a high a computational cost in general.

6. **Shortest-path predictor (SHP)**: This predictor assigns scores to node couples according to their shortest-path distance:

$$s_{ij} = \frac{1}{d_{ij}}. \tag{6.22}$$

It can also assign scores to existing edges:

$$s_{ij} = \frac{1}{\bar{d}_{ij}}, \tag{6.23}$$

where $\bar{d}_{ij}$ is the distance between i and j obtained by first removing the edge (i, j). The shortest-path predictor is implemented by the class `USHPPredictor`. The distances are computed using Dijkstra's algorithm, which is executed during the `predict` method. The distances are therefore not pre-calculated, but rather computed on-demand according to the set of edges to be predicted. Nevertheless, to improve the time performance the distances can be cached. The cache strategy can be read set using the method `CacheLevel getCacheLevel() const` and `void setCacheLevel(CacheLevel cacheLevel)` respectively (see Section 4.2.2 for more details).

■ **Example 6.16** The following code shows how to compute the scores of all non-existing links using SBM:

Listing 6.4: code/predictors/sbm.cpp

```
#include <linkpred.hpp>
#include <iostream>
using namespace LinkPred;
int main() {
```

```
  auto net = UNetwork<>::read("Zakarays_Karate_Club.edges");
  USBMPredictor<> predictor(net, 777);
  predictor.init();
  predictor.learn();
  std::cout << "#Start\tEnd\tScore\n";
  for (auto it = net->nonEdgesBegin(); it != net->nonEdgesEnd();
     ++it) {
    auto i = net->getLabel(net->start(*it));
    auto j = net->getLabel(net->end(*it));
    double sc = predictor.score(*it);
    std::cout << i << "\t" << j << "\t" << sc << std::endl;
  }
  return 0;
}
```

■

■ **Example 6.17** The following code shows how to compute the top *k* scores using KAB:

Listing 6.5: code/predictors/kabtop.cpp

```
#include <linkpred.hpp>
#include <iostream>
using namespace LinkPred;
int main() {
  int k = 10;
  auto net = UNetwork<>::read("Zakarays_Karate_Club.edges");
  UKABPredictor<> predictor(net);
  predictor.init();
  predictor.learn();
  std::vector<typename UNetwork<>::Edge> edges(k);
  std::vector<double> scores(k);
  k = predictor.top(k, edges.begin(), scores.begin());
  std::cout << "#Start\tEnd\tScore\n";
  for (int i = 0; i < k; i++) {
    std::cout << net->getLabel(net->start(edges[i])) << "\t" <<
        net->getLabel(net->end(edges[i])) << "\t" << scores[i] <<
        std::endl;
  }
  return 0;
}
```

■

6.2.3 Network embedding methods

In these methods, the network is first embedded into a low dimensional vector space, whereby nodes are assigned coordinates in that space while preserving the network's structural properties. These coordinates can be used either to compute the similarity between nodes or as features to train a classifier to discriminate between existing edges (the positive class) and non-existing edges (the negative class) [10].

LinkPred provides two classes that can be used to build link prediction algorithms based on graph embedding: the class `UECLPredictor`, which combines an encoder (a graph embedding algorithm) and a classifier (Figure 6.3), and the class `UESMPredictor`,

which pairs the encoder with a similarity measure (Figure 6.4).

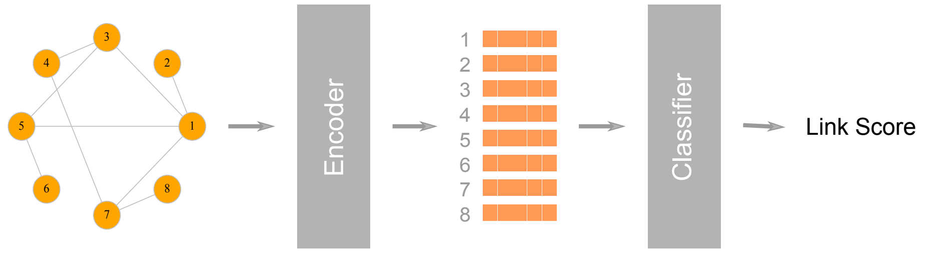

Figure 6.3: The class `UECLPredictor` uses an encoder to embed the graph followed by a classifier to predict link scores.

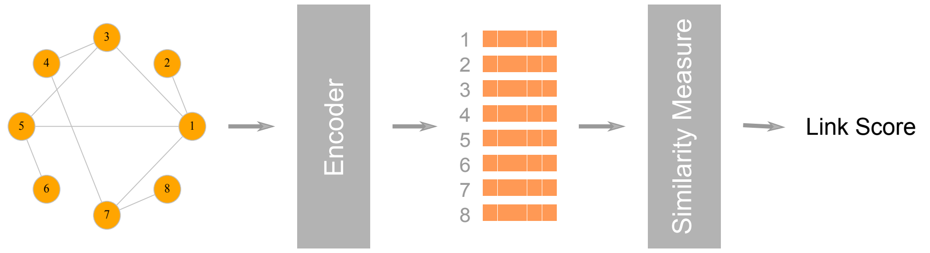

Figure 6.4: The class `UESMPredictor` uses an encoder to embed the graph followed by a similarity measure to predict link scores.

The components of these predictors are passed to their constructors:

```
UECLPredictor<> predictor(net, encoder, classifier, seed);
UESMPredictor<> predictor(net, encoder, simMeasure);
```

The argument encoder must be a shared pointer to an object of type `Encoder`, for example:

```
auto encoder = std::make_shared<Node2Vec<>>(net, 777);
```

Section 4.3 gives a detailed presentation about the graph embedding algorithms available in LinkPred and the interface `Encoder`.

The following code shows how to create a prediction algorithm that uses Node2Vec and logistic regression to predict links. As mentioned earlier, since we are predicting using a classifier, we will use the class `UECLPredictor`:

Listing 6.6: code/predictors/ecl.cpp

```
#include <linkpred.hpp>
#include <iostream>
using namespace LinkPred;
int main(int argc, char*argv[]) {
  // Load network
  auto net = UNetwork<>::read("Zakarays_Karate_Club.edges");
  // Create the encoder
  auto encoder = std::make_shared<Node2Vec<>>(net, 777);
  // Create the classifier
  auto classifier = std::make_shared<LogisticRegresser<>>(0.001,
      888);
```

```
  // Create the predictor
  UECLPredictor<> predictor(net, encoder, classifier, 999);
  // Initialize and train
  predictor.init();
  predictor.learn();
  // Print the score of all non-existing edges
  std::cout << "#Start\tEnd\tScore\n";
  for (auto it = net->nonEdgesBegin(); it != net->nonEdgesEnd();
      ++it) {
    auto i = net->getLabel(net->start(*it));
    auto j = net->getLabel(net->end(*it));
    double sc = predictor.score(*it);
    std::cout << i << "\t" << j << "\t" << sc << std::endl;
  }
  return 0;
}
```

If we want to use a similarity measure instead of a classifier, we use the class `UESMPredictor` (here, we are using LINE as encoder):

Listing 6.7: code/predictors/esm.cpp

```
#include <linkpred.hpp>
#include <iostream>
using namespace LinkPred;
int main(int argc, char*argv[]) {
  // Load network
  auto net = UNetwork<>::read("Zakarays_Karate_Club.edges");
  // Create the encoder
  auto encoder = std::make_shared<LINE<>>(net, 777);
  // Create the similarity measure
  auto simMeasure = std::make_shared<CosineSim>();
  // Create the predictor
  UESMPredictor<> predictor(net, encoder, simMeasure);
  // Initialize and train
  predictor.init();
  predictor.learn();
  // Print the score of all non-existing edges
  std::cout << "#Start\tEnd\tScore\n";
  for (auto it = net->nonEdgesBegin(); it != net->nonEdgesEnd();
      ++it) {
    auto i = net->getLabel(net->start(*it));
    auto j = net->getLabel(net->end(*it));
    double sc = predictor.score(*it);
    std::cout << i << "\t" << j << "\t" << sc << std::endl;
  }
  return 0;
}
```

6.2.4 Utility predictors

LinkPred includes a number of link prediction classes that can be useful for debugging and similar purposes:

- **The constant predictor**: This is a predictor that assigns a constant score, namely 0, to all links. It is useful for debugging performance measures as its performance

on a given test data can be easily calculated theoretically. This algorithm is implemented in the class `UCSTPredictor` .

- **The random predictor**: This is a predictor that assigns a random score uniformly distributed in $[0,1)$. Similar to the constant predictor, the random predictor is useful for debugging performance measures as its expected performance on a given test data can also be easily calculated theoretically. This algorithm is implemented in the class `URNDPredictor` .
- **Predictor with pre-stored scores**: This algorithm loads edge scores from a file and merely serves as a lookup table. It is useful for evaluating the results of link prediction algorithms implemented outside LinkPred. It is intended to play a proxy role on behalf of the external algorithm and uses the pre-stored data to predict links. This algorithm is implemented in the class `UPSTPredictor` . The scores are loaded using the method `loadEdgeScores` . The format of this file is as follows (the first is just a comment and can be omitted):

```
#Start   End      Score
1        31       0.41374
1        10       0.276687
1        28       0.283587
1        29       0.374494
1        33       0.463135
1        17       0.531863
1        34       0.49409
1        26       0.325087
1        25       0.325087
...
```

■ **Example 6.18** The following code shows how to use `UPSTPredictor` :

Listing 6.8: code/predictors/pst.cpp

```
#include <linkpred.hpp>
#include <iostream>
using namespace LinkPred;
int main(int argc, char*argv[]) {
  auto net = UNetwork<>::read("Zakarays_Karate_Club.edges");
  // First we compute scores using ADA and store them on file
  {
    UADAPredictor<> predictor(net);
    predictor.init();
    predictor.learn();
    std::vector<Utilities::EdgeScore<std::string>> esv;
    for (auto it=net->nonEdgesBegin();it!=net->nonEdgesEnd()
        ;++it){
      auto i = net->getLabel(net->start(*it));
      auto j = net->getLabel(net->end(*it));
      double sc = predictor.score(*it);
      esv.push_back({ i,  j, sc });
    }
    Utilities::writeEdgeScores("pstscores.csv", esv);
  }
  // We then load the scores into UPSTPredictor
  {
    UPSTPredictor<> predictor(net);
```

```
    predictor.loadEdgeScores("pstscores.csv");
    predictor.init();
    predictor.learn();
    std::vector<Utilities::EdgeScore<std::string>> esv;
    for (auto it = net->nonEdgesBegin(); it != net->
       nonEdgesEnd(); ++it){
      auto i = net->getLabel(net->start(*it));
      auto j = net->getLabel(net->end(*it));
      double sc = predictor.score(*it);
      std::cout << i << "\t" << j << "\t" << sc << std::endl;
    }
  }
  return 0;
}
```

■

Since `UPSTPredictor` requires knowing the test set beforehand, it is typically used with pre-generated data that is loaded from file (see Chapter 7). The typical workflow for using this predictor is as follows:

1. Generate test data using one of the methods available in `NetworkManipulator` (see Chapter 7) or using `Simp::Evaluator::genTestData` (see Chapter 2).
2. Save the test data to file.
3. Use the training part of the data to train the external algorithm.
4. Compute the scores of all links in the test set and save it to a file.
5. Load the test data from file using the method `NetworkManipulator::loadTestData` (see Chapter 7).
6. Load the scores from file using `UPSTPredictor::loadEdgeScores`.
7. Now, the results from the external algorithms on this test data can be compared against any algorithm implemented in LinkPred.

6.3 Link predictors for directed networks

LinkPred contains the implementations of several link prediction algorithms that work on directed networks. These are basically adaptations of topological-ranking methods shown earlier for the undirected case:

1. **Directed Adamic-Adar index (DADA)**: In this method, a couple (i, j) is assigned the score:

$$s_{ij} = \sum_{k \in \Gamma_{i \to j}} \frac{1}{\log(\kappa_k)}, \tag{6.24}$$

where $\Gamma_{i \to j}$ is the set of nodes k such that there exists and edge between i and k and an edge between k and j. Here, κ_k is the degree of node k (the sum of out and in-degrees). If $\Gamma_{i \to j}$ is empty, the score is set to 0. Eq. (6.24) is well defined, because $\kappa_k \neq 1$. The directed Adamic-Adar index method is implemented in the class `DADAPredictor`.
2. **Directed common neighbors (DCNE)**: In this approach, the score of a couple (i, j) is simply the size of the set $\Gamma_{i \to j}$. The directed common neighbors index method is implemented in the class `DCNEPredictor`.

3. **Directed hub depromoted index (DHDI)**: In this approach, the score of a couple (i, j) is given by:

$$s_{ij} = \frac{|\Gamma_{i \to j}|}{\max(\kappa_i^{out}, \kappa_j^{in})}, \tag{6.25}$$

where κ_i^{out} is the out-degree of i, and κ_j^{in} is the in-degree of j. The hub depromoted index method is implemented in the class `DHDIPredictor`.

4. **Directed hub promoted index (DHPI)**: In this approach, the score of a couple (i, j) is given by:

$$s_{ij} = \frac{|\Gamma_{i \to j}|}{\min(\kappa_i^{out}, \kappa_j^{in})}, \tag{6.26}$$

The hub promoted index method is implemented in the class `DHDIPredictor`.

5. **Directed Jackard index (DJID)**: In this approach, the score of a couple (i, j) is given by:

$$s_{ij} = \frac{|\Gamma_{i \to j}|}{\kappa_i^{out} + \kappa_j^{in} - |\Gamma_{i \to j}|}. \tag{6.27}$$

If $\Gamma_{i \to j}$ is empty, the score 0 is assigned. The directed Jackard index method is implemented in the class `DJIDPredictor`.

6. **Directed local path index (DLCP)**: This method can be thought of as higher order correction to directed common neighbors. It assigns the score:

$$s_{ij} = |\Gamma_{i \to j}| + \varepsilon \Pi^3_{i \to j}, \tag{6.28}$$

where Π^3_{ij} stands for the number of directed paths of length three, and ε is an algorithm parameter, which usually takes small values (1e-3 for instance). The directed local path index method is implemented in the class `DLCPPredictor`. The default value of ε is 1e-3. To read the value ε use the method `double getEpsilon()const`, and to modify it use `void setEpsilon(double epsilon)`.

7. **Directed Leicht-Holme-Newman index (DLHN)**: In this approach, the score of a couple (i, j) is given by:

$$s_{ij} = \frac{|\Gamma_{i \to j}|}{\kappa_i^{out} \kappa_j^{in}}. \tag{6.29}$$

The directed Leicht-Holme-Newman index method is implemented in the class `DLHNPredictor`.

8. **Directed preferential attachment index (DPAT)**: In this approach, the score of a couple (i, j) is simply given by:

$$s_{ij} = \kappa_i^{out} \kappa_j^{in}. \tag{6.30}$$

The directed preferential attachment index method is implemented in the class `DPATPredictor`.

9. **Directed Salton index (DSAI)**: In this approach, the score of a couple (i, j) is given by:

$$s_{ij} = \frac{|\Gamma_{i \to j}|}{\sqrt{\kappa_i^{out} \kappa_j^{in}}}. \tag{6.31}$$

The directed Salton index method is implemented in the class `DSAIPredictor`.

10. **Directed Sorensen index (DSOI)**: In this approach, the score of a couple (i, j) is given by:

$$s_{ij} = \frac{|\Gamma_{i \to j}|}{\kappa_i^{out} + \kappa_j^{in}}. \tag{6.32}$$

The directed Sorensen index method is implemented in the class `DSOIPredictor`.

6.4 Implementing a new link prediction algorithm

The first step in implementing a new link prediction algorithm is to inherit from `ULPredictor` and implement the necessary methods. For a minimal implementation, the three methods `init`, `learn` and `score` must at least be defined. If you want to achieve better performance you may want to redefine the three other methods (`top`, `predict` and `predictNeg`).

■ **Example 6.19** Suppose you want to create a very simple link prediction algorithm that assigns as score to (i, j) the score $\kappa_i + \kappa_j$, the sum of the degrees of the two nodes[1]. In a file named `usdpredictor.hpp`, write the following code:

Listing 6.9: code/predictors/usdpredictor.hpp

```
#ifndef USDPREDICTOR_HPP_
#define USDPREDICTOR_HPP_
#include <linkpred.hpp>
class USDPredictor: public LinkPred::ULPredictor<> {
  using LinkPred::ULPredictor<>::net;
  using LinkPred::ULPredictor<>::name;
public:
  using EdgeType = typename LinkPred::ULPredictor<>::EdgeType;
  USDPredictor(std::shared_ptr<Network const> net) :
      LinkPred::ULPredictor<>(net) {
    name = "USD";
  }
  virtual void init();
  virtual void learn();
  virtual double score(EdgeType const & e);
  virtual ~USDPredictor() = default;
};
#endif
```

[1] LinkPred already contains a sum-of-degree predictor named `USUMPredictor`.

R The abstract class `ULPredictor` is in fact a class template, but in the code above we are extending it with the default template parameters. Although a bit restrictive, this approach is the quickest and easiest way to add a new predictor.

In a file named `usdpredictor.cpp` write the implementation of the abstract methods:

Listing 6.10: code/predictors/usdpredictor.cpp

```
#include "usdpredictor.hpp"
void USDPredictor::init() {}
void USDPredictor::learn() {}
double USDPredictor::score(EdgeType const & e) {
  auto i = net->start(e);
  auto j = net->end(e);
  return net->getDeg(i) + net->getDeg(j);
}
```

Note that this predictor does not require initialization or learning. This predictor is now ready to be used with LinkPred classes and methods. We can write a code that uses this predictor to find the top k missing links:

Listing 6.11: code/predictors/usdtop.cpp

```
#include <linkpred.hpp>
#include "usdpredictor.hpp"
#include <iostream>
using namespace LinkPred;
int main(int argc, char*argv[]) {
  std::size_t k = 10;
  auto net = UNetwork<>::read("Infectious.edges");
  USDPredictor predictor(net);
  predictor.init();
  predictor.learn();
  std::vector<typename UNetwork<>::EdgeType> edges;
  edges.resize(k);
  std::vector<double> scores;
  scores.resize(k);
  k = predictor.top(k, edges.begin(), scores.begin());
  std::cout << "#Start\tEnd\tScore\n";
  for (std::size_t l = 0; l < k; l++) {
    auto i = net->getLabel(net->start(edges[l]));
    auto j = net->getLabel(net->end(edges[l]));
    std::cout << i << "\t" << j << "\t" << scores[l] <<std::endl;
  }
  return 0;
}
```

■

7. Performance Evaluation

Performance evaluation is a crucial phase in the development of new link prediction algorithms as well as in the study of their effectiveness for a given type or family of networks. LinkPred offers a set of tools that help streamlining the performance evaluation procedure. This includes data setup functionalities, which can be used to create test data, efficient implementations of the most important performance measures used in link prediction literature, and and helper classes that facilitates the comparative evaluation of multiple link prediction algorithms using multiple performance measures.

7.1 Data setup

To measure the performance of a link prediction algorithm, it is presented with a distorted version of a fully known network that serves as ground truth data. The distortions to which the network is subjected can be categorized into three types[1]:

1. Removing existing links.
2. Adding new links.
3. A combination of the above.

The task of the link prediction algorithm is to determine the actual status of couples based on the observed relationships. Notice that traditionally, the role of link prediction methods has been limited to detecting missing links. As a result, most link prediction algorithms can only handle distortions of the first kind (link removal). Nevertheless, LinkPred offers the possibility of performing all three types of distortions. Before presenting the relevant classes and methods, some terminology is of the order:

- *The reference network* is the ground truth network used to measure the performance of the algorithm. This network is unknown to the algorithm.
- *The observed network* is the network obtained after distortion and presented to the algorithm.
- *A true positive link* is a link that is present in the reference network as well as the observed network.

[1]Notice that distortions are limited to edges. No nodes are added or removed from the network

- *A true negative link* is a link that is missing from the reference network as well as the observed network.
- *A false positive link* is a link that is missing from the reference network but present in the observed network.
- *A false negative link* is a link that is present in the reference network but missing form the observed network.

Notice that depending on the type of distortions applied to the network, some of the sets defined above (true positive links, true negative links, false positive links and false negative links) may be empty. For instance, if the network is only modified by removing existing links, the set of false positive links contains no elements[2].

The data used for performance evaluation is stored within a class named `TestData`. This class provides a smart pointer to the reference network via `getRefNet()`, a smart pointer to the observed network via `getObsNet()` and the following ranges:

- The set of positive links included in the test set: `posBegin()` and `posEnd()`.
- The set of negative links included in the test set: `negBegin()` and `negEnd()`.

A `TestData` object can be created by calling the constructor:

```
TestData testData(refNet, obsNet, remLinks, addLinks, tpLinks,
    tnLinks, posClass, negClass);
```

The two last arguments are of the type `LinkClass`:

```
enum LinkClass {
  TP, /**< True positive link. */
  FN, /**< False negative link. */
  FP, /**< False positive link. */
  TN /**< True negative link. */
};
```

and are used to specify the set of links used, respectively, as positive instances and negative instances in the test set. This allows for instance to consider non-existing links as the positive instances.

It is clear from the constructor's signature that `TestData` is intended to be merely a container to store the test data elements together. To generate the test data, LinkPred provides the class `NetworkManipulator`, which contains a set of static methods that can be used to that end. These methods are explained in detail in the next sections.

7.1.1 Creating test data by removing edges

The first method distorts the network by removing existing links:

```
createTestDataRem(NetworkCSP refNet, double remRatio, bool
    keepConnected, bool aTP, double tpRatio, bool aTN, double
    tnRatio, long int seed, bool preGenerateTPN = true);
```

The parameters of this method are as follows:

- `refNet`: A constant shared pointer to the reference network.

[2] It is important to keep in mind that the class of a link as defined here is determined solely by specifying the reference and observed networks and is independent of any classification results. Hence, if a classifier is used on the network, a true negative link may for example be classified as positive and will therefore constitute a false positive instance. This may render the discussion a bit confusing by times but is necessary to keep in line with existing conventions.

- `remRatio` : Value between 0 and 1 that specifies the percentage of edges to be removed.
- `keepConnected` : Specifies whether to keep the network connected. If the reference network is disconnected or the ratio of edges to be removed is too large to keep the network connected, an exception is raised.
- `aTP` : Specifies whether to use all true positive links in the test set.
- `tpRatio` : Ratio of true positive links to be used in the test set. This parameter is only relevant when `aTP` is false.
- `aTN` : Specifies whether to use all true negative links in the test set.
- `tnRatio` : Ratio of true negative links to be used in the test set. This parameter is only relevant when `aTN` is false.
- `seed` : The random number generator's seed.
- `preGenerateTPN` : Whether to pre-generate true positives and true negatives.

The set of false negative links is used as the set of positive instances in the test set, whereas the set of true negative links is used as the set of negative instances.

R If the parameter `preGenerateTPN` is set to false, edges are only generated on-demand. The class `TestData` can also *stream* edges without storing them in memory. This is particularity useful for very large networks. The streamed edges are accessed through the following methods of the class `TestData` :

```
auto posStrmBegin() const;
auto posStrmEnd() const;
auto negStrmBegin() const;
auto negStrmEnd() const;
```

7.1.2 Creating test data by adding edges

The second method distorts the network by adding new links:

```
createTestDataAdd(NetworkCSP refNet, double remRatio, bool aTP,
    double tpRati, bool aTN, double tnRatio, long int seed, bool
    preGenerateTPN = true);
```

The parameters of this method are as follows:

- `refNet` : A constant shared pointer to the reference network.
- `addRatio` : Value between 0 and 1 that specifies the percentage of edges to be added.
- `aTP` : Specifies whether to use all true positive links in the test set.
- `tpRatio` : Ratio of true positive links to be used in the test set. This parameter is only relevant when `aTP` is false.
- `aTN` : Specifies whether to use all true negative links in the test set.
- `tnRatio` : Ratio of true negative links to be used in the test set. This parameter is only relevant when `aTN` is false.
- `seed` : The random number generator's seed.
- `preGenerateTPN` : Whether to pre-generate true positives and true negatives.

The set of true positive links is used as the set of positive instances in the test set, whereas the set of false positive links is used as the set of negative instances.

7.1.3 Creating test data by adding and removing edges

The last method is more flexible and can be used to create a new network by both adding and removing links. The method starts first by removing existing links, then proceeds to add new links:

```
createTestData(NetworkCSP refNet, double remRatio, double
    addRatio, bool keepConnected, bool aTP, double tpRatio, bool
    aTN, double tnRatio, LinkClass posClass, LinkClass negClass,
    long int seed, bool preGenerateTPN = true);
```

The parameters of this method are as follows:

- `refNet`: A constant shared pointer to the reference network.
- `remRatio`: Value between 0 and 1 that specifies the percentage of edges to be removed.
- `addRatio`: Value between 0 and 1 that specifies the percentage of edges to be added.
- `keepConnected`: Specifies whether to keep the network connected. If the reference network is disconnected or the ratio of edges to be removed is too large to keep the network connected, an exception is raised.
- `aTP`: Specifies whether to use all true positive links in the test set.
- `tpRatio`: Ratio of true positive links to be used in the test set. This parameter is only relevant when `aTP` is false.
- `aTN`: Specifies whether to use all true negative links in the test set.
- `tnRatio`: Ratio of true negative links to be used in the test set. This parameter is only relevant when `aTN` is false.
- `posClass`: Indicates which links will be considered the positive links.
- `negClass`: Indicates which links will be considered the negative links.
- `seed`: The random number generator's seed.
- `preGenerateTPN`: Whether to pre-generate true positives and true negatives.

■ **Example 7.1** Consider the network shown in the right side of Figure 7.1. The following code creates a distorted version of this network by adding and removing edges. The resulting network is shown in the right side of Figure 7.1.

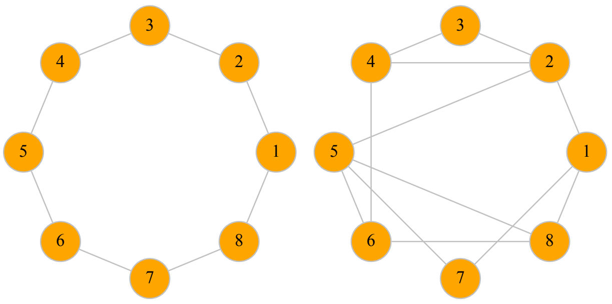

Figure 7.1: Example of network distortion. To the right, the reference network. To the left, the observed network.

```
#include <linkpred.hpp>
#include <iostream>
using namespace LinkPred;
```

```
int main(int argc, char*argv[]) {
  int n = 8;
  auto net = std::make_shared<UNetwork<>>();
  for (int i = 1; i <= n; i++) {
    std::string il = std::to_string(i);
    std::string jl = std::to_string(i % n + 1);
    net->addEdge(net->addNode(il).first, net->addNode(jl).first);
    jl = std::to_string((i + 1) % n + 1);
    net->addEdge(net->addNode(il).first, net->addNode(jl).first);
  }
  net->assemble();
  auto testData = NetworkManipulator<>::createTestData(net, 0.4,
     0.3, true, true, 0, true, 0, FN, TN, 777);
  std::cout << "Reference␣network:\n";
  testData.getRefNet()->print();
  std::cout << "Observed␣network:\n";
  testData.getObsNet()->print();
  std::cout << "Positive␣links:" << std::endl;
  for (auto it = testData.posBegin(); it != testData.posEnd(); ++
     it) {
    std::cout << net->getLabel(net->start(*it)) << "\t" << net->
       getLabel(net->end(*it)) << std::endl;
  }
  std::cout << "Negative␣links:" << std::endl;
  for (auto it = testData.negBegin(); it != testData.negEnd(); ++
     it) {
    std::cout << net->getLabel(net->start(*it)) << "\t" << net->
       getLabel(net->end(*it)) << std::endl;
  }
  return 0;
}
```

The following is the output of this code:

```
Reference network:
2	1
2	3
2	4
2	8
1	3
1	7
1	8
3	4
3	5
4	5
4	6
5	6
5	7
6	7
6	8
7	8
Observed network:
2	1
2	3
2	4
1	6
1	7
```

```
1       8
3       4
4       6
5       6
5       7
5       8
6       8
Positive links:
6       7
7       8
2       8
4       5
3       5
1       3
Negative links:
2       5
2       6
2       7
1       4
1       5
3       6
3       7
3       8
4       7
4       8
```

■

7.1.4 Loading test data from file

The method `loadTestData` allows to read test data from file:

```
loadTestData(std::string obsEdgesFileName, std::string
    remEdgesFileName, std::string addEdgesFileName, bool aTP,
    double tpRatio, bool aTN, double tnRatio, LinkClass posClass,
    LinkClass negClass, long int seed, bool preGenerateTPN = true)
    ;
```

The parameters of this method are as follows:

- `obsEdgesFileName` : A file containing the observed edges (edge list format).
- `remEdgesFileName` : A file containing the removed edges (edge list format). This is ignored if equal to empty string "".
- `addEdgesFileName` : A file containing the add edges (edge list format). This is ignored if equal to empty string "".
- `aTP` : Specifies whether to use all true positive links in the test set.
- `tpRatio` : Ratio of true positive links to be used in the test set. This parameter is only relevant when `aTP` is false.
- `aTN` : Specifies whether to use all true negative links in the test set.
- `tnRatio` : Ratio of true negative links to be used in the test set. This parameter is only relevant when `aTN` is false.
- `posClass` : Indicates which links will be considered the positive links.
- `negClass` : Indicates which links will be considered the negative links.
- `seed` : The random number generator's seed.
- `preGenerateTPN` : Whether to pre-generate true positives and true negatives.

■ **Example 7.2** This example shows how to load test data from file. Consider the test data shown in Figure 7.2.

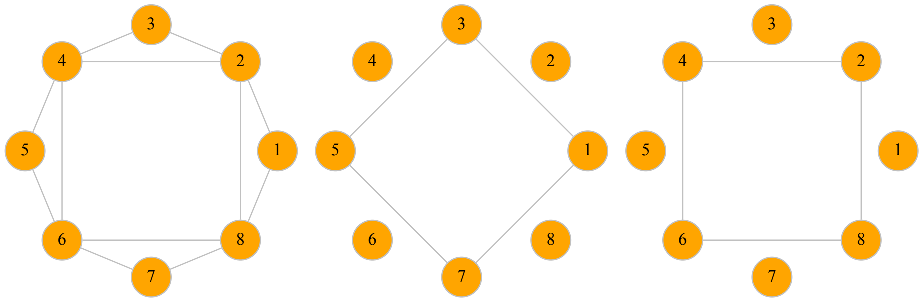

Figure 7.2: Example of test data to be loaded from file. Left: the observed edges. Middle: the removed edges. Right: added edges

This data is stored in three files. The file `net-obs.edges` contains the list of observed edges:

```
1       2
2       3
3       4
4       5
5       6
6       7
7       8
8       1
#The following are added (spurious) edges
2       4
4       6
6       8
8       2
```

The file `net-rem.edges` contains the list of removed edges:

```
1       3
3       5
5       7
7       1
```

The file `net-add.edges` contains the list of added edges:

```
2       4
4       6
6       8
8       2
```

In the following program, we use `loadTestData` to load this data from file. We will consider both added and removed edges and use false negative edges (removed edges) as the positive class and true negative edges as the negative class.

```
#include <linkpred.hpp>
#include <iostream>
using namespace LinkPred;
int main(int argc, char *argv[]) {
  std::cout << "Loading␣test␣data..." << std::endl;
```

```
  auto testData = NetworkManipulator<>::loadTestData("net-obs.
     edges", "net-rem.edges", "net-add.edges", true, 0, true, 0,
     FN, TN, 777, true);
  std::cout << "Test␣data␣reference␣network:\n";
  auto refNet = testData.getRefNet();
  refNet->print();
  std::cout << "Test␣data␣observed␣network:\n";
  auto obsNet = testData.getObsNet();
  obsNet->print();
  std::cout << "Positive␣links␣in␣the␣test␣set:\n";
  for (auto it = testData.posBegin(); it != testData.posEnd(); ++
     it) {
    std::cout << refNet->getLabel(refNet->start(*it)) << "\t" <<
       refNet->getLabel(refNet->end(*it)) << std::endl;
  }
  std::cout << "Negative␣links␣in␣the␣test␣set:\n";
  for (auto it = testData.negBegin(); it != testData.negEnd(); ++
     it) {
    std::cout << refNet->getLabel(refNet->start(*it)) << "\t" <<
       refNet->getLabel(refNet->end(*it)) << std::endl;
  }
  return 0;
}
```

The following is the output of this code:

```
Loading test data...
Test data reference network:
1       2
1       8
1       3
1       7
2       3
8       7
3       4
3       5
4       5
5       6
5       7
6       7
Test data observed network:
1       2
1       8
2       8
2       3
2       4
8       6
8       7
3       4
4       5
4       6
5       6
6       7
Positive links in the test set:
1       3
1       7
3       5
```

```
5       7
Negative links in the test set:
1       4
1       5
1       6
2       5
2       6
2       7
8       3
8       4
8       5
3       6
3       7
4       7
```

In the second program, we use true positive edges (non-added edges) as the positive class and false positive edges (added edges() as the negative class.

```
#include <linkpred.hpp>
#include <iostream>
using namespace LinkPred;
int main(int argc, char *argv[]) {
  std::cout << "Loading␣test␣data..." << std::endl;
  auto testData = NetworkManipulator<>::loadTestData("net-obs.
     edges", "net-rem.edges", "net-add.edges", true, 0, true, 0,
     TP, FP, 777, true);
  std::cout << "Test␣data␣reference␣network:\n";
  auto refNet = testData.getRefNet();
  refNet->print();
  std::cout << "Test␣data␣observed␣network:\n";
  auto obsNet = testData.getObsNet();
  obsNet->print();
  std::cout << "Positive␣links␣in␣the␣test␣set:\n";
  for (auto it = testData.posBegin(); it != testData.posEnd(); ++
     it) {
    std::cout << refNet->getLabel(refNet->start(*it)) << "\t" <<
       refNet->getLabel(refNet->end(*it)) << std::endl;
  }
  std::cout << "Negative␣links␣in␣the␣test␣set:\n";
  for (auto it = testData.negBegin(); it != testData.negEnd(); ++
     it) {
    std::cout << refNet->getLabel(refNet->start(*it)) << "\t" <<
       refNet->getLabel(refNet->end(*it)) << std::endl;
  }
  return 0;
}
```

The following is the output of this code:

```
Loading test data...
Test data reference network:
1       2
1       8
1       3
1       7
2       3
8       7
3       4
```

```
3       5
4       5
5       6
5       7
6       7
Test data observed network:
1       2
1       8
2       8
2       3
2       4
8       6
8       7
3       4
4       5
4       6
5       6
6       7
Positive links in the test set:
1       2
1       8
2       3
8       7
3       4
4       5
5       6
6       7
Negative links in the test set:
2       8
2       4
8       6
4       6
```

In the third program, we consider only removed edges and use false negative edges (removed edges) as the positive class and true negative edges as the negative class.

```
#include <linkpred.hpp>
#include <iostream>
using namespace LinkPred;
int main(int argc, char *argv[]) {
  std::cout << "Loading test data..." << std::endl;
  auto testData = NetworkManipulator<>::loadTestData("net-obs.
     edges", "net-rem.edges", "", true, 0, true, 0, FN, TN, 777,
     true);
  std::cout << "Test data reference network:\n";
  auto refNet = testData.getRefNet();
  refNet->print();
  std::cout << "Test data observed network:\n";
  auto obsNet = testData.getObsNet();
  obsNet->print();
  std::cout << "Positive links in the test set:\n";
  for (auto it = testData.posBegin(); it != testData.posEnd(); ++
     it) {
    std::cout << refNet->getLabel(refNet->start(*it)) << "\t" <<
       refNet->getLabel(refNet->end(*it)) << std::endl;
  }
  std::cout << "Negative links in the test set:\n";
```

```
  for (auto it = testData.negBegin(); it != testData.negEnd(); ++
      it) {
    std::cout << refNet->getLabel(refNet->start(*it)) << "\t" <<
        refNet->getLabel(refNet->end(*it)) << std::endl;
  }
  return 0;
}
```

The following is the output of this code:

```
Loading test data...
Test data reference network:
1	2
1	8
1	3
1	7
2	8
2	3
2	4
8	6
8	7
3	4
3	5
4	5
4	6
5	6
5	7
6	7
Test data observed network:
1	2
1	8
2	8
2	3
2	4
8	6
8	7
3	4
4	5
4	6
5	6
6	7
Positive links in the test set:
1	3
1	7
3	5
5	7
Negative links in the test set:
1	4
1	5
1	6
2	5
2	6
2	7
8	3
8	4
8	5
3	6
```

```
3       7
4       7
```

In the last program, we consider only added edges and use true positive edges (non-added edges) as the positive class and false positive edges (added edges() as the negative class.

```
#include <linkpred.hpp>
#include <iostream>
using namespace LinkPred;
int main(int argc, char *argv[]) {
  std::cout << "Loading␣test␣data..." << std::endl;
  auto testData = NetworkManipulator<>::loadTestData("net-obs.
     edges", "", "net-add.edges", true, 0, true, 0, TP, FP, 777,
     true);
  std::cout << "Test␣data␣reference␣network:\n";
  auto refNet = testData.getRefNet();
  refNet->print();
  std::cout << "Test␣data␣observed␣network:\n";
  auto obsNet = testData.getObsNet();
  obsNet->print();
  std::cout << "Positive␣links␣in␣the␣test␣set:\n";
  for (auto it = testData.posBegin(); it != testData.posEnd(); ++
     it) {
    std::cout << refNet->getLabel(refNet->start(*it)) << "\t" <<
       refNet->getLabel(refNet->end(*it)) << std::endl;
  }
  std::cout << "Negative␣links␣in␣the␣test␣set:\n";
  for (auto it = testData.negBegin(); it != testData.negEnd(); ++
     it) {
    std::cout << refNet->getLabel(refNet->start(*it)) << "\t" <<
       refNet->getLabel(refNet->end(*it)) << std::endl;
  }
  return 0;
}
```

The following is the output of this code:

```
Loading test data...
Test data reference network:
1       2
1       8
2       3
8       7
3       4
4       5
5       6
6       7
Test data observed network:
1       2
1       8
2       8
2       3
2       4
8       6
8       7
3       4
```

```
4       5
4       6
5       6
6       7
Positive links in the test set:
1       2
1       8
2       3
8       7
3       4
4       5
5       6
6       7
Negative links in the test set:
2       8
2       4
8       6
4       6
```

■

7.1.5 Creating test data from two snapshots of an evolving network

The class `NetworkManipulator` offers two other methods for generating test data by comparing two snapshots of the same network: `createTestDataSeq` and `createTestDataSeqInter`. These methods are useful when evaluating link prediction algorithms' performance on evolving networks, where the task is to predict future links. The prediction algorithm is trained using a snapshot `firstNet` of the network at a given time, and another snapshot `secondNet` taken later is used as a reference network to evaluate its performance. New nodes can appear in the second snapshot, and existing nodes can disappear, and the same goes for links. In the method `createTestDataSeq`, nodes that are not present in `secondNet` (the observed network) and edges incident to them are removed from the reference network. The method `createTestDataSeqInter`, on the other hand, only keeps nodes common to both networks.

The signatures of these methods are as follows:

```
createTestDataSeq(NetworkCSP firstNet, NetworkCSP secondNet, bool
    aTP, double tpRatio, bool aTN, double tnRatio, LinkClass
   posClass, LinkClass negClass, long int seed, bool
   preGenerateTPN = true);

createTestDataSeqInter(NetworkCSP firstNet, NetworkCSP secondNet,
    bool aTP, double tpRatio, bool aTN, double tnRatio, LinkClass
    posClass, LinkClass negClass, long int seed, bool
   preGenerateTPN = true);
```

The parameters of this method are as follows:

- `firstNet` The first network.
- `secondNet` The second network.
- `aTP`: Specifies whether to use all true positive links in the test set.
- `tpRatio`: Ratio of true positive links to be used in the test set. This parameter is only relevant when `aTP` is false.
- `aTN`: Specifies whether to use all true negative links in the test set.

- `tnRatio` : Ratio of true negative links to be used in the test set. This parameter is only relevant when `aTN` is false.
- `posClass` : Indicates which links will be considered the positive links.
- `negClass` : Indicates which links will be considered the negative links.
- `seed` : The random number generator's seed.
- `preGenerateTPN` : Whether to pre-generate true positives and true negatives.

■ **Example 7.3** Consider the two snapshots of the same evolving network shown in Figure 7.3.

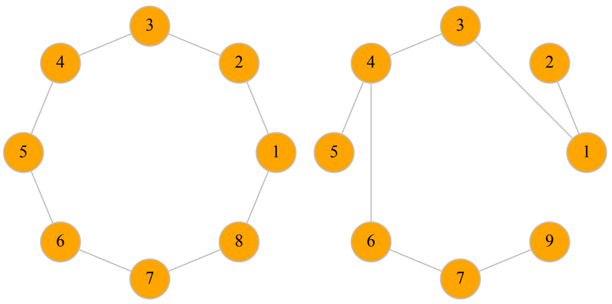

Figure 7.3: Two snapshots of the same evolving network. The first snapshot (left) is used to predict the second snapshot. Note that node 8 disappeared in snapshot 2, whereas node 9 has appeared.

In the following program, we use `createTestDataSeq` to generate test data to assess the performance of the algorithms in predicting links that will appear in the second snapshot.

```
#include <linkpred.hpp>
#include <iostream>
using namespace LinkPred;
int main(int argc, char *argv[]) {
  std::cout << "Reading␣networks...\n";
  auto net1 = UNetwork<>::read("net-seq1.edges");
  auto net2 = UNetwork<>::read("net-seq2.edges");
  std::cout << "First␣network:\n";
  net1->print();
  std::cout << "Second␣network:\n";
  net2->print();
  std::cout << "Creating␣test␣set.␣Detecting␣links␣that␣will␣
      appear:␣Positive␣class:␣FN.␣Negative␣class:␣TN\n";
  auto testData = NetworkManipulator<>::createTestDataSeq(net1,
      net2, true, 0, true, 0, FN, TN, 777, true);
  std::cout << "Test␣data␣reference␣network:\n";
  auto refNet = testData.getRefNet();
  refNet->print();
  std::cout << "Test␣data␣observed␣network:\n";
  auto obsNet = testData.getObsNet();
  obsNet->print();
  std::cout << "Positive␣links␣in␣the␣test␣set:\n";
  for (auto it = testData.posBegin(); it != testData.posEnd(); ++
      it) {
    std::cout << refNet->getLabel(refNet->start(*it)) << "\t" <<
        refNet->getLabel(refNet->end(*it)) << std::endl;
```

```
  }
  std::cout << "Negative␣links␣in␣the␣test␣set:\n";
  for (auto it = testData.negBegin(); it != testData.negEnd(); ++
     it) {
    std::cout << refNet->getLabel(refNet->start(*it)) << "\t" <<
       refNet->getLabel(refNet->end(*it)) << std::endl;
  }
  return 0;
}
```

The following is the output of this code:

```
Reading networks...
First network:
1       2
1       8
2       3
3       4
4       5
5       6
6       7
7       8
Second network:
1       2
1       3
3       4
4       6
4       5
6       7
7       9
Creating test set. Detecting links that will appear: Positive class: FN.
   Negative class: TN
Test data reference network:
1       2
1       3
3       4
4       5
4       6
6       7
Test data observed network:
1       2
1       8
2       3
3       4
4       5
5       6
6       7
7       8
Positive links in the test set:
1       3
4       6
Negative links in the test set:
1       4
1       5
1       6
1       7
2       4
```

```
2	5
2	6
2	7
2	8
3	5
3	6
3	7
3	8
4	7
4	8
5	7
5	8
6	8
```

In this second program, we use `createTestDataSeq` to generate test data to assess the performance of the algorithms in predicting links that will disappear in the second snapshot.

```
#include <linkpred.hpp>
#include <iostream>
using namespace LinkPred;
int main(int argc, char *argv[]) {
  std::cout << "Reading networks...\n";
  auto net1 = UNetwork<>::read("net-seq1.edges");
  auto net2 = UNetwork<>::read("net-seq2.edges");
  std::cout << "First network:\n";
  net1->print();
  std::cout << "Second network:\n";
  net2->print();
  std::cout << "Creating test set. Detecting links that will 
     disappear: Positive class: TP. Negative class: FP\n";
  auto testData = NetworkManipulator<>::createTestDataSeq(net1,
     net2, true, 0, true, 0, TP, FP, 777, true);
  std::cout << "Test data reference network:\n";
  auto refNet = testData.getRefNet();
  refNet->print();
  std::cout << "Test data observed network:\n";
  auto obsNet = testData.getObsNet();
  obsNet->print();
  std::cout << "Positive links in the test set:\n";
  for (auto it = testData.posBegin(); it != testData.posEnd(); ++
     it) {
    std::cout << refNet->getLabel(refNet->start(*it)) << "\t" <<
       refNet->getLabel(refNet->end(*it)) << std::endl;
  }
  std::cout << "Negative links in the test set:\n";
  for (auto it = testData.negBegin(); it != testData.negEnd(); ++
     it) {
    std::cout << refNet->getLabel(refNet->start(*it)) << "\t" <<
       refNet->getLabel(refNet->end(*it)) << std::endl;
  }
  return 0;
}
```

The following is the output of this code:

```
Reading networks...
```

```
First network:
1       2
1       8
2       3
3       4
4       5
5       6
6       7
7       8
Second network:
1       2
1       3
3       4
4       6
4       5
6       7
7       9
Creating test set. Detecting links that will disappear: Positive class: TP.
    Negative class: FP
Test data reference network:
1       2
1       3
3       4
4       5
4       6
6       7
Test data observed network:
1       2
1       8
2       3
3       4
4       5
5       6
6       7
7       8
Positive links in the test set:
1       2
3       4
4       5
6       7
Negative links in the test set:
1       8
2       3
5       6
7       8
```

In the third program, we use `createTestDataSeqInter` to generate test data to assess the performance of the algorithms in predicting links that will appear in the second snapshot.

```
#include <linkpred.hpp>
#include <iostream>
using namespace LinkPred;
int main(int argc, char *argv[]) {
  std::cout << "Reading␣networks...\n";
  auto net1 = UNetwork<>::read("net-seq1.edges");
```

```
  auto net2 = UNetwork<>::read("net-seq2.edges");
  std::cout << "First␣network:\n";
  net1->print();
  std::cout << "Second␣network:\n";
  net2->print();
  std::cout << "Creating␣test␣set.␣Detecting␣links␣that␣will␣
     appear:␣Positive␣class:␣FN.␣Negative␣class:␣TN\n";
  auto testData = NetworkManipulator<>::createTestDataSeqInter(
     net1, net2, true, 0, true, 0, FN, TN, 777, true);
  std::cout << "Test␣data␣reference␣network:\n";
  auto refNet = testData.getRefNet();
  refNet->print();
  std::cout << "Test␣data␣observed␣network:\n";
  auto obsNet = testData.getObsNet();
  obsNet->print();
  std::cout << "Positive␣links␣in␣the␣test␣set:\n";
  for (auto it = testData.posBegin(); it != testData.posEnd(); ++
     it) {
    std::cout << refNet->getLabel(refNet->start(*it)) << "\t" <<
       refNet->getLabel(refNet->end(*it)) << std::endl;
  }
  std::cout << "Negative␣links␣in␣the␣test␣set:\n";
  for (auto it = testData.negBegin(); it != testData.negEnd(); ++
     it) {
    std::cout << refNet->getLabel(refNet->start(*it)) << "\t" <<
       refNet->getLabel(refNet->end(*it)) << std::endl;
  }
  return 0;
}
```

The following is the output of this code:

```
Reading networks...
First network:
1       2
1       8
2       3
3       4
4       5
5       6
6       7
7       8
Second network:
1       2
1       3
3       4
4       6
4       5
6       7
7       9
Creating test set. Detecting links that will appear: Positive class: FN.
   Negative class: TN
Test data reference network:
1       2
1       3
3       4
4       6
```

```
4       5
6       7
Test data observed network:
1       2
2       3
3       4
4       5
6       5
6       7
Positive links in the test set:
1       3
4       6
Negative links in the test set:
1       4
1       6
1       5
1       7
2       4
2       6
2       5
2       7
3       6
3       5
3       7
4       7
5       7
```

In the last program, we use `createTestDataSeqInter` to generate test data to assess the performance of the algorithms in predicting links that will disappear in the second snapshot.

```
#include <linkpred.hpp>
#include <iostream>
using namespace LinkPred;
int main(int argc, char *argv[]) {
  std::cout << "Reading networks...\n";
  auto net1 = UNetwork<>::read("net-seq1.edges");
  auto net2 = UNetwork<>::read("net-seq2.edges");
  std::cout << "First network:\n";
  net1->print();
  std::cout << "Second network:\n";
  net2->print();
  std::cout << "Creating test set. Detecting links that will 
     disappear: Positive class: TP. Negative class: FP\n";
  auto testData = NetworkManipulator<>::createTestDataSeqInter(
     net1, net2, true, 0, true, 0, TP, FP, 777, true);
  std::cout << "Test data reference network:\n";
  auto refNet = testData.getRefNet();
  refNet->print();
  std::cout << "Test data observed network:\n";
  auto obsNet = testData.getObsNet();
  obsNet->print();
  std::cout << "Positive links in the test set:\n";
  for (auto it = testData.posBegin(); it != testData.posEnd(); ++
     it) {
    std::cout << refNet->getLabel(refNet->start(*it)) << "\t" <<
```

```
        refNet->getLabel(refNet->end(*it)) << std::endl;
  }
  std::cout << "Negative␣links␣in␣the␣test␣set:\n";
  for (auto it = testData.negBegin(); it != testData.negEnd(); ++
      it) {
    std::cout << refNet->getLabel(refNet->start(*it)) << "\t" <<
        refNet->getLabel(refNet->end(*it)) << std::endl;
  }
  return 0;
}
```

The following is the output of this code:

```
Reading networks...
First network:
1       2
1       8
2       3
3       4
4       5
5       6
6       7
7       8
Second network:
1       2
1       3
3       4
4       6
4       5
6       7
7       9
Creating test set. Detecting links that will disappear: Positive class: TP.
    Negative class: FP
Test data reference network:
1       2
1       3
3       4
4       6
4       5
6       7
Test data observed network:
1       2
2       3
3       4
4       5
6       5
6       7
Positive links in the test set:
1       2
3       4
4       5
6       7
Negative links in the test set:
2       3
5       6
```

■

7.2 Prediction results

For performance purposes and to avoid redundant computations, link prediction results are stored in an object of the class `PredResults`. The constructor of this class takes int two parameters a `TestData` object and an `std::shared_ptr` to a link predictor. The most important methods provided by this class are:

- `bool isPosComputed()const`: Check whether the positive links scores have been computed.
- `void compPosScores()`: Compute the scores of positive links. The method performs the computation only once.
- `bool isNegComputed()const`: Check whether the negative links scores have been computed.
- `void compNegScores()`: Compute the scores of negative links. The method performs the computation only once.
- `SortStatus getNegSortStatus()const`: Return the sort status of negative links scores. The type `SortStatus` is an enumeration containing the following values:

```
enum SortStatus {
  None, /**< Not sorted. */
  Inc, /**< Sorted in increasing order. */
  Dec /**< Sorted in decreasing order. */
};
```

- `void sortNeg(SortStatus negSortStatus)`: Sort the negative links scores according to the specified sorting direction. The method only sorts the scores if necessary.
- `SortStatus getPosSortStatus()const`: Return the sort status of positive links scores.
- `void sortPos(SortStatus posSortStatus)`: Sort the positive links scores according to the specified sorting direction. The method only sorts the scores if necessary.

7.3 Performance measures

All performance measures in LinkPred inherit from the abstract class `PerfMeasure`. Every performance should be uniquely identified by its name, which can be passed as parameter to the constructor. The most important method in the class `PerfMeasure` is `eval` which evaluates the value of the performance measure given an object `predResult` (see Section 7.1). The results of the performance measure are written to an object of type `PerfResults` passed as the second parameter of the method. The class `PerfResults` is defined as `std::map<std::string, double>`. This allows the possibility of associating several result values with a single performance measure.

An important class of performance measures are performance curves such as the receiver operating characteristic (ROC) curve and the precision-recall (PR) curve. These are represented by the abstract class `PerfCurve`, which inherits from the class `PerfMeasure`. The class `PerfCurve` defines a new virtual method:

```
virtual std::vector<std::pair<double, double>> getCurve(std::
    shared_ptr<PredResultsT>& predResults) = 0;
```

which returns the performance curve in the form of an `std::vector` of points. Each data point is represented by an `std::pair`, where the first element is the x coordinate, whereas the second element is the corresponding y coordinate. Although not mandatory, the

area under the curve, computed using numerical integration, is the typical performance value associated with a performance curve, and its is that value which is returned by the method `eval`.

LinkPred includes the implementation of number of performance measures, including the most important performance curves (ROC and PR) and a generic (parameterized) curve class. These performance measures are presented in the rest of this section.

7.3.1 Receiver operating characteristic curve (ROC)

One of the most important performance measure used in the field of link prediction is the receiver operating (ROC) curve, in which the true positive rate (recall) is plotted against the false positive rate. The ROC curve can be computed using the class `ROC`. The following code show how to calculate the ROC curve and the associate area under the curve. Notice that the two operations are independent of each other, and if the AUC is the only result required, it is enough (and computationally better) to call the method `eval`. An example ROC curve obtained using this code is plotted in Figure 7.4.

```
#include <linkpred.hpp>
#include <iostream>
using namespace LinkPred;
int main(int argc, char*argv[]) {
  std::string netFileName(argv[1]);
  auto fullNet = UNetwork<>::read(netFileName, false, true);
  auto testData = NetworkManipulator<>::createTestData(fullNet,
     0.3, 0, true, true, 0, true, 0, FN, TN, 777);
  testData.lock();
  auto predictor = std::make_shared<UHRGPredictor<>>(testData.
     getObsNet(), 333);
  predictor->init();
  predictor->learn();
  auto predResults = std::make_shared<PredResults<>>(testData,
     predictor);
  auto roc = std::make_shared<ROC<>>("ROC");
  auto curve = roc->getCurve(predResults);
  std::cout << "#x\ty\n";
  for (std::size_t i = 0; i < curve.size(); i++) {
    std::cout << curve[i].first << "\t" << curve[i].second << std
       ::endl;
  }
  PerfResults res;
  roc->eval(predResults, res);
  std::cout << "#ROCAUC:␣" << res.at(roc->getName()) << std::endl
     ;
  return 0;
}
```

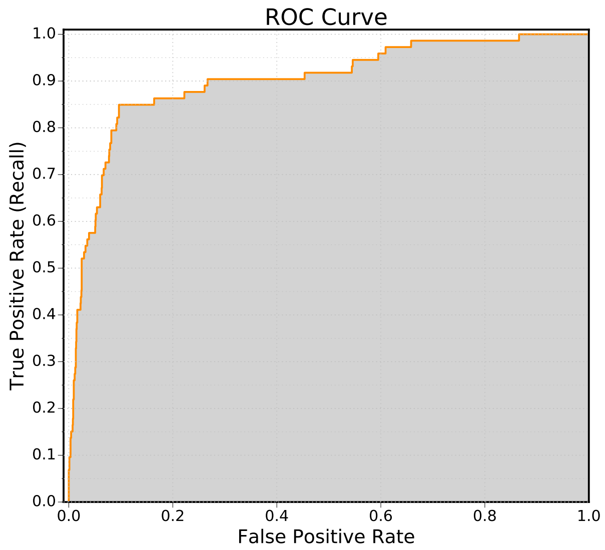

Figure 7.4: Example ROC curve. The area under the curve (shown in gray) is the value associated with this performance curve.

The default behavior of the `ROC` performance measure is to compute the positive and negative edge scores and then compute the area under the curve. This may lead to memory issues with large graphs. To compute the ROCAUC without storing both types of scores, the class `ROC` offers a method that *streams* scores without storing them. To enable this method, call `setStrmEnabled(bool)` on the `ROC` object. To specify which scores to steam use the method `setStrmNeg(bool)`. By default the negative scores are streamed, while the positive scores are stored. Passing **false** to `setStrmNeg` switches this.

■ **Example 7.4** This is an example of using the streaming method with `ROC`.

```
#include <linkpred.hpp>
#include <iostream>
using namespace LinkPred;
int main(int argc, char *argv[]) {
  std::string netFileName(argv[1]);
  auto refNet = UNetwork<>::read(netFileName);
  auto testData = NetworkManipulator<>::createTestData(refNet,
      0.1, 0, false, true, 0, true, 0, FN, TN, 777, false);
  testData.lock();
  auto predictor = std::make_shared<UADAPredictor<>>(testData.
      getObsNet());
  predictor->init();
  predictor->learn();
  auto predResults = std::make_shared<PredResults<>>(testData,
      predictor);
  auto roc = std::make_shared<ROC<>>("ROC");
  roc->setStrmEnabled(true);
  PerfResults res;
  roc->eval(predResults, res);
  std::cout << "#ROCAUC␣(streaming):␣" << res.at(roc->getName())
      << std::endl;
  return 0;
```

```
}
```

■

In addition to consuming little memory, the streaming method supports distributed processing (in addition to shared memory parallelism), which makes it suitable for large networks (see Chapter 8).

7.3.2 Precision-recall curve

The precision-recall (PR) curve is also a widely used measure of performance of link prediction algorithms. In this curve, the precision is plotted as a function of the recall. The PR curve can be computed using the class `PR`. The area under the PR curve can be computed using two integration methods:

- The trapezoidal rule which assumes a linear interpolation between the PR points.
- Nonlinear interpolation as proposed by Jesse Davis and Mark Goadrich [9].

The second method is more accurate, as linear integration tends to overestimate the area under the curve [9]. Furthermore, the implementation of Davis-Goadrich nonlinear interpolation in LinkPred ensures little to no additional cost compared to the trapezoidal method. Nevertheless, the user can choose the integration method using the method `void setInterpolMethod(InterpolMethod interpolMethod)`. The active integration method can be queried using `InterpolMethod getInterpolMethod()const`. The type `InterpolMethod` is a public enumeration of the class `PR` with two possible values:

```
enum InterpolMethod {
  LIN, /**< Linear interpolation (Trapezoidal rule). */
  DGI /**< Davis-Goadrich nonlinear interpolation. */
};
```

By default, the integration method used is `DGI`.

The following code shows how to calculate the PR curve and the associate area under the curve. Notice that the two operations are independent of each other, and if the AUC is the only result required, it is enough to call the method `eval`. An example PR curve obtained using this code is plotted in Figure 7.5.

```
#include <linkpred.hpp>
#include <iostream>
using namespace LinkPred;
int main(int argc, char*argv[]) {
  std::string netFileName(argv[1]);
  auto fullNet = UNetwork<>::read(netFileName, false, true);
  auto testData = NetworkManipulator<>::createTestData(fullNet,
      0.3, 0, true, true, 0, true, 0, FN, TN, 777);
  testData.lock();
  auto predictor = std::make_shared<UHRGPredictor<>>(testData.
      getObsNet(), 333);
  predictor->init();
  predictor->learn();
  auto predResults = std::make_shared<PredResults<>>(testData,
      predictor);
  auto pr = std::make_shared<PR<>>("PR");
  auto curve = pr->getCurve(predResults);
  std::cout << "#x\ty\n";
  for (std::size_t i = 0; i < curve.size(); i++) {
```

```
    std::cout << curve[i].first << "\t" << curve[i].second << std
        ::endl;
  }
  PerfResults res;
  pr->eval(predResults, res);
  std::cout << "#PRAUC:␣" << res.at(pr->getName()) << std::endl;
  return 0;
}
```

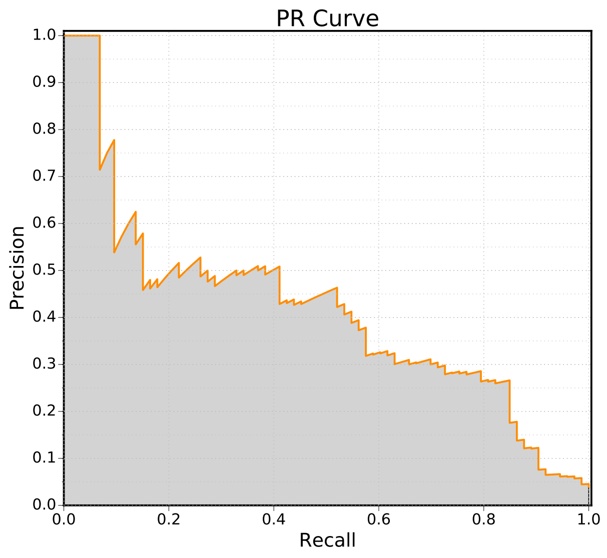

Figure 7.5: Example PR curve. The area under the curve (shown in gray) is the value associated with this performance curve.

7.3.3 General performance curves

LinkPred offers the possibility of calculating general performance curves using the class `GCurve`. A performance curve is in general defined by giving the x and y coordinates functions. These are passed as parameters -in the form of lambdas- to the constructor of the class `GCurve`. The associated performance value is the area under the curve computed using the trapezoidal rule (linear interpolation). For example, the ROC curve can be defined as:

```
GCurve<> cur(fpr, rec, "ROC");
```

The two first parameters of the constructors are lambdas having the signature:

```
double(std::size_t tp, std::size_t fn, std::size_t tn, std::
    size_t fp, std::size_t P, std::size_t N)
```

where:

- `tp`: Number of true positives.
- `fn`: Number of false negatives.
- `tn`: Number of true negatives.
- `fp`: Number of false positives.
- `P`: Number of positives. Notice that: `P` = `tp` + `fn`.
- `N`: Number of negatives. Here also: Notice that: `N` = `tn` + `fp`.

LinkPred contains the definition of several useful lambdas that can be used to define performance curves. These are defined in the name space `PerfLambda`:

- Recall (`rec`):

$$\frac{tp}{P}. \tag{7.1}$$

- False positive rate (`fpr`):

$$\frac{fp}{N}. \tag{7.2}$$

- Precision (`pre`):

$$\frac{tp}{tp+fp}. \tag{7.3}$$

- False negative rate (`fnr`):

$$\frac{fn}{P}. \tag{7.4}$$

- True negative rate (`tnr`):

$$\frac{tn}{N}. \tag{7.5}$$

- False omission rate (`fmr`):

$$\frac{fn}{tn+fn}. \tag{7.6}$$

- Accuracy (`acc`):

$$\frac{tp+tp}{P+N}. \tag{7.7}$$

- False discovery rate (`fdr`):

$$\frac{fp}{tp+fp}. \tag{7.8}$$

- Negative predictive value (`npv`):

$$\frac{tn}{tn+fn}. \tag{7.9}$$

Notice that some of these functions may be undefined for certain boundary values of the threshold, and therefore particular care must be taken when using them with `GCurve`. In particular, the curve, and consequently the area under it, may become undefined in some cases. For instance, it is possible to define the PR curve using `GCurve` in the same way we previously defined the ROC curve:

```
GCurve<> pr(rec, pre, "PR");
```

However, there are two important reasons to avoid such practice. First, as explained before, the area under the curve may be undefined in some cases. Second, the class `PR` offers a more accurate method for calculating the area under the curve (Davis-Goadrich interpolation) than the method used by `GCurve` (trapezoidal rule).

The following code shows how to calculate the ROC curve with negatives replacing positives (we denote this curve by NROC) and the associated area under the curve. This can be achieved by using `GCurve` with `fnr` (false negative rate) as the x-coordinates and `tnr` (true negative rate) as the y-coordinates. An example NROC curve obtained using this code is plotted in Figure 7.6.

```
#include <linkpred.hpp>
#include <iostream>
using namespace LinkPred;
int main(int argc, char*argv[]) {
  std::string netFileName(argv[1]);
  auto fullNet = UNetwork<>::read(netFileName, false, true);
  auto testData = NetworkManipulator<>::createTestData(fullNet,
     0.3, 0, true, true, 0, true, 0, FN, TN, 777);
  testData.lock();
  auto predictor = std::make_shared<UHRGPredictor<>>(testData.
     getObsNet(), 333);
  predictor->init();
  predictor->learn();
  auto predResults = std::make_shared<PredResults<>>(testData,
     predictor);
  auto nroc = std::make_shared<GCurve<>>(PerfLambda::fnr,
     PerfLambda::tnr, "NROC");
  auto curve = nroc->getCurve(predResults);
  std::cout << "#x\ty\n";
  for (std::size_t i = 0; i < curve.size(); i++) {
    std::cout << curve[i].first << "\t" << curve[i].second << std
       ::endl;
  }
  PerfResults res;
  nroc->eval(predResults, res);
  std::cout << "#NROCAUC: " << res.at(nroc->getName()) << std::
     endl;
  return 0;
}
```

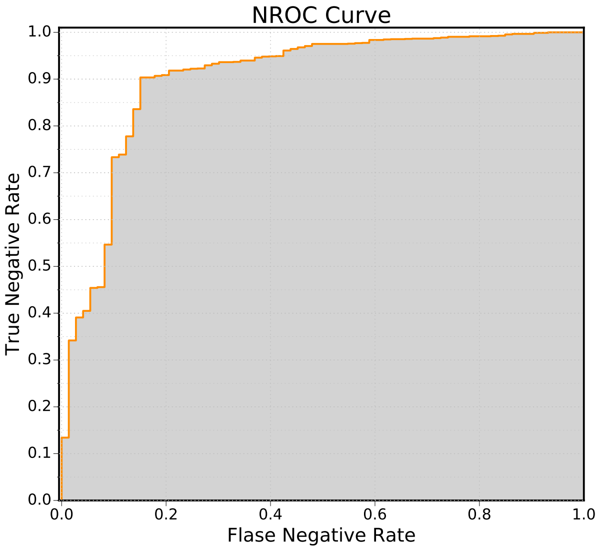

Figure 7.6: Example ROC curve with negative instances replacing positive ones. The area under the curve (shown in gray) is the value associated with this performance curve.

7.3.4 Top precision

The top precision measure is defined as the ratio of true positives within the top l scored edges, $l > 0$ being a parameter of the measure (usually l is set to the number of links removed from the network). Top precision is implemented by the class `TPR`, and since it is not a curve measure, this class inherits directly from `PerfMeasure`. The class `TPR` offers two approaches for computing top-precision. The first approach requires computing the score of all negative links, whereas the second approach calls the method `top` of the predictor. The first approach is in general more precise than the second one but may require more memory and time. The reason behind this is that it is possible to write efficient implementations of the method `top` (finding top scored edges) for most prediction algorithms. Indeed for most link predictors computing top scored edges does not require generating true negative links nor computing their scores. As a result, the second approach is the performance measure of choice for very large networks. Note that if `ROC` or `PR` are requested, it is better to use the first approach, since the computation of these two performance measures require the computation of the scores of all negative links anyways. To toggle between the two approaches simply call `setUseTopMethod`.

R The implementation of the method `top` in link predictors may be biased based on node IDs. This may skew the results when computing top-precision. To obtain unbiased results over multiple runs, it is advised to reshuffle the node IDs from run to run. This can be done by simply calling the method `shuffle` on the reference network between runs.

The following code shows how to use the class `TPR` using the first approach.

```
#include <linkpred.hpp>
#include <iostream>
using namespace LinkPred;
int main(int argc, char*argv[]) {
```

```
  std::string netFileName(argv[1]);
  auto fullNet = UNetwork<>::read(netFileName, false, true);
  auto testData = NetworkManipulator<>::createTestData(fullNet,
     0.3, 0, true, true, 0, true, 0, FN, TN, 777);
  testData.lock();
  auto predictor = std::make_shared<UHRGPredictor<>>(testData.
     getObsNet(), 333);
  predictor->init();
  predictor->learn();
  auto predResults = std::make_shared<PredResults<>>(testData,
     predictor);
  auto tpr = std::make_shared<TPR<>>(testData.getNbPos(), "TPR");
  PerfResults res;
  tpr->eval(predResults, res);
  std::cout << "TPR:␣" << res.at(tpr->getName()) << std::endl;
  return 0;
}
```

In the next code, we compute top-precision using the method `top`:

```
#include <linkpred.hpp>
#include <iostream>
using namespace LinkPred;
int main(int argc, char*argv[]) {
  std::string netFileName(argv[1]);
  auto fullNet = UNetwork<>::read(netFileName, false, true);
  auto testData = NetworkManipulator<>::createTestData(fullNet,
     0.3, 0, true, true, 0, true, 0, FN, TN, 777);
  testData.lock();
  auto predictor = std::make_shared<URALPredictor<>>(testData.
     getObsNet());
  predictor->init();
  predictor->learn();
  auto predResults = std::make_shared<PredResults<>>(testData,
     predictor);
  auto tpr = std::make_shared<TPR<>>(testData.getNbPos(),"TPRT");
  tpr->setUseTopMethod(true);
  PerfResults res;
  tpr->eval(predResults, res);
  std::cout << "TPRT:␣" << res.at(tpr->getName()) << std::endl;
  return 0;
}
```

7.4 Performance evaluation classes

LinkPred offers two helper classes that simplify the task of evaluating and comparing the performance of link prediction algorithms: `PerfEvaluator` and `PerfEvalExp`.

7.4.1 The class `PerfEvalExp`

The class `PerfEvalExp` allows to evaluate the performance of several link predictors based on several performance measures. The experimental setting in `PerfEvalExp` consists in removing a certain ratio of existing links from a reference network and presenting the algorithms with the obtained network (the observed networks). The

performance measures specified by the user are then applied to assess the predictive power of the algorithms. The parameters of the experiment are passed to `PerfEvalExp` as an instance of the `struct` named `PerfEvalExpDesc`. These include the reference network, the number of iterations, the range of removal ratios (defined as `ratioStart`, `ratioEnd` and `ratioStep`), the ratio of false negative links and true negative links used in the test set, etc.

```
template<typename Network=UNetwork<>> struct PerfeEvalExpDescp{
  ...
  std::shared_ptr<Network> refNet;
  std::size_t nbTestRuns = 1;
  double ratioStart = 0.1;
  double ratioEnd = 0.1;
  double ratioStep = 0.1;
  bool keepConnected = false;
  double fnRatio = 1;
  double tnRatio = 1;
  bool timingEnabled = false;
  long int seed = 0;
  std::ostream* out = &std::cout;
};
```

`PerfEvalExp` requires also a callback object to create link predictors and performance measures. This object must implement the interface `PEFactory`, which contains two methods one for creating link predictors `getPredictors` and the other for creating performance measures `getPerfMeasures`:

```
template<...> class PEFactory {
public:
  virtual std::vector<std::shared_ptr<LPredictorT>> getPredictors
     (std::shared_ptr<Network const> obsNet) = 0;
  virtual std::vector<std::shared_ptr<PerfMeasureT>>
     getPerfMeasures(TestDataT const & testData) = 0;
};
```

■ **Example 7.5** The following code shows how to use `PerfEvalExp` to compare three link prediction methods (ADA, JID and RAL) using top-precision (calling the `top` method). The experiment is repeated ten times and the default ratio of removed edges is used (0.1).

```
#include <linkpred.hpp>
#include <iostream>
#include <vector>
#include <memory>
using namespace LinkPred;
class Factory: public PEFactory<> {
public:
  virtual std::vector<std::shared_ptr<ULPredictor<>>>
     getPredictors(std::shared_ptr<UNetwork<> const> obsNet) {
    std::vector<std::shared_ptr<ULPredictor<>>> prs;
    prs.push_back(std::make_shared<UADAPredictor<>>(obsNet));
    prs.push_back(std::make_shared<UJIDPredictor<>>(obsNet));
    prs.push_back(std::make_shared<URALPredictor<>>(obsNet));
    return prs;
  }
```

```
  virtual std::vector<std::shared_ptr<PerfMeasure<>>>
     getPerfMeasures(TestData<> const & testData) {
    std::vector<std::shared_ptr<PerfMeasure<>>> pms;
    auto tpr = std::make_shared<TPR<>>(testData.getNbPos(), "TPRT
       ");
    tpr->setUseTopMethod(true);
    pms.push_back(tpr);
    return pms;
  }
  virtual ~Factory() = default;
};
int main(int argc, char*argv[]) {
  PerfeEvalExpDescp<> ped;
  ped.refNet = UNetwork<>::read("Infectious.edges");
  ped.nbTestRuns = 10;
  ped.seed = 777;
  auto factory = std::make_shared<Factory>();
  PerfEvalExp<> exp(ped, factory);
  exp.run();
  return 0;
}
```

The output of this code is as follows:

```
# n: 410 m: 2765
#ratio  TPRTADA TPRTJID TPRTRAL
0.10    0.3225  0.3225  0.3297
0.10    0.3225  0.3696  0.3514
0.10    0.3370  0.3406  0.3406
0.10    0.3188  0.3406  0.3297
0.10    0.3007  0.3623  0.3297
0.10    0.3188  0.3406  0.3442
0.10    0.3406  0.3370  0.3551
0.10    0.3116  0.3225  0.3442
0.10    0.3841  0.3696  0.3949
0.10    0.3406  0.3478  0.3696
#Time: 688.532 ms
```

■

Enabling timing in `PerfEvalExp` (by setting `timingEnabled` to true), results in three time measures being calculated:

- `ITN` (Init Time Nano): The time spent in the method `init` in nanoseconds.
- `LTN` (Learn Time Nano): The time spent in the method `learn` in nanoseconds.
- `PTN` (Predict Time Nano): The time spent in the method `predict` in nanoseconds.

Since different predictors split the processing differently between the three methods, time comparison should be based on the sum of the three methods rather than that of a single one.

■ **Example 7.6** The following code shows how to use `PerfEvalExp` to compare the time performance of two algorithms ADA and HRG (note that no learning performance measures are used). The experiment is repeated ten times and the default ratio of removed edges is used (0.1).

```
#include <linkpred.hpp>
#include <iostream>
```

```
#include <vector>
#include <memory>
using namespace LinkPred;
class Factory: public PEFactory<> {
public:
  virtual std::vector<std::shared_ptr<ULPredictor<>>>
     getPredictors(std::shared_ptr<UNetwork<> const> obsNet) {
    std::vector<std::shared_ptr<ULPredictor<>>> prs;
    prs.push_back(std::make_shared<UADAPredictor<>>(obsNet));
    prs.push_back(std::make_shared<UHRGPredictor<>>(obsNet, 333))
        ;
    return prs;
  }
  virtual std::vector<std::shared_ptr<PerfMeasure<>>>
     getPerfMeasures(TestData<> const & testData) {
    std::vector<std::shared_ptr<PerfMeasure<>>> pms;
    return pms;
  }
  virtual ~Factory() = default;
};
int main(int argc, char*argv[]) {
  PerfeEvalExpDescp<> ped;
  ped.refNet = UNetwork<>::read("Zakarays_Karate_Club.edges");
  ped.nbTestRuns = 10;
  ped.seed = 777;
  ped.timingEnabled = true;
  auto factory = std::make_shared<Factory>();
  PerfEvalExp<> exp(ped, factory);
  exp.run();
  return 0;
}
```

The output of this code is as follows:

```
# n: 34 m: 78
#ratio  ITNADA  ITNHRG  LTNADA  LTNHRG  PTNADA  PTNHRG  TTNADA  TTNHRG
0.10    344     608972  145     2242406166      32789   16438   33278
    2243031576
0.10    309     137817  74      1528222331      9713    22062   10096
    1528382210
0.10    305     139456  78      1505922677      8470    16297   8853
    1506078430
0.10    315     181203  77      2240478339      8308    21287   8700
    2240680829
0.10    465     142991  80      1861618290      9255    17122   9800
    1861778403
0.10    293     141502  76      1623068910      9964    19323   10333
    1623229735
0.10    319     145763  80      1628975047      10742   27264   11141
    1629148074
0.10    298     146415  78      1974976422      9563    18218   9939
    1975141055
0.10    287     142000  80      1729851112      9260    15397   9627
    1730008509
0.10    290     146339  78      2402748124      9593    15082   9961
    2402909545
#Time: 18745.5 ms
```

■

R The time spent in computing the performance measures is not computed as it is not part of the link prediction task.

R Enabling timing causes automatically the computation of scores for all links in the test set. If you add a performance measure that does not require these scores but rather calls directly the link predictor methods (such as top-precision with the option `useTopMethod` enabled), then the time spent in these methods is not measured.

7.4.2 The class `PerfEvaluator`

The class `PerfEvaluator` offers more flexibility than `PerfEvalExp`, since it takes the object `TestData` as input. However, `PerfEvaluator` performs a single iteration comparison, and it is up to he user to repeat the experiment. To use the class `PerfEvaluator`, we proceed as follows:

1. First, the `TestData` object is passed as parameter to the constructor:

```
PerfEvaluator<> perf(testData);
```

2. Add the link predictors:

```
perf.addPredictor(std::make_shared<ADAPredictor<>>(testData
    .getObsNet()));
perf.addPredictor(std::make_shared<CNEPredictor<>>(testData
    .getObsNet()));
```

3. Add the performance measures:

```
perf.addPerfMeasure(std::make_shared<ROC<>>());
perf.addPerfMeasure(std::make_shared<PR<>>());
```

   Notice that this step can be exchanged or interleaved with the previous one.

4. Run the evaluation (the predictors are initialized by the performance evaluator):

```
perf.eval();
```

   The evaluator can be set to take time measurements by enabling timing before running the method `eval`:

```
perf.setTimeEnabled(true); // Enable timing. Timing is
    disabled by default.
perf.eval();
```

   Three time measures are calculated by `PerfEval`:
   - `ITN` (Init Time Nano): The time spent in the method `init` in nanoseconds.
   - `LTN` (Learn Time Nano): The time spent in the method `learn` in nanoseconds.
   - `PTN` (Predict Time Nano): The time spent in the method `predict` in nanoseconds.

   Since different predictors split the processing differently between the three methods, time comparison should be based on the sum of the three methods rather than that of a single one.

5. Finally, retrieve the performance values. The class `PerfEval` provides a range for retrieving results: `resultsBegin()` and `resultsEnd()`. The provided iterator points to a pair the first element of which is the name of the performance measure (of type `std::string`), and the second element is the value of the measure (of type **double**). Since there are several predictors, the name of the performance measures results reported is the concatenation of the performance name (`measure->getName()`) and the predictor's name (`predictor->getName()`).

The following code shows how to use `PerfEval` to evaluate the performance of two link predictors using two measures.

```
#include <linkpred.hpp>
#include <iostream>
using namespace LinkPred;
int main(int argc, char*argv[]) {
  std::string netFileName(argv[1]);
  long int seed = std::atol(argv[2]);
  std::size_t nbTests = std::atol(argv[3]);
  RandomGen rng(seed);
  auto fullNet = UNetwork<>::read(netFileName, false, true);
  for (std::size_t i = 0; i < nbTests; i++) {
    auto testData = NetworkManipulator<>::createTestData(fullNet,
        0.1, 0, true, true, 0, true, 0, FN, TN, rng.getInt());
    testData.lock();
    PerfEvaluator<> perf(testData);

    perf.addPredictor(std::make_shared<UADAPredictor<>>(testData.
       getObsNet()));
    perf.addPredictor(std::make_shared<UCNEPredictor<>>(testData.
       getObsNet()));

    perf.addPerfMeasure(std::make_shared<ROC<>>());
    perf.addPerfMeasure(std::make_shared<PR<>>());

    perf.eval();

    if (i == 0) {
      std::cout << "#";
      for (auto it = perf.resultsBegin(); it != perf.resultsEnd()
          ; ++it) {
        std::cout << it->first << "\t";
      }
      std::cout << std::endl;
    }
    for (auto it = perf.resultsBegin(); it != perf.resultsEnd();
       ++it) {
      std::cout << std::setprecision(4) << it->second << "\t";
    }
    std::cout << std::endl;
  }
  return 0;
}
```

This is an example output of this code:

```
#ADAPR   ADAROC   CNEPR    CNEROC
0.8064   0.9694   0.8036   0.9637
```

```
0.7427  0.9878  0.6466  0.9704
0.8079  0.9687  0.7557  0.9573
0.7892  0.991   0.7871  0.9839
0.8453  0.9703  0.8122  0.9677
0.729   0.8982  0.6902  0.8964
0.7356  0.9768  0.6272  0.9557
0.8014  0.9641  0.7294  0.9504
0.7796  0.9614  0.7378  0.9624
0.6416  0.9348  0.5392  0.9221
```

8. Parallelism, Templates and Library Extension

This chapter deals with time performance issues and how to harness the power of LinkPred on parallel/distributed machines. This may become a necessity when dealing with large data as predicting links in large networks can be time and memory consuming even for the most efficient of algorithms. We will also discuss template arguments of LinkPred classes and how to add new instantiations to the library. Finally, we will show how to extend the library with new prediction algorithms.

8.1 Parallelism

LinkPred offers two types of parallelism, shared memory parallelism using OpenMP, and distributed parallelism via MPI. These two types of parallelism can be used in conjunction or separately, or completely disabled at compilation time.

8.1.1 Shared memory parallelism

Most LinkPred classes support shared memory parallelism using OpenMP for the computationally intensive parts of their code. Enabling parallelism can result in significant improvement in running time. However, depending on the algorithms implemented in the methods under consideration, parallelism may result in different degrees of speedup. For example, Figure 8.1 shows the running time speed up obtained using parallel execution of four link predictors on the Yeast network. Once can see that different algorithms exhibit different speed ups depending on the details of the prediction procedure.

Instead of a using a ”global switch” to enable and disable parallelism, LinkPred offers a fine grain control of parallelism at the object level. This allows more flexibility to handle different use scenarios. To turn on parallelism for a predictor, we need to call the method `setParallel`. Notice that by default parallelism is turned off in all classes.

```
predictor->setParallel(true);
```

The same applies for performance measures:

```
measure->setParallel(true);
```

There are other classes in LinkPred that support parallelism, and they can all be set to run their code in parallel using the method `setParallel`.

In general, and especially in the case where several classes are set to run parallel code, it is important to allow for nested parallelism:

```
omp_set_nested(1);
```

This should typically be done at the start of the `main` function, or at least before running the LinkPred code.

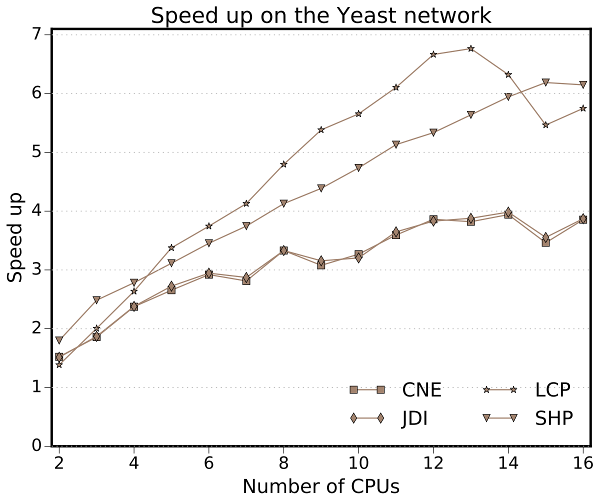

Figure 8.1: Runtime speed up of several link predictors on the Yeast network.

Choosing the level at which parallelism should be activated is important to achieve the best possible performance. In what follows, we present a number of scenarios that LinkPred users may face and the suggested parallelization strategies.

1. Running a single predictor on a single network: in this case, parallelism should activated at the predictor level as it is the only to take advantage of the parallel execution capability.
2. Evaluating a single link predictor: In general, performance evaluation involves running the link predictor multiple times with different training and test sets. Enabling parallelism at the predictor can lead to some improvement (depending on the type of the predictor). A better strategy, however, would be to parallelize the execution at the outer level, that is executing the predictor with different test data in parallel.
3. Evaluating the performance of several link predictors: This is similar to the previous case, except that we can run the predictors in parallel. This can be beneficial if the predictors have comparable runtime, but if there is a large discrepancy between runtimes it is better to prallelize at the predictor level or over test runs.

The following code how to compute the scores for all negative links fo a network using CNE predictor in parallel.

```
#include <linkpred.hpp>
#include <iostream>
#include <algorithm>
using namespace LinkPred;
```

```
int main(int argc, char*argv[]) {
  omp_set_nested(1); // enable nested parallelism
  auto net = UNetwork<>::read("Infectious.edges");
  UCNEPredictor<> predictor(net);
  predictor.setParallel(true);
  predictor.init();
  predictor.learn();
  std::vector<double> scores;
  scores.resize(net->getNbNonEdges());
  auto its = predictor.predictNeg(scores.begin());
  std::cout << "#Start\tEnd\tScore\n";
  std::size_t i = 0;
  for (auto it = its.first; it != its.second; ++it, i++) {
    std::cout << net->getLabel(net->start(*it)) << "\t"<< net->
        getLabel(net->end(*it)) << "\t" << scores[i] << std::endl;
  }
  return 0;
}
```

The following is an extract of this code's output:

```
#Start  End     Score
100     10      0
100     11      0
100     113     7
100     12      0
100     13      0
100     14      0
100     15      0
100     16      0
100     107     10
100     23      0
...
```

8.1.2 Distributed parallelism

Distributed parallelism is implemented in LinkPred using MPI (Message Passing Interface) unless this deactivated during compilation time (the corresponding flag is `LINKPRED_WITH_MPI`). Several (but not all) link predictors offer distributed implementations of the methods `predictNeg` and `top`. Note, however, that the network data structure is not distributed, and consequently, each processor must have access to the whole network data either by reading it from file or otherwise.

The `ROC` performance measure supports distributed processing when using the streaming method, and the same applies to `TPR` (top-precision) when using the `top` method. In both cases, the result of the performance measure is only available at processor 0. The default methods in `ROC` and `TPR` as well as the `PR` class dot not support distributed parallelism.

Similarly to shared memory parallelism, distributed processing is controlled at the object level. To activate/deactivate distributed processing for a given predictor just set the attributed `distributed`. For example:

```
predictor->setDistributed(true);
```

The following code shows how to find the top k edges distributively.

```
#include <linkpred.hpp>
#include <iostream>
using namespace LinkPred;
int main(int argc, char*argv[]) {
  MPI_Init(&argc, &argv);
  std::size_t k = 10;
  auto net = UNetwork<>::read("Infectious.edges");
  URALPredictor<> predictor(net);
  predictor.setComm(MPI_COMM_WORLD); // Optional when using the
     default communicator MPI_COMM_WORLD
  predictor.setDistributed(true);
  predictor.init();
  predictor.learn();
  std::vector<typename UNetwork<>::Edge> edges;
  edges.resize(k);
  std::vector<double> scores;
  scores.resize(k);
  k = predictor.top(k, edges.begin(), scores.begin());

  int procID;
  MPI_Comm_rank(MPI_COMM_WORLD, &procID);
  if (procID == 0) {
    std::cout << "#Start\tEnd\tScore\n";
  }
  for (std::size_t i = 0; i < k; i++) {
    std::cout << net->getLabel(net->start(edges[i])) << "\t"
        << net->getLabel(net->end(edges[i])) << "\t" << scores[i]
        << std::endl;
  }
  MPI_Finalize();
  return 0;
}
```

If you compile this code into the executable `ratop`, for example, the program can be run as follows:

```
mpirun -n 4 ./raltop
```

This is an example output of this code:

```
#Start  End     Score
169     178     0.912642
144     142     0.886008
51      39      0.985052
265     297     0.811915
300     295     0.806431
197     237     0.836456
257     299     0.864928
257     294     0.887479
261     292     0.973033
389     367     0.965622
```

R In this example, you may need to set `OMP_NUM_THREADS` to 1, because we are not using shared memory parallelism. On Linux, this can be achieved by running the command `export OMP_NUM_THREADS=1`.

The following shows how to compute the area under the ROC in a distributed way:

```
#include <linkpred.hpp>
#include <iostream>
using namespace LinkPred;
int main(int argc, char *argv[]) {
  MPI_Init(&argc, &argv);
  int procID = 0;
  MPI_Comm_rank(MPI_COMM_WORLD, &procID);
  std::string netFileName(argv[1]);
  auto refNet = UNetwork<>::read(netFileName);
  auto testData = NetworkManipulator<>::createTestData(refNet,
     0.1, 0, false, true, 0, true, 0, FN, TN, 777, false);
  testData.lock();
  auto predictor = std::make_shared<UADAPredictor<>>(testData.
     getObsNet());
  predictor->init();
  predictor->learn();
  auto predResults = std::make_shared<PredResults<>>(testData,
     predictor);
  auto roc = std::make_shared<ROC<>>("ROC");
  roc->setComm(MPI_COMM_WORLD); // Optional when using the
     default communicator MPI_COMM_WORLD
  roc->setParallel(true);
  roc->setDistributed(true);
  roc->setStrmEnabled(true);
  PerfResults res;
  roc->eval(predResults, res);
  if (procID == 0) {
    std::cout << "#ROCAUC (streaming): " << res.at(roc->getName()
       ) << std::endl;
  }
  MPI_Finalize();
  return 0;
}
```

If you compile this code into the executable `rocstrmdist`, for example, the program can be run as follows:

```
mpirun -n 4 ./rocstrmdist AS_Internet.edges
```

This is an example output of this code:

```
#ROCAUC (streaming): 0.8466
```

8.2 Templates

LinkPred is mainly a pre-instantiated template library, a design choice that offers fast compilation while keeping the library extensible and customizable. The default templates arguments used in the pre-instantiated classes are chosen to give the best performance possible, but it is often the case that classes are instantiated with arguments other then the default ones. For example, the class `UNetwork` is instantiated with `std::string` as the default type for nodes labels, and it is also instantiated with **unsigned int**. The latter is a more restrictive option but may be useful for saving memory, especially that most network datasets have integer node IDs.

R You can find the list of pre-instantiated classes in the file `include/instantiations.hpp`.

The class templates instantiations found in `instantiations.hpp` can be readily used. If you want to instantiate a class with a template argument other than those already available, you have to edit the file `instantiations.hpp`.

■ **Example 8.1** If you want to instantiate the class `UNetwork` with `short int`, locate the following section in `instantiations.hpp`:

```
#ifdef UNETWORK_CPP
template class UNetwork<>;
template class UNetwork<unsigned int>;
#endif
```

and change it to (stay within the `ifdef`):

```
#ifdef UNETWORK_CPP
template class UNetwork<>;
template class UNetwork<unsigned int>;
template class UNetwork<short int>;
#endif
```

You need than to recompile the library to use the new instantiation. ■

R Note that adding a new instantiation may require to add other new instantiations in classes that use it. For example, if you want to use the new instantiation `UNetwork<short int>` with the CNE predictor, you need to add a new instantiation of the class `UCNEPredictor` as follows:

```
#ifdef UCNEPREDICTOR_CPP
template class UCNEPredictor<UNetwork<>, typename
   UNetwork<>::NonEdgeIt>;
template class UCNEPredictor<UNetwork<short int>,
   typename UNetwork<short int>::NonEdgeIt>; // new
   instantiation
#endif
```

8.3 Extending LinkPred

The practice followed in LinkPred is to define clear and easy interfaces for its components and use these interfaces to connect these components. This makes integrating new implementations of these interfaces easy and seamless. In this section, we will demonstrate through an example how to extend LinkPred with new link prediction algorithm. We will use the same example presented at the end of Chapter 6, but this time we will added it to the library. Suppose you want to create a very simple link prediction algorithm that assigns as score to (i, j) the score $\kappa_i + \kappa_j$, the sum of the degrees of the two nodes[1]. To add this predictor to the library proceed as follows:

[1] LinkPred already contains a sum-of-degree predictor named `USUMPredictor`.

1. In the source directory of LinkPred, create the file `include/linkpred/predictors/undirected/usdpredictor.hpp`, and write the following code:

Listing 8.1: code/parallel/usdpredictor.hpp

```
#ifndef USDPREDICTOR_HPP_
#define USDPREDICTOR_HPP_
#include <linkpred/predictors/undirected/ulpredictor.hpp>
namespace LinkPred {
template<typename Network = UNetwork<>, typename EdgeRndIt =
    typename std::vector<typename Network::Edge>::
    const_iterator, typename ScoreRndIt = typename std::vector
    <double>::iterator, typename EdgeRndOutIt = typename std::
    vector<typename Network::Edge>::iterator> class
    USDPredictor: public ULPredictor<Network, EdgeRndIt,
    ScoreRndIt, EdgeRndOutIt> {
  using ULPredictor<Network, EdgeRndIt, ScoreRndIt,
      EdgeRndOutIt>::net; /**< The network. */
  using ULPredictor<Network, EdgeRndIt, ScoreRndIt,
      EdgeRndOutIt>::name; /**< The name of the predictor. */
public:
  USDPredictor(std::shared_ptr<Network const> net) :
      ULPredictor<Network, EdgeRndIt, ScoreRndIt, EdgeRndOutIt
      >(net) {
    name = "SD";
  }
  virtual void init(){}
  virtual void learn(){}
  virtual double score(typename Network::Edge const & e);
  virtual ~USDPredictor() = default;
};
} /* namespace LinkPred */
#endif /* USDPREDICTOR_HPP_ */
```

> **R** This predictor does not require any initialization or learning, hence the empty implementations of the two methods `init` and `learn`.

2. In the file `src/predictors/undirected/usdpredictor.cpp` write the implementation of the abstract method `score`:

Listing 8.2: code/parallel/usdpredictor.cpp

```
#include <linkpred/predictors/undirected/usdpredictor.hpp>
namespace LinkPred {
template<typename Network, typename EdgeRndIt, typename
    ScoreRndIt, typename EdgeRndOutIt> double USDPredictor<
    Network, EdgeRndIt, ScoreRndIt, EdgeRndOutIt>::score(
    typename Network::Edge const & e) {
  auto srcNode = Network::start(e);
  auto endNode = Network::end(e);
  return net->getDeg(srcNode) + net->getDeg(endNode);
}
#define USDPREDICTOR_CPP
#include "linkpred/instantiations.hpp"
```

```
#undef USDPREDICTOR_CPP
} /* namespace LinkPred */
```

3. Add the predictor to the header file: `include/linkpred/predictors/undirected/undirected.hpp`. To do this, locate the line:

```
#define UNDIRECTED_HPP_
```

then add after it the following:

```
#include "linkpred/predictors/undirected/usdpredictor.hpp"
```

4. In the file `include/linkpred/instantiations.hpp` add the following:

```
#ifdef USDPREDICTOR_CPP
template class USDPredictor<>;
template class USDPredictor<UNetwork<>, typename UNetwork<>::
    NonEdgeIt>;
#endif
```

5. In the top `CMakeLists.txt`, locate the line:

```
  src/predictors/undirected/uadapredictor.cpp
```

below it or above it add the following line:

```
  src/predictors/undirected/usdpredictor.cpp
```

6. Finally, recompile LinkPred (see Chapter 1). The new predictor is now part of linkPred and can be used as any other built-in predictor.

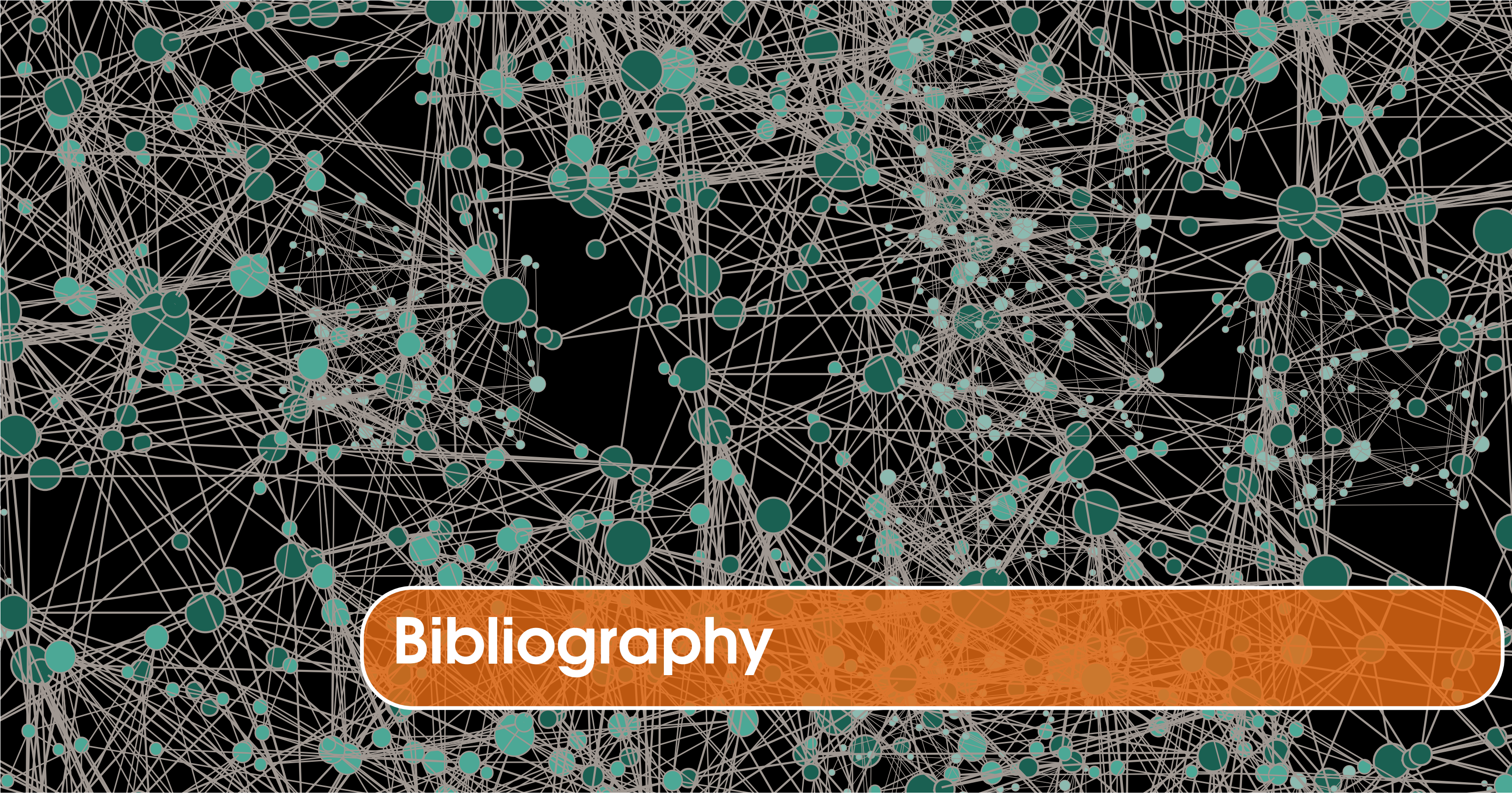

Bibliography

[1] Amr Ahmed et al. “Distributed Large-Scale Natural Graph Factorization”. In: *Proceedings of the 22nd International Conference on World Wide Web*. WWW ’13. Rio de Janeiro, Brazil: Association for Computing Machinery, 2013, pages 37–48. ISBN: 9781450320351 (cited on page 60).

[2] R. Alharbi, H. Benhidour, and S. Kerrache. “Link Prediction in Complex Networks Based on a Hidden Variables Model”. In: *2016 UKSim-AMSS 18th International Conference on Computer Modelling and Simulation (UKSim)*. 2016, pages 119–124 (cited on page 60).

[3] Vladimir Batagelj and Andrej Mrvar. *Pajek Datasets*. `http://vlado.fmf.uni-lj.si/pub/networks/data`. 2006 (cited on page 12).

[4] Mikhail Belkin and Partha Niyogi. “Laplacian Eigenmaps and Spectral Techniques for Embedding and Clustering.” In: *NIPS*. Edited by Thomas G. Dietterich, Suzanna Becker, and Zoubin Ghahramani. MIT Press, 2001, pages 585–591 (cited on page 60).

[5] Smriti Bhagat, Graham Cormode, and S. Muthukrishnan. “Node Classification in Social Networks.” In: *Social Network Data Analytics*. Edited by Charu C. Aggarwal. Springer, 2011, pages 115–148. ISBN: 978-1-4419-8461-6 (cited on page 60).

[6] Shaosheng Cao, Wei Lu, and Qiongkai Xu. “Deep Neural Networks for Learning Graph Representations”. In: *AAAI Conference on Artificial Intelligence*. 2016 (cited on page 60).

[7] Aaron Clauset, Cristopher Moore, and Mark EJ Newman. “Hierarchical structure and the prediction of missing links in networks”. In: *Nature* 453.7191 (2008), pages 98–101 (cited on pages 11, 78).

[8] Aaron Clauset, Cosma Rohilla Shalizi, and Mark EJ Newman. “Power-law distributions in empirical data”. In: *SIAM review* 51.4 (2009), pages 661–703 (cited on pages 12, 80).

[9] Jesse Davis and Mark Goadrich. "The Relationship Between Precision-Recall and ROC Curves". In: *Proceedings of the 23rd International Conference on Machine Learning*. ICML '06. Pittsburgh, Pennsylvania, USA: ACM, 2006, pages 233–240. ISBN: 1-59593-383-2 (cited on page 112).

[10] Palash Goyal and Emilio Ferrara. "Graph embedding techniques, applications, and performance: A survey". In: *Knowledge-Based Systems* 151 (2018), pages 78–94. ISSN: 0950-7051 (cited on pages 60, 81).

[11] Aditya Grover and Jure Leskovec. "Node2vec: Scalable Feature Learning for Networks". In: *Proceedings of the 22nd ACM SIGKDD International Conference on Knowledge Discovery and Data Mining*. KDD '16. San Francisco, California, USA: Association for Computing Machinery, 2016, pages 855–864. ISBN: 9781450342322 (cited on pages 12, 60, 61).

[12] Roger Guimerà and Marta Sales-Pardo. "Missing and spurious interactions and the reconstruction of complex networks". In: *Proceedings of the National Academy of Sciences* 106.52 (2009), pages 22073–22078 (cited on pages 11, 78).

[13] William W. Hager and Hongchao Zhang. "Algorithm 851: CG_DESCENT, a conjugate gradient method with guaranteed descent". In: *ACM Trans. Math. Softw.* 32.1 (Mar. 2006), pages 113–137. ISSN: 0098-3500 (cited on page 12).

[14] Lorenzo Isella et al. "What's in a crowd? Analysis of face-to-face behavioral networks". In: *Journal of Theoretical Biology* 271.1 (2011), pages 166–180. ISSN: 0022-5193 (cited on page 12).

[15] Seyed Mehran Kazemi and David Poole. "SimplE Embedding for Link Prediction in Knowledge Graphs". In: *Advances in Neural Information Processing Systems*. Edited by S. Bengio et al. Volume 31. Curran Associates, Inc., 2018, pages 4284–4295 (cited on page 60).

[16] Said Kerrache, Ruwayda Alharbi, and Hafida Benhidour. "A Scalable Similarity-Popularity Link Prediction Method". In: *Scientific Reports* 10.1 (Apr. 2020), page 6394. ISSN: 2045-2322 (cited on page 77).

[17] Yehuda Koren, Robert Bell, and Chris Volinsky. "Matrix Factorization Techniques for Recommender Systems". In: *Computer* 42.8 (2009), pages 30–37. ISSN: 0018-9162 (cited on pages 60, 61).

[18] Jérôme Kunegis. "KONECT: The Koblenz Network Collection". In: *Proceedings of the 22Nd International Conference on World Wide Web*. WWW '13 Companion. Rio de Janeiro, Brazil: ACM, 2013, pages 1343–1350. ISBN: 978-1-4503-2038-2. URL: `http://konect.uni-koblenz.de/networks` (cited on page 12).

[19] Jure Leskovec and Andrej Krevl. *SNAP Datasets: Stanford Large Network Dataset Collection*. `http://snap.stanford.edu/data`. June 2014 (cited on page 12).

[20] Zhen Liu et al. "Correlations between community structure and link formation in complex networks". In: *PloS one* 8.9 (2013) (cited on pages 11, 79).

[21] Linyuan Lü and Tao Zhou. "Link prediction in complex networks: A survey". In: *Physica A: Statistical Mechanics and its Applications* 390.6 (2011), pages 1150–1170 (cited on page 73).

[22] Laurens van der Maaten and Geoffrey Hinton. "Visualizing Data using t-SNE". In: *Journal of Machine Learning Research* 9 (2008), pages 2579–2605 (cited on page 60).

[23] Fragkiskos Papadopoulos, Constantinos Psomas, and Dmitri Krioukov. "Network mapping by replaying hyperbolic growth". In: *IEEE/ACM Transactions on Networking (TON)* 23.1 (2015), pages 198–211 (cited on pages 12, 79).

[24] Fragkiskos Papadopoulos et al. "Popularity versus similarity in growing networks". In: *Nature* 489.7417 (2012), pages 537–540 (cited on pages 12, 79).

[25] Bryan Perozzi, Rami Al-Rfou, and Steven Skiena. "DeepWalk: Online Learning of Social Representations". In: *Proceedings of the 20th ACM SIGKDD International Conference on Knowledge Discovery and Data Mining*. KDD '14. New York, New York, USA: Association for Computing Machinery, 2014, pages 701–710. ISBN: 9781450329569 (cited on pages 12, 60).

[26] Ryan A. Rossi and Nesreen K. Ahmed. "The Network Data Repository with Interactive Graph Analytics and Visualization". In: *AAAI*. 2015. URL: `http://networkrepository.com` (cited on page 12).

[27] Sam T. Roweis and Lawrence K. Saul. "Nonlinear Dimensionality Reduction by Locally Linear Embedding". In: *Science* 290.5500 (2000), pages 2323–2326. ISSN: 0036-8075 (cited on pages 60, 61).

[28] Damian Szklarczyk et al. "STRING v11: protein–protein association networks with increased coverage, supporting functional discovery in genome-wide experimental datasets". In: *Nucleic Acids Research* 47.D1 (Nov. 2018), pages D607–D613. ISSN: 0305-1048 (cited on page 12).

[29] Jian Tang et al. "LINE: Large-Scale Information Network Embedding". In: *Proceedings of the 24th International Conference on World Wide Web*. Republic and Canton of Geneva, CHE: International World Wide Web Conferences Steering Committee, 2015, pages 1067–1077. ISBN: 9781450334693 (cited on pages 12, 60, 61).

[30] Jian Tang et al. "Visualizing Large-scale and High-dimensional Data." In: *WWW*. Edited by Jacqueline Bourdeau et al. ACM, 2016, pages 287–297. ISBN: 978-1-4503-4143-1 (cited on pages 12, 60).

[31] Jiliang Tang, Charu C. Aggarwal, and Huan Liu. "Node Classification in Signed Social Networks." In: *SDM*. Edited by Sanjay Chawla Venkatasubramanian and Wagner Meira Jr. SIAM, 2016, pages 54–62. ISBN: 978-1-61197-434-8 (cited on page 60).

[32] Daixin Wang, Peng Cui, and Wenwu Zhu. "Structural Deep Network Embedding". In: *Proceedings of the 22nd ACM SIGKDD International Conference on Knowledge Discovery and Data Mining*. KDD '16. San Francisco, California, USA: Association for Computing Machinery, 2016, pages 1225–1234. ISBN: 9781450342322 (cited on page 60).

[33] Peng Wang et al. "Link prediction in social networks: the state-of-the-art". In: *Science China Information Sciences* 58.1 (2015), pages 1–38 (cited on page 73).

[34] Wayne W Zachary. "An information flow model for conflict and fission in small groups". In: *Journal of anthropological research* 33 (1977), pages 452–473 (cited on page 12).

[35] R. Zafarani and H. Liu. *Social Computing Data Repository at ASU*. 2009. URL: http://socialcomputing.asu.edu (cited on page 12).

[36] Marinka Zitnik et al. *BioSNAP Datasets: Stanford Biomedical Network Dataset Collection*. http://snap.stanford.edu/biodata. Aug. 2018 (cited on page 12).
