# Supplementary material for: LinkPred: a high performance library for link prediction in complex networks
Source: PeerJ Comput Sci. 2021 May 21;7:e521. doi: 10.7717/peerj-cs.521 (PMC8157017; doi:10.7717/peerj-cs.521)
Supplement: Supplemental Information 1 [file peerj-cs-07-521-s001.zip › LinkPred/doc/RefMan.pdf]

LinkPred

..

Generated by Doxygen 1.8.17

1 LinkPred: A high performance library for link prediction in complex networks 1

2 Module Index 3

2.1 Modules . . . 3

3 Namespace Index 5

3.1 Namespace List . . . 5

4 Hierarchical Index 7

4.1 Class Hierarchy . . . 7

5 Class Index 11

5.1 Class List . . . 11

6 File Index 17

6.1 File List . . . 17

7 Module Documentation 23

7.1 Core classes . . . 23

7.1.1 Detailed Description . . . 23

7.2 Graph algorithms. . . . 24

7.2.1 Detailed Description . . . 24

7.3 Machine learning classes . . . 25

7.3.1 Detailed Description . . . 25

7.4 Numerical classes and algorithms . . . 26

7.4.1 Detailed Description . . . 26

7.5 Performance evaluation classes . . . 27

7.5.1 Detailed Description . . . 27

7.6 Link prediction algorithms . . . 28

7.6.1 Detailed Description . . . 29

7.7 Simplified interface . . . 30

7.7.1 Detailed Description . . . 30

7.8 Utility functions and classes . . . 31

7.8.1 Detailed Description . . . 31

8 Namespace Documentation 33

8.1 LinkPred Namespace Reference . . . 33

8.1.1 Detailed Description . . . 38

8.1.2 Typedef Documentation . . . 38

8.1.2.1 PerfResults . . . 39

8.1.3 Enumeration Type Documentation . . . 39

8.1.3.1 CacheLevel . . . 39

8.1.3.2 LinkClass . . . 39

8.1.3.3 LogLevel . . . 39

8.1.3.4 MDSAlg . . . 40

8.1.3.5 SortOrder 40
8.1.4 Function Documentation 40
8.1.4.1 operator*() [1/3] 40
8.1.4.2 operator*() [2/3] 42
8.1.4.3 operator*() [3/3] 42
8.1.4.4 operator+() [1/3] 43
8.1.4.5 operator+() [2/3] 43
8.1.4.6 operator+() [3/3] 43
8.1.4.7 operator-() [1/3] 44
8.1.4.8 operator-() [2/3] 44
8.1.4.9 operator-() [3/3] 44
8.1.4.10 operator/() [1/3] 45
8.1.4.11 operator/() [2/3] 45
8.1.4.12 operator/() [3/3] 46
8.1.4.13 operator^() 46
8.1.5 Variable Documentation 46
8.1.5.1 MathPI 46
8.2 LinkPred::Simp Namespace Reference 47
8.2.1 Detailed Description 47
8.3 LinkPred::Utils Namespace Reference 47
8.3.1 Detailed Description 48
8.3.2 Function Documentation 48
8.3.2.1 assertNoNaN() 49
8.3.2.2 clear() 49
8.3.2.3 filter() 49
8.3.2.4 flip() 49
8.3.2.5 flipMap() 50
8.3.2.6 getRandom() 50
8.3.2.7 getRndPerm() [1/2] 50
8.3.2.8 getRndPerm() [2/2] 51
8.3.2.9 int_cast() 51
8.3.2.10 localRange() 51
8.3.2.11 ltrim() 51
8.3.2.12 norm() 52
8.3.2.13 plFit() [1/2] 52
8.3.2.14 plFit() [2/2] 52
8.3.2.15 print() [1/3] 53
8.3.2.16 print() [2/3] 53
8.3.2.17 print() [3/3] 53
8.3.2.18 printEdges() [1/2] 54
8.3.2.19 printEdges() [2/2] 54
8.3.2.20 readEdges() 54

8.3.2.21 readEdgeScores() . . . 54
8.3.2.22 rtrim() . . . 55
8.3.2.23 selectRandom() . . . 55
8.3.2.24 selectRandomInPlace() . . . 56
8.3.2.25 selectTopK() . . . 56
8.3.2.26 shuffle() . . . 56
8.3.2.27 sort() . . . 57
8.3.2.28 trim() . . . 57
8.3.2.29 writeEdgeScores() . . . 57

9 Class Documentation **59**

9.1 LinkPred::ASPDistCalculator< Network, DistType, NbHopsType > Class Template Reference . . . 59
9.1.1 Detailed Description . . . 60
9.1.2 Member Typedef Documentation . . . 61
9.1.2.1 Edge . . . 61
9.1.2.2 EdgeLengthMap . . . 61
9.1.2.3 EdgeLengthMapSP . . . 61
9.1.2.4 Label . . . 61
9.1.2.5 LengthMapIdType . . . 62
9.1.2.6 NetworkSP . . . 62
9.1.2.7 NodeDistMap . . . 62
9.1.2.8 NodeDistMapSP . . . 62
9.1.2.9 NodeID . . . 62
9.1.3 Constructor & Destructor Documentation . . . 62
9.1.3.1 ASPDistCalculator() [1/3] . . . 62
9.1.3.2 ASPDistCalculator() [2/3] . . . 63
9.1.3.3 ASPDistCalculator() [3/3] . . . 63
9.1.3.4 ∼ASPDistCalculator() . . . 63
9.1.4 Member Function Documentation . . . 63
9.1.4.1 getDist() [1/2] . . . 64
9.1.4.2 getDist() [2/2] . . . 64
9.1.4.3 getIndDist() . . . 64
9.1.4.4 operator=() [1/2] . . . 65
9.1.4.5 operator=() [2/2] . . . 65
9.1.4.6 setLandmarks() . . . 65
9.2 LinkPred::ASPDsimCalculator< Network, DsimType, NbHopsType > Class Template Reference . . . 66
9.2.1 Detailed Description . . . 67
9.2.2 Member Typedef Documentation . . . 67
9.2.2.1 Edge . . . 67
9.2.2.2 EdgeLengthMap . . . 68
9.2.2.3 EdgeLengthMapSP . . . 68
9.2.2.4 Label . . . 68

9.2.2.5 LengthMapIdType . . . 68
9.2.2.6 NetworkSP . . . 68
9.2.2.7 NodeDsimMap . . . 69
9.2.2.8 NodeDsimMapSP . . . 69
9.2.2.9 NodeID . . . 69
9.2.3 Constructor & Destructor Documentation . . . 69
9.2.3.1 ASPDsimCalculator() [1/3] . . . 69
9.2.3.2 ASPDsimCalculator() [2/3] . . . 70
9.2.3.3 ASPDsimCalculator() [3/3] . . . 70
9.2.3.4 ∼ASPDsimCalculator() . . . 70
9.2.4 Member Function Documentation . . . 70
9.2.4.1 getDist() [1/2] . . . 70
9.2.4.2 getDist() [2/2] . . . 71
9.2.4.3 getIndDist() . . . 71
9.2.4.4 operator=() [1/2] . . . 72
9.2.4.5 operator=() [2/2] . . . 72
9.2.4.6 setLandmarks() . . . 72
9.3 LinkPred::BFS< Network, NodeProcessor > Class Template Reference . . . 73
9.3.1 Detailed Description . . . 74
9.3.2 Constructor & Destructor Documentation . . . 74
9.3.2.1 BFS() [1/3] . . . 74
9.3.2.2 BFS() [2/3] . . . 74
9.3.2.3 BFS() [3/3] . . . 74
9.3.2.4 ∼BFS() . . . 75
9.3.3 Member Function Documentation . . . 75
9.3.3.1 operator=() [1/2] . . . 75
9.3.3.2 operator=() [2/2] . . . 75
9.3.3.3 traverse() . . . 76
9.4 LinkPred::BHeap< T, P, ComparatorT, ComparatorP > Class Template Reference . . . 76
9.4.1 Detailed Description . . . 77
9.4.2 Constructor & Destructor Documentation . . . 78
9.4.2.1 BHeap() [1/3] . . . 78
9.4.2.2 BHeap() [2/3] . . . 78
9.4.2.3 BHeap() [3/3] . . . 78
9.4.2.4 ∼BHeap() . . . 79
9.4.3 Member Function Documentation . . . 79
9.4.3.1 contains() . . . 79
9.4.3.2 decrease() . . . 79
9.4.3.3 increase() . . . 80
9.4.3.4 operator=() [1/2] . . . 80
9.4.3.5 operator=() [2/2] . . . 80
9.4.3.6 pop() . . . 81

9.4.3.7 print() 81
9.4.3.8 push() 81
9.4.3.9 set() 81
9.4.3.10 size() 82
9.4.3.11 top() 82
9.4.3.12 tryDecrease() 82
9.4.3.13 tryIncrease() 83
9.5 LinkPred::Bhmap< K, P, Comparator > Class Template Reference 83
9.5.1 Detailed Description 84
9.5.2 Member Typedef Documentation 84
9.5.2.1 k_const_iterator 84
9.5.2.2 p_const_iterator 84
9.5.3 Constructor & Destructor Documentation 84
9.5.3.1 Bhmap() [1/3] 84
9.5.3.2 Bhmap() [2/3] 85
9.5.3.3 Bhmap() [3/3] 85
9.5.3.4 ∼Bhmap() 85
9.5.4 Member Function Documentation 85
9.5.4.1 insert() 85
9.5.4.2 kbegin() 86
9.5.4.3 kend() 86
9.5.4.4 key() 86
9.5.4.5 kfind() 86
9.5.4.6 operator=() [1/2] 87
9.5.4.7 operator=() [2/2] 87
9.5.4.8 pbegin() 87
9.5.4.9 pend() 88
9.5.4.10 pfind() 88
9.5.4.11 pos() 88
9.5.4.12 size() 88
9.6 LinkPred::Classifier< InRndIt, OutRndIt, ScoreRndIt > Class Template Reference 89
9.6.1 Detailed Description 89
9.6.2 Constructor & Destructor Documentation 89
9.6.2.1 Classifier() [1/3] 89
9.6.2.2 Classifier() [2/3] 90
9.6.2.3 Classifier() [3/3] 90
9.6.2.4 ∼Classifier() 90
9.6.3 Member Function Documentation 90
9.6.3.1 getName() 91
9.6.3.2 learn() 91
9.6.3.3 operator=() [1/2] 91
9.6.3.4 operator=() [2/2] 92

9.6.3.5 predict() 92
9.6.3.6 setName() 92
9.7 LinkPred::Collector< Network > Class Template Reference 93
9.7.1 Detailed Description 93
9.7.2 Constructor & Destructor Documentation 93
9.7.2.1 Collector() [1/3] 93
9.7.2.2 Collector() [2/3] 93
9.7.2.3 Collector() [3/3] 94
9.7.2.4 ∼Collector() 94
9.7.3 Member Function Documentation 94
9.7.3.1 getVisited() 94
9.7.3.2 operator=() [1/2] 94
9.7.3.3 operator=() [2/2] 95
9.7.3.4 process() 95
9.8 LinkPred::CosineSim Class Reference 95
9.8.1 Detailed Description 96
9.8.2 Constructor & Destructor Documentation 96
9.8.2.1 CosineSim() [1/3] 97
9.8.2.2 CosineSim() [2/3] 97
9.8.2.3 CosineSim() [3/3] 97
9.8.2.4 ∼CosineSim() 97
9.8.3 Member Function Documentation 97
9.8.3.1 operator=() [1/2] 97
9.8.3.2 operator=() [2/2] 98
9.8.3.3 sim() 98
9.9 LinkPred::Counter< Network > Class Template Reference 98
9.9.1 Detailed Description 99
9.9.2 Constructor & Destructor Documentation 99
9.9.2.1 Counter() [1/3] 99
9.9.2.2 Counter() [2/3] 99
9.9.2.3 Counter() [3/3] 100
9.9.2.4 ∼Counter() 100
9.9.3 Member Function Documentation 100
9.9.3.1 getCount() 100
9.9.3.2 operator=() [1/2] 100
9.9.3.3 operator=() [2/2] 101
9.9.3.4 process() 101
9.9.3.5 resetCount() 101
9.10 LinkPred::DADAPredictor< Network, EdgeRndIt, ScoreRndIt, EdgeRndOutIt > Class Template Reference 102
9.10.1 Detailed Description 103
9.10.2 Constructor & Destructor Documentation 103

9.10.2.1 DADAPredictor() [1/3] 103
9.10.2.2 DADAPredictor() [2/3] 103
9.10.2.3 DADAPredictor() [3/3] 104
9.10.2.4 ~DADAPredictor() 104
9.10.3 Member Function Documentation 104
9.10.3.1 init() 104
9.10.3.2 learn() 105
9.10.3.3 operator=() [1/2] 105
9.10.3.4 operator=() [2/2] 105
9.10.3.5 predict() 106
9.10.3.6 score() 106
9.10.3.7 top() 106
9.11 LinkPred::DCNEPredictor< Network, EdgeRndIt, ScoreRndIt, EdgeRndOutIt > Class Template Reference 107
9.11.1 Detailed Description 108
9.11.2 Constructor & Destructor Documentation 109
9.11.2.1 DCNEPredictor() [1/3] 109
9.11.2.2 DCNEPredictor() [2/3] 109
9.11.2.3 DCNEPredictor() [3/3] 109
9.11.2.4 ~DCNEPredictor() 110
9.11.3 Member Function Documentation 110
9.11.3.1 init() 110
9.11.3.2 learn() 110
9.11.3.3 operator=() [1/2] 110
9.11.3.4 operator=() [2/2] 111
9.11.3.5 predict() 111
9.11.3.6 score() 111
9.11.3.7 top() 112
9.12 LinkPred::DeepWalk< Network > Class Template Reference 113
9.12.1 Detailed Description 114
9.12.2 Constructor & Destructor Documentation 114
9.12.2.1 DeepWalk() [1/3] 114
9.12.2.2 DeepWalk() [2/3] 115
9.12.2.3 DeepWalk() [3/3] 115
9.12.2.4 ~DeepWalk() 115
9.12.3 Member Function Documentation 115
9.12.3.1 encode() 116
9.12.3.2 getAlpha() 116
9.12.3.3 getDefaultDim() 116
9.12.3.4 getInitLR() 116
9.12.3.5 getMaxCodeLength() 117
9.12.3.6 getNbNodeWalks() 117

9.12.3.7 getNbWalks() 117
9.12.3.8 getStepInterval() 117
9.12.3.9 getTotalSteps() 118
9.12.3.10 getWalkLength() 118
9.12.3.11 getWindowSize() 118
9.12.3.12 init() 118
9.12.3.13 operator=() [1/2] 118
9.12.3.14 operator=() [2/2] 119
9.12.3.15 setAlpha() 119
9.12.3.16 setInitLR() 119
9.12.3.17 setNbNodeWalks() 120
9.12.3.18 setNbWalks() 120
9.12.3.19 setStepInterval() 120
9.12.3.20 setWalkLength() 120
9.12.3.21 setWeightMap() 121
9.12.3.22 setWindowSize() 121

9.13 LinkPred::DESPLDistCalculator< Network, DistType, NbHopsType > Class Template Reference . . 121

9.13.1 Detailed Description 123
9.13.2 Member Typedef Documentation 123
9.13.2.1 Edge 123
9.13.2.2 EdgeLengthMap 124
9.13.2.3 EdgeLengthMapSP 124
9.13.2.4 Label 124
9.13.2.5 LengthMapIdType 124
9.13.2.6 NetworkSP 124
9.13.2.7 NodeDistMap 124
9.13.2.8 NodeDistMapSP 125
9.13.2.9 NodeID 125
9.13.2.10 NodeSDistMap 125
9.13.2.11 NodeSDistMapSP 125
9.13.3 Constructor & Destructor Documentation 125
9.13.3.1 DESPLDistCalculator() [1/3] 125
9.13.3.2 DESPLDistCalculator() [2/3] 126
9.13.3.3 DESPLDistCalculator() [3/3] 126
9.13.3.4 ∼ DESPLDistCalculator() 126
9.13.4 Member Function Documentation 127
9.13.4.1 getDist() [1/3] 127
9.13.4.2 getDist() [2/3] 127
9.13.4.3 getDist() [3/3] 127
9.13.4.4 getFinDistMapNoNeighb() 128
9.13.4.5 getIndDist() 128
9.13.4.6 getLim() 129

9.13.4.7 getMaxNbNodesInCache() 129
9.13.4.8 getSDistMap() 129
9.13.4.9 operator=() [1/2] 129
9.13.4.10 operator=() [2/2] 130
9.13.4.11 setMaxNbNodesInCache() 130
9.14 LinkPred::DFS< Network, NodeProcessor > Class Template Reference 130
9.14.1 Detailed Description 131
9.14.2 Constructor & Destructor Documentation 132
9.14.2.1 DFS() [1/3] 132
9.14.2.2 DFS() [2/3] 132
9.14.2.3 DFS() [3/3] 132
9.14.2.4 ∼DFS() 133
9.14.3 Member Function Documentation 133
9.14.3.1 operator=() [1/2] 133
9.14.3.2 operator=() [2/2] 133
9.14.3.3 traverse() 134
9.15 LinkPred::DHDIPredictor< Network, EdgeRndIt, ScoreRndIt, EdgeRndOutIt > Class Template Reference 134
9.15.1 Detailed Description 135
9.15.2 Constructor & Destructor Documentation 135
9.15.2.1 DHDIPredictor() [1/3] 135
9.15.2.2 DHDIPredictor() [2/3] 136
9.15.2.3 DHDIPredictor() [3/3] 136
9.15.2.4 ∼DHDIPredictor() 136
9.15.3 Member Function Documentation 136
9.15.3.1 init() 137
9.15.3.2 learn() 137
9.15.3.3 operator=() [1/2] 137
9.15.3.4 operator=() [2/2] 137
9.15.3.5 predict() 138
9.15.3.6 score() 138
9.15.3.7 top() 139
9.16 LinkPred::DHPIPredictor< Network, EdgeRndIt, ScoreRndIt, EdgeRndOutIt > Class Template Reference 139
9.16.1 Detailed Description 140
9.16.2 Constructor & Destructor Documentation 141
9.16.2.1 DHPIPredictor() [1/3] 141
9.16.2.2 DHPIPredictor() [2/3] 141
9.16.2.3 DHPIPredictor() [3/3] 141
9.16.2.4 ∼DHPIPredictor() 142
9.16.3 Member Function Documentation 142
9.16.3.1 init() 142
9.16.3.2 learn() 142

9.16.3.3 operator=() [1/2] 143
9.16.3.4 operator=() [2/2] 143
9.16.3.5 predict() 143
9.16.3.6 score() 144
9.16.3.7 top() 144
9.17 LinkPred::Dijkstra< Network, DistType, NbHopsType > Class Template Reference 145
9.17.1 Detailed Description 146
9.17.2 Member Typedef Documentation 146
9.17.2.1 Edge 146
9.17.2.2 EdgeLengthMap 147
9.17.2.3 EdgeLengthMapSP 147
9.17.2.4 Label 147
9.17.2.5 LengthMapIdType 147
9.17.2.6 NetworkSP 147
9.17.2.7 NodeDistMap 147
9.17.2.8 NodeDistMapSP 148
9.17.2.9 NodeID 148
9.17.2.10 NodeSDistMapSP 148
9.17.2.11 PathType 148
9.17.2.12 PathTypeSP 148
9.17.3 Constructor & Destructor Documentation 148
9.17.3.1 Dijkstra() [1/4] 149
9.17.3.2 Dijkstra() [2/4] 149
9.17.3.3 Dijkstra() [3/4] 149
9.17.3.4 Dijkstra() [4/4] 149
9.17.3.5 ∼Dijkstra() 150
9.17.4 Member Function Documentation 150
9.17.4.1 getDist() [1/2] 150
9.17.4.2 getDist() [2/2] 150
9.17.4.3 getDistL() 151
9.17.4.4 getDsim() [1/2] 151
9.17.4.5 getDsim() [2/2] 153
9.17.4.6 getIndDist() 153
9.17.4.7 getIndDistToAll() 154
9.17.4.8 getIndDsim() 155
9.17.4.9 getNet() 155
9.17.4.10 getSDist() 155
9.17.4.11 getShortestPath() 156
9.17.4.12 operator=() [1/2] 156
9.17.4.13 operator=() [2/2] 157
9.17.4.14 registerLengthMap() 157
9.17.4.15 setNet() 157

9.17.4.16 unregisterLengthMap() 158
9.18 LinkPred::DJIDPredictor< Network, EdgeRndIt, ScoreRndIt, EdgeRndOutIt > Class Template Reference 158
9.18.1 Detailed Description 159
9.18.2 Constructor & Destructor Documentation 160
9.18.2.1 DJIDPredictor() [1/3] 160
9.18.2.2 DJIDPredictor() [2/3] 160
9.18.2.3 DJIDPredictor() [3/3] 160
9.18.2.4 ~DJIDPredictor() 161
9.18.3 Member Function Documentation 161
9.18.3.1 init() 161
9.18.3.2 learn() 161
9.18.3.3 operator=() [1/2] 161
9.18.3.4 operator=() [2/2] 162
9.18.3.5 predict() 162
9.18.3.6 score() 162
9.18.3.7 top() 163
9.19 LinkPred::DLCPPredictor< Network, EdgeRndIt, ScoreRndIt, EdgeRndOutIt > Class Template Reference 164
9.19.1 Detailed Description 165
9.19.2 Constructor & Destructor Documentation 165
9.19.2.1 DLCPPredictor() [1/3] 165
9.19.2.2 DLCPPredictor() [2/3] 165
9.19.2.3 DLCPPredictor() [3/3] 166
9.19.2.4 ~DLCPPredictor() 166
9.19.3 Member Function Documentation 166
9.19.3.1 init() 167
9.19.3.2 learn() 167
9.19.3.3 operator=() [1/2] 167
9.19.3.4 operator=() [2/2] 167
9.19.3.5 predict() 168
9.19.3.6 score() 168
9.20 LinkPred::DLHNPredictor< Network, EdgeRndIt, ScoreRndIt, EdgeRndOutIt > Class Template Reference 169
9.20.1 Detailed Description 170
9.20.2 Constructor & Destructor Documentation 170
9.20.2.1 DLHNPredictor() [1/3] 170
9.20.2.2 DLHNPredictor() [2/3] 171
9.20.2.3 DLHNPredictor() [3/3] 171
9.20.2.4 ~DLHNPredictor() 171
9.20.3 Member Function Documentation 171
9.20.3.1 init() 172
9.20.3.2 learn() 172

9.20.3.3 operator=() [1/2] 172
9.20.3.4 operator=() [2/2] 172
9.20.3.5 predict() 173
9.20.3.6 score() 173
9.20.3.7 top() 174
9.21 LinkPred::DLPredictor< NetworkT, EdgeRndItT, ScoreRndItT, EdgeRndOutItT > Class Template Reference 174
9.21.1 Detailed Description 175
9.21.2 Member Typedef Documentation 175
9.21.2.1 Edge 175
9.21.2.2 EdgeRndIt 176
9.21.2.3 EdgeRndOutIt 176
9.21.2.4 Network 176
9.21.2.5 NodeID 176
9.21.2.6 ScoreRndIt 176
9.21.3 Constructor & Destructor Documentation 177
9.21.3.1 DLPredictor() [1/3] 177
9.21.3.2 DLPredictor() [2/3] 177
9.21.3.3 DLPredictor() [3/3] 177
9.21.3.4 ∼DLPredictor() 178
9.21.4 Member Function Documentation 178
9.21.4.1 getName() 178
9.21.4.2 getNet() 178
9.21.4.3 init() 179
9.21.4.4 learn() 179
9.21.4.5 operator=() [1/2] 179
9.21.4.6 operator=() [2/2] 180
9.21.4.7 predict() 180
9.21.4.8 predictNeg() 180
9.21.4.9 score() 181
9.21.4.10 setName() 181
9.21.4.11 top() 182
9.22 LinkPred::DNetwork< LabelT, NodeIDT, EdgeT > Class Template Reference 182
9.22.1 Detailed Description 185
9.22.2 Member Typedef Documentation 185
9.22.2.1 Edge 185
9.22.2.2 EdgeIt 186
9.22.2.3 EdgeMapSP 186
9.22.2.4 Label 186
9.22.2.5 LabelIt 186
9.22.2.6 NodeID 186
9.22.2.7 NodeIt 186

9.22.2.8 NodeMapSP 187
9.22.2.9 NodeSMapSP 187
9.22.3 Constructor & Destructor Documentation 187
9.22.3.1 DNetwork() [1/4] 187
9.22.3.2 DNetwork() [2/4] 187
9.22.3.3 DNetwork() [3/4] 188
9.22.3.4 DNetwork() [4/4] 188
9.22.3.5 ∼DNetwork() 188
9.22.4 Member Function Documentation 188
9.22.4.1 addEdge() 188
9.22.4.2 addNode() 189
9.22.4.3 assemble() 189
9.22.4.4 compareEdgeEnd() 189
9.22.4.5 coupleAtOrd() 190
9.22.4.6 coupleOrd() 190
9.22.4.7 createEdgeMap() 190
9.22.4.8 createEdgeMapSP() 191
9.22.4.9 createNodeMap() 191
9.22.4.10 createNodeMapSP() 191
9.22.4.11 createNodeSMap() 192
9.22.4.12 createNodeSMapSP() 192
9.22.4.13 edgesBegin() 193
9.22.4.14 edgesEnd() 193
9.22.4.15 end() 193
9.22.4.16 findLabel() 193
9.22.4.17 findNode() 194
9.22.4.18 getAvgDeg() 194
9.22.4.19 getAvgInDeg() 194
9.22.4.20 getAvgOutDeg() 195
9.22.4.21 getDeg() 195
9.22.4.22 getDegStat() 195
9.22.4.23 getID() 196
9.22.4.24 getInDeg() 196
9.22.4.25 getInDegStat() 196
9.22.4.26 getInOutDeg() 197
9.22.4.27 getLabel() 197
9.22.4.28 getMaxDeg() 197
9.22.4.29 getMaxInDeg() 198
9.22.4.30 getMaxOutDeg() 198
9.22.4.31 getMinDeg() 198
9.22.4.32 getMinInDeg() 198
9.22.4.33 getMinOutDeg() 199

9.22.4.34 getNbCouples() 199
9.22.4.35 getNbEdges() 199
9.22.4.36 getNbNodes() 199
9.22.4.37 getNbNonEdges() 200
9.22.4.38 getNbPaths() 200
9.22.4.39 getOutDeg() 200
9.22.4.40 getOutDegStat() 201
9.22.4.41 inEdgesBegin() 201
9.22.4.42 inEdgesEnd() 201
9.22.4.43 inNeighborsBegin() 201
9.22.4.44 inNeighborsEnd() 202
9.22.4.45 isEdge() [1/2] 202
9.22.4.46 isEdge() [2/2] 203
9.22.4.47 labelsBegin() 203
9.22.4.48 labelsEnd() 203
9.22.4.49 makeEdge() 203
9.22.4.50 neighbBegin() 204
9.22.4.51 neighbEnd() 204
9.22.4.52 nodesBegin() 204
9.22.4.53 nodesDegBegin() 205
9.22.4.54 nodesDegEnd() 205
9.22.4.55 nodesEnd() 205
9.22.4.56 nonEdgesBegin() 205
9.22.4.57 nonEdgesEnd() 206
9.22.4.58 operator=() [1/2] 206
9.22.4.59 operator=() [2/2] 206
9.22.4.60 outEdgesBegin() 206
9.22.4.61 outEdgesEnd() 207
9.22.4.62 outNeighborsBegin() 207
9.22.4.63 outNeighborsEnd() 207
9.22.4.64 print() 208
9.22.4.65 printEdges() 208
9.22.4.66 read() 208
9.22.4.67 readEdges() 209
9.22.4.68 reverseEdge() 209
9.22.4.69 rndEdgesBegin() 209
9.22.4.70 rndEdgesEnd() 210
9.22.4.71 rndNodesBegin() 210
9.22.4.72 rndNodesEnd() 210
9.22.4.73 rndNonEdgesBegin() 211
9.22.4.74 rndNonEdgesEnd() 211
9.22.4.75 shuffle() 211

9.22.4.76 start() 212
9.22.4.77 write() 212
9.23 LinkPred::DotProd Class Reference 212
9.23.1 Detailed Description 213
9.23.2 Constructor & Destructor Documentation 213
9.23.2.1 DotProd() [1/3] 214
9.23.2.2 DotProd() [2/3] 214
9.23.2.3 DotProd() [3/3] 214
9.23.2.4 ∼DotProd() 214
9.23.3 Member Function Documentation 214
9.23.3.1 operator=() [1/2] 214
9.23.3.2 operator=() [2/2] 215
9.23.3.3 sim() 215
9.24 LinkPred::DPATPredictor< Network, EdgeRndIt, ScoreRndIt, EdgeRndOutIt > Class Template Reference 215
9.24.1 Detailed Description 216
9.24.2 Constructor & Destructor Documentation 217
9.24.2.1 DPATPredictor() [1/3] 217
9.24.2.2 DPATPredictor() [2/3] 217
9.24.2.3 DPATPredictor() [3/3] 217
9.24.2.4 ∼DPATPredictor() 218
9.24.3 Member Function Documentation 218
9.24.3.1 init() 218
9.24.3.2 learn() 219
9.24.3.3 operator=() [1/2] 219
9.24.3.4 operator=() [2/2] 219
9.24.3.5 predict() 220
9.24.3.6 score() 220
9.25 LinkPred::DPSTPredictor< Network, EdgeRndIt, ScoreRndIt, EdgeRndOutIt > Class Template Reference 220
9.25.1 Detailed Description 222
9.25.2 Constructor & Destructor Documentation 222
9.25.2.1 DPSTPredictor() [1/3] 222
9.25.2.2 DPSTPredictor() [2/3] 222
9.25.2.3 DPSTPredictor() [3/3] 223
9.25.2.4 ∼DPSTPredictor() 223
9.25.3 Member Function Documentation 223
9.25.3.1 init() 223
9.25.3.2 learn() 224
9.25.3.3 loadEdgeScores() 224
9.25.3.4 operator=() [1/2] 224
9.25.3.5 operator=() [2/2] 225
9.25.3.6 score() 225

9.25.3.7 setEdgeScores() 225
9.26 LinkPred::DSAIPredictor< Network, EdgeRndIt, ScoreRndIt, EdgeRndOutIt > Class Template Reference 226
9.26.1 Detailed Description 227
9.26.2 Constructor & Destructor Documentation 227
9.26.2.1 DSAIPredictor() [1/3] 227
9.26.2.2 DSAIPredictor() [2/3] 228
9.26.2.3 DSAIPredictor() [3/3] 228
9.26.2.4 ∼DSAIPredictor() 228
9.26.3 Member Function Documentation 228
9.26.3.1 init() 229
9.26.3.2 learn() 229
9.26.3.3 operator=() [1/2] 229
9.26.3.4 operator=() [2/2] 229
9.26.3.5 predict() 230
9.26.3.6 score() 230
9.26.3.7 top() 231
9.27 LinkPred::DSOIPredictor< Network, EdgeRndIt, ScoreRndIt, EdgeRndOutIt > Class Template Reference 231
9.27.1 Detailed Description 232
9.27.2 Constructor & Destructor Documentation 233
9.27.2.1 DSOIPredictor() [1/3] 233
9.27.2.2 DSOIPredictor() [2/3] 233
9.27.2.3 DSOIPredictor() [3/3] 233
9.27.2.4 ∼DSOIPredictor() 234
9.27.3 Member Function Documentation 234
9.27.3.1 init() 234
9.27.3.2 learn() 234
9.27.3.3 operator=() [1/2] 235
9.27.3.4 operator=() [2/2] 235
9.27.3.5 predict() 235
9.27.3.6 score() 236
9.27.3.7 top() 236
9.28 LinkPred::Simp::Evaluator::Factory::ECLParams Struct Reference 237
9.28.1 Detailed Description 237
9.28.2 Member Data Documentation 237
9.28.2.1 classifierName 237
9.28.2.2 dim 237
9.28.2.3 encoderName 237
9.28.2.4 negRatio 238
9.28.2.5 posRatio 238
9.28.2.6 seed 238
9.29 LinkPred::UNetwork< LabelT, NodeIDT, EdgeT >::EdgeMap< ValueType > Class Template Reference238

9.29.1 Detailed Description 239
9.29.2 Member Typedef Documentation 239
9.29.2.1 EdgeMapConstIt 239
9.29.2.2 EdgeMapIt 239
9.29.3 Constructor & Destructor Documentation 239
9.29.3.1 EdgeMap() [1/2] 240
9.29.3.2 EdgeMap() [2/2] 240
9.29.3.3 ∼EdgeMap() 240
9.29.4 Member Function Documentation 240
9.29.4.1 at() 240
9.29.4.2 begin() 241
9.29.4.3 cbegin() 241
9.29.4.4 cend() 241
9.29.4.5 end() 242
9.29.4.6 find() [1/2] 242
9.29.4.7 find() [2/2] 242
9.29.4.8 operator=() [1/2] 243
9.29.4.9 operator=() [2/2] 243
9.29.4.10 operator[]() [1/2] 243
9.29.4.11 operator[]() [2/2] 244
9.29.4.12 size() 244
9.30 LinkPred::DNetwork< LabelT, NodeIDT, EdgeT >::EdgeMap< ValueType > Class Template Reference 244
9.30.1 Detailed Description 245
9.30.2 Member Typedef Documentation 245
9.30.2.1 EdgeMapConstIt 245
9.30.2.2 EdgeMapIt 246
9.30.3 Constructor & Destructor Documentation 246
9.30.3.1 EdgeMap() [1/2] 246
9.30.3.2 EdgeMap() [2/2] 246
9.30.3.3 ∼EdgeMap() 246
9.30.4 Member Function Documentation 247
9.30.4.1 at() 247
9.30.4.2 begin() 247
9.30.4.3 cbegin() 247
9.30.4.4 cend() 248
9.30.4.5 end() 248
9.30.4.6 find() [1/2] 248
9.30.4.7 find() [2/2] 248
9.30.4.8 operator=() [1/2] 249
9.30.4.9 operator=() [2/2] 249
9.30.4.10 operator[]() [1/2] 249
9.30.4.11 operator[]() [2/2] 250

9.30.4.12 size() 250
9.31 LinkPred::Utils::EdgeScore< Label > Struct Template Reference 251
9.31.1 Detailed Description 251
9.31.2 Member Data Documentation 251
9.31.2.1 i 251
9.31.2.2 j 251
9.31.2.3 score 252
9.32 LinkPred::Simp::EdgeScore Struct Reference 252
9.32.1 Detailed Description 252
9.32.2 Member Data Documentation 252
9.32.2.1 i 252
9.32.2.2 j 252
9.32.2.3 score 253
9.33 LinkPred::Simp::EdgeScoreByID Struct Reference 253
9.33.1 Detailed Description 253
9.33.2 Member Data Documentation 253
9.33.2.1 i 253
9.33.2.2 j 253
9.33.2.3 score 254
9.34 LinkPred::Encoder< Network > Class Template Reference 254
9.34.1 Detailed Description 254
9.34.2 Member Typedef Documentation 255
9.34.2.1 CodeMap 255
9.34.2.2 CodeMapSP 255
9.34.2.3 Edge 255
9.34.2.4 NodeID 255
9.34.2.5 WeightMap 255
9.34.2.6 WeightMapSP 256
9.34.3 Constructor & Destructor Documentation 256
9.34.3.1 Encoder() [1/3] 256
9.34.3.2 Encoder() [2/3] 256
9.34.3.3 Encoder() [3/3] 256
9.34.3.4 ∼Encoder() 257
9.34.4 Member Function Documentation 257
9.34.4.1 encode() 257
9.34.4.2 getDim() 257
9.34.4.3 getEdgeCode() [1/2] 257
9.34.4.4 getEdgeCode() [2/2] 258
9.34.4.5 getEdgeCodeDim() 258
9.34.4.6 getName() 258
9.34.4.7 getNodeCode() 259
9.34.4.8 getNodeCodeMap() 259

9.34.4.9 getWeightMap() 259
9.34.4.10 init() 259
9.34.4.11 operator=() [1/2] 259
9.34.4.12 operator=() [2/2] 260
9.34.4.13 setDim() 260
9.34.4.14 setName() 260
9.34.4.15 setWeightMap() 261
9.35 LinkPred::Simp::Evaluator::Factory::ESMParams Struct Reference 261
9.35.1 Detailed Description 261
9.35.2 Member Data Documentation 261
9.35.2.1 dim 261
9.35.2.2 encoderName 262
9.35.2.3 seed 262
9.35.2.4 simMeasureName 262
9.36 LinkPred::ESPDistCalculator< Network, DistType, NbHopsType > Class Template Reference . . . 262
9.36.1 Detailed Description 263
9.36.2 Member Typedef Documentation 264
9.36.2.1 Edge 264
9.36.2.2 EdgeLengthMap 264
9.36.2.3 EdgeLengthMapSP 264
9.36.2.4 Label 264
9.36.2.5 LengthMapIdType 265
9.36.2.6 NetworkSP 265
9.36.2.7 NodeDistMap 265
9.36.2.8 NodeDistMapSP 265
9.36.2.9 NodeID 265
9.36.3 Constructor & Destructor Documentation 265
9.36.3.1 ESPDistCalculator() [1/3] 266
9.36.3.2 ESPDistCalculator() [2/3] 267
9.36.3.3 ESPDistCalculator() [3/3] 267
9.36.3.4 ∼ESPDistCalculator() 267
9.36.4 Member Function Documentation 268
9.36.4.1 getCacheHits() 268
9.36.4.2 getCacheMiss() 268
9.36.4.3 getDist() [1/2] 268
9.36.4.4 getDist() [2/2] 269
9.36.4.5 getIndDist() 269
9.36.4.6 operator=() [1/2] 269
9.36.4.7 operator=() [2/2] 270
9.37 LinkPred::ESPDsimCalculator< Network, DsimType, NbHopsType > Class Template Reference . . 270
9.37.1 Detailed Description 271
9.37.2 Member Typedef Documentation 272

9.37.2.1 Edge 272
9.37.2.2 EdgeLengthMap 272
9.37.2.3 EdgeLengthMapSP 272
9.37.2.4 Label 272
9.37.2.5 LengthMapIdType 273
9.37.2.6 NetworkSP 273
9.37.2.7 NodeDsimMap 273
9.37.2.8 NodeDsimMapSP 273
9.37.2.9 NodeID 273
9.37.3 Constructor & Destructor Documentation 273
9.37.3.1 ESPDsimCalculator() [1/3] 274
9.37.3.2 ESPDsimCalculator() [2/3] 275
9.37.3.3 ESPDsimCalculator() [3/3] 275
9.37.3.4 ∼ESPDsimCalculator() 275
9.37.4 Member Function Documentation 276
9.37.4.1 getDist() [1/2] 276
9.37.4.2 getDist() [2/2] 276
9.37.4.3 getIndDist() 276
9.37.4.4 operator=() [1/2] 277
9.37.4.5 operator=() [2/2] 277
9.38 LinkPred::ESPIndSimlCalculator< Network, DistType, NbHopsType > Class Template Reference . 277
9.38.1 Detailed Description 279
9.38.2 Member Typedef Documentation 279
9.38.2.1 Edge 279
9.38.2.2 EdgeLengthMap 279
9.38.2.3 EdgeLengthMapSP 280
9.38.2.4 Label 280
9.38.2.5 LengthMapIdType 280
9.38.2.6 NetworkSP 280
9.38.2.7 NodeDistMap 280
9.38.2.8 NodeDistMapSP 280
9.38.2.9 NodeID 281
9.38.3 Constructor & Destructor Documentation 281
9.38.3.1 ESPIndSimlCalculator() [1/3] 281
9.38.3.2 ESPIndSimlCalculator() [2/3] 281
9.38.3.3 ESPIndSimlCalculator() [3/3] 281
9.38.3.4 ∼ESPIndSimlCalculator() 282
9.38.4 Member Function Documentation 282
9.38.4.1 getDirIndSiml() 282
9.38.4.2 getIndSiml() 282
9.38.4.3 operator=() [1/2] 283
9.38.4.4 operator=() [2/2] 283

9.39 LinkPred::ESPLDistCalculator< Network, DistType, NbHopsType > Class Template Reference . . . 284
9.39.1 Detailed Description . . . 285
9.39.2 Member Typedef Documentation . . . 285
9.39.2.1 Edge . . . 286
9.39.2.2 EdgeLengthMap . . . 286
9.39.2.3 EdgeLengthMapSP . . . 286
9.39.2.4 Label . . . 286
9.39.2.5 LengthMapIdType . . . 286
9.39.2.6 NetworkSP . . . 286
9.39.2.7 NodeDistMap . . . 287
9.39.2.8 NodeDistMapSP . . . 287
9.39.2.9 NodeID . . . 287
9.39.2.10 NodeSDistMap . . . 287
9.39.2.11 NodeSDistMapSP . . . 287
9.39.3 Constructor & Destructor Documentation . . . 287
9.39.3.1 ESPLDistCalculator() [1/3] . . . 288
9.39.3.2 ESPLDistCalculator() [2/3] . . . 288
9.39.3.3 ESPLDistCalculator() [3/3] . . . 288
9.39.3.4 ∼ ESPLDistCalculator() . . . 289
9.39.4 Member Function Documentation . . . 289
9.39.4.1 getDist() [1/3] . . . 289
9.39.4.2 getDist() [2/3] . . . 289
9.39.4.3 getDist() [3/3] . . . 290
9.39.4.4 getFinDistMapNoNeighb() . . . 290
9.39.4.5 getIndDist() . . . 290
9.39.4.6 getLim() . . . 291
9.39.4.7 getMaxNbNodesInCache() . . . 291
9.39.4.8 getSDistMap() . . . 291
9.39.4.9 operator=() [1/2] . . . 292
9.39.4.10 operator=() [2/2] . . . 292
9.39.4.11 setMaxNbNodesInCache() . . . 292
9.40 LinkPred::ESPLSimlCalculator< Network, DistType, NbHopsType > Class Template Reference . . . 293
9.40.1 Detailed Description . . . 294
9.40.2 Member Typedef Documentation . . . 295
9.40.2.1 Edge . . . 295
9.40.2.2 EdgeLengthMap . . . 295
9.40.2.3 EdgeLengthMapSP . . . 295
9.40.2.4 Label . . . 295
9.40.2.5 LengthMapIdType . . . 295
9.40.2.6 NetworkSP . . . 296
9.40.2.7 NodeID . . . 296
9.40.2.8 NodeSDistMap . . . 296

9.40.2.9 NodeSDistMapSP 296
9.40.3 Constructor & Destructor Documentation 296
9.40.3.1 ESPLSimlCalculator() [1/3] 296
9.40.3.2 ESPLSimlCalculator() [2/3] 297
9.40.3.3 ESPLSimlCalculator() [3/3] 297
9.40.3.4 ∼ ESPLSimlCalculator() 297
9.40.4 Member Function Documentation 298
9.40.4.1 getDirSiml() [1/2] 298
9.40.4.2 getDirSiml() [2/2] 298
9.40.4.3 getLim() 298
9.40.4.4 getMaxNbNodesInCache() 299
9.40.4.5 getNnzSimlMap() 299
9.40.4.6 getNnzSimlMapNoNeighb() 299
9.40.4.7 getSiml() [1/2] 300
9.40.4.8 getSiml() [2/2] 300
9.40.4.9 isUseHops() 300
9.40.4.10 operator=() [1/2] 301
9.40.4.11 operator=() [2/2] 301
9.40.4.12 setMaxNbNodesInCache() 301
9.40.4.13 setUseHops() 302
9.41 LinkPred::ESPSimlCalculator< Network, DistType, NbHopsType > Class Template Reference . . . 302
9.41.1 Detailed Description 304
9.41.2 Member Typedef Documentation 304
9.41.2.1 Edge 304
9.41.2.2 EdgeLengthMap 304
9.41.2.3 EdgeLengthMapSP 304
9.41.2.4 Label 305
9.41.2.5 LengthMapIdType 305
9.41.2.6 NetworkSP 305
9.41.2.7 NodeDistMap 305
9.41.2.8 NodeDistMapSP 305
9.41.2.9 NodeID 305
9.41.3 Constructor & Destructor Documentation 306
9.41.3.1 ESPSimlCalculator() [1/3] 306
9.41.3.2 ESPSimlCalculator() [2/3] 306
9.41.3.3 ESPSimlCalculator() [3/3] 306
9.41.3.4 ∼ESPSimlCalculator() 307
9.41.4 Member Function Documentation 307
9.41.4.1 getDirSiml() [1/2] 307
9.41.4.2 getDirSiml() [2/2] 307
9.41.4.3 getMaxNbNodesInCache() 308
9.41.4.4 getSiml() [1/2] 308

9.41.4.5 getSiml() [2/2] 308
9.41.4.6 operator=() [1/2] 309
9.41.4.7 operator=() [2/2] 309
9.41.4.8 setMaxNbNodesInCache() 309
9.42 LinkPred::Simp::Evaluator Class Reference 310
9.42.1 Detailed Description 311
9.42.2 Constructor & Destructor Documentation 311
9.42.2.1 Evaluator() [1/2] 311
9.42.2.2 Evaluator() [2/2] 311
9.42.2.3 ∼Evaluator() 311
9.42.3 Member Function Documentation 311
9.42.3.1 addADA() 311
9.42.3.2 addCNE() 312
9.42.3.3 addCRA() 312
9.42.3.4 addECL() 312
9.42.3.5 addESM() 313
9.42.3.6 addFBM() 313
9.42.3.7 addHDI() 314
9.42.3.8 addHPI() 314
9.42.3.9 addHRG() 314
9.42.3.10 addHYP() 315
9.42.3.11 addJID() 315
9.42.3.12 addKAB() 315
9.42.3.13 addLCP() 316
9.42.3.14 addLHN() 316
9.42.3.15 addPAT() 316
9.42.3.16 addPR() 317
9.42.3.17 addPST() 317
9.42.3.18 addRAL() 317
9.42.3.19 addRND() 317
9.42.3.20 addROC() 318
9.42.3.21 addSAI() 318
9.42.3.22 addSBM() 318
9.42.3.23 addSHP() 318
9.42.3.24 addSOI() 319
9.42.3.25 addTPR() 319
9.42.3.26 genTestData() 319
9.42.3.27 getPerfRes() 320
9.42.3.28 run() [1/2] 320
9.42.3.29 run() [2/2] 320
9.43 LinkPred::FastSig< T > Class Template Reference 321
9.43.1 Detailed Description 321

9.43.2 Constructor & Destructor Documentation 321
9.43.2.1 FastSig() [1/4] 321
9.43.2.2 FastSig() [2/4] 322
9.43.2.3 FastSig() [3/4] 322
9.43.2.4 FastSig() [4/4] 322
9.43.2.5 ∼FastSig() 323
9.43.3 Member Function Documentation 323
9.43.3.1 operator()() 323
9.43.3.2 operator=() [1/2] 323
9.43.3.3 operator=() [2/2] 323
9.44 LinkPred::Simp::Evaluator::Factory::FBMParams Struct Reference 324
9.44.1 Detailed Description 324
9.44.2 Member Data Documentation 324
9.44.2.1 maxIter 324
9.44.2.2 seed 324
9.45 LinkPred::GCurve< PredResultsT > Class Template Reference 325
9.45.1 Detailed Description 326
9.45.2 Member Typedef Documentation 326
9.45.2.1 ScoresItT 326
9.45.3 Constructor & Destructor Documentation 327
9.45.3.1 GCurve() [1/4] 327
9.45.3.2 GCurve() [2/4] 327
9.45.3.3 GCurve() [3/4] 327
9.45.3.4 GCurve() [4/4] 328
9.45.3.5 ∼GCurve() 328
9.45.4 Member Function Documentation 328
9.45.4.1 eval() [1/2] 328
9.45.4.2 eval() [2/2] 329
9.45.4.3 getCurve() [1/2] 329
9.45.4.4 getCurve() [2/2] 330
9.45.4.5 getGCurve() 330
9.45.4.6 getGCurveAuc() 330
9.45.4.7 getThresholds() 331
9.45.4.8 operator=() [1/2] 331
9.45.4.9 operator=() [2/2] 332
9.46 LinkPred::GFMatrix Class Reference 332
9.46.1 Detailed Description 333
9.46.2 Constructor & Destructor Documentation 333
9.46.2.1 GFMatrix() [1/4] 333
9.46.2.2 GFMatrix() [2/4] 333
9.46.2.3 GFMatrix() [3/4] 334
9.46.2.4 GFMatrix() [4/4] 334

9.46.2.5 ∼GFMatrix() . . . 334
9.46.3 Member Function Documentation . . . 334
9.46.3.1 diag() . . . 335
9.46.3.2 elemMult() . . . 335
9.46.3.3 get() . . . 335
9.46.3.4 getCol() . . . 335
9.46.3.5 getCols() . . . 336
9.46.3.6 getM() . . . 336
9.46.3.7 getN() . . . 336
9.46.3.8 getName() . . . 337
9.46.3.9 getRow() . . . 337
9.46.3.10 getRows() . . . 337
9.46.3.11 mult() [1/2] . . . 337
9.46.3.12 mult() [2/2] . . . 338
9.46.3.13 operator"!=() . . . 338
9.46.3.14 operator=() [1/2] . . . 339
9.46.3.15 operator=() [2/2] . . . 339
9.46.3.16 operator==() . . . 339
9.46.3.17 print() [1/2] . . . 339
9.46.3.18 print() [2/2] . . . 340
9.46.3.19 removeNaN() . . . 340
9.46.3.20 set() . . . 340
9.46.3.21 setCol() . . . 340
9.46.3.22 setDiag() . . . 341
9.46.3.23 setName() . . . 341
9.46.3.24 setRow() . . . 341
9.46.3.25 sum() . . . 341
9.46.3.26 sumCols() . . . 342
9.46.3.27 sumRows() . . . 342
9.46.4 Friends And Related Function Documentation . . . 342
9.46.4.1 operator∗ [1/4] . . . 342
9.46.4.2 operator∗ [2/4] . . . 343
9.46.4.3 operator∗ [3/4] . . . 343
9.46.4.4 operator∗ [4/4] . . . 343
9.46.4.5 operator+ [1/5] . . . 344
9.46.4.6 operator+ [2/5] . . . 344
9.46.4.7 operator+ [3/5] . . . 344
9.46.4.8 operator+ [4/5] . . . 345
9.46.4.9 operator+ [5/5] . . . 345
9.46.4.10 operator- [1/5] . . . 346
9.46.4.11 operator- [2/5] . . . 346
9.46.4.12 operator- [3/5] . . . 346

9.46.4.13 operator- [4/5] 347
9.46.4.14 operator- [5/5] 347
9.46.4.15 operator/ 347
9.47 LinkPred::GraphTraversal< Network, NodeProcessor > Class Template Reference 348
9.47.1 Detailed Description 348
9.47.2 Constructor & Destructor Documentation 348
9.47.2.1 GraphTraversal() [1/3] 349
9.47.2.2 GraphTraversal() [2/3] 349
9.47.2.3 GraphTraversal() [3/3] 349
9.47.2.4 ∼GraphTraversal() 349
9.47.3 Member Function Documentation 350
9.47.3.1 operator=() [1/2] 350
9.47.3.2 operator=() [2/2] 350
9.47.3.3 traverse() 350
9.48 LinkPred::HMSM< Network > Class Template Reference 351
9.48.1 Detailed Description 352
9.48.2 Constructor & Destructor Documentation 352
9.48.2.1 HMSM() [1/3] 352
9.48.2.2 HMSM() [2/3] 353
9.48.2.3 HMSM() [3/3] 353
9.48.2.4 ∼HMSM() 353
9.48.3 Member Function Documentation 353
9.48.3.1 encode() 354
9.48.3.2 getAlpha() 354
9.48.3.3 getDefaultDim() 354
9.48.3.4 getEps() 354
9.48.3.5 getHProb() 354
9.48.3.6 getLProb() 355
9.48.3.7 getNbMdsRuns() 355
9.48.3.8 getTol() 355
9.48.3.9 init() 355
9.48.3.10 operator=() [1/2] 355
9.48.3.11 operator=() [2/2] 356
9.48.3.12 setAlpha() 356
9.48.3.13 setEps() 356
9.48.3.14 setHProb() 357
9.48.3.15 setNbMdsRuns() 357
9.48.3.16 setProb() 357
9.48.3.17 setTol() 357
9.48.3.18 setWeightMap() 358
9.49 LinkPred::Simp::Evaluator::Factory::HRGParams Struct Reference 358
9.49.1 Detailed Description 358

9.49.2 Member Data Documentation 358
9.49.2.1 nbBeans 359
9.49.2.2 nbSamples 359
9.49.2.3 seed 359
9.50 LinkPred::Simp::Evaluator::Factory::HYPParams Struct Reference 359
9.50.1 Detailed Description 359
9.50.2 Member Data Documentation 359
9.50.2.1 gamma 360
9.50.2.2 L 360
9.50.2.3 m 360
9.50.2.4 seed 360
9.50.2.5 T 360
9.50.2.6 zeta 360
9.51 LinkPred::Simp::Evaluator::Factory::KABParams Struct Reference 360
9.51.1 Detailed Description 361
9.51.2 Member Data Documentation 361
9.51.2.1 horizLim 361
9.52 LinkPred::L1Sim Class Reference 361
9.52.1 Detailed Description 362
9.52.2 Constructor & Destructor Documentation 362
9.52.2.1 L1Sim() [1/3] 362
9.52.2.2 L1Sim() [2/3] 362
9.52.2.3 L1Sim() [3/3] 363
9.52.2.4 ∼L1Sim() 363
9.52.3 Member Function Documentation 363
9.52.3.1 operator=() [1/2] 363
9.52.3.2 operator=() [2/2] 363
9.52.3.3 sim() 364
9.53 LinkPred::L2Sim Class Reference 364
9.53.1 Detailed Description 365
9.53.2 Constructor & Destructor Documentation 365
9.53.2.1 L2Sim() [1/3] 365
9.53.2.2 L2Sim() [2/3] 365
9.53.2.3 L2Sim() [3/3] 366
9.53.2.4 ∼L2Sim() 366
9.53.3 Member Function Documentation 366
9.53.3.1 operator=() [1/2] 366
9.53.3.2 operator=() [2/2] 366
9.53.3.3 sim() 367
9.54 LinkPred::LargeVis< Network > Class Template Reference 367
9.54.1 Detailed Description 369
9.54.2 Constructor & Destructor Documentation 369

9.54.2.1 LargeVis() [1/3] 369
9.54.2.2 LargeVis() [2/3] 369
9.54.2.3 LargeVis() [3/3] 370
9.54.2.4 ∼LargeVis() 370
9.54.3 Member Function Documentation 370
9.54.3.1 encode() 370
9.54.3.2 getDefaultDim() 371
9.54.3.3 getGamma() 371
9.54.3.4 getInitLR() 371
9.54.3.5 getNbNegSamples() 371
9.54.3.6 getNbSamples() 371
9.54.3.7 getNegSamplingPower() 371
9.54.3.8 getNegSize() 372
9.54.3.9 init() 372
9.54.3.10 operator=() [1/2] 372
9.54.3.11 operator=() [2/2] 372
9.54.3.12 setGamma() 374
9.54.3.13 setInitLR() 374
9.54.3.14 setNbNegSamples() 374
9.54.3.15 setNbSamples() [1/2] 375
9.54.3.16 setNbSamples() [2/2] 375
9.54.3.17 setNegSamplingPower() 375
9.54.3.18 setNegSize() 375
9.55 LinkPred::Simp::Evaluator::Factory::LCPParams Struct Reference 376
9.55.1 Detailed Description 376
9.55.2 Member Data Documentation 376
9.55.2.1 epsilon 376
9.56 LinkPred::LINE< Network > Class Template Reference 377
9.56.1 Detailed Description 378
9.56.2 Constructor & Destructor Documentation 378
9.56.2.1 LINE() [1/3] 378
9.56.2.2 LINE() [2/3] 379
9.56.2.3 LINE() [3/3] 379
9.56.2.4 ∼LINE() 379
9.56.3 Member Function Documentation 379
9.56.3.1 encode() 379
9.56.3.2 getDefaultDim() 380
9.56.3.3 getInitLr() 380
9.56.3.4 getNbNegSamples() 380
9.56.3.5 getNegSamplingPower() 380
9.56.3.6 getNegSize() 380
9.56.3.7 getOrder() 381

9.56.3.8 getRecNet() 381
9.56.3.9 getRecWeightMap() 381
9.56.3.10 init() 381
9.56.3.11 isEnableReconstruct() 382
9.56.3.12 operator=() [1/2] 382
9.56.3.13 operator=() [2/2] 382
9.56.3.14 setEnableReconstruct() 382
9.56.3.15 setInitLr() 383
9.56.3.16 setNbNegSamples() 383
9.56.3.17 setNegSamplingPower() 383
9.56.3.18 setNegSize() 384
9.56.3.19 setOrder() 384
9.57 LinkPred::LMapQueue< K, P, KComparator, PComparator > Class Template Reference 384
9.57.1 Detailed Description 385
9.57.2 Constructor & Destructor Documentation 385
9.57.2.1 LMapQueue() [1/3] 385
9.57.2.2 LMapQueue() [2/3] 386
9.57.2.3 LMapQueue() [3/3] 386
9.57.2.4 ∼LMapQueue() 386
9.57.3 Member Function Documentation 387
9.57.3.1 at() [1/2] 387
9.57.3.2 at() [2/2] 387
9.57.3.3 begin() [1/2] 387
9.57.3.4 begin() [2/2] 388
9.57.3.5 cbegin() 388
9.57.3.6 cend() 388
9.57.3.7 clear() 388
9.57.3.8 compareTop() 388
9.57.3.9 count() 389
9.57.3.10 empty() 389
9.57.3.11 end() [1/2] 389
9.57.3.12 end() [2/2] 390
9.57.3.13 find() 390
9.57.3.14 getL() 390
9.57.3.15 merge() 390
9.57.3.16 operator=() [1/2] 391
9.57.3.17 operator=() [2/2] 391
9.57.3.18 pop() [1/2] 391
9.57.3.19 pop() [2/2] 391
9.57.3.20 printPQ() 392
9.57.3.21 push() [1/2] 392
9.57.3.22 push() [2/2] 392

9.57.3.23 size() 393
9.57.3.24 top() 393
9.58 LinkPred::Log Class Reference 393
9.58.1 Detailed Description 394
9.58.2 Constructor & Destructor Documentation 394
9.58.2.1 Log() 394
9.58.2.2 ∼Log() 394
9.58.3 Member Function Documentation 394
9.58.3.1 operator<<() 394
9.58.4 Member Data Documentation 395
9.58.4.1 DefaultLogLevel 395
9.58.4.2 DefaultOutputStream 395
9.58.4.3 logLevel 395
9.58.4.4 out 395
9.59 LinkPred::LogisticRegresser< InRndIt, OutRndIt, ScoreRndIt > Class Template Reference 396
9.59.1 Detailed Description 397
9.59.2 Constructor & Destructor Documentation 397
9.59.2.1 LogisticRegresser() [1/3] 397
9.59.2.2 LogisticRegresser() [2/3] 397
9.59.2.3 LogisticRegresser() [3/3] 398
9.59.2.4 ∼LogisticRegresser() 398
9.59.3 Member Function Documentation 398
9.59.3.1 getLambda() 398
9.59.3.2 getTheta() 399
9.59.3.3 getTol() 399
9.59.3.4 learn() 399
9.59.3.5 operator=() [1/2] 399
9.59.3.6 operator=() [2/2] 400
9.59.3.7 predict() 400
9.59.3.8 setLambda() 400
9.59.3.9 setTol() 401
9.60 LinkPred::LogMDSCG Class Reference 401
9.60.1 Detailed Description 402
9.60.2 Constructor & Destructor Documentation 402
9.60.2.1 LogMDSCG() [1/3] 402
9.60.2.2 LogMDSCG() [2/3] 403
9.60.2.3 LogMDSCG() [3/3] 403
9.60.2.4 ∼LogMDSCG() 403
9.60.3 Member Function Documentation 404
9.60.3.1 f() 404
9.60.3.2 fgrad() 404
9.60.3.3 finalize() 404

9.60.3.4 grad() 405
9.60.3.5 init() 405
9.60.3.6 operator=() [1/2] 405
9.60.3.7 operator=() [2/2] 406
9.61 LinkPred::LogRegCG< InRndIt, OutRndIt > Class Template Reference 406
9.61.1 Detailed Description 407
9.61.2 Constructor & Destructor Documentation 408
9.61.2.1 LogRegCG() [1/3] 408
9.61.2.2 LogRegCG() [2/3] 408
9.61.2.3 LogRegCG() [3/3] 408
9.61.2.4 ∼LogRegCG() 409
9.61.3 Member Function Documentation 409
9.61.3.1 f() 409
9.61.3.2 fgrad() 409
9.61.3.3 finalize() 410
9.61.3.4 getLambda() 410
9.61.3.5 grad() 411
9.61.3.6 init() 411
9.61.3.7 operator=() [1/2] 411
9.61.3.8 operator=() [2/2] 412
9.61.3.9 setLambda() 412
9.62 LinkPred::LPSim Class Reference 412
9.62.1 Detailed Description 413
9.62.2 Constructor & Destructor Documentation 413
9.62.2.1 LPSim() [1/3] 414
9.62.2.2 LPSim() [2/3] 414
9.62.2.3 LPSim() [3/3] 414
9.62.2.4 ∼LPSim() 414
9.62.3 Member Function Documentation 414
9.62.3.1 getP() 415
9.62.3.2 operator=() [1/2] 415
9.62.3.3 operator=() [2/2] 415
9.62.3.4 setP() 415
9.62.3.5 sim() 416
9.63 LinkPred::MatFact< Network > Class Template Reference 416
9.63.1 Detailed Description 417
9.63.2 Constructor & Destructor Documentation 418
9.63.2.1 MatFact() [1/3] 418
9.63.2.2 MatFact() [2/3] 418
9.63.2.3 MatFact() [3/3] 418
9.63.2.4 ∼MatFact() 419
9.63.3 Member Function Documentation 419

9.63.3.1 encode() 419
9.63.3.2 getDefaultDim() 419
9.63.3.3 getLambda() 419
9.63.3.4 getNegRatio() 420
9.63.3.5 getPosRatio() 420
9.63.3.6 getTol() 420
9.63.3.7 init() 420
9.63.3.8 operator=() [1/2] 420
9.63.3.9 operator=() [2/2] 421
9.63.3.10 setLambda() 421
9.63.3.11 setNegRatio() 421
9.63.3.12 setPosRatio() 422
9.63.3.13 setTol() 422
9.63.3.14 setWeightMap() 422
9.64 LinkPred::MatFactCG Class Reference 423
9.64.1 Detailed Description 424
9.64.2 Constructor & Destructor Documentation 424
9.64.2.1 MatFactCG() [1/3] 424
9.64.2.2 MatFactCG() [2/3] 424
9.64.2.3 MatFactCG() [3/3] 424
9.64.2.4 ∼MatFactCG() 425
9.64.3 Member Function Documentation 425
9.64.3.1 f() 425
9.64.3.2 fgrad() 425
9.64.3.3 finalize() 426
9.64.3.4 grad() 426
9.64.3.5 init() 426
9.64.3.6 operator=() [1/2] 427
9.64.3.7 operator=() [2/2] 427
9.65 LinkPred::MatFactPbData Struct Reference 427
9.65.1 Detailed Description 428
9.65.2 Member Data Documentation 428
9.65.2.1 i 428
9.65.2.2 j 428
9.65.2.3 target 428
9.66 LinkPred::MDS Class Reference 428
9.66.1 Detailed Description 429
9.66.2 Constructor & Destructor Documentation 429
9.66.2.1 MDS() [1/2] 429
9.66.2.2 MDS() [2/2] 429
9.66.2.3 ∼MDS() 430
9.66.3 Member Function Documentation 430

9.66.3.1 cgMDS() 430
9.66.3.2 getAlg() 430
9.66.3.3 getNbRuns() 431
9.66.3.4 getTol() 431
9.66.3.5 setAlg() 431
9.66.3.6 setNbRuns() 431
9.66.3.7 setTol() 432
9.66.3.8 solve() 432
9.67 LinkPred::NetDistCalculator< Network, DistType, NbHopsType > Class Template Reference 433
9.67.1 Detailed Description 433
9.67.2 Member Typedef Documentation 433
9.67.2.1 Edge 434
9.67.2.2 EdgeLengthMap 434
9.67.2.3 EdgeLengthMapSP 434
9.67.2.4 Label 434
9.67.2.5 LengthMapIdType 434
9.67.2.6 NetworkSP 434
9.67.2.7 NodeDistMap 435
9.67.2.8 NodeDistMapSP 435
9.67.2.9 NodeID 435
9.67.3 Constructor & Destructor Documentation 435
9.67.3.1 ∼NetDistCalculator() 435
9.67.4 Member Function Documentation 435
9.67.4.1 getDiscDist() 435
9.67.4.2 getDiscNbHops() 436
9.67.4.3 getDist() [1/2] 436
9.67.4.4 getDist() [2/2] 436
9.67.4.5 getIndDist() 437
9.67.4.6 getMaxDist() 437
9.67.4.7 setDiscDist() 437
9.67.4.8 setDiscNbHops() 438
9.68 LinkPred::NetIndSimlCalculator< Network, DistType, NbHopsType > Class Template Reference . . 438
9.68.1 Detailed Description 439
9.68.2 Member Typedef Documentation 439
9.68.2.1 Edge 439
9.68.2.2 EdgeLengthMap 439
9.68.2.3 EdgeLengthMapSP 439
9.68.2.4 Label 439
9.68.2.5 LengthMapIdType 440
9.68.2.6 NetworkSP 440
9.68.2.7 NodeID 440
9.68.3 Constructor & Destructor Documentation 440

9.68.3.1 ∼NetIndSimlCalculator() 440
9.68.4 Member Function Documentation 440
9.68.4.1 getDirIndSiml() 440
9.68.4.2 getDiscDist() 441
9.68.4.3 getDiscNbHops() 441
9.68.4.4 getIndSiml() 441
9.68.4.5 getLambda() 442
9.68.4.6 getSelfSiml() 442
9.68.4.7 setDiscDist() 442
9.68.4.8 setDiscNbHops() 442
9.68.4.9 setLambda() 443
9.68.4.10 setSelfSiml() 443
9.69 LinkPred::NetSimlCalculator< Network, DistType, NbHopsType > Class Template Reference 443
9.69.1 Detailed Description 444
9.69.2 Member Typedef Documentation 444
9.69.2.1 Edge 445
9.69.2.2 EdgeLengthMap 445
9.69.2.3 EdgeLengthMapSP 445
9.69.2.4 Label 445
9.69.2.5 LengthMapIdType 445
9.69.2.6 NetworkSP 445
9.69.2.7 NodeID 446
9.69.2.8 NodeSDistMap 446
9.69.2.9 NodeSDistMapSP 446
9.69.3 Constructor & Destructor Documentation 446
9.69.3.1 ∼NetSimlCalculator() 446
9.69.4 Member Function Documentation 446
9.69.4.1 getDirSiml() 446
9.69.4.2 getDiscDist() 447
9.69.4.3 getDiscNbHops() 447
9.69.4.4 getLambda() 447
9.69.4.5 getNnzSimlMap() 447
9.69.4.6 getNnzSimlMapNoNeighb() 448
9.69.4.7 getSelfSiml() 448
9.69.4.8 getSiml() 448
9.69.4.9 setDiscDist() 449
9.69.4.10 setDiscNbHops() 449
9.69.4.11 setLambda() 449
9.69.4.12 setSelfSiml() 450
9.70 LinkPred::NetworkManipulator< Network > Class Template Reference 450
9.70.1 Detailed Description 451
9.70.2 Member Typedef Documentation 452

9.70.2.1 Edge 452
9.70.2.2 EdgeMap 452
9.70.2.3 Label 452
9.70.2.4 NetworkCSP 452
9.70.2.5 NetworkSP 452
9.70.2.6 NodeID 453
9.70.2.7 NodeMap 453
9.70.3 Constructor & Destructor Documentation 453
9.70.3.1 NetworkManipulator() 453
9.70.3.2 ∼NetworkManipulator() 453
9.70.4 Member Function Documentation 453
9.70.4.1 createTestData() [1/2] 453
9.70.4.2 createTestData() [2/2] 454
9.70.4.3 createTestDataAdd() [1/2] 455
9.70.4.4 createTestDataAdd() [2/2] 455
9.70.4.5 createTestDataRem() [1/2] 456
9.70.4.6 createTestDataRem() [2/2] 457
9.70.4.7 createTestDataSeq() 457
9.70.4.8 createTestDataSeqInter() 458
9.70.4.9 getAllNegLinksExcept() 459
9.70.4.10 getRndNegLinksExcept() 459
9.70.4.11 getRndPosLinksExcept() 459
9.70.4.12 isConnected() 460
9.70.4.13 loadTestData() 460
9.70.4.14 loadTestDataAdd() 462
9.70.4.15 loadTestDataRem() 462
9.70.4.16 rndConExtract() 463
9.70.4.17 rndExtract() 463
9.70.4.18 rst() 464
9.71 LinkPred::Node2Vec< Network > Class Template Reference 464
9.71.1 Detailed Description 466
9.71.2 Constructor & Destructor Documentation 466
9.71.2.1 Node2Vec() [1/3] 466
9.71.2.2 Node2Vec() [2/3] 467
9.71.2.3 Node2Vec() [3/3] 467
9.71.2.4 ∼Node2Vec() 467
9.71.3 Member Function Documentation 467
9.71.3.1 encode() 468
9.71.3.2 getDefaultDim() 468
9.71.3.3 getInitLR() 468
9.71.3.4 getNbNegSamples() 468
9.71.3.5 getNbWalks() 469

9.71.3.6 getP() 469
9.71.3.7 getQ() 469
9.71.3.8 getStepInterval() 469
9.71.3.9 getSubSample() 470
9.71.3.10 getTotalSteps() 470
9.71.3.11 getWalkLength() 470
9.71.3.12 getWindowSize() 470
9.71.3.13 init() 470
9.71.3.14 operator=() [1/2] 470
9.71.3.15 operator=() [2/2] 471
9.71.3.16 setInitLR() 471
9.71.3.17 setNbNegSamples() 471
9.71.3.18 setNbWalks() 472
9.71.3.19 setP() 472
9.71.3.20 setQ() 472
9.71.3.21 setStepInterval() 472
9.71.3.22 setSubSample() 473
9.71.3.23 setWalkLength() 473
9.71.3.24 setWeightMap() 473
9.71.3.25 setWindowSize() 474
9.72 LinkPred::DNetwork< LabelT, NodeIDT, EdgeT >::NodeDegIt Class Reference 474
9.72.1 Detailed Description 475
9.72.2 Member Typedef Documentation 475
9.72.2.1 difference_type 476
9.72.2.2 pointer 476
9.72.2.3 reference 476
9.72.3 Constructor & Destructor Documentation 476
9.72.3.1 NodeDegIt() [1/2] 476
9.72.3.2 NodeDegIt() [2/2] 477
9.72.4 Member Function Documentation 477
9.72.4.1 operator"!=() 477
9.72.4.2 operator*() 477
9.72.4.3 operator+() 478
9.72.4.4 operator++() [1/2] 478
9.72.4.5 operator++() [2/2] 478
9.72.4.6 operator+=() 478
9.72.4.7 operator-() [1/2] 479
9.72.4.8 operator-() [2/2] 479
9.72.4.9 operator--() [1/2] 480
9.72.4.10 operator--() [2/2] 480
9.72.4.11 operator-=() 480
9.72.4.12 operator->() 481

9.72.4.13 operator<() 481
9.72.4.14 operator<=() 481
9.72.4.15 operator=() [1/2] 482
9.72.4.16 operator=() [2/2] 482
9.72.4.17 operator==() 482
9.72.4.18 operator>() 483
9.72.4.19 operator>=() 483
9.72.5 Friends And Related Function Documentation 483
9.72.5.1 DNetwork 483
9.73 LinkPred::UNetwork< LabelT, NodeIDT, EdgeT >::NodeDegIt Class Reference 484
9.73.1 Detailed Description 485
9.73.2 Member Typedef Documentation 485
9.73.2.1 difference_type 485
9.73.2.2 pointer 485
9.73.2.3 reference 486
9.73.3 Constructor & Destructor Documentation 486
9.73.3.1 NodeDegIt() [1/2] 486
9.73.3.2 NodeDegIt() [2/2] 486
9.73.4 Member Function Documentation 486
9.73.4.1 operator"!=() 487
9.73.4.2 operator*() 487
9.73.4.3 operator+() 487
9.73.4.4 operator++() [1/2] 488
9.73.4.5 operator++() [2/2] 488
9.73.4.6 operator+=() 488
9.73.4.7 operator-() [1/2] 489
9.73.4.8 operator-() [2/2] 489
9.73.4.9 operator--() [1/2] 489
9.73.4.10 operator--() [2/2] 490
9.73.4.11 operator-=() 490
9.73.4.12 operator->() 490
9.73.4.13 operator<() 490
9.73.4.14 operator<=() 491
9.73.4.15 operator=() [1/2] 491
9.73.4.16 operator=() [2/2] 491
9.73.4.17 operator==() 493
9.73.4.18 operator>() 493
9.73.4.19 operator>=() 493
9.73.5 Friends And Related Function Documentation 494
9.73.5.1 UNetwork 494
9.74 LinkPred::UNetwork< LabelT, NodeIDT, EdgeT >::NodeMap< ValueType > Class Template Reference 494
9.74.1 Detailed Description 495

9.74.2 Member Typedef Documentation 495
9.74.2.1 NodeMapConstIt 495
9.74.2.2 NodeMapIt 495
9.74.3 Constructor & Destructor Documentation 495
9.74.3.1 NodeMap() [1/2] 496
9.74.3.2 NodeMap() [2/2] 496
9.74.3.3 ∼NodeMap() 496
9.74.4 Member Function Documentation 496
9.74.4.1 at() 496
9.74.4.2 begin() 497
9.74.4.3 cbegin() 497
9.74.4.4 cend() 497
9.74.4.5 end() 498
9.74.4.6 operator=() [1/2] 498
9.74.4.7 operator=() [2/2] 498
9.74.4.8 operator[]() [1/2] 498
9.74.4.9 operator[]() [2/2] 499
9.75 LinkPred::DNetwork< LabelT, NodeIDT, EdgeT >::NodeMap< ValueType > Class Template Reference 499
9.75.1 Detailed Description 500
9.75.2 Member Typedef Documentation 500
9.75.2.1 NodeMapConstIt 500
9.75.2.2 NodeMapIt 501
9.75.3 Constructor & Destructor Documentation 501
9.75.3.1 NodeMap() [1/2] 501
9.75.3.2 NodeMap() [2/2] 501
9.75.3.3 ∼NodeMap() 501
9.75.4 Member Function Documentation 502
9.75.4.1 at() 502
9.75.4.2 begin() 502
9.75.4.3 cbegin() 502
9.75.4.4 cend() 503
9.75.4.5 end() 503
9.75.4.6 operator=() [1/2] 503
9.75.4.7 operator=() [2/2] 503
9.75.4.8 operator[]() [1/2] 504
9.75.4.9 operator[]() [2/2] 504
9.76 LinkPred::DNetwork< LabelT, NodeIDT, EdgeT >::NodeSMap< ValueType > Class Template Reference 505
9.76.1 Detailed Description 505
9.76.2 Member Typedef Documentation 506
9.76.2.1 NodeSMapConstIterator 506
9.76.2.2 NodeSMapIterator 506

9.76.3 Constructor & Destructor Documentation 506
9.76.3.1 NodeSMap() [1/2] 506
9.76.3.2 NodeSMap() [2/2] 506
9.76.3.3 ∼NodeSMap() 507
9.76.4 Member Function Documentation 507
9.76.4.1 at() 507
9.76.4.2 begin() 507
9.76.4.3 cbegin() 508
9.76.4.4 cend() 508
9.76.4.5 end() 508
9.76.4.6 find() 508
9.76.4.7 operator=() [1/2] 509
9.76.4.8 operator=() [2/2] 509
9.76.4.9 operator[]() [1/2] 509
9.76.4.10 operator[]() [2/2] 510
9.76.4.11 size() 510
9.77 LinkPred::UNetwork< LabelT, NodeIDT, EdgeT >::NodeSMap< ValueType > Class Template Reference 510
9.77.1 Detailed Description 511
9.77.2 Member Typedef Documentation 511
9.77.2.1 NodeSMapConstIterator 511
9.77.2.2 NodeSMapIterator 512
9.77.3 Constructor & Destructor Documentation 512
9.77.3.1 NodeSMap() [1/2] 512
9.77.3.2 NodeSMap() [2/2] 512
9.77.3.3 ∼NodeSMap() 513
9.77.4 Member Function Documentation 513
9.77.4.1 at() 513
9.77.4.2 begin() 513
9.77.4.3 cbegin() 514
9.77.4.4 cend() 514
9.77.4.5 end() 514
9.77.4.6 find() 514
9.77.4.7 operator=() [1/2] 515
9.77.4.8 operator=() [2/2] 515
9.77.4.9 operator[]() [1/2] 515
9.77.4.10 operator[]() [2/2] 516
9.77.4.11 size() 516
9.78 LinkPred::UNetwork< LabelT, NodeIDT, EdgeT >::NonEdgeIt Class Reference 517
9.78.1 Detailed Description 518
9.78.2 Member Typedef Documentation 518
9.78.2.1 difference_type 518

9.78.2.2 pointer 519
9.78.2.3 reference 519
9.78.3 Constructor & Destructor Documentation 519
9.78.3.1 NonEdgeIt() [1/2] 519
9.78.3.2 NonEdgeIt() [2/2] 519
9.78.4 Member Function Documentation 520
9.78.4.1 operator"!=() 520
9.78.4.2 operator*() 520
9.78.4.3 operator+() 520
9.78.4.4 operator++() [1/2] 521
9.78.4.5 operator++() [2/2] 521
9.78.4.6 operator+=() 521
9.78.4.7 operator-() [1/2] 522
9.78.4.8 operator-() [2/2] 522
9.78.4.9 operator--() [1/2] 522
9.78.4.10 operator--() [2/2] 523
9.78.4.11 operator-=() 523
9.78.4.12 operator->() 523
9.78.4.13 operator<() 524
9.78.4.14 operator<=() 524
9.78.4.15 operator=() [1/2] 524
9.78.4.16 operator=() [2/2] 525
9.78.4.17 operator==() 525
9.78.4.18 operator>() 525
9.78.4.19 operator>=() 526
9.78.5 Friends And Related Function Documentation 526
9.78.5.1 UNetwork 526
9.79 LinkPred::DNetwork< LabelT, NodeIDT, EdgeT >::NonEdgeIt Class Reference 526
9.79.1 Detailed Description 528
9.79.2 Member Typedef Documentation 528
9.79.2.1 difference_type 528
9.79.2.2 pointer 528
9.79.2.3 reference 528
9.79.3 Constructor & Destructor Documentation 528
9.79.3.1 NonEdgeIt() [1/2] 528
9.79.3.2 NonEdgeIt() [2/2] 529
9.79.4 Member Function Documentation 529
9.79.4.1 operator"!=() 529
9.79.4.2 operator*() 529
9.79.4.3 operator+() 530
9.79.4.4 operator++() [1/2] 530
9.79.4.5 operator++() [2/2] 530

9.79.4.6 operator+=() . . . 531
9.79.4.7 operator-() [1/2] . . . 531
9.79.4.8 operator-() [2/2] . . . 531
9.79.4.9 operator--() [1/2] . . . 532
9.79.4.10 operator--() [2/2] . . . 532
9.79.4.11 operator-=() . . . 532
9.79.4.12 operator->() . . . 533
9.79.4.13 operator<() . . . 533
9.79.4.14 operator<=() . . . 533
9.79.4.15 operator=() [1/2] . . . 534
9.79.4.16 operator=() [2/2] . . . 534
9.79.4.17 operator==() . . . 534
9.79.4.18 operator>() . . . 535
9.79.4.19 operator>=() . . . 535
9.79.5 Friends And Related Function Documentation . . . 535
9.79.5.1 DNetwork . . . 535
9.80 LinkPred::Utils::PairCompRight< FirstT, SecondT, CompareT > Struct Template Reference . . . 536
9.80.1 Detailed Description . . . 536
9.80.2 Member Function Documentation . . . 536
9.80.2.1 operator()() . . . 536
9.81 LinkPred::Pearson Class Reference . . . 537
9.81.1 Detailed Description . . . 538
9.81.2 Constructor & Destructor Documentation . . . 538
9.81.2.1 Pearson() [1/3] . . . 538
9.81.2.2 Pearson() [2/3] . . . 538
9.81.2.3 Pearson() [3/3] . . . 538
9.81.2.4 ∼Pearson() . . . 538
9.81.3 Member Function Documentation . . . 539
9.81.3.1 operator=() [1/2] . . . 539
9.81.3.2 operator=() [2/2] . . . 539
9.81.3.3 sim() . . . 539
9.82 LinkPred::PEFactory< Network, LPredictorT, TestDataT, PerfMeasureT > Class Template Reference 540
9.82.1 Detailed Description . . . 540
9.82.2 Constructor & Destructor Documentation . . . 540
9.82.2.1 ∼PEFactory() . . . 540
9.82.3 Member Function Documentation . . . 541
9.82.3.1 getPerfMeasures() . . . 541
9.82.3.2 getPredictors() . . . 541
9.83 LinkPred::PerfCurve< PredResultsT > Class Template Reference . . . 541
9.83.1 Detailed Description . . . 543
9.83.2 Member Typedef Documentation . . . 543
9.83.2.1 ScoresItT . . . 543

9.83.3 Constructor & Destructor Documentation 543
9.83.3.1 PerfCurve() [1/4] 543
9.83.3.2 PerfCurve() [2/4] 543
9.83.3.3 PerfCurve() [3/4] 544
9.83.3.4 PerfCurve() [4/4] 544
9.83.3.5 ∼PerfCurve() 544
9.83.4 Member Function Documentation 544
9.83.4.1 eval() [1/2] 545
9.83.4.2 eval() [2/2] 545
9.83.4.3 getCurve() [1/2] 545
9.83.4.4 getCurve() [2/2] 546
9.83.4.5 operator=() [1/2] 546
9.83.4.6 operator=() [2/2] 547
9.84 LinkPred::PerfeEvalExpDescp< Network > Struct Template Reference 547
9.84.1 Detailed Description 547
9.84.2 Member Data Documentation 548
9.84.2.1 fnRatio 548
9.84.2.2 keepConnected 548
9.84.2.3 nbTestRuns 548
9.84.2.4 out 548
9.84.2.5 ratioEnd 548
9.84.2.6 ratioStart 548
9.84.2.7 ratioStep 549
9.84.2.8 refNet 549
9.84.2.9 seed 549
9.84.2.10 timingEnabled 549
9.84.2.11 tnRatio 549
9.85 LinkPred::PerfEvalExp< Network, TestDataT, LPredictorT, PredResultsT, PerfMeasureT > Class Template Reference 549
9.85.1 Detailed Description 550
9.85.2 Constructor & Destructor Documentation 550
9.85.2.1 PerfEvalExp() [1/3] 550
9.85.2.2 PerfEvalExp() [2/3] 551
9.85.2.3 PerfEvalExp() [3/3] 551
9.85.2.4 ∼PerfEvalExp() 551
9.85.3 Member Function Documentation 552
9.85.3.1 getFactory() 552
9.85.3.2 getOutPrec() 552
9.85.3.3 getPed() 552
9.85.3.4 operator=() [1/2] 553
9.85.3.5 operator=() [2/2] 553
9.85.3.6 resultsBegin() 553

9.85.3.7 resultsEnd() 554
9.85.3.8 run() 554
9.85.3.9 runNoTiming() 554
9.85.3.10 runTiming() 554
9.85.3.11 setOutPrec() 554
9.86 LinkPred::PerfEvaluator< TestDataT, LPredictorT, PredResultsT, PerfMeasureT > Class Template Reference 555
9.86.1 Detailed Description 555
9.86.2 Constructor & Destructor Documentation 556
9.86.2.1 PerfEvaluator() [1/3] 556
9.86.2.2 PerfEvaluator() [2/3] 556
9.86.2.3 PerfEvaluator() [3/3] 556
9.86.2.4 ∼PerfEvaluator() 557
9.86.3 Member Function Documentation 557
9.86.3.1 addPerfMeasure() 557
9.86.3.2 addPredictor() 557
9.86.3.3 eval() 559
9.86.3.4 evalNoTiming() 559
9.86.3.5 evalTiming() 559
9.86.3.6 getNbPerfMeasures() 559
9.86.3.7 getNbPredictors() 560
9.86.3.8 getPredictor() 560
9.86.3.9 isTimingEnabled() 560
9.86.3.10 operator=() [1/2] 561
9.86.3.11 operator=() [2/2] 561
9.86.3.12 resultsBegin() 561
9.86.3.13 resultsEnd() 562
9.86.3.14 setTimingEnabled() 562
9.87 LinkPred::PerfMeasure< PredResultsT > Class Template Reference 562
9.87.1 Detailed Description 563
9.87.2 Member Typedef Documentation 563
9.87.2.1 ScoresItT 563
9.87.3 Constructor & Destructor Documentation 563
9.87.3.1 PerfMeasure() [1/4] 564
9.87.3.2 PerfMeasure() [2/4] 564
9.87.3.3 PerfMeasure() [3/4] 564
9.87.3.4 PerfMeasure() [4/4] 564
9.87.3.5 ∼PerfMeasure() 565
9.87.4 Member Function Documentation 565
9.87.4.1 eval() [1/2] 565
9.87.4.2 eval() [2/2] 565
9.87.4.3 getName() 566

9.87.4.4 operator=() [1/2] 566
9.87.4.5 operator=() [2/2] 566
9.87.4.6 requiresNeg() 567
9.87.4.7 requiresPos() 567
9.87.4.8 requiresShuffling() 567
9.87.4.9 setName() 567
9.88 LinkPred::Simp::PerfRes Struct Reference 568
9.88.1 Detailed Description 568
9.88.2 Member Data Documentation 568
9.88.2.1 name 568
9.88.2.2 res 568
9.89 LinkPred::PR< PredResultsT > Class Template Reference 569
9.89.1 Detailed Description 570
9.89.2 Member Typedef Documentation 571
9.89.2.1 ScoresItT 571
9.89.3 Member Enumeration Documentation 571
9.89.3.1 InterpolMethod 571
9.89.4 Constructor & Destructor Documentation 571
9.89.4.1 PR() [1/4] 571
9.89.4.2 PR() [2/4] 571
9.89.4.3 PR() [3/4] 572
9.89.4.4 PR() [4/4] 572
9.89.4.5 ∼PR() 572
9.89.5 Member Function Documentation 572
9.89.5.1 eval() [1/2] 573
9.89.5.2 eval() [2/2] 573
9.89.5.3 getCurve() [1/2] 573
9.89.5.4 getCurve() [2/2] 574
9.89.5.5 getInterpolMethod() 574
9.89.5.6 getPRAuc() 575
9.89.5.7 getPRAucDGI() 575
9.89.5.8 getPRAucLIN() 576
9.89.5.9 getPRCurve() 576
9.89.5.10 getThresholds() 577
9.89.5.11 operator=() [1/2] 577
9.89.5.12 operator=() [2/2] 577
9.89.5.13 setInterpolMethod() 578
9.90 LinkPred::Simp::Predictor Class Reference 578
9.90.1 Detailed Description 580
9.90.2 Constructor & Destructor Documentation 580
9.90.2.1 Predictor() [1/2] 580
9.90.2.2 Predictor() [2/2] 581

9.90.2.3 ~Predictor() 581
9.90.3 Member Function Documentation 581
9.90.3.1 getID() 581
9.90.3.2 getLabel() 581
9.90.3.3 getNbNodes() 582
9.90.3.4 isEdgeByID() 582
9.90.3.5 isEdgeByLabel() 582
9.90.3.6 loadnet() 583
9.90.3.7 predADA() 583
9.90.3.8 predAllADA() 583
9.90.3.9 predAllADAByID() 584
9.90.3.10 predAllCNE() 584
9.90.3.11 predAllCNEByID() 584
9.90.3.12 predAllCRA() 584
9.90.3.13 predAllCRAByID() 585
9.90.3.14 predAllECL() 585
9.90.3.15 predAllECLByID() 585
9.90.3.16 predAllESM() 586
9.90.3.17 predAllESMByID() 587
9.90.3.18 predAllFBM() 587
9.90.3.19 predAllFBMByID() 587
9.90.3.20 predAllHDI() 589
9.90.3.21 predAllHDIByID() 589
9.90.3.22 predAllHPI() 589
9.90.3.23 predAllHPIByID() 590
9.90.3.24 predAllHRG() 590
9.90.3.25 predAllHRGByID() 590
9.90.3.26 predAllHYP() 591
9.90.3.27 predAllHYPByID() 591
9.90.3.28 predAllJID() 592
9.90.3.29 predAllJIDByID() 592
9.90.3.30 predAllKAB() 592
9.90.3.31 predAllKABByID() 593
9.90.3.32 predAllLCP() 593
9.90.3.33 predAllLCPByID() 593
9.90.3.34 predAllLHN() 594
9.90.3.35 predAllLHNByID() 594
9.90.3.36 predAllPAT() 594
9.90.3.37 predAllPATByID() 594
9.90.3.38 predAllRAL() 595
9.90.3.39 predAllRALByID() 595
9.90.3.40 predAllRND() 595

9.90.3.41 predAllRNDByID() . . . 595
9.90.3.42 predAllSAI() . . . 596
9.90.3.43 predAllSAIByID() . . . 596
9.90.3.44 predAllSBM() . . . 596
9.90.3.45 predAllSBMByID() . . . 597
9.90.3.46 predAllSHP() . . . 597
9.90.3.47 predAllSHPByID() . . . 597
9.90.3.48 predAllSOI() . . . 598
9.90.3.49 predAllSOIByID() . . . 598
9.90.3.50 predCNE() . . . 598
9.90.3.51 predCRA() . . . 599
9.90.3.52 predECL() . . . 599
9.90.3.53 predESM() . . . 599
9.90.3.54 predFBM() . . . 600
9.90.3.55 predHDI() . . . 600
9.90.3.56 predHPI() . . . 601
9.90.3.57 predHRG() . . . 601
9.90.3.58 predHYP() . . . 601
9.90.3.59 predJID() . . . 602
9.90.3.60 predKAB() . . . 602
9.90.3.61 predLCP() . . . 602
9.90.3.62 predLHN() . . . 603
9.90.3.63 predPAT() . . . 603
9.90.3.64 predRAL() . . . 603
9.90.3.65 predRND() . . . 604
9.90.3.66 predSAI() . . . 604
9.90.3.67 predSBM() . . . 604
9.90.3.68 predSHP() . . . 604
9.90.3.69 predSOI() . . . 605
9.90.3.70 predTopADA() . . . 605
9.90.3.71 predTopCNE() . . . 605
9.90.3.72 predTopCRA() . . . 606
9.90.3.73 predTopECL() . . . 606
9.90.3.74 predTopESM() . . . 607
9.90.3.75 predTopFBM() . . . 607
9.90.3.76 predTopHDI() . . . 608
9.90.3.77 predTopHPI() . . . 608
9.90.3.78 predTopHRG() . . . 609
9.90.3.79 predTopHYP() . . . 609
9.90.3.80 predTopJID() . . . 610
9.90.3.81 predTopKAB() . . . 610
9.90.3.82 predTopLCP() . . . 610

9.90.3.83 predTopLHN() . . . 611
9.90.3.84 predTopPAT() . . . 611
9.90.3.85 predTopRAL() . . . 611
9.90.3.86 predTopRND() . . . 612
9.90.3.87 predTopSAI() . . . 612
9.90.3.88 predTopSBM() . . . 613
9.90.3.89 predTopSHP() . . . 613
9.90.3.90 predTopSOI() . . . 613
9.91 LinkPred::PredResults< TestDataT, LPredictorT, ScoresContainerT > Class Template Reference . . . 614
9.91.1 Detailed Description . . . 615
9.91.2 Member Typedef Documentation . . . 615
9.91.2.1 ScoresItT . . . 615
9.91.3 Constructor & Destructor Documentation . . . 615
9.91.3.1 PredResults() [1/3] . . . 616
9.91.3.2 PredResults() [2/3] . . . 616
9.91.3.3 PredResults() [3/3] . . . 616
9.91.3.4 ∼PredResults() . . . 616
9.91.4 Member Function Documentation . . . 617
9.91.4.1 compNegScores() . . . 617
9.91.4.2 compPosScores() . . . 617
9.91.4.3 compTopScores() . . . 617
9.91.4.4 getNbTop() . . . 617
9.91.4.5 getNegSortOrder() . . . 618
9.91.4.6 getPosSortOrder() . . . 618
9.91.4.7 getPredictor() . . . 618
9.91.4.8 getTestData() . . . 619
9.91.4.9 isNegComputed() . . . 619
9.91.4.10 isPosComputed() . . . 619
9.91.4.11 isTopComputed() . . . 619
9.91.4.12 negBegin() . . . 620
9.91.4.13 negEnd() . . . 620
9.91.4.14 negStrmBegin() . . . 620
9.91.4.15 negStrmEnd() . . . 620
9.91.4.16 operator=() [1/2] . . . 621
9.91.4.17 operator=() [2/2] . . . 622
9.91.4.18 posBegin() . . . 622
9.91.4.19 posEnd() . . . 622
9.91.4.20 posStrmBegin() . . . 623
9.91.4.21 posStrmEnd() . . . 623
9.91.4.22 sortNeg() . . . 623
9.91.4.23 sortPos() . . . 623
9.91.4.24 topBegin() . . . 625

9.91.4.25 topEdgesBegin() 625
9.91.4.26 topEdgesEnd() 625
9.91.4.27 topEnd() 626
9.92 LinkPred::Simp::Evaluator::Factory::PSTParams Struct Reference 626
9.92.1 Detailed Description 626
9.92.2 Member Data Documentation 626
9.92.2.1 fileName 626
9.93 LinkPred::RandomGen Class Reference 627
9.93.1 Detailed Description 627
9.93.2 Constructor & Destructor Documentation 627
9.93.2.1 RandomGen() [1/4] 627
9.93.2.2 RandomGen() [2/4] 627
9.93.2.3 RandomGen() [3/4] 628
9.93.2.4 RandomGen() [4/4] 628
9.93.2.5 ∼RandomGen() 628
9.93.3 Member Function Documentation 628
9.93.3.1 getBool() 628
9.93.3.2 getDouble() 629
9.93.3.3 getGeo() 629
9.93.3.4 getInt() 629
9.93.3.5 getPL() 630
9.93.3.6 getSeed() 630
9.93.3.7 getSInt() 630
9.93.3.8 getUInt() 631
9.93.3.9 operator=() [1/2] 631
9.93.3.10 operator=() [2/2] 631
9.94 LinkPred::RndClassifier< InRndIt, OutRndIt, ScoreRndIt > Class Template Reference 632
9.94.1 Detailed Description 632
9.94.2 Constructor & Destructor Documentation 633
9.94.2.1 RndClassifier() [1/3] 633
9.94.2.2 RndClassifier() [2/3] 633
9.94.2.3 RndClassifier() [3/3] 633
9.94.2.4 ∼RndClassifier() 635
9.94.3 Member Function Documentation 635
9.94.3.1 learn() 635
9.94.3.2 operator=() [1/2] 635
9.94.3.3 operator=() [2/2] 636
9.94.3.4 predict() 636
9.95 LinkPred::UNetwork< LabelT, NodeIDT, EdgeT >::RndEdgeIt Class Reference 636
9.95.1 Detailed Description 638
9.95.2 Member Typedef Documentation 638
9.95.2.1 pointer 638

9.95.2.2 reference . . . 638
9.95.3 Constructor & Destructor Documentation . . . 638
9.95.3.1 RndEdgeIt() [1/2] . . . 638
9.95.3.2 RndEdgeIt() [2/2] . . . 639
9.95.4 Member Function Documentation . . . 639
9.95.4.1 operator"!=() . . . 639
9.95.4.2 operator*() . . . 639
9.95.4.3 operator++() [1/2] . . . 640
9.95.4.4 operator++() [2/2] . . . 640
9.95.4.5 operator->() . . . 640
9.95.4.6 operator=() [1/2] . . . 640
9.95.4.7 operator=() [2/2] . . . 641
9.95.4.8 operator==() . . . 641
9.96 LinkPred::DNetwork< LabelT, NodeIDT, EdgeT >::RndEdgeIt Class Reference . . . 641
9.96.1 Detailed Description . . . 643
9.96.2 Member Typedef Documentation . . . 643
9.96.2.1 pointer . . . 643
9.96.2.2 reference . . . 643
9.96.3 Constructor & Destructor Documentation . . . 643
9.96.3.1 RndEdgeIt() [1/2] . . . 643
9.96.3.2 RndEdgeIt() [2/2] . . . 644
9.96.4 Member Function Documentation . . . 644
9.96.4.1 operator"!=() . . . 644
9.96.4.2 operator*() . . . 644
9.96.4.3 operator++() [1/2] . . . 645
9.96.4.4 operator++() [2/2] . . . 645
9.96.4.5 operator->() . . . 645
9.96.4.6 operator=() [1/2] . . . 645
9.96.4.7 operator=() [2/2] . . . 646
9.96.4.8 operator==() . . . 646
9.97 LinkPred::DNetwork< LabelT, NodeIDT, EdgeT >::RndNodeIt Class Reference . . . 646
9.97.1 Detailed Description . . . 648
9.97.2 Member Typedef Documentation . . . 648
9.97.2.1 pointer . . . 648
9.97.2.2 reference . . . 648
9.97.3 Constructor & Destructor Documentation . . . 648
9.97.3.1 RndNodeIt() [1/2] . . . 648
9.97.3.2 RndNodeIt() [2/2] . . . 649
9.97.4 Member Function Documentation . . . 649
9.97.4.1 operator"!=() . . . 649
9.97.4.2 operator*() . . . 649
9.97.4.3 operator++() [1/2] . . . 650

9.97.4.4 operator++() [2/2] 650
9.97.4.5 operator->() 650
9.97.4.6 operator=() [1/2] 650
9.97.4.7 operator=() [2/2] 651
9.97.4.8 operator==() 651
9.98 LinkPred::UNetwork< LabelT, NodeIDT, EdgeT >::RndNodeIt Class Reference 651
9.98.1 Detailed Description 653
9.98.2 Member Typedef Documentation 653
9.98.2.1 pointer 653
9.98.2.2 reference 653
9.98.3 Constructor & Destructor Documentation 653
9.98.3.1 RndNodeIt() [1/2] 653
9.98.3.2 RndNodeIt() [2/2] 654
9.98.4 Member Function Documentation 654
9.98.4.1 operator"!=() 654
9.98.4.2 operator*() 654
9.98.4.3 operator++() [1/2] 655
9.98.4.4 operator++() [2/2] 655
9.98.4.5 operator->() 655
9.98.4.6 operator=() [1/2] 655
9.98.4.7 operator=() [2/2] 656
9.98.4.8 operator==() 656
9.99 LinkPred::UNetwork< LabelT, NodeIDT, EdgeT >::RndNonEdgeIt Class Reference 656
9.99.1 Detailed Description 658
9.99.2 Member Typedef Documentation 658
9.99.2.1 pointer 658
9.99.2.2 reference 658
9.99.3 Constructor & Destructor Documentation 658
9.99.3.1 RndNonEdgeIt() [1/2] 658
9.99.3.2 RndNonEdgeIt() [2/2] 659
9.99.4 Member Function Documentation 659
9.99.4.1 operator"!=() 659
9.99.4.2 operator*() 659
9.99.4.3 operator++() [1/2] 660
9.99.4.4 operator++() [2/2] 660
9.99.4.5 operator->() 660
9.99.4.6 operator=() [1/2] 660
9.99.4.7 operator=() [2/2] 661
9.99.4.8 operator==() 661
9.100 LinkPred::DNetwork< LabelT, NodeIDT, EdgeT >::RndNonEdgeIt Class Reference 661
9.100.1 Detailed Description 663
9.100.2 Member Typedef Documentation 663

9.100.2.1 pointer 663
9.100.2.2 reference 663
9.100.3 Constructor & Destructor Documentation 663
9.100.3.1 RndNonEdgeIt() [1/2] 663
9.100.3.2 RndNonEdgeIt() [2/2] 664
9.100.4 Member Function Documentation 664
9.100.4.1 operator"!=() 664
9.100.4.2 operator*() 664
9.100.4.3 operator++() [1/2] 665
9.100.4.4 operator++() [2/2] 665
9.100.4.5 operator->() 665
9.100.4.6 operator=() [1/2] 665
9.100.4.7 operator=() [2/2] 666
9.100.4.8 operator==() 666
9.101 LinkPred::Simp::Evaluator::Factory::RNDParams Struct Reference 666
9.101.1 Detailed Description 667
9.101.2 Member Data Documentation 667
9.101.2.1 seed 667
9.102 LinkPred::ROC< PredResultsT > Class Template Reference 667
9.102.1 Detailed Description 669
9.102.2 Member Typedef Documentation 669
9.102.2.1 ScoresItT 669
9.102.3 Constructor & Destructor Documentation 669
9.102.3.1 ROC() [1/4] 669
9.102.3.2 ROC() [2/4] 669
9.102.3.3 ROC() [3/4] 670
9.102.3.4 ROC() [4/4] 670
9.102.3.5 ~ROC() 670
9.102.4 Member Function Documentation 670
9.102.4.1 eval() [1/2] 671
9.102.4.2 eval() [2/2] 671
9.102.4.3 evalNoStream() 671
9.102.4.4 evalStream() 672
9.102.4.5 getCurve() [1/2] 672
9.102.4.6 getCurve() [2/2] 673
9.102.4.7 getROCAuc() 673
9.102.4.8 getROCAucStrm() 673
9.102.4.9 getROCCurve() 674
9.102.4.10 getThresholds() 674
9.102.4.11 isStrmEnabled() 675
9.102.4.12 isStrmNeg() 675
9.102.4.13 operator=() [1/2] 675

9.102.4.14 operator=() [2/2] 676
9.102.4.15 setStrmEnabled() 676
9.102.4.16 setStrmNeg() 676
9.103 LinkPred::Simp::Evaluator::Factory::SBMParams Struct Reference 677
9.103.1 Detailed Description 677
9.103.2 Member Data Documentation 677
9.103.2.1 maxIter 677
9.103.2.2 seed 677
9.104 LinkPred::PredResults< TestDataT, LPredictorT, ScoresContainerT >::ScoreIterator Class Reference 678
9.104.1 Detailed Description 679
9.104.2 Member Typedef Documentation 679
9.104.2.1 difference_type 679
9.104.2.2 pointer 680
9.104.2.3 reference 680
9.104.3 Constructor & Destructor Documentation 680
9.104.3.1 ScoreIterator() [1/3] 680
9.104.3.2 ScoreIterator() [2/3] 680
9.104.3.3 ScoreIterator() [3/3] 682
9.104.4 Member Function Documentation 682
9.104.4.1 operator"!=() 682
9.104.4.2 operator*() 682
9.104.4.3 operator+() 683
9.104.4.4 operator++() [1/2] 683
9.104.4.5 operator++() [2/2] 683
9.104.4.6 operator+=() 684
9.104.4.7 operator-() [1/2] 684
9.104.4.8 operator-() [2/2] 684
9.104.4.9 operator--() [1/2] 685
9.104.4.10 operator--() [2/2] 685
9.104.4.11 operator-=() 685
9.104.4.12 operator->() 686
9.104.4.13 operator<() 686
9.104.4.14 operator<=() 686
9.104.4.15 operator=() [1/2] 687
9.104.4.16 operator=() [2/2] 687
9.104.4.17 operator==() 687
9.104.4.18 operator>() 688
9.104.4.19 operator>=() 688
9.104.5 Friends And Related Function Documentation 688
9.104.5.1 PredResults 689
9.105 LinkPred::Simp::Evaluator::Factory::SHPParams Struct Reference 689
9.105.1 Detailed Description 689

9.105.2 Member Data Documentation 689
9.105.2.1 seed 689
9.106 LinkPred::SimMeasure Class Reference 690
9.106.1 Detailed Description 690
9.106.2 Constructor & Destructor Documentation 690
9.106.2.1 SimMeasure() [1/3] 691
9.106.2.2 SimMeasure() [2/3] 691
9.106.2.3 SimMeasure() [3/3] 691
9.106.2.4 ∼SimMeasure() 691
9.106.3 Member Function Documentation 691
9.106.3.1 getName() 691
9.106.3.2 operator=() [1/2] 692
9.106.3.3 operator=() [2/2] 692
9.106.3.4 setName() 692
9.106.3.5 sim() 692
9.107 LinkPred::TestData< Network, EdgeContT > Class Template Reference 693
9.107.1 Detailed Description 694
9.107.2 Member Typedef Documentation 694
9.107.2.1 Edge 695
9.107.2.2 EdgeIt 695
9.107.2.3 NetworkCSP 695
9.107.2.4 NetworkSP 695
9.107.2.5 NonEdgeIt 695
9.107.3 Member Enumeration Documentation 695
9.107.3.1 IteratorType 695
9.107.4 Constructor & Destructor Documentation 696
9.107.4.1 TestData() [1/4] 696
9.107.4.2 TestData() [2/4] 696
9.107.4.3 TestData() [3/4] 697
9.107.4.4 TestData() [4/4] 697
9.107.4.5 ∼TestData() 697
9.107.5 Member Function Documentation 698
9.107.5.1 genNeg() 698
9.107.5.2 genPos() 698
9.107.5.3 getEg() 698
9.107.5.4 getNbNeg() 698
9.107.5.5 getNbPos() 699
9.107.5.6 getNegClass() 699
9.107.5.7 getObsNet() 699
9.107.5.8 getPosClass() 699
9.107.5.9 getRefNet() 700
9.107.5.10 getRemLinksMap() 700

9.107.5.11 isLocked() 700
9.107.5.12 isTnGenerated() 700
9.107.5.13 isTpGenerated() 701
9.107.5.14 lock() 701
9.107.5.15 negBegin() 701
9.107.5.16 negEnd() 701
9.107.5.17 negStrmBegin() 702
9.107.5.18 negStrmEnd() 702
9.107.5.19 operator=() [1/2] 702
9.107.5.20 operator=() [2/2] 702
9.107.5.21 posBegin() 703
9.107.5.22 posEnd() 703
9.107.5.23 posStrmBegin() 703
9.107.5.24 posStrmEnd() 704
9.108 LinkPred::TestEdgeGen< Network, EdgeContT > Class Template Reference 704
9.108.1 Detailed Description 704
9.108.2 Constructor & Destructor Documentation 705
9.108.2.1 TestEdgeGen() [1/3] 705
9.108.2.2 TestEdgeGen() [2/3] 705
9.108.2.3 TestEdgeGen() [3/3] 706
9.108.2.4 ∼TestEdgeGen() 706
9.108.3 Member Function Documentation 706
9.108.3.1 generateTN() 706
9.108.3.2 generateTP() 707
9.108.3.3 operator=() [1/2] 707
9.108.3.4 operator=() [2/2] 707
9.108.3.5 tnBegin() 708
9.108.3.6 tnEnd() 708
9.108.3.7 tpBegin() 708
9.108.3.8 tpEnd() 709
9.109 LinkPred::TestData< Network, EdgeContT >::TestEdgeIt Class Reference 709
9.109.1 Detailed Description 711
9.109.2 Member Typedef Documentation 711
9.109.2.1 difference_type 711
9.109.2.2 pointer 711
9.109.2.3 reference 711
9.109.3 Constructor & Destructor Documentation 711
9.109.3.1 TestEdgeIt() [1/3] 711
9.109.3.2 TestEdgeIt() [2/3] 712
9.109.3.3 TestEdgeIt() [3/3] 712
9.109.4 Member Function Documentation 712
9.109.4.1 operator"!=() 712

9.109.4.2 operator∗() . . . 713
9.109.4.3 operator+() . . . 713
9.109.4.4 operator++() [1/2] . . . 713
9.109.4.5 operator++() [2/2] . . . 714
9.109.4.6 operator+=() . . . 714
9.109.4.7 operator-() [1/2] . . . 714
9.109.4.8 operator-() [2/2] . . . 715
9.109.4.9 operator--() [1/2] . . . 715
9.109.4.10 operator--() [2/2] . . . 715
9.109.4.11 operator-=() . . . 716
9.109.4.12 operator->() . . . 716
9.109.4.13 operator<() . . . 716
9.109.4.14 operator<=() . . . 717
9.109.4.15 operator=() [1/2] . . . 717
9.109.4.16 operator=() [2/2] . . . 717
9.109.4.17 operator==() . . . 718
9.109.4.18 operator>() . . . 718
9.109.4.19 operator>=() . . . 718
9.109.5 Friends And Related Function Documentation . . . 719
9.109.5.1 TestData . . . 719
9.110 LinkPred::TestEdgeGen< Network, EdgeContT >::TNEdgeIt Class Reference . . . 719
9.110.1 Detailed Description . . . 721
9.110.2 Member Typedef Documentation . . . 721
9.110.2.1 difference_type . . . 721
9.110.2.2 pointer . . . 721
9.110.2.3 reference . . . 721
9.110.3 Constructor & Destructor Documentation . . . 721
9.110.3.1 TNEdgeIt() [1/2] . . . 721
9.110.3.2 TNEdgeIt() [2/2] . . . 722
9.110.4 Member Function Documentation . . . 722
9.110.4.1 operator"!=() . . . 722
9.110.4.2 operator∗() . . . 722
9.110.4.3 operator+() . . . 723
9.110.4.4 operator++() [1/2] . . . 723
9.110.4.5 operator++() [2/2] . . . 723
9.110.4.6 operator+=() . . . 724
9.110.4.7 operator-() [1/2] . . . 724
9.110.4.8 operator-() [2/2] . . . 724
9.110.4.9 operator--() [1/2] . . . 725
9.110.4.10 operator--() [2/2] . . . 725
9.110.4.11 operator-=() . . . 725
9.110.4.12 operator->() . . . 726

9.110.4.13 operator<() 726
9.110.4.14 operator<=() 726
9.110.4.15 operator=() [1/2] 727
9.110.4.16 operator=() [2/2] 727
9.110.4.17 operator==() 727
9.110.4.18 operator>() 728
9.110.4.19 operator>=() 728
9.110.5 Friends And Related Function Documentation 728
9.110.5.1 TestEdgeGen 728
9.111 LinkPred::TestEdgeGen< Network, EdgeContT >::TPEdgeIt Class Reference 729
9.111.1 Detailed Description 730
9.111.2 Member Typedef Documentation 730
9.111.2.1 difference_type 730
9.111.2.2 pointer 731
9.111.2.3 reference 731
9.111.3 Constructor & Destructor Documentation 731
9.111.3.1 TPEdgeIt() [1/2] 731
9.111.3.2 TPEdgeIt() [2/2] 731
9.111.4 Member Function Documentation 732
9.111.4.1 operator"!=() 732
9.111.4.2 operator*() 732
9.111.4.3 operator+() 732
9.111.4.4 operator++() [1/2] 733
9.111.4.5 operator++() [2/2] 733
9.111.4.6 operator+=() 733
9.111.4.7 operator-() [1/2] 734
9.111.4.8 operator-() [2/2] 734
9.111.4.9 operator--() [1/2] 734
9.111.4.10 operator--() [2/2] 735
9.111.4.11 operator-=() 735
9.111.4.12 operator->() 735
9.111.4.13 operator<() 736
9.111.4.14 operator<=() 736
9.111.4.15 operator=() [1/2] 736
9.111.4.16 operator=() [2/2] 737
9.111.4.17 operator==() 737
9.111.4.18 operator>() 737
9.111.4.19 operator>=() 738
9.111.5 Friends And Related Function Documentation 738
9.111.5.1 TestEdgeGen 738
9.112 LinkPred::TPR< PredResultsT > Class Template Reference 738
9.112.1 Detailed Description 739

9.112.2 Member Typedef Documentation 740
9.112.2.1 ScoresItT 740
9.112.3 Constructor & Destructor Documentation 740
9.112.3.1 TPR() [1/4] 740
9.112.3.2 TPR() [2/4] 740
9.112.3.3 TPR() [3/4] 741
9.112.3.4 TPR() [4/4] 741
9.112.3.5 ∼TPR() 741
9.112.4 Member Function Documentation 741
9.112.4.1 eval() [1/2] 742
9.112.4.2 eval() [2/2] 742
9.112.4.3 evalUsingPredict() 742
9.112.4.4 evalUsingTop() 743
9.112.4.5 getTPR() 743
9.112.4.6 isUseTopMethod() 743
9.112.4.7 operator=() [1/2] 744
9.112.4.8 operator=() [2/2] 744
9.112.4.9 requiresNeg() 744
9.112.4.10 requiresShuffling() 745
9.112.4.11 setUseTopMethod() 745
9.113 LinkPred::UADAPredictor< Network, EdgeRndIt, ScoreRndIt, EdgeRndOutIt > Class Template Reference 745
9.113.1 Detailed Description 746
9.113.2 Constructor & Destructor Documentation 747
9.113.2.1 UADAPredictor() [1/3] 747
9.113.2.2 UADAPredictor() [2/3] 747
9.113.2.3 UADAPredictor() [3/3] 747
9.113.2.4 ∼UADAPredictor() 748
9.113.3 Member Function Documentation 748
9.113.3.1 init() 748
9.113.3.2 learn() 748
9.113.3.3 operator=() [1/2] 748
9.113.3.4 operator=() [2/2] 749
9.113.3.5 predict() 749
9.113.3.6 score() 749
9.113.3.7 top() 750
9.114 LinkPred::UCNEPredictor< Network, EdgeRndIt, ScoreRndIt, EdgeRndOutIt > Class Template Reference 751
9.114.1 Detailed Description 752
9.114.2 Constructor & Destructor Documentation 752
9.114.2.1 UCNEPredictor() [1/3] 752
9.114.2.2 UCNEPredictor() [2/3] 752
9.114.2.3 UCNEPredictor() [3/3] 753

9.114.2.4 ~UCNEPredictor() . . . 753
9.114.3 Member Function Documentation . . . 753
9.114.3.1 init() . . . 753
9.114.3.2 learn() . . . 754
9.114.3.3 operator=() [1/2] . . . 754
9.114.3.4 operator=() [2/2] . . . 754
9.114.3.5 predict() . . . 755
9.114.3.6 score() . . . 755
9.114.3.7 top() . . . 755
9.115 LinkPred::UCRAPredictor< Network, EdgeRndIt, ScoreRndIt, EdgeRndOutIt > Class Template Reference . . . 756
9.115.1 Detailed Description . . . 757
9.115.2 Constructor & Destructor Documentation . . . 758
9.115.2.1 UCRAPredictor() [1/3] . . . 758
9.115.2.2 UCRAPredictor() [2/3] . . . 758
9.115.2.3 UCRAPredictor() [3/3] . . . 758
9.115.2.4 ~UCRAPredictor() . . . 759
9.115.3 Member Function Documentation . . . 759
9.115.3.1 init() . . . 759
9.115.3.2 learn() . . . 759
9.115.3.3 operator=() [1/2] . . . 759
9.115.3.4 operator=() [2/2] . . . 760
9.115.3.5 predict() . . . 760
9.115.3.6 score() . . . 760
9.115.3.7 top() . . . 761
9.116 LinkPred::UCSTPredictor< Network, EdgeRndIt, ScoreRndIt, EdgeRndOutIt > Class Template Reference . . . 762
9.116.1 Detailed Description . . . 763
9.116.2 Constructor & Destructor Documentation . . . 763
9.116.2.1 UCSTPredictor() [1/3] . . . 763
9.116.2.2 UCSTPredictor() [2/3] . . . 763
9.116.2.3 UCSTPredictor() [3/3] . . . 765
9.116.2.4 ~UCSTPredictor() . . . 765
9.116.3 Member Function Documentation . . . 765
9.116.3.1 init() . . . 765
9.116.3.2 learn() . . . 766
9.116.3.3 operator=() [1/2] . . . 766
9.116.3.4 operator=() [2/2] . . . 766
9.116.3.5 predict() . . . 767
9.116.3.6 score() . . . 767
9.116.3.7 top() . . . 767
9.117 LinkPred::UECLPredictor< Network, EdgeRndIt, ScoreRndIt, EdgeRndOutIt > Class Template Reference . . . 768

9.117.1 Detailed Description 769
9.117.2 Constructor & Destructor Documentation 770
9.117.2.1 UECLPredictor() [1/3] 770
9.117.2.2 UECLPredictor() [2/3] 770
9.117.2.3 UECLPredictor() [3/3] 770
9.117.2.4 ∼UECLPredictor() 771
9.117.3 Member Function Documentation 771
9.117.3.1 getClassifier() 771
9.117.3.2 getEncoder() 771
9.117.3.3 getNegRatio() 772
9.117.3.4 getPosRatio() 772
9.117.3.5 init() 772
9.117.3.6 learn() 772
9.117.3.7 operator=() [1/2] 773
9.117.3.8 operator=() [2/2] 773
9.117.3.9 score() 773
9.117.3.10 setClassifier() 774
9.117.3.11 setEncoder() 774
9.117.3.12 setNegRatio() 774
9.117.3.13 setPosRatio() 775
9.118 LinkPred::UESMPredictor< Network, EdgeRndIt, ScoreRndIt, EdgeRndOutIt > Class Template Reference 775
9.118.1 Detailed Description 776
9.118.2 Constructor & Destructor Documentation 777
9.118.2.1 UESMPredictor() [1/3] 777
9.118.2.2 UESMPredictor() [2/3] 777
9.118.2.3 UESMPredictor() [3/3] 777
9.118.2.4 ∼UESMPredictor() 778
9.118.3 Member Function Documentation 778
9.118.3.1 getEncoder() 778
9.118.3.2 getSimMeasure() 778
9.118.3.3 init() 779
9.118.3.4 learn() 779
9.118.3.5 operator=() [1/2] 779
9.118.3.6 operator=() [2/2] 779
9.118.3.7 score() 780
9.118.3.8 setEncoder() 780
9.118.3.9 setSimMeasure() 781
9.119 LinkPred::UFBMPredictor< Network, EdgeRndIt, ScoreRndIt, EdgeRndOutIt > Class Template Reference 781
9.119.1 Detailed Description 782
9.119.2 Constructor & Destructor Documentation 782
9.119.2.1 UFBMPredictor() [1/3] 782

9.119.2.2 UFBMPredictor() [2/3] 783
9.119.2.3 UFBMPredictor() [3/3] 783
9.119.2.4 ∼UFBMPredictor() 783
9.119.3 Member Function Documentation 784
9.119.3.1 getMaxIter() 784
9.119.3.2 init() 784
9.119.3.3 learn() 784
9.119.3.4 operator=() [1/2] 784
9.119.3.5 operator=() [2/2] 785
9.119.3.6 predict() 785
9.119.3.7 score() 785
9.119.3.8 setMaxIter() 786
9.120 LinkPred::UHDIPredictor< Network, EdgeRndIt, ScoreRndIt, EdgeRndOutIt > Class Template Reference 786
9.120.1 Detailed Description 787
9.120.2 Constructor & Destructor Documentation 788
9.120.2.1 UHDIPredictor() [1/3] 788
9.120.2.2 UHDIPredictor() [2/3] 788
9.120.2.3 UHDIPredictor() [3/3] 788
9.120.2.4 ∼UHDIPredictor() 789
9.120.3 Member Function Documentation 789
9.120.3.1 init() 789
9.120.3.2 learn() 789
9.120.3.3 operator=() [1/2] 790
9.120.3.4 operator=() [2/2] 790
9.120.3.5 predict() 790
9.120.3.6 score() 791
9.120.3.7 top() 791
9.121 LinkPred::UHPIPredictor< Network, EdgeRndIt, ScoreRndIt, EdgeRndOutIt > Class Template Reference 792
9.121.1 Detailed Description 793
9.121.2 Constructor & Destructor Documentation 793
9.121.2.1 UHPIPredictor() [1/3] 793
9.121.2.2 UHPIPredictor() [2/3] 793
9.121.2.3 UHPIPredictor() [3/3] 794
9.121.2.4 ∼UHPIPredictor() 794
9.121.3 Member Function Documentation 794
9.121.3.1 init() 794
9.121.3.2 learn() 795
9.121.3.3 operator=() [1/2] 795
9.121.3.4 operator=() [2/2] 795
9.121.3.5 predict() 796
9.121.3.6 score() 796

9.121.3.7 top() 796
9.122 LinkPred::UHRGPredictor< Network, EdgeRndIt, ScoreRndIt, EdgeRndOutIt > Class Template Reference 797
9.122.1 Detailed Description 798
9.122.2 Constructor & Destructor Documentation 799
9.122.2.1 UHRGPredictor() [1/3] 799
9.122.2.2 UHRGPredictor() [2/3] 799
9.122.2.3 UHRGPredictor() [3/3] 799
9.122.2.4 ∼UHRGPredictor() 799
9.122.3 Member Function Documentation 800
9.122.3.1 getNbBeans() 800
9.122.3.2 getNbSamples() 800
9.122.3.3 init() 800
9.122.3.4 learn() 801
9.122.3.5 operator=() [1/2] 801
9.122.3.6 operator=() [2/2] 801
9.122.3.7 predict() 801
9.122.3.8 score() 802
9.122.3.9 setNbBeans() 802
9.122.3.10 setNbSamples() 803
9.123 LinkPred::UHYPPredictor< Network, EdgeRndIt, ScoreRndIt, EdgeRndOutIt > Class Template Reference 803
9.123.1 Detailed Description 804
9.123.2 Constructor & Destructor Documentation 804
9.123.2.1 UHYPPredictor() [1/3] 805
9.123.2.2 UHYPPredictor() [2/3] 805
9.123.2.3 UHYPPredictor() [3/3] 805
9.123.2.4 ∼UHYPPredictor() 806
9.123.3 Member Function Documentation 806
9.123.3.1 getGamma() 806
9.123.3.2 getL() 806
9.123.3.3 getM() 806
9.123.3.4 getSeed() 807
9.123.3.5 getT() 807
9.123.3.6 getZeta() 807
9.123.3.7 init() 807
9.123.3.8 learn() 808
9.123.3.9 operator=() [1/2] 808
9.123.3.10 operator=() [2/2] 808
9.123.3.11 predict() 809
9.123.3.12 score() 809
9.123.3.13 setGamma() 809
9.123.3.14 setL() 810

9.123.3.15 setM() 810
9.123.3.16 setT() 810
9.123.3.17 setZeta() 811
9.124 LinkPred::UJIDPredictor< Network, EdgeRndIt, ScoreRndIt, EdgeRndOutIt > Class Template Reference 811
9.124.1 Detailed Description 812
9.124.2 Constructor & Destructor Documentation 813
9.124.2.1 UJIDPredictor() [1/3] 813
9.124.2.2 UJIDPredictor() [2/3] 813
9.124.2.3 UJIDPredictor() [3/3] 813
9.124.2.4 ∼UJIDPredictor() 814
9.124.3 Member Function Documentation 814
9.124.3.1 init() 814
9.124.3.2 learn() 814
9.124.3.3 operator=() [1/2] 814
9.124.3.4 operator=() [2/2] 815
9.124.3.5 predict() 815
9.124.3.6 score() 815
9.124.3.7 top() 816
9.125 LinkPred::UKABPredictor< Network, EdgeRndIt, ScoreRndIt, EdgeRndOutIt > Class Template Reference 817
9.125.1 Detailed Description 818
9.125.2 Member Typedef Documentation 818
9.125.2.1 Edge 818
9.125.2.2 NodeID 818
9.125.3 Constructor & Destructor Documentation 819
9.125.3.1 UKABPredictor() [1/3] 819
9.125.3.2 UKABPredictor() [2/3] 819
9.125.3.3 UKABPredictor() [3/3] 819
9.125.3.4 ∼UKABPredictor() 820
9.125.4 Member Function Documentation 820
9.125.4.1 getCacheLevel() 820
9.125.4.2 getHorizLim() 820
9.125.4.3 init() 821
9.125.4.4 learn() 821
9.125.4.5 operator=() [1/2] 821
9.125.4.6 operator=() [2/2] 821
9.125.4.7 predict() 822
9.125.4.8 score() 822
9.125.4.9 setCacheLevel() 823
9.125.4.10 setHorizLim() 823
9.125.4.11 top() 823

9.126 LinkPred::ULCPPredictor< Network, EdgeRndIt, ScoreRndIt, EdgeRndOutIt > Class Template Reference 824
9.126.1 Detailed Description 825
9.126.2 Constructor & Destructor Documentation 825
9.126.2.1 ULCPPredictor() [1/3] 825
9.126.2.2 ULCPPredictor() [2/3] 826
9.126.2.3 ULCPPredictor() [3/3] 826
9.126.2.4 ~ULCPPredictor() 826
9.126.3 Member Function Documentation 826
9.126.3.1 getEpsilon() 827
9.126.3.2 init() 827
9.126.3.3 learn() 827
9.126.3.4 operator=() [1/2] 827
9.126.3.5 operator=() [2/2] 828
9.126.3.6 predict() 828
9.126.3.7 score() 828
9.126.3.8 setEpsilon() 829
9.127 LinkPred::ULHNPredictor< Network, EdgeRndIt, ScoreRndIt, EdgeRndOutIt > Class Template Reference 829
9.127.1 Detailed Description 830
9.127.2 Constructor & Destructor Documentation 831
9.127.2.1 ULHNPredictor() [1/3] 831
9.127.2.2 ULHNPredictor() [2/3] 831
9.127.2.3 ULHNPredictor() [3/3] 831
9.127.2.4 ~ULHNPredictor() 832
9.127.3 Member Function Documentation 832
9.127.3.1 init() 832
9.127.3.2 learn() 832
9.127.3.3 operator=() [1/2] 833
9.127.3.4 operator=() [2/2] 833
9.127.3.5 predict() 833
9.127.3.6 score() 834
9.127.3.7 top() 834
9.128 LinkPred::ULPredictor< NetworkT, EdgeRndItT, ScoreRndItT, EdgeRndOutItT > Class Template Reference 835
9.128.1 Detailed Description 835
9.128.2 Member Typedef Documentation 836
9.128.2.1 Edge 836
9.128.2.2 EdgeRndIt 836
9.128.2.3 EdgeRndOutIt 836
9.128.2.4 Network 837
9.128.2.5 NodeID 837
9.128.2.6 ScoreRndIt 837

9.128.3 Constructor & Destructor Documentation 837
9.128.3.1 ULPredictor() [1/3] 837
9.128.3.2 ULPredictor() [2/3] 838
9.128.3.3 ULPredictor() [3/3] 838
9.128.3.4 ∼ULPredictor() 838
9.128.4 Member Function Documentation 838
9.128.4.1 getName() 839
9.128.4.2 getNet() 839
9.128.4.3 init() 839
9.128.4.4 learn() 840
9.128.4.5 operator=() [1/2] 840
9.128.4.6 operator=() [2/2] 840
9.128.4.7 predict() 841
9.128.4.8 predictNeg() 841
9.128.4.9 score() 842
9.128.4.10 setName() 842
9.128.4.11 top() 843
9.129 LinkPred::UMPSPredictor< Network, EdgeRndIt, ScoreRndIt, EdgeRndOutIt > Class Template Reference 843
9.129.1 Detailed Description 845
9.129.2 Member Enumeration Documentation 846
9.129.2.1 EdgeLengthMethod 846
9.129.2.2 LambdaMethod 846
9.129.2.3 LandmarkStrategy 846
9.129.3 Constructor & Destructor Documentation 847
9.129.3.1 UMPSPredictor() [1/3] 847
9.129.3.2 UMPSPredictor() [2/3] 847
9.129.3.3 UMPSPredictor() [3/3] 847
9.129.3.4 ∼UMPSPredictor() 848
9.129.4 Member Function Documentation 848
9.129.4.1 getAsp() 848
9.129.4.2 getCacheLevel() 848
9.129.4.3 getEdgeLengthMethod() 849
9.129.4.4 getLambda() 849
9.129.4.5 getLambdaMethod() 849
9.129.4.6 getLambdaNegEstRatio() 849
9.129.4.7 getLambdaPosEstRatio() 850
9.129.4.8 getLambdaStep() 850
9.129.4.9 getLandmarkRatio() 850
9.129.4.10 getLandmarkStrategy() 850
9.129.4.11 getLength() 851
9.129.4.12 getMaxLambda() 851

9.129.4.13 getMinLambda() 851
9.129.4.14 getNegScore() 851
9.129.4.15 getPerfMeasure() 852
9.129.4.16 getPosScore() 852
9.129.4.17 getTol() 852
9.129.4.18 init() 852
9.129.4.19 learn() 853
9.129.4.20 operator=() [1/2] 853
9.129.4.21 operator=() [2/2] 853
9.129.4.22 predict() 854
9.129.4.23 score() 854
9.129.4.24 setAsp() 854
9.129.4.25 setCacheLevel() 855
9.129.4.26 setEdgeLengthMethod() 855
9.129.4.27 setLambda() 855
9.129.4.28 setLambdaMethod() 856
9.129.4.29 setLambdaNegEstRatio() 856
9.129.4.30 setLambdaPosEstRatio() 856
9.129.4.31 setLambdaStep() 858
9.129.4.32 setLandmarkRatio() 858
9.129.4.33 setLandmarkStrategy() 858
9.129.4.34 setMaxLambda() 859
9.129.4.35 setMinLambda() 859
9.129.4.36 setNegScore() 859
9.129.4.37 setPerfMeasure() 860
9.129.4.38 setPosScore() 860
9.129.4.39 setTol() 860
9.130 LinkPred::UNEDPredictor< Network, EdgeRndIt, ScoreRndIt, EdgeRndOutIt > Class Template Reference 861
9.130.1 Detailed Description 862
9.130.2 Member Typedef Documentation 862
9.130.2.1 Edge 862
9.130.2.2 NodeID 863
9.130.3 Constructor & Destructor Documentation 863
9.130.3.1 UNEDPredictor() [1/3] 863
9.130.3.2 UNEDPredictor() [2/3] 863
9.130.3.3 UNEDPredictor() [3/3] 863
9.130.3.4 ∼UNEDPredictor() 864
9.130.4 Member Function Documentation 864
9.130.4.1 init() 864
9.130.4.2 learn() 864
9.130.4.3 operator=() [1/2] 865

9.130.4.4 operator=() [2/2] 865
9.130.4.5 predict() 865
9.130.4.6 score() 866
9.130.4.7 top() 866
9.131 LinkPred::UNetwork< LabelT, NodeIDT, EdgeT > Class Template Reference 867
9.131.1 Detailed Description 869
9.131.2 Member Typedef Documentation 870
9.131.2.1 Edge 870
9.131.2.2 EdgeIt 870
9.131.2.3 EdgeMapSP 870
9.131.2.4 Label 870
9.131.2.5 LabelIt 870
9.131.2.6 NodeID 871
9.131.2.7 NodeIt 871
9.131.2.8 NodeMapSP 871
9.131.2.9 NodeSMapSP 871
9.131.3 Constructor & Destructor Documentation 871
9.131.3.1 UNetwork() [1/4] 871
9.131.3.2 UNetwork() [2/4] 871
9.131.3.3 UNetwork() [3/4] 872
9.131.3.4 UNetwork() [4/4] 872
9.131.3.5 ∼UNetwork() 872
9.131.4 Member Function Documentation 872
9.131.4.1 addEdge() 873
9.131.4.2 addNode() 873
9.131.4.3 assemble() 873
9.131.4.4 compareEdgeEnd() 873
9.131.4.5 coupleAtOrd() 874
9.131.4.6 coupleOrd() 874
9.131.4.7 createEdgeMap() 874
9.131.4.8 createEdgeMapSP() 875
9.131.4.9 createNodeMap() 875
9.131.4.10 createNodeMapSP() 876
9.131.4.11 createNodeSMap() 876
9.131.4.12 createNodeSMapSP() 876
9.131.4.13 edgesBegin() 877
9.131.4.14 edgesEnd() 877
9.131.4.15 end() 877
9.131.4.16 findLabel() 878
9.131.4.17 findNode() 878
9.131.4.18 generateERN() 878
9.131.4.19 generateREG() 879

9.131.4.20 generateRNC() . . . 879
9.131.4.21 getAvgDeg() . . . 879
9.131.4.22 getCC() [1/2] . . . 880
9.131.4.23 getCC() [2/2] . . . 880
9.131.4.24 getCommonNeighbors() [1/2] . . . 880
9.131.4.25 getCommonNeighbors() [2/2] . . . 881
9.131.4.26 getCSR() . . . 881
9.131.4.27 getDeg() . . . 881
9.131.4.28 getDegStat() . . . 882
9.131.4.29 getID() . . . 882
9.131.4.30 getLabel() . . . 882
9.131.4.31 getMaxDeg() . . . 883
9.131.4.32 getMinDeg() . . . 883
9.131.4.33 getNbCommonNeighbors() . . . 883
9.131.4.34 getNbCouples() . . . 884
9.131.4.35 getNbEdges() . . . 884
9.131.4.36 getNbInEdges() . . . 884
9.131.4.37 getNbNodes() . . . 885
9.131.4.38 getNbNonEdges() . . . 885
9.131.4.39 getNbPaths() . . . 885
9.131.4.40 isEdge() [1/2] . . . 885
9.131.4.41 isEdge() [2/2] . . . 886
9.131.4.42 labelsBegin() . . . 886
9.131.4.43 labelsEnd() . . . 887
9.131.4.44 makeEdge() . . . 887
9.131.4.45 neighbBegin() . . . 887
9.131.4.46 neighbEnd() . . . 888
9.131.4.47 nodesBegin() . . . 888
9.131.4.48 nodesDegBegin() . . . 888
9.131.4.49 nodesDegEnd() . . . 888
9.131.4.50 nodesEnd() . . . 889
9.131.4.51 nonEdgesBegin() . . . 889
9.131.4.52 nonEdgesEnd() . . . 889
9.131.4.53 operator=() [1/2] . . . 889
9.131.4.54 operator=() [2/2] . . . 890
9.131.4.55 print() . . . 890
9.131.4.56 printEdges() . . . 890
9.131.4.57 read() . . . 890
9.131.4.58 readEdges() . . . 891
9.131.4.59 reverseEdge() . . . 891
9.131.4.60 rndEdgesBegin() . . . 892
9.131.4.61 rndEdgesEnd() . . . 892

9.131.4.62 rndNodesBegin() 892
9.131.4.63 rndNodesEnd() 893
9.131.4.64 rndNonEdgesBegin() 893
9.131.4.65 rndNonEdgesEnd() 893
9.131.4.66 shuffle() 894
9.131.4.67 start() 894
9.131.4.68 write() 894
9.132 LinkPred::UPATPredictor< Network, EdgeRndIt, ScoreRndIt, EdgeRndOutIt > Class Template Reference 895
9.132.1 Detailed Description 896
9.132.2 Constructor & Destructor Documentation 896
9.132.2.1 UPATPredictor() [1/3] 896
9.132.2.2 UPATPredictor() [2/3] 896
9.132.2.3 UPATPredictor() [3/3] 897
9.132.2.4 ∼UPATPredictor() 897
9.132.3 Member Function Documentation 897
9.132.3.1 init() 897
9.132.3.2 learn() 898
9.132.3.3 operator=() [1/2] 898
9.132.3.4 operator=() [2/2] 898
9.132.3.5 predict() 899
9.132.3.6 score() 899
9.132.3.7 top() 899
9.133 LinkPred::UPSTPredictor< Network, EdgeRndIt, ScoreRndIt, EdgeRndOutIt > Class Template Reference 900
9.133.1 Detailed Description 901
9.133.2 Constructor & Destructor Documentation 902
9.133.2.1 UPSTPredictor() [1/3] 902
9.133.2.2 UPSTPredictor() [2/3] 902
9.133.2.3 UPSTPredictor() [3/3] 902
9.133.2.4 ∼UPSTPredictor() 903
9.133.3 Member Function Documentation 903
9.133.3.1 init() 903
9.133.3.2 learn() 903
9.133.3.3 loadEdgeScores() 903
9.133.3.4 operator=() [1/2] 904
9.133.3.5 operator=() [2/2] 904
9.133.3.6 score() 904
9.133.3.7 setEdgeScores() 905
9.134 LinkPred::URALPredictor< Network, EdgeRndIt, ScoreRndIt, EdgeRndOutIt > Class Template Reference 905
9.134.1 Detailed Description 906
9.134.2 Constructor & Destructor Documentation 907

9.134.2.1 URALPredictor() [1/3] 907
9.134.2.2 URALPredictor() [2/3] 907
9.134.2.3 URALPredictor() [3/3] 907
9.134.2.4 ∼URALPredictor() 908
9.134.3 Member Function Documentation 908
9.134.3.1 init() 908
9.134.3.2 learn() 908
9.134.3.3 operator=() [1/2] 909
9.134.3.4 operator=() [2/2] 909
9.134.3.5 predict() 909
9.134.3.6 score() 910
9.134.3.7 top() 910
9.135 LinkPred::URNDPredictor< Network, EdgeRndIt, ScoreRndIt, EdgeRndOutIt > Class Template Reference 911
9.135.1 Detailed Description 912
9.135.2 Constructor & Destructor Documentation 912
9.135.2.1 URNDPredictor() [1/3] 912
9.135.2.2 URNDPredictor() [2/3] 912
9.135.2.3 URNDPredictor() [3/3] 913
9.135.2.4 ∼URNDPredictor() 913
9.135.3 Member Function Documentation 913
9.135.3.1 init() 913
9.135.3.2 learn() 914
9.135.3.3 operator=() [1/2] 914
9.135.3.4 operator=() [2/2] 914
9.135.3.5 predict() 915
9.135.3.6 score() 915
9.136 LinkPred::USAIPredictor< Network, EdgeRndIt, ScoreRndIt, EdgeRndOutIt > Class Template Reference 915
9.136.1 Detailed Description 917
9.136.2 Constructor & Destructor Documentation 918
9.136.2.1 USAIPredictor() [1/3] 918
9.136.2.2 USAIPredictor() [2/3] 918
9.136.2.3 USAIPredictor() [3/3] 918
9.136.2.4 ∼USAIPredictor() 919
9.136.3 Member Function Documentation 919
9.136.3.1 init() 919
9.136.3.2 learn() 919
9.136.3.3 operator=() [1/2] 920
9.136.3.4 operator=() [2/2] 920
9.136.3.5 predict() 920
9.136.3.6 score() 921
9.136.3.7 top() 921

9.137 LinkPred::USBMPredictor< Network, EdgeRndIt, ScoreRndIt, EdgeRndOutIt > Class Template Reference 922
9.137.1 Detailed Description 923
9.137.2 Constructor & Destructor Documentation 923
9.137.2.1 USBMPredictor() [1/3] 923
9.137.2.2 USBMPredictor() [2/3] 923
9.137.2.3 USBMPredictor() [3/3] 924
9.137.2.4 ∼USBMPredictor() 924
9.137.3 Member Function Documentation 924
9.137.3.1 getMaxIter() 924
9.137.3.2 init() 925
9.137.3.3 learn() 925
9.137.3.4 operator=() [1/2] 925
9.137.3.5 operator=() [2/2] 925
9.137.3.6 predict() 926
9.137.3.7 score() 926
9.137.3.8 setMaxIter() 927
9.138 LinkPred::USHPPredictor< Network, EdgeRndIt, ScoreRndIt, EdgeRndOutIt > Class Template Reference 927
9.138.1 Detailed Description 928
9.138.2 Member Enumeration Documentation 929
9.138.2.1 LandmarkStrategy 929
9.138.3 Constructor & Destructor Documentation 929
9.138.3.1 USHPPredictor() [1/3] 929
9.138.3.2 USHPPredictor() [2/3] 930
9.138.3.3 USHPPredictor() [3/3] 930
9.138.3.4 ∼USHPPredictor() 930
9.138.4 Member Function Documentation 930
9.138.4.1 getAsp() 931
9.138.4.2 getCacheLevel() 931
9.138.4.3 getLandmarkRatio() 931
9.138.4.4 getLandmarkStrategy() 931
9.138.4.5 init() 932
9.138.4.6 learn() 932
9.138.4.7 operator=() [1/2] 932
9.138.4.8 operator=() [2/2] 932
9.138.4.9 predict() 933
9.138.4.10 score() 933
9.138.4.11 setAsp() 934
9.138.4.12 setCacheLevel() 934
9.138.4.13 setLandmarkRatio() 934
9.138.4.14 setLandmarkStrategy() 935

9.139 LinkPred::USOIPredictor< Network, EdgeRndIt, ScoreRndIt, EdgeRndOutIt > Class Template Reference . . . 935
9.139.1 Detailed Description . . . 936
9.139.2 Constructor & Destructor Documentation . . . 937
9.139.2.1 USOIPredictor() [1/3] . . . 937
9.139.2.2 USOIPredictor() [2/3] . . . 937
9.139.2.3 USOIPredictor() [3/3] . . . 937
9.139.2.4 ∼USOIPredictor() . . . 938
9.139.3 Member Function Documentation . . . 938
9.139.3.1 init() . . . 938
9.139.3.2 learn() . . . 938
9.139.3.3 operator=() [1/2] . . . 938
9.139.3.4 operator=() [2/2] . . . 939
9.139.3.5 predict() . . . 939
9.139.3.6 score() . . . 939
9.139.3.7 top() . . . 940
9.140 LinkPred::USUMPredictor< Network, EdgeRndIt, ScoreRndIt, EdgeRndOutIt > Class Template Reference . . . 941
9.140.1 Detailed Description . . . 942
9.140.2 Constructor & Destructor Documentation . . . 942
9.140.2.1 USUMPredictor() [1/3] . . . 942
9.140.2.2 USUMPredictor() [2/3] . . . 942
9.140.2.3 USUMPredictor() [3/3] . . . 943
9.140.2.4 ∼USUMPredictor() . . . 943
9.140.3 Member Function Documentation . . . 943
9.140.3.1 init() . . . 943
9.140.3.2 learn() . . . 944
9.140.3.3 operator=() [1/2] . . . 944
9.140.3.4 operator=() [2/2] . . . 944
9.140.3.5 predict() . . . 945
9.140.3.6 score() . . . 945
9.140.3.7 top() . . . 945
9.141 LinkPred::Vec Class Reference . . . 946
9.141.1 Detailed Description . . . 948
9.141.2 Constructor & Destructor Documentation . . . 948
9.141.2.1 Vec() [1/8] . . . 948
9.141.2.2 Vec() [2/8] . . . 948
9.141.2.3 Vec() [3/8] . . . 948
9.141.2.4 Vec() [4/8] . . . 949
9.141.2.5 Vec() [5/8] . . . 949
9.141.2.6 Vec() [6/8] . . . 949
9.141.2.7 Vec() [7/8] . . . 950
9.141.2.8 Vec() [8/8] . . . 950

9.141.2.9 ~Vec() 950
9.141.3 Member Function Documentation 950
9.141.3.1 amax() [1/2] 950
9.141.3.2 amax() [2/2] 951
9.141.3.3 amin() [1/2] 951
9.141.3.4 amin() [2/2] 951
9.141.3.5 axpy() 951
9.141.3.6 center() [1/2] 952
9.141.3.7 center() [2/2] 952
9.141.3.8 find() 952
9.141.3.9 findNz() 952
9.141.3.10 getValues() 953
9.141.3.11 ifmax() 953
9.141.3.12 ifmin() 953
9.141.3.13 ilmax() 953
9.141.3.14 ilmin() 953
9.141.3.15 imax() 954
9.141.3.16 imin() 954
9.141.3.17 isConstant() 954
9.141.3.18 makeConstant() 954
9.141.3.19 max() [1/2] 955
9.141.3.20 max() [2/2] 955
9.141.3.21 mean() [1/2] 955
9.141.3.22 mean() [2/2] 955
9.141.3.23 min() [1/2] 956
9.141.3.24 min() [2/2] 956
9.141.3.25 norm() [1/2] 956
9.141.3.26 norm() [2/2] 956
9.141.3.27 operator"!=() 957
9.141.3.28 operator=() [1/2] 957
9.141.3.29 operator=() [2/2] 957
9.141.3.30 operator==() 958
9.141.3.31 operator[]() [1/2] 958
9.141.3.32 operator[]() [2/2] 958
9.141.3.33 partScale() 959
9.141.3.34 print() [1/4] 959
9.141.3.35 print() [2/4] 959
9.141.3.36 print() [3/4] 959
9.141.3.37 print() [4/4] 960
9.141.3.38 read() [1/2] 960
9.141.3.39 read() [2/2] 960
9.141.3.40 reset() [1/2] 961

9.141.3.41 reset() [2/2] 961
9.141.3.42 resize() [1/2] 961
9.141.3.43 resize() [2/2] 961
9.141.3.44 scale() 962
9.141.3.45 scatter() 962
9.141.3.46 setConstant() 962
9.141.3.47 size() 962
9.141.3.48 sum() 963
9.141.3.49 write() [1/2] 963
9.141.3.50 write() [2/2] 963
9.141.4 Friends And Related Function Documentation 963
9.141.4.1 operator* [1/5] 963
9.141.4.2 operator* [2/5] 964
9.141.4.3 operator* [3/5] 964
9.141.4.4 operator* [4/5] 965
9.141.4.5 operator* [5/5] 965
9.141.4.6 operator+ [1/3] 965
9.141.4.7 operator+ [2/3] 966
9.141.4.8 operator+ [3/3] 966
9.141.4.9 operator- [1/3] 966
9.141.4.10 operator- [2/3] 967
9.141.4.11 operator- [3/3] 967
9.141.4.12 operator/ [1/3] 968
9.141.4.13 operator/ [2/3] 968
9.141.4.14 operator/ [3/3] 968
9.141.4.15 operator^ 969

10 File Documentation **971**
10.1 bindings/Java/include/LinkPredJavaConfig.hpp File Reference 971
10.1.1 Detailed Description 971
10.2 bindings/Python/include/LinkPredPythonConfig.hpp File Reference 971
10.2.1 Detailed Description 971
10.3 include/LinkPredConfig.hpp File Reference 972
10.3.1 Detailed Description 972
10.4 include/linkpred.hpp File Reference 972
10.4.1 Detailed Description 973
10.5 include/linkpred/core/core.hpp File Reference 973
10.5.1 Detailed Description 973
10.6 include/linkpred/core/dnetwork/dnetwork.hpp File Reference 974
10.6.1 Detailed Description 975
10.7 include/linkpred/core/ds/bheap.hpp File Reference 975
10.7.1 Detailed Description 976

10.8 include/linkpred/core/ds/bhmap.hpp File Reference 976
10.8.1 Detailed Description 977
10.9 include/linkpred/core/ds/ds.hpp File Reference 977
10.9.1 Detailed Description 978
10.10 include/linkpred/core/ds/lmapqueue.hpp File Reference 978
10.10.1 Detailed Description 979
10.11 include/linkpred/core/unetwork/unetwork.hpp File Reference 979
10.11.1 Detailed Description 980
10.12 include/linkpred/graphalg/encoders/deepwalk/deepwalk.hpp File Reference 981
10.12.1 Detailed Description 982
10.13 include/linkpred/graphalg/encoders/encoder.hpp File Reference 983
10.13.1 Detailed Description 983
10.14 include/linkpred/graphalg/encoders/encoders.hpp File Reference 984
10.14.1 Detailed Description 985
10.15 include/linkpred/graphalg/encoders/hmsm/hmsm.hpp File Reference 985
10.15.1 Detailed Description 986
10.16 include/linkpred/graphalg/encoders/largevis/largevis.hpp File Reference 987
10.16.1 Detailed Description 988
10.17 include/linkpred/graphalg/encoders/lem/lem.hpp File Reference 988
10.17.1 Detailed Description 989
10.18 include/linkpred/graphalg/encoders/line/line.hpp File Reference 989
10.18.1 Detailed Description 991
10.19 include/linkpred/graphalg/encoders/lle/lle.hpp File Reference 991
10.19.1 Detailed Description 992
10.20 include/linkpred/graphalg/encoders/matfact/matfact.hpp File Reference 992
10.20.1 Detailed Description 994
10.21 include/linkpred/graphalg/encoders/matfact/matfactcg.hpp File Reference 994
10.21.1 Detailed Description 995
10.22 include/linkpred/graphalg/encoders/node2vec/node2vec.hpp File Reference 996
10.22.1 Detailed Description 997
10.23 include/linkpred/graphalg/graphalg.hpp File Reference 997
10.23.1 Detailed Description 998
10.24 include/linkpred/graphalg/shortestpaths/dijkstra.hpp File Reference 998
10.24.1 Detailed Description 999
10.25 include/linkpred/graphalg/shortestpaths/netdistcalculator.hpp File Reference 999
10.25.1 Detailed Description 1001
10.26 include/linkpred/graphalg/shortestpaths/shortestpaths.hpp File Reference 1001
10.26.1 Detailed Description 1002
10.27 include/linkpred/graphalg/traversal/graphtraversal.hpp File Reference 1002
10.27.1 Detailed Description 1004
10.28 include/linkpred/graphalg/traversal/traversal.hpp File Reference 1004
10.28.1 Detailed Description 1004

10.29 include/linkpred/instantiations.hpp File Reference 1004
10.29.1 Detailed Description 1005
10.30 include/linkpred/ml/classifiers/classifier.hpp File Reference 1005
10.30.1 Detailed Description 1007
10.31 include/linkpred/ml/classifiers/classifiers.hpp File Reference 1007
10.31.1 Detailed Description 1008
10.32 include/linkpred/ml/classifiers/ffn/ffn.hpp File Reference 1008
10.32.1 Detailed Description 1010
10.33 include/linkpred/ml/classifiers/linearsvm/linearsvm.hpp File Reference 1010
10.33.1 Detailed Description 1012
10.34 include/linkpred/ml/classifiers/logistic/logisticregresser.hpp File Reference 1012
10.34.1 Detailed Description 1015
10.35 include/linkpred/ml/classifiers/logistic/logregcg.hpp File Reference 1015
10.35.1 Detailed Description 1015
10.36 include/linkpred/ml/classifiers/naivebayes/naivebayes.hpp File Reference 1016
10.36.1 Detailed Description 1017
10.37 include/linkpred/ml/classifiers/rndclassifier/rndclassifier.hpp File Reference 1017
10.37.1 Detailed Description 1020
10.38 include/linkpred/ml/ml.hpp File Reference 1020
10.38.1 Detailed Description 1020
10.39 include/linkpred/ml/simmeasures/cosinesim.hpp File Reference 1021
10.39.1 Detailed Description 1022
10.40 include/linkpred/ml/simmeasures/dotprod.hpp File Reference 1023
10.40.1 Detailed Description 1024
10.41 include/linkpred/ml/simmeasures/l1sim.hpp File Reference 1025
10.41.1 Detailed Description 1026
10.42 include/linkpred/ml/simmeasures/l2sim.hpp File Reference 1027
10.42.1 Detailed Description 1028
10.43 include/linkpred/ml/simmeasures/lpsim.hpp File Reference 1029
10.43.1 Detailed Description 1030
10.44 include/linkpred/ml/simmeasures/pearson.hpp File Reference 1031
10.44.1 Detailed Description 1032
10.45 include/linkpred/ml/simmeasures/simmeasure.hpp File Reference 1033
10.45.1 Detailed Description 1034
10.46 include/linkpred/ml/simmeasures/simmeasures.hpp File Reference 1034
10.46.1 Detailed Description 1035
10.47 include/linkpred/numerical/linear/gfmatrix.hpp File Reference 1035
10.47.1 Detailed Description 1037
10.48 include/linkpred/numerical/linear/vec.hpp File Reference 1037
10.48.1 Detailed Description 1038
10.49 include/linkpred/numerical/mds/logmdscg.hpp File Reference 1038
10.49.1 Detailed Description 1039

10.50 include/linkpred/numerical/mds/mds.hpp File Reference 1039
10.50.1 Detailed Description 1041
10.51 include/linkpred/numerical/mf/fastsig.hpp File Reference 1041
10.51.1 Detailed Description 1042
10.52 include/linkpred/numerical/numerical.hpp File Reference 1043
10.52.1 Detailed Description 1043
10.53 include/linkpred/perf/networkmanipulator.hpp File Reference 1043
10.53.1 Detailed Description 1046
10.54 include/linkpred/perf/perf.hpp File Reference 1046
10.54.1 Detailed Description 1047
10.55 include/linkpred/perf/perfevaluator.hpp File Reference 1047
10.55.1 Detailed Description 1049
10.56 include/linkpred/perf/perfmeasure.hpp File Reference 1049
10.56.1 Detailed Description 1051
10.56.2 Typedef Documentation 1051
10.56.2.1 PerfLambdaT 1051
10.56.3 Variable Documentation 1051
10.56.3.1 acc 1051
10.56.3.2 fdr 1052
10.56.3.3 fmr 1052
10.56.3.4 fnr 1053
10.56.3.5 fpr 1053
10.56.3.6 npv 1053
10.56.3.7 pre 1054
10.56.3.8 rec 1054
10.56.3.9 tnr 1055
10.57 include/linkpred/perf/predresults.hpp File Reference 1055
10.57.1 Detailed Description 1057
10.58 include/linkpred/predictors/directed/dadapredictor.hpp File Reference 1057
10.58.1 Detailed Description 1058
10.59 include/linkpred/predictors/directed/dcnepredictor.hpp File Reference 1059
10.59.1 Detailed Description 1060
10.60 include/linkpred/predictors/directed/dhdipredictor.hpp File Reference 1060
10.60.1 Detailed Description 1061
10.61 include/linkpred/predictors/directed/dhpipredictor.hpp File Reference 1062
10.61.1 Detailed Description 1063
10.62 include/linkpred/predictors/directed/directed.hpp File Reference 1063
10.62.1 Detailed Description 1064
10.63 include/linkpred/predictors/directed/djidpredictor.hpp File Reference 1064
10.63.1 Detailed Description 1065
10.64 include/linkpred/predictors/directed/dlcppredictor.hpp File Reference 1066
10.64.1 Detailed Description 1067

10.65 include/linkpred/predictors/directed/dlhnpredictor.hpp File Reference 1067
10.65.1 Detailed Description 1068
10.66 include/linkpred/predictors/directed/dlpredictor.hpp File Reference 1069
10.66.1 Detailed Description 1069
10.67 include/linkpred/predictors/directed/dpatpredictor.hpp File Reference 1070
10.67.1 Detailed Description 1071
10.68 include/linkpred/predictors/directed/dpstpredictor.hpp File Reference 1071
10.68.1 Detailed Description 1072
10.69 include/linkpred/predictors/directed/dsaipredictor.hpp File Reference 1073
10.69.1 Detailed Description 1074
10.70 include/linkpred/predictors/directed/dsoipredictor.hpp File Reference 1074
10.70.1 Detailed Description 1075
10.71 include/linkpred/predictors/predictors.hpp File Reference 1076
10.71.1 Detailed Description 1076
10.72 include/linkpred/predictors/undirected/uadapredictor.hpp File Reference 1076
10.72.1 Detailed Description 1078
10.73 include/linkpred/predictors/undirected/ucnepredictor.hpp File Reference 1078
10.73.1 Detailed Description 1079
10.74 include/linkpred/predictors/undirected/ucrapredictor.hpp File Reference 1080
10.74.1 Detailed Description 1081
10.75 include/linkpred/predictors/undirected/ucstpredictor.hpp File Reference 1081
10.75.1 Detailed Description 1082
10.76 include/linkpred/predictors/undirected/ueclpredictor.hpp File Reference 1083
10.76.1 Detailed Description 1084
10.77 include/linkpred/predictors/undirected/uesmpredictor.hpp File Reference 1084
10.77.1 Detailed Description 1085
10.78 include/linkpred/predictors/undirected/ufbmpredictor.hpp File Reference 1086
10.78.1 Detailed Description 1087
10.79 include/linkpred/predictors/undirected/uhdipredictor.hpp File Reference 1087
10.79.1 Detailed Description 1088
10.80 include/linkpred/predictors/undirected/uhpipredictor.hpp File Reference 1089
10.80.1 Detailed Description 1090
10.81 include/linkpred/predictors/undirected/uhrgpredictor.hpp File Reference 1090
10.81.1 Detailed Description 1091
10.82 include/linkpred/predictors/undirected/uhyppredictor.hpp File Reference 1092
10.82.1 Detailed Description 1094
10.83 include/linkpred/predictors/undirected/ujidpredictor.hpp File Reference 1094
10.83.1 Detailed Description 1095
10.84 include/linkpred/predictors/undirected/ukabpredictor.hpp File Reference 1096
10.84.1 Detailed Description 1097
10.85 include/linkpred/predictors/undirected/ulcppredictor.hpp File Reference 1098
10.85.1 Detailed Description 1099

10.86 include/linkpred/predictors/undirected/ulhnpredictor.hpp File Reference 1099
10.86.1 Detailed Description 1100
10.87 include/linkpred/predictors/undirected/ulpredictor.hpp File Reference 1101
10.87.1 Detailed Description 1101
10.88 include/linkpred/predictors/undirected/umpspredictor.hpp File Reference 1102
10.88.1 Detailed Description 1102
10.89 include/linkpred/predictors/undirected/undirected.hpp File Reference 1102
10.89.1 Detailed Description 1104
10.90 include/linkpred/predictors/undirected/unedpredictor.hpp File Reference 1104
10.90.1 Detailed Description 1106
10.91 include/linkpred/predictors/undirected/upatpredictor.hpp File Reference 1106
10.91.1 Detailed Description 1107
10.92 include/linkpred/predictors/undirected/upstpredictor.hpp File Reference 1108
10.92.1 Detailed Description 1109
10.93 include/linkpred/predictors/undirected/uralpredictor.hpp File Reference 1109
10.93.1 Detailed Description 1110
10.94 include/linkpred/predictors/undirected/urndpredictor.hpp File Reference 1111
10.94.1 Detailed Description 1112
10.95 include/linkpred/predictors/undirected/usaipredictor.hpp File Reference 1113
10.95.1 Detailed Description 1114
10.96 include/linkpred/predictors/undirected/usbmpredictor.hpp File Reference 1114
10.96.1 Detailed Description 1115
10.97 include/linkpred/predictors/undirected/ushppredictor.hpp File Reference 1116
10.97.1 Detailed Description 1117
10.98 include/linkpred/predictors/undirected/usoipredictor.hpp File Reference 1118
10.98.1 Detailed Description 1119
10.99 include/linkpred/predictors/undirected/usumpredictor.hpp File Reference 1119
10.99.1 Detailed Description 1120
10.100 include/linkpred/simp/edgescore.hpp File Reference 1121
10.100.1 Detailed Description 1122
10.101 include/linkpred/simp/evaluator.hpp File Reference 1122
10.101.1 Detailed Description 1123
10.102 include/linkpred/simp/perfres.hpp File Reference 1123
10.102.1 Detailed Description 1125
10.103 include/linkpred/simp/predictor.hpp File Reference 1125
10.103.1 Detailed Description 1126
10.104 include/linkpred/simp/simp.hpp File Reference 1126
10.104.1 Detailed Description 1127
10.105 include/linkpred/utils/log.hpp File Reference 1127
10.105.1 Detailed Description 1128
10.105.2 Macro Definition Documentation 1128
10.105.2.1 logger 1128

10.106 include/linkpred/utils/loglevel.hpp File Reference 1128
10.106.1 Detailed Description 1129
10.107 include/linkpred/utils/miscutils.hpp File Reference 1129
10.107.1 Detailed Description 1132
10.108 include/linkpred/utils/randomgen.hpp File Reference 1132
10.108.1 Detailed Description 1132
10.109 include/linkpred/utils/utils.hpp File Reference 1133
10.109.1 Detailed Description 1133

Index **1135**

Chapter 1

LinkPred: A high performance library for link prediction in complex networks

This is LinkPred, a high performance library for link prediction in complex networks.

LinkPred provides the following functionalities:

Basic data structures to efficiently store and access network data.

Basic graph algorithms such graph traversal, shortest path algorithms, and graph embedding methods.

Implementation of several topological similarity index predictors, for example: common neighbors, Adamic-Adard index and Jackard index among other predictors (a full list is available in the library documentation).

Implementation of several state-of-the-art global link predictors (a full list is available in the library documentation).

Implementation of several link prediction algorithms based on graph embedding techniques.

Test data generation from ground truth networks.

Performance evaluation functionalities.

Chapter 2

Module Index

2.1 Modules

Here is a list of all modules:

Core classes 23
Graph algorithms. 24
Machine learning classes 25
Numerical classes and algorithms 26
Performance evaluation classes 27
Link prediction algorithms 28
Simplified interface 30
Utility functions and classes 31

Chapter 3

Namespace Index

3.1 Namespace List

Here is a list of all documented namespaces with brief descriptions:

LinkPred
Main namespace 33
LinkPred::Simp
Simplified interface. Contains a simplified interface for LinkPred that includes the essential functionalities 47
LinkPred::Utils
Some utility functions 47

Chapter 4

Hierarchical Index

4.1 Class Hierarchy

This inheritance list is sorted roughly, but not completely, alphabetically:

- LinkPred::BHeap< T, P, ComparatorT, ComparatorP > 76
- LinkPred::Bhmap< K, P, Comparator > 83
- LinkPred::Bhmap< Label, NodeID > 83
- CGDProblem
  - LinkPred::LogMDSCG 401
  - LinkPred::LogRegCG< InRndIt, OutRndIt > 406
  - LinkPred::MatFactCG 423
- LinkPred::Classifier< InRndIt, OutRndIt, ScoreRndIt > 89
- LinkPred::Classifier< typename std::vector< Vec >::iterator, typename std::vector< bool >::iterator, typename std::vector< double >::iterator > 89
  - LinkPred::LogisticRegresser< InRndIt, OutRndIt, ScoreRndIt > 396
  - LinkPred::RndClassifier< InRndIt, OutRndIt, ScoreRndIt > 632
- LinkPred::Collector< Network > 93
- LinkPred::Counter< Network > 98
- LinkPred::Dijkstra< Network, DistType, NbHopsType > 145
- LinkPred::Dijkstra< DNetwork<>, double, std::size_t > 145
- LinkPred::Dijkstra< UNetwork<>, double, std::size_t > 145
- LinkPred::DLPredictor< NetworkT, EdgeRndItT, ScoreRndItT, EdgeRndOutItT > 174
- LinkPred::DLPredictor< DNetwork<>, typename std::vector< typename DNetwork<> ::Edge >::const_iterator, typename std::vector< double >::iterator, typename std::vector< typename DNetwork<> ::Edge >::iterator > 174
  - LinkPred::DADAPredictor< Network, EdgeRndIt, ScoreRndIt, EdgeRndOutIt > 102
  - LinkPred::DCNEPredictor< Network, EdgeRndIt, ScoreRndIt, EdgeRndOutIt > 107
  - LinkPred::DHDIPredictor< Network, EdgeRndIt, ScoreRndIt, EdgeRndOutIt > 134
  - LinkPred::DHPIPredictor< Network, EdgeRndIt, ScoreRndIt, EdgeRndOutIt > 139
  - LinkPred::DJIDPredictor< Network, EdgeRndIt, ScoreRndIt, EdgeRndOutIt > 158
  - LinkPred::DLCPPredictor< Network, EdgeRndIt, ScoreRndIt, EdgeRndOutIt > 164
  - LinkPred::DLHNPredictor< Network, EdgeRndIt, ScoreRndIt, EdgeRndOutIt > 169
  - LinkPred::DPATPredictor< Network, EdgeRndIt, ScoreRndIt, EdgeRndOutIt > 215
  - LinkPred::DPSTPredictor< Network, EdgeRndIt, ScoreRndIt, EdgeRndOutIt > 220
  - LinkPred::DSAIPredictor< Network, EdgeRndIt, ScoreRndIt, EdgeRndOutIt > 226
  - LinkPred::DSOIPredictor< Network, EdgeRndIt, ScoreRndIt, EdgeRndOutIt > 231
- LinkPred::DNetwork< LabelT, NodeIDT, EdgeT > 182
- LinkPred::DNetwork<> 182
- LinkPred::Simp::Evaluator::Factory::ECLParams 237

- LinkPred::UNetwork< LabelT, NodeIDT, EdgeT >::EdgeMap< ValueType > . . . 238
- LinkPred::DNetwork< LabelT, NodeIDT, EdgeT >::EdgeMap< ValueType > . . . 244
- LinkPred::Utils::EdgeScore< Label > . . . 251
- LinkPred::Simp::EdgeScore . . . 252
- LinkPred::Simp::EdgeScoreByID . . . 253
- LinkPred::Encoder< Network > . . . 254
- LinkPred::Encoder< UNetwork<> > . . . 254
  - LinkPred::DeepWalk< Network > . . . 113
  - LinkPred::HMSM< Network > . . . 351
  - LinkPred::LargeVis< Network > . . . 367
  - LinkPred::LINE< Network > . . . 377
  - LinkPred::MatFact< Network > . . . 416
  - LinkPred::Node2Vec< Network > . . . 464
- LinkPred::Simp::Evaluator::Factory::ESMParams . . . 261
- LinkPred::Simp::Evaluator . . . 310
- LinkPred::FastSig< T > . . . 321
- LinkPred::FastSig< float > . . . 321
- LinkPred::Simp::Evaluator::Factory::FBMParams . . . 324
- LinkPred::GFMatrix . . . 332
- LinkPred::GraphTraversal< Network, NodeProcessor > . . . 348
- LinkPred::GraphTraversal< UNetwork<>, Collector< UNetwork<> > > . . . 348
  - LinkPred::BFS< Network, NodeProcessor > . . . 73
  - LinkPred::DFS< Network, NodeProcessor > . . . 130
- LinkPred::Simp::Evaluator::Factory::HRGParams . . . 358
- LinkPred::Simp::Evaluator::Factory::HYPParams . . . 359
- iterator
  - LinkPred::DNetwork< LabelT, NodeIDT, EdgeT >::NodeDegIt . . . 474
  - LinkPred::DNetwork< LabelT, NodeIDT, EdgeT >::NonEdgeIt . . . 526
  - LinkPred::DNetwork< LabelT, NodeIDT, EdgeT >::RndEdgeIt . . . 641
  - LinkPred::DNetwork< LabelT, NodeIDT, EdgeT >::RndNodeIt . . . 646
  - LinkPred::DNetwork< LabelT, NodeIDT, EdgeT >::RndNonEdgeIt . . . 661
  - LinkPred::PredResults< TestDataT, LPredictorT, ScoresContainerT >::ScoreIterator . . . 678
  - LinkPred::TestData< Network, EdgeContT >::TestEdgeIt . . . 709
  - LinkPred::TestEdgeGen< Network, EdgeContT >::TNEdgeIt . . . 719
  - LinkPred::TestEdgeGen< Network, EdgeContT >::TPEdgeIt . . . 729
  - LinkPred::UNetwork< LabelT, NodeIDT, EdgeT >::NodeDegIt . . . 484
  - LinkPred::UNetwork< LabelT, NodeIDT, EdgeT >::NonEdgeIt . . . 517
  - LinkPred::UNetwork< LabelT, NodeIDT, EdgeT >::RndEdgeIt . . . 636
  - LinkPred::UNetwork< LabelT, NodeIDT, EdgeT >::RndNodeIt . . . 651
  - LinkPred::UNetwork< LabelT, NodeIDT, EdgeT >::RndNonEdgeIt . . . 656
- LinkPred::Simp::Evaluator::Factory::KABParams . . . 360
- LinkPred::Simp::Evaluator::Factory::LCPParams . . . 376
- LinkPred::LMapQueue< K, P, KComparator, PComparator > . . . 384
- LinkPred::Log . . . 393
- LinkPred::MatFactPbData . . . 427
- LinkPred::MDS . . . 428
- LinkPred::NetDistCalculator< Network, DistType, NbHopsType > . . . 433
- LinkPred::NetDistCalculator< DNetwork<>, double, std::size_t > . . . 433
  - LinkPred::DESPLDistCalculator< Network, DistType, NbHopsType > . . . 121
- LinkPred::NetDistCalculator< UNetwork<>, double, std::size_t > . . . 433
  - LinkPred::ESPLDistCalculator< UNetwork<>, double, std::size_t > . . . 284
  - LinkPred::ASPDistCalculator< Network, DistType, NbHopsType > . . . 59
  - LinkPred::ASPDsimCalculator< Network, DsimType, NbHopsType > . . . 66
  - LinkPred::ESPDistCalculator< Network, DistType, NbHopsType > . . . 262
  - LinkPred::ESPDsimCalculator< Network, DsimType, NbHopsType > . . . 270
  - LinkPred::ESPLDistCalculator< Network, DistType, NbHopsType > . . . 284
- LinkPred::NetIndSimlCalculator< Network, DistType, NbHopsType > . . . 438

- LinkPred::NetIndSimlCalculator< UNetwork<>, double, std::size_t > . . . 438
  - LinkPred::ESPIndSimlCalculator< Network, DistType, NbHopsType > . . . 277
- LinkPred::NetSimlCalculator< Network, DistType, NbHopsType > . . . 443
- LinkPred::NetSimlCalculator< UNetwork<>, double, std::size_t > . . . 443
  - LinkPred::ESPLSimlCalculator< Network, DistType, NbHopsType > . . . 293
  - LinkPred::ESPSimlCalculator< Network, DistType, NbHopsType > . . . 302
- LinkPred::NetworkManipulator< Network > . . . 450
- LinkPred::UNetwork< LabelT, NodeIDT, EdgeT >::NodeMap< ValueType > . . . 494
- LinkPred::DNetwork< LabelT, NodeIDT, EdgeT >::NodeMap< ValueType > . . . 499
- LinkPred::DNetwork< LabelT, NodeIDT, EdgeT >::NodeSMap< ValueType > . . . 505
- LinkPred::UNetwork< LabelT, NodeIDT, EdgeT >::NodeSMap< ValueType > . . . 510
- LinkPred::Utils::PairCompRight< FirstT, SecondT, CompareT > . . . 536
- LinkPred::PEFactory< Network, LPredictorT, TestDataT, PerfMeasureT > . . . 540
- LinkPred::PEFactory<> . . . 540
- LinkPred::PerfeEvalExpDescp< Network > . . . 547
- LinkPred::PerfeEvalExpDescp< UNetwork<> > . . . 547
- LinkPred::PerfEvalExp< Network, TestDataT, LPredictorT, PredResultsT, PerfMeasureT > . . . 549
- LinkPred::PerfEvaluator< TestDataT, LPredictorT, PredResultsT, PerfMeasureT > . . . 555
- LinkPred::PerfMeasure< PredResultsT > . . . 562
- LinkPred::PerfMeasure< PredResults<> > . . . 562
  - LinkPred::PerfCurve< PredResultsT > . . . 541
  - LinkPred::TPR< PredResultsT > . . . 738
  - LinkPred::PerfCurve< PredResults<> > . . . 541
    - LinkPred::GCurve< PredResultsT > . . . 325
    - LinkPred::PR< PredResultsT > . . . 569
    - LinkPred::ROC< PredResultsT > . . . 667
- LinkPred::Simp::PerfRes . . . 568
- LinkPred::Simp::Predictor . . . 578
- LinkPred::PredResults< TestDataT, LPredictorT, ScoresContainerT > . . . 614
- LinkPred::Simp::Evaluator::Factory::PSTParams . . . 626
- LinkPred::RandomGen . . . 627
- LinkPred::Simp::Evaluator::Factory::RNDParams . . . 666
- LinkPred::Simp::Evaluator::Factory::SBMParams . . . 677
- LinkPred::Simp::Evaluator::Factory::SHPParams . . . 689
- LinkPred::SimMeasure . . . 690
  - LinkPred::CosineSim . . . 95
  - LinkPred::DotProd . . . 212
  - LinkPred::L1Sim . . . 361
  - LinkPred::L2Sim . . . 364
  - LinkPred::LPSim . . . 412
  - LinkPred::Pearson . . . 537
- LinkPred::TestData< Network, EdgeContT > . . . 693
- LinkPred::TestData<> . . . 693
- LinkPred::TestEdgeGen< Network, EdgeContT > . . . 704
- LinkPred::ULPredictor< NetworkT, EdgeRndItT, ScoreRndItT, EdgeRndOutItT > . . . 835
- LinkPred::ULPredictor< Network, EdgeRndIt, ScoreRndIt, EdgeRndOutIt > . . . 835
- LinkPred::ULPredictor< UNetwork<>, typename std::vector< typename UNetwork<> ::Edge >↩ ::const_iterator, typename std::vector< double >::iterator, typename std::vector< typename U↩ Network<> ::Edge >::iterator > . . . 835
  - LinkPred::UADAPredictor< Network, EdgeRndIt, ScoreRndIt, EdgeRndOutIt > . . . 745
  - LinkPred::UCNEPredictor< Network, EdgeRndIt, ScoreRndIt, EdgeRndOutIt > . . . 751
  - LinkPred::UCRAPredictor< Network, EdgeRndIt, ScoreRndIt, EdgeRndOutIt > . . . 756
  - LinkPred::UCSTPredictor< Network, EdgeRndIt, ScoreRndIt, EdgeRndOutIt > . . . 762
  - LinkPred::UECLPredictor< Network, EdgeRndIt, ScoreRndIt, EdgeRndOutIt > . . . 768
  - LinkPred::UESMPredictor< Network, EdgeRndIt, ScoreRndIt, EdgeRndOutIt > . . . 775
  - LinkPred::UFBMPredictor< Network, EdgeRndIt, ScoreRndIt, EdgeRndOutIt > . . . 781
  - LinkPred::UHDIPredictor< Network, EdgeRndIt, ScoreRndIt, EdgeRndOutIt > . . . 786

LinkPred::UHPIPredictor< Network, EdgeRndIt, ScoreRndIt, EdgeRndOutIt > 792
LinkPred::UHRGPredictor< Network, EdgeRndIt, ScoreRndIt, EdgeRndOutIt > 797
LinkPred::UHYPPredictor< Network, EdgeRndIt, ScoreRndIt, EdgeRndOutIt > 803
LinkPred::UJIDPredictor< Network, EdgeRndIt, ScoreRndIt, EdgeRndOutIt > 811
LinkPred::UKABPredictor< Network, EdgeRndIt, ScoreRndIt, EdgeRndOutIt > 817
LinkPred::ULCPPredictor< Network, EdgeRndIt, ScoreRndIt, EdgeRndOutIt > 824
LinkPred::ULHNPredictor< Network, EdgeRndIt, ScoreRndIt, EdgeRndOutIt > 829
LinkPred::UMPSPredictor< Network, EdgeRndIt, ScoreRndIt, EdgeRndOutIt > 843
LinkPred::UNEDPredictor< Network, EdgeRndIt, ScoreRndIt, EdgeRndOutIt > 861
LinkPred::UPATPredictor< Network, EdgeRndIt, ScoreRndIt, EdgeRndOutIt > 895
LinkPred::UPSTPredictor< Network, EdgeRndIt, ScoreRndIt, EdgeRndOutIt > 900
LinkPred::URALPredictor< Network, EdgeRndIt, ScoreRndIt, EdgeRndOutIt > 905
LinkPred::URNDPredictor< Network, EdgeRndIt, ScoreRndIt, EdgeRndOutIt > 911
LinkPred::USAIPredictor< Network, EdgeRndIt, ScoreRndIt, EdgeRndOutIt > 915
LinkPred::USBMPredictor< Network, EdgeRndIt, ScoreRndIt, EdgeRndOutIt > 922
LinkPred::USHPPredictor< Network, EdgeRndIt, ScoreRndIt, EdgeRndOutIt > 927
LinkPred::USOIPredictor< Network, EdgeRndIt, ScoreRndIt, EdgeRndOutIt > 935
LinkPred::USUMPredictor< Network, EdgeRndIt, ScoreRndIt, EdgeRndOutIt > 941
LinkPred::UNetwork< LabelT, NodeIDT, EdgeT > 867
LinkPred::UNetwork<> 867
LinkPred::Vec 946

Chapter 5

Class Index

5.1 Class List

Here are the classes, structs, unions and interfaces with brief descriptions:

LinkPred::ASPDistCalculator< Network, DistType, NbHopsType >
Approximate shortest path distance calculator 59
LinkPred::ASPDsimCalculator< Network, DsimType, NbHopsType >
Approximate shortest path dissimilarity calculator 66
LinkPred::BFS< Network, NodeProcessor >
BFS graph traversal 73
LinkPred::BHeap< T, P, ComparatorT, ComparatorP >
A binary heap 76
LinkPred::Bhmap< K, P, Comparator >
A bidirectional half map 83
LinkPred::Classifier< InRndIt, OutRndIt, ScoreRndIt >
Interface of a binary classifier 89
LinkPred::Collector< Network >
A class that collects nodes during traversal 93
LinkPred::CosineSim
Cosine similarity 95
LinkPred::Counter< Network >
A class that counts nodes during traversal 98
LinkPred::DADAPredictor< Network, EdgeRndIt, ScoreRndIt, EdgeRndOutIt >
Common neighbor link predictor adapted to directed networks 102
LinkPred::DCNEPredictor< Network, EdgeRndIt, ScoreRndIt, EdgeRndOutIt >
Common neighbor link predictor adapted to directed networks 107
LinkPred::DeepWalk< Network >
DeepWalk encoder. Reference: Perozzi, B., Al-Rfou, R., and Skiena, S. (2014). Deepwalk: Online learning of social representations. In Proceedings of the 20th ACM SIGKDD International Conference on Knowledge Discovery and Data Mining, KDD '14, pages 701–710, New York, NY, USA. Association for Computing Machinery. This implementation is based on the code `https://github.com/xgfs/deepwalk-c` 113
LinkPred::DESPLDistCalculator< Network, DistType, NbHopsType >
Exact shortest path distance calculator on a directed network with limits on the number of hops 121
LinkPred::DFS< Network, NodeProcessor >
DFS graph traversal 130
LinkPred::DHDIPredictor< Network, EdgeRndIt, ScoreRndIt, EdgeRndOutIt >
Common neighbor link predictor adapted to directed networks 134
LinkPred::DHPIPredictor< Network, EdgeRndIt, ScoreRndIt, EdgeRndOutIt >
Common neighbor link predictor adapted to directed networks 139

LinkPred::Dijkstra< Network, DistType, NbHopsType >
An implementation of Dijkstra's algorithm 145
LinkPred::DJIDPredictor< Network, EdgeRndIt, ScoreRndIt, EdgeRndOutIt >
Common neighbor link predictor adapted to directed networks 158
LinkPred::DLCPPredictor< Network, EdgeRndIt, ScoreRndIt, EdgeRndOutIt >
Common neighbor link predictor adapted to directed networks 164
LinkPred::DLHNPredictor< Network, EdgeRndIt, ScoreRndIt, EdgeRndOutIt >
Common neighbor link predictor adapted to directed networks 169
LinkPred::DLPredictor< NetworkT, EdgeRndItT, ScoreRndItT, EdgeRndOutItT >
The interface of a link predictor in a directed network 174
LinkPred::DNetwork< LabelT, NodeIDT, EdgeT >
This class represents a directed network in the sense of graph theory 182
LinkPred::DotProd
A simple dot product similarity measure 212
LinkPred::DPATPredictor< Network, EdgeRndIt, ScoreRndIt, EdgeRndOutIt >
Common neighbor link predictor adapted to directed networks 215
LinkPred::DPSTPredictor< Network, EdgeRndIt, ScoreRndIt, EdgeRndOutIt >
A link predictor that prestores edge scores. This allows to seemingly integrate results from external link prediction algorithms to LinkPred (for example, users may implement their own link prediction algorithm and then use this link predictor to use compare their results to algorithms available in LinkPred) 220
LinkPred::DSAIPredictor< Network, EdgeRndIt, ScoreRndIt, EdgeRndOutIt >
Common neighbor link predictor adapted to directed networks 226
LinkPred::DSOIPredictor< Network, EdgeRndIt, ScoreRndIt, EdgeRndOutIt >
Common neighbor link predictor adapted to directed networks 231
LinkPred::Simp::Evaluator::Factory::ECLParams
Parameters of ECL 237
LinkPred::UNetwork< LabelT, NodeIDT, EdgeT >::EdgeMap< ValueType >
An edge map 238
LinkPred::DNetwork< LabelT, NodeIDT, EdgeT >::EdgeMap< ValueType >
An edge map 244
LinkPred::Utils::EdgeScore< Label >
A structure to store the score of an edge 251
LinkPred::Simp::EdgeScore
A structure to store the score of an edge 252
LinkPred::Simp::EdgeScoreByID
A structure to store the score of an edge. The node IDs are used instead of labels 253
LinkPred::Encoder< Network >
The interface of a network encoder 254
LinkPred::Simp::Evaluator::Factory::ESMParams
Parameters of ESM 261
LinkPred::ESPDistCalculator< Network, DistType, NbHopsType >
Exact shortest path distance calculator 262
LinkPred::ESPDsimCalculator< Network, DsimType, NbHopsType >
Exact shortest path dissimilarity calculator 270
LinkPred::ESPIndSimlCalculator< Network, DistType, NbHopsType >
Exact shortest path distance calculator 277
LinkPred::ESPLDistCalculator< Network, DistType, NbHopsType >
Exact shortest path distance calculator with limits on the number of hops 284
LinkPred::ESPLSimlCalculator< Network, DistType, NbHopsType >
Exact shortest path distance calculator 293
LinkPred::ESPSimlCalculator< Network, DistType, NbHopsType >
Exact shortest path distance calculator 302
LinkPred::Simp::Evaluator
A class that simplifies the evaluation of link prediction algorithms 310
LinkPred::FastSig< T >
A fast sigmoid 321

LinkPred::Simp::Evaluator::Factory::FBMParams
Parameters of FBM 324
LinkPred::GCurve< PredResultsT >
General performance curve 325
LinkPred::GFMatrix
Generalized full matrix. The storage scheme used is column-major 332
LinkPred::GraphTraversal< Network, NodeProcessor >
Graph traversal interface 348
LinkPred::HMSM< Network >
Contains the implementation of an algorithm for embedding a network using a a hidden metric space model. Reference: R. Alharbi, H. Benhidour, and S. Kerrache. “Link Prediction in Complex Net-works Based on a Hidden Variables Model”. In: 2016 UKSim-AMSS 18th Inter-national Conference on Computer Modelling and Simulation (UKSim). 2016,pages 119–124 351
LinkPred::Simp::Evaluator::Factory::HRGParams
Parameters of HRG 358
LinkPred::Simp::Evaluator::Factory::HYPParams
Parameters of HYP 359
LinkPred::Simp::Evaluator::Factory::KABParams
Parameters of KAB 360
LinkPred::L1Sim
L1 similarity (negative the L1 norm or Manhattan distance) 361
LinkPred::L2Sim
L2 similarity (negative the Euclidean distance) 364
LinkPred::LargeVis< Network >
LargeVis encoder. Reference: Tang, J., Liu, J., Zhang, M., and Mei, Q. (2016b). Visualizing large-scale and high-dimensional data. In Bourdeau, J., Hendler, J., Nkambou, R., Horrocks, I., and Zhao, B. Y., editors, WWW, pages 287–297. ACM. This implementation is based on the code `https://github.com/lferry007/LargeVis` 367
LinkPred::Simp::Evaluator::Factory::LCPParams
Parameters of LCP 376
LinkPred::LINE< Network >
LINE encoder 377
LinkPred::LMapQueue< K, P, KComparator, PComparator >
A map-priority queue with limit on the capacity 384
LinkPred::Log
A log class 393
LinkPred::LogisticRegresser< InRndIt, OutRndIt, ScoreRndIt >
Logistic regression algorithm 396
LinkPred::LogMDSCG
Optimization problem associated with multidimensional scaling using the logarithmic (MULTIS↩CALE) loss function 401
LinkPred::LogRegCG< InRndIt, OutRndIt >
Logistic regression optimization problem 406
LinkPred::LPSim
LP similarity (negative the Lp norm) 412
LinkPred::MatFact< Network >
Contains the implementation of an algorithm for embedding a network using matrix factoriza-tion. Reference: Koren, Y., Bell, R., and Volinsky, C. (2009). Matrix factorization techniques for recommender systems. Computer, 42(8):30–37 Ahmed, A., Shervashidze, N., Narayanamurthy, S., Josifovski, V., and Smola, A. J. (2013). Distributed large-scale natural graph factorization. In Proceedings of the 22nd International Conference on World Wide Web, WWW ’13, pages 37–48, New York, NY, USA. Association for Computing Machinery 416
LinkPred::MatFactCG
Optimization problem associated with matrix factorization 423
LinkPred::MatFactPbData
A simple structure to store matrix factoization problem data 427
LinkPred::MDS
Solve the MDS problem 428

LinkPred::NetDistCalculator< Network, DistType, NbHopsType >
Interface for calculating the distance between nodes in a network 433
LinkPred::NetIndSimlCalculator< Network, DistType, NbHopsType >
Interface for calculating the indirect similarity between nodes in a network 438
LinkPred::NetSimlCalculator< Network, DistType, NbHopsType >
Interface for calculating the similarity between nodes in a network 443
LinkPred::NetworkManipulator< Network >
Class to manipulate network by removing or adding edges 450
LinkPred::Node2Vec< Network >
Node2Vec encoder. References: Grover, A. and Leskovec, J. (2016). Node2vec: Scalable feature learning for networks. In Proceedings of the 22nd ACM SIGKDD International Conference on Knowledge Discovery and Data Mining, KDD'16, pages 855–864, New York, NY, USA. Association for Computing Machinery. This implementation is based on the code https↩://github.com/xgfs/node2vec-c 464
LinkPred::DNetwork< LabelT, NodeIDT, EdgeT >::NodeDegIt
Node-degree iterator. This class can be used to iterate over pairs of node IDs and in and out degrees 474
LinkPred::UNetwork< LabelT, NodeIDT, EdgeT >::NodeDegIt
Node-degree iterator. This class can be used to iterate over pairs of node IDs and degrees . . 484
LinkPred::UNetwork< LabelT, NodeIDT, EdgeT >::NodeMap< ValueType >
A node map 494
LinkPred::DNetwork< LabelT, NodeIDT, EdgeT >::NodeMap< ValueType >
A node map 499
LinkPred::DNetwork< LabelT, NodeIDT, EdgeT >::NodeSMap< ValueType >
A sparse node map 505
LinkPred::UNetwork< LabelT, NodeIDT, EdgeT >::NodeSMap< ValueType >
A sparse node map 510
LinkPred::UNetwork< LabelT, NodeIDT, EdgeT >::NonEdgeIt
Nonedges iterator 517
LinkPred::DNetwork< LabelT, NodeIDT, EdgeT >::NonEdgeIt
Nonedges iterator 526
LinkPred::Utils::PairCompRight< FirstT, SecondT, CompareT >
Class for comparing pairs based on second elements only 536
LinkPred::Pearson
Pearson similarity (Pearson correlation coefficient) 537
LinkPred::PEFactory< Network, LPredictorT, TestDataT, PerfMeasureT >
Factory class to create link predictors and performance measures 540
LinkPred::PerfCurve< PredResultsT >
Abstract performance curve 541
LinkPred::PerfeEvalExpDescp< Network >
Structure storing experiment description 547
LinkPred::PerfEvalExp< Network, TestDataT, LPredictorT, PredResultsT, PerfMeasureT >
Performance evaluation experiment 549
LinkPred::PerfEvaluator< TestDataT, LPredictorT, PredResultsT, PerfMeasureT >
Performance evaluator 555
LinkPred::PerfMeasure< PredResultsT >
Abstract performance measure 562
LinkPred::Simp::PerfRes
A structure to store performance results 568
LinkPred::PR< PredResultsT >
The precision recall curve 569
LinkPred::Simp::Predictor
A class that simplifies the use of link prediction algorithms 578
LinkPred::PredResults< TestDataT, LPredictorT, ScoresContainerT >
A class to store and manage prediction results 614
LinkPred::Simp::Evaluator::Factory::PSTParams
Parameters of PST 626

LinkPred::RandomGen
A random number generator 627
LinkPred::RndClassifier< InRndIt, OutRndIt, ScoreRndIt >
Random classifier 632
LinkPred::UNetwork< LabelT, NodeIDT, EdgeT >::RndEdgeIt
Randomized edges iterator 636
LinkPred::DNetwork< LabelT, NodeIDT, EdgeT >::RndEdgeIt
Randomized edges iterator 641
LinkPred::DNetwork< LabelT, NodeIDT, EdgeT >::RndNodeIt
Randomized Nodes iterator 646
LinkPred::UNetwork< LabelT, NodeIDT, EdgeT >::RndNodeIt
Randomized Nodes iterator 651
LinkPred::UNetwork< LabelT, NodeIDT, EdgeT >::RndNonEdgeIt
Randomized nonedges iterator 656
LinkPred::DNetwork< LabelT, NodeIDT, EdgeT >::RndNonEdgeIt
Randomized nonedges iterator 661
LinkPred::Simp::Evaluator::Factory::RNDParams
Parameters of RND 666
LinkPred::ROC< PredResultsT >
Receiver Operating Characteristic curve 667
LinkPred::Simp::Evaluator::Factory::SBMParams
Parameters of SBM 677
LinkPred::PredResults< TestDataT, LPredictorT, ScoresContainerT >::ScoreIterator
Score iterator 678
LinkPred::Simp::Evaluator::Factory::SHPParams
Parameters of SHP 689
LinkPred::SimMeasure
Interface of a similarity measure 690
LinkPred::TestData< Network, EdgeContT >
Test data 693
LinkPred::TestEdgeGen< Network, EdgeContT >
Generate true positives and true negatives 704
LinkPred::TestData< Network, EdgeContT >::TestEdgeIt
Test edges iterator 709
LinkPred::TestEdgeGen< Network, EdgeContT >::TNEdgeIt
TN edges iterator 719
LinkPred::TestEdgeGen< Network, EdgeContT >::TPEdgeIt
TP edges iterator 729
LinkPred::TPR< PredResultsT >
Compute top precision 738
LinkPred::UADAPredictor< Network, EdgeRndIt, ScoreRndIt, EdgeRndOutIt >
Adamic Adar index link predictor 745
LinkPred::UCNEPredictor< Network, EdgeRndIt, ScoreRndIt, EdgeRndOutIt >
Common neighbor link predictor 751
LinkPred::UCRAPredictor< Network, EdgeRndIt, ScoreRndIt, EdgeRndOutIt >
Local path link predictor 756
LinkPred::UCSTPredictor< Network, EdgeRndIt, ScoreRndIt, EdgeRndOutIt >
Constant link predictor 762
LinkPred::UECLPredictor< Network, EdgeRndIt, ScoreRndIt, EdgeRndOutIt >
Encoder-classifier link predictor 768
LinkPred::UESMPredictor< Network, EdgeRndIt, ScoreRndIt, EdgeRndOutIt >
Encoder-Similarity measure link predictor 775
LinkPred::UFBMPredictor< Network, EdgeRndIt, ScoreRndIt, EdgeRndOutIt >
Fast blocking model link predictor 781
LinkPred::UHDIPredictor< Network, EdgeRndIt, ScoreRndIt, EdgeRndOutIt >
Hub depromoted index link predictor 786
LinkPred::UHPIPredictor< Network, EdgeRndIt, ScoreRndIt, EdgeRndOutIt >
Hub promoted index link predictor 792

LinkPred::UHRGPredictor< Network, EdgeRndIt, ScoreRndIt, EdgeRndOutIt >
HRG predictor 797
LinkPred::UHYPPredictor< Network, EdgeRndIt, ScoreRndIt, EdgeRndOutIt >
Hypermap predictor 803
LinkPred::UJIDPredictor< Network, EdgeRndIt, ScoreRndIt, EdgeRndOutIt >
Jackard index link predictor 811
LinkPred::UKABPredictor< Network, EdgeRndIt, ScoreRndIt, EdgeRndOutIt >
A scalable popularity similarity link predictor proposed in: "Kerrache, S., Alharbi, R. & Benhidour, H. A Scalable Similarity-Popularity Link Prediction Method. Sci Rep 10, 6394 (2020)". `https://doi.org/10.1038/s41598-020-62636-1` 817
LinkPred::ULCPPredictor< Network, EdgeRndIt, ScoreRndIt, EdgeRndOutIt >
Local path link predictor 824
LinkPred::ULHNPredictor< Network, EdgeRndIt, ScoreRndIt, EdgeRndOutIt >
Leicht-Holme-Newman index link predictor 829
LinkPred::ULPredictor< NetworkT, EdgeRndItT, ScoreRndItT, EdgeRndOutItT >
The interface of a link predictor in an undirected network 835
LinkPred::UMPSPredictor< Network, EdgeRndIt, ScoreRndIt, EdgeRndOutIt >
A scalable popularity similarity link predictor 843
LinkPred::UNEDPredictor< Network, EdgeRndIt, ScoreRndIt, EdgeRndOutIt >
A neighbors degree link predictor 861
LinkPred::UNetwork< LabelT, NodeIDT, EdgeT >
This class represents an undirected network in the sense of graph theory 867
LinkPred::UPATPredictor< Network, EdgeRndIt, ScoreRndIt, EdgeRndOutIt >
A preferential attachment link predictor 895
LinkPred::UPSTPredictor< Network, EdgeRndIt, ScoreRndIt, EdgeRndOutIt >
A link predictor that prestores edge scores. This allows to seemingly integrate results from external link prediction algorithms to LinkPred (for example, users may implement their own link prediction algorithm and then use this link predictor to use compare their results to algorithms available in LinkPred) 900
LinkPred::URALPredictor< Network, EdgeRndIt, ScoreRndIt, EdgeRndOutIt >
A resource allocation link predictor 905
LinkPred::URNDPredictor< Network, EdgeRndIt, ScoreRndIt, EdgeRndOutIt >
A random link predictor 911
LinkPred::USAIPredictor< Network, EdgeRndIt, ScoreRndIt, EdgeRndOutIt >
A Salton index link predictor 915
LinkPred::USBMPredictor< Network, EdgeRndIt, ScoreRndIt, EdgeRndOutIt >
The stochastic block model link predictor 922
LinkPred::USHPPredictor< Network, EdgeRndIt, ScoreRndIt, EdgeRndOutIt >
A shortest path link predictor link predictor 927
LinkPred::USOIPredictor< Network, EdgeRndIt, ScoreRndIt, EdgeRndOutIt >
A Sorensen index link predictor 935
LinkPred::USUMPredictor< Network, EdgeRndIt, ScoreRndIt, EdgeRndOutIt >
A sum-of-degrees link predictor 941
LinkPred::Vec
This class represent a vector (in the sense of linear algebra) 946

Chapter 6

File Index

6.1 File List

Here is a list of all documented files with brief descriptions:

bindings/Java/include/LinkPredJavaConfig.hpp
Contains configuration options . . . 971
bindings/Python/include/LinkPredPythonConfig.hpp
Contains configuration options . . . 971
include/LinkPredConfig.hpp
Contains configuration options . . . 972
include/linkpred.hpp
Includes all headers of the library . . . 972
include/linkpred/instantiations.hpp
Contains explicit instantiations of template classes . . . 1004
include/linkpred/core/core.hpp
Includes the headers related to core classes . . . 973
include/linkpred/core/dnetwork/dnetwork.hpp
Contains the implementation of a directed network data structure . . . 974
include/linkpred/core/ds/bheap.hpp
Contains the implementation of a templated binary heap . . . 975
include/linkpred/core/ds/bhmap.hpp
Contains the implementation of a templated bidirectional half map . . . 976
include/linkpred/core/ds/ds.hpp
Includes the headers related to data structures . . . 977
include/linkpred/core/ds/lmapqueue.hpp
Contains the implementation of a templated map-priority queue with limit on the capacity . . . 978
include/linkpred/core/unetwork/unetwork.hpp
Contains the implementation of an undirected network data structure . . . 979
include/linkpred/graphalg/graphalg.hpp
Includes the headers related to graph algorithms . . . 997
include/linkpred/graphalg/encoders/encoder.hpp
Contains the interface of a network encoder . . . 983
include/linkpred/graphalg/encoders/encoders.hpp
Includes the headers related to network encoders . . . 984
include/linkpred/graphalg/encoders/deepwalk/deepwalk.hpp
DeepWalk encoder . . . 981
include/linkpred/graphalg/encoders/hmsm/hmsm.hpp
Contains the implementation of an algorithm for embedding a network using a a hidden metric space model . . . 985

include/linkpred/graphalg/encoders/largevis/largevis.hpp
LargeVis encoder . . . 987
include/linkpred/graphalg/encoders/lem/lem.hpp
Contains the implementation of Laplacian eigenmaps embedding (LEM) . . . 988
include/linkpred/graphalg/encoders/line/line.hpp
The LINE (Large-scale Information Network Embedding) encoder . . . 989
include/linkpred/graphalg/encoders/lle/lle.hpp
Contains the implementation of locally linear graph embedding (LLE) . . . 991
include/linkpred/graphalg/encoders/matfact/matfact.hpp
Contains the implementation of an algorithm for embedding a network using matrix factorization. Reference: Koren, Y., Bell, R., and Volinsky, C. (2009). Matrix factorization techniques for recommender systems. Computer, 42(8):30–37 Ahmed, A., Shervashidze, N., Narayanamurthy, S., Josifovski, V., and Smola, A. J. (2013). Distributed large-scale natural graph factorization. In Proceedings of the 22nd International Conference on World Wide Web, WWW '13, pages 37–48, New York, NY, USA. Association for Computing Machinery . . . 992
include/linkpred/graphalg/encoders/matfact/matfactcg.hpp
Contains the implementation of the optimization problem associated with matrix factorization . 994
include/linkpred/graphalg/encoders/node2vec/node2vec.hpp
A node2vec encoder . . . 996
include/linkpred/graphalg/shortestpaths/dijkstra.hpp
Contains an implementation of Dijkstra's algorithm . . . 998
include/linkpred/graphalg/shortestpaths/netdistcalculator.hpp
Contains the implementation of classes for computing distances in network . . . 999
include/linkpred/graphalg/shortestpaths/shortestpaths.hpp
Includes the headers related to shortest paths algorithms . . . 1001
include/linkpred/graphalg/traversal/graphtraversal.hpp
Contains the implementation of graph traversal algorithms . . . 1002
include/linkpred/graphalg/traversal/traversal.hpp
Includes the headers related to graph traversal algorithms . . . 1004
include/linkpred/ml/ml.hpp
Includes the headers related to learning-based predictors . . . 1020
include/linkpred/ml/classifiers/classifier.hpp
Contains the interface of a classifier . . . 1005
include/linkpred/ml/classifiers/classifiers.hpp
Includes the headers related to classifiers . . . 1007
include/linkpred/ml/classifiers/ffn/ffn.hpp
Contains a wrapper of mlpack::ann::FFN (feed-forward neural network) . . . 1008
include/linkpred/ml/classifiers/linearsvm/linearsvm.hpp
Contains a wrapper of mlpack::smv::LinearSVM . . . 1010
include/linkpred/ml/classifiers/logistic/logisticregresser.hpp
Contains the implementation of a logistic regression algorithm . . . 1012
include/linkpred/ml/classifiers/logistic/logregcg.hpp
Contains the implementation of a logistic regression optimization problem . . . 1015
include/linkpred/ml/classifiers/naivebayes/naivebayes.hpp
Contains a wrapper of mlpack::maive_bayes::NaiveBayesClassifier . . . 1016
include/linkpred/ml/classifiers/rndclassifier/rndclassifier.hpp
Contains a random classiffier (for debugging purposes) . . . 1017
include/linkpred/ml/simmeasures/cosinesim.hpp
Contains the implementation of cosine similarity . . . 1021
include/linkpred/ml/simmeasures/dotprod.hpp
Contains the implementation of the dot product similarity measure . . . 1023
include/linkpred/ml/simmeasures/l1sim.hpp
Contains the implementation of L1 similarity . . . 1025
include/linkpred/ml/simmeasures/l2sim.hpp
Contains the implementation of L2 similarity . . . 1027
include/linkpred/ml/simmeasures/lpsim.hpp
Contains the implementation of LP similarity . . . 1029

include/linkpred/ml/simmeasures/pearson.hpp
Contains the implementation of Perason similarity 1031
include/linkpred/ml/simmeasures/simmeasure.hpp
Contains the interface of a similarity measure 1033
include/linkpred/ml/simmeasures/simmeasures.hpp
Includes the headers related to similarity measures 1034
include/linkpred/numerical/numerical.hpp
Includes the headers related to numerical algorithms 1043
include/linkpred/numerical/linear/gfmatrix.hpp
Contains the implementation of a full matrix 1035
include/linkpred/numerical/linear/vec.hpp
Contains the implementation of a vector (in the sense of linear algebra) 1037
include/linkpred/numerical/mds/logmdscg.hpp
Contains the implementation of the optimization problem associated with multidimensional scaling using the logarithmic (MULTISCALE) loss function 1038
include/linkpred/numerical/mds/mds.hpp
Contains the implementation of a solver of the multidimensional scaling problem using the logarithmic (MULTISCALE) loss function 1039
include/linkpred/numerical/mf/fastsig.hpp
Contains the implementation of a fast sigmoid 1041
include/linkpred/perf/networkmanipulator.hpp
Contains the implementation of test data related classes 1043
include/linkpred/perf/perf.hpp
Includes the headers related to performance evaluation classes 1046
include/linkpred/perf/perfevaluator.hpp
Includes the headers related to core classes 1047
include/linkpred/perf/perfmeasure.hpp
Contains the implementation of several performance measures 1049
include/linkpred/perf/predresults.hpp
Contains the implementation of a class to store and manage prediction results 1055
include/linkpred/predictors/predictors.hpp
Includes the headers of link predictors 1076
include/linkpred/predictors/directed/dadapredictor.hpp
Contains the implementation of a common neighbor link predictor 1057
include/linkpred/predictors/directed/dcnepredictor.hpp
Contains the implementation of a common neighbor link predictor 1059
include/linkpred/predictors/directed/dhdipredictor.hpp
Contains the implementation of a common neighbor link predictor 1060
include/linkpred/predictors/directed/dhpipredictor.hpp
Contains the implementation of a common neighbor link predictor 1062
include/linkpred/predictors/directed/directed.hpp
Includes the headers of link predictors for directed networks 1063
include/linkpred/predictors/directed/djidpredictor.hpp
Contains the implementation of a common neighbor link predictor 1064
include/linkpred/predictors/directed/dlcppredictor.hpp
Contains the implementation of a directed LCP link predictor 1066
include/linkpred/predictors/directed/dlhnpredictor.hpp
Contains the implementation of a common neighbor link predictor 1067
include/linkpred/predictors/directed/dlpredictor.hpp
Contains the interface of a link predictor 1069
include/linkpred/predictors/directed/dpatpredictor.hpp
Contains the implementation of a common neighbor link predictor 1070
include/linkpred/predictors/directed/dpstpredictor.hpp
Contains the implementation of a link predictor that prestores the scores of edges 1071
include/linkpred/predictors/directed/dsaipredictor.hpp
Contains the implementation of a common neighbor link predictor 1073
include/linkpred/predictors/directed/dsoipredictor.hpp
Contains the implementation of a common neighbor link predictor 1074

include/linkpred/predictors/undirected/uadapredictor.hpp
Contains the implementation of an Adamic Adar index link predictor . . . 1076
include/linkpred/predictors/undirected/ucnepredictor.hpp
Contains the implementation of a common neighbor link predictor . . . 1078
include/linkpred/predictors/undirected/ucrapredictor.hpp
Contains the implementation of the Cannistraci Resource Allocation link predictor . . . 1080
include/linkpred/predictors/undirected/ucstpredictor.hpp
Contains the implementation of a constant link predictor . . . 1081
include/linkpred/predictors/undirected/ueclpredictor.hpp
Contains the implementation of an encoder-classifier link predictor . . . 1083
include/linkpred/predictors/undirected/uesmpredictor.hpp
Contains the implementation of an encoder-similarity measure link predictor . . . 1084
include/linkpred/predictors/undirected/ufbmpredictor.hpp
Contains the implementation of the fast blocking model link predictor . . . 1086
include/linkpred/predictors/undirected/uhdipredictor.hpp
Contains the implementation of a hub depromoted index link predictor . . . 1087
include/linkpred/predictors/undirected/uhpipredictor.hpp
Contains the implementation of a hub promoted index link predictor . . . 1089
include/linkpred/predictors/undirected/uhrgpredictor.hpp
Contains the implementation of the HRG link predictor . . . 1090
include/linkpred/predictors/undirected/uhyppredictor.hpp
Contains the implementation of the hypermap link predictor . . . 1092
include/linkpred/predictors/undirected/ujidpredictor.hpp
Contains the implementation of a Jackard index link predictor . . . 1094
include/linkpred/predictors/undirected/ukabpredictor.hpp
Contains the implementation of a scalable popularity-similarity link predictor . . . 1096
include/linkpred/predictors/undirected/ulcppredictor.hpp
Contains the implementation of a local path link predictor . . . 1098
include/linkpred/predictors/undirected/ulhnpredictor.hpp
Contains the implementation of a Leicht-Holme-Newman index link predictor . . . 1099
include/linkpred/predictors/undirected/ulpredictor.hpp
Contains the interface of a link predictor . . . 1101
include/linkpred/predictors/undirected/umpspredictor.hpp
Contains the implementation of a scalable popularity similarity link predictor . . . 1102
include/linkpred/predictors/undirected/undirected.hpp
Includes the headers of link predictors for undirected networks . . . 1102
include/linkpred/predictors/undirected/unedpredictor.hpp
Contains the implementation of a link prediction algorithm based on the degrees of neighbors . 1104
include/linkpred/predictors/undirected/upatpredictor.hpp
Contains the implementation of a preferential attachment link predictor . . . 1106
include/linkpred/predictors/undirected/upstpredictor.hpp
Contains the implementation of a link predictor that prestores the scores of edges . . . 1108
include/linkpred/predictors/undirected/uralpredictor.hpp
Contains the implementation of a resource allocation link predictor . . . 1109
include/linkpred/predictors/undirected/urndpredictor.hpp
Contains the implementation of a random link predictor . . . 1111
include/linkpred/predictors/undirected/usaipredictor.hpp
Contains the implementation of a Salton index link predictor . . . 1113
include/linkpred/predictors/undirected/usbmpredictor.hpp
Contains the implementation of the stochastic block model link predictor . . . 1114
include/linkpred/predictors/undirected/ushppredictor.hpp
Contains the implementation of a shortest path link predictor . . . 1116
include/linkpred/predictors/undirected/usoipredictor.hpp
Contains the implementation of a Sorensen index link predictor . . . 1118
include/linkpred/predictors/undirected/usumpredictor.hpp
Contains the implementation of a sum-of-degrees link predictor . . . 1119
include/linkpred/simp/edgescore.hpp
Contains the definition of a structure to store the score of an edge . . . 1121

include/linkpred/simp/evaluator.hpp
Contains the definition of a class that simplifies the evaluation of link prediction algorithms . . . 1122
include/linkpred/simp/perfres.hpp
Contains the definition of a structure to store performance results 1123
include/linkpred/simp/predictor.hpp
Contains the definition of a class that simplifies the use of link prediction algorithms 1125
include/linkpred/simp/simp.hpp
Includes all headers of the essential interface 1126
include/linkpred/utils/log.hpp
Contains the implementation of a log class 1127
include/linkpred/utils/loglevel.hpp
Contains the definition of log levels 1128
include/linkpred/utils/miscutils.hpp
Contains the implementation of miscellaneous useful methods 1129
include/linkpred/utils/randomgen.hpp
Contains the implementation of a random number generator 1132
include/linkpred/utils/utils.hpp
Includes the headers related to utility classes 1133

Chapter 7

Module Documentation

7.1 Core classes

This group contains core classes.

Files

- file core.hpp

    Includes the headers related to core classes.
- file dnetwork.hpp

    Contains the implementation of a directed network data structure.
- file bheap.hpp

    Contains the implementation of a templated binary heap.
- file ds.hpp

    Includes the headers related to data structures.
- file lmapqueue.hpp

    Contains the implementation of a templated map-priority queue with limit on the capacity.
- file unetwork.hpp

    Contains the implementation of an undirected network data structure.

7.1.1 Detailed Description

This group contains core classes.

7.2 Graph algorithms.

This group contains graph algorithms.

Files

- file deepwalk.hpp

  DeepWalk encoder.
- file encoder.hpp

  Contains the interface of a network encoder.
- file encoders.hpp

  Includes the headers related to network encoders.
- file hmsm.hpp

  Contains the implementation of an algorithm for embedding a network using a a hidden metric space model.
- file largevis.hpp

  LargeVis encoder.
- file lem.hpp

  Contains the implementation of Laplacian eigenmaps embedding (LEM).
- file line.hpp

  The LINE (Large-scale Information Network Embedding) encoder.
- file lle.hpp

  Contains the implementation of locally linear graph embedding (LLE).
- file matfact.hpp

  Contains the implementation of an algorithm for embedding a network using matrix factorization. Reference: Koren, Y., Bell, R., and Volinsky, C. (2009). Matrix factorization techniques for recommender systems. Computer, 42(8)↩ :30–37 Ahmed, A., Shervashidze, N., Narayanamurthy, S., Josifovski, V., and Smola, A. J. (2013). Distributed large-scale natural graph factorization. In Proceedings of the 22nd International Conference on World Wide Web, WWW '13, pages 37–48, New York, NY, USA. Association for Computing Machinery.
- file matfactcg.hpp

  Contains the implementation of the optimization problem associated with matrix factorization.
- file node2vec.hpp

  A node2vec encoder.
- file graphalg.hpp

  Includes the headers related to graph algorithms.
- file dijkstra.hpp

  Contains an implementation of Dijkstra's algorithm.
- file netdistcalculator.hpp

  Contains the implementation of classes for computing distances in network.
- file shortestpaths.hpp

  Includes the headers related to shortest paths algorithms.
- file graphtraversal.hpp

  Contains the implementation of graph traversal algorithms.
- file traversal.hpp

  Includes the headers related to graph traversal algorithms.

7.2.1 Detailed Description

This group contains graph algorithms.

7.3 Machine learning classes

This group contains classes related to supervised learning algorithms.

Files

- file classifier.hpp

  Contains the interface of a classifier.
- file classifiers.hpp

  Includes the headers related to classifiers.
- file ffn.hpp

  Contains a wrapper of mlpack::ann::FFN (feed-forward neural network).
- file linearsvm.hpp

  Contains a wrapper of mlpack::smv::LinearSVM.
- file logisticregresser.hpp

  Contains the implementation of a logistic regression algorithm.
- file logregcg.hpp

  Contains the implementation of a logistic regression optimization problem.
- file naivebayes.hpp

  Contains a wrapper of mlpack::maive_bayes::NaiveBayesClassifier.
- file rndclassifier.hpp

  Contains a random classiffier (for debugging purposes).
- file ml.hpp

  Includes the headers related to learning-based predictors.
- file cosinesim.hpp

  Contains the implementation of cosine similarity.
- file dotprod.hpp

  Contains the implementation of the dot product similarity measure.
- file l1sim.hpp

  Contains the implementation of L1 similarity.
- file l2sim.hpp

  Contains the implementation of L2 similarity.
- file lpsim.hpp

  Contains the implementation of LP similarity.
- file pearson.hpp

  Contains the implementation of Perason similarity.
- file simmeasure.hpp

  Contains the interface of a similarity measure.
- file simmeasures.hpp

  Includes the headers related to similarity measures.

7.3.1 Detailed Description

This group contains classes related to supervised learning algorithms.

7.4 Numerical classes and algorithms

This group contains classes related to numerical data structures and algroithms.

Files

- file gfmatrix.hpp

  Contains the implementation of a full matrix.
- file vec.hpp

  Contains the implementation of a vector (in the sense of linear algebra).
- file logmdscg.hpp

  Contains the implementation of the optimization problem associated with multidimensional scaling using the logarithmic (MULTISCALE) loss function.
- file mds.hpp

  Contains the implementation of a solver of the multidimensional scaling problem using the logarithmic (MULTISCALE) loss function.
- file fastsig.hpp

  Contains the implementation of a fast sigmoid.
- file numerical.hpp

  Includes the headers related to numerical algorithms.

7.4.1 Detailed Description

This group contains classes related to numerical data structures and algroithms.

7.5 Performance evaluation classes

This group contains classes related to performance evaluation.

Files

- file networkmanipulator.hpp

  Contains the implementation of test data related classes.
- file perf.hpp

  Includes the headers related to performance evaluation classes.
- file perfevaluator.hpp

  Includes the headers related to core classes.
- file perfmeasure.hpp

  Contains the implementation of several performance measures.
- file predresults.hpp

  Contains the implementation of a class to store and manage prediction results.

7.5.1 Detailed Description

This group contains classes related to performance evaluation.

7.6 Link prediction algorithms

This group contains link prediction algorithms.

Files

- file dadapredictor.hpp

  Contains the implementation of a common neighbor link predictor.
- file dcnepredictor.hpp

  Contains the implementation of a common neighbor link predictor.
- file dhdipredictor.hpp

  Contains the implementation of a common neighbor link predictor.
- file dhpipredictor.hpp

  Contains the implementation of a common neighbor link predictor.
- file directed.hpp

  Includes the headers of link predictors for directed networks.
- file djidpredictor.hpp

  Contains the implementation of a common neighbor link predictor.
- file dlcppredictor.hpp

  Contains the implementation of a directed LCP link predictor.
- file dlhnpredictor.hpp

  Contains the implementation of a common neighbor link predictor.
- file dlpredictor.hpp

  Contains the interface of a link predictor.
- file dpatpredictor.hpp

  Contains the implementation of a common neighbor link predictor.
- file dpstpredictor.hpp

  Contains the implementation of a link predictor that prestores the scores of edges.
- file dsaipredictor.hpp

  Contains the implementation of a common neighbor link predictor.
- file dsoipredictor.hpp

  Contains the implementation of a common neighbor link predictor.
- file predictors.hpp

  Includes the headers of link predictors.
- file uadapredictor.hpp

  Contains the implementation of an Adamic Adar index link predictor.
- file ucnepredictor.hpp

  Contains the implementation of a common neighbor link predictor.
- file ucrapredictor.hpp

  Contains the implementation of the Cannistraci Resource Allocation link predictor.
- file ucstpredictor.hpp

  Contains the implementation of a constant link predictor.
- file ueclpredictor.hpp

  Contains the implementation of an encoder-classifier link predictor.
- file uesmpredictor.hpp

  Contains the implementation of an encoder-similarity measure link predictor.
- file ufbmpredictor.hpp

  Contains the implementation of the fast blocking model link predictor.
- file uhdipredictor.hpp

Contains the implementation of a hub depromoted index link predictor.

- file uhpipredictor.hpp

  Contains the implementation of a hub promoted index link predictor.
- file uhrgpredictor.hpp

  Contains the implementation of the HRG link predictor.
- file uhyppredictor.hpp

  Contains the implementation of the hypermap link predictor.
- file ujidpredictor.hpp

  Contains the implementation of a Jackard index link predictor.
- file ukabpredictor.hpp

  Contains the implementation of a scalable popularity-similarity link predictor.
- file ulcppredictor.hpp

  Contains the implementation of a local path link predictor.
- file ulhnpredictor.hpp

  Contains the implementation of a Leicht-Holme-Newman index link predictor.
- file ulpredictor.hpp

  Contains the interface of a link predictor.
- file umpspredictor.hpp

  Contains the implementation of a scalable popularity similarity link predictor.
- file undirected.hpp

  Includes the headers of link predictors for undirected networks.
- file unedpredictor.hpp

  Contains the implementation of a link prediction algorithm based on the degrees of neighbors.
- file upatpredictor.hpp

  Contains the implementation of a preferential attachment link predictor.
- file upstpredictor.hpp

  Contains the implementation of a link predictor that prestores the scores of edges.
- file uralpredictor.hpp

  Contains the implementation of a resource allocation link predictor.
- file urndpredictor.hpp

  Contains the implementation of a random link predictor.
- file usaipredictor.hpp

  Contains the implementation of a Salton index link predictor.
- file usbmpredictor.hpp

  Contains the implementation of the stochastic block model link predictor.
- file ushppredictor.hpp

  Contains the implementation of a shortest path link predictor.
- file usoipredictor.hpp

  Contains the implementation of a Sorensen index link predictor.
- file usumpredictor.hpp

  Contains the implementation of a sum-of-degrees link predictor.

7.6.1 Detailed Description

This group contains link prediction algorithms.

7.7 Simplified interface

This group contains a simplified interface for LinkPred that includes the essential functionalities.

Files

- file edgescore.hpp

    Contains the definition of a structure to store the score of an edge.
- file evaluator.hpp

    Contains the definition of a class that simplifies the evaluation of link prediction algorithms.
- file perfres.hpp

    Contains the definition of a structure to store performance results.
- file predictor.hpp

    Contains the definition of a class that simplifies the use of link prediction algorithms.
- file simp.hpp

    Includes all headers of the essential interface.

7.7.1 Detailed Description

This group contains a simplified interface for LinkPred that includes the essential functionalities.

7.8 Utility functions and classes

This group contains various utility functions and classes.

Files

- file log.hpp

  Contains the implementation of a log class.
- file loglevel.hpp

  Contains the definition of log levels.
- file miscutils.hpp

  Contains the implementation of miscellaneous useful methods.
- file randomgen.hpp

  Contains the implementation of a random number generator.
- file utils.hpp

  Includes the headers related to utility classes.

7.8.1 Detailed Description

This group contains various utility functions and classes.

Chapter 8

Namespace Documentation

8.1 LinkPred Namespace Reference

Main namespace.

Namespaces

- Simp

  Simplified interface. Contains a simplified interface for LinkPred that includes the essential functionalities.
- Utils

  Some utility functions.

Classes

- class ASPDistCalculator

  Approximate shortest path distance calculator.
- class ASPDsimCalculator

  Approximate shortest path dissimilarity calculator.
- class BFS

  BFS graph traversal.
- class BHeap

  A binary heap.
- class Bhmap

  A bidirectional half map.
- class Classifier

  Interface of a binary classifier.
- class Collector

  A class that collects nodes during traversal.
- class CosineSim

  Cosine similarity.
- class Counter

  A class that counts nodes during traversal.
- class DADAPredictor

  Common neighbor link predictor adapted to directed networks.

- class DCNEPredictor

  Common neighbor link predictor adapted to directed networks.
- class DeepWalk

  DeepWalk encoder. Reference: Perozzi, B., Al-Rfou, R., and Skiena, S. (2014). Deepwalk: Online learning of social representations. In Proceedings of the 20th ACM SIGKDD International Conference on Knowledge Discovery and Data Mining, KDD '14, pages 701–710, New York, NY, USA. Association for Computing Machinery. This implementation is based on the code `https://github.com/xgfs/deepwalk-c`.
- class DESPLDistCalculator

  Exact shortest path distance calculator on a directed network with limits on the number of hops.
- class DFS

  DFS graph traversal.
- class DHDIPredictor

  Common neighbor link predictor adapted to directed networks.
- class DHPIPredictor

  Common neighbor link predictor adapted to directed networks.
- class Dijkstra

  An implementation of Dijkstra's algorithm.
- class DJIDPredictor

  Common neighbor link predictor adapted to directed networks.
- class DLCPPredictor

  Common neighbor link predictor adapted to directed networks.
- class DLHNPredictor

  Common neighbor link predictor adapted to directed networks.
- class DLPredictor

  The interface of a link predictor in a directed network.
- class DNetwork

  This class represents a directed network in the sense of graph theory.
- class DotProd

  A simple dot product similarity measure.
- class DPATPredictor

  Common neighbor link predictor adapted to directed networks.
- class DPSTPredictor

  A link predictor that prestores edge scores. This allows to seemingly integrate results from external link prediction algorithms to LinkPred (for example, users may implement their own link prediction algorithm and then use this link predictor to use compare their results to algorithms available in LinkPred).
- class DSAIPredictor

  Common neighbor link predictor adapted to directed networks.
- class DSOIPredictor

  Common neighbor link predictor adapted to directed networks.
- class Encoder

  The interface of a network encoder.
- class ESPDistCalculator

  Exact shortest path distance calculator.
- class ESPDsimCalculator

  Exact shortest path dissimilarity calculator.
- class ESPIndSimlCalculator

  Exact shortest path distance calculator.
- class ESPLDistCalculator

  Exact shortest path distance calculator with limits on the number of hops.
- class ESPLSimlCalculator

  Exact shortest path distance calculator.
- class ESPSimlCalculator

Exact shortest path distance calculator.
- class FastSig

  A fast sigmoid.
- class GCurve

  General performance curve.
- class GFMatrix

  Generalized full matrix. The storage scheme used is column-major.
- class GraphTraversal

  Graph traversal interface.
- class HMSM

  Contains the implementation of an algorithm for embedding a network using a a hidden metric space model. Reference: R. Alharbi, H. Benhidour, and S. Kerrache. "Link Prediction in Complex Net-works Based on a Hidden Variables Model". In: 2016 UKSim-AMSS 18th Inter-national Conference on Computer Modelling and Simulation (UKSim). 2016,pages 119–124.
- class L1Sim

  L1 similarity (negative the L1 norm or Manhattan distance).
- class L2Sim

  L2 similarity (negative the Euclidean distance).
- class LargeVis

  LargeVis encoder. Reference: Tang, J., Liu, J., Zhang, M., and Mei, Q. (2016b). Visualizing large-scale and high-dimensional data. In Bourdeau, J., Hendler, J., Nkambou, R., Horrocks, I., and Zhao, B. Y., editors, WWW, pages 287–297. ACM. This implementation is based on the code `https://github.com/lferry007/LargeVis`.
- class LINE

  LINE encoder.
- class LMapQueue

  A map-priority queue with limit on the capacity.
- class Log

  A log class.
- class LogisticRegresser

  Logistic regression algorithm.
- class LogMDSCG

  Optimization problem associated with multidimensional scaling using the logarithmic (MULTISCALE) loss function.
- class LogRegCG

  Logistic regression optimization problem.
- class LPSim

  LP similarity (negative the Lp norm).
- class MatFact

  Contains the implementation of an algorithm for embedding a network using matrix factorization. Reference: Koren, Y., Bell, R., and Volinsky, C. (2009). Matrix factorization techniques for recommender systems. Computer, 42(8)↩ :30–37 Ahmed, A., Shervashidze, N., Narayanamurthy, S., Josifovski, V., and Smola, A. J. (2013). Distributed large-scale natural graph factorization. In Proceedings of the 22nd International Conference on World Wide Web, WWW '13, pages 37–48, New York, NY, USA. Association for Computing Machinery.
- class MatFactCG

  Optimization problem associated with matrix factorization.
- struct MatFactPbData

  A simple structure to store matrix factoization problem data.
- class MDS

  Solve the MDS problem.
- class NetDistCalculator

  Interface for calculating the distance between nodes in a network.
- class NetIndSimlCalculator

  Interface for calculating the indirect similarity between nodes in a network.
- class NetSimlCalculator

Interface for calculating the similarity between nodes in a network.
- class NetworkManipulator

  Class to manipulate network by removing or adding edges.
- class Node2Vec

  Node2Vec encoder. References: Grover, A. and Leskovec, J. (2016). Node2vec: Scalable feature learning for networks. In Proceedings of the 22nd ACM SIGKDD International Conference on Knowledge Discovery and Data Mining, KDD'16, pages 855–864, New York, NY, USA. Association for Computing Machinery. This implementation is based on the code `https://github.com/xgfs/node2vec-c`.
- class Pearson

  Pearson similarity (Pearson correlation coefficient).
- class PEFactory

  Factory class to create link predictors and performance measures.
- class PerfCurve

  Abstract performance curve.
- struct PerfeEvalExpDescp

  Structure storing experiment description.
- class PerfEvalExp

  Performance evaluation experiment.
- class PerfEvaluator

  Performance evaluator.
- class PerfMeasure

  Abstract performance measure.
- class PR

  The precision recall curve.
- class PredResults

  A class to store and manage prediction results.
- class RandomGen

  A random number generator.
- class RndClassifier

  Random classifier.
- class ROC

  Receiver Operating Characteristic curve.
- class SimMeasure

  Interface of a similarity measure.
- class TestData

  Test data.
- class TestEdgeGen

  Generate true positives and true negatives.
- class TPR

  Compute top precision.
- class UADAPredictor

  Adamic Adar index link predictor.
- class UCNEPredictor

  Common neighbor link predictor.
- class UCRAPredictor

  Local path link predictor.
- class UCSTPredictor

  Constant link predictor.
- class UECLPredictor

  Encoder-classifier link predictor.
- class UESMPredictor

Encoder-Similarity measure link predictor.
- class UFBMPredictor

  Fast blocking model link predictor.
- class UHDIPredictor

  Hub depromoted index link predictor.
- class UHPIPredictor

  Hub promoted index link predictor.
- class UHRGPredictor

  HRG predictor.
- class UHYPPredictor

  Hypermap predictor.
- class UJIDPredictor

  Jackard index link predictor.
- class UKABPredictor

  A scalable popularity similarity link predictor proposed in: "Kerrache, S., Alharbi, R. & Benhidour, H. A Scalable Similarity-Popularity Link Prediction Method. Sci Rep 10, 6394 (2020)". https://doi.org/10.1038/s41598-020-62636-1.
- class ULCPPredictor

  Local path link predictor.
- class ULHNPredictor

  Leicht-Holme-Newman index link predictor.
- class ULPredictor

  The interface of a link predictor in an undirected network.
- class UMPSPredictor

  A scalable popularity similarity link predictor.
- class UNEDPredictor

  A neighbors degree link predictor.
- class UNetwork

  This class represents an undirected network in the sense of graph theory.
- class UPATPredictor

  A preferential attachment link predictor.
- class UPSTPredictor

  A link predictor that prestores edge scores. This allows to seemingly integrate results from external link prediction algorithms to LinkPred (for example, users may implement their own link prediction algorithm and then use this link predictor to use compare their results to algorithms available in LinkPred).
- class URALPredictor

  A resource allocation link predictor.
- class URNDPredictor

  A random link predictor.
- class USAIPredictor

  A Salton index link predictor.
- class USBMPredictor

  The stochastic block model link predictor.
- class USHPPredictor

  A shortest path link predictor link predictor.
- class USOIPredictor

  A Sorensen index link predictor.
- class USUMPredictor

  A sum-of-degrees link predictor.
- class Vec

  This class represent a vector (in the sense of linear algebra).

Typedefs

- using PerfResults = std::map< std::string, double >

Enumerations

- enum CacheLevel { NoCache, NodeCache, NetworkCache }

  Cache levels.
- enum MDSAlg { IpoptMDS, CGMDS }

  MDS solution methods.
- enum LinkClass { TP, FN, FP, TN }

  Enumeration of all classes of links.
- enum LogLevel {
  logError, logWarning, logInfo, logDebug,
  logDebug1, logDebug2, logDebug3 }

  Enumeration of log levels.
- enum SortOrder { None, Inc, Dec }

  Enumeration of different sorting orders.

Functions

- Vec operator+ (Vec const &v1, Vec const &v2)
- Vec operator- (Vec const &v1, Vec const &v2)
- Vec operator* (Vec const &v1, Vec const &v2)
- Vec operator/ (Vec const &v1, Vec const &v2)
- Vec operator+ (double a, Vec const &v)
- Vec operator- (double a, Vec const &v)
- Vec operator* (double a, Vec const &v)
- Vec operator/ (double a, Vec const &v)
- Vec operator+ (Vec const &v, double a)
- Vec operator- (Vec const &v, double a)
- Vec operator* (Vec const &v, double a)
- Vec operator/ (Vec const &v, double a)
- double operator^ (Vec const &v1, Vec const &v2)

Variables

- constexpr double MathPI = 3.141592653589793238462643383279502884L

8.1.1 Detailed Description

Main namespace.

The main namespace of the library.

8.1.2 Typedef Documentation

8.1.2.1 PerfResults

```
using LinkPred::PerfResults = typedef std::map<std::string, double>
```

Map of performance results.Performance results are stored in a map, with the key being the identifier of the performance measure and the data being the value.

8.1.3 Enumeration Type Documentation

8.1.3.1 CacheLevel

```
enum LinkPred::CacheLevel
```

Cache levels.

Enumerator

NoCache	No cache.
NodeCache	Cache at node level.
NetworkCache	Cache at network level.

8.1.3.2 LinkClass

```
enum LinkPred::LinkClass
```

Enumeration of all classes of links.

Enumerator

TP	True positive link.
FN	False negative link.
FP	False positive link.
TN	True negative link.

8.1.3.3 LogLevel

```
enum LinkPred::LogLevel
```

Enumeration of log levels.

Enumerator

logError	At this level, only errors are reported.
logWarning	Warnings are included at this level.
logInfo	Running information included at this level.
logDebug	Debug level.
logDebug1	Debug level 1.
logDebug2	Debug level 2.
logDebug3	Debug level 3.

8.1.3.4 MDSAlg

```
enum LinkPred::MDSAlg
```

MDS solution methods.

Enumerator

IpoptMDS	Solution using Ipopt.
CGMDS	Solution using the CG Descent algorithm.

8.1.3.5 SortOrder

```
enum LinkPred::SortOrder
```

Enumeration of different sorting orders.

Enumerator

None	Not sorted.
Inc	Sorted in increasing order.
Dec	Sorted in decreasing order.

8.1.4 Function Documentation

8.1.4.1 operator*() [1/3]

```
Vec LinkPred::operator* (
            double a,
            Vec const & v )
```

Scalar-vector multiplication.

Parameters

a	The scalar.
v	The vector.

Returns

a ∗ v.

8.1.4.2 operator∗() [2/3]

```
Vec LinkPred::operator* (
            Vec const & v,
            double a )
```

Vector-scalar multiplication.

Parameters

v	The vector.
a	The scalar.

Returns

v ∗ a.

8.1.4.3 operator∗() [3/3]

```
Vec LinkPred::operator* (
            Vec const & v1,
            Vec const & v2 )
```

Vector element-wise multiplication.

Parameters

v1	The first vector.
v2	The second vector.

Returns

v1 .∗ v2.

8.1.4.4 operator+() [1/3]

```
Vec LinkPred::operator+ (
            double a,
            Vec const & v )
```

Scalar-vector addition.

Parameters

a	The scalar.
v	The vector.

Returns

a + v.

8.1.4.5 operator+() [2/3]

```
Vec LinkPred::operator+ (
            Vec const & v,
            double a )
```

Vector-scalar addition.

Parameters

v	The vector.
a	The scalar.

Returns

v + a.

8.1.4.6 operator+() [3/3]

```
Vec LinkPred::operator+ (
            Vec const & v1,
            Vec const & v2 )
```

Vector addition.

Parameters

v1	The first vector.
v2	The second vector.

Returns

v1 + v2.

8.1.4.7 operator-() [1/3]

```
Vec LinkPred::operator- (
            double a,
            Vec const & v )
```

Scalar-vector subtraction.

Parameters

a	The scalar.
v	The vector.

Returns

a - v.

8.1.4.8 operator-() [2/3]

```
Vec LinkPred::operator- (
            Vec const & v,
            double a )
```

Vector-scalar subtraction.

Parameters

v	The vector.
a	The scalar.

Returns

v - a.

8.1.4.9 operator-() [3/3]

```
Vec LinkPred::operator- (
            Vec const & v1,
            Vec const & v2 )
```

Vector subtraction.

Parameters

v1	The first vector.
v2	The second vector.

Returns

v1 - v2.

8.1.4.10 operator/() [1/3]

```
Vec LinkPred::operator/ (
            double a,
            Vec const & v )
```

Scalar-vector division.

Parameters

a	The scalar.
v	The vector.

Returns

a / v.

8.1.4.11 operator/() [2/3]

```
Vec LinkPred::operator/ (
            Vec const & v,
            double a )
```

Vector-scalar division.

Parameters

v	The vector.
a	The scalar.

Returns

v / a.

8.1.4.12 operator/() [3/3]

```
Vec LinkPred::operator/ (
            Vec const & v1,
            Vec const & v2 )
```

Vector element-wise division.

Parameters

v1	The first vector.
v2	The second vector.

Returns

v1 ./ v2.

8.1.4.13 operator^()

```
double LinkPred::operator^ (
            Vec const & v1,
            Vec const & v2 )
```

Dot product.

Parameters

v1	The first vector.
v2	The second vector.

Returns

v1' ∗ v2.

8.1.5 Variable Documentation

8.1.5.1 MathPI

```
constexpr double LinkPred::MathPI = 3.14159265358979323846264338327950288 4L  [constexpr]
```

Pi.

8.2 LinkPred::Simp Namespace Reference

Simplified interface. Contains a simplified interface for LinkPred that includes the essential functionalities.

Classes

- struct EdgeScore

    A structure to store the score of an edge.
- struct EdgeScoreByID

    A structure to store the score of an edge. The node IDs are used instead of labels.
- class Evaluator

    A class that simplifies the evaluation of link prediction algorithms.
- struct PerfRes

    A structure to store performance results.
- class Predictor

    A class that simplifies the use of link prediction algorithms.

8.2.1 Detailed Description

Simplified interface. Contains a simplified interface for LinkPred that includes the essential functionalities.

8.3 LinkPred::Utils Namespace Reference

Some utility functions.

Classes

- struct EdgeScore

    A structure to store the score of an edge.
- struct PairCompRight

    Class for comparing pairs based on second elements only.

Functions

- std::string & ltrim (std::string &str, const char ∗spaces=" \t\n\r\f\v")
- std::string & rtrim (std::string &str, const char ∗spaces=" \t\n\r\f\v")
- std::string & trim (std::string &str, const char ∗spaces=" \t\n\r\f\v")
- template< typename T >
  void clear (std::queue< T > &q)
- template< typename IteratorT >
  void sort (IteratorT begin, IteratorT end, SortOrder sortOrder)
- std::vector< std::size_t > getRndPerm (std::size_t n)
- std::vector< std::size_t > getRndPerm (std::size_t n, long int seed)
- std::pair< double, double > plFit (std::vector< std::size_t > const &data)
- std::pair< double, double > plFit (std::vector< double > const &data)

- template<typename T >
  void print (std::vector< T > const &v, std::string name)
- template<typename U , typename V >
  std::pair< V, U > flip (const std::pair< U, V > &p)
- template<typename U , typename V >
  std::multimap< V, U > flipMap (const std::map< U, V > &map)
- template<typename RandomIterator >
  std::set< typename std::iterator_traits< RandomIterator >::value_type > selectRandom (RandomIterator begin, RandomIterator end, std::size_t k, long int seed)
- template<typename RandomIterator >
  void selectRandomInPlace (RandomIterator begin, RandomIterator end, std::size_t k, long int seed)
- template<typename T , typename InputIterator , typename InserterIt , typename Compare >
  void selectTopK (InputIterator begin, InputIterator end, InserterIt inserter, std::size_t k)
- template<typename RandomIterator >
  RandomIterator getRandom (RandomIterator begin, RandomIterator end, long int seed)
- template<typename T , typename InputIterator , typename InserterIt >
  void filter (InputIterator begin, InputIterator end, std::set< T > const &excepts, InserterIt inserter)
- template<typename InputIterator >
  void print (InputIterator begin, InputIterator end, std::string const &title, std::ostream &out)
- template<typename InputIterator >
  void print (InputIterator begin, InputIterator end, std::string const &title)
- template<typename InputIterator , typename Network >
  void printEdges (InputIterator begin, InputIterator end, Network const &net, std::string const &title, std::ostream &out)
- template<typename InputIterator , typename Network >
  void printEdges (InputIterator begin, InputIterator end, Network const &net, std::string const &title)
- template<typename InputIterator >
  double norm (InputIterator begin, InputIterator end)
- int int_cast (std::size_t source)
- template<typename InputIterator >
  void assertNoNaN (InputIterator begin, InputIterator end)
- std::pair< std::size_t, std::size_t > localRange (std::size_t n, int nbProcs, int procID)
- template<typename RandomIterator >
  void shuffle (RandomIterator begin, RandomIterator end, long int seed)
- template<typename Label = std::string>
  std::vector< std::pair< Label, Label > > readEdges (std::string fileName, bool ignoreLoops=true)
- template<typename Label = std::string>
  std::vector< EdgeScore< Label > > readEdgeScores (std::string fileName)
- template<typename Label = std::string>
  void writeEdgeScores (std::string fileName, std::vector< EdgeScore< Label >> const &esv)

8.3.1 Detailed Description

Some utility functions.

8.3.2 Function Documentation

8.3.2.1 assertNoNaN()

```
template<typename InputIterator >
void LinkPred::Utils::assertNoNaN (
            InputIterator begin,
            InputIterator end )
```

Check for NaN and throws exception if it finds it.

8.3.2.2 clear()

```
template<typename T >
void LinkPred::Utils::clear (
            std::queue< T > & q )
```

Clear the queue (q becomes empty after the call).

Template Parameters

T	the data type stored in the queue.

Parameters

q	The queue.

8.3.2.3 filter()

```
template<typename T , typename InputIterator , typename InserterIt >
void LinkPred::Utils::filter (
            InputIterator begin,
            InputIterator end,
            std::set< T > const & excepts,
            InserterIt inserter )
```

Filter a range.

8.3.2.4 flip()

```
template<typename U , typename V >
std::pair<V, U> LinkPred::Utils::flip (
            const std::pair< U, V > & p )
```

Flip a pair.

Parameters

p	The original pair.

Returns

The flipped pair.

8.3.2.5 flipMap()

```
template<typename U , typename V >
std::multimap<V, U> LinkPred::Utils::flipMap (
            const std::map< U, V > & map )
```

Flip a map.

Parameters

map	The original map.

Returns

The flipped map.

8.3.2.6 getRandom()

```
template<typename RandomIterator >
RandomIterator LinkPred::Utils::getRandom (
            RandomIterator begin,
            RandomIterator end,
            long int seed )
```

Select a random element from a range.

8.3.2.7 getRndPerm() [1/2]

```
std::vector<std::size_t> LinkPred::Utils::getRndPerm (
            std::size_t n )
```

Parameters

n	Size of the permutation.

Returns

A vector containing a random permutation of [0..n-1].

8.3.2.8 getRndPerm() [2/2]

```
std::vector<std::size_t> LinkPred::Utils::getRndPerm (
            std::size_t n,
            long int seed )
```

Parameters

n	Size of the permutation.
seed	The seed.

Returns

A vector containing a random permutation of [0..n-1].

8.3.2.9 int_cast()

```
int LinkPred::Utils::int_cast (
            std::size_t source ) [inline]
```

Controlled numerical cast.

8.3.2.10 localRange()

```
std::pair<std::size_t, std::size_t> LinkPred::Utils::localRange (
            std::size_t n,
            int nbProcs,
            int procID )
```

Compute local range in a distributed setting.

Parameters

n	Total range size.
nbProcs	Number of processors.
procID	The processor ID.

Returns

Pair containing start and end of the range (end is not included in the range).

8.3.2.11 ltrim()

```
std::string& LinkPred::Utils::ltrim (
            std::string & str,
            const char * spaces = " \t\n\r\f\v" ) [inline]
```

Trim the string from left.

Parameters

str	Input string.
spaces	List of spaces.

8.3.2.12 norm()

```
template<typename InputIterator >
double LinkPred::Utils::norm (
            InputIterator begin,
            InputIterator end )
```

Returns

The norm of a range.

8.3.2.13 plFit() [1/2]

```
std::pair<double, double> LinkPred::Utils::plFit (
            std::vector< double > const & data )
```

Fit a continuous power law.

Parameters

data	The data to be fitted.

Returns

A pair containing respectively, the power of the power law and the minimum value.

8.3.2.14 plFit() [2/2]

```
std::pair<double, double> LinkPred::Utils::plFit (
            std::vector< std::size_t > const & data )
```

Fit a discrete power law.

Parameters

data	The data to be fitted.

Returns

A pair containing respectively, the power of the power law and the minimum value.

8.3.2.15 print() [1/3]

```
template<typename InputIterator >
void LinkPred::Utils::print (
            InputIterator begin,
            InputIterator end,
            std::string const & title )
```

Print data.

8.3.2.16 print() [2/3]

```
template<typename InputIterator >
void LinkPred::Utils::print (
            InputIterator begin,
            InputIterator end,
            std::string const & title,
            std::ostream & out )
```

Print data.

8.3.2.17 print() [3/3]

```
template<typename T >
void LinkPred::Utils::print (
            std::vector< T > const & v,
            std::string name )
```

Print vector to standard output.

Parameters

v	The vector.
name	String prefixed to the print.

8.3.2.18 printEdges() [1/2]

```
template<typename InputIterator , typename Network >
void LinkPred::Utils::printEdges (
            InputIterator begin,
            InputIterator end,
            Network const & net,
            std::string const & title )
```

Print edges.

8.3.2.19 printEdges() [2/2]

```
template<typename InputIterator , typename Network >
void LinkPred::Utils::printEdges (
            InputIterator begin,
            InputIterator end,
            Network const & net,
            std::string const & title,
            std::ostream & out )
```

Print edges.

8.3.2.20 readEdges()

```
template<typename Label = std::string>
std::vector<std::pair<Label, Label> > LinkPred::Utils::readEdges (
            std::string fileName,
            bool ignoreLoops = true )
```

Read edges from file.

Parameters

fileName	The file containing the edges.
ignoreLoops	Whether to ignore loops (an edge from a node to itself).

Returns

A vector containing the edge represented as std::pair.

8.3.2.21 readEdgeScores()

```
template<typename Label = std::string>
std::vector<EdgeScore<Label> > LinkPred::Utils::readEdgeScores (
            std::string fileName )
```

Read edges and their scores from file.

Parameters

fileName	The file containing the edges and their scores.

Returns

A vector containing the edges with their scores.

8.3.2.22 rtrim()

```
std::string& LinkPred::Utils::rtrim (
            std::string & str,
            const char * spaces = " \t\n\r\f\v" )  [inline]
```

Trim the string from right.

Parameters

str	Input string.
spaces	List of spaces.

8.3.2.23 selectRandom()

```
template<typename RandomIterator >
std::set< typename std::iterator_traits<RandomIterator>::value_type> LinkPred::Utils::select←
Random (
            RandomIterator begin,
            RandomIterator end,
            std::size_t k,
            long int seed )
```

Randomly select k elements without repetition using Floyd's selection algorithm.

Parameters

begin	Iterator pointing to the start of elements.
end	Iterator pointing to the end of elements.
k	The number of elements to select. If k is larger than the actual number of elements, std::out_of_range is thrown.
seed	Seed for the random number generator.

8.3.2.24 selectRandomInPlace()

```
template<typename RandomIterator >
void LinkPred::Utils::selectRandomInPlace (
            RandomIterator begin,
            RandomIterator end,
            std::size_t k,
            long int seed )
```

Randomly select k elements without repetition using Floyd's selection algorithm. The selected elements are put at the end of the range.

Parameters

begin	Iterator pointing to the start of elements.
end	Iterator pointing to the end of elements.
k	The number of elements to select. If k is larger than the actual number of elements, std::out_of_range is thrown.
seed	Seed for the random number generator.

8.3.2.25 selectTopK()

```
template<typename T , typename InputIterator , typename InserterIt , typename Compare >
void LinkPred::Utils::selectTopK (
            InputIterator begin,
            InputIterator end,
            InserterIt inserter,
            std::size_t k )
```

Randomly select k largest or smallest elements.

Parameters

begin	Iterator pointing to the start of elements.
end	Iterator pointing to the end of elements.
inserter	An inserter iterator where the selected elements will be inserted.
k	The number of elements to select. If k is larger than the actual number of elements, std::out_of_range is thrown.

8.3.2.26 shuffle()

```
template<typename RandomIterator >
void LinkPred::Utils::shuffle (
            RandomIterator begin,
            RandomIterator end,
            long int seed )
```

Randomly shuffle a range.

8.3.2.27 sort()

```
template<typename IteratorT >
void LinkPred::Utils::sort (
            IteratorT begin,
            IteratorT end,
            SortOrder sortOrder )
```

Sort a range.

Template Parameters

T	The data type.
IteratorT	The iterator type.

Parameters

begin	Iterator to the first element in the range.
end	Iterator to one-past-the-last element in the range.
sortOrder	The requested sorting order.

8.3.2.28 trim()

```
std::string& LinkPred::Utils::trim (
            std::string & str,
            const char * spaces = " \t\n\r\f\v" )  [inline]
```

Trim the string from left and right.

Parameters

str	Input string.
spaces	List of spaces.

8.3.2.29 writeEdgeScores()

```
template<typename Label = std::string>
void LinkPred::Utils::writeEdgeScores (
            std::string fileName,
            std::vector< EdgeScore< Label >> const & esv )
```

Write edges and their scores to file.

Parameters

fileName	The file where the edges and their scores will be written.
esv	A vector containing the edges with their scores.

Chapter 9

Class Documentation

9.1 LinkPred::ASPDistCalculator< Network, DistType, NbHopsType > Class Template Reference

Approximate shortest path distance calculator.

`#include <netdistcalculator.hpp>`

Inheritance diagram for LinkPred::ASPDistCalculator< Network, DistType, NbHopsType >:

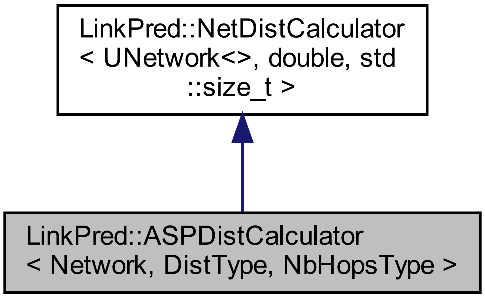

Collaboration diagram for LinkPred::ASPDistCalculator< Network, DistType, NbHopsType >:

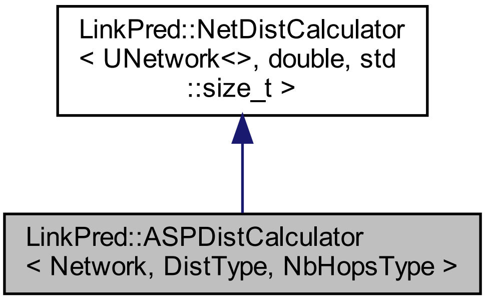

Public Types

- using LengthMapIdType = typename NetDistCalculator< Network, DistType, NbHopsType >::LengthMapIdType
- using NetworkSP = typename NetDistCalculator< Network, DistType, NbHopsType >::NetworkSP
- using NodeID = typename NetDistCalculator< Network, DistType, NbHopsType >::NodeID
- using Label = typename NetDistCalculator< Network, DistType, NbHopsType >::Label
- using Edge = typename NetDistCalculator< Network, DistType, NbHopsType >::Edge
- using NodeDistMap = typename NetDistCalculator< Network, DistType, NbHopsType >::NodeDistMap
- using NodeDistMapSP = typename NetDistCalculator< Network, DistType, NbHopsType >::NodeDistMapSP
- using EdgeLengthMap = typename NetDistCalculator< Network, DistType, NbHopsType >::EdgeLengthMap
- using EdgeLengthMapSP = typename NetDistCalculator< Network, DistType, NbHopsType >::EdgeLengthMapSP

Public Member Functions

- ASPDistCalculator (Dijkstra< Network, DistType, NbHopsType > &dijkstra, EdgeLengthMapSP length)
- ASPDistCalculator (ASPDistCalculator const &that)=default
- ASPDistCalculator & operator= (ASPDistCalculator const &that)=default
- ASPDistCalculator (ASPDistCalculator &&that)=default
- ASPDistCalculator & operator= (ASPDistCalculator &&that)=default
- template<typename InputIterator >
  void setLandmarks (InputIterator landmarksBegin, InputIterator landmarksEnd)
- virtual std::pair< DistType, NbHopsType > getDist (NodeID const &i, NodeID const &j)
- virtual std::pair< DistType, NbHopsType > getIndDist (NodeID const &i, NodeID const &j)
- virtual NodeDistMapSP getDist (NodeID const &i)
- virtual ∼ASPDistCalculator ()

9.1.1 Detailed Description

template<typename Network = UNetwork<>, typename DistType = double, typename NbHopsType = std::size_t>
class LinkPred::ASPDistCalculator< Network, DistType, NbHopsType >

Approximate shortest path distance calculator.

This class implements a fast method to approximate shortest path distances in large networks. First, a set of landmarks is provided, then the distance between any two nodes is computed as the minimum over the sums of distances between the two nodes and any landmark.

Template Parameters

Network	The network type.
DistType	The distance type (can be an integer or floating point type).
NbHopsType	The type of the number of hops (must be an integer type).

9.1.2 Member Typedef Documentation

9.1.2.1 Edge

```
template<typename Network = UNetwork<>, typename DistType = double, typename NbHopsType =
std::size_t>
using LinkPred::ASPDistCalculator< Network, DistType, NbHopsType >::Edge = typename NetDistCalculator<Network
DistType, NbHopsType>::Edge
```

Edge type.

9.1.2.2 EdgeLengthMap

```
template<typename Network = UNetwork<>, typename DistType = double, typename NbHopsType =
std::size_t>
using LinkPred::ASPDistCalculator< Network, DistType, NbHopsType >::EdgeLengthMap = typename
NetDistCalculator<Network, DistType, NbHopsType>::EdgeLengthMap
```

Edge length map.

9.1.2.3 EdgeLengthMapSP

```
template<typename Network = UNetwork<>, typename DistType = double, typename NbHopsType =
std::size_t>
using LinkPred::ASPDistCalculator< Network, DistType, NbHopsType >::EdgeLengthMapSP = typename
NetDistCalculator<Network, DistType, NbHopsType>::EdgeLengthMapSP
```

Shared pointer to an edge length map.

9.1.2.4 Label

```
template<typename Network = UNetwork<>, typename DistType = double, typename NbHopsType =
std::size_t>
using LinkPred::ASPDistCalculator< Network, DistType, NbHopsType >::Label = typename NetDistCalculator<Networ
DistType, NbHopsType>::Label
```

Nodes label type.

9.1.2.5 LengthMapIdType

```
template<typename Network = UNetwork<>, typename DistType = double, typename NbHopsType =
std::size_t>
using LinkPred::ASPDistCalculator< Network, DistType, NbHopsType >::LengthMapIdType = typename
NetDistCalculator<Network, DistType, NbHopsType>::LengthMapIdType
```

Length map ID type.

9.1.2.6 NetworkSP

```
template<typename Network = UNetwork<>, typename DistType = double, typename NbHopsType =
std::size_t>
using LinkPred::ASPDistCalculator< Network, DistType, NbHopsType >::NetworkSP = typename
NetDistCalculator<Network, DistType, NbHopsType>::NetworkSP
```

Shared pointer to network.

9.1.2.7 NodeDistMap

```
template<typename Network = UNetwork<>, typename DistType = double, typename NbHopsType =
std::size_t>
using LinkPred::ASPDistCalculator< Network, DistType, NbHopsType >::NodeDistMap = typename
NetDistCalculator<Network, DistType, NbHopsType>::NodeDistMap
```

Distance map.

9.1.2.8 NodeDistMapSP

```
template<typename Network = UNetwork<>, typename DistType = double, typename NbHopsType =
std::size_t>
using LinkPred::ASPDistCalculator< Network, DistType, NbHopsType >::NodeDistMapSP = typename
NetDistCalculator<Network, DistType, NbHopsType>::NodeDistMapSP
```

Shared pointer to a distance map.

9.1.2.9 NodeID

```
template<typename Network = UNetwork<>, typename DistType = double, typename NbHopsType =
std::size_t>
using LinkPred::ASPDistCalculator< Network, DistType, NbHopsType >::NodeID = typename NetDistCalculator<Netwo[illegible]
DistType, NbHopsType>::NodeID
```

Nodes ID type.

9.1.3 Constructor & Destructor Documentation

9.1.3.1 ASPDistCalculator() [1/3]

```
template<typename Network = UNetwork<>, typename DistType = double, typename NbHopsType =
std::size_t>
LinkPred::ASPDistCalculator< Network, DistType, NbHopsType >::ASPDistCalculator (
            Dijkstra< Network, DistType, NbHopsType > & dijkstra,
            EdgeLengthMapSP length )  [inline]
```

Constructor.

Parameters

dijkstra	A Dijkstra algorithm object.
length	The length map.

9.1.3.2 ASPDistCalculator() [2/3]

```
template<typename Network = UNetwork<>, typename DistType = double, typename NbHopsType =
std::size_t>
LinkPred::ASPDistCalculator< Network, DistType, NbHopsType >::ASPDistCalculator (
             ASPDistCalculator< Network, DistType, NbHopsType > const & that )  [default]
```

Copy constructor.

Parameters

that	The object to copy.

9.1.3.3 ASPDistCalculator() [3/3]

```
template<typename Network = UNetwork<>, typename DistType = double, typename NbHopsType =
std::size_t>
LinkPred::ASPDistCalculator< Network, DistType, NbHopsType >::ASPDistCalculator (
             ASPDistCalculator< Network, DistType, NbHopsType > && that )  [default]
```

Move constructor.

Parameters

that	The object to move.

9.1.3.4 ∼ASPDistCalculator()

```
template<typename Network = UNetwork<>, typename DistType = double, typename NbHopsType =
std::size_t>
virtual LinkPred::ASPDistCalculator< Network, DistType, NbHopsType >::~ASPDistCalculator ( )
[inline], [virtual]
```

Destructor.

9.1.4 Member Function Documentation

9.1.4.1 getDist() [1/2]

```
template<typename Network = UNetwork<>, typename DistType = double, typename NbHopsType =
std::size_t>
virtual NodeDistMapSP LinkPred::ASPDistCalculator< Network, DistType, NbHopsType >::getDist (
            NodeID const & i )  [virtual]
```

Parameters

i	Source node.

Returns

The distance from i to all other nodes.

9.1.4.2 getDist() [2/2]

```
template<typename Network = UNetwork<>, typename DistType = double, typename NbHopsType =
std::size_t>
virtual std::pair<DistType, NbHopsType> LinkPred::ASPDistCalculator< Network, DistType, Nb↩
HopsType >::getDist (
            NodeID const & i,
            NodeID const & j )  [virtual]
```

Parameters

i	Index of the source node.
j	Index of the end node.

Returns

The distance between i and j.

9.1.4.3 getIndDist()

```
template<typename Network = UNetwork<>, typename DistType = double, typename NbHopsType =
std::size_t>
virtual std::pair<DistType, NbHopsType> LinkPred::ASPDistCalculator< Network, DistType, Nb↩
HopsType >::getIndDist (
            NodeID const & i,
            NodeID const & j )  [inline], [virtual]
```

Parameters

i	Index of the source node.
j	Index of the end node.

Returns

The distance between i and j ignoring the edge between i and j.

9.1.4.4 operator=() [1/2]

```
template<typename Network = UNetwork<>, typename DistType = double, typename NbHopsType =
std::size_t>
ASPDistCalculator& LinkPred::ASPDistCalculator< Network, DistType, NbHopsType >::operator= (
            ASPDistCalculator< Network, DistType, NbHopsType > && that )  [default]
```

Move assignment operator.

Parameters

that	The object to move.

9.1.4.5 operator=() [2/2]

```
template<typename Network = UNetwork<>, typename DistType = double, typename NbHopsType =
std::size_t>
ASPDistCalculator& LinkPred::ASPDistCalculator< Network, DistType, NbHopsType >::operator= (
            ASPDistCalculator< Network, DistType, NbHopsType > const & that )  [default]
```

Copy assignment operator.

Parameters

that	The object to copy.

9.1.4.6 setLandmarks()

```
template<typename Network = UNetwork<>, typename DistType = double, typename NbHopsType =
std::size_t>
template<typename InputIterator >
void LinkPred::ASPDistCalculator< Network, DistType, NbHopsType >::setLandmarks (
            InputIterator landmarksBegin,
            InputIterator landmarksEnd )  [inline]
```

Set the landmarks.

Parameters

landmarksBegin	Iterator to the first landmarks.
landmarksEnd	Iterator to one past the last landmarks.

The documentation for this class was generated from the following file:

- include/linkpred/graphalg/shortestpaths/netdistcalculator.hpp

9.2 LinkPred::ASPDsimCalculator< Network, DsimType, NbHopsType > Class Template Reference

Approximate shortest path dissimilarity calculator.

```
#include <netdistcalculator.hpp>
```

Inheritance diagram for LinkPred::ASPDsimCalculator< Network, DsimType, NbHopsType >:

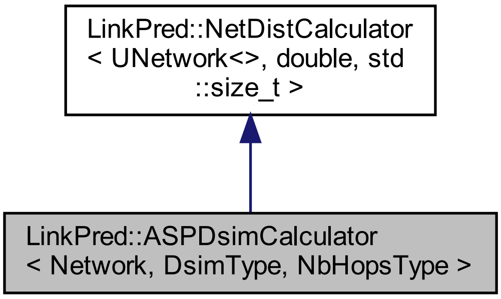

Collaboration diagram for LinkPred::ASPDsimCalculator< Network, DsimType, NbHopsType >:

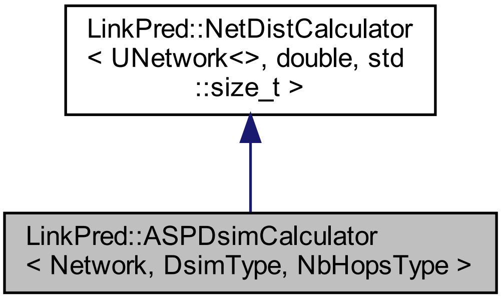

Public Types

- using LengthMapIdType = typename NetDistCalculator< Network, DsimType, NbHopsType >↩ ::LengthMapIdType
- using NetworkSP = typename NetDistCalculator< Network, DsimType, NbHopsType >::NetworkSP
- using NodeID = typename NetDistCalculator< Network, DsimType, NbHopsType >::NodeID
- using Label = typename NetDistCalculator< Network, DsimType, NbHopsType >::Label
- using Edge = typename NetDistCalculator< Network, DsimType, NbHopsType >::Edge
- using NodeDsimMap = typename NetDistCalculator< Network, DsimType, NbHopsType >::NodeDistMap
- using NodeDsimMapSP = typename NetDistCalculator< Network, DsimType, NbHopsType >↩ ::NodeDistMapSP
- using EdgeLengthMap = typename NetDistCalculator< Network, DsimType, NbHopsType >::EdgeLengthMap
- using EdgeLengthMapSP = typename NetDistCalculator< Network, DsimType, NbHopsType >↩ ::EdgeLengthMapSP

Public Member Functions

- ASPDsimCalculator (Dijkstra< Network, DsimType, NbHopsType > &dijkstra, EdgeLengthMapSP length)
- ASPDsimCalculator (ASPDsimCalculator const &that)=default
- ASPDsimCalculator & operator= (ASPDsimCalculator const &that)=default
- ASPDsimCalculator (ASPDsimCalculator &&that)=default
- ASPDsimCalculator & operator= (ASPDsimCalculator &&that)=default
- template<typename InputIterator >
  void setLandmarks (InputIterator landmarksBegin, InputIterator landmarksEnd)
- virtual std::pair< DsimType, NbHopsType > getDist (NodeID const &i, NodeID const &j)
- virtual std::pair< DsimType, NbHopsType > getIndDist (NodeID const &i, NodeID const &j)
- virtual NodeDsimMapSP getDist (NodeID const &i)
- virtual ∼ASPDsimCalculator ()

9.2.1 Detailed Description

template<typename Network = UNetwork<>, typename DsimType = double, typename NbHopsType = std::size_t>
class LinkPred::ASPDsimCalculator< Network, DsimType, NbHopsType >

Approximate shortest path dissimilarity calculator.

Template Parameters

Network	The network type.
DistType	The distance type (can be an integer or floating point type).
NbHopsType	The type of the number of hops (must be an integer type).

9.2.2 Member Typedef Documentation

9.2.2.1 Edge

```
template<typename Network = UNetwork<>, typename DsimType = double, typename NbHopsType =
std::size_t>
```

```
using LinkPred::ASPDsimCalculator< Network, DsimType, NbHopsType >::Edge = typename NetDistCalculator<Network
DsimType, NbHopsType>::Edge
```

Edge type.

9.2.2.2 EdgeLengthMap

```
template<typename Network = UNetwork<>, typename DsimType = double, typename NbHopsType =
std::size_t>
using LinkPred::ASPDsimCalculator< Network, DsimType, NbHopsType >::EdgeLengthMap = typename
NetDistCalculator<Network, DsimType, NbHopsType>::EdgeLengthMap
```

Edge length map.

9.2.2.3 EdgeLengthMapSP

```
template<typename Network = UNetwork<>, typename DsimType = double, typename NbHopsType =
std::size_t>
using LinkPred::ASPDsimCalculator< Network, DsimType, NbHopsType >::EdgeLengthMapSP = typename
NetDistCalculator<Network, DsimType, NbHopsType>::EdgeLengthMapSP
```

Shared pointer to an edge length map.

9.2.2.4 Label

```
template<typename Network = UNetwork<>, typename DsimType = double, typename NbHopsType =
std::size_t>
using LinkPred::ASPDsimCalculator< Network, DsimType, NbHopsType >::Label = typename NetDistCalculator<Networ
DsimType, NbHopsType>::Label
```

Nodes label type.

9.2.2.5 LengthMapIdType

```
template<typename Network = UNetwork<>, typename DsimType = double, typename NbHopsType =
std::size_t>
using LinkPred::ASPDsimCalculator< Network, DsimType, NbHopsType >::LengthMapIdType = typename
NetDistCalculator<Network, DsimType, NbHopsType>::LengthMapIdType
```

Length map ID type.

9.2.2.6 NetworkSP

```
template<typename Network = UNetwork<>, typename DsimType = double, typename NbHopsType =
std::size_t>
using LinkPred::ASPDsimCalculator< Network, DsimType, NbHopsType >::NetworkSP = typename
NetDistCalculator<Network, DsimType, NbHopsType>::NetworkSP
```

Shared pointer to network.

9.2.2.7 NodeDsimMap

```
template<typename Network = UNetwork<>, typename DsimType = double, typename NbHopsType =
std::size_t>
using LinkPred::ASPDsimCalculator< Network, DsimType, NbHopsType >::NodeDsimMap = typename
NetDistCalculator<Network, DsimType, NbHopsType>::NodeDistMap
```

Dissimilarity map.

9.2.2.8 NodeDsimMapSP

```
template<typename Network = UNetwork<>, typename DsimType = double, typename NbHopsType =
std::size_t>
using LinkPred::ASPDsimCalculator< Network, DsimType, NbHopsType >::NodeDsimMapSP = typename
NetDistCalculator<Network, DsimType, NbHopsType>::NodeDistMapSP
```

Shared pointer to a Dissimilarity map.

9.2.2.9 NodeID

```
template<typename Network = UNetwork<>, typename DsimType = double, typename NbHopsType =
std::size_t>
using LinkPred::ASPDsimCalculator< Network, DsimType, NbHopsType >::NodeID = typename NetDistCalculator<Netwo
DsimType, NbHopsType>::NodeID
```

Nodes ID type.

9.2.3 Constructor & Destructor Documentation

9.2.3.1 ASPDsimCalculator() [1/3]

```
template<typename Network = UNetwork<>, typename DsimType = double, typename NbHopsType =
std::size_t>
LinkPred::ASPDsimCalculator< Network, DsimType, NbHopsType >::ASPDsimCalculator (
            Dijkstra< Network, DsimType, NbHopsType > & dijkstra,
            EdgeLengthMapSP length )  [inline]
```

Constructor.

Parameters

dijkstra	A Dijkstra algorithm object.
length	The length map.

9.2.3.2 ASPDsimCalculator() [2/3]

```
template<typename Network = UNetwork<>, typename DsimType = double, typename NbHopsType =
std::size_t>
LinkPred::ASPDsimCalculator< Network, DsimType, NbHopsType >::ASPDsimCalculator (
            ASPDsimCalculator< Network, DsimType, NbHopsType > const & that )  [default]
```

Copy constructor.

Parameters

that	The object to copy.

9.2.3.3 ASPDsimCalculator() [3/3]

```
template<typename Network = UNetwork<>, typename DsimType = double, typename NbHopsType =
std::size_t>
LinkPred::ASPDsimCalculator< Network, DsimType, NbHopsType >::ASPDsimCalculator (
            ASPDsimCalculator< Network, DsimType, NbHopsType > && that )  [default]
```

Move constructor.

Parameters

that	The object to move.

9.2.3.4 ∼ASPDsimCalculator()

```
template<typename Network = UNetwork<>, typename DsimType = double, typename NbHopsType =
std::size_t>
virtual LinkPred::ASPDsimCalculator< Network, DsimType, NbHopsType >::~ASPDsimCalculator ( )
[inline], [virtual]
```

Destructor.

9.2.4 Member Function Documentation

9.2.4.1 getDist() [1/2]

```
template<typename Network = UNetwork<>, typename DsimType = double, typename NbHopsType =
std::size_t>
virtual NodeDsimMapSP LinkPred::ASPDsimCalculator< Network, DsimType, NbHopsType >::getDist (
            NodeID const & i )  [virtual]
```

Parameters

i	Source node.

Returns

The distance from i to all other nodes.

9.2.4.2 getDist() [2/2]

```
template<typename Network = UNetwork<>, typename DsimType = double, typename NbHopsType =
std::size_t>
virtual std::pair<DsimType, NbHopsType> LinkPred::ASPDsimCalculator< Network, DsimType, Nb↩
HopsType >::getDist (
            NodeID const & i,
            NodeID const & j )  [virtual]
```

Parameters

i	Index of the source node.
j	Index of the end node.

Returns

The distance between i and j.

9.2.4.3 getIndDist()

```
template<typename Network = UNetwork<>, typename DsimType = double, typename NbHopsType =
std::size_t>
virtual std::pair<DsimType, NbHopsType> LinkPred::ASPDsimCalculator< Network, DsimType, Nb↩
HopsType >::getIndDist (
            NodeID const & i,
            NodeID const & j )  [inline], [virtual]
```

Parameters

i	Index of the source node.
j	Index of the end node.

Returns

The distance between i and j ignoring the edge between i and j.

9.2.4.4 operator=() [1/2]

```
template<typename Network = UNetwork<>, typename DsimType = double, typename NbHopsType =
std::size_t>
ASPDsimCalculator& LinkPred::ASPDsimCalculator< Network, DsimType, NbHopsType >::operator= (
            ASPDsimCalculator< Network, DsimType, NbHopsType > && that )  [default]
```

Move assignment operator.

Parameters

that	The object to move.

9.2.4.5 operator=() [2/2]

```
template<typename Network = UNetwork<>, typename DsimType = double, typename NbHopsType =
std::size_t>
ASPDsimCalculator& LinkPred::ASPDsimCalculator< Network, DsimType, NbHopsType >::operator= (
            ASPDsimCalculator< Network, DsimType, NbHopsType > const & that )  [default]
```

Copy assignment operator.

Parameters

that	The object to copy.

9.2.4.6 setLandmarks()

```
template<typename Network = UNetwork<>, typename DsimType = double, typename NbHopsType =
std::size_t>
template<typename InputIterator >
void LinkPred::ASPDsimCalculator< Network, DsimType, NbHopsType >::setLandmarks (
            InputIterator landmarksBegin,
            InputIterator landmarksEnd )  [inline]
```

Set the landmarks.

Parameters

landmarksBegin	Iterator to the first landmarks.
landmarksEnd	Iterator to one past the last landmarks.

The documentation for this class was generated from the following file:

- include/linkpred/graphalg/shortestpaths/netdistcalculator.hpp

9.3 LinkPred::BFS< Network, NodeProcessor > Class Template Reference

BFS graph traversal.

`#include <graphtraversal.hpp>`

Inheritance diagram for LinkPred::BFS< Network, NodeProcessor >:

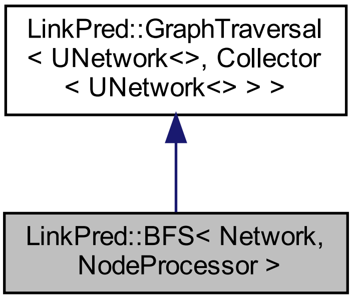

Collaboration diagram for LinkPred::BFS< Network, NodeProcessor >:

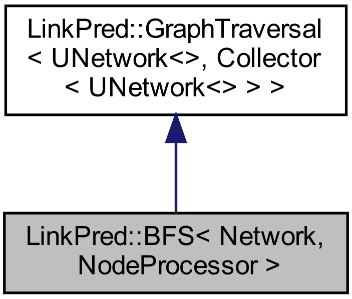

Public Member Functions

- BFS (std::shared_ptr< Network const > net)
- BFS (BFS const &that)=default
- BFS & operator= (BFS const &that)=default
- BFS (BFS &&that)=default
- BFS & operator= (BFS &&that)=default
- virtual void traverse (typename Network::NodeID srcNode, NodeProcessor &processor)
- virtual ∼BFS ()=default

9.3.1 Detailed Description

template<typename Network = UNetwork<>, typename NodeProcessor = Collector< Network>>
class LinkPred::BFS< Network, NodeProcessor >

BFS graph traversal.

Template Parameters

Network	The network type.
NodeProcessor	The node processor type.

9.3.2 Constructor & Destructor Documentation

9.3.2.1 BFS() [1/3]

```
template<typename Network = UNetwork<>, typename NodeProcessor = Collector< Network>>
LinkPred::BFS< Network, NodeProcessor >::BFS (
            std::shared_ptr< Network const > net )  [inline]
```

< The network on which traversal is done. Constructor.

Parameters

net	The network on which traversal is done.

9.3.2.2 BFS() [2/3]

```
template<typename Network = UNetwork<>, typename NodeProcessor = Collector< Network>>
LinkPred::BFS< Network, NodeProcessor >::BFS (
            BFS< Network, NodeProcessor > const & that )  [default]
```

Copy constructor.

Parameters

that	The object to copy.

9.3.2.3 BFS() [3/3]

```
template<typename Network = UNetwork<>, typename NodeProcessor = Collector< Network>>
```

```
LinkPred::BFS< Network, NodeProcessor >::BFS (
            BFS< Network, NodeProcessor > && that )  [default]
```

Move constructor.

Parameters

that	The object to move.

9.3.2.4 ∼BFS()

```
template<typename Network = UNetwork<>, typename NodeProcessor = Collector< Network>>
virtual LinkPred::BFS< Network, NodeProcessor >::~BFS ( )  [virtual], [default]
```

Destructor.

9.3.3 Member Function Documentation

9.3.3.1 operator=() [1/2]

```
template<typename Network = UNetwork<>, typename NodeProcessor = Collector< Network>>
BFS& LinkPred::BFS< Network, NodeProcessor >::operator= (
            BFS< Network, NodeProcessor > && that )  [default]
```

Move assignment operator.

Parameters

that	The object to move.

9.3.3.2 operator=() [2/2]

```
template<typename Network = UNetwork<>, typename NodeProcessor = Collector< Network>>
BFS& LinkPred::BFS< Network, NodeProcessor >::operator= (
            BFS< Network, NodeProcessor > const & that )  [default]
```

Copy assignment operator.

Parameters

that	The object to copy.

9.3.3.3 traverse()

```
template<typename Network = UNetwork<>, typename NodeProcessor = Collector< Network>>
virtual void LinkPred::BFS< Network, NodeProcessor >::traverse (
            typename Network::NodeID srcNode,
            NodeProcessor & processor ) [inline], [virtual]
```

Traverse the graph in BFS order.

Parameters

srcNode	The source node.
processor	The node processor.

The documentation for this class was generated from the following file:

- include/linkpred/graphalg/traversal/graphtraversal.hpp

9.4 LinkPred::BHeap< T, P, ComparatorT, ComparatorP > Class Template Reference

A binary heap.

`#include <bheap.hpp>`

Public Member Functions

- BHeap ()
- BHeap (BHeap const &that)=default
- BHeap & operator= (BHeap const &that)=default
- BHeap (BHeap &&that)=default
- BHeap & operator= (BHeap &&that)=default
- std::size_t size ()
- bool push (T const &elem, P const &pr)
- const std::pair< T, P > & top () const
- void pop ()
- void set (T const &elem, P const &pr)
- void increase (T const &elem, P const &pr)
- void decrease (T const &elem, P const &pr)
- bool tryIncrease (T const &elem, P const &pr)
- bool tryDecrease (T const &elem, P const &pr)
- bool contains (T const &elem)
- void print ()
- virtual ~BHeap ()=default

9.4.1 Detailed Description

**template<typename T, typename P = int, typename ComparatorT = std::less<T>, typename ComparatorP = std::less<P>>
class LinkPred::BHeap< T, P, ComparatorT, ComparatorP >**

A binary heap.

This class implements a binary heap priority queue offering O(log n) insert, remove, and O(log n)^2 priority update.

Template Parameters

T	The stored data type.
P	The priority type.
ComparatorT	The data comparator.
ComparatorP	The priority comparator.

9.4.2 Constructor & Destructor Documentation

9.4.2.1 BHeap() [1/3]

```
template<typename T , typename P = int, typename ComparatorT = std::less<T>, typename ComparatorP
= std::less<P>>
LinkPred::BHeap< T, P, ComparatorT, ComparatorP >::BHeap ( ) [inline]
```

Constructor.

9.4.2.2 BHeap() [2/3]

```
template<typename T , typename P = int, typename ComparatorT = std::less<T>, typename ComparatorP
= std::less<P>>
LinkPred::BHeap< T, P, ComparatorT, ComparatorP >::BHeap (
            BHeap< T, P, ComparatorT, ComparatorP > const & that ) [default]
```

Copy constructor.

Parameters

that	The object to copy.

9.4.2.3 BHeap() [3/3]

```
template<typename T , typename P = int, typename ComparatorT = std::less<T>, typename ComparatorP
= std::less<P>>
LinkPred::BHeap< T, P, ComparatorT, ComparatorP >::BHeap (
            BHeap< T, P, ComparatorT, ComparatorP > && that ) [default]
```

Move constructor.

Parameters

that	The object to move.

9.4.2.4 ~BHeap()

```
template<typename T , typename P = int, typename ComparatorT = std::less<T>, typename ComparatorP
= std::less<P>>
virtual LinkPred::BHeap< T, P, ComparatorT, ComparatorP >::~BHeap ( ) [virtual], [default]
```

Destructor.

9.4.3 Member Function Documentation

9.4.3.1 contains()

```
template<typename T , typename P = int, typename ComparatorT = std::less<T>, typename ComparatorP
= std::less<P>>
bool LinkPred::BHeap< T, P, ComparatorT, ComparatorP >::contains (
            T const & elem ) [inline]
```

Check if an element exists.

Parameters

elem	The element to be checked.

Returns

True if `elem` exists in the heap, false otherwise.

9.4.3.2 decrease()

```
template<typename T , typename P = int, typename ComparatorT = std::less<T>, typename ComparatorP
= std::less<P>>
void LinkPred::BHeap< T, P, ComparatorT, ComparatorP >::decrease (
            T const & elem,
            P const & pr ) [inline]
```

Decrease the priority of an element.

Parameters

elem	The element.
pr	The new priority. This must not be greater than the current one.

9.4.3.3 increase()

```
template<typename T , typename P = int, typename ComparatorT = std::less<T>, typename ComparatorP
= std::less<P>>
void LinkPred::BHeap< T, P, ComparatorT, ComparatorP >::increase (
            T const & elem,
            P const & pr ) [inline]
```

Increase the priority of an element.

Parameters

elem	The element.
pr	The new priority. This must not be smaller than the current one.

9.4.3.4 operator=() [1/2]

```
template<typename T , typename P = int, typename ComparatorT = std::less<T>, typename ComparatorP
= std::less<P>>
BHeap& LinkPred::BHeap< T, P, ComparatorT, ComparatorP >::operator= (
            BHeap< T, P, ComparatorT, ComparatorP > && that ) [default]
```

Move assignment operator.

Parameters

that	The object to move.

9.4.3.5 operator=() [2/2]

```
template<typename T , typename P = int, typename ComparatorT = std::less<T>, typename ComparatorP
= std::less<P>>
BHeap& LinkPred::BHeap< T, P, ComparatorT, ComparatorP >::operator= (
            BHeap< T, P, ComparatorT, ComparatorP > const & that ) [default]
```

Copy assignment operator.

Parameters

that	The object to copy.

9.4.3.6 pop()

```
template<typename T , typename P = int, typename ComparatorT = std::less<T>, typename ComparatorP
= std::less<P>>
void LinkPred::BHeap< T, P, ComparatorT, ComparatorP >::pop ( ) [inline]
```

Remove the element with the highest priority.

9.4.3.7 print()

```
template<typename T , typename P = int, typename ComparatorT = std::less<T>, typename ComparatorP
= std::less<P>>
void LinkPred::BHeap< T, P, ComparatorT, ComparatorP >::print ( ) [inline]
```

Print the heap content to std::cout.

9.4.3.8 push()

```
template<typename T , typename P = int, typename ComparatorT = std::less<T>, typename ComparatorP
= std::less<P>>
bool LinkPred::BHeap< T, P, ComparatorT, ComparatorP >::push (
            T const & elem,
            P const & pr ) [inline]
```

Push an element. If the element already exists, its priority is updated.

Parameters

elem	The element to be pushed.
pr	The priority.

Returns

True if the element is inserted, false if it already exists.

9.4.3.9 set()

```
template<typename T , typename P = int, typename ComparatorT = std::less<T>, typename ComparatorP
= std::less<P>>
void LinkPred::BHeap< T, P, ComparatorT, ComparatorP >::set (
            T const & elem,
            P const & pr ) [inline]
```

Set the priority of an element.

Parameters

elem	The element.
pr	The new priority.

9.4.3.10 size()

```
template<typename T , typename P = int, typename ComparatorT = std::less<T>, typename ComparatorP
= std::less<P>>
std::size_t LinkPred::BHeap< T, P, ComparatorT, ComparatorP >::size ( ) [inline]
```

Returns

The size of the heap.

9.4.3.11 top()

```
template<typename T , typename P = int, typename ComparatorT = std::less<T>, typename ComparatorP
= std::less<P>>
const std::pair<T, P>& LinkPred::BHeap< T, P, ComparatorT, ComparatorP >::top ( ) const [inline]
```

Return the element with the highest priority. This method does not remove the element.

Returns

A constant reference to pair, where the first element is the data and the second is the associated priority.

9.4.3.12 tryDecrease()

```
template<typename T , typename P = int, typename ComparatorT = std::less<T>, typename ComparatorP
= std::less<P>>
bool LinkPred::BHeap< T, P, ComparatorT, ComparatorP >::tryDecrease (
            T const & elem,
            P const & pr ) [inline]
```

Try to decrease the priority.

Parameters

elem	The element.
pr	The new priority. The priority is updated only if this is smaller than the current one.

Returns

True if the priority is updated, false otherwise.

9.4.3.13 tryIncrease()

```
template<typename T , typename P = int, typename ComparatorT = std::less<T>, typename ComparatorP
= std::less<P>>
bool LinkPred::BHeap< T, P, ComparatorT, ComparatorP >::tryIncrease (
            T const & elem,
            P const & pr )  [inline]
```

Try to increase the priority.

Parameters

elem	The element.
pr	The new priority. The priority is updated only if this is greater than the current one.

Returns

True if the priority is updated, false otherwise.

The documentation for this class was generated from the following file:

- include/linkpred/core/ds/bheap.hpp

9.5 LinkPred::Bhmap< K, P, Comparator > Class Template Reference

A bidirectional half map.

`#include <bhmap.hpp>`

Public Types

- using k_const_iterator = typename std::map< K, P, Comparator >::const_iterator
- using p_const_iterator = typename std::vector< std::pair< P, K > >::const_iterator

Public Member Functions

- Bhmap ()=default
- Bhmap (Bhmap const &that)=default
- Bhmap & operator= (Bhmap const &that)=default
- Bhmap (Bhmap &&that)=default
- Bhmap & operator= (Bhmap &&that)=default
- std::pair< P, bool > insert (K const &key)
- std::size_t size () const
- p_const_iterator pbegin () const
- p_const_iterator pend () const
- k_const_iterator kbegin () const
- k_const_iterator kend () const
- const P & pos (K const &key) const
- const K & key (P const &pos) const
- k_const_iterator pfind (K const &key) const
- p_const_iterator kfind (P const &pos) const
- virtual ~Bhmap ()=default

9.5.1 Detailed Description

template<typename K, typename P = std::size_t, typename Comparator = std::less<K>>
class LinkPred::Bhmap< K, P, Comparator >

A bidirectional half map.

This is a special type of a bidirectional map, where user-provided keys are mapped to positions. The latter are contiguous from 0 to n-1, and are assigned according to insertion order. The lookup of position given the key is done in O(log n), whereas the reverse lookup is done is O(1).

Template Parameters

K	The key type.
P	The position type (must be integer).
Comparator	Key comparator.

9.5.2 Member Typedef Documentation

9.5.2.1 k_const_iterator

```
template<typename K , typename P = std::size_t, typename Comparator = std::less<K>>
using LinkPred::Bhmap< K, P, Comparator >::k_const_iterator = typename std::map<K, P, Comparator>↩
::const_iterator
```

Constant iterator over keys.

9.5.2.2 p_const_iterator

```
template<typename K , typename P = std::size_t, typename Comparator = std::less<K>>
using LinkPred::Bhmap< K, P, Comparator >::p_const_iterator = typename std::vector<std↩
::pair<P, K> >::const_iterator
```

Constant iterator over positions.

9.5.3 Constructor & Destructor Documentation

9.5.3.1 Bhmap() [1/3]

```
template<typename K , typename P = std::size_t, typename Comparator = std::less<K>>
LinkPred::Bhmap< K, P, Comparator >::Bhmap ( )  [default]
```

Constructor.

9.5.3.2 Bhmap() [2/3]

```
template<typename K , typename P = std::size_t, typename Comparator = std::less<K>>
LinkPred::Bhmap< K, P, Comparator >::Bhmap (
            Bhmap< K, P, Comparator > const & that ) [default]
```

Copy constructor.

Parameters

that	The object to copy.

9.5.3.3 Bhmap() [3/3]

```
template<typename K , typename P = std::size_t, typename Comparator = std::less<K>>
LinkPred::Bhmap< K, P, Comparator >::Bhmap (
            Bhmap< K, P, Comparator > && that ) [default]
```

Move constructor.

Parameters

that	The object to move.

9.5.3.4 ∼Bhmap()

```
template<typename K , typename P = std::size_t, typename Comparator = std::less<K>>
virtual LinkPred::Bhmap< K, P, Comparator >::~Bhmap ( ) [virtual], [default]
```

Destructor.

9.5.4 Member Function Documentation

9.5.4.1 insert()

```
template<typename K , typename P = std::size_t, typename Comparator = std::less<K>>
std::pair<P, bool> LinkPred::Bhmap< K, P, Comparator >::insert (
            K const & key ) [inline]
```

Add an element if it does not already exist.

Parameters

key	The key.

Returns

True if the key is added, false if it already exists.

9.5.4.2 kbegin()

```
template<typename K , typename P = std::size_t, typename Comparator = std::less<K>>
k_const_iterator LinkPred::Bhmap< K, P, Comparator >::kbegin ( ) const [inline]
```

Returns

Begin iterator.

9.5.4.3 kend()

```
template<typename K , typename P = std::size_t, typename Comparator = std::less<K>>
k_const_iterator LinkPred::Bhmap< K, P, Comparator >::kend ( ) const [inline]
```

Returns

End iterator.

9.5.4.4 key()

```
template<typename K , typename P = std::size_t, typename Comparator = std::less<K>>
const K& LinkPred::Bhmap< K, P, Comparator >::key (
             P const & pos ) const [inline]
```

Returns

A reference to the mapped key.

9.5.4.5 kfind()

```
template<typename K , typename P = std::size_t, typename Comparator = std::less<K>>
p_const_iterator LinkPred::Bhmap< K, P, Comparator >::kfind (
             P const & pos ) const [inline]
```

Find a position. This operation is O(1).

Parameters

pos	The position.

Returns

An iterator to the position.

9.5.4.6 operator=() [1/2]

```
template<typename K , typename P = std::size_t, typename Comparator = std::less<K>>
Bhmap& LinkPred::Bhmap< K, P, Comparator >::operator= (
            Bhmap< K, P, Comparator > && that )  [default]
```

Move assignment operator.

Parameters

that	The object to move.

9.5.4.7 operator=() [2/2]

```
template<typename K , typename P = std::size_t, typename Comparator = std::less<K>>
Bhmap& LinkPred::Bhmap< K, P, Comparator >::operator= (
            Bhmap< K, P, Comparator > const & that )  [default]
```

Copy assignment operator.

Parameters

that	The object to copy.

9.5.4.8 pbegin()

```
template<typename K , typename P = std::size_t, typename Comparator = std::less<K>>
p_const_iterator LinkPred::Bhmap< K, P, Comparator >::pbegin ( ) const  [inline]
```

Returns

Begin iterator.

9.5.4.9 pend()

```
template<typename K , typename P = std::size_t, typename Comparator = std::less<K>>
p_const_iterator LinkPred::Bhmap< K, P, Comparator >::pend ( ) const  [inline]
```

Returns

End iterator.

9.5.4.10 pfind()

```
template<typename K , typename P = std::size_t, typename Comparator = std::less<K>>
k_const_iterator LinkPred::Bhmap< K, P, Comparator >::pfind (
            K const & key ) const  [inline]
```

Find a key. This operation is O(log(n)).

Parameters

key	The key.

Returns

An iterator to the key.

9.5.4.11 pos()

```
template<typename K , typename P = std::size_t, typename Comparator = std::less<K>>
const P& LinkPred::Bhmap< K, P, Comparator >::pos (
            K const & key ) const  [inline]
```

Returns

A reference to the mapped key.

9.5.4.12 size()

```
template<typename K , typename P = std::size_t, typename Comparator = std::less<K>>
std::size_t LinkPred::Bhmap< K, P, Comparator >::size ( ) const  [inline]
```

Returns

The size of the map.

The documentation for this class was generated from the following file:

- include/linkpred/core/ds/bhmap.hpp

9.6 LinkPred::Classifier< InRndIt, OutRndIt, ScoreRndIt > Class Template Reference

Interface of a binary classifier.

```
#include <classifier.hpp>
```

Public Member Functions

- Classifier ()=default
- Classifier (Classifier const &that)=default
- Classifier & operator= (Classifier const &that)=default
- Classifier (Classifier &&that)=default
- Classifier & operator= (Classifier &&that)=default
- virtual void learn (InRndIt trInBegin, InRndIt trInEnd, OutRndIt trOutBegin, OutRndIt trOutEnd)=0
- virtual void predict (InRndIt inBegin, InRndIt inEnd, ScoreRndIt scoresBegin)=0
- const std::string & getName () const
- void setName (const std::string &name)
- virtual ~Classifier ()=default

9.6.1 Detailed Description

**template<typename InRndIt = typename std::vector<Vec>::iterator, typename OutRndIt = typename std::vector<bool>↩ ::iterator, typename ScoreRndIt = typename std::vector<double>::iterator>
class LinkPred::Classifier< InRndIt, OutRndIt, ScoreRndIt >**

Interface of a binary classifier.

Template Parameters

InRndIt	Input (features) iterator type. Must be a random iterator to LinkPred::Vec.
OutRndIt	Output (class) iterator type. Must be a random iterator to LinkPred::Vec.
Score↩ RndIt	Classification scores iterator type. Must be a random iterator to bool.

9.6.2 Constructor & Destructor Documentation

9.6.2.1 Classifier() [1/3]

```
template<typename InRndIt = typename std::vector<Vec>::iterator, typename OutRndIt = typename
std::vector<bool>::iterator, typename ScoreRndIt = typename std::vector<double>::iterator>
LinkPred::Classifier< InRndIt, OutRndIt, ScoreRndIt >::Classifier ( )  [default]
```

Default constructor.

9.6.2.2 Classifier() [2/3]

```
template<typename InRndIt = typename std::vector<Vec>::iterator, typename OutRndIt = typename
std::vector<bool>::iterator, typename ScoreRndIt = typename std::vector<double>::iterator>
LinkPred::Classifier< InRndIt, OutRndIt, ScoreRndIt >::Classifier (
            Classifier< InRndIt, OutRndIt, ScoreRndIt > const & that )  [default]
```

Copy constructor.

Parameters

that	The object to copy.

9.6.2.3 Classifier() [3/3]

```
template<typename InRndIt = typename std::vector<Vec>::iterator, typename OutRndIt = typename
std::vector<bool>::iterator, typename ScoreRndIt = typename std::vector<double>::iterator>
LinkPred::Classifier< InRndIt, OutRndIt, ScoreRndIt >::Classifier (
            Classifier< InRndIt, OutRndIt, ScoreRndIt > && that )  [default]
```

Move constructor.

Parameters

that	The object to move.

9.6.2.4 ∼Classifier()

```
template<typename InRndIt = typename std::vector<Vec>::iterator, typename OutRndIt = typename
std::vector<bool>::iterator, typename ScoreRndIt = typename std::vector<double>::iterator>
virtual LinkPred::Classifier< InRndIt, OutRndIt, ScoreRndIt >::~Classifier ( )  [virtual],
[default]
```

Destructor.

9.6.3 Member Function Documentation

9.6.3.1 getName()

```
template<typename InRndIt = typename std::vector<Vec>::iterator, typename OutRndIt = typename
std::vector<bool>::iterator, typename ScoreRndIt = typename std::vector<double>::iterator>
const std::string& LinkPred::Classifier< InRndIt, OutRndIt, ScoreRndIt >::getName ( ) const
[inline]
```

Returns

The name of the classifier.

9.6.3.2 learn()

```
template<typename InRndIt = typename std::vector<Vec>::iterator, typename OutRndIt = typename
std::vector<bool>::iterator, typename ScoreRndIt = typename std::vector<double>::iterator>
virtual void LinkPred::Classifier< InRndIt, OutRndIt, ScoreRndIt >::learn (
            InRndIt trInBegin,
            InRndIt trInEnd,
            OutRndIt trOutBegin,
            OutRndIt trOutEnd ) [pure virtual]
```

Learn from data.

Parameters

trInBegin	Iterator to the first example features (input).
trInEnd	Iterator to one-past-the-last example features (input).
trOutBegin	Iterator to the first example class (output).
trOutEnd	Iterator to one-past-the-last example class (output).

9.6.3.3 operator=() [1/2]

```
template<typename InRndIt = typename std::vector<Vec>::iterator, typename OutRndIt = typename
std::vector<bool>::iterator, typename ScoreRndIt = typename std::vector<double>::iterator>
Classifier& LinkPred::Classifier< InRndIt, OutRndIt, ScoreRndIt >::operator= (
            Classifier< InRndIt, OutRndIt, ScoreRndIt > && that ) [default]
```

Move assignment operator.

Parameters

that	The object to move.

9.6.3.4 operator=() [2/2]

```
template<typename InRndIt = typename std::vector<Vec>::iterator, typename OutRndIt = typename
std::vector<bool>::iterator, typename ScoreRndIt = typename std::vector<double>::iterator>
Classifier& LinkPred::Classifier< InRndIt, OutRndIt, ScoreRndIt >::operator= (
            Classifier< InRndIt, OutRndIt, ScoreRndIt > const & that )  [default]
```

Copy assignment operator.

Parameters

that	The object to copy.

9.6.3.5 predict()

```
template<typename InRndIt = typename std::vector<Vec>::iterator, typename OutRndIt = typename
std::vector<bool>::iterator, typename ScoreRndIt = typename std::vector<double>::iterator>
virtual void LinkPred::Classifier< InRndIt, OutRndIt, ScoreRndIt >::predict (
            InRndIt inBegin,
            InRndIt inEnd,
            ScoreRndIt scoresBegin )  [pure virtual]
```

Predict.

Parameters

inBegin	Iterator to the first instance features (input).
inEnd	Iterator to one-past-the-last instance features (input).
scoresBegin	Iterator to the first location where to store prediction scores. Memory must be pre-allocated.

9.6.3.6 setName()

```
template<typename InRndIt = typename std::vector<Vec>::iterator, typename OutRndIt = typename
std::vector<bool>::iterator, typename ScoreRndIt = typename std::vector<double>::iterator>
void LinkPred::Classifier< InRndIt, OutRndIt, ScoreRndIt >::setName (
            const std::string & name )  [inline]
```

Set the name of the classifier.

Parameters

name	The new name of the classifier.

The documentation for this class was generated from the following file:

- include/linkpred/ml/classifiers/classifier.hpp

9.7 LinkPred::Collector< Network > Class Template Reference

A class that collects nodes during traversal.

`#include <graphtraversal.hpp>`

Public Member Functions

- Collector ()=default
- Collector (Collector const &that)=default
- Collector & operator= (Collector const &that)=default
- Collector (Collector &&that)=default
- Collector & operator= (Collector &&that)=default
- bool process (typename Network::NodeID const &i)
- const std::queue< typename Network::NodeID > & getVisited () const
- virtual ~Collector ()=default

9.7.1 Detailed Description

template<typename Network = UNetwork<>>
class LinkPred::Collector< Network >

A class that collects nodes during traversal.

Template Parameters

Network	The network type.

9.7.2 Constructor & Destructor Documentation

9.7.2.1 Collector() [1/3]

```
template<typename Network = UNetwork<>>
LinkPred::Collector< Network >::Collector ( )  [default]
```

Constructor.

9.7.2.2 Collector() [2/3]

```
template<typename Network = UNetwork<>>
LinkPred::Collector< Network >::Collector (
            Collector< Network > const & that )  [default]
```

Copy constructor.

Parameters

that	The object to copy.

9.7.2.3 Collector() [3/3]

```
template<typename Network = UNetwork<>>
LinkPred::Collector< Network >::Collector (
            Collector< Network > && that )  [default]
```

Move constructor.

Parameters

that	The object to move.

9.7.2.4 ∼Collector()

```
template<typename Network = UNetwork<>>
virtual LinkPred::Collector< Network >::~Collector ( )  [virtual], [default]
```

Destructor.

9.7.3 Member Function Documentation

9.7.3.1 getVisited()

```
template<typename Network = UNetwork<>>
const std::queue<typename Network::NodeID>& LinkPred::Collector< Network >::getVisited ( )
const  [inline]
```

Returns

The visited nodes.

9.7.3.2 operator=() [1/2]

```
template<typename Network = UNetwork<>>
Collector& LinkPred::Collector< Network >::operator= (
            Collector< Network > && that )  [default]
```

Move assignment operator.

Parameters

that	The object to move.

9.7.3.3 operator=() [2/2]

```
template<typename Network = UNetwork<>>
Collector& LinkPred::Collector< Network >::operator= (
            Collector< Network > const & that )  [default]
```

Copy assignment operator.

Parameters

that	The object to copy.

9.7.3.4 process()

```
template<typename Network = UNetwork<>>
bool LinkPred::Collector< Network >::process (
            typename Network::NodeID const & i )  [inline]
```

Node processing.

Parameters

i	The node's ID.

The documentation for this class was generated from the following file:

- include/linkpred/graphalg/traversal/graphtraversal.hpp

9.8 LinkPred::CosineSim Class Reference

Cosine similarity.

```
#include <cosinesim.hpp>
```

Inheritance diagram for LinkPred::CosineSim:

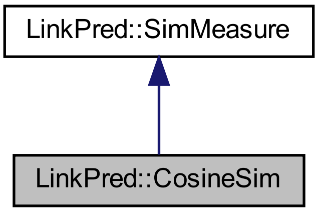

Collaboration diagram for LinkPred::CosineSim:

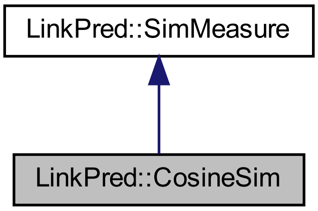

Public Member Functions

- CosineSim ()
- CosineSim (CosineSim const &that)=default
- CosineSim & operator= (CosineSim const &that)=default
- CosineSim (CosineSim &&that)=default
- CosineSim & operator= (CosineSim &&that)=default
- virtual double sim (Vec const &v1, Vec const &v2)
- virtual ∼CosineSim ()=default

9.8.1 Detailed Description

Cosine similarity.

9.8.2 Constructor & Destructor Documentation

9.8.2.1 CosineSim() [1/3]

```
LinkPred::CosineSim::CosineSim ( ) [inline]
```

Constructor.

9.8.2.2 CosineSim() [2/3]

```
LinkPred::CosineSim::CosineSim (
            CosineSim const & that ) [default]
```

Copy constructor.

Parameters

that	The object to copy.

9.8.2.3 CosineSim() [3/3]

```
LinkPred::CosineSim::CosineSim (
            CosineSim && that ) [default]
```

Move constructor.

Parameters

that	The object to move.

9.8.2.4 ∼CosineSim()

```
virtual LinkPred::CosineSim::~CosineSim ( ) [virtual], [default]
```

Destructor.

9.8.3 Member Function Documentation

9.8.3.1 operator=() [1/2]

```
CosineSim& LinkPred::CosineSim::operator= (
            CosineSim && that ) [default]
```

Move assignment operator.

Parameters

that	The object to move.

9.8.3.2 operator=() [2/2]

```
CosineSim& LinkPred::CosineSim::operator= (
            CosineSim const & that )  [default]
```

Copy assignment operator.

Parameters

that	The object to copy.

9.8.3.3 sim()

```
virtual double LinkPred::CosineSim::sim (
            Vec const & v1,
            Vec const & v2 )  [virtual]
```

Compute the similarity between two vectors.

Parameters

v1	First vector.
v2	Second vector. Must be of the same dimension as v1.

Returns

The similarity between v1 and v2.

Implements LinkPred::SimMeasure.

The documentation for this class was generated from the following file:

- include/linkpred/ml/simmeasures/cosinesim.hpp

9.9 LinkPred::Counter< Network > Class Template Reference

A class that counts nodes during traversal.

```
#include <graphtraversal.hpp>
```

Public Member Functions

- Counter ()=default
- Counter (Counter const &that)=default
- Counter & operator= (Counter const &that)=default
- Counter (Counter &&that)=default
- Counter & operator= (Counter &&that)=default
- bool process (typename Network::NodeID const &i)
- std::size_t getCount () const
- void resetCount ()
- virtual ~Counter ()=default

9.9.1 Detailed Description

template<typename Network = UNetwork<>>
class LinkPred::Counter< Network >

A class that counts nodes during traversal.

Template Parameters

Network	The network type.

9.9.2 Constructor & Destructor Documentation

9.9.2.1 Counter() [1/3]

```
template<typename Network = UNetwork<>>
LinkPred::Counter< Network >::Counter ( )  [default]
```

Constructor.

9.9.2.2 Counter() [2/3]

```
template<typename Network = UNetwork<>>
LinkPred::Counter< Network >::Counter (
            Counter< Network > const & that )  [default]
```

Copy constructor.

Parameters

that	The object to copy.

9.9.2.3 Counter() [3/3]

```
template<typename Network = UNetwork<>>
LinkPred::Counter< Network >::Counter (
            Counter< Network > && that )  [default]
```

Move constructor.

Parameters

that	The object to move.

9.9.2.4 ∼Counter()

```
template<typename Network = UNetwork<>>
virtual LinkPred::Counter< Network >::~Counter ( )  [virtual], [default]
```

Destructor.

9.9.3 Member Function Documentation

9.9.3.1 getCount()

```
template<typename Network = UNetwork<>>
std::size_t LinkPred::Counter< Network >::getCount ( ) const  [inline]
```

Returns

The nodes count.

9.9.3.2 operator=() [1/2]

```
template<typename Network = UNetwork<>>
Counter& LinkPred::Counter< Network >::operator= (
            Counter< Network > && that )  [default]
```

Move assignment operator.

Parameters

that	The object to move.

9.9.3.3 operator=() [2/2]

```
template<typename Network = UNetwork<>>
Counter& LinkPred::Counter< Network >::operator= (
            Counter< Network > const & that )  [default]
```

Copy assignment operator.

Parameters

that	The object to copy.

9.9.3.4 process()

```
template<typename Network = UNetwork<>>
bool LinkPred::Counter< Network >::process (
            typename Network::NodeID const & i )  [inline]
```

Node processing.

Parameters

i	The node's ID.

9.9.3.5 resetCount()

```
template<typename Network = UNetwork<>>
void LinkPred::Counter< Network >::resetCount ( )  [inline]
```

Reset he nodes count to 0.

The documentation for this class was generated from the following file:

- include/linkpred/graphalg/traversal/graphtraversal.hpp

9.10 LinkPred::DADAPredictor< Network, EdgeRndIt, ScoreRndIt, EdgeRndOutIt > Class Template Reference

Common neighbor link predictor adapted to directed networks.

```
#include <dadapredictor.hpp>
```

Inheritance diagram for LinkPred::DADAPredictor< Network, EdgeRndIt, ScoreRndIt, EdgeRndOutIt >:

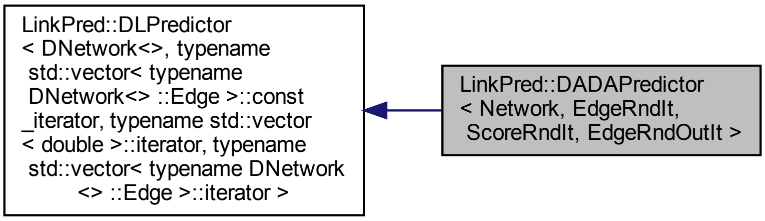

Collaboration diagram for LinkPred::DADAPredictor< Network, EdgeRndIt, ScoreRndIt, EdgeRndOutIt >:

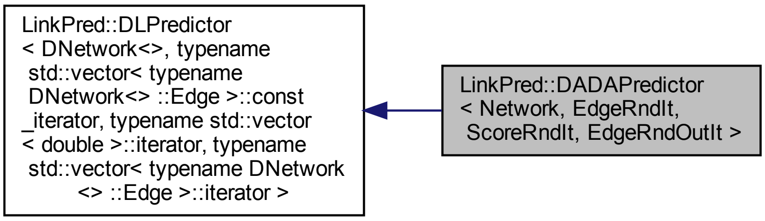

Public Member Functions

- DADAPredictor (std::shared_ptr< Network const > net)
- DADAPredictor (DADAPredictor const &that)=default
- DADAPredictor & operator= (DADAPredictor const &that)=default
- DADAPredictor (DADAPredictor &&that)=default
- DADAPredictor & operator= (DADAPredictor &&that)=default
- virtual void init ()
- virtual void learn ()
- virtual void predict (EdgeRndIt begin, EdgeRndIt end, ScoreRndIt scores)
- virtual double score (Edge const &e)
- virtual std::size_t top (std::size_t k, EdgeRndOutIt eit, ScoreRndIt sit)
- virtual ~DADAPredictor ()=default

Additional Inherited Members

9.10.1 Detailed Description

template<typename Network = DNetwork<>, typename EdgeRndIt = typename std::vector<typename Network::Edge>↵ ::const_iterator, typename ScoreRndIt = typename std::vector<double>::iterator, typename EdgeRndOutIt = typename std↵ ::vector<typename Network::Edge>::iterator>
class LinkPred::DADAPredictor< Network, EdgeRndIt, ScoreRndIt, EdgeRndOutIt >

Common neighbor link predictor adapted to directed networks.

Template Parameters

Network	The network type.
Edge↵ RndIt	A random iterator type used to iterate on edges.
Score↵ RndIt	A random iterator type used to iterate on scores.

9.10.2 Constructor & Destructor Documentation

9.10.2.1 DADAPredictor() [1/3]

```
template<typename Network = DNetwork<>, typename EdgeRndIt = typename std::vector<typename
Network::Edge>::const_iterator, typename ScoreRndIt = typename std::vector<double>::iterator,
typename EdgeRndOutIt = typename std::vector<typename Network::Edge>::iterator>
LinkPred::DADAPredictor< Network, EdgeRndIt, ScoreRndIt, EdgeRndOutIt >::DADAPredictor (
            std::shared_ptr< Network const > net )  [inline]
```

Parameters

net	The network.

9.10.2.2 DADAPredictor() [2/3]

```
template<typename Network = DNetwork<>, typename EdgeRndIt = typename std::vector<typename
Network::Edge>::const_iterator, typename ScoreRndIt = typename std::vector<double>::iterator,
typename EdgeRndOutIt = typename std::vector<typename Network::Edge>::iterator>
LinkPred::DADAPredictor< Network, EdgeRndIt, ScoreRndIt, EdgeRndOutIt >::DADAPredictor (
            DADAPredictor< Network, EdgeRndIt, ScoreRndIt, EdgeRndOutIt > const & that )
[default]
```

Copy constructor.

Parameters

that	The object to copy.

9.10.2.3 DADAPredictor() [3/3]

```
template<typename Network = DNetwork<>, typename EdgeRndIt = typename std::vector<typename
Network::Edge>::const_iterator, typename ScoreRndIt = typename std::vector<double>::iterator,
typename EdgeRndOutIt = typename std::vector<typename Network::Edge>::iterator>
LinkPred::DADAPredictor< Network, EdgeRndIt, ScoreRndIt, EdgeRndOutIt >::DADAPredictor (
            DADAPredictor< Network, EdgeRndIt, ScoreRndIt, EdgeRndOutIt > && that )  [default]
```

Move constructor.

Parameters

that	The object to move.

9.10.2.4 ∼DADAPredictor()

```
template<typename Network = DNetwork<>, typename EdgeRndIt = typename std::vector<typename
Network::Edge>::const_iterator, typename ScoreRndIt = typename std::vector<double>::iterator,
typename EdgeRndOutIt = typename std::vector<typename Network::Edge>::iterator>
virtual LinkPred::DADAPredictor< Network, EdgeRndIt, ScoreRndIt, EdgeRndOutIt >::∼DADAPredictor
( )  [virtual], [default]
```

Destructor.

9.10.3 Member Function Documentation

9.10.3.1 init()

```
template<typename Network = DNetwork<>, typename EdgeRndIt = typename std::vector<typename
Network::Edge>::const_iterator, typename ScoreRndIt = typename std::vector<double>::iterator,
typename EdgeRndOutIt = typename std::vector<typename Network::Edge>::iterator>
virtual void LinkPred::DADAPredictor< Network, EdgeRndIt, ScoreRndIt, EdgeRndOutIt >::init ( )
[inline], [virtual]
```

Initialize the solver.

Implements LinkPred::DLPredictor< DNetwork<>, typename std::vector< typename DNetwork<> ::Edge >::const_iterator, typena

9.10.3.2 learn()

```
template<typename Network = DNetwork<>, typename EdgeRndIt = typename std::vector<typename
Network::Edge>::const_iterator, typename ScoreRndIt = typename std::vector<double>::iterator,
typename EdgeRndOutIt = typename std::vector<typename Network::Edge>::iterator>
virtual void LinkPred::DADAPredictor< Network, EdgeRndIt, ScoreRndIt, EdgeRndOutIt >::learn (
) [inline], [virtual]
```

Learn.

Implements LinkPred::DLPredictor< DNetwork<>, typename std::vector< typename DNetwork<> ::Edge >::const_iterator, typena [illegible]

9.10.3.3 operator=() [1/2]

```
template<typename Network = DNetwork<>, typename EdgeRndIt = typename std::vector<typename
Network::Edge>::const_iterator, typename ScoreRndIt = typename std::vector<double>::iterator,
typename EdgeRndOutIt = typename std::vector<typename Network::Edge>::iterator>
DADAPredictor& LinkPred::DADAPredictor< Network, EdgeRndIt, ScoreRndIt, EdgeRndOutIt >::operator=
(
            DADAPredictor< Network, EdgeRndIt, ScoreRndIt, EdgeRndOutIt > && that ) [default]
```

Move assignment operator.

Parameters

that	The object to move.

9.10.3.4 operator=() [2/2]

```
template<typename Network = DNetwork<>, typename EdgeRndIt = typename std::vector<typename
Network::Edge>::const_iterator, typename ScoreRndIt = typename std::vector<double>::iterator,
typename EdgeRndOutIt = typename std::vector<typename Network::Edge>::iterator>
DADAPredictor& LinkPred::DADAPredictor< Network, EdgeRndIt, ScoreRndIt, EdgeRndOutIt >::operator=
(
            DADAPredictor< Network, EdgeRndIt, ScoreRndIt, EdgeRndOutIt > const & that )
[default]
```

Copy assignment operator.

Parameters

that	The object to copy.

9.10.3.5 predict()

```
template<typename Network = DNetwork<>, typename EdgeRndIt = typename std::vector<typename
Network::Edge>::const_iterator, typename ScoreRndIt = typename std::vector<double>::iterator,
typename EdgeRndOutIt = typename std::vector<typename Network::Edge>::iterator>
virtual void LinkPred::DADAPredictor< Network, EdgeRndIt, ScoreRndIt, EdgeRndOutIt >::predict
(
            EdgeRndIt begin,
            EdgeRndIt end,
            ScoreRndIt scores )  [virtual]
```

Predict the links.

Parameters

begin	Beginning of the links to be predicted.
end	end of the links to be predicted.
scores	Beginning of scores.

Reimplemented from LinkPred::DLPredictor< DNetwork<>, typename std::vector< typename DNetwork<> ::Edge >::const_iterat[illegible]

9.10.3.6 score()

```
template<typename Network = DNetwork<>, typename EdgeRndIt = typename std::vector<typename
Network::Edge>::const_iterator, typename ScoreRndIt = typename std::vector<double>::iterator,
typename EdgeRndOutIt = typename std::vector<typename Network::Edge>::iterator>
virtual double LinkPred::DADAPredictor< Network, EdgeRndIt, ScoreRndIt, EdgeRndOutIt >::score
(
            Edge const & e )  [virtual]
```

Compute the score of a single edge.

Parameters

e	The edge.

Returns

The score of e.

9.10.3.7 top()

```
template<typename Network = DNetwork<>, typename EdgeRndIt = typename std::vector<typename
Network::Edge>::const_iterator, typename ScoreRndIt = typename std::vector<double>::iterator,
typename EdgeRndOutIt = typename std::vector<typename Network::Edge>::iterator>
```

```
virtual std::size_t LinkPred::DADAPredictor< Network, EdgeRndIt, ScoreRndIt, EdgeRndOutIt >↩
::top (
            std::size_t k,
            EdgeRndOutIt eit,
            ScoreRndIt sit )  [virtual]
```

Finds the k negative edges with the top score. Ties are broken randomly.

Parameters

k	The number of edges to find.
eit	An output iterator where the edges are written.
sit	An output iterator where the scores are written. The scores are written in the same order as the edges.

Returns

The number of negative edges inserted. It is the minimum between k and the number of negative edges in the network.

Reimplemented from LinkPred::DLPredictor< DNetwork<>, typename std::vector< typename DNetwork<> ::Edge >::const_iterato

The documentation for this class was generated from the following file:

- include/linkpred/predictors/directed/dadapredictor.hpp

9.11 LinkPred::DCNEPredictor< Network, EdgeRndIt, ScoreRndIt, EdgeRndOutIt > Class Template Reference

Common neighbor link predictor adapted to directed networks.

```
#include <dcnepredictor.hpp>
```

Inheritance diagram for LinkPred::DCNEPredictor< Network, EdgeRndIt, ScoreRndIt, EdgeRndOutIt >:

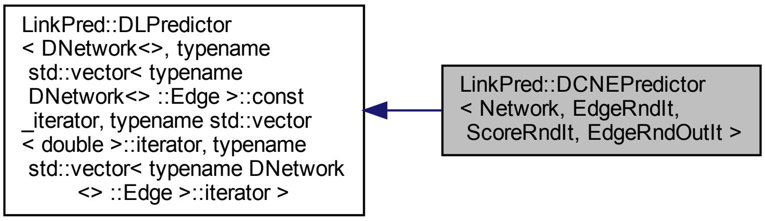

Collaboration diagram for LinkPred::DCNEPredictor< Network, EdgeRndIt, ScoreRndIt, EdgeRndOutIt >:

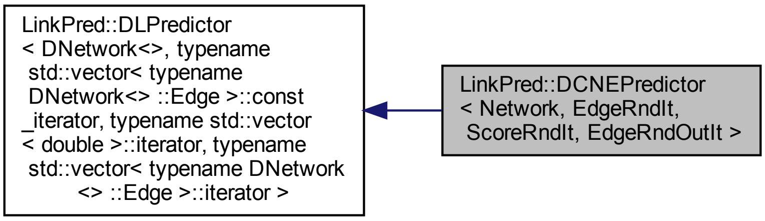

Public Member Functions

- DCNEPredictor (std::shared_ptr< Network const > net)
- DCNEPredictor (DCNEPredictor const &that)=default
- DCNEPredictor & operator= (DCNEPredictor const &that)=default
- DCNEPredictor (DCNEPredictor &&that)=default
- DCNEPredictor & operator= (DCNEPredictor &&that)=default
- virtual void init ()
- virtual void learn ()
- virtual void predict (EdgeRndIt begin, EdgeRndIt end, ScoreRndIt scores)
- virtual double score (Edge const &e)
- virtual std::size_t top (std::size_t k, EdgeRndOutIt eit, ScoreRndIt sit)
- virtual ~DCNEPredictor ()=default

Additional Inherited Members

9.11.1 Detailed Description

template<typename Network = DNetwork<>, typename EdgeRndIt = typename std::vector<typename Network::Edge>::const_iterator, typename ScoreRndIt = typename std::vector<double>::iterator, typename EdgeRndOutIt = typename std::vector<typename Network::Edge>::iterator>
class LinkPred::DCNEPredictor< Network, EdgeRndIt, ScoreRndIt, EdgeRndOutIt >

Common neighbor link predictor adapted to directed networks.

Template Parameters

Network	The network type.
EdgeRndIt	A random iterator type used to iterate on edges.
ScoreRndIt	A random iterator type used to iterate on scores.

9.11.2 Constructor & Destructor Documentation

9.11.2.1 DCNEPredictor() [1/3]

```
template<typename Network = DNetwork<>, typename EdgeRndIt = typename std::vector<typename
Network::Edge>::const_iterator, typename ScoreRndIt = typename std::vector<double>::iterator,
typename EdgeRndOutIt = typename std::vector<typename Network::Edge>::iterator>
LinkPred::DCNEPredictor< Network, EdgeRndIt, ScoreRndIt, EdgeRndOutIt >::DCNEPredictor (
            std::shared_ptr< Network const > net )  [inline]
```

Parameters

net	The network.

9.11.2.2 DCNEPredictor() [2/3]

```
template<typename Network = DNetwork<>, typename EdgeRndIt = typename std::vector<typename
Network::Edge>::const_iterator, typename ScoreRndIt = typename std::vector<double>::iterator,
typename EdgeRndOutIt = typename std::vector<typename Network::Edge>::iterator>
LinkPred::DCNEPredictor< Network, EdgeRndIt, ScoreRndIt, EdgeRndOutIt >::DCNEPredictor (
            DCNEPredictor< Network, EdgeRndIt, ScoreRndIt, EdgeRndOutIt > const & that )
[default]
```

Copy constructor.

Parameters

that	The object to copy.

9.11.2.3 DCNEPredictor() [3/3]

```
template<typename Network = DNetwork<>, typename EdgeRndIt = typename std::vector<typename
Network::Edge>::const_iterator, typename ScoreRndIt = typename std::vector<double>::iterator,
typename EdgeRndOutIt = typename std::vector<typename Network::Edge>::iterator>
LinkPred::DCNEPredictor< Network, EdgeRndIt, ScoreRndIt, EdgeRndOutIt >::DCNEPredictor (
            DCNEPredictor< Network, EdgeRndIt, ScoreRndIt, EdgeRndOutIt > && that )  [default]
```

Move constructor.

Parameters

that	The object to move.

9.11.2.4 ∼DCNEPredictor()

```
template<typename Network = DNetwork<>, typename EdgeRndIt = typename std::vector<typename
Network::Edge>::const_iterator, typename ScoreRndIt = typename std::vector<double>::iterator,
typename EdgeRndOutIt = typename std::vector<typename Network::Edge>::iterator>
virtual LinkPred::DCNEPredictor< Network, EdgeRndIt, ScoreRndIt, EdgeRndOutIt >::∼DCNEPredictor
( ) [virtual], [default]
```

Destructor.

9.11.3 Member Function Documentation

9.11.3.1 init()

```
template<typename Network = DNetwork<>, typename EdgeRndIt = typename std::vector<typename
Network::Edge>::const_iterator, typename ScoreRndIt = typename std::vector<double>::iterator,
typename EdgeRndOutIt = typename std::vector<typename Network::Edge>::iterator>
virtual void LinkPred::DCNEPredictor< Network, EdgeRndIt, ScoreRndIt, EdgeRndOutIt >::init ( )
[inline], [virtual]
```

Initialize the solver.

Implements LinkPred::DLPredictor< DNetwork<>, typename std::vector< typename DNetwork<> ::Edge >::const_iterator, typena

9.11.3.2 learn()

```
template<typename Network = DNetwork<>, typename EdgeRndIt = typename std::vector<typename
Network::Edge>::const_iterator, typename ScoreRndIt = typename std::vector<double>::iterator,
typename EdgeRndOutIt = typename std::vector<typename Network::Edge>::iterator>
virtual void LinkPred::DCNEPredictor< Network, EdgeRndIt, ScoreRndIt, EdgeRndOutIt >::learn (
) [inline], [virtual]
```

Learn.

Implements LinkPred::DLPredictor< DNetwork<>, typename std::vector< typename DNetwork<> ::Edge >::const_iterator, typena

9.11.3.3 operator=() [1/2]

```
template<typename Network = DNetwork<>, typename EdgeRndIt = typename std::vector<typename
Network::Edge>::const_iterator, typename ScoreRndIt = typename std::vector<double>::iterator,
typename EdgeRndOutIt = typename std::vector<typename Network::Edge>::iterator>
DCNEPredictor& LinkPred::DCNEPredictor< Network, EdgeRndIt, ScoreRndIt, EdgeRndOutIt >::operator=
(
            DCNEPredictor< Network, EdgeRndIt, ScoreRndIt, EdgeRndOutIt > && that ) [default]
```

Move assignment operator.

Parameters

that	The object to move.

9.11.3.4 operator=() [2/2]

```
template<typename Network = DNetwork<>, typename EdgeRndIt = typename std::vector<typename
Network::Edge>::const_iterator, typename ScoreRndIt = typename std::vector<double>::iterator,
typename EdgeRndOutIt = typename std::vector<typename Network::Edge>::iterator>
DCNEPredictor& LinkPred::DCNEPredictor< Network, EdgeRndIt, ScoreRndIt, EdgeRndOutIt >::operator=
(
            DCNEPredictor< Network, EdgeRndIt, ScoreRndIt, EdgeRndOutIt > const & that )
[default]
```

Copy assignment operator.

Parameters

that	The object to copy.

9.11.3.5 predict()

```
template<typename Network = DNetwork<>, typename EdgeRndIt = typename std::vector<typename
Network::Edge>::const_iterator, typename ScoreRndIt = typename std::vector<double>::iterator,
typename EdgeRndOutIt = typename std::vector<typename Network::Edge>::iterator>
virtual void LinkPred::DCNEPredictor< Network, EdgeRndIt, ScoreRndIt, EdgeRndOutIt >::predict
(
            EdgeRndIt begin,
            EdgeRndIt end,
            ScoreRndIt scores )  [virtual]
```

Predict the links.

Parameters

begin	Beginning of the links to be predicted.
end	end of the links to be predicted.
scores	Beginning of scores.

Reimplemented from LinkPred::DLPredictor< DNetwork<>, typename std::vector< typename DNetwork<> ::Edge >::const_iterat

9.11.3.6 score()

```
template<typename Network = DNetwork<>, typename EdgeRndIt = typename std::vector<typename
Network::Edge>::const_iterator, typename ScoreRndIt = typename std::vector<double>::iterator,
```

```
typename EdgeRndOutIt = typename std::vector<typename Network::Edge>::iterator>
virtual double LinkPred::DCNEPredictor< Network, EdgeRndIt, ScoreRndIt, EdgeRndOutIt >::score
(
            Edge const & e )  [virtual]
```

Compute the score of a single edge.

Parameters

e	The edge.

Returns

The score of e.

9.11.3.7 top()

```
template<typename Network = DNetwork<>, typename EdgeRndIt = typename std::vector<typename
Network::Edge>::const_iterator, typename ScoreRndIt = typename std::vector<double>::iterator,
typename EdgeRndOutIt = typename std::vector<typename Network::Edge>::iterator>
virtual std::size_t LinkPred::DCNEPredictor< Network, EdgeRndIt, ScoreRndIt, EdgeRndOutIt >↩
::top (
            std::size_t k,
            EdgeRndOutIt eit,
            ScoreRndIt sit )  [virtual]
```

Finds the k negative edges with the top score. Ties are broken randomly.

Parameters

k	The number of edges to find.
eit	An output iterator where the edges are written.
sit	An output iterator where the scores are written. The scores are written in the same order as the edges.

Returns

The number of negative edges inserted. It is the minimum between k and the number of negative edges in the network.

Reimplemented from LinkPred::DLPredictor< DNetwork<>, typename std::vector< typename DNetwork<> ::Edge >::const_iterat

The documentation for this class was generated from the following file:

- include/linkpred/predictors/directed/dcnepredictor.hpp

9.12 LinkPred::DeepWalk< Network > Class Template Reference

DeepWalk encoder. Reference: Perozzi, B., Al-Rfou, R., and Skiena, S. (2014). Deepwalk: Online learning of social representations. In Proceedings of the 20th ACM SIGKDD International Conference on Knowledge Discovery and Data Mining, KDD '14, pages 701–710, New York, NY, USA. Association for Computing Machinery. This implementation is based on the code https://github.com/xgfs/deepwalk-c.

```
#include <deepwalk.hpp>
```

Inheritance diagram for LinkPred::DeepWalk< Network >:

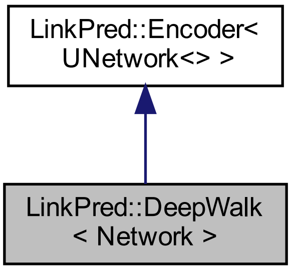

Collaboration diagram for LinkPred::DeepWalk< Network >:

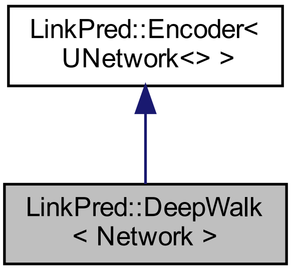

Public Member Functions

- DeepWalk (std::shared_ptr< Network const > net, long int seed)
- DeepWalk (DeepWalk const &that)=default
- DeepWalk & operator= (DeepWalk const &that)=default
- DeepWalk (DeepWalk &&that)=default
- DeepWalk & operator= (DeepWalk &&that)=default
- virtual void init ()

- virtual void encode ()
- float getInitLR () const
- void setInitLR (float initLR)
- int getNbWalks () const
- void setNbWalks (int nbWalks)
- int getWalkLength () const
- void setWalkLength (int walkLength)
- int getWindowSize () const
- void setWindowSize (int windowSize)
- virtual void setWeightMap (const WeightMapSP &weightMap)
- float getAlpha () const
- void setAlpha (float alpha)
- const int getMaxCodeLength () const
- int getNbNodeWalks () const
- void setNbNodeWalks (int nbNodeWalks)
- long long getStepInterval () const
- void setStepInterval (long long stepInterval)
- long long getTotalSteps () const
- const int getDefaultDim () const
- virtual ∼DeepWalk ()=default

Additional Inherited Members

9.12.1 Detailed Description

template<typename Network = UNetwork<>>
class LinkPred::DeepWalk< Network >

DeepWalk encoder. Reference: Perozzi, B., Al-Rfou, R., and Skiena, S. (2014). Deepwalk: Online learning of social representations. In Proceedings of the 20th ACM SIGKDD International Conference on Knowledge Discovery and Data Mining, KDD '14, pages 701–710, New York, NY, USA. Association for Computing Machinery. This implementation is based on the code https://github.com/xgfs/deepwalk-c.

Template Parameters

Network	The network type.

9.12.2 Constructor & Destructor Documentation

9.12.2.1 DeepWalk() [1/3]

```
template<typename Network = UNetwork<>>
LinkPred::DeepWalk< Network >::DeepWalk (
            std::shared_ptr< Network const > net,
            long int seed ) [inline]
```

Constructor.

Parameters

net	The network.
seed	Random number generator seed.

9.12.2.2 DeepWalk() [2/3]

```
template<typename Network = UNetwork<>>
LinkPred::DeepWalk< Network >::DeepWalk (
            DeepWalk< Network > const & that )  [default]
```

Copy constructor.

Parameters

that	The object to copy.

9.12.2.3 DeepWalk() [3/3]

```
template<typename Network = UNetwork<>>
LinkPred::DeepWalk< Network >::DeepWalk (
            DeepWalk< Network > && that )  [default]
```

Move constructor.

Parameters

that	The object to move.

9.12.2.4 ∼DeepWalk()

```
template<typename Network = UNetwork<>>
virtual LinkPred::DeepWalk< Network >::∼DeepWalk ( )  [virtual], [default]
```

Destructor.

9.12.3 Member Function Documentation

9.12.3.1 encode()

```
template<typename Network = UNetwork<>>
virtual void LinkPred::DeepWalk< Network >::encode ( )  [virtual]
```

Encode the network.

Implements LinkPred::Encoder< UNetwork<> >.

9.12.3.2 getAlpha()

```
template<typename Network = UNetwork<>>
float LinkPred::DeepWalk< Network >::getAlpha ( ) const  [inline]
```

Returns

The probability of continuing random walk in initialization.

9.12.3.3 getDefaultDim()

```
template<typename Network = UNetwork<>>
const int LinkPred::DeepWalk< Network >::getDefaultDim ( ) const  [inline]
```

Returns

Default embedding dimension.

9.12.3.4 getInitLR()

```
template<typename Network = UNetwork<>>
float LinkPred::DeepWalk< Network >::getInitLR ( ) const  [inline]
```

Returns

Initial learning rate.

9.12.3.5 getMaxCodeLength()

```
template<typename Network = UNetwork<>>
const int LinkPred::DeepWalk< Network >::getMaxCodeLength ( ) const [inline]
```

Returns

Maximum code length.

9.12.3.6 getNbNodeWalks()

```
template<typename Network = UNetwork<>>
int LinkPred::DeepWalk< Network >::getNbNodeWalks ( ) const [inline]
```

Returns

The number of walks per node in the PageRank initialization.

9.12.3.7 getNbWalks()

```
template<typename Network = UNetwork<>>
int LinkPred::DeepWalk< Network >::getNbWalks ( ) const [inline]
```

Returns

Number of walks per vertex ("\gamma").

9.12.3.8 getStepInterval()

```
template<typename Network = UNetwork<>>
long long LinkPred::DeepWalk< Network >::getStepInterval ( ) const [inline]
```

Returns

The number of steps after which the learning rate is updated.

9.12.3.9 getTotalSteps()

```
template<typename Network = UNetwork<>>
long long LinkPred::DeepWalk< Network >::getTotalSteps ( ) const [inline]
```

Returns

The total number of steps.

9.12.3.10 getWalkLength()

```
template<typename Network = UNetwork<>>
int LinkPred::DeepWalk< Network >::getWalkLength ( ) const [inline]
```

Returns

DeepWalk parameter "t" = length of the walk.

9.12.3.11 getWindowSize()

```
template<typename Network = UNetwork<>>
int LinkPred::DeepWalk< Network >::getWindowSize ( ) const [inline]
```

Returns

DeepWalk parameter "w" = window size.

9.12.3.12 init()

```
template<typename Network = UNetwork<>>
virtual void LinkPred::DeepWalk< Network >::init ( ) [virtual]
```

Initialize encoder.

Implements LinkPred::Encoder< UNetwork<> >.

9.12.3.13 operator=() [1/2]

```
template<typename Network = UNetwork<>>
DeepWalk& LinkPred::DeepWalk< Network >::operator= (
            DeepWalk< Network > && that ) [default]
```

Move assignment operator.

Parameters

that	The object to move.

9.12.3.14 operator=() [2/2]

```
template<typename Network = UNetwork<>>
DeepWalk& LinkPred::DeepWalk< Network >::operator= (
            DeepWalk< Network > const & that ) [default]
```

Copy assignment operator.

Parameters

that	The object to copy.

9.12.3.15 setAlpha()

```
template<typename Network = UNetwork<>>
void LinkPred::DeepWalk< Network >::setAlpha (
            float alpha ) [inline]
```

Set the probability of continuing random walk in initialization.

Parameters

alpha	Probability of continuing random walk in initialization.

9.12.3.16 setInitLR()

```
template<typename Network = UNetwork<>>
void LinkPred::DeepWalk< Network >::setInitLR (
            float initLR ) [inline]
```

Set the initial learning rate.

Parameters

initLR	Initial learning rate.

9.12.3.17 setNbNodeWalks()

```
template<typename Network = UNetwork<>>
void LinkPred::DeepWalk< Network >::setNbNodeWalks (
            int nbNodeWalks )  [inline]
```

Set the number of walks per node in the PageRank initialization.

Parameters

nbNodeWalks	The number of walks per node in the PageRank initialization.

9.12.3.18 setNbWalks()

```
template<typename Network = UNetwork<>>
void LinkPred::DeepWalk< Network >::setNbWalks (
            int nbWalks )  [inline]
```

Set the number of walks per vertex ("\gamma").

Parameters

nbWalks	Number of walks per vertex ("\gamma").

9.12.3.19 setStepInterval()

```
template<typename Network = UNetwork<>>
void LinkPred::DeepWalk< Network >::setStepInterval (
            long long stepInterval )  [inline]
```

Set the number of steps after which the learning rate is updated.

Parameters

stepInterval	Number of steps after which the learning rate is updated.

9.12.3.20 setWalkLength()

```
template<typename Network = UNetwork<>>
void LinkPred::DeepWalk< Network >::setWalkLength (
            int walkLength )  [inline]
```

Set the DeepWalk parameter "t" = length of the walk.

Parameters

walkLength	DeepWalk parameter "t" = length of the walk.

9.12.3.21 setWeightMap()

```
template<typename Network = UNetwork<>>
virtual void LinkPred::DeepWalk< Network >::setWeightMap (
            const WeightMapSP & weightMap ) [inline], [virtual]
```

Set edge weight map.

Reimplemented from LinkPred::Encoder< UNetwork<> >.

9.12.3.22 setWindowSize()

```
template<typename Network = UNetwork<>>
void LinkPred::DeepWalk< Network >::setWindowSize (
            int windowSize ) [inline]
```

Set the DeepWalk parameter "w" = window size.

Parameters

windowSize	DeepWalk parameter "w" = window size.

The documentation for this class was generated from the following file:

- include/linkpred/graphalg/encoders/deepwalk/deepwalk.hpp

9.13 LinkPred::DESPLDistCalculator< Network, DistType, NbHopsType > Class Template Reference

Exact shortest path distance calculator on a directed network with limits on the number of hops.

```
#include <netdistcalculator.hpp>
```

Inheritance diagram for LinkPred::DESPLDistCalculator< Network, DistType, NbHopsType >:

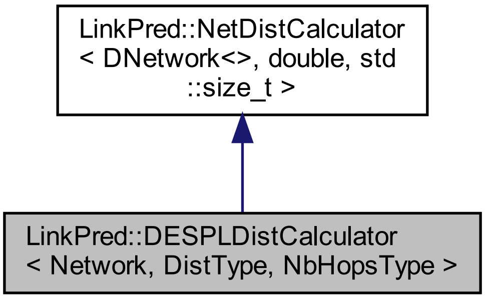

Collaboration diagram for LinkPred::DESPLDistCalculator< Network, DistType, NbHopsType >:

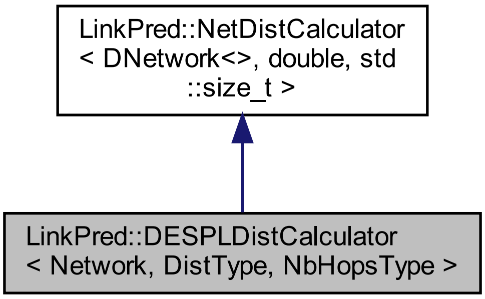

Public Types

- using LengthMapIdType = typename NetDistCalculator< Network, DistType, NbHopsType >↵ ::LengthMapIdType
- using NetworkSP = typename NetDistCalculator< Network, DistType, NbHopsType >::NetworkSP
- using NodeID = typename NetDistCalculator< Network, DistType, NbHopsType >::NodeID
- using Label = typename NetDistCalculator< Network, DistType, NbHopsType >::Label
- using Edge = typename NetDistCalculator< Network, DistType, NbHopsType >::Edge
- using NodeDistMap = typename NetDistCalculator< Network, DistType, NbHopsType >::NodeDistMap
- using NodeDistMapSP = typename NetDistCalculator< Network, DistType, NbHopsType >::NodeDistMapSP
- using NodeSDistMap = typename Network::template NodeSMap< std::pair< DistType, NbHopsType > >
- using NodeSDistMapSP = typename Network::template NodeSMapSP< std::pair< DistType, NbHopsType > >
- using EdgeLengthMap = typename NetDistCalculator< Network, DistType, NbHopsType >::EdgeLengthMap
- using EdgeLengthMapSP = typename NetDistCalculator< Network, DistType, NbHopsType >↵ ::EdgeLengthMapSP

Public Member Functions

- DESPLDistCalculator (Dijkstra< Network, DistType, NbHopsType > &dijkstra, EdgeLengthMapSP length, std::size_t lim, CacheLevel cacheLevel=CacheLevel::NetworkCache)
- DESPLDistCalculator (DESPLDistCalculator const &that)=default
- DESPLDistCalculator & operator= (DESPLDistCalculator const &that)=default
- DESPLDistCalculator (DESPLDistCalculator &&that)=default
- DESPLDistCalculator & operator= (DESPLDistCalculator &&that)=default
- virtual std::pair< DistType, NbHopsType > getDist (NodeID const &i, NodeID const &j)
- virtual std::pair< DistType, NbHopsType > getIndDist (NodeID const &i, NodeID const &j)
- virtual NodeDistMapSP getDist (NodeID const &i)
- virtual NodeSDistMapSP getSDistMap (NodeID const &i)
- virtual NodeSDistMapSP getFinDistMapNoNeighb (NodeID const &srcNode)
- virtual std::pair< DistType, NbHopsType > getDist (Edge const &e)
- std::size_t getMaxNbNodesInCache () const
- void setMaxNbNodesInCache (std::size_t maxNbNodesInCache)
- std::size_t getLim () const
- virtual ∼ DESPLDistCalculator ()

9.13.1 Detailed Description

template<typename Network = DNetwork<>, typename DistType = double, typename NbHopsType = std::size_t>
class LinkPred::DESPLDistCalculator< Network, DistType, NbHopsType >

Exact shortest path distance calculator on a directed network with limits on the number of hops.

This class offers an additional layer over Dijkstra which provides memory management functionalities by caching computed distances.

Template Parameters

Network	The network type.
DistType	The distance type (can be an integer or floating point type).
NbHopsType	The type of the number of hops (must be an integer type).

9.13.2 Member Typedef Documentation

9.13.2.1 Edge

```
template<typename Network = DNetwork<>, typename DistType = double, typename NbHopsType =
std::size_t>
using LinkPred::DESPLDistCalculator< Network, DistType, NbHopsType >::Edge = typename NetDistCalculator<Netwo
DistType, NbHopsType>::Edge
```

Edge type.

9.13.2.2 EdgeLengthMap

```
template<typename Network = DNetwork<>, typename DistType = double, typename NbHopsType =
std::size_t>
using LinkPred::DESPLDistCalculator< Network, DistType, NbHopsType >::EdgeLengthMap = typename
NetDistCalculator<Network, DistType, NbHopsType>::EdgeLengthMap
```

Edge length map.

9.13.2.3 EdgeLengthMapSP

```
template<typename Network = DNetwork<>, typename DistType = double, typename NbHopsType =
std::size_t>
using LinkPred::DESPLDistCalculator< Network, DistType, NbHopsType >::EdgeLengthMapSP = typename
NetDistCalculator<Network, DistType, NbHopsType>::EdgeLengthMapSP
```

Shared pointer to an edge length map.

9.13.2.4 Label

```
template<typename Network = DNetwork<>, typename DistType = double, typename NbHopsType =
std::size_t>
using LinkPred::DESPLDistCalculator< Network, DistType, NbHopsType >::Label = typename NetDistCalculator<Netw[illegible]
DistType, NbHopsType>::Label
```

Nodes label type.

9.13.2.5 LengthMapIdType

```
template<typename Network = DNetwork<>, typename DistType = double, typename NbHopsType =
std::size_t>
using LinkPred::DESPLDistCalculator< Network, DistType, NbHopsType >::LengthMapIdType = typename
NetDistCalculator<Network, DistType, NbHopsType>::LengthMapIdType
```

Length map ID type.

9.13.2.6 NetworkSP

```
template<typename Network = DNetwork<>, typename DistType = double, typename NbHopsType =
std::size_t>
using LinkPred::DESPLDistCalculator< Network, DistType, NbHopsType >::NetworkSP = typename
NetDistCalculator<Network, DistType, NbHopsType>::NetworkSP
```

Shared pointer to network.

9.13.2.7 NodeDistMap

```
template<typename Network = DNetwork<>, typename DistType = double, typename NbHopsType =
std::size_t>
using LinkPred::DESPLDistCalculator< Network, DistType, NbHopsType >::NodeDistMap = typename
NetDistCalculator<Network, DistType, NbHopsType>::NodeDistMap
```

Distance map.

9.13.2.8 NodeDistMapSP

```
template<typename Network = DNetwork<>, typename DistType = double, typename NbHopsType =
std::size_t>
using LinkPred::DESPLDistCalculator< Network, DistType, NbHopsType >::NodeDistMapSP = typename
NetDistCalculator<Network, DistType, NbHopsType>::NodeDistMapSP
```

Shared pointer to a distance map.

9.13.2.9 NodeID

```
template<typename Network = DNetwork<>, typename DistType = double, typename NbHopsType =
std::size_t>
using LinkPred::DESPLDistCalculator< Network, DistType, NbHopsType >::NodeID = typename NetDistCalculator<Net
DistType, NbHopsType>::NodeID
```

Nodes ID type.

9.13.2.10 NodeSDistMap

```
template<typename Network = DNetwork<>, typename DistType = double, typename NbHopsType =
std::size_t>
using LinkPred::DESPLDistCalculator< Network, DistType, NbHopsType >::NodeSDistMap = typename
Network::template NodeSMap<std::pair<DistType, NbHopsType> >
```

Distance map.

9.13.2.11 NodeSDistMapSP

```
template<typename Network = DNetwork<>, typename DistType = double, typename NbHopsType =
std::size_t>
using LinkPred::DESPLDistCalculator< Network, DistType, NbHopsType >::NodeSDistMapSP = typename
Network::template NodeSMapSP<std::pair<DistType, NbHopsType> >
```

Shared pointer to a distance map.

9.13.3 Constructor & Destructor Documentation

9.13.3.1 DESPLDistCalculator() [1/3]

```
template<typename Network = DNetwork<>, typename DistType = double, typename NbHopsType =
std::size_t>
LinkPred::DESPLDistCalculator< Network, DistType, NbHopsType >::DESPLDistCalculator (
            Dijkstra< Network, DistType, NbHopsType > & dijkstra,
            EdgeLengthMapSP length,
            std::size_t lim,
            CacheLevel cacheLevel = CacheLevel::NetworkCache ) [inline]
```

Constructor.

Parameters

dijkstra	A Dijkstra algorithm object.
lim	Horizon limit.
length	The length map.
cacheLevel	The cache level.

9.13.3.2 DESPLDistCalculator() [2/3]

```
template<typename Network = DNetwork<>, typename DistType = double, typename NbHopsType =
std::size_t>
LinkPred::DESPLDistCalculator< Network, DistType, NbHopsType >::DESPLDistCalculator (
            DESPLDistCalculator< Network, DistType, NbHopsType > const & that )  [default]
```

Copy constructor.

Parameters

that	The object to copy.

9.13.3.3 DESPLDistCalculator() [3/3]

```
template<typename Network = DNetwork<>, typename DistType = double, typename NbHopsType =
std::size_t>
LinkPred::DESPLDistCalculator< Network, DistType, NbHopsType >::DESPLDistCalculator (
            DESPLDistCalculator< Network, DistType, NbHopsType > && that )  [default]
```

Move constructor.

Parameters

that	The object to move.

9.13.3.4 ∼ DESPLDistCalculator()

```
template<typename Network = DNetwork<>, typename DistType = double, typename NbHopsType =
std::size_t>
virtual LinkPred::DESPLDistCalculator< Network, DistType, NbHopsType >::∼ DESPLDistCalculator
( )  [inline], [virtual]
```

Destructor.

9.13.4 Member Function Documentation

9.13.4.1 getDist() [1/3]

```
template<typename Network = DNetwork<>, typename DistType = double, typename NbHopsType =
std::size_t>
virtual std::pair<DistType, NbHopsType> LinkPred::DESPLDistCalculator< Network, DistType,
NbHopsType >::getDist (
            Edge const & e ) [inline], [virtual]
```

Parameters

e	An input edge.

Returns

The distance between start and end of e.

9.13.4.2 getDist() [2/3]

```
template<typename Network = DNetwork<>, typename DistType = double, typename NbHopsType =
std::size_t>
virtual NodeDistMapSP LinkPred::DESPLDistCalculator< Network, DistType, NbHopsType >::getDist
(
            NodeID const & i ) [virtual]
```

Returns

The distance from srcNode.

Parameters

i	The source node.

9.13.4.3 getDist() [3/3]

```
template<typename Network = DNetwork<>, typename DistType = double, typename NbHopsType =
std::size_t>
virtual std::pair<DistType, NbHopsType> LinkPred::DESPLDistCalculator< Network, DistType,
NbHopsType >::getDist (
            NodeID const & i,
            NodeID const & j ) [virtual]
```

Parameters

i	Index of the source node.
j	Index of the end node.

Returns

The distance between i and j.

9.13.4.4 getFinDistMapNoNeighb()

```
template<typename Network = DNetwork<>, typename DistType = double, typename NbHopsType =
std::size_t>
virtual NodeSDistMapSP LinkPred::DESPLDistCalculator< Network, DistType, NbHopsType >::get←
FinDistMapNoNeighb (
            NodeID const & srcNode )  [virtual]
```

Returns

A sparse distance map of nodes having finite distance to a given source node. Only nodes not connected to srcNode are considered. The node srcNode itself is also excluded.

Parameters

srcNode	The source node.

9.13.4.5 getIndDist()

```
template<typename Network = DNetwork<>, typename DistType = double, typename NbHopsType =
std::size_t>
virtual std::pair<DistType, NbHopsType> LinkPred::DESPLDistCalculator< Network, DistType,
NbHopsType >::getIndDist (
            NodeID const & i,
            NodeID const & j )  [inline], [virtual]
```

Parameters

i	Index of the source node.
j	Index of the end node.

Returns

The distance between i and j ignoring the edge between i and j.

9.13.4.6 getLim()

```
template<typename Network = DNetwork<>, typename DistType = double, typename NbHopsType =
std::size_t>
std::size_t LinkPred::DESPLDistCalculator< Network, DistType, NbHopsType >::getLim ( ) const
[inline]
```

Returns

The limit on the number of hops.

9.13.4.7 getMaxNbNodesInCache()

```
template<typename Network = DNetwork<>, typename DistType = double, typename NbHopsType =
std::size_t>
std::size_t LinkPred::DESPLDistCalculator< Network, DistType, NbHopsType >::getMaxNbNodesIn←
Cache ( ) const [inline]
```

Returns

The maximum number of nodes allowed in cache.

9.13.4.8 getSDistMap()

```
template<typename Network = DNetwork<>, typename DistType = double, typename NbHopsType =
std::size_t>
virtual NodeSDistMapSP LinkPred::DESPLDistCalculator< Network, DistType, NbHopsType >::getS←
DistMap (
            NodeID const & i ) [virtual]
```

Returns

The sparse distance from srcNode.

Parameters

i	The source node.

9.13.4.9 operator=() [1/2]

```
template<typename Network = DNetwork<>, typename DistType = double, typename NbHopsType =
std::size_t>
```

```
DESPLDistCalculator& LinkPred::DESPLDistCalculator< Network, DistType, NbHopsType >::operator=
(
            DESPLDistCalculator< Network, DistType, NbHopsType > && that )  [default]
```

Move assignment operator.

Parameters

that	The object to move.

9.13.4.10 operator=() [2/2]

```
template<typename Network = DNetwork<>, typename DistType = double, typename NbHopsType =
std::size_t>
DESPLDistCalculator& LinkPred::DESPLDistCalculator< Network, DistType, NbHopsType >::operator=
(
            DESPLDistCalculator< Network, DistType, NbHopsType > const & that )  [default]
```

Copy assignment operator.

Parameters

that	The object to copy.

9.13.4.11 setMaxNbNodesInCache()

```
template<typename Network = DNetwork<>, typename DistType = double, typename NbHopsType =
std::size_t>
void LinkPred::DESPLDistCalculator< Network, DistType, NbHopsType >::setMaxNbNodesInCache (
            std::size_t maxNbNodesInCache )  [inline]
```

Set the maximum number of nodes in cache (for each node a map of distance to all other nodes is kept in memory).

Parameters

maxNbNodesInCache	New value for the maximum number of nodes in cache.

The documentation for this class was generated from the following file:

- include/linkpred/graphalg/shortestpaths/netdistcalculator.hpp

9.14 LinkPred::DFS< Network, NodeProcessor > Class Template Reference

DFS graph traversal.

`#include <graphtraversal.hpp>`

Inheritance diagram for LinkPred::DFS< Network, NodeProcessor >:

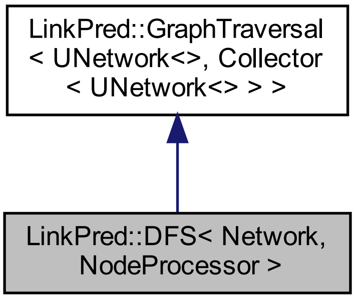

Collaboration diagram for LinkPred::DFS< Network, NodeProcessor >:

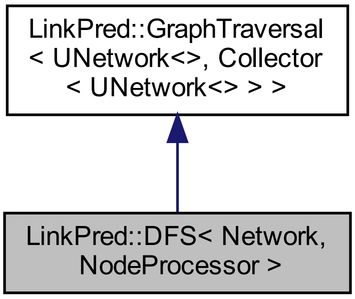

Public Member Functions

- DFS (std::shared_ptr< Network const > net)
- DFS (DFS const &that)=default
- DFS & operator= (DFS const &that)=default
- DFS (DFS &&that)=default
- DFS & operator= (DFS &&that)=default
- virtual void traverse (typename Network::NodeID srcNode, NodeProcessor &processor)
- virtual ~DFS ()=default

9.14.1 Detailed Description

template< typename Network = UNetwork<>, typename NodeProcessor = Collector< Network>>
class LinkPred::DFS< Network, NodeProcessor >

DFS graph traversal.

Template Parameters

Network	The network type.
NodeProcessor	The node processor type.

9.14.2 Constructor & Destructor Documentation

9.14.2.1 DFS() [1/3]

```
template<typename Network = UNetwork<>, typename NodeProcessor = Collector< Network>>
LinkPred::DFS< Network, NodeProcessor >::DFS (
            std::shared_ptr< Network const > net )  [inline]
```

< The network on which traversal is done. Constructor.

Parameters

net	The network on which traversal is done.

9.14.2.2 DFS() [2/3]

```
template<typename Network = UNetwork<>, typename NodeProcessor = Collector< Network>>
LinkPred::DFS< Network, NodeProcessor >::DFS (
            DFS< Network, NodeProcessor > const & that )  [default]
```

Copy constructor.

Parameters

that	The object to copy.

9.14.2.3 DFS() [3/3]

```
template<typename Network = UNetwork<>, typename NodeProcessor = Collector< Network>>
LinkPred::DFS< Network, NodeProcessor >::DFS (
            DFS< Network, NodeProcessor > && that )  [default]
```

Move constructor.

Parameters

that	The object to move.

9.14.2.4 ∼DFS()

```
template<typename Network = UNetwork<>, typename NodeProcessor = Collector< Network>>
virtual LinkPred::DFS< Network, NodeProcessor >::~DFS ( )  [virtual], [default]
```

Destructor.

9.14.3 Member Function Documentation

9.14.3.1 operator=() [1/2]

```
template<typename Network = UNetwork<>, typename NodeProcessor = Collector< Network>>
DFS& LinkPred::DFS< Network, NodeProcessor >::operator= (
            DFS< Network, NodeProcessor > && that )  [default]
```

Move assignment operator.

Parameters

that	The object to move.

9.14.3.2 operator=() [2/2]

```
template<typename Network = UNetwork<>, typename NodeProcessor = Collector< Network>>
DFS& LinkPred::DFS< Network, NodeProcessor >::operator= (
            DFS< Network, NodeProcessor > const & that )  [default]
```

Copy assignment operator.

Parameters

that	The object to copy.

9.14.3.3 traverse()

```
template<typename Network = UNetwork<>, typename NodeProcessor = Collector< Network>>
virtual void LinkPred::DFS< Network, NodeProcessor >::traverse (
            typename Network::NodeID srcNode,
            NodeProcessor & processor )  [inline], [virtual]
```

Traverse the graph in DFS order.

Parameters

srcNode	The source node.
processor	The node processor.

The documentation for this class was generated from the following file:

- include/linkpred/graphalg/traversal/graphtraversal.hpp

9.15 LinkPred::DHDIPredictor< Network, EdgeRndIt, ScoreRndIt, EdgeRndOutIt > Class Template Reference

Common neighbor link predictor adapted to directed networks.

```
#include <dhdipredictor.hpp>
```

Inheritance diagram for LinkPred::DHDIPredictor< Network, EdgeRndIt, ScoreRndIt, EdgeRndOutIt >:

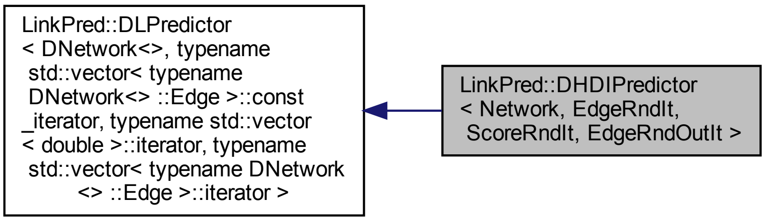

Collaboration diagram for LinkPred::DHDIPredictor< Network, EdgeRndIt, ScoreRndIt, EdgeRndOutIt >:

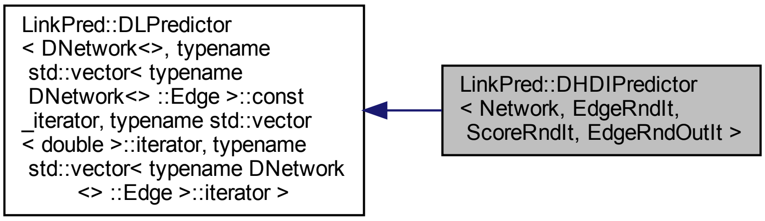

Public Member Functions

- DHDIPredictor (std::shared_ptr< Network const > net)
- DHDIPredictor (DHDIPredictor const &that)=default
- DHDIPredictor & operator= (DHDIPredictor const &that)=default
- DHDIPredictor (DHDIPredictor &&that)=default
- DHDIPredictor & operator= (DHDIPredictor &&that)=default
- virtual void init ()
- virtual void learn ()
- virtual void predict (EdgeRndIt begin, EdgeRndIt end, ScoreRndIt scores)
- virtual double score (Edge const &e)
- virtual std::size_t top (std::size_t k, EdgeRndOutIt eit, ScoreRndIt sit)
- virtual ~DHDIPredictor ()=default

Additional Inherited Members

9.15.1 Detailed Description

template<typename Network = DNetwork<>, typename EdgeRndIt = typename std::vector<typename Network::Edge>↩ ::const_iterator, typename ScoreRndIt = typename std::vector<double>::iterator, typename EdgeRndOutIt = typename std↩ ::vector<typename Network::Edge>::iterator>
class LinkPred::DHDIPredictor< Network, EdgeRndIt, ScoreRndIt, EdgeRndOutIt >

Common neighbor link predictor adapted to directed networks.

Template Parameters

Network	The network type.
Edge↩ RndIt	A random iterator type used to iterate on edges.
Score↩ RndIt	A random iterator type used to iterate on scores.

9.15.2 Constructor & Destructor Documentation

9.15.2.1 DHDIPredictor() [1/3]

```
template<typename Network = DNetwork<>, typename EdgeRndIt = typename std::vector<typename
Network::Edge>::const_iterator, typename ScoreRndIt = typename std::vector<double>::iterator,
typename EdgeRndOutIt = typename std::vector<typename Network::Edge>::iterator>
LinkPred::DHDIPredictor< Network, EdgeRndIt, ScoreRndIt, EdgeRndOutIt >::DHDIPredictor (
            std::shared_ptr< Network const > net )  [inline]
```

Parameters

net	The network.

9.15.2.2 DHDIPredictor() [2/3]

```
template<typename Network = DNetwork<>, typename EdgeRndIt = typename std::vector<typename
Network::Edge>::const_iterator, typename ScoreRndIt = typename std::vector<double>::iterator,
typename EdgeRndOutIt = typename std::vector<typename Network::Edge>::iterator>
LinkPred::DHDIPredictor< Network, EdgeRndIt, ScoreRndIt, EdgeRndOutIt >::DHDIPredictor (
            DHDIPredictor< Network, EdgeRndIt, ScoreRndIt, EdgeRndOutIt > const & that )
[default]
```

Copy constructor.

Parameters

that	The object to copy.

9.15.2.3 DHDIPredictor() [3/3]

```
template<typename Network = DNetwork<>, typename EdgeRndIt = typename std::vector<typename
Network::Edge>::const_iterator, typename ScoreRndIt = typename std::vector<double>::iterator,
typename EdgeRndOutIt = typename std::vector<typename Network::Edge>::iterator>
LinkPred::DHDIPredictor< Network, EdgeRndIt, ScoreRndIt, EdgeRndOutIt >::DHDIPredictor (
            DHDIPredictor< Network, EdgeRndIt, ScoreRndIt, EdgeRndOutIt > && that )  [default]
```

Move constructor.

Parameters

that	The object to move.

9.15.2.4 ∼DHDIPredictor()

```
template<typename Network = DNetwork<>, typename EdgeRndIt = typename std::vector<typename
Network::Edge>::const_iterator, typename ScoreRndIt = typename std::vector<double>::iterator,
typename EdgeRndOutIt = typename std::vector<typename Network::Edge>::iterator>
virtual LinkPred::DHDIPredictor< Network, EdgeRndIt, ScoreRndIt, EdgeRndOutIt >::~DHDIPredictor
( )  [virtual], [default]
```

Destructor.

9.15.3 Member Function Documentation

9.15.3.1 init()

```
template<typename Network = DNetwork<>, typename EdgeRndIt = typename std::vector<typename
Network::Edge>::const_iterator, typename ScoreRndIt = typename std::vector<double>::iterator,
typename EdgeRndOutIt = typename std::vector<typename Network::Edge>::iterator>
virtual void LinkPred::DHDIPredictor< Network, EdgeRndIt, ScoreRndIt, EdgeRndOutIt >::init ( )
[inline], [virtual]
```

Initialize the solver.

Implements LinkPred::DLPredictor< DNetwork<>, typename std::vector< typename DNetwork<> ::Edge >::const_iterator, typena

9.15.3.2 learn()

```
template<typename Network = DNetwork<>, typename EdgeRndIt = typename std::vector<typename
Network::Edge>::const_iterator, typename ScoreRndIt = typename std::vector<double>::iterator,
typename EdgeRndOutIt = typename std::vector<typename Network::Edge>::iterator>
virtual void LinkPred::DHDIPredictor< Network, EdgeRndIt, ScoreRndIt, EdgeRndOutIt >::learn (
) [inline], [virtual]
```

Learn.

Implements LinkPred::DLPredictor< DNetwork<>, typename std::vector< typename DNetwork<> ::Edge >::const_iterator, typena

9.15.3.3 operator=() [1/2]

```
template<typename Network = DNetwork<>, typename EdgeRndIt = typename std::vector<typename
Network::Edge>::const_iterator, typename ScoreRndIt = typename std::vector<double>::iterator,
typename EdgeRndOutIt = typename std::vector<typename Network::Edge>::iterator>
DHDIPredictor& LinkPred::DHDIPredictor< Network, EdgeRndIt, ScoreRndIt, EdgeRndOutIt >::operator=
(
            DHDIPredictor< Network, EdgeRndIt, ScoreRndIt, EdgeRndOutIt > && that )  [default]
```

Move assignment operator.

Parameters

that	The object to move.

9.15.3.4 operator=() [2/2]

```
template<typename Network = DNetwork<>, typename EdgeRndIt = typename std::vector<typename
Network::Edge>::const_iterator, typename ScoreRndIt = typename std::vector<double>::iterator,
typename EdgeRndOutIt = typename std::vector<typename Network::Edge>::iterator>
```

```
DHDIPredictor& LinkPred::DHDIPredictor< Network, EdgeRndIt, ScoreRndIt, EdgeRndOutIt >::operator=
(
            DHDIPredictor< Network, EdgeRndIt, ScoreRndIt, EdgeRndOutIt > const & that )
[default]
```

Copy assignment operator.

Parameters

that	The object to copy.

9.15.3.5 predict()

```
template<typename Network = DNetwork<>, typename EdgeRndIt = typename std::vector<typename
Network::Edge>::const_iterator, typename ScoreRndIt = typename std::vector<double>::iterator,
typename EdgeRndOutIt = typename std::vector<typename Network::Edge>::iterator>
virtual void LinkPred::DHDIPredictor< Network, EdgeRndIt, ScoreRndIt, EdgeRndOutIt >::predict
(
            EdgeRndIt begin,
            EdgeRndIt end,
            ScoreRndIt scores )  [virtual]
```

Predict the links.

Parameters

begin	Beginning of the links to be predicted.
end	end of the links to be predicted.
scores	Beginning of scores.

Reimplemented from LinkPred::DLPredictor< DNetwork<>, typename std::vector< typename DNetwork<> ::Edge >::const_iterat

9.15.3.6 score()

```
template<typename Network = DNetwork<>, typename EdgeRndIt = typename std::vector<typename
Network::Edge>::const_iterator, typename ScoreRndIt = typename std::vector<double>::iterator,
typename EdgeRndOutIt = typename std::vector<typename Network::Edge>::iterator>
virtual double LinkPred::DHDIPredictor< Network, EdgeRndIt, ScoreRndIt, EdgeRndOutIt >::score
(
            Edge const & e )  [virtual]
```

Compute the score of a single edge.

Parameters

e	The edge.

Returns

The score of e.

9.15.3.7 top()

```
template<typename Network = DNetwork<>, typename EdgeRndIt = typename std::vector<typename
Network::Edge>::const_iterator, typename ScoreRndIt = typename std::vector<double>::iterator,
typename EdgeRndOutIt = typename std::vector<typename Network::Edge>::iterator>
virtual std::size_t LinkPred::DHDIPredictor< Network, EdgeRndIt, ScoreRndIt, EdgeRndOutIt >↩
::top (
            std::size_t k,
            EdgeRndOutIt eit,
            ScoreRndIt sit )  [virtual]
```

Finds the k negative edges with the top score. Ties are broken randomly.

Parameters

k	The number of edges to find.
eit	An output iterator where the edges are written.
sit	An output iterator where the scores are written. The scores are written in the same order as the edges.

Returns

The number of negative edges inserted. It is the minimum between k and the number of negative edges in the network.

Reimplemented from LinkPred::DLPredictor< DNetwork<>, typename std::vector< typename DNetwork<> ::Edge >::const_iterat

The documentation for this class was generated from the following file:

- include/linkpred/predictors/directed/dhdipredictor.hpp

9.16 LinkPred::DHPIPredictor< Network, EdgeRndIt, ScoreRndIt, EdgeRndOutIt > Class Template Reference

Common neighbor link predictor adapted to directed networks.

```
#include <dhpipredictor.hpp>
```

Inheritance diagram for LinkPred::DHPIPredictor< Network, EdgeRndIt, ScoreRndIt, EdgeRndOutIt >:

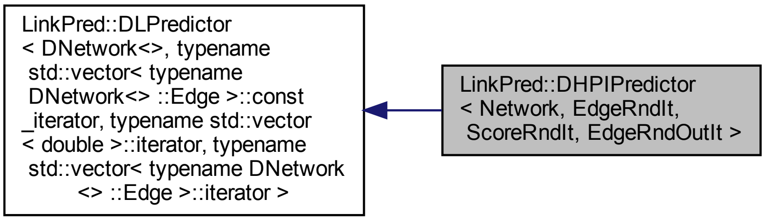

Collaboration diagram for LinkPred::DHPIPredictor< Network, EdgeRndIt, ScoreRndIt, EdgeRndOutIt >:

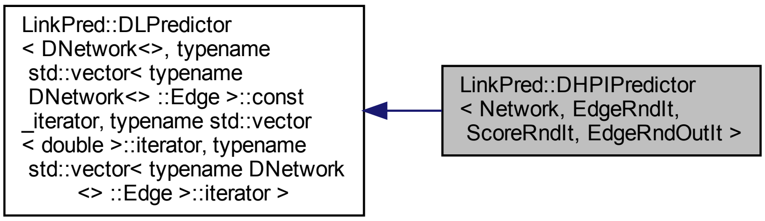

Public Member Functions

- DHPIPredictor (std::shared_ptr< Network const > net)
- DHPIPredictor (DHPIPredictor const &that)=default
- DHPIPredictor & operator= (DHPIPredictor const &that)=default
- DHPIPredictor (DHPIPredictor &&that)=default
- DHPIPredictor & operator= (DHPIPredictor &&that)=default
- virtual void init ()
- virtual void learn ()
- virtual void predict (EdgeRndIt begin, EdgeRndIt end, ScoreRndIt scores)
- virtual double score (Edge const &e)
- virtual std::size_t top (std::size_t k, EdgeRndOutIt eit, ScoreRndIt sit)
- virtual ~DHPIPredictor ()=default

Additional Inherited Members

9.16.1 Detailed Description

template<typename Network = DNetwork<>, typename EdgeRndIt = typename std::vector<typename Network::Edge>↩ ::const_iterator, typename ScoreRndIt = typename std::vector<double>::iterator, typename EdgeRndOutIt = typename std↩ ::vector<typename Network::Edge>::iterator>
class LinkPred::DHPIPredictor< Network, EdgeRndIt, ScoreRndIt, EdgeRndOutIt >

Common neighbor link predictor adapted to directed networks.

Template Parameters

Network	The network type.
Edge↩ RndIt	A random iterator type used to iterate on edges.
Score↩ RndIt	A random iterator type used to iterate on scores.

9.16.2 Constructor & Destructor Documentation

9.16.2.1 DHPIPredictor() [1/3]

```
template<typename Network = DNetwork<>, typename EdgeRndIt = typename std::vector<typename
Network::Edge>::const_iterator, typename ScoreRndIt = typename std::vector<double>::iterator,
typename EdgeRndOutIt = typename std::vector<typename Network::Edge>::iterator>
LinkPred::DHPIPredictor< Network, EdgeRndIt, ScoreRndIt, EdgeRndOutIt >::DHPIPredictor (
            std::shared_ptr< Network const > net ) [inline]
```

Parameters

net	The network.

9.16.2.2 DHPIPredictor() [2/3]

```
template<typename Network = DNetwork<>, typename EdgeRndIt = typename std::vector<typename
Network::Edge>::const_iterator, typename ScoreRndIt = typename std::vector<double>::iterator,
typename EdgeRndOutIt = typename std::vector<typename Network::Edge>::iterator>
LinkPred::DHPIPredictor< Network, EdgeRndIt, ScoreRndIt, EdgeRndOutIt >::DHPIPredictor (
            DHPIPredictor< Network, EdgeRndIt, ScoreRndIt, EdgeRndOutIt > const & that )
[default]
```

Copy constructor.

Parameters

that	The object to copy.

9.16.2.3 DHPIPredictor() [3/3]

```
template<typename Network = DNetwork<>, typename EdgeRndIt = typename std::vector<typename
Network::Edge>::const_iterator, typename ScoreRndIt = typename std::vector<double>::iterator,
```

```
typename EdgeRndOutIt = typename std::vector<typename Network::Edge>::iterator>
LinkPred::DHPIPredictor< Network, EdgeRndIt, ScoreRndIt, EdgeRndOutIt >::DHPIPredictor (
            DHPIPredictor< Network, EdgeRndIt, ScoreRndIt, EdgeRndOutIt > && that )  [default]
```

Move constructor.

Parameters

that	The object to move.

9.16.2.4 ∼DHPIPredictor()

```
template<typename Network = DNetwork<>, typename EdgeRndIt = typename std::vector<typename
Network::Edge>::const_iterator, typename ScoreRndIt = typename std::vector<double>::iterator,
typename EdgeRndOutIt = typename std::vector<typename Network::Edge>::iterator>
virtual LinkPred::DHPIPredictor< Network, EdgeRndIt, ScoreRndIt, EdgeRndOutIt >::~DHPIPredictor
( )  [virtual], [default]
```

Destructor.

9.16.3 Member Function Documentation

9.16.3.1 init()

```
template<typename Network = DNetwork<>, typename EdgeRndIt = typename std::vector<typename
Network::Edge>::const_iterator, typename ScoreRndIt = typename std::vector<double>::iterator,
typename EdgeRndOutIt = typename std::vector<typename Network::Edge>::iterator>
virtual void LinkPred::DHPIPredictor< Network, EdgeRndIt, ScoreRndIt, EdgeRndOutIt >::init ( )
[inline], [virtual]
```

Initialize the solver.

Implements LinkPred::DLPredictor< DNetwork<>, typename std::vector< typename DNetwork<> ::Edge >::const_iterator, typena

9.16.3.2 learn()

```
template<typename Network = DNetwork<>, typename EdgeRndIt = typename std::vector<typename
Network::Edge>::const_iterator, typename ScoreRndIt = typename std::vector<double>::iterator,
typename EdgeRndOutIt = typename std::vector<typename Network::Edge>::iterator>
virtual void LinkPred::DHPIPredictor< Network, EdgeRndIt, ScoreRndIt, EdgeRndOutIt >::learn (
)  [inline], [virtual]
```

Learn.

Implements LinkPred::DLPredictor< DNetwork<>, typename std::vector< typename DNetwork<> ::Edge >::const_iterator, typena

9.16.3.3 operator=() [1/2]

```
template<typename Network = DNetwork<>, typename EdgeRndIt = typename std::vector<typename
Network::Edge>::const_iterator, typename ScoreRndIt = typename std::vector<double>::iterator,
typename EdgeRndOutIt = typename std::vector<typename Network::Edge>::iterator>
DHPIPredictor& LinkPred::DHPIPredictor< Network, EdgeRndIt, ScoreRndIt, EdgeRndOutIt >::operator=
(
            DHPIPredictor< Network, EdgeRndIt, ScoreRndIt, EdgeRndOutIt > && that )  [default]
```

Move assignment operator.

Parameters

that	The object to move.

9.16.3.4 operator=() [2/2]

```
template<typename Network = DNetwork<>, typename EdgeRndIt = typename std::vector<typename
Network::Edge>::const_iterator, typename ScoreRndIt = typename std::vector<double>::iterator,
typename EdgeRndOutIt = typename std::vector<typename Network::Edge>::iterator>
DHPIPredictor& LinkPred::DHPIPredictor< Network, EdgeRndIt, ScoreRndIt, EdgeRndOutIt >::operator=
(
            DHPIPredictor< Network, EdgeRndIt, ScoreRndIt, EdgeRndOutIt > const & that )
[default]
```

Copy assignment operator.

Parameters

that	The object to copy.

9.16.3.5 predict()

```
template<typename Network = DNetwork<>, typename EdgeRndIt = typename std::vector<typename
Network::Edge>::const_iterator, typename ScoreRndIt = typename std::vector<double>::iterator,
typename EdgeRndOutIt = typename std::vector<typename Network::Edge>::iterator>
virtual void LinkPred::DHPIPredictor< Network, EdgeRndIt, ScoreRndIt, EdgeRndOutIt >::predict
(
            EdgeRndIt begin,
            EdgeRndIt end,
            ScoreRndIt scores )  [virtual]
```

Predict the links.

Parameters

begin	Beginning of the links to be predicted.
end	end of the links to be predicted.
scores	Beginning of scores.

Reimplemented from LinkPred::DLPredictor< DNetwork<>, typename std::vector< typename DNetwork<> ::Edge >::const_iterat

9.16.3.6 score()

```
template<typename Network = DNetwork<>, typename EdgeRndIt = typename std::vector<typename
Network::Edge>::const_iterator, typename ScoreRndIt = typename std::vector<double>::iterator,
typename EdgeRndOutIt = typename std::vector<typename Network::Edge>::iterator>
virtual double LinkPred::DHPIPredictor< Network, EdgeRndIt, ScoreRndIt, EdgeRndOutIt >::score
(
            Edge const & e )  [virtual]
```

Compute the score of a single edge.

Parameters

e	The edge.

Returns

The score of e.

9.16.3.7 top()

```
template<typename Network = DNetwork<>, typename EdgeRndIt = typename std::vector<typename
Network::Edge>::const_iterator, typename ScoreRndIt = typename std::vector<double>::iterator,
typename EdgeRndOutIt = typename std::vector<typename Network::Edge>::iterator>
virtual std::size_t LinkPred::DHPIPredictor< Network, EdgeRndIt, ScoreRndIt, EdgeRndOutIt >↩
::top (
            std::size_t k,
            EdgeRndOutIt eit,
            ScoreRndIt sit )  [virtual]
```

Finds the k negative edges with the top score. Ties are broken randomly.

Parameters

k	The number of edges to find.
eit	An output iterator where the edges are written.
sit	An output iterator where the scores are written. The scores are written in the same order as the edges.

Returns

The number of negative edges inserted. It is the minimum between k and the number of negative edges in the network.

Reimplemented from LinkPred::DLPredictor< DNetwork<>, typename std::vector< typename DNetwork<> ::Edge >::const_iterat

The documentation for this class was generated from the following file:

- include/linkpred/predictors/directed/dhpipredictor.hpp

9.17 LinkPred::Dijkstra< Network, DistType, NbHopsType > Class Template Reference

An implementation of Dijkstra's algorithm.

```
#include <dijkstra.hpp>
```

Public Types

- using LengthMapIdType = long int
- using NetworkSP = std::shared_ptr< Network >
- using NodeID = typename Network::NodeID
- using Label = typename Network::Label
- using Edge = typename Network::Edge
- using NodeDistMap = typename Network::template NodeMap< std::pair< DistType, NbHopsType > >
- using NodeDistMapSP = typename Network::template NodeMapSP< std::pair< DistType, NbHopsType > >
- using NodeSDistMapSP = typename Network::template NodeSMapSP< std::pair< DistType, NbHopsType > >
- using EdgeLengthMap = typename Network::template EdgeMap< DistType >
- using EdgeLengthMapSP = typename Network::template EdgeMapSP< DistType >
- using PathType = std::vector< NodeID >
- using PathTypeSP = std::shared_ptr< PathType >

Public Member Functions

- Dijkstra (std::shared_ptr< Network const > net)
- Dijkstra ()=default
- Dijkstra (Dijkstra const &that)=default
- Dijkstra & operator= (Dijkstra const &that)=default
- Dijkstra (Dijkstra &&that)=default
- Dijkstra & operator= (Dijkstra &&that)=default
- std::shared_ptr< Network const > getNet () const
- void setNet (std::shared_ptr< const Network > net)
- LengthMapIdType registerLengthMap (EdgeLengthMapSP length)
- void unregisterLengthMap (LengthMapIdType const &lengthMapId)
- std::pair< DistType, NbHopsType > getIndDist (NodeID const &srcId, NodeID const &dstId, LengthMapIdType lengthMapId, DistType discDist=std::numeric_limits< DistType >::infinity(), NbHopsType discNbHops=std::numeric_limits< NbHopsType >::max()) const
- NodeDistMapSP getIndDistToAll (NodeID const &srcId, NodeID const &dstId, LengthMapIdType lengthMapId, DistType discDist=std::numeric_limits< DistType >::infinity(), NbHopsType discNbHops=std::numeric_limits< NbHopsType >::max()) const
- std::pair< PathTypeSP, double > getShortestPath (NodeID const &srcId, NodeID const &dstId, LengthMapIdType lengthMapId, DistType discDist=std::numeric_limits< DistType >::infinity(), NbHopsType discNbHops=std::numeric_limits< NbHopsType >::max()) const
- NodeDistMapSP getDist (NodeID const &srcId, LengthMapIdType lengthMapId, DistType discDist=std::numeric_limits< DistType >::infinity(), NbHopsType discNbHops=std::numeric_limits< NbHopsType >::max()) const

- NodeSDistMapSP getDistL (NodeID const &srcId, LengthMapIdType lengthMapId, std::size_t lim, DistType discDist=std::numeric_limits< DistType >::infinity(), NbHopsType discNbHops=std::numeric_limits< Nb↵ HopsType >::max()) const
- template<typename InputIterator , typename OuputIterator >
  void getDist (InputIterator begin, InputIterator end, OuputIterator outit, LengthMapIdType lengthMapId, Dist↵ Type discDist=std::numeric_limits< DistType >::infinity(), NbHopsType discNbHops=std::numeric_limits< NbHopsType >::max()) const
- template<typename InputIterator , typename OuputIterator >
  void getSDist (InputIterator begin, InputIterator end, OuputIterator outit, LengthMapIdType lengthMap↵ Id, DistType discDist=std::numeric_limits< DistType >::infinity(), NbHopsType discNbHops=std::numeric↵ _limits< NbHopsType >::max()) const
- NodeDistMapSP getDsim (NodeID const &srcId, LengthMapIdType lengthMapId, DistType discDist=std↵ ::numeric_limits< DistType >::infinity(), NbHopsType discNbHops=std::numeric_limits< NbHopsType >↵ ::max()) const
- std::pair< DistType, NbHopsType > getIndDsim (NodeID const &srcId, NodeID const &dstId, LengthMapIdType lengthMapId, DistType discDist=std::numeric_limits< DistType >::infinity(), NbHopsType discNbHops=std↵ ::numeric_limits< NbHopsType >::max()) const
- std::map< NodeID, NodeDistMapSP > getDsim (std::set< NodeID > const &srcIds, LengthMapIdType lengthMapId, DistType discDist=std::numeric_limits< DistType >::infinity(), NbHopsType discNbHops=std↵ ::numeric_limits< NbHopsType >::max()) const
- virtual ∼Dijkstra ()=default

9.17.1 Detailed Description

template<typename Network = UNetwork<>, typename DistType = double, typename NbHopsType = std::size_t>
class LinkPred::Dijkstra< Network, DistType, NbHopsType >

An implementation of Dijkstra's algorithm.

This class currently implements Dijkstra's algorithm using a binary heap, which means that the algorithm runs in O(m + n (log n)^2) instead of O(m + n log n).

Template Parameters

Network	The network type.
DistType	The distance type (can be an integer or floating point type).
NbHopsType	The type of the number of hops (must be an integer type).

9.17.2 Member Typedef Documentation

9.17.2.1 Edge

```
template<typename Network = UNetwork<>, typename DistType = double, typename NbHopsType =
std::size_t>
using LinkPred::Dijkstra< Network, DistType, NbHopsType >::Edge = typename Network::Edge
```

Edge type.

9.17.2.2 EdgeLengthMap

```
template<typename Network = UNetwork<>, typename DistType = double, typename NbHopsType =
std::size_t>
using LinkPred::Dijkstra< Network, DistType, NbHopsType >::EdgeLengthMap = typename Network←
::template EdgeMap<DistType>
```

Edge length map.

9.17.2.3 EdgeLengthMapSP

```
template<typename Network = UNetwork<>, typename DistType = double, typename NbHopsType =
std::size_t>
using LinkPred::Dijkstra< Network, DistType, NbHopsType >::EdgeLengthMapSP = typename Network←
::template EdgeMapSP<DistType>
```

Shared pointer to an edge length map.

9.17.2.4 Label

```
template<typename Network = UNetwork<>, typename DistType = double, typename NbHopsType =
std::size_t>
using LinkPred::Dijkstra< Network, DistType, NbHopsType >::Label = typename Network::Label
```

Nodes label type.

9.17.2.5 LengthMapIdType

```
template<typename Network = UNetwork<>, typename DistType = double, typename NbHopsType =
std::size_t>
using LinkPred::Dijkstra< Network, DistType, NbHopsType >::LengthMapIdType = long int
```

Length map ID type.

9.17.2.6 NetworkSP

```
template<typename Network = UNetwork<>, typename DistType = double, typename NbHopsType =
std::size_t>
using LinkPred::Dijkstra< Network, DistType, NbHopsType >::NetworkSP = std::shared_ptr<Network>
```

Shared pointer to network.

9.17.2.7 NodeDistMap

```
template<typename Network = UNetwork<>, typename DistType = double, typename NbHopsType =
std::size_t>
using LinkPred::Dijkstra< Network, DistType, NbHopsType >::NodeDistMap = typename Network←
::template NodeMap<std::pair<DistType, NbHopsType> >
```

Distance map.

9.17.2.8 NodeDistMapSP

```
template<typename Network = UNetwork<>, typename DistType = double, typename NbHopsType =
std::size_t>
using LinkPred::Dijkstra< Network, DistType, NbHopsType >::NodeDistMapSP = typename Network↩
::template NodeMapSP<std::pair<DistType, NbHopsType> >
```

Shared pointer to a distance map.

9.17.2.9 NodeID

```
template<typename Network = UNetwork<>, typename DistType = double, typename NbHopsType =
std::size_t>
using LinkPred::Dijkstra< Network, DistType, NbHopsType >::NodeID = typename Network::NodeID
```

Nodes ID type.

9.17.2.10 NodeSDistMapSP

```
template<typename Network = UNetwork<>, typename DistType = double, typename NbHopsType =
std::size_t>
using LinkPred::Dijkstra< Network, DistType, NbHopsType >::NodeSDistMapSP = typename Network↩
::template NodeSMapSP<std::pair<DistType, NbHopsType> >
```

Shared pointer to a sparse distance map.

9.17.2.11 PathType

```
template<typename Network = UNetwork<>, typename DistType = double, typename NbHopsType =
std::size_t>
using LinkPred::Dijkstra< Network, DistType, NbHopsType >::PathType = std::vector<NodeID>
```

Path.

9.17.2.12 PathTypeSP

```
template<typename Network = UNetwork<>, typename DistType = double, typename NbHopsType =
std::size_t>
using LinkPred::Dijkstra< Network, DistType, NbHopsType >::PathTypeSP = std::shared_ptr<PathType>
```

Shared pointer to a path.

9.17.3 Constructor & Destructor Documentation

9.17.3.1 Dijkstra() [1/4]

```
template<typename Network = UNetwork<>, typename DistType = double, typename NbHopsType =
std::size_t>
LinkPred::Dijkstra< Network, DistType, NbHopsType >::Dijkstra (
            std::shared_ptr< Network const > net )  [inline]
```

Constructor.

9.17.3.2 Dijkstra() [2/4]

```
template<typename Network = UNetwork<>, typename DistType = double, typename NbHopsType =
std::size_t>
LinkPred::Dijkstra< Network, DistType, NbHopsType >::Dijkstra ( )  [default]
```

Default constructor.

9.17.3.3 Dijkstra() [3/4]

```
template<typename Network = UNetwork<>, typename DistType = double, typename NbHopsType =
std::size_t>
LinkPred::Dijkstra< Network, DistType, NbHopsType >::Dijkstra (
            Dijkstra< Network, DistType, NbHopsType > const & that )  [default]
```

Copy constructor.

Parameters

that	The object to copy.

9.17.3.4 Dijkstra() [4/4]

```
template<typename Network = UNetwork<>, typename DistType = double, typename NbHopsType =
std::size_t>
LinkPred::Dijkstra< Network, DistType, NbHopsType >::Dijkstra (
            Dijkstra< Network, DistType, NbHopsType > && that )  [default]
```

Move constructor.

Parameters

that	The object to move.

9.17.3.5 ∼Dijkstra()

```
template<typename Network = UNetwork<>, typename DistType = double, typename NbHopsType =
std::size_t>
virtual LinkPred::Dijkstra< Network, DistType, NbHopsType >::~Dijkstra ( )  [virtual], [default]
```

Destructor.

9.17.4 Member Function Documentation

9.17.4.1 getDist() [1/2]

```
template<typename Network = UNetwork<>, typename DistType = double, typename NbHopsType =
std::size_t>
template<typename InputIterator , typename OuputIterator >
void LinkPred::Dijkstra< Network, DistType, NbHopsType >::getDist (
            InputIterator begin,
            InputIterator end,
            OuputIterator outit,
            LengthMapIdType lengthMapId,
            DistType discDist = std::numeric_limits<DistType>::infinity(),
            NbHopsType discNbHops = std::numeric_limits<NbHopsType>::max() ) const  [inline]
```

Computes the distance from a list of nodes to all other nodes.

Parameters

begin	Iterator to the first element in the list of source nodes.
end	Iterator to one past the last element in the list of source nodes.
outit	Output iterator.
length←↩ MapId	The ID of the map length (as returned by UNetwork::registerLengthMap).
discDist	The value that should be assigned as distance between disconnected nodes.
discNbHops	The value that should be assigned as number of hops between disconnected nodes.

9.17.4.2 getDist() [2/2]

```
template<typename Network = UNetwork<>, typename DistType = double, typename NbHopsType =
std::size_t>
NodeDistMapSP LinkPred::Dijkstra< Network, DistType, NbHopsType >::getDist (
            NodeID const & srcId,
            LengthMapIdType lengthMapId,
            DistType discDist = std::numeric_limits< DistType >::infinity(),
            NbHopsType discNbHops = std::numeric_limits< NbHopsType >::max() ) const
```

Computes the distance from a single source node to all other nodes.

Parameters

srcId	The ID of the source node.
length←↩ MapId	The ID of the map length (as returned by UNetwork::registerLengthMap).
discDist	The value that should be assigned as distance between disconnected nodes.
discNbHops	The value that should be assigned as number of hops between disconnected nodes.

Returns

The distance map.

9.17.4.3 getDistL()

```
template<typename Network = UNetwork<>, typename DistType = double, typename NbHopsType =
std::size_t>
NodeSDistMapSP LinkPred::Dijkstra< Network, DistType, NbHopsType >::getDistL (
            NodeID const & srcId,
            LengthMapIdType lengthMapId,
            std::size_t lim,
            DistType discDist = std::numeric_limits< DistType >::infinity(),
            NbHopsType discNbHops = std::numeric_limits< NbHopsType >::max() ) const
```

Computes the distance with a limit on the number of hops from a single source node to all other nodes.

Parameters

srcId	The ID of the source node.
length←↩ MapId	The ID of the map length (as returned by UNetwork::registerLengthMap).
lim	The limit on the number of hops.
discDist	The value that should be assigned as distance between disconnected nodes.
discNbHops	The value that should be assigned as number of hops between disconnected nodes.

Returns

The distance map.

9.17.4.4 getDsim() [1/2]

```
template<typename Network = UNetwork<>, typename DistType = double, typename NbHopsType =
std::size_t>
NodeDistMapSP LinkPred::Dijkstra< Network, DistType, NbHopsType >::getDsim (
            NodeID const & srcId,
            LengthMapIdType lengthMapId,
```

```
DistType discDist = std::numeric_limits< DistType >::infinity(),
NbHopsType discNbHops = std::numeric_limits< NbHopsType >::max() ) const
```

Computes the dissimilarity of a single source node to all other nodes.

Parameters

srcId	The ID of the source node.
length↩ MapId	The ID of the map length (as returned by UNetwork::registerLengthMap).
discDist	The value that should be assigned as distance between disconnected nodes.
discNbHops	The value that should be assigned as number of hops between disconnected nodes.

Returns

The distance map.

9.17.4.5 getDsim() [2/2]

```
template<typename Network = UNetwork<>, typename DistType = double, typename NbHopsType =
std::size_t>
std::map<NodeID, NodeDistMapSP> LinkPred::Dijkstra< Network, DistType, NbHopsType >::getDsim
(
            std::set< NodeID > const & srcIds,
            LengthMapIdType lengthMapId,
            DistType discDist = std::numeric_limits< DistType >::infinity(),
            NbHopsType discNbHops = std::numeric_limits< NbHopsType >::max() ) const
```

Computes the dissimilarity of a set of nodes to all other nodes.

Parameters

srcIds	The IDs of the source node.
length↩ MapId	The ID of the map length (as returned by UNetwork::registerLengthMap).
discDist	The value that should be assigned as distance between disconnected nodes.
discNbHops	The value that should be assigned as number of hops between disconnected nodes.

Returns

The distance map.

9.17.4.6 getIndDist()

```
template<typename Network = UNetwork<>, typename DistType = double, typename NbHopsType =
std::size_t>
std::pair<DistType, NbHopsType> LinkPred::Dijkstra< Network, DistType, NbHopsType >::getInd↩
Dist (
            NodeID const & srcId,
            NodeID const & dstId,
```

```
LengthMapIdType lengthMapId,
DistType discDist = std::numeric_limits< DistType >::infinity(),
NbHopsType discNbHops = std::numeric_limits< NbHopsType >::max() ) const
```

Computes the distance between two nodes while ignoring the edge between them.

Parameters

srcId	The ID of the source node.
dstId	The ID of the destination node.
length↩ MapId	The ID of the map length (as returned by UNetwork::registerLengthMap).
discDist	The value that should be assigned as distance between disconnected nodes.
discNbHops	The value that should be assigned as number of hops between disconnected nodes.

Returns

The distance map.

9.17.4.7 getIndDistToAll()

```
template<typename Network = UNetwork<>, typename DistType = double, typename NbHopsType =
std::size_t>
NodeDistMapSP LinkPred::Dijkstra< Network, DistType, NbHopsType >::getIndDistToAll (
NodeID const & srcId,
NodeID const & dstId,
LengthMapIdType lengthMapId,
DistType discDist = std::numeric_limits< DistType >::infinity(),
NbHopsType discNbHops = std::numeric_limits< NbHopsType >::max() ) const
```

Computes the distance from a single source node to all other nodes while ignoring the edge between the source node and a specific node.

Parameters

srcId	The ID of the source node.
dstId	The ID of the destination node of the edge to be ignored.
length↩ MapId	The ID of the map length (as returned by UNetwork::registerLengthMap).
discDist	The value that should be assigned as distance between disconnected nodes.
discNbHops	The value that should be assigned as number of hops between disconnected nodes.

Returns

The distance map.

9.17.4.8 getIndDsim()

```
template<typename Network = UNetwork<>, typename DistType = double, typename NbHopsType =
std::size_t>
std::pair<DistType, NbHopsType> LinkPred::Dijkstra< Network, DistType, NbHopsType >::getInd↩
Dsim (
            NodeID const & srcId,
            NodeID const & dstId,
            LengthMapIdType lengthMapId,
            DistType discDist = std::numeric_limits< DistType >::infinity(),
            NbHopsType discNbHops = std::numeric_limits< NbHopsType >::max() ) const
```

Computes the dissimilarity between two nodes while ignoring the direction edge between them.

Parameters

srcId	The ID of the source node.
dstId	The ID of the destination node.
length↩ MapId	The ID of the map length (as returned by UNetwork::registerLengthMap).
discDist	The value that should be assigned as distance between disconnected nodes.
discNbHops	The value that should be assigned as number of hops between disconnected nodes.

Returns

The distance map.

9.17.4.9 getNet()

```
template<typename Network = UNetwork<>, typename DistType = double, typename NbHopsType =
std::size_t>
std::shared_ptr<Network const> LinkPred::Dijkstra< Network, DistType, NbHopsType >::getNet (
) const [inline]
```

Returns

The network.

9.17.4.10 getSDist()

```
template<typename Network = UNetwork<>, typename DistType = double, typename NbHopsType =
std::size_t>
template<typename InputIterator , typename OuputIterator >
void LinkPred::Dijkstra< Network, DistType, NbHopsType >::getSDist (
            InputIterator begin,
            InputIterator end,
            OuputIterator outit,
            LengthMapIdType lengthMapId,
            DistType discDist = std::numeric_limits<DistType>::infinity(),
            NbHopsType discNbHops = std::numeric_limits<NbHopsType>::max() ) const [inline]
```

Computes the inverse similarity from a list of nodes to all other nodes.

Parameters

begin	Iterator to the first element in the list of source nodes.
end	Iterator to one past the last element in the list of source nodes.
outit	Output iterator.
length←→ MapId	The ID of the map length (as returned by UNetwork::registerLengthMap).
discDist	The value that should be assigned as distance between disconnected nodes.
discNbHops	The value that should be assigned as number of hops between disconnected nodes.

9.17.4.11 getShortestPath()

```
template<typename Network = UNetwork<>, typename DistType = double, typename NbHopsType =
std::size_t>
std::pair<PathTypeSP, double> LinkPred::Dijkstra< Network, DistType, NbHopsType >::get←
ShortestPath (
            NodeID const & srcId,
            NodeID const & dstId,
            LengthMapIdType lengthMapId,
            DistType discDist = std::numeric_limits< DistType >::infinity(),
            NbHopsType discNbHops = std::numeric_limits< NbHopsType >::max() ) const
```

Finds the shortest path from a node to another.

Parameters

srcId	The ID of the source node.
dstId	The ID of the destination node.
length←→ MapId	The ID of the map length (as returned by UNetwork::registerLengthMap).
discDist	The value that should be assigned as distance between disconnected nodes.
discNbHops	The value that should be assigned as number of hops between disconnected nodes.

Returns

A pair containing a pointer to the path and its length.

9.17.4.12 operator=() [1/2]

```
template<typename Network = UNetwork<>, typename DistType = double, typename NbHopsType =
std::size_t>
Dijkstra& LinkPred::Dijkstra< Network, DistType, NbHopsType >::operator= (
            Dijkstra< Network, DistType, NbHopsType > && that )  [default]
```

Move assignment operator.

Parameters

that	The object to move.

9.17.4.13 operator=() [2/2]

```
template<typename Network = UNetwork<>, typename DistType = double, typename NbHopsType =
std::size_t>
Dijkstra& LinkPred::Dijkstra< Network, DistType, NbHopsType >::operator= (
            Dijkstra< Network, DistType, NbHopsType > const & that )  [default]
```

Copy assignment operator.

Parameters

that	The object to copy.

9.17.4.14 registerLengthMap()

```
template<typename Network = UNetwork<>, typename DistType = double, typename NbHopsType =
std::size_t>
LengthMapIdType LinkPred::Dijkstra< Network, DistType, NbHopsType >::registerLengthMap (
            EdgeLengthMapSP length )
```

Register a length map.

Parameters

length	The length map.

Returns

The ID of the length map.

9.17.4.15 setNet()

```
template<typename Network = UNetwork<>, typename DistType = double, typename NbHopsType =
std::size_t>
void LinkPred::Dijkstra< Network, DistType, NbHopsType >::setNet (
            std::shared_ptr< const Network > net )  [inline]
```

Update the network. Alllength maps are invalidated.

Parameters

net	

9.17.4.16 unregisterLengthMap()

```
template<typename Network = UNetwork<>, typename DistType = double, typename NbHopsType =
std::size_t>
void LinkPred::Dijkstra< Network, DistType, NbHopsType >::unregisterLengthMap (
            LengthMapIdType const & lengthMapId )
```

Unregister a length map.

Parameters

length↵ MapId	The ID of the length map.

The documentation for this class was generated from the following file:

- include/linkpred/graphalg/shortestpaths/dijkstra.hpp

9.18 LinkPred::DJIDPredictor< Network, EdgeRndIt, ScoreRndIt, EdgeRndOutIt > Class Template Reference

Common neighbor link predictor adapted to directed networks.

```
#include <djidpredictor.hpp>
```

Inheritance diagram for LinkPred::DJIDPredictor< Network, EdgeRndIt, ScoreRndIt, EdgeRndOutIt >:

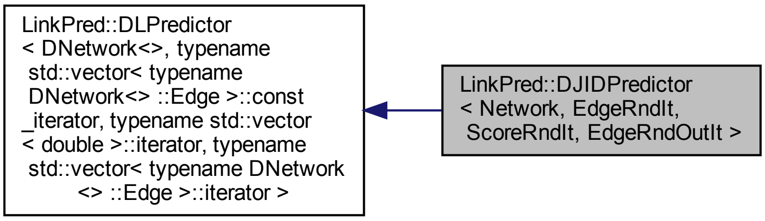

Collaboration diagram for LinkPred::DJIDPredictor< Network, EdgeRndIt, ScoreRndIt, EdgeRndOutIt >:

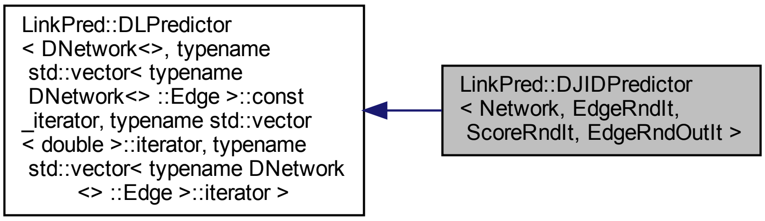

Public Member Functions

- DJIDPredictor (std::shared_ptr< Network const > net)
- DJIDPredictor (DJIDPredictor const &that)=default
- DJIDPredictor & operator= (DJIDPredictor const &that)=default
- DJIDPredictor (DJIDPredictor &&that)=default
- DJIDPredictor & operator= (DJIDPredictor &&that)=default
- virtual void init ()
- virtual void learn ()
- virtual void predict (EdgeRndIt begin, EdgeRndIt end, ScoreRndIt scores)
- virtual double score (Edge const &e)
- virtual std::size_t top (std::size_t k, EdgeRndOutIt eit, ScoreRndIt sit)
- virtual ~DJIDPredictor ()=default

Additional Inherited Members

9.18.1 Detailed Description

template<typename Network = DNetwork<>, typename EdgeRndIt = typename std::vector<typename Network::Edge>↩ ::const_iterator, typename ScoreRndIt = typename std::vector<double>::iterator, typename EdgeRndOutIt = typename std↩ ::vector<typename Network::Edge>::iterator>
class LinkPred::DJIDPredictor< Network, EdgeRndIt, ScoreRndIt, EdgeRndOutIt >

Common neighbor link predictor adapted to directed networks.

Template Parameters

Network	The network type.
Edge↩ RndIt	A random iterator type used to iterate on edges.
Score↩ RndIt	A random iterator type used to iterate on scores.

9.18.2 Constructor & Destructor Documentation

9.18.2.1 DJIDPredictor() [1/3]

template<typename Network = DNetwork<>, typename EdgeRndIt = typename std::vector<typename Network::Edge>::const_iterator, typename ScoreRndIt = typename std::vector<double>::iterator, typename EdgeRndOutIt = typename std::vector<typename Network::Edge>::iterator>
LinkPred::DJIDPredictor< Network, EdgeRndIt, ScoreRndIt, EdgeRndOutIt >::DJIDPredictor (
std::shared_ptr< Network const > *net*) [inline]

Parameters

net	The network.

9.18.2.2 DJIDPredictor() [2/3]

template<typename Network = DNetwork<>, typename EdgeRndIt = typename std::vector<typename Network::Edge>::const_iterator, typename ScoreRndIt = typename std::vector<double>::iterator, typename EdgeRndOutIt = typename std::vector<typename Network::Edge>::iterator>
LinkPred::DJIDPredictor< Network, EdgeRndIt, ScoreRndIt, EdgeRndOutIt >::DJIDPredictor (
DJIDPredictor< Network, EdgeRndIt, ScoreRndIt, EdgeRndOutIt > const & *that*)
[default]

Copy constructor.

Parameters

that	The object to copy.

9.18.2.3 DJIDPredictor() [3/3]

template<typename Network = DNetwork<>, typename EdgeRndIt = typename std::vector<typename Network::Edge>::const_iterator, typename ScoreRndIt = typename std::vector<double>::iterator, typename EdgeRndOutIt = typename std::vector<typename Network::Edge>::iterator>
LinkPred::DJIDPredictor< Network, EdgeRndIt, ScoreRndIt, EdgeRndOutIt >::DJIDPredictor (
DJIDPredictor< Network, EdgeRndIt, ScoreRndIt, EdgeRndOutIt > && *that*) [default]

Move constructor.

Parameters

that	The object to move.

9.18.2.4 ∼DJIDPredictor()

```
template<typename Network = DNetwork<>, typename EdgeRndIt = typename std::vector<typename
Network::Edge>::const_iterator, typename ScoreRndIt = typename std::vector<double>::iterator,
typename EdgeRndOutIt = typename std::vector<typename Network::Edge>::iterator>
virtual LinkPred::DJIDPredictor< Network, EdgeRndIt, ScoreRndIt, EdgeRndOutIt >::∼DJIDPredictor
( ) [virtual], [default]
```

Destructor.

9.18.3 Member Function Documentation

9.18.3.1 init()

```
template<typename Network = DNetwork<>, typename EdgeRndIt = typename std::vector<typename
Network::Edge>::const_iterator, typename ScoreRndIt = typename std::vector<double>::iterator,
typename EdgeRndOutIt = typename std::vector<typename Network::Edge>::iterator>
virtual void LinkPred::DJIDPredictor< Network, EdgeRndIt, ScoreRndIt, EdgeRndOutIt >::init ( )
[inline], [virtual]
```

Initialize the solver.

Implements LinkPred::DLPredictor< DNetwork<>, typename std::vector< typename DNetwork<> ::Edge >::const_iterator, typena

9.18.3.2 learn()

```
template<typename Network = DNetwork<>, typename EdgeRndIt = typename std::vector<typename
Network::Edge>::const_iterator, typename ScoreRndIt = typename std::vector<double>::iterator,
typename EdgeRndOutIt = typename std::vector<typename Network::Edge>::iterator>
virtual void LinkPred::DJIDPredictor< Network, EdgeRndIt, ScoreRndIt, EdgeRndOutIt >::learn (
) [inline], [virtual]
```

Learn.

Implements LinkPred::DLPredictor< DNetwork<>, typename std::vector< typename DNetwork<> ::Edge >::const_iterator, typena

9.18.3.3 operator=() [1/2]

```
template<typename Network = DNetwork<>, typename EdgeRndIt = typename std::vector<typename
Network::Edge>::const_iterator, typename ScoreRndIt = typename std::vector<double>::iterator,
typename EdgeRndOutIt = typename std::vector<typename Network::Edge>::iterator>
DJIDPredictor& LinkPred::DJIDPredictor< Network, EdgeRndIt, ScoreRndIt, EdgeRndOutIt >::operator=
(
            DJIDPredictor< Network, EdgeRndIt, ScoreRndIt, EdgeRndOutIt > && that ) [default]
```

Move assignment operator.

Parameters

that	The object to move.

9.18.3.4 operator=() [2/2]

```
template<typename Network = DNetwork<>, typename EdgeRndIt = typename std::vector<typename
Network::Edge>::const_iterator, typename ScoreRndIt = typename std::vector<double>::iterator,
typename EdgeRndOutIt = typename std::vector<typename Network::Edge>::iterator>
DJIDPredictor& LinkPred::DJIDPredictor< Network, EdgeRndIt, ScoreRndIt, EdgeRndOutIt >::operator=
(
            DJIDPredictor< Network, EdgeRndIt, ScoreRndIt, EdgeRndOutIt > const & that )
[default]
```

Copy assignment operator.

Parameters

that	The object to copy.

9.18.3.5 predict()

```
template<typename Network = DNetwork<>, typename EdgeRndIt = typename std::vector<typename
Network::Edge>::const_iterator, typename ScoreRndIt = typename std::vector<double>::iterator,
typename EdgeRndOutIt = typename std::vector<typename Network::Edge>::iterator>
virtual void LinkPred::DJIDPredictor< Network, EdgeRndIt, ScoreRndIt, EdgeRndOutIt >::predict
(
            EdgeRndIt begin,
            EdgeRndIt end,
            ScoreRndIt scores )  [virtual]
```

Predict the links.

Parameters

begin	Beginning of the links to be predicted.
end	end of the links to be predicted.
scores	Beginning of scores.

Reimplemented from LinkPred::DLPredictor< DNetwork<>, typename std::vector< typename DNetwork<> ::Edge >::const_iterat

9.18.3.6 score()

```
template<typename Network = DNetwork<>, typename EdgeRndIt = typename std::vector<typename
Network::Edge>::const_iterator, typename ScoreRndIt = typename std::vector<double>::iterator,
```

```
typename EdgeRndOutIt = typename std::vector<typename Network::Edge>::iterator>
virtual double LinkPred::DJIDPredictor< Network, EdgeRndIt, ScoreRndIt, EdgeRndOutIt >::score
(
            Edge const & e )  [virtual]
```

Compute the score of a single edge.

Parameters

e	The edge.

Returns

The score of e.

9.18.3.7 top()

```
template<typename Network = DNetwork<>, typename EdgeRndIt = typename std::vector<typename
Network::Edge>::const_iterator, typename ScoreRndIt = typename std::vector<double>::iterator,
typename EdgeRndOutIt = typename std::vector<typename Network::Edge>::iterator>
virtual std::size_t LinkPred::DJIDPredictor< Network, EdgeRndIt, ScoreRndIt, EdgeRndOutIt >↩
::top (
            std::size_t k,
            EdgeRndOutIt eit,
            ScoreRndIt sit )  [virtual]
```

Finds the k negative edges with the top score. Ties are broken randomly.

Parameters

k	The number of edges to find.
eit	An output iterator where the edges are written.
sit	An output iterator where the scores are written. The scores are written in the same order as the edges.

Returns

The number of negative edges inserted. It is the minimum between k and the number of negative edges in the network.

Reimplemented from LinkPred::DLPredictor< DNetwork<>, typename std::vector< typename DNetwork<> ::Edge >::const_iterat

The documentation for this class was generated from the following file:

- include/linkpred/predictors/directed/djidpredictor.hpp

9.19 LinkPred::DLCPPredictor< Network, EdgeRndIt, ScoreRndIt, EdgeRndOutIt > Class Template Reference

Common neighbor link predictor adapted to directed networks.

```
#include <dlcppredictor.hpp>
```

Inheritance diagram for LinkPred::DLCPPredictor< Network, EdgeRndIt, ScoreRndIt, EdgeRndOutIt >:

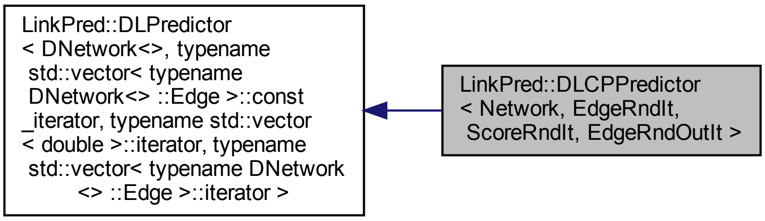

Collaboration diagram for LinkPred::DLCPPredictor< Network, EdgeRndIt, ScoreRndIt, EdgeRndOutIt >:

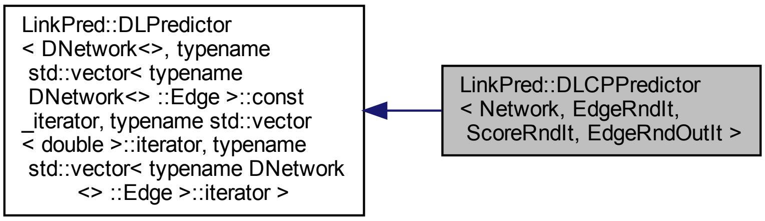

Public Member Functions

- DLCPPredictor (std::shared_ptr< Network const > net)
- DLCPPredictor (DLCPPredictor const &that)=default
- DLCPPredictor & operator= (DLCPPredictor const &that)=default
- DLCPPredictor (DLCPPredictor &&that)=default
- DLCPPredictor & operator= (DLCPPredictor &&that)=default
- virtual void init ()
- virtual void learn ()
- virtual void predict (EdgeRndIt begin, EdgeRndIt end, ScoreRndIt scores)
- virtual double score (Edge const &e)
- virtual ~DLCPPredictor ()=default

Additional Inherited Members

9.19.1 Detailed Description

template<typename Network = DNetwork<>, typename EdgeRndIt = typename std::vector<typename Network::Edge>↩ ::const_iterator, typename ScoreRndIt = typename std::vector<double>::iterator, typename EdgeRndOutIt = typename std↩ ::vector<typename Network::Edge>::iterator>
class LinkPred::DLCPPredictor< Network, EdgeRndIt, ScoreRndIt, EdgeRndOutIt >

Common neighbor link predictor adapted to directed networks.

Template Parameters

Network	The network type.
Edge↩ RndIt	A random iterator type used to iterate on edges.
Score↩ RndIt	A random iterator type used to iterate on scores.

9.19.2 Constructor & Destructor Documentation

9.19.2.1 DLCPPredictor() [1/3]

```
template<typename Network = DNetwork<>, typename EdgeRndIt = typename std::vector<typename
Network::Edge>::const_iterator, typename ScoreRndIt = typename std::vector<double>::iterator,
typename EdgeRndOutIt = typename std::vector<typename Network::Edge>::iterator>
LinkPred::DLCPPredictor< Network, EdgeRndIt, ScoreRndIt, EdgeRndOutIt >::DLCPPredictor (
            std::shared_ptr< Network const > net )  [inline]
```

Parameters

net	The network.

9.19.2.2 DLCPPredictor() [2/3]

```
template<typename Network = DNetwork<>, typename EdgeRndIt = typename std::vector<typename
Network::Edge>::const_iterator, typename ScoreRndIt = typename std::vector<double>::iterator,
typename EdgeRndOutIt = typename std::vector<typename Network::Edge>::iterator>
LinkPred::DLCPPredictor< Network, EdgeRndIt, ScoreRndIt, EdgeRndOutIt >::DLCPPredictor (
            DLCPPredictor< Network, EdgeRndIt, ScoreRndIt, EdgeRndOutIt > const & that )
[default]
```

Copy constructor.

Parameters

that	The object to copy.

9.19.2.3 DLCPPredictor() [3/3]

```
template<typename Network = DNetwork<>, typename EdgeRndIt = typename std::vector<typename
Network::Edge>::const_iterator, typename ScoreRndIt = typename std::vector<double>::iterator,
typename EdgeRndOutIt = typename std::vector<typename Network::Edge>::iterator>
LinkPred::DLCPPredictor< Network, EdgeRndIt, ScoreRndIt, EdgeRndOutIt >::DLCPPredictor (
            DLCPPredictor< Network, EdgeRndIt, ScoreRndIt, EdgeRndOutIt > && that )  [default]
```

Move constructor.

Parameters

that	The object to move.

9.19.2.4 ∼DLCPPredictor()

```
template<typename Network = DNetwork<>, typename EdgeRndIt = typename std::vector<typename
Network::Edge>::const_iterator, typename ScoreRndIt = typename std::vector<double>::iterator,
typename EdgeRndOutIt = typename std::vector<typename Network::Edge>::iterator>
virtual LinkPred::DLCPPredictor< Network, EdgeRndIt, ScoreRndIt, EdgeRndOutIt >::~DLCPPredictor
( )  [virtual], [default]
```

Finds the k negative edges with the top score. Ties are broken randomly.

Parameters

k	The number of edges to find.
eit	An output iterator where the edges are written.
sit	An output iterator where the scores are written. The scores are written in the same order as the edges.

Returns

The number of negative edges inserted. It is the minimum between k and the number of negative edges in the network. Destructor.

9.19.3 Member Function Documentation

9.19.3.1 init()

```
template<typename Network = DNetwork<>, typename EdgeRndIt = typename std::vector<typename
Network::Edge>::const_iterator, typename ScoreRndIt = typename std::vector<double>::iterator,
typename EdgeRndOutIt = typename std::vector<typename Network::Edge>::iterator>
virtual void LinkPred::DLCPPredictor< Network, EdgeRndIt, ScoreRndIt, EdgeRndOutIt >::init ( )
[inline], [virtual]
```

Initialize the solver.

Implements LinkPred::DLPredictor< DNetwork<>, typename std::vector< typename DNetwork<> ::Edge >::const_iterator, typena

9.19.3.2 learn()

```
template<typename Network = DNetwork<>, typename EdgeRndIt = typename std::vector<typename
Network::Edge>::const_iterator, typename ScoreRndIt = typename std::vector<double>::iterator,
typename EdgeRndOutIt = typename std::vector<typename Network::Edge>::iterator>
virtual void LinkPred::DLCPPredictor< Network, EdgeRndIt, ScoreRndIt, EdgeRndOutIt >::learn (
) [inline], [virtual]
```

Learn.

Implements LinkPred::DLPredictor< DNetwork<>, typename std::vector< typename DNetwork<> ::Edge >::const_iterator, typena

9.19.3.3 operator=() [1/2]

```
template<typename Network = DNetwork<>, typename EdgeRndIt = typename std::vector<typename
Network::Edge>::const_iterator, typename ScoreRndIt = typename std::vector<double>::iterator,
typename EdgeRndOutIt = typename std::vector<typename Network::Edge>::iterator>
DLCPPredictor& LinkPred::DLCPPredictor< Network, EdgeRndIt, ScoreRndIt, EdgeRndOutIt >::operator=
(
            DLCPPredictor< Network, EdgeRndIt, ScoreRndIt, EdgeRndOutIt > && that )  [default]
```

Move assignment operator.

Parameters

that	The object to move.

9.19.3.4 operator=() [2/2]

```
template<typename Network = DNetwork<>, typename EdgeRndIt = typename std::vector<typename
Network::Edge>::const_iterator, typename ScoreRndIt = typename std::vector<double>::iterator,
typename EdgeRndOutIt = typename std::vector<typename Network::Edge>::iterator>
```

```
DLCPPredictor& LinkPred::DLCPPredictor< Network, EdgeRndIt, ScoreRndIt, EdgeRndOutIt >::operator=
(
            DLCPPredictor< Network, EdgeRndIt, ScoreRndIt, EdgeRndOutIt > const & that )
[default]
```

Copy assignment operator.

Parameters

that	The object to copy.

9.19.3.5 predict()

```
template<typename Network = DNetwork<>, typename EdgeRndIt = typename std::vector<typename
Network::Edge>::const_iterator, typename ScoreRndIt = typename std::vector<double>::iterator,
typename EdgeRndOutIt = typename std::vector<typename Network::Edge>::iterator>
virtual void LinkPred::DLCPPredictor< Network, EdgeRndIt, ScoreRndIt, EdgeRndOutIt >::predict
(
            EdgeRndIt begin,
            EdgeRndIt end,
            ScoreRndIt scores )  [virtual]
```

Predict the links.

Parameters

begin	Beginning of the links to be predicted.
end	end of the links to be predicted.
scores	Beginning of scores.

Reimplemented from LinkPred::DLPredictor< DNetwork<>, typename std::vector< typename DNetwork<> ::Edge >::const_iterat

9.19.3.6 score()

```
template<typename Network = DNetwork<>, typename EdgeRndIt = typename std::vector<typename
Network::Edge>::const_iterator, typename ScoreRndIt = typename std::vector<double>::iterator,
typename EdgeRndOutIt = typename std::vector<typename Network::Edge>::iterator>
virtual double LinkPred::DLCPPredictor< Network, EdgeRndIt, ScoreRndIt, EdgeRndOutIt >::score
(
            Edge const & e )  [virtual]
```

Compute the score of a single edge.

Parameters

e	The edge.

Returns

The score of e.

The documentation for this class was generated from the following file:

- include/linkpred/predictors/directed/dlcppredictor.hpp

9.20 LinkPred::DLHNPredictor< Network, EdgeRndIt, ScoreRndIt, EdgeRndOutIt > Class Template Reference

Common neighbor link predictor adapted to directed networks.

```
#include <dlhnpredictor.hpp>
```

Inheritance diagram for LinkPred::DLHNPredictor< Network, EdgeRndIt, ScoreRndIt, EdgeRndOutIt >:

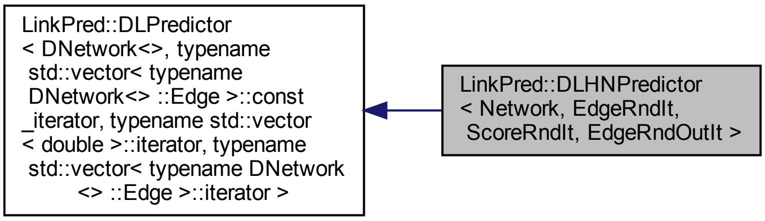

Collaboration diagram for LinkPred::DLHNPredictor< Network, EdgeRndIt, ScoreRndIt, EdgeRndOutIt >:

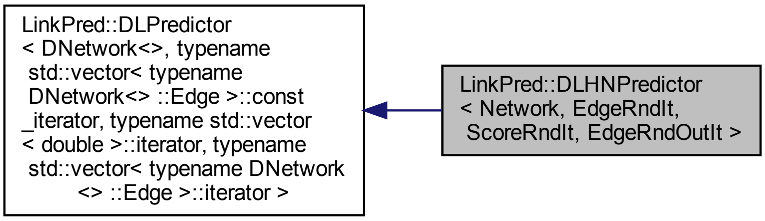

Public Member Functions

- DLHNPredictor (std::shared_ptr< Network const > net)
- DLHNPredictor (DLHNPredictor const &that)=default
- DLHNPredictor & operator= (DLHNPredictor const &that)=default
- DLHNPredictor (DLHNPredictor &&that)=default
- DLHNPredictor & operator= (DLHNPredictor &&that)=default
- virtual void init ()
- virtual void learn ()
- virtual void predict (EdgeRndIt begin, EdgeRndIt end, ScoreRndIt scores)
- virtual double score (Edge const &e)
- virtual std::size_t top (std::size_t k, EdgeRndOutIt eit, ScoreRndIt sit)
- virtual ∼DLHNPredictor ()=default

Additional Inherited Members

9.20.1 Detailed Description

template<typename Network = DNetwork<>, typename EdgeRndIt = typename std::vector<typename Network::Edge>↩ ::const_iterator, typename ScoreRndIt = typename std::vector<double>::iterator, typename EdgeRndOutIt = typename std↩ ::vector<typename Network::Edge>::iterator>
class LinkPred::DLHNPredictor< Network, EdgeRndIt, ScoreRndIt, EdgeRndOutIt >

Common neighbor link predictor adapted to directed networks.

Template Parameters

Network	The network type.
Edge↩ RndIt	A random iterator type used to iterate on edges.
Score↩ RndIt	A random iterator type used to iterate on scores.

9.20.2 Constructor & Destructor Documentation

9.20.2.1 DLHNPredictor() [1/3]

```
template<typename Network = DNetwork<>, typename EdgeRndIt = typename std::vector<typename
Network::Edge>::const_iterator, typename ScoreRndIt = typename std::vector<double>::iterator,
typename EdgeRndOutIt = typename std::vector<typename Network::Edge>::iterator>
LinkPred::DLHNPredictor< Network, EdgeRndIt, ScoreRndIt, EdgeRndOutIt >::DLHNPredictor (
            std::shared_ptr< Network const > net )  [inline]
```

Parameters

net	The network.

9.20.2.2 DLHNPredictor() [2/3]

```
template<typename Network = DNetwork<>, typename EdgeRndIt = typename std::vector<typename
Network::Edge>::const_iterator, typename ScoreRndIt = typename std::vector<double>::iterator,
typename EdgeRndOutIt = typename std::vector<typename Network::Edge>::iterator>
LinkPred::DLHNPredictor< Network, EdgeRndIt, ScoreRndIt, EdgeRndOutIt >::DLHNPredictor (
            DLHNPredictor< Network, EdgeRndIt, ScoreRndIt, EdgeRndOutIt > const & that )
[default]
```

Copy constructor.

Parameters

that	The object to copy.

9.20.2.3 DLHNPredictor() [3/3]

```
template<typename Network = DNetwork<>, typename EdgeRndIt = typename std::vector<typename
Network::Edge>::const_iterator, typename ScoreRndIt = typename std::vector<double>::iterator,
typename EdgeRndOutIt = typename std::vector<typename Network::Edge>::iterator>
LinkPred::DLHNPredictor< Network, EdgeRndIt, ScoreRndIt, EdgeRndOutIt >::DLHNPredictor (
            DLHNPredictor< Network, EdgeRndIt, ScoreRndIt, EdgeRndOutIt > && that )  [default]
```

Move constructor.

Parameters

that	The object to move.

9.20.2.4 ∼DLHNPredictor()

```
template<typename Network = DNetwork<>, typename EdgeRndIt = typename std::vector<typename
Network::Edge>::const_iterator, typename ScoreRndIt = typename std::vector<double>::iterator,
typename EdgeRndOutIt = typename std::vector<typename Network::Edge>::iterator>
virtual LinkPred::DLHNPredictor< Network, EdgeRndIt, ScoreRndIt, EdgeRndOutIt >::~DLHNPredictor
( )  [virtual], [default]
```

Destructor.

9.20.3 Member Function Documentation

9.20.3.1 init()

```
template<typename Network = DNetwork<>, typename EdgeRndIt = typename std::vector<typename
Network::Edge>::const_iterator, typename ScoreRndIt = typename std::vector<double>::iterator,
typename EdgeRndOutIt = typename std::vector<typename Network::Edge>::iterator>
virtual void LinkPred::DLHNPredictor< Network, EdgeRndIt, ScoreRndIt, EdgeRndOutIt >::init ( )
[inline], [virtual]
```

Initialize the solver.

Implements LinkPred::DLPredictor< DNetwork<>, typename std::vector< typename DNetwork<> ::Edge >::const_iterator, typena

9.20.3.2 learn()

```
template<typename Network = DNetwork<>, typename EdgeRndIt = typename std::vector<typename
Network::Edge>::const_iterator, typename ScoreRndIt = typename std::vector<double>::iterator,
typename EdgeRndOutIt = typename std::vector<typename Network::Edge>::iterator>
virtual void LinkPred::DLHNPredictor< Network, EdgeRndIt, ScoreRndIt, EdgeRndOutIt >::learn (
) [inline], [virtual]
```

Learn.

Implements LinkPred::DLPredictor< DNetwork<>, typename std::vector< typename DNetwork<> ::Edge >::const_iterator, typena

9.20.3.3 operator=() [1/2]

```
template<typename Network = DNetwork<>, typename EdgeRndIt = typename std::vector<typename
Network::Edge>::const_iterator, typename ScoreRndIt = typename std::vector<double>::iterator,
typename EdgeRndOutIt = typename std::vector<typename Network::Edge>::iterator>
DLHNPredictor& LinkPred::DLHNPredictor< Network, EdgeRndIt, ScoreRndIt, EdgeRndOutIt >::operator=
(
            DLHNPredictor< Network, EdgeRndIt, ScoreRndIt, EdgeRndOutIt > && that ) [default]
```

Move assignment operator.

Parameters

that	The object to move.

9.20.3.4 operator=() [2/2]

```
template<typename Network = DNetwork<>, typename EdgeRndIt = typename std::vector<typename
Network::Edge>::const_iterator, typename ScoreRndIt = typename std::vector<double>::iterator,
typename EdgeRndOutIt = typename std::vector<typename Network::Edge>::iterator>
```

```
DLHNPredictor& LinkPred::DLHNPredictor< Network, EdgeRndIt, ScoreRndIt, EdgeRndOutIt >::operator=
(
             DLHNPredictor< Network, EdgeRndIt, ScoreRndIt, EdgeRndOutIt > const & that )
[default]
```

Copy assignment operator.

Parameters

that	The object to copy.

9.20.3.5 predict()

```
template<typename Network = DNetwork<>, typename EdgeRndIt = typename std::vector<typename
Network::Edge>::const_iterator, typename ScoreRndIt = typename std::vector<double>::iterator,
typename EdgeRndOutIt = typename std::vector<typename Network::Edge>::iterator>
virtual void LinkPred::DLHNPredictor< Network, EdgeRndIt, ScoreRndIt, EdgeRndOutIt >::predict
(
             EdgeRndIt begin,
             EdgeRndIt end,
             ScoreRndIt scores )  [virtual]
```

Predict the links.

Parameters

begin	Beginning of the links to be predicted.
end	end of the links to be predicted.
scores	Beginning of scores.

Reimplemented from LinkPred::DLPredictor< DNetwork<>, typename std::vector< typename DNetwork<> ::Edge >::const_iterat

9.20.3.6 score()

```
template<typename Network = DNetwork<>, typename EdgeRndIt = typename std::vector<typename
Network::Edge>::const_iterator, typename ScoreRndIt = typename std::vector<double>::iterator,
typename EdgeRndOutIt = typename std::vector<typename Network::Edge>::iterator>
virtual double LinkPred::DLHNPredictor< Network, EdgeRndIt, ScoreRndIt, EdgeRndOutIt >::score
(
             Edge const & e )  [virtual]
```

Compute the score of a single edge.

Parameters

e	The edge.

Returns

The score of e.

9.20.3.7 top()

```
template<typename Network = DNetwork<>, typename EdgeRndIt = typename std::vector<typename
Network::Edge>::const_iterator, typename ScoreRndIt = typename std::vector<double>::iterator,
typename EdgeRndOutIt = typename std::vector<typename Network::Edge>::iterator>
virtual std::size_t LinkPred::DLHNPredictor< Network, EdgeRndIt, ScoreRndIt, EdgeRndOutIt >↩
::top (
            std::size_t k,
            EdgeRndOutIt eit,
            ScoreRndIt sit )  [virtual]
```

Finds the k negative edges with the top score. Ties are broken randomly.

Parameters

k	The number of edges to find.
eit	An output iterator where the edges are written.
sit	An output iterator where the scores are written. The scores are written in the same order as the edges.

Returns

The number of negative edges inserted. It is the minimum between k and the number of negative edges in the network.

Reimplemented from LinkPred::DLPredictor< DNetwork<>, typename std::vector< typename DNetwork<> ::Edge >::const_iterat

The documentation for this class was generated from the following file:

- include/linkpred/predictors/directed/dlhnpredictor.hpp

9.21 LinkPred::DLPredictor< NetworkT, EdgeRndItT, ScoreRndItT, EdgeRndOutItT > Class Template Reference

The interface of a link predictor in a directed network.

`#include <dlpredictor.hpp>`

Public Types

- using Network = NetworkT
- using EdgeRndIt = EdgeRndItT
- using ScoreRndIt = ScoreRndItT
- using EdgeRndOutIt = EdgeRndOutItT
- using NodeID = typename Network::NodeID
- using Edge = typename Network::Edge

Public Member Functions

- DLPredictor (std::shared_ptr< Network const > net)
- DLPredictor (DLPredictor const &that)=default
- DLPredictor & operator= (DLPredictor const &that)=default
- DLPredictor (DLPredictor &&that)=default
- DLPredictor & operator= (DLPredictor &&that)=default
- virtual void init ()=0
- virtual void learn ()=0
- virtual void predict (EdgeRndIt begin, EdgeRndIt end, ScoreRndIt scores)
- virtual std::pair< typename Network::NonEdgeIt, typename Network::NonEdgeIt > predictNeg (ScoreRndIt scores)
- virtual double score (Edge const &e)=0
- virtual std::size_t top (std::size_t k, EdgeRndOutIt eit, ScoreRndIt sit)
- auto getNet () const
- const std::string & getName () const
- void setName (const std::string &name)
- virtual ~DLPredictor ()=default

9.21.1 Detailed Description

template<typename NetworkT = DNetwork<>, typename EdgeRndItT = typename std::vector<typename NetworkT::Edge>::const_iterator, typename ScoreRndItT = typename std::vector<double>::iterator, typename EdgeRndOutItT = typename std::vector<typename NetworkT::Edge>::iterator>
class LinkPred::DLPredictor< NetworkT, EdgeRndItT, ScoreRndItT, EdgeRndOutItT >

The interface of a link predictor in a directed network.

Template Parameters

NetworkT	The network type.
EdgeRndItT	A random iterator type used to iterate on edges.
ScoreRndItT	A random iterator type used to iterate on scores.

Parameters

EdgeRndOutItT	A random output iterator to write edges.

9.21.2 Member Typedef Documentation

9.21.2.1 Edge

```
template<typename NetworkT = DNetwork<>, typename EdgeRndItT = typename std::vector<typename NetworkT::Edge>::const_iterator, typename ScoreRndItT = typename std::vector<double>::iterator, typename EdgeRndOutItT = typename std::vector<typename NetworkT::Edge>::iterator>
using LinkPred::DLPredictor< NetworkT, EdgeRndItT, ScoreRndItT, EdgeRndOutItT >::Edge = typename Network::Edge
```

The edges type.

9.21.2.2 EdgeRndIt

```
template<typename NetworkT = DNetwork<>, typename EdgeRndItT = typename std::vector<typename
NetworkT::Edge>::const_iterator, typename ScoreRndItT = typename std::vector<double>::iterator,
typename EdgeRndOutItT = typename std::vector<typename NetworkT::Edge>::iterator>
using LinkPred::DLPredictor< NetworkT, EdgeRndItT, ScoreRndItT, EdgeRndOutItT >::EdgeRndIt =
EdgeRndItT
```

A random iterator type used to iterate on edges.

9.21.2.3 EdgeRndOutIt

```
template<typename NetworkT = DNetwork<>, typename EdgeRndItT = typename std::vector<typename
NetworkT::Edge>::const_iterator, typename ScoreRndItT = typename std::vector<double>::iterator,
typename EdgeRndOutItT = typename std::vector<typename NetworkT::Edge>::iterator>
using LinkPred::DLPredictor< NetworkT, EdgeRndItT, ScoreRndItT, EdgeRndOutItT >::EdgeRndOutIt
= EdgeRndOutItT
```

A random output iterator to write edges.

9.21.2.4 Network

```
template<typename NetworkT = DNetwork<>, typename EdgeRndItT = typename std::vector<typename
NetworkT::Edge>::const_iterator, typename ScoreRndItT = typename std::vector<double>::iterator,
typename EdgeRndOutItT = typename std::vector<typename NetworkT::Edge>::iterator>
using LinkPred::DLPredictor< NetworkT, EdgeRndItT, ScoreRndItT, EdgeRndOutItT >::Network =
NetworkT
```

The network type.

9.21.2.5 NodeID

```
template<typename NetworkT = DNetwork<>, typename EdgeRndItT = typename std::vector<typename
NetworkT::Edge>::const_iterator, typename ScoreRndItT = typename std::vector<double>::iterator,
typename EdgeRndOutItT = typename std::vector<typename NetworkT::Edge>::iterator>
using LinkPred::DLPredictor< NetworkT, EdgeRndItT, ScoreRndItT, EdgeRndOutItT >::NodeID =
typename Network::NodeID
```

The node IDs type.

9.21.2.6 ScoreRndIt

```
template<typename NetworkT = DNetwork<>, typename EdgeRndItT = typename std::vector<typename
NetworkT::Edge>::const_iterator, typename ScoreRndItT = typename std::vector<double>::iterator,
typename EdgeRndOutItT = typename std::vector<typename NetworkT::Edge>::iterator>
using LinkPred::DLPredictor< NetworkT, EdgeRndItT, ScoreRndItT, EdgeRndOutItT >::ScoreRndIt =
ScoreRndItT
```

A random iterator type used to iterate on scores.

9.21.3 Constructor & Destructor Documentation

9.21.3.1 DLPredictor() [1/3]

```
template<typename NetworkT = DNetwork<>, typename EdgeRndItT = typename std::vector<typename
NetworkT::Edge>::const_iterator, typename ScoreRndItT = typename std::vector<double>::iterator,
typename EdgeRndOutItT = typename std::vector<typename NetworkT::Edge>::iterator>
LinkPred::DLPredictor< NetworkT, EdgeRndItT, ScoreRndItT, EdgeRndOutItT >::DLPredictor (
            std::shared_ptr< Network const > net )  [inline]
```

Constructor.

Parameters

net	The network.

9.21.3.2 DLPredictor() [2/3]

```
template<typename NetworkT = DNetwork<>, typename EdgeRndItT = typename std::vector<typename
NetworkT::Edge>::const_iterator, typename ScoreRndItT = typename std::vector<double>::iterator,
typename EdgeRndOutItT = typename std::vector<typename NetworkT::Edge>::iterator>
LinkPred::DLPredictor< NetworkT, EdgeRndItT, ScoreRndItT, EdgeRndOutItT >::DLPredictor (
            DLPredictor< NetworkT, EdgeRndItT, ScoreRndItT, EdgeRndOutItT > const & that )
[default]
```

Copy constructor.

Parameters

that	The object to copy.

9.21.3.3 DLPredictor() [3/3]

```
template<typename NetworkT = DNetwork<>, typename EdgeRndItT = typename std::vector<typename
NetworkT::Edge>::const_iterator, typename ScoreRndItT = typename std::vector<double>::iterator,
typename EdgeRndOutItT = typename std::vector<typename NetworkT::Edge>::iterator>
LinkPred::DLPredictor< NetworkT, EdgeRndItT, ScoreRndItT, EdgeRndOutItT >::DLPredictor (
            DLPredictor< NetworkT, EdgeRndItT, ScoreRndItT, EdgeRndOutItT > && that )  [default]
```

Move constructor.

Parameters

that	The object to move.

9.21.3.4 ∼DLPredictor()

```
template<typename NetworkT = DNetwork<>, typename EdgeRndItT = typename std::vector<typename
NetworkT::Edge>::const_iterator, typename ScoreRndItT = typename std::vector<double>::iterator,
typename EdgeRndOutItT = typename std::vector<typename NetworkT::Edge>::iterator>
virtual LinkPred::DLPredictor< NetworkT, EdgeRndItT, ScoreRndItT, EdgeRndOutItT >::∼DLPredictor
( ) [virtual], [default]
```

Destructor.

9.21.4 Member Function Documentation

9.21.4.1 getName()

```
template<typename NetworkT = DNetwork<>, typename EdgeRndItT = typename std::vector<typename
NetworkT::Edge>::const_iterator, typename ScoreRndItT = typename std::vector<double>::iterator,
typename EdgeRndOutItT = typename std::vector<typename NetworkT::Edge>::iterator>
const std::string& LinkPred::DLPredictor< NetworkT, EdgeRndItT, ScoreRndItT, EdgeRndOutItT >↩
::getName ( ) const [inline]
```

Returns

The name of the predictor.

9.21.4.2 getNet()

```
template<typename NetworkT = DNetwork<>, typename EdgeRndItT = typename std::vector<typename
NetworkT::Edge>::const_iterator, typename ScoreRndItT = typename std::vector<double>::iterator,
typename EdgeRndOutItT = typename std::vector<typename NetworkT::Edge>::iterator>
auto LinkPred::DLPredictor< NetworkT, EdgeRndItT, ScoreRndItT, EdgeRndOutItT >::getNet ( )
const [inline]
```

Returns

The network.

9.21.4.3 init()

```
template<typename NetworkT = DNetwork<>, typename EdgeRndItT = typename std::vector<typename
NetworkT::Edge>::const_iterator, typename ScoreRndItT = typename std::vector<double>::iterator,
typename EdgeRndOutItT = typename std::vector<typename NetworkT::Edge>::iterator>
virtual void LinkPred::DLPredictor< NetworkT, EdgeRndItT, ScoreRndItT, EdgeRndOutItT >::init (
)  [pure virtual]
```

Initialize the solver.

Implemented in LinkPred::DPSTPredictor< Network, EdgeRndIt, ScoreRndIt, EdgeRndOutIt >, LinkPred::DADAPredictor< Network
LinkPred::DCNEPredictor< Network, EdgeRndIt, ScoreRndIt, EdgeRndOutIt >, LinkPred::DHDIPredictor< Network, EdgeRndIt, Sc
LinkPred::DHPIPredictor< Network, EdgeRndIt, ScoreRndIt, EdgeRndOutIt >, LinkPred::DJIDPredictor< Network, EdgeRndIt, Scor
LinkPred::DLCPPredictor< Network, EdgeRndIt, ScoreRndIt, EdgeRndOutIt >, LinkPred::DLHNPredictor< Network, EdgeRndIt, Sc
LinkPred::DPATPredictor< Network, EdgeRndIt, ScoreRndIt, EdgeRndOutIt >, LinkPred::DSAIPredictor< Network, EdgeRndIt, Sco
and LinkPred::DSOIPredictor< Network, EdgeRndIt, ScoreRndIt, EdgeRndOutIt >.

9.21.4.4 learn()

```
template<typename NetworkT = DNetwork<>, typename EdgeRndItT = typename std::vector<typename
NetworkT::Edge>::const_iterator, typename ScoreRndItT = typename std::vector<double>::iterator,
typename EdgeRndOutItT = typename std::vector<typename NetworkT::Edge>::iterator>
virtual void LinkPred::DLPredictor< NetworkT, EdgeRndItT, ScoreRndItT, EdgeRndOutItT >::learn
( )  [pure virtual]
```

Learning.

Implemented in LinkPred::DPSTPredictor< Network, EdgeRndIt, ScoreRndIt, EdgeRndOutIt >, LinkPred::DADAPredictor< Network
LinkPred::DCNEPredictor< Network, EdgeRndIt, ScoreRndIt, EdgeRndOutIt >, LinkPred::DHDIPredictor< Network, EdgeRndIt, Sc
LinkPred::DHPIPredictor< Network, EdgeRndIt, ScoreRndIt, EdgeRndOutIt >, LinkPred::DJIDPredictor< Network, EdgeRndIt, Scor
LinkPred::DLCPPredictor< Network, EdgeRndIt, ScoreRndIt, EdgeRndOutIt >, LinkPred::DLHNPredictor< Network, EdgeRndIt, Sc
LinkPred::DPATPredictor< Network, EdgeRndIt, ScoreRndIt, EdgeRndOutIt >, LinkPred::DSAIPredictor< Network, EdgeRndIt, Sco
and LinkPred::DSOIPredictor< Network, EdgeRndIt, ScoreRndIt, EdgeRndOutIt >.

9.21.4.5 operator=() [1/2]

```
template<typename NetworkT = DNetwork<>, typename EdgeRndItT = typename std::vector<typename
NetworkT::Edge>::const_iterator, typename ScoreRndItT = typename std::vector<double>::iterator,
typename EdgeRndOutItT = typename std::vector<typename NetworkT::Edge>::iterator>
DLPredictor& LinkPred::DLPredictor< NetworkT, EdgeRndItT, ScoreRndItT, EdgeRndOutItT >::operator=
(
            DLPredictor< NetworkT, EdgeRndItT, ScoreRndItT, EdgeRndOutItT > && that )  [default]
```

Move assignment operator.

Parameters

that	The object to move.

9.21.4.6 operator=() [2/2]

```
template<typename NetworkT = DNetwork<>, typename EdgeRndItT = typename std::vector<typename
NetworkT::Edge>::const_iterator, typename ScoreRndItT = typename std::vector<double>::iterator,
typename EdgeRndOutItT = typename std::vector<typename NetworkT::Edge>::iterator>
DLPredictor& LinkPred::DLPredictor< NetworkT, EdgeRndItT, ScoreRndItT, EdgeRndOutItT >::operator=
(
            DLPredictor< NetworkT, EdgeRndItT, ScoreRndItT, EdgeRndOutItT > const & that )
[default]
```

Copy assignment operator.

Parameters

that	The object to copy.

9.21.4.7 predict()

```
template<typename NetworkT = DNetwork<>, typename EdgeRndItT = typename std::vector<typename
NetworkT::Edge>::const_iterator, typename ScoreRndItT = typename std::vector<double>::iterator,
typename EdgeRndOutItT = typename std::vector<typename NetworkT::Edge>::iterator>
virtual void LinkPred::DLPredictor< NetworkT, EdgeRndItT, ScoreRndItT, EdgeRndOutItT >::predict
(
            EdgeRndIt begin,
            EdgeRndIt end,
            ScoreRndIt scores )  [inline], [virtual]
```

Predict links.

Parameters

begin	Iterator to the first edge to be predicted.
end	end Iterator to one past the last edge to be predicted.
scores	Random output iterator to store the scores.

Reimplemented in LinkPred::DADAPredictor< Network, EdgeRndIt, ScoreRndIt, EdgeRndOutIt >, LinkPred::DCNEPredictor< Netw[illegible]
LinkPred::DHDIPredictor< Network, EdgeRndIt, ScoreRndIt, EdgeRndOutIt >, LinkPred::DHPIPredictor< Network, EdgeRndIt, Sco[illegible]
LinkPred::DJIDPredictor< Network, EdgeRndIt, ScoreRndIt, EdgeRndOutIt >, LinkPred::DLCPPredictor< Network, EdgeRndIt, Sco[illegible]
LinkPred::DLHNPredictor< Network, EdgeRndIt, ScoreRndIt, EdgeRndOutIt >, LinkPred::DPATPredictor< Network, EdgeRndIt, Sc[illegible]
LinkPred::DSAIPredictor< Network, EdgeRndIt, ScoreRndIt, EdgeRndOutIt >, and LinkPred::DSOIPredictor< Network, EdgeRndIt, [illegible]

9.21.4.8 predictNeg()

```
template<typename NetworkT = DNetwork<>, typename EdgeRndItT = typename std::vector<typename
NetworkT::Edge>::const_iterator, typename ScoreRndItT = typename std::vector<double>::iterator,
```

```
typename EdgeRndOutItT = typename std::vector<typename NetworkT::Edge>::iterator>
virtual std::pair<typename Network::NonEdgeIt, typename Network::NonEdgeIt> LinkPred::DLPredictor<
NetworkT, EdgeRndItT, ScoreRndItT, EdgeRndOutItT >::predictNeg (
            ScoreRndIt scores )  [inline], [virtual]
```

Predict score for all negative (non-existing) links in the network.

Parameters

scores	Random output iterator to store the scores.

Returns

A pair of iterators begin and end to the range of non-existing links predicted by the method.

9.21.4.9 score()

```
template<typename NetworkT = DNetwork<>, typename EdgeRndItT = typename std::vector<typename
NetworkT::Edge>::const_iterator, typename ScoreRndItT = typename std::vector<double>::iterator,
typename EdgeRndOutItT = typename std::vector<typename NetworkT::Edge>::iterator>
virtual double LinkPred::DLPredictor< NetworkT, EdgeRndItT, ScoreRndItT, EdgeRndOutItT >↩
::score (
            Edge const & e )  [pure virtual]
```

Compute the score of a single edge.

Parameters

e	The edge.

Returns

The score of e.

9.21.4.10 setName()

```
template<typename NetworkT = DNetwork<>, typename EdgeRndItT = typename std::vector<typename
NetworkT::Edge>::const_iterator, typename ScoreRndItT = typename std::vector<double>::iterator,
typename EdgeRndOutItT = typename std::vector<typename NetworkT::Edge>::iterator>
void LinkPred::DLPredictor< NetworkT, EdgeRndItT, ScoreRndItT, EdgeRndOutItT >::setName (
            const std::string & name )  [inline]
```

Set the name of the predictor.

Parameters

name	The new name of the predictor.

9.21.4.11 top()

```
template<typename NetworkT = DNetwork<>, typename EdgeRndItT = typename std::vector<typename
NetworkT::Edge>::const_iterator, typename ScoreRndItT = typename std::vector<double>::iterator,
typename EdgeRndOutItT = typename std::vector<typename NetworkT::Edge>::iterator>
virtual std::size_t LinkPred::DLPredictor< NetworkT, EdgeRndItT, ScoreRndItT, EdgeRndOutItT
>::top (
            std::size_t k,
            EdgeRndOutIt eit,
            ScoreRndIt sit )  [inline], [virtual]
```

Finds the k negative edges with the top score. Ties are broken randomly.

Parameters

k	The number of edges to find.
eit	An output iterator where the edges are written.
sit	An output iterator where the scores are written. The scores are written in the same order as the edges.

Returns

The number of negative edges inserted. It is the minimum between k and the number of negative edges in the network.

Reimplemented in LinkPred::DADAPredictor< Network, EdgeRndIt, ScoreRndIt, EdgeRndOutIt >, LinkPred::DCNEPredictor< Netw[illegible]
LinkPred::DHDIPredictor< Network, EdgeRndIt, ScoreRndIt, EdgeRndOutIt >, LinkPred::DHPIPredictor< Network, EdgeRndIt, Sco[illegible]
LinkPred::DJIDPredictor< Network, EdgeRndIt, ScoreRndIt, EdgeRndOutIt >, LinkPred::DLHNPredictor< Network, EdgeRndIt, Sco[illegible]
LinkPred::DSAIPredictor< Network, EdgeRndIt, ScoreRndIt, EdgeRndOutIt >, and LinkPred::DSOIPredictor< Network, EdgeRndIt, [illegible]

The documentation for this class was generated from the following file:

- include/linkpred/predictors/directed/dlpredictor.hpp

9.22 LinkPred::DNetwork< LabelT, NodeIDT, EdgeT > Class Template Reference

This class represents a directed network in the sense of graph theory.

```
#include <dnetwork.hpp>
```

Classes

- class EdgeMap

  An edge map.
- class NodeDegIt

  Node-degree iterator. This class can be used to iterate over pairs of node IDs and in and out degrees.
- class NodeMap

  A node map.
- class NodeSMap

  A sparse node map.
- class NonEdgeIt

  Nonedges iterator.
- class RndEdgeIt

  Randomized edges iterator.
- class RndNodeIt

  Randomized Nodes iterator.
- class RndNonEdgeIt

  Randomized nonedges iterator.

Public Types

- using Label = LabelT
- using NodeID = NodeIDT
- using Edge = EdgeT
- using LabelIt = typename Bhmap< Label, NodeID >::k_const_iterator
- using NodeIt = typename Bhmap< Label, NodeID >::p_const_iterator
- using EdgeIt = typename std::vector< Edge >::const_iterator
- template<typename ValueT >
  using NodeMapSP = std::shared_ptr< NodeMap< ValueT > >
- template<typename ValueT >
  using NodeSMapSP = std::shared_ptr< NodeSMap< ValueT > >
- template<typename ValueT >
  using EdgeMapSP = std::shared_ptr< EdgeMap< ValueT > >

Public Member Functions

- DNetwork ()=default
- DNetwork (std::vector< std::pair< Label, Label >> const &edges)
- DNetwork (DNetwork const &that)=default
- DNetwork & operator= (DNetwork const &that)=default
- DNetwork (DNetwork &&that)=default
- DNetwork & operator= (DNetwork &&that)=default
- std::pair< NodeID, bool > addNode (Label const &nodeId)
- NodeID getID (Label const &label) const
- Label getLabel (NodeID const &iid) const
- LabelIt findLabel (Label const &label) const
- NodeIt findNode (NodeID const &iid) const
- void addEdge (NodeID const &i, NodeID const &j)
- std::size_t coupleOrd (Edge const &e) const
- std::size_t coupleAtOrd (std::size_t ord) const
- EdgeIt neighbBegin (NodeID const &iid) const
- EdgeIt neighbEnd (NodeID const &iid) const

- EdgeIt outNeighborsBegin (NodeID const &iid) const
- EdgeIt outNeighborsEnd (NodeID const &iid) const
- EdgeIt inNeighborsBegin (NodeID const &iid) const
- EdgeIt inNeighborsEnd (NodeID const &iid) const
- std::size_t getDeg (NodeID const &iid) const
- std::pair< std::size_t, std::size_t > getInOutDeg (NodeID const &iid) const
- std::size_t getOutDeg (NodeID const &iid) const
- std::size_t getInDeg (NodeID const &iid) const
- bool isEdge (NodeID const &ii, NodeID const &ij) const
- bool isEdge (Edge const &edge) const
- std::size_t getNbNodes () const
- std::size_t getNbCouples () const
- std::size_t getNbEdges () const
- std::size_t getNbNonEdges () const
- double getAvgDeg () const
- std::size_t getMaxDeg () const
- std::size_t getMinDeg () const
- double getAvgOutDeg () const
- std::size_t getMaxOutDeg () const
- std::size_t getMinOutDeg () const
- double getAvgInDeg () const
- std::size_t getMaxInDeg () const
- std::size_t getMinInDeg () const
- void assemble ()
- void shuffle (long int seed)
- LabelIt labelsBegin () const
- LabelIt labelsEnd () const
- NodeIt nodesBegin () const
- NodeIt nodesEnd () const
- NodeDegIt nodesDegBegin () const
- NodeDegIt nodesDegEnd () const
- EdgeIt edgesBegin () const
- EdgeIt edgesEnd () const
- EdgeIt outEdgesBegin () const
- EdgeIt outEdgesEnd () const
- EdgeIt inEdgesBegin () const
- EdgeIt inEdgesEnd () const
- NonEdgeIt nonEdgesBegin () const
- NonEdgeIt nonEdgesEnd () const
- RndNodeIt rndNodesBegin (double ratio, long int seed) const
- RndNodeIt rndNodesEnd () const
- RndNonEdgeIt rndNonEdgesBegin (double ratio, long int seed) const
- RndNonEdgeIt rndNonEdgesEnd () const
- RndEdgeIt rndEdgesBegin (double ratio, long int seed) const
- RndEdgeIt rndEdgesEnd () const
- void getDegStat (std::size_t &minDeg, std::size_t &maxDeg, double &avgDeg) const
- void getOutDegStat (std::size_t &minOutDeg, std::size_t &maxOutDeg, double &avgOutDeg) const
- void getInDegStat (std::size_t &minInDeg, std::size_t &maxInDeg, double &avgInDeg) const
- template<typename ValueT >
  NodeMap< ValueT > createNodeMap () const
- template<typename ValueT >
  NodeMapSP< ValueT > createNodeMapSP () const
- template<typename ValueT >
  NodeSMap< ValueT > createNodeSMap (ValueT const &defVal) const

- template< typename ValueT >
  NodeSMapSP< ValueT > createNodeSMapSP (ValueT const &defVal) const
- template< typename ValueT >
  EdgeMap< ValueT > createEdgeMap () const
- template< typename ValueT >
  EdgeMapSP< ValueT > createEdgeMapSP () const
- std::shared_ptr< std::vector< Edge > > readEdges (std::string fileName) const
- void write (std::string fileName) const
- void print () const
- std::size_t getNbPaths (NodeID const &srcId, NodeID const &endId, std::size_t length) const
- template< typename ForwardIterator >
  void printEdges (ForwardIterator edgesBegin, ForwardIterator edgesEnd) const
- virtual ~DNetwork ()=default

Static Public Member Functions

- static Edge makeEdge (NodeID const &i, NodeID const &j)
- static Edge reverseEdge (Edge const &e)
- static const NodeID start (Edge const &edge)
- static const NodeID end (Edge const &edge)
- static bool compareEdgeEnd (Edge const &e1, Edge const &e2)
- static std::shared_ptr< DNetwork< Label, NodeID, Edge > > read (std::string fileName, bool ignore↩ Repetitions=false, bool ignoreLoops=false)

9.22.1 Detailed Description

template<typename LabelT = std::string, typename NodeIDT = unsigned int, typename EdgeT = unsigned long long int>
class LinkPred::DNetwork< LabelT, NodeIDT, EdgeT >

This class represents a directed network in the sense of graph theory.

Template Parameters

LabelT	Type of external labels.
NodeIDT	Type of internal node IDs. This must be an unsigned integral type.
EdgeT	Type of edges. This must be an unsigned integral type having at least double the size of `NodeID` DNetwork.

9.22.2 Member Typedef Documentation

9.22.2.1 Edge

```
template<typename LabelT = std::string, typename NodeIDT = unsigned int, typename EdgeT =
unsigned long long int>
using LinkPred::DNetwork< LabelT, NodeIDT, EdgeT >::Edge = EdgeT
```

Internal edge type.

9.22.2.2 EdgeIt

```
template<typename LabelT = std::string, typename NodeIDT = unsigned int, typename EdgeT =
unsigned long long int>
using LinkPred::DNetwork< LabelT, NodeIDT, EdgeT >::EdgeIt = typename std::vector<Edge>↩
::const_iterator
```

Edge iterator.

9.22.2.3 EdgeMapSP

```
template<typename LabelT = std::string, typename NodeIDT = unsigned int, typename EdgeT =
unsigned long long int>
template<typename ValueT >
using LinkPred::DNetwork< LabelT, NodeIDT, EdgeT >::EdgeMapSP = std::shared_ptr<EdgeMap<ValueT>
>
```

Shared pointer to an edge map.

9.22.2.4 Label

```
template<typename LabelT = std::string, typename NodeIDT = unsigned int, typename EdgeT =
unsigned long long int>
using LinkPred::DNetwork< LabelT, NodeIDT, EdgeT >::Label = LabelT
```

External label type.

9.22.2.5 LabelIt

```
template<typename LabelT = std::string, typename NodeIDT = unsigned int, typename EdgeT =
unsigned long long int>
using LinkPred::DNetwork< LabelT, NodeIDT, EdgeT >::LabelIt = typename Bhmap<Label, NodeID>↩
::k_const_iterator
```

External node iterator that offers the mapping to internal IDs.

9.22.2.6 NodeID

```
template<typename LabelT = std::string, typename NodeIDT = unsigned int, typename EdgeT =
unsigned long long int>
using LinkPred::DNetwork< LabelT, NodeIDT, EdgeT >::NodeID = NodeIDT
```

Internal node ID type.

9.22.2.7 NodeIt

```
template<typename LabelT = std::string, typename NodeIDT = unsigned int, typename EdgeT =
unsigned long long int>
using LinkPred::DNetwork< LabelT, NodeIDT, EdgeT >::NodeIt = typename Bhmap<Label, NodeID>↩
::p_const_iterator
```

Internal node iterator (random access iterator).

9.22.2.8 NodeMapSP

```
template<typename LabelT = std::string, typename NodeIDT = unsigned int, typename EdgeT =
unsigned long long int>
template<typename ValueT >
using LinkPred::DNetwork< LabelT, NodeIDT, EdgeT >::NodeMapSP = std::shared_ptr<NodeMap<ValueT>
>
```

Shared pointer to a node map.

9.22.2.9 NodeSMapSP

```
template<typename LabelT = std::string, typename NodeIDT = unsigned int, typename EdgeT =
unsigned long long int>
template<typename ValueT >
using LinkPred::DNetwork< LabelT, NodeIDT, EdgeT >::NodeSMapSP = std::shared_ptr<NodeSMap<ValueT>
>
```

Shared pointer to a node map.

9.22.3 Constructor & Destructor Documentation

9.22.3.1 DNetwork() [1/4]

```
template<typename LabelT = std::string, typename NodeIDT = unsigned int, typename EdgeT =
unsigned long long int>
LinkPred::DNetwork< LabelT, NodeIDT, EdgeT >::DNetwork ( ) [default]
```

Default constructor.

9.22.3.2 DNetwork() [2/4]

```
template<typename LabelT = std::string, typename NodeIDT = unsigned int, typename EdgeT =
unsigned long long int>
LinkPred::DNetwork< LabelT, NodeIDT, EdgeT >::DNetwork (
            std::vector< std::pair< Label, Label >> const & edges )
```

Build the network form a list of edges. The network is assembled within this constructor.

Parameters

edges	List of edges.

9.22.3.3 DNetwork() [3/4]

```
template<typename LabelT = std::string, typename NodeIDT = unsigned int, typename EdgeT =
unsigned long long int>
LinkPred::DNetwork< LabelT, NodeIDT, EdgeT >::DNetwork (
            DNetwork< LabelT, NodeIDT, EdgeT > const & that )  [default]
```

Copy constructor.

Parameters

that	The object to copy.

9.22.3.4 DNetwork() [4/4]

```
template<typename LabelT = std::string, typename NodeIDT = unsigned int, typename EdgeT =
unsigned long long int>
LinkPred::DNetwork< LabelT, NodeIDT, EdgeT >::DNetwork (
            DNetwork< LabelT, NodeIDT, EdgeT > && that )  [default]
```

Move constructor.

Parameters

that	The object to move.

9.22.3.5 ∼DNetwork()

```
template<typename LabelT = std::string, typename NodeIDT = unsigned int, typename EdgeT =
unsigned long long int>
virtual LinkPred::DNetwork< LabelT, NodeIDT, EdgeT >::∼DNetwork ( )  [virtual], [default]
```

Destructor.

9.22.4 Member Function Documentation

9.22.4.1 addEdge()

```
template<typename LabelT = std::string, typename NodeIDT = unsigned int, typename EdgeT =
unsigned long long int>
void LinkPred::DNetwork< LabelT, NodeIDT, EdgeT >::addEdge (
            NodeID const & i,
            NodeID const & j )
```

Add an edge.

Parameters

i	The starting node.
j	The end node.

9.22.4.2 addNode()

```
template<typename LabelT = std::string, typename NodeIDT = unsigned int, typename EdgeT =
unsigned long long int>
std::pair<NodeID, bool> LinkPred::DNetwork< LabelT, NodeIDT, EdgeT >::addNode (
            Label const & nodeId )
```

Add a node.

Parameters

node←<br>*Id*	The ID of the node.

Returns

An std::pair, where first is the internal ID, and second is a boolean which is true if the node is actually added.

9.22.4.3 assemble()

```
template<typename LabelT = std::string, typename NodeIDT = unsigned int, typename EdgeT =
unsigned long long int>
void LinkPred::DNetwork< LabelT, NodeIDT, EdgeT >::assemble ( )
```

Assemble the network. No changes to the network are allowed after calling this method.

9.22.4.4 compareEdgeEnd()

```
template<typename LabelT = std::string, typename NodeIDT = unsigned int, typename EdgeT =
unsigned long long int>
static bool LinkPred::DNetwork< LabelT, NodeIDT, EdgeT >::compareEdgeEnd (
            Edge const & e1,
            Edge const & e2 ) [inline], [static]
```

Compare edge ends.

Parameters

e1	First edge.
e2	Second edge.

Returns

True if the end of e1 is smaller than that of e2 (comparison is based on node IDs).

9.22.4.5 coupleAtOrd()

```
template<typename LabelT = std::string, typename NodeIDT = unsigned int, typename EdgeT =
unsigned long long int>
std::size_t LinkPred::DNetwork< LabelT, NodeIDT, EdgeT >::coupleAtOrd (
            std::size_t ord ) const [inline]
```

Parameters

ord	The order of an edge.

Returns

The edge given its order.

9.22.4.6 coupleOrd()

```
template<typename LabelT = std::string, typename NodeIDT = unsigned int, typename EdgeT =
unsigned long long int>
std::size_t LinkPred::DNetwork< LabelT, NodeIDT, EdgeT >::coupleOrd (
            Edge const & e ) const [inline]
```

Parameters

e	An edge.

Returns

The order of the edge

9.22.4.7 createEdgeMap()

```
template<typename LabelT = std::string, typename NodeIDT = unsigned int, typename EdgeT =
unsigned long long int>
template<typename ValueT >
EdgeMap<ValueT> LinkPred::DNetwork< LabelT, NodeIDT, EdgeT >::createEdgeMap ( ) const [inline]
```

Template Parameters

ValueT	Value type.

Returns

An edge map.

9.22.4.8 createEdgeMapSP()

```
template<typename LabelT = std::string, typename NodeIDT = unsigned int, typename EdgeT =
unsigned long long int>
template<typename ValueT >
EdgeMapSP<ValueT> LinkPred::DNetwork< LabelT, NodeIDT, EdgeT >::createEdgeMapSP ( ) const
[inline]
```

Template Parameters

ValueT	Value type.

Returns

A pointer to an edge map.

9.22.4.9 createNodeMap()

```
template<typename LabelT = std::string, typename NodeIDT = unsigned int, typename EdgeT =
unsigned long long int>
template<typename ValueT >
NodeMap<ValueT> LinkPred::DNetwork< LabelT, NodeIDT, EdgeT >::createNodeMap ( ) const [inline]
```

Template Parameters

ValueT	Value type.

Returns

A node map.

9.22.4.10 createNodeMapSP()

```
template<typename LabelT = std::string, typename NodeIDT = unsigned int, typename EdgeT =
unsigned long long int>
```

```
template<typename ValueT >
NodeMapSP<ValueT> LinkPred::DNetwork< LabelT, NodeIDT, EdgeT >::createNodeMapSP ( ) const
[inline]
```

Template Parameters

ValueT	Value type.

Returns

A pointer to a node map.

9.22.4.11 createNodeSMap()

```
template<typename LabelT = std::string, typename NodeIDT = unsigned int, typename EdgeT =
unsigned long long int>
template<typename ValueT >
NodeSMap<ValueT> LinkPred::DNetwork< LabelT, NodeIDT, EdgeT >::createNodeSMap (
            ValueT const & defVal ) const  [inline]
```

Template Parameters

ValueT	Value type.

Returns

A sparse node map.

9.22.4.12 createNodeSMapSP()

```
template<typename LabelT = std::string, typename NodeIDT = unsigned int, typename EdgeT =
unsigned long long int>
template<typename ValueT >
NodeSMapSP<ValueT> LinkPred::DNetwork< LabelT, NodeIDT, EdgeT >::createNodeSMapSP (
            ValueT const & defVal ) const  [inline]
```

Template Parameters

ValueT	Value type.

Returns

A pointer to a sparse node map.

9.22.4.13 edgesBegin()

```
template<typename LabelT = std::string, typename NodeIDT = unsigned int, typename EdgeT =
unsigned long long int>
EdgeIt LinkPred::DNetwork< LabelT, NodeIDT, EdgeT >::edgesBegin ( ) const  [inline]
```

Returns

A read-only (constant) iterator that points to the first edge (with internal ID). Edges are ordered by source node.

9.22.4.14 edgesEnd()

```
template<typename LabelT = std::string, typename NodeIDT = unsigned int, typename EdgeT =
unsigned long long int>
EdgeIt LinkPred::DNetwork< LabelT, NodeIDT, EdgeT >::edgesEnd ( ) const  [inline]
```

Returns

A read-only (constant) iterator that points one past the last edge (with internal ID). Edges are ordered by source node.

9.22.4.15 end()

```
template<typename LabelT = std::string, typename NodeIDT = unsigned int, typename EdgeT =
unsigned long long int>
static const NodeID LinkPred::DNetwork< LabelT, NodeIDT, EdgeT >::end (
            Edge const & edge )  [inline], [static]
```

Parameters

edge	An edge.

Returns

The end node of edge.

9.22.4.16 findLabel()

```
template<typename LabelT = std::string, typename NodeIDT = unsigned int, typename EdgeT =
unsigned long long int>
LabelIt LinkPred::DNetwork< LabelT, NodeIDT, EdgeT >::findLabel (
            Label const & label ) const  [inline]
```

Parameters

label	An external node ID.

Returns

Iterator to the external node ID.

9.22.4.17 findNode()

```
template<typename LabelT = std::string, typename NodeIDT = unsigned int, typename EdgeT =
unsigned long long int>
NodeIt LinkPred::DNetwork< LabelT, NodeIDT, EdgeT >::findNode (
            NodeID const & iid ) const [inline]
```

Parameters

iid	An internal node ID.

Returns

Iterator to the internal node ID.

9.22.4.18 getAvgDeg()

```
template<typename LabelT = std::string, typename NodeIDT = unsigned int, typename EdgeT =
unsigned long long int>
double LinkPred::DNetwork< LabelT, NodeIDT, EdgeT >::getAvgDeg ( ) const [inline]
```

Returns

Average degree. Can only be called after the network is assembled.

9.22.4.19 getAvgInDeg()

```
template<typename LabelT = std::string, typename NodeIDT = unsigned int, typename EdgeT =
unsigned long long int>
double LinkPred::DNetwork< LabelT, NodeIDT, EdgeT >::getAvgInDeg ( ) const [inline]
```

Returns

Average in-degree. Can only be called after the network is assembled.

9.22.4.20 getAvgOutDeg()

```
template<typename LabelT = std::string, typename NodeIDT = unsigned int, typename EdgeT =
unsigned long long int>
double LinkPred::DNetwork< LabelT, NodeIDT, EdgeT >::getAvgOutDeg ( ) const [inline]
```

Returns

Average out-degree. Can only be called after the network is assembled.

9.22.4.21 getDeg()

```
template<typename LabelT = std::string, typename NodeIDT = unsigned int, typename EdgeT =
unsigned long long int>
std::size_t LinkPred::DNetwork< LabelT, NodeIDT, EdgeT >::getDeg (
            NodeID const & iid ) const [inline]
```

Parameters

iid	The node internal ID.

Returns

The degree of node iid (sum of in and out-degrees).

9.22.4.22 getDegStat()

```
template<typename LabelT = std::string, typename NodeIDT = unsigned int, typename EdgeT =
unsigned long long int>
void LinkPred::DNetwork< LabelT, NodeIDT, EdgeT >::getDegStat (
            std::size_t & minDeg,
            std::size_t & maxDeg,
            double & avgDeg ) const [inline]
```

Compute some degree statistics.

Parameters

minDeg	(output parameter) minimum degree.
maxDeg	(output parameter) maximum degree.
avgDeg	(output parameter) average degree.

9.22.4.23 getID()

```
template<typename LabelT = std::string, typename NodeIDT = unsigned int, typename EdgeT =
unsigned long long int>
NodeID LinkPred::DNetwork< LabelT, NodeIDT, EdgeT >::getID (
            Label const & label ) const [inline]
```

Translates from external label to internal IDs. This method is O(log n), where n is the number of nodes.

Parameters

label	An external node label.

Returns

The internal ID of label;

9.22.4.24 getInDeg()

```
template<typename LabelT = std::string, typename NodeIDT = unsigned int, typename EdgeT =
unsigned long long int>
std::size_t LinkPred::DNetwork< LabelT, NodeIDT, EdgeT >::getInDeg (
            NodeID const & iid ) const [inline]
```

Parameters

iid	The node internal ID.

Returns

The in-degree of node iid.

9.22.4.25 getInDegStat()

```
template<typename LabelT = std::string, typename NodeIDT = unsigned int, typename EdgeT =
unsigned long long int>
void LinkPred::DNetwork< LabelT, NodeIDT, EdgeT >::getInDegStat (
            std::size_t & minInDeg,
            std::size_t & maxInDeg,
            double & avgInDeg ) const [inline]
```

Compute some in-degree statistics.

Parameters

minInDeg	(input parameter) minimum in-degree.
maxInDeg	(input parameter) maximum in-degree.
avgInDeg	(input parameter) average in-degree.

9.22.4.26 getInOutDeg()

```
template<typename LabelT = std::string, typename NodeIDT = unsigned int, typename EdgeT =
unsigned long long int>
std::pair<std::size_t, std::size_t> LinkPred::DNetwork< LabelT, NodeIDT, EdgeT >::getInOutDeg
(
            NodeID const & iid ) const [inline]
```

Parameters

iid	The node internal ID.

Returns

The in and out-degrees of node iid.

9.22.4.27 getLabel()

```
template<typename LabelT = std::string, typename NodeIDT = unsigned int, typename EdgeT =
unsigned long long int>
Label LinkPred::DNetwork< LabelT, NodeIDT, EdgeT >::getLabel (
            NodeID const & iid ) const [inline]
```

Parameters

iid	An internal node ID.

Returns

The external label of the node iid.

9.22.4.28 getMaxDeg()

```
template<typename LabelT = std::string, typename NodeIDT = unsigned int, typename EdgeT =
unsigned long long int>
std::size_t LinkPred::DNetwork< LabelT, NodeIDT, EdgeT >::getMaxDeg ( ) const [inline]
```

Returns

Maximum degree. Can only be called after the network is assembled.

9.22.4.29 getMaxInDeg()

```
template<typename LabelT = std::string, typename NodeIDT = unsigned int, typename EdgeT =
unsigned long long int>
std::size_t LinkPred::DNetwork< LabelT, NodeIDT, EdgeT >::getMaxInDeg ( ) const [inline]
```

Returns

Maximum in-degree. Can only be called after the network is assembled.

9.22.4.30 getMaxOutDeg()

```
template<typename LabelT = std::string, typename NodeIDT = unsigned int, typename EdgeT =
unsigned long long int>
std::size_t LinkPred::DNetwork< LabelT, NodeIDT, EdgeT >::getMaxOutDeg ( ) const [inline]
```

Returns

Maximum out-degree. Can only be called after the network is assembled.

9.22.4.31 getMinDeg()

```
template<typename LabelT = std::string, typename NodeIDT = unsigned int, typename EdgeT =
unsigned long long int>
std::size_t LinkPred::DNetwork< LabelT, NodeIDT, EdgeT >::getMinDeg ( ) const [inline]
```

Returns

Minimum degree. Can only be called after the network is assembled.

9.22.4.32 getMinInDeg()

```
template<typename LabelT = std::string, typename NodeIDT = unsigned int, typename EdgeT =
unsigned long long int>
std::size_t LinkPred::DNetwork< LabelT, NodeIDT, EdgeT >::getMinInDeg ( ) const [inline]
```

Returns

Minimum in-degree. Can only be called after the network is assembled.

9.22.4.33 getMinOutDeg()

```
template<typename LabelT = std::string, typename NodeIDT = unsigned int, typename EdgeT =
unsigned long long int>
std::size_t LinkPred::DNetwork< LabelT, NodeIDT, EdgeT >::getMinOutDeg ( ) const  [inline]
```

Returns

Minimum out-degree. Can only be called after the network is assembled.

9.22.4.34 getNbCouples()

```
template<typename LabelT = std::string, typename NodeIDT = unsigned int, typename EdgeT =
unsigned long long int>
std::size_t LinkPred::DNetwork< LabelT, NodeIDT, EdgeT >::getNbCouples ( ) const  [inline]
```

Returns

The number of couples in the network.

9.22.4.35 getNbEdges()

```
template<typename LabelT = std::string, typename NodeIDT = unsigned int, typename EdgeT =
unsigned long long int>
std::size_t LinkPred::DNetwork< LabelT, NodeIDT, EdgeT >::getNbEdges ( ) const  [inline]
```

Returns

The number of edges in the network.

9.22.4.36 getNbNodes()

```
template<typename LabelT = std::string, typename NodeIDT = unsigned int, typename EdgeT =
unsigned long long int>
std::size_t LinkPred::DNetwork< LabelT, NodeIDT, EdgeT >::getNbNodes ( ) const  [inline]
```

Returns

The number of nodes in the network.

9.22.4.37 getNbNonEdges()

```
template<typename LabelT = std::string, typename NodeIDT = unsigned int, typename EdgeT =
unsigned long long int>
std::size_t LinkPred::DNetwork< LabelT, NodeIDT, EdgeT >::getNbNonEdges ( ) const [inline]
```

Returns

The number of non-edges in the network.

9.22.4.38 getNbPaths()

```
template<typename LabelT = std::string, typename NodeIDT = unsigned int, typename EdgeT =
unsigned long long int>
std::size_t LinkPred::DNetwork< LabelT, NodeIDT, EdgeT >::getNbPaths (
            NodeID const & srcId,
            NodeID const & endId,
            std::size_t length ) const
```

Parameters

srcId	The source node ID.
endId	The end node ID.
length	Specified length.

Returns

The number of paths of length exactly length joining srcNode and endNode.

9.22.4.39 getOutDeg()

```
template<typename LabelT = std::string, typename NodeIDT = unsigned int, typename EdgeT =
unsigned long long int>
std::size_t LinkPred::DNetwork< LabelT, NodeIDT, EdgeT >::getOutDeg (
            NodeID const & iid ) const [inline]
```

Parameters

iid	The node internal ID.

Returns

The out-degree of node iid.

9.22.4.40 getOutDegStat()

```
template<typename LabelT = std::string, typename NodeIDT = unsigned int, typename EdgeT =
unsigned long long int>
void LinkPred::DNetwork< LabelT, NodeIDT, EdgeT >::getOutDegStat (
            std::size_t & minOutDeg,
            std::size_t & maxOutDeg,
            double & avgOutDeg ) const [inline]
```

Compute some out-degree statistics.

Parameters

minOutDeg	(output parameter) minimum out-degree.
maxOutDeg	(output parameter) maximum out-degree.
avgOutDeg	(output parameter) average out-degree.

9.22.4.41 inEdgesBegin()

```
template<typename LabelT = std::string, typename NodeIDT = unsigned int, typename EdgeT =
unsigned long long int>
EdgeIt LinkPred::DNetwork< LabelT, NodeIDT, EdgeT >::inEdgesBegin ( ) const [inline]
```

Returns

A read-only (constant) iterator that points to the first edge (with internal ID). Edges are ordered by end node.

9.22.4.42 inEdgesEnd()

```
template<typename LabelT = std::string, typename NodeIDT = unsigned int, typename EdgeT =
unsigned long long int>
EdgeIt LinkPred::DNetwork< LabelT, NodeIDT, EdgeT >::inEdgesEnd ( ) const [inline]
```

Returns

A read-only (constant) iterator that points one past the last edge (with internal ID). Edges are ordered by end node.

9.22.4.43 inNeighborsBegin()

```
template<typename LabelT = std::string, typename NodeIDT = unsigned int, typename EdgeT =
unsigned long long int>
EdgeIt LinkPred::DNetwork< LabelT, NodeIDT, EdgeT >::inNeighborsBegin (
            NodeID const & iid ) const [inline]
```

Parameters

iid	An internal node ID.

Returns

An iterator to the first in-neighbor of iid.

9.22.4.44 inNeighborsEnd()

```
template<typename LabelT = std::string, typename NodeIDT = unsigned int, typename EdgeT =
unsigned long long int>
EdgeIt LinkPred::DNetwork< LabelT, NodeIDT, EdgeT >::inNeighborsEnd (
            NodeID const & iid ) const  [inline]
```

Parameters

iid	An internal node ID.

Returns

An iterator to one past the last in-neighbor of iid.

9.22.4.45 isEdge() [1/2]

```
template<typename LabelT = std::string, typename NodeIDT = unsigned int, typename EdgeT =
unsigned long long int>
bool LinkPred::DNetwork< LabelT, NodeIDT, EdgeT >::isEdge (
            Edge const & edge ) const
```

Check if an edge exists in O(k_max).

Parameters

edge	An edge.

Returns

True if edge exists in the network, false otherwise.

9.22.4.46 isEdge() [2/2]

```
template<typename LabelT = std::string, typename NodeIDT = unsigned int, typename EdgeT =
unsigned long long int>
bool LinkPred::DNetwork< LabelT, NodeIDT, EdgeT >::isEdge (
            NodeID const & ii,
            NodeID const & ij ) const  [inline]
```

Check if an edge exists in O(k_max).

Parameters

ii	An internal node ID.
ij	An internal node ID.

Returns

true if the edge (ii, ij) exists, false otherwise.

9.22.4.47 labelsBegin()

```
template<typename LabelT = std::string, typename NodeIDT = unsigned int, typename EdgeT =
unsigned long long int>
LabelIt LinkPred::DNetwork< LabelT, NodeIDT, EdgeT >::labelsBegin ( ) const  [inline]
```

Returns

A read-only (constant) external node ID iterator that points to the first node.

9.22.4.48 labelsEnd()

```
template<typename LabelT = std::string, typename NodeIDT = unsigned int, typename EdgeT =
unsigned long long int>
LabelIt LinkPred::DNetwork< LabelT, NodeIDT, EdgeT >::labelsEnd ( ) const  [inline]
```

Returns

A read-only (constant) external node ID iterator that points one past the last node.

9.22.4.49 makeEdge()

```
template<typename LabelT = std::string, typename NodeIDT = unsigned int, typename EdgeT =
unsigned long long int>
static Edge LinkPred::DNetwork< LabelT, NodeIDT, EdgeT >::makeEdge (
            NodeID const & i,
            NodeID const & j )  [inline], [static]
```

Make an edge in internal representation out of two nodes' internal IDs.

Parameters

i	The starting node.
j	The end node.

Returns

The edge (i, j).

9.22.4.50 neighbBegin()

```
template<typename LabelT = std::string, typename NodeIDT = unsigned int, typename EdgeT =
unsigned long long int>
EdgeIt LinkPred::DNetwork< LabelT, NodeIDT, EdgeT >::neighbBegin (
            NodeID const & iid ) const  [inline]
```

Parameters

iid	An internal node ID.

Returns

An iterator to the first out-neighbor of iid.

9.22.4.51 neighbEnd()

```
template<typename LabelT = std::string, typename NodeIDT = unsigned int, typename EdgeT =
unsigned long long int>
EdgeIt LinkPred::DNetwork< LabelT, NodeIDT, EdgeT >::neighbEnd (
            NodeID const & iid ) const  [inline]
```

Parameters

iid	An internal node ID.

Returns

An iterator to one past the last out-neighbor of iid.

9.22.4.52 nodesBegin()

```
template<typename LabelT = std::string, typename NodeIDT = unsigned int, typename EdgeT =
unsigned long long int>
NodeIt LinkPred::DNetwork< LabelT, NodeIDT, EdgeT >::nodesBegin ( ) const  [inline]
```

Returns

A read-only (constant) external node ID iterator that points to the first node.

9.22.4.53 nodesDegBegin()

```
template<typename LabelT = std::string, typename NodeIDT = unsigned int, typename EdgeT =
unsigned long long int>
NodeDegIt LinkPred::DNetwork< LabelT, NodeIDT, EdgeT >::nodesDegBegin ( ) const [inline]
```

Returns

A read-only (constant) external node ID iterator that points to the first node-degree couple.

9.22.4.54 nodesDegEnd()

```
template<typename LabelT = std::string, typename NodeIDT = unsigned int, typename EdgeT =
unsigned long long int>
NodeDegIt LinkPred::DNetwork< LabelT, NodeIDT, EdgeT >::nodesDegEnd ( ) const [inline]
```

Returns

A read-only (constant) external node ID iterator that points one past the last node-degree couple.

9.22.4.55 nodesEnd()

```
template<typename LabelT = std::string, typename NodeIDT = unsigned int, typename EdgeT =
unsigned long long int>
NodeIt LinkPred::DNetwork< LabelT, NodeIDT, EdgeT >::nodesEnd ( ) const [inline]
```

Returns

A read-only (constant) external node ID iterator that points one past the last node.

9.22.4.56 nonEdgesBegin()

```
template<typename LabelT = std::string, typename NodeIDT = unsigned int, typename EdgeT =
unsigned long long int>
NonEdgeIt LinkPred::DNetwork< LabelT, NodeIDT, EdgeT >::nonEdgesBegin ( ) const [inline]
```

Returns

A read-only (constant) iterator that points to the first non-edge (with internal ID).

9.22.4.57 nonEdgesEnd()

```
template<typename LabelT = std::string, typename NodeIDT = unsigned int, typename EdgeT =
unsigned long long int>
NonEdgeIt LinkPred::DNetwork< LabelT, NodeIDT, EdgeT >::nonEdgesEnd ( ) const [inline]
```

Returns

A read-only (constant) iterator that points one past the last non-edge (with internal ID).

9.22.4.58 operator=() [1/2]

```
template<typename LabelT = std::string, typename NodeIDT = unsigned int, typename EdgeT =
unsigned long long int>
DNetwork& LinkPred::DNetwork< LabelT, NodeIDT, EdgeT >::operator= (
            DNetwork< LabelT, NodeIDT, EdgeT > && that ) [default]
```

Move assignment operator.

Parameters

that	The object to move.

9.22.4.59 operator=() [2/2]

```
template<typename LabelT = std::string, typename NodeIDT = unsigned int, typename EdgeT =
unsigned long long int>
DNetwork& LinkPred::DNetwork< LabelT, NodeIDT, EdgeT >::operator= (
            DNetwork< LabelT, NodeIDT, EdgeT > const & that ) [default]
```

Copy assignment operator.

Parameters

that	The object to copy.

9.22.4.60 outEdgesBegin()

```
template<typename LabelT = std::string, typename NodeIDT = unsigned int, typename EdgeT =
unsigned long long int>
EdgeIt LinkPred::DNetwork< LabelT, NodeIDT, EdgeT >::outEdgesBegin ( ) const [inline]
```

Returns

A read-only (constant) iterator that points to the first edge (with internal ID). Edges are ordered by source node.

9.22.4.61 outEdgesEnd()

```
template<typename LabelT = std::string, typename NodeIDT = unsigned int, typename EdgeT =
unsigned long long int>
EdgeIt LinkPred::DNetwork< LabelT, NodeIDT, EdgeT >::outEdgesEnd ( ) const  [inline]
```

Returns

A read-only (constant) iterator that points one past the last edge (with internal ID). Edges are ordered by source node.

9.22.4.62 outNeighborsBegin()

```
template<typename LabelT = std::string, typename NodeIDT = unsigned int, typename EdgeT =
unsigned long long int>
EdgeIt LinkPred::DNetwork< LabelT, NodeIDT, EdgeT >::outNeighborsBegin (
            NodeID const & iid ) const  [inline]
```

Parameters

iid	An internal node ID.

Returns

An iterator to the first out-neighbor of iid.

9.22.4.63 outNeighborsEnd()

```
template<typename LabelT = std::string, typename NodeIDT = unsigned int, typename EdgeT =
unsigned long long int>
EdgeIt LinkPred::DNetwork< LabelT, NodeIDT, EdgeT >::outNeighborsEnd (
            NodeID const & iid ) const  [inline]
```

Parameters

iid	An internal node ID.

Returns

An iterator to one past the last out-neighbor of iid.

9.22.4.64 print()

```
template<typename LabelT = std::string, typename NodeIDT = unsigned int, typename EdgeT =
unsigned long long int>
void LinkPred::DNetwork< LabelT, NodeIDT, EdgeT >::print ( ) const
```

Print edges to std::cout.

9.22.4.65 printEdges()

```
template<typename LabelT = std::string, typename NodeIDT = unsigned int, typename EdgeT =
unsigned long long int>
template<typename ForwardIterator >
void LinkPred::DNetwork< LabelT, NodeIDT, EdgeT >::printEdges (
            ForwardIterator edgesBegin,
            ForwardIterator edgesEnd ) const [inline]
```

Print edges to std::cout.

Parameters

edgesBegin	Iterator to the beginning of the edges.
edgesEnd	Iterator to one past the the end of the edges.

9.22.4.66 read()

```
template<typename LabelT = std::string, typename NodeIDT = unsigned int, typename EdgeT =
unsigned long long int>
static std::shared_ptr<DNetwork<Label, NodeID, Edge> > LinkPred::DNetwork< LabelT, NodeIDT,
EdgeT >::read (
            std::string fileName,
            bool ignoreRepetitions = false,
            bool ignoreLoops = false ) [static]
```

Read network from file.

Parameters

fileName	The file name.
ignoreRepetitions	Whether to ignore repeated edges.
ignoreLoops	Whether to ignore loops.

Returns

The read network.

9.22.4.67 readEdges()

```
template<typename LabelT = std::string, typename NodeIDT = unsigned int, typename EdgeT =
unsigned long long int>
std::shared_ptr<std::vector<Edge> > LinkPred::DNetwork< LabelT, NodeIDT, EdgeT >::readEdges
(
            std::string fileName ) const
```

Read couples from file.

Parameters

fileName	The file name.

Returns

The edges.

9.22.4.68 reverseEdge()

```
template<typename LabelT = std::string, typename NodeIDT = unsigned int, typename EdgeT =
unsigned long long int>
static Edge LinkPred::DNetwork< LabelT, NodeIDT, EdgeT >::reverseEdge (
            Edge const & e )  [inline], [static]
```

Parameters

e	An edge (i,j).

Returns

The edge (j, i).

9.22.4.69 rndEdgesBegin()

```
template<typename LabelT = std::string, typename NodeIDT = unsigned int, typename EdgeT =
unsigned long long int>
RndEdgeIt LinkPred::DNetwork< LabelT, NodeIDT, EdgeT >::rndEdgesBegin (
            double ratio,
            long int seed ) const  [inline]
```

Parameters

ratio	Ratio of edges that are selected.
seed	The random number gnerator's seed.

Returns

a read-only (constant) randomized iterator that points to the first edge (with internal ID).

9.22.4.70 rndEdgesEnd()

```
template<typename LabelT = std::string, typename NodeIDT = unsigned int, typename EdgeT =
unsigned long long int>
RndEdgeIt LinkPred::DNetwork< LabelT, NodeIDT, EdgeT >::rndEdgesEnd ( ) const  [inline]
```

Returns

A read-only (constant) randomized iterator that points one past the last edge (with internal ID).

9.22.4.71 rndNodesBegin()

```
template<typename LabelT = std::string, typename NodeIDT = unsigned int, typename EdgeT =
unsigned long long int>
RndNodeIt LinkPred::DNetwork< LabelT, NodeIDT, EdgeT >::rndNodesBegin (
             double ratio,
             long int seed ) const  [inline]
```

Parameters

ratio	Ratio of nodes that are selected.
seed	The random number gnerator's seed.

Returns

a read-only (constant) randomized iterator that points to the first node (with internal ID).

9.22.4.72 rndNodesEnd()

```
template<typename LabelT = std::string, typename NodeIDT = unsigned int, typename EdgeT =
unsigned long long int>
RndNodeIt LinkPred::DNetwork< LabelT, NodeIDT, EdgeT >::rndNodesEnd ( ) const  [inline]
```

Returns

A read-only (constant) randomized iterator that points one past the last node (with internal ID).

9.22.4.73 rndNonEdgesBegin()

```
template<typename LabelT = std::string, typename NodeIDT = unsigned int, typename EdgeT =
unsigned long long int>
RndNonEdgeIt LinkPred::DNetwork< LabelT, NodeIDT, EdgeT >::rndNonEdgesBegin (
            double ratio,
            long int seed ) const [inline]
```

Parameters

ratio	Ratio of nonedges that are selected.
seed	The random number gnerator's seed.

Returns

a read-only (constant) randomized iterator that points to the first non-edge (with internal ID).

9.22.4.74 rndNonEdgesEnd()

```
template<typename LabelT = std::string, typename NodeIDT = unsigned int, typename EdgeT =
unsigned long long int>
RndNonEdgeIt LinkPred::DNetwork< LabelT, NodeIDT, EdgeT >::rndNonEdgesEnd ( ) const [inline]
```

Returns

A read-only (constant) randomized iterator that points one past the last non-edge (with internal ID).

9.22.4.75 shuffle()

```
template<typename LabelT = std::string, typename NodeIDT = unsigned int, typename EdgeT =
unsigned long long int>
void LinkPred::DNetwork< LabelT, NodeIDT, EdgeT >::shuffle (
            long int seed )
```

Shuffles the nodes' internal IDs. This is useful to eliminate bias in methods that depend on node/edge order. Upon calling this method, all iterators and maps associated with the network are invalidated.

Parameters

seed	Random number generator's seed.

9.22.4.76 start()

```
template<typename LabelT = std::string, typename NodeIDT = unsigned int, typename EdgeT =
unsigned long long int>
static const NodeID LinkPred::DNetwork< LabelT, NodeIDT, EdgeT >::start (
            Edge const & edge )  [inline], [static]
```

Parameters

edge	An edge.

Returns

The starting node of edge.

9.22.4.77 write()

```
template<typename LabelT = std::string, typename NodeIDT = unsigned int, typename EdgeT =
unsigned long long int>
void LinkPred::DNetwork< LabelT, NodeIDT, EdgeT >::write (
            std::string fileName ) const
```

Write adjacency matrix in sparse form to file.

Parameters

fileName	the file name.

The documentation for this class was generated from the following file:

- include/linkpred/core/dnetwork/dnetwork.hpp

9.23 LinkPred::DotProd Class Reference

A simple dot product similarity measure.

```
#include <dotprod.hpp>
```

Inheritance diagram for LinkPred::DotProd:

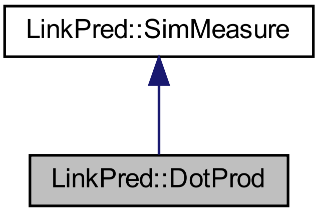

Collaboration diagram for LinkPred::DotProd:

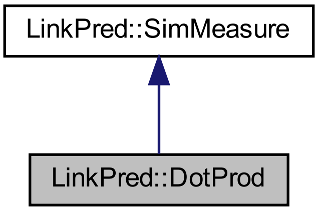

Public Member Functions

- DotProd ()
- DotProd (DotProd const &that)=default
- DotProd & operator= (DotProd const &that)=default
- DotProd (DotProd &&that)=default
- DotProd & operator= (DotProd &&that)=default
- virtual double sim (Vec const &v1, Vec const &v2)
- virtual ∼DotProd ()=default

9.23.1 Detailed Description

A simple dot product similarity measure.

9.23.2 Constructor & Destructor Documentation

9.23.2.1 DotProd() [1/3]

```
LinkPred::DotProd::DotProd ( )  [inline]
```

Constructor.

9.23.2.2 DotProd() [2/3]

```
LinkPred::DotProd::DotProd (
            DotProd const & that )  [default]
```

Copy constructor.

Parameters

that	The object to copy.

9.23.2.3 DotProd() [3/3]

```
LinkPred::DotProd::DotProd (
            DotProd && that )  [default]
```

Move constructor.

Parameters

that	The object to move.

9.23.2.4 ∼DotProd()

```
virtual LinkPred::DotProd::∼DotProd ( )  [virtual], [default]
```

Destructor.

9.23.3 Member Function Documentation

9.23.3.1 operator=() [1/2]

```
DotProd& LinkPred::DotProd::operator= (
            DotProd && that )  [default]
```

Move assignment operator.

Parameters

that	The object to move.

9.23.3.2 operator=() [2/2]

```
DotProd& LinkPred::DotProd::operator= (
            DotProd const & that )  [default]
```

Copy assignment operator.

Parameters

that	The object to copy.

9.23.3.3 sim()

```
virtual double LinkPred::DotProd::sim (
            Vec const & v1,
            Vec const & v2 )  [virtual]
```

Compute the similarity between two vectors.

Parameters

v1	First vector.
v2	Second vector. Must be of the same dimension as v1.

Returns

The similarity between v1 and v2.

Implements LinkPred::SimMeasure.

The documentation for this class was generated from the following file:

- include/linkpred/ml/simmeasures/dotprod.hpp

9.24 LinkPred::DPATPredictor< Network, EdgeRndIt, ScoreRndIt, EdgeRndOutIt > Class Template Reference

Common neighbor link predictor adapted to directed networks.

```
#include <dpatpredictor.hpp>
```

Inheritance diagram for LinkPred::DPATPredictor< Network, EdgeRndIt, ScoreRndIt, EdgeRndOutIt >:

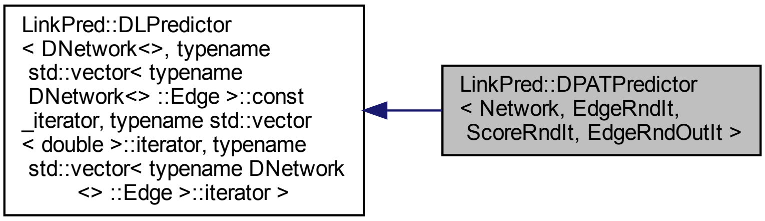

Collaboration diagram for LinkPred::DPATPredictor< Network, EdgeRndIt, ScoreRndIt, EdgeRndOutIt >:

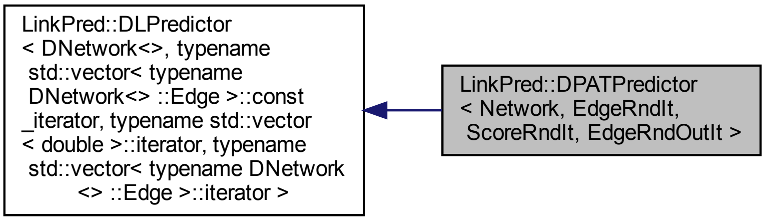

Public Member Functions

- DPATPredictor (std::shared_ptr< Network const > net)
- DPATPredictor (DPATPredictor const &that)=default
- DPATPredictor & operator= (DPATPredictor const &that)=default
- DPATPredictor (DPATPredictor &&that)=default
- DPATPredictor & operator= (DPATPredictor &&that)=default
- virtual void init ()
- virtual void learn ()
- virtual void predict (EdgeRndIt begin, EdgeRndIt end, ScoreRndIt scores)
- virtual double score (Edge const &e)
- virtual ~DPATPredictor ()=default

Additional Inherited Members

9.24.1 Detailed Description

template<typename Network = DNetwork<>, typename EdgeRndIt = typename std::vector<typename Network::Edge>↩ ::const_iterator, typename ScoreRndIt = typename std::vector<double>::iterator, typename EdgeRndOutIt = typename std↩ ::vector<typename Network::Edge>::iterator>
class LinkPred::DPATPredictor< Network, EdgeRndIt, ScoreRndIt, EdgeRndOutIt >

Common neighbor link predictor adapted to directed networks.

Template Parameters

Network	The network type.
Edge↩ RndIt	A random iterator type used to iterate on edges.
Score↩ RndIt	A random iterator type used to iterate on scores.

9.24.2 Constructor & Destructor Documentation

9.24.2.1 DPATPredictor() [1/3]

```
template<typename Network = DNetwork<>, typename EdgeRndIt = typename std::vector<typename
Network::Edge>::const_iterator, typename ScoreRndIt = typename std::vector<double>::iterator,
typename EdgeRndOutIt = typename std::vector<typename Network::Edge>::iterator>
LinkPred::DPATPredictor< Network, EdgeRndIt, ScoreRndIt, EdgeRndOutIt >::DPATPredictor (
            std::shared_ptr< Network const > net )  [inline]
```

Parameters

net	The network.

9.24.2.2 DPATPredictor() [2/3]

```
template<typename Network = DNetwork<>, typename EdgeRndIt = typename std::vector<typename
Network::Edge>::const_iterator, typename ScoreRndIt = typename std::vector<double>::iterator,
typename EdgeRndOutIt = typename std::vector<typename Network::Edge>::iterator>
LinkPred::DPATPredictor< Network, EdgeRndIt, ScoreRndIt, EdgeRndOutIt >::DPATPredictor (
            DPATPredictor< Network, EdgeRndIt, ScoreRndIt, EdgeRndOutIt > const & that )
[default]
```

Copy constructor.

Parameters

that	The object to copy.

9.24.2.3 DPATPredictor() [3/3]

```
template<typename Network = DNetwork<>, typename EdgeRndIt = typename std::vector<typename
Network::Edge>::const_iterator, typename ScoreRndIt = typename std::vector<double>::iterator,
```

```
typename EdgeRndOutIt = typename std::vector<typename Network::Edge>::iterator>
LinkPred::DPATPredictor< Network, EdgeRndIt, ScoreRndIt, EdgeRndOutIt >::DPATPredictor (
            DPATPredictor< Network, EdgeRndIt, ScoreRndIt, EdgeRndOutIt > && that )  [default]
```

Move constructor.

Parameters

that	The object to move.

9.24.2.4 ∼DPATPredictor()

```
template<typename Network = DNetwork<>, typename EdgeRndIt = typename std::vector<typename
Network::Edge>::const_iterator, typename ScoreRndIt = typename std::vector<double>::iterator,
typename EdgeRndOutIt = typename std::vector<typename Network::Edge>::iterator>
virtual LinkPred::DPATPredictor< Network, EdgeRndIt, ScoreRndIt, EdgeRndOutIt >::~DPATPredictor
( )  [virtual], [default]
```

Finds the k negative edges with the top score. Ties are broken randomly.

Parameters

k	The number of edges to find.
eit	An output iterator where the edges are written.
sit	An output iterator where the scores are written. The scores are written in the same order as the edges.

Returns

The number of negative edges inserted. It is the minimum between k and the number of negative edges in the network. Destructor.

9.24.3 Member Function Documentation

9.24.3.1 init()

```
template<typename Network = DNetwork<>, typename EdgeRndIt = typename std::vector<typename
Network::Edge>::const_iterator, typename ScoreRndIt = typename std::vector<double>::iterator,
typename EdgeRndOutIt = typename std::vector<typename Network::Edge>::iterator>
virtual void LinkPred::DPATPredictor< Network, EdgeRndIt, ScoreRndIt, EdgeRndOutIt >::init ( )
[inline], [virtual]
```

Initialize the solver.

Implements LinkPred::DLPredictor< DNetwork<>, typename std::vector< typename DNetwork<> ::Edge >::const_iterator, typena

9.24.3.2 learn()

```
template<typename Network = DNetwork<>, typename EdgeRndIt = typename std::vector<typename
Network::Edge>::const_iterator, typename ScoreRndIt = typename std::vector<double>::iterator,
typename EdgeRndOutIt = typename std::vector<typename Network::Edge>::iterator>
virtual void LinkPred::DPATPredictor< Network, EdgeRndIt, ScoreRndIt, EdgeRndOutIt >::learn (
) [inline], [virtual]
```

Learn.

Implements LinkPred::DLPredictor< DNetwork<>, typename std::vector< typename DNetwork<> ::Edge >::const_iterator, typena

9.24.3.3 operator=() [1/2]

```
template<typename Network = DNetwork<>, typename EdgeRndIt = typename std::vector<typename
Network::Edge>::const_iterator, typename ScoreRndIt = typename std::vector<double>::iterator,
typename EdgeRndOutIt = typename std::vector<typename Network::Edge>::iterator>
DPATPredictor& LinkPred::DPATPredictor< Network, EdgeRndIt, ScoreRndIt, EdgeRndOutIt >::operator=
(
            DPATPredictor< Network, EdgeRndIt, ScoreRndIt, EdgeRndOutIt > && that )  [default]
```

Move assignment operator.

Parameters

that	The object to move.

9.24.3.4 operator=() [2/2]

```
template<typename Network = DNetwork<>, typename EdgeRndIt = typename std::vector<typename
Network::Edge>::const_iterator, typename ScoreRndIt = typename std::vector<double>::iterator,
typename EdgeRndOutIt = typename std::vector<typename Network::Edge>::iterator>
DPATPredictor& LinkPred::DPATPredictor< Network, EdgeRndIt, ScoreRndIt, EdgeRndOutIt >::operator=
(
            DPATPredictor< Network, EdgeRndIt, ScoreRndIt, EdgeRndOutIt > const & that )
[default]
```

Copy assignment operator.

Parameters

that	The object to copy.

9.24.3.5 predict()

```
template<typename Network = DNetwork<>, typename EdgeRndIt = typename std::vector<typename
Network::Edge>::const_iterator, typename ScoreRndIt = typename std::vector<double>::iterator,
typename EdgeRndOutIt = typename std::vector<typename Network::Edge>::iterator>
virtual void LinkPred::DPATPredictor< Network, EdgeRndIt, ScoreRndIt, EdgeRndOutIt >::predict
(
            EdgeRndIt begin,
            EdgeRndIt end,
            ScoreRndIt scores )  [virtual]
```

Predict the links.

Parameters

begin	Beginning of the links to be predicted.
end	end of the links to be predicted.
scores	Beginning of scores.

Reimplemented from LinkPred::DLPredictor< DNetwork<>, typename std::vector< typename DNetwork<> ::Edge >::const_iterat

9.24.3.6 score()

```
template<typename Network = DNetwork<>, typename EdgeRndIt = typename std::vector<typename
Network::Edge>::const_iterator, typename ScoreRndIt = typename std::vector<double>::iterator,
typename EdgeRndOutIt = typename std::vector<typename Network::Edge>::iterator>
virtual double LinkPred::DPATPredictor< Network, EdgeRndIt, ScoreRndIt, EdgeRndOutIt >::score
(
            Edge const & e )  [virtual]
```

Compute the score of a single edge.

Parameters

e	The edge.

Returns

The score of e.

The documentation for this class was generated from the following file:

- include/linkpred/predictors/directed/dpatpredictor.hpp

9.25 LinkPred::DPSTPredictor< Network, EdgeRndIt, ScoreRndIt, EdgeRndOutIt > Class Template Reference

A link predictor that prestores edge scores. This allows to seemingly integrate results from external link prediction algorithms to LinkPred (for example, users may implement their own link prediction algorithm and then use this link predictor to use compare their results to algorithms available in LinkPred).

```
#include <dpstpredictor.hpp>
```

Inheritance diagram for LinkPred::DPSTPredictor< Network, EdgeRndIt, ScoreRndIt, EdgeRndOutIt >:

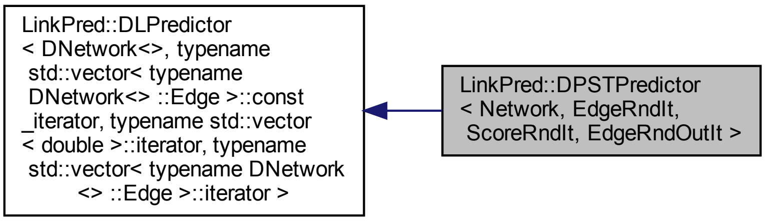

Collaboration diagram for LinkPred::DPSTPredictor< Network, EdgeRndIt, ScoreRndIt, EdgeRndOutIt >:

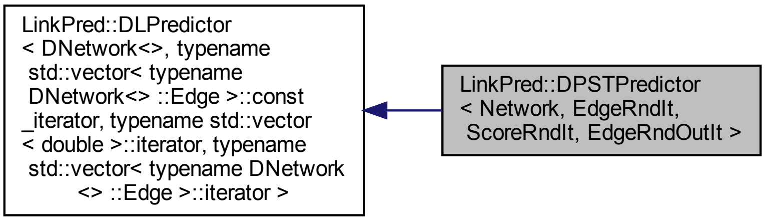

Public Member Functions

- DPSTPredictor (std::shared_ptr< Network const > net)
- DPSTPredictor (DPSTPredictor const &that)=default
- DPSTPredictor & operator= (DPSTPredictor const &that)=default
- DPSTPredictor (DPSTPredictor &&that)=default
- DPSTPredictor & operator= (DPSTPredictor &&that)=default
- void setEdgeScores (typename Network::template EdgeMapSP< double > edgeScores)
- void loadEdgeScores (std::string fileName)
- virtual void init ()
- virtual void learn ()
- virtual double score (Edge const &e)
- virtual ~DPSTPredictor ()=default

Additional Inherited Members

9.25.1 Detailed Description

template<typename Network = DNetwork<>, typename EdgeRndIt = typename std::vector<typename Network::Edge>::const_iterator, typename ScoreRndIt = typename std::vector<double>::iterator, typename EdgeRndOutIt = typename std::vector<typename Network::Edge>::iterator>
class LinkPred::DPSTPredictor< Network, EdgeRndIt, ScoreRndIt, EdgeRndOutIt >

A link predictor that prestores edge scores. This allows to seemingly integrate results from external link prediction algorithms to LinkPred (for example, users may implement their own link prediction algorithm and then use this link predictor to use compare their results to algorithms available in LinkPred).

Template Parameters

Network	The network type.
EdgeRndIt	A random iterator type used to iterate on edges.
ScoreRndIt	A random iterator type used to iterate on scores.

9.25.2 Constructor & Destructor Documentation

9.25.2.1 DPSTPredictor() [1/3]

```
template<typename Network = DNetwork<>, typename EdgeRndIt = typename std::vector<typename
Network::Edge>::const_iterator, typename ScoreRndIt = typename std::vector<double>::iterator,
typename EdgeRndOutIt = typename std::vector<typename Network::Edge>::iterator>
LinkPred::DPSTPredictor< Network, EdgeRndIt, ScoreRndIt, EdgeRndOutIt >::DPSTPredictor (
            std::shared_ptr< Network const > net ) [inline]
```

Parameters

net	The network.

9.25.2.2 DPSTPredictor() [2/3]

```
template<typename Network = DNetwork<>, typename EdgeRndIt = typename std::vector<typename
Network::Edge>::const_iterator, typename ScoreRndIt = typename std::vector<double>::iterator,
typename EdgeRndOutIt = typename std::vector<typename Network::Edge>::iterator>
LinkPred::DPSTPredictor< Network, EdgeRndIt, ScoreRndIt, EdgeRndOutIt >::DPSTPredictor (
            DPSTPredictor< Network, EdgeRndIt, ScoreRndIt, EdgeRndOutIt > const & that )
[default]
```

Copy constructor.

Parameters

that	The object to copy.

9.25.2.3 DPSTPredictor() [3/3]

```
template<typename Network = DNetwork<>, typename EdgeRndIt = typename std::vector<typename
Network::Edge>::const_iterator, typename ScoreRndIt = typename std::vector<double>::iterator,
typename EdgeRndOutIt = typename std::vector<typename Network::Edge>::iterator>
LinkPred::DPSTPredictor< Network, EdgeRndIt, ScoreRndIt, EdgeRndOutIt >::DPSTPredictor (
            DPSTPredictor< Network, EdgeRndIt, ScoreRndIt, EdgeRndOutIt > && that )  [default]
```

Move constructor.

Parameters

that	The object to move.

9.25.2.4 ∼DPSTPredictor()

```
template<typename Network = DNetwork<>, typename EdgeRndIt = typename std::vector<typename
Network::Edge>::const_iterator, typename ScoreRndIt = typename std::vector<double>::iterator,
typename EdgeRndOutIt = typename std::vector<typename Network::Edge>::iterator>
virtual LinkPred::DPSTPredictor< Network, EdgeRndIt, ScoreRndIt, EdgeRndOutIt >::∼DPSTPredictor
( )  [virtual], [default]
```

Destructor.

9.25.3 Member Function Documentation

9.25.3.1 init()

```
template<typename Network = DNetwork<>, typename EdgeRndIt = typename std::vector<typename
Network::Edge>::const_iterator, typename ScoreRndIt = typename std::vector<double>::iterator,
typename EdgeRndOutIt = typename std::vector<typename Network::Edge>::iterator>
virtual void LinkPred::DPSTPredictor< Network, EdgeRndIt, ScoreRndIt, EdgeRndOutIt >::init ( )
[inline], [virtual]
```

Initialize the solver.

Implements LinkPred::DLPredictor< DNetwork<>, typename std::vector< typename DNetwork<> ::Edge >::const_iterator, typena

9.25.3.2 learn()

```
template<typename Network = DNetwork<>, typename EdgeRndIt = typename std::vector<typename
Network::Edge>::const_iterator, typename ScoreRndIt = typename std::vector<double>::iterator,
typename EdgeRndOutIt = typename std::vector<typename Network::Edge>::iterator>
virtual void LinkPred::DPSTPredictor< Network, EdgeRndIt, ScoreRndIt, EdgeRndOutIt >::learn (
) [inline], [virtual]
```

Learn.

Implements LinkPred::DLPredictor< DNetwork<>, typename std::vector< typename DNetwork<> ::Edge >::const_iterator, typena

9.25.3.3 loadEdgeScores()

```
template<typename Network = DNetwork<>, typename EdgeRndIt = typename std::vector<typename
Network::Edge>::const_iterator, typename ScoreRndIt = typename std::vector<double>::iterator,
typename EdgeRndOutIt = typename std::vector<typename Network::Edge>::iterator>
void LinkPred::DPSTPredictor< Network, EdgeRndIt, ScoreRndIt, EdgeRndOutIt >::loadEdgeScores (
            std::string fileName )
```

Load edge scores from file. This file should contain the scores of all non-exisitng links of net.

Parameters

fileName	The name of the file containing edge scores. This should be a text file in which each line contains three columns separate by a space character (one or multiple spaces or tabs). The first two columns contain the the labels (not the internal IDs) of two nodes composing the edge. The third column contains the score. For example: A B 0.35 or: 5 8 2.6 All node labels that appear in this file must already exist in the network.

9.25.3.4 operator=() [1/2]

```
template<typename Network = DNetwork<>, typename EdgeRndIt = typename std::vector<typename
Network::Edge>::const_iterator, typename ScoreRndIt = typename std::vector<double>::iterator,
typename EdgeRndOutIt = typename std::vector<typename Network::Edge>::iterator>
DPSTPredictor& LinkPred::DPSTPredictor< Network, EdgeRndIt, ScoreRndIt, EdgeRndOutIt >::operator=
(
            DPSTPredictor< Network, EdgeRndIt, ScoreRndIt, EdgeRndOutIt > && that ) [default]
```

Move assignment operator.

Parameters

that	The object to move.

9.25.3.5 operator=() [2/2]

```
template<typename Network = DNetwork<>, typename EdgeRndIt = typename std::vector<typename
Network::Edge>::const_iterator, typename ScoreRndIt = typename std::vector<double>::iterator,
typename EdgeRndOutIt = typename std::vector<typename Network::Edge>::iterator>
DPSTPredictor& LinkPred::DPSTPredictor< Network, EdgeRndIt, ScoreRndIt, EdgeRndOutIt >::operator=
(
            DPSTPredictor< Network, EdgeRndIt, ScoreRndIt, EdgeRndOutIt > const & that )
[default]
```

Copy assignment operator.

Parameters

that	The object to copy.

9.25.3.6 score()

```
template<typename Network = DNetwork<>, typename EdgeRndIt = typename std::vector<typename
Network::Edge>::const_iterator, typename ScoreRndIt = typename std::vector<double>::iterator,
typename EdgeRndOutIt = typename std::vector<typename Network::Edge>::iterator>
virtual double LinkPred::DPSTPredictor< Network, EdgeRndIt, ScoreRndIt, EdgeRndOutIt >::score
(
            Edge const & e )  [virtual]
```

Compute the score of a single edge.

Parameters

e	The edge.

Returns

The score of e.

9.25.3.7 setEdgeScores()

```
template<typename Network = DNetwork<>, typename EdgeRndIt = typename std::vector<typename
Network::Edge>::const_iterator, typename ScoreRndIt = typename std::vector<double>::iterator,
typename EdgeRndOutIt = typename std::vector<typename Network::Edge>::iterator>
void LinkPred::DPSTPredictor< Network, EdgeRndIt, ScoreRndIt, EdgeRndOutIt >::setEdgeScores (
            typename Network::template EdgeMapSP< double > edgeScores )  [inline]
```

Set the edge scores. This should contain the scores of all non-exisitng links of net.

Parameters

edgeScores	A map contatining the scores of non-exisitng links of net.

The documentation for this class was generated from the following file:

- include/linkpred/predictors/directed/dpstpredictor.hpp

9.26 LinkPred::DSAIPredictor< Network, EdgeRndIt, ScoreRndIt, EdgeRndOutIt > Class Template Reference

Common neighbor link predictor adapted to directed networks.

`#include <dsaipredictor.hpp>`

Inheritance diagram for LinkPred::DSAIPredictor< Network, EdgeRndIt, ScoreRndIt, EdgeRndOutIt >:

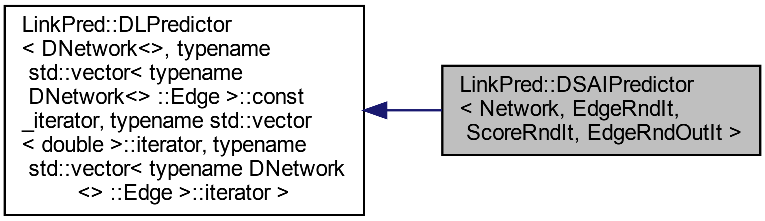

Collaboration diagram for LinkPred::DSAIPredictor< Network, EdgeRndIt, ScoreRndIt, EdgeRndOutIt >:

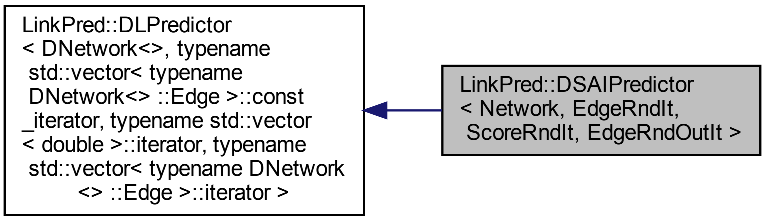

Public Member Functions

- DSAIPredictor (std::shared_ptr< Network const > net)
- DSAIPredictor (DSAIPredictor const &that)=default
- DSAIPredictor & operator= (DSAIPredictor const &that)=default
- DSAIPredictor (DSAIPredictor &&that)=default
- DSAIPredictor & operator= (DSAIPredictor &&that)=default
- virtual void init ()
- virtual void learn ()
- virtual void predict (EdgeRndIt begin, EdgeRndIt end, ScoreRndIt scores)
- virtual double score (Edge const &e)
- virtual std::size_t top (std::size_t k, EdgeRndOutIt eit, ScoreRndIt sit)
- virtual ∼DSAIPredictor ()=default

Additional Inherited Members

9.26.1 Detailed Description

template<typename Network = DNetwork<>, typename EdgeRndIt = typename std::vector<typename Network::Edge>::const_iterator, typename ScoreRndIt = typename std::vector<double>::iterator, typename EdgeRndOutIt = typename std::vector<typename Network::Edge>::iterator>
class LinkPred::DSAIPredictor< Network, EdgeRndIt, ScoreRndIt, EdgeRndOutIt >

Common neighbor link predictor adapted to directed networks.

Template Parameters

Network	The network type.
EdgeRndIt	A random iterator type used to iterate on edges.
ScoreRndIt	A random iterator type used to iterate on scores.

9.26.2 Constructor & Destructor Documentation

9.26.2.1 DSAIPredictor() [1/3]

```
template<typename Network = DNetwork<>, typename EdgeRndIt = typename std::vector<typename
Network::Edge>::const_iterator, typename ScoreRndIt = typename std::vector<double>::iterator,
typename EdgeRndOutIt = typename std::vector<typename Network::Edge>::iterator>
LinkPred::DSAIPredictor< Network, EdgeRndIt, ScoreRndIt, EdgeRndOutIt >::DSAIPredictor (
            std::shared_ptr< Network const > net )  [inline]
```

Parameters

net	The network.

9.26.2.2 DSAIPredictor() [2/3]

```
template<typename Network = DNetwork<>, typename EdgeRndIt = typename std::vector<typename
Network::Edge>::const_iterator, typename ScoreRndIt = typename std::vector<double>::iterator,
typename EdgeRndOutIt = typename std::vector<typename Network::Edge>::iterator>
LinkPred::DSAIPredictor< Network, EdgeRndIt, ScoreRndIt, EdgeRndOutIt >::DSAIPredictor (
            DSAIPredictor< Network, EdgeRndIt, ScoreRndIt, EdgeRndOutIt > const & that )
[default]
```

Copy constructor.

Parameters

that	The object to copy.

9.26.2.3 DSAIPredictor() [3/3]

```
template<typename Network = DNetwork<>, typename EdgeRndIt = typename std::vector<typename
Network::Edge>::const_iterator, typename ScoreRndIt = typename std::vector<double>::iterator,
typename EdgeRndOutIt = typename std::vector<typename Network::Edge>::iterator>
LinkPred::DSAIPredictor< Network, EdgeRndIt, ScoreRndIt, EdgeRndOutIt >::DSAIPredictor (
            DSAIPredictor< Network, EdgeRndIt, ScoreRndIt, EdgeRndOutIt > && that )  [default]
```

Move constructor.

Parameters

that	The object to move.

9.26.2.4 ∼DSAIPredictor()

```
template<typename Network = DNetwork<>, typename EdgeRndIt = typename std::vector<typename
Network::Edge>::const_iterator, typename ScoreRndIt = typename std::vector<double>::iterator,
typename EdgeRndOutIt = typename std::vector<typename Network::Edge>::iterator>
virtual LinkPred::DSAIPredictor< Network, EdgeRndIt, ScoreRndIt, EdgeRndOutIt >::~DSAIPredictor
( )  [virtual], [default]
```

Destructor.

9.26.3 Member Function Documentation

9.26.3.1 init()

```
template<typename Network = DNetwork<>, typename EdgeRndIt = typename std::vector<typename
Network::Edge>::const_iterator, typename ScoreRndIt = typename std::vector<double>::iterator,
typename EdgeRndOutIt = typename std::vector<typename Network::Edge>::iterator>
virtual void LinkPred::DSAIPredictor< Network, EdgeRndIt, ScoreRndIt, EdgeRndOutIt >::init ( )
[inline], [virtual]
```

Initialize the solver.

Implements LinkPred::DLPredictor< DNetwork<>, typename std::vector< typename DNetwork<> ::Edge >::const_iterator, typena

9.26.3.2 learn()

```
template<typename Network = DNetwork<>, typename EdgeRndIt = typename std::vector<typename
Network::Edge>::const_iterator, typename ScoreRndIt = typename std::vector<double>::iterator,
typename EdgeRndOutIt = typename std::vector<typename Network::Edge>::iterator>
virtual void LinkPred::DSAIPredictor< Network, EdgeRndIt, ScoreRndIt, EdgeRndOutIt >::learn (
) [inline], [virtual]
```

Learn.

Implements LinkPred::DLPredictor< DNetwork<>, typename std::vector< typename DNetwork<> ::Edge >::const_iterator, typena

9.26.3.3 operator=() [1/2]

```
template<typename Network = DNetwork<>, typename EdgeRndIt = typename std::vector<typename
Network::Edge>::const_iterator, typename ScoreRndIt = typename std::vector<double>::iterator,
typename EdgeRndOutIt = typename std::vector<typename Network::Edge>::iterator>
DSAIPredictor& LinkPred::DSAIPredictor< Network, EdgeRndIt, ScoreRndIt, EdgeRndOutIt >::operator=
(
            DSAIPredictor< Network, EdgeRndIt, ScoreRndIt, EdgeRndOutIt > && that )   [default]
```

Move assignment operator.

Parameters

that	The object to move.

9.26.3.4 operator=() [2/2]

```
template<typename Network = DNetwork<>, typename EdgeRndIt = typename std::vector<typename
Network::Edge>::const_iterator, typename ScoreRndIt = typename std::vector<double>::iterator,
typename EdgeRndOutIt = typename std::vector<typename Network::Edge>::iterator>
```

```
DSAIPredictor& LinkPred::DSAIPredictor< Network, EdgeRndIt, ScoreRndIt, EdgeRndOutIt >::operator=
(
            DSAIPredictor< Network, EdgeRndIt, ScoreRndIt, EdgeRndOutIt > const & that )
[default]
```

Copy assignment operator.

Parameters

that	The object to copy.

9.26.3.5 predict()

```
template<typename Network = DNetwork<>, typename EdgeRndIt = typename std::vector<typename
Network::Edge>::const_iterator, typename ScoreRndIt = typename std::vector<double>::iterator,
typename EdgeRndOutIt = typename std::vector<typename Network::Edge>::iterator>
virtual void LinkPred::DSAIPredictor< Network, EdgeRndIt, ScoreRndIt, EdgeRndOutIt >::predict
(
            EdgeRndIt begin,
            EdgeRndIt end,
            ScoreRndIt scores )  [virtual]
```

Predict the links.

Parameters

begin	Beginning of the links to be predicted.
end	end of the links to be predicted.
scores	Beginning of scores.

Reimplemented from LinkPred::DLPredictor< DNetwork<>, typename std::vector< typename DNetwork<> ::Edge >::const_iterat

9.26.3.6 score()

```
template<typename Network = DNetwork<>, typename EdgeRndIt = typename std::vector<typename
Network::Edge>::const_iterator, typename ScoreRndIt = typename std::vector<double>::iterator,
typename EdgeRndOutIt = typename std::vector<typename Network::Edge>::iterator>
virtual double LinkPred::DSAIPredictor< Network, EdgeRndIt, ScoreRndIt, EdgeRndOutIt >::score
(
            Edge const & e )  [virtual]
```

Compute the score of a single edge.

Parameters

e	The edge.

Returns

The score of e.

9.26.3.7 top()

```
template<typename Network = DNetwork<>, typename EdgeRndIt = typename std::vector<typename
Network::Edge>::const_iterator, typename ScoreRndIt = typename std::vector<double>::iterator,
typename EdgeRndOutIt = typename std::vector<typename Network::Edge>::iterator>
virtual std::size_t LinkPred::DSAIPredictor< Network, EdgeRndIt, ScoreRndIt, EdgeRndOutIt >↩
::top (
            std::size_t k,
            EdgeRndOutIt eit,
            ScoreRndIt sit )  [virtual]
```

Finds the k negative edges with the top score. Ties are broken randomly.

Parameters

k	The number of edges to find.
eit	An output iterator where the edges are written.
sit	An output iterator where the scores are written. The scores are written in the same order as the edges.

Returns

The number of negative edges inserted. It is the minimum between k and the number of negative edges in the network.

Reimplemented from LinkPred::DLPredictor< DNetwork<>, typename std::vector< typename DNetwork<> ::Edge >::const_iterat

The documentation for this class was generated from the following file:

- include/linkpred/predictors/directed/dsaipredictor.hpp

9.27 LinkPred::DSOIPredictor< Network, EdgeRndIt, ScoreRndIt, EdgeRndOutIt > Class Template Reference

Common neighbor link predictor adapted to directed networks.

```
#include <dsoipredictor.hpp>
```

Inheritance diagram for LinkPred::DSOIPredictor< Network, EdgeRndIt, ScoreRndIt, EdgeRndOutIt >:

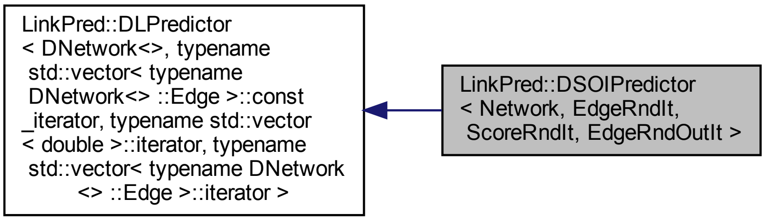

Collaboration diagram for LinkPred::DSOIPredictor< Network, EdgeRndIt, ScoreRndIt, EdgeRndOutIt >:

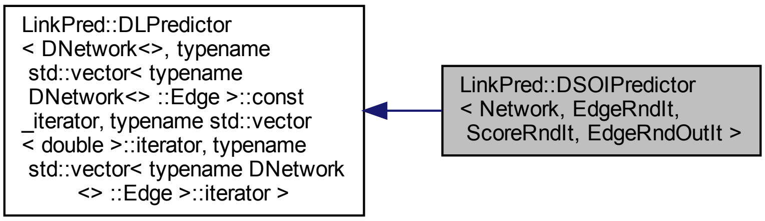

Public Member Functions

- DSOIPredictor (std::shared_ptr< Network const > net)
- DSOIPredictor (DSOIPredictor const &that)=default
- DSOIPredictor & operator= (DSOIPredictor const &that)=default
- DSOIPredictor (DSOIPredictor &&that)=default
- DSOIPredictor & operator= (DSOIPredictor &&that)=default
- virtual void init ()
- virtual void learn ()
- virtual void predict (EdgeRndIt begin, EdgeRndIt end, ScoreRndIt scores)
- virtual double score (Edge const &e)
- virtual std::size_t top (std::size_t k, EdgeRndOutIt eit, ScoreRndIt sit)
- virtual ~DSOIPredictor ()=default

Additional Inherited Members

9.27.1 Detailed Description

**template<typename Network = DNetwork<>, typename EdgeRndIt = typename std::vector<typename Network::Edge>::const_iterator, typename ScoreRndIt = typename std::vector<double>::iterator, typename EdgeRndOutIt = typename std::vector<typename Network::Edge>::iterator>
class LinkPred::DSOIPredictor< Network, EdgeRndIt, ScoreRndIt, EdgeRndOutIt >**

Common neighbor link predictor adapted to directed networks.

Template Parameters

Network	The network type.
Edge↩ RndIt	A random iterator type used to iterate on edges.
Score↩ RndIt	A random iterator type used to iterate on scores.

9.27.2 Constructor & Destructor Documentation

9.27.2.1 DSOIPredictor() [1/3]

```
template<typename Network = DNetwork<>, typename EdgeRndIt = typename std::vector<typename
Network::Edge>::const_iterator, typename ScoreRndIt = typename std::vector<double>::iterator,
typename EdgeRndOutIt = typename std::vector<typename Network::Edge>::iterator>
LinkPred::DSOIPredictor< Network, EdgeRndIt, ScoreRndIt, EdgeRndOutIt >::DSOIPredictor (
            std::shared_ptr< Network const > net )  [inline]
```

Parameters

net	The network.

9.27.2.2 DSOIPredictor() [2/3]

```
template<typename Network = DNetwork<>, typename EdgeRndIt = typename std::vector<typename
Network::Edge>::const_iterator, typename ScoreRndIt = typename std::vector<double>::iterator,
typename EdgeRndOutIt = typename std::vector<typename Network::Edge>::iterator>
LinkPred::DSOIPredictor< Network, EdgeRndIt, ScoreRndIt, EdgeRndOutIt >::DSOIPredictor (
            DSOIPredictor< Network, EdgeRndIt, ScoreRndIt, EdgeRndOutIt > const & that )
[default]
```

Copy constructor.

Parameters

that	The object to copy.

9.27.2.3 DSOIPredictor() [3/3]

```
template<typename Network = DNetwork<>, typename EdgeRndIt = typename std::vector<typename
Network::Edge>::const_iterator, typename ScoreRndIt = typename std::vector<double>::iterator,
```

```
typename EdgeRndOutIt = typename std::vector<typename Network::Edge>::iterator>
LinkPred::DSOIPredictor< Network, EdgeRndIt, ScoreRndIt, EdgeRndOutIt >::DSOIPredictor (
            DSOIPredictor< Network, EdgeRndIt, ScoreRndIt, EdgeRndOutIt > && that )  [default]
```

Move constructor.

Parameters

that	The object to move.

9.27.2.4 ∼DSOIPredictor()

```
template<typename Network = DNetwork<>, typename EdgeRndIt = typename std::vector<typename
Network::Edge>::const_iterator, typename ScoreRndIt = typename std::vector<double>::iterator,
typename EdgeRndOutIt = typename std::vector<typename Network::Edge>::iterator>
virtual LinkPred::DSOIPredictor< Network, EdgeRndIt, ScoreRndIt, EdgeRndOutIt >::∼DSOIPredictor
( )  [virtual], [default]
```

Destructor.

9.27.3 Member Function Documentation

9.27.3.1 init()

```
template<typename Network = DNetwork<>, typename EdgeRndIt = typename std::vector<typename
Network::Edge>::const_iterator, typename ScoreRndIt = typename std::vector<double>::iterator,
typename EdgeRndOutIt = typename std::vector<typename Network::Edge>::iterator>
virtual void LinkPred::DSOIPredictor< Network, EdgeRndIt, ScoreRndIt, EdgeRndOutIt >::init ( )
[inline], [virtual]
```

Initialize the solver.

Implements LinkPred::DLPredictor< DNetwork<>, typename std::vector< typename DNetwork<> ::Edge >::const_iterator, typena

9.27.3.2 learn()

```
template<typename Network = DNetwork<>, typename EdgeRndIt = typename std::vector<typename
Network::Edge>::const_iterator, typename ScoreRndIt = typename std::vector<double>::iterator,
typename EdgeRndOutIt = typename std::vector<typename Network::Edge>::iterator>
virtual void LinkPred::DSOIPredictor< Network, EdgeRndIt, ScoreRndIt, EdgeRndOutIt >::learn (
)  [inline], [virtual]
```

Learn.

Implements LinkPred::DLPredictor< DNetwork<>, typename std::vector< typename DNetwork<> ::Edge >::const_iterator, typena

9.27.3.3 operator=() [1/2]

```
template<typename Network = DNetwork<>, typename EdgeRndIt = typename std::vector<typename
Network::Edge>::const_iterator, typename ScoreRndIt = typename std::vector<double>::iterator,
typename EdgeRndOutIt = typename std::vector<typename Network::Edge>::iterator>
DSOIPredictor& LinkPred::DSOIPredictor< Network, EdgeRndIt, ScoreRndIt, EdgeRndOutIt >::operator=
(
            DSOIPredictor< Network, EdgeRndIt, ScoreRndIt, EdgeRndOutIt > && that )   [default]
```

Move assignment operator.

Parameters

that	The object to move.

9.27.3.4 operator=() [2/2]

```
template<typename Network = DNetwork<>, typename EdgeRndIt = typename std::vector<typename
Network::Edge>::const_iterator, typename ScoreRndIt = typename std::vector<double>::iterator,
typename EdgeRndOutIt = typename std::vector<typename Network::Edge>::iterator>
DSOIPredictor& LinkPred::DSOIPredictor< Network, EdgeRndIt, ScoreRndIt, EdgeRndOutIt >::operator=
(
            DSOIPredictor< Network, EdgeRndIt, ScoreRndIt, EdgeRndOutIt > const & that )
[default]
```

Copy assignment operator.

Parameters

that	The object to copy.

9.27.3.5 predict()

```
template<typename Network = DNetwork<>, typename EdgeRndIt = typename std::vector<typename
Network::Edge>::const_iterator, typename ScoreRndIt = typename std::vector<double>::iterator,
typename EdgeRndOutIt = typename std::vector<typename Network::Edge>::iterator>
virtual void LinkPred::DSOIPredictor< Network, EdgeRndIt, ScoreRndIt, EdgeRndOutIt >::predict
(
            EdgeRndIt begin,
            EdgeRndIt end,
            ScoreRndIt scores )   [virtual]
```

Predict the links.

Parameters

begin	Beginning of the links to be predicted.
end	end of the links to be predicted.
scores	Beginning of scores.

Reimplemented from LinkPred::DLPredictor< DNetwork<>, typename std::vector< typename DNetwork<> ::Edge >::const_iterat

9.27.3.6 score()

```
template<typename Network = DNetwork<>, typename EdgeRndIt = typename std::vector<typename
Network::Edge>::const_iterator, typename ScoreRndIt = typename std::vector<double>::iterator,
typename EdgeRndOutIt = typename std::vector<typename Network::Edge>::iterator>
virtual double LinkPred::DSOIPredictor< Network, EdgeRndIt, ScoreRndIt, EdgeRndOutIt >::score
(
            Edge const & e )  [virtual]
```

Compute the score of a single edge.

Parameters

e	The edge.

Returns

The score of e.

9.27.3.7 top()

```
template<typename Network = DNetwork<>, typename EdgeRndIt = typename std::vector<typename
Network::Edge>::const_iterator, typename ScoreRndIt = typename std::vector<double>::iterator,
typename EdgeRndOutIt = typename std::vector<typename Network::Edge>::iterator>
virtual std::size_t LinkPred::DSOIPredictor< Network, EdgeRndIt, ScoreRndIt, EdgeRndOutIt >↩
::top (
            std::size_t k,
            EdgeRndOutIt eit,
            ScoreRndIt sit )  [virtual]
```

Finds the k negative edges with the top score. Ties are broken randomly.

Parameters

k	The number of edges to find.
eit	An output iterator where the edges are written.
sit	An output iterator where the scores are written. The scores are written in the same order as the edges.

Returns

The number of negative edges inserted. It is the minimum between k and the number of negative edges in the network.

Reimplemented from LinkPred::DLPredictor< DNetwork<>, typename std::vector< typename DNetwork<> ::Edge >::const_iterat

The documentation for this class was generated from the following file:

- include/linkpred/predictors/directed/dsoipredictor.hpp

9.28 LinkPred::Simp::Evaluator::Factory::ECLParams Struct Reference

Parameters of ECL.

```
#include <evaluator.hpp>
```

Public Attributes

- std::string encoderName = "N2V"
- std::string classifierName = "LGR"
- int dim = 0
- double posRatio = 1.0
- double negRatio = 1.0
- long int seed = 0

9.28.1 Detailed Description

Parameters of ECL.

9.28.2 Member Data Documentation

9.28.2.1 classifierName

```
std::string LinkPred::Simp::Evaluator::Factory::ECLParams::classifierName = "LGR"
```

The name of the classifier. Possible values are: DTP (simple dot poroduct between the features), FFN (feed-forward neural network with default architecture), LSVM (linear SVM), LGR (logistic regression), NVB (naive Bayes). All classifiers except logistic regression requirte compilation with mlpack.

9.28.2.2 dim

```
int LinkPred::Simp::Evaluator::Factory::ECLParams::dim = 0
```

The dimension of the embedding space. If set to zero, the default value is used (the default dimension depends on the encoder).

9.28.2.3 encoderName

```
std::string LinkPred::Simp::Evaluator::Factory::ECLParams::encoderName = "N2V"
```

The name of the encoder. Possible values are: DPW (DeepWalk), HMSM (Hidden Metric Space Model), LVS (LargeVis), LEM (Laplacian Eigenmaps), LIN (LINE), LLE (Locally Linear Embedding), MFC (Matrix Factorization) and N2V (Node2Vec).

9.28.2.4 negRatio

```
double LinkPred::Simp::Evaluator::Factory::ECLParams::negRatio = 1.0
```

Ratio of negative edges used in the training of the classifier.

9.28.2.5 posRatio

```
double LinkPred::Simp::Evaluator::Factory::ECLParams::posRatio = 1.0
```

Ratio of positive edges used in the training of the classifier.

9.28.2.6 seed

```
long int LinkPred::Simp::Evaluator::Factory::ECLParams::seed = 0
```

Seed of the random number generator.

The documentation for this struct was generated from the following file:

- include/linkpred/simp/evaluator.hpp

9.29 LinkPred::UNetwork< LabelT, NodeIDT, EdgeT >::EdgeMap< ValueType > Class Template Reference

An edge map.

```
#include <unetwork.hpp>
```

Public Types

- using EdgeMapIt = typename std::map< Edge, ValueType >::iterator
- using EdgeMapConstIt = typename std::map< Edge, ValueType >::const_iterator

Public Member Functions

- EdgeMap (EdgeMap const &that)=default
- EdgeMap & operator= (EdgeMap const &that)=default
- EdgeMap (EdgeMap &&that)=default
- EdgeMap & operator= (EdgeMap &&that)=default
- ValueType operator[ ] (Edge const &e) const
- ValueType & operator[ ] (Edge const &e)
- ValueType at (Edge const &e) const
- EdgeMapConstIt find (Edge const &e) const
- EdgeMapIt find (Edge const &e)
- EdgeMapIt begin ()
- EdgeMapIt end ()
- EdgeMapConstIt cbegin () const
- EdgeMapConstIt cend () const
- std::size_t size ()
- ∼EdgeMap ()=default

Friends

- class **UNetwork**

9.29.1 Detailed Description

template<typename LabelT = std::string, typename NodeIDT = unsigned int, typename EdgeT = unsigned long long int>
template<typename ValueType>
class LinkPred::UNetwork< LabelT, NodeIDT, EdgeT >::EdgeMap< ValueType >

An edge map.

This class can be used to assign a value to every edge in the network. Access to values is done in logarithmic time.

Template Parameters

ValueType	Type of mapped values.

9.29.2 Member Typedef Documentation

9.29.2.1 EdgeMapConstIt

```
template<typename LabelT = std::string, typename NodeIDT = unsigned int, typename EdgeT =
unsigned long long int>
template<typename ValueType >
using LinkPred::UNetwork< LabelT, NodeIDT, EdgeT >::EdgeMap< ValueType >::EdgeMapConstIt =
typename std::map<Edge,ValueType>::const_iterator
```

A constant iterator on the map values.

9.29.2.2 EdgeMapIt

```
template<typename LabelT = std::string, typename NodeIDT = unsigned int, typename EdgeT =
unsigned long long int>
template<typename ValueType >
using LinkPred::UNetwork< LabelT, NodeIDT, EdgeT >::EdgeMap< ValueType >::EdgeMapIt = typename
std::map<Edge, ValueType>::iterator
```

Iterator on the map values.

9.29.3 Constructor & Destructor Documentation

9.29.3.1 EdgeMap() [1/2]

```
template<typename LabelT = std::string, typename NodeIDT = unsigned int, typename EdgeT =
unsigned long long int>
template<typename ValueType >
LinkPred::UNetwork< LabelT, NodeIDT, EdgeT >::EdgeMap< ValueType >::EdgeMap (
            EdgeMap< ValueType > const & that )  [default]
```

Copy constructor.

Parameters

that	The object to copy.

9.29.3.2 EdgeMap() [2/2]

```
template<typename LabelT = std::string, typename NodeIDT = unsigned int, typename EdgeT =
unsigned long long int>
template<typename ValueType >
LinkPred::UNetwork< LabelT, NodeIDT, EdgeT >::EdgeMap< ValueType >::EdgeMap (
            EdgeMap< ValueType > && that )  [default]
```

Move constructor.

Parameters

that	The object to move.

9.29.3.3 ∼EdgeMap()

```
template<typename LabelT = std::string, typename NodeIDT = unsigned int, typename EdgeT =
unsigned long long int>
template<typename ValueType >
LinkPred::UNetwork< LabelT, NodeIDT, EdgeT >::EdgeMap< ValueType >::~EdgeMap ( )  [default]
```

Destructor.

9.29.4 Member Function Documentation

9.29.4.1 at()

```
template<typename LabelT = std::string, typename NodeIDT = unsigned int, typename EdgeT =
unsigned long long int>
template<typename ValueType >
ValueType LinkPred::UNetwork< LabelT, NodeIDT, EdgeT >::EdgeMap< ValueType >::at (
            Edge const & e ) const  [inline]
```

Parameters

e	An edge.

Returns

The value associated with the edge e.

9.29.4.2 begin()

```
template<typename LabelT = std::string, typename NodeIDT = unsigned int, typename EdgeT =
unsigned long long int>
template<typename ValueType >
EdgeMapIt LinkPred::UNetwork< LabelT, NodeIDT, EdgeT >::EdgeMap< ValueType >::begin ( )  [inline]
```

Returns

An iterator to the first element in the map.

9.29.4.3 cbegin()

```
template<typename LabelT = std::string, typename NodeIDT = unsigned int, typename EdgeT =
unsigned long long int>
template<typename ValueType >
EdgeMapConstIt LinkPred::UNetwork< LabelT, NodeIDT, EdgeT >::EdgeMap< ValueType >::cbegin ( )
const  [inline]
```

Returns

A constant iterator to the first element in the map.

9.29.4.4 cend()

```
template<typename LabelT = std::string, typename NodeIDT = unsigned int, typename EdgeT =
unsigned long long int>
template<typename ValueType >
EdgeMapConstIt LinkPred::UNetwork< LabelT, NodeIDT, EdgeT >::EdgeMap< ValueType >::cend ( )
const  [inline]
```

Returns

A constant iterator to one-past-the-last element in the map.

9.29.4.5 end()

```
template<typename LabelT = std::string, typename NodeIDT = unsigned int, typename EdgeT =
unsigned long long int>
template<typename ValueType >
EdgeMapIt LinkPred::UNetwork< LabelT, NodeIDT, EdgeT >::EdgeMap< ValueType >::end ( )  [inline]
```

Returns

An iterator to one-past-the-last element in the map.

9.29.4.6 find() [1/2]

```
template<typename LabelT = std::string, typename NodeIDT = unsigned int, typename EdgeT =
unsigned long long int>
template<typename ValueType >
EdgeMapIt LinkPred::UNetwork< LabelT, NodeIDT, EdgeT >::EdgeMap< ValueType >::find (
            Edge const & e )  [inline]
```

Parameters

e	An edge.

Returns

Searches and return an iterator to the edge e and its value.

9.29.4.7 find() [2/2]

```
template<typename LabelT = std::string, typename NodeIDT = unsigned int, typename EdgeT =
unsigned long long int>
template<typename ValueType >
EdgeMapConstIt LinkPred::UNetwork< LabelT, NodeIDT, EdgeT >::EdgeMap< ValueType >::find (
            Edge const & e ) const  [inline]
```

Parameters

e	An edge.

Returns

Searches and return a const iterator to the edge e and its value.

9.29.4.8 operator=() [1/2]

```
template<typename LabelT = std::string, typename NodeIDT = unsigned int, typename EdgeT =
unsigned long long int>
template<typename ValueType >
EdgeMap& LinkPred::UNetwork< LabelT, NodeIDT, EdgeT >::EdgeMap< ValueType >::operator= (
            EdgeMap< ValueType > && that )  [default]
```

Move assignment operator.

Parameters

that	The object to move.

9.29.4.9 operator=() [2/2]

```
template<typename LabelT = std::string, typename NodeIDT = unsigned int, typename EdgeT =
unsigned long long int>
template<typename ValueType >
EdgeMap& LinkPred::UNetwork< LabelT, NodeIDT, EdgeT >::EdgeMap< ValueType >::operator= (
            EdgeMap< ValueType > const & that )  [default]
```

Copy assignment operator.

Parameters

that	The object to copy.

9.29.4.10 operator[]() [1/2]

```
template<typename LabelT = std::string, typename NodeIDT = unsigned int, typename EdgeT =
unsigned long long int>
template<typename ValueType >
ValueType& LinkPred::UNetwork< LabelT, NodeIDT, EdgeT >::EdgeMap< ValueType >::operator[] (
            Edge const & e )  [inline]
```

Parameters

e	An edge.

Returns

A reference to the value associated with the edge e.

9.29.4.11 operator[]() [2/2]

```
template<typename LabelT = std::string, typename NodeIDT = unsigned int, typename EdgeT =
unsigned long long int>
template<typename ValueType >
ValueType LinkPred::UNetwork< LabelT, NodeIDT, EdgeT >::EdgeMap< ValueType >::operator[] (
            Edge const & e ) const [inline]
```

Parameters

e	An edge.

Returns

The value associated with the edge e.

9.29.4.12 size()

```
template<typename LabelT = std::string, typename NodeIDT = unsigned int, typename EdgeT =
unsigned long long int>
template<typename ValueType >
std::size_t LinkPred::UNetwork< LabelT, NodeIDT, EdgeT >::EdgeMap< ValueType >::size ( )
[inline]
```

Returns

The size of the map.

The documentation for this class was generated from the following file:

- include/linkpred/core/unetwork/unetwork.hpp

9.30 LinkPred::DNetwork< LabelT, NodeIDT, EdgeT >::EdgeMap< ValueType > Class Template Reference

An edge map.

```
#include <dnetwork.hpp>
```

Public Types

- using EdgeMapIt = typename std::map< Edge, ValueType >::iterator
- using EdgeMapConstIt = typename std::map< Edge, ValueType >::const_iterator

Public Member Functions

- EdgeMap (EdgeMap const &that)=default
- EdgeMap & operator= (EdgeMap const &that)=default
- EdgeMap (EdgeMap &&that)=default
- EdgeMap & operator= (EdgeMap &&that)=default
- ValueType operator[ ] (Edge const &e) const
- ValueType & operator[ ] (Edge const &e)
- ValueType at (Edge const &e) const
- EdgeMapConstIt find (Edge const &e) const
- EdgeMapIt find (Edge const &e)
- EdgeMapIt begin ()
- EdgeMapIt end ()
- EdgeMapConstIt cbegin () const
- EdgeMapConstIt cend () const
- std::size_t size ()
- ∼EdgeMap ()=default

Friends

- class **DNetwork**

9.30.1 Detailed Description

template<typename LabelT = std::string, typename NodeIDT = unsigned int, typename EdgeT = unsigned long long int>
template<typename ValueType>
class LinkPred::DNetwork< LabelT, NodeIDT, EdgeT >::EdgeMap< ValueType >

An edge map.

This class can be used to assign a value to every edge in the network. Access to values is done in logarithmic time.

Template Parameters

ValueType	Type of mapped values.

9.30.2 Member Typedef Documentation

9.30.2.1 EdgeMapConstIt

```
template<typename LabelT = std::string, typename NodeIDT = unsigned int, typename EdgeT =
unsigned long long int>
template<typename ValueType >
using LinkPred::DNetwork< LabelT, NodeIDT, EdgeT >::EdgeMap< ValueType >::EdgeMapConstIt =
typename std::map<Edge,ValueType>::const_iterator
```

A constant iterator on the map values.

9.30.2.2 EdgeMapIt

```
template<typename LabelT = std::string, typename NodeIDT = unsigned int, typename EdgeT =
unsigned long long int>
template<typename ValueType >
using LinkPred::DNetwork< LabelT, NodeIDT, EdgeT >::EdgeMap< ValueType >::EdgeMapIt = typename
std::map<Edge, ValueType>::iterator
```

Iterator on the map values.

9.30.3 Constructor & Destructor Documentation

9.30.3.1 EdgeMap() [1/2]

```
template<typename LabelT = std::string, typename NodeIDT = unsigned int, typename EdgeT =
unsigned long long int>
template<typename ValueType >
LinkPred::DNetwork< LabelT, NodeIDT, EdgeT >::EdgeMap< ValueType >::EdgeMap (
            EdgeMap< ValueType > const & that ) [default]
```

Copy constructor.

Parameters

that	The object to copy.

9.30.3.2 EdgeMap() [2/2]

```
template<typename LabelT = std::string, typename NodeIDT = unsigned int, typename EdgeT =
unsigned long long int>
template<typename ValueType >
LinkPred::DNetwork< LabelT, NodeIDT, EdgeT >::EdgeMap< ValueType >::EdgeMap (
            EdgeMap< ValueType > && that ) [default]
```

Move constructor.

Parameters

that	The object to move.

9.30.3.3 ∼EdgeMap()

```
template<typename LabelT = std::string, typename NodeIDT = unsigned int, typename EdgeT =
```

```
unsigned long long int>
template<typename ValueType >
LinkPred::DNetwork< LabelT, NodeIDT, EdgeT >::EdgeMap< ValueType >::~EdgeMap ( )  [default]
```

Destructor.

9.30.4 Member Function Documentation

9.30.4.1 at()

```
template<typename LabelT = std::string, typename NodeIDT = unsigned int, typename EdgeT =
unsigned long long int>
template<typename ValueType >
ValueType LinkPred::DNetwork< LabelT, NodeIDT, EdgeT >::EdgeMap< ValueType >::at (
            Edge const & e ) const  [inline]
```

Parameters

e	An edge.

Returns

The value associated with the edge e.

9.30.4.2 begin()

```
template<typename LabelT = std::string, typename NodeIDT = unsigned int, typename EdgeT =
unsigned long long int>
template<typename ValueType >
EdgeMapIt LinkPred::DNetwork< LabelT, NodeIDT, EdgeT >::EdgeMap< ValueType >::begin ( )  [inline]
```

Returns

An iterator to the first element in the map.

9.30.4.3 cbegin()

```
template<typename LabelT = std::string, typename NodeIDT = unsigned int, typename EdgeT =
unsigned long long int>
template<typename ValueType >
EdgeMapConstIt LinkPred::DNetwork< LabelT, NodeIDT, EdgeT >::EdgeMap< ValueType >::cbegin ( )
const  [inline]
```

Returns

A constant iterator to the first element in the map.

9.30.4.4 cend()

```
template<typename LabelT = std::string, typename NodeIDT = unsigned int, typename EdgeT =
unsigned long long int>
template<typename ValueType >
EdgeMapConstIt LinkPred::DNetwork< LabelT, NodeIDT, EdgeT >::EdgeMap< ValueType >::cend ( )
const [inline]
```

Returns

A constant iterator to one-past-the-last element in the map.

9.30.4.5 end()

```
template<typename LabelT = std::string, typename NodeIDT = unsigned int, typename EdgeT =
unsigned long long int>
template<typename ValueType >
EdgeMapIt LinkPred::DNetwork< LabelT, NodeIDT, EdgeT >::EdgeMap< ValueType >::end ( ) [inline]
```

Returns

An iterator to one-past-the-last element in the map.

9.30.4.6 find() [1/2]

```
template<typename LabelT = std::string, typename NodeIDT = unsigned int, typename EdgeT =
unsigned long long int>
template<typename ValueType >
EdgeMapIt LinkPred::DNetwork< LabelT, NodeIDT, EdgeT >::EdgeMap< ValueType >::find (
            Edge const & e ) [inline]
```

Parameters

e	An edge.

Returns

Searches and return an iterator to the edge e and its value.

9.30.4.7 find() [2/2]

```
template<typename LabelT = std::string, typename NodeIDT = unsigned int, typename EdgeT =
unsigned long long int>
```

```
template<typename ValueType >
EdgeMapConstIt LinkPred::DNetwork< LabelT, NodeIDT, EdgeT >::EdgeMap< ValueType >::find (
            Edge const & e ) const  [inline]
```

Parameters

e	An edge.

Returns

Searches and return a const iterator to the edge e and its value.

9.30.4.8 operator=() [1/2]

```
template<typename LabelT = std::string, typename NodeIDT = unsigned int, typename EdgeT =
unsigned long long int>
template<typename ValueType >
EdgeMap& LinkPred::DNetwork< LabelT, NodeIDT, EdgeT >::EdgeMap< ValueType >::operator= (
            EdgeMap< ValueType > && that )  [default]
```

Move assignment operator.

Parameters

that	The object to move.

9.30.4.9 operator=() [2/2]

```
template<typename LabelT = std::string, typename NodeIDT = unsigned int, typename EdgeT =
unsigned long long int>
template<typename ValueType >
EdgeMap& LinkPred::DNetwork< LabelT, NodeIDT, EdgeT >::EdgeMap< ValueType >::operator= (
            EdgeMap< ValueType > const & that )  [default]
```

Copy assignment operator.

Parameters

that	The object to copy.

9.30.4.10 operator[]() [1/2]

```
template<typename LabelT = std::string, typename NodeIDT = unsigned int, typename EdgeT =
```

```
unsigned long long int>
template<typename ValueType >
ValueType& LinkPred::DNetwork< LabelT, NodeIDT, EdgeT >::EdgeMap< ValueType >::operator[] (
            Edge const & e ) [inline]
```

Parameters

e	An edge.

Returns

A reference to the value associated with the edge e.

9.30.4.11 operator[]() [2/2]

```
template<typename LabelT = std::string, typename NodeIDT = unsigned int, typename EdgeT =
unsigned long long int>
template<typename ValueType >
ValueType LinkPred::DNetwork< LabelT, NodeIDT, EdgeT >::EdgeMap< ValueType >::operator[] (
            Edge const & e ) const [inline]
```

Parameters

e	An edge.

Returns

The value associated with the edge e.

9.30.4.12 size()

```
template<typename LabelT = std::string, typename NodeIDT = unsigned int, typename EdgeT =
unsigned long long int>
template<typename ValueType >
std::size_t LinkPred::DNetwork< LabelT, NodeIDT, EdgeT >::EdgeMap< ValueType >::size ( )
[inline]
```

Returns

The size of the map.

The documentation for this class was generated from the following file:

- include/linkpred/core/dnetwork/dnetwork.hpp

9.31 LinkPred::Utils::EdgeScore< Label > Struct Template Reference

A structure to store the score of an edge.

```
#include <miscutils.hpp>
```

Public Attributes

- Label i
- Label j
- double score

9.31.1 Detailed Description

template<typename Label = std::string>
struct LinkPred::Utils::EdgeScore< Label >

A structure to store the score of an edge.

Template Parameters

Label	The label type.

9.31.2 Member Data Documentation

9.31.2.1 i

```
template<typename Label = std::string>
Label LinkPred::Utils::EdgeScore< Label >::i
```

The start node.

9.31.2.2 j

```
template<typename Label = std::string>
Label LinkPred::Utils::EdgeScore< Label >::j
```

The end node.

9.31.2.3 score

```
template<typename Label = std::string>
double LinkPred::Utils::EdgeScore< Label >::score
```

The score.

The documentation for this struct was generated from the following file:

- include/linkpred/utils/miscutils.hpp

9.32 LinkPred::Simp::EdgeScore Struct Reference

A structure to store the score of an edge.

```
#include <edgescore.hpp>
```

Public Attributes

- std::string i
- std::string j
- double score

9.32.1 Detailed Description

A structure to store the score of an edge.

9.32.2 Member Data Documentation

9.32.2.1 i

```
std::string LinkPred::Simp::EdgeScore::i
```

The label of the start node.

9.32.2.2 j

```
std::string LinkPred::Simp::EdgeScore::j
```

The label of the end node.

9.32.2.3 score

`double LinkPred::Simp::EdgeScore::score`

The score.

The documentation for this struct was generated from the following file:

- include/linkpred/simp/edgescore.hpp

9.33 LinkPred::Simp::EdgeScoreByID Struct Reference

A structure to store the score of an edge. The node IDs are used instead of labels.

`#include <edgescore.hpp>`

Public Attributes

- int i
- int j
- double score

9.33.1 Detailed Description

A structure to store the score of an edge. The node IDs are used instead of labels.

This is useful for reducing memory consumption for large networks.

9.33.2 Member Data Documentation

9.33.2.1 i

`int LinkPred::Simp::EdgeScoreByID::i`

The ID of the start node.

9.33.2.2 j

`int LinkPred::Simp::EdgeScoreByID::j`

The ID of the end node.

9.33.2.3 score

```
double LinkPred::Simp::EdgeScoreByID::score
```

The score.

The documentation for this struct was generated from the following file:

- include/linkpred/simp/edgescore.hpp

9.34 LinkPred::Encoder< Network > Class Template Reference

The interface of a network encoder.

```
#include <encoder.hpp>
```

Public Types

- using NodeID = typename Network::NodeID
- using Edge = typename Network::Edge
- using CodeMap = typename Network::template NodeMap< Vec >
- using CodeMapSP = typename Network::template NodeMapSP< Vec >
- using WeightMap = typename Network::template EdgeMap< double >
- using WeightMapSP = typename Network::template EdgeMapSP< double >

Public Member Functions

- Encoder (std::shared_ptr< Network const > net)
- Encoder (Encoder const &that)=default
- Encoder & operator= (Encoder const &that)=default
- Encoder (Encoder &&that)=default
- Encoder & operator= (Encoder &&that)=default
- virtual void init ()=0
- virtual void encode ()=0
- CodeMapSP getNodeCodeMap ()
- Vec getNodeCode (NodeID const &i)
- virtual Vec getEdgeCode (NodeID const &i, NodeID const &j)
- Vec getEdgeCode (Edge const &e)
- virtual int getEdgeCodeDim () const
- int getDim () const
- void setDim (int dim)
- const std::string & getName () const
- void setName (const std::string &name)
- const WeightMapSP & getWeightMap () const
- virtual void setWeightMap (const WeightMapSP &weightMap)
- virtual ~Encoder ()=default

9.34.1 Detailed Description

template<typename Network = UNetwork<>>
class LinkPred::Encoder< Network >

The interface of a network encoder.

Template Parameters

Network	The network type.

9.34.2 Member Typedef Documentation

9.34.2.1 CodeMap

```
template<typename Network = UNetwork<>>
using LinkPred::Encoder< Network >::CodeMap = typename Network::template NodeMap<Vec>
```

Code map type.

9.34.2.2 CodeMapSP

```
template<typename Network = UNetwork<>>
using LinkPred::Encoder< Network >::CodeMapSP = typename Network::template NodeMapSP<Vec>
```

Shared pointer to a code map.

9.34.2.3 Edge

```
template<typename Network = UNetwork<>>
using LinkPred::Encoder< Network >::Edge = typename Network::Edge
```

Edge type.

9.34.2.4 NodeID

```
template<typename Network = UNetwork<>>
using LinkPred::Encoder< Network >::NodeID = typename Network::NodeID
```

Node ID type.

9.34.2.5 WeightMap

```
template<typename Network = UNetwork<>>
using LinkPred::Encoder< Network >::WeightMap = typename Network::template EdgeMap<double>
```

Edge weight map type.

9.34.2.6 WeightMapSP

```
template<typename Network = UNetwork<>>
using LinkPred::Encoder< Network >::WeightMapSP = typename Network::template EdgeMapSP<double>
```

Shared pointer to an edge weight map.

9.34.3 Constructor & Destructor Documentation

9.34.3.1 Encoder() [1/3]

```
template<typename Network = UNetwork<>>
LinkPred::Encoder< Network >::Encoder (
            std::shared_ptr< Network const > net )  [inline]
```

Constructor.

Parameters

net	The network.

9.34.3.2 Encoder() [2/3]

```
template<typename Network = UNetwork<>>
LinkPred::Encoder< Network >::Encoder (
            Encoder< Network > const & that )  [default]
```

Copy constructor.

Parameters

that	The object to copy.

9.34.3.3 Encoder() [3/3]

```
template<typename Network = UNetwork<>>
LinkPred::Encoder< Network >::Encoder (
            Encoder< Network > && that )  [default]
```

Move constructor.

Parameters

that	The object to move.

9.34.3.4 ∼Encoder()

```
template<typename Network = UNetwork<>>
virtual LinkPred::Encoder< Network >::∼Encoder ( )  [virtual], [default]
```

Destructor.

9.34.4 Member Function Documentation

9.34.4.1 encode()

```
template<typename Network = UNetwork<>>
virtual void LinkPred::Encoder< Network >::encode ( )  [pure virtual]
```

Encode the network.

Implemented in LinkPred::LINE< Network >, LinkPred::Node2Vec< Network >, LinkPred::DeepWalk< Network >, LinkPred::LargeVis< Network >, LinkPred::MatFact< Network >, and LinkPred::HMSM< Network >.

9.34.4.2 getDim()

```
template<typename Network = UNetwork<>>
int LinkPred::Encoder< Network >::getDim ( ) const  [inline]
```

Returns

The dimension of the embedding (node embedding).

9.34.4.3 getEdgeCode() [1/2]

```
template<typename Network = UNetwork<>>
Vec LinkPred::Encoder< Network >::getEdgeCode (
            Edge const & e )  [inline]
```

Shortcut for the previous method.

Parameters

e	The edge.

Returns

The encoding of e.

9.34.4.4 getEdgeCode() [2/2]

```
template<typename Network = UNetwork<>>
virtual Vec LinkPred::Encoder< Network >::getEdgeCode (
            NodeID const & i,
            NodeID const & j )  [inline], [virtual]
```

Return the code of given edge. This is a default implementation that simply concatenates the two node codes. Sub-classes can override this implementation and provide a more complex one.

Parameters

i	The start node ID.
j	The end node ID.

Returns

The encoding of edge (i,j).

9.34.4.5 getEdgeCodeDim()

```
template<typename Network = UNetwork<>>
virtual int LinkPred::Encoder< Network >::getEdgeCodeDim ( ) const  [inline], [virtual]
```

This corresponds to the default edge encoding (see the method getEdgeCode).

Returns

The dimension of the edge codes.

9.34.4.6 getName()

```
template<typename Network = UNetwork<>>
const std::string& LinkPred::Encoder< Network >::getName ( ) const  [inline]
```

Returns

The name of the encoder.

9.34.4.7 getNodeCode()

```
template<typename Network = UNetwork<>>
Vec LinkPred::Encoder< Network >::getNodeCode (
            NodeID const & i )  [inline]
```

Return the code of given node.

Parameters

i	The node ID.

Returns

The encoding of node i.

9.34.4.8 getNodeCodeMap()

```
template<typename Network = UNetwork<>>
CodeMapSP LinkPred::Encoder< Network >::getNodeCodeMap ( )  [inline]
```

Return the encoding of all nodes in the network.

Returns

The network encoding in the form of a node map.

9.34.4.9 getWeightMap()

```
template<typename Network = UNetwork<>>
const WeightMapSP& LinkPred::Encoder< Network >::getWeightMap ( ) const  [inline]
```

Returns

The edge weight map.

9.34.4.10 init()

```
template<typename Network = UNetwork<>>
virtual void LinkPred::Encoder< Network >::init ( )  [pure virtual]
```

Initialize encoder.

Implemented in LinkPred::LINE< Network >, LinkPred::Node2Vec< Network >, LinkPred::DeepWalk< Network >, LinkPred::LargeVis< Network >, LinkPred::MatFact< Network >, and LinkPred::HMSM< Network >.

9.34.4.11 operator=() [1/2]

```
template<typename Network = UNetwork<>>
Encoder& LinkPred::Encoder< Network >::operator= (
            Encoder< Network > && that )  [default]
```

Move assignment operator.

Parameters

that	The object to move.

9.34.4.12 operator=() [2/2]

```
template<typename Network = UNetwork<>>
Encoder& LinkPred::Encoder< Network >::operator= (
            Encoder< Network > const & that )  [default]
```

Copy assignment operator.

Parameters

that	The object to copy.

9.34.4.13 setDim()

```
template<typename Network = UNetwork<>>
void LinkPred::Encoder< Network >::setDim (
            int dim )  [inline]
```

Set the dimension of the embedding (node embedding).

Parameters

dim	The dimension of the embedding (node embedding).

9.34.4.14 setName()

```
template<typename Network = UNetwork<>>
void LinkPred::Encoder< Network >::setName (
            const std::string & name )  [inline]
```

Set the name of the encoder.

Parameters

name	The new name of the encoder.

9.34.4.15 setWeightMap()

```
template<typename Network = UNetwork<>>
virtual void LinkPred::Encoder< Network >::setWeightMap (
            const WeightMapSP & weightMap )  [inline], [virtual]
```

Set The edge weight map.

Parameters

weightMap	The edge weight map.

Reimplemented in LinkPred::Node2Vec< Network >, LinkPred::DeepWalk< Network >, LinkPred::MatFact< Network >, and LinkPred::HMSM< Network >.

The documentation for this class was generated from the following file:

- include/linkpred/graphalg/encoders/encoder.hpp

9.35 LinkPred::Simp::Evaluator::Factory::ESMParams Struct Reference

Parameters of ESM.

```
#include <evaluator.hpp>
```

Public Attributes

- std::string encoderName = "N2V"
- std::string simMeasureName = "L2"
- int dim = 0
- long int seed = 0

9.35.1 Detailed Description

Parameters of ESM.

9.35.2 Member Data Documentation

9.35.2.1 dim

```
int LinkPred::Simp::Evaluator::Factory::ESMParams::dim = 0
```

The dimension of the embedding space. If set to zero, the default value is used (the default dimension depends on the encoder).

9.35.2.2 encoderName

```
std::string LinkPred::Simp::Evaluator::Factory::ESMParams::encoderName = "N2V"
```

The name of the encoder. Possible values are: DPW (DeepWalk), HMSM (Hidden Metric Space Model), LVS (LargeVis), LEM (Laplacian Eigenmaps), LIN (LINE), LLE (Locally Linear Embedding), MFC (Matrix Factorization) and N2V (Node2Vec).

9.35.2.3 seed

```
long int LinkPred::Simp::Evaluator::Factory::ESMParams::seed = 0
```

Seed of the random number generator.

9.35.2.4 simMeasureName

```
std::string LinkPred::Simp::Evaluator::Factory::ESMParams::simMeasureName = "L2"
```

The name of the similarity measure. Possible values are: CSM (cosine similarity), DTP (dot product), L1 (L1 similarity), L2 (L2 similarity), PRS (Pearson similarity).

The documentation for this struct was generated from the following file:

- include/linkpred/simp/evaluator.hpp

9.36 LinkPred::ESPDistCalculator< Network, DistType, NbHopsType > Class Template Reference

Exact shortest path distance calculator.

```
#include <netdistcalculator.hpp>
```

Inheritance diagram for LinkPred::ESPDistCalculator< Network, DistType, NbHopsType >:

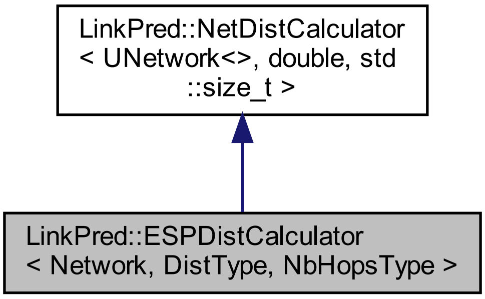

Collaboration diagram for LinkPred::ESPDistCalculator< Network, DistType, NbHopsType >:

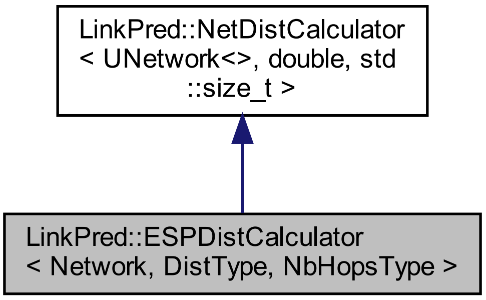

Public Types

- using LengthMapIdType = typename NetDistCalculator< Network, DistType, NbHopsType >::LengthMapIdType
- using NetworkSP = typename NetDistCalculator< Network, DistType, NbHopsType >::NetworkSP
- using NodeID = typename NetDistCalculator< Network, DistType, NbHopsType >::NodeID
- using Label = typename NetDistCalculator< Network, DistType, NbHopsType >::Label
- using Edge = typename NetDistCalculator< Network, DistType, NbHopsType >::Edge
- using NodeDistMap = typename NetDistCalculator< Network, DistType, NbHopsType >::NodeDistMap
- using NodeDistMapSP = typename NetDistCalculator< Network, DistType, NbHopsType >::NodeDistMapSP
- using EdgeLengthMap = typename NetDistCalculator< Network, DistType, NbHopsType >::EdgeLengthMap
- using EdgeLengthMapSP = typename NetDistCalculator< Network, DistType, NbHopsType >::EdgeLengthMapSP

Public Member Functions

- ESPDistCalculator (Dijkstra< Network, DistType, NbHopsType > &dijkstra, EdgeLengthMapSP length, CacheLevel cacheLevel=CacheLevel::NetworkCache)
- ESPDistCalculator (ESPDistCalculator const &that)=default
- ESPDistCalculator & operator= (ESPDistCalculator const &that)=default
- ESPDistCalculator (ESPDistCalculator &&that)=default
- ESPDistCalculator & operator= (ESPDistCalculator &&that)=default
- virtual std::pair< DistType, NbHopsType > getDist (NodeID const &i, NodeID const &j)
- virtual NodeDistMapSP getDist (NodeID const &i)
- virtual std::pair< DistType, NbHopsType > getIndDist (NodeID const &i, NodeID const &j)
- std::size_t getCacheHits () const
- std::size_t getCacheMiss () const
- virtual ~ESPDistCalculator ()

9.36.1 Detailed Description

template<typename Network = UNetwork<>, typename DistType = double, typename NbHopsType = std::size_t>
class LinkPred::ESPDistCalculator< Network, DistType, NbHopsType >

Exact shortest path distance calculator.

This class offers an additional layer over Dijkstra which provides memory management functionalities by caching computed distances.

Template Parameters

Network	The network type.
DistType	The distance type (can be an integer or floating point type).
NbHopsType	The type of the number of hops (must be an integer type).

9.36.2 Member Typedef Documentation

9.36.2.1 Edge

```
template<typename Network = UNetwork<>, typename DistType = double, typename NbHopsType =
std::size_t>
using LinkPred::ESPDistCalculator< Network, DistType, NbHopsType >::Edge = typename NetDistCalculator<Network
DistType, NbHopsType>::Edge
```

Edge type.

9.36.2.2 EdgeLengthMap

```
template<typename Network = UNetwork<>, typename DistType = double, typename NbHopsType =
std::size_t>
using LinkPred::ESPDistCalculator< Network, DistType, NbHopsType >::EdgeLengthMap = typename
NetDistCalculator<Network, DistType, NbHopsType>::EdgeLengthMap
```

Edge length map.

9.36.2.3 EdgeLengthMapSP

```
template<typename Network = UNetwork<>, typename DistType = double, typename NbHopsType =
std::size_t>
using LinkPred::ESPDistCalculator< Network, DistType, NbHopsType >::EdgeLengthMapSP = typename
NetDistCalculator<Network, DistType, NbHopsType>::EdgeLengthMapSP
```

Shared pointer to an edge length map.

9.36.2.4 Label

```
template<typename Network = UNetwork<>, typename DistType = double, typename NbHopsType =
std::size_t>
using LinkPred::ESPDistCalculator< Network, DistType, NbHopsType >::Label = typename NetDistCalculator<Networ
DistType, NbHopsType>::Label
```

Nodes label type.

9.36.2.5 LengthMapIdType

```
template<typename Network = UNetwork<>, typename DistType = double, typename NbHopsType =
std::size_t>
using LinkPred::ESPDistCalculator< Network, DistType, NbHopsType >::LengthMapIdType = typename
NetDistCalculator<Network, DistType, NbHopsType>::LengthMapIdType
```

Length map ID type.

9.36.2.6 NetworkSP

```
template<typename Network = UNetwork<>, typename DistType = double, typename NbHopsType =
std::size_t>
using LinkPred::ESPDistCalculator< Network, DistType, NbHopsType >::NetworkSP = typename
NetDistCalculator<Network, DistType, NbHopsType>::NetworkSP
```

Shared pointer to network.

9.36.2.7 NodeDistMap

```
template<typename Network = UNetwork<>, typename DistType = double, typename NbHopsType =
std::size_t>
using LinkPred::ESPDistCalculator< Network, DistType, NbHopsType >::NodeDistMap = typename
NetDistCalculator<Network, DistType, NbHopsType>::NodeDistMap
```

Distance map.

9.36.2.8 NodeDistMapSP

```
template<typename Network = UNetwork<>, typename DistType = double, typename NbHopsType =
std::size_t>
using LinkPred::ESPDistCalculator< Network, DistType, NbHopsType >::NodeDistMapSP = typename
NetDistCalculator<Network, DistType, NbHopsType>::NodeDistMapSP
```

Shared pointer to a distance map.

9.36.2.9 NodeID

```
template<typename Network = UNetwork<>, typename DistType = double, typename NbHopsType =
std::size_t>
using LinkPred::ESPDistCalculator< Network, DistType, NbHopsType >::NodeID = typename NetDistCalculator<Netwo
DistType, NbHopsType>::NodeID
```

Nodes ID type.

9.36.3 Constructor & Destructor Documentation

9.36.3.1 ESPDistCalculator() [1/3]

```
template<typename Network = UNetwork<>, typename DistType = double, typename NbHopsType =
std::size_t>
LinkPred::ESPDistCalculator< Network, DistType, NbHopsType >::ESPDistCalculator (
            Dijkstra< Network, DistType, NbHopsType > & dijkstra,
            EdgeLengthMapSP length,
            CacheLevel cacheLevel = CacheLevel::NetworkCache )  [inline]
```

Constructor.

Parameters

dijkstra	A Dijkstra algorithm object.
length	The length map.
cacheLevel	The cache level.

9.36.3.2 ESPDistCalculator() [2/3]

```
template<typename Network = UNetwork<>, typename DistType = double, typename NbHopsType =
std::size_t>
LinkPred::ESPDistCalculator< Network, DistType, NbHopsType >::ESPDistCalculator (
            ESPDistCalculator< Network, DistType, NbHopsType > const & that )  [default]
```

Copy constructor.

Parameters

that	The object to copy.

9.36.3.3 ESPDistCalculator() [3/3]

```
template<typename Network = UNetwork<>, typename DistType = double, typename NbHopsType =
std::size_t>
LinkPred::ESPDistCalculator< Network, DistType, NbHopsType >::ESPDistCalculator (
            ESPDistCalculator< Network, DistType, NbHopsType > && that )  [default]
```

Move constructor.

Parameters

that	The object to move.

9.36.3.4 ∼ESPDistCalculator()

```
template<typename Network = UNetwork<>, typename DistType = double, typename NbHopsType =
std::size_t>
virtual LinkPred::ESPDistCalculator< Network, DistType, NbHopsType >::~ESPDistCalculator ( )
[inline], [virtual]
```

Destructor.

9.36.4 Member Function Documentation

9.36.4.1 getCacheHits()

```
template<typename Network = UNetwork<>, typename DistType = double, typename NbHopsType =
std::size_t>
std::size_t LinkPred::ESPDistCalculator< Network, DistType, NbHopsType >::getCacheHits ( )
const [inline]
```

Returns

The number of cache hits.

9.36.4.2 getCacheMiss()

```
template<typename Network = UNetwork<>, typename DistType = double, typename NbHopsType =
std::size_t>
std::size_t LinkPred::ESPDistCalculator< Network, DistType, NbHopsType >::getCacheMiss ( )
const [inline]
```

Returns

The number of cache misses.

9.36.4.3 getDist() [1/2]

```
template<typename Network = UNetwork<>, typename DistType = double, typename NbHopsType =
std::size_t>
virtual NodeDistMapSP LinkPred::ESPDistCalculator< Network, DistType, NbHopsType >::getDist (
            NodeID const & i ) [virtual]
```

Parameters

i	Source node.

Returns

The distance from i to all other nodes.

9.36.4.4 getDist() [2/2]

```
template<typename Network = UNetwork<>, typename DistType = double, typename NbHopsType =
std::size_t>
virtual std::pair<DistType, NbHopsType> LinkPred::ESPDistCalculator< Network, DistType, Nb↩
HopsType >::getDist (
            NodeID const & i,
            NodeID const & j )  [virtual]
```

Parameters

i	Index of the source node.
j	Index of the end node.

Returns

The distance between i and j.

9.36.4.5 getIndDist()

```
template<typename Network = UNetwork<>, typename DistType = double, typename NbHopsType =
std::size_t>
virtual std::pair<DistType, NbHopsType> LinkPred::ESPDistCalculator< Network, DistType, Nb↩
HopsType >::getIndDist (
            NodeID const & i,
            NodeID const & j )  [virtual]
```

Parameters

i	Index of the source node.
j	Index of the end node.

Returns

The distance between i and j ignoring the edge between i and j.

9.36.4.6 operator=() [1/2]

```
template<typename Network = UNetwork<>, typename DistType = double, typename NbHopsType =
std::size_t>
ESPDistCalculator& LinkPred::ESPDistCalculator< Network, DistType, NbHopsType >::operator= (
            ESPDistCalculator< Network, DistType, NbHopsType > && that )  [default]
```

Move assignment operator.

Parameters

that	The object to move.

9.36.4.7 operator=() [2/2]

```
template<typename Network = UNetwork<>, typename DistType = double, typename NbHopsType =
std::size_t>
ESPDistCalculator& LinkPred::ESPDistCalculator< Network, DistType, NbHopsType >::operator= (
            ESPDistCalculator< Network, DistType, NbHopsType > const & that )  [default]
```

Copy assignment operator.

Parameters

that	The object to copy.

The documentation for this class was generated from the following file:

- include/linkpred/graphalg/shortestpaths/netdistcalculator.hpp

9.37 LinkPred::ESPDsimCalculator< Network, DsimType, NbHopsType > Class Template Reference

Exact shortest path dissimilarity calculator.

```
#include <netdistcalculator.hpp>
```

Inheritance diagram for LinkPred::ESPDsimCalculator< Network, DsimType, NbHopsType >:

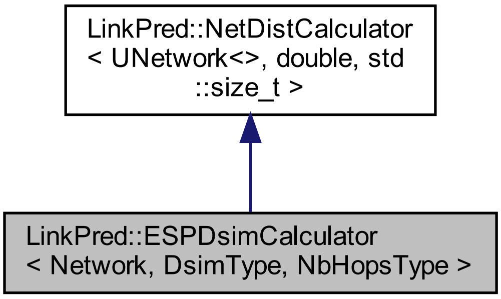

Collaboration diagram for LinkPred::ESPDsimCalculator< Network, DsimType, NbHopsType >:

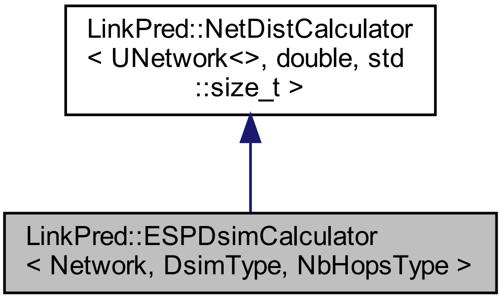

Public Types

- using LengthMapIdType = typename NetDistCalculator< Network, DsimType, NbHopsType >::LengthMapIdType
- using NetworkSP = typename NetDistCalculator< Network, DsimType, NbHopsType >::NetworkSP
- using NodeID = typename NetDistCalculator< Network, DsimType, NbHopsType >::NodeID
- using Label = typename NetDistCalculator< Network, DsimType, NbHopsType >::Label
- using Edge = typename NetDistCalculator< Network, DsimType, NbHopsType >::Edge
- using NodeDsimMap = typename NetDistCalculator< Network, DsimType, NbHopsType >::NodeDistMap
- using NodeDsimMapSP = typename NetDistCalculator< Network, DsimType, NbHopsType >::NodeDistMapSP
- using EdgeLengthMap = typename NetDistCalculator< Network, DsimType, NbHopsType >::EdgeLengthMap
- using EdgeLengthMapSP = typename NetDistCalculator< Network, DsimType, NbHopsType >::EdgeLengthMapSP

Public Member Functions

- ESPDsimCalculator (Dijkstra< Network, DsimType, NbHopsType > &dijkstra, EdgeLengthMapSP length, CacheLevel cacheLevel=CacheLevel::NodeCache)
- ESPDsimCalculator (ESPDsimCalculator const &that)=default
- ESPDsimCalculator & operator= (ESPDsimCalculator const &that)=default
- ESPDsimCalculator (ESPDsimCalculator &&that)=default
- ESPDsimCalculator & operator= (ESPDsimCalculator &&that)=default
- virtual std::pair< DsimType, NbHopsType > getDist (NodeID const &i, NodeID const &j)
- virtual std::pair< DsimType, NbHopsType > getIndDist (NodeID const &i, NodeID const &j)
- virtual NodeDsimMapSP getDist (NodeID const &i)
- virtual ∼ESPDsimCalculator ()

9.37.1 Detailed Description

**template<typename Network = UNetwork<>, typename DsimType = double, typename NbHopsType = std::size_t>
class LinkPred::ESPDsimCalculator< Network, DsimType, NbHopsType >**

Exact shortest path dissimilarity calculator.

Template Parameters

Network	The network type.
DistType	The distance type (can be an integer or floating point type).
NbHopsType	The type of the number of hops (must be an integer type).

9.37.2 Member Typedef Documentation

9.37.2.1 Edge

```
template<typename Network = UNetwork<>, typename DsimType = double, typename NbHopsType =
std::size_t>
using LinkPred::ESPDsimCalculator< Network, DsimType, NbHopsType >::Edge = typename NetDistCalculator<Network
DsimType, NbHopsType>::Edge
```

Edge type.

9.37.2.2 EdgeLengthMap

```
template<typename Network = UNetwork<>, typename DsimType = double, typename NbHopsType =
std::size_t>
using LinkPred::ESPDsimCalculator< Network, DsimType, NbHopsType >::EdgeLengthMap = typename
NetDistCalculator<Network, DsimType, NbHopsType>::EdgeLengthMap
```

Edge length map.

9.37.2.3 EdgeLengthMapSP

```
template<typename Network = UNetwork<>, typename DsimType = double, typename NbHopsType =
std::size_t>
using LinkPred::ESPDsimCalculator< Network, DsimType, NbHopsType >::EdgeLengthMapSP = typename
NetDistCalculator<Network, DsimType, NbHopsType>::EdgeLengthMapSP
```

Shared pointer to an edge length map.

9.37.2.4 Label

```
template<typename Network = UNetwork<>, typename DsimType = double, typename NbHopsType =
std::size_t>
using LinkPred::ESPDsimCalculator< Network, DsimType, NbHopsType >::Label = typename NetDistCalculator<Netwo
DsimType, NbHopsType>::Label
```

Nodes label type.

9.37.2.5 LengthMapIdType

```
template<typename Network = UNetwork<>, typename DsimType = double, typename NbHopsType =
std::size_t>
using LinkPred::ESPDsimCalculator< Network, DsimType, NbHopsType >::LengthMapIdType = typename
NetDistCalculator<Network, DsimType, NbHopsType>::LengthMapIdType
```

Length map ID type.

9.37.2.6 NetworkSP

```
template<typename Network = UNetwork<>, typename DsimType = double, typename NbHopsType =
std::size_t>
using LinkPred::ESPDsimCalculator< Network, DsimType, NbHopsType >::NetworkSP = typename
NetDistCalculator<Network, DsimType, NbHopsType>::NetworkSP
```

Shared pointer to network.

9.37.2.7 NodeDsimMap

```
template<typename Network = UNetwork<>, typename DsimType = double, typename NbHopsType =
std::size_t>
using LinkPred::ESPDsimCalculator< Network, DsimType, NbHopsType >::NodeDsimMap = typename
NetDistCalculator<Network, DsimType, NbHopsType>::NodeDistMap
```

Dissimilarity map.

9.37.2.8 NodeDsimMapSP

```
template<typename Network = UNetwork<>, typename DsimType = double, typename NbHopsType =
std::size_t>
using LinkPred::ESPDsimCalculator< Network, DsimType, NbHopsType >::NodeDsimMapSP = typename
NetDistCalculator<Network, DsimType, NbHopsType>::NodeDistMapSP
```

Shared pointer to a Dissimilarity map.

9.37.2.9 NodeID

```
template<typename Network = UNetwork<>, typename DsimType = double, typename NbHopsType =
std::size_t>
using LinkPred::ESPDsimCalculator< Network, DsimType, NbHopsType >::NodeID = typename NetDistCalculator<Netwo
DsimType, NbHopsType>::NodeID
```

Nodes ID type.

9.37.3 Constructor & Destructor Documentation

9.37.3.1 ESPDsimCalculator() [1/3]

```
template<typename Network = UNetwork<>, typename DsimType = double, typename NbHopsType =
std::size_t>
LinkPred::ESPDsimCalculator< Network, DsimType, NbHopsType >::ESPDsimCalculator (
            Dijkstra< Network, DsimType, NbHopsType > & dijkstra,
            EdgeLengthMapSP length,
            CacheLevel cacheLevel = CacheLevel::NodeCache )  [inline]
```

Constructor.

Parameters

dijkstra	A Dijkstra algorithm object.
length	The length map.
cacheLevel	The cache level.

9.37.3.2 ESPDsimCalculator() [2/3]

```
template<typename Network = UNetwork<>, typename DsimType = double, typename NbHopsType =
std::size_t>
LinkPred::ESPDsimCalculator< Network, DsimType, NbHopsType >::ESPDsimCalculator (
            ESPDsimCalculator< Network, DsimType, NbHopsType > const & that )  [default]
```

Copy constructor.

Parameters

that	The object to copy.

9.37.3.3 ESPDsimCalculator() [3/3]

```
template<typename Network = UNetwork<>, typename DsimType = double, typename NbHopsType =
std::size_t>
LinkPred::ESPDsimCalculator< Network, DsimType, NbHopsType >::ESPDsimCalculator (
            ESPDsimCalculator< Network, DsimType, NbHopsType > && that )  [default]
```

Move constructor.

Parameters

that	The object to move.

9.37.3.4 ∼ESPDsimCalculator()

```
template<typename Network = UNetwork<>, typename DsimType = double, typename NbHopsType =
std::size_t>
virtual LinkPred::ESPDsimCalculator< Network, DsimType, NbHopsType >::~ESPDsimCalculator ( )
[inline], [virtual]
```

Destructor.

9.37.4 Member Function Documentation

9.37.4.1 getDist() [1/2]

```
template<typename Network = UNetwork<>, typename DsimType = double, typename NbHopsType =
std::size_t>
virtual NodeDsimMapSP LinkPred::ESPDsimCalculator< Network, DsimType, NbHopsType >::getDist (
            NodeID const & i )  [virtual]
```

Parameters

i	Source node.

Returns

The distance from i to all other nodes.

9.37.4.2 getDist() [2/2]

```
template<typename Network = UNetwork<>, typename DsimType = double, typename NbHopsType =
std::size_t>
virtual std::pair<DsimType, NbHopsType> LinkPred::ESPDsimCalculator< Network, DsimType, Nb←
HopsType >::getDist (
            NodeID const & i,
            NodeID const & j )  [virtual]
```

Parameters

i	Index of the source node.
j	Index of the end node.

Returns

The distance between i and j.

9.37.4.3 getIndDist()

```
template<typename Network = UNetwork<>, typename DsimType = double, typename NbHopsType =
std::size_t>
virtual std::pair<DsimType, NbHopsType> LinkPred::ESPDsimCalculator< Network, DsimType, Nb←
HopsType >::getIndDist (
            NodeID const & i,
            NodeID const & j )  [virtual]
```

Parameters

i	Index of the source node.
j	Index of the end node.

Returns

The distance between i and j ignoring the edge between i and j.

9.37.4.4 operator=() [1/2]

```
template<typename Network = UNetwork<>, typename DsimType = double, typename NbHopsType =
std::size_t>
ESPDsimCalculator& LinkPred::ESPDsimCalculator< Network, DsimType, NbHopsType >::operator= (
            ESPDsimCalculator< Network, DsimType, NbHopsType > && that )  [default]
```

Move assignment operator.

Parameters

that	The object to move.

9.37.4.5 operator=() [2/2]

```
template<typename Network = UNetwork<>, typename DsimType = double, typename NbHopsType =
std::size_t>
ESPDsimCalculator& LinkPred::ESPDsimCalculator< Network, DsimType, NbHopsType >::operator= (
            ESPDsimCalculator< Network, DsimType, NbHopsType > const & that )  [default]
```

Copy assignment operator.

Parameters

that	The object to copy.

The documentation for this class was generated from the following file:

- include/linkpred/graphalg/shortestpaths/netdistcalculator.hpp

9.38 LinkPred::ESPIndSimlCalculator< Network, DistType, NbHopsType > Class Template Reference

Exact shortest path distance calculator.

```
#include <netdistcalculator.hpp>
```

Inheritance diagram for LinkPred::ESPIndSimlCalculator< Network, DistType, NbHopsType >:

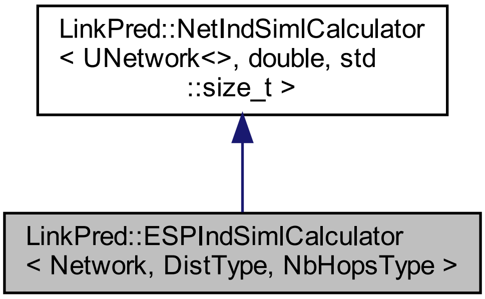

Collaboration diagram for LinkPred::ESPIndSimlCalculator< Network, DistType, NbHopsType >:

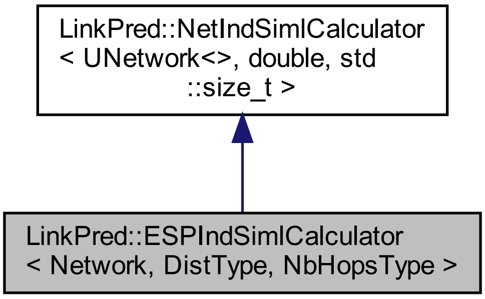

Public Types

- using LengthMapIdType = typename NetIndSimlCalculator< Network, DistType, NbHopsType >↩ ::LengthMapIdType
- using NetworkSP = typename NetIndSimlCalculator< Network, DistType, NbHopsType >::NetworkSP
- using NodeID = typename NetIndSimlCalculator< Network, DistType, NbHopsType >::NodeID
- using Label = typename NetIndSimlCalculator< Network, DistType, NbHopsType >::Label
- using Edge = typename NetIndSimlCalculator< Network, DistType, NbHopsType >::Edge
- using NodeDistMap = typename Network::template NodeMap< std::pair< DistType, NbHopsType > >
- using NodeDistMapSP = typename Network::template NodeMapSP< std::pair< DistType, NbHopsType > >
- using EdgeLengthMap = typename NetIndSimlCalculator< Network, DistType, NbHopsType >↩ ::EdgeLengthMap
- using EdgeLengthMapSP = typename NetIndSimlCalculator< Network, DistType, NbHopsType >↩ ::EdgeLengthMapSP

Public Member Functions

- ESPIndSimlCalculator (Dijkstra< Network, DistType, NbHopsType > &dijkstra, EdgeLengthMapSP length)
- ESPIndSimlCalculator (ESPIndSimlCalculator const &that)=default
- ESPIndSimlCalculator & operator= (ESPIndSimlCalculator const &that)=default
- ESPIndSimlCalculator (ESPIndSimlCalculator &&that)=default
- ESPIndSimlCalculator & operator= (ESPIndSimlCalculator &&that)=default
- virtual std::tuple< DistType, DistType, NbHopsType > getIndSiml (NodeID const &i, NodeID const &j)
- virtual std::tuple< DistType, DistType, NbHopsType > getDirIndSiml (NodeID const &i, NodeID const &j)
- virtual ∼ESPIndSimlCalculator ()

9.38.1 Detailed Description

template<typename Network = UNetwork<>, typename DistType = double, typename NbHopsType = std::size_t>
class LinkPred::ESPIndSimlCalculator< Network, DistType, NbHopsType >

Exact shortest path distance calculator.

This class offers an additional layer over Dijkstra which provides memory management functionalities by caching computed distances.

Template Parameters

Network	The network type.
DistType	The distance type (can be an integer or floating point type).
NbHopsType	The type of the number of hops (must be an integer type).

9.38.2 Member Typedef Documentation

9.38.2.1 Edge

```
template<typename Network = UNetwork<>, typename DistType = double, typename NbHopsType =
std::size_t>
using LinkPred::ESPIndSimlCalculator< Network, DistType, NbHopsType >::Edge = typename NetIndSimlCalculator<N
DistType, NbHopsType>::Edge
```

Edge type.

9.38.2.2 EdgeLengthMap

```
template<typename Network = UNetwork<>, typename DistType = double, typename NbHopsType =
std::size_t>
using LinkPred::ESPIndSimlCalculator< Network, DistType, NbHopsType >::EdgeLengthMap = typename
NetIndSimlCalculator<Network, DistType, NbHopsType>::EdgeLengthMap
```

Edge length map.

9.38.2.3 EdgeLengthMapSP

```
template<typename Network = UNetwork<>, typename DistType = double, typename NbHopsType =
std::size_t>
using LinkPred::ESPIndSimlCalculator< Network, DistType, NbHopsType >::EdgeLengthMapSP =
typename NetIndSimlCalculator<Network, DistType, NbHopsType>::EdgeLengthMapSP
```

Shared pointer to an edge length map.

9.38.2.4 Label

```
template<typename Network = UNetwork<>, typename DistType = double, typename NbHopsType =
std::size_t>
using LinkPred::ESPIndSimlCalculator< Network, DistType, NbHopsType >::Label = typename NetIndSimlCalculator<
DistType, NbHopsType>::Label
```

Nodes label type.

9.38.2.5 LengthMapIdType

```
template<typename Network = UNetwork<>, typename DistType = double, typename NbHopsType =
std::size_t>
using LinkPred::ESPIndSimlCalculator< Network, DistType, NbHopsType >::LengthMapIdType =
typename NetIndSimlCalculator<Network, DistType, NbHopsType>::LengthMapIdType
```

Length map ID type.

9.38.2.6 NetworkSP

```
template<typename Network = UNetwork<>, typename DistType = double, typename NbHopsType =
std::size_t>
using LinkPred::ESPIndSimlCalculator< Network, DistType, NbHopsType >::NetworkSP = typename
NetIndSimlCalculator<Network, DistType, NbHopsType>::NetworkSP
```

Shared pointer to network.

9.38.2.7 NodeDistMap

```
template<typename Network = UNetwork<>, typename DistType = double, typename NbHopsType =
std::size_t>
using LinkPred::ESPIndSimlCalculator< Network, DistType, NbHopsType >::NodeDistMap = typename
Network::template NodeMap<std::pair<DistType, NbHopsType> >
```

Distance map.

9.38.2.8 NodeDistMapSP

```
template<typename Network = UNetwork<>, typename DistType = double, typename NbHopsType =
std::size_t>
using LinkPred::ESPIndSimlCalculator< Network, DistType, NbHopsType >::NodeDistMapSP = typename
Network::template NodeMapSP<std::pair<DistType, NbHopsType> >
```

Shared pointer to a distance map.

9.38.2.9 NodeID

```
template<typename Network = UNetwork<>, typename DistType = double, typename NbHopsType =
std::size_t>
using LinkPred::ESPIndSimlCalculator< Network, DistType, NbHopsType >::NodeID = typename
NetIndSimlCalculator<Network, DistType, NbHopsType>::NodeID
```

Nodes ID type.

9.38.3 Constructor & Destructor Documentation

9.38.3.1 ESPIndSimlCalculator() [1/3]

```
template<typename Network = UNetwork<>, typename DistType = double, typename NbHopsType =
std::size_t>
LinkPred::ESPIndSimlCalculator< Network, DistType, NbHopsType >::ESPIndSimlCalculator (
            Dijkstra< Network, DistType, NbHopsType > & dijkstra,
            EdgeLengthMapSP length ) [inline]
```

Constructor.

Parameters

dijkstra	A Dijkstra algorithm object.
length	The length map.

9.38.3.2 ESPIndSimlCalculator() [2/3]

```
template<typename Network = UNetwork<>, typename DistType = double, typename NbHopsType =
std::size_t>
LinkPred::ESPIndSimlCalculator< Network, DistType, NbHopsType >::ESPIndSimlCalculator (
            ESPIndSimlCalculator< Network, DistType, NbHopsType > const & that ) [default]
```

Copy constructor.

Parameters

that	The object to copy.

9.38.3.3 ESPIndSimlCalculator() [3/3]

```
template<typename Network = UNetwork<>, typename DistType = double, typename NbHopsType =
std::size_t>
```

```
LinkPred::ESPIndSimlCalculator< Network, DistType, NbHopsType >::ESPIndSimlCalculator (
            ESPIndSimlCalculator< Network, DistType, NbHopsType > && that )  [default]
```

Move constructor.

Parameters

that	The object to move.

9.38.3.4 ∼ESPIndSimlCalculator()

```
template<typename Network = UNetwork<>, typename DistType = double, typename NbHopsType =
std::size_t>
virtual LinkPred::ESPIndSimlCalculator< Network, DistType, NbHopsType >::~ESPIndSimlCalculator
( )  [inline], [virtual]
```

Destructor.

9.38.4 Member Function Documentation

9.38.4.1 getDirIndSiml()

```
template<typename Network = UNetwork<>, typename DistType = double, typename NbHopsType =
std::size_t>
virtual std::tuple<DistType, DistType, NbHopsType> LinkPred::ESPIndSimlCalculator< Network,
DistType, NbHopsType >::getDirIndSiml (
            NodeID const & i,
            NodeID const & j )  [virtual]
```

Parameters

i	Index of the source node.
j	Index of the end node.

Returns

The directed similarity between i and j ignoring the edge between i and j.

9.38.4.2 getIndSiml()

```
template<typename Network = UNetwork<>, typename DistType = double, typename NbHopsType =
std::size_t>
```

```
virtual std::tuple<DistType, DistType, NbHopsType> LinkPred::ESPIndSimlCalculator< Network,
DistType, NbHopsType >::getIndSiml (
            NodeID const & i,
            NodeID const & j )  [virtual]
```

Parameters

i	Index of the source node.
j	Index of the end node.

Returns

The similarity between i and j ignoring the edge between i and j.

9.38.4.3 operator=() [1/2]

```
template<typename Network = UNetwork<>, typename DistType = double, typename NbHopsType =
std::size_t>
ESPIndSimlCalculator& LinkPred::ESPIndSimlCalculator< Network, DistType, NbHopsType >::operator=
(
            ESPIndSimlCalculator< Network, DistType, NbHopsType > && that )  [default]
```

Move assignment operator.

Parameters

that	The object to move.

9.38.4.4 operator=() [2/2]

```
template<typename Network = UNetwork<>, typename DistType = double, typename NbHopsType =
std::size_t>
ESPIndSimlCalculator& LinkPred::ESPIndSimlCalculator< Network, DistType, NbHopsType >::operator=
(
            ESPIndSimlCalculator< Network, DistType, NbHopsType > const & that )  [default]
```

Copy assignment operator.

Parameters

that	The object to copy.

The documentation for this class was generated from the following file:

- include/linkpred/graphalg/shortestpaths/netdistcalculator.hpp

9.39 LinkPred::ESPLDistCalculator< Network, DistType, NbHopsType > Class Template Reference

Exact shortest path distance calculator with limits on the number of hops.

```
#include <netdistcalculator.hpp>
```

Inheritance diagram for LinkPred::ESPLDistCalculator< Network, DistType, NbHopsType >:

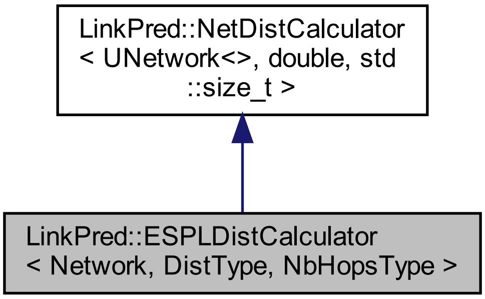

Collaboration diagram for LinkPred::ESPLDistCalculator< Network, DistType, NbHopsType >:

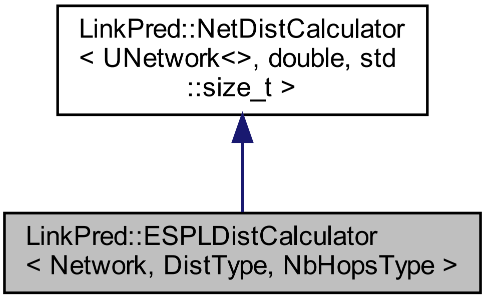

Public Types

- using LengthMapIdType = typename NetDistCalculator< Network, DistType, NbHopsType >↩ ::LengthMapIdType
- using NetworkSP = typename NetDistCalculator< Network, DistType, NbHopsType >::NetworkSP
- using NodeID = typename NetDistCalculator< Network, DistType, NbHopsType >::NodeID
- using Label = typename NetDistCalculator< Network, DistType, NbHopsType >::Label
- using Edge = typename NetDistCalculator< Network, DistType, NbHopsType >::Edge

- using NodeDistMap = typename NetDistCalculator< Network, DistType, NbHopsType >::NodeDistMap
- using NodeDistMapSP = typename NetDistCalculator< Network, DistType, NbHopsType >::NodeDistMapSP
- using NodeSDistMap = typename Network::template NodeSMap< std::pair< DistType, NbHopsType > >
- using NodeSDistMapSP = typename Network::template NodeSMapSP< std::pair< DistType, NbHopsType > >
- using EdgeLengthMap = typename NetDistCalculator< Network, DistType, NbHopsType >::EdgeLengthMap
- using EdgeLengthMapSP = typename NetDistCalculator< Network, DistType, NbHopsType >↩ ::EdgeLengthMapSP

Public Member Functions

- ESPLDistCalculator (Dijkstra< Network, DistType, NbHopsType > &dijkstra, EdgeLengthMapSP length, std::size_t lim, CacheLevel cacheLevel=CacheLevel::NetworkCache)
- ESPLDistCalculator (ESPLDistCalculator const &that)=default
- ESPLDistCalculator & operator= (ESPLDistCalculator const &that)=default
- ESPLDistCalculator (ESPLDistCalculator &&that)=default
- ESPLDistCalculator & operator= (ESPLDistCalculator &&that)=default
- virtual std::pair< DistType, NbHopsType > getDist (NodeID const &i, NodeID const &j)
- virtual std::pair< DistType, NbHopsType > getIndDist (NodeID const &i, NodeID const &j)
- virtual NodeDistMapSP getDist (NodeID const &i)
- virtual NodeSDistMapSP getSDistMap (NodeID const &i)
- virtual NodeSDistMapSP getFinDistMapNoNeighb (NodeID const &srcNode)
- virtual std::pair< DistType, NbHopsType > getDist (Edge const &e)
- std::size_t getMaxNbNodesInCache () const
- void setMaxNbNodesInCache (std::size_t maxNbNodesInCache)
- std::size_t getLim () const
- virtual ∼ ESPLDistCalculator ()

9.39.1 Detailed Description

template<typename Network = UNetwork<>, typename DistType = double, typename NbHopsType = std::size_t>
class LinkPred::ESPLDistCalculator< Network, DistType, NbHopsType >

Exact shortest path distance calculator with limits on the number of hops.

This class offers an additional layer over Dijkstra which provides memory management functionalities by caching computed distances.

Template Parameters

Network	The network type.
DistType	The distance type (can be an integer or floating point type).
NbHopsType	The type of the number of hops (must be an integer type).

9.39.2 Member Typedef Documentation

9.39.2.1 Edge

```
template<typename Network = UNetwork<>, typename DistType = double, typename NbHopsType =
std::size_t>
using LinkPred::ESPLDistCalculator< Network, DistType, NbHopsType >::Edge = typename NetDistCalculator<Networ
DistType, NbHopsType>::Edge
```

Edge type.

9.39.2.2 EdgeLengthMap

```
template<typename Network = UNetwork<>, typename DistType = double, typename NbHopsType =
std::size_t>
using LinkPred::ESPLDistCalculator< Network, DistType, NbHopsType >::EdgeLengthMap = typename
NetDistCalculator<Network, DistType, NbHopsType>::EdgeLengthMap
```

Edge length map.

9.39.2.3 EdgeLengthMapSP

```
template<typename Network = UNetwork<>, typename DistType = double, typename NbHopsType =
std::size_t>
using LinkPred::ESPLDistCalculator< Network, DistType, NbHopsType >::EdgeLengthMapSP = typename
NetDistCalculator<Network, DistType, NbHopsType>::EdgeLengthMapSP
```

Shared pointer to an edge length map.

9.39.2.4 Label

```
template<typename Network = UNetwork<>, typename DistType = double, typename NbHopsType =
std::size_t>
using LinkPred::ESPLDistCalculator< Network, DistType, NbHopsType >::Label = typename NetDistCalculator<Netwo
DistType, NbHopsType>::Label
```

Nodes label type.

9.39.2.5 LengthMapIdType

```
template<typename Network = UNetwork<>, typename DistType = double, typename NbHopsType =
std::size_t>
using LinkPred::ESPLDistCalculator< Network, DistType, NbHopsType >::LengthMapIdType = typename
NetDistCalculator<Network, DistType, NbHopsType>::LengthMapIdType
```

Length map ID type.

9.39.2.6 NetworkSP

```
template<typename Network = UNetwork<>, typename DistType = double, typename NbHopsType =
std::size_t>
using LinkPred::ESPLDistCalculator< Network, DistType, NbHopsType >::NetworkSP = typename
NetDistCalculator<Network, DistType, NbHopsType>::NetworkSP
```

Shared pointer to network.

9.39.2.7 NodeDistMap

```
template<typename Network = UNetwork<>, typename DistType = double, typename NbHopsType =
std::size_t>
using LinkPred::ESPLDistCalculator< Network, DistType, NbHopsType >::NodeDistMap = typename
NetDistCalculator<Network, DistType, NbHopsType>::NodeDistMap
```

Distance map.

9.39.2.8 NodeDistMapSP

```
template<typename Network = UNetwork<>, typename DistType = double, typename NbHopsType =
std::size_t>
using LinkPred::ESPLDistCalculator< Network, DistType, NbHopsType >::NodeDistMapSP = typename
NetDistCalculator<Network, DistType, NbHopsType>::NodeDistMapSP
```

Shared pointer to a distance map.

9.39.2.9 NodeID

```
template<typename Network = UNetwork<>, typename DistType = double, typename NbHopsType =
std::size_t>
using LinkPred::ESPLDistCalculator< Network, DistType, NbHopsType >::NodeID = typename NetDistCalculator<Netw[illegible]
DistType, NbHopsType>::NodeID
```

Nodes ID type.

9.39.2.10 NodeSDistMap

```
template<typename Network = UNetwork<>, typename DistType = double, typename NbHopsType =
std::size_t>
using LinkPred::ESPLDistCalculator< Network, DistType, NbHopsType >::NodeSDistMap = typename
Network::template NodeSMap<std::pair<DistType, NbHopsType> >
```

Distance map.

9.39.2.11 NodeSDistMapSP

```
template<typename Network = UNetwork<>, typename DistType = double, typename NbHopsType =
std::size_t>
using LinkPred::ESPLDistCalculator< Network, DistType, NbHopsType >::NodeSDistMapSP = typename
Network::template NodeSMapSP<std::pair<DistType, NbHopsType> >
```

Shared pointer to a distance map.

9.39.3 Constructor & Destructor Documentation

9.39.3.1 ESPLDistCalculator() [1/3]

```
template<typename Network = UNetwork<>, typename DistType = double, typename NbHopsType =
std::size_t>
LinkPred::ESPLDistCalculator< Network, DistType, NbHopsType >::ESPLDistCalculator (
            Dijkstra< Network, DistType, NbHopsType > & dijkstra,
            EdgeLengthMapSP length,
            std::size_t lim,
            CacheLevel cacheLevel = CacheLevel::NetworkCache )  [inline]
```

Constructor.

Parameters

dijkstra	A Dijkstra algorithm object.
length	The length map.
lim	Horizon limit.
cacheLevel	The cache level.

9.39.3.2 ESPLDistCalculator() [2/3]

```
template<typename Network = UNetwork<>, typename DistType = double, typename NbHopsType =
std::size_t>
LinkPred::ESPLDistCalculator< Network, DistType, NbHopsType >::ESPLDistCalculator (
            ESPLDistCalculator< Network, DistType, NbHopsType > const & that )  [default]
```

Copy constructor.

Parameters

that	The object to copy.

9.39.3.3 ESPLDistCalculator() [3/3]

```
template<typename Network = UNetwork<>, typename DistType = double, typename NbHopsType =
std::size_t>
LinkPred::ESPLDistCalculator< Network, DistType, NbHopsType >::ESPLDistCalculator (
            ESPLDistCalculator< Network, DistType, NbHopsType > && that )  [default]
```

Move constructor.

Parameters

that	The object to move.

9.39.3.4 ∼ ESPLDistCalculator()

```
template<typename Network = UNetwork<>, typename DistType = double, typename NbHopsType =
std::size_t>
virtual LinkPred::ESPLDistCalculator< Network, DistType, NbHopsType >::∼ ESPLDistCalculator (
) [inline], [virtual]
```

Destructor.

9.39.4 Member Function Documentation

9.39.4.1 getDist() [1/3]

```
template<typename Network = UNetwork<>, typename DistType = double, typename NbHopsType =
std::size_t>
virtual std::pair<DistType, NbHopsType> LinkPred::ESPLDistCalculator< Network, DistType, Nb↩
HopsType >::getDist (
            Edge const & e ) [inline], [virtual]
```

Parameters

e	An input edge.

Returns

The distance between start and end of e.

9.39.4.2 getDist() [2/3]

```
template<typename Network = UNetwork<>, typename DistType = double, typename NbHopsType =
std::size_t>
virtual NodeDistMapSP LinkPred::ESPLDistCalculator< Network, DistType, NbHopsType >::getDist (
            NodeID const & i ) [virtual]
```

Returns

The distance from srcNode.

Parameters

i	The source node.

9.39.4.3 getDist() [3/3]

```
template<typename Network = UNetwork<>, typename DistType = double, typename NbHopsType =
std::size_t>
virtual std::pair<DistType, NbHopsType> LinkPred::ESPLDistCalculator< Network, DistType, Nb↩
HopsType >::getDist (
            NodeID const & i,
            NodeID const & j )  [virtual]
```

Parameters

i	Index of the source node.
j	Index of the end node.

Returns

The distance between i and j.

9.39.4.4 getFinDistMapNoNeighb()

```
template<typename Network = UNetwork<>, typename DistType = double, typename NbHopsType =
std::size_t>
virtual NodeSDistMapSP LinkPred::ESPLDistCalculator< Network, DistType, NbHopsType >::getFin↩
DistMapNoNeighb (
            NodeID const & srcNode )  [virtual]
```

Returns

A sparse distance map of nodes having finite distance to a given source node. Only nodes not connected to srcNode are considered. The node srcNode itself is also excluded.

Parameters

srcNode	The source node.

9.39.4.5 getIndDist()

```
template<typename Network = UNetwork<>, typename DistType = double, typename NbHopsType =
std::size_t>
virtual std::pair<DistType, NbHopsType> LinkPred::ESPLDistCalculator< Network, DistType, Nb↩
HopsType >::getIndDist (
            NodeID const & i,
            NodeID const & j )  [inline], [virtual]
```

Parameters

i	Index of the source node.
j	Index of the end node.

Returns

The distance between i and j ignoring the edge between i and j.

9.39.4.6 getLim()

```
template<typename Network = UNetwork<>, typename DistType = double, typename NbHopsType =
std::size_t>
std::size_t LinkPred::ESPLDistCalculator< Network, DistType, NbHopsType >::getLim ( ) const
[inline]
```

Returns

The limit on the number of hops.

9.39.4.7 getMaxNbNodesInCache()

```
template<typename Network = UNetwork<>, typename DistType = double, typename NbHopsType =
std::size_t>
std::size_t LinkPred::ESPLDistCalculator< Network, DistType, NbHopsType >::getMaxNbNodesIn←
Cache ( ) const [inline]
```

Returns

The maximum number of nodes allowed in cache.

9.39.4.8 getSDistMap()

```
template<typename Network = UNetwork<>, typename DistType = double, typename NbHopsType =
std::size_t>
virtual NodeSDistMapSP LinkPred::ESPLDistCalculator< Network, DistType, NbHopsType >::getS←
DistMap (
            NodeID const & i ) [virtual]
```

Returns

The sparse distance from srcNode.

Parameters

i	The source node.

9.39.4.9 operator=() [1/2]

```
template<typename Network = UNetwork<>, typename DistType = double, typename NbHopsType =
std::size_t>
ESPLDistCalculator& LinkPred::ESPLDistCalculator< Network, DistType, NbHopsType >::operator= (
            ESPLDistCalculator< Network, DistType, NbHopsType > && that )  [default]
```

Move assignment operator.

Parameters

that	The object to move.

9.39.4.10 operator=() [2/2]

```
template<typename Network = UNetwork<>, typename DistType = double, typename NbHopsType =
std::size_t>
ESPLDistCalculator& LinkPred::ESPLDistCalculator< Network, DistType, NbHopsType >::operator= (
            ESPLDistCalculator< Network, DistType, NbHopsType > const & that )  [default]
```

Copy assignment operator.

Parameters

that	The object to copy.

9.39.4.11 setMaxNbNodesInCache()

```
template<typename Network = UNetwork<>, typename DistType = double, typename NbHopsType =
std::size_t>
void LinkPred::ESPLDistCalculator< Network, DistType, NbHopsType >::setMaxNbNodesInCache (
            std::size_t maxNbNodesInCache )  [inline]
```

Set the maximum number of nodes in cache (for each node a map of distance to all other nodes is kept in memory).

Parameters

maxNbNodesInCache	New value for the maximum number of nodes in cache.

The documentation for this class was generated from the following file:

- include/linkpred/graphalg/shortestpaths/netdistcalculator.hpp

9.40 LinkPred::ESPLSimlCalculator< Network, DistType, NbHopsType > Class Template Reference

Exact shortest path distance calculator.

`#include <netdistcalculator.hpp>`

Inheritance diagram for LinkPred::ESPLSimlCalculator< Network, DistType, NbHopsType >:

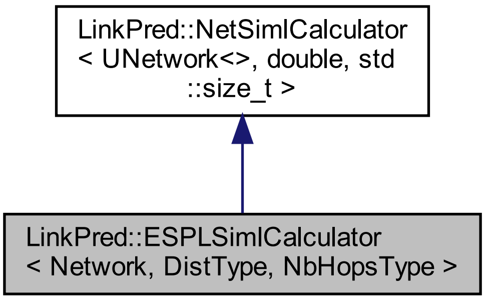

Collaboration diagram for LinkPred::ESPLSimlCalculator< Network, DistType, NbHopsType >:

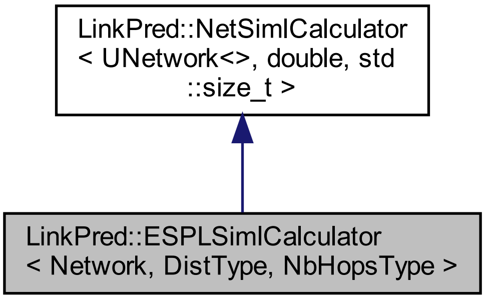

Public Types

- using LengthMapIdType = typename NetSimlCalculator< Network, DistType, NbHopsType >↩ ::LengthMapIdType
- using NetworkSP = typename NetSimlCalculator< Network, DistType, NbHopsType >::NetworkSP
- using NodeID = typename NetSimlCalculator< Network, DistType, NbHopsType >::NodeID
- using Label = typename NetSimlCalculator< Network, DistType, NbHopsType >::Label
- using Edge = typename NetSimlCalculator< Network, DistType, NbHopsType >::Edge
- using NodeSDistMap = typename Network::template NodeSMap< std::pair< DistType, NbHopsType > >
- using NodeSDistMapSP = typename Network::template NodeSMapSP< std::pair< DistType, NbHopsType > >
- using EdgeLengthMap = typename NetSimlCalculator< Network, DistType, NbHopsType >::EdgeLengthMap
- using EdgeLengthMapSP = typename NetSimlCalculator< Network, DistType, NbHopsType >↩ ::EdgeLengthMapSP

Public Member Functions

- ESPLSimlCalculator (Dijkstra< Network, DistType, NbHopsType > &dijkstra, EdgeLengthMapSP length, std::size_t lim, CacheLevel cacheLevel=CacheLevel::NetworkCache)
- ESPLSimlCalculator (ESPLSimlCalculator const &that)=default
- ESPLSimlCalculator & operator= (ESPLSimlCalculator const &that)=default
- ESPLSimlCalculator (ESPLSimlCalculator &&that)=default
- ESPLSimlCalculator & operator= (ESPLSimlCalculator &&that)=default
- virtual std::tuple< DistType, DistType, NbHopsType > getSiml (NodeID const &i, NodeID const &j)
- virtual std::tuple< DistType, DistType, NbHopsType > getDirSiml (NodeID const &i, NodeID const &j)
- virtual NodeSDistMapSP getNnzSimlMap (NodeID const &srcNode)
- virtual NodeSDistMapSP getNnzSimlMapNoNeighb (NodeID const &srcNode)
- virtual std::tuple< DistType, DistType, NbHopsType > getSiml (Edge const &e)
- virtual std::tuple< DistType, DistType, NbHopsType > getDirSiml (Edge const &e)
- std::size_t getMaxNbNodesInCache () const
- void setMaxNbNodesInCache (std::size_t maxNbNodesInCache)
- std::size_t getLim () const
- bool isUseHops () const
- void setUseHops (bool useHops)
- virtual ∼ ESPLSimlCalculator ()

9.40.1 Detailed Description

template<typename Network = UNetwork<>, typename DistType = double, typename NbHopsType = std::size_t>
class LinkPred::ESPLSimlCalculator< Network, DistType, NbHopsType >

Exact shortest path distance calculator.

This class offers an additional layer over Dijkstra which provides memory management functionalities by caching computed distances.

Template Parameters

Network	The network type.
DistType	The distance type (can be an integer or floating point type).
NbHopsType	The type of the number of hops (must be an integer type).

9.40.2 Member Typedef Documentation

9.40.2.1 Edge

```
template<typename Network = UNetwork<>, typename DistType = double, typename NbHopsType =
std::size_t>
using LinkPred::ESPLSimlCalculator< Network, DistType, NbHopsType >::Edge = typename NetSimlCalculator<Networ[illegible]
DistType, NbHopsType>::Edge
```

Edge type.

9.40.2.2 EdgeLengthMap

```
template<typename Network = UNetwork<>, typename DistType = double, typename NbHopsType =
std::size_t>
using LinkPred::ESPLSimlCalculator< Network, DistType, NbHopsType >::EdgeLengthMap = typename
NetSimlCalculator<Network, DistType, NbHopsType>::EdgeLengthMap
```

Edge length map.

9.40.2.3 EdgeLengthMapSP

```
template<typename Network = UNetwork<>, typename DistType = double, typename NbHopsType =
std::size_t>
using LinkPred::ESPLSimlCalculator< Network, DistType, NbHopsType >::EdgeLengthMapSP = typename
NetSimlCalculator<Network, DistType, NbHopsType>::EdgeLengthMapSP
```

Shared pointer to an edge length map.

9.40.2.4 Label

```
template<typename Network = UNetwork<>, typename DistType = double, typename NbHopsType =
std::size_t>
using LinkPred::ESPLSimlCalculator< Network, DistType, NbHopsType >::Label = typename NetSimlCalculator<Netwo[illegible]
DistType, NbHopsType>::Label
```

Nodes label type.

9.40.2.5 LengthMapIdType

```
template<typename Network = UNetwork<>, typename DistType = double, typename NbHopsType =
std::size_t>
using LinkPred::ESPLSimlCalculator< Network, DistType, NbHopsType >::LengthMapIdType = typename
NetSimlCalculator<Network, DistType, NbHopsType>::LengthMapIdType
```

Length map ID type.

9.40.2.6 NetworkSP

```
template<typename Network = UNetwork<>, typename DistType = double, typename NbHopsType =
std::size_t>
using LinkPred::ESPLSimlCalculator< Network, DistType, NbHopsType >::NetworkSP = typename
NetSimlCalculator<Network, DistType, NbHopsType>::NetworkSP
```

Shared pointer to network.

9.40.2.7 NodeID

```
template<typename Network = UNetwork<>, typename DistType = double, typename NbHopsType =
std::size_t>
using LinkPred::ESPLSimlCalculator< Network, DistType, NbHopsType >::NodeID = typename NetSimlCalculator<Netw
DistType, NbHopsType>::NodeID
```

Nodes ID type.

9.40.2.8 NodeSDistMap

```
template<typename Network = UNetwork<>, typename DistType = double, typename NbHopsType =
std::size_t>
using LinkPred::ESPLSimlCalculator< Network, DistType, NbHopsType >::NodeSDistMap = typename
Network::template NodeSMap<std::pair<DistType, NbHopsType> >
```

Distance map.

9.40.2.9 NodeSDistMapSP

```
template<typename Network = UNetwork<>, typename DistType = double, typename NbHopsType =
std::size_t>
using LinkPred::ESPLSimlCalculator< Network, DistType, NbHopsType >::NodeSDistMapSP = typename
Network::template NodeSMapSP<std::pair<DistType, NbHopsType> >
```

Shared pointer to a distance map.

9.40.3 Constructor & Destructor Documentation

9.40.3.1 ESPLSimlCalculator() [1/3]

```
template<typename Network = UNetwork<>, typename DistType = double, typename NbHopsType =
std::size_t>
LinkPred::ESPLSimlCalculator< Network, DistType, NbHopsType >::ESPLSimlCalculator (
            Dijkstra< Network, DistType, NbHopsType > & dijkstra,
            EdgeLengthMapSP length,
            std::size_t lim,
            CacheLevel cacheLevel = CacheLevel::NetworkCache )  [inline]
```

Constructor.

Parameters

dijkstra	A Dijkstra algorithm object.
length	The length map.
lim	Horizon limit.
cacheLevel	The cache level.

9.40.3.2 ESPLSimlCalculator() [2/3]

```
template<typename Network = UNetwork<>, typename DistType = double, typename NbHopsType =
std::size_t>
LinkPred::ESPLSimlCalculator< Network, DistType, NbHopsType >::ESPLSimlCalculator (
            ESPLSimlCalculator< Network, DistType, NbHopsType > const & that )  [default]
```

Copy constructor.

Parameters

that	The object to copy.

9.40.3.3 ESPLSimlCalculator() [3/3]

```
template<typename Network = UNetwork<>, typename DistType = double, typename NbHopsType =
std::size_t>
LinkPred::ESPLSimlCalculator< Network, DistType, NbHopsType >::ESPLSimlCalculator (
            ESPLSimlCalculator< Network, DistType, NbHopsType > && that )  [default]
```

Move constructor.

Parameters

that	The object to move.

9.40.3.4 ∼ ESPLSimlCalculator()

```
template<typename Network = UNetwork<>, typename DistType = double, typename NbHopsType =
std::size_t>
virtual LinkPred::ESPLSimlCalculator< Network, DistType, NbHopsType >::∼ ESPLSimlCalculator (
)  [inline], [virtual]
```

Destructor.

9.40.4 Member Function Documentation

9.40.4.1 getDirSiml() [1/2]

```
template<typename Network = UNetwork<>, typename DistType = double, typename NbHopsType =
std::size_t>
virtual std::tuple<DistType, DistType, NbHopsType> LinkPred::ESPLSimlCalculator< Network,
DistType, NbHopsType >::getDirSiml (
            Edge const & e ) [inline], [virtual]
```

Parameters

e	An input edge.

Returns

The directed similarity between start and end of e.

9.40.4.2 getDirSiml() [2/2]

```
template<typename Network = UNetwork<>, typename DistType = double, typename NbHopsType =
std::size_t>
virtual std::tuple<DistType, DistType, NbHopsType> LinkPred::ESPLSimlCalculator< Network,
DistType, NbHopsType >::getDirSiml (
            NodeID const & i,
            NodeID const & j ) [virtual]
```

Parameters

i	Index of the source node.
j	Index of the end node.

Returns

The directed similarity between i and j.

9.40.4.3 getLim()

```
template<typename Network = UNetwork<>, typename DistType = double, typename NbHopsType =
std::size_t>
std::size_t LinkPred::ESPLSimlCalculator< Network, DistType, NbHopsType >::getLim ( ) const
[inline]
```

Returns

The limit on the number of hops.

9.40.4.4 getMaxNbNodesInCache()

```
template<typename Network = UNetwork<>, typename DistType = double, typename NbHopsType =
std::size_t>
std::size_t LinkPred::ESPLSimlCalculator< Network, DistType, NbHopsType >::getMaxNbNodesIn←
Cache ( ) const  [inline]
```

Returns

The maximum number of nodes allowed in cache.

9.40.4.5 getNnzSimlMap()

```
template<typename Network = UNetwork<>, typename DistType = double, typename NbHopsType =
std::size_t>
virtual NodeSDistMapSP LinkPred::ESPLSimlCalculator< Network, DistType, NbHopsType >::getNnz←
SimlMap (
            NodeID const & srcNode )  [virtual]
```

Returns

A sparse similarity map of nodes having non-zero similarity to a given source node.

Parameters

srcNode	The source node.

9.40.4.6 getNnzSimlMapNoNeighb()

```
template<typename Network = UNetwork<>, typename DistType = double, typename NbHopsType =
std::size_t>
virtual NodeSDistMapSP LinkPred::ESPLSimlCalculator< Network, DistType, NbHopsType >::getNnz←
SimlMapNoNeighb (
            NodeID const & srcNode )  [virtual]
```

Returns

A sparse similarity map of nodes having non-zero similarity to a given source node. Only nodes not connected to srcNode are considered. The node srcNode itself is also excluded.

Parameters

srcNode	The source node.

9.40.4.7 getSiml() [1/2]

```
template<typename Network = UNetwork<>, typename DistType = double, typename NbHopsType =
std::size_t>
virtual std::tuple<DistType, DistType, NbHopsType> LinkPred::ESPLSimlCalculator< Network,
DistType, NbHopsType >::getSiml (
             Edge const & e )  [inline], [virtual]
```

Parameters

e	An input edge.

Returns

The similarity between start and end of e.

9.40.4.8 getSiml() [2/2]

```
template<typename Network = UNetwork<>, typename DistType = double, typename NbHopsType =
std::size_t>
virtual std::tuple<DistType, DistType, NbHopsType> LinkPred::ESPLSimlCalculator< Network,
DistType, NbHopsType >::getSiml (
             NodeID const & i,
             NodeID const & j )  [virtual]
```

Parameters

i	Index of the source node.
j	Index of the end node.

Returns

The similarity between i and j.

9.40.4.9 isUseHops()

```
template<typename Network = UNetwork<>, typename DistType = double, typename NbHopsType =
std::size_t>
bool LinkPred::ESPLSimlCalculator< Network, DistType, NbHopsType >::isUseHops ( ) const  [inline]
```

Returns

True if the number of hops is used to compute similarity.

9.40.4.10 operator=() [1/2]

```
template<typename Network = UNetwork<>, typename DistType = double, typename NbHopsType =
std::size_t>
ESPLSimlCalculator& LinkPred::ESPLSimlCalculator< Network, DistType, NbHopsType >::operator= (
            ESPLSimlCalculator< Network, DistType, NbHopsType > && that )  [default]
```

Move assignment operator.

Parameters

that	The object to move.

9.40.4.11 operator=() [2/2]

```
template<typename Network = UNetwork<>, typename DistType = double, typename NbHopsType =
std::size_t>
ESPLSimlCalculator& LinkPred::ESPLSimlCalculator< Network, DistType, NbHopsType >::operator= (
            ESPLSimlCalculator< Network, DistType, NbHopsType > const & that )  [default]
```

Copy assignment operator.

Parameters

that	The object to copy.

9.40.4.12 setMaxNbNodesInCache()

```
template<typename Network = UNetwork<>, typename DistType = double, typename NbHopsType =
std::size_t>
void LinkPred::ESPLSimlCalculator< Network, DistType, NbHopsType >::setMaxNbNodesInCache (
            std::size_t maxNbNodesInCache )  [inline]
```

Set the maximum number of nodes in cache (for each node a map of similarity to all other nodes is kept in memory).

Parameters

maxNbNodesInCache	New value for the maximum number of nodes in cache.

9.40.4.13 setUseHops()

```
template<typename Network = UNetwork<>, typename DistType = double, typename NbHopsType =
std::size_t>
void LinkPred::ESPLSimlCalculator< Network, DistType, NbHopsType >::setUseHops (
            bool useHops ) [inline]
```

Parameters

useHops	Set whether the number of hops is used to compute similarity.

The documentation for this class was generated from the following file:

- include/linkpred/graphalg/shortestpaths/netdistcalculator.hpp

9.41 LinkPred::ESPSimlCalculator< Network, DistType, NbHopsType > Class Template Reference

Exact shortest path distance calculator.

```
#include <netdistcalculator.hpp>
```

Inheritance diagram for LinkPred::ESPSimlCalculator< Network, DistType, NbHopsType >:

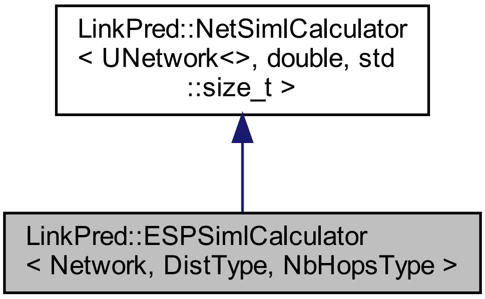

Collaboration diagram for LinkPred::ESPSimlCalculator< Network, DistType, NbHopsType >:

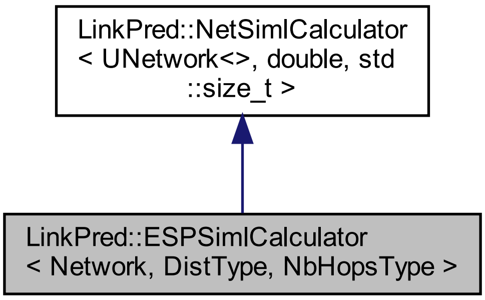

Public Types

- using LengthMapIdType = typename NetSimlCalculator< Network, DistType, NbHopsType >::LengthMapIdType
- using NetworkSP = typename NetSimlCalculator< Network, DistType, NbHopsType >::NetworkSP
- using NodeID = typename NetSimlCalculator< Network, DistType, NbHopsType >::NodeID
- using Label = typename NetSimlCalculator< Network, DistType, NbHopsType >::Label
- using Edge = typename NetSimlCalculator< Network, DistType, NbHopsType >::Edge
- using NodeDistMap = typename Network::template NodeMap< std::pair< DistType, NbHopsType > >
- using NodeDistMapSP = typename Network::template NodeMapSP< std::pair< DistType, NbHopsType > >
- using EdgeLengthMap = typename NetSimlCalculator< Network, DistType, NbHopsType >::EdgeLengthMap
- using EdgeLengthMapSP = typename NetSimlCalculator< Network, DistType, NbHopsType >::EdgeLengthMapSP

Public Member Functions

- ESPSimlCalculator (Dijkstra< Network, DistType, NbHopsType > &dijkstra, EdgeLengthMapSP length, CacheLevel cacheLevel=CacheLevel::NetworkCache)
- ESPSimlCalculator (ESPSimlCalculator const &that)=default
- ESPSimlCalculator & operator= (ESPSimlCalculator const &that)=default
- ESPSimlCalculator (ESPSimlCalculator &&that)=default
- ESPSimlCalculator & operator= (ESPSimlCalculator &&that)=default
- virtual std::tuple< DistType, DistType, NbHopsType > getSiml (NodeID const &i, NodeID const &j)
- virtual std::tuple< DistType, DistType, NbHopsType > getDirSiml (NodeID const &i, NodeID const &j)
- virtual std::tuple< DistType, DistType, NbHopsType > getSiml (Edge const &e)
- virtual std::tuple< DistType, DistType, NbHopsType > getDirSiml (Edge const &e)
- std::size_t getMaxNbNodesInCache () const
- void setMaxNbNodesInCache (std::size_t maxNbNodesInCache)
- virtual ∼ESPSimlCalculator ()

9.41.1 Detailed Description

**template<typename Network = UNetwork<>, typename DistType = double, typename NbHopsType = std::size_t>
class LinkPred::ESPSimlCalculator< Network, DistType, NbHopsType >**

Exact shortest path distance calculator.

This class offers an additional layer over Dijkstra which provides memory management functionalities by caching computed distances.

Template Parameters

Network	The network type.
DistType	The distance type (can be an integer or floating point type).
NbHopsType	The type of the number of hops (must be an integer type).

9.41.2 Member Typedef Documentation

9.41.2.1 Edge

```
template<typename Network = UNetwork<>, typename DistType = double, typename NbHopsType =
std::size_t>
using LinkPred::ESPSimlCalculator< Network, DistType, NbHopsType >::Edge = typename NetSimlCalculator<Network
DistType, NbHopsType>::Edge
```

Edge type.

9.41.2.2 EdgeLengthMap

```
template<typename Network = UNetwork<>, typename DistType = double, typename NbHopsType =
std::size_t>
using LinkPred::ESPSimlCalculator< Network, DistType, NbHopsType >::EdgeLengthMap = typename
NetSimlCalculator<Network, DistType, NbHopsType>::EdgeLengthMap
```

Edge length map.

9.41.2.3 EdgeLengthMapSP

```
template<typename Network = UNetwork<>, typename DistType = double, typename NbHopsType =
std::size_t>
using LinkPred::ESPSimlCalculator< Network, DistType, NbHopsType >::EdgeLengthMapSP = typename
NetSimlCalculator<Network, DistType, NbHopsType>::EdgeLengthMapSP
```

Shared pointer to an edge length map.

9.41.2.4 Label

```
template<typename Network = UNetwork<>, typename DistType = double, typename NbHopsType =
std::size_t>
using LinkPred::ESPSimlCalculator< Network, DistType, NbHopsType >::Label = typename NetSimlCalculator<Networ
DistType, NbHopsType>::Label
```

Nodes label type.

9.41.2.5 LengthMapIdType

```
template<typename Network = UNetwork<>, typename DistType = double, typename NbHopsType =
std::size_t>
using LinkPred::ESPSimlCalculator< Network, DistType, NbHopsType >::LengthMapIdType = typename
NetSimlCalculator<Network, DistType, NbHopsType>::LengthMapIdType
```

Length map ID type.

9.41.2.6 NetworkSP

```
template<typename Network = UNetwork<>, typename DistType = double, typename NbHopsType =
std::size_t>
using LinkPred::ESPSimlCalculator< Network, DistType, NbHopsType >::NetworkSP = typename
NetSimlCalculator<Network, DistType, NbHopsType>::NetworkSP
```

Shared pointer to network.

9.41.2.7 NodeDistMap

```
template<typename Network = UNetwork<>, typename DistType = double, typename NbHopsType =
std::size_t>
using LinkPred::ESPSimlCalculator< Network, DistType, NbHopsType >::NodeDistMap = typename
Network::template NodeMap<std::pair<DistType, NbHopsType> >
```

Distance map.

9.41.2.8 NodeDistMapSP

```
template<typename Network = UNetwork<>, typename DistType = double, typename NbHopsType =
std::size_t>
using LinkPred::ESPSimlCalculator< Network, DistType, NbHopsType >::NodeDistMapSP = typename
Network::template NodeMapSP<std::pair<DistType, NbHopsType> >
```

Shared pointer to a distance map.

9.41.2.9 NodeID

```
template<typename Network = UNetwork<>, typename DistType = double, typename NbHopsType =
std::size_t>
using LinkPred::ESPSimlCalculator< Network, DistType, NbHopsType >::NodeID = typename NetSimlCalculator<Netwo
DistType, NbHopsType>::NodeID
```

Nodes ID type.

9.41.3 Constructor & Destructor Documentation

9.41.3.1 ESPSimlCalculator() [1/3]

```
template<typename Network = UNetwork<>, typename DistType = double, typename NbHopsType =
std::size_t>
LinkPred::ESPSimlCalculator< Network, DistType, NbHopsType >::ESPSimlCalculator (
            Dijkstra< Network, DistType, NbHopsType > & dijkstra,
            EdgeLengthMapSP length,
            CacheLevel cacheLevel = CacheLevel::NetworkCache )  [inline]
```

Constructor.

Parameters

dijkstra	A Dijkstra algorithm object.
length	The length map.
cacheLevel	The cache level.

9.41.3.2 ESPSimlCalculator() [2/3]

```
template<typename Network = UNetwork<>, typename DistType = double, typename NbHopsType =
std::size_t>
LinkPred::ESPSimlCalculator< Network, DistType, NbHopsType >::ESPSimlCalculator (
            ESPSimlCalculator< Network, DistType, NbHopsType > const & that )  [default]
```

Copy constructor.

Parameters

that	The object to copy.

9.41.3.3 ESPSimlCalculator() [3/3]

```
template<typename Network = UNetwork<>, typename DistType = double, typename NbHopsType =
std::size_t>
LinkPred::ESPSimlCalculator< Network, DistType, NbHopsType >::ESPSimlCalculator (
            ESPSimlCalculator< Network, DistType, NbHopsType > && that )  [default]
```

Move constructor.

Parameters

that	The object to move.

9.41.3.4 ∼ESPSimlCalculator()

```
template<typename Network = UNetwork<>, typename DistType = double, typename NbHopsType =
std::size_t>
virtual LinkPred::ESPSimlCalculator< Network, DistType, NbHopsType >::∼ESPSimlCalculator ( )
[inline], [virtual]
```

Destructor.

9.41.4 Member Function Documentation

9.41.4.1 getDirSiml() [1/2]

```
template<typename Network = UNetwork<>, typename DistType = double, typename NbHopsType =
std::size_t>
virtual std::tuple<DistType, DistType, NbHopsType> LinkPred::ESPSimlCalculator< Network,
DistType, NbHopsType >::getDirSiml (
            Edge const & e )  [inline], [virtual]
```

Parameters

e	An input edge.

Returns

The directed similarity between start and end of e.

9.41.4.2 getDirSiml() [2/2]

```
template<typename Network = UNetwork<>, typename DistType = double, typename NbHopsType =
std::size_t>
virtual std::tuple<DistType, DistType, NbHopsType> LinkPred::ESPSimlCalculator< Network,
DistType, NbHopsType >::getDirSiml (
            NodeID const & i,
            NodeID const & j )  [virtual]
```

Parameters

i	Index of the source node.
j	Index of the end node.

Returns

The directed similarity between i and j.

9.41.4.3 getMaxNbNodesInCache()

```
template<typename Network = UNetwork<>, typename DistType = double, typename NbHopsType =
std::size_t>
std::size_t LinkPred::ESPSimlCalculator< Network, DistType, NbHopsType >::getMaxNbNodesInCache
( ) const  [inline]
```

Returns

The maximum number of nodes allowed in cache.

9.41.4.4 getSiml() [1/2]

```
template<typename Network = UNetwork<>, typename DistType = double, typename NbHopsType =
std::size_t>
virtual std::tuple<DistType, DistType, NbHopsType> LinkPred::ESPSimlCalculator< Network,
DistType, NbHopsType >::getSiml (
            Edge const & e )  [inline], [virtual]
```

Parameters

e	Edge.

Returns

The similarity between the two ends of the edge.

9.41.4.5 getSiml() [2/2]

```
template<typename Network = UNetwork<>, typename DistType = double, typename NbHopsType =
std::size_t>
virtual std::tuple<DistType, DistType, NbHopsType> LinkPred::ESPSimlCalculator< Network,
DistType, NbHopsType >::getSiml (
            NodeID const & i,
            NodeID const & j )  [virtual]
```

Parameters

i	Index of the source node.
j	Index of the end node.

Returns

The similarity between i and j.

9.41.4.6 operator=() [1/2]

```
template<typename Network = UNetwork<>, typename DistType = double, typename NbHopsType =
std::size_t>
ESPSimlCalculator& LinkPred::ESPSimlCalculator< Network, DistType, NbHopsType >::operator= (
            ESPSimlCalculator< Network, DistType, NbHopsType > && that )  [default]
```

Move assignment operator.

Parameters

that	The object to move.

9.41.4.7 operator=() [2/2]

```
template<typename Network = UNetwork<>, typename DistType = double, typename NbHopsType =
std::size_t>
ESPSimlCalculator& LinkPred::ESPSimlCalculator< Network, DistType, NbHopsType >::operator= (
            ESPSimlCalculator< Network, DistType, NbHopsType > const & that )  [default]
```

Copy assignment operator.

Parameters

that	The object to copy.

9.41.4.8 setMaxNbNodesInCache()

```
template<typename Network = UNetwork<>, typename DistType = double, typename NbHopsType =
std::size_t>
void LinkPred::ESPSimlCalculator< Network, DistType, NbHopsType >::setMaxNbNodesInCache (
            std::size_t maxNbNodesInCache )  [inline]
```

Set the maximum number of nodes in cache (for each node a map of similarity to all other nodes is kept in memory).

Parameters

maxNbNodesInCache	New value for the maximum number of nodes in cache.

The documentation for this class was generated from the following file:

- include/linkpred/graphalg/shortestpaths/netdistcalculator.hpp

9.42 LinkPred::Simp::Evaluator Class Reference

A class that simplifies the evaluation of link prediction algorithms.

`#include <evaluator.hpp>`

Public Member Functions

- Evaluator ()
- Evaluator (Evaluator const &that)=default
- virtual ∼Evaluator ()=default
- void addADA (std::string const &name="ADA")
- void addCNE (std::string const &name="CNE")
- void addCRA (std::string const &name="CRA")
- void addECL (std::string const &name="ECL-N2V-LGR", std::string encoderName="N2V", std::string classifierName="LGR", int dim=0, double posRatio=1.0, double negRatio=1.0, long int seed=0)
- void addESM (std::string const &name="ESM-N2V-L2", std::string encoderName="N2V", std::string sim←MeasureName="L2", int dim=0, long int seed=0)
- void addFBM (std::string const &name="FBM", int maxIter=50, long int seed=0)
- void addHDI (std::string const &name="HDI")
- void addHPI (std::string const &name="HPI")
- void addHRG (std::string const &name="HRG", int nbBeans=25, int nbSamples=10000, long int seed=0)
- void addHYP (std::string const &name="HYP", double m=1.5, double L=1, double gamma=2.1, double zeta=1, double T=0.8, long int seed=0)
- void addJID (std::string const &name="JID")
- void addKAB (std::string const &name="KAB", int horizLim=2)
- void addLCP (std::string const &name="LCP", double epsilon=0.001)
- void addLHN (std::string const &name="LHN")
- void addPAT (std::string const &name="PAT")
- void addPST (std::string const &name="PST", std::string fileName="pst.csv")
- void addRAL (std::string const &name="RAL")
- void addRND (std::string const &name="RND", long int seed=0)
- void addSAI (std::string const &name="SAI")
- void addSBM (std::string const &name="SBM", int maxIter=1000, long int seed=0)
- void addSHP (std::string const &name="SHP", long int seed=0)
- void addSOI (std::string const &name="SOI")
- void addROC ()
- void addPR ()
- void addTPR ()
- void genTestData (std::string const &fullNetFileName, std::string const &obsEdgesFileName, std::string const &remEdgesFileName, double remRatio=0.1, bool keepConnected=false, long int seed=0)
- void run (std::string const &fullNetFileName, int nbRuns=10, double remRatio=0.1, bool keep←Connected=false, long int seed=0)
- void run (std::string const &obsEdgesFileName, std::string const &remEdgesFileName)
- std::vector< PerfRes > getPerfRes (int iter)

9.42.1 Detailed Description

A class that simplifies the evaluation of link prediction algorithms.

9.42.2 Constructor & Destructor Documentation

9.42.2.1 Evaluator() [1/2]

```
LinkPred::Simp::Evaluator::Evaluator ( )
```

Parameters

net	The network.

9.42.2.2 Evaluator() [2/2]

```
LinkPred::Simp::Evaluator::Evaluator (
            Evaluator const & that )  [default]
```

Copy constructor.

Parameters

that	The object to copy.

9.42.2.3 ∼Evaluator()

```
virtual LinkPred::Simp::Evaluator::~Evaluator ( )  [virtual], [default]
```

Destructor.

9.42.3 Member Function Documentation

9.42.3.1 addADA()

```
void LinkPred::Simp::Evaluator::addADA (
            std::string const & name = "ADA" )
```

Add Adamic Adar predictor.

Parameters

name	A unique identifier for the predictor.

9.42.3.2 addCNE()

```
void LinkPred::Simp::Evaluator::addCNE (
            std::string const & name = "CNE" )
```

Add common neighbors.

Parameters

name	A unique identifier for the predictor.

9.42.3.3 addCRA()

```
void LinkPred::Simp::Evaluator::addCRA (
            std::string const & name = "CRA" )
```

Add Cannistraci resource allocation.

Parameters

name	A unique identifier for the predictor.

9.42.3.4 addECL()

```
void LinkPred::Simp::Evaluator::addECL (
            std::string const & name = "ECL-N2V-LGR",
            std::string encoderName = "N2V",
            std::string classifierName = "LGR",
            int dim = 0,
            double posRatio = 1.0,
            double negRatio = 1.0,
            long int seed = 0 )
```

Add an encoder-classifier link predictor.

Parameters

name	A unique identifier for the predictor.

Parameters

encoderName	The name of the encoder. Possible values are: DPW (DeepWalk), HMSM (Hidden Metric Space Model), LVS (LargeVis), LEM (Laplacian Eigenmaps), LIN (LINE), LLE (Locally Linear Embedding), MFC (Matrix Factorization), and N2V (Node2Vec).
classifierName	The name of the classifier. Possible values are: FFN (feed-forward neural network withn default architecture), LSVM (linear SVM), LGR (logistic regression), NVB (naive Bayes). All classifiers except logistic regression requirte compilation with mlpack.
dim	The dimension of the embedding space. If set to zero, the default value is used (the default dimension depends on the ecnoder).
posRatio	Ratio of positive edges used in the training of the classifier.
negRatio	Ratio of negative edges used in the training of the classifier.
seed	Seed of the random number generator.

9.42.3.5 addESM()

```
void LinkPred::Simp::Evaluator::addESM (
            std::string const & name = "ESM-N2V-L2",
            std::string encoderName = "N2V",
            std::string simMeasureName = "L2",
            int dim = 0,
            long int seed = 0 )
```

Add an encoder-similarity measure link predictor.

Parameters

name	A unique identifier for the predictor.
encoderName	The name of the encoder. Possible values are: DPW (DeepWalk), HMSM (Hidden Metric Space Model), LVS (LargeVis), LEM (Laplacian Eigenmaps), LIN (LINE), LLE (Locally Linear Embedding), MFC (Matrix Factorization), and N2V (Node2Vec).
simMeasureName	The name of the similarity measure. Possible values are: CSM (cosine similarity), DTP (dot product), L1 (L1 similarity), L2 (L2 similarity), PRS (Pearson similarity).
dim	The dimension of the embedding space. If set to zero, the default value is used (the default dimension depends on the ecnoder).
seed	Seed of the random number generator.

9.42.3.6 addFBM()

```
void LinkPred::Simp::Evaluator::addFBM (
            std::string const & name = "FBM",
            int maxIter = 50,
            long int seed = 0 )
```

Add fast blocking model.

Parameters

name	A unique identifier for the predictor.
maxIter	Maximum number of iterations.
seed	Seed of the random number generator.

9.42.3.7 addHDI()

```
void LinkPred::Simp::Evaluator::addHDI (
            std::string const & name = "HDI" )
```

Add hub depromoted index.

Parameters

name	A unique identifier for the predictor.

9.42.3.8 addHPI()

```
void LinkPred::Simp::Evaluator::addHPI (
            std::string const & name = "HPI" )
```

Add Hub promoted index.

Parameters

name	A unique identifier for the predictor.

9.42.3.9 addHRG()

```
void LinkPred::Simp::Evaluator::addHRG (
            std::string const & name = "HRG",
            int nbBeans = 25,
            int nbSamples = 10000,
            long int seed = 0 )
```

Add hierarchical random graph.

Parameters

name	A unique identifier for the predictor.
nbBeans	Number of bins in edge statistics histogram.
nbSamples	Number of samples to take for predictions.
seed	Seed of the random number generator.

9.42.3.10 addHYP()

```
void LinkPred::Simp::Evaluator::addHYP (
            std::string const & name = "HYP",
            double m = 1.5,
            double L = 1,
            double gamma = 2.1,
            double zeta = 1,
            double T = 0.8,
            long int seed = 0 )
```

Add Hypermap.

Parameters

name	A unique identifier for the predictor.
m	The parameter m (see the algorithm description).
L	The parameter L (see the algorithm description).
gamma	The power law exponent gamma (see the algorithm description).
zeta	The parameter zeta (see the algorithm description).
T	The parameter T (see the algorithm description).
seed	The random number generator seed.

9.42.3.11 addJID()

```
void LinkPred::Simp::Evaluator::addJID (
            std::string const & name = "JID" )
```

Add Jackard index predictor.

Parameters

name	A unique identifier for the predictor.

9.42.3.12 addKAB()

```
void LinkPred::Simp::Evaluator::addKAB (
            std::string const & name = "KAB",
            int horizLim = 2 )
```

Add the scalable popularity similarity link predictor proposed in: "Kerrache, S., Alharbi, R. & Benhidour, H. A Scalable Similarity-Popularity Link Prediction Method. Sci Rep 10, 6394 (2020)".

Parameters

name	A unique identifier for the predictor.
horizLim	Horizon limit.

9.42.3.13 addLCP()

```
void LinkPred::Simp::Evaluator::addLCP (
            std::string const & name = "LCP",
            double epsilon = 0.001 )
```

Add local path.

Parameters

name	A unique identifier for the predictor.
epsilon	The weight of paths of length 3.

9.42.3.14 addLHN()

```
void LinkPred::Simp::Evaluator::addLHN (
            std::string const & name = "LHN" )
```

Add Leicht-Holme-Newman index.

Parameters

name	A unique identifier for the predictor.

9.42.3.15 addPAT()

```
void LinkPred::Simp::Evaluator::addPAT (
            std::string const & name = "PAT" )
```

Add preferential attachment index.

Parameters

name	A unique identifier for the predictor.

9.42.3.16 addPR()

```
void LinkPred::Simp::Evaluator::addPR ( )
```

Add area under the precision-recall curve.

9.42.3.17 addPST()

```
void LinkPred::Simp::Evaluator::addPST (
            std::string const & name = "PST",
            std::string fileName = "pst.csv" )
```

Add pre-stored results. This is designed to work with run(std::string const &obsEdgesFileName, std::string const &remEdgesFileName, long int seed) to measure the performance of user-defined prediction algorithms.

Parameters

name	A unique identifier for the predictor.
fileName	File containing the scores of all non-exisityng links in the training network.

9.42.3.18 addRAL()

```
void LinkPred::Simp::Evaluator::addRAL (
            std::string const & name = "RAL" )
```

Add resource allocation index.

Parameters

name	A unique identifier for the predictor.

9.42.3.19 addRND()

```
void LinkPred::Simp::Evaluator::addRND (
            std::string const & name = "RND",
            long int seed = 0 )
```

Add random predictor.

Parameters

name	A unique identifier for the predictor.
seed	The random number generator seed.

9.42.3.20 addROC()

```
void LinkPred::Simp::Evaluator::addROC ( )
```

Add area under the ROC curve.

9.42.3.21 addSAI()

```
void LinkPred::Simp::Evaluator::addSAI (
            std::string const & name = "SAI" )
```

Add Salton index.

Parameters

name	A unique identifier for the predictor.

9.42.3.22 addSBM()

```
void LinkPred::Simp::Evaluator::addSBM (
            std::string const & name = "SBM",
            int maxIter = 1000,
            long int seed = 0 )
```

Add stochastic blocking model.

Parameters

name	A unique identifier for the predictor.
maxIter	Maximum number of iterations.
seed	Seed of the random number generator.

9.42.3.23 addSHP()

```
void LinkPred::Simp::Evaluator::addSHP (
            std::string const & name = "SHP",
            long int seed = 0 )
```

Add shortest path predictor.

Parameters

name	A unique identifier for the predictor.
seed	The random number generator seed.

9.42.3.24 addSOI()

```
void LinkPred::Simp::Evaluator::addSOI (
            std::string const & name = "SOI" )
```

Add Sorensen index.

Parameters

name	A unique identifier for the predictor.

9.42.3.25 addTPR()

```
void LinkPred::Simp::Evaluator::addTPR ( )
```

Add top precision.

9.42.3.26 genTestData()

```
void LinkPred::Simp::Evaluator::genTestData (
            std::string const & fullNetFileName,
            std::string const & obsEdgesFileName,
            std::string const & remEdgesFileName,
            double remRatio = 0.1,
            bool keepConnected = false,
            long int seed = 0 )
```

Generate test data and save it to file.

Parameters

fullNetFileName	This the file containing the ground truth network.
obsEdgesFileName	The method writes the remaining edges into this file. This constitutes the training set.
remEdgesFileName	The method writes the removed edges into this file. This constitutes the positive examples of the test set.
remRatio	Edge remove ratio.
keepConnected	Whether to keep the graph connected when removing edges. This may be impossible for high ratios or if the network is initially disconnected.
seed	Seed for the andom number generator

9.42.3.27 getPerfRes()

```
std::vector<PerfRes> LinkPred::Simp::Evaluator::getPerfRes (
            int iter ) [inline]
```

Parameters

iter	Iteration number.

Returns

The performance results at the specified iteration.

9.42.3.28 run() [1/2]

```
void LinkPred::Simp::Evaluator::run (
            std::string const & fullNetFileName,
            int nbRuns = 10,
            double remRatio = 0.1,
            bool keepConnected = false,
            long int seed = 0 )
```

Run a performance evaluation. This method creates test data by randomnly removing edges from the full network.

Parameters

fullNetFileName	The network file name. This is the full network (ground truth) containing all edges.
nbRuns	Number of times the experiment is run. Each time the test set is changed.
remRatio	Edge remove ratio.
keepConnected	Whether to keep the graph connected when removing edges. This may be impossible for high ratios or if the network is initially disconnected.
seed	Seed for the andom number generator

9.42.3.29 run() [2/2]

```
void LinkPred::Simp::Evaluator::run (
            std::string const & obsEdgesFileName,
            std::string const & remEdgesFileName )
```

Run a performance evaluation. This method uses the test data given as input

Parameters

obsEdgesFileName	A file containing observed edges (training set).
remEdgesFileName	A file containing removed edges (the positive examples of the test set).

The documentation for this class was generated from the following file:

- include/linkpred/simp/evaluator.hpp

9.43 LinkPred::FastSig< T > Class Template Reference

A fast sigmoid.

`#include <fastsig.hpp>`

Public Member Functions

- FastSig ()
- FastSig (int n, T lowBound, T uppBound)
- FastSig (FastSig const &that)=default
- FastSig & operator= (FastSig const &that)=default
- FastSig (FastSig &&that)=default
- FastSig & operator= (FastSig &&that)=default
- T operator() (T x)
- virtual ∼FastSig ()=default

9.43.1 Detailed Description

template<typename T>
class LinkPred::FastSig< T >

A fast sigmoid.

Template Parameters

T	Function value type (must be floating point).

9.43.2 Constructor & Destructor Documentation

9.43.2.1 FastSig() [1/4]

```
template<typename T >
LinkPred::FastSig< T >::FastSig ( )  [inline]
```

Default constructor.

9.43.2.2 FastSig() [2/4]

```
template<typename T >
LinkPred::FastSig< T >::FastSig (
            int n,
            T lowBound,
            T uppBound ) [inline]
```

Constructor.

Parameters

n	Sigmoid table size.
lowBound	Lower bound. Any input smaller than lowBound is assigned the value 0.
uppBound	Upper bound. Any input greater than uppBound is assigned the value 1.

9.43.2.3 FastSig() [3/4]

```
template<typename T >
LinkPred::FastSig< T >::FastSig (
            FastSig< T > const & that ) [default]
```

Copy constructor.

Parameters

that	The object to copy.

9.43.2.4 FastSig() [4/4]

```
template<typename T >
LinkPred::FastSig< T >::FastSig (
            FastSig< T > && that ) [default]
```

Move constructor.

Parameters

that	The object to move.

9.43.2.5 ∼FastSig()

```
template<typename T >
virtual LinkPred::FastSig< T >::~FastSig ( ) [virtual], [default]
```

Destructor.

9.43.3 Member Function Documentation

9.43.3.1 operator()()

```
template<typename T >
T LinkPred::FastSig< T >::operator() (
            T x ) [inline]
```

Parameters

x	Input value.

Returns

Sigmoid(x) (approximated of course).

9.43.3.2 operator=() [1/2]

```
template<typename T >
FastSig& LinkPred::FastSig< T >::operator= (
            FastSig< T > && that ) [default]
```

Move assignment operator.

Parameters

that	The object to move.

9.43.3.3 operator=() [2/2]

```
template<typename T >
FastSig& LinkPred::FastSig< T >::operator= (
            FastSig< T > const & that ) [default]
```

Copy assignment operator.

Parameters

that	The object to copy.

The documentation for this class was generated from the following file:

- include/linkpred/numerical/mf/fastsig.hpp

9.44 LinkPred::Simp::Evaluator::Factory::FBMParams Struct Reference

Parameters of FBM.

```
#include <evaluator.hpp>
```

Public Attributes

- int maxIter = 50
- long int seed = 0

9.44.1 Detailed Description

Parameters of FBM.

9.44.2 Member Data Documentation

9.44.2.1 maxIter

```
int LinkPred::Simp::Evaluator::Factory::FBMParams::maxIter = 50
```

Max iterations for FBM.

9.44.2.2 seed

```
long int LinkPred::Simp::Evaluator::Factory::FBMParams::seed = 0
```

Seed for FBM.

The documentation for this struct was generated from the following file:

- include/linkpred/simp/evaluator.hpp

9.45 LinkPred::GCurve< PredResultsT > Class Template Reference

General performance curve.

`#include <perfmeasure.hpp>`

Inheritance diagram for LinkPred::GCurve< PredResultsT >:

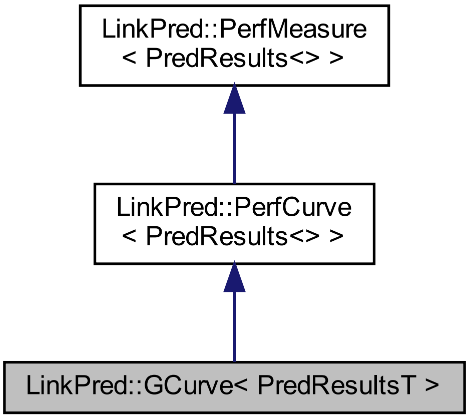

Collaboration diagram for LinkPred::GCurve< PredResultsT >:

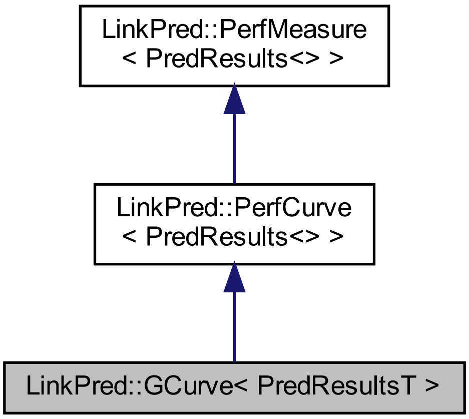

Public Types

- using ScoresItT = typename PerfMeasure< PredResultsT >::ScoresItT

Public Member Functions

- GCurve (typename PerfLambda::PerfLambdaT const &xLambda, typename PerfLambda::PerfLambdaT const &yLambda)
- GCurve (typename PerfLambda::PerfLambdaT const &xLambda, typename PerfLambda::PerfLambdaT const &yLambda, std::string name)
- GCurve (GCurve const &that)=default
- GCurve & operator= (GCurve const &that)=default
- GCurve (GCurve &&that)=default
- GCurve & operator= (GCurve &&that)=default
- virtual void eval (std::shared_ptr< PredResultsT > &predResults, PerfResults &results)
- virtual void eval (ScoresItT posScoresBegin, ScoresItT posScoresEnd, ScoresItT negScoresBegin, ScoresItT negScoresEnd, SortOrder &posSortOrder, SortOrder &negSortOrder, PerfResults &results)
- template<typename ScoresItT >
  std::vector< std::pair< double, double > > getGCurve (ScoresItT posScoresBegin, ScoresItT posScores↩End, ScoresItT negScoresBegin, ScoresItT negScoresEnd, bool parallel=false)
- virtual std::vector< std::pair< double, double > > getCurve (std::shared_ptr< PredResultsT > &pred↩Results)
- virtual std::vector< std::pair< double, double > > getCurve (ScoresItT posScoresBegin, ScoresItT pos↩ScoresEnd, ScoresItT negScoresBegin, ScoresItT negScoresEnd, SortOrder &posSortOrder, SortOrder &negSortOrder)
- virtual ~GCurve ()=default

Static Public Member Functions

- template<typename ScoresItT >
  static std::vector< double > getThresholds (ScoresItT posScoresBegin, ScoresItT posScoresEnd, ScoresItT negScoresBegin, ScoresItT negScoresEnd)
- static double getGCurveAuc (std::vector< std::pair< double, double >> const &curve, bool parallel=false)

9.45.1 Detailed Description

template<typename PredResultsT = PredResults<>>
class LinkPred::GCurve< PredResultsT >

General performance curve.

Template Parameters

PredResultsT	The prediction results type.

9.45.2 Member Typedef Documentation

9.45.2.1 ScoresItT

```
template<typename PredResultsT = PredResults<>>
using LinkPred::GCurve< PredResultsT >::ScoresItT = typename PerfMeasure<PredResultsT>↩
::ScoresItT
```

Scores iterator type.

9.45.3 Constructor & Destructor Documentation

9.45.3.1 GCurve() [1/4]

```
template<typename PredResultsT = PredResults<>>
LinkPred::GCurve< PredResultsT >::GCurve (
            typename PerfLambda::PerfLambdaT const & xLambda,
            typename PerfLambda::PerfLambdaT const & yLambda )  [inline]
```

Constructor.

Parameters

xLambda	A lambda to compute the x-coordinates.
yLambda	A lambda to compute the y-coordinates.

9.45.3.2 GCurve() [2/4]

```
template<typename PredResultsT = PredResults<>>
LinkPred::GCurve< PredResultsT >::GCurve (
            typename PerfLambda::PerfLambdaT const & xLambda,
            typename PerfLambda::PerfLambdaT const & yLambda,
            std::string name )  [inline]
```

Constructor.

Parameters

xLambda	A lambda to compute the x-coordinates.
yLambda	A lambda to compute the y-coordinates.
name	The name of the performance measure.

9.45.3.3 GCurve() [3/4]

```
template<typename PredResultsT = PredResults<>>
LinkPred::GCurve< PredResultsT >::GCurve (
            GCurve< PredResultsT > const & that )  [default]
```

Copy constructor.

Parameters

that	The object to copy.

9.45.3.4 GCurve() [4/4]

```
template<typename PredResultsT = PredResults<>>
LinkPred::GCurve< PredResultsT >::GCurve (
            GCurve< PredResultsT > && that ) [default]
```

Move constructor.

Parameters

that	The object to move.

9.45.3.5 ∼GCurve()

```
template<typename PredResultsT = PredResults<>>
virtual LinkPred::GCurve< PredResultsT >::~GCurve ( ) [virtual], [default]
```

Destructor.

9.45.4 Member Function Documentation

9.45.4.1 eval() [1/2]

```
template<typename PredResultsT = PredResults<>>
virtual void LinkPred::GCurve< PredResultsT >::eval (
            ScoresItT posScoresBegin,
            ScoresItT posScoresEnd,
            ScoresItT negScoresBegin,
            ScoresItT negScoresEnd,
            SortOrder & posSortOrder,
            SortOrder & negSortOrder,
            PerfResults & results ) [inline], [virtual]
```

Computes the area under the curve.

Parameters

posScoresBegin	Iterator to the first positive score.

Parameters

posScoresEnd	Iterator to one-past-the-last positive score.
negScoresBegin	Iterator to the first negative score.
negScoresEnd	Iterator to one-past-the-last negative score.
posSortOrder	The sorting order of positive scores. This may be modified by the method.
negSortOrder	The sorting order of negative scores. This may be modified by the method.
results	To write results.

9.45.4.2 eval() [2/2]

```
template<typename PredResultsT = PredResults<>>
virtual void LinkPred::GCurve< PredResultsT >::eval (
            std::shared_ptr< PredResultsT > & predResults,
            PerfResults & results )  [inline], [virtual]
```

Computes the area under the curve.

Parameters

predResults	The prediction results.
results	Iterator to write results.

9.45.4.3 getCurve() [1/2]

```
template<typename PredResultsT = PredResults<>>
virtual std::vector<std::pair<double, double> > LinkPred::GCurve< PredResultsT >::getCurve (
            ScoresItT posScoresBegin,
            ScoresItT posScoresEnd,
            ScoresItT negScoresBegin,
            ScoresItT negScoresEnd,
            SortOrder & posSortOrder,
            SortOrder & negSortOrder )  [inline], [virtual]
```

Computes the performance curve.

Parameters

posScoresBegin	Iterator to the first positive score.
posScoresEnd	Iterator to one-past-the-last positive score.
negScoresBegin	Iterator to the first negative score.
negScoresEnd	Iterator to one-past-the-last negative score.
posSortOrder	The sorting order of positive scores. This may be modified by the method.
negSortOrder	The sorting order of negative scores. This may be modified by the method.

Returns

A curve in the form of an std::vector of pairs representing the x and y coordinates.

9.45.4.4 getCurve() [2/2]

```
template<typename PredResultsT = PredResults<>>
virtual std::vector<std::pair<double, double> > LinkPred::GCurve< PredResultsT >::getCurve (
            std::shared_ptr< PredResultsT > & predResults ) [inline], [virtual]
```

Computes the performance curve.

Parameters

predResults	The prediction results.

Returns

A curve in the form of an std::vector of pairs representing the x and y coordinates.

9.45.4.5 getGCurve()

```
template<typename PredResultsT = PredResults<>>
template<typename ScoresItT >
std::vector<std::pair<double, double> > LinkPred::GCurve< PredResultsT >::getGCurve (
            ScoresItT posScoresBegin,
            ScoresItT posScoresEnd,
            ScoresItT negScoresBegin,
            ScoresItT negScoresEnd,
            bool parallel = false ) [inline]
```

Compute the curve. Both ranges must be sorted.

Parameters

posScoresBegin	Iterator to the first positive score.
posScoresEnd	Iterator to one-past-the-last positive score.
negScoresBegin	Iterator to the first negative score.
negScoresEnd	Iterator to one-past-the-last negative score.
parallel	Whether to run in parallel.

9.45.4.6 getGCurveAuc()

```
template<typename PredResultsT = PredResults<>>
```

```
static double LinkPred::GCurve< PredResultsT >::getGCurveAuc (
            std::vector< std::pair< double, double >> const & curve,
            bool parallel = false ) [inline], [static]
```

Computes the area under the curve using integration by linear interpolation.

Parameters

curve	The curve represented by its x-y coordinates.
parallel	Whether to run in parallel.

Returns

The area under the curve.

9.45.4.7 getThresholds()

```
template<typename PredResultsT = PredResults<>>
template<typename ScoresItT >
static std::vector<double> LinkPred::GCurve< PredResultsT >::getThresholds (
            ScoresItT posScoresBegin,
            ScoresItT posScoresEnd,
            ScoresItT negScoresBegin,
            ScoresItT negScoresEnd ) [inline], [static]
```

Compute the threshold of the GCurve curve. Ranges must be sorted in increasing order.

Parameters

posScoresBegin	Iterator to the first positive score.
posScoresEnd	Iterator to one-past-the-last positive score.
negScoresBegin	Iterator to the first negative score.
negScoresEnd	Iterator to one-past-the-last negative score.

9.45.4.8 operator=() [1/2]

```
template<typename PredResultsT = PredResults<>>
GCurve& LinkPred::GCurve< PredResultsT >::operator= (
            GCurve< PredResultsT > && that ) [default]
```

Move assignment operator.

Parameters

that	The object to move.

9.45.4.9 operator=() [2/2]

```
template<typename PredResultsT = PredResults<>>
GCurve& LinkPred::GCurve< PredResultsT >::operator= (
            GCurve< PredResultsT > const & that )  [default]
```

Copy assignment operator.

Parameters

that	The object to copy.

The documentation for this class was generated from the following file:

- include/linkpred/perf/perfmeasure.hpp

9.46 LinkPred::GFMatrix Class Reference

Generalized full matrix. The storage scheme used is column-major.

`#include <gfmatrix.hpp>`

Public Member Functions

- GFMatrix ()=delete
- GFMatrix (int m, int n, bool initZero=false)
- GFMatrix (GFMatrix const &that)
- GFMatrix & operator= (GFMatrix const &that)
- GFMatrix (GFMatrix &&that)
- GFMatrix & operator= (GFMatrix &&that)
- bool operator== (const GFMatrix &that) const
- bool operator!= (const GFMatrix &that) const
- void set (int i, int j, double v)
- double get (int i, int j) const
- void setRow (int i, Vec const &vec)
- Vec getRow (int i) const
- void setCol (int j, Vec const &vec)
- Vec getCol (int j) const
- GFMatrix getRows (std::vector< int > const &rowInd) const
- GFMatrix getCols (std::vector< int > const &colInd) const
- Vec sumCols () const
- Vec sumRows () const
- double sum ()
- void print () const
- void print (std::string name) const
- Vec diag () const
- void setDiag (double v)
- void removeNaN ()
- int getM () const
- int getN () const
- std::string getName () const
- void setName (const std::string &name)
- virtual ∼GFMatrix ()

Static Public Member Functions

- static GFMatrix mult (GFMatrix const &mat1, GFMatrix const &mat2, bool trans1=false, bool trans2=false)
- static GFMatrix elemMult (GFMatrix const &mat1, GFMatrix const &mat2)
- static GFMatrix mult (Vec const &v1, Vec const &v2)

Friends

- Vec operator* (const GFMatrix &mat, const Vec &vec)
- GFMatrix operator* (const GFMatrix &mat1, const GFMatrix &mat2)
- GFMatrix operator+ (const GFMatrix &mat1, const GFMatrix &mat2)
- GFMatrix operator- (const GFMatrix &mat1, const GFMatrix &mat2)
- GFMatrix operator/ (const GFMatrix &mat1, const GFMatrix &mat2)
- GFMatrix operator+ (const GFMatrix &mat1, const Vec &mat2)
- GFMatrix operator+ (const Vec &mat1, const GFMatrix &mat2)
- GFMatrix operator- (const GFMatrix &mat1, const Vec &mat2)
- GFMatrix operator- (const Vec &mat1, const GFMatrix &mat2)
- GFMatrix operator* (double a, const GFMatrix &mat)
- GFMatrix operator* (const GFMatrix &mat, double a)
- GFMatrix operator+ (double a, const GFMatrix &mat)
- GFMatrix operator+ (const GFMatrix &mat, double a)
- GFMatrix operator- (double a, const GFMatrix &mat)
- GFMatrix operator- (const GFMatrix &mat, double a)

9.46.1 Detailed Description

Generalized full matrix. The storage scheme used is column-major.

9.46.2 Constructor & Destructor Documentation

9.46.2.1 GFMatrix() [1/4]

```
LinkPred::GFMatrix::GFMatrix ( )  [delete]
```

Default constructor.

9.46.2.2 GFMatrix() [2/4]

```
LinkPred::GFMatrix::GFMatrix (
            int m,
            int n,
            bool initZero = false )
```

Constructor.

Parameters

m	The number of rows.
n	The number of columns.
initZero	If set to true, the matrix is initialized to zero.

9.46.2.3 GFMatrix() [3/4]

```
LinkPred::GFMatrix::GFMatrix (
            GFMatrix const & that )
```

Copy constructor.

Parameters

that	The object to copy.

9.46.2.4 GFMatrix() [4/4]

```
LinkPred::GFMatrix::GFMatrix (
            GFMatrix && that )
```

Move constructor.

Parameters

that	The object to move.

9.46.2.5 ∼GFMatrix()

```
virtual LinkPred::GFMatrix::∼GFMatrix ( )  [virtual]
```

Destructor.

9.46.3 Member Function Documentation

9.46.3.1 diag()

```
Vec LinkPred::GFMatrix::diag ( ) const
```

Returns

The diagonal of the matrix.

9.46.3.2 elemMult()

```
static GFMatrix LinkPred::GFMatrix::elemMult (
            GFMatrix const & mat1,
            GFMatrix const & mat2 ) [static]
```

Element-wise matrix multiplication.

Parameters

mat1	First matrix.
mat2	Second matrix.

Returns

The resulting matrix.

9.46.3.3 get()

```
double LinkPred::GFMatrix::get (
            int i,
            int j ) const [inline]
```

Get a matrix entry.

Parameters

i	Row index.
j	Column index.

9.46.3.4 getCol()

```
Vec LinkPred::GFMatrix::getCol (
            int j ) const
```

Parameters

j	The column index.

Returns

The column vector j.

9.46.3.5 getCols()

```
GFMatrix LinkPred::GFMatrix::getCols (
            std::vector< int > const & colInd ) const
```

Parameters

colInd	The column indexes.

Returns

The matrix composed from the specified columns.

9.46.3.6 getM()

```
int LinkPred::GFMatrix::getM ( ) const  [inline]
```

Returns

The number of rows.

9.46.3.7 getN()

```
int LinkPred::GFMatrix::getN ( ) const  [inline]
```

Returns

The number of columns.

9.46.3.8 getName()

```
std::string LinkPred::GFMatrix::getName ( ) const  [inline]
```

Returns

The name of the matrix.

9.46.3.9 getRow()

```
Vec LinkPred::GFMatrix::getRow (
            int i ) const
```

Parameters

i	The row index.

Returns

The row vector i.

9.46.3.10 getRows()

```
GFMatrix LinkPred::GFMatrix::getRows (
            std::vector< int > const & rowInd ) const
```

Parameters

rowInd	The row indexes.

Returns

The matrix composed from the specified rows.

9.46.3.11 mult() [1/2]

```
static GFMatrix LinkPred::GFMatrix::mult (
            GFMatrix const & mat1,
            GFMatrix const & mat2,
            bool trans1 = false,
            bool trans2 = false )  [static]
```

Matrix-matrix multiplication with possibility of transposing.

Parameters

mat1	First matrix.
mat2	Second matrix.
trans1	Transposing mat1.
trans2	Transposing mat2.

Returns

The resulting matrix.

9.46.3.12 mult() [2/2]

```
static GFMatrix LinkPred::GFMatrix::mult (
            Vec const & v1,
            Vec const & v2 )  [static]
```

Vector'-Vector multiplication.

Parameters

v1	First vector.
v2	Second vector.

Returns

The resulting matrix.

9.46.3.13 operator"!=()

```
bool LinkPred::GFMatrix::operator!= (
            const GFMatrix & that ) const
```

Parameters

that	The other matrix.

Returns

True if the two matrices are not equal.

9.46.3.14 operator=() [1/2]

```
GFMatrix& LinkPred::GFMatrix::operator= (
            GFMatrix && that )
```

Move assignment operator.

Parameters

that	The object to move.

9.46.3.15 operator=() [2/2]

```
GFMatrix& LinkPred::GFMatrix::operator= (
            GFMatrix const & that )
```

Copy assignment operator.

Parameters

that	The object to copy.

9.46.3.16 operator==()

```
bool LinkPred::GFMatrix::operator== (
            const GFMatrix & that ) const
```

Parameters

that	The other matrix.

Returns

True if the two matrices are equal.

9.46.3.17 print() [1/2]

```
void LinkPred::GFMatrix::print ( ) const
```

Print matrix to standard output.

9.46.3.18 print() [2/2]

```
void LinkPred::GFMatrix::print (
            std::string name ) const
```

Print matrix to standard output adding the name.

Parameters

name	Name added to the output.

9.46.3.19 removeNaN()

```
void LinkPred::GFMatrix::removeNaN ( )
```

Remove NaN entries.

9.46.3.20 set()

```
void LinkPred::GFMatrix::set (
            int i,
            int j,
            double v ) [inline]
```

Set a matrix entry.

Parameters

i	Row index.
j	Column index.
v	The new value.

9.46.3.21 setCol()

```
void LinkPred::GFMatrix::setCol (
            int j,
            Vec const & vec )
```

Set column vector.

Parameters

j	The column index.
vec	The column vector to set.

9.46.3.22 setDiag()

```
void LinkPred::GFMatrix::setDiag (
            double v )
```

Set diagonal.

Parameters

v	The value to be put in the diagonal.

9.46.3.23 setName()

```
void LinkPred::GFMatrix::setName (
            const std::string & name ) [inline]
```

Set the name of the matrix.

Parameters

name	The new matrix name.

9.46.3.24 setRow()

```
void LinkPred::GFMatrix::setRow (
            int i,
            Vec const & vec )
```

Set row vector.

Parameters

i	The row index.
vec	The row vector to set.

9.46.3.25 sum()

```
double LinkPred::GFMatrix::sum ( )
```

Returns

The sum of all elements.

9.46.3.26 sumCols()

```
Vec LinkPred::GFMatrix::sumCols ( ) const
```

Returns

The sum of the columns of the matrix.

9.46.3.27 sumRows()

```
Vec LinkPred::GFMatrix::sumRows ( ) const
```

Returns

The sum of the rows of the matrix.

9.46.4 Friends And Related Function Documentation

9.46.4.1 operator* [1/4]

```
Vec operator* (
            const GFMatrix & mat,
            const Vec & vec ) [friend]
```

GFMatrix-vector multiplication.

Parameters

mat	The matrix.
vec	The vector that will be multiplied by the matrix.

Returns

The resulting vector.

9.46.4.2 operator* [2/4]

```
GFMatrix operator* (
            const GFMatrix & mat,
            double a ) [friend]
```

GFMatrix * a.

Parameters

mat	The second matrix.
a	scalar.

Returns

The resulting matrix.

9.46.4.3 operator* [3/4]

```
GFMatrix operator* (
            const GFMatrix & mat1,
            const GFMatrix & mat2 ) [friend]
```

GFMatrix * GFMatrix.

Parameters

mat1	The first matrix.
mat2	The second matrix.

Returns

The resulting matrix.

9.46.4.4 operator* [4/4]

```
GFMatrix operator* (
            double a,
            const GFMatrix & mat ) [friend]
```

a * GFMatrix.

Parameters

a	scalar.
mat	The second matrix.

Returns

The resulting matrix.

9.46.4.5 operator+ [1/5]

```
GFMatrix operator+ (
            const GFMatrix & mat,
            double a )  [friend]
```

GFMatrix + a.

Parameters

mat	The second matrix.
a	A scalar.

Returns

The resulting matrix.

9.46.4.6 operator+ [2/5]

```
GFMatrix operator+ (
            const GFMatrix & mat1,
            const GFMatrix & mat2 )  [friend]
```

GFMatrix + GFMatrix.

Parameters

mat1	The first matrix.
mat2	The second matrix.

Returns

The resulting matrix.

9.46.4.7 operator+ [3/5]

```
GFMatrix operator+ (
            const GFMatrix & mat1,
            const Vec & mat2 )  [friend]
```

GFMatrix + Vec.

Parameters

mat1	The first matrix.
mat2	The second matrix (diagonal).

Returns

The resulting matrix.

9.46.4.8 operator+ [4/5]

```
GFMatrix operator+ (
            const Vec & mat1,
            const GFMatrix & mat2 )  [friend]
```

Vec + GFMatrix.

Parameters

mat1	The first matrix (diagonal).
mat2	The second matrix.

Returns

The resulting matrix.

9.46.4.9 operator+ [5/5]

```
GFMatrix operator+ (
            double a,
            const GFMatrix & mat )  [friend]
```

a + GFMatrix.

Parameters

a	scalar.
mat	The second matrix.

Returns

The resulting matrix.

9.46.4.10 operator- [1/5]

```
GFMatrix operator- (
            const GFMatrix & mat,
            double a ) [friend]
```

GFMatrix - a.

Parameters

mat	The second matrix.
a	scalar.

Returns

The resulting matrix.

9.46.4.11 operator- [2/5]

```
GFMatrix operator- (
            const GFMatrix & mat1,
            const GFMatrix & mat2 ) [friend]
```

GFMatrix - GFMatrix.

Parameters

mat1	The first matrix.
mat2	The second matrix.

Returns

The resulting matrix.

9.46.4.12 operator- [3/5]

```
GFMatrix operator- (
            const GFMatrix & mat1,
            const Vec & mat2 ) [friend]
```

GFMatrix - Vec.

Parameters

mat1	The first matrix.
mat2	The second matrix (diagonal).

Returns

The resulting matrix.

9.46.4.13 operator- [4/5]

```
GFMatrix operator- (
            const Vec & mat1,
            const GFMatrix & mat2 )  [friend]
```

Vec - GFMatrix.

Parameters

mat1	The first matrix (diagonal).
mat2	The second matrix.

Returns

The resulting matrix.

9.46.4.14 operator- [5/5]

```
GFMatrix operator- (
            double a,
            const GFMatrix & mat )  [friend]
```

a - GFMatrix.

Parameters

a	scalar.
mat	The second matrix.

Returns

The resulting matrix.

9.46.4.15 operator/

```
GFMatrix operator/ (
            const GFMatrix & mat1,
            const GFMatrix & mat2 )  [friend]
```

GFMatrix / GFMatrix (element-wise division).

Parameters

mat1	The first matrix.
mat2	The second matrix.

Returns

The resulting matrix.

The documentation for this class was generated from the following file:

- include/linkpred/numerical/linear/gfmatrix.hpp

9.47 LinkPred::GraphTraversal< Network, NodeProcessor > Class Template Reference

Graph traversal interface.

`#include <graphtraversal.hpp>`

Public Member Functions

- GraphTraversal (std::shared_ptr< Network const > net)
- GraphTraversal (GraphTraversal const &that)=default
- GraphTraversal & operator= (GraphTraversal const &that)=default
- GraphTraversal (GraphTraversal &&that)=default
- GraphTraversal & operator= (GraphTraversal &&that)=default
- virtual void traverse (typename Network::NodeID srcNode, NodeProcessor &processor)=0
- virtual ~GraphTraversal ()=default

9.47.1 Detailed Description

template<typename Network = UNetwork<>, typename NodeProcessor = Collector< Network>>
class LinkPred::GraphTraversal< Network, NodeProcessor >

Graph traversal interface.

Template Parameters

Network	The network type.
NodeProcessor	The node processor type.

9.47.2 Constructor & Destructor Documentation

9.47.2.1 GraphTraversal() [1/3]

```
template<typename Network = UNetwork<>, typename NodeProcessor = Collector< Network>>
LinkPred::GraphTraversal< Network, NodeProcessor >::GraphTraversal (
            std::shared_ptr< Network const > net ) [inline]
```

Constructor.

Parameters

net	The network on which traversal is done.

9.47.2.2 GraphTraversal() [2/3]

```
template<typename Network = UNetwork<>, typename NodeProcessor = Collector< Network>>
LinkPred::GraphTraversal< Network, NodeProcessor >::GraphTraversal (
            GraphTraversal< Network, NodeProcessor > const & that ) [default]
```

Copy constructor.

Parameters

that	The object to copy.

9.47.2.3 GraphTraversal() [3/3]

```
template<typename Network = UNetwork<>, typename NodeProcessor = Collector< Network>>
LinkPred::GraphTraversal< Network, NodeProcessor >::GraphTraversal (
            GraphTraversal< Network, NodeProcessor > && that ) [default]
```

Move constructor.

Parameters

that	The object to move.

9.47.2.4 ∼GraphTraversal()

```
template<typename Network = UNetwork<>, typename NodeProcessor = Collector< Network>>
virtual LinkPred::GraphTraversal< Network, NodeProcessor >::~GraphTraversal ( ) [virtual],
[default]
```

Destructor.

9.47.3 Member Function Documentation

9.47.3.1 operator=() [1/2]

```
template<typename Network = UNetwork<>, typename NodeProcessor = Collector< Network>>
GraphTraversal& LinkPred::GraphTraversal< Network, NodeProcessor >::operator= (
            GraphTraversal< Network, NodeProcessor > && that )  [default]
```

Move assignment operator.

Parameters

that	The object to move.

9.47.3.2 operator=() [2/2]

```
template<typename Network = UNetwork<>, typename NodeProcessor = Collector< Network>>
GraphTraversal& LinkPred::GraphTraversal< Network, NodeProcessor >::operator= (
            GraphTraversal< Network, NodeProcessor > const & that )  [default]
```

Copy assignment operator.

Parameters

that	The object to copy.

9.47.3.3 traverse()

```
template<typename Network = UNetwork<>, typename NodeProcessor = Collector< Network>>
virtual void LinkPred::GraphTraversal< Network, NodeProcessor >::traverse (
            typename Network::NodeID srcNode,
            NodeProcessor & processor )  [pure virtual]
```

Traverse the graph.

Parameters

srcNode	The source node.
processor	The node processor.

The documentation for this class was generated from the following file:

- include/linkpred/graphalg/traversal/graphtraversal.hpp

9.48 LinkPred::HMSM< Network > Class Template Reference

Contains the implementation of an algorithm for embedding a network using a a hidden metric space model. Reference: R. Alharbi, H. Benhidour, and S. Kerrache. “Link Prediction in Complex Net-works Based on a Hidden Variables Model”. In: 2016 UKSim-AMSS 18th Inter-national Conference on Computer Modelling and Simulation (UKSim). 2016,pages 119–124.

```
#include <hmsm.hpp>
```

Inheritance diagram for LinkPred::HMSM< Network >:

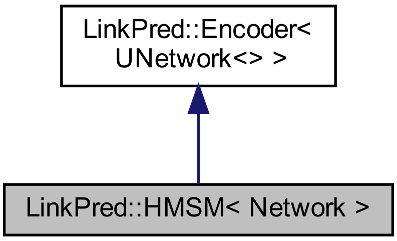

Collaboration diagram for LinkPred::HMSM< Network >:

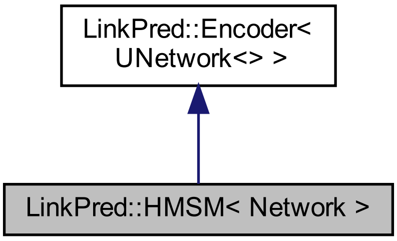

Public Member Functions

- HMSM (std::shared_ptr< Network const > net, long int seed)
- HMSM (HMSM const &that)=default
- HMSM & operator= (HMSM const &that)=default
- HMSM (HMSM &&that)=default

- HMSM & operator= (HMSM &&that)=default
- virtual void init ()
- virtual void encode ()
- virtual void setWeightMap (const WeightMapSP &weightMap)
- const int getDefaultDim () const
- double getAlpha () const
- void setAlpha (double alpha)
- double getEps () const
- void setEps (double eps)
- double getHProb () const
- void setHProb (double hProb)
- double getLProb () const
- void setProb (double lProb)
- int getNbMdsRuns () const
- void setNbMdsRuns (int nbMdsRuns)
- double getTol () const
- void setTol (double tol)
- virtual ∼HMSM ()=default

Additional Inherited Members

9.48.1 Detailed Description

template<typename Network = UNetwork<>>
class LinkPred::HMSM< Network >

Contains the implementation of an algorithm for embedding a network using a a hidden metric space model. Reference: R. Alharbi, H. Benhidour, and S. Kerrache. “Link Prediction in Complex Net-works Based on a Hidden Variables Model”. In: 2016 UKSim-AMSS 18th Inter-national Conference on Computer Modelling and Simulation (UKSim). 2016,pages 119–124.

Template Parameters

Network	The network type.

9.48.2 Constructor & Destructor Documentation

9.48.2.1 HMSM() [1/3]

```
template<typename Network = UNetwork<>>
LinkPred::HMSM< Network >::HMSM (
            std::shared_ptr< Network const > net,
            long int seed )  [inline]
```

Constructor.

Parameters

net	The network.
seed	Random number generator seed.

9.48.2.2 HMSM() [2/3]

```
template<typename Network = UNetwork<>>
LinkPred::HMSM< Network >::HMSM (
            HMSM< Network > const & that )  [default]
```

Copy constructor.

Parameters

that	The object to copy.

9.48.2.3 HMSM() [3/3]

```
template<typename Network = UNetwork<>>
LinkPred::HMSM< Network >::HMSM (
            HMSM< Network > && that )  [default]
```

Move constructor.

Parameters

that	The object to move.

9.48.2.4 ∼HMSM()

```
template<typename Network = UNetwork<>>
virtual LinkPred::HMSM< Network >::~HMSM ( )  [virtual], [default]
```

Destructor.

9.48.3 Member Function Documentation

9.48.3.1 encode()

```
template<typename Network = UNetwork<>>
virtual void LinkPred::HMSM< Network >::encode ( )  [virtual]
```

Encode the network.

Implements LinkPred::Encoder< UNetwork<> >.

9.48.3.2 getAlpha()

```
template<typename Network = UNetwork<>>
double LinkPred::HMSM< Network >::getAlpha ( ) const  [inline]
```

Returns

The parameter alpha of the HMSM model.

9.48.3.3 getDefaultDim()

```
template<typename Network = UNetwork<>>
const int LinkPred::HMSM< Network >::getDefaultDim ( ) const  [inline]
```

Returns

The default embedding dimension.

9.48.3.4 getEps()

```
template<typename Network = UNetwork<>>
double LinkPred::HMSM< Network >::getEps ( ) const  [inline]
```

Returns

Degree product when of the nodes has degree 0.

9.48.3.5 getHProb()

```
template<typename Network = UNetwork<>>
double LinkPred::HMSM< Network >::getHProb ( ) const  [inline]
```

Probability assigned to connected couples.

9.48.3.6 getLProb()

```
template<typename Network = UNetwork<>>
double LinkPred::HMSM< Network >::getLProb ( ) const [inline]
```

Probability assigned to disconnected couples.

9.48.3.7 getNbMdsRuns()

```
template<typename Network = UNetwork<>>
int LinkPred::HMSM< Network >::getNbMdsRuns ( ) const [inline]
```

Returns

The number of times MDS is run (with default starting point).

9.48.3.8 getTol()

```
template<typename Network = UNetwork<>>
double LinkPred::HMSM< Network >::getTol ( ) const [inline]
```

Returns

Tolerance for stopping the MDS optimization problem.

9.48.3.9 init()

```
template<typename Network = UNetwork<>>
virtual void LinkPred::HMSM< Network >::init ( ) [virtual]
```

Initialize encoder.

Implements LinkPred::Encoder< UNetwork<> >.

9.48.3.10 operator=() [1/2]

```
template<typename Network = UNetwork<>>
HMSM& LinkPred::HMSM< Network >::operator= (
            HMSM< Network > && that ) [default]
```

Move assignment operator.

Parameters

that	The object to move.

9.48.3.11 operator=() [2/2]

```
template<typename Network = UNetwork<>>
HMSM& LinkPred::HMSM< Network >::operator= (
            HMSM< Network > const & that )  [default]
```

Copy assignment operator.

Parameters

that	The object to copy.

9.48.3.12 setAlpha()

```
template<typename Network = UNetwork<>>
void LinkPred::HMSM< Network >::setAlpha (
            double alpha )  [inline]
```

Set the parameter alpha of the HMSM model.

Parameters

alpha	The parameter alpha of the HMSM model.

9.48.3.13 setEps()

```
template<typename Network = UNetwork<>>
void LinkPred::HMSM< Network >::setEps (
            double eps )  [inline]
```

Set degree product when of the nodes has degree 0.

Parameters

eps	Degree product when of the nodes has degree 0.

9.48.3.14 setHProb()

```
template<typename Network = UNetwork<>>
void LinkPred::HMSM< Network >::setHProb (
            double hProb )  [inline]
```

Set the probability assigned to connected couples.

Parameters

hProb	Probability assigned to connected couples.

9.48.3.15 setNbMdsRuns()

```
template<typename Network = UNetwork<>>
void LinkPred::HMSM< Network >::setNbMdsRuns (
            int nbMdsRuns )  [inline]
```

Set the number of times MDS is run (with default starting point).

Parameters

nbMdsRuns	Number of times MDS is run (with default starting point).

9.48.3.16 setProb()

```
template<typename Network = UNetwork<>>
void LinkPred::HMSM< Network >::setProb (
            double lProb )  [inline]
```

Set the probability assigned to disconnected couples.

Parameters

lProb	Probability assigned to disconnected couples.

9.48.3.17 setTol()

```
template<typename Network = UNetwork<>>
void LinkPred::HMSM< Network >::setTol (
            double tol )  [inline]
```

Set the tolerance for stopping the MDS optimization problem.

Parameters

tol	Tolerance for stopping the MDS optimization problem.

9.48.3.18 setWeightMap()

```
template<typename Network = UNetwork<>>
virtual void LinkPred::HMSM< Network >::setWeightMap (
            const WeightMapSP & weightMap )  [inline], [virtual]
```

Set edge weight map.

Parameters

weightMap	Edge weight map.

Reimplemented from LinkPred::Encoder< UNetwork<> >.

The documentation for this class was generated from the following file:

- include/linkpred/graphalg/encoders/hmsm/hmsm.hpp

9.49 LinkPred::Simp::Evaluator::Factory::HRGParams Struct Reference

Parameters of HRG.

```
#include <evaluator.hpp>
```

Public Attributes

- int nbBeans = 25
- int nbSamples = 10000
- long int seed = 0

9.49.1 Detailed Description

Parameters of HRG.

9.49.2 Member Data Documentation

9.49.2.1 nbBeans

```
int LinkPred::Simp::Evaluator::Factory::HRGParams::nbBeans = 25
```

Number of bins in edge statistics histogram in HRG.

9.49.2.2 nbSamples

```
int LinkPred::Simp::Evaluator::Factory::HRGParams::nbSamples = 10000
```

Number of samples to take for predictions in HRG.

9.49.2.3 seed

```
long int LinkPred::Simp::Evaluator::Factory::HRGParams::seed = 0
```

Seed for HRG

The documentation for this struct was generated from the following file:

- include/linkpred/simp/evaluator.hpp

9.50 LinkPred::Simp::Evaluator::Factory::HYPParams Struct Reference

Parameters of HYP.

```
#include <evaluator.hpp>
```

Public Attributes

- double m = 1.5
- double L = 1
- double gamma = 2.1
- double zeta = 1
- double T = 0.8
- long int seed = 0

9.50.1 Detailed Description

Parameters of HYP.

9.50.2 Member Data Documentation

9.50.2.1 gamma

```
double LinkPred::Simp::Evaluator::Factory::HYPParams::gamma = 2.1
```

The power law exponent gamma (see the HYP algorithm description).

9.50.2.2 L

```
double LinkPred::Simp::Evaluator::Factory::HYPParams::L = 1
```

The parameter L (see the HYP algorithm description).

9.50.2.3 m

```
double LinkPred::Simp::Evaluator::Factory::HYPParams::m = 1.5
```

The parameter m (see the HYP algorithm description).

9.50.2.4 seed

```
long int LinkPred::Simp::Evaluator::Factory::HYPParams::seed = 0
```

The random number generator seed for HYP.

9.50.2.5 T

```
double LinkPred::Simp::Evaluator::Factory::HYPParams::T = 0.8
```

The parameter T (see the HYP algorithm description).

9.50.2.6 zeta

```
double LinkPred::Simp::Evaluator::Factory::HYPParams::zeta = 1
```

The parameter zeta (see the HYP algorithm description).

The documentation for this struct was generated from the following file:

- include/linkpred/simp/evaluator.hpp

9.51 LinkPred::Simp::Evaluator::Factory::KABParams Struct Reference

Parameters of KAB.

```
#include <evaluator.hpp>
```

Public Attributes

- int horizLim = 2

9.51.1 Detailed Description

Parameters of KAB.

9.51.2 Member Data Documentation

9.51.2.1 horizLim

```
int LinkPred::Simp::Evaluator::Factory::KABParams::horizLim = 2
```

Horizon limit for KAB.

The documentation for this struct was generated from the following file:

- include/linkpred/simp/evaluator.hpp

9.52 LinkPred::L1Sim Class Reference

L1 similarity (negative the L1 norm or Manhattan distance).

```
#include <l1sim.hpp>
```

Inheritance diagram for LinkPred::L1Sim:

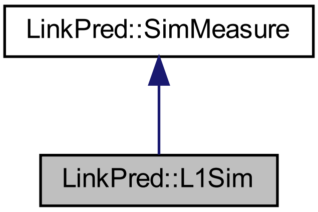

Collaboration diagram for LinkPred::L1Sim:

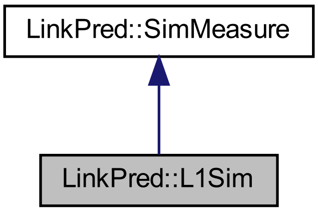

Public Member Functions

- L1Sim ()
- L1Sim (L1Sim const &that)=default
- L1Sim & operator= (L1Sim const &that)=default
- L1Sim (L1Sim &&that)=default
- L1Sim & operator= (L1Sim &&that)=default
- virtual double sim (Vec const &v1, Vec const &v2)
- virtual ∼L1Sim ()=default

9.52.1 Detailed Description

L1 similarity (negative the L1 norm or Manhattan distance).

9.52.2 Constructor & Destructor Documentation

9.52.2.1 L1Sim() [1/3]

```
LinkPred::L1Sim::L1Sim ( )  [inline]
```

Constructor.

9.52.2.2 L1Sim() [2/3]

```
LinkPred::L1Sim::L1Sim (
            L1Sim const & that )  [default]
```

Copy constructor.

Parameters

that	The object to copy.

9.52.2.3 L1Sim() [3/3]

```
LinkPred::L1Sim::L1Sim (
            L1Sim && that )  [default]
```

Move constructor.

Parameters

that	The object to move.

9.52.2.4 ∼L1Sim()

```
virtual LinkPred::L1Sim::∼L1Sim ( )  [virtual], [default]
```

Destructor.

9.52.3 Member Function Documentation

9.52.3.1 operator=() [1/2]

```
L1Sim& LinkPred::L1Sim::operator= (
            L1Sim && that )  [default]
```

Move assignment operator.

Parameters

that	The object to move.

9.52.3.2 operator=() [2/2]

```
L1Sim& LinkPred::L1Sim::operator= (
            L1Sim const & that )  [default]
```

Copy assignment operator.

Parameters

that	The object to copy.

9.52.3.3 sim()

```
virtual double LinkPred::L1Sim::sim (
            Vec const & v1,
            Vec const & v2 )  [virtual]
```

Compute the similarity between two vectors.

Parameters

v1	First vector.
v2	Second vector. Must be of the same dimension as v1.

Returns

The similarity between v1 and v2.

Implements LinkPred::SimMeasure.

The documentation for this class was generated from the following file:

- include/linkpred/ml/simmeasures/l1sim.hpp

9.53 LinkPred::L2Sim Class Reference

L2 similarity (negative the Euclidean distance).

`#include <l2sim.hpp>`

Inheritance diagram for LinkPred::L2Sim:

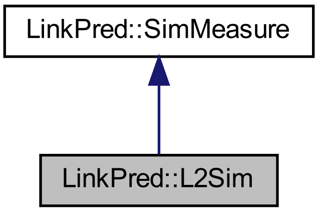

Collaboration diagram for LinkPred::L2Sim:

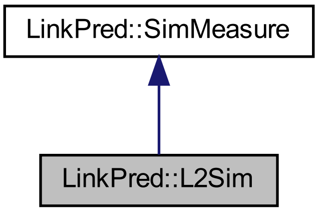

Public Member Functions

- L2Sim ()
- L2Sim (L2Sim const &that)=default
- L2Sim & operator= (L2Sim const &that)=default
- L2Sim (L2Sim &&that)=default
- L2Sim & operator= (L2Sim &&that)=default
- virtual double sim (Vec const &v1, Vec const &v2)
- virtual ~L2Sim ()=default

9.53.1 Detailed Description

L2 similarity (negative the Euclidean distance).

9.53.2 Constructor & Destructor Documentation

9.53.2.1 L2Sim() [1/3]

```
LinkPred::L2Sim::L2Sim ( )  [inline]
```

Constructor.

9.53.2.2 L2Sim() [2/3]

```
LinkPred::L2Sim::L2Sim (
            L2Sim const & that )  [default]
```

Copy constructor.

Parameters

that	The object to copy.

9.53.2.3 L2Sim() [3/3]

```
LinkPred::L2Sim::L2Sim (
            L2Sim && that )  [default]
```

Move constructor.

Parameters

that	The object to move.

9.53.2.4 ∼L2Sim()

```
virtual LinkPred::L2Sim::∼L2Sim ( )  [virtual], [default]
```

Destructor.

9.53.3 Member Function Documentation

9.53.3.1 operator=() [1/2]

```
L2Sim& LinkPred::L2Sim::operator= (
            L2Sim && that )  [default]
```

Move assignment operator.

Parameters

that	The object to move.

9.53.3.2 operator=() [2/2]

```
L2Sim& LinkPred::L2Sim::operator= (
            L2Sim const & that )  [default]
```

Copy assignment operator.

Parameters

that	The object to copy.

9.53.3.3 sim()

```
virtual double LinkPred::L2Sim::sim (
            Vec const & v1,
            Vec const & v2 )  [virtual]
```

Compute the similarity between two vectors.

Parameters

v1	First vector.
v2	Second vector. Must be of the same dimension as v1.

Returns

The similarity between v1 and v2.

Implements LinkPred::SimMeasure.

The documentation for this class was generated from the following file:

- include/linkpred/ml/simmeasures/l2sim.hpp

9.54 LinkPred::LargeVis< Network > Class Template Reference

LargeVis encoder. Reference: Tang, J., Liu, J., Zhang, M., and Mei, Q. (2016b). Visualizing large-scale and high-dimensional data. In Bourdeau, J., Hendler, J., Nkambou, R., Horrocks, I., and Zhao, B. Y., editors, WWW, pages 287–297. ACM. This implementation is based on the code `https://github.com/lferry007/Large↩ Vis`.

```
#include <largevis.hpp>
```

Inheritance diagram for LinkPred::LargeVis< Network >:

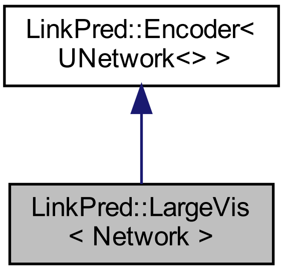

Collaboration diagram for LinkPred::LargeVis< Network >:

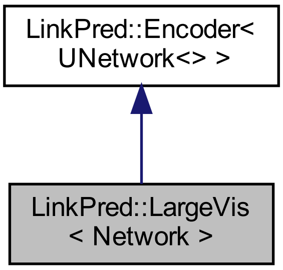

Public Member Functions

- LargeVis (std::shared_ptr< Network const > net, long int seed)
- LargeVis (LargeVis const &that)=default
- LargeVis & operator= (LargeVis const &that)=default
- LargeVis (LargeVis &&that)=default
- LargeVis & operator= (LargeVis &&that)=default
- virtual void init ()
- virtual void encode ()
- void setNbSamples ()
- float getGamma () const
- void setGamma (float gamma)
- float getInitLR () const
- void setInitLR (float initLR)
- long long getNbNegSamples () const
- void setNbNegSamples (long long nbNegSamples)
- long long getNbSamples () const

- void setNbSamples (long long nbSamples)
- long long getNegSize () const
- void setNegSize (long long negSize)
- float getNegSamplingPower () const
- void setNegSamplingPower (float negSamplingPower)
- const int getDefaultDim () const
- virtual ~LargeVis ()=default

Additional Inherited Members

9.54.1 Detailed Description

template<typename Network = UNetwork<>>
class LinkPred::LargeVis< Network >

LargeVis encoder. Reference: Tang, J., Liu, J., Zhang, M., and Mei, Q. (2016b). Visualizing large-scale and high-dimensional data. In Bourdeau, J., Hendler, J., Nkambou, R., Horrocks, I., and Zhao, B. Y., editors, WWW, pages 287–297. ACM. This implementation is based on the code `https://github.com/lferry007/LargeVis`.

Template Parameters

Network	The network type.

9.54.2 Constructor & Destructor Documentation

9.54.2.1 LargeVis() [1/3]

```
template<typename Network = UNetwork<>>
LinkPred::LargeVis< Network >::LargeVis (
            std::shared_ptr< Network const > net,
            long int seed ) [inline]
```

Constructor.

Parameters

net	The network.
seed	Random number generator seed.

9.54.2.2 LargeVis() [2/3]

```
template<typename Network = UNetwork<>>
```

```
LinkPred::LargeVis< Network >::LargeVis (
            LargeVis< Network > const & that )  [default]
```

Copy constructor.

Parameters

that	The object to copy.

9.54.2.3 LargeVis() [3/3]

```
template<typename Network = UNetwork<>>
LinkPred::LargeVis< Network >::LargeVis (
            LargeVis< Network > && that )  [default]
```

Move constructor.

Parameters

that	The object to move.

9.54.2.4 ∼LargeVis()

```
template<typename Network = UNetwork<>>
virtual LinkPred::LargeVis< Network >::~LargeVis ( )  [virtual], [default]
```

Destructor.

9.54.3 Member Function Documentation

9.54.3.1 encode()

```
template<typename Network = UNetwork<>>
virtual void LinkPred::LargeVis< Network >::encode ( )  [virtual]
```

Encode the network.

Implements LinkPred::Encoder< UNetwork<> >.

9.54.3.2 getDefaultDim()

```
template<typename Network = UNetwork<>>
const int LinkPred::LargeVis< Network >::getDefaultDim ( ) const  [inline]
```

Returns

The default embedding dimension.

9.54.3.3 getGamma()

```
template<typename Network = UNetwork<>>
float LinkPred::LargeVis< Network >::getGamma ( ) const  [inline]
```

Returns

The parameter gamma.

9.54.3.4 getInitLR()

```
template<typename Network = UNetwork<>>
float LinkPred::LargeVis< Network >::getInitLR ( ) const  [inline]
```

Returns

Initial learning rate.

9.54.3.5 getNbNegSamples()

```
template<typename Network = UNetwork<>>
long long LinkPred::LargeVis< Network >::getNbNegSamples ( ) const  [inline]
```

Returns

Number of negative samples.

9.54.3.6 getNbSamples()

```
template<typename Network = UNetwork<>>
long long LinkPred::LargeVis< Network >::getNbSamples ( ) const  [inline]
```

Returns

Number of samples.

9.54.3.7 getNegSamplingPower()

```
template<typename Network = UNetwork<>>
float LinkPred::LargeVis< Network >::getNegSamplingPower ( ) const  [inline]
```

Parameters

Power	used in negative sampling.

9.54.3.8 getNegSize()

```
template<typename Network = UNetwork<>>
long long LinkPred::LargeVis< Network >::getNegSize ( ) const  [inline]
```

Returns

The size of the table used in negative sampling.

9.54.3.9 init()

```
template<typename Network = UNetwork<>>
virtual void LinkPred::LargeVis< Network >::init ( )  [virtual]
```

Initialize encoder.

Implements LinkPred::Encoder< UNetwork<> >.

9.54.3.10 operator=() [1/2]

```
template<typename Network = UNetwork<>>
LargeVis& LinkPred::LargeVis< Network >::operator= (
            LargeVis< Network > && that )  [default]
```

Move assignment operator.

Parameters

that	The object to move.

9.54.3.11 operator=() [2/2]

```
template<typename Network = UNetwork<>>
LargeVis& LinkPred::LargeVis< Network >::operator= (
            LargeVis< Network > const & that )  [default]
```

Copy assignment operator.

Parameters

that	The object to copy.

9.54.3.12 setGamma()

```
template<typename Network = UNetwork<>>
void LinkPred::LargeVis< Network >::setGamma (
            float gamma ) [inline]
```

Set the parameter gamma.

Parameters

gamma	The parameter gamma.

9.54.3.13 setInitLR()

```
template<typename Network = UNetwork<>>
void LinkPred::LargeVis< Network >::setInitLR (
            float initLR ) [inline]
```

Set the initial learning rate.

Parameters

initLR	The initial learning rate.

9.54.3.14 setNbNegSamples()

```
template<typename Network = UNetwork<>>
void LinkPred::LargeVis< Network >::setNbNegSamples (
            long long nbNegSamples ) [inline]
```

Set the number of negative samples.

Parameters

nbNegSamples	The number of negative samples.

9.54.3.15 setNbSamples() [1/2]

```
template<typename Network = UNetwork<>>
void LinkPred::LargeVis< Network >::setNbSamples ( )
```

Set nbSamples using a simple rule of thumb.

9.54.3.16 setNbSamples() [2/2]

```
template<typename Network = UNetwork<>>
void LinkPred::LargeVis< Network >::setNbSamples (
             long long nbSamples )  [inline]
```

Set the number of samples.

Parameters

nbSamples	The number of samples.

9.54.3.17 setNegSamplingPower()

```
template<typename Network = UNetwork<>>
void LinkPred::LargeVis< Network >::setNegSamplingPower (
             float negSamplingPower )  [inline]
```

Set the power used in negative sampling.

Parameters

negSamplingPower	Power used in negative sampling.

9.54.3.18 setNegSize()

```
template<typename Network = UNetwork<>>
void LinkPred::LargeVis< Network >::setNegSize (
             long long negSize )  [inline]
```

Set the size of the table used in negative sampling.

Parameters

negSize	Size of the table used in negative sampling.

The documentation for this class was generated from the following file:

- include/linkpred/graphalg/encoders/largevis/largevis.hpp

9.55 LinkPred::Simp::Evaluator::Factory::LCPParams Struct Reference

Parameters of LCP.

`#include <evaluator.hpp>`

Public Attributes

- double epsilon = 0.001

9.55.1 Detailed Description

Parameters of LCP.

9.55.2 Member Data Documentation

9.55.2.1 epsilon

```
double LinkPred::Simp::Evaluator::Factory::LCPParams::epsilon = 0.001
```

The weight of paths of length 3 in LCP.

The documentation for this struct was generated from the following file:

- include/linkpred/simp/evaluator.hpp

9.56 LinkPred::LINE< Network > Class Template Reference

LINE encoder.

```
#include <line.hpp>
```

Inheritance diagram for LinkPred::LINE< Network >:

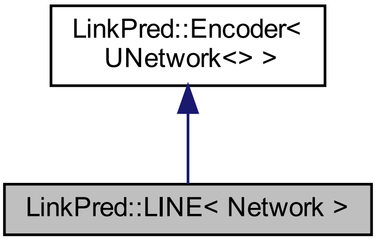

Collaboration diagram for LinkPred::LINE< Network >:

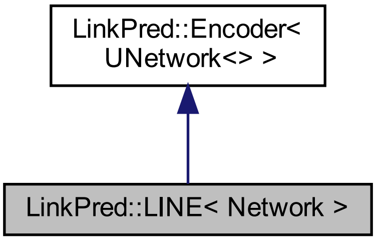

Public Member Functions

- LINE (std::shared_ptr< Network const > net, long int seed)
- LINE (LINE const &that)=default
- LINE & operator= (LINE const &that)=default
- LINE (LINE &&that)=default
- LINE & operator= (LINE &&that)=default
- virtual void init ()
- virtual void encode ()
- float getInitLr () const
- void setInitLr (float initLR)
- int getNbNegSamples () const
- void setNbNegSamples (int nbNegSamples)

- int getOrder () const
- void setOrder (int order)
- float getNegSamplingPower () const
- void setNegSamplingPower (float negSamplingPower)
- long long getNegSize () const
- void setNegSize (long long negSize)
- bool isEnableReconstruct () const
- void setEnableReconstruct (bool enableReconstruct)
- const std::shared_ptr< const Network > getRecNet () const
- const WeightMapSP & getRecWeightMap () const
- const int getDefaultDim () const
- virtual ∼LINE ()=default

Additional Inherited Members

9.56.1 Detailed Description

template<typename Network = UNetwork<>>
class LinkPred::LINE< Network >

LINE encoder.

This implementation is based on the code https://github.com/tangjianpku/LINE Reference: Tang, J., Qu, M., Wang, M., Zhang, M., Yan, J., and Mei, Q. (2015). LINE: Large-Scale Information Network Embedding, page 1067–1077. International World Wide Web Conferences Steering Committee, Republic and Canton of Geneva, CHE.

Template Parameters

Network	The network type.

9.56.2 Constructor & Destructor Documentation

9.56.2.1 LINE() [1/3]

```
template<typename Network = UNetwork<>>
LinkPred::LINE< Network >::LINE (
            std::shared_ptr< Network const > net,
            long int seed )  [inline]
```

Constructor.

Parameters

net	The network.
seed	Random number generator seed.

9.56.2.2 LINE() [2/3]

```
template<typename Network = UNetwork<>>
LinkPred::LINE< Network >::LINE (
             LINE< Network > const & that )  [default]
```

Copy constructor.

Parameters

that	The object to copy.

9.56.2.3 LINE() [3/3]

```
template<typename Network = UNetwork<>>
LinkPred::LINE< Network >::LINE (
             LINE< Network > && that )  [default]
```

Move constructor.

Parameters

that	The object to move.

9.56.2.4 ~LINE()

```
template<typename Network = UNetwork<>>
virtual LinkPred::LINE< Network >::~LINE ( )  [virtual], [default]
```

Destructor.

9.56.3 Member Function Documentation

9.56.3.1 encode()

```
template<typename Network = UNetwork<>>
virtual void LinkPred::LINE< Network >::encode ( )  [virtual]
```

Encode the network.

Implements LinkPred::Encoder< UNetwork<> >.

9.56.3.2 getDefaultDim()

```
template<typename Network = UNetwork<>>
const int LinkPred::LINE< Network >::getDefaultDim ( ) const  [inline]
```

Returns

The default embedding dimension.

9.56.3.3 getInitLr()

```
template<typename Network = UNetwork<>>
float LinkPred::LINE< Network >::getInitLr ( ) const  [inline]
```

Returns

Initial learning rate.

9.56.3.4 getNbNegSamples()

```
template<typename Network = UNetwork<>>
int LinkPred::LINE< Network >::getNbNegSamples ( ) const  [inline]
```

Returns

Number of negative samples.

9.56.3.5 getNegSamplingPower()

```
template<typename Network = UNetwork<>>
float LinkPred::LINE< Network >::getNegSamplingPower ( ) const  [inline]
```

Parameters

Power	used in negative sampling.

9.56.3.6 getNegSize()

```
template<typename Network = UNetwork<>>
long long LinkPred::LINE< Network >::getNegSize ( ) const  [inline]
```

Returns

The size of the table used in negative sampling.

9.56.3.7 getOrder()

```
template<typename Network = UNetwork<>>
int LinkPred::LINE< Network >::getOrder ( ) const [inline]
```

Returns

The order of similarity. Possible values are: 1 (first order), 2 (second order), and 12 (concatenate first and second order). When order = 12, the embedding dimension is split between first order codes and second order codes.

9.56.3.8 getRecNet()

```
template<typename Network = UNetwork<>>
const std::shared_ptr<const Network> LinkPred::LINE< Network >::getRecNet ( ) const [inline]
```

Returns

The reconstructed network.

9.56.3.9 getRecWeightMap()

```
template<typename Network = UNetwork<>>
const WeightMapSP& LinkPred::LINE< Network >::getRecWeightMap ( ) const [inline]
```

Returns

The weight map of the reconstructed network.

9.56.3.10 init()

```
template<typename Network = UNetwork<>>
virtual void LinkPred::LINE< Network >::init ( ) [virtual]
```

Initialize encoder.

Implements LinkPred::Encoder< UNetwork<> >.

9.56.3.11 isEnableReconstruct()

```
template<typename Network = UNetwork<>>
bool LinkPred::LINE< Network >::isEnableReconstruct ( ) const [inline]
```

Returns

Whether the network is reconstructed (expanded).

9.56.3.12 operator=() [1/2]

```
template<typename Network = UNetwork<>>
LINE& LinkPred::LINE< Network >::operator= (
            LINE< Network > && that ) [default]
```

Move assignment operator.

Parameters

that	The object to move.

9.56.3.13 operator=() [2/2]

```
template<typename Network = UNetwork<>>
LINE& LinkPred::LINE< Network >::operator= (
            LINE< Network > const & that ) [default]
```

Copy assignment operator.

Parameters

that	The object to copy.

9.56.3.14 setEnableReconstruct()

```
template<typename Network = UNetwork<>>
void LinkPred::LINE< Network >::setEnableReconstruct (
            bool enableReconstruct ) [inline]
```

Set whether the network is reconstructed (expanded).

Parameters

enableReconstruct	Whether the network is reconstructed (expanded).

9.56.3.15 setInitLr()

```
template<typename Network = UNetwork<>>
void LinkPred::LINE< Network >::setInitLr (
            float initLR )  [inline]
```

Set the initial learning rate.

Parameters

initLR	The initial learning rate.

9.56.3.16 setNbNegSamples()

```
template<typename Network = UNetwork<>>
void LinkPred::LINE< Network >::setNbNegSamples (
            int nbNegSamples )  [inline]
```

Set the number of negative samples.

Parameters

nbNegSamples	The number of negative samples.

9.56.3.17 setNegSamplingPower()

```
template<typename Network = UNetwork<>>
void LinkPred::LINE< Network >::setNegSamplingPower (
            float negSamplingPower )  [inline]
```

Set the power used in negative sampling.

Parameters

negSamplingPower	Power used in negative sampling.

9.56.3.18 setNegSize()

```
template<typename Network = UNetwork<>>
void LinkPred::LINE< Network >::setNegSize (
             long long negSize ) [inline]
```

Set the size of the table used in negative sampling.

Parameters

negSize	Size of the table used in negative sampling.

9.56.3.19 setOrder()

```
template<typename Network = UNetwork<>>
void LinkPred::LINE< Network >::setOrder (
             int order ) [inline]
```

Set the order of similarity. Possible values are: 1 (first order), 2 (second order), and 12 (concatenate first and second order). When order = 12, the embedding dimension is split between first order codes and second order codes.

Parameters

order	Order of similarity.

The documentation for this class was generated from the following file:

- include/linkpred/graphalg/encoders/line/line.hpp

9.57 LinkPred::LMapQueue< K, P, KComparator, PComparator > Class Template Reference

A map-priority queue with limit on the capacity.

```
#include <lmapqueue.hpp>
```

Public Member Functions

- LMapQueue (std::size_t l)
- LMapQueue (LMapQueue const &that)=default
- LMapQueue & operator= (LMapQueue const &that)=default
- LMapQueue (LMapQueue &&that)=default
- LMapQueue & operator= (LMapQueue &&that)=default
- const std::pair< K, P > & top () const
- bool empty () const

- std::size_t size () const
- void printPQ ()
- bool push (std::pair< K, P > const &elem)
- bool push (K const &key, P const &pr)
- bool compareTop (P const &pr)
- P & at (K const &key)
- const P & at (K const &key) const
- void pop ()
- void pop (P const pr)
- auto begin ()
- auto begin () const
- auto cbegin () const
- auto end ()
- auto end () const
- auto cend () const
- std::size_t getL () const
- void clear ()
- std::size_t count (K const &key) const
- auto find (K const &key) const
- virtual ~LMapQueue ()=default

Static Public Member Functions

- template<typename RandomIterator >
  static LMapQueue< K, P, KComparator, PComparator > merge (std::size_t k, RandomIterator begin, RandomIterator end)

9.57.1 Detailed Description

**template<typename K, typename P, typename KComparator = std::less<K>, typename PComparator = std::greater<P>>
class LinkPred::LMapQueue< K, P, KComparator, PComparator >**

A map-priority queue with limit on the capacity.

Template Parameters

K	The key type.
P	The priority type.
KComparator	Key comparator.
PComparator	Priority comparator.

9.57.2 Constructor & Destructor Documentation

9.57.2.1 LMapQueue() [1/3]

```
template<typename K , typename P , typename KComparator = std::less<K>, typename PComparator
= std::greater<P>>
```

```
LinkPred::LMapQueue< K, P, KComparator, PComparator >::LMapQueue (
            std::size_t l ) [inline]
```

Constructor.

Parameters

l	The capacity limit of the container.

9.57.2.2 LMapQueue() [2/3]

```
template<typename K , typename P , typename KComparator = std::less<K>, typename PComparator
= std::greater<P>>
LinkPred::LMapQueue< K, P, KComparator, PComparator >::LMapQueue (
            LMapQueue< K, P, KComparator, PComparator > const & that ) [default]
```

Copy constructor.

Parameters

that	The object to copy.

9.57.2.3 LMapQueue() [3/3]

```
template<typename K , typename P , typename KComparator = std::less<K>, typename PComparator
= std::greater<P>>
LinkPred::LMapQueue< K, P, KComparator, PComparator >::LMapQueue (
            LMapQueue< K, P, KComparator, PComparator > && that ) [default]
```

Move constructor.

Parameters

that	The object to move.

9.57.2.4 ∼LMapQueue()

```
template<typename K , typename P , typename KComparator = std::less<K>, typename PComparator
= std::greater<P>>
virtual LinkPred::LMapQueue< K, P, KComparator, PComparator >::~LMapQueue ( ) [virtual],
[default]
```

Destructor.

9.57.3 Member Function Documentation

9.57.3.1 at() [1/2]

```
template<typename K , typename P , typename KComparator = std::less<K>, typename PComparator
= std::greater<P>>
P& LinkPred::LMapQueue< K, P, KComparator, PComparator >::at (
            K const & key )  [inline]
```

Returns a reference to the priority of the element with key equivalent to key. If no such element exists, an exception of type std::out_of_range is thrown.

Parameters

key	The key of the element to find.

Returns

Reference to the priority of the requested element

9.57.3.2 at() [2/2]

```
template<typename K , typename P , typename KComparator = std::less<K>, typename PComparator
= std::greater<P>>
const P& LinkPred::LMapQueue< K, P, KComparator, PComparator >::at (
            K const & key ) const  [inline]
```

Returns a reference to the priority of the element with key equivalent to key. If no such element exists, an exception of type std::out_of_range is thrown.

Parameters

key	The key of the element to find.

Returns

Reference to the priority of the requested element

9.57.3.3 begin() [1/2]

```
template<typename K , typename P , typename KComparator = std::less<K>, typename PComparator
= std::greater<P>>
auto LinkPred::LMapQueue< K, P, KComparator, PComparator >::begin ( )  [inline]
```

Returns

An iterator to the first element in the map.

9.57.3.4 begin() [2/2]

```
template<typename K , typename P , typename KComparator = std::less<K>, typename PComparator
= std::greater<P>>
auto LinkPred::LMapQueue< K, P, KComparator, PComparator >::begin ( ) const [inline]
```

Returns

A constant iterator to the first element in the map.

9.57.3.5 cbegin()

```
template<typename K , typename P , typename KComparator = std::less<K>, typename PComparator
= std::greater<P>>
auto LinkPred::LMapQueue< K, P, KComparator, PComparator >::cbegin ( ) const [inline]
```

Returns

A constant iterator to the first element in the map.

9.57.3.6 cend()

```
template<typename K , typename P , typename KComparator = std::less<K>, typename PComparator
= std::greater<P>>
auto LinkPred::LMapQueue< K, P, KComparator, PComparator >::cend ( ) const [inline]
```

Returns

A constant iterator to the one-past-the-last element in the map.

9.57.3.7 clear()

```
template<typename K , typename P , typename KComparator = std::less<K>, typename PComparator
= std::greater<P>>
void LinkPred::LMapQueue< K, P, KComparator, PComparator >::clear ( ) [inline]
```

Clear the content of the container.

9.57.3.8 compareTop()

```
template<typename K , typename P , typename KComparator = std::less<K>, typename PComparator
= std::greater<P>>
bool LinkPred::LMapQueue< K, P, KComparator, PComparator >::compareTop (
            P const & pr ) [inline]
```

Compares the input priority to the priority of the top element in the queue. The queue must not be empty. A true return value means that an element with priority pr will be inserted to the queue.

Parameters

pr	The priority to be compared.

Returns

True result of comparing the priority of top to pr.

9.57.3.9 count()

```
template<typename K , typename P , typename KComparator = std::less<K>, typename PComparator
= std::greater<P>>
std::size_t LinkPred::LMapQueue< K, P, KComparator, PComparator >::count (
            K const & key ) const  [inline]
```

Parameters

key	key value of the elements to count.

Returns

Number of elements with key that compares equivalent to key, which is either 1 or 0.

9.57.3.10 empty()

```
template<typename K , typename P , typename KComparator = std::less<K>, typename PComparator
= std::greater<P>>
bool LinkPred::LMapQueue< K, P, KComparator, PComparator >::empty ( ) const  [inline]
```

Checks whether the container is empty.

Returns

True if the container is empty, false otherwise.

9.57.3.11 end() [1/2]

```
template<typename K , typename P , typename KComparator = std::less<K>, typename PComparator
= std::greater<P>>
auto LinkPred::LMapQueue< K, P, KComparator, PComparator >::end ( )  [inline]
```

Returns

An iterator to the one-past-the-last element in the map.

9.57.3.12 end() [2/2]

```
template<typename K , typename P , typename KComparator = std::less<K>, typename PComparator
= std::greater<P>>
auto LinkPred::LMapQueue< K, P, KComparator, PComparator >::end ( ) const [inline]
```

Returns

A constant iterator to the one-past-the-last element in the map.

9.57.3.13 find()

```
template<typename K , typename P , typename KComparator = std::less<K>, typename PComparator
= std::greater<P>>
auto LinkPred::LMapQueue< K, P, KComparator, PComparator >::find (
            K const & key ) const [inline]
```

Parameters

key	value of the element to search for.

Returns

Iterator to an element with key equivalent to key. If no such element is found, past-the-end (see end()) iterator is returned.

9.57.3.14 getL()

```
template<typename K , typename P , typename KComparator = std::less<K>, typename PComparator
= std::greater<P>>
std::size_t LinkPred::LMapQueue< K, P, KComparator, PComparator >::getL ( ) const [inline]
```

Returns

The value of the limiting capacity.

9.57.3.15 merge()

```
template<typename K , typename P , typename KComparator = std::less<K>, typename PComparator
= std::greater<P>>
template<typename RandomIterator >
static LMapQueue<K, P, KComparator, PComparator> LinkPred::LMapQueue< K, P, KComparator, P←
Comparator >::merge (
            std::size_t k,
            RandomIterator begin,
            RandomIterator end ) [inline], [static]
```

Merge several queues by selecting at most the top k elements. The elements selected are unique. In case of duplication, the highest priority is considered. The input queues are emptied.

9.57.3.16 operator=() [1/2]

```
template<typename K , typename P , typename KComparator = std::less<K>, typename PComparator
= std::greater<P>>
LMapQueue& LinkPred::LMapQueue< K, P, KComparator, PComparator >::operator= (
            LMapQueue< K, P, KComparator, PComparator > && that )  [default]
```

Move assignment operator.

Parameters

that	The object to move.

9.57.3.17 operator=() [2/2]

```
template<typename K , typename P , typename KComparator = std::less<K>, typename PComparator
= std::greater<P>>
LMapQueue& LinkPred::LMapQueue< K, P, KComparator, PComparator >::operator= (
            LMapQueue< K, P, KComparator, PComparator > const & that )  [default]
```

Copy assignment operator.

Parameters

that	The object to copy.

9.57.3.18 pop() [1/2]

```
template<typename K , typename P , typename KComparator = std::less<K>, typename PComparator
= std::greater<P>>
void LinkPred::LMapQueue< K, P, KComparator, PComparator >::pop ( )  [inline]
```

Removes the top element from the priority queue.

9.57.3.19 pop() [2/2]

```
template<typename K , typename P , typename KComparator = std::less<K>, typename PComparator
= std::greater<P>>
void LinkPred::LMapQueue< K, P, KComparator, PComparator >::pop (
            P const pr )  [inline]
```

Removes all element with priority strictly less than the specified value.

Parameters

pr	The threshold priority.

9.57.3.20 printPQ()

```
template<typename K , typename P , typename KComparator = std::less<K>, typename PComparator
= std::greater<P>>
void LinkPred::LMapQueue< K, P, KComparator, PComparator >::printPQ ( )  [inline]
```

Print the content of the queue.

9.57.3.21 push() [1/2]

```
template<typename K , typename P , typename KComparator = std::less<K>, typename PComparator
= std::greater<P>>
bool LinkPred::LMapQueue< K, P, KComparator, PComparator >::push (
            K const & key,
            P const & pr )  [inline]
```

Insert an element into the map. Only the top l elements are kept.

Parameters

key	The key of the element top insert.
pr	The priority of the element.

Returns

True if the insertion takes place, false otherwise.

9.57.3.22 push() [2/2]

```
template<typename K , typename P , typename KComparator = std::less<K>, typename PComparator
= std::greater<P>>
bool LinkPred::LMapQueue< K, P, KComparator, PComparator >::push (
            std::pair< K, P > const & elem )  [inline]
```

Insert an element into the map. Only the top l elements are kept.

Parameters

elem	A pair where the first element is the key of the element top insert, and the second element is the priority of the element.

Returns

True if the insertion takes place, false otherwise.

9.57.3.23 size()

```
template<typename K , typename P , typename KComparator = std::less<K>, typename PComparator
= std::greater<P>>
std::size_t LinkPred::LMapQueue< K, P, KComparator, PComparator >::size ( ) const [inline]
```

Returns

The number of elements in the container.

9.57.3.24 top()

```
template<typename K , typename P , typename KComparator = std::less<K>, typename PComparator
= std::greater<P>>
const std::pair<K, P>& LinkPred::LMapQueue< K, P, KComparator, PComparator >::top ( ) const
[inline]
```

Returns

A reference to the element with the highest priority.

The documentation for this class was generated from the following file:

- include/linkpred/core/ds/lmapqueue.hpp

9.58 LinkPred::Log Class Reference

A log class.

```
#include <log.hpp>
```

Public Member Functions

- Log (LogLevel level=logError)
- template<typename T >
  Log & operator<< (T const &value)
- ∼Log ()

Static Public Attributes

- static constexpr std::ostream * DefaultOutputStream = &std::cerr
- static const LogLevel DefaultLogLevel = logError
- static std::ostream * out
- static LogLevel logLevel

9.58.1 Detailed Description

A log class.

9.58.2 Constructor & Destructor Documentation

9.58.2.1 Log()

```
LinkPred::Log::Log (
            LogLevel level = logError )  [inline]
```

Constructor.

9.58.2.2 ∼Log()

```
LinkPred::Log::∼Log ( )  [inline]
```

Destructor.

9.58.3 Member Function Documentation

9.58.3.1 operator<<()

```
template<typename T >
Log& LinkPred::Log::operator<< (
            T const & value )  [inline]
```

Copy constructor.

Parameters

value	Value to be logged.

9.58.4 Member Data Documentation

9.58.4.1 DefaultLogLevel

```
const LogLevel LinkPred::Log::DefaultLogLevel = logError  [static]
```

Default log level.

9.58.4.2 DefaultOutputStream

```
constexpr std::ostream* LinkPred::Log::DefaultOutputStream = &std::cerr  [static], [constexpr]
```

Default output stream.

9.58.4.3 logLevel

```
LogLevel LinkPred::Log::logLevel  [static]
```

Log level.

9.58.4.4 out

```
std::ostream* LinkPred::Log::out  [static]
```

Output file.

The documentation for this class was generated from the following file:

- include/linkpred/utils/log.hpp

9.59 LinkPred::LogisticRegresser< InRndIt, OutRndIt, ScoreRndIt > Class Template Reference

Logistic regression algorithm.

```
#include <logisticregresser.hpp>
```

Inheritance diagram for LinkPred::LogisticRegresser< InRndIt, OutRndIt, ScoreRndIt >:

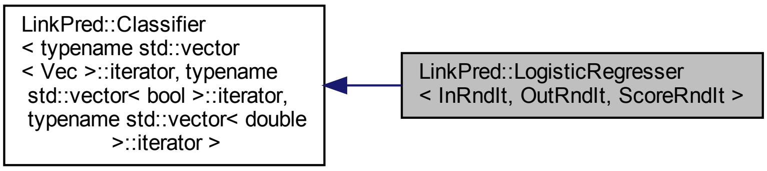

Collaboration diagram for LinkPred::LogisticRegresser< InRndIt, OutRndIt, ScoreRndIt >:

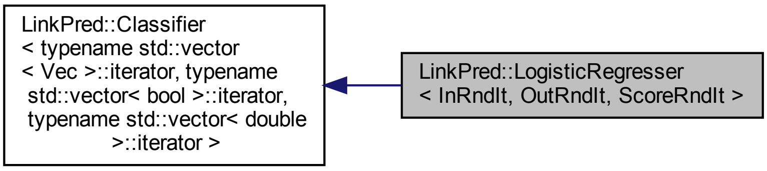

Public Member Functions

- LogisticRegresser (double lambda, long int seed)
- LogisticRegresser (LogisticRegresser const &that)=default
- LogisticRegresser & operator= (LogisticRegresser const &that)=default
- LogisticRegresser (LogisticRegresser &&that)=default
- LogisticRegresser & operator= (LogisticRegresser &&that)=default
- virtual void learn (InRndIt trInBegin, InRndIt trInEnd, OutRndIt trOutBegin, OutRndIt trOutEnd)
- virtual void predict (InRndIt inBegin, InRndIt inEnd, ScoreRndIt scoresBegin)
- double getLambda () const
- void setLambda (double lambda)
- double getTol () const
- void setTol (double tol)
- const Vec & getTheta () const
- virtual ∼LogisticRegresser ()=default

9.59.1 Detailed Description

template<typename InRndIt = typename std::vector<Vec>::iterator, typename OutRndIt = typename std::vector<bool>↩ ::iterator, typename ScoreRndIt = typename std::vector<double>::iterator>
class LinkPred::LogisticRegresser< InRndIt, OutRndIt, ScoreRndIt >

Logistic regression algorithm.

Template Parameters

InRndIt	Input (features) iterator type.
OutRndIt	Output (class) iterator type.
Score↩ RndIt	Classification scores iterator type.

9.59.2 Constructor & Destructor Documentation

9.59.2.1 LogisticRegresser() [1/3]

```
template<typename InRndIt = typename std::vector<Vec>::iterator, typename OutRndIt = typename
std::vector<bool>::iterator, typename ScoreRndIt = typename std::vector<double>::iterator>
LinkPred::LogisticRegresser< InRndIt, OutRndIt, ScoreRndIt >::LogisticRegresser (
            double lambda,
            long int seed )  [inline]
```

Constructor.

Parameters

lambda	Regularization coefficient.
seed	The random number generator's seed.

9.59.2.2 LogisticRegresser() [2/3]

```
template<typename InRndIt = typename std::vector<Vec>::iterator, typename OutRndIt = typename
std::vector<bool>::iterator, typename ScoreRndIt = typename std::vector<double>::iterator>
LinkPred::LogisticRegresser< InRndIt, OutRndIt, ScoreRndIt >::LogisticRegresser (
            LogisticRegresser< InRndIt, OutRndIt, ScoreRndIt > const & that )  [default]
```

Copy constructor.

Parameters

that	The object to copy.

9.59.2.3 LogisticRegresser() [3/3]

```
template<typename InRndIt = typename std::vector<Vec>::iterator, typename OutRndIt = typename
std::vector<bool>::iterator, typename ScoreRndIt = typename std::vector<double>::iterator>
LinkPred::LogisticRegresser< InRndIt, OutRndIt, ScoreRndIt >::LogisticRegresser (
            LogisticRegresser< InRndIt, OutRndIt, ScoreRndIt > && that )  [default]
```

Move constructor.

Parameters

that	The object to move.

9.59.2.4 ∼LogisticRegresser()

```
template<typename InRndIt = typename std::vector<Vec>::iterator, typename OutRndIt = typename
std::vector<bool>::iterator, typename ScoreRndIt = typename std::vector<double>::iterator>
virtual LinkPred::LogisticRegresser< InRndIt, OutRndIt, ScoreRndIt >::~LogisticRegresser ( )
[virtual], [default]
```

Destructor.

9.59.3 Member Function Documentation

9.59.3.1 getLambda()

```
template<typename InRndIt = typename std::vector<Vec>::iterator, typename OutRndIt = typename
std::vector<bool>::iterator, typename ScoreRndIt = typename std::vector<double>::iterator>
double LinkPred::LogisticRegresser< InRndIt, OutRndIt, ScoreRndIt >::getLambda ( ) const
[inline]
```

Returns

The regularization coefficient.

9.59.3.2 getTheta()

```
template<typename InRndIt = typename std::vector<Vec>::iterator, typename OutRndIt = typename
std::vector<bool>::iterator, typename ScoreRndIt = typename std::vector<double>::iterator>
const Vec& LinkPred::LogisticRegresser< InRndIt, OutRndIt, ScoreRndIt >::getTheta ( ) const
[inline]
```

Returns

The model parameters.

9.59.3.3 getTol()

```
template<typename InRndIt = typename std::vector<Vec>::iterator, typename OutRndIt = typename
std::vector<bool>::iterator, typename ScoreRndIt = typename std::vector<double>::iterator>
double LinkPred::LogisticRegresser< InRndIt, OutRndIt, ScoreRndIt >::getTol ( ) const  [inline]
```

Returns

The tolerance.

9.59.3.4 learn()

```
template<typename InRndIt = typename std::vector<Vec>::iterator, typename OutRndIt = typename
std::vector<bool>::iterator, typename ScoreRndIt = typename std::vector<double>::iterator>
virtual void LinkPred::LogisticRegresser< InRndIt, OutRndIt, ScoreRndIt >::learn (
            InRndIt trInBegin,
            InRndIt trInEnd,
            OutRndIt trOutBegin,
            OutRndIt trOutEnd )  [virtual]
```

Learn from data.

Parameters

trInBegin	Iterator to the first example features (input).
trInEnd	Iterator to one-past-the-last example features (input).
trOutBegin	Iterator to the first example class (output).
trOutEnd	Iterator to one-past-the-last example class (output).

9.59.3.5 operator=() [1/2]

```
template<typename InRndIt = typename std::vector<Vec>::iterator, typename OutRndIt = typename
std::vector<bool>::iterator, typename ScoreRndIt = typename std::vector<double>::iterator>
```

```
LogisticRegresser& LinkPred::LogisticRegresser< InRndIt, OutRndIt, ScoreRndIt >::operator= (
            LogisticRegresser< InRndIt, OutRndIt, ScoreRndIt > && that )  [default]
```

Move assignment operator.

Parameters

that	The object to move.

9.59.3.6 operator=() [2/2]

```
template<typename InRndIt = typename std::vector<Vec>::iterator, typename OutRndIt = typename
std::vector<bool>::iterator, typename ScoreRndIt = typename std::vector<double>::iterator>
LogisticRegresser& LinkPred::LogisticRegresser< InRndIt, OutRndIt, ScoreRndIt >::operator= (
            LogisticRegresser< InRndIt, OutRndIt, ScoreRndIt > const & that )  [default]
```

Copy assignment operator.

Parameters

that	The object to copy.

9.59.3.7 predict()

```
template<typename InRndIt = typename std::vector<Vec>::iterator, typename OutRndIt = typename
std::vector<bool>::iterator, typename ScoreRndIt = typename std::vector<double>::iterator>
virtual void LinkPred::LogisticRegresser< InRndIt, OutRndIt, ScoreRndIt >::predict (
            InRndIt inBegin,
            InRndIt inEnd,
            ScoreRndIt scoresBegin )  [virtual]
```

Predict.

Parameters

inBegin	Iterator to the first instance features (input).
inEnd	Iterator to one-past-the-last instance features (input).
scoresBegin	Iterator to the first location where to store prediction scores.

9.59.3.8 setLambda()

```
template<typename InRndIt = typename std::vector<Vec>::iterator, typename OutRndIt = typename
std::vector<bool>::iterator, typename ScoreRndIt = typename std::vector<double>::iterator>
```

```
void LinkPred::LogisticRegresser< InRndIt, OutRndIt, ScoreRndIt >::setLambda (
            double lambda )  [inline]
```

Set the regularization coefficient.

Parameters

lambda	The new regularization coefficient.

9.59.3.9 setTol()

```
template<typename InRndIt = typename std::vector<Vec>::iterator, typename OutRndIt = typename
std::vector<bool>::iterator, typename ScoreRndIt = typename std::vector<double>::iterator>
void LinkPred::LogisticRegresser< InRndIt, OutRndIt, ScoreRndIt >::setTol (
            double tol )  [inline]
```

Set the tolerance.

Parameters

tol	The new value of the tolerance.

The documentation for this class was generated from the following file:

- include/linkpred/ml/classifiers/logistic/logisticregresser.hpp

9.60 LinkPred::LogMDSCG Class Reference

Optimization problem associated with multidimensional scaling using the logarithmic (MULTISCALE) loss function.

```
#include <logmdscg.hpp>
```

Inheritance diagram for LinkPred::LogMDSCG:

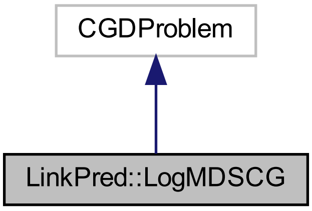

Collaboration diagram for LinkPred::LogMDSCG:

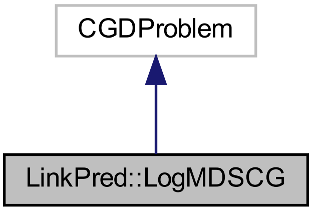

Public Member Functions

- LogMDSCG (std::size_t nbNodes, std::size_t nbKnownCouples, double ∗sqDist, double ∗weight, std::size_t dim, double ∗coords, long int seed)
- LogMDSCG (const LogMDSCG &that)=delete
- LogMDSCG & operator= (const LogMDSCG &that)=delete
- LogMDSCG (LogMDSCG &&that)=delete
- LogMDSCG & operator= (LogMDSCG &&that)=delete
- virtual void init (double ∗x, CG::INT n)
- virtual double f (double ∗x, CG::INT n)
- virtual void grad (double ∗grad_f, double ∗x, CG::INT n)
- virtual double fgrad (double ∗grad_f, double ∗x, CG::INT n)
- virtual void finalize (double ∗x, CG::INT n)
- virtual ∼LogMDSCG ()=default

9.60.1 Detailed Description

Optimization problem associated with multidimensional scaling using the logarithmic (MULTISCALE) loss function.

9.60.2 Constructor & Destructor Documentation

9.60.2.1 LogMDSCG() [1/3]

```
LinkPred::LogMDSCG::LogMDSCG (
            std::size_t nbNodes,
            std::size_t nbKnownCouples,
            double ∗ sqDist,
            double ∗ weight,
            std::size_t dim,
            double ∗ coords,
            long int seed )
```

Constructor.

Parameters

nbNodes	Number of nodes (or more generally, the number of data points).
nbKnownCouples	Number of couples for which the target distance is specified.
sqDist	Upper triangular matrix of squared distances without the diagonal stored sequentially as one vector.
weight	The weight associated with every distance (can be zero, if the distance is not relevant or unknown).
dim	The problem dimensionality.
coords	The coordinates (this is the solution to the problem).
seed	The random number generator's seed.

9.60.2.2 LogMDSCG() [2/3]

```
LinkPred::LogMDSCG::LogMDSCG (
            const LogMDSCG & that )  [delete]
```

Deleted copy constructor.

Parameters

that	The object to copy.

9.60.2.3 LogMDSCG() [3/3]

```
LinkPred::LogMDSCG::LogMDSCG (
            LogMDSCG && that )  [delete]
```

Deleted move constructor.

Parameters

that	The object to move.

9.60.2.4 ∼LogMDSCG()

```
virtual LinkPred::LogMDSCG::∼LogMDSCG ( )  [virtual], [default]
```

Default destructor.

9.60.3 Member Function Documentation

9.60.3.1 f()

```
virtual double LinkPred::LogMDSCG::f (
            double * x,
            CG::INT n ) [virtual]
```

Objective function.

Parameters

x	The variables.
n	The size.

Returns

The value of the objective.

9.60.3.2 fgrad()

```
virtual double LinkPred::LogMDSCG::fgrad (
            double * grad_f,
            double * x,
            CG::INT n ) [virtual]
```

Compute objective and gradient at the same time. This is a default implementation that should be overloaded if needed.

Parameters

grad_f	The gradient (output).
x	The variables.
n	The size.

Returns

The value of the objective.

9.60.3.3 finalize()

```
virtual void LinkPred::LogMDSCG::finalize (
            double * x,
            CG::INT n ) [virtual]
```

Finalize the solution.

Parameters

x	The variables.
n	The size.

9.60.3.4 grad()

```
virtual void LinkPred::LogMDSCG::grad (
            double * grad_f,
            double * x,
            CG::INT n ) [virtual]
```

Gradient.

Parameters

grad←_f	The gradient (output).
x	The variables.
n	The size.

9.60.3.5 init()

```
virtual void LinkPred::LogMDSCG::init (
            double * x,
            CG::INT n ) [virtual]
```

Initializes x.

Parameters

x	The variables.
n	The size.

9.60.3.6 operator=() [1/2]

```
LogMDSCG& LinkPred::LogMDSCG::operator= (
            const LogMDSCG & that ) [delete]
```

Deleted copy assignment operator.

Parameters

that	The object to copy.

9.60.3.7 operator=() [2/2]

```
LogMDSCG& LinkPred::LogMDSCG::operator= (
            LogMDSCG && that )  [delete]
```

Deleted move assignment operator.

Parameters

that	The object to move.

The documentation for this class was generated from the following file:

- include/linkpred/numerical/mds/logmdscg.hpp

9.61 LinkPred::LogRegCG< InRndIt, OutRndIt > Class Template Reference

Logistic regression optimization problem.

```
#include <logregcg.hpp>
```

Inheritance diagram for LinkPred::LogRegCG< InRndIt, OutRndIt >:

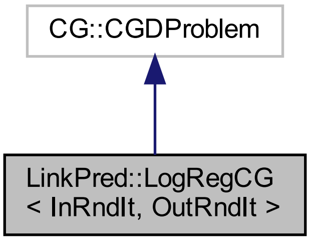

Collaboration diagram for LinkPred::LogRegCG< InRndIt, OutRndIt >:

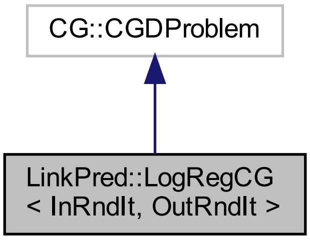

Public Member Functions

- LogRegCG (InRndIt trInBegin, InRndIt trInEnd, OutRndIt trOutBegin, OutRndIt trOutEnd, double lambda, long int seed)
- LogRegCG (const LogRegCG &that)=delete
- LogRegCG & operator= (const LogRegCG &that)=delete
- LogRegCG (LogRegCG &&that)=delete
- LogRegCG & operator= (LogRegCG &&that)=delete
- virtual void init (double ∗theta, CG::INT n)
- virtual double f (double ∗theta, CG::INT n)
- virtual void grad (double ∗grad_f, double ∗theta, CG::INT n)
- virtual double fgrad (double ∗grad_f, double ∗theta, CG::INT n)
- virtual void finalize (double ∗theta, CG::INT n)
- double getLambda () const
- void setLambda (double lambda)
- virtual ∼LogRegCG ()=default

9.61.1 Detailed Description

template<typename InRndIt = typename std::vector<Vec>::iterator, typename OutRndIt = typename std::vector<bool>↩ ::iterator>
class LinkPred::LogRegCG< InRndIt, OutRndIt >

Logistic regression optimization problem.

Template Parameters

InRndIt	Input (features) iterator type.
Out↩ RndIt	Output (class) iterator type.

9.61.2 Constructor & Destructor Documentation

9.61.2.1 LogRegCG() [1/3]

```
template<typename InRndIt = typename std::vector<Vec>::iterator, typename OutRndIt = typename
std::vector<bool>::iterator>
LinkPred::LogRegCG< InRndIt, OutRndIt >::LogRegCG (
            InRndIt trInBegin,
            InRndIt trInEnd,
            OutRndIt trOutBegin,
            OutRndIt trOutEnd,
            double lambda,
            long int seed )  [inline]
```

Constructor.

Parameters

trInBegin	Iterator to the first example features (input).
trInEnd	Iterator to one-past-the-last example features (input).
trOutBegin	Iterator to the first example class (output).
trOutEnd	Iterator to one-past-the-last example class (output).
lambda	Regularization coefficient.
seed	The random number generator's seed.

9.61.2.2 LogRegCG() [2/3]

```
template<typename InRndIt = typename std::vector<Vec>::iterator, typename OutRndIt = typename
std::vector<bool>::iterator>
LinkPred::LogRegCG< InRndIt, OutRndIt >::LogRegCG (
            const LogRegCG< InRndIt, OutRndIt > & that )  [delete]
```

Deleted copy constructor.

Parameters

that	The object to copy.

9.61.2.3 LogRegCG() [3/3]

```
template<typename InRndIt = typename std::vector<Vec>::iterator, typename OutRndIt = typename
std::vector<bool>::iterator>
```

```
LinkPred::LogRegCG< InRndIt, OutRndIt >::LogRegCG (
            LogRegCG< InRndIt, OutRndIt > && that )  [delete]
```

Deleted move constructor.

Parameters

that	The object to move.

9.61.2.4 ∼LogRegCG()

```
template<typename InRndIt = typename std::vector<Vec>::iterator, typename OutRndIt = typename
std::vector<bool>::iterator>
virtual LinkPred::LogRegCG< InRndIt, OutRndIt >::~LogRegCG ( )  [virtual], [default]
```

Destructor.

9.61.3 Member Function Documentation

9.61.3.1 f()

```
template<typename InRndIt = typename std::vector<Vec>::iterator, typename OutRndIt = typename
std::vector<bool>::iterator>
virtual double LinkPred::LogRegCG< InRndIt, OutRndIt >::f (
            double * theta,
            CG::INT n )  [virtual]
```

Objective function.

Parameters

theta	The variables.
n	The size.

Returns

The value of the objective.

9.61.3.2 fgrad()

```
template<typename InRndIt = typename std::vector<Vec>::iterator, typename OutRndIt = typename
std::vector<bool>::iterator>
```

```
virtual double LinkPred::LogRegCG< InRndIt, OutRndIt >::fgrad (
            double * grad_f,
            double * theta,
            CG::INT n ) [virtual]
```

Compute objective and gradient at the same time. This is a default implementation that should be overloaded if needed.

Parameters

grad_f	The gradient (output).
theta	The variables.
n	The size.

Returns

The value of the objective.

9.61.3.3 finalize()

```
template<typename InRndIt = typename std::vector<Vec>::iterator, typename OutRndIt = typename
std::vector<bool>::iterator>
virtual void LinkPred::LogRegCG< InRndIt, OutRndIt >::finalize (
            double * theta,
            CG::INT n ) [inline], [virtual]
```

Finalize the solution.

Parameters

theta	The variables.
n	The size.

9.61.3.4 getLambda()

```
template<typename InRndIt = typename std::vector<Vec>::iterator, typename OutRndIt = typename
std::vector<bool>::iterator>
double LinkPred::LogRegCG< InRndIt, OutRndIt >::getLambda ( ) const [inline]
```

Returns

The regularization coefficient.

9.61.3.5 grad()

```
template<typename InRndIt = typename std::vector<Vec>::iterator, typename OutRndIt = typename
std::vector<bool>::iterator>
virtual void LinkPred::LogRegCG< InRndIt, OutRndIt >::grad (
            double * grad_f,
            double * theta,
            CG::INT n )  [virtual]
```

Gradient.

Parameters

grad←_f	The gradient (output).
theta	The variables.
n	The size.

9.61.3.6 init()

```
template<typename InRndIt = typename std::vector<Vec>::iterator, typename OutRndIt = typename
std::vector<bool>::iterator>
virtual void LinkPred::LogRegCG< InRndIt, OutRndIt >::init (
            double * theta,
            CG::INT n )  [virtual]
```

Initializes theta.

Parameters

theta	The variables.
n	The size.

9.61.3.7 operator=() [1/2]

```
template<typename InRndIt = typename std::vector<Vec>::iterator, typename OutRndIt = typename
std::vector<bool>::iterator>
LogRegCG& LinkPred::LogRegCG< InRndIt, OutRndIt >::operator= (
            const LogRegCG< InRndIt, OutRndIt > & that )  [delete]
```

Deleted copy assignment operator.

Parameters

that	The object to copy.

9.61.3.8 operator=() [2/2]

```
template<typename InRndIt = typename std::vector<Vec>::iterator, typename OutRndIt = typename
std::vector<bool>::iterator>
LogRegCG& LinkPred::LogRegCG< InRndIt, OutRndIt >::operator= (
            LogRegCG< InRndIt, OutRndIt > && that )  [delete]
```

Deleted move assignment operator.

Parameters

that	The object to move.

9.61.3.9 setLambda()

```
template<typename InRndIt = typename std::vector<Vec>::iterator, typename OutRndIt = typename
std::vector<bool>::iterator>
void LinkPred::LogRegCG< InRndIt, OutRndIt >::setLambda (
            double lambda )  [inline]
```

Set the regularization coefficient.

Parameters

lambda	The new regularization coefficient.

The documentation for this class was generated from the following file:

- include/linkpred/ml/classifiers/logistic/logregcg.hpp

9.62 LinkPred::LPSim Class Reference

LP similarity (negative the Lp norm).

```
#include <lpsim.hpp>
```

Inheritance diagram for LinkPred::LPSim:

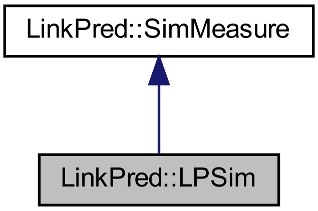

Collaboration diagram for LinkPred::LPSim:

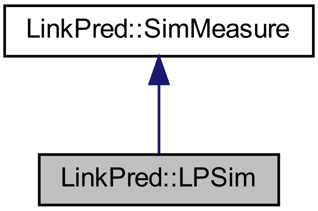

Public Member Functions

- LPSim (double p)
- LPSim (LPSim const &that)=default
- LPSim & operator= (LPSim const &that)=default
- LPSim (LPSim &&that)=default
- LPSim & operator= (LPSim &&that)=default
- virtual double sim (Vec const &v1, Vec const &v2)
- double getP () const
- void setP (double p)
- virtual ∼LPSim ()=default

9.62.1 Detailed Description

LP similarity (negative the Lp norm).

9.62.2 Constructor & Destructor Documentation

9.62.2.1 LPSim() [1/3]

```
LinkPred::LPSim::LPSim (
            double p ) [inline]
```

Constructor.

Parameters

p	The p of the norm.

9.62.2.2 LPSim() [2/3]

```
LinkPred::LPSim::LPSim (
            LPSim const & that ) [default]
```

Copy constructor.

Parameters

that	The object to copy.

9.62.2.3 LPSim() [3/3]

```
LinkPred::LPSim::LPSim (
            LPSim && that ) [default]
```

Move constructor.

Parameters

that	The object to move.

9.62.2.4 ∼LPSim()

```
virtual LinkPred::LPSim::∼LPSim ( ) [virtual], [default]
```

Destructor.

9.62.3 Member Function Documentation

9.62.3.1 getP()

```
double LinkPred::LPSim::getP ( ) const  [inline]
```

Returns

The power p of the norm.

9.62.3.2 operator=() [1/2]

```
LPSim& LinkPred::LPSim::operator= (
            LPSim && that )  [default]
```

Move assignment operator.

Parameters

that	The object to move.

9.62.3.3 operator=() [2/2]

```
LPSim& LinkPred::LPSim::operator= (
            LPSim const & that )  [default]
```

Copy assignment operator.

Parameters

that	The object to copy.

9.62.3.4 setP()

```
void LinkPred::LPSim::setP (
            double p )  [inline]
```

Set the power p of the norm.

Parameters

p	The power p of the norm.

9.62.3.5 sim()

```
virtual double LinkPred::LPSim::sim (
            Vec const & v1,
            Vec const & v2 )  [virtual]
```

Compute the similarity between two vectors.

Parameters

v1	First vector.
v2	Second vector. Must be of the same dimension as v1.

Returns

The similarity between v1 and v2.

Implements LinkPred::SimMeasure.

The documentation for this class was generated from the following file:

- include/linkpred/ml/simmeasures/lpsim.hpp

9.63 LinkPred::MatFact< Network > Class Template Reference

Contains the implementation of an algorithm for embedding a network using matrix factorization. Reference: Koren, Y., Bell, R., and Volinsky, C. (2009). Matrix factorization techniques for recommender systems. Computer, 42(8)↩ :30–37 Ahmed, A., Shervashidze, N., Narayanamurthy, S., Josifovski, V., and Smola, A. J. (2013). Distributed large-scale natural graph factorization. In Proceedings of the 22nd International Conference on World Wide Web, WWW ’13, pages 37–48, New York, NY, USA. Association for Computing Machinery.

```
#include <matfact.hpp>
```

Inheritance diagram for LinkPred::MatFact< Network >:

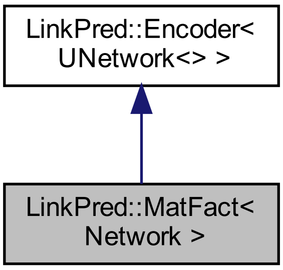

Collaboration diagram for LinkPred::MatFact< Network >:

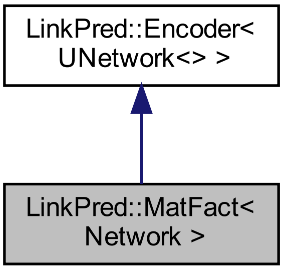

Public Member Functions

- MatFact (std::shared_ptr< Network const > net, long int seed)
- MatFact (MatFact const &that)=default
- MatFact & operator= (MatFact const &that)=default
- MatFact (MatFact &&that)=default
- MatFact & operator= (MatFact &&that)=default
- virtual void init ()
- virtual void encode ()
- const int getDefaultDim () const
- double getLambda () const
- void setLambda (double lambda)
- double getNegRatio () const
- void setNegRatio (double negRatio)
- double getPosRatio () const
- void setPosRatio (double posRatio)
- double getTol () const
- void setTol (double tol)
- virtual void setWeightMap (const WeightMapSP &weightMap)
- virtual ∼MatFact ()=default

Additional Inherited Members

9.63.1 Detailed Description

template<typename Network = UNetwork<>>
class LinkPred::MatFact< Network >

Contains the implementation of an algorithm for embedding a network using matrix factorization. Reference: Koren, Y., Bell, R., and Volinsky, C. (2009). Matrix factorization techniques for recommender systems. Computer, 42(8)↩ :30–37 Ahmed, A., Shervashidze, N., Narayanamurthy, S., Josifovski, V., and Smola, A. J. (2013). Distributed large-scale natural graph factorization. In Proceedings of the 22nd International Conference on World Wide Web, WWW '13, pages 37–48, New York, NY, USA. Association for Computing Machinery.

Template Parameters

Network	The network type.

9.63.2 Constructor & Destructor Documentation

9.63.2.1 MatFact() [1/3]

```
template<typename Network = UNetwork<>>
LinkPred::MatFact< Network >::MatFact (
            std::shared_ptr< Network const > net,
            long int seed )  [inline]
```

Constructor.

Parameters

net	The network.
seed	Random number generator seed.

9.63.2.2 MatFact() [2/3]

```
template<typename Network = UNetwork<>>
LinkPred::MatFact< Network >::MatFact (
            MatFact< Network > const & that )  [default]
```

Copy constructor.

Parameters

that	The object to copy.

9.63.2.3 MatFact() [3/3]

```
template<typename Network = UNetwork<>>
LinkPred::MatFact< Network >::MatFact (
            MatFact< Network > && that )  [default]
```

Move constructor.

Parameters

that	The object to move.

9.63.2.4 ~MatFact()

```
template<typename Network = UNetwork<>>
virtual LinkPred::MatFact< Network >::~MatFact ( )  [virtual], [default]
```

Destructor.

9.63.3 Member Function Documentation

9.63.3.1 encode()

```
template<typename Network = UNetwork<>>
virtual void LinkPred::MatFact< Network >::encode ( )  [virtual]
```

Encode the network.

Implements LinkPred::Encoder< UNetwork<> >.

9.63.3.2 getDefaultDim()

```
template<typename Network = UNetwork<>>
const int LinkPred::MatFact< Network >::getDefaultDim ( ) const  [inline]
```

Returns

The default embedding dimension.

9.63.3.3 getLambda()

```
template<typename Network = UNetwork<>>
double LinkPred::MatFact< Network >::getLambda ( ) const  [inline]
```

Returns

The regularization coeffcient.

9.63.3.4 getNegRatio()

```
template<typename Network = UNetwork<>>
double LinkPred::MatFact< Network >::getNegRatio ( ) const  [inline]
```

Returns

The ratio of negative edges used for fitting the model.

9.63.3.5 getPosRatio()

```
template<typename Network = UNetwork<>>
double LinkPred::MatFact< Network >::getPosRatio ( ) const  [inline]
```

Returns

The ratio of positive edges used for fitting the model.

9.63.3.6 getTol()

```
template<typename Network = UNetwork<>>
double LinkPred::MatFact< Network >::getTol ( ) const  [inline]
```

Returns

The tolerance for stopping the optimization.

9.63.3.7 init()

```
template<typename Network = UNetwork<>>
virtual void LinkPred::MatFact< Network >::init ( )  [virtual]
```

Initialize encoder.

Implements LinkPred::Encoder< UNetwork<> >.

9.63.3.8 operator=() [1/2]

```
template<typename Network = UNetwork<>>
MatFact& LinkPred::MatFact< Network >::operator= (
            MatFact< Network > && that )  [default]
```

Move assignment operator.

Parameters

that	The object to move.

9.63.3.9 operator=() [2/2]

```
template<typename Network = UNetwork<>>
MatFact& LinkPred::MatFact< Network >::operator= (
            MatFact< Network > const & that )  [default]
```

Copy assignment operator.

Parameters

that	The object to copy.

9.63.3.10 setLambda()

```
template<typename Network = UNetwork<>>
void LinkPred::MatFact< Network >::setLambda (
            double lambda )  [inline]
```

Set the regularization coeffcient.

Parameters

lambda	The regularization coeffcient.

9.63.3.11 setNegRatio()

```
template<typename Network = UNetwork<>>
void LinkPred::MatFact< Network >::setNegRatio (
            double negRatio )  [inline]
```

Set the ratio of negative edges used for fitting the model.

Parameters

negRatio	The ratio of negative edges used for fitting the model.

9.63.3.12 setPosRatio()

```
template<typename Network = UNetwork<>>
void LinkPred::MatFact< Network >::setPosRatio (
             double posRatio )  [inline]
```

Set the ratio of positive edges used for fitting the model.

Parameters

posRatio	The ratio of positive edges used for fitting the model.

9.63.3.13 setTol()

```
template<typename Network = UNetwork<>>
void LinkPred::MatFact< Network >::setTol (
             double tol )  [inline]
```

Set the tolerance for stopping the optimization.

Parameters

tol	The tolerance for stopping the optimization.

9.63.3.14 setWeightMap()

```
template<typename Network = UNetwork<>>
virtual void LinkPred::MatFact< Network >::setWeightMap (
             const WeightMapSP & weightMap )  [inline], [virtual]
```

Set the edge weight map.

Parameters

weightMap	The edge weight map.

Reimplemented from LinkPred::Encoder< UNetwork<> >.

The documentation for this class was generated from the following file:

- include/linkpred/graphalg/encoders/matfact/matfact.hpp

9.64 LinkPred::MatFactCG Class Reference

Optimization problem associated with matrix factorization.

`#include <matfactcg.hpp>`

Inheritance diagram for LinkPred::MatFactCG:

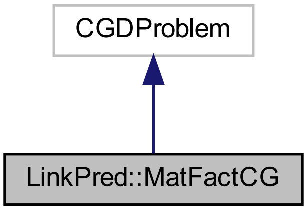

Collaboration diagram for LinkPred::MatFactCG:

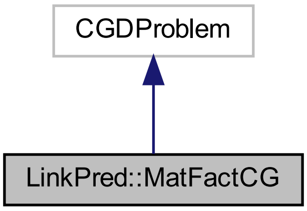

Public Member Functions

- MatFactCG (long int nbNodes, std::vector< MatFactPbData > const &pbData, int dim, double lambda, long int seed)
- MatFactCG (const MatFactCG &that)=delete
- MatFactCG & operator= (const MatFactCG &that)=delete
- MatFactCG (MatFactCG &&that)=delete
- MatFactCG & operator= (MatFactCG &&that)=delete
- virtual void init (double ∗x, CG::INT n)
- virtual double f (double ∗x, CG::INT n)
- virtual void grad (double ∗grad_f, double ∗x, CG::INT n)
- virtual double fgrad (double ∗grad_f, double ∗x, CG::INT n)
- virtual void finalize (double ∗x, CG::INT n)
- virtual ∼MatFactCG ()=default

9.64.1 Detailed Description

Optimization problem associated with matrix factorization.

9.64.2 Constructor & Destructor Documentation

9.64.2.1 MatFactCG() [1/3]

```
LinkPred::MatFactCG::MatFactCG (
            long int nbNodes,
            std::vector< MatFactPbData > const & pbData,
            int dim,
            double lambda,
            long int seed )
```

Constructor.

Parameters

nbNodes	The number of nodes in the network.
pbData	The problem data.
dim	The problem dimensionality.
lambda	Regularization coeffcient.
seed	The random number generator's seed.

9.64.2.2 MatFactCG() [2/3]

```
LinkPred::MatFactCG::MatFactCG (
            const MatFactCG & that )  [delete]
```

Deleted copy constructor.

Parameters

that	The object to copy.

9.64.2.3 MatFactCG() [3/3]

```
LinkPred::MatFactCG::MatFactCG (
            MatFactCG && that )  [delete]
```

Deleted move constructor.

Parameters

that	The object to move.

9.64.2.4 ∼MatFactCG()

```
virtual LinkPred::MatFactCG::∼MatFactCG ( )  [virtual], [default]
```

Default destructor.

9.64.3 Member Function Documentation

9.64.3.1 f()

```
virtual double LinkPred::MatFactCG::f (
            double * x,
            CG::INT n )  [virtual]
```

Objective function.

Parameters

x	The variables.
n	The size.

Returns

The value of the objective.

9.64.3.2 fgrad()

```
virtual double LinkPred::MatFactCG::fgrad (
            double * grad_f,
            double * x,
            CG::INT n )  [virtual]
```

Compute objective and gradient at the same time. This is a default implementation that should be overloaded if needed.

Parameters

grad_f	The gradient (output).
x	The variables.
n	The size.

Returns

The value of the objective.

9.64.3.3 finalize()

```
virtual void LinkPred::MatFactCG::finalize (
            double * x,
            CG::INT n ) [virtual]
```

Finalize the solution.

Parameters

x	The variables.
n	The size.

9.64.3.4 grad()

```
virtual void LinkPred::MatFactCG::grad (
            double * grad_f,
            double * x,
            CG::INT n ) [virtual]
```

Gradient.

Parameters

grad_f	The gradient (output).
x	The variables.
n	The size.

9.64.3.5 init()

```
virtual void LinkPred::MatFactCG::init (
```

```
            double * x,
            CG::INT n )  [virtual]
```

Initializes x.

Parameters

x	The variables.
n	The size.

9.64.3.6 operator=() [1/2]

```
MatFactCG& LinkPred::MatFactCG::operator= (
            const MatFactCG & that )  [delete]
```

Deleted copy assignment operator.

Parameters

that	The object to copy.

9.64.3.7 operator=() [2/2]

```
MatFactCG& LinkPred::MatFactCG::operator= (
            MatFactCG && that )  [delete]
```

Deleted move assignment operator.

Parameters

that	The object to move.

The documentation for this class was generated from the following file:

- include/linkpred/graphalg/encoders/matfact/matfactcg.hpp

9.65 LinkPred::MatFactPbData Struct Reference

A simple structure to store matrix factoization problem data.

```
#include <matfactcg.hpp>
```

Public Attributes

- long int i
- long int j
- double target

9.65.1 Detailed Description

A simple structure to store matrix factoization problem data.

9.65.2 Member Data Documentation

9.65.2.1 i

`long int LinkPred::MatFactPbData::i`

Start node.

9.65.2.2 j

`long int LinkPred::MatFactPbData::j`

End node.

9.65.2.3 target

`double LinkPred::MatFactPbData::target`

Target value (0/1 for unweighted graphs.

The documentation for this struct was generated from the following file:

- include/linkpred/graphalg/encoders/matfact/matfactcg.hpp

9.66 LinkPred::MDS Class Reference

Solve the MDS problem.

`#include <mds.hpp>`

Public Member Functions

- MDS ()=default
- MDS (MDSAlg alg, double tol, std::size_t nbRuns)
- MDSAlg getAlg ()
- void setAlg (MDSAlg alg)
- double getTol ()
- void setTol (double tol)
- std::size_t getNbRuns ()
- void setNbRuns (std::size_t nbRuns)
- double solve (std::size_t nbNodes, std::size_t nbKnownCouples, double ∗sqDist, double ∗weight, std::size_t dim, double ∗coord, long int seed, bool init=true)
- double cgMDS (std::size_t nbNodes, std::size_t nbKnownCouples, std::size_t dim, double ∗sqDist, double ∗weight, double ∗coord, long int seed, bool init)
- virtual ∼MDS ()=default

9.66.1 Detailed Description

Solve the MDS problem.

9.66.2 Constructor & Destructor Documentation

9.66.2.1 MDS() [1/2]

```
LinkPred::MDS::MDS ( )  [default]
```

Constructor.

9.66.2.2 MDS() [2/2]

```
LinkPred::MDS::MDS (
            MDSAlg alg,
            double tol,
            std::size_t nbRuns )  [inline]
```

Constructor.

Parameters

alg	The algorithm used to solve the MDS problem.
tol	The tolerance.
nbRuns	The number of times multidimensional scaling is run using different random initial positions.

9.66.2.3 ∼MDS()

```
virtual LinkPred::MDS::~MDS ( ) [virtual], [default]
```

Destructor.

9.66.3 Member Function Documentation

9.66.3.1 cgMDS()

```
double LinkPred::MDS::cgMDS (
            std::size_t nbNodes,
            std::size_t nbKnownCouples,
            std::size_t dim,
            double * sqDist,
            double * weight,
            double * coord,
            long int seed,
            bool init )
```

MDS using CG.

Parameters

nbNodes	Number of nodes (or more generally, the number of data points).
nbKnownCouples	Number of couples for which the target distance is specified.
sqDist	Upper triangular matrix of squared distances without the diagonal stored sequentially as one vector.
weight	The weight associated with every distance (can be zero, if the distance is not relevant or unknown).
dim	The problem dimensionality.
coord	The coordinates (this is the solution to the problem).
seed	The random number generator's seed.
init	Whether to initialize points randomly or use the current values of the coordinates as the initial positions of the data points.

Returns

The objective function value.

9.66.3.2 getAlg()

```
MDSAlg LinkPred::MDS::getAlg ( ) [inline]
```

Returns

The algorithm used to solve the MDS problem.

9.66.3.3 getNbRuns()

```
std::size_t LinkPred::MDS::getNbRuns ( ) [inline]
```

Returns

The number of times multidimensional scaling is run using different random initial positions.

9.66.3.4 getTol()

```
double LinkPred::MDS::getTol ( ) [inline]
```

Returns

The tolerance.

9.66.3.5 setAlg()

```
void LinkPred::MDS::setAlg (
            MDSAlg alg ) [inline]
```

Set the MDS algorithm.

Parameters

alg	The algorithm used to solve the MDS problem.

9.66.3.6 setNbRuns()

```
void LinkPred::MDS::setNbRuns (
            std::size_t nbRuns ) [inline]
```

Set the number of runs.

Parameters

nbRuns	The new value of the number of runs.

9.66.3.7 setTol()

```
void LinkPred::MDS::setTol (
            double tol )  [inline]
```

Set the tolerance.

Parameters

tol	The new value of the tolerance.

9.66.3.8 solve()

```
double LinkPred::MDS::solve (
            std::size_t nbNodes,
            std::size_t nbKnownCouples,
            double * sqDist,
            double * weight,
            std::size_t dim,
            double * coord,
            long int seed,
            bool init = true )
```

Solve the MDS problem.

Parameters

nbNodes	Number of nodes (or more generally, the number of data points).
nbKnownCouples	Number of couples for which the target distance is specified.
sqDist	Upper triangular matrix of squared distances without the diagonal stored sequentially as one vector.
weight	The weight associated with every distance (can be zero, if the distance is not relevant or unknown).
dim	The problem dimensionality.
coord	The coordinates (this is the solution to the problem).
seed	The random number generator's seed.
init	Whether to initialize points randomly or use the current values of the coordinates as the initial positions of the data points.

Returns

The objective function value.

The documentation for this class was generated from the following file:

- include/linkpred/numerical/mds/mds.hpp

9.67 LinkPred::NetDistCalculator< Network, DistType, NbHopsType > Class Template Reference

Interface for calculating the distance between nodes in a network.

`#include <netdistcalculator.hpp>`

Public Types

- using LengthMapIdType = long int
- using NetworkSP = std::shared_ptr< Network >
- using NodeID = typename Network::NodeID
- using Label = typename Network::Label
- using Edge = typename Network::Edge
- using NodeDistMap = typename Network::template NodeMap< std::pair< DistType, NbHopsType > >
- using NodeDistMapSP = typename Network::template NodeMapSP< std::pair< DistType, NbHopsType > >
- using EdgeLengthMap = typename Network::template EdgeMap< DistType >
- using EdgeLengthMapSP = typename Network::template EdgeMapSP< DistType >

Public Member Functions

- virtual std::pair< DistType, NbHopsType > getDist (NodeID const &i, NodeID const &j)=0
- virtual std::pair< DistType, NbHopsType > getIndDist (NodeID const &i, NodeID const &j)=0
- virtual NodeDistMapSP getDist (NodeID const &i)=0
- virtual std::pair< DistType, NbHopsType > getMaxDist (NodeID const &i)
- double getDiscDist () const
- void setDiscDist (DistType discDist)
- std::size_t getDiscNbHops () const
- void setDiscNbHops (NbHopsType discNbHops)
- virtual ~NetDistCalculator ()=default

9.67.1 Detailed Description

template<typename Network, typename DistType, typename NbHopsType>
class LinkPred::NetDistCalculator< Network, DistType, NbHopsType >

Interface for calculating the distance between nodes in a network.

Template Parameters

Network	The network type.
DistType	The distance type (can be an integer or floating point type).
NbHopsType	The type of the number of hops (must be an integer type).

9.67.2 Member Typedef Documentation

9.67.2.1 Edge

```
template<typename Network , typename DistType , typename NbHopsType >
using LinkPred::NetDistCalculator< Network, DistType, NbHopsType >::Edge = typename Network↩
::Edge
```

Edge type.

9.67.2.2 EdgeLengthMap

```
template<typename Network , typename DistType , typename NbHopsType >
using LinkPred::NetDistCalculator< Network, DistType, NbHopsType >::EdgeLengthMap = typename
Network::template EdgeMap<DistType>
```

Edge length map.

9.67.2.3 EdgeLengthMapSP

```
template<typename Network , typename DistType , typename NbHopsType >
using LinkPred::NetDistCalculator< Network, DistType, NbHopsType >::EdgeLengthMapSP = typename
Network::template EdgeMapSP<DistType>
```

Shared pointer to an edge length map.

9.67.2.4 Label

```
template<typename Network , typename DistType , typename NbHopsType >
using LinkPred::NetDistCalculator< Network, DistType, NbHopsType >::Label = typename Network↩
::Label
```

Nodes label type.

9.67.2.5 LengthMapIdType

```
template<typename Network , typename DistType , typename NbHopsType >
using LinkPred::NetDistCalculator< Network, DistType, NbHopsType >::LengthMapIdType = long int
```

Length map ID type.

9.67.2.6 NetworkSP

```
template<typename Network , typename DistType , typename NbHopsType >
using LinkPred::NetDistCalculator< Network, DistType, NbHopsType >::NetworkSP = std::shared_↩
ptr<Network>
```

Shared pointer to network.

9.67.2.7 NodeDistMap

```
template<typename Network , typename DistType , typename NbHopsType >
using LinkPred::NetDistCalculator< Network, DistType, NbHopsType >::NodeDistMap = typename
Network::template NodeMap<std::pair<DistType, NbHopsType> >
```

Distance map.

9.67.2.8 NodeDistMapSP

```
template<typename Network , typename DistType , typename NbHopsType >
using LinkPred::NetDistCalculator< Network, DistType, NbHopsType >::NodeDistMapSP = typename
Network::template NodeMapSP<std::pair<DistType, NbHopsType> >
```

Shared pointer to a distance map.

9.67.2.9 NodeID

```
template<typename Network , typename DistType , typename NbHopsType >
using LinkPred::NetDistCalculator< Network, DistType, NbHopsType >::NodeID = typename Network↩
::NodeID
```

Nodes ID type.

9.67.3 Constructor & Destructor Documentation

9.67.3.1 ∼NetDistCalculator()

```
template<typename Network , typename DistType , typename NbHopsType >
virtual LinkPred::NetDistCalculator< Network, DistType, NbHopsType >::~NetDistCalculator ( )
[virtual], [default]
```

Destructor.

9.67.4 Member Function Documentation

9.67.4.1 getDiscDist()

```
template<typename Network , typename DistType , typename NbHopsType >
double LinkPred::NetDistCalculator< Network, DistType, NbHopsType >::getDiscDist ( ) const
[inline]
```

Returns

The distance assigned to disconnected nodes.

9.67.4.2 getDiscNbHops()

```
template<typename Network , typename DistType , typename NbHopsType >
std::size_t LinkPred::NetDistCalculator< Network, DistType, NbHopsType >::getDiscNbHops ( )
const [inline]
```

Returns

The value that should be assigned as number of hops between disconnected nodes.

9.67.4.3 getDist() [1/2]

```
template<typename Network , typename DistType , typename NbHopsType >
virtual NodeDistMapSP LinkPred::NetDistCalculator< Network, DistType, NbHopsType >::getDist (
            NodeID const & i ) [pure virtual]
```

Parameters

i	Source node.

Returns

The distance from i to all other nodes.

Implemented in LinkPred::ESPLDistCalculator< UNetwork<>, double, std::size_t >.

9.67.4.4 getDist() [2/2]

```
template<typename Network , typename DistType , typename NbHopsType >
virtual std::pair<DistType, NbHopsType> LinkPred::NetDistCalculator< Network, DistType, Nb←
HopsType >::getDist (
            NodeID const & i,
            NodeID const & j ) [pure virtual]
```

Parameters

i	Index of the source node.
j	Index of the end node.

Returns

The distance between i and j.

Implemented in LinkPred::ESPLDistCalculator< UNetwork<>, double, std::size_t >.

9.67.4.5 getIndDist()

```
template<typename Network , typename DistType , typename NbHopsType >
virtual std::pair<DistType, NbHopsType> LinkPred::NetDistCalculator< Network, DistType, Nb↩
HopsType >::getIndDist (
            NodeID const & i,
            NodeID const & j )  [pure virtual]
```

Parameters

i	Index of the source node.
j	Index of the end node.

Returns

The distance between i and j ignoring the edge between i and j.

Implemented in LinkPred::ESPLDistCalculator< UNetwork<>, double, std::size_t >.

9.67.4.6 getMaxDist()

```
template<typename Network , typename DistType , typename NbHopsType >
virtual std::pair<DistType, NbHopsType> LinkPred::NetDistCalculator< Network, DistType, Nb↩
HopsType >::getMaxDist (
            NodeID const & i )  [inline], [virtual]
```

Parameters

i	Index of the source node.

Returns

The maximum distance from i to any other node in the same connected component.

9.67.4.7 setDiscDist()

```
template<typename Network , typename DistType , typename NbHopsType >
void LinkPred::NetDistCalculator< Network, DistType, NbHopsType >::setDiscDist (
            DistType discDist )  [inline]
```

Set the distance assigned to disconnected nodes.

Parameters

discDist	The distance assigned to disconnected nodes.

9.67.4.8 setDiscNbHops()

```
template<typename Network , typename DistType , typename NbHopsType >
void LinkPred::NetDistCalculator< Network, DistType, NbHopsType >::setDiscNbHops (
            NbHopsType discNbHops )  [inline]
```

Set the value that should be assigned as number of hops between disconnected nodes.

Parameters

discNbHops	The value that should be assigned as number of hops between disconnected nodes.

The documentation for this class was generated from the following file:

- include/linkpred/graphalg/shortestpaths/netdistcalculator.hpp

9.68 LinkPred::NetIndSimlCalculator< Network, DistType, NbHopsType > Class Template Reference

Interface for calculating the indirect similarity between nodes in a network.

`#include <netdistcalculator.hpp>`

Public Types

- using LengthMapIdType = long int
- using NetworkSP = std::shared_ptr< Network >
- using NodeID = typename Network::NodeID
- using Label = typename Network::Label
- using Edge = typename Network::Edge
- using EdgeLengthMap = typename Network::template EdgeMap< DistType >
- using EdgeLengthMapSP = typename Network::template EdgeMapSP< DistType >

Public Member Functions

- virtual std::tuple< DistType, DistType, NbHopsType > getIndSiml (NodeID const &i, NodeID const &j)=0
- virtual std::tuple< DistType, DistType, NbHopsType > getDirIndSiml (NodeID const &i, NodeID const &j)=0
- double getSelfSiml () const
- void setSelfSiml (DistType selfSiml)
- double getDiscDist () const
- void setDiscDist (DistType discDist)
- std::size_t getDiscNbHops () const
- void setDiscNbHops (NbHopsType discNbHops)
- double getLambda () const
- void setLambda (double lambda)
- virtual ~NetIndSimlCalculator ()=default

9.68.1 Detailed Description

template<typename Network, typename DistType, typename NbHopsType>
class LinkPred::NetIndSimlCalculator< Network, DistType, NbHopsType >

Interface for calculating the indirect similarity between nodes in a network.

Template Parameters

Network	The network type.
DistType	The distance and similarity type (can be an integer or floating point type).
NbHopsType	The type of the number of hops (must be an integer type).

9.68.2 Member Typedef Documentation

9.68.2.1 Edge

```
template<typename Network , typename DistType , typename NbHopsType >
using LinkPred::NetIndSimlCalculator< Network, DistType, NbHopsType >::Edge = typename Network↩
::Edge
```

Edge type.

9.68.2.2 EdgeLengthMap

```
template<typename Network , typename DistType , typename NbHopsType >
using LinkPred::NetIndSimlCalculator< Network, DistType, NbHopsType >::EdgeLengthMap = typename
Network::template EdgeMap<DistType>
```

Edge length map.

9.68.2.3 EdgeLengthMapSP

```
template<typename Network , typename DistType , typename NbHopsType >
using LinkPred::NetIndSimlCalculator< Network, DistType, NbHopsType >::EdgeLengthMapSP =
typename Network::template EdgeMapSP<DistType>
```

Shared pointer to an edge length map.

9.68.2.4 Label

```
template<typename Network , typename DistType , typename NbHopsType >
using LinkPred::NetIndSimlCalculator< Network, DistType, NbHopsType >::Label = typename Network↩
::Label
```

Nodes label type.

9.68.2.5 LengthMapIdType

```
template<typename Network , typename DistType , typename NbHopsType >
using LinkPred::NetIndSimlCalculator< Network, DistType, NbHopsType >::LengthMapIdType = long
int
```

Length map ID type.

9.68.2.6 NetworkSP

```
template<typename Network , typename DistType , typename NbHopsType >
using LinkPred::NetIndSimlCalculator< Network, DistType, NbHopsType >::NetworkSP = std::shared←
_ptr<Network>
```

Shared pointer to network.

9.68.2.7 NodeID

```
template<typename Network , typename DistType , typename NbHopsType >
using LinkPred::NetIndSimlCalculator< Network, DistType, NbHopsType >::NodeID = typename
Network::NodeID
```

Nodes ID type.

9.68.3 Constructor & Destructor Documentation

9.68.3.1 ∼NetIndSimlCalculator()

```
template<typename Network , typename DistType , typename NbHopsType >
virtual LinkPred::NetIndSimlCalculator< Network, DistType, NbHopsType >::∼NetIndSimlCalculator
( ) [virtual], [default]
```

Destructor.

9.68.4 Member Function Documentation

9.68.4.1 getDirIndSiml()

```
template<typename Network , typename DistType , typename NbHopsType >
virtual std::tuple<DistType, DistType, NbHopsType> LinkPred::NetIndSimlCalculator< Network,
DistType, NbHopsType >::getDirIndSiml (
            NodeID const & i,
            NodeID const & j ) [pure virtual]
```

Parameters

i	Index of the source node.
j	Index of the end node.

Returns

The directed similarity between i and j ignoring the edge between i and j.

9.68.4.2 getDiscDist()

```
template<typename Network , typename DistType , typename NbHopsType >
double LinkPred::NetIndSimlCalculator< Network, DistType, NbHopsType >::getDiscDist ( ) const
[inline]
```

Returns

The distance assigned to disconnected nodes.

9.68.4.3 getDiscNbHops()

```
template<typename Network , typename DistType , typename NbHopsType >
std::size_t LinkPred::NetIndSimlCalculator< Network, DistType, NbHopsType >::getDiscNbHops ( )
const [inline]
```

Returns

The value that should be assigned as number of hops between disconnected nodes.

9.68.4.4 getIndSiml()

```
template<typename Network , typename DistType , typename NbHopsType >
virtual std::tuple<DistType, DistType, NbHopsType> LinkPred::NetIndSimlCalculator< Network,
DistType, NbHopsType >::getIndSiml (
            NodeID const & i,
            NodeID const & j ) [pure virtual]
```

Parameters

i	Index of the source node.
j	Index of the end node.

Returns

The similarity between i and j ignoring the edge between i and j.

9.68.4.5 getLambda()

```
template<typename Network , typename DistType , typename NbHopsType >
double LinkPred::NetIndSimlCalculator< Network, DistType, NbHopsType >::getLambda ( ) const
[inline]
```

Returns

The conductance.

9.68.4.6 getSelfSiml()

```
template<typename Network , typename DistType , typename NbHopsType >
double LinkPred::NetIndSimlCalculator< Network, DistType, NbHopsType >::getSelfSiml ( ) const
[inline]
```

Returns

The similarity between a node and itself.

9.68.4.7 setDiscDist()

```
template<typename Network , typename DistType , typename NbHopsType >
void LinkPred::NetIndSimlCalculator< Network, DistType, NbHopsType >::setDiscDist (
            DistType discDist )  [inline]
```

Set the distance assigned to disconnected nodes.

Parameters

discDist	The distance assigned to disconnected nodes.

9.68.4.8 setDiscNbHops()

```
template<typename Network , typename DistType , typename NbHopsType >
void LinkPred::NetIndSimlCalculator< Network, DistType, NbHopsType >::setDiscNbHops (
            NbHopsType discNbHops )  [inline]
```

Set the value that should be assigned as number of hops between disconnected nodes.

Parameters

discNbHops	The value that should be assigned as number of hops between disconnected nodes.

9.68.4.9 setLambda()

```
template<typename Network , typename DistType , typename NbHopsType >
void LinkPred::NetIndSimlCalculator< Network, DistType, NbHopsType >::setLambda (
            double lambda )  [inline]
```

Set the conductance.

Parameters

lambda	The new value of the weight.

9.68.4.10 setSelfSiml()

```
template<typename Network , typename DistType , typename NbHopsType >
void LinkPred::NetIndSimlCalculator< Network, DistType, NbHopsType >::setSelfSiml (
            DistType selfSiml )  [inline]
```

Set the similarity between a node and itself.

Parameters

selfSiml	The similarity similarity between a node and itself.

The documentation for this class was generated from the following file:

- include/linkpred/graphalg/shortestpaths/netdistcalculator.hpp

9.69 LinkPred::NetSimlCalculator< Network, DistType, NbHopsType > Class Template Reference

Interface for calculating the similarity between nodes in a network.

```
#include <netdistcalculator.hpp>
```

Public Types

- using LengthMapIdType = long int
- using NetworkSP = std::shared_ptr< Network >
- using NodeID = typename Network::NodeID
- using Label = typename Network::Label
- using Edge = typename Network::Edge
- using EdgeLengthMap = typename Network::template EdgeMap< DistType >
- using EdgeLengthMapSP = typename Network::template EdgeMapSP< DistType >
- using NodeSDistMap = typename Network::template NodeSMap< std::pair< DistType, NbHopsType > >
- using NodeSDistMapSP = typename Network::template NodeSMapSP< std::pair< DistType, NbHopsType > >

Public Member Functions

- virtual std::tuple< DistType, DistType, NbHopsType > getSiml (NodeID const &i, NodeID const &j)=0
- virtual std::tuple< DistType, DistType, NbHopsType > getDirSiml (NodeID const &i, NodeID const &j)=0
- virtual NodeSDistMapSP getNnzSimlMap (NodeID const &srcNode)
- virtual NodeSDistMapSP getNnzSimlMapNoNeighb (NodeID const &srcNode)
- double getSelfSiml () const
- void setSelfSiml (DistType selfSiml)
- double getDiscDist () const
- void setDiscDist (DistType discDist)
- std::size_t getDiscNbHops () const
- void setDiscNbHops (NbHopsType discNbHops)
- double getLambda () const
- void setLambda (double lambda)
- virtual ~NetSimlCalculator ()=default

9.69.1 Detailed Description

template<typename Network, typename DistType, typename NbHopsType>
class LinkPred::NetSimlCalculator< Network, DistType, NbHopsType >

Interface for calculating the similarity between nodes in a network.

Template Parameters

Network	The network type.
DistType	The distance and similarity type (can be an integer or floating point type).
NbHopsType	The type of the number of hops (must be an integer type).

9.69.2 Member Typedef Documentation

9.69.2.1 Edge

```
template<typename Network , typename DistType , typename NbHopsType >
using LinkPred::NetSimlCalculator< Network, DistType, NbHopsType >::Edge = typename Network↩
::Edge
```

Edge type.

9.69.2.2 EdgeLengthMap

```
template<typename Network , typename DistType , typename NbHopsType >
using LinkPred::NetSimlCalculator< Network, DistType, NbHopsType >::EdgeLengthMap = typename
Network::template EdgeMap<DistType>
```

Edge length map.

9.69.2.3 EdgeLengthMapSP

```
template<typename Network , typename DistType , typename NbHopsType >
using LinkPred::NetSimlCalculator< Network, DistType, NbHopsType >::EdgeLengthMapSP = typename
Network::template EdgeMapSP<DistType>
```

Shared pointer to an edge length map.

9.69.2.4 Label

```
template<typename Network , typename DistType , typename NbHopsType >
using LinkPred::NetSimlCalculator< Network, DistType, NbHopsType >::Label = typename Network↩
::Label
```

Nodes label type.

9.69.2.5 LengthMapIdType

```
template<typename Network , typename DistType , typename NbHopsType >
using LinkPred::NetSimlCalculator< Network, DistType, NbHopsType >::LengthMapIdType = long int
```

Length map ID type.

9.69.2.6 NetworkSP

```
template<typename Network , typename DistType , typename NbHopsType >
using LinkPred::NetSimlCalculator< Network, DistType, NbHopsType >::NetworkSP = std::shared_↩
ptr<Network>
```

Shared pointer to network.

9.69.2.7 NodeID

```
template<typename Network , typename DistType , typename NbHopsType >
using LinkPred::NetSimlCalculator< Network, DistType, NbHopsType >::NodeID = typename Network↩
::NodeID
```

Nodes ID type.

9.69.2.8 NodeSDistMap

```
template<typename Network , typename DistType , typename NbHopsType >
using LinkPred::NetSimlCalculator< Network, DistType, NbHopsType >::NodeSDistMap = typename
Network::template NodeSMap<std::pair<DistType, NbHopsType> >
```

Distance map.

9.69.2.9 NodeSDistMapSP

```
template<typename Network , typename DistType , typename NbHopsType >
using LinkPred::NetSimlCalculator< Network, DistType, NbHopsType >::NodeSDistMapSP = typename
Network::template NodeSMapSP<std::pair<DistType, NbHopsType> >
```

Shared pointer to a distance map.

9.69.3 Constructor & Destructor Documentation

9.69.3.1 ∼NetSimlCalculator()

```
template<typename Network , typename DistType , typename NbHopsType >
virtual LinkPred::NetSimlCalculator< Network, DistType, NbHopsType >::∼NetSimlCalculator ( )
[virtual], [default]
```

Destructor.

9.69.4 Member Function Documentation

9.69.4.1 getDirSiml()

```
template<typename Network , typename DistType , typename NbHopsType >
virtual std::tuple<DistType, DistType, NbHopsType> LinkPred::NetSimlCalculator< Network,
DistType, NbHopsType >::getDirSiml (
            NodeID const & i,
            NodeID const & j ) [pure virtual]
```

Parameters

i	Index of the source node.
j	Index of the end node.

Returns

The directed similarity between i and j.

9.69.4.2 getDiscDist()

```
template<typename Network , typename DistType , typename NbHopsType >
double LinkPred::NetSimlCalculator< Network, DistType, NbHopsType >::getDiscDist ( ) const
[inline]
```

Returns

The distance assigned to disconnected nodes.

9.69.4.3 getDiscNbHops()

```
template<typename Network , typename DistType , typename NbHopsType >
std::size_t LinkPred::NetSimlCalculator< Network, DistType, NbHopsType >::getDiscNbHops ( )
const [inline]
```

Returns

The value that should be assigned as number of hops between disconnected nodes.

9.69.4.4 getLambda()

```
template<typename Network , typename DistType , typename NbHopsType >
double LinkPred::NetSimlCalculator< Network, DistType, NbHopsType >::getLambda ( ) const
[inline]
```

Returns

The conductance.

9.69.4.5 getNnzSimlMap()

```
template<typename Network , typename DistType , typename NbHopsType >
virtual NodeSDistMapSP LinkPred::NetSimlCalculator< Network, DistType, NbHopsType >::getNnz←
SimlMap (
            NodeID const & srcNode ) [inline], [virtual]
```

Returns

A sparse similarity map of nodes having non-zero similarity to a given source node. Only nodes not connected to srcNode ar considered.

Parameters

srcNode	The source node.

9.69.4.6 getNnzSimlMapNoNeighb()

```
template<typename Network , typename DistType , typename NbHopsType >
virtual NodeSDistMapSP LinkPred::NetSimlCalculator< Network, DistType, NbHopsType >::getNnz←
SimlMapNoNeighb (
            NodeID const & srcNode )  [inline], [virtual]
```

Returns

A sparse similarity map of nodes having non-zero similarity to a given source node. Only nodes not connected to srcNode are considered. The node srcNode itself is also excluded.

Parameters

srcNode	The source node.

9.69.4.7 getSelfSiml()

```
template<typename Network , typename DistType , typename NbHopsType >
double LinkPred::NetSimlCalculator< Network, DistType, NbHopsType >::getSelfSiml ( ) const
[inline]
```

Returns

The similarity between a node and itself.

9.69.4.8 getSiml()

```
template<typename Network , typename DistType , typename NbHopsType >
virtual std::tuple<DistType, DistType, NbHopsType> LinkPred::NetSimlCalculator< Network,
DistType, NbHopsType >::getSiml (
            NodeID const & i,
            NodeID const & j )  [pure virtual]
```

Parameters

i	Index of the source node.
j	Index of the end node.

Returns

The similarity between i and j.

9.69.4.9 setDiscDist()

```
template<typename Network , typename DistType , typename NbHopsType >
void LinkPred::NetSimlCalculator< Network, DistType, NbHopsType >::setDiscDist (
            DistType discDist ) [inline]
```

Set the distance assigned to disconnected nodes.

Parameters

discDist	The distance assigned to disconnected nodes.

9.69.4.10 setDiscNbHops()

```
template<typename Network , typename DistType , typename NbHopsType >
void LinkPred::NetSimlCalculator< Network, DistType, NbHopsType >::setDiscNbHops (
            NbHopsType discNbHops ) [inline]
```

Set the value that should be assigned as number of hops between disconnected nodes.

Parameters

discNbHops	The value that should be assigned as number of hops between disconnected nodes.

9.69.4.11 setLambda()

```
template<typename Network , typename DistType , typename NbHopsType >
void LinkPred::NetSimlCalculator< Network, DistType, NbHopsType >::setLambda (
            double lambda ) [inline]
```

Set the conductance.

Parameters

lambda	The new value of the conductance.

9.69.4.12 setSelfSiml()

```
template<typename Network , typename DistType , typename NbHopsType >
void LinkPred::NetSimlCalculator< Network, DistType, NbHopsType >::setSelfSiml (
            DistType selfSiml ) [inline]
```

Set the similarity between a node and itself.

Parameters

selfSiml	The similarity similarity between a node and itself.

The documentation for this class was generated from the following file:

- include/linkpred/graphalg/shortestpaths/netdistcalculator.hpp

9.70 LinkPred::NetworkManipulator< Network > Class Template Reference

Class to manipulate network by removing or adding edges.

```
#include <networkmanipulator.hpp>
```

Public Types

- using NetworkSP = std::shared_ptr< Network >
- using NetworkCSP = std::shared_ptr< const Network >
- using NodeID = typename Network::NodeID
- using Label = typename Network::Label
- using Edge = typename Network::Edge
- template<typename ValueT >
  using NodeMap = typename Network::template NodeMap< ValueT >
- template<typename ValueT >
  using EdgeMap = typename Network::template EdgeMap< ValueT >

Public Member Functions

- NetworkManipulator ()=default
- virtual ~NetworkManipulator ()=default

Static Public Member Functions

- template< typename InserterIt >
  static void rst (NetworkCSP net, long int seed, InserterIt inserter)
- static std::pair< NetworkCSP, std::shared_ptr< std::vector< Edge > > > rndConExtract (NetworkCSP net, double ratio, long int seed)
- static std::pair< NetworkCSP, std::shared_ptr< std::vector< Edge > > > rndExtract (NetworkCSP net, double ratio, long int seed)
- template< typename InserterIt >
  static void getAllNegLinksExcept (NetworkCSP net, std::set< Edge > const &excepts, InserterIt inserter)
- template< typename InserterIt >
  static void getRndNegLinksExcept (NetworkCSP net, double ratio, long int seed, std::set< Edge > const &excepts, InserterIt inserter)
- template< typename InserterIt >
  static void getRndPosLinksExcept (NetworkCSP net, double ratio, long int seed, std::set< Edge > const &excepts, InserterIt inserter)
- static TestData< Network, std::vector< Edge > > createTestDataRem (NetworkCSP refNet, double remRatio, bool keepConnected, bool aTP, double tpRatio, bool aTN, double tnRatio, long int seed, bool preGenerateTPN=true)
- static TestData< Network, std::vector< Edge > > createTestDataRem (NetworkCSP refNet, double remRatio, long int seed, bool preGenerateTPN=true)
- static TestData< Network, std::vector< Edge > > createTestDataAdd (NetworkCSP refNet, double addRatio, bool aTP, double tpRatio, bool aTN, double tnRatio, long int seed, bool preGenerateTPN=true)
- static TestData< Network, std::vector< Edge > > createTestDataAdd (NetworkCSP refNet, double addRatio, long int seed, bool preGenerateTPN=true)
- static TestData< Network, std::vector< Edge > > createTestData (NetworkCSP refNet, double remRatio, double addRatio, bool keepConnected, bool aTP, double tpRatio, bool aTN, double tnRatio, LinkClass posClass, LinkClass negClass, long int seed, bool preGenerateTPN=true)
- static TestData< Network, std::vector< Edge > > createTestData (NetworkCSP refNet, double remRatio, double addRatio, LinkClass posClass, LinkClass negClass, long int seed, bool preGenerateTPN=true)
- static TestData< Network, std::vector< Edge > > createTestDataSeqInter (NetworkCSP firstNet, NetworkCSP secondNet, bool aTP, double tpRatio, bool aTN, double tnRatio, LinkClass posClass, LinkClass negClass, long int seed, bool preGenerateTPN=true)
- static TestData< Network, std::vector< Edge > > createTestDataSeq (NetworkCSP firstNet, NetworkCSP secondNet, bool aTP, double tpRatio, bool aTN, double tnRatio, LinkClass posClass, LinkClass negClass, long int seed, bool preGenerateTPN=true)
- static TestData< Network, std::vector< Edge > > loadTestData (std::string obsEdgesFileName, std::string remEdgesFileName, std::string addEdgesFileName, bool aTP, double tpRatio, bool aTN, double tnRatio, LinkClass posClass, LinkClass negClass, long int seed, bool preGenerateTPN=true)
- static TestData< Network, std::vector< Edge > > loadTestDataRem (std::string obsEdgesFileName, std::string remEdgesFileName, bool preGenerateTPN=true)
- static TestData< Network, std::vector< Edge > > loadTestDataAdd (std::string obsEdgesFileName, std::string addEdgesFileName, bool preGenerateTPN=true)
- static bool isConnected (NetworkCSP net)

9.70.1 Detailed Description

template< typename Network = UNetwork<>>
class LinkPred::NetworkManipulator< Network >

Class to manipulate network by removing or adding edges.

The following terminology is used: When removing links: TP: Edges of observed network TN: Non-edges of reference network FP: None FN: Removed links When adding links: TP: Edges of reference network TN: Non-edges of observed network FP: Added links FN: None When adding/removing links: TP: Edges of observed network - added links = Edges of reference network - removed links TN: Non-edges of observed network - removed links = Non-edges of reference network - added links FP: Added links FN: Removed links

Template Parameters

Network	The network type.

9.70.2 Member Typedef Documentation

9.70.2.1 Edge

```
template<typename Network = UNetwork<>>
using LinkPred::NetworkManipulator< Network >::Edge = typename Network::Edge
```

The edge type.

9.70.2.2 EdgeMap

```
template<typename Network = UNetwork<>>
template<typename ValueT >
using LinkPred::NetworkManipulator< Network >::EdgeMap = typename Network::template EdgeMap<ValueT>
```

Edge map.

9.70.2.3 Label

```
template<typename Network = UNetwork<>>
using LinkPred::NetworkManipulator< Network >::Label = typename Network::Label
```

Nodes labels type.

9.70.2.4 NetworkCSP

```
template<typename Network = UNetwork<>>
using LinkPred::NetworkManipulator< Network >::NetworkCSP = std::shared_ptr<const Network>
```

Constant shared pointer to a network.

9.70.2.5 NetworkSP

```
template<typename Network = UNetwork<>>
using LinkPred::NetworkManipulator< Network >::NetworkSP = std::shared_ptr<Network>
```

Shared pointer to a network.

9.70.2.6 NodeID

```
template<typename Network = UNetwork<>>
using LinkPred::NetworkManipulator< Network >::NodeID = typename Network::NodeID
```

Nodes IDs type.

9.70.2.7 NodeMap

```
template<typename Network = UNetwork<>>
template<typename ValueT >
using LinkPred::NetworkManipulator< Network >::NodeMap = typename Network::template NodeMap<ValueT>
```

Node map.

9.70.3 Constructor & Destructor Documentation

9.70.3.1 NetworkManipulator()

```
template<typename Network = UNetwork<>>
LinkPred::NetworkManipulator< Network >::NetworkManipulator ( ) [default]
```

Constructor.

9.70.3.2 ∼NetworkManipulator()

```
template<typename Network = UNetwork<>>
virtual LinkPred::NetworkManipulator< Network >::~NetworkManipulator ( ) [virtual], [default]
```

Destructor.

9.70.4 Member Function Documentation

9.70.4.1 createTestData() [1/2]

```
template<typename Network = UNetwork<>>
static TestData<Network, std::vector<Edge> > LinkPred::NetworkManipulator< Network >::create↩
TestData (
            NetworkCSP refNet,
            double remRatio,
            double addRatio,
            bool keepConnected,
            bool aTP,
            double tpRatio,
            bool aTN,
            double tnRatio,
            LinkClass posClass,
            LinkClass negClass,
            long int seed,
            bool preGenerateTPN = true ) [static]
```

Creates test data by adding/removing edges from a network. The reference network is not modified. The two networks have the same external-internal ID mapping.

Parameters

refNet	The reference network.
remRatio	Value between 0 and 1 that specifies the percentage of edges that are removed.
addRatio	Value between 0 and 1 that specifies the percentage of edges that are added.
keepConnected	Whether to keep the network connected.
aTP	Whether to use all true positive links in the test set.
tpRatio	Ratio of true positive inks to e used in the test set. This parameter is only relevant when aTP is false.
aTN	Whether to use all true negative links in the test set.
tnRatio	Ratio of true negative inks to e used in the test set. This parameter is only relevant when aTN is false.
posClass	Indicates which links will be considered the positive links.
negClass	Indicates which links will be considered the negative links.
seed	The random number generator's seed.
preGenerateTPN	Whether to pre-generate true positives and true negatives.

Returns

The test data.

9.70.4.2 createTestData() [2/2]

```
template<typename Network = UNetwork<>>
static TestData<Network, std::vector<Edge> > LinkPred::NetworkManipulator< Network >::create↵
TestData (
            NetworkCSP refNet,
            double remRatio,
            double addRatio,
            LinkClass posClass,
            LinkClass negClass,
            long int seed,
            bool preGenerateTPN = true )  [static]
```

Creates test data by adding/removing edges from a network. The reference network is not modified. The two networks have the same external-internal ID mapping.

Parameters

refNet	The reference network.
remRatio	Value between 0 and 1 that specifies the percentage of edges that are removed.
addRatio	Value between 0 and 1 that specifies the percentage of edges that are added.
posClass	Indicates which links will be considered the positive links.
negClass	Indicates which links will be considered the negative links.
seed	The random number generator's seed.
preGenerateTPN	Whether to pre-generate true positives and true negatives.

Returns

The test data.

9.70.4.3 createTestDataAdd() [1/2]

```
template<typename Network = UNetwork<>>
static TestData<Network, std::vector<Edge> > LinkPred::NetworkManipulator< Network >::create←
TestDataAdd (
            NetworkCSP refNet,
            double addRatio,
            bool aTP,
            double tpRatio,
            bool aTN,
            double tnRatio,
            long int seed,
            bool preGenerateTPN = true )   [static]
```

Creates test data by adding edges to a network. The reference network is not modified. The two networks have the same external-internal ID mapping.

Parameters

refNet	The reference network.
addRatio	Value between 0 and 1 that specifies the percentage of edges that are added.
aTP	Whether to use all true positive links in the test set.
tpRatio	Ratio of true positive inks to e used in the test set. This parameter is only relevant when aTP is false.
aTN	Whether to use all true negative links in the test set.
tnRatio	Ratio of true negative inks to e used in the test set. This parameter is only relevant when aTN is false.
seed	The random number generator's seed.
preGenerateTPN	Whether to pre-generate true positives and true negatives.

Returns

The test data.

9.70.4.4 createTestDataAdd() [2/2]

```
template<typename Network = UNetwork<>>
static TestData<Network, std::vector<Edge> > LinkPred::NetworkManipulator< Network >::create←
TestDataAdd (
            NetworkCSP refNet,
            double addRatio,
            long int seed,
            bool preGenerateTPN = true )   [static]
```

Creates test data by adding edges to a network. The reference network is not modified. The two networks have the same external-internal ID mapping.

Parameters

refNet	The reference network.
addRatio	Value between 0 and 1 that specifies the percentage of edges that are added.
seed	The random number generator's seed.
preGenerateTPN	Whether to pre-generate true positives and true negatives.

Returns

The test data.

9.70.4.5 createTestDataRem() [1/2]

```
template<typename Network = UNetwork<>>
static TestData<Network, std::vector<Edge> > LinkPred::NetworkManipulator< Network >::create↩
TestDataRem (
            NetworkCSP refNet,
            double remRatio,
            bool keepConnected,
            bool aTP,
            double tpRatio,
            bool aTN,
            double tnRatio,
            long int seed,
            bool preGenerateTPN = true )  [static]
```

Creates test data by removing edges from a network. The reference network is not modified. The two networks have the same external-internal ID mapping.

Parameters

refNet	The reference network.
remRatio	Value between 0 and 1 that specifies the percentage of edges that are removed.
keepConnected	Whether to keep the network connected.
aTP	Whether to use all true positive links in the test set.
tpRatio	Ratio of true positive inks to e used in the test set. This parameter is only relevant when aTP is false.
aTN	Whether to use all true negative links in the test set.
tnRatio	Ratio of true negative inks to e used in the test set. This parameter is only relevant when aTN is false.
seed	The random number generator's seed.
preGenerateTPN	Whether to pre-generate true positives and true negatives.

Returns

The test data.

9.70.4.6 createTestDataRem() [2/2]

```
template<typename Network = UNetwork<>>
static TestData<Network, std::vector<Edge> > LinkPred::NetworkManipulator< Network >::create↩
TestDataRem (
            NetworkCSP refNet,
            double remRatio,
            long int seed,
            bool preGenerateTPN = true )  [static]
```

Creates test data by removing edges from a network. The reference network is not modified. The two networks have the same external-internal ID mapping.

Parameters

refNet	The reference network.
remRatio	Value between 0 and 1 that specifies the percentage of edges that are removed.
seed	The random number generator's seed.
preGenerateTPN	Whether to pre-generate true positives and true negatives.

Returns

The test data.

9.70.4.7 createTestDataSeq()

```
template<typename Network = UNetwork<>>
static TestData<Network, std::vector<Edge> > LinkPred::NetworkManipulator< Network >::create↩
TestDataSeq (
            NetworkCSP firstNet,
            NetworkCSP secondNet,
            bool aTP,
            double tpRatio,
            bool aTN,
            double tnRatio,
            LinkClass posClass,
            LinkClass negClass,
            long int seed,
            bool preGenerateTPN = true )  [static]
```

Creates test data from two networks. Nodes not present in the second network and associated edges are removed from the test set.

Parameters

firstNet	The first network.
secondNet	The second network.
aTP	Whether to use all true positive links in the test set.
tpRatio	Ratio of true positive inks to e used in the test set. This parameter is only relevant when aTP is false.
aTN	Whether to use all true negative links in the test set.

Parameters

tnRatio	Ratio of true negative inks to e used in the test set. This parameter is only relevant when aTN is false.
posClass	Indicates which links will be considered the positive links.
negClass	Indicates which links will be considered the negative links.
seed	The random number generator's seed.
preGenerateTPN	Whether to pre-generate true positives and true negatives.

Returns

The test data.

9.70.4.8 createTestDataSeqInter()

```
template<typename Network = UNetwork<>>
static TestData<Network, std::vector<Edge> > LinkPred::NetworkManipulator< Network >::create↵
TestDataSeqInter (
            NetworkCSP firstNet,
            NetworkCSP secondNet,
            bool aTP,
            double tpRatio,
            bool aTN,
            double tnRatio,
            LinkClass posClass,
            LinkClass negClass,
            long int seed,
            bool preGenerateTPN = true )  [static]
```

Creates test data from two networks. Only nodes common to both networks are considered.

Parameters

firstNet	The first network.
secondNet	The second network.
aTP	Whether to use all true positive links in the test set.
tpRatio	Ratio of true positive inks to e used in the test set. This parameter is only relevant when aTP is false.
aTN	Whether to use all true negative links in the test set.
tnRatio	Ratio of true negative inks to e used in the test set. This parameter is only relevant when aTN is false.
posClass	Indicates which links will be considered the positive links.
negClass	Indicates which links will be considered the negative links.
seed	The random number generator's seed.
preGenerateTPN	Whether to pre-generate true positives and true negatives.

Returns

The test data.

9.70.4.9 getAllNegLinksExcept()

```
template<typename Network = UNetwork<>>
template<typename InserterIt >
static void LinkPred::NetworkManipulator< Network >::getAllNegLinksExcept (
            NetworkCSP net,
            std::set< Edge > const & excepts,
            InserterIt inserter )  [inline], [static]
```

Return all negative links except those passed in parameter. Be aware that this can be computationally intensive both in term of time and space.

Parameters

net	The network.
excepts	The links that are excepted.
inserter	An inserter iterator.

9.70.4.10 getRndNegLinksExcept()

```
template<typename Network = UNetwork<>>
template<typename InserterIt >
static void LinkPred::NetworkManipulator< Network >::getRndNegLinksExcept (
            NetworkCSP net,
            double ratio,
            long int seed,
            std::set< Edge > const & excepts,
            InserterIt inserter )  [inline], [static]
```

Select random negative links except those passed in parameter.

Parameters

net	The network.
ratio	The ratio of negative links to be selected.
seed	The random seed.
excepts	The links that are excepted.
inserter	The inserter iterator where the selected edges will be inserted.

9.70.4.11 getRndPosLinksExcept()

```
template<typename Network = UNetwork<>>
template<typename InserterIt >
```

```
static void LinkPred::NetworkManipulator< Network >::getRndPosLinksExcept (
            NetworkCSP net,
            double ratio,
            long int seed,
            std::set< Edge > const & excepts,
            InserterIt inserter ) [inline], [static]
```

Select random negative links except those passed in parameter.

Parameters

net	The network.
ratio	The ratio of positive links to be selected.
seed	The random seed.
excepts	The links that are excepted.
inserter	The inserter iterator where the selected edges will be inserted.

9.70.4.12 isConnected()

```
template<typename Network = UNetwork<>>
static bool LinkPred::NetworkManipulator< Network >::isConnected (
            NetworkCSP net ) [inline], [static]
```

Check if a network is connected.

Parameters

net	The network.

Returns

True if the network net is connected, false otherwise.

9.70.4.13 loadTestData()

```
template<typename Network = UNetwork<>>
static TestData<Network, std::vector<Edge> > LinkPred::NetworkManipulator< Network >::load←
TestData (
            std::string obsEdgesFileName,
            std::string remEdgesFileName,
            std::string addEdgesFileName,
            bool aTP,
            double tpRatio,
            bool aTN,
            double tnRatio,
            LinkClass posClass,
            LinkClass negClass,
```

```
long int seed,
bool preGenerateTPN = true )  [static]
```

Read test data from file.

Parameters

obsEdgesFileName	A file containing the observed edges (edge list format).
remEdgesFileName	A file containing the removed edges (edge list format). This is ignored if equal to empty string "".
addEdgesFileName	A file containing the add edges (edge list format). This is ignored if equal to empty string "".
aTP	Whether to use all true positive links in the test set.
tpRatio	Ratio of true positive inks to e used in the test set. This parameter is only relevant when aTP is false.
aTN	Whether to use all true negative links in the test set.
tnRatio	Ratio of true negative inks to e used in the test set. This parameter is only relevant when aTN is false.
posClass	Indicates which links will be considered the positive links.
negClass	Indicates which links will be considered the negative links.
seed	The random number generator's seed.
preGenerateTPN	Whether to pre-generate true positives and true negatives.

Returns

The test data.

9.70.4.14 loadTestDataAdd()

```
template<typename Network = UNetwork<>>
static TestData<Network, std::vector<Edge> > LinkPred::NetworkManipulator< Network >::load↩
TestDataAdd (
            std::string obsEdgesFileName,
            std::string addEdgesFileName,
            bool preGenerateTPN = true )  [static]
```

Read test data from file (test data obtained by adding edges).

Parameters

obsEdgesFileName	A file containing the observed edges (edge list format).
addEdgesFileName	A file containing the added edges (edge list format).
preGenerateTPN	Whether to pre-generate true positives and true negatives.

Returns

The test data.

9.70.4.15 loadTestDataRem()

```
template<typename Network = UNetwork<>>
static TestData<Network, std::vector<Edge> > LinkPred::NetworkManipulator< Network >::load↩
```

```
TestDataRem (
            std::string obsEdgesFileName,
            std::string remEdgesFileName,
            bool preGenerateTPN = true )  [static]
```

Read test data from file (test data obtained by removing edges).

Parameters

obsEdgesFileName	A file containing the observed edges (edge list format).
remEdgesFileName	A file containing the removed edges (edge list format).
preGenerateTPN	Whether to pre-generate true positives and true negatives.

Returns

The test data.

9.70.4.16 rndConExtract()

```
template<typename Network = UNetwork<>>
static std::pair<NetworkCSP, std::shared_ptr<std::vector<Edge> > > LinkPred::NetworkManipulator<
Network >::rndConExtract (
            NetworkCSP net,
            double ratio,
            long int seed )  [static]
```

Creates a new connected network from the current by extracting uniformly randomly a specified ratio of edges. The reference network is not modified. The two networks have the same external-internal ID mapping.

Parameters

net	The reference network.
ratio	Value between 0 and 1 that specifies the percentage of edges that are removed.
seed	The random number generator's seed.

Returns

A pair that contains a pointer to the resulting network and pointer to the set of extracted links.

9.70.4.17 rndExtract()

```
template<typename Network = UNetwork<>>
static std::pair<NetworkCSP, std::shared_ptr<std::vector<Edge> > > LinkPred::NetworkManipulator<
Network >::rndExtract (
            NetworkCSP net,
```

```
            double ratio,
            long int seed )  [static]
```

Creates a new network from the current by extracting uniformly randomly a specified ratio of edges. The reference network is not modified. The two networks have the same external-internal ID mapping.

Parameters

net	The reference network.
ratio	Value between 0 and 1 that specifies the percentage of edges that are removed.
seed	The random number generator's seed.

Returns

A pair that contains a pointer to the resulting network and pointer to the set of extracted links.

9.70.4.18 rst()

```
template<typename Network = UNetwork<>>
template<typename InserterIt >
static void LinkPred::NetworkManipulator< Network >::rst (
            NetworkCSP net,
            long int seed,
            InserterIt inserter )  [inline], [static]
```

Generate a random spanning tree.

Template Parameters

Inserter↵ It	Type of iterator to insert tree edges.

Parameters

net	The network.
seed	The random number generator's seed.
inserter	An inserter iterator to insert the tree edges.

The documentation for this class was generated from the following file:

- include/linkpred/perf/networkmanipulator.hpp

9.71 LinkPred::Node2Vec< Network > Class Template Reference

Node2Vec encoder. References: Grover, A. and Leskovec, J. (2016). Node2vec: Scalable feature learning for networks. In Proceedings of the 22nd ACM SIGKDD International Conference on Knowledge Discovery and Data

Mining, KDD'16, pages 855–864, New York, NY, USA. Association for Computing Machinery. This implementation is based on the code https://github.com/xgfs/node2vec-c.

```
#include <node2vec.hpp>
```

Inheritance diagram for LinkPred::Node2Vec< Network >:

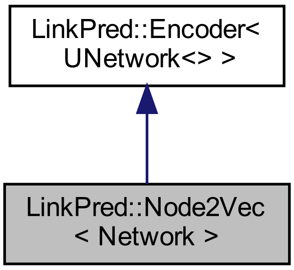

Collaboration diagram for LinkPred::Node2Vec< Network >:

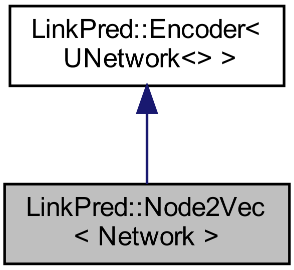

Public Member Functions

- Node2Vec (std::shared_ptr< Network const > net, long int seed)
- Node2Vec (Node2Vec const &that)=default
- Node2Vec & operator= (Node2Vec const &that)=default
- Node2Vec (Node2Vec &&that)=default
- Node2Vec & operator= (Node2Vec &&that)=default
- virtual void init ()
- virtual void encode ()
- float getInitLR () const
- void setInitLR (float initLR)
- int getNbNegSamples () const
- void setNbNegSamples (int nbNegSamples)

- int getNbWalks () const
- void setNbWalks (int nbWalks)
- float getP () const
- void setP (float p)
- float getQ () const
- void setQ (float q)
- int getWalkLength () const
- void setWalkLength (int walkLength)
- int getWindowSize () const
- void setWindowSize (int windowSize)
- virtual void setWeightMap (const WeightMapSP &weightMap)
- long long getStepInterval () const
- void setStepInterval (long long stepInterval)
- long long getTotalSteps () const
- const int getDefaultDim () const
- float getSubSample () const
- void setSubSample (float subSample)
- virtual ∼Node2Vec ()=default

Additional Inherited Members

9.71.1 Detailed Description

template<typename Network = UNetwork<>>
class LinkPred::Node2Vec< Network >

Node2Vec encoder. References: Grover, A. and Leskovec, J. (2016). Node2vec: Scalable feature learning for networks. In Proceedings of the 22nd ACM SIGKDD International Conference on Knowledge Discovery and Data Mining, KDD'16, pages 855–864, New York, NY, USA. Association for Computing Machinery. This implementation is based on the code `https://github.com/xgfs/node2vec-c`.

Template Parameters

Network	The network type.

9.71.2 Constructor & Destructor Documentation

9.71.2.1 Node2Vec() [1/3]

```
template<typename Network = UNetwork<>>
LinkPred::Node2Vec< Network >::Node2Vec (
            std::shared_ptr< Network const > net,
            long int seed )  [inline]
```

Constructor.

Parameters

net	The network.
seed	Random number generator seed.

9.71.2.2 Node2Vec() [2/3]

```
template<typename Network = UNetwork<>>
LinkPred::Node2Vec< Network >::Node2Vec (
            Node2Vec< Network > const & that )  [default]
```

Copy constructor.

Parameters

that	The object to copy.

9.71.2.3 Node2Vec() [3/3]

```
template<typename Network = UNetwork<>>
LinkPred::Node2Vec< Network >::Node2Vec (
            Node2Vec< Network > && that )  [default]
```

Move constructor.

Parameters

that	The object to move.

9.71.2.4 ∼Node2Vec()

```
template<typename Network = UNetwork<>>
virtual LinkPred::Node2Vec< Network >::~Node2Vec ( )  [virtual], [default]
```

Destructor.

9.71.3 Member Function Documentation

9.71.3.1 encode()

```
template<typename Network = UNetwork<>>
virtual void LinkPred::Node2Vec< Network >::encode ( ) [virtual]
```

Encode the network.

Implements LinkPred::Encoder< UNetwork<> >.

9.71.3.2 getDefaultDim()

```
template<typename Network = UNetwork<>>
const int LinkPred::Node2Vec< Network >::getDefaultDim ( ) const [inline]
```

Returns

Default embedding dimension.

9.71.3.3 getInitLR()

```
template<typename Network = UNetwork<>>
float LinkPred::Node2Vec< Network >::getInitLR ( ) const [inline]
```

Returns

Initial learning rate.

9.71.3.4 getNbNegSamples()

```
template<typename Network = UNetwork<>>
int LinkPred::Node2Vec< Network >::getNbNegSamples ( ) const [inline]
```

Returns

Number of negative samples.

9.71.3.5 getNbWalks()

```
template<typename Network = UNetwork<>>
int LinkPred::Node2Vec< Network >::getNbWalks ( ) const  [inline]
```

Returns

Number of walks per vertex ("\gamma").

9.71.3.6 getP()

```
template<typename Network = UNetwork<>>
float LinkPred::Node2Vec< Network >::getP ( ) const  [inline]
```

Returns

The parameter p.

9.71.3.7 getQ()

```
template<typename Network = UNetwork<>>
float LinkPred::Node2Vec< Network >::getQ ( ) const  [inline]
```

Returns

The parameter q.

9.71.3.8 getStepInterval()

```
template<typename Network = UNetwork<>>
long long LinkPred::Node2Vec< Network >::getStepInterval ( ) const  [inline]
```

Returns

The number of steps after which the learning rate is updated.

9.71.3.9 getSubSample()

```
template<typename Network = UNetwork<>>
float LinkPred::Node2Vec< Network >::getSubSample ( ) const  [inline]
```

Returns

Sub-sample size.

9.71.3.10 getTotalSteps()

```
template<typename Network = UNetwork<>>
long long LinkPred::Node2Vec< Network >::getTotalSteps ( ) const  [inline]
```

Returns

The total number of steps.

9.71.3.11 getWalkLength()

```
template<typename Network = UNetwork<>>
int LinkPred::Node2Vec< Network >::getWalkLength ( ) const  [inline]
```

Returns

DeepWalk parameter "t" = length of the walk.

9.71.3.12 getWindowSize()

```
template<typename Network = UNetwork<>>
int LinkPred::Node2Vec< Network >::getWindowSize ( ) const  [inline]
```

Returns

DeepWalk parameter "w" = window size.

9.71.3.13 init()

```
template<typename Network = UNetwork<>>
virtual void LinkPred::Node2Vec< Network >::init ( )  [virtual]
```

Initialize encoder.

Implements LinkPred::Encoder< UNetwork<> >.

9.71.3.14 operator=() [1/2]

```
template<typename Network = UNetwork<>>
Node2Vec& LinkPred::Node2Vec< Network >::operator= (
            Node2Vec< Network > && that )  [default]
```

Move assignment operator.

Parameters

that	The object to move.

9.71.3.15 operator=() [2/2]

```
template<typename Network = UNetwork<>>
Node2Vec& LinkPred::Node2Vec< Network >::operator= (
            Node2Vec< Network > const & that )  [default]
```

Copy assignment operator.

Parameters

that	The object to copy.

9.71.3.16 setInitLR()

```
template<typename Network = UNetwork<>>
void LinkPred::Node2Vec< Network >::setInitLR (
            float initLR )  [inline]
```

Set the initial learning rate.

Parameters

initLR	Initial learning rate.

9.71.3.17 setNbNegSamples()

```
template<typename Network = UNetwork<>>
void LinkPred::Node2Vec< Network >::setNbNegSamples (
            int nbNegSamples )  [inline]
```

Set the number of negative samples.

Parameters

nbNegSamples	The number of negative samples.

9.71.3.18 setNbWalks()

```
template<typename Network = UNetwork<>>
void LinkPred::Node2Vec< Network >::setNbWalks (
            int nbWalks ) [inline]
```

Set the number of walks per vertex ("\gamma").

Parameters

nbWalks	Number of walks per vertex ("\gamma").

9.71.3.19 setP()

```
template<typename Network = UNetwork<>>
void LinkPred::Node2Vec< Network >::setP (
            float p ) [inline]
```

Set the parameter p.

Parameters

p	The parameter p.

9.71.3.20 setQ()

```
template<typename Network = UNetwork<>>
void LinkPred::Node2Vec< Network >::setQ (
            float q ) [inline]
```

Set the parameter q.

Parameters

q	The parameter q.

9.71.3.21 setStepInterval()

```
template<typename Network = UNetwork<>>
void LinkPred::Node2Vec< Network >::setStepInterval (
            long long stepInterval ) [inline]
```

Set the number of steps after which the learning rate is updated.

Parameters

stepInterval	Number of steps after which the learning rate is updated.

9.71.3.22 setSubSample()

```
template<typename Network = UNetwork<>>
void LinkPred::Node2Vec< Network >::setSubSample (
            float subSample ) [inline]
```

Set sub-sample size.

Parameters

subSample	Sub-sample size.

9.71.3.23 setWalkLength()

```
template<typename Network = UNetwork<>>
void LinkPred::Node2Vec< Network >::setWalkLength (
            int walkLength ) [inline]
```

Set the DeepWalk parameter "t" = length of the walk.

Parameters

walkLength	DeepWalk parameter "t" = length of the walk.

9.71.3.24 setWeightMap()

```
template<typename Network = UNetwork<>>
virtual void LinkPred::Node2Vec< Network >::setWeightMap (
            const WeightMapSP & weightMap ) [inline], [virtual]
```

Set edge weight map.

Reimplemented from LinkPred::Encoder< UNetwork<> >.

9.71.3.25 setWindowSize()

```
template<typename Network = UNetwork<>>
void LinkPred::Node2Vec< Network >::setWindowSize (
            int windowSize ) [inline]
```

Set the DeepWalk parameter "w" = window size.

Parameters

windowSize	DeepWalk parameter "w" = window size.

The documentation for this class was generated from the following file:

- include/linkpred/graphalg/encoders/node2vec/node2vec.hpp

9.72 LinkPred::DNetwork< LabelT, NodeIDT, EdgeT >::NodeDegIt Class Reference

Node-degree iterator. This class can be used to iterate over pairs of node IDs and in and out degrees.

```
#include <dnetwork.hpp>
```

Inheritance diagram for LinkPred::DNetwork< LabelT, NodeIDT, EdgeT >::NodeDegIt:

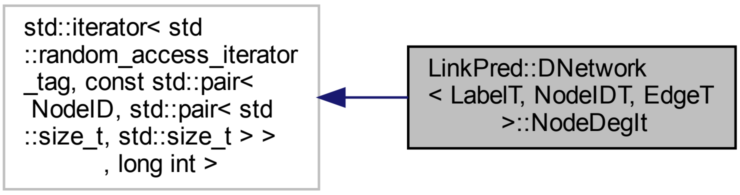

Collaboration diagram for LinkPred::DNetwork< LabelT, NodeIDT, EdgeT >::NodeDegIt:

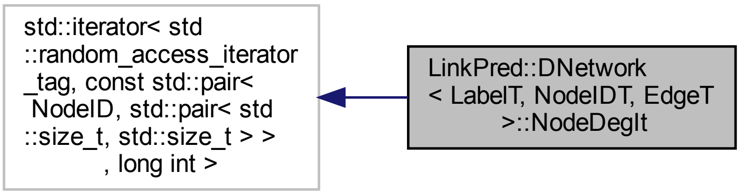

Public Types

- using pointer = typename std::iterator< std::random_access_iterator_tag, const std::pair< NodeID, std::pair< std::size_t, std::size_t > >, long int >::pointer
- using reference = typename std::iterator< std::random_access_iterator_tag, const std::pair< NodeID, std::pair< std::size_t, std::size_t > >, long int >::reference
- using difference_type = typename std::iterator< std::random_access_iterator_tag, const std::pair< NodeID, std::pair< std::size_t, std::size_t > >, long int >::difference_type

Public Member Functions

- NodeDegIt (NodeDegIt const &that)=default
- NodeDegIt & operator= (NodeDegIt const &that)=default
- NodeDegIt (NodeDegIt &&that)=default
- NodeDegIt & operator= (NodeDegIt &&that)=default
- reference operator* ()
- pointer operator-> ()
- NodeDegIt & operator++ ()
- NodeDegIt & operator-- ()
- NodeDegIt operator++ (int)
- NodeDegIt operator-- (int)
- NodeDegIt operator+ (const difference_type &n) const
- NodeDegIt & operator+= (const difference_type &n)
- NodeDegIt operator- (const difference_type &n) const
- NodeDegIt & operator-= (const difference_type &n)
- difference_type operator- (const NodeDegIt &that) const
- bool operator== (const NodeDegIt &that) const
- bool operator!= (const NodeDegIt &that) const
- bool operator< (const NodeDegIt &that) const
- bool operator> (const NodeDegIt &that) const
- bool operator<= (const NodeDegIt &that) const
- bool operator>= (const NodeDegIt &that) const

Friends

- class DNetwork

9.72.1 Detailed Description

**template<typename LabelT = std::string, typename NodeIDT = unsigned int, typename EdgeT = unsigned long long int>
class LinkPred::DNetwork< LabelT, NodeIDT, EdgeT >::NodeDegIt**

Node-degree iterator. This class can be used to iterate over pairs of node IDs and in and out degrees.

9.72.2 Member Typedef Documentation

9.72.2.1 difference_type

```
template<typename LabelT = std::string, typename NodeIDT = unsigned int, typename EdgeT =
unsigned long long int>
using LinkPred::DNetwork< LabelT, NodeIDT, EdgeT >::NodeDegIt::difference_type = typename
std::iterator<std::random_access_iterator_tag, const std::pair<NodeID, std::pair<std::size↩
_t, std::size_t> >, long int>::difference_type
```

The difference type associated with the iterator.

9.72.2.2 pointer

```
template<typename LabelT = std::string, typename NodeIDT = unsigned int, typename EdgeT =
unsigned long long int>
using LinkPred::DNetwork< LabelT, NodeIDT, EdgeT >::NodeDegIt::pointer = typename std::iterator<std↩
::random_access_iterator_tag, const std::pair<NodeID, std::pair<std::size_t, std::size_t> >,
long int>::pointer
```

The pointer type associated with the iterator.

9.72.2.3 reference

```
template<typename LabelT = std::string, typename NodeIDT = unsigned int, typename EdgeT =
unsigned long long int>
using LinkPred::DNetwork< LabelT, NodeIDT, EdgeT >::NodeDegIt::reference = typename std↩
::iterator<std::random_access_iterator_tag, const std::pair<NodeID, std::pair<std::size_t,
std::size_t> >, long int>::reference
```

The reference type associated with the iterator.

9.72.3 Constructor & Destructor Documentation

9.72.3.1 NodeDegIt() [1/2]

```
template<typename LabelT = std::string, typename NodeIDT = unsigned int, typename EdgeT =
unsigned long long int>
LinkPred::DNetwork< LabelT, NodeIDT, EdgeT >::NodeDegIt::NodeDegIt (
            NodeDegIt const & that )  [default]
```

Copy constructor.

Parameters

that	The object to copy.

9.72.3.2 NodeDegIt() [2/2]

```
template<typename LabelT = std::string, typename NodeIDT = unsigned int, typename EdgeT =
unsigned long long int>
LinkPred::DNetwork< LabelT, NodeIDT, EdgeT >::NodeDegIt::NodeDegIt (
            NodeDegIt && that )  [default]
```

Move constructor.

Parameters

that	The object to move.

9.72.4 Member Function Documentation

9.72.4.1 operator"!=()

```
template<typename LabelT = std::string, typename NodeIDT = unsigned int, typename EdgeT =
unsigned long long int>
bool LinkPred::DNetwork< LabelT, NodeIDT, EdgeT >::NodeDegIt::operator!= (
            const NodeDegIt & that ) const  [inline]
```

Parameters

that	The other iterator.

Returns

True if this is not equal to that.

9.72.4.2 operator*()

```
template<typename LabelT = std::string, typename NodeIDT = unsigned int, typename EdgeT =
unsigned long long int>
reference LinkPred::DNetwork< LabelT, NodeIDT, EdgeT >::NodeDegIt::operator* ( )  [inline]
```

Dereference operator.

Returns

A reference to the object to which the iterator points.

9.72.4.3 operator+()

```
template<typename LabelT = std::string, typename NodeIDT = unsigned int, typename EdgeT =
unsigned long long int>
NodeDegIt LinkPred::DNetwork< LabelT, NodeIDT, EdgeT >::NodeDegIt::operator+ (
            const difference_type & n ) const  [inline]
```

Arithmetic + operator.

Parameters

n	Increment value.

Returns

The new iterator.

9.72.4.4 operator++() [1/2]

```
template<typename LabelT = std::string, typename NodeIDT = unsigned int, typename EdgeT =
unsigned long long int>
NodeDegIt& LinkPred::DNetwork< LabelT, NodeIDT, EdgeT >::NodeDegIt::operator++ ( )  [inline]
```

Pre-increment operator.

Returns

A reference to the new iterator.

9.72.4.5 operator++() [2/2]

```
template<typename LabelT = std::string, typename NodeIDT = unsigned int, typename EdgeT =
unsigned long long int>
NodeDegIt LinkPred::DNetwork< LabelT, NodeIDT, EdgeT >::NodeDegIt::operator++ (
            int  )  [inline]
```

Post-increment operator.

Returns

A reference to the new iterator.

9.72.4.6 operator+=()

```
template<typename LabelT = std::string, typename NodeIDT = unsigned int, typename EdgeT =
unsigned long long int>
NodeDegIt& LinkPred::DNetwork< LabelT, NodeIDT, EdgeT >::NodeDegIt::operator+= (
            const difference_type & n )  [inline]
```

Arithmetic += operator.

Parameters

n	Increment value.

Returns

A reference to the new iterator.

9.72.4.7 operator-() [1/2]

```
template<typename LabelT = std::string, typename NodeIDT = unsigned int, typename EdgeT =
unsigned long long int>
NodeDegIt LinkPred::DNetwork< LabelT, NodeIDT, EdgeT >::NodeDegIt::operator- (
            const difference_type & n ) const [inline]
```

Arithmetic - operator.

Parameters

n	Decrement value.

Returns

The new iterator.

9.72.4.8 operator-() [2/2]

```
template<typename LabelT = std::string, typename NodeIDT = unsigned int, typename EdgeT =
unsigned long long int>
difference_type LinkPred::DNetwork< LabelT, NodeIDT, EdgeT >::NodeDegIt::operator- (
            const NodeDegIt & that ) const [inline]
```

Difference between the present iterator and the one passed as parameter.

Parameters

that	The other iterator.

Returns

The difference between the current and that iterator.

9.72.4.9 operator--() [1/2]

```
template<typename LabelT = std::string, typename NodeIDT = unsigned int, typename EdgeT =
unsigned long long int>
NodeDegIt& LinkPred::DNetwork< LabelT, NodeIDT, EdgeT >::NodeDegIt::operator-- ( )  [inline]
```

Pre-decrement operator.

Returns

A reference to the new iterator.

9.72.4.10 operator--() [2/2]

```
template<typename LabelT = std::string, typename NodeIDT = unsigned int, typename EdgeT =
unsigned long long int>
NodeDegIt LinkPred::DNetwork< LabelT, NodeIDT, EdgeT >::NodeDegIt::operator-- (
             int  )  [inline]
```

Post-decrement operator.

Returns

A reference to the new iterator.

9.72.4.11 operator-=()

```
template<typename LabelT = std::string, typename NodeIDT = unsigned int, typename EdgeT =
unsigned long long int>
NodeDegIt& LinkPred::DNetwork< LabelT, NodeIDT, EdgeT >::NodeDegIt::operator-= (
             const difference_type & n )  [inline]
```

Arithmetic -= operator.

Parameters

n	Decrement value.

Returns

A reference to the new iterator.

9.72.4.12 operator->()

```
template<typename LabelT = std::string, typename NodeIDT = unsigned int, typename EdgeT =
unsigned long long int>
pointer LinkPred::DNetwork< LabelT, NodeIDT, EdgeT >::NodeDegIt::operator-> ( ) [inline]
```

Arrow operator.

Returns

A pointer to the object to which the iterator points.

9.72.4.13 operator<()

```
template<typename LabelT = std::string, typename NodeIDT = unsigned int, typename EdgeT =
unsigned long long int>
bool LinkPred::DNetwork< LabelT, NodeIDT, EdgeT >::NodeDegIt::operator< (
            const NodeDegIt & that ) const [inline]
```

Parameters

that	The other iterator.

Returns

True if this is less than that.

9.72.4.14 operator<=()

```
template<typename LabelT = std::string, typename NodeIDT = unsigned int, typename EdgeT =
unsigned long long int>
bool LinkPred::DNetwork< LabelT, NodeIDT, EdgeT >::NodeDegIt::operator<= (
            const NodeDegIt & that ) const [inline]
```

Parameters

that	The other iterator.

Returns

True if this is greater or equal to that.

9.72.4.15 operator=() [1/2]

```
template<typename LabelT = std::string, typename NodeIDT = unsigned int, typename EdgeT =
unsigned long long int>
NodeDegIt& LinkPred::DNetwork< LabelT, NodeIDT, EdgeT >::NodeDegIt::operator= (
            NodeDegIt && that )  [default]
```

Move assignment operator.

Parameters

that	The object to move.

9.72.4.16 operator=() [2/2]

```
template<typename LabelT = std::string, typename NodeIDT = unsigned int, typename EdgeT =
unsigned long long int>
NodeDegIt& LinkPred::DNetwork< LabelT, NodeIDT, EdgeT >::NodeDegIt::operator= (
            NodeDegIt const & that )  [default]
```

Copy assignment operator.

Parameters

that	The object to copy.

9.72.4.17 operator==()

```
template<typename LabelT = std::string, typename NodeIDT = unsigned int, typename EdgeT =
unsigned long long int>
bool LinkPred::DNetwork< LabelT, NodeIDT, EdgeT >::NodeDegIt::operator== (
            const NodeDegIt & that ) const  [inline]
```

Parameters

that	The other iterator.

Returns

True if this equals that.

9.72.4.18 operator>()

```
template<typename LabelT = std::string, typename NodeIDT = unsigned int, typename EdgeT =
unsigned long long int>
bool LinkPred::DNetwork< LabelT, NodeIDT, EdgeT >::NodeDegIt::operator> (
            const NodeDegIt & that ) const  [inline]
```

Parameters

that	The other iterator.

Returns

True if this is greater that.

9.72.4.19 operator>=()

```
template<typename LabelT = std::string, typename NodeIDT = unsigned int, typename EdgeT =
unsigned long long int>
bool LinkPred::DNetwork< LabelT, NodeIDT, EdgeT >::NodeDegIt::operator>= (
            const NodeDegIt & that ) const  [inline]
```

Parameters

that	The other iterator.

Returns

True if this is less or equal to that.

9.72.5 Friends And Related Function Documentation

9.72.5.1 DNetwork

```
template<typename LabelT = std::string, typename NodeIDT = unsigned int, typename EdgeT =
unsigned long long int>
friend class DNetwork  [friend]
```

DNetwork is a friend.

The documentation for this class was generated from the following file:

- include/linkpred/core/dnetwork/dnetwork.hpp

9.73 LinkPred::UNetwork< LabelT, NodeIDT, EdgeT >::NodeDegIt Class Reference

Node-degree iterator. This class can be used to iterate over pairs of node IDs and degrees.

```
#include <unetwork.hpp>
```

Inheritance diagram for LinkPred::UNetwork< LabelT, NodeIDT, EdgeT >::NodeDegIt:

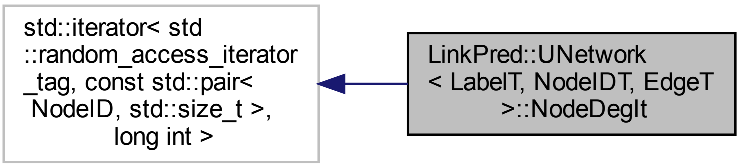

Collaboration diagram for LinkPred::UNetwork< LabelT, NodeIDT, EdgeT >::NodeDegIt:

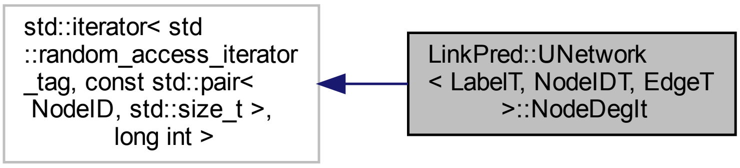

Public Types

- using pointer = typename std::iterator< std::random_access_iterator_tag, const std::pair< NodeID, std::size_t >, long int >::pointer
- using reference = typename std::iterator< std::random_access_iterator_tag, const std::pair< NodeID, std::size_t >, long int >::reference
- using difference_type = typename std::iterator< std::random_access_iterator_tag, const std::pair< NodeID, std::size_t >, long int >::difference_type

Public Member Functions

- NodeDegIt (NodeDegIt const &that)=default
- NodeDegIt & operator= (NodeDegIt const &that)=default
- NodeDegIt (NodeDegIt &&that)=default
- NodeDegIt & operator= (NodeDegIt &&that)=default

- reference operator* ()
- pointer operator-> ()
- NodeDegIt & operator++ ()
- NodeDegIt & operator-- ()
- NodeDegIt operator++ (int)
- NodeDegIt operator-- (int)
- NodeDegIt operator+ (const difference_type &n) const
- NodeDegIt & operator+= (const difference_type &n)
- NodeDegIt operator- (const difference_type &n) const
- NodeDegIt & operator-= (const difference_type &n)
- difference_type operator- (const NodeDegIt &that) const
- bool operator== (const NodeDegIt &that) const
- bool operator!= (const NodeDegIt &that) const
- bool operator< (const NodeDegIt &that) const
- bool operator> (const NodeDegIt &that) const
- bool operator<= (const NodeDegIt &that) const
- bool operator>= (const NodeDegIt &that) const

Friends

- class UNetwork

9.73.1 Detailed Description

template<typename LabelT = std::string, typename NodeIDT = unsigned int, typename EdgeT = unsigned long long int>
class LinkPred::UNetwork< LabelT, NodeIDT, EdgeT >::NodeDegIt

Node-degree iterator. This class can be used to iterate over pairs of node IDs and degrees.

9.73.2 Member Typedef Documentation

9.73.2.1 difference_type

```
template<typename LabelT = std::string, typename NodeIDT = unsigned int, typename EdgeT =
unsigned long long int>
using LinkPred::UNetwork< LabelT, NodeIDT, EdgeT >::NodeDegIt::difference_type = typename
std::iterator<std::random_access_iterator_tag, const std::pair<NodeID, std::size_t>, long
int>::difference_type
```

The difference type associated with the iterator.

9.73.2.2 pointer

```
template<typename LabelT = std::string, typename NodeIDT = unsigned int, typename EdgeT =
unsigned long long int>
using LinkPred::UNetwork< LabelT, NodeIDT, EdgeT >::NodeDegIt::pointer = typename std::iterator<std↩
::random_access_iterator_tag, const std::pair<NodeID, std::size_t>, long int>::pointer
```

The pointer type associated with the iterator.

9.73.2.3 reference

```
template<typename LabelT = std::string, typename NodeIDT = unsigned int, typename EdgeT =
unsigned long long int>
using LinkPred::UNetwork< LabelT, NodeIDT, EdgeT >::NodeDegIt::reference = typename std↩
::iterator<std::random_access_iterator_tag, const std::pair<NodeID, std::size_t>, long int>↩
::reference
```

The reference type associated with the iterator.

9.73.3 Constructor & Destructor Documentation

9.73.3.1 NodeDegIt() [1/2]

```
template<typename LabelT = std::string, typename NodeIDT = unsigned int, typename EdgeT =
unsigned long long int>
LinkPred::UNetwork< LabelT, NodeIDT, EdgeT >::NodeDegIt::NodeDegIt (
            NodeDegIt const & that )  [default]
```

Copy constructor.

Parameters

that	The object to copy.

9.73.3.2 NodeDegIt() [2/2]

```
template<typename LabelT = std::string, typename NodeIDT = unsigned int, typename EdgeT =
unsigned long long int>
LinkPred::UNetwork< LabelT, NodeIDT, EdgeT >::NodeDegIt::NodeDegIt (
            NodeDegIt && that )  [default]
```

Move constructor.

Parameters

that	The object to move.

9.73.4 Member Function Documentation

9.73.4.1 operator"!=()

```
template<typename LabelT = std::string, typename NodeIDT = unsigned int, typename EdgeT =
unsigned long long int>
bool LinkPred::UNetwork< LabelT, NodeIDT, EdgeT >::NodeDegIt::operator!= (
            const NodeDegIt & that ) const  [inline]
```

Parameters

that	The other iterator.

Returns

True if this is not equal to that.

9.73.4.2 operator*()

```
template<typename LabelT = std::string, typename NodeIDT = unsigned int, typename EdgeT =
unsigned long long int>
reference LinkPred::UNetwork< LabelT, NodeIDT, EdgeT >::NodeDegIt::operator* ( )  [inline]
```

Dereference operator.

Returns

A reference to the object to which the iterator points.

9.73.4.3 operator+()

```
template<typename LabelT = std::string, typename NodeIDT = unsigned int, typename EdgeT =
unsigned long long int>
NodeDegIt LinkPred::UNetwork< LabelT, NodeIDT, EdgeT >::NodeDegIt::operator+ (
            const difference_type & n ) const  [inline]
```

Arithmetic + operator.

Parameters

n	Increment value.

Returns

The new iterator.

9.73.4.4 operator++() [1/2]

```
template<typename LabelT = std::string, typename NodeIDT = unsigned int, typename EdgeT =
unsigned long long int>
NodeDegIt& LinkPred::UNetwork< LabelT, NodeIDT, EdgeT >::NodeDegIt::operator++ ( )  [inline]
```

Pre-increment operator.

Returns

A reference to the new iterator.

9.73.4.5 operator++() [2/2]

```
template<typename LabelT = std::string, typename NodeIDT = unsigned int, typename EdgeT =
unsigned long long int>
NodeDegIt LinkPred::UNetwork< LabelT, NodeIDT, EdgeT >::NodeDegIt::operator++ (
             int  )  [inline]
```

Post-increment operator.

Returns

A reference to the new iterator.

9.73.4.6 operator+=()

```
template<typename LabelT = std::string, typename NodeIDT = unsigned int, typename EdgeT =
unsigned long long int>
NodeDegIt& LinkPred::UNetwork< LabelT, NodeIDT, EdgeT >::NodeDegIt::operator+= (
             const difference_type & n )  [inline]
```

Arithmetic += operator.

Parameters

n	Increment value.

Returns

A reference to the new iterator.

9.73.4.7 operator-() [1/2]

```
template<typename LabelT = std::string, typename NodeIDT = unsigned int, typename EdgeT =
unsigned long long int>
NodeDegIt LinkPred::UNetwork< LabelT, NodeIDT, EdgeT >::NodeDegIt::operator- (
            const difference_type & n ) const  [inline]
```

Arithmetic - operator.

Parameters

n	Decrement value.

Returns

The new iterator.

9.73.4.8 operator-() [2/2]

```
template<typename LabelT = std::string, typename NodeIDT = unsigned int, typename EdgeT =
unsigned long long int>
difference_type LinkPred::UNetwork< LabelT, NodeIDT, EdgeT >::NodeDegIt::operator- (
            const NodeDegIt & that ) const  [inline]
```

Difference between the present iterator and the one passed as parameter.

Parameters

that	The other iterator.

Returns

The difference between the current and that iterator.

9.73.4.9 operator--() [1/2]

```
template<typename LabelT = std::string, typename NodeIDT = unsigned int, typename EdgeT =
unsigned long long int>
NodeDegIt& LinkPred::UNetwork< LabelT, NodeIDT, EdgeT >::NodeDegIt::operator-- ( )  [inline]
```

Pre-decrement operator.

Returns

A reference to the new iterator.

9.73.4.10 operator--() [2/2]

```
template<typename LabelT = std::string, typename NodeIDT = unsigned int, typename EdgeT =
unsigned long long int>
NodeDegIt LinkPred::UNetwork< LabelT, NodeIDT, EdgeT >::NodeDegIt::operator-- (
             int ) [inline]
```

Post-decrement operator.

Returns

A reference to the new iterator.

9.73.4.11 operator-=()

```
template<typename LabelT = std::string, typename NodeIDT = unsigned int, typename EdgeT =
unsigned long long int>
NodeDegIt& LinkPred::UNetwork< LabelT, NodeIDT, EdgeT >::NodeDegIt::operator-= (
             const difference_type & n ) [inline]
```

Arithmetic -= operator.

Parameters

n	Decrement value.

Returns

A reference to the new iterator.

9.73.4.12 operator->()

```
template<typename LabelT = std::string, typename NodeIDT = unsigned int, typename EdgeT =
unsigned long long int>
pointer LinkPred::UNetwork< LabelT, NodeIDT, EdgeT >::NodeDegIt::operator-> ( ) [inline]
```

Arrow operator.

Returns

A pointer to the object to which the iterator points.

9.73.4.13 operator<()

```
template<typename LabelT = std::string, typename NodeIDT = unsigned int, typename EdgeT =
unsigned long long int>
bool LinkPred::UNetwork< LabelT, NodeIDT, EdgeT >::NodeDegIt::operator< (
             const NodeDegIt & that ) const [inline]
```

Parameters

that	The other iterator.

Returns

True if this is less than that.

9.73.4.14 operator<=()

```
template<typename LabelT = std::string, typename NodeIDT = unsigned int, typename EdgeT =
unsigned long long int>
bool LinkPred::UNetwork< LabelT, NodeIDT, EdgeT >::NodeDegIt::operator<= (
            const NodeDegIt & that ) const  [inline]
```

Parameters

that	The other iterator.

Returns

True if this is greater or equal to that.

9.73.4.15 operator=() [1/2]

```
template<typename LabelT = std::string, typename NodeIDT = unsigned int, typename EdgeT =
unsigned long long int>
NodeDegIt& LinkPred::UNetwork< LabelT, NodeIDT, EdgeT >::NodeDegIt::operator= (
            NodeDegIt && that )  [default]
```

Move assignment operator.

Parameters

that	The object to move.

9.73.4.16 operator=() [2/2]

```
template<typename LabelT = std::string, typename NodeIDT = unsigned int, typename EdgeT =
unsigned long long int>
```

```
NodeDegIt& LinkPred::UNetwork< LabelT, NodeIDT, EdgeT >::NodeDegIt::operator= (
            NodeDegIt const & that )  [default]
```

Copy assignment operator.

Parameters

that	The object to copy.

9.73.4.17 operator==()

```
template<typename LabelT = std::string, typename NodeIDT = unsigned int, typename EdgeT =
unsigned long long int>
bool LinkPred::UNetwork< LabelT, NodeIDT, EdgeT >::NodeDegIt::operator== (
            const NodeDegIt & that ) const [inline]
```

Parameters

that	The other iterator.

Returns

True if this equals that.

9.73.4.18 operator>()

```
template<typename LabelT = std::string, typename NodeIDT = unsigned int, typename EdgeT =
unsigned long long int>
bool LinkPred::UNetwork< LabelT, NodeIDT, EdgeT >::NodeDegIt::operator> (
            const NodeDegIt & that ) const [inline]
```

Parameters

that	The other iterator.

Returns

True if this is greater that.

9.73.4.19 operator>=()

```
template<typename LabelT = std::string, typename NodeIDT = unsigned int, typename EdgeT =
unsigned long long int>
bool LinkPred::UNetwork< LabelT, NodeIDT, EdgeT >::NodeDegIt::operator>= (
            const NodeDegIt & that ) const [inline]
```

Parameters

that	The other iterator.

Returns

True if this is less or equal to that.

9.73.5 Friends And Related Function Documentation

9.73.5.1 UNetwork

```
template<typename LabelT = std::string, typename NodeIDT = unsigned int, typename EdgeT =
unsigned long long int>
friend class UNetwork [friend]
```

UNetwork is a friend.

The documentation for this class was generated from the following file:

- include/linkpred/core/unetwork/unetwork.hpp

9.74 LinkPred::UNetwork< LabelT, NodeIDT, EdgeT >::NodeMap< ValueType > Class Template Reference

A node map.

```
#include <unetwork.hpp>
```

Public Types

- using NodeMapIt = typename std::vector< ValueType >::iterator
- using NodeMapConstIt = typename std::vector< ValueType >::const_iterator

Public Member Functions

- NodeMap (NodeMap const &that)=default
- NodeMap & operator= (NodeMap const &that)=default
- NodeMap (NodeMap &&that)=default
- NodeMap & operator= (NodeMap &&that)=default
- ValueType operator[ ] (NodeID const &i) const
- ValueType & operator[ ] (NodeID const &i)
- ValueType at (NodeID const &i) const
- NodeMapIt begin ()
- NodeMapIt end ()
- NodeMapConstIt cbegin () const
- NodeMapConstIt cend () const
- ∼NodeMap ()=default

Friends

- class **UNetwork**

9.74.1 Detailed Description

template<typename LabelT = std::string, typename NodeIDT = unsigned int, typename EdgeT = unsigned long long int>
template<typename ValueType>
class LinkPred::UNetwork< LabelT, NodeIDT, EdgeT >::NodeMap< ValueType >

A node map.

This class can be used to assign a value to every node in the network. Access to values is done in constant time.

Template Parameters

ValueType	Type of mapped values.

9.74.2 Member Typedef Documentation

9.74.2.1 NodeMapConstIt

```
template<typename LabelT = std::string, typename NodeIDT = unsigned int, typename EdgeT =
unsigned long long int>
template<typename ValueType >
using LinkPred::UNetwork< LabelT, NodeIDT, EdgeT >::NodeMap< ValueType >::NodeMapConstIt =
typename std::vector<ValueType>::const_iterator
```

A constant iterator on the map values.

9.74.2.2 NodeMapIt

```
template<typename LabelT = std::string, typename NodeIDT = unsigned int, typename EdgeT =
unsigned long long int>
template<typename ValueType >
using LinkPred::UNetwork< LabelT, NodeIDT, EdgeT >::NodeMap< ValueType >::NodeMapIt = typename
std::vector<ValueType>::iterator
```

Iterator on the map values.

9.74.3 Constructor & Destructor Documentation

9.74.3.1 NodeMap() [1/2]

```
template<typename LabelT = std::string, typename NodeIDT = unsigned int, typename EdgeT =
unsigned long long int>
template<typename ValueType >
LinkPred::UNetwork< LabelT, NodeIDT, EdgeT >::NodeMap< ValueType >::NodeMap (
            NodeMap< ValueType > const & that )  [default]
```

Copy constructor.

Parameters

that	The object to copy.

9.74.3.2 NodeMap() [2/2]

```
template<typename LabelT = std::string, typename NodeIDT = unsigned int, typename EdgeT =
unsigned long long int>
template<typename ValueType >
LinkPred::UNetwork< LabelT, NodeIDT, EdgeT >::NodeMap< ValueType >::NodeMap (
            NodeMap< ValueType > && that )  [default]
```

Move constructor.

Parameters

that	The object to move.

9.74.3.3 ∼NodeMap()

```
template<typename LabelT = std::string, typename NodeIDT = unsigned int, typename EdgeT =
unsigned long long int>
template<typename ValueType >
LinkPred::UNetwork< LabelT, NodeIDT, EdgeT >::NodeMap< ValueType >::~NodeMap ( )  [default]
```

Destructor.

9.74.4 Member Function Documentation

9.74.4.1 at()

```
template<typename LabelT = std::string, typename NodeIDT = unsigned int, typename EdgeT =
unsigned long long int>
template<typename ValueType >
ValueType LinkPred::UNetwork< LabelT, NodeIDT, EdgeT >::NodeMap< ValueType >::at (
            NodeID const & i ) const  [inline]
```

Parameters

i	A node ID.

Returns

The value associated with the node i.

9.74.4.2 begin()

```
template<typename LabelT = std::string, typename NodeIDT = unsigned int, typename EdgeT =
unsigned long long int>
template<typename ValueType >
NodeMapIt LinkPred::UNetwork< LabelT, NodeIDT, EdgeT >::NodeMap< ValueType >::begin ( )  [inline]
```

Returns

An iterator to the first element in the map.

9.74.4.3 cbegin()

```
template<typename LabelT = std::string, typename NodeIDT = unsigned int, typename EdgeT =
unsigned long long int>
template<typename ValueType >
NodeMapConstIt LinkPred::UNetwork< LabelT, NodeIDT, EdgeT >::NodeMap< ValueType >::cbegin ( )
const  [inline]
```

Returns

A constant iterator to the first element in the map.

9.74.4.4 cend()

```
template<typename LabelT = std::string, typename NodeIDT = unsigned int, typename EdgeT =
unsigned long long int>
template<typename ValueType >
NodeMapConstIt LinkPred::UNetwork< LabelT, NodeIDT, EdgeT >::NodeMap< ValueType >::cend ( )
const  [inline]
```

Returns

A constant iterator to one-past-the-last element in the map.

9.74.4.5 end()

```
template<typename LabelT = std::string, typename NodeIDT = unsigned int, typename EdgeT =
unsigned long long int>
template<typename ValueType >
NodeMapIt LinkPred::UNetwork< LabelT, NodeIDT, EdgeT >::NodeMap< ValueType >::end ( ) [inline]
```

Returns

An iterator to one-past-the-last element in the map.

9.74.4.6 operator=() [1/2]

```
template<typename LabelT = std::string, typename NodeIDT = unsigned int, typename EdgeT =
unsigned long long int>
template<typename ValueType >
NodeMap& LinkPred::UNetwork< LabelT, NodeIDT, EdgeT >::NodeMap< ValueType >::operator= (
            NodeMap< ValueType > && that ) [default]
```

Move assignment operator.

Parameters

that	The object to move.

9.74.4.7 operator=() [2/2]

```
template<typename LabelT = std::string, typename NodeIDT = unsigned int, typename EdgeT =
unsigned long long int>
template<typename ValueType >
NodeMap& LinkPred::UNetwork< LabelT, NodeIDT, EdgeT >::NodeMap< ValueType >::operator= (
            NodeMap< ValueType > const & that ) [default]
```

Copy assignment operator.

Parameters

that	The object to copy.

9.74.4.8 operator[]() [1/2]

```
template<typename LabelT = std::string, typename NodeIDT = unsigned int, typename EdgeT =
unsigned long long int>
```

```
template<typename ValueType >
ValueType& LinkPred::UNetwork< LabelT, NodeIDT, EdgeT >::NodeMap< ValueType >::operator[] (
            NodeID const & i ) [inline]
```

Parameters

i	A node ID.

Returns

A reference to the value associated with the node i.

9.74.4.9 operator[]() [2/2]

```
template<typename LabelT = std::string, typename NodeIDT = unsigned int, typename EdgeT =
unsigned long long int>
template<typename ValueType >
ValueType LinkPred::UNetwork< LabelT, NodeIDT, EdgeT >::NodeMap< ValueType >::operator[] (
            NodeID const & i ) const [inline]
```

Parameters

i	A node ID.

Returns

The value associated with the node i.

The documentation for this class was generated from the following file:

- include/linkpred/core/unetwork/unetwork.hpp

9.75 LinkPred::DNetwork< LabelT, NodeIDT, EdgeT >::NodeMap< ValueType > Class Template Reference

A node map.

```
#include <dnetwork.hpp>
```

Public Types

- using NodeMapIt = typename std::vector< ValueType >::iterator
- using NodeMapConstIt = typename std::vector< ValueType >::const_iterator

Public Member Functions

- NodeMap (NodeMap const &that)=default
- NodeMap & operator= (NodeMap const &that)=default
- NodeMap (NodeMap &&that)=default
- NodeMap & operator= (NodeMap &&that)=default
- ValueType operator[ ] (NodeID const &i) const
- ValueType & operator[ ] (NodeID const &i)
- ValueType at (NodeID const &i) const
- NodeMapIt begin ()
- NodeMapIt end ()
- NodeMapConstIt cbegin () const
- NodeMapConstIt cend () const
- ~NodeMap ()=default

Friends

- class **DNetwork**

9.75.1 Detailed Description

template<typename LabelT = std::string, typename NodeIDT = unsigned int, typename EdgeT = unsigned long long int>
template<typename ValueType>
class LinkPred::DNetwork< LabelT, NodeIDT, EdgeT >::NodeMap< ValueType >

A node map.

This class can be used to assign a value to every node in the network. Access to values is done in constant time.

Template Parameters

ValueType	Type of mapped values.

9.75.2 Member Typedef Documentation

9.75.2.1 NodeMapConstIt

```
template<typename LabelT = std::string, typename NodeIDT = unsigned int, typename EdgeT =
unsigned long long int>
template<typename ValueType >
using LinkPred::DNetwork< LabelT, NodeIDT, EdgeT >::NodeMap< ValueType >::NodeMapConstIt =
typename std::vector<ValueType>::const_iterator
```

A constant iterator on the map values.

9.75.2.2 NodeMapIt

```
template<typename LabelT = std::string, typename NodeIDT = unsigned int, typename EdgeT =
unsigned long long int>
template<typename ValueType >
using LinkPred::DNetwork< LabelT, NodeIDT, EdgeT >::NodeMap< ValueType >::NodeMapIt = typename
std::vector<ValueType>::iterator
```

Iterator on the map values.

9.75.3 Constructor & Destructor Documentation

9.75.3.1 NodeMap() [1/2]

```
template<typename LabelT = std::string, typename NodeIDT = unsigned int, typename EdgeT =
unsigned long long int>
template<typename ValueType >
LinkPred::DNetwork< LabelT, NodeIDT, EdgeT >::NodeMap< ValueType >::NodeMap (
            NodeMap< ValueType > const & that )  [default]
```

Copy constructor.

Parameters

that	The object to copy.

9.75.3.2 NodeMap() [2/2]

```
template<typename LabelT = std::string, typename NodeIDT = unsigned int, typename EdgeT =
unsigned long long int>
template<typename ValueType >
LinkPred::DNetwork< LabelT, NodeIDT, EdgeT >::NodeMap< ValueType >::NodeMap (
            NodeMap< ValueType > && that )  [default]
```

Move constructor.

Parameters

that	The object to move.

9.75.3.3 ~NodeMap()

```
template<typename LabelT = std::string, typename NodeIDT = unsigned int, typename EdgeT =
```

```
unsigned long long int>
template<typename ValueType >
LinkPred::DNetwork< LabelT, NodeIDT, EdgeT >::NodeMap< ValueType >::~NodeMap ( ) [default]
```

Destructor.

9.75.4 Member Function Documentation

9.75.4.1 at()

```
template<typename LabelT = std::string, typename NodeIDT = unsigned int, typename EdgeT =
unsigned long long int>
template<typename ValueType >
ValueType LinkPred::DNetwork< LabelT, NodeIDT, EdgeT >::NodeMap< ValueType >::at (
            NodeID const & i ) const [inline]
```

Parameters

i	A node ID.

Returns

The value associated with the node i.

9.75.4.2 begin()

```
template<typename LabelT = std::string, typename NodeIDT = unsigned int, typename EdgeT =
unsigned long long int>
template<typename ValueType >
NodeMapIt LinkPred::DNetwork< LabelT, NodeIDT, EdgeT >::NodeMap< ValueType >::begin ( ) [inline]
```

Returns

An iterator to the first element in the map.

9.75.4.3 cbegin()

```
template<typename LabelT = std::string, typename NodeIDT = unsigned int, typename EdgeT =
unsigned long long int>
template<typename ValueType >
NodeMapConstIt LinkPred::DNetwork< LabelT, NodeIDT, EdgeT >::NodeMap< ValueType >::cbegin ( )
const [inline]
```

Returns

A constant iterator to the first element in the map.

9.75.4.4 cend()

```
template<typename LabelT = std::string, typename NodeIDT = unsigned int, typename EdgeT =
unsigned long long int>
template<typename ValueType >
NodeMapConstIt LinkPred::DNetwork< LabelT, NodeIDT, EdgeT >::NodeMap< ValueType >::cend ( )
const [inline]
```

Returns

A constant iterator to one-past-the-last element in the map.

9.75.4.5 end()

```
template<typename LabelT = std::string, typename NodeIDT = unsigned int, typename EdgeT =
unsigned long long int>
template<typename ValueType >
NodeMapIt LinkPred::DNetwork< LabelT, NodeIDT, EdgeT >::NodeMap< ValueType >::end ( ) [inline]
```

Returns

An iterator to one-past-the-last element in the map.

9.75.4.6 operator=() [1/2]

```
template<typename LabelT = std::string, typename NodeIDT = unsigned int, typename EdgeT =
unsigned long long int>
template<typename ValueType >
NodeMap& LinkPred::DNetwork< LabelT, NodeIDT, EdgeT >::NodeMap< ValueType >::operator= (
            NodeMap< ValueType > && that ) [default]
```

Move assignment operator.

Parameters

that	The object to move.

9.75.4.7 operator=() [2/2]

```
template<typename LabelT = std::string, typename NodeIDT = unsigned int, typename EdgeT =
unsigned long long int>
template<typename ValueType >
```

```
NodeMap& LinkPred::DNetwork< LabelT, NodeIDT, EdgeT >::NodeMap< ValueType >::operator= (
            NodeMap< ValueType > const & that )  [default]
```

Copy assignment operator.

Parameters

that	The object to copy.

9.75.4.8 operator[]() [1/2]

```
template<typename LabelT = std::string, typename NodeIDT = unsigned int, typename EdgeT =
unsigned long long int>
template<typename ValueType >
ValueType& LinkPred::DNetwork< LabelT, NodeIDT, EdgeT >::NodeMap< ValueType >::operator[] (
            NodeID const & i )  [inline]
```

Parameters

i	A node ID.

Returns

A reference to the value associated with the node i.

9.75.4.9 operator[]() [2/2]

```
template<typename LabelT = std::string, typename NodeIDT = unsigned int, typename EdgeT =
unsigned long long int>
template<typename ValueType >
ValueType LinkPred::DNetwork< LabelT, NodeIDT, EdgeT >::NodeMap< ValueType >::operator[] (
            NodeID const & i ) const  [inline]
```

Parameters

i	A node ID.

Returns

The value associated with the node i.

The documentation for this class was generated from the following file:

- include/linkpred/core/dnetwork/dnetwork.hpp

9.76 LinkPred::DNetwork< LabelT, NodeIDT, EdgeT >::NodeSMap< ValueType > Class Template Reference

A sparse node map.

```
#include <dnetwork.hpp>
```

Public Types

- using NodeSMapIterator = typename std::map< NodeID, ValueType >::iterator
- using NodeSMapConstIterator = typename std::map< NodeID, ValueType >::const_iterator

Public Member Functions

- NodeSMap (NodeSMap const &that)=default
- NodeSMap & operator= (NodeSMap const &that)=default
- NodeSMap (NodeSMap &&that)=default
- NodeSMap & operator= (NodeSMap &&that)=default
- auto find (NodeID const &i)
- ValueType operator[ ] (NodeID const &i) const
- ValueType & operator[ ] (NodeID const &i)
- ValueType at (NodeID const &i) const
- NodeSMapIterator begin ()
- NodeSMapIterator end ()
- NodeSMapConstIterator cbegin () const
- NodeSMapConstIterator cend () const
- std::size_t size () const
- ~NodeSMap ()=default

Friends

- class **DNetwork**

9.76.1 Detailed Description

template<typename LabelT = std::string, typename NodeIDT = unsigned int, typename EdgeT = unsigned long long int>
template<typename ValueType>
class LinkPred::DNetwork< LabelT, NodeIDT, EdgeT >::NodeSMap< ValueType >

A sparse node map.

This class can be used to assign a value to every node in the network. Access to values is done in constant time.

Template Parameters

ValueType	Type of mapped values.

9.76.2 Member Typedef Documentation

9.76.2.1 NodeSMapConstIterator

```
template<typename LabelT = std::string, typename NodeIDT = unsigned int, typename EdgeT =
unsigned long long int>
template<typename ValueType >
using LinkPred::DNetwork< LabelT, NodeIDT, EdgeT >::NodeSMap< ValueType >::NodeSMapConstIterator
= typename std::map<NodeID, ValueType>::const_iterator
```

A constant iterator on the map values.

9.76.2.2 NodeSMapIterator

```
template<typename LabelT = std::string, typename NodeIDT = unsigned int, typename EdgeT =
unsigned long long int>
template<typename ValueType >
using LinkPred::DNetwork< LabelT, NodeIDT, EdgeT >::NodeSMap< ValueType >::NodeSMapIterator =
typename std::map<NodeID, ValueType>::iterator
```

Iterator on the map values.

9.76.3 Constructor & Destructor Documentation

9.76.3.1 NodeSMap() [1/2]

```
template<typename LabelT = std::string, typename NodeIDT = unsigned int, typename EdgeT =
unsigned long long int>
template<typename ValueType >
LinkPred::DNetwork< LabelT, NodeIDT, EdgeT >::NodeSMap< ValueType >::NodeSMap (
            NodeSMap< ValueType > const & that )  [default]
```

Copy constructor.

Parameters

that	The object to copy.

9.76.3.2 NodeSMap() [2/2]

```
template<typename LabelT = std::string, typename NodeIDT = unsigned int, typename EdgeT =
unsigned long long int>
```

```
template<typename ValueType >
LinkPred::DNetwork< LabelT, NodeIDT, EdgeT >::NodeSMap< ValueType >::NodeSMap (
            NodeSMap< ValueType > && that ) [default]
```

Move constructor.

Parameters

that	The object to move.

9.76.3.3 ∼NodeSMap()

```
template<typename LabelT = std::string, typename NodeIDT = unsigned int, typename EdgeT =
unsigned long long int>
template<typename ValueType >
LinkPred::DNetwork< LabelT, NodeIDT, EdgeT >::NodeSMap< ValueType >::~NodeSMap ( ) [default]
```

Destructor.

9.76.4 Member Function Documentation

9.76.4.1 at()

```
template<typename LabelT = std::string, typename NodeIDT = unsigned int, typename EdgeT =
unsigned long long int>
template<typename ValueType >
ValueType LinkPred::DNetwork< LabelT, NodeIDT, EdgeT >::NodeSMap< ValueType >::at (
            NodeID const & i ) const [inline]
```

Parameters

i	A node ID.

Returns

The value associated with the node i.

9.76.4.2 begin()

```
template<typename LabelT = std::string, typename NodeIDT = unsigned int, typename EdgeT =
unsigned long long int>
template<typename ValueType >
NodeSMapIterator LinkPred::DNetwork< LabelT, NodeIDT, EdgeT >::NodeSMap< ValueType >::begin (
) [inline]
```

Returns

An iterator to the first element in the map.

9.76.4.3 cbegin()

```
template<typename LabelT = std::string, typename NodeIDT = unsigned int, typename EdgeT =
unsigned long long int>
template<typename ValueType >
NodeSMapConstIterator LinkPred::DNetwork< LabelT, NodeIDT, EdgeT >::NodeSMap< ValueType >↩
::cbegin ( ) const  [inline]
```

Returns

A constant iterator to the first element in the map.

9.76.4.4 cend()

```
template<typename LabelT = std::string, typename NodeIDT = unsigned int, typename EdgeT =
unsigned long long int>
template<typename ValueType >
NodeSMapConstIterator LinkPred::DNetwork< LabelT, NodeIDT, EdgeT >::NodeSMap< ValueType >↩
::cend ( ) const  [inline]
```

Returns

A constant iterator to one-past-the-last element in the map.

9.76.4.5 end()

```
template<typename LabelT = std::string, typename NodeIDT = unsigned int, typename EdgeT =
unsigned long long int>
template<typename ValueType >
NodeSMapIterator LinkPred::DNetwork< LabelT, NodeIDT, EdgeT >::NodeSMap< ValueType >::end ( )
[inline]
```

Returns

An iterator to one-past-the-last element in the map.

9.76.4.6 find()

```
template<typename LabelT = std::string, typename NodeIDT = unsigned int, typename EdgeT =
unsigned long long int>
template<typename ValueType >
auto LinkPred::DNetwork< LabelT, NodeIDT, EdgeT >::NodeSMap< ValueType >::find (
            NodeID const & i )  [inline]
```

Parameters

i	A node ID.

Returns

An iterator to the element with key i.

9.76.4.7 operator=() [1/2]

```
template<typename LabelT = std::string, typename NodeIDT = unsigned int, typename EdgeT =
unsigned long long int>
template<typename ValueType >
NodeSMap& LinkPred::DNetwork< LabelT, NodeIDT, EdgeT >::NodeSMap< ValueType >::operator= (
            NodeSMap< ValueType > && that ) [default]
```

Move assignment operator.

Parameters

that	The object to move.

9.76.4.8 operator=() [2/2]

```
template<typename LabelT = std::string, typename NodeIDT = unsigned int, typename EdgeT =
unsigned long long int>
template<typename ValueType >
NodeSMap& LinkPred::DNetwork< LabelT, NodeIDT, EdgeT >::NodeSMap< ValueType >::operator= (
            NodeSMap< ValueType > const & that ) [default]
```

Copy assignment operator.

Parameters

that	The object to copy.

9.76.4.9 operator[]() [1/2]

```
template<typename LabelT = std::string, typename NodeIDT = unsigned int, typename EdgeT =
unsigned long long int>
template<typename ValueType >
ValueType& LinkPred::DNetwork< LabelT, NodeIDT, EdgeT >::NodeSMap< ValueType >::operator[] (
            NodeID const & i ) [inline]
```

Parameters

i	A node ID.

Returns

A reference to the value associated with the node i.

9.76.4.10 operator[]() [2/2]

```
template<typename LabelT = std::string, typename NodeIDT = unsigned int, typename EdgeT =
unsigned long long int>
template<typename ValueType >
ValueType LinkPred::DNetwork< LabelT, NodeIDT, EdgeT >::NodeSMap< ValueType >::operator[] (
            NodeID const & i ) const [inline]
```

Parameters

i	A node ID.

Returns

The value associated with the node i.

9.76.4.11 size()

```
template<typename LabelT = std::string, typename NodeIDT = unsigned int, typename EdgeT =
unsigned long long int>
template<typename ValueType >
std::size_t LinkPred::DNetwork< LabelT, NodeIDT, EdgeT >::NodeSMap< ValueType >::size ( )
const [inline]
```

Returns

The number of elements in the map (those that were explicitly inserted).

The documentation for this class was generated from the following file:

- include/linkpred/core/dnetwork/dnetwork.hpp

9.77 LinkPred::UNetwork< LabelT, NodeIDT, EdgeT >::NodeSMap< ValueType > Class Template Reference

A sparse node map.

```
#include <unetwork.hpp>
```

Public Types

- using NodeSMapIterator = typename std::map< NodeID, ValueType >::iterator
- using NodeSMapConstIterator = typename std::map< NodeID, ValueType >::const_iterator

Public Member Functions

- NodeSMap (NodeSMap const &that)=default
- NodeSMap & operator= (NodeSMap const &that)=default
- NodeSMap (NodeSMap &&that)=default
- NodeSMap & operator= (NodeSMap &&that)=default
- auto find (NodeID const &i)
- ValueType operator[ ] (NodeID const &i) const
- ValueType & operator[ ] (NodeID const &i)
- ValueType at (NodeID const &i) const
- NodeSMapIterator begin ()
- NodeSMapIterator end ()
- NodeSMapConstIterator cbegin () const
- NodeSMapConstIterator cend () const
- std::size_t size () const
- ∼NodeSMap ()=default

Friends

- class **UNetwork**

9.77.1 Detailed Description

template<typename LabelT = std::string, typename NodeIDT = unsigned int, typename EdgeT = unsigned long long int>
template<typename ValueType>
class LinkPred::UNetwork< LabelT, NodeIDT, EdgeT >::NodeSMap< ValueType >

A sparse node map.

This class can be used to assign a value to every node in the network. Access to values is done in constant time.

Template Parameters

ValueType	Type of mapped values.

9.77.2 Member Typedef Documentation

9.77.2.1 NodeSMapConstIterator

```
template<typename LabelT = std::string, typename NodeIDT = unsigned int, typename EdgeT =
unsigned long long int>
```

```
template<typename ValueType >
using LinkPred::UNetwork< LabelT, NodeIDT, EdgeT >::NodeSMap< ValueType >::NodeSMapConstIterator
= typename std::map<NodeID, ValueType>::const_iterator
```

A constant iterator on the map values.

9.77.2.2 NodeSMapIterator

```
template<typename LabelT = std::string, typename NodeIDT = unsigned int, typename EdgeT =
unsigned long long int>
template<typename ValueType >
using LinkPred::UNetwork< LabelT, NodeIDT, EdgeT >::NodeSMap< ValueType >::NodeSMapIterator =
typename std::map<NodeID, ValueType>::iterator
```

Iterator on the map values.

9.77.3 Constructor & Destructor Documentation

9.77.3.1 NodeSMap() [1/2]

```
template<typename LabelT = std::string, typename NodeIDT = unsigned int, typename EdgeT =
unsigned long long int>
template<typename ValueType >
LinkPred::UNetwork< LabelT, NodeIDT, EdgeT >::NodeSMap< ValueType >::NodeSMap (
            NodeSMap< ValueType > const & that )  [default]
```

Copy constructor.

Parameters

that	The object to copy.

9.77.3.2 NodeSMap() [2/2]

```
template<typename LabelT = std::string, typename NodeIDT = unsigned int, typename EdgeT =
unsigned long long int>
template<typename ValueType >
LinkPred::UNetwork< LabelT, NodeIDT, EdgeT >::NodeSMap< ValueType >::NodeSMap (
            NodeSMap< ValueType > && that )  [default]
```

Move constructor.

Parameters

that	The object to move.

9.77.3.3 ~NodeSMap()

```
template<typename LabelT = std::string, typename NodeIDT = unsigned int, typename EdgeT =
unsigned long long int>
template<typename ValueType >
LinkPred::UNetwork< LabelT, NodeIDT, EdgeT >::NodeSMap< ValueType >::~NodeSMap ( ) [default]
```

Destructor.

9.77.4 Member Function Documentation

9.77.4.1 at()

```
template<typename LabelT = std::string, typename NodeIDT = unsigned int, typename EdgeT =
unsigned long long int>
template<typename ValueType >
ValueType LinkPred::UNetwork< LabelT, NodeIDT, EdgeT >::NodeSMap< ValueType >::at (
            NodeID const & i ) const [inline]
```

Parameters

i	A node ID.

Returns

The value associated with the node i.

9.77.4.2 begin()

```
template<typename LabelT = std::string, typename NodeIDT = unsigned int, typename EdgeT =
unsigned long long int>
template<typename ValueType >
NodeSMapIterator LinkPred::UNetwork< LabelT, NodeIDT, EdgeT >::NodeSMap< ValueType >::begin (
) [inline]
```

Returns

An iterator to the first element in the map.

9.77.4.3 cbegin()

```
template<typename LabelT = std::string, typename NodeIDT = unsigned int, typename EdgeT =
unsigned long long int>
template<typename ValueType >
NodeSMapConstIterator LinkPred::UNetwork< LabelT, NodeIDT, EdgeT >::NodeSMap< ValueType >↩
::cbegin ( ) const [inline]
```

Returns

A constant iterator to the first element in the map.

9.77.4.4 cend()

```
template<typename LabelT = std::string, typename NodeIDT = unsigned int, typename EdgeT =
unsigned long long int>
template<typename ValueType >
NodeSMapConstIterator LinkPred::UNetwork< LabelT, NodeIDT, EdgeT >::NodeSMap< ValueType >↩
::cend ( ) const [inline]
```

Returns

A constant iterator to one-past-the-last element in the map.

9.77.4.5 end()

```
template<typename LabelT = std::string, typename NodeIDT = unsigned int, typename EdgeT =
unsigned long long int>
template<typename ValueType >
NodeSMapIterator LinkPred::UNetwork< LabelT, NodeIDT, EdgeT >::NodeSMap< ValueType >::end ( )
[inline]
```

Returns

An iterator to one-past-the-last element in the map.

9.77.4.6 find()

```
template<typename LabelT = std::string, typename NodeIDT = unsigned int, typename EdgeT =
unsigned long long int>
template<typename ValueType >
auto LinkPred::UNetwork< LabelT, NodeIDT, EdgeT >::NodeSMap< ValueType >::find (
            NodeID const & i ) [inline]
```

Parameters

i	A node ID.

Returns

An iterator to the element with key i.

9.77.4.7 operator=() [1/2]

```
template<typename LabelT = std::string, typename NodeIDT = unsigned int, typename EdgeT =
unsigned long long int>
template<typename ValueType >
NodeSMap& LinkPred::UNetwork< LabelT, NodeIDT, EdgeT >::NodeSMap< ValueType >::operator= (
            NodeSMap< ValueType > && that )  [default]
```

Move assignment operator.

Parameters

that	The object to move.

9.77.4.8 operator=() [2/2]

```
template<typename LabelT = std::string, typename NodeIDT = unsigned int, typename EdgeT =
unsigned long long int>
template<typename ValueType >
NodeSMap& LinkPred::UNetwork< LabelT, NodeIDT, EdgeT >::NodeSMap< ValueType >::operator= (
            NodeSMap< ValueType > const & that )  [default]
```

Copy assignment operator.

Parameters

that	The object to copy.

9.77.4.9 operator[]() [1/2]

```
template<typename LabelT = std::string, typename NodeIDT = unsigned int, typename EdgeT =
unsigned long long int>
template<typename ValueType >
ValueType& LinkPred::UNetwork< LabelT, NodeIDT, EdgeT >::NodeSMap< ValueType >::operator[] (
            NodeID const & i )  [inline]
```

Parameters

i	A node ID.

Returns

A reference to the value associated with the node i.

9.77.4.10 operator[]() [2/2]

```
template<typename LabelT = std::string, typename NodeIDT = unsigned int, typename EdgeT =
unsigned long long int>
template<typename ValueType >
ValueType LinkPred::UNetwork< LabelT, NodeIDT, EdgeT >::NodeSMap< ValueType >::operator[] (
            NodeID const & i ) const [inline]
```

Parameters

i	A node ID.

Returns

The value associated with the node i.

9.77.4.11 size()

```
template<typename LabelT = std::string, typename NodeIDT = unsigned int, typename EdgeT =
unsigned long long int>
template<typename ValueType >
std::size_t LinkPred::UNetwork< LabelT, NodeIDT, EdgeT >::NodeSMap< ValueType >::size ( )
const [inline]
```

Returns

The number of elements in the map (those that were explicitly inserted).

The documentation for this class was generated from the following file:

- include/linkpred/core/unetwork/unetwork.hpp

9.78 LinkPred::UNetwork< LabelT, NodeIDT, EdgeT >::NonEdgeIt Class Reference

Nonedges iterator.

`#include <unetwork.hpp>`

Inheritance diagram for LinkPred::UNetwork< LabelT, NodeIDT, EdgeT >::NonEdgeIt:

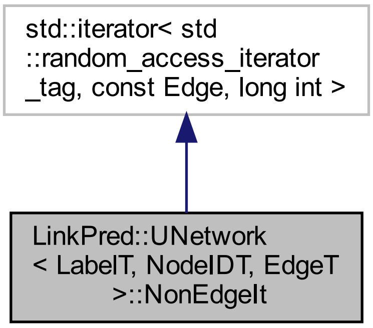

Collaboration diagram for LinkPred::UNetwork< LabelT, NodeIDT, EdgeT >::NonEdgeIt:

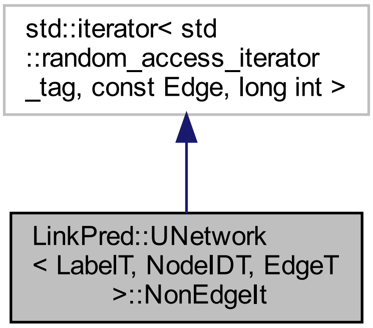

Public Types

- using pointer = typename std::iterator< std::random_access_iterator_tag, const Edge, long int >::pointer
- using reference = typename std::iterator< std::random_access_iterator_tag, const Edge, long int >↩ ::reference
- using difference_type = typename std::iterator< std::random_access_iterator_tag, const Edge, long int >↩ ::difference_type

Public Member Functions

- NonEdgeIt (NonEdgeIt const &that)=default
- NonEdgeIt & operator= (NonEdgeIt const &that)=default
- NonEdgeIt (NonEdgeIt &&that)=default
- NonEdgeIt & operator= (NonEdgeIt &&that)=default
- reference operator* ()
- pointer operator-> ()
- NonEdgeIt & operator++ ()
- NonEdgeIt & operator-- ()
- NonEdgeIt operator++ (int)
- NonEdgeIt operator-- (int)
- NonEdgeIt operator+ (const difference_type &n) const
- NonEdgeIt & operator+= (const difference_type &n)
- NonEdgeIt operator- (const difference_type &n) const
- NonEdgeIt & operator-= (const difference_type &n)
- difference_type operator- (const NonEdgeIt &that) const
- bool operator== (const NonEdgeIt &that) const
- bool operator!= (const NonEdgeIt &that) const
- bool operator< (const NonEdgeIt &that) const
- bool operator> (const NonEdgeIt &that) const
- bool operator<= (const NonEdgeIt &that) const
- bool operator>= (const NonEdgeIt &that) const

Friends

- class UNetwork

9.78.1 Detailed Description

**template<typename LabelT = std::string, typename NodeIDT = unsigned int, typename EdgeT = unsigned long long int>
class LinkPred::UNetwork< LabelT, NodeIDT, EdgeT >::NonEdgeIt**

Nonedges iterator.

9.78.2 Member Typedef Documentation

9.78.2.1 difference_type

```
template<typename LabelT = std::string, typename NodeIDT = unsigned int, typename EdgeT =
unsigned long long int>
using LinkPred::UNetwork< LabelT, NodeIDT, EdgeT >::NonEdgeIt::difference_type = typename
std::iterator<std::random_access_iterator_tag, const Edge, long int>::difference_type
```

The difference type associated with the iterator.

9.78.2.2 pointer

```
template<typename LabelT = std::string, typename NodeIDT = unsigned int, typename EdgeT =
unsigned long long int>
using LinkPred::UNetwork< LabelT, NodeIDT, EdgeT >::NonEdgeIt::pointer = typename std::iterator<std↩
::random_access_iterator_tag, const Edge, long int>::pointer
```

The pointer type associated with the iterator.

9.78.2.3 reference

```
template<typename LabelT = std::string, typename NodeIDT = unsigned int, typename EdgeT =
unsigned long long int>
using LinkPred::UNetwork< LabelT, NodeIDT, EdgeT >::NonEdgeIt::reference = typename std↩
::iterator<std::random_access_iterator_tag, const Edge, long int>::reference
```

The reference type associated with the iterator.

9.78.3 Constructor & Destructor Documentation

9.78.3.1 NonEdgeIt() [1/2]

```
template<typename LabelT = std::string, typename NodeIDT = unsigned int, typename EdgeT =
unsigned long long int>
LinkPred::UNetwork< LabelT, NodeIDT, EdgeT >::NonEdgeIt::NonEdgeIt (
            NonEdgeIt const & that )  [default]
```

Copy constructor.

Parameters

that	The object to copy.

9.78.3.2 NonEdgeIt() [2/2]

```
template<typename LabelT = std::string, typename NodeIDT = unsigned int, typename EdgeT =
unsigned long long int>
LinkPred::UNetwork< LabelT, NodeIDT, EdgeT >::NonEdgeIt::NonEdgeIt (
            NonEdgeIt && that )  [default]
```

Move constructor.

Parameters

that	The object to move.

9.78.4 Member Function Documentation

9.78.4.1 operator"!=()

```
template<typename LabelT = std::string, typename NodeIDT = unsigned int, typename EdgeT =
unsigned long long int>
bool LinkPred::UNetwork< LabelT, NodeIDT, EdgeT >::NonEdgeIt::operator!= (
            const NonEdgeIt & that ) const  [inline]
```

Parameters

that	The other iterator.

Returns

True if this is not equal to that.

9.78.4.2 operator∗()

```
template<typename LabelT = std::string, typename NodeIDT = unsigned int, typename EdgeT =
unsigned long long int>
reference LinkPred::UNetwork< LabelT, NodeIDT, EdgeT >::NonEdgeIt::operator* ( )  [inline]
```

Dereference operator.

Returns

A reference to the object to which the iterator points.

9.78.4.3 operator+()

```
template<typename LabelT = std::string, typename NodeIDT = unsigned int, typename EdgeT =
unsigned long long int>
NonEdgeIt LinkPred::UNetwork< LabelT, NodeIDT, EdgeT >::NonEdgeIt::operator+ (
            const difference_type & n ) const  [inline]
```

Arithmetic + operator.

Parameters

n	Increment value.

Returns

The new iterator.

9.78.4.4 operator++() [1/2]

```
template<typename LabelT = std::string, typename NodeIDT = unsigned int, typename EdgeT =
unsigned long long int>
NonEdgeIt& LinkPred::UNetwork< LabelT, NodeIDT, EdgeT >::NonEdgeIt::operator++ ( )  [inline]
```

Pre-increment operator.

Returns

A reference to the new iterator.

9.78.4.5 operator++() [2/2]

```
template<typename LabelT = std::string, typename NodeIDT = unsigned int, typename EdgeT =
unsigned long long int>
NonEdgeIt LinkPred::UNetwork< LabelT, NodeIDT, EdgeT >::NonEdgeIt::operator++ (
            int  )  [inline]
```

Post-increment operator.

Returns

A reference to the new iterator.

9.78.4.6 operator+=()

```
template<typename LabelT = std::string, typename NodeIDT = unsigned int, typename EdgeT =
unsigned long long int>
NonEdgeIt& LinkPred::UNetwork< LabelT, NodeIDT, EdgeT >::NonEdgeIt::operator+= (
            const difference_type & n )  [inline]
```

Arithmetic += operator.

Parameters

n	Increment value.

Returns

A reference to the new iterator.

9.78.4.7 operator-() [1/2]

```
template<typename LabelT = std::string, typename NodeIDT = unsigned int, typename EdgeT =
unsigned long long int>
NonEdgeIt LinkPred::UNetwork< LabelT, NodeIDT, EdgeT >::NonEdgeIt::operator- (
            const difference_type & n ) const [inline]
```

Arithmetic - operator.

Parameters

n	Decrement value.

Returns

The new iterator.

9.78.4.8 operator-() [2/2]

```
template<typename LabelT = std::string, typename NodeIDT = unsigned int, typename EdgeT =
unsigned long long int>
difference_type LinkPred::UNetwork< LabelT, NodeIDT, EdgeT >::NonEdgeIt::operator- (
            const NonEdgeIt & that ) const [inline]
```

Difference between the present iterator and the one passed as parameter.

Parameters

that	The other iterator.

Returns

The difference between the current and that iterator.

9.78.4.9 operator--() [1/2]

```
template<typename LabelT = std::string, typename NodeIDT = unsigned int, typename EdgeT =
unsigned long long int>
NonEdgeIt& LinkPred::UNetwork< LabelT, NodeIDT, EdgeT >::NonEdgeIt::operator-- ( ) [inline]
```

Pre-decrement operator.

Returns

A reference to the new iterator.

9.78.4.10 operator--() [2/2]

```
template<typename LabelT = std::string, typename NodeIDT = unsigned int, typename EdgeT =
unsigned long long int>
NonEdgeIt LinkPred::UNetwork< LabelT, NodeIDT, EdgeT >::NonEdgeIt::operator-- (
            int ) [inline]
```

Post-decrement operator.

Returns

A reference to the new iterator.

9.78.4.11 operator-=()

```
template<typename LabelT = std::string, typename NodeIDT = unsigned int, typename EdgeT =
unsigned long long int>
NonEdgeIt& LinkPred::UNetwork< LabelT, NodeIDT, EdgeT >::NonEdgeIt::operator-= (
            const difference_type & n ) [inline]
```

Arithmetic -= operator.

Parameters

n	Decrement value.

Returns

A reference to the new iterator.

9.78.4.12 operator->()

```
template<typename LabelT = std::string, typename NodeIDT = unsigned int, typename EdgeT =
unsigned long long int>
pointer LinkPred::UNetwork< LabelT, NodeIDT, EdgeT >::NonEdgeIt::operator-> ( ) [inline]
```

Arrow operator.

Returns

A pointer to the object to which the iterator points.

9.78.4.13 operator<()

```
template<typename LabelT = std::string, typename NodeIDT = unsigned int, typename EdgeT =
unsigned long long int>
bool LinkPred::UNetwork< LabelT, NodeIDT, EdgeT >::NonEdgeIt::operator< (
            const NonEdgeIt & that ) const  [inline]
```

Parameters

that	The other iterator.

Returns

True if this is less than that.

9.78.4.14 operator<=()

```
template<typename LabelT = std::string, typename NodeIDT = unsigned int, typename EdgeT =
unsigned long long int>
bool LinkPred::UNetwork< LabelT, NodeIDT, EdgeT >::NonEdgeIt::operator<= (
            const NonEdgeIt & that ) const  [inline]
```

Parameters

that	The other iterator.

Returns

True if this is greater or equal to that.

9.78.4.15 operator=() [1/2]

```
template<typename LabelT = std::string, typename NodeIDT = unsigned int, typename EdgeT =
unsigned long long int>
NonEdgeIt& LinkPred::UNetwork< LabelT, NodeIDT, EdgeT >::NonEdgeIt::operator= (
            NonEdgeIt && that )  [default]
```

Move assignment operator.

Parameters

that	The object to move.

9.78.4.16 operator=() [2/2]

```
template<typename LabelT = std::string, typename NodeIDT = unsigned int, typename EdgeT =
unsigned long long int>
NonEdgeIt& LinkPred::UNetwork< LabelT, NodeIDT, EdgeT >::NonEdgeIt::operator= (
            NonEdgeIt const & that )  [default]
```

Copy assignment operator.

Parameters

that	The object to copy.

9.78.4.17 operator==()

```
template<typename LabelT = std::string, typename NodeIDT = unsigned int, typename EdgeT =
unsigned long long int>
bool LinkPred::UNetwork< LabelT, NodeIDT, EdgeT >::NonEdgeIt::operator== (
            const NonEdgeIt & that ) const  [inline]
```

Parameters

that	The other iterator.

Returns

True if this equals that.

9.78.4.18 operator>()

```
template<typename LabelT = std::string, typename NodeIDT = unsigned int, typename EdgeT =
unsigned long long int>
bool LinkPred::UNetwork< LabelT, NodeIDT, EdgeT >::NonEdgeIt::operator> (
            const NonEdgeIt & that ) const  [inline]
```

Parameters

that	The other iterator.

Returns

True if this is greater that.

9.78.4.19 operator>=()

```
template<typename LabelT = std::string, typename NodeIDT = unsigned int, typename EdgeT =
unsigned long long int>
bool LinkPred::UNetwork< LabelT, NodeIDT, EdgeT >::NonEdgeIt::operator>= (
            const NonEdgeIt & that ) const  [inline]
```

Parameters

that	The other iterator.

Returns

True if this is less or equal to that.

9.78.5 Friends And Related Function Documentation

9.78.5.1 UNetwork

```
template<typename LabelT = std::string, typename NodeIDT = unsigned int, typename EdgeT =
unsigned long long int>
friend class UNetwork  [friend]
```

UNetwork is a friend.

The documentation for this class was generated from the following file:

- include/linkpred/core/unetwork/unetwork.hpp

9.79 LinkPred::DNetwork< LabelT, NodeIDT, EdgeT >::NonEdgeIt Class Reference

Nonedges iterator.

`#include <dnetwork.hpp>`

Inheritance diagram for LinkPred::DNetwork< LabelT, NodeIDT, EdgeT >::NonEdgeIt:

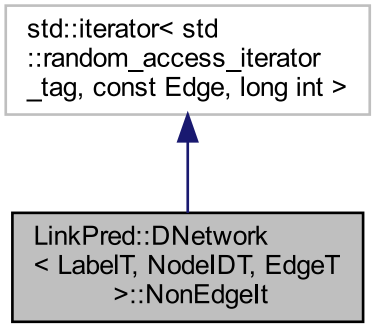

Collaboration diagram for LinkPred::DNetwork< LabelT, NodeIDT, EdgeT >::NonEdgeIt:

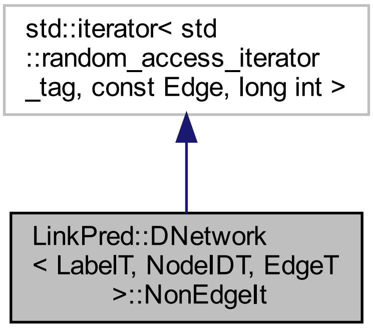

Public Types

- using pointer = typename std::iterator< std::random_access_iterator_tag, const Edge, long int >::pointer
- using reference = typename std::iterator< std::random_access_iterator_tag, const Edge, long int >::reference
- using difference_type = typename std::iterator< std::random_access_iterator_tag, const Edge, long int >::difference_type

Public Member Functions

- NonEdgeIt (NonEdgeIt const &that)=default
- NonEdgeIt & operator= (NonEdgeIt const &that)=default
- NonEdgeIt (NonEdgeIt &&that)=default
- NonEdgeIt & operator= (NonEdgeIt &&that)=default
- reference operator∗ ()
- pointer operator-> ()
- NonEdgeIt & operator++ ()
- NonEdgeIt & operator-- ()
- NonEdgeIt operator++ (int)
- NonEdgeIt operator-- (int)
- NonEdgeIt operator+ (const difference_type &n) const
- NonEdgeIt & operator+= (const difference_type &n)
- NonEdgeIt operator- (const difference_type &n) const
- NonEdgeIt & operator-= (const difference_type &n)
- difference_type operator- (const NonEdgeIt &that) const
- bool operator== (const NonEdgeIt &that) const
- bool operator!= (const NonEdgeIt &that) const
- bool operator< (const NonEdgeIt &that) const
- bool operator> (const NonEdgeIt &that) const
- bool operator<= (const NonEdgeIt &that) const
- bool operator>= (const NonEdgeIt &that) const

Friends

- class DNetwork

9.79.1 Detailed Description

**template<typename LabelT = std::string, typename NodeIDT = unsigned int, typename EdgeT = unsigned long long int>
class LinkPred::DNetwork< LabelT, NodeIDT, EdgeT >::NonEdgeIt**

Nonedges iterator.

9.79.2 Member Typedef Documentation

9.79.2.1 difference_type

```
template<typename LabelT = std::string, typename NodeIDT = unsigned int, typename EdgeT =
unsigned long long int>
using LinkPred::DNetwork< LabelT, NodeIDT, EdgeT >::NonEdgeIt::difference_type = typename
std::iterator<std::random_access_iterator_tag, const Edge, long int>::difference_type
```

The difference type associated with the iterator.

9.79.2.2 pointer

```
template<typename LabelT = std::string, typename NodeIDT = unsigned int, typename EdgeT =
unsigned long long int>
using LinkPred::DNetwork< LabelT, NodeIDT, EdgeT >::NonEdgeIt::pointer = typename std::iterator<std↩
::random_access_iterator_tag, const Edge, long int>::pointer
```

The pointer type associated with the iterator.

9.79.2.3 reference

```
template<typename LabelT = std::string, typename NodeIDT = unsigned int, typename EdgeT =
unsigned long long int>
using LinkPred::DNetwork< LabelT, NodeIDT, EdgeT >::NonEdgeIt::reference = typename std↩
::iterator<std::random_access_iterator_tag, const Edge, long int>::reference
```

The reference type associated with the iterator.

9.79.3 Constructor & Destructor Documentation

9.79.3.1 NonEdgeIt() [1/2]

```
template<typename LabelT = std::string, typename NodeIDT = unsigned int, typename EdgeT =
unsigned long long int>
LinkPred::DNetwork< LabelT, NodeIDT, EdgeT >::NonEdgeIt::NonEdgeIt (
            NonEdgeIt const & that )  [default]
```

Copy constructor.

Parameters

that	The object to copy.

9.79.3.2 NonEdgeIt() [2/2]

```
template<typename LabelT = std::string, typename NodeIDT = unsigned int, typename EdgeT =
unsigned long long int>
LinkPred::DNetwork< LabelT, NodeIDT, EdgeT >::NonEdgeIt::NonEdgeIt (
            NonEdgeIt && that )  [default]
```

Move constructor.

Parameters

that	The object to move.

9.79.4 Member Function Documentation

9.79.4.1 operator"!=()

```
template<typename LabelT = std::string, typename NodeIDT = unsigned int, typename EdgeT =
unsigned long long int>
bool LinkPred::DNetwork< LabelT, NodeIDT, EdgeT >::NonEdgeIt::operator!= (
            const NonEdgeIt & that ) const  [inline]
```

Parameters

that	The other iterator.

Returns

True if this is not equal to that.

9.79.4.2 operator*()

```
template<typename LabelT = std::string, typename NodeIDT = unsigned int, typename EdgeT =
unsigned long long int>
reference LinkPred::DNetwork< LabelT, NodeIDT, EdgeT >::NonEdgeIt::operator* ( )  [inline]
```

Dereference operator.

Returns

A reference to the object to which the iterator points.

9.79.4.3 operator+()

```
template<typename LabelT = std::string, typename NodeIDT = unsigned int, typename EdgeT =
unsigned long long int>
NonEdgeIt LinkPred::DNetwork< LabelT, NodeIDT, EdgeT >::NonEdgeIt::operator+ (
            const difference_type & n ) const  [inline]
```

Arithmetic + operator.

Parameters

n	Increment value.

Returns

The new iterator.

9.79.4.4 operator++() [1/2]

```
template<typename LabelT = std::string, typename NodeIDT = unsigned int, typename EdgeT =
unsigned long long int>
NonEdgeIt& LinkPred::DNetwork< LabelT, NodeIDT, EdgeT >::NonEdgeIt::operator++ ( )  [inline]
```

Pre-increment operator.

Returns

A reference to the new iterator.

9.79.4.5 operator++() [2/2]

```
template<typename LabelT = std::string, typename NodeIDT = unsigned int, typename EdgeT =
unsigned long long int>
NonEdgeIt LinkPred::DNetwork< LabelT, NodeIDT, EdgeT >::NonEdgeIt::operator++ (
            int  )  [inline]
```

Post-increment operator.

Returns

A reference to the new iterator.

9.79.4.6 operator+=()

```
template<typename LabelT = std::string, typename NodeIDT = unsigned int, typename EdgeT =
unsigned long long int>
NonEdgeIt& LinkPred::DNetwork< LabelT, NodeIDT, EdgeT >::NonEdgeIt::operator+= (
            const difference_type & n )  [inline]
```

Arithmetic += operator.

Parameters

n	Increment value.

Returns

A reference to the new iterator.

9.79.4.7 operator-() [1/2]

```
template<typename LabelT = std::string, typename NodeIDT = unsigned int, typename EdgeT =
unsigned long long int>
NonEdgeIt LinkPred::DNetwork< LabelT, NodeIDT, EdgeT >::NonEdgeIt::operator- (
            const difference_type & n ) const  [inline]
```

Arithmetic - operator.

Parameters

n	Decrement value.

Returns

The new iterator.

9.79.4.8 operator-() [2/2]

```
template<typename LabelT = std::string, typename NodeIDT = unsigned int, typename EdgeT =
unsigned long long int>
difference_type LinkPred::DNetwork< LabelT, NodeIDT, EdgeT >::NonEdgeIt::operator- (
            const NonEdgeIt & that ) const  [inline]
```

Difference between the present iterator and the one passed as parameter.

Parameters

that	The other iterator.

Returns

The difference between the current and that iterator.

9.79.4.9 operator--() [1/2]

```
template<typename LabelT = std::string, typename NodeIDT = unsigned int, typename EdgeT =
unsigned long long int>
NonEdgeIt& LinkPred::DNetwork< LabelT, NodeIDT, EdgeT >::NonEdgeIt::operator-- ( ) [inline]
```

Pre-decrement operator.

Returns

A reference to the new iterator.

9.79.4.10 operator--() [2/2]

```
template<typename LabelT = std::string, typename NodeIDT = unsigned int, typename EdgeT =
unsigned long long int>
NonEdgeIt LinkPred::DNetwork< LabelT, NodeIDT, EdgeT >::NonEdgeIt::operator-- (
             int ) [inline]
```

Post-decrement operator.

Returns

A reference to the new iterator.

9.79.4.11 operator-=()

```
template<typename LabelT = std::string, typename NodeIDT = unsigned int, typename EdgeT =
unsigned long long int>
NonEdgeIt& LinkPred::DNetwork< LabelT, NodeIDT, EdgeT >::NonEdgeIt::operator-= (
             const difference_type & n ) [inline]
```

Arithmetic -= operator.

Parameters

n	Decrement value.

Returns

A reference to the new iterator.

9.79.4.12 operator->()

```
template<typename LabelT = std::string, typename NodeIDT = unsigned int, typename EdgeT =
unsigned long long int>
pointer LinkPred::DNetwork< LabelT, NodeIDT, EdgeT >::NonEdgeIt::operator-> ( )  [inline]
```

Arrow operator.

Returns

A pointer to the object to which the iterator points.

9.79.4.13 operator<()

```
template<typename LabelT = std::string, typename NodeIDT = unsigned int, typename EdgeT =
unsigned long long int>
bool LinkPred::DNetwork< LabelT, NodeIDT, EdgeT >::NonEdgeIt::operator< (
            const NonEdgeIt & that ) const  [inline]
```

Parameters

that	The other iterator.

Returns

True if this is less than that.

9.79.4.14 operator<=()

```
template<typename LabelT = std::string, typename NodeIDT = unsigned int, typename EdgeT =
unsigned long long int>
bool LinkPred::DNetwork< LabelT, NodeIDT, EdgeT >::NonEdgeIt::operator<= (
            const NonEdgeIt & that ) const  [inline]
```

Parameters

that	The other iterator.

Returns

True if this is greater or equal to that.

9.79.4.15 operator=() [1/2]

```
template<typename LabelT = std::string, typename NodeIDT = unsigned int, typename EdgeT =
unsigned long long int>
NonEdgeIt& LinkPred::DNetwork< LabelT, NodeIDT, EdgeT >::NonEdgeIt::operator= (
            NonEdgeIt && that )  [default]
```

Move assignment operator.

Parameters

that	The object to move.

9.79.4.16 operator=() [2/2]

```
template<typename LabelT = std::string, typename NodeIDT = unsigned int, typename EdgeT =
unsigned long long int>
NonEdgeIt& LinkPred::DNetwork< LabelT, NodeIDT, EdgeT >::NonEdgeIt::operator= (
            NonEdgeIt const & that )  [default]
```

Copy assignment operator.

Parameters

that	The object to copy.

9.79.4.17 operator==()

```
template<typename LabelT = std::string, typename NodeIDT = unsigned int, typename EdgeT =
unsigned long long int>
bool LinkPred::DNetwork< LabelT, NodeIDT, EdgeT >::NonEdgeIt::operator== (
            const NonEdgeIt & that ) const  [inline]
```

Parameters

that	The other iterator.

Returns

True if this equals that.

9.79.4.18 operator>()

```
template<typename LabelT = std::string, typename NodeIDT = unsigned int, typename EdgeT =
unsigned long long int>
bool LinkPred::DNetwork< LabelT, NodeIDT, EdgeT >::NonEdgeIt::operator> (
            const NonEdgeIt & that ) const  [inline]
```

Parameters

that	The other iterator.

Returns

True if this is greater that.

9.79.4.19 operator>=()

```
template<typename LabelT = std::string, typename NodeIDT = unsigned int, typename EdgeT =
unsigned long long int>
bool LinkPred::DNetwork< LabelT, NodeIDT, EdgeT >::NonEdgeIt::operator>= (
            const NonEdgeIt & that ) const  [inline]
```

Parameters

that	The other iterator.

Returns

True if this is less or equal to that.

9.79.5 Friends And Related Function Documentation

9.79.5.1 DNetwork

```
template<typename LabelT = std::string, typename NodeIDT = unsigned int, typename EdgeT =
unsigned long long int>
friend class DNetwork  [friend]
```

DNetwork is a friend.

The documentation for this class was generated from the following file:

- include/linkpred/core/dnetwork/dnetwork.hpp

9.80 LinkPred::Utils::PairCompRight$<$ FirstT, SecondT, CompareT $>$ Struct Template Reference

Class for comparing pairs based on second elements only.

`#include <miscutils.hpp>`

Public Member Functions

- bool operator() (std::pair$<$ FirstT, SecondT $>$ const &l, std::pair$<$ FirstT, SecondT $>$ const &r) const

9.80.1 Detailed Description

template$<$typename FirstT, typename SecondT, typename CompareT$>$
struct LinkPred::Utils::PairCompRight$<$ FirstT, SecondT, CompareT $>$

Class for comparing pairs based on second elements only.

Template Parameters

FirstT	The first pair type.
SecondT	The second pair type.
CompareT	The comparator type.

9.80.2 Member Function Documentation

9.80.2.1 operator()()

```
template<typename FirstT , typename SecondT , typename CompareT >
bool LinkPred::Utils::PairCompRight< FirstT, SecondT, CompareT >::operator() (
            std::pair< FirstT, SecondT > const & l,
            std::pair< FirstT, SecondT > const & r ) const  [inline]
```

Compare pair based on second elements.

Parameters

l	The first pair.
r	The second pair.

Returns

The result of the comparison.

The documentation for this struct was generated from the following file:

- include/linkpred/utils/miscutils.hpp

9.81 LinkPred::Pearson Class Reference

Pearson similarity (Pearson correlation coefficient).

`#include <pearson.hpp>`

Inheritance diagram for LinkPred::Pearson:

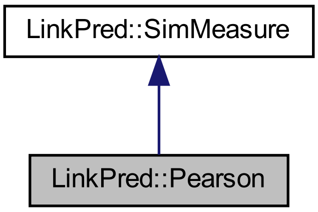

Collaboration diagram for LinkPred::Pearson:

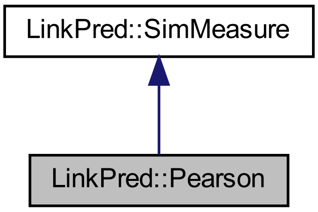

Public Member Functions

- Pearson ()
- Pearson (Pearson const &that)=default
- Pearson & operator= (Pearson const &that)=default
- Pearson (Pearson &&that)=default
- Pearson & operator= (Pearson &&that)=default
- virtual double sim (Vec const &v1, Vec const &v2)
- virtual ∼Pearson ()=default

9.81.1 Detailed Description

Pearson similarity (Pearson correlation coefficient).

9.81.2 Constructor & Destructor Documentation

9.81.2.1 Pearson() [1/3]

```
LinkPred::Pearson::Pearson ( ) [inline]
```

Constructor.

9.81.2.2 Pearson() [2/3]

```
LinkPred::Pearson::Pearson (
            Pearson const & that ) [default]
```

Copy constructor.

Parameters

that	The object to copy.

9.81.2.3 Pearson() [3/3]

```
LinkPred::Pearson::Pearson (
            Pearson && that ) [default]
```

Move constructor.

Parameters

that	The object to move.

9.81.2.4 ∼Pearson()

```
virtual LinkPred::Pearson::~Pearson ( ) [virtual], [default]
```

Destructor.

9.81.3 Member Function Documentation

9.81.3.1 operator=() [1/2]

```
Pearson& LinkPred::Pearson::operator= (
            Pearson && that )  [default]
```

Move assignment operator.

Parameters

that	The object to move.

9.81.3.2 operator=() [2/2]

```
Pearson& LinkPred::Pearson::operator= (
            Pearson const & that )  [default]
```

Copy assignment operator.

Parameters

that	The object to copy.

9.81.3.3 sim()

```
virtual double LinkPred::Pearson::sim (
            Vec const & v1,
            Vec const & v2 )  [virtual]
```

Compute the similarity between two vectors.

Parameters

v1	First vector.
v2	Second vector. Must be of the same dimension as v1.

Returns

The similarity between v1 and v2.

Implements LinkPred::SimMeasure.

The documentation for this class was generated from the following file:

- include/linkpred/ml/simmeasures/pearson.hpp

9.82 LinkPred::PEFactory< Network, LPredictorT, TestDataT, PerfMeasureT > Class Template Reference

Factory class to create link predictors and performance measures.

```
#include <perfevaluator.hpp>
```

Public Member Functions

- virtual std::vector< std::shared_ptr< LPredictorT > > getPredictors (std::shared_ptr< Network const > obsNet)=0
- virtual std::vector< std::shared_ptr< PerfMeasureT > > getPerfMeasures (TestDataT const &testData)=0
- virtual ~PEFactory ()=default

9.82.1 Detailed Description

template<typename Network = UNetwork<>, typename LPredictorT = ULPredictor<>, typename TestDataT = TestData<>, typename PerfMeasureT = PerfMeasure< PredResults<TestDataT, LPredictorT>>>
class LinkPred::PEFactory< Network, LPredictorT, TestDataT, PerfMeasureT >

Factory class to create link predictors and performance measures.

Template Parameters

Network	The network data type.
LPredictorT	The link predictor type.
TestDataT	The test data type.
PerfMeasureT	The performance measure type.

9.82.2 Constructor & Destructor Documentation

9.82.2.1 ~PEFactory()

```
template<typename Network = UNetwork<>, typename LPredictorT = ULPredictor<>, typename Test↩
DataT = TestData<>, typename PerfMeasureT = PerfMeasure< PredResults<TestDataT, LPredictor↩
T>>>
virtual LinkPred::PEFactory< Network, LPredictorT, TestDataT, PerfMeasureT >::~PEFactory ( )
[virtual], [default]
```

Destructor.

9.82.3 Member Function Documentation

9.82.3.1 getPerfMeasures()

```
template<typename Network = UNetwork<>, typename LPredictorT = ULPredictor<>, typename Test←
DataT = TestData<>, typename PerfMeasureT = PerfMeasure< PredResults<TestDataT, LPredictor←
T>>>
virtual std::vector<std::shared_ptr<PerfMeasureT> > LinkPred::PEFactory< Network, LPredictorT,
TestDataT, PerfMeasureT >::getPerfMeasures (
            TestDataT const & testData )  [pure virtual]
```

Returns

A vector of performance measures.

9.82.3.2 getPredictors()

```
template<typename Network = UNetwork<>, typename LPredictorT = ULPredictor<>, typename Test←
DataT = TestData<>, typename PerfMeasureT = PerfMeasure< PredResults<TestDataT, LPredictor←
T>>>
virtual std::vector<std::shared_ptr<LPredictorT> > LinkPred::PEFactory< Network, LPredictorT,
TestDataT, PerfMeasureT >::getPredictors (
            std::shared_ptr< Network const > obsNet )  [pure virtual]
```

Returns

A vector of predictors.

The documentation for this class was generated from the following file:

- include/linkpred/perf/perfevaluator.hpp

9.83 LinkPred::PerfCurve< PredResultsT > Class Template Reference

Abstract performance curve.

```
#include <perfmeasure.hpp>
```

Inheritance diagram for LinkPred::PerfCurve< PredResultsT >:

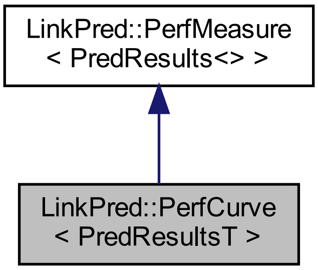

Collaboration diagram for LinkPred::PerfCurve< PredResultsT >:

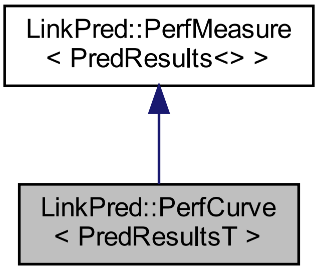

Public Types

- using ScoresItT = typename PerfMeasure< PredResultsT >::ScoresItT

Public Member Functions

- PerfCurve ()=default
- PerfCurve (std::string name)
- PerfCurve (PerfCurve const &that)=default
- PerfCurve & operator= (PerfCurve const &that)=default
- PerfCurve (PerfCurve &&that)=default
- PerfCurve & operator= (PerfCurve &&that)=default
- virtual void eval (std::shared_ptr< PredResultsT > &predResults, PerfResults &results)=0
- virtual void eval (ScoresItT posScoresBegin, ScoresItT posScoresEnd, ScoresItT negScoresBegin, ScoresItT negScoresEnd, SortOrder &posSortOrder, SortOrder &negSortOrder, PerfResults &results)=0
- virtual std::vector< std::pair< double, double > > getCurve (std::shared_ptr< PredResultsT > &pred↔ Results)=0

- virtual std::vector< std::pair< double, double > > getCurve (ScoresItT posScoresBegin, ScoresItT pos↩ ScoresEnd, ScoresItT negScoresBegin, ScoresItT negScoresEnd, SortOrder &posSortOrder, SortOrder &negSortOrder)=0
- virtual ∼PerfCurve ()=default

9.83.1 Detailed Description

template<typename PredResultsT = PredResults<>>
class LinkPred::PerfCurve< PredResultsT >

Abstract performance curve.

Template Parameters

PredResultsT	The prediction results type.

9.83.2 Member Typedef Documentation

9.83.2.1 ScoresItT

```
template<typename PredResultsT = PredResults<>>
using LinkPred::PerfCurve< PredResultsT >::ScoresItT = typename PerfMeasure<PredResultsT>↩
::ScoresItT
```

Scores iterator type.

9.83.3 Constructor & Destructor Documentation

9.83.3.1 PerfCurve() [1/4]

```
template<typename PredResultsT = PredResults<>>
LinkPred::PerfCurve< PredResultsT >::PerfCurve ( )  [default]
```

< The name of the performance measure. Constructor.

9.83.3.2 PerfCurve() [2/4]

```
template<typename PredResultsT = PredResults<>>
LinkPred::PerfCurve< PredResultsT >::PerfCurve (
            std::string name )  [inline]
```

Constructor.

Parameters

name	The name of the performance measure.

9.83.3.3 PerfCurve() [3/4]

```
template<typename PredResultsT = PredResults<>>
LinkPred::PerfCurve< PredResultsT >::PerfCurve (
            PerfCurve< PredResultsT > const & that )  [default]
```

Copy constructor.

Parameters

that	The object to copy.

9.83.3.4 PerfCurve() [4/4]

```
template<typename PredResultsT = PredResults<>>
LinkPred::PerfCurve< PredResultsT >::PerfCurve (
            PerfCurve< PredResultsT > && that )  [default]
```

Move constructor.

Parameters

that	The object to move.

9.83.3.5 ∼PerfCurve()

```
template<typename PredResultsT = PredResults<>>
virtual LinkPred::PerfCurve< PredResultsT >::~PerfCurve ( )  [virtual], [default]
```

Destructor.

9.83.4 Member Function Documentation

9.83.4.1 eval() [1/2]

```
template<typename PredResultsT = PredResults<>>
virtual void LinkPred::PerfCurve< PredResultsT >::eval (
            ScoresItT posScoresBegin,
            ScoresItT posScoresEnd,
            ScoresItT negScoresBegin,
            ScoresItT negScoresEnd,
            SortOrder & posSortOrder,
            SortOrder & negSortOrder,
            PerfResults & results ) [pure virtual]
```

Computes the performance measure.

Parameters

posScoresBegin	Iterator to the first positive score.
posScoresEnd	Iterator to one-past-the-last positive score.
negScoresBegin	Iterator to the first negative score.
negScoresEnd	Iterator to one-past-the-last negative score.
posSortOrder	The sorting order of positive scores. This may be modified by the method.
negSortOrder	The sorting order of negative scores. This may be modified by the method.
results	To write results.

9.83.4.2 eval() [2/2]

```
template<typename PredResultsT = PredResults<>>
virtual void LinkPred::PerfCurve< PredResultsT >::eval (
            std::shared_ptr< PredResultsT > & predResults,
            PerfResults & results ) [pure virtual]
```

Computes the performance measure (typically the area under the curve or AUC).

Parameters

predResults	The prediction results.
results	To write results.

9.83.4.3 getCurve() [1/2]

```
template<typename PredResultsT = PredResults<>>
virtual std::vector<std::pair<double, double> > LinkPred::PerfCurve< PredResultsT >::get↩
Curve (
            ScoresItT posScoresBegin,
            ScoresItT posScoresEnd,
            ScoresItT negScoresBegin,
```

```
              ScoresItT negScoresEnd,
              SortOrder & posSortOrder,
              SortOrder & negSortOrder )  [pure virtual]
```

Computes the performance curve.

Parameters

posScoresBegin	Iterator to the first positive score.
posScoresEnd	Iterator to one-past-the-last positive score.
negScoresBegin	Iterator to the first negative score.
negScoresEnd	Iterator to one-past-the-last negative score.
posSortOrder	The sorting order of positive scores. This may be modified by the method.
negSortOrder	The sorting order of negative scores. This may be modified by the method.

Returns

A curve in the form of an std::vector of pairs representing the x and y coordinates.

9.83.4.4 getCurve() [2/2]

```
template<typename PredResultsT = PredResults<>>
virtual std::vector<std::pair<double, double> > LinkPred::PerfCurve< PredResultsT >::get↩
Curve (
              std::shared_ptr< PredResultsT > & predResults )  [pure virtual]
```

Computes the performance curve.

Parameters

predResults	The prediction results.

Returns

A curve in the form of an std::vector of pairs representing the x and y coordinates.

9.83.4.5 operator=() [1/2]

```
template<typename PredResultsT = PredResults<>>
PerfCurve& LinkPred::PerfCurve< PredResultsT >::operator= (
              PerfCurve< PredResultsT > && that )  [default]
```

Move assignment operator.

Parameters

that	The object to move.

9.83.4.6 operator=() [2/2]

```
template<typename PredResultsT = PredResults<>>
PerfCurve& LinkPred::PerfCurve< PredResultsT >::operator= (
            PerfCurve< PredResultsT > const & that )  [default]
```

Copy assignment operator.

Parameters

that	The object to copy.

The documentation for this class was generated from the following file:

- include/linkpred/perf/perfmeasure.hpp

9.84 LinkPred::PerfeEvalExpDescp< Network > Struct Template Reference

Structure storing experiment description.

`#include <perfevaluator.hpp>`

Public Attributes

- bool timingEnabled = false
- std::ostream ∗ out = &std::cout
- std::size_t nbTestRuns = 1
- std::shared_ptr< Network > refNet
- bool keepConnected = false
- double fnRatio = 1
- double tnRatio = 1
- double ratioStart = 0.1
- double ratioEnd = 0.1
- double ratioStep = 0.1
- long int seed = 0

9.84.1 Detailed Description

template<typename Network = UNetwork<>>
struct LinkPred::PerfeEvalExpDescp< Network >

Structure storing experiment description.

9.84.2 Member Data Documentation

9.84.2.1 fnRatio

```
template<typename Network = UNetwork<>>
double LinkPred::PerfeEvalExpDescp< Network >::fnRatio = 1
```

Ratio of false negatives used in the test set.

9.84.2.2 keepConnected

```
template<typename Network = UNetwork<>>
bool LinkPred::PerfeEvalExpDescp< Network >::keepConnected = false
```

Whether to keep the network connected.

9.84.2.3 nbTestRuns

```
template<typename Network = UNetwork<>>
std::size_t LinkPred::PerfeEvalExpDescp< Network >::nbTestRuns = 1
```

Number of test runs.

9.84.2.4 out

```
template<typename Network = UNetwork<>>
std::ostream* LinkPred::PerfeEvalExpDescp< Network >::out = &std::cout
```

Output file.

9.84.2.5 ratioEnd

```
template<typename Network = UNetwork<>>
double LinkPred::PerfeEvalExpDescp< Network >::ratioEnd = 0.1
```

End value of the ratio of removed edges. This is adjusted if keepConnected is set to true.

9.84.2.6 ratioStart

```
template<typename Network = UNetwork<>>
double LinkPred::PerfeEvalExpDescp< Network >::ratioStart = 0.1
```

Start value of the ratio of removed edges.

9.84.2.7 ratioStep

```
template<typename Network = UNetwork<>>
double LinkPred::PerfeEvalExpDescp< Network >::ratioStep = 0.1
```

Step size of the ratio of removed edges.

9.84.2.8 refNet

```
template<typename Network = UNetwork<>>
std::shared_ptr<Network> LinkPred::PerfeEvalExpDescp< Network >::refNet
```

Reference network.

9.84.2.9 seed

```
template<typename Network = UNetwork<>>
long int LinkPred::PerfeEvalExpDescp< Network >::seed = 0
```

Seed for the random number generator.

9.84.2.10 timingEnabled

```
template<typename Network = UNetwork<>>
bool LinkPred::PerfeEvalExpDescp< Network >::timingEnabled = false
```

Enable/disable timing.

9.84.2.11 tnRatio

```
template<typename Network = UNetwork<>>
double LinkPred::PerfeEvalExpDescp< Network >::tnRatio = 1
```

Ratio of true negatives used in the test set.

The documentation for this struct was generated from the following file:

- include/linkpred/perf/perfevaluator.hpp

9.85 LinkPred::PerfEvalExp< Network, TestDataT, LPredictorT, PredResultsT, PerfMeasureT > Class Template Reference

Performance evaluation experiment.

```
#include <perfevaluator.hpp>
```

Public Member Functions

- PerfEvalExp (PerfeEvalExpDescp< Network > const &ped, std::shared_ptr< PEFactory< Network, LPredictorT, TestDataT, PerfMeasureT >> const &factory)
- PerfEvalExp (PerfEvalExp const &that)=default
- PerfEvalExp & operator= (PerfEvalExp const &that)=default
- PerfEvalExp (PerfEvalExp &&that)=default
- PerfEvalExp & operator= (PerfEvalExp &&that)=default
- void run ()
- void runNoTiming ()
- void runTiming ()
- auto resultsBegin () const
- auto resultsEnd () const
- const std::shared_ptr< PEFactory< Network, LPredictorT, TestDataT, PerfMeasureT > > & getFactory () const
- int getOutPrec () const
- void setOutPrec (int outPrec)
- const PerfeEvalExpDescp< Network > & getPed () const
- virtual ∼PerfEvalExp ()=default

9.85.1 Detailed Description

template<typename Network = UNetwork<>, typename TestDataT = TestData<>, typename LPredictorT = ULPredictor<>, typename PredResultsT = PredResults<TestDataT, LPredictorT>, typename PerfMeasureT = PerfMeasure<PredResultsT>> class LinkPred::PerfEvalExp< Network, TestDataT, LPredictorT, PredResultsT, PerfMeasureT >

Performance evaluation experiment.

Template Parameters

Network	The network data type.
TestDataT	The test data type.
PredResultsT	The prediction results type.
PerfMeasureT	The performance measure type.

9.85.2 Constructor & Destructor Documentation

9.85.2.1 PerfEvalExp() [1/3]

```
template<typename Network = UNetwork<>, typename TestDataT = TestData<>, typename LPredictorT
= ULPredictor<>, typename PredResultsT = PredResults<TestDataT, LPredictorT>, typename PerfMeasureT = PerfMeasure<PredResultsT>>
LinkPred::PerfEvalExp< Network, TestDataT, LPredictorT, PredResultsT, PerfMeasureT >::PerfEvalExp
(
            PerfeEvalExpDescp< Network > const & ped,
            std::shared_ptr< PEFactory< Network, LPredictorT, TestDataT, PerfMeasureT >>
const & factory )  [inline]
```

Constructor.

Parameters

ped	The experiment description.
factory	Factory bject to create link predictor and performance measures.

9.85.2.2 PerfEvalExp() [2/3]

```
template<typename Network = UNetwork<>, typename TestDataT = TestData<>, typename LPredictorT
= ULPredictor<>, typename PredResultsT = PredResults<TestDataT, LPredictorT>, typename Perf↩
MeasureT = PerfMeasure<PredResultsT>>
LinkPred::PerfEvalExp< Network, TestDataT, LPredictorT, PredResultsT, PerfMeasureT >::PerfEvalExp
(
            PerfEvalExp< Network, TestDataT, LPredictorT, PredResultsT, PerfMeasureT > const
& that )  [default]
```

Copy constructor.

Parameters

that	The object to copy.

9.85.2.3 PerfEvalExp() [3/3]

```
template<typename Network = UNetwork<>, typename TestDataT = TestData<>, typename LPredictorT
= ULPredictor<>, typename PredResultsT = PredResults<TestDataT, LPredictorT>, typename Perf↩
MeasureT = PerfMeasure<PredResultsT>>
LinkPred::PerfEvalExp< Network, TestDataT, LPredictorT, PredResultsT, PerfMeasureT >::PerfEvalExp
(
            PerfEvalExp< Network, TestDataT, LPredictorT, PredResultsT, PerfMeasureT > &&
that )  [default]
```

Move constructor.

Parameters

that	The object to move.

9.85.2.4 ∼PerfEvalExp()

```
template<typename Network = UNetwork<>, typename TestDataT = TestData<>, typename LPredictorT
= ULPredictor<>, typename PredResultsT = PredResults<TestDataT, LPredictorT>, typename Perf↩
MeasureT = PerfMeasure<PredResultsT>>
```

```
virtual LinkPred::PerfEvalExp< Network, TestDataT, LPredictorT, PredResultsT, PerfMeasureT >↩
::~PerfEvalExp ( ) [virtual], [default]
```

Destructor.

9.85.3 Member Function Documentation

9.85.3.1 getFactory()

```
template<typename Network = UNetwork<>, typename TestDataT = TestData<>, typename LPredictorT
= ULPredictor<>, typename PredResultsT = PredResults<TestDataT, LPredictorT>, typename Perf↩
MeasureT = PerfMeasure<PredResultsT>>
const std::shared_ptr< PEFactory<Network, LPredictorT, TestDataT, PerfMeasureT> >& LinkPred::PerfEvalExp<
Network, TestDataT, LPredictorT, PredResultsT, PerfMeasureT >::getFactory ( ) const [inline]
```

Returns

The fatory.

9.85.3.2 getOutPrec()

```
template<typename Network = UNetwork<>, typename TestDataT = TestData<>, typename LPredictorT
= ULPredictor<>, typename PredResultsT = PredResults<TestDataT, LPredictorT>, typename Perf↩
MeasureT = PerfMeasure<PredResultsT>>
int LinkPred::PerfEvalExp< Network, TestDataT, LPredictorT, PredResultsT, PerfMeasureT >↩
::getOutPrec ( ) const [inline]
```

Returns

Output precision (for double).

9.85.3.3 getPed()

```
template<typename Network = UNetwork<>, typename TestDataT = TestData<>, typename LPredictorT
= ULPredictor<>, typename PredResultsT = PredResults<TestDataT, LPredictorT>, typename Perf↩
MeasureT = PerfMeasure<PredResultsT>>
const PerfeEvalExpDescp<Network>& LinkPred::PerfEvalExp< Network, TestDataT, LPredictorT,
PredResultsT, PerfMeasureT >::getPed ( ) const [inline]
```

Returns

The experience descriptor.

9.85.3.4 operator=() [1/2]

```
template<typename Network = UNetwork<>, typename TestDataT = TestData<>, typename LPredictorT
= ULPredictor<>, typename PredResultsT = PredResults<TestDataT, LPredictorT>, typename Perf↩
MeasureT = PerfMeasure<PredResultsT>>
PerfEvalExp& LinkPred::PerfEvalExp< Network, TestDataT, LPredictorT, PredResultsT, Perf↩
MeasureT >::operator= (
            PerfEvalExp< Network, TestDataT, LPredictorT, PredResultsT, PerfMeasureT > &&
that ) [default]
```

Move assignment operator.

Parameters

that	The object to move.

9.85.3.5 operator=() [2/2]

```
template<typename Network = UNetwork<>, typename TestDataT = TestData<>, typename LPredictorT
= ULPredictor<>, typename PredResultsT = PredResults<TestDataT, LPredictorT>, typename Perf↩
MeasureT = PerfMeasure<PredResultsT>>
PerfEvalExp& LinkPred::PerfEvalExp< Network, TestDataT, LPredictorT, PredResultsT, Perf↩
MeasureT >::operator= (
            PerfEvalExp< Network, TestDataT, LPredictorT, PredResultsT, PerfMeasureT > const
& that ) [default]
```

Copy assignment operator.

Parameters

that	The object to copy.

9.85.3.6 resultsBegin()

```
template<typename Network = UNetwork<>, typename TestDataT = TestData<>, typename LPredictorT
= ULPredictor<>, typename PredResultsT = PredResults<TestDataT, LPredictorT>, typename Perf↩
MeasureT = PerfMeasure<PredResultsT>>
auto LinkPred::PerfEvalExp< Network, TestDataT, LPredictorT, PredResultsT, PerfMeasureT >↩
::resultsBegin ( ) const [inline]
```

Returns

An iterator to the first performance result.

9.85.3.7 resultsEnd()

```
template<typename Network = UNetwork<>, typename TestDataT = TestData<>, typename LPredictorT
= ULPredictor<>, typename PredResultsT = PredResults<TestDataT, LPredictorT>, typename Perf↩
MeasureT = PerfMeasure<PredResultsT>>
auto LinkPred::PerfEvalExp< Network, TestDataT, LPredictorT, PredResultsT, PerfMeasureT >↩
::resultsEnd ( ) const  [inline]
```

Returns

An iterator to one-past-the-last first performance result.

9.85.3.8 run()

```
template<typename Network = UNetwork<>, typename TestDataT = TestData<>, typename LPredictorT
= ULPredictor<>, typename PredResultsT = PredResults<TestDataT, LPredictorT>, typename Perf↩
MeasureT = PerfMeasure<PredResultsT>>
void LinkPred::PerfEvalExp< Network, TestDataT, LPredictorT, PredResultsT, PerfMeasureT >::run
( )
```

Run the performance evaluation experiment.

9.85.3.9 runNoTiming()

```
template<typename Network = UNetwork<>, typename TestDataT = TestData<>, typename LPredictorT
= ULPredictor<>, typename PredResultsT = PredResults<TestDataT, LPredictorT>, typename Perf↩
MeasureT = PerfMeasure<PredResultsT>>
void LinkPred::PerfEvalExp< Network, TestDataT, LPredictorT, PredResultsT, PerfMeasureT >↩
::runNoTiming ( )
```

Runs the performance evaluation without timing.

9.85.3.10 runTiming()

```
template<typename Network = UNetwork<>, typename TestDataT = TestData<>, typename LPredictorT
= ULPredictor<>, typename PredResultsT = PredResults<TestDataT, LPredictorT>, typename Perf↩
MeasureT = PerfMeasure<PredResultsT>>
void LinkPred::PerfEvalExp< Network, TestDataT, LPredictorT, PredResultsT, PerfMeasureT >↩
::runTiming ( )
```

Runs the performance evaluation with timing.

9.85.3.11 setOutPrec()

```
template<typename Network = UNetwork<>, typename TestDataT = TestData<>, typename LPredictorT
= ULPredictor<>, typename PredResultsT = PredResults<TestDataT, LPredictorT>, typename Perf↩
MeasureT = PerfMeasure<PredResultsT>>
void LinkPred::PerfEvalExp< Network, TestDataT, LPredictorT, PredResultsT, PerfMeasureT >↩
::setOutPrec (
             int outPrec )  [inline]
```

Parameters

outPrec	Output precision (for double).

The documentation for this class was generated from the following file:

- include/linkpred/perf/perfevaluator.hpp

9.86 LinkPred::PerfEvaluator< TestDataT, LPredictorT, PredResultsT, PerfMeasureT > Class Template Reference

Performance evaluator.

```
#include <perfevaluator.hpp>
```

Public Member Functions

- PerfEvaluator (TestDataT testData)
- PerfEvaluator (PerfEvaluator const &that)=default
- PerfEvaluator & operator= (PerfEvaluator const &that)=default
- PerfEvaluator (PerfEvaluator &&that)=default
- PerfEvaluator & operator= (PerfEvaluator &&that)=default
- std::size_t addPredictor (std::shared_ptr< LPredictorT > predictor)
- std::size_t addPerfMeasure (std::shared_ptr< PerfMeasureT > measure)
- std::size_t getNbPredictors () const
- std::size_t getNbPerfMeasures () const
- auto getPredictor (std::size_t i) const
- void eval ()
- void evalNoTiming ()
- void evalTiming ()
- bool isTimingEnabled () const
- void setTimingEnabled (bool timingEnabled)
- auto resultsBegin () const
- auto resultsEnd () const
- virtual ∼PerfEvaluator ()=default

9.86.1 Detailed Description

template<typename TestDataT = TestData<>, typename LPredictorT = ULPredictor<>, typename PredResultsT = Pred↩ Results<TestDataT, LPredictorT>, typename PerfMeasureT = PerfMeasure<PredResultsT>> class LinkPred::PerfEvaluator< TestDataT, LPredictorT, PredResultsT, PerfMeasureT >

Performance evaluator.

Template Parameters

TestDataT	The test data type.
PredResultsT	The prediction results type.
PerfMeasureT	The performance measure type.

9.86.2 Constructor & Destructor Documentation

9.86.2.1 PerfEvaluator() [1/3]

```
template<typename TestDataT = TestData<>, typename LPredictorT = ULPredictor<>, typename
PredResultsT = PredResults<TestDataT, LPredictorT>, typename PerfMeasureT = PerfMeasure<↩
PredResultsT>>
LinkPred::PerfEvaluator< TestDataT, LPredictorT, PredResultsT, PerfMeasureT >::PerfEvaluator (
            TestDataT testData )  [inline]
```

Constructor.

Parameters

testData	The test data.

9.86.2.2 PerfEvaluator() [2/3]

```
template<typename TestDataT = TestData<>, typename LPredictorT = ULPredictor<>, typename
PredResultsT = PredResults<TestDataT, LPredictorT>, typename PerfMeasureT = PerfMeasure<↩
PredResultsT>>
LinkPred::PerfEvaluator< TestDataT, LPredictorT, PredResultsT, PerfMeasureT >::PerfEvaluator (
            PerfEvaluator< TestDataT, LPredictorT, PredResultsT, PerfMeasureT > const & that
)  [default]
```

Copy constructor.

Parameters

that	The object to copy.

9.86.2.3 PerfEvaluator() [3/3]

```
template<typename TestDataT = TestData<>, typename LPredictorT = ULPredictor<>, typename
PredResultsT = PredResults<TestDataT, LPredictorT>, typename PerfMeasureT = PerfMeasure<↩
PredResultsT>>
LinkPred::PerfEvaluator< TestDataT, LPredictorT, PredResultsT, PerfMeasureT >::PerfEvaluator (
            PerfEvaluator< TestDataT, LPredictorT, PredResultsT, PerfMeasureT > && that )
[default]
```

Move constructor.

Parameters

that	The object to move.

9.86.2.4 ∼PerfEvaluator()

```
template<typename TestDataT = TestData<>, typename LPredictorT = ULPredictor<>, typename
PredResultsT = PredResults<TestDataT, LPredictorT>, typename PerfMeasureT = PerfMeasure<←
PredResultsT>>
virtual LinkPred::PerfEvaluator< TestDataT, LPredictorT, PredResultsT, PerfMeasureT >::∼PerfEvaluator
( ) [virtual], [default]
```

Destructor.

9.86.3 Member Function Documentation

9.86.3.1 addPerfMeasure()

```
template<typename TestDataT = TestData<>, typename LPredictorT = ULPredictor<>, typename
PredResultsT = PredResults<TestDataT, LPredictorT>, typename PerfMeasureT = PerfMeasure<←
PredResultsT>>
std::size_t LinkPred::PerfEvaluator< TestDataT, LPredictorT, PredResultsT, PerfMeasureT >←
::addPerfMeasure (
            std::shared_ptr< PerfMeasureT > measure ) [inline]
```

Add a performance measure.

Parameters

measure	A performanmce measure.

Returns

An ID.

9.86.3.2 addPredictor()

```
template<typename TestDataT = TestData<>, typename LPredictorT = ULPredictor<>, typename
PredResultsT = PredResults<TestDataT, LPredictorT>, typename PerfMeasureT = PerfMeasure<←
PredResultsT>>
```

```
std::size_t LinkPred::PerfEvaluator< TestDataT, LPredictorT, PredResultsT, PerfMeasureT >↩
::addPredictor (
            std::shared_ptr< LPredictorT > predictor ) [inline]
```

Add a predictor.

Parameters

predictor	A link predictor.

Returns

An ID.

9.86.3.3 eval()

```
template<typename TestDataT = TestData<>, typename LPredictorT = ULPredictor<>, typename
PredResultsT = PredResults<TestDataT, LPredictorT>, typename PerfMeasureT = PerfMeasure<↩
PredResultsT>>
void LinkPred::PerfEvaluator< TestDataT, LPredictorT, PredResultsT, PerfMeasureT >::eval ( )
[inline]
```

Run the performance evaluation.

9.86.3.4 evalNoTiming()

```
template<typename TestDataT = TestData<>, typename LPredictorT = ULPredictor<>, typename
PredResultsT = PredResults<TestDataT, LPredictorT>, typename PerfMeasureT = PerfMeasure<↩
PredResultsT>>
void LinkPred::PerfEvaluator< TestDataT, LPredictorT, PredResultsT, PerfMeasureT >::evalNo↩
Timing ( )
```

Runs the performance evaluation without timing.

9.86.3.5 evalTiming()

```
template<typename TestDataT = TestData<>, typename LPredictorT = ULPredictor<>, typename
PredResultsT = PredResults<TestDataT, LPredictorT>, typename PerfMeasureT = PerfMeasure<↩
PredResultsT>>
void LinkPred::PerfEvaluator< TestDataT, LPredictorT, PredResultsT, PerfMeasureT >::evalTiming
( )
```

Runs the performance evaluation with timing.

9.86.3.6 getNbPerfMeasures()

```
template<typename TestDataT = TestData<>, typename LPredictorT = ULPredictor<>, typename
PredResultsT = PredResults<TestDataT, LPredictorT>, typename PerfMeasureT = PerfMeasure<↩
PredResultsT>>
std::size_t LinkPred::PerfEvaluator< TestDataT, LPredictorT, PredResultsT, PerfMeasureT >↩
::getNbPerfMeasures ( ) const [inline]
```

Returns

The number of performance measures.

9.86.3.7 getNbPredictors()

```
template<typename TestDataT = TestData<>, typename LPredictorT = ULPredictor<>, typename
PredResultsT = PredResults<TestDataT, LPredictorT>, typename PerfMeasureT = PerfMeasure<←
PredResultsT>>
std::size_t LinkPred::PerfEvaluator< TestDataT, LPredictorT, PredResultsT, PerfMeasureT >←
::getNbPredictors ( ) const [inline]
```

Returns

Ther number of predictors.

9.86.3.8 getPredictor()

```
template<typename TestDataT = TestData<>, typename LPredictorT = ULPredictor<>, typename
PredResultsT = PredResults<TestDataT, LPredictorT>, typename PerfMeasureT = PerfMeasure<←
PredResultsT>>
auto LinkPred::PerfEvaluator< TestDataT, LPredictorT, PredResultsT, PerfMeasureT >::get←
Predictor (
            std::size_t i ) const [inline]
```

Parameters

i	Index of the predictor.

Returns

The predictor of index i.

9.86.3.9 isTimingEnabled()

```
template<typename TestDataT = TestData<>, typename LPredictorT = ULPredictor<>, typename
PredResultsT = PredResults<TestDataT, LPredictorT>, typename PerfMeasureT = PerfMeasure<←
PredResultsT>>
bool LinkPred::PerfEvaluator< TestDataT, LPredictorT, PredResultsT, PerfMeasureT >::isTiming←
Enabled ( ) const [inline]
```

Returns

Whether timing is enabled.

9.86.3.10 operator=() [1/2]

```
template<typename TestDataT = TestData<>, typename LPredictorT = ULPredictor<>, typename
PredResultsT = PredResults<TestDataT, LPredictorT>, typename PerfMeasureT = PerfMeasure<↩
PredResultsT>>
PerfEvaluator& LinkPred::PerfEvaluator< TestDataT, LPredictorT, PredResultsT, PerfMeasureT >↩
::operator= (
            PerfEvaluator< TestDataT, LPredictorT, PredResultsT, PerfMeasureT > && that )
[default]
```

Move assignment operator.

Parameters

that	The object to move.

9.86.3.11 operator=() [2/2]

```
template<typename TestDataT = TestData<>, typename LPredictorT = ULPredictor<>, typename
PredResultsT = PredResults<TestDataT, LPredictorT>, typename PerfMeasureT = PerfMeasure<↩
PredResultsT>>
PerfEvaluator& LinkPred::PerfEvaluator< TestDataT, LPredictorT, PredResultsT, PerfMeasureT >↩
::operator= (
            PerfEvaluator< TestDataT, LPredictorT, PredResultsT, PerfMeasureT > const & that
)  [default]
```

Copy assignment operator.

Parameters

that	The object to copy.

9.86.3.12 resultsBegin()

```
template<typename TestDataT = TestData<>, typename LPredictorT = ULPredictor<>, typename
PredResultsT = PredResults<TestDataT, LPredictorT>, typename PerfMeasureT = PerfMeasure<↩
PredResultsT>>
auto LinkPred::PerfEvaluator< TestDataT, LPredictorT, PredResultsT, PerfMeasureT >::results↩
Begin ( ) const  [inline]
```

Returns

An iterator to the first performance result.

9.86.3.13 resultsEnd()

```
template<typename TestDataT = TestData<>, typename LPredictorT = ULPredictor<>, typename
PredResultsT = PredResults<TestDataT, LPredictorT>, typename PerfMeasureT = PerfMeasure<↩
PredResultsT>>
auto LinkPred::PerfEvaluator< TestDataT, LPredictorT, PredResultsT, PerfMeasureT >::resultsEnd
( ) const [inline]
```

Returns

An iterator to one-past-the-last first performance result.

9.86.3.14 setTimingEnabled()

```
template<typename TestDataT = TestData<>, typename LPredictorT = ULPredictor<>, typename
PredResultsT = PredResults<TestDataT, LPredictorT>, typename PerfMeasureT = PerfMeasure<↩
PredResultsT>>
void LinkPred::PerfEvaluator< TestDataT, LPredictorT, PredResultsT, PerfMeasureT >::set↩
TimingEnabled (
            bool timingEnabled ) [inline]
```

Enable/disable timing.

Parameters

timingEnabled	The new value.

The documentation for this class was generated from the following file:

- include/linkpred/perf/perfevaluator.hpp

9.87 LinkPred::PerfMeasure< PredResultsT > Class Template Reference

Abstract performance measure.

```
#include <perfmeasure.hpp>
```

Public Types

- using ScoresItT = typename PredResultsT::ScoresItT

Public Member Functions

- PerfMeasure ()=default
- PerfMeasure (std::string const &name)
- PerfMeasure (PerfMeasure const &that)=default
- PerfMeasure & operator= (PerfMeasure const &that)=default
- PerfMeasure (PerfMeasure &&that)=default
- PerfMeasure & operator= (PerfMeasure &&that)=default
- const std::string & getName () const
- void setName (std::string const &name)
- virtual bool requiresPos () const
- virtual bool requiresNeg () const
- virtual bool requiresShuffling () const
- virtual void eval (std::shared_ptr< PredResultsT > &predResults, PerfResults &results)=0
- virtual void eval (ScoresItT posScoresBegin, ScoresItT posScoresEnd, ScoresItT negScoresBegin, ScoresItT negScoresEnd, SortOrder &posSortOrder, SortOrder &negSortOrder, PerfResults &results)=0
- virtual ∼PerfMeasure ()=default

9.87.1 Detailed Description

template<typename PredResultsT = PredResults<>>
class LinkPred::PerfMeasure< PredResultsT >

Abstract performance measure.

Template Parameters

PredResultsT	The prediction results type.

9.87.2 Member Typedef Documentation

9.87.2.1 ScoresItT

```
template<typename PredResultsT = PredResults<>>
using LinkPred::PerfMeasure< PredResultsT >::ScoresItT = typename PredResultsT::ScoresItT
```

Scores iterator type.

9.87.3 Constructor & Destructor Documentation

9.87.3.1 PerfMeasure() [1/4]

```
template<typename PredResultsT = PredResults<>>
LinkPred::PerfMeasure< PredResultsT >::PerfMeasure ( )  [default]
```

Constructor.

9.87.3.2 PerfMeasure() [2/4]

```
template<typename PredResultsT = PredResults<>>
LinkPred::PerfMeasure< PredResultsT >::PerfMeasure (
            std::string const & name )  [inline]
```

Constructor.

Parameters

name	The name of the performance measure.

9.87.3.3 PerfMeasure() [3/4]

```
template<typename PredResultsT = PredResults<>>
LinkPred::PerfMeasure< PredResultsT >::PerfMeasure (
            PerfMeasure< PredResultsT > const & that )  [default]
```

Copy constructor.

Parameters

that	The object to copy.

9.87.3.4 PerfMeasure() [4/4]

```
template<typename PredResultsT = PredResults<>>
LinkPred::PerfMeasure< PredResultsT >::PerfMeasure (
            PerfMeasure< PredResultsT > && that )  [default]
```

Move constructor.

Parameters

that	The object to move.

9.87.3.5 ∼PerfMeasure()

```
template<typename PredResultsT = PredResults<>>
virtual LinkPred::PerfMeasure< PredResultsT >::∼PerfMeasure ( ) [virtual], [default]
```

Destructor.

9.87.4 Member Function Documentation

9.87.4.1 eval() [1/2]

```
template<typename PredResultsT = PredResults<>>
virtual void LinkPred::PerfMeasure< PredResultsT >::eval (
            ScoresItT posScoresBegin,
            ScoresItT posScoresEnd,
            ScoresItT negScoresBegin,
            ScoresItT negScoresEnd,
            SortOrder & posSortOrder,
            SortOrder & negSortOrder,
            PerfResults & results ) [pure virtual]
```

Computes the performance measure.

Parameters

posScoresBegin	Iterator to the first positive score.
posScoresEnd	Iterator to one-past-the-last positive score.
negScoresBegin	Iterator to the first negative score.
negScoresEnd	Iterator to one-past-the-last negative score.
posSortOrder	The sorting order of positive scores. This may be modified by the method.
negSortOrder	The sorting order of negative scores. This may be modified by the method.
results	To write results.

Implemented in LinkPred::PerfCurve< PredResults<> >.

9.87.4.2 eval() [2/2]

```
template<typename PredResultsT = PredResults<>>
virtual void LinkPred::PerfMeasure< PredResultsT >::eval (
            std::shared_ptr< PredResultsT > & predResults,
            PerfResults & results ) [pure virtual]
```

Computes the performance measure.

Parameters

predResults	The prediction results.
results	To write results.

Implemented in LinkPred::PerfCurve< PredResults<> >.

9.87.4.3 getName()

```
template<typename PredResultsT = PredResults<>>
const std::string& LinkPred::PerfMeasure< PredResultsT >::getName ( ) const [inline]
```

Returns

The name of the performance measure.

9.87.4.4 operator=() [1/2]

```
template<typename PredResultsT = PredResults<>>
PerfMeasure& LinkPred::PerfMeasure< PredResultsT >::operator= (
            PerfMeasure< PredResultsT > && that ) [default]
```

Move assignment operator.

Parameters

that	The object to move.

9.87.4.5 operator=() [2/2]

```
template<typename PredResultsT = PredResults<>>
PerfMeasure& LinkPred::PerfMeasure< PredResultsT >::operator= (
            PerfMeasure< PredResultsT > const & that ) [default]
```

Copy assignment operator.

Parameters

that	The object to copy.

9.87.4.6 requiresNeg()

```
template<typename PredResultsT = PredResults<>>
virtual bool LinkPred::PerfMeasure< PredResultsT >::requiresNeg ( ) const  [inline], [virtual]
```

Returns

Whether the performance measure requires the generation of negative set. The default value is true.

9.87.4.7 requiresPos()

```
template<typename PredResultsT = PredResults<>>
virtual bool LinkPred::PerfMeasure< PredResultsT >::requiresPos ( ) const  [inline], [virtual]
```

Returns

Whether the performance measure requires the generation of positive set. The default value is true.

9.87.4.8 requiresShuffling()

```
template<typename PredResultsT = PredResults<>>
virtual bool LinkPred::PerfMeasure< PredResultsT >::requiresShuffling ( ) const  [inline],
[virtual]
```

Returns

Whether the performance measure requires network shuffling.

9.87.4.9 setName()

```
template<typename PredResultsT = PredResults<>>
void LinkPred::PerfMeasure< PredResultsT >::setName (
             std::string const & name )  [inline]
```

Parameters

name	The name of the performance measure.

The documentation for this class was generated from the following file:

- include/linkpred/perf/perfmeasure.hpp

9.88 LinkPred::Simp::PerfRes Struct Reference

A structure to store performance results.

```
#include <perfres.hpp>
```

Public Attributes

- std::string name
- double res

9.88.1 Detailed Description

A structure to store performance results.

9.88.2 Member Data Documentation

9.88.2.1 name

```
std::string LinkPred::Simp::PerfRes::name
```

Concatenation of the name of the performance mneasure and that of the predictor.

9.88.2.2 res

```
double LinkPred::Simp::PerfRes::res
```

The result.

The documentation for this struct was generated from the following file:

- include/linkpred/simp/perfres.hpp

9.89 LinkPred::PR< PredResultsT > Class Template Reference

The precision recall curve.

```
#include <perfmeasure.hpp>
```

Inheritance diagram for LinkPred::PR< PredResultsT >:

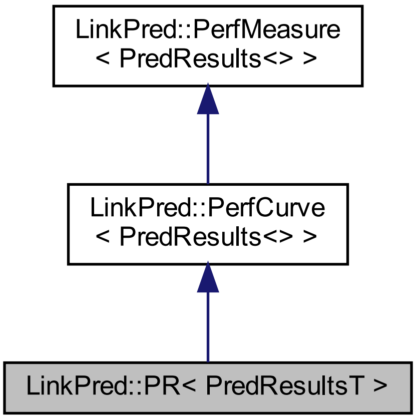

Collaboration diagram for LinkPred::PR< PredResultsT >:

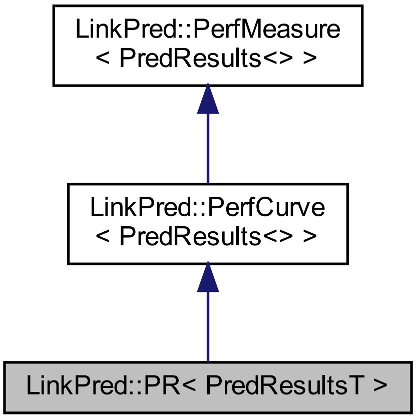

Public Types

- enum InterpolMethod { LIN, DGI }

    Interpolation methods used for the computation of the PR-AUC.
- using ScoresItT = typename PerfMeasure< PredResultsT >::ScoresItT

Public Member Functions

- PR ()
- PR (std::string name)
- PR (PR const &that)=default
- PR & operator= (PR const &that)=default
- PR (PR &&that)=default
- PR & operator= (PR &&that)=default
- virtual void eval (std::shared_ptr< PredResultsT > &predResults, PerfResults &results)
- virtual void eval (ScoresItT posScoresBegin, ScoresItT posScoresEnd, ScoresItT negScoresBegin, ScoresItT negScoresEnd, SortOrder &posSortOrder, SortOrder &negSortOrder, PerfResults &results)
- virtual std::vector< std::pair< double, double > > getCurve (std::shared_ptr< PredResultsT > &predResults)
- virtual std::vector< std::pair< double, double > > getCurve (ScoresItT posScoresBegin, ScoresItT posScoresEnd, ScoresItT negScoresBegin, ScoresItT negScoresEnd, SortOrder &posSortOrder, SortOrder &negSortOrder)
- InterpolMethod getInterpolMethod () const
- void setInterpolMethod (InterpolMethod interpolMethod)
- virtual ∼PR ()=default

Static Public Member Functions

- template<typename CountItT >
  static double getPRAucLIN (CountItT tpsBegin, CountItT tpsEnd, CountItT fpsBegin, CountItT fpsEnd, std::size_t P, bool bcz, bool parallel=false)
- template<typename CountItT >
  static double getPRAucDGI (CountItT tpsBegin, CountItT tpsEnd, CountItT fpsBegin, CountItT fpsEnd, std::size_t P, bool bcz, bool parallel=false)
- template<typename ScoresItT >
  static double getPRAuc (ScoresItT posScoresBegin, ScoresItT posScoresEnd, ScoresItT negScoresBegin, ScoresItT negScoresEnd, InterpolMethod interpolMethod, bool parallel=false)
- template<typename ScoresItT >
  static std::vector< double > getThresholds (ScoresItT posScoresBegin, ScoresItT posScoresEnd, ScoresItT negScoresBegin, ScoresItT negScoresEnd)
- template<typename ScoresItT >
  static std::vector< std::pair< double, double > > getPRCurve (ScoresItT posScoresBegin, ScoresItT posScoresEnd, ScoresItT negScoresBegin, ScoresItT negScoresEnd, bool parallel=false)

9.89.1 Detailed Description

template<typename PredResultsT = PredResults<>>
class LinkPred::PR< PredResultsT >

The precision recall curve.

Template Parameters

PredResultsT	The prediction results type.

9.89.2 Member Typedef Documentation

9.89.2.1 ScoresItT

```
template<typename PredResultsT = PredResults<>>
using LinkPred::PR< PredResultsT >::ScoresItT = typename PerfMeasure<PredResultsT>::ScoresItT
```

Scores iterator type.

9.89.3 Member Enumeration Documentation

9.89.3.1 InterpolMethod

```
template<typename PredResultsT = PredResults<>>
enum LinkPred::PR::InterpolMethod
```

Interpolation methods used for the computation of the PR-AUC.

< The name of the performance measure.

Enumerator

LIN	Linear interpolation (Trapezoidal rule).
DGI	Davis-Goadrich nonlinear interpolation.

9.89.4 Constructor & Destructor Documentation

9.89.4.1 PR() [1/4]

```
template<typename PredResultsT = PredResults<>>
LinkPred::PR< PredResultsT >::PR ( )  [inline]
```

Constructor.

9.89.4.2 PR() [2/4]

```
template<typename PredResultsT = PredResults<>>
LinkPred::PR< PredResultsT >::PR (
            std::string name )  [inline]
```

Constructor.

Parameters

name	The name of the performance measure.

9.89.4.3 PR() [3/4]

```
template<typename PredResultsT = PredResults<>>
LinkPred::PR< PredResultsT >::PR (
            PR< PredResultsT > const & that )  [default]
```

Copy constructor.

Parameters

that	The object to copy.

9.89.4.4 PR() [4/4]

```
template<typename PredResultsT = PredResults<>>
LinkPred::PR< PredResultsT >::PR (
            PR< PredResultsT > && that )  [default]
```

Move constructor.

Parameters

that	The object to move.

9.89.4.5 ∼PR()

```
template<typename PredResultsT = PredResults<>>
virtual LinkPred::PR< PredResultsT >::~PR ( )  [virtual], [default]
```

Destructor.

9.89.5 Member Function Documentation

9.89.5.1 eval() [1/2]

```
template<typename PredResultsT = PredResults<>>
virtual void LinkPred::PR< PredResultsT >::eval (
            ScoresItT posScoresBegin,
            ScoresItT posScoresEnd,
            ScoresItT negScoresBegin,
            ScoresItT negScoresEnd,
            SortOrder & posSortOrder,
            SortOrder & negSortOrder,
            PerfResults & results )  [inline], [virtual]
```

Computes the area under the PR curve.

Parameters

posScoresBegin	Iterator to the first positive score.
posScoresEnd	Iterator to one-past-the-last positive score.
negScoresBegin	Iterator to the first negative score.
negScoresEnd	Iterator to one-past-the-last negative score.
posSortOrder	The sorting order of positive scores. This may be modified by the method.
negSortOrder	The sorting order of negative scores. This may be modified by the method.
results	To write results.

9.89.5.2 eval() [2/2]

```
template<typename PredResultsT = PredResults<>>
virtual void LinkPred::PR< PredResultsT >::eval (
            std::shared_ptr< PredResultsT > & predResults,
            PerfResults & results )  [inline], [virtual]
```

Computes the performance measure.

Parameters

predResults	The prediction results.
results	Iterator to write results.

9.89.5.3 getCurve() [1/2]

```
template<typename PredResultsT = PredResults<>>
virtual std::vector<std::pair<double, double> > LinkPred::PR< PredResultsT >::getCurve (
            ScoresItT posScoresBegin,
            ScoresItT posScoresEnd,
            ScoresItT negScoresBegin,
            ScoresItT negScoresEnd,
```

```
            SortOrder & posSortOrder,
            SortOrder & negSortOrder )  [inline], [virtual]
```

Compute the PR curve.

Parameters

posScoresBegin	Iterator to the first positive score.
posScoresEnd	Iterator to one-past-the-last positive score.
negScoresBegin	Iterator to the first negative score.
negScoresEnd	Iterator to one-past-the-last negative score.
posSortOrder	The sorting order of positive scores. This may be modified by the method.
negSortOrder	The sorting order of negative scores. This may be modified by the method.

Returns

A curve in the form of an std::vector of pairs representing the x and y coordinates.

9.89.5.4 getCurve() [2/2]

```
template<typename PredResultsT = PredResults<>>
virtual std::vector<std::pair<double, double> > LinkPred::PR< PredResultsT >::getCurve (
            std::shared_ptr< PredResultsT > & predResults )  [inline], [virtual]
```

Compute the PR curve.

Parameters

predResults	The prediction results.

Returns

A curve in the form of an std::vector of pairs representing the x and y coordinates.

9.89.5.5 getInterpolMethod()

```
template<typename PredResultsT = PredResults<>>
InterpolMethod LinkPred::PR< PredResultsT >::getInterpolMethod ( ) const  [inline]
```

Returns

The interpolation method.

9.89.5.6 getPRAuc()

```
template<typename PredResultsT = PredResults<>>
template<typename ScoresItT >
static double LinkPred::PR< PredResultsT >::getPRAuc (
            ScoresItT posScoresBegin,
            ScoresItT posScoresEnd,
            ScoresItT negScoresBegin,
            ScoresItT negScoresEnd,
            InterpolMethod interpolMethod,
            bool parallel = false ) [inline], [static]
```

Compute the area under the Precision-Recall Curve. Ranges must be sorted in increasing order.

Parameters

posScoresBegin	Iterator to the first positive score.
posScoresEnd	Iterator to one-past-the-last positive score.
negScoresBegin	Iterator to the first negative score.
negScoresEnd	Iterator to one-past-the-last negative score.
interpolMethod	Interpolation method used in the integral.
parallel	Whether to run in parallel.

9.89.5.7 getPRAucDGI()

```
template<typename PredResultsT = PredResults<>>
template<typename CountItT >
static double LinkPred::PR< PredResultsT >::getPRAucDGI (
            CountItT tpsBegin,
            CountItT tpsEnd,
            CountItT fpsBegin,
            CountItT fpsEnd,
            std::size_t P,
            bool bcz,
            bool parallel = false ) [inline], [static]
```

Compute the area under the Precision-Recall Curve using Davis-Goadrich nonlinear interpolation. See: Jesse Davis and Mark Goadrich (2006) The relationship between Precision-Recall and ROC curves. Proceedings of the 23rd international conference on Machine learning. pp.233–240

Parameters

tpsBegin	Iterator to the first true positive count.
tpsEnd	Iterator to one-past-the-last true positive count.
fpsBegin	Iterator to the first false positive count.
fpsEnd	Iterator to one-past-the-last false positive count.
P	The number of positive instances.
bcz	Whether the boundary value of precision is zero.
parallel	Whether to run in parallel.

9.89.5.8 getPRAucLIN()

```
template<typename PredResultsT = PredResults<>>
template<typename CountItT >
static double LinkPred::PR< PredResultsT >::getPRAucLIN (
            CountItT tpsBegin,
            CountItT tpsEnd,
            CountItT fpsBegin,
            CountItT fpsEnd,
            std::size_t P,
            bool bcz,
            bool parallel = false )  [inline], [static]
```

Compute the area under the Precision-Recall Curve using linear interpolation (trapezoidal rule). Notice that this method may over-estimate the actual area especially when the points on the curve are distant from one-another.

Parameters

tpsBegin	Iterator to the first true positive count.
tpsEnd	Iterator to one-past-the-last true positive count.
fpsBegin	Iterator to the first false positive count.
fpsEnd	Iterator to one-past-the-last false positive count.
P	The number of positive instances.
bcz	Whether the boundary value of precision is zero.
parallel	Whether to run in parallel.

9.89.5.9 getPRCurve()

```
template<typename PredResultsT = PredResults<>>
template<typename ScoresItT >
static std::vector<std::pair<double, double> > LinkPred::PR< PredResultsT >::getPRCurve (
            ScoresItT posScoresBegin,
            ScoresItT posScoresEnd,
            ScoresItT negScoresBegin,
            ScoresItT negScoresEnd,
            bool parallel = false )  [inline], [static]
```

Compute the Precision-Recall Curve. Ranges must sorted in increasing order.

Parameters

posScoresBegin	Iterator to the first positive score.
posScoresEnd	Iterator to one-past-the-last positive score.
negScoresBegin	Iterator to the first negative score.
negScoresEnd	Iterator to one-past-the-last negative score.
parallel	Whether to run in parallel.

9.89.5.10 getThresholds()

```
template<typename PredResultsT = PredResults<>>
template<typename ScoresItT >
static std::vector<double> LinkPred::PR< PredResultsT >::getThresholds (
            ScoresItT posScoresBegin,
            ScoresItT posScoresEnd,
            ScoresItT negScoresBegin,
            ScoresItT negScoresEnd )  [inline], [static]
```

Compute the threshold of the Precision-Recall Curve. Ranges must be sorted in increasing order.

Parameters

posScoresBegin	Iterator to the first positive score.
posScoresEnd	Iterator to one-past-the-last positive score.
negScoresBegin	Iterator to the first negative score.
negScoresEnd	Iterator to one-past-the-last negative score.

9.89.5.11 operator=() [1/2]

```
template<typename PredResultsT = PredResults<>>
PR& LinkPred::PR< PredResultsT >::operator= (
            PR< PredResultsT > && that )  [default]
```

Move assignment operator.

Parameters

that	The object to move.

9.89.5.12 operator=() [2/2]

```
template<typename PredResultsT = PredResults<>>
PR& LinkPred::PR< PredResultsT >::operator= (
            PR< PredResultsT > const & that )  [default]
```

Copy assignment operator.

Parameters

that	The object to copy.

9.89.5.13 setInterpolMethod()

```
template<typename PredResultsT = PredResults<>>
void LinkPred::PR< PredResultsT >::setInterpolMethod (
            InterpolMethod interpolMethod ) [inline]
```

Set the interpolation method.

Parameters

interpolMethod	The new value of the interpolation method.

The documentation for this class was generated from the following file:

- include/linkpred/perf/perfmeasure.hpp

9.90 LinkPred::Simp::Predictor Class Reference

A class that simplifies the use of link prediction algorithms.

```
#include <predictor.hpp>
```

Public Member Functions

- Predictor ()=default
- Predictor (Predictor const &that)=default
- virtual ∼Predictor ()=default
- int getNbNodes () const
- std::string getLabel (int i) const
- int getID (std::string const &i) const
- bool isEdgeByID (int i, int j) const
- bool isEdgeByLabel (std::string const &i, std::string const &j) const
- void loadnet (std::string fileName)
- std::vector< EdgeScore > predAllADA ()
- void predADA (std::vector< EdgeScore > &edgeScores)
- std::vector< EdgeScore > predTopADA (int k)
- std::vector< EdgeScore > predAllCNE ()
- void predCNE (std::vector< EdgeScore > &edgeScores)
- std::vector< EdgeScore > predTopCNE (int k)
- std::vector< EdgeScore > predAllCRA ()
- void predCRA (std::vector< EdgeScore > &edgeScores)
- std::vector< EdgeScore > predTopCRA (int k)
- std::vector< EdgeScore > predAllECL (std::string encoderName="N2V", std::string classifierName="LGR", int dim=0, double posRatio=1.0, double negRatio=1.0, long int seed=0)
- void predECL (std::vector< EdgeScore > &edgeScores, std::string encoderName="N2V", std::string classifierName="LGR", int dim=0, double posRatio=1.0, double negRatio=1.0, long int seed=0)

- std::vector< EdgeScore > predTopECL (int k, std::string encoderName="N2V", std::string classifierName="LGR", int dim=0, double posRatio=1.0, double negRatio=1.0, long int seed=0)
- std::vector< EdgeScore > predAllESM (std::string encoderName="N2V", std::string simMeasureName="L2", int dim=0, long int seed=0)
- void predESM (std::vector< EdgeScore > &edgeScores, std::string encoderName="N2V", std::string simMeasureName="L2", int dim=0, long int seed=0)
- std::vector< EdgeScore > predTopESM (int k, std::string encoderName="N2V", std::string simMeasureName="L2", int dim=0, long int seed=0)
- std::vector< EdgeScore > predAllFBM (int maxIter=50, long int seed=0)
- void predFBM (std::vector< EdgeScore > &edgeScores, int maxIter=50, long int seed=0)
- std::vector< EdgeScore > predTopFBM (int k, int maxIter=50, long int seed=0)
- std::vector< EdgeScore > predAllHDI ()
- void predHDI (std::vector< EdgeScore > &edgeScores)
- std::vector< EdgeScore > predTopHDI (int k)
- std::vector< EdgeScore > predAllHPI ()
- void predHPI (std::vector< EdgeScore > &edgeScores)
- std::vector< EdgeScore > predTopHPI (int k)
- std::vector< EdgeScore > predAllHRG (int nbBeans=25, int nbSamples=10000, long int seed=0)
- void predHRG (std::vector< EdgeScore > &edgeScores, int nbBeans=25, int nbSamples=10000, long int seed=0)
- std::vector< EdgeScore > predTopHRG (int k, int nbBeans=25, int nbSamples=10000, long int seed=0)
- std::vector< EdgeScore > predAllHYP (double m=1.5, double L=1, double gamma=2.1, double zeta=1, double T=0.8, long int seed=0)
- void predHYP (std::vector< EdgeScore > &edgeScores, double m=1.5, double L=1, double gamma=2.1, double zeta=1, double T=0.8, long int seed=0)
- std::vector< EdgeScore > predTopHYP (int k, double m=1.5, double L=1, double gamma=2.1, double zeta=1, double T=0.8, long int seed=0)
- std::vector< EdgeScore > predAllJID ()
- void predJID (std::vector< EdgeScore > &edgeScores)
- std::vector< EdgeScore > predTopJID (int k)
- std::vector< EdgeScore > predAllKAB (int horizLim=2)
- void predKAB (std::vector< EdgeScore > &edgeScores, int horizLim=2)
- std::vector< EdgeScore > predTopKAB (int k, int horizLim=2)
- std::vector< EdgeScore > predAllLCP (double epsilon=0.001)
- void predLCP (std::vector< EdgeScore > &edgeScores, double epsilon=0.001)
- std::vector< EdgeScore > predTopLCP (int k, double epsilon=0.001)
- std::vector< EdgeScore > predAllLHN ()
- void predLHN (std::vector< EdgeScore > &edgeScores)
- std::vector< EdgeScore > predTopLHN (int k)
- std::vector< EdgeScore > predAllPAT ()
- void predPAT (std::vector< EdgeScore > &edgeScores)
- std::vector< EdgeScore > predTopPAT (int k)
- std::vector< EdgeScore > predAllRAL ()
- void predRAL (std::vector< EdgeScore > &edgeScores)
- std::vector< EdgeScore > predTopRAL (int k)
- std::vector< EdgeScore > predAllRND (long int seed=0)
- void predRND (std::vector< EdgeScore > &edgeScores, long int seed=0)
- std::vector< EdgeScore > predTopRND (int k, long int seed=0)
- std::vector< EdgeScore > predAllSAI ()
- void predSAI (std::vector< EdgeScore > &edgeScores)
- std::vector< EdgeScore > predTopSAI (int k)
- std::vector< EdgeScore > predAllSBM (int maxIter=1000, long int seed=0)
- void predSBM (std::vector< EdgeScore > &edgeScores, int maxIter=1000, long int seed=0)
- std::vector< EdgeScore > predTopSBM (int k, int maxIter=1000, long int seed=0)
- std::vector< EdgeScore > predAllSHP (long int seed=0)

- void predSHP (std::vector$<$ EdgeScore $>$ &edgeScores, long int seed=0)
- std::vector$<$ EdgeScore $>$ predTopSHP (int k, long int seed=0)
- std::vector$<$ EdgeScore $>$ predAllSOI ()
- void predSOI (std::vector$<$ EdgeScore $>$ &edgeScores)
- std::vector$<$ EdgeScore $>$ predTopSOI (int k)
- std::vector$<$ EdgeScoreByID $>$ predAllADAByID ()
- std::vector$<$ EdgeScoreByID $>$ predAllCNEByID ()
- std::vector$<$ EdgeScoreByID $>$ predAllCRAByID ()
- std::vector$<$ EdgeScoreByID $>$ predAllECLByID (std::string encoderName="N2V", std::string classifier←Name="LGR", int dim=0, double posRatio=1.0, double negRatio=1.0, long int seed=0)
- std::vector$<$ EdgeScoreByID $>$ predAllESMByID (std::string encoderName="N2V", std::string simMeasure←Name="L2", int dim=0, long int seed=0)
- std::vector$<$ EdgeScoreByID $>$ predAllFBMByID (int maxIter=50, long int seed=0)
- std::vector$<$ EdgeScoreByID $>$ predAllHDIByID ()
- std::vector$<$ EdgeScoreByID $>$ predAllHPIByID ()
- std::vector$<$ EdgeScoreByID $>$ predAllHRGByID (int nbBeans=25, int nbSamples=10000, long int seed=0)
- std::vector$<$ EdgeScoreByID $>$ predAllHYPByID (double m=1.5, double L=1, double gamma=2.1, double zeta=1, double T=0.8, long int seed=0)
- std::vector$<$ EdgeScoreByID $>$ predAllJIDByID ()
- std::vector$<$ EdgeScoreByID $>$ predAllKABByID (int horizLim=2)
- std::vector$<$ EdgeScoreByID $>$ predAllLCPByID (double epsilon=0.001)
- std::vector$<$ EdgeScoreByID $>$ predAllLHNByID ()
- std::vector$<$ EdgeScoreByID $>$ predAllPATByID ()
- std::vector$<$ EdgeScoreByID $>$ predAllRALByID ()
- std::vector$<$ EdgeScoreByID $>$ predAllRNDByID (long int seed=0)
- std::vector$<$ EdgeScoreByID $>$ predAllSAIByID ()
- std::vector$<$ EdgeScoreByID $>$ predAllSBMByID (int maxIter=1000, long int seed=0)
- std::vector$<$ EdgeScoreByID $>$ predAllSHPByID (long int seed=0)
- std::vector$<$ EdgeScoreByID $>$ predAllSOIByID ()

9.90.1 Detailed Description

A class that simplifies the use of link prediction algorithms.

9.90.2 Constructor & Destructor Documentation

9.90.2.1 Predictor() [1/2]

```
LinkPred::Simp::Predictor::Predictor ( )  [default]
```

Parameters

net	The network.

9.90.2.2 Predictor() [2/2]

```
LinkPred::Simp::Predictor::Predictor (
            Predictor const & that )  [default]
```

Copy constructor.

Parameters

that	The object to copy.

9.90.2.3 ∼Predictor()

```
virtual LinkPred::Simp::Predictor::~Predictor ( )  [virtual], [default]
```

Destructor.

9.90.3 Member Function Documentation

9.90.3.1 getID()

```
int LinkPred::Simp::Predictor::getID (
            std::string const & i ) const
```

Translate node label to ID.

Parameters

i	Node label.

Returns

The node ID.

9.90.3.2 getLabel()

```
std::string LinkPred::Simp::Predictor::getLabel (
            int i ) const
```

Translate node ID to label.

Parameters

i	Node internal ID (sequential from 0 to nbNodes-1).

Returns

The node label.

9.90.3.3 getNbNodes()

```
int LinkPred::Simp::Predictor::getNbNodes ( ) const
```

Returns the number of nodes in the network.

Returns

The number of nodes in the network.

9.90.3.4 isEdgeByID()

```
bool LinkPred::Simp::Predictor::isEdgeByID (
            int i,
            int j ) const
```

Check if an edge exists using internal node IDs.

Parameters

i	ID of start node.
j	ID of end node.

Returns

True if (i, j) is an edge, false otherwise.

9.90.3.5 isEdgeByLabel()

```
bool LinkPred::Simp::Predictor::isEdgeByLabel (
            std::string const & i,
            std::string const & j ) const
```

Check if an edge exists using internal node IDs.

Parameters

i	ID of start node.
j	ID of end node.

Returns

True if (i, j) is an edge, false otherwise.

9.90.3.6 loadnet()

```
void LinkPred::Simp::Predictor::loadnet (
            std::string fileName )
```

Load network from file. The format is list of edges. Comments must be on separate lines and start with #.

Parameters

fileName	The file name.

9.90.3.7 predADA()

```
void LinkPred::Simp::Predictor::predADA (
            std::vector< EdgeScore > & edgeScores )
```

Adamic Adar predictor.

Parameters

edgeScores	A input vector of negative edges. The score of each edge will be written in the member score.

9.90.3.8 predAllADA()

```
std::vector<EdgeScore> LinkPred::Simp::Predictor::predAllADA ( )
```

Adamic Adar predictor.

Returns

The score of all negative edges.

9.90.3.9 predAllADAByID()

```
std::vector<EdgeScoreByID> LinkPred::Simp::Predictor::predAllADAByID ( )
```

Adamic Adar predictor.

Returns

The score of all negative edges.

9.90.3.10 predAllCNE()

```
std::vector<EdgeScore> LinkPred::Simp::Predictor::predAllCNE ( )
```

Common neighbors.

Returns

The score of all negative edges.

9.90.3.11 predAllCNEByID()

```
std::vector<EdgeScoreByID> LinkPred::Simp::Predictor::predAllCNEByID ( )
```

Common neighbors.

Returns

The score of all negative edges.

9.90.3.12 predAllCRA()

```
std::vector<EdgeScore> LinkPred::Simp::Predictor::predAllCRA ( )
```

Cannistraci resource allocation.

Returns

The score of all negative edges.

9.90.3.13 predAllCRAByID()

```
std::vector<EdgeScoreByID> LinkPred::Simp::Predictor::predAllCRAByID ( )
```

Cannistraci resource allocation.

Returns

The score of all negative edges.

9.90.3.14 predAllECL()

```
std::vector<EdgeScore> LinkPred::Simp::Predictor::predAllECL (
            std::string encoderName = "N2V",
            std::string classifierName = "LGR",
            int dim = 0,
            double posRatio = 1.0,
            double negRatio = 1.0,
            long int seed = 0 )
```

Encoder-classifier link predictor.

Parameters

encoderName	The name of the encoder. Possible values are: DPW (DeepWalk), HMSM (Hidden Metric Space Model), LVS (LargeVis), LEM (Laplacian Eigenmaps), LIN (LINE), LLE (Locally Linear Embedding), MFC (Matrix Factorization), and N2V (Node2Vec).
classifierName	The name of the classifier. Possible values are: FFN (feed-forward neural network withn default architecture), LSVM (linear SVM), LGR (logistic regression), NVB (naive Bayes). All classifiers except logistic regression requirte compilation with mlpack.
dim	The dimension of the embedding space. If set to zero, the default value is used (the default dimension depends on the ecnoder).
posRatio	Ratio of positive edges used in the training of the classifier.
negRatio	Ratio of negative edges used in the training of the classifier.
seed	Seed of the random number generator.

Returns

The score of all negative edges.

9.90.3.15 predAllECLByID()

```
std::vector<EdgeScoreByID> LinkPred::Simp::Predictor::predAllECLByID (
            std::string encoderName = "N2V",
            std::string classifierName = "LGR",
            int dim = 0,
```

```
            double posRatio = 1.0,
            double negRatio = 1.0,
            long int seed = 0 )
```

Encoder-classifier link predictor.

Parameters

encoderName	The name of the encoder. Possible values are: DPW (DeepWalk), HMSM (Hidden Metric Space Model), LVS (LargeVis), LEM (Laplacian Eigenmaps), LIN (LINE), LLE (Locally Linear Embedding), MFC (Matrix Factorization), and N2V (Node2Vec).
classifierName	The name of the classifier. Possible values are: FFN (feed-forward neural network withn default architecture), LSVM (linear SVM), LGR (logistic regression), NVB (naive Bayes). All classifiers except logistic regression requirte compilation with mlpack.
dim	The dimension of the embedding space. If set to zero, the default value is used (the default dimension depends on the ecnoder).
posRatio	Ratio of positive edges used in the training of the classifier.
negRatio	Ratio of negative edges used in the training of the classifier.
seed	Seed of the random number generator.

Returns

The score of all negative edges.

9.90.3.16 predAllESM()

```
std::vector<EdgeScore> LinkPred::Simp::Predictor::predAllESM (
            std::string encoderName = "N2V",
            std::string simMeasureName = "L2",
            int dim = 0,
            long int seed = 0 )
```

Encoder-similarity measure link predictor.

Parameters

encoderName	The name of the encoder. Possible values are: DPW (DeepWalk), HMSM (Hidden Metric Space Model), LVS (LargeVis), LEM (Laplacian Eigenmaps), LIN (LINE), LLE (Locally Linear Embedding), MFC (Matrix Factorization), and N2V (Node2Vec).
simMeasureName	The name of the similarity measure. Possible values are: CSM (cosine similarity), DTP (dot product), L1 (L1 similarity), L2 (L2 similarity), PRS (Pearson similarity).
dim	The dimension of the embedding space. If set to zero, the default value is used (the default dimension depends on the ecnoder).
seed	Seed of the random number generator.

Returns

The score of all negative edges.

9.90.3.17 predAllESMByID()

```
std::vector<EdgeScoreByID> LinkPred::Simp::Predictor::predAllESMByID (
            std::string encoderName = "N2V",
            std::string simMeasureName = "L2",
            int dim = 0,
            long int seed = 0 )
```

Encoder-similarity measure link predictor.

Parameters

encoderName	The name of the encoder. Possible values are: DPW (DeepWalk), HMSM (Hidden Metric Space Model), LVS (LargeVis), LEM (Laplacian Eigenmaps), LIN (LINE), LLE (Locally Linear Embedding), MFC (Matrix Factorization), and N2V (Node2Vec).
simMeasureName	The name of the similarity measure. Possible values are: CSM (cosine similarity), DTP (dot product), L1 (L1 similarity), L2 (L2 similarity), PRS (Pearson similarity).
dim	The dimension of the embedding space. If set to zero, the default value is used (the default dimension depends on the ecnoder).
seed	Seed of the random number generator.

Returns

The score of all negative edges.

9.90.3.18 predAllFBM()

```
std::vector<EdgeScore> LinkPred::Simp::Predictor::predAllFBM (
            int maxIter = 50,
            long int seed = 0 )
```

Fast blocking model.

Parameters

maxIter	Maximum number of iterations.
seed	Seed of the random number generator.

Returns

The score of all negative edges.

9.90.3.19 predAllFBMByID()

```
std::vector<EdgeScoreByID> LinkPred::Simp::Predictor::predAllFBMById (
            int maxIter = 50,
            long int seed = 0 )
```

Fast blocking model.

Parameters

maxIter	Maximum number of iterations.
seed	Seed of the random number generator.

Returns

The score of all negative edges.

9.90.3.20 predAllHDI()

`std::vector<EdgeScore> LinkPred::Simp::Predictor::predAllHDI ( )`

Hub depromoted index.

Returns

The score of all negative edges.

9.90.3.21 predAllHDIByID()

`std::vector<EdgeScoreByID> LinkPred::Simp::Predictor::predAllHDIByID ( )`

Hub depromoted index.

Returns

The score of all negative edges.

9.90.3.22 predAllHPI()

`std::vector<EdgeScore> LinkPred::Simp::Predictor::predAllHPI ( )`

Hub promoted index.

Returns

The score of all negative edges.

9.90.3.23 predAllHPIByID()

```
std::vector<EdgeScoreByID> LinkPred::Simp::Predictor::predAllHPIByID ( )
```

Hub promoted index.

Returns

The score of all negative edges.

9.90.3.24 predAllHRG()

```
std::vector<EdgeScore> LinkPred::Simp::Predictor::predAllHRG (
            int nbBeans = 25,
            int nbSamples = 10000,
            long int seed = 0 )
```

Hierarchical random graph.

Parameters

nbBeans	Number of bins in edge statistics histogram.
nbSamples	Number of samples to take for predictions.
seed	Seed of the random number generator.

Returns

The score of all negative edges.

9.90.3.25 predAllHRGByID()

```
std::vector<EdgeScoreByID> LinkPred::Simp::Predictor::predAllHRGByID (
            int nbBeans = 25,
            int nbSamples = 10000,
            long int seed = 0 )
```

Hierarchical random graph.

Parameters

nbBeans	Number of bins in edge statistics histogram.
nbSamples	Number of samples to take for predictions.
seed	Seed of the random number generator.

Returns

The score of all negative edges.

9.90.3.26 predAllHYP()

```
std::vector<EdgeScore> LinkPred::Simp::Predictor::predAllHYP (
            double m = 1.5,
            double L = 1,
            double gamma = 2.1,
            double zeta = 1,
            double T = 0.8,
            long int seed = 0 )
```

Hypermap.

Parameters

m	The parameter m (see the algorithm description).
L	The parameter L (see the algorithm description).
gamma	The power law exponent gamma (see the algorithm description).
zeta	The parameter zeta (see the algorithm description).
T	The parameter T (see the algorithm description).
seed	The random number generator seed.

Returns

The score of all negative edges.

9.90.3.27 predAllHYPByID()

```
std::vector<EdgeScoreByID> LinkPred::Simp::Predictor::predAllHYPByID (
            double m = 1.5,
            double L = 1,
            double gamma = 2.1,
            double zeta = 1,
            double T = 0.8,
            long int seed = 0 )
```

Hypermap.

Parameters

m	The parameter m (see the algorithm description).
L	The parameter L (see the algorithm description).
gamma	The power law exponent gamma (see the algorithm description).
zeta	The parameter zeta (see the algorithm description).
T	The parameter T (see the algorithm description).
seed	The random number generator seed.

Returns

The score of all negative edges.

9.90.3.28 predAllJID()

```
std::vector<EdgeScore> LinkPred::Simp::Predictor::predAllJID ( )
```

Jackard index.

Returns

The score of all negative edges.

9.90.3.29 predAllJIDByID()

```
std::vector<EdgeScoreByID> LinkPred::Simp::Predictor::predAllJIDByID ( )
```

Jackard index.

Returns

The score of all negative edges.

9.90.3.30 predAllKAB()

```
std::vector<EdgeScore> LinkPred::Simp::Predictor::predAllKAB (
            int horizLim = 2 )
```

A scalable popularity similarity link predictor proposed in: "Kerrache, S., Alharbi, R. & Benhidour, H. A Scalable Similarity-Popularity Link Prediction Method. Sci Rep 10, 6394 (2020)".

Parameters

horizLim	Horizon limit.

Returns

The score of all negative edges.

9.90.3.31 predAllKABByID()

```
std::vector<EdgeScoreByID> LinkPred::Simp::Predictor::predAllKABByID (
            int horizLim = 2 )
```

A scalable popularity similarity link predictor proposed in: "Kerrache, S., Alharbi, R. & Benhidour, H. A Scalable Similarity-Popularity Link Prediction Method. Sci Rep 10, 6394 (2020)".

Parameters

horizLim	Horizon limit.

Returns

The score of all negative edges.

9.90.3.32 predAllLCP()

```
std::vector<EdgeScore> LinkPred::Simp::Predictor::predAllLCP (
            double epsilon = 0.001 )
```

Local path.

Parameters

epsilon	The weight of paths of length 3.

Returns

The score of all negative edges.

9.90.3.33 predAllLCPByID()

```
std::vector<EdgeScoreByID> LinkPred::Simp::Predictor::predAllLCPByID (
            double epsilon = 0.001 )
```

Local path.

Parameters

epsilon	The weight of paths of length 3.

Returns

The score of all negative edges.

9.90.3.34 predAllLHN()

```
std::vector<EdgeScore> LinkPred::Simp::Predictor::predAllLHN ( )
```

Leicht-Holme-Newman index.

Returns

The score of all negative edges.

9.90.3.35 predAllLHNByID()

```
std::vector<EdgeScoreByID> LinkPred::Simp::Predictor::predAllLHNByID ( )
```

Leicht-Holme-Newman index.

Returns

The score of all negative edges.

9.90.3.36 predAllPAT()

```
std::vector<EdgeScore> LinkPred::Simp::Predictor::predAllPAT ( )
```

Preferential attachment index.

Returns

The score of all negative edges.

9.90.3.37 predAllPATByID()

```
std::vector<EdgeScoreByID> LinkPred::Simp::Predictor::predAllPATByID ( )
```

Preferential attachment index.

Returns

The score of all negative edges.

9.90.3.38 predAllRAL()

```
std::vector<EdgeScore> LinkPred::Simp::Predictor::predAllRAL ( )
```

Resource allocation index.

Returns

The score of all negative edges.

9.90.3.39 predAllRALByID()

```
std::vector<EdgeScoreByID> LinkPred::Simp::Predictor::predAllRALByID ( )
```

Resource allocation index.

Returns

The score of all negative edges.

9.90.3.40 predAllRND()

```
std::vector<EdgeScore> LinkPred::Simp::Predictor::predAllRND (
            long int seed = 0 )
```

Random predictor.

Parameters

seed	The random number generator seed.

Returns

The score of all negative edges.

9.90.3.41 predAllRNDByID()

```
std::vector<EdgeScoreByID> LinkPred::Simp::Predictor::predAllRNDByID (
            long int seed = 0 )
```

Random predictor.

Parameters

seed	The random number generator seed.

Returns

The score of all negative edges.

9.90.3.42 predAllSAI()

```
std::vector<EdgeScore> LinkPred::Simp::Predictor::predAllSAI ( )
```

Salton index.

Returns

The score of all negative edges.

9.90.3.43 predAllSAIByID()

```
std::vector<EdgeScoreByID> LinkPred::Simp::Predictor::predAllSAIByID ( )
```

Salton index.

Returns

The score of all negative edges.

9.90.3.44 predAllSBM()

```
std::vector<EdgeScore> LinkPred::Simp::Predictor::predAllSBM (
            int maxIter = 1000,
            long int seed = 0 )
```

Stochastic blocking model.

Parameters

maxIter	Maximum number of iterations.
seed	Seed of the random number generator.

Returns

The score of all negative edges.

9.90.3.45 predAllSBMByID()

```
std::vector<EdgeScoreByID> LinkPred::Simp::Predictor::predAllSBMByID (
            int maxIter = 1000,
            long int seed = 0 )
```

Stochastic blocking model.

Parameters

maxIter	Maximum number of iterations.
seed	Seed of the random number generator.

Returns

The score of all negative edges.

9.90.3.46 predAllSHP()

```
std::vector<EdgeScore> LinkPred::Simp::Predictor::predAllSHP (
            long int seed = 0 )
```

Shortest path predictor.

Parameters

seed	The random number generator seed.

Returns

The score of all negative edges.

9.90.3.47 predAllSHPByID()

```
std::vector<EdgeScoreByID> LinkPred::Simp::Predictor::predAllSHPByID (
            long int seed = 0 )
```

Shortest path predictor.

Parameters

seed	The random number generator seed.

Returns

The score of all negative edges.

9.90.3.48 predAllSOI()

```
std::vector<EdgeScore> LinkPred::Simp::Predictor::predAllSOI ( )
```

Sorensen index.

Returns

The score of all negative edges.

9.90.3.49 predAllSOIByID()

```
std::vector<EdgeScoreByID> LinkPred::Simp::Predictor::predAllSOIByID ( )
```

Sorensen index.

Returns

The score of all negative edges.

9.90.3.50 predCNE()

```
void LinkPred::Simp::Predictor::predCNE (
            std::vector< EdgeScore > & edgeScores )
```

Common neighbors.

Parameters

edgeScores	A input vector of negative edges. The score of each edge will be written in the member score.

9.90.3.51 predCRA()

```
void LinkPred::Simp::Predictor::predCRA (
            std::vector< EdgeScore > & edgeScores )
```

Cannistraci resource allocation.

Parameters

edgeScores	A input vector of negative edges. The score of each edge will be written in the member score.

9.90.3.52 predECL()

```
void LinkPred::Simp::Predictor::predECL (
            std::vector< EdgeScore > & edgeScores,
            std::string encoderName = "N2V",
            std::string classifierName = "LGR",
            int dim = 0,
            double posRatio = 1.0,
            double negRatio = 1.0,
            long int seed = 0 )
```

Encoder-classifier link predictor.

Parameters

edgeScores	A input vector of negative edges. The score of each edge will be written in the member score.
encoderName	The name of the encoder. Possible values are: DPW (DeepWalk), HMSM (Hidden Metric Space Model), LVS (LargeVis), LEM (Laplacian Eigenmaps), LIN (LINE), LLE (Locally Linear Embedding), MFC (Matrix Factorization), and N2V (Node2Vec).
classifierName	The name of the classifier. Possible values are: FFN (feed-forward neural network withn default architecture), LSVM (linear SVM), LGR (logistic regression), NVB (naive Bayes). All classifiers except logistic regression requirte compilation with mlpack.
dim	The dimension of the embedding space. If set to zero, the default value is used (the default dimension depends on the ecnoder).
posRatio	Ratio of positive edges used in the training of the classifier.
negRatio	Ratio of negative edges used in the training of the classifier.
seed	Seed of the random number generator.

9.90.3.53 predESM()

```
void LinkPred::Simp::Predictor::predESM (
            std::vector< EdgeScore > & edgeScores,
            std::string encoderName = "N2V",
            std::string simMeasureName = "L2",
```

```
            int dim = 0,
            long int seed = 0 )
```

Encoder-similarity measure link predictor.

Parameters

edgeScores	A input vector of negative edges. The score of each edge will be written in the member score.
encoderName	The name of the encoder. Possible values are: DPW (DeepWalk), HMSM (Hidden Metric Space Model), LVS (LargeVis),LEM (Laplacian Eigenmaps), LIN (LINE), LLE (Locally Linear Embedding), MFC (Matrix Factorization), and N2V (Node2Vec).
simMeasureName	The name of the similarity measure. Possible values are: CSM (cosine similarity), DTP (dot product), L1 (L1 similarity), L2 (L2 similarity), PRS (Pearson similarity).
dim	The dimension of the embedding space. If set to zero, the default value is used (the default dimension depends on the ecnoder).
seed	Seed of the random number generator.

9.90.3.54 predFBM()

```
void LinkPred::Simp::Predictor::predFBM (
            std::vector< EdgeScore > & edgeScores,
            int maxIter = 50,
            long int seed = 0 )
```

Fast blocking model.

Parameters

edgeScores	A input vector of negative edges. The score of each edge will be written in the member score.
maxIter	Maximum number of iterations.
seed	Seed of the random number generator.

9.90.3.55 predHDI()

```
void LinkPred::Simp::Predictor::predHDI (
            std::vector< EdgeScore > & edgeScores )
```

Hub depromoted index.

Parameters

edgeScores	A input vector of negative edges. The score of each edge will be written in the member score.

9.90.3.56 predHPI()

```
void LinkPred::Simp::Predictor::predHPI (
            std::vector< EdgeScore > & edgeScores )
```

Hub promoted index.

Parameters

edgeScores	A input vector of negative edges. The score of each edge will be written in the member score.

9.90.3.57 predHRG()

```
void LinkPred::Simp::Predictor::predHRG (
            std::vector< EdgeScore > & edgeScores,
            int nbBeans = 25,
            int nbSamples = 10000,
            long int seed = 0 )
```

Hierarchical random graph.

Parameters

edgeScores	A input vector of negative edges. The score of each edge will be written in the member score.
nbBeans	Number of bins in edge statistics histogram.
nbSamples	Number of samples to take for predictions.
seed	Seed of the random number generator.

9.90.3.58 predHYP()

```
void LinkPred::Simp::Predictor::predHYP (
            std::vector< EdgeScore > & edgeScores,
            double m = 1.5,
            double L = 1,
            double gamma = 2.1,
            double zeta = 1,
            double T = 0.8,
            long int seed = 0 )
```

Hypermap.

Parameters

edgeScores	A input vector of negative edges. The score of each edge will be written in the member score.
m	The parameter m (see the algorithm description).
L	The parameter L (see the algorithm description).

Parameters

gamma	The power law exponent gamma (see the algorithm description).
zeta	The parameter zeta (see the algorithm description).
T	The parameter T (see the algorithm description).
seed	The random number generator seed.

9.90.3.59 predJID()

```
void LinkPred::Simp::Predictor::predJID (
            std::vector< EdgeScore > & edgeScores )
```

Jackard index.

Parameters

edgeScores	A input vector of negative edges. The score of each edge will be written in the member score.

9.90.3.60 predKAB()

```
void LinkPred::Simp::Predictor::predKAB (
            std::vector< EdgeScore > & edgeScores,
            int horizLim = 2 )
```

A scalable popularity similarity link predictor proposed in: "Kerrache, S., Alharbi, R. & Benhidour, H. A Scalable Similarity-Popularity Link Prediction Method. Sci Rep 10, 6394 (2020)".

Parameters

edgeScores	A input vector of negative edges. The score of each edge will be written in the member score.
horizLim	Horizon limit.

9.90.3.61 predLCP()

```
void LinkPred::Simp::Predictor::predLCP (
            std::vector< EdgeScore > & edgeScores,
            double epsilon = 0.001 )
```

Local path.

Parameters

edgeScores	A input vector of negative edges. The score of each edge will be written in the member score.
epsilon	The weight of paths of length 3.

9.90.3.62 predLHN()

```
void LinkPred::Simp::Predictor::predLHN (
            std::vector< EdgeScore > & edgeScores )
```

Leicht-Holme-Newman index.

Parameters

edgeScores	A input vector of negative edges. The score of each edge will be written in the member score.

9.90.3.63 predPAT()

```
void LinkPred::Simp::Predictor::predPAT (
            std::vector< EdgeScore > & edgeScores )
```

Preferential attachment index.

Parameters

edgeScores	A input vector of negative edges. The score of each edge will be written in the member score.

9.90.3.64 predRAL()

```
void LinkPred::Simp::Predictor::predRAL (
            std::vector< EdgeScore > & edgeScores )
```

Resource allocation index.

Parameters

edgeScores	A input vector of negative edges. The score of each edge will be written in the member score.

9.90.3.65 predRND()

```
void LinkPred::Simp::Predictor::predRND (
            std::vector< EdgeScore > & edgeScores,
            long int seed = 0 )
```

Random predictor.

Parameters

edgeScores	A input vector of negative edges. The score of each edge will be written in the member score.
seed	The random number generator seed.

9.90.3.66 predSAI()

```
void LinkPred::Simp::Predictor::predSAI (
            std::vector< EdgeScore > & edgeScores )
```

Salton index.

Parameters

edgeScores	A input vector of negative edges. The score of each edge will be written in the member score.

9.90.3.67 predSBM()

```
void LinkPred::Simp::Predictor::predSBM (
            std::vector< EdgeScore > & edgeScores,
            int maxIter = 1000,
            long int seed = 0 )
```

Stochastic blocking model.

Parameters

edgeScores	A input vector of negative edges. The score of each edge will be written in the member score.
maxIter	Maximum number of iterations.
seed	Seed of the random number generator.

9.90.3.68 predSHP()

```
void LinkPred::Simp::Predictor::predSHP (
```

```
            std::vector< EdgeScore > & edgeScores,
            long int seed = 0 )
```

Shortest path predictor.

Parameters

edgeScores	A input vector of negative edges. The score of each edge will be written in the member score.
seed	The random number generator seed.

9.90.3.69 predSOI()

```
void LinkPred::Simp::Predictor::predSOI (
            std::vector< EdgeScore > & edgeScores )
```

Sorensen index.

Parameters

edgeScores	A input vector of negative edges. The score of each edge will be written in the member score.

9.90.3.70 predTopADA()

```
std::vector<EdgeScore> LinkPred::Simp::Predictor::predTopADA (
            int k )
```

Adamic Adar predictor.

Parameters

k	Number of edges to be returned (the actual number may be smaller).

Returns

The top k negative edge scores (the actual size may of the output be smaller than k).

9.90.3.71 predTopCNE()

```
std::vector<EdgeScore> LinkPred::Simp::Predictor::predTopCNE (
            int k )
```

Common neighbors.

Parameters

k	Number of edges to be returned (the actual number may be smaller).

Returns

The top k negative edge scores (the actual size may of the output be smaller than k).

9.90.3.72 predTopCRA()

```
std::vector<EdgeScore> LinkPred::Simp::Predictor::predTopCRA (
            int k )
```

Cannistraci resource allocation.

Parameters

k	Number of edges to be returned (the actual number may be smaller).

Returns

The top k negative edge scores (the actual size may of the output be smaller than k).

9.90.3.73 predTopECL()

```
std::vector<EdgeScore> LinkPred::Simp::Predictor::predTopECL (
            int k,
            std::string encoderName = "N2V",
            std::string classifierName = "LGR",
            int dim = 0,
            double posRatio = 1.0,
            double negRatio = 1.0,
            long int seed = 0 )
```

Encoder-classifier link predictor.

Parameters

k	Number of edges to be returned (the actual number may be smaller).
encoderName	The name of the encoder. Possible values are: DPW (DeepWalk), HMSM (Hidden Metric Space Model), LVS (LargeVis),LEM (Laplacian Eigenmaps), LIN (LINE), LLE (Locally Linear Embedding), MFC (Matrix Factorization), and N2V (Node2Vec).
classifierName	The name of the classifier. Possible values are: FFN (feed-forward neural network withn default architecture), LSVM (linear SVM), LGR (logistic regression), NVB (naive Bayes). All classifiers except logistic regression requirte compilation with mlpack.

Parameters

dim	The dimension of the embedding space. If set to zero, the default value is used (the default dimension depends on the ecnoder).
posRatio	Ratio of positive edges used in the training of the classifier.
negRatio	Ratio of negative edges used in the training of the classifier.
seed	Seed of the random number generator.

Returns

The top k negative edge scores (the actual size may of the output be smaller than k).

9.90.3.74 predTopESM()

```
std::vector<EdgeScore> LinkPred::Simp::Predictor::predTopESM (
            int k,
            std::string encoderName = "N2V",
            std::string simMeasureName = "L2",
            int dim = 0,
            long int seed = 0 )
```

Encoder-similarity measure link predictor.

Parameters

k	Number of edges to be returned (the actual number may be smaller).
encoderName	The name of the encoder. Possible values are: DPW (DeepWalk), HMSM (Hidden Metric Space Model), LVS (LargeVis), LEM (Laplacian Eigenmaps), LIN (LINE), LLE (Locally Linear Embedding), MFC (Matrix Factorization), and N2V (Node2Vec).
simMeasureName	The name of the similarity measure. Possible values are: CSM (cosine similarity), DTP (dot product), L1 (L1 similarity), L2 (L2 similarity), PRS (Pearson similarity).
dim	The dimension of the embedding space. If set to zero, the default value is used (the default dimension depends on the ecnoder).
seed	Seed of the random number generator.

Returns

The top k negative edge scores (the actual size may of the output be smaller than k).

9.90.3.75 predTopFBM()

```
std::vector<EdgeScore> LinkPred::Simp::Predictor::predTopFBM (
            int k,
            int maxIter = 50,
            long int seed = 0 )
```

Fast blocking model.

Parameters

k	Number of edges to be returned (the actual number may be smaller).
maxIter	Maximum number of iterations.
seed	Seed of the random number generator.

Returns

The top k negative edge scores (the actual size may of the output be smaller than k).

9.90.3.76 predTopHDI()

```
std::vector<EdgeScore> LinkPred::Simp::Predictor::predTopHDI (
            int k )
```

Hub depromoted index.

Parameters

k	Number of edges to be returned (the actual number may be smaller).

Returns

The top k negative edge scores (the actual size may of the output be smaller than k).

9.90.3.77 predTopHPI()

```
std::vector<EdgeScore> LinkPred::Simp::Predictor::predTopHPI (
            int k )
```

Hub promoted index.

Parameters

k	Number of edges to be returned (the actual number may be smaller).

Returns

The top k negative edge scores (the actual size may of the output be smaller than k).

9.90.3.78 predTopHRG()

```
std::vector<EdgeScore> LinkPred::Simp::Predictor::predTopHRG (
            int k,
            int nbBeans = 25,
            int nbSamples = 10000,
            long int seed = 0 )
```

Hierarchical random graph.

Parameters

k	Number of edges to be returned (the actual number may be smaller).
nbBeans	Number of bins in edge statistics histogram.
nbSamples	Number of samples to take for predictions.
seed	Seed of the random number generator.

Returns

The top k negative edge scores (the actual size may of the output be smaller than k).

9.90.3.79 predTopHYP()

```
std::vector<EdgeScore> LinkPred::Simp::Predictor::predTopHYP (
            int k,
            double m = 1.5,
            double L = 1,
            double gamma = 2.1,
            double zeta = 1,
            double T = 0.8,
            long int seed = 0 )
```

Hypermap.

Parameters

k	Number of edges to be returned (the actual number may be smaller).
m	The parameter m (see the algorithm description).
L	The parameter L (see the algorithm description).
gamma	The power law exponent gamma (see the algorithm description).
zeta	The parameter zeta (see the algorithm description).
T	The parameter T (see the algorithm description).
seed	The random number generator seed.

Returns

The top k negative edge scores (the actual size may of the output be smaller than k).

9.90.3.80 predTopJID()

```
std::vector<EdgeScore> LinkPred::Simp::Predictor::predTopJID (
            int k )
```

Jackard index.

Parameters

k	Number of edges to be returned (the actual number may be smaller).

Returns

The top k negative edge scores (the actual size may of the output be smaller than k).

9.90.3.81 predTopKAB()

```
std::vector<EdgeScore> LinkPred::Simp::Predictor::predTopKAB (
            int k,
            int horizLim = 2 )
```

A scalable popularity similarity link predictor proposed in: "Kerrache, S., Alharbi, R. & Benhidour, H. A Scalable Similarity-Popularity Link Prediction Method. Sci Rep 10, 6394 (2020)".

Parameters

k	Number of edges to be returned (the actual number may be smaller).
horizLim	Horizon limit.

Returns

The top k negative edge scores (the actual size may of the output be smaller than k).

9.90.3.82 predTopLCP()

```
std::vector<EdgeScore> LinkPred::Simp::Predictor::predTopLCP (
            int k,
            double epsilon = 0.001 )
```

Local path.

Parameters

k	Number of edges to be returned (the actual number may be smaller).
epsilon	The weight of paths of length 3.

Returns

The top k negative edge scores (the actual size may of the output be smaller than k).

9.90.3.83 predTopLHN()

```
std::vector<EdgeScore> LinkPred::Simp::Predictor::predTopLHN (
            int k )
```

Leicht-Holme-Newman index.

Parameters

k	Number of edges to be returned (the actual number may be smaller).

Returns

The top k negative edge scores (the actual size may of the output be smaller than k).

9.90.3.84 predTopPAT()

```
std::vector<EdgeScore> LinkPred::Simp::Predictor::predTopPAT (
            int k )
```

Preferential attachment index.

Parameters

k	Number of edges to be returned (the actual number may be smaller).

Returns

The top k negative edge scores (the actual size may of the output be smaller than k).

9.90.3.85 predTopRAL()

```
std::vector<EdgeScore> LinkPred::Simp::Predictor::predTopRAL (
            int k )
```

Resource allocation index.

Parameters

k	Number of edges to be returned (the actual number may be smaller).

Returns

The top k negative edge scores (the actual size may of the output be smaller than k).

9.90.3.86 predTopRND()

```
std::vector<EdgeScore> LinkPred::Simp::Predictor::predTopRND (
            int k,
            long int seed = 0 )
```

Random predictor.

Parameters

k	Number of edges to be returned (the actual number may be smaller).
seed	The random number generator seed.

Returns

The top k negative edge scores (the actual size may of the output be smaller than k).

9.90.3.87 predTopSAI()

```
std::vector<EdgeScore> LinkPred::Simp::Predictor::predTopSAI (
            int k )
```

Salton index.

Parameters

k	Number of edges to be returned (the actual number may be smaller).

Returns

The top k negative edge scores (the actual size may of the output be smaller than k).

9.90.3.88 predTopSBM()

```
std::vector<EdgeScore> LinkPred::Simp::Predictor::predTopSBM (
            int k,
            int maxIter = 1000,
            long int seed = 0 )
```

Stochastic blocking model.

Parameters

k	Number of edges to be returned (the actual number may be smaller).
maxIter	Maximum number of iterations.
seed	Seed of the random number generator.

Returns

The top k negative edge scores (the actual size may of the output be smaller than k).

9.90.3.89 predTopSHP()

```
std::vector<EdgeScore> LinkPred::Simp::Predictor::predTopSHP (
            int k,
            long int seed = 0 )
```

Shortest path predictor.

Parameters

k	Number of edges to be returned (the actual number may be smaller).
seed	The random number generator seed.

Returns

The top k negative edge scores (the actual size may of the output be smaller than k).

9.90.3.90 predTopSOI()

```
std::vector<EdgeScore> LinkPred::Simp::Predictor::predTopSOI (
            int k )
```

Sorensen index.

Parameters

k	Number of edges to be returned (the actual number may be smaller).

Returns

The top k negative edge scores (the actual size may of the output be smaller than k).

The documentation for this class was generated from the following file:

- include/linkpred/simp/predictor.hpp

9.91 LinkPred::PredResults< TestDataT, LPredictorT, ScoresContainerT > Class Template Reference

A class to store and manage prediction results.

```
#include <predresults.hpp>
```

Classes

- class ScoreIterator

  Score iterator.

Public Types

- using ScoresItT = typename ScoresContainerT::iterator

Public Member Functions

- PredResults (TestDataT testData, std::shared_ptr< LPredictorT > const &predictor)
- PredResults (PredResults const &that)=default
- PredResults & operator= (PredResults const &that)=default
- PredResults (PredResults &&that)=default
- PredResults & operator= (PredResults &&that)=default
- void compPosScores ()
- void compNegScores ()
- void compTopScores (std::size_t l)
- auto posBegin () const
- auto posEnd () const
- auto negBegin () const
- auto negEnd () const
- auto posStrmBegin () const
- auto posStrmEnd () const
- auto negStrmBegin () const
- auto negStrmEnd () const
- auto topBegin () const

- auto topEnd () const
- auto topEdgesBegin () const
- auto topEdgesEnd () const
- bool isNegComputed () const
- SortOrder getNegSortOrder () const
- void sortNeg (SortOrder negSortOrder)
- bool isPosComputed () const
- SortOrder getPosSortOrder () const
- void sortPos (SortOrder posSortOrder)
- bool isTopComputed () const
- const std::shared_ptr< LPredictorT > & getPredictor () const
- TestDataT & getTestData ()
- std::size_t getNbTop () const
- virtual ~PredResults ()=default

9.91.1 Detailed Description

template<typename TestDataT = TestData<>, typename LPredictorT = ULPredictor<>, typename ScoresContainerT = std↵ ::vector<double>>
class LinkPred::PredResults< TestDataT, LPredictorT, ScoresContainerT >

A class to store and manage prediction results.

Template Parameters

TestDataT	The test data type.
LPredictorT	The link predictor type.
ScoresContainerT	The type of container storing scores.

9.91.2 Member Typedef Documentation

9.91.2.1 ScoresItT

```
template<typename TestDataT = TestData<>, typename LPredictorT = ULPredictor<>, typename
ScoresContainerT = std::vector<double>>
using LinkPred::PredResults< TestDataT, LPredictorT, ScoresContainerT >::ScoresItT = typename
ScoresContainerT::iterator
```

Scores iterator type.

9.91.3 Constructor & Destructor Documentation

9.91.3.1 PredResults() [1/3]

```
template<typename TestDataT = TestData<>, typename LPredictorT = ULPredictor<>, typename
ScoresContainerT = std::vector<double>>
LinkPred::PredResults< TestDataT, LPredictorT, ScoresContainerT >::PredResults (
            TestDataT testData,
            std::shared_ptr< LPredictorT > const & predictor )  [inline]
```

Constructor.

Parameters

testData	The test data.
predictor	Link predictor.

9.91.3.2 PredResults() [2/3]

```
template<typename TestDataT = TestData<>, typename LPredictorT = ULPredictor<>, typename
ScoresContainerT = std::vector<double>>
LinkPred::PredResults< TestDataT, LPredictorT, ScoresContainerT >::PredResults (
            PredResults< TestDataT, LPredictorT, ScoresContainerT > const & that )  [default]
```

Copy constructor.

Parameters

that	The object to copy.

9.91.3.3 PredResults() [3/3]

```
template<typename TestDataT = TestData<>, typename LPredictorT = ULPredictor<>, typename
ScoresContainerT = std::vector<double>>
LinkPred::PredResults< TestDataT, LPredictorT, ScoresContainerT >::PredResults (
            PredResults< TestDataT, LPredictorT, ScoresContainerT > && that )  [default]
```

Move constructor.

Parameters

that	The object to move.

9.91.3.4 ∼PredResults()

```
template<typename TestDataT = TestData<>, typename LPredictorT = ULPredictor<>, typename
```

```
ScoresContainerT = std::vector<double>>
virtual LinkPred::PredResults< TestDataT, LPredictorT, ScoresContainerT >::~PredResults ( )
[virtual], [default]
```

Destructor.

9.91.4 Member Function Documentation

9.91.4.1 compNegScores()

```
template<typename TestDataT = TestData<>, typename LPredictorT = ULPredictor<>, typename
ScoresContainerT = std::vector<double>>
void LinkPred::PredResults< TestDataT, LPredictorT, ScoresContainerT >::compNegScores ( )
[inline]
```

Compute scores for negative links.

9.91.4.2 compPosScores()

```
template<typename TestDataT = TestData<>, typename LPredictorT = ULPredictor<>, typename
ScoresContainerT = std::vector<double>>
void LinkPred::PredResults< TestDataT, LPredictorT, ScoresContainerT >::compPosScores ( )
[inline]
```

Compute scores for positive links.

9.91.4.3 compTopScores()

```
template<typename TestDataT = TestData<>, typename LPredictorT = ULPredictor<>, typename
ScoresContainerT = std::vector<double>>
void LinkPred::PredResults< TestDataT, LPredictorT, ScoresContainerT >::compTopScores (
            std::size_t l )  [inline]
```

Compute top scores. This method finds the negative edges with the highest scores.

Parameters

l	Number of top links to compute.

9.91.4.4 getNbTop()

```
template<typename TestDataT = TestData<>, typename LPredictorT = ULPredictor<>, typename
ScoresContainerT = std::vector<double>>
std::size_t LinkPred::PredResults< TestDataT, LPredictorT, ScoresContainerT >::getNbTop ( )
const  [inline]
```

Returns

The number of computed top edges.

9.91.4.5 getNegSortOrder()

```
template<typename TestDataT = TestData<>, typename LPredictorT = ULPredictor<>, typename
ScoresContainerT = std::vector<double>>
SortOrder LinkPred::PredResults< TestDataT, LPredictorT, ScoresContainerT >::getNegSortOrder (
) const [inline]
```

Returns

The sorting order of the negative scores.

9.91.4.6 getPosSortOrder()

```
template<typename TestDataT = TestData<>, typename LPredictorT = ULPredictor<>, typename
ScoresContainerT = std::vector<double>>
SortOrder LinkPred::PredResults< TestDataT, LPredictorT, ScoresContainerT >::getPosSortOrder (
) const [inline]
```

Returns

The sorting order of the positive scores. }

9.91.4.7 getPredictor()

```
template<typename TestDataT = TestData<>, typename LPredictorT = ULPredictor<>, typename
ScoresContainerT = std::vector<double>>
const std::shared_ptr<LPredictorT>& LinkPred::PredResults< TestDataT, LPredictorT, Scores←
ContainerT >::getPredictor ( ) const [inline]
```

Returns

The link predictor.

9.91.4.8 getTestData()

```
template<typename TestDataT = TestData<>, typename LPredictorT = ULPredictor<>, typename
ScoresContainerT = std::vector<double>>
TestDataT& LinkPred::PredResults< TestDataT, LPredictorT, ScoresContainerT >::getTestData ( )
[inline]
```

Returns

The test data.

9.91.4.9 isNegComputed()

```
template<typename TestDataT = TestData<>, typename LPredictorT = ULPredictor<>, typename
ScoresContainerT = std::vector<double>>
bool LinkPred::PredResults< TestDataT, LPredictorT, ScoresContainerT >::isNegComputed ( )
const [inline]
```

Returns

Whether the negative scores have been computed.

9.91.4.10 isPosComputed()

```
template<typename TestDataT = TestData<>, typename LPredictorT = ULPredictor<>, typename
ScoresContainerT = std::vector<double>>
bool LinkPred::PredResults< TestDataT, LPredictorT, ScoresContainerT >::isPosComputed ( )
const [inline]
```

Returns

Whether the positive scores have been computed.

9.91.4.11 isTopComputed()

```
template<typename TestDataT = TestData<>, typename LPredictorT = ULPredictor<>, typename
ScoresContainerT = std::vector<double>>
bool LinkPred::PredResults< TestDataT, LPredictorT, ScoresContainerT >::isTopComputed ( )
const [inline]
```

Returns

Whether the top scores have been computed.

9.91.4.12 negBegin()

```
template<typename TestDataT = TestData<>, typename LPredictorT = ULPredictor<>, typename
ScoresContainerT = std::vector<double>>
auto LinkPred::PredResults< TestDataT, LPredictorT, ScoresContainerT >::negBegin ( ) const
[inline]
```

Returns

Iterator to the first element in the scores of negative links.

9.91.4.13 negEnd()

```
template<typename TestDataT = TestData<>, typename LPredictorT = ULPredictor<>, typename
ScoresContainerT = std::vector<double>>
auto LinkPred::PredResults< TestDataT, LPredictorT, ScoresContainerT >::negEnd ( ) const
[inline]
```

Returns

Iterator to one-past the last element in the scores of negative links.

9.91.4.14 negStrmBegin()

```
template<typename TestDataT = TestData<>, typename LPredictorT = ULPredictor<>, typename
ScoresContainerT = std::vector<double>>
auto LinkPred::PredResults< TestDataT, LPredictorT, ScoresContainerT >::negStrmBegin ( ) const
[inline]
```

Returns

Iterator to the first element in the scores of negative links. The scores here may be streamed and not pre-computed.

9.91.4.15 negStrmEnd()

```
template<typename TestDataT = TestData<>, typename LPredictorT = ULPredictor<>, typename
ScoresContainerT = std::vector<double>>
auto LinkPred::PredResults< TestDataT, LPredictorT, ScoresContainerT >::negStrmEnd ( ) const
[inline]
```

Returns

Iterator to one-past the last element in the scores of negative links. The scores here may be streamed and not pre-computed.

9.91.4.16 operator=() [1/2]

```
template<typename TestDataT = TestData<>, typename LPredictorT = ULPredictor<>, typename
ScoresContainerT = std::vector<double>>
PredResults& LinkPred::PredResults< TestDataT, LPredictorT, ScoresContainerT >::operator= (
            PredResults< TestDataT, LPredictorT, ScoresContainerT > && that )  [default]
```

Move assignment operator.

Parameters

that	The object to move.

9.91.4.17 operator=() [2/2]

```
template<typename TestDataT = TestData<>, typename LPredictorT = ULPredictor<>, typename
ScoresContainerT = std::vector<double>>
PredResults& LinkPred::PredResults< TestDataT, LPredictorT, ScoresContainerT >::operator= (
            PredResults< TestDataT, LPredictorT, ScoresContainerT > const & that )  [default]
```

Copy assignment operator.

Parameters

that	The object to copy.

9.91.4.18 posBegin()

```
template<typename TestDataT = TestData<>, typename LPredictorT = ULPredictor<>, typename
ScoresContainerT = std::vector<double>>
auto LinkPred::PredResults< TestDataT, LPredictorT, ScoresContainerT >::posBegin ( ) const
[inline]
```

Returns

Iterator to the first element in the scores of positive links.

9.91.4.19 posEnd()

```
template<typename TestDataT = TestData<>, typename LPredictorT = ULPredictor<>, typename
ScoresContainerT = std::vector<double>>
auto LinkPred::PredResults< TestDataT, LPredictorT, ScoresContainerT >::posEnd ( ) const
[inline]
```

Returns

Iterator to one-past the last element in the scores of positive links.

9.91.4.20 posStrmBegin()

```
template<typename TestDataT = TestData<>, typename LPredictorT = ULPredictor<>, typename
ScoresContainerT = std::vector<double>>
auto LinkPred::PredResults< TestDataT, LPredictorT, ScoresContainerT >::posStrmBegin ( ) const
[inline]
```

Returns

Iterator to the first element in the scores of positive links. The scores here may be streamed and not pre-computed.

9.91.4.21 posStrmEnd()

```
template<typename TestDataT = TestData<>, typename LPredictorT = ULPredictor<>, typename
ScoresContainerT = std::vector<double>>
auto LinkPred::PredResults< TestDataT, LPredictorT, ScoresContainerT >::posStrmEnd ( ) const
[inline]
```

Returns

Iterator to one-past the last element in the scores of positive links. The scores here may be streamed and not pre-computed.

9.91.4.22 sortNeg()

```
template<typename TestDataT = TestData<>, typename LPredictorT = ULPredictor<>, typename
ScoresContainerT = std::vector<double>>
void LinkPred::PredResults< TestDataT, LPredictorT, ScoresContainerT >::sortNeg (
            SortOrder negSortOrder )  [inline]
```

Sort the negative scores.

Parameters

negSortOrder	The request sorting order.

9.91.4.23 sortPos()

```
template<typename TestDataT = TestData<>, typename LPredictorT = ULPredictor<>, typename
ScoresContainerT = std::vector<double>>
void LinkPred::PredResults< TestDataT, LPredictorT, ScoresContainerT >::sortPos (
            SortOrder posSortOrder )  [inline]
```

Sort the positive scores.

Parameters

posSortOrder	The request sorting order.

9.91.4.24 topBegin()

```
template<typename TestDataT = TestData<>, typename LPredictorT = ULPredictor<>, typename
ScoresContainerT = std::vector<double>>
auto LinkPred::PredResults< TestDataT, LPredictorT, ScoresContainerT >::topBegin ( ) const
[inline]
```

Returns

Iterator to the first element in the top scores.

9.91.4.25 topEdgesBegin()

```
template<typename TestDataT = TestData<>, typename LPredictorT = ULPredictor<>, typename
ScoresContainerT = std::vector<double>>
auto LinkPred::PredResults< TestDataT, LPredictorT, ScoresContainerT >::topEdgesBegin ( )
const [inline]
```

Returns

Iterator to the first element in the top edges.

9.91.4.26 topEdgesEnd()

```
template<typename TestDataT = TestData<>, typename LPredictorT = ULPredictor<>, typename
ScoresContainerT = std::vector<double>>
auto LinkPred::PredResults< TestDataT, LPredictorT, ScoresContainerT >::topEdgesEnd ( ) const
[inline]
```

Returns

Iterator to one-past the last element in the top edges.

9.91.4.27 topEnd()

```
template<typename TestDataT = TestData<>, typename LPredictorT = ULPredictor<>, typename
ScoresContainerT = std::vector<double>>
auto LinkPred::PredResults< TestDataT, LPredictorT, ScoresContainerT >::topEnd ( ) const
[inline]
```

Returns

Iterator to one-past the last element in the top scores.

The documentation for this class was generated from the following file:

- include/linkpred/perf/predresults.hpp

9.92 LinkPred::Simp::Evaluator::Factory::PSTParams Struct Reference

Parameters of PST.

```
#include <evaluator.hpp>
```

Public Attributes

- std::string fileName

9.92.1 Detailed Description

Parameters of PST.

9.92.2 Member Data Documentation

9.92.2.1 fileName

```
std::string LinkPred::Simp::Evaluator::Factory::PSTParams::fileName
```

File containing the scores of all non-exisityng links in the training network.

The documentation for this struct was generated from the following file:

- include/linkpred/simp/evaluator.hpp

9.93 LinkPred::RandomGen Class Reference

A random number generator.

```
#include <randomgen.hpp>
```

Public Member Functions

- RandomGen ()
- RandomGen (long int seed)
- RandomGen (RandomGen const &that)=default
- RandomGen & operator= (RandomGen const &that)=default
- RandomGen (RandomGen &&that)=default
- RandomGen & operator= (RandomGen &&that)=default
- auto getSeed ()
- auto getInt ()
- std::size_t getUInt (std::size_t low, std::size_t high)
- int getSInt (int low, int high)
- double getDouble (double low, double high)
- bool getBool ()
- double getPL (double minV, double maxV, double gamma)
- unsigned long int getGeo (double p)
- virtual ∼RandomGen ()=default

9.93.1 Detailed Description

A random number generator.

This is mainly a wrapper that simplifies access to C++11 random generating classes/methods.

9.93.2 Constructor & Destructor Documentation

9.93.2.1 RandomGen() [1/4]

```
LinkPred::RandomGen::RandomGen ( ) [inline]
```

Constructor.

9.93.2.2 RandomGen() [2/4]

```
LinkPred::RandomGen::RandomGen (
            long int seed ) [inline]
```

Constructor with seed.

Parameters

seed	The seed.

9.93.2.3 RandomGen() [3/4]

```
LinkPred::RandomGen::RandomGen (
            RandomGen const & that ) [default]
```

Copy constructor.

Parameters

that	The object to copy.

9.93.2.4 RandomGen() [4/4]

```
LinkPred::RandomGen::RandomGen (
            RandomGen && that ) [default]
```

Move constructor.

Parameters

that	The object to move.

9.93.2.5 ∼RandomGen()

```
virtual LinkPred::RandomGen::∼RandomGen ( ) [virtual], [default]
```

Destructor.

9.93.3 Member Function Documentation

9.93.3.1 getBool()

```
bool LinkPred::RandomGen::getBool ( ) [inline]
```

Returns

A uniformly distributed boolean (a fair coin toss).

9.93.3.2 getDouble()

```
double LinkPred::RandomGen::getDouble (
            double low,
            double high )  [inline]
```

Generate a uniformly distributed double in the specified interval.

Parameters

low	The left limit of the interval.
high	The right limit of the interval.

Returns

A double uniformly distributed in the interval [low, high).

9.93.3.3 getGeo()

```
unsigned long int LinkPred::RandomGen::getGeo (
            double p )  [inline]
```

Parameters

p	The probability of success of the associated Bernouli distribution.

Returns

A sample from a geometric distribution.

9.93.3.4 getInt()

```
auto LinkPred::RandomGen::getInt ( )  [inline]
```

Returns

A random integer.

9.93.3.5 getPL()

```
double LinkPred::RandomGen::getPL (
            double minV,
            double maxV,
            double gamma )  [inline]
```

Returns

Sample from a power law distribution.

Parameters

minV	The minimum value.
maxV	The maximum value.
gamma	The exponent of the power law.

9.93.3.6 getSeed()

```
auto LinkPred::RandomGen::getSeed ( )  [inline]
```

Returns

The seed.

9.93.3.7 getSInt()

```
int LinkPred::RandomGen::getSInt (
            int low,
            int high )  [inline]
```

Returns

A random signed integer in the interval [low, high] (inclusive of the boundaries).

Parameters

low	The lower bound of the interval.
high	The upper bound of the interval.

9.93.3.8 getUInt()

```
std::size_t LinkPred::RandomGen::getUInt (
            std::size_t low,
            std::size_t high )  [inline]
```

Returns

A random unsigned integer in the interval [low, high] (inclusive of the boundaries).

Parameters

low	The lower bound of the interval.
high	The upper bound of the interval.

9.93.3.9 operator=() [1/2]

```
RandomGen& LinkPred::RandomGen::operator= (
            RandomGen && that )  [default]
```

Move assignment operator.

Parameters

that	The object to move.

9.93.3.10 operator=() [2/2]

```
RandomGen& LinkPred::RandomGen::operator= (
            RandomGen const & that )  [default]
```

Copy assignment operator.

Parameters

that	The object to copy.

The documentation for this class was generated from the following file:

- include/linkpred/utils/randomgen.hpp

9.94 LinkPred::RndClassifier< InRndIt, OutRndIt, ScoreRndIt > Class Template Reference

Random classifier.

`#include <rndclassifier.hpp>`

Inheritance diagram for LinkPred::RndClassifier< InRndIt, OutRndIt, ScoreRndIt >:

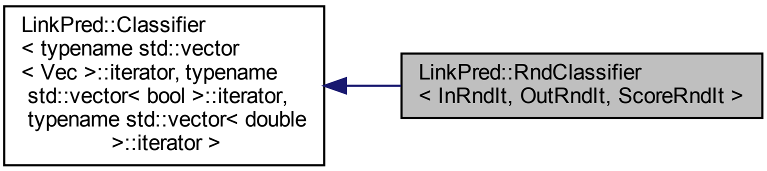

Collaboration diagram for LinkPred::RndClassifier< InRndIt, OutRndIt, ScoreRndIt >:

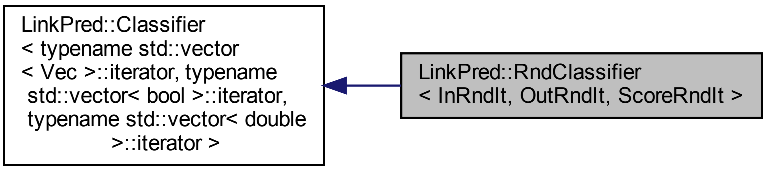

Public Member Functions

- RndClassifier (long int seed)
- RndClassifier (RndClassifier const &that)=default
- RndClassifier & operator= (RndClassifier const &that)=default
- RndClassifier (RndClassifier &&that)=default
- RndClassifier & operator= (RndClassifier &&that)=default
- virtual void learn (InRndIt trInBegin, InRndIt trInEnd, OutRndIt trOutBegin, OutRndIt trOutEnd)
- virtual void predict (InRndIt inBegin, InRndIt inEnd, ScoreRndIt scoresBegin)
- virtual ~RndClassifier ()=default

9.94.1 Detailed Description

template<typename InRndIt = typename std::vector<Vec>::iterator, typename OutRndIt = typename std::vector<bool>↩ ::iterator, typename ScoreRndIt = typename std::vector<double>::iterator>
class LinkPred::RndClassifier< InRndIt, OutRndIt, ScoreRndIt >

Random classifier.

Template Parameters

InRndIt	Input (features) iterator type.
OutRndIt	Output (class) iterator type.
Score↩ RndIt	Classification scores iterator type.

9.94.2 Constructor & Destructor Documentation

9.94.2.1 RndClassifier() [1/3]

```
template<typename InRndIt = typename std::vector<Vec>::iterator, typename OutRndIt = typename
std::vector<bool>::iterator, typename ScoreRndIt = typename std::vector<double>::iterator>
LinkPred::RndClassifier< InRndIt, OutRndIt, ScoreRndIt >::RndClassifier (
            long int seed )  [inline]
```

Constructor.

Parameters

seed	The random number generator's seed.

9.94.2.2 RndClassifier() [2/3]

```
template<typename InRndIt = typename std::vector<Vec>::iterator, typename OutRndIt = typename
std::vector<bool>::iterator, typename ScoreRndIt = typename std::vector<double>::iterator>
LinkPred::RndClassifier< InRndIt, OutRndIt, ScoreRndIt >::RndClassifier (
            RndClassifier< InRndIt, OutRndIt, ScoreRndIt > const & that )  [default]
```

Copy constructor.

Parameters

that	The object to copy.

9.94.2.3 RndClassifier() [3/3]

```
template<typename InRndIt = typename std::vector<Vec>::iterator, typename OutRndIt = typename
std::vector<bool>::iterator, typename ScoreRndIt = typename std::vector<double>::iterator>
```

```
LinkPred::RndClassifier< InRndIt, OutRndIt, ScoreRndIt >::RndClassifier (
            RndClassifier< InRndIt, OutRndIt, ScoreRndIt > && that )  [default]
```

Move constructor.

Parameters

that	The object to move.

9.94.2.4 ∼RndClassifier()

```
template<typename InRndIt = typename std::vector<Vec>::iterator, typename OutRndIt = typename
std::vector<bool>::iterator, typename ScoreRndIt = typename std::vector<double>::iterator>
virtual LinkPred::RndClassifier< InRndIt, OutRndIt, ScoreRndIt >::~RndClassifier ( )  [virtual],
[default]
```

Destructor.

9.94.3 Member Function Documentation

9.94.3.1 learn()

```
template<typename InRndIt = typename std::vector<Vec>::iterator, typename OutRndIt = typename
std::vector<bool>::iterator, typename ScoreRndIt = typename std::vector<double>::iterator>
virtual void LinkPred::RndClassifier< InRndIt, OutRndIt, ScoreRndIt >::learn (
            InRndIt trInBegin,
            InRndIt trInEnd,
            OutRndIt trOutBegin,
            OutRndIt trOutEnd )  [virtual]
```

Learn from data.

Parameters

trInBegin	Iterator to the first example features (input).
trInEnd	Iterator to one-past-the-last example features (input).
trOutBegin	Iterator to the first example class (output).
trOutEnd	Iterator to one-past-the-last example class (output).

9.94.3.2 operator=() [1/2]

```
template<typename InRndIt = typename std::vector<Vec>::iterator, typename OutRndIt = typename
std::vector<bool>::iterator, typename ScoreRndIt = typename std::vector<double>::iterator>
RndClassifier& LinkPred::RndClassifier< InRndIt, OutRndIt, ScoreRndIt >::operator= (
            RndClassifier< InRndIt, OutRndIt, ScoreRndIt > && that )  [default]
```

Move assignment operator.

Parameters

that	The object to move.

9.94.3.3 operator=() [2/2]

```
template<typename InRndIt = typename std::vector<Vec>::iterator, typename OutRndIt = typename
std::vector<bool>::iterator, typename ScoreRndIt = typename std::vector<double>::iterator>
RndClassifier& LinkPred::RndClassifier< InRndIt, OutRndIt, ScoreRndIt >::operator= (
            RndClassifier< InRndIt, OutRndIt, ScoreRndIt > const & that )  [default]
```

Copy assignment operator.

Parameters

that	The object to copy.

9.94.3.4 predict()

```
template<typename InRndIt = typename std::vector<Vec>::iterator, typename OutRndIt = typename
std::vector<bool>::iterator, typename ScoreRndIt = typename std::vector<double>::iterator>
virtual void LinkPred::RndClassifier< InRndIt, OutRndIt, ScoreRndIt >::predict (
            InRndIt inBegin,
            InRndIt inEnd,
            ScoreRndIt scoresBegin )  [virtual]
```

Predict.

Parameters

inBegin	Iterator to the first instance features (input).
inEnd	Iterator to one-past-the-last instance features (input).
scoresBegin	Iterator to the first location where to store prediction scores.

The documentation for this class was generated from the following file:

- include/linkpred/ml/classifiers/rndclassifier/rndclassifier.hpp

9.95 LinkPred::UNetwork< LabelT, NodeIDT, EdgeT >::RndEdgeIt Class Reference

Randomized edges iterator.

```
#include <unetwork.hpp>
```

Inheritance diagram for LinkPred::UNetwork< LabelT, NodeIDT, EdgeT >::RndEdgeIt:

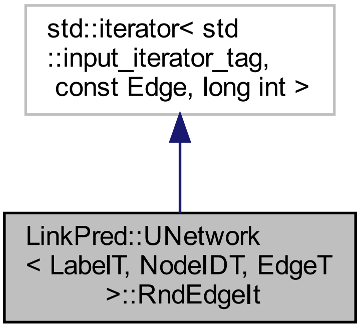

Collaboration diagram for LinkPred::UNetwork< LabelT, NodeIDT, EdgeT >::RndEdgeIt:

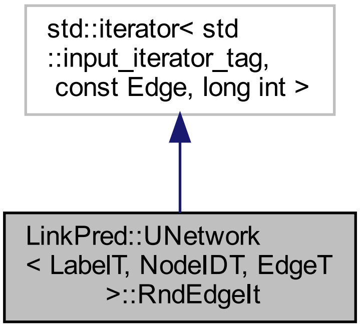

Public Types

- using pointer = typename std::iterator< std::input_iterator_tag, const Edge, long int >::pointer
- using reference = typename std::iterator< std::input_iterator_tag, const Edge, long int >::reference

Public Member Functions

- RndEdgeIt (RndEdgeIt const &that)=default
- RndEdgeIt & operator= (RndEdgeIt const &that)=default
- RndEdgeIt (RndEdgeIt &&that)=default
- RndEdgeIt & operator= (RndEdgeIt &&that)=default

- reference operator* () const
- pointer operator-> () const
- RndEdgeIt & operator++ ()
- RndEdgeIt operator++ (int)
- bool operator== (const RndEdgeIt &that) const
- bool operator!= (const RndEdgeIt &that) const

Friends

- class **UNetwork**

9.95.1 Detailed Description

template<typename LabelT = std::string, typename NodeIDT = unsigned int, typename EdgeT = unsigned long long int>
class LinkPred::UNetwork< LabelT, NodeIDT, EdgeT >::RndEdgeIt

Randomized edges iterator.

This is forward iterator that can be used to randomly sample a subset of the edges.

9.95.2 Member Typedef Documentation

9.95.2.1 pointer

```
template<typename LabelT = std::string, typename NodeIDT = unsigned int, typename EdgeT =
unsigned long long int>
using LinkPred::UNetwork< LabelT, NodeIDT, EdgeT >::RndEdgeIt::pointer = typename std::iterator<std↩
::input_iterator_tag, const Edge, long int>::pointer
```

The pointer type associated with the iterator.

9.95.2.2 reference

```
template<typename LabelT = std::string, typename NodeIDT = unsigned int, typename EdgeT =
unsigned long long int>
using LinkPred::UNetwork< LabelT, NodeIDT, EdgeT >::RndEdgeIt::reference = typename std↩
::iterator<std::input_iterator_tag, const Edge, long int>::reference
```

The reference type associated with the iterator.

9.95.3 Constructor & Destructor Documentation

9.95.3.1 RndEdgeIt() [1/2]

```
template<typename LabelT = std::string, typename NodeIDT = unsigned int, typename EdgeT =
unsigned long long int>
LinkPred::UNetwork< LabelT, NodeIDT, EdgeT >::RndEdgeIt::RndEdgeIt (
            RndEdgeIt const & that )  [default]
```

Copy constructor.

Parameters

that	The object to copy.

9.95.3.2 RndEdgeIt() [2/2]

```
template<typename LabelT = std::string, typename NodeIDT = unsigned int, typename EdgeT =
unsigned long long int>
LinkPred::UNetwork< LabelT, NodeIDT, EdgeT >::RndEdgeIt::RndEdgeIt (
             RndEdgeIt && that )  [default]
```

Move constructor.

Parameters

that	The object to move.

9.95.4 Member Function Documentation

9.95.4.1 operator"!=()

```
template<typename LabelT = std::string, typename NodeIDT = unsigned int, typename EdgeT =
unsigned long long int>
bool LinkPred::UNetwork< LabelT, NodeIDT, EdgeT >::RndEdgeIt::operator!= (
             const RndEdgeIt & that ) const  [inline]
```

Parameters

that	The other iterator.

Returns

True if this is not equal to that.

9.95.4.2 operator*()

```
template<typename LabelT = std::string, typename NodeIDT = unsigned int, typename EdgeT =
unsigned long long int>
reference LinkPred::UNetwork< LabelT, NodeIDT, EdgeT >::RndEdgeIt::operator* ( ) const  [inline]
```

Dereference operator.

Returns

A reference to the object to which the iterator points.

9.95.4.3 operator++() [1/2]

```
template<typename LabelT = std::string, typename NodeIDT = unsigned int, typename EdgeT =
unsigned long long int>
RndEdgeIt& LinkPred::UNetwork< LabelT, NodeIDT, EdgeT >::RndEdgeIt::operator++ ( ) [inline]
```

Pre-increment operator.

Returns

A reference to the new iterator.

9.95.4.4 operator++() [2/2]

```
template<typename LabelT = std::string, typename NodeIDT = unsigned int, typename EdgeT =
unsigned long long int>
RndEdgeIt LinkPred::UNetwork< LabelT, NodeIDT, EdgeT >::RndEdgeIt::operator++ (
             int ) [inline]
```

Post-increment operator.

Returns

A reference to the new iterator.

9.95.4.5 operator->()

```
template<typename LabelT = std::string, typename NodeIDT = unsigned int, typename EdgeT =
unsigned long long int>
pointer LinkPred::UNetwork< LabelT, NodeIDT, EdgeT >::RndEdgeIt::operator-> ( ) const [inline]
```

Arrow operator.

Returns

A pointer to the object to which the iterator points.

9.95.4.6 operator=() [1/2]

```
template<typename LabelT = std::string, typename NodeIDT = unsigned int, typename EdgeT =
unsigned long long int>
RndEdgeIt& LinkPred::UNetwork< LabelT, NodeIDT, EdgeT >::RndEdgeIt::operator= (
             RndEdgeIt && that ) [default]
```

Move assignment operator.

Parameters

that	The object to move.

9.95.4.7 operator=() [2/2]

```
template<typename LabelT = std::string, typename NodeIDT = unsigned int, typename EdgeT =
unsigned long long int>
RndEdgeIt& LinkPred::UNetwork< LabelT, NodeIDT, EdgeT >::RndEdgeIt::operator= (
            RndEdgeIt const & that )  [default]
```

Copy assignment operator.

Parameters

that	The object to copy.

9.95.4.8 operator==()

```
template<typename LabelT = std::string, typename NodeIDT = unsigned int, typename EdgeT =
unsigned long long int>
bool LinkPred::UNetwork< LabelT, NodeIDT, EdgeT >::RndEdgeIt::operator== (
            const RndEdgeIt & that ) const  [inline]
```

Parameters

that	The other iterator.

Returns

True if this equals that.

The documentation for this class was generated from the following file:

- include/linkpred/core/unetwork/unetwork.hpp

9.96 LinkPred::DNetwork< LabelT, NodeIDT, EdgeT >::RndEdgeIt Class Reference

Randomized edges iterator.

```
#include <dnetwork.hpp>
```

Inheritance diagram for LinkPred::DNetwork< LabelT, NodeIDT, EdgeT >::RndEdgeIt:

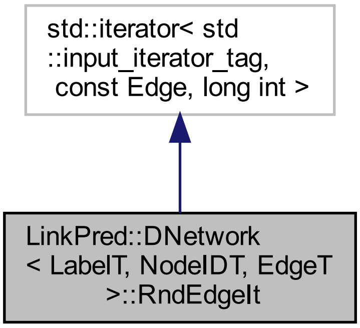

Collaboration diagram for LinkPred::DNetwork< LabelT, NodeIDT, EdgeT >::RndEdgeIt:

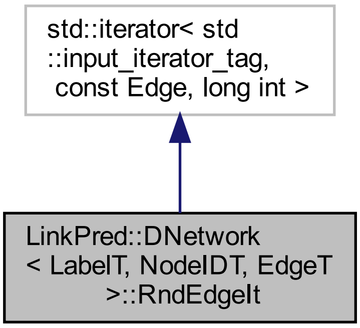

Public Types

- using pointer = typename std::iterator< std::input_iterator_tag, const Edge, long int >::pointer
- using reference = typename std::iterator< std::input_iterator_tag, const Edge, long int >::reference

Public Member Functions

- RndEdgeIt (RndEdgeIt const &that)=default
- RndEdgeIt & operator= (RndEdgeIt const &that)=default
- RndEdgeIt (RndEdgeIt &&that)=default
- RndEdgeIt & operator= (RndEdgeIt &&that)=default
- reference operator* () const
- pointer operator-> () const
- RndEdgeIt & operator++ ()
- RndEdgeIt operator++ (int)
- bool operator== (const RndEdgeIt &that) const
- bool operator!= (const RndEdgeIt &that) const

Friends

- class **DNetwork**

9.96.1 Detailed Description

**template<typename LabelT = std::string, typename NodeIDT = unsigned int, typename EdgeT = unsigned long long int>
class LinkPred::DNetwork< LabelT, NodeIDT, EdgeT >::RndEdgeIt**

Randomized edges iterator.

This is forward iterator that can be used to randomly sample a subset of the edges.

9.96.2 Member Typedef Documentation

9.96.2.1 pointer

```
template<typename LabelT = std::string, typename NodeIDT = unsigned int, typename EdgeT =
unsigned long long int>
using LinkPred::DNetwork< LabelT, NodeIDT, EdgeT >::RndEdgeIt::pointer = typename std::iterator<std↩
::input_iterator_tag, const Edge, long int>::pointer
```

The pointer type associated with the iterator.

9.96.2.2 reference

```
template<typename LabelT = std::string, typename NodeIDT = unsigned int, typename EdgeT =
unsigned long long int>
using LinkPred::DNetwork< LabelT, NodeIDT, EdgeT >::RndEdgeIt::reference = typename std↩
::iterator<std::input_iterator_tag, const Edge, long int>::reference
```

The reference type associated with the iterator.

9.96.3 Constructor & Destructor Documentation

9.96.3.1 RndEdgeIt() [1/2]

```
template<typename LabelT = std::string, typename NodeIDT = unsigned int, typename EdgeT =
unsigned long long int>
LinkPred::DNetwork< LabelT, NodeIDT, EdgeT >::RndEdgeIt::RndEdgeIt (
            RndEdgeIt const & that )  [default]
```

Copy constructor.

Parameters

that	The object to copy.

9.96.3.2 RndEdgeIt() [2/2]

```
template<typename LabelT = std::string, typename NodeIDT = unsigned int, typename EdgeT =
unsigned long long int>
LinkPred::DNetwork< LabelT, NodeIDT, EdgeT >::RndEdgeIt::RndEdgeIt (
            RndEdgeIt && that )  [default]
```

Move constructor.

Parameters

that	The object to move.

9.96.4 Member Function Documentation

9.96.4.1 operator"!=()

```
template<typename LabelT = std::string, typename NodeIDT = unsigned int, typename EdgeT =
unsigned long long int>
bool LinkPred::DNetwork< LabelT, NodeIDT, EdgeT >::RndEdgeIt::operator!= (
            const RndEdgeIt & that ) const  [inline]
```

Parameters

that	The other iterator.

Returns

True if this is not equal to that.

9.96.4.2 operator*()

```
template<typename LabelT = std::string, typename NodeIDT = unsigned int, typename EdgeT =
unsigned long long int>
reference LinkPred::DNetwork< LabelT, NodeIDT, EdgeT >::RndEdgeIt::operator* ( ) const  [inline]
```

Dereference operator.

Returns

A reference to the object to which the iterator points.

9.96.4.3 operator++() [1/2]

```
template<typename LabelT = std::string, typename NodeIDT = unsigned int, typename EdgeT =
unsigned long long int>
RndEdgeIt& LinkPred::DNetwork< LabelT, NodeIDT, EdgeT >::RndEdgeIt::operator++ ( )  [inline]
```

Pre-increment operator.

Returns

A reference to the new iterator.

9.96.4.4 operator++() [2/2]

```
template<typename LabelT = std::string, typename NodeIDT = unsigned int, typename EdgeT =
unsigned long long int>
RndEdgeIt LinkPred::DNetwork< LabelT, NodeIDT, EdgeT >::RndEdgeIt::operator++ (
             int  )  [inline]
```

Post-increment operator.

Returns

A reference to the new iterator.

9.96.4.5 operator->()

```
template<typename LabelT = std::string, typename NodeIDT = unsigned int, typename EdgeT =
unsigned long long int>
pointer LinkPred::DNetwork< LabelT, NodeIDT, EdgeT >::RndEdgeIt::operator-> ( ) const  [inline]
```

Arrow operator.

Returns

A pointer to the object to which the iterator points.

9.96.4.6 operator=() [1/2]

```
template<typename LabelT = std::string, typename NodeIDT = unsigned int, typename EdgeT =
unsigned long long int>
RndEdgeIt& LinkPred::DNetwork< LabelT, NodeIDT, EdgeT >::RndEdgeIt::operator= (
             RndEdgeIt && that )  [default]
```

Move assignment operator.

Parameters

that	The object to move.

9.96.4.7 operator=() [2/2]

```
template<typename LabelT = std::string, typename NodeIDT = unsigned int, typename EdgeT =
unsigned long long int>
RndEdgeIt& LinkPred::DNetwork< LabelT, NodeIDT, EdgeT >::RndEdgeIt::operator= (
            RndEdgeIt const & that )  [default]
```

Copy assignment operator.

Parameters

that	The object to copy.

9.96.4.8 operator==()

```
template<typename LabelT = std::string, typename NodeIDT = unsigned int, typename EdgeT =
unsigned long long int>
bool LinkPred::DNetwork< LabelT, NodeIDT, EdgeT >::RndEdgeIt::operator== (
            const RndEdgeIt & that ) const  [inline]
```

Parameters

that	The other iterator.

Returns

True if this equals that.

The documentation for this class was generated from the following file:

- include/linkpred/core/dnetwork/dnetwork.hpp

9.97 LinkPred::DNetwork< LabelT, NodeIDT, EdgeT >::RndNodeIt Class Reference

Randomized Nodes iterator.

```
#include <dnetwork.hpp>
```

Inheritance diagram for LinkPred::DNetwork< LabelT, NodeIDT, EdgeT >::RndNodeIt:

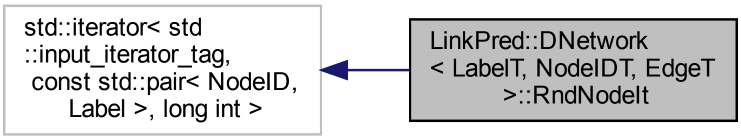

Collaboration diagram for LinkPred::DNetwork< LabelT, NodeIDT, EdgeT >::RndNodeIt:

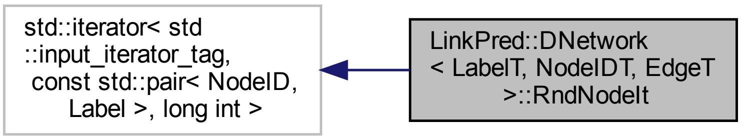

Public Types

- using pointer = typename std::iterator< std::input_iterator_tag, const std::pair< NodeID, Label >, long int >::pointer
- using reference = typename std::iterator< std::input_iterator_tag, const std::pair< NodeID, Label >, long int >::reference

Public Member Functions

- RndNodeIt (RndNodeIt const &that)=default
- RndNodeIt & operator= (RndNodeIt const &that)=default
- RndNodeIt (RndNodeIt &&that)=default
- RndNodeIt & operator= (RndNodeIt &&that)=default
- reference operator* () const
- pointer operator-> () const
- RndNodeIt & operator++ ()
- RndNodeIt operator++ (int)
- bool operator== (const RndNodeIt &that) const
- bool operator!= (const RndNodeIt &that) const

Friends

- class **DNetwork**

9.97.1 Detailed Description

**template<typename LabelT = std::string, typename NodeIDT = unsigned int, typename EdgeT = unsigned long long int>
class LinkPred::DNetwork< LabelT, NodeIDT, EdgeT >::RndNodeIt**

Randomized Nodes iterator.

This is forward iterator that can be used to randomly sample a subset of the nodes.

9.97.2 Member Typedef Documentation

9.97.2.1 pointer

```
template<typename LabelT = std::string, typename NodeIDT = unsigned int, typename EdgeT =
unsigned long long int>
using LinkPred::DNetwork< LabelT, NodeIDT, EdgeT >::RndNodeIt::pointer = typename std::iterator<std::input_iterator_tag, const std::pair<NodeID, Label>, long int>::pointer
```

The pointer type associated with the iterator.

9.97.2.2 reference

```
template<typename LabelT = std::string, typename NodeIDT = unsigned int, typename EdgeT =
unsigned long long int>
using LinkPred::DNetwork< LabelT, NodeIDT, EdgeT >::RndNodeIt::reference = typename std::iterator<std::input_iterator_tag, const std::pair<NodeID, Label>, long int>::reference
```

The reference type associated with the iterator.

9.97.3 Constructor & Destructor Documentation

9.97.3.1 RndNodeIt() [1/2]

```
template<typename LabelT = std::string, typename NodeIDT = unsigned int, typename EdgeT =
unsigned long long int>
LinkPred::DNetwork< LabelT, NodeIDT, EdgeT >::RndNodeIt::RndNodeIt (
            RndNodeIt const & that )  [default]
```

Copy constructor.

Parameters

that	The object to copy.

9.97.3.2 RndNodeIt() [2/2]

```
template<typename LabelT = std::string, typename NodeIDT = unsigned int, typename EdgeT =
unsigned long long int>
LinkPred::DNetwork< LabelT, NodeIDT, EdgeT >::RndNodeIt::RndNodeIt (
            RndNodeIt && that ) [default]
```

Move constructor.

Parameters

that	The object to move.

9.97.4 Member Function Documentation

9.97.4.1 operator"!=()

```
template<typename LabelT = std::string, typename NodeIDT = unsigned int, typename EdgeT =
unsigned long long int>
bool LinkPred::DNetwork< LabelT, NodeIDT, EdgeT >::RndNodeIt::operator!= (
            const RndNodeIt & that ) const [inline]
```

Parameters

that	The other iterator.

Returns

True if this is not equal to that.

9.97.4.2 operator∗()

```
template<typename LabelT = std::string, typename NodeIDT = unsigned int, typename EdgeT =
unsigned long long int>
reference LinkPred::DNetwork< LabelT, NodeIDT, EdgeT >::RndNodeIt::operator∗ ( ) const [inline]
```

Dereference operator.

Returns

A reference to the object to which the iterator points.

9.97.4.3 operator++() [1/2]

```
template<typename LabelT = std::string, typename NodeIDT = unsigned int, typename EdgeT =
unsigned long long int>
RndNodeIt& LinkPred::DNetwork< LabelT, NodeIDT, EdgeT >::RndNodeIt::operator++ ( )  [inline]
```

Pre-increment operator.

Returns

A reference to the new iterator.

9.97.4.4 operator++() [2/2]

```
template<typename LabelT = std::string, typename NodeIDT = unsigned int, typename EdgeT =
unsigned long long int>
RndNodeIt LinkPred::DNetwork< LabelT, NodeIDT, EdgeT >::RndNodeIt::operator++ (
            int  )  [inline]
```

Post-increment operator.

Returns

A reference to the new iterator.

9.97.4.5 operator->()

```
template<typename LabelT = std::string, typename NodeIDT = unsigned int, typename EdgeT =
unsigned long long int>
pointer LinkPred::DNetwork< LabelT, NodeIDT, EdgeT >::RndNodeIt::operator-> ( ) const  [inline]
```

Arrow operator.

Returns

A pointer to the object to which the iterator points.

9.97.4.6 operator=() [1/2]

```
template<typename LabelT = std::string, typename NodeIDT = unsigned int, typename EdgeT =
unsigned long long int>
RndNodeIt& LinkPred::DNetwork< LabelT, NodeIDT, EdgeT >::RndNodeIt::operator= (
            RndNodeIt && that )  [default]
```

Move assignment operator.

Parameters

that	The object to move.

9.97.4.7 operator=() [2/2]

```
template<typename LabelT = std::string, typename NodeIDT = unsigned int, typename EdgeT =
unsigned long long int>
RndNodeIt& LinkPred::DNetwork< LabelT, NodeIDT, EdgeT >::RndNodeIt::operator= (
            RndNodeIt const & that )  [default]
```

Copy assignment operator.

Parameters

that	The object to copy.

9.97.4.8 operator==()

```
template<typename LabelT = std::string, typename NodeIDT = unsigned int, typename EdgeT =
unsigned long long int>
bool LinkPred::DNetwork< LabelT, NodeIDT, EdgeT >::RndNodeIt::operator== (
            const RndNodeIt & that ) const  [inline]
```

Parameters

that	The other iterator.

Returns

True if this equals that.

The documentation for this class was generated from the following file:

- include/linkpred/core/dnetwork/dnetwork.hpp

9.98 LinkPred::UNetwork< LabelT, NodeIDT, EdgeT >::RndNodeIt Class Reference

Randomized Nodes iterator.

```
#include <unetwork.hpp>
```

Inheritance diagram for LinkPred::UNetwork< LabelT, NodeIDT, EdgeT >::RndNodeIt:

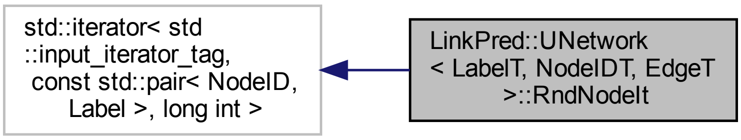

Collaboration diagram for LinkPred::UNetwork< LabelT, NodeIDT, EdgeT >::RndNodeIt:

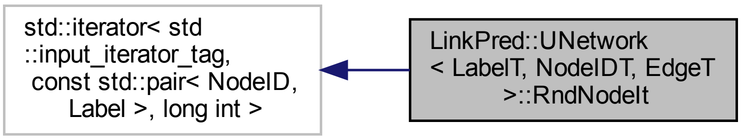

Public Types

- using pointer = typename std::iterator< std::input_iterator_tag, const std::pair< NodeID, Label >, long int >::pointer
- using reference = typename std::iterator< std::input_iterator_tag, const std::pair< NodeID, Label >, long int >::reference

Public Member Functions

- RndNodeIt (RndNodeIt const &that)=default
- RndNodeIt & operator= (RndNodeIt const &that)=default
- RndNodeIt (RndNodeIt &&that)=default
- RndNodeIt & operator= (RndNodeIt &&that)=default
- reference operator* () const
- pointer operator-> () const
- RndNodeIt & operator++ ()
- RndNodeIt operator++ (int)
- bool operator== (const RndNodeIt &that) const
- bool operator!= (const RndNodeIt &that) const

Friends

- class **UNetwork**

9.98.1 Detailed Description

**template<typename LabelT = std::string, typename NodeIDT = unsigned int, typename EdgeT = unsigned long long int>
class LinkPred::UNetwork< LabelT, NodeIDT, EdgeT >::RndNodeIt**

Randomized Nodes iterator.

This is forward iterator that can be used to randomly sample a subset of the nodes.

9.98.2 Member Typedef Documentation

9.98.2.1 pointer

```
template<typename LabelT = std::string, typename NodeIDT = unsigned int, typename EdgeT =
unsigned long long int>
using LinkPred::UNetwork< LabelT, NodeIDT, EdgeT >::RndNodeIt::pointer = typename std::iterator<std↩
::input_iterator_tag, const std::pair<NodeID, Label>, long int>::pointer
```

The pointer type associated with the iterator.

9.98.2.2 reference

```
template<typename LabelT = std::string, typename NodeIDT = unsigned int, typename EdgeT =
unsigned long long int>
using LinkPred::UNetwork< LabelT, NodeIDT, EdgeT >::RndNodeIt::reference = typename std↩
::iterator<std::input_iterator_tag, const std::pair<NodeID, Label>, long int>::reference
```

The reference type associated with the iterator.

9.98.3 Constructor & Destructor Documentation

9.98.3.1 RndNodeIt() [1/2]

```
template<typename LabelT = std::string, typename NodeIDT = unsigned int, typename EdgeT =
unsigned long long int>
LinkPred::UNetwork< LabelT, NodeIDT, EdgeT >::RndNodeIt::RndNodeIt (
            RndNodeIt const & that ) [default]
```

Copy constructor.

Parameters

that	The object to copy.

9.98.3.2 RndNodeIt() [2/2]

```
template<typename LabelT = std::string, typename NodeIDT = unsigned int, typename EdgeT =
unsigned long long int>
LinkPred::UNetwork< LabelT, NodeIDT, EdgeT >::RndNodeIt::RndNodeIt (
            RndNodeIt && that ) [default]
```

Move constructor.

Parameters

that	The object to move.

9.98.4 Member Function Documentation

9.98.4.1 operator"!=()

```
template<typename LabelT = std::string, typename NodeIDT = unsigned int, typename EdgeT =
unsigned long long int>
bool LinkPred::UNetwork< LabelT, NodeIDT, EdgeT >::RndNodeIt::operator!= (
            const RndNodeIt & that ) const [inline]
```

Parameters

that	The other iterator.

Returns

True if this is not equal to that.

9.98.4.2 operator*()

```
template<typename LabelT = std::string, typename NodeIDT = unsigned int, typename EdgeT =
unsigned long long int>
reference LinkPred::UNetwork< LabelT, NodeIDT, EdgeT >::RndNodeIt::operator* ( ) const [inline]
```

Dereference operator.

Returns

A reference to the object to which the iterator points.

9.98.4.3 operator++() [1/2]

```
template<typename LabelT = std::string, typename NodeIDT = unsigned int, typename EdgeT =
unsigned long long int>
RndNodeIt& LinkPred::UNetwork< LabelT, NodeIDT, EdgeT >::RndNodeIt::operator++ ( ) [inline]
```

Pre-increment operator.

Returns

A reference to the new iterator.

9.98.4.4 operator++() [2/2]

```
template<typename LabelT = std::string, typename NodeIDT = unsigned int, typename EdgeT =
unsigned long long int>
RndNodeIt LinkPred::UNetwork< LabelT, NodeIDT, EdgeT >::RndNodeIt::operator++ (
            int ) [inline]
```

Post-increment operator.

Returns

A reference to the new iterator.

9.98.4.5 operator->()

```
template<typename LabelT = std::string, typename NodeIDT = unsigned int, typename EdgeT =
unsigned long long int>
pointer LinkPred::UNetwork< LabelT, NodeIDT, EdgeT >::RndNodeIt::operator-> ( ) const [inline]
```

Arrow operator.

Returns

A pointer to the object to which the iterator points.

9.98.4.6 operator=() [1/2]

```
template<typename LabelT = std::string, typename NodeIDT = unsigned int, typename EdgeT =
unsigned long long int>
RndNodeIt& LinkPred::UNetwork< LabelT, NodeIDT, EdgeT >::RndNodeIt::operator= (
            RndNodeIt && that ) [default]
```

Move assignment operator.

Parameters

that	The object to move.

9.98.4.7 operator=() [2/2]

```
template<typename LabelT = std::string, typename NodeIDT = unsigned int, typename EdgeT =
unsigned long long int>
RndNodeIt& LinkPred::UNetwork< LabelT, NodeIDT, EdgeT >::RndNodeIt::operator= (
            RndNodeIt const & that )  [default]
```

Copy assignment operator.

Parameters

that	The object to copy.

9.98.4.8 operator==()

```
template<typename LabelT = std::string, typename NodeIDT = unsigned int, typename EdgeT =
unsigned long long int>
bool LinkPred::UNetwork< LabelT, NodeIDT, EdgeT >::RndNodeIt::operator== (
            const RndNodeIt & that ) const  [inline]
```

Parameters

that	The other iterator.

Returns

True if this equals that.

The documentation for this class was generated from the following file:

- include/linkpred/core/unetwork/unetwork.hpp

9.99 LinkPred::UNetwork< LabelT, NodeIDT, EdgeT >::RndNonEdgeIt Class Reference

Randomized nonedges iterator.

```
#include <unetwork.hpp>
```

Inheritance diagram for LinkPred::UNetwork< LabelT, NodeIDT, EdgeT >::RndNonEdgeIt:

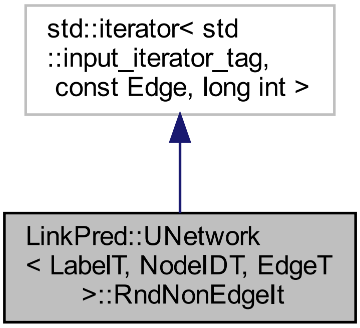

Collaboration diagram for LinkPred::UNetwork< LabelT, NodeIDT, EdgeT >::RndNonEdgeIt:

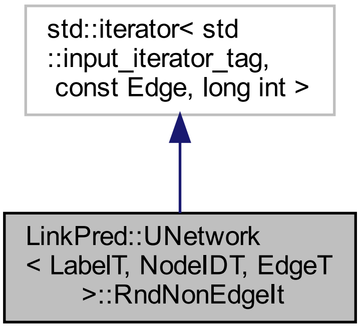

Public Types

- using pointer = typename std::iterator< std::input_iterator_tag, const Edge, long int >::pointer
- using reference = typename std::iterator< std::input_iterator_tag, const Edge, long int >::reference

Public Member Functions

- RndNonEdgeIt (RndNonEdgeIt const &that)=default
- RndNonEdgeIt & operator= (RndNonEdgeIt const &that)=default
- RndNonEdgeIt (RndNonEdgeIt &&that)=default
- RndNonEdgeIt & operator= (RndNonEdgeIt &&that)=default
- reference operator* ()
- pointer operator-> ()
- RndNonEdgeIt & operator++ ()
- RndNonEdgeIt operator++ (int)
- bool operator== (const RndNonEdgeIt &that) const
- bool operator!= (const RndNonEdgeIt &that) const

Friends

- class **UNetwork**

9.99.1 Detailed Description

**template<typename LabelT = std::string, typename NodeIDT = unsigned int, typename EdgeT = unsigned long long int>
class LinkPred::UNetwork< LabelT, NodeIDT, EdgeT >::RndNonEdgeIt**

Randomized nonedges iterator.

This is forward iterator that can be used to randomly sample a subset of the negative edges.

9.99.2 Member Typedef Documentation

9.99.2.1 pointer

```
template<typename LabelT = std::string, typename NodeIDT = unsigned int, typename EdgeT =
unsigned long long int>
using LinkPred::UNetwork< LabelT, NodeIDT, EdgeT >::RndNonEdgeIt::pointer = typename std↩
::iterator<std::input_iterator_tag, const Edge, long int>::pointer
```

The pointer type associated with the iterator.

9.99.2.2 reference

```
template<typename LabelT = std::string, typename NodeIDT = unsigned int, typename EdgeT =
unsigned long long int>
using LinkPred::UNetwork< LabelT, NodeIDT, EdgeT >::RndNonEdgeIt::reference = typename std↩
::iterator<std::input_iterator_tag, const Edge, long int>::reference
```

The reference type associated with the iterator.

9.99.3 Constructor & Destructor Documentation

9.99.3.1 RndNonEdgeIt() [1/2]

```
template<typename LabelT = std::string, typename NodeIDT = unsigned int, typename EdgeT =
unsigned long long int>
LinkPred::UNetwork< LabelT, NodeIDT, EdgeT >::RndNonEdgeIt::RndNonEdgeIt (
            RndNonEdgeIt const & that )  [default]
```

Copy constructor.

Parameters

that	The object to copy.

9.99.3.2 RndNonEdgeIt() [2/2]

```
template<typename LabelT = std::string, typename NodeIDT = unsigned int, typename EdgeT =
unsigned long long int>
LinkPred::UNetwork< LabelT, NodeIDT, EdgeT >::RndNonEdgeIt::RndNonEdgeIt (
            RndNonEdgeIt && that )  [default]
```

Move constructor.

Parameters

that	The object to move.

9.99.4 Member Function Documentation

9.99.4.1 operator"!=()

```
template<typename LabelT = std::string, typename NodeIDT = unsigned int, typename EdgeT =
unsigned long long int>
bool LinkPred::UNetwork< LabelT, NodeIDT, EdgeT >::RndNonEdgeIt::operator!= (
            const RndNonEdgeIt & that ) const  [inline]
```

Parameters

that	The other iterator.

Returns

True if this is not equal to that.

9.99.4.2 operator*()

```
template<typename LabelT = std::string, typename NodeIDT = unsigned int, typename EdgeT =
unsigned long long int>
reference LinkPred::UNetwork< LabelT, NodeIDT, EdgeT >::RndNonEdgeIt::operator* ( )  [inline]
```

Dereference operator.

Returns

A reference to the object to which the iterator points.

9.99.4.3 operator++() [1/2]

```
template<typename LabelT = std::string, typename NodeIDT = unsigned int, typename EdgeT =
unsigned long long int>
RndNonEdgeIt& LinkPred::UNetwork< LabelT, NodeIDT, EdgeT >::RndNonEdgeIt::operator++ ( )
[inline]
```

Pre-increment operator.

Returns

A reference to the new iterator.

9.99.4.4 operator++() [2/2]

```
template<typename LabelT = std::string, typename NodeIDT = unsigned int, typename EdgeT =
unsigned long long int>
RndNonEdgeIt LinkPred::UNetwork< LabelT, NodeIDT, EdgeT >::RndNonEdgeIt::operator++ (
            int ) [inline]
```

Post-increment operator.

Returns

A reference to the new iterator.

9.99.4.5 operator->()

```
template<typename LabelT = std::string, typename NodeIDT = unsigned int, typename EdgeT =
unsigned long long int>
pointer LinkPred::UNetwork< LabelT, NodeIDT, EdgeT >::RndNonEdgeIt::operator-> ( ) [inline]
```

Arrow operator.

Returns

A pointer to the object to which the iterator points.

9.99.4.6 operator=() [1/2]

```
template<typename LabelT = std::string, typename NodeIDT = unsigned int, typename EdgeT =
unsigned long long int>
RndNonEdgeIt& LinkPred::UNetwork< LabelT, NodeIDT, EdgeT >::RndNonEdgeIt::operator= (
            RndNonEdgeIt && that ) [default]
```

Move assignment operator.

Parameters

that	The object to move.

9.99.4.7 operator=() [2/2]

```
template<typename LabelT = std::string, typename NodeIDT = unsigned int, typename EdgeT =
unsigned long long int>
RndNonEdgeIt& LinkPred::UNetwork< LabelT, NodeIDT, EdgeT >::RndNonEdgeIt::operator= (
            RndNonEdgeIt const & that )  [default]
```

Copy assignment operator.

Parameters

that	The object to copy.

9.99.4.8 operator==()

```
template<typename LabelT = std::string, typename NodeIDT = unsigned int, typename EdgeT =
unsigned long long int>
bool LinkPred::UNetwork< LabelT, NodeIDT, EdgeT >::RndNonEdgeIt::operator== (
            const RndNonEdgeIt & that ) const  [inline]
```

Parameters

that	The other iterator.

Returns

True if this equals that.

The documentation for this class was generated from the following file:

- include/linkpred/core/unetwork/unetwork.hpp

9.100 LinkPred::DNetwork< LabelT, NodeIDT, EdgeT >::RndNonEdgeIt Class Reference

Randomized nonedges iterator.

```
#include <dnetwork.hpp>
```

Inheritance diagram for LinkPred::DNetwork< LabelT, NodeIDT, EdgeT >::RndNonEdgeIt:

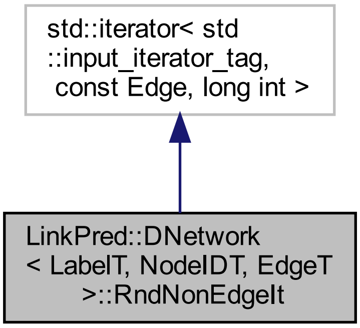

Collaboration diagram for LinkPred::DNetwork< LabelT, NodeIDT, EdgeT >::RndNonEdgeIt:

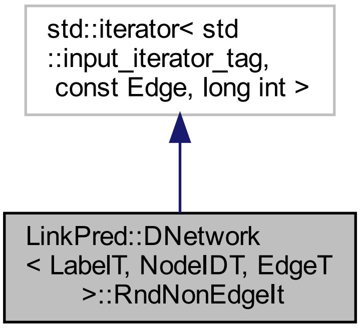

Public Types

- using pointer = typename std::iterator< std::input_iterator_tag, const Edge, long int >::pointer
- using reference = typename std::iterator< std::input_iterator_tag, const Edge, long int >::reference

Public Member Functions

- RndNonEdgeIt (RndNonEdgeIt const &that)=default
- RndNonEdgeIt & operator= (RndNonEdgeIt const &that)=default
- RndNonEdgeIt (RndNonEdgeIt &&that)=default
- RndNonEdgeIt & operator= (RndNonEdgeIt &&that)=default
- reference operator* ()
- pointer operator-> ()
- RndNonEdgeIt & operator++ ()
- RndNonEdgeIt operator++ (int)
- bool operator== (const RndNonEdgeIt &that) const
- bool operator!= (const RndNonEdgeIt &that) const

Friends

- class **DNetwork**

9.100.1 Detailed Description

template<typename LabelT = std::string, typename NodeIDT = unsigned int, typename EdgeT = unsigned long long int>
class LinkPred::DNetwork< LabelT, NodeIDT, EdgeT >::RndNonEdgeIt

Randomized nonedges iterator.

This is forward iterator that can be used to randomly sample a subset of the negative edges.

9.100.2 Member Typedef Documentation

9.100.2.1 pointer

```
template<typename LabelT = std::string, typename NodeIDT = unsigned int, typename EdgeT =
unsigned long long int>
using LinkPred::DNetwork< LabelT, NodeIDT, EdgeT >::RndNonEdgeIt::pointer = typename std↩
::iterator<std::input_iterator_tag, const Edge, long int>::pointer
```

The pointer type associated with the iterator.

9.100.2.2 reference

```
template<typename LabelT = std::string, typename NodeIDT = unsigned int, typename EdgeT =
unsigned long long int>
using LinkPred::DNetwork< LabelT, NodeIDT, EdgeT >::RndNonEdgeIt::reference = typename std↩
::iterator<std::input_iterator_tag, const Edge, long int>::reference
```

The reference type associated with the iterator.

9.100.3 Constructor & Destructor Documentation

9.100.3.1 RndNonEdgeIt() [1/2]

```
template<typename LabelT = std::string, typename NodeIDT = unsigned int, typename EdgeT =
unsigned long long int>
LinkPred::DNetwork< LabelT, NodeIDT, EdgeT >::RndNonEdgeIt::RndNonEdgeIt (
            RndNonEdgeIt const & that )  [default]
```

Copy constructor.

Parameters

that	The object to copy.

9.100.3.2 RndNonEdgeIt() [2/2]

```
template<typename LabelT = std::string, typename NodeIDT = unsigned int, typename EdgeT =
unsigned long long int>
LinkPred::DNetwork< LabelT, NodeIDT, EdgeT >::RndNonEdgeIt::RndNonEdgeIt (
            RndNonEdgeIt && that )  [default]
```

Move constructor.

Parameters

that	The object to move.

9.100.4 Member Function Documentation

9.100.4.1 operator"!=()

```
template<typename LabelT = std::string, typename NodeIDT = unsigned int, typename EdgeT =
unsigned long long int>
bool LinkPred::DNetwork< LabelT, NodeIDT, EdgeT >::RndNonEdgeIt::operator!= (
            const RndNonEdgeIt & that ) const  [inline]
```

Parameters

that	The other iterator.

Returns

True if this is not equal to that.

9.100.4.2 operator∗()

```
template<typename LabelT = std::string, typename NodeIDT = unsigned int, typename EdgeT =
unsigned long long int>
reference LinkPred::DNetwork< LabelT, NodeIDT, EdgeT >::RndNonEdgeIt::operator* ( )  [inline]
```

Dereference operator.

Returns

A reference to the object to which the iterator points.

9.100.4.3 operator++() [1/2]

```
template<typename LabelT = std::string, typename NodeIDT = unsigned int, typename EdgeT =
unsigned long long int>
RndNonEdgeIt& LinkPred::DNetwork< LabelT, NodeIDT, EdgeT >::RndNonEdgeIt::operator++ ( )
[inline]
```

Pre-increment operator.

Returns

A reference to the new iterator.

9.100.4.4 operator++() [2/2]

```
template<typename LabelT = std::string, typename NodeIDT = unsigned int, typename EdgeT =
unsigned long long int>
RndNonEdgeIt LinkPred::DNetwork< LabelT, NodeIDT, EdgeT >::RndNonEdgeIt::operator++ (
            int ) [inline]
```

Post-increment operator.

Returns

A reference to the new iterator.

9.100.4.5 operator->()

```
template<typename LabelT = std::string, typename NodeIDT = unsigned int, typename EdgeT =
unsigned long long int>
pointer LinkPred::DNetwork< LabelT, NodeIDT, EdgeT >::RndNonEdgeIt::operator-> ( ) [inline]
```

Arrow operator.

Returns

A pointer to the object to which the iterator points.

9.100.4.6 operator=() [1/2]

```
template<typename LabelT = std::string, typename NodeIDT = unsigned int, typename EdgeT =
unsigned long long int>
RndNonEdgeIt& LinkPred::DNetwork< LabelT, NodeIDT, EdgeT >::RndNonEdgeIt::operator= (
            RndNonEdgeIt && that ) [default]
```

Move assignment operator.

Parameters

that	The object to move.

9.100.4.7 operator=() [2/2]

```
template<typename LabelT = std::string, typename NodeIDT = unsigned int, typename EdgeT =
unsigned long long int>
RndNonEdgeIt& LinkPred::DNetwork< LabelT, NodeIDT, EdgeT >::RndNonEdgeIt::operator= (
            RndNonEdgeIt const & that )  [default]
```

Copy assignment operator.

Parameters

that	The object to copy.

9.100.4.8 operator==()

```
template<typename LabelT = std::string, typename NodeIDT = unsigned int, typename EdgeT =
unsigned long long int>
bool LinkPred::DNetwork< LabelT, NodeIDT, EdgeT >::RndNonEdgeIt::operator== (
            const RndNonEdgeIt & that ) const  [inline]
```

Parameters

that	The other iterator.

Returns

True if this equals that.

The documentation for this class was generated from the following file:

- include/linkpred/core/dnetwork/dnetwork.hpp

9.101 LinkPred::Simp::Evaluator::Factory::RNDParams Struct Reference

Parameters of RND.

```
#include <evaluator.hpp>
```

Public Attributes

- long int seed = 0

9.101.1 Detailed Description

Parameters of RND.

9.101.2 Member Data Documentation

9.101.2.1 seed

```
long int LinkPred::Simp::Evaluator::Factory::RNDParams::seed = 0
```

The random number generator seed for RND.

The documentation for this struct was generated from the following file:

- include/linkpred/simp/evaluator.hpp

9.102 LinkPred::ROC< PredResultsT > Class Template Reference

Receiver Operating Characteristic curve.

```
#include <perfmeasure.hpp>
```

Inheritance diagram for LinkPred::ROC< PredResultsT >:

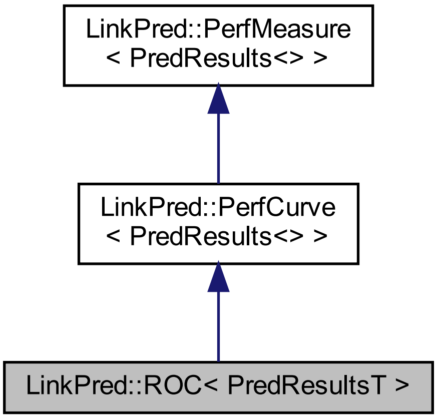

Collaboration diagram for LinkPred::ROC< PredResultsT >:

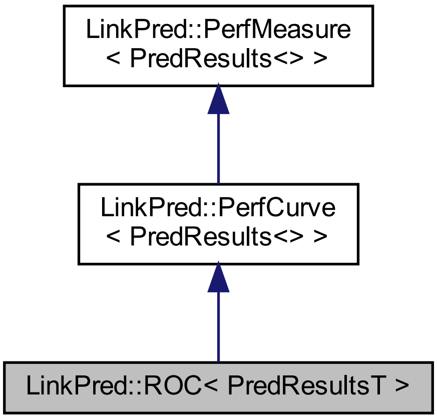

Public Types

- using ScoresItT = typename PerfMeasure< PredResultsT >::ScoresItT

Public Member Functions

- ROC ()
- ROC (std::string name)
- ROC (ROC const &that)=default
- ROC & operator= (ROC const &that)=default
- ROC (ROC &&that)=default
- ROC & operator= (ROC &&that)=default
- bool isStrmEnabled () const
- void setStrmEnabled (bool strmEnabled)
- bool isStrmNeg () const
- void setStrmNeg (bool strmNeg)
- virtual void eval (ScoresItT posScoresBegin, ScoresItT posScoresEnd, ScoresItT negScoresBegin, ScoresItT negScoresEnd, SortOrder &posSortOrder, SortOrder &negSortOrder, PerfResults &results)
- virtual void eval (std::shared_ptr< PredResultsT > &predResults, PerfResults &results)
- virtual void evalNoStream (std::shared_ptr< PredResultsT > &predResults, PerfResults &results)
- template<typename StrmScoresItT , typename StoredScoresItT >
  double getROCAucStrm (StrmScoresItT strmBegin, StrmScoresItT strmEnd, StoredScoresItT strdBegin, StoredScoresItT strdEnd)
- virtual void evalStream (std::shared_ptr< PredResultsT > &predResults, PerfResults &results)
- virtual std::vector< std::pair< double, double > > getCurve (std::shared_ptr< PredResultsT > &pred↩ Results)
- virtual std::vector< std::pair< double, double > > getCurve (ScoresItT posScoresBegin, ScoresItT pos↩ ScoresEnd, ScoresItT negScoresBegin, ScoresItT negScoresEnd, SortOrder &posSortOrder, SortOrder &negSortOrder)
- virtual ~ROC ()=default

Static Public Member Functions

- template<typename ScoresItT >
  static double getROCAuc (ScoresItT posScoresBegin, ScoresItT posScoresEnd, ScoresItT negScoresBegin, ScoresItT negScoresEnd, bool parallel=false)
- template<typename ScoresItT >
  static std::vector< double > getThresholds (ScoresItT posScoresBegin, ScoresItT posScoresEnd, ScoresItT negScoresBegin, ScoresItT negScoresEnd)
- template<typename ScoresItT >
  static std::vector< std::pair< double, double > > getROCCurve (ScoresItT posScoresBegin, ScoresItT posScoresEnd, ScoresItT negScoresBegin, ScoresItT negScoresEnd, bool parallel=false)

9.102.1 Detailed Description

template<typename PredResultsT = PredResults<>>
class LinkPred::ROC< PredResultsT >

Receiver Operating Characteristic curve.

Template Parameters

PredResultsT	The prediction results type.

9.102.2 Member Typedef Documentation

9.102.2.1 ScoresItT

```
template<typename PredResultsT = PredResults<>>
using LinkPred::ROC< PredResultsT >::ScoresItT = typename PerfMeasure<PredResultsT>::ScoresItT
```

Scores iterator type.

9.102.3 Constructor & Destructor Documentation

9.102.3.1 ROC() [1/4]

```
template<typename PredResultsT = PredResults<>>
LinkPred::ROC< PredResultsT >::ROC ( )  [inline]
```

< The name of the performance measure. Constructor.

9.102.3.2 ROC() [2/4]

```
template<typename PredResultsT = PredResults<>>
LinkPred::ROC< PredResultsT >::ROC (
            std::string name )  [inline]
```

Constructor.

Parameters

name	The name of the performance measure.

9.102.3.3 ROC() [3/4]

```
template<typename PredResultsT = PredResults<>>
LinkPred::ROC< PredResultsT >::ROC (
            ROC< PredResultsT > const & that )  [default]
```

Copy constructor.

Parameters

that	The object to copy.

9.102.3.4 ROC() [4/4]

```
template<typename PredResultsT = PredResults<>>
LinkPred::ROC< PredResultsT >::ROC (
            ROC< PredResultsT > && that )  [default]
```

Move constructor.

Parameters

that	The object to move.

9.102.3.5 ∼ROC()

```
template<typename PredResultsT = PredResults<>>
virtual LinkPred::ROC< PredResultsT >::~ROC ( )  [virtual], [default]
```

Destructor.

9.102.4 Member Function Documentation

9.102.4.1 eval() [1/2]

```
template<typename PredResultsT = PredResults<>>
virtual void LinkPred::ROC< PredResultsT >::eval (
            ScoresItT posScoresBegin,
            ScoresItT posScoresEnd,
            ScoresItT negScoresBegin,
            ScoresItT negScoresEnd,
            SortOrder & posSortOrder,
            SortOrder & negSortOrder,
            PerfResults & results )  [inline], [virtual]
```

Computes the area under the ROC curve.

Parameters

posScoresBegin	Iterator to the first positive score.
posScoresEnd	Iterator to one-past-the-last positive score.
negScoresBegin	Iterator to the first negative score.
negScoresEnd	Iterator to one-past-the-last negative score.
posSortOrder	The sorting order of positive scores. This may be modified by the method.
negSortOrder	The sorting order of negative scores. This may be modified by the method.
results	To write results.

9.102.4.2 eval() [2/2]

```
template<typename PredResultsT = PredResults<>>
virtual void LinkPred::ROC< PredResultsT >::eval (
            std::shared_ptr< PredResultsT > & predResults,
            PerfResults & results )  [inline], [virtual]
```

Computes the area under the ROC curve.

Parameters

predResults	The prediction results.
results	Iterator to write results.

9.102.4.3 evalNoStream()

```
template<typename PredResultsT = PredResults<>>
virtual void LinkPred::ROC< PredResultsT >::evalNoStream (
            std::shared_ptr< PredResultsT > & predResults,
            PerfResults & results )  [inline], [virtual]
```

Computes the area under the ROC curve.

Parameters

predResults	The prediction results.
results	Iterator to write results.

9.102.4.4 evalStream()

```
template<typename PredResultsT = PredResults<>>
virtual void LinkPred::ROC< PredResultsT >::evalStream (
            std::shared_ptr< PredResultsT > & predResults,
            PerfResults & results ) [inline], [virtual]
```

Computes the area under the ROC curve with streaming scores.

Parameters

predResults	The prediction results.
results	Iterator to write results.

9.102.4.5 getCurve() [1/2]

```
template<typename PredResultsT = PredResults<>>
virtual std::vector<std::pair<double, double> > LinkPred::ROC< PredResultsT >::getCurve (
            ScoresItT posScoresBegin,
            ScoresItT posScoresEnd,
            ScoresItT negScoresBegin,
            ScoresItT negScoresEnd,
            SortOrder & posSortOrder,
            SortOrder & negSortOrder ) [inline], [virtual]
```

Computes the ROC curve.

Parameters

posScoresBegin	Iterator to the first positive score.
posScoresEnd	Iterator to one-past-the-last positive score.
negScoresBegin	Iterator to the first negative score.
negScoresEnd	Iterator to one-past-the-last negative score.
posSortOrder	The sorting order of positive scores. This may be modified by the method.
negSortOrder	The sorting order of negative scores. This may be modified by the method.

Returns

A curve in the form of an std::vector of pairs representing the x and y coordinates.

9.102.4.6 getCurve() [2/2]

```
template<typename PredResultsT = PredResults<>>
virtual std::vector<std::pair<double, double> > LinkPred::ROC< PredResultsT >::getCurve (
            std::shared_ptr< PredResultsT > & predResults ) [inline], [virtual]
```

Computes the ROC curve.

Parameters

predResults	The prediction results.

Returns

A curve in the form of an std::vector of pairs representing the x and y coordinates.

9.102.4.7 getROCAuc()

```
template<typename PredResultsT = PredResults<>>
template<typename ScoresItT >
static double LinkPred::ROC< PredResultsT >::getROCAuc (
            ScoresItT posScoresBegin,
            ScoresItT posScoresEnd,
            ScoresItT negScoresBegin,
            ScoresItT negScoresEnd,
            bool parallel = false ) [inline], [static]
```

Compute the area under the ROC curve. The smallest range must be sorted in increasing order (in case of a tie, the false negative ranges must be sorted).

Parameters

posScoresBegin	Iterator to the first positive score.
posScoresEnd	Iterator to one-past-the-last positive score.
negScoresBegin	Iterator to the first negative score.
negScoresEnd	Iterator to one-past-the-last negative score.
parallel	Whether to run in parallel.

Returns

The area under the ROC curve.

9.102.4.8 getROCAucStrm()

```
template<typename PredResultsT = PredResults<>>
template<typename StrmScoresItT , typename StoredScoresItT >
```

```
double LinkPred::ROC< PredResultsT >::getROCAucStrm (
            StrmScoresItT strmBegin,
            StrmScoresItT strmEnd,
            StoredScoresItT strdBegin,
            StoredScoresItT strdEnd ) [inline]
```

Compute the area under the ROC curve with streaming. The smallest range must be sorted in increasing order (in case of a tie, the false negative ranges must be sorted).

Parameters

strmBegin	Iterator to the first streamed score.
strmEnd	Iterator to one-past-the-last streamed score.
strdBegin	Iterator to the first stored score.
strdEnd	Iterator to one-past-the-last stored score.

Returns

The area under the ROC curve.

9.102.4.9 getROCCurve()

```
template<typename PredResultsT = PredResults<>>
template<typename ScoresItT >
static std::vector<std::pair<double, double> > LinkPred::ROC< PredResultsT >::getROCCurve (
            ScoresItT posScoresBegin,
            ScoresItT posScoresEnd,
            ScoresItT negScoresBegin,
            ScoresItT negScoresEnd,
            bool parallel = false ) [inline], [static]
```

Compute the ROC curve. Both ranges must be sorted.

Parameters

posScoresBegin	Iterator to the first positive score.
posScoresEnd	Iterator to one-past-the-last positive score.
negScoresBegin	Iterator to the first negative score.
negScoresEnd	Iterator to one-past-the-last negative score.
parallel	Whether to run in parallel.

9.102.4.10 getThresholds()

```
template<typename PredResultsT = PredResults<>>
template<typename ScoresItT >
```

```
static std::vector<double> LinkPred::ROC< PredResultsT >::getThresholds (
            ScoresItT posScoresBegin,
            ScoresItT posScoresEnd,
            ScoresItT negScoresBegin,
            ScoresItT negScoresEnd )  [inline], [static]
```

Compute the threshold of the ROC curve. Ranges must be sorted in increasing order.

Parameters

posScoresBegin	Iterator to the first positive score.
posScoresEnd	Iterator to one-past-the-last positive score.
negScoresBegin	Iterator to the first negative score.
negScoresEnd	Iterator to one-past-the-last negative score.

9.102.4.11 isStrmEnabled()

```
template<typename PredResultsT = PredResults<>>
bool LinkPred::ROC< PredResultsT >::isStrmEnabled ( ) const  [inline]
```

Returns

Whether streaming is enabled.

9.102.4.12 isStrmNeg()

```
template<typename PredResultsT = PredResults<>>
bool LinkPred::ROC< PredResultsT >::isStrmNeg ( ) const  [inline]
```

Returns

Which class to stream. If true negative scores are streamed, else positive scores are. Only used if enableStrm is true.

9.102.4.13 operator=() [1/2]

```
template<typename PredResultsT = PredResults<>>
ROC& LinkPred::ROC< PredResultsT >::operator= (
            ROC< PredResultsT > && that )  [default]
```

Move assignment operator.

Parameters

that	The object to move.

9.102.4.14 operator=() [2/2]

```
template<typename PredResultsT = PredResults<>>
ROC& LinkPred::ROC< PredResultsT >::operator= (
            ROC< PredResultsT > const & that )  [default]
```

Copy assignment operator.

Parameters

that	The object to copy.

9.102.4.15 setStrmEnabled()

```
template<typename PredResultsT = PredResults<>>
void LinkPred::ROC< PredResultsT >::setStrmEnabled (
            bool strmEnabled )  [inline]
```

Parameters

strmEnabled	Whether to enable streaming.

9.102.4.16 setStrmNeg()

```
template<typename PredResultsT = PredResults<>>
void LinkPred::ROC< PredResultsT >::setStrmNeg (
            bool strmNeg )  [inline]
```

Set which class to stream. If true negative scores are streamed, else positive scores are. Only used if enableStrm is true.

Parameters

strmNeg	If true negative scores are streamed, else positive scores are. Only used if enableStrm is true.

The documentation for this class was generated from the following file:

- include/linkpred/perf/perfmeasure.hpp

9.103 LinkPred::Simp::Evaluator::Factory::SBMParams Struct Reference

Parameters of SBM.

```
#include <evaluator.hpp>
```

Public Attributes

- int maxIter = 1000
- long int seed = 0

9.103.1 Detailed Description

Parameters of SBM.

9.103.2 Member Data Documentation

9.103.2.1 maxIter

```
int LinkPred::Simp::Evaluator::Factory::SBMParams::maxIter = 1000
```

Max iterations for SBM.

9.103.2.2 seed

```
long int LinkPred::Simp::Evaluator::Factory::SBMParams::seed = 0
```

Seed for SBM.

The documentation for this struct was generated from the following file:

- include/linkpred/simp/evaluator.hpp

9.104 LinkPred::PredResults< TestDataT, LPredictorT, ScoresContainerT >::ScoreIterator Class Reference

Score iterator.

```
#include <predresults.hpp>
```

Inheritance diagram for LinkPred::PredResults< TestDataT, LPredictorT, ScoresContainerT >::ScoreIterator:

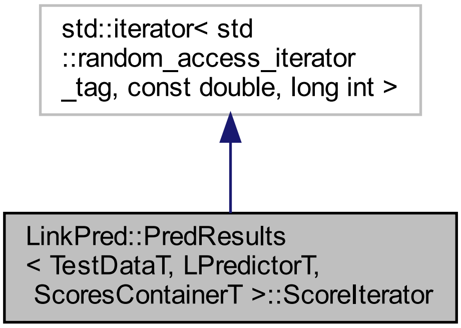

Collaboration diagram for LinkPred::PredResults< TestDataT, LPredictorT, ScoresContainerT >::ScoreIterator:

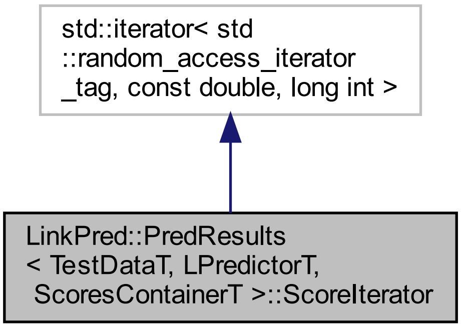

Public Types

- using pointer = typename std::iterator< std::random_access_iterator_tag, const std::pair< typename TestDataT::Edge, double >, long int >::pointer
- using reference = typename std::iterator< std::random_access_iterator_tag, const std::pair< typename TestDataT::Edge, double >, long int >::reference
- using difference_type = typename std::iterator< std::random_access_iterator_tag, const std::pair< typename TestDataT::Edge, double >, long int >::difference_type

Public Member Functions

- ScoreIterator (std::shared_ptr< LPredictorT > const &predictor, typename TestDataT::TestEdgeIt const &eit)
- ScoreIterator (ScoreIterator const &that)=default
- ScoreIterator & operator= (ScoreIterator const &that)=default
- ScoreIterator (ScoreIterator &&that)=default
- ScoreIterator & operator= (ScoreIterator &&that)=default
- reference operator* ()
- pointer operator-> ()
- ScoreIterator & operator++ ()
- ScoreIterator & operator-- ()
- ScoreIterator operator++ (int)
- ScoreIterator operator-- (int)
- ScoreIterator operator+ (const difference_type &n) const
- ScoreIterator & operator+= (const difference_type &n)
- ScoreIterator operator- (const difference_type &n) const
- ScoreIterator & operator-= (const difference_type &n)
- difference_type operator- (const ScoreIterator &that) const
- bool operator== (const ScoreIterator &that) const
- bool operator!= (const ScoreIterator &that) const
- bool operator< (const ScoreIterator &that) const
- bool operator> (const ScoreIterator &that) const
- bool operator<= (const ScoreIterator &that) const
- bool operator>= (const ScoreIterator &that) const

Friends

- class PredResults

9.104.1 Detailed Description

**template<typename TestDataT = TestData<>, typename LPredictorT = ULPredictor<>, typename ScoresContainerT = std↩
::vector<double>>
class LinkPred::PredResults< TestDataT, LPredictorT, ScoresContainerT >::ScoreIterator**

Score iterator.

9.104.2 Member Typedef Documentation

9.104.2.1 difference_type

```
template<typename TestDataT = TestData<>, typename LPredictorT = ULPredictor<>, typename
ScoresContainerT = std::vector<double>>
using LinkPred::PredResults< TestDataT, LPredictorT, ScoresContainerT >::ScoreIterator::difference_type
= typename std::iterator<std::random_access_iterator_tag, const std::pair<typename TestDataT↩
::Edge, double>, long int>::difference_type
```

The difference type associated with the iterator.

9.104.2.2 pointer

```
template<typename TestDataT = TestData<>, typename LPredictorT = ULPredictor<>, typename
ScoresContainerT = std::vector<double>>
using LinkPred::PredResults< TestDataT, LPredictorT, ScoresContainerT >::ScoreIterator::pointer
= typename std::iterator<std::random_access_iterator_tag, const std::pair<typename TestDataT↩
::Edge, double>, long int>::pointer
```

The pointer type associated with the iterator.

9.104.2.3 reference

```
template<typename TestDataT = TestData<>, typename LPredictorT = ULPredictor<>, typename
ScoresContainerT = std::vector<double>>
using LinkPred::PredResults< TestDataT, LPredictorT, ScoresContainerT >::ScoreIterator::reference
= typename std::iterator<std::random_access_iterator_tag, const std::pair<typename TestDataT↩
::Edge, double>, long int>::reference
```

The reference type associated with the iterator.

9.104.3 Constructor & Destructor Documentation

9.104.3.1 ScoreIterator() [1/3]

```
template<typename TestDataT = TestData<>, typename LPredictorT = ULPredictor<>, typename
ScoresContainerT = std::vector<double>>
LinkPred::PredResults< TestDataT, LPredictorT, ScoresContainerT >::ScoreIterator::Score↩
Iterator (
            std::shared_ptr< LPredictorT > const & predictor,
            typename TestDataT::TestEdgeIt const & eit )  [inline]
```

Constructor.

Parameters

predictor	The link predictor.
eit	Test edge iterator.

9.104.3.2 ScoreIterator() [2/3]

```
template<typename TestDataT = TestData<>, typename LPredictorT = ULPredictor<>, typename
ScoresContainerT = std::vector<double>>
LinkPred::PredResults< TestDataT, LPredictorT, ScoresContainerT >::ScoreIterator::Score↩
Iterator (
            ScoreIterator const & that )  [default]
```

Copy constructor.

Parameters

that	The object to copy.

9.104.3.3 ScoreIterator() [3/3]

```
template<typename TestDataT = TestData<>, typename LPredictorT = ULPredictor<>, typename
ScoresContainerT = std::vector<double>>
LinkPred::PredResults< TestDataT, LPredictorT, ScoresContainerT >::ScoreIterator::Score↩
Iterator (
            ScoreIterator && that )  [default]
```

Move constructor.

Parameters

that	The object to move.

9.104.4 Member Function Documentation

9.104.4.1 operator"!=()

```
template<typename TestDataT = TestData<>, typename LPredictorT = ULPredictor<>, typename
ScoresContainerT = std::vector<double>>
bool LinkPred::PredResults< TestDataT, LPredictorT, ScoresContainerT >::ScoreIterator::operator!=
(
            const ScoreIterator & that ) const  [inline]
```

Parameters

that	The other iterator.

Returns

True if this is not equal to that.

9.104.4.2 operator∗()

```
template<typename TestDataT = TestData<>, typename LPredictorT = ULPredictor<>, typename
ScoresContainerT = std::vector<double>>
reference LinkPred::PredResults< TestDataT, LPredictorT, ScoresContainerT >::ScoreIterator↩
::operator* ( )  [inline]
```

Dereference operator.

Returns

A reference to the object to which the iterator points.

9.104.4.3 operator+()

```
template<typename TestDataT = TestData<>, typename LPredictorT = ULPredictor<>, typename
ScoresContainerT = std::vector<double>>
ScoreIterator LinkPred::PredResults< TestDataT, LPredictorT, ScoresContainerT >::ScoreIterator↩
::operator+ (
            const difference_type & n ) const  [inline]
```

Arithmetic + operator.

Parameters

n	Increment value.

Returns

The new iterator.

9.104.4.4 operator++() [1/2]

```
template<typename TestDataT = TestData<>, typename LPredictorT = ULPredictor<>, typename
ScoresContainerT = std::vector<double>>
ScoreIterator& LinkPred::PredResults< TestDataT, LPredictorT, ScoresContainerT >::Score↩
Iterator::operator++ ( )  [inline]
```

Pre-increment operator.

Returns

A reference to the new iterator.

9.104.4.5 operator++() [2/2]

```
template<typename TestDataT = TestData<>, typename LPredictorT = ULPredictor<>, typename
ScoresContainerT = std::vector<double>>
ScoreIterator LinkPred::PredResults< TestDataT, LPredictorT, ScoresContainerT >::ScoreIterator↩
::operator++ (
            int  )  [inline]
```

Post-increment operator.

Returns

A reference to the new iterator.

9.104.4.6 operator+=()

```
template<typename TestDataT = TestData<>, typename LPredictorT = ULPredictor<>, typename
ScoresContainerT = std::vector<double>>
ScoreIterator& LinkPred::PredResults< TestDataT, LPredictorT, ScoresContainerT >::Score↩
Iterator::operator+= (
            const difference_type & n )  [inline]
```

Arithmetic += operator.

Parameters

n	Increment value.

Returns

A reference to the new iterator.

9.104.4.7 operator-() [1/2]

```
template<typename TestDataT = TestData<>, typename LPredictorT = ULPredictor<>, typename
ScoresContainerT = std::vector<double>>
ScoreIterator LinkPred::PredResults< TestDataT, LPredictorT, ScoresContainerT >::ScoreIterator↩
::operator- (
            const difference_type & n ) const  [inline]
```

Arithmetic - operator.

Parameters

n	Decrement value.

Returns

The new iterator.

9.104.4.8 operator-() [2/2]

```
template<typename TestDataT = TestData<>, typename LPredictorT = ULPredictor<>, typename
ScoresContainerT = std::vector<double>>
difference_type LinkPred::PredResults< TestDataT, LPredictorT, ScoresContainerT >::Score↩
Iterator::operator- (
            const ScoreIterator & that ) const  [inline]
```

Difference between the present iterator and the one passed as parameter.

Parameters

that	The other iterator.

Returns

The difference between the current and that iterator.

9.104.4.9 operator--() [1/2]

```
template<typename TestDataT = TestData<>, typename LPredictorT = ULPredictor<>, typename
ScoresContainerT = std::vector<double>>
ScoreIterator& LinkPred::PredResults< TestDataT, LPredictorT, ScoresContainerT >::Score↩
Iterator::operator-- ( ) [inline]
```

Pre-decrement operator.

Returns

A reference to the new iterator.

9.104.4.10 operator--() [2/2]

```
template<typename TestDataT = TestData<>, typename LPredictorT = ULPredictor<>, typename
ScoresContainerT = std::vector<double>>
ScoreIterator LinkPred::PredResults< TestDataT, LPredictorT, ScoresContainerT >::ScoreIterator↩
::operator-- (
             int ) [inline]
```

Post-decrement operator.

Returns

A reference to the new iterator.

9.104.4.11 operator-=()

```
template<typename TestDataT = TestData<>, typename LPredictorT = ULPredictor<>, typename
ScoresContainerT = std::vector<double>>
ScoreIterator& LinkPred::PredResults< TestDataT, LPredictorT, ScoresContainerT >::Score↩
Iterator::operator-= (
             const difference_type & n ) [inline]
```

Arithmetic -= operator.

Parameters

n	Decrement value.

Returns

A reference to the new iterator.

9.104.4.12 operator->()

```
template<typename TestDataT = TestData<>, typename LPredictorT = ULPredictor<>, typename
ScoresContainerT = std::vector<double>>
pointer LinkPred::PredResults< TestDataT, LPredictorT, ScoresContainerT >::ScoreIterator↩
::operator-> ( ) [inline]
```

Arrow operator.

Returns

A pointer to the object to which the iterator points.

9.104.4.13 operator<()

```
template<typename TestDataT = TestData<>, typename LPredictorT = ULPredictor<>, typename
ScoresContainerT = std::vector<double>>
bool LinkPred::PredResults< TestDataT, LPredictorT, ScoresContainerT >::ScoreIterator::operator<
(
            const ScoreIterator & that ) const [inline]
```

Parameters

that	The other iterator.

Returns

True if this is less than that.

9.104.4.14 operator<=()

```
template<typename TestDataT = TestData<>, typename LPredictorT = ULPredictor<>, typename
ScoresContainerT = std::vector<double>>
bool LinkPred::PredResults< TestDataT, LPredictorT, ScoresContainerT >::ScoreIterator::operator<=
(
            const ScoreIterator & that ) const [inline]
```

Parameters

that	The other iterator.

Returns

True if this is greater or equal to that.

9.104.4.15 operator=() [1/2]

```
template<typename TestDataT = TestData<>, typename LPredictorT = ULPredictor<>, typename
ScoresContainerT = std::vector<double>>
ScoreIterator& LinkPred::PredResults< TestDataT, LPredictorT, ScoresContainerT >::Score↩
Iterator::operator= (
            ScoreIterator && that )  [default]
```

Move assignment operator.

Parameters

that	The object to move.

9.104.4.16 operator=() [2/2]

```
template<typename TestDataT = TestData<>, typename LPredictorT = ULPredictor<>, typename
ScoresContainerT = std::vector<double>>
ScoreIterator& LinkPred::PredResults< TestDataT, LPredictorT, ScoresContainerT >::Score↩
Iterator::operator= (
            ScoreIterator const & that )  [default]
```

Copy assignment operator.

Parameters

that	The object to copy.

9.104.4.17 operator==()

```
template<typename TestDataT = TestData<>, typename LPredictorT = ULPredictor<>, typename
ScoresContainerT = std::vector<double>>
bool LinkPred::PredResults< TestDataT, LPredictorT, ScoresContainerT >::ScoreIterator::operator==
(
            const ScoreIterator & that ) const  [inline]
```

Parameters

that	The other iterator.

Returns

True if this equals that.

9.104.4.18 operator>()

```
template<typename TestDataT = TestData<>, typename LPredictorT = ULPredictor<>, typename
ScoresContainerT = std::vector<double>>
bool LinkPred::PredResults< TestDataT, LPredictorT, ScoresContainerT >::ScoreIterator::operator>
(
            const ScoreIterator & that ) const  [inline]
```

Parameters

that	The other iterator.

Returns

True if this is greater that.

9.104.4.19 operator>=()

```
template<typename TestDataT = TestData<>, typename LPredictorT = ULPredictor<>, typename
ScoresContainerT = std::vector<double>>
bool LinkPred::PredResults< TestDataT, LPredictorT, ScoresContainerT >::ScoreIterator::operator>=
(
            const ScoreIterator & that ) const  [inline]
```

Parameters

that	The other iterator.

Returns

True if this is less or equal to that.

9.104.5 Friends And Related Function Documentation

9.104.5.1 PredResults

```
template<typename TestDataT = TestData<>, typename LPredictorT = ULPredictor<>, typename
ScoresContainerT = std::vector<double>>
friend class PredResults [friend]
```

PredResults is a friend.

The documentation for this class was generated from the following file:

- include/linkpred/perf/predresults.hpp

9.105 LinkPred::Simp::Evaluator::Factory::SHPParams Struct Reference

Parameters of SHP.

```
#include <evaluator.hpp>
```

Public Attributes

- long int seed = 0

9.105.1 Detailed Description

Parameters of SHP.

9.105.2 Member Data Documentation

9.105.2.1 seed

```
long int LinkPred::Simp::Evaluator::Factory::SHPParams::seed = 0
```

The random number generator seed for SHP.

The documentation for this struct was generated from the following file:

- include/linkpred/simp/evaluator.hpp

9.106 LinkPred::SimMeasure Class Reference

Interface of a similarity measure.

`#include <simmeasure.hpp>`

Inheritance diagram for LinkPred::SimMeasure:

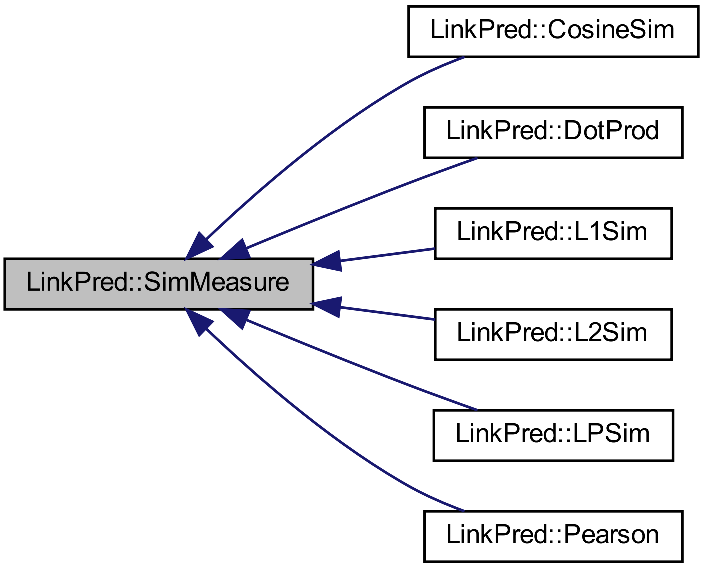

Public Member Functions

- SimMeasure ()=default
- SimMeasure (SimMeasure const &that)=default
- SimMeasure & operator= (SimMeasure const &that)=default
- SimMeasure (SimMeasure &&that)=default
- SimMeasure & operator= (SimMeasure &&that)=default
- virtual double sim (Vec const &v1, Vec const &v2)=0
- const std::string & getName () const
- void setName (const std::string &name)
- virtual ∼SimMeasure ()=default

9.106.1 Detailed Description

Interface of a similarity measure.

9.106.2 Constructor & Destructor Documentation

9.106.2.1 SimMeasure() [1/3]

```
LinkPred::SimMeasure::SimMeasure ( ) [default]
```

Default constructor.

9.106.2.2 SimMeasure() [2/3]

```
LinkPred::SimMeasure::SimMeasure (
            SimMeasure const & that ) [default]
```

Copy constructor.

Parameters

that	The object to copy.

9.106.2.3 SimMeasure() [3/3]

```
LinkPred::SimMeasure::SimMeasure (
            SimMeasure && that ) [default]
```

Move constructor.

Parameters

that	The object to move.

9.106.2.4 ∼SimMeasure()

```
virtual LinkPred::SimMeasure::∼SimMeasure ( ) [virtual], [default]
```

Destructor.

9.106.3 Member Function Documentation

9.106.3.1 getName()

```
const std::string& LinkPred::SimMeasure::getName ( ) const [inline]
```

Returns

The name of the SimMeasure.

9.106.3.2 operator=() [1/2]

```
SimMeasure& LinkPred::SimMeasure::operator= (
            SimMeasure && that )  [default]
```

Move assignment operator.

Parameters

that	The object to move.

9.106.3.3 operator=() [2/2]

```
SimMeasure& LinkPred::SimMeasure::operator= (
            SimMeasure const & that )  [default]
```

Copy assignment operator.

Parameters

that	The object to copy.

9.106.3.4 setName()

```
void LinkPred::SimMeasure::setName (
            const std::string & name )  [inline]
```

Set the name of the SimMeasure.

Parameters

name	The new name of the SimMeasure.

9.106.3.5 sim()

```
virtual double LinkPred::SimMeasure::sim (
            Vec const & v1,
            Vec const & v2 )  [pure virtual]
```

Compute the similarity between two vectors.

Parameters

v1	First vector.
v2	Second vector. Must be of the same dimension as v1.

Returns

The similarity between v1 and v2.

Implemented in LinkPred::LPSim, LinkPred::CosineSim, LinkPred::DotProd, LinkPred::L1Sim, LinkPred::L2Sim, and LinkPred::Pearson.

The documentation for this class was generated from the following file:

- include/linkpred/ml/simmeasures/simmeasure.hpp

9.107 LinkPred::TestData< Network, EdgeContT > Class Template Reference

Test data.

```
#include <networkmanipulator.hpp>
```

Classes

- class TestEdgeIt

  Test edges iterator.

Public Types

- enum IteratorType { ECEIT, TPEIT, TNEIT }

  Enumeration of iterator types.
- using NetworkSP = std::shared_ptr< Network >
- using NetworkCSP = std::shared_ptr< const Network >
- using Edge = typename Network::Edge
- using NonEdgeIt = typename Network::NonEdgeIt
- using EdgeIt = typename Network::EdgeIt

Public Member Functions

- TestData (NetworkCSP refNet, NetworkCSP obsNet, std::shared_ptr< EdgeContT > remLinks, std::shared_ptr< EdgeContT > addLinks, std::shared_ptr< EdgeContT > tpLinks, std::shared_ptr< EdgeContT > tnLinks, LinkClass posClass, LinkClass negClass)
- TestData (NetworkCSP refNet, NetworkCSP obsNet, std::shared_ptr< EdgeContT > remLinks, std::shared_ptr< EdgeContT > addLinks, std::shared_ptr< TestEdgeGen< Network, EdgeContT >> eg, LinkClass posClass, LinkClass negClass)
- void lock ()
- void genPos ()
- void genNeg ()
- TestData (TestData const &that)=default
- TestData & operator= (TestData const &that)=default
- TestData (TestData &&that)=default
- TestData & operator= (TestData &&that)=default
- auto getObsNet () const
- auto getRefNet () const
- auto posBegin () const
- auto posEnd () const
- auto negBegin () const
- auto negEnd () const
- auto posStrmBegin () const
- auto posStrmEnd () const
- auto negStrmBegin () const
- auto negStrmEnd () const
- std::size_t getNbPos () const
- std::size_t getNbNeg () const
- std::shared_ptr< std::set< Edge > const > getRemLinksMap () const
- LinkClass getNegClass () const
- LinkClass getPosClass () const
- bool isTnGenerated () const
- bool isTpGenerated () const
- bool isLocked () const
- const std::shared_ptr< TestEdgeGen< Network, EdgeContT > > & getEg () const
- virtual ~TestData ()=default

9.107.1 Detailed Description

template<typename Network = UNetwork<>, typename EdgeContT = std::vector< typename Network::Edge>>
class LinkPred::TestData< Network, EdgeContT >

Test data.

Template Parameters

Network	The network type.
EdgeContT	The container used to store edges.

9.107.2 Member Typedef Documentation

9.107.2.1 Edge

```
template<typename Network = UNetwork<>, typename EdgeContT = std::vector< typename Network↵
::Edge>>
using LinkPred::TestData< Network, EdgeContT >::Edge = typename Network::Edge
```

The edge type.

9.107.2.2 EdgeIt

```
template<typename Network = UNetwork<>, typename EdgeContT = std::vector< typename Network↵
::Edge>>
using LinkPred::TestData< Network, EdgeContT >::EdgeIt = typename Network::EdgeIt
```

Edge iterator type.

9.107.2.3 NetworkCSP

```
template<typename Network = UNetwork<>, typename EdgeContT = std::vector< typename Network↵
::Edge>>
using LinkPred::TestData< Network, EdgeContT >::NetworkCSP = std::shared_ptr<const Network>
```

Constant shared pointer to anetwork.

9.107.2.4 NetworkSP

```
template<typename Network = UNetwork<>, typename EdgeContT = std::vector< typename Network↵
::Edge>>
using LinkPred::TestData< Network, EdgeContT >::NetworkSP = std::shared_ptr<Network>
```

Shared pointer to a network.

9.107.2.5 NonEdgeIt

```
template<typename Network = UNetwork<>, typename EdgeContT = std::vector< typename Network↵
::Edge>>
using LinkPred::TestData< Network, EdgeContT >::NonEdgeIt = typename Network::NonEdgeIt
```

Negative edge iterator type.

9.107.3 Member Enumeration Documentation

9.107.3.1 IteratorType

```
template<typename Network = UNetwork<>, typename EdgeContT = std::vector< typename Network↵
::Edge>>
enum LinkPred::TestData::IteratorType
```

Enumeration of iterator types.

Enumerator

ECEIT	Edge container iterator.
TPEIT	True negative edge iterator.
TNEIT	True positive edge iterator.

9.107.4 Constructor & Destructor Documentation

9.107.4.1 TestData() [1/4]

```
template<typename Network = UNetwork<>, typename EdgeContT = std::vector< typename Network↩
::Edge>>
LinkPred::TestData< Network, EdgeContT >::TestData (
            NetworkCSP refNet,
            NetworkCSP obsNet,
            std::shared_ptr< EdgeContT > remLinks,
            std::shared_ptr< EdgeContT > addLinks,
            std::shared_ptr< EdgeContT > tpLinks,
            std::shared_ptr< EdgeContT > tnLinks,
            LinkClass posClass,
            LinkClass negClass )  [inline]
```

Parameters

refNet	The reference network.
obsNet	The network.
remLinks	Removed edges.
addLinks	Added edges.
tpLinks	True positive links.
tnLinks	True negative links.
posClass	The class of edges used as the positive instances in the test set.
negClass	The class of edges used as the negative instances in the test set.

9.107.4.2 TestData() [2/4]

```
template<typename Network = UNetwork<>, typename EdgeContT = std::vector< typename Network↩
::Edge>>
LinkPred::TestData< Network, EdgeContT >::TestData (
            NetworkCSP refNet,
            NetworkCSP obsNet,
            std::shared_ptr< EdgeContT > remLinks,
            std::shared_ptr< EdgeContT > addLinks,
            std::shared_ptr< TestEdgeGen< Network, EdgeContT >> eg,
            LinkClass posClass,
            LinkClass negClass )  [inline]
```

Parameters

refNet	The reference network.
obsNet	The network.
remLinks	Removed edges.
addLinks	Added edges.
eg	Edge generator.
posClass	The class of edges used as the positive instances in the test set.
negClass	The class of edges used as the negative instances in the test set.

eg->template generateTP(std::back_inserter(*tpLinks)); eg->template generateTN(std::back_inserter(*tnLinks));

tpGenerated = true; tnGenerated = true;

9.107.4.3 TestData() [3/4]

```
template<typename Network = UNetwork<>, typename EdgeContT = std::vector< typename Network←
::Edge>>
LinkPred::TestData< Network, EdgeContT >::TestData (
            TestData< Network, EdgeContT > const & that )  [default]
```

Copy constructor.

Parameters

that	The object to copy.

9.107.4.4 TestData() [4/4]

```
template<typename Network = UNetwork<>, typename EdgeContT = std::vector< typename Network←
::Edge>>
LinkPred::TestData< Network, EdgeContT >::TestData (
            TestData< Network, EdgeContT > && that )  [default]
```

Move constructor.

Parameters

that	The object to move.

9.107.4.5 ∼TestData()

```
template<typename Network = UNetwork<>, typename EdgeContT = std::vector< typename Network←
::Edge>>
virtual LinkPred::TestData< Network, EdgeContT >::∼TestData ( )  [virtual], [default]
```

Destructor.

9.107.5 Member Function Documentation

9.107.5.1 genNeg()

```
template<typename Network = UNetwork<>, typename EdgeContT = std::vector< typename Network↩
::Edge>>
void LinkPred::TestData< Network, EdgeContT >::genNeg ( ) [inline]
```

Generate negative instances.

9.107.5.2 genPos()

```
template<typename Network = UNetwork<>, typename EdgeContT = std::vector< typename Network↩
::Edge>>
void LinkPred::TestData< Network, EdgeContT >::genPos ( ) [inline]
```

Generate positive instances.

9.107.5.3 getEg()

```
template<typename Network = UNetwork<>, typename EdgeContT = std::vector< typename Network↩
::Edge>>
const std::shared_ptr<TestEdgeGen<Network, EdgeContT> >& LinkPred::TestData< Network, Edge↩
ContT >::getEg ( ) const [inline]
```

Returns

Edge generator.

9.107.5.4 getNbNeg()

```
template<typename Network = UNetwork<>, typename EdgeContT = std::vector< typename Network↩
::Edge>>
std::size_t LinkPred::TestData< Network, EdgeContT >::getNbNeg ( ) const [inline]
```

Returns

Number of negative links in the test set.

9.107.5.5 getNbPos()

```
template<typename Network = UNetwork<>, typename EdgeContT = std::vector< typename Network↩
::Edge>>
std::size_t LinkPred::TestData< Network, EdgeContT >::getNbPos ( ) const  [inline]
```

Returns

Number of positive links in the test set.

9.107.5.6 getNegClass()

```
template<typename Network = UNetwork<>, typename EdgeContT = std::vector< typename Network↩
::Edge>>
LinkClass LinkPred::TestData< Network, EdgeContT >::getNegClass ( ) const  [inline]
```

Returns

negClass.

9.107.5.7 getObsNet()

```
template<typename Network = UNetwork<>, typename EdgeContT = std::vector< typename Network↩
::Edge>>
auto LinkPred::TestData< Network, EdgeContT >::getObsNet ( ) const  [inline]
```

Returns

The observed network.

9.107.5.8 getPosClass()

```
template<typename Network = UNetwork<>, typename EdgeContT = std::vector< typename Network↩
::Edge>>
LinkClass LinkPred::TestData< Network, EdgeContT >::getPosClass ( ) const  [inline]
```

Returns

posClass.

9.107.5.9 getRefNet()

```
template<typename Network = UNetwork<>, typename EdgeContT = std::vector< typename Network↩
::Edge>>
auto LinkPred::TestData< Network, EdgeContT >::getRefNet ( ) const [inline]
```

Returns

The reference network.

9.107.5.10 getRemLinksMap()

```
template<typename Network = UNetwork<>, typename EdgeContT = std::vector< typename Network↩
::Edge>>
std::shared_ptr<std::set<Edge> const> LinkPred::TestData< Network, EdgeContT >::getRem↩
LinksMap ( ) const [inline]
```

Returns

The removed links in a set.

9.107.5.11 isLocked()

```
template<typename Network = UNetwork<>, typename EdgeContT = std::vector< typename Network↩
::Edge>>
bool LinkPred::TestData< Network, EdgeContT >::isLocked ( ) const [inline]
```

Returns

Whether the test data is locked.

9.107.5.12 isTnGenerated()

```
template<typename Network = UNetwork<>, typename EdgeContT = std::vector< typename Network↩
::Edge>>
bool LinkPred::TestData< Network, EdgeContT >::isTnGenerated ( ) const [inline]
```

Returns

tnGenerated.

9.107.5.13 isTpGenerated()

```
template<typename Network = UNetwork<>, typename EdgeContT = std::vector< typename Network↩
::Edge>>
bool LinkPred::TestData< Network, EdgeContT >::isTpGenerated ( ) const [inline]
```

Returns

tpGenerated.

9.107.5.14 lock()

```
template<typename Network = UNetwork<>, typename EdgeContT = std::vector< typename Network↩
::Edge>>
void LinkPred::TestData< Network, EdgeContT >::lock ( ) [inline]
```

Lock the dataset. No modifications can be done on the object after calling this method.

9.107.5.15 negBegin()

```
template<typename Network = UNetwork<>, typename EdgeContT = std::vector< typename Network↩
::Edge>>
auto LinkPred::TestData< Network, EdgeContT >::negBegin ( ) const [inline]
```

Returns

An iterator to the first negative link.

9.107.5.16 negEnd()

```
template<typename Network = UNetwork<>, typename EdgeContT = std::vector< typename Network↩
::Edge>>
auto LinkPred::TestData< Network, EdgeContT >::negEnd ( ) const [inline]
```

Returns

An iterator to one-past-the-last negative link.

9.107.5.17 negStrmBegin()

```
template<typename Network = UNetwork<>, typename EdgeContT = std::vector< typename Network↩
::Edge>>
auto LinkPred::TestData< Network, EdgeContT >::negStrmBegin ( ) const [inline]
```

Returns

An iterator to the first negative link. The edges here may be streamed and not pre-generated.

9.107.5.18 negStrmEnd()

```
template<typename Network = UNetwork<>, typename EdgeContT = std::vector< typename Network↩
::Edge>>
auto LinkPred::TestData< Network, EdgeContT >::negStrmEnd ( ) const [inline]
```

Returns

An iterator to one-past-the-last negative link. The edges here may be streamed and not pre-generated.

9.107.5.19 operator=() [1/2]

```
template<typename Network = UNetwork<>, typename EdgeContT = std::vector< typename Network↩
::Edge>>
TestData& LinkPred::TestData< Network, EdgeContT >::operator= (
            TestData< Network, EdgeContT > && that ) [default]
```

Move assignment operator.

Parameters

that	The object to move.

9.107.5.20 operator=() [2/2]

```
template<typename Network = UNetwork<>, typename EdgeContT = std::vector< typename Network↩
::Edge>>
TestData& LinkPred::TestData< Network, EdgeContT >::operator= (
            TestData< Network, EdgeContT > const & that ) [default]
```

Copy assignment operator.

Parameters

that	The object to copy.

9.107.5.21 posBegin()

```
template<typename Network = UNetwork<>, typename EdgeContT = std::vector< typename Network↵
::Edge>>
auto LinkPred::TestData< Network, EdgeContT >::posBegin ( ) const [inline]
```

Returns

An iterator to the first positive link.

9.107.5.22 posEnd()

```
template<typename Network = UNetwork<>, typename EdgeContT = std::vector< typename Network↵
::Edge>>
auto LinkPred::TestData< Network, EdgeContT >::posEnd ( ) const [inline]
```

Returns

An iterator to one-past-the-last positive link.

9.107.5.23 posStrmBegin()

```
template<typename Network = UNetwork<>, typename EdgeContT = std::vector< typename Network↵
::Edge>>
auto LinkPred::TestData< Network, EdgeContT >::posStrmBegin ( ) const [inline]
```

Returns

An iterator to the first positive link. The edges here may be streamed and not pre-generated.

9.107.5.24 posStrmEnd()

```
template<typename Network = UNetwork<>, typename EdgeContT = std::vector< typename Network↩
::Edge>>
auto LinkPred::TestData< Network, EdgeContT >::posStrmEnd ( ) const [inline]
```

Returns

An iterator to one-past-the-last positive link. The edges here may be streamed and not pre-generated.

The documentation for this class was generated from the following file:

- include/linkpred/perf/networkmanipulator.hpp

9.108 LinkPred::TestEdgeGen< Network, EdgeContT > Class Template Reference

Generate true positives and true negatives.

```
#include <networkmanipulator.hpp>
```

Classes

- class TNEdgeIt
  TN edges iterator.
- class TPEdgeIt
  TP edges iterator.

Public Member Functions

- TestEdgeGen (NetworkCSP refNet, NetworkCSP obsNet, std::shared_ptr< EdgeContT > remLinks, std↩ ::shared_ptr< EdgeContT > addLinks, bool aTP, double tpRatio, bool aTN, double tnRatio, long int seed)
- TestEdgeGen (TestEdgeGen const &that)=default
- TestEdgeGen & operator= (TestEdgeGen const &that)=default
- TestEdgeGen (TestEdgeGen &&that)=default
- TestEdgeGen & operator= (TestEdgeGen &&that)=default
- template<typename OutputEdgeItT = typename std::vector< typename Network::Edge>::iterator>
  void generateTP (OutputEdgeItT oit)
- template<typename OutputEdgeItT = typename std::vector< typename Network::Edge>::iterator>
  void generateTN (OutputEdgeItT oit)
- auto tpBegin () const
- auto tpEnd () const
- auto tnBegin () const
- auto tnEnd () const
- virtual ~TestEdgeGen ()=default

9.108.1 Detailed Description

template<typename Network = UNetwork<>, typename EdgeContT = std::vector< typename Network::Edge>>
class LinkPred::TestEdgeGen< Network, EdgeContT >

Generate true positives and true negatives.

Template Parameters

Network	Network type.
EdgeContT	The container used to store edges.

9.108.2 Constructor & Destructor Documentation

9.108.2.1 TestEdgeGen() [1/3]

```
template<typename Network = UNetwork<>, typename EdgeContT = std::vector< typename Network↩
::Edge>>
LinkPred::TestEdgeGen< Network, EdgeContT >::TestEdgeGen (
            NetworkCSP refNet,
            NetworkCSP obsNet,
            std::shared_ptr< EdgeContT > remLinks,
            std::shared_ptr< EdgeContT > addLinks,
            bool aTP,
            double tpRatio,
            bool aTN,
            double tnRatio,
            long int seed )  [inline]
```

Parameters

refNet	The reference network.
obsNet	The network.
remLinks	Removed edges.
addLinks	Added edges.
aTP	Whether to use all true positive links in the test set.
tpRatio	Ratio of true positive inks to e used in the test set. This parameter is only relevant when aTP is false.
aTN	Whether to use all true negative links in the test set.
tnRatio	Ratio of true negative inks to e used in the test set. This parameter is only relevant when aTN is false.
seed	The random number generator's seed.

9.108.2.2 TestEdgeGen() [2/3]

```
template<typename Network = UNetwork<>, typename EdgeContT = std::vector< typename Network↩
::Edge>>
LinkPred::TestEdgeGen< Network, EdgeContT >::TestEdgeGen (
            TestEdgeGen< Network, EdgeContT > const & that )  [default]
```

Copy constructor.

Parameters

that	The object to copy.

9.108.2.3 TestEdgeGen() [3/3]

```
template<typename Network = UNetwork<>, typename EdgeContT = std::vector< typename Network↩
::Edge>>
LinkPred::TestEdgeGen< Network, EdgeContT >::TestEdgeGen (
            TestEdgeGen< Network, EdgeContT > && that )  [default]
```

Move constructor.

Parameters

that	The object to move.

9.108.2.4 ∼TestEdgeGen()

```
template<typename Network = UNetwork<>, typename EdgeContT = std::vector< typename Network↩
::Edge>>
virtual LinkPred::TestEdgeGen< Network, EdgeContT >::∼TestEdgeGen ( )  [virtual], [default]
```

Destructor.

9.108.3 Member Function Documentation

9.108.3.1 generateTN()

```
template<typename Network = UNetwork<>, typename EdgeContT = std::vector< typename Network↩
::Edge>>
template<typename OutputEdgeItT = typename std::vector< typename Network::Edge>::iterator>
void LinkPred::TestEdgeGen< Network, EdgeContT >::generateTN (
            OutputEdgeItT oit )  [inline]
```

Generate true negative links.

Template Parameters

OutputEdgeItT	Iterator write edges.

Parameters

oit	Output edge iterator.

9.108.3.2 generateTP()

```
template<typename Network = UNetwork<>, typename EdgeContT = std::vector< typename Network↩
::Edge>>
template<typename OutputEdgeItT = typename std::vector< typename Network::Edge>::iterator>
void LinkPred::TestEdgeGen< Network, EdgeContT >::generateTP (
            OutputEdgeItT oit )  [inline]
```

Generate true positive links.

Template Parameters

OutputEdgeItT	Iterator write edges.

Parameters

oit	Output edge iterator.

9.108.3.3 operator=() [1/2]

```
template<typename Network = UNetwork<>, typename EdgeContT = std::vector< typename Network↩
::Edge>>
TestEdgeGen& LinkPred::TestEdgeGen< Network, EdgeContT >::operator= (
            TestEdgeGen< Network, EdgeContT > && that )  [default]
```

Move assignment operator.

Parameters

that	The object to move.

9.108.3.4 operator=() [2/2]

```
template<typename Network = UNetwork<>, typename EdgeContT = std::vector< typename Network↩
::Edge>>
TestEdgeGen& LinkPred::TestEdgeGen< Network, EdgeContT >::operator= (
            TestEdgeGen< Network, EdgeContT > const & that )  [default]
```

Copy assignment operator.

Parameters

that	The object to copy.

9.108.3.5 tnBegin()

```
template<typename Network = UNetwork<>, typename EdgeContT = std::vector< typename Network↩
::Edge>>
auto LinkPred::TestEdgeGen< Network, EdgeContT >::tnBegin ( ) const  [inline]
```

Returns

Iterator to the first true positive link.

9.108.3.6 tnEnd()

```
template<typename Network = UNetwork<>, typename EdgeContT = std::vector< typename Network↩
::Edge>>
auto LinkPred::TestEdgeGen< Network, EdgeContT >::tnEnd ( ) const  [inline]
```

Returns

Iterator to one-past the last true positive link.

9.108.3.7 tpBegin()

```
template<typename Network = UNetwork<>, typename EdgeContT = std::vector< typename Network↩
::Edge>>
auto LinkPred::TestEdgeGen< Network, EdgeContT >::tpBegin ( ) const  [inline]
```

Returns

Iterator to the first true positive link.

9.108.3.8 tpEnd()

```
template<typename Network = UNetwork<>, typename EdgeContT = std::vector< typename Network↩
::Edge>>
auto LinkPred::TestEdgeGen< Network, EdgeContT >::tpEnd ( ) const  [inline]
```

Returns

Iterator to one-past the last true positive link.

The documentation for this class was generated from the following file:

- include/linkpred/perf/networkmanipulator.hpp

9.109 LinkPred::TestData< Network, EdgeContT >::TestEdgeIt Class Reference

Test edges iterator.

```
#include <networkmanipulator.hpp>
```

Inheritance diagram for LinkPred::TestData< Network, EdgeContT >::TestEdgeIt:

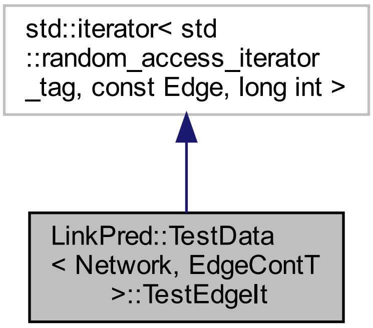

Collaboration diagram for LinkPred::TestData< Network, EdgeContT >::TestEdgeIt:

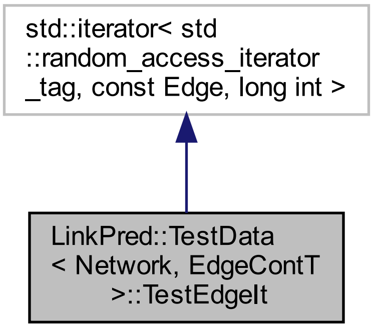

Public Types

- using pointer = typename std::iterator< std::random_access_iterator_tag, const Edge, long int >::pointer
- using reference = typename std::iterator< std::random_access_iterator_tag, const Edge, long int >↩ ::reference
- using difference_type = typename std::iterator< std::random_access_iterator_tag, const Edge, long int >↩ ::difference_type

Public Member Functions

- TestEdgeIt (IteratorType const &itType, typename EdgeContT::const_iterator const &eceit, typename TestEdgeGen< Network, EdgeContT >::TPEdgeIt const &tpeit, typename TestEdgeGen< Network, Edge↩ ContT >::TNEdgeIt const &tneit)
- TestEdgeIt (TestEdgeIt const &that)=default
- TestEdgeIt & operator= (TestEdgeIt const &that)=default
- TestEdgeIt (TestEdgeIt &&that)=default
- TestEdgeIt & operator= (TestEdgeIt &&that)=default
- reference operator∗ ()
- pointer operator-> ()
- TestEdgeIt & operator++ ()
- TestEdgeIt & operator-- ()
- TestEdgeIt operator++ (int)
- TestEdgeIt operator-- (int)
- TestEdgeIt operator+ (const difference_type &n) const
- TestEdgeIt & operator+= (const difference_type &n)
- TestEdgeIt operator- (const difference_type &n) const
- TestEdgeIt & operator-= (const difference_type &n)
- difference_type operator- (const TestEdgeIt &that) const
- bool operator== (const TestEdgeIt &that) const
- bool operator!= (const TestEdgeIt &that) const
- bool operator< (const TestEdgeIt &that) const
- bool operator> (const TestEdgeIt &that) const
- bool operator<= (const TestEdgeIt &that) const
- bool operator>= (const TestEdgeIt &that) const

Friends

- class TestData

9.109.1 Detailed Description

template<typename Network = UNetwork<>, typename EdgeContT = std::vector< typename Network::Edge>>
class LinkPred::TestData< Network, EdgeContT >::TestEdgeIt

Test edges iterator.

9.109.2 Member Typedef Documentation

9.109.2.1 difference_type

```
template<typename Network = UNetwork<>, typename EdgeContT = std::vector< typename Network↩
::Edge>>
using LinkPred::TestData< Network, EdgeContT >::TestEdgeIt::difference_type = typename std↩
::iterator<std::random_access_iterator_tag, const Edge, long int>::difference_type
```

The difference type associated with the iterator.

9.109.2.2 pointer

```
template<typename Network = UNetwork<>, typename EdgeContT = std::vector< typename Network↩
::Edge>>
using LinkPred::TestData< Network, EdgeContT >::TestEdgeIt::pointer = typename std::iterator<std↩
::random_access_iterator_tag, const Edge, long int>::pointer
```

The pointer type associated with the iterator.

9.109.2.3 reference

```
template<typename Network = UNetwork<>, typename EdgeContT = std::vector< typename Network↩
::Edge>>
using LinkPred::TestData< Network, EdgeContT >::TestEdgeIt::reference = typename std::iterator<std↩
::random_access_iterator_tag, const Edge, long int>::reference
```

The reference type associated with the iterator.

9.109.3 Constructor & Destructor Documentation

9.109.3.1 TestEdgeIt() [1/3]

```
template<typename Network = UNetwork<>, typename EdgeContT = std::vector< typename Network↩
::Edge>>
LinkPred::TestData< Network, EdgeContT >::TestEdgeIt::TestEdgeIt (
             IteratorType const & itType,
             typename EdgeContT::const_iterator const & eceit,
             typename TestEdgeGen< Network, EdgeContT >::TPEdgeIt const & tpeit,
             typename TestEdgeGen< Network, EdgeContT >::TNEdgeIt const & tneit )  [inline]
```

Constructor.

Parameters

itType	The iterator type.
eceit	Edge container iterator.
tpeit	True positive edge iterator.
tneit	True negative edge iterator.

9.109.3.2 TestEdgeIt() [2/3]

```
template<typename Network = UNetwork<>, typename EdgeContT = std::vector< typename Network↩
::Edge>>
LinkPred::TestData< Network, EdgeContT >::TestEdgeIt::TestEdgeIt (
            TestEdgeIt const & that )  [default]
```

Copy constructor.

Parameters

that	The object to copy.

9.109.3.3 TestEdgeIt() [3/3]

```
template<typename Network = UNetwork<>, typename EdgeContT = std::vector< typename Network↩
::Edge>>
LinkPred::TestData< Network, EdgeContT >::TestEdgeIt::TestEdgeIt (
            TestEdgeIt && that )  [default]
```

Move constructor.

Parameters

that	The object to move.

9.109.4 Member Function Documentation

9.109.4.1 operator"!=()

```
template<typename Network = UNetwork<>, typename EdgeContT = std::vector< typename Network↩
::Edge>>
bool LinkPred::TestData< Network, EdgeContT >::TestEdgeIt::operator!= (
            const TestEdgeIt & that ) const  [inline]
```

Parameters

that	The other iterator.

Returns

True if this is not equal to that.

9.109.4.2 operator*()

```
template<typename Network = UNetwork<>, typename EdgeContT = std::vector< typename Network↩
::Edge>>
reference LinkPred::TestData< Network, EdgeContT >::TestEdgeIt::operator* ( ) [inline]
```

Dereference operator.

Returns

A reference to the object to which the iterator points.

9.109.4.3 operator+()

```
template<typename Network = UNetwork<>, typename EdgeContT = std::vector< typename Network↩
::Edge>>
TestEdgeIt LinkPred::TestData< Network, EdgeContT >::TestEdgeIt::operator+ (
            const difference_type & n ) const [inline]
```

Arithmetic + operator.

Parameters

n	Increment value.

Returns

The new iterator.

9.109.4.4 operator++() [1/2]

```
template<typename Network = UNetwork<>, typename EdgeContT = std::vector< typename Network↩
::Edge>>
TestEdgeIt& LinkPred::TestData< Network, EdgeContT >::TestEdgeIt::operator++ ( ) [inline]
```

Pre-increment operator.

Returns

A reference to the new iterator.

9.109.4.5 operator++() [2/2]

```
template<typename Network = UNetwork<>, typename EdgeContT = std::vector< typename Network↩
::Edge>>
TestEdgeIt LinkPred::TestData< Network, EdgeContT >::TestEdgeIt::operator++ (
            int ) [inline]
```

Post-increment operator.

Returns

A reference to the new iterator.

9.109.4.6 operator+=()

```
template<typename Network = UNetwork<>, typename EdgeContT = std::vector< typename Network↩
::Edge>>
TestEdgeIt& LinkPred::TestData< Network, EdgeContT >::TestEdgeIt::operator+= (
            const difference_type & n ) [inline]
```

Arithmetic += operator.

Parameters

n	Increment value.

Returns

A reference to the new iterator.

9.109.4.7 operator-() [1/2]

```
template<typename Network = UNetwork<>, typename EdgeContT = std::vector< typename Network↩
::Edge>>
TestEdgeIt LinkPred::TestData< Network, EdgeContT >::TestEdgeIt::operator- (
            const difference_type & n ) const [inline]
```

Arithmetic - operator.

Parameters

n	Decrement value.

Returns

The new iterator.

9.109.4.8 operator-() [2/2]

```
template<typename Network = UNetwork<>, typename EdgeContT = std::vector< typename Network↩
::Edge>>
difference_type LinkPred::TestData< Network, EdgeContT >::TestEdgeIt::operator- (
            const TestEdgeIt & that ) const [inline]
```

Difference between the present iterator and the one passed as parameter.

Parameters

that	The other iterator.

Returns

The difference between the current and that iterator.

9.109.4.9 operator--() [1/2]

```
template<typename Network = UNetwork<>, typename EdgeContT = std::vector< typename Network↩
::Edge>>
TestEdgeIt& LinkPred::TestData< Network, EdgeContT >::TestEdgeIt::operator-- ( ) [inline]
```

Pre-decrement operator.

Returns

A reference to the new iterator.

9.109.4.10 operator--() [2/2]

```
template<typename Network = UNetwork<>, typename EdgeContT = std::vector< typename Network↩
::Edge>>
TestEdgeIt LinkPred::TestData< Network, EdgeContT >::TestEdgeIt::operator-- (
            int ) [inline]
```

Post-decrement operator.

Returns

A reference to the new iterator.

9.109.4.11 operator-=()

```
template<typename Network = UNetwork<>, typename EdgeContT = std::vector< typename Network↩
::Edge>>
TestEdgeIt& LinkPred::TestData< Network, EdgeContT >::TestEdgeIt::operator-= (
            const difference_type & n ) [inline]
```

Arithmetic -= operator.

Parameters

n	Decrement value.

Returns

A reference to the new iterator.

9.109.4.12 operator->()

```
template<typename Network = UNetwork<>, typename EdgeContT = std::vector< typename Network↩
::Edge>>
pointer LinkPred::TestData< Network, EdgeContT >::TestEdgeIt::operator-> ( ) [inline]
```

Arrow operator.

Returns

A pointer to the object to which the iterator points.

9.109.4.13 operator<()

```
template<typename Network = UNetwork<>, typename EdgeContT = std::vector< typename Network↩
::Edge>>
bool LinkPred::TestData< Network, EdgeContT >::TestEdgeIt::operator< (
            const TestEdgeIt & that ) const [inline]
```

Parameters

that	The other iterator.

Returns

True if this is less than that.

9.109.4.14 operator<=()

```
template<typename Network = UNetwork<>, typename EdgeContT = std::vector< typename Network↩
::Edge>>
bool LinkPred::TestData< Network, EdgeContT >::TestEdgeIt::operator<= (
            const TestEdgeIt & that ) const  [inline]
```

Parameters

that	The other iterator.

Returns

True if this is greater or equal to that.

9.109.4.15 operator=() [1/2]

```
template<typename Network = UNetwork<>, typename EdgeContT = std::vector< typename Network↩
::Edge>>
TestEdgeIt& LinkPred::TestData< Network, EdgeContT >::TestEdgeIt::operator= (
            TestEdgeIt && that )  [default]
```

Move assignment operator.

Parameters

that	The object to move.

9.109.4.16 operator=() [2/2]

```
template<typename Network = UNetwork<>, typename EdgeContT = std::vector< typename Network↩
::Edge>>
TestEdgeIt& LinkPred::TestData< Network, EdgeContT >::TestEdgeIt::operator= (
            TestEdgeIt const & that )  [default]
```

Copy assignment operator.

Parameters

that	The object to copy.

9.109.4.17 operator==()

```
template<typename Network = UNetwork<>, typename EdgeContT = std::vector< typename Network↩
::Edge>>
bool LinkPred::TestData< Network, EdgeContT >::TestEdgeIt::operator== (
            const TestEdgeIt & that ) const  [inline]
```

Parameters

that	The other iterator.

Returns

True if this equals that.

9.109.4.18 operator>()

```
template<typename Network = UNetwork<>, typename EdgeContT = std::vector< typename Network↩
::Edge>>
bool LinkPred::TestData< Network, EdgeContT >::TestEdgeIt::operator> (
            const TestEdgeIt & that ) const  [inline]
```

Parameters

that	The other iterator.

Returns

True if this is greater that.

9.109.4.19 operator>=()

```
template<typename Network = UNetwork<>, typename EdgeContT = std::vector< typename Network↩
::Edge>>
bool LinkPred::TestData< Network, EdgeContT >::TestEdgeIt::operator>= (
            const TestEdgeIt & that ) const  [inline]
```

Parameters

that	The other iterator.

Returns

True if this is less or equal to that.

9.109.5 Friends And Related Function Documentation

9.109.5.1 TestData

```
template<typename Network = UNetwork<>, typename EdgeContT = std::vector< typename Network↵
::Edge>>
friend class TestData  [friend]
```

TestData is a friend.

The documentation for this class was generated from the following file:

- include/linkpred/perf/networkmanipulator.hpp

9.110 LinkPred::TestEdgeGen< Network, EdgeContT >::TNEdgeIt Class Reference

TN edges iterator.

```
#include <networkmanipulator.hpp>
```

Inheritance diagram for LinkPred::TestEdgeGen< Network, EdgeContT >::TNEdgeIt:

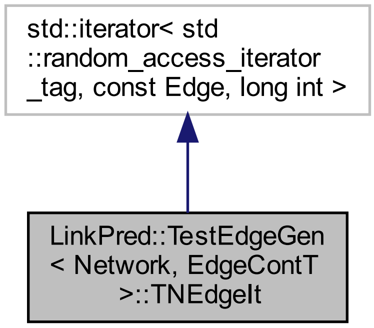

Collaboration diagram for LinkPred::TestEdgeGen< Network, EdgeContT >::TNEdgeIt:

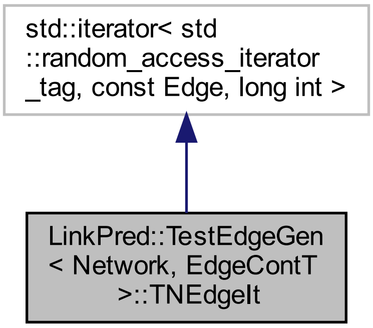

Public Types

- using pointer = typename std::iterator< std::random_access_iterator_tag, const Edge, long int >::pointer
- using reference = typename std::iterator< std::random_access_iterator_tag, const Edge, long int >↩ ::reference
- using difference_type = typename std::iterator< std::random_access_iterator_tag, const Edge, long int >↩ ::difference_type

Public Member Functions

- TNEdgeIt (TNEdgeIt const &that)=default
- TNEdgeIt & operator= (TNEdgeIt const &that)=default
- TNEdgeIt (TNEdgeIt &&that)=default
- TNEdgeIt & operator= (TNEdgeIt &&that)=default
- reference operator* ()
- pointer operator-> ()
- TNEdgeIt & operator++ ()
- TNEdgeIt & operator-- ()
- TNEdgeIt operator++ (int)
- TNEdgeIt operator-- (int)
- TNEdgeIt operator+ (const difference_type &n) const
- TNEdgeIt & operator+= (const difference_type &n)
- TNEdgeIt operator- (const difference_type &n) const
- TNEdgeIt & operator-= (const difference_type &n)
- difference_type operator- (const TNEdgeIt &that) const
- bool operator== (const TNEdgeIt &that) const
- bool operator!= (const TNEdgeIt &that) const
- bool operator< (const TNEdgeIt &that) const
- bool operator> (const TNEdgeIt &that) const
- bool operator<= (const TNEdgeIt &that) const
- bool operator>= (const TNEdgeIt &that) const

Friends

- class TestEdgeGen

9.110.1 Detailed Description

**template<typename Network = UNetwork<>, typename EdgeContT = std::vector< typename Network::Edge>>
class LinkPred::TestEdgeGen< Network, EdgeContT >::TNEdgeIt**

TN edges iterator.

9.110.2 Member Typedef Documentation

9.110.2.1 difference_type

```
template<typename Network = UNetwork<>, typename EdgeContT = std::vector< typename Network↩
::Edge>>
using LinkPred::TestEdgeGen< Network, EdgeContT >::TNEdgeIt::difference_type = typename std↩
::iterator<std::random_access_iterator_tag, const Edge, long int>::difference_type
```

The difference type associated with the iterator.

9.110.2.2 pointer

```
template<typename Network = UNetwork<>, typename EdgeContT = std::vector< typename Network↩
::Edge>>
using LinkPred::TestEdgeGen< Network, EdgeContT >::TNEdgeIt::pointer = typename std::iterator<std↩
::random_access_iterator_tag, const Edge, long int>::pointer
```

The pointer type associated with the iterator.

9.110.2.3 reference

```
template<typename Network = UNetwork<>, typename EdgeContT = std::vector< typename Network↩
::Edge>>
using LinkPred::TestEdgeGen< Network, EdgeContT >::TNEdgeIt::reference = typename std::iterator<std↩
::random_access_iterator_tag, const Edge, long int>::reference
```

The reference type associated with the iterator.

9.110.3 Constructor & Destructor Documentation

9.110.3.1 TNEdgeIt() [1/2]

```
template<typename Network = UNetwork<>, typename EdgeContT = std::vector< typename Network↩
::Edge>>
LinkPred::TestEdgeGen< Network, EdgeContT >::TNEdgeIt::TNEdgeIt (
            TNEdgeIt const & that ) [default]
```

Copy constructor.

Parameters

that	The object to copy.

9.110.3.2 TNEdgeIt() [2/2]

```
template<typename Network = UNetwork<>, typename EdgeContT = std::vector< typename Network↩
::Edge>>
LinkPred::TestEdgeGen< Network, EdgeContT >::TNEdgeIt::TNEdgeIt (
            TNEdgeIt && that ) [default]
```

Move constructor.

Parameters

that	The object to move.

9.110.4 Member Function Documentation

9.110.4.1 operator"!=()

```
template<typename Network = UNetwork<>, typename EdgeContT = std::vector< typename Network↩
::Edge>>
bool LinkPred::TestEdgeGen< Network, EdgeContT >::TNEdgeIt::operator!= (
            const TNEdgeIt & that ) const [inline]
```

Parameters

that	The other iterator.

Returns

True if this is not equal to that.

9.110.4.2 operator∗()

```
template<typename Network = UNetwork<>, typename EdgeContT = std::vector< typename Network↩
::Edge>>
reference LinkPred::TestEdgeGen< Network, EdgeContT >::TNEdgeIt::operator* ( ) [inline]
```

Dereference operator.

Returns

A reference to the object to which the iterator points.

9.110.4.3 operator+()

```
template<typename Network = UNetwork<>, typename EdgeContT = std::vector< typename Network↩
::Edge>>
TNEdgeIt LinkPred::TestEdgeGen< Network, EdgeContT >::TNEdgeIt::operator+ (
            const difference_type & n ) const  [inline]
```

Arithmetic + operator.

Parameters

n	Increment value.

Returns

The new iterator.

9.110.4.4 operator++() [1/2]

```
template<typename Network = UNetwork<>, typename EdgeContT = std::vector< typename Network↩
::Edge>>
TNEdgeIt& LinkPred::TestEdgeGen< Network, EdgeContT >::TNEdgeIt::operator++ ( )  [inline]
```

Pre-increment operator.

Returns

A reference to the new iterator.

9.110.4.5 operator++() [2/2]

```
template<typename Network = UNetwork<>, typename EdgeContT = std::vector< typename Network↩
::Edge>>
TNEdgeIt LinkPred::TestEdgeGen< Network, EdgeContT >::TNEdgeIt::operator++ (
            int  )  [inline]
```

Post-increment operator.

Returns

A reference to the new iterator.

9.110.4.6 operator+=()

```
template<typename Network = UNetwork<>, typename EdgeContT = std::vector< typename Network↩
::Edge>>
TNEdgeIt& LinkPred::TestEdgeGen< Network, EdgeContT >::TNEdgeIt::operator+= (
            const difference_type & n )  [inline]
```

Arithmetic += operator.

Parameters

n	Increment value.

Returns

A reference to the new iterator.

9.110.4.7 operator-() [1/2]

```
template<typename Network = UNetwork<>, typename EdgeContT = std::vector< typename Network↩
::Edge>>
TNEdgeIt LinkPred::TestEdgeGen< Network, EdgeContT >::TNEdgeIt::operator- (
            const difference_type & n ) const  [inline]
```

Arithmetic - operator.

Parameters

n	Decrement value.

Returns

The new iterator.

9.110.4.8 operator-() [2/2]

```
template<typename Network = UNetwork<>, typename EdgeContT = std::vector< typename Network↩
::Edge>>
difference_type LinkPred::TestEdgeGen< Network, EdgeContT >::TNEdgeIt::operator- (
            const TNEdgeIt & that ) const  [inline]
```

Difference between the present iterator and the one passed as parameter.

Parameters

that	The other iterator.

Returns

The difference between the current and that iterator.

9.110.4.9 operator--() [1/2]

```
template<typename Network = UNetwork<>, typename EdgeContT = std::vector< typename Network↩
::Edge>>
TNEdgeIt& LinkPred::TestEdgeGen< Network, EdgeContT >::TNEdgeIt::operator-- ( ) [inline]
```

Pre-decrement operator.

Returns

A reference to the new iterator.

9.110.4.10 operator--() [2/2]

```
template<typename Network = UNetwork<>, typename EdgeContT = std::vector< typename Network↩
::Edge>>
TNEdgeIt LinkPred::TestEdgeGen< Network, EdgeContT >::TNEdgeIt::operator-- (
            int ) [inline]
```

Post-decrement operator.

Returns

A reference to the new iterator.

9.110.4.11 operator-=()

```
template<typename Network = UNetwork<>, typename EdgeContT = std::vector< typename Network↩
::Edge>>
TNEdgeIt& LinkPred::TestEdgeGen< Network, EdgeContT >::TNEdgeIt::operator-= (
            const difference_type & n ) [inline]
```

Arithmetic -= operator.

Parameters

n	Decrement value.

Returns

A reference to the new iterator.

9.110.4.12 operator->()

```
template<typename Network = UNetwork<>, typename EdgeContT = std::vector< typename Network↩
::Edge>>
pointer LinkPred::TestEdgeGen< Network, EdgeContT >::TNEdgeIt::operator-> ( ) [inline]
```

Arrow operator.

Returns

A pointer to the object to which the iterator points.

9.110.4.13 operator<()

```
template<typename Network = UNetwork<>, typename EdgeContT = std::vector< typename Network↩
::Edge>>
bool LinkPred::TestEdgeGen< Network, EdgeContT >::TNEdgeIt::operator< (
            const TNEdgeIt & that ) const [inline]
```

Parameters

that	The other iterator.

Returns

True if this is less than that.

9.110.4.14 operator<=()

```
template<typename Network = UNetwork<>, typename EdgeContT = std::vector< typename Network↩
::Edge>>
bool LinkPred::TestEdgeGen< Network, EdgeContT >::TNEdgeIt::operator<= (
            const TNEdgeIt & that ) const [inline]
```

Parameters

that	The other iterator.

Returns

True if this is greater or equal to that.

9.110.4.15 operator=() [1/2]

```
template<typename Network = UNetwork<>, typename EdgeContT = std::vector< typename Network↩
::Edge>>
TNEdgeIt& LinkPred::TestEdgeGen< Network, EdgeContT >::TNEdgeIt::operator= (
            TNEdgeIt && that ) [default]
```

Move assignment operator.

Parameters

that	The object to move.

9.110.4.16 operator=() [2/2]

```
template<typename Network = UNetwork<>, typename EdgeContT = std::vector< typename Network↩
::Edge>>
TNEdgeIt& LinkPred::TestEdgeGen< Network, EdgeContT >::TNEdgeIt::operator= (
            TNEdgeIt const & that ) [default]
```

Copy assignment operator.

Parameters

that	The object to copy.

9.110.4.17 operator==()

```
template<typename Network = UNetwork<>, typename EdgeContT = std::vector< typename Network↩
::Edge>>
bool LinkPred::TestEdgeGen< Network, EdgeContT >::TNEdgeIt::operator== (
            const TNEdgeIt & that ) const [inline]
```

Parameters

that	The other iterator.

Returns

True if this equals that.

9.110.4.18 operator>()

```
template<typename Network = UNetwork<>, typename EdgeContT = std::vector< typename Network↩
::Edge>>
bool LinkPred::TestEdgeGen< Network, EdgeContT >::TNEdgeIt::operator> (
            const TNEdgeIt & that ) const  [inline]
```

Parameters

that	The other iterator.

Returns

True if this is greater that.

9.110.4.19 operator>=()

```
template<typename Network = UNetwork<>, typename EdgeContT = std::vector< typename Network↩
::Edge>>
bool LinkPred::TestEdgeGen< Network, EdgeContT >::TNEdgeIt::operator>= (
            const TNEdgeIt & that ) const  [inline]
```

Parameters

that	The other iterator.

Returns

True if this is less or equal to that.

9.110.5 Friends And Related Function Documentation

9.110.5.1 TestEdgeGen

```
template<typename Network = UNetwork<>, typename EdgeContT = std::vector< typename Network↩
::Edge>>
friend class TestEdgeGen  [friend]
```

TestEdgeGen is a friend.

The documentation for this class was generated from the following file:

- include/linkpred/perf/networkmanipulator.hpp

9.111 LinkPred::TestEdgeGen< Network, EdgeContT >::TPEdgeIt Class Reference

TP edges iterator.

```
#include <networkmanipulator.hpp>
```

Inheritance diagram for LinkPred::TestEdgeGen< Network, EdgeContT >::TPEdgeIt:

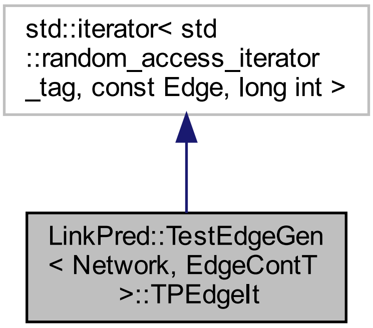

Collaboration diagram for LinkPred::TestEdgeGen< Network, EdgeContT >::TPEdgeIt:

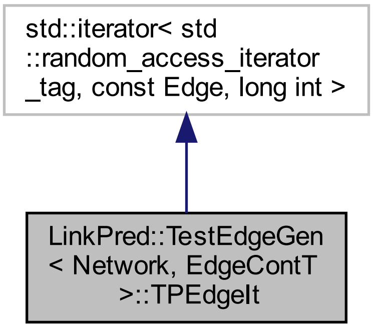

Public Types

- using pointer = typename std::iterator< std::random_access_iterator_tag, const Edge, long int >::pointer
- using reference = typename std::iterator< std::random_access_iterator_tag, const Edge, long int >↩ ::reference
- using difference_type = typename std::iterator< std::random_access_iterator_tag, const Edge, long int >↩ ::difference_type

Public Member Functions

- TPEdgeIt (TPEdgeIt const &that)=default
- TPEdgeIt & operator= (TPEdgeIt const &that)=default
- TPEdgeIt (TPEdgeIt &&that)=default
- TPEdgeIt & operator= (TPEdgeIt &&that)=default
- reference operator* ()
- pointer operator-> ()
- TPEdgeIt & operator++ ()
- TPEdgeIt & operator-- ()
- TPEdgeIt operator++ (int)
- TPEdgeIt operator-- (int)
- TPEdgeIt operator+ (const difference_type &n) const
- TPEdgeIt & operator+= (const difference_type &n)
- TPEdgeIt operator- (const difference_type &n) const
- TPEdgeIt & operator-= (const difference_type &n)
- difference_type operator- (const TPEdgeIt &that) const
- bool operator== (const TPEdgeIt &that) const
- bool operator!= (const TPEdgeIt &that) const
- bool operator< (const TPEdgeIt &that) const
- bool operator> (const TPEdgeIt &that) const
- bool operator<= (const TPEdgeIt &that) const
- bool operator>= (const TPEdgeIt &that) const

Friends

- class TestEdgeGen

9.111.1 Detailed Description

**template<typename Network = UNetwork<>, typename EdgeContT = std::vector< typename Network::Edge>>
class LinkPred::TestEdgeGen< Network, EdgeContT >::TPEdgeIt**

TP edges iterator.

9.111.2 Member Typedef Documentation

9.111.2.1 difference_type

```
template<typename Network = UNetwork<>, typename EdgeContT = std::vector< typename Network↩
::Edge>>
using LinkPred::TestEdgeGen< Network, EdgeContT >::TPEdgeIt::difference_type = typename std↩
::iterator<std::random_access_iterator_tag, const Edge, long int>::difference_type
```

The difference type associated with the iterator.

9.111.2.2 pointer

```
template<typename Network = UNetwork<>, typename EdgeContT = std::vector< typename Network↩
::Edge>>
using LinkPred::TestEdgeGen< Network, EdgeContT >::TPEdgeIt::pointer = typename std::iterator<std↩
::random_access_iterator_tag, const Edge, long int>::pointer
```

The pointer type associated with the iterator.

9.111.2.3 reference

```
template<typename Network = UNetwork<>, typename EdgeContT = std::vector< typename Network↩
::Edge>>
using LinkPred::TestEdgeGen< Network, EdgeContT >::TPEdgeIt::reference = typename std::iterator<std↩
::random_access_iterator_tag, const Edge, long int>::reference
```

The reference type associated with the iterator.

9.111.3 Constructor & Destructor Documentation

9.111.3.1 TPEdgeIt() [1/2]

```
template<typename Network = UNetwork<>, typename EdgeContT = std::vector< typename Network↩
::Edge>>
LinkPred::TestEdgeGen< Network, EdgeContT >::TPEdgeIt::TPEdgeIt (
            TPEdgeIt const & that ) [default]
```

Copy constructor.

Parameters

that	The object to copy.

9.111.3.2 TPEdgeIt() [2/2]

```
template<typename Network = UNetwork<>, typename EdgeContT = std::vector< typename Network↩
::Edge>>
LinkPred::TestEdgeGen< Network, EdgeContT >::TPEdgeIt::TPEdgeIt (
            TPEdgeIt && that ) [default]
```

Move constructor.

Parameters

that	The object to move.

9.111.4 Member Function Documentation

9.111.4.1 operator"!=()

```
template<typename Network = UNetwork<>, typename EdgeContT = std::vector< typename Network↵
::Edge>>
bool LinkPred::TestEdgeGen< Network, EdgeContT >::TPEdgeIt::operator!= (
            const TPEdgeIt & that ) const  [inline]
```

Parameters

that	The other iterator.

Returns

True if this is not equal to that.

9.111.4.2 operator*()

```
template<typename Network = UNetwork<>, typename EdgeContT = std::vector< typename Network↵
::Edge>>
reference LinkPred::TestEdgeGen< Network, EdgeContT >::TPEdgeIt::operator* ( )  [inline]
```

Dereference operator.

Returns

A reference to the object to which the iterator points.

9.111.4.3 operator+()

```
template<typename Network = UNetwork<>, typename EdgeContT = std::vector< typename Network↵
::Edge>>
TPEdgeIt LinkPred::TestEdgeGen< Network, EdgeContT >::TPEdgeIt::operator+ (
            const difference_type & n ) const  [inline]
```

Arithmetic + operator.

Parameters

n	Increment value.

Returns

The new iterator.

9.111.4.4 operator++() [1/2]

```
template<typename Network = UNetwork<>, typename EdgeContT = std::vector< typename Network↩
::Edge>>
TPEdgeIt& LinkPred::TestEdgeGen< Network, EdgeContT >::TPEdgeIt::operator++ ( )  [inline]
```

Pre-increment operator.

Returns

A reference to the new iterator.

9.111.4.5 operator++() [2/2]

```
template<typename Network = UNetwork<>, typename EdgeContT = std::vector< typename Network↩
::Edge>>
TPEdgeIt LinkPred::TestEdgeGen< Network, EdgeContT >::TPEdgeIt::operator++ (
            int  )  [inline]
```

Post-increment operator.

Returns

A reference to the new iterator.

9.111.4.6 operator+=()

```
template<typename Network = UNetwork<>, typename EdgeContT = std::vector< typename Network↩
::Edge>>
TPEdgeIt& LinkPred::TestEdgeGen< Network, EdgeContT >::TPEdgeIt::operator+= (
            const difference_type & n )  [inline]
```

Arithmetic += operator.

Parameters

n	Increment value.

Returns

A reference to the new iterator.

9.111.4.7 operator-() [1/2]

```
template<typename Network = UNetwork<>, typename EdgeContT = std::vector< typename Network↩
::Edge>>
TPEdgeIt LinkPred::TestEdgeGen< Network, EdgeContT >::TPEdgeIt::operator- (
            const difference_type & n ) const  [inline]
```

Arithmetic - operator.

Parameters

n	Decrement value.

Returns

The new iterator.

9.111.4.8 operator-() [2/2]

```
template<typename Network = UNetwork<>, typename EdgeContT = std::vector< typename Network↩
::Edge>>
difference_type LinkPred::TestEdgeGen< Network, EdgeContT >::TPEdgeIt::operator- (
            const TPEdgeIt & that ) const  [inline]
```

Difference between the present iterator and the one passed as parameter.

Parameters

that	The other iterator.

Returns

The difference between the current and that iterator.

9.111.4.9 operator--() [1/2]

```
template<typename Network = UNetwork<>, typename EdgeContT = std::vector< typename Network↩
::Edge>>
TPEdgeIt& LinkPred::TestEdgeGen< Network, EdgeContT >::TPEdgeIt::operator-- ( )  [inline]
```

Pre-decrement operator.

Returns

A reference to the new iterator.

9.111.4.10 operator--() [2/2]

```
template<typename Network = UNetwork<>, typename EdgeContT = std::vector< typename Network↵
::Edge>>
TPEdgeIt LinkPred::TestEdgeGen< Network, EdgeContT >::TPEdgeIt::operator-- (
            int ) [inline]
```

Post-decrement operator.

Returns

A reference to the new iterator.

9.111.4.11 operator-=()

```
template<typename Network = UNetwork<>, typename EdgeContT = std::vector< typename Network↵
::Edge>>
TPEdgeIt& LinkPred::TestEdgeGen< Network, EdgeContT >::TPEdgeIt::operator-= (
            const difference_type & n ) [inline]
```

Arithmetic -= operator.

Parameters

n	Decrement value.

Returns

A reference to the new iterator.

9.111.4.12 operator->()

```
template<typename Network = UNetwork<>, typename EdgeContT = std::vector< typename Network↵
::Edge>>
pointer LinkPred::TestEdgeGen< Network, EdgeContT >::TPEdgeIt::operator-> ( ) [inline]
```

Arrow operator.

Returns

A pointer to the object to which the iterator points.

9.111.4.13 operator<()

```
template<typename Network = UNetwork<>, typename EdgeContT = std::vector< typename Network↩
::Edge>>
bool LinkPred::TestEdgeGen< Network, EdgeContT >::TPEdgeIt::operator< (
            const TPEdgeIt & that ) const  [inline]
```

Parameters

that	The other iterator.

Returns

True if this is less than that.

9.111.4.14 operator<=()

```
template<typename Network = UNetwork<>, typename EdgeContT = std::vector< typename Network↩
::Edge>>
bool LinkPred::TestEdgeGen< Network, EdgeContT >::TPEdgeIt::operator<= (
            const TPEdgeIt & that ) const  [inline]
```

Parameters

that	The other iterator.

Returns

True if this is greater or equal to that.

9.111.4.15 operator=() [1/2]

```
template<typename Network = UNetwork<>, typename EdgeContT = std::vector< typename Network↩
::Edge>>
TPEdgeIt& LinkPred::TestEdgeGen< Network, EdgeContT >::TPEdgeIt::operator= (
            TPEdgeIt && that )  [default]
```

Move assignment operator.

Parameters

that	The object to move.

9.111.4.16 operator=() [2/2]

```
template<typename Network = UNetwork<>, typename EdgeContT = std::vector< typename Network↩
::Edge>>
TPEdgeIt& LinkPred::TestEdgeGen< Network, EdgeContT >::TPEdgeIt::operator= (
            TPEdgeIt const & that )  [default]
```

Copy assignment operator.

Parameters

that	The object to copy.

9.111.4.17 operator==()

```
template<typename Network = UNetwork<>, typename EdgeContT = std::vector< typename Network↩
::Edge>>
bool LinkPred::TestEdgeGen< Network, EdgeContT >::TPEdgeIt::operator== (
            const TPEdgeIt & that ) const  [inline]
```

Parameters

that	The other iterator.

Returns

True if this equals that.

9.111.4.18 operator>()

```
template<typename Network = UNetwork<>, typename EdgeContT = std::vector< typename Network↩
::Edge>>
bool LinkPred::TestEdgeGen< Network, EdgeContT >::TPEdgeIt::operator> (
            const TPEdgeIt & that ) const  [inline]
```

Parameters

that	The other iterator.

Returns

True if this is greater that.

9.111.4.19 operator>=()

```
template<typename Network = UNetwork<>, typename EdgeContT = std::vector< typename Network↩
::Edge>>
bool LinkPred::TestEdgeGen< Network, EdgeContT >::TPEdgeIt::operator>= (
            const TPEdgeIt & that ) const  [inline]
```

Parameters

that	The other iterator.

Returns

True if this is less or equal to that.

9.111.5 Friends And Related Function Documentation

9.111.5.1 TestEdgeGen

```
template<typename Network = UNetwork<>, typename EdgeContT = std::vector< typename Network↩
::Edge>>
friend class TestEdgeGen  [friend]
```

TestEdgeGen is a friend.

The documentation for this class was generated from the following file:

- include/linkpred/perf/networkmanipulator.hpp

9.112 LinkPred::TPR< PredResultsT > Class Template Reference

Compute top precision.

`#include <perfmeasure.hpp>`

Inheritance diagram for LinkPred::TPR< PredResultsT >:

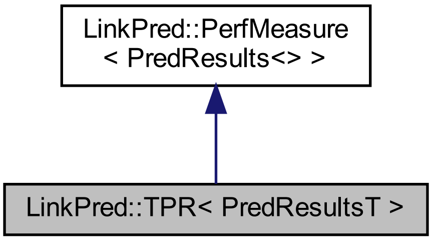

Collaboration diagram for LinkPred::TPR< PredResultsT >:

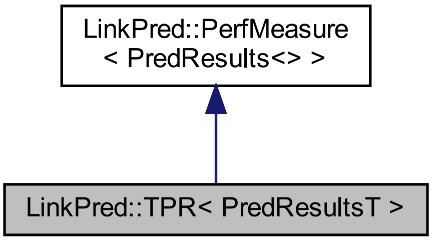

Public Types

- using ScoresItT = typename PerfMeasure< PredResultsT >::ScoresItT

Public Member Functions

- TPR (std::size_t l)
- TPR (std::size_t l, std::string name)
- TPR (TPR const &that)=default
- TPR & operator= (TPR const &that)=default
- TPR (TPR &&that)=default
- TPR & operator= (TPR &&that)=default
- virtual void evalUsingTop (std::shared_ptr< PredResultsT > &predResults, PerfResults &results)
- virtual void evalUsingPredict (std::shared_ptr< PredResultsT > &predResults, PerfResults &results)
- virtual void eval (std::shared_ptr< PredResultsT > &predResults, PerfResults &results)
- virtual void eval (ScoresItT posScoresBegin, ScoresItT posScoresEnd, ScoresItT negScoresBegin, ScoresItT negScoresEnd, SortOrder &posSortOrder, SortOrder &negSortOrder, PerfResults &results)
- bool isUseTopMethod () const
- void setUseTopMethod (bool useTopMethod)
- virtual bool requiresNeg () const
- virtual bool requiresShuffling () const
- virtual ∼TPR ()=default

Static Public Member Functions

- template<typename ScoresItT >
  static double getTPR (ScoresItT posScoresBegin, ScoresItT posScoresEnd, ScoresItT negScoresBegin, ScoresItT negScoresEnd, std::size_t l)

9.112.1 Detailed Description

template<typename PredResultsT = PredResults<>>
class LinkPred::TPR< PredResultsT >

Compute top precision.

Template Parameters

PredResultsT	The prediction results type.

9.112.2 Member Typedef Documentation

9.112.2.1 ScoresItT

```
template<typename PredResultsT = PredResults<>>
using LinkPred::TPR< PredResultsT >::ScoresItT = typename PerfMeasure<PredResultsT>::ScoresItT
```

Scores iterator type.

9.112.3 Constructor & Destructor Documentation

9.112.3.1 TPR() [1/4]

```
template<typename PredResultsT = PredResults<>>
LinkPred::TPR< PredResultsT >::TPR (
            std::size_t l )  [inline]
```

Constructor.

Parameters

l	The number of links to consider.

9.112.3.2 TPR() [2/4]

```
template<typename PredResultsT = PredResults<>>
LinkPred::TPR< PredResultsT >::TPR (
            std::size_t l,
            std::string name )  [inline]
```

Constructor.

Parameters

l	The number of links to consider.
name	The name of the performance measure.

9.112.3.3 TPR() [3/4]

```
template<typename PredResultsT = PredResults<>>
LinkPred::TPR< PredResultsT >::TPR (
            TPR< PredResultsT > const & that ) [default]
```

Copy constructor.

Parameters

that	The object to copy.

9.112.3.4 TPR() [4/4]

```
template<typename PredResultsT = PredResults<>>
LinkPred::TPR< PredResultsT >::TPR (
            TPR< PredResultsT > && that ) [default]
```

Move constructor.

Parameters

that	The object to move.

9.112.3.5 ∼TPR()

```
template<typename PredResultsT = PredResults<>>
virtual LinkPred::TPR< PredResultsT >::~TPR ( ) [virtual], [default]
```

Destructor.

9.112.4 Member Function Documentation

9.112.4.1 eval() [1/2]

```
template<typename PredResultsT = PredResults<>>
virtual void LinkPred::TPR< PredResultsT >::eval (
            ScoresItT posScoresBegin,
            ScoresItT posScoresEnd,
            ScoresItT negScoresBegin,
            ScoresItT negScoresEnd,
            SortOrder & posSortOrder,
            SortOrder & negSortOrder,
            PerfResults & results ) [inline], [virtual]
```

Computes the area under the PR curve.

Parameters

posScoresBegin	Iterator to the first positive score.
posScoresEnd	Iterator to one-past-the-last positive score.
negScoresBegin	Iterator to the first negative score.
negScoresEnd	Iterator to one-past-the-last negative score.
posSortOrder	The sorting order of positive scores. This may be modified by the method.
negSortOrder	The sorting order of negative scores. This may be modified by the method.
results	To write results.

9.112.4.2 eval() [2/2]

```
template<typename PredResultsT = PredResults<>>
virtual void LinkPred::TPR< PredResultsT >::eval (
            std::shared_ptr< PredResultsT > & predResults,
            PerfResults & results ) [inline], [virtual]
```

Computes the performance measure.

Parameters

predResults	The prediction results.
results	Iterator to write results.

9.112.4.3 evalUsingPredict()

```
template<typename PredResultsT = PredResults<>>
virtual void LinkPred::TPR< PredResultsT >::evalUsingPredict (
            std::shared_ptr< PredResultsT > & predResults,
            PerfResults & results ) [inline], [virtual]
```

Computes the TPR using the predict method.

Parameters

predResults	The prediction results.
results	Iterator to write results.

9.112.4.4 evalUsingTop()

```
template<typename PredResultsT = PredResults<>>
virtual void LinkPred::TPR< PredResultsT >::evalUsingTop (
            std::shared_ptr< PredResultsT > & predResults,
            PerfResults & results )  [inline], [virtual]
```

Computes the TPR using the top method.

Parameters

predResults	The prediction results.
results	Iterator to write results.

9.112.4.5 getTPR()

```
template<typename PredResultsT = PredResults<>>
template<typename ScoresItT >
static double LinkPred::TPR< PredResultsT >::getTPR (
            ScoresItT posScoresBegin,
            ScoresItT posScoresEnd,
            ScoresItT negScoresBegin,
            ScoresItT negScoresEnd,
            std::size_t l )  [inline], [static]
```

Ranges must be sorted in decreasing order.

Parameters

posScoresBegin	Iterator to the first positive score.
posScoresEnd	Iterator to one-past-the-last positive score.
negScoresBegin	Iterator to the first negative score.
negScoresEnd	Iterator to one-past-the-last negative score.
l	The number of links to consider.

9.112.4.6 isUseTopMethod()

```
template<typename PredResultsT = PredResults<>>
```

```
bool LinkPred::TPR< PredResultsT >::isUseTopMethod ( ) const  [inline]
```

Returns

True if the method top is used to compute TPR, false otherwise.

9.112.4.7 operator=() [1/2]

```
template<typename PredResultsT = PredResults<>>
TPR& LinkPred::TPR< PredResultsT >::operator= (
            TPR< PredResultsT > && that )  [default]
```

Move assignment operator.

Parameters

that	The object to move.

9.112.4.8 operator=() [2/2]

```
template<typename PredResultsT = PredResults<>>
TPR& LinkPred::TPR< PredResultsT >::operator= (
            TPR< PredResultsT > const & that )  [default]
```

Copy assignment operator.

Parameters

that	The object to copy.

9.112.4.9 requiresNeg()

```
template<typename PredResultsT = PredResults<>>
virtual bool LinkPred::TPR< PredResultsT >::requiresNeg ( ) const  [inline], [virtual]
```

Returns

Whether the performance measure requires the generation of negative set. The default value is true.

9.112.4.10 requiresShuffling()

```
template<typename PredResultsT = PredResults<>>
virtual bool LinkPred::TPR< PredResultsT >::requiresShuffling ( ) const [inline], [virtual]
```

Returns

Whether the performance measure requires network shuffling.

9.112.4.11 setUseTopMethod()

```
template<typename PredResultsT = PredResults<>>
void LinkPred::TPR< PredResultsT >::setUseTopMethod (
            bool useTopMethod ) [inline]
```

Enable/disable the use of the method top is used to compute TPR.

Parameters

useTopMethod	If true, enable the use of the method top, otherwise disable it.

The documentation for this class was generated from the following file:

- include/linkpred/perf/perfmeasure.hpp

9.113 LinkPred::UADAPredictor< Network, EdgeRndIt, ScoreRndIt, EdgeRndOutIt > Class Template Reference

Adamic Adar index link predictor.

`#include <uadapredictor.hpp>`

Inheritance diagram for LinkPred::UADAPredictor< Network, EdgeRndIt, ScoreRndIt, EdgeRndOutIt >:

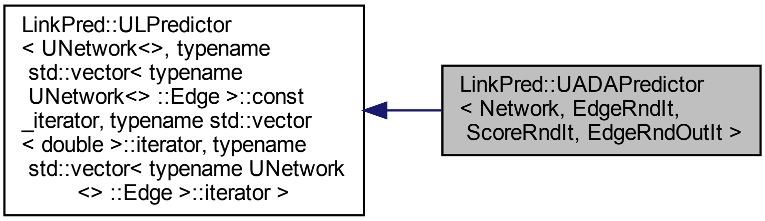

Collaboration diagram for LinkPred::UADAPredictor< Network, EdgeRndIt, ScoreRndIt, EdgeRndOutIt >:

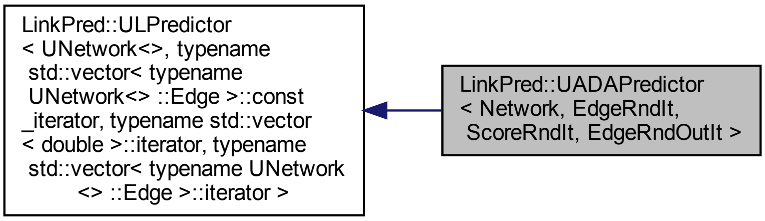

Public Member Functions

- UADAPredictor (std::shared_ptr< Network const > net)
- UADAPredictor (UADAPredictor const &that)=default
- UADAPredictor & operator= (UADAPredictor const &that)=default
- UADAPredictor (UADAPredictor &&that)=default
- UADAPredictor & operator= (UADAPredictor &&that)=default
- virtual void init ()
- virtual void learn ()
- virtual double score (Edge const &e)
- virtual void predict (EdgeRndIt begin, EdgeRndIt end, ScoreRndIt scores)
- virtual std::size_t top (std::size_t k, EdgeRndOutIt eit, ScoreRndIt sit)
- virtual ~UADAPredictor ()=default

Additional Inherited Members

9.113.1 Detailed Description

template<typename Network = UNetwork<>, typename EdgeRndIt = typename std::vector<typename Network::Edge>↩ ::const_iterator, typename ScoreRndIt = typename std::vector<double>::iterator, typename EdgeRndOutIt = typename std↩ ::vector<typename Network::Edge>::iterator>
class LinkPred::UADAPredictor< Network, EdgeRndIt, ScoreRndIt, EdgeRndOutIt >

Adamic Adar index link predictor.

Template Parameters

Network	The network type.
Edge↩ RndIt	A random iterator type used to iterate on edges.
Score↩ RndIt	A random iterator type used to iterate on scores.

9.113.2 Constructor & Destructor Documentation

9.113.2.1 UADAPredictor() [1/3]

```
template<typename Network = UNetwork<>, typename EdgeRndIt = typename std::vector<typename
Network::Edge>::const_iterator, typename ScoreRndIt = typename std::vector<double>::iterator,
typename EdgeRndOutIt = typename std::vector<typename Network::Edge>::iterator>
LinkPred::UADAPredictor< Network, EdgeRndIt, ScoreRndIt, EdgeRndOutIt >::UADAPredictor (
            std::shared_ptr< Network const > net )  [inline]
```

Parameters

net	The network.

9.113.2.2 UADAPredictor() [2/3]

```
template<typename Network = UNetwork<>, typename EdgeRndIt = typename std::vector<typename
Network::Edge>::const_iterator, typename ScoreRndIt = typename std::vector<double>::iterator,
typename EdgeRndOutIt = typename std::vector<typename Network::Edge>::iterator>
LinkPred::UADAPredictor< Network, EdgeRndIt, ScoreRndIt, EdgeRndOutIt >::UADAPredictor (
            UADAPredictor< Network, EdgeRndIt, ScoreRndIt, EdgeRndOutIt > const & that )
[default]
```

Copy constructor.

Parameters

that	The object to copy.

9.113.2.3 UADAPredictor() [3/3]

```
template<typename Network = UNetwork<>, typename EdgeRndIt = typename std::vector<typename
Network::Edge>::const_iterator, typename ScoreRndIt = typename std::vector<double>::iterator,
typename EdgeRndOutIt = typename std::vector<typename Network::Edge>::iterator>
LinkPred::UADAPredictor< Network, EdgeRndIt, ScoreRndIt, EdgeRndOutIt >::UADAPredictor (
            UADAPredictor< Network, EdgeRndIt, ScoreRndIt, EdgeRndOutIt > && that )  [default]
```

Move constructor.

Parameters

that	The object to move.

9.113.2.4 ∼UADAPredictor()

```
template<typename Network = UNetwork<>, typename EdgeRndIt = typename std::vector<typename
Network::Edge>::const_iterator, typename ScoreRndIt = typename std::vector<double>::iterator,
typename EdgeRndOutIt = typename std::vector<typename Network::Edge>::iterator>
virtual LinkPred::UADAPredictor< Network, EdgeRndIt, ScoreRndIt, EdgeRndOutIt >::∼UADAPredictor
( ) [virtual], [default]
```

Destructor.

9.113.3 Member Function Documentation

9.113.3.1 init()

```
template<typename Network = UNetwork<>, typename EdgeRndIt = typename std::vector<typename
Network::Edge>::const_iterator, typename ScoreRndIt = typename std::vector<double>::iterator,
typename EdgeRndOutIt = typename std::vector<typename Network::Edge>::iterator>
virtual void LinkPred::UADAPredictor< Network, EdgeRndIt, ScoreRndIt, EdgeRndOutIt >::init ( )
[inline], [virtual]
```

Initialize the predictor.

Implements LinkPred::ULPredictor< UNetwork<>, typename std::vector< typename UNetwork<> ::Edge >::const_iterator, typena

9.113.3.2 learn()

```
template<typename Network = UNetwork<>, typename EdgeRndIt = typename std::vector<typename
Network::Edge>::const_iterator, typename ScoreRndIt = typename std::vector<double>::iterator,
typename EdgeRndOutIt = typename std::vector<typename Network::Edge>::iterator>
virtual void LinkPred::UADAPredictor< Network, EdgeRndIt, ScoreRndIt, EdgeRndOutIt >::learn (
) [inline], [virtual]
```

Learning.

Implements LinkPred::ULPredictor< UNetwork<>, typename std::vector< typename UNetwork<> ::Edge >::const_iterator, typena

9.113.3.3 operator=() [1/2]

```
template<typename Network = UNetwork<>, typename EdgeRndIt = typename std::vector<typename
Network::Edge>::const_iterator, typename ScoreRndIt = typename std::vector<double>::iterator,
typename EdgeRndOutIt = typename std::vector<typename Network::Edge>::iterator>
UADAPredictor& LinkPred::UADAPredictor< Network, EdgeRndIt, ScoreRndIt, EdgeRndOutIt >::operator=
(
            UADAPredictor< Network, EdgeRndIt, ScoreRndIt, EdgeRndOutIt > && that )  [default]
```

Move assignment operator.

Parameters

that	The object to move.

9.113.3.4 operator=() [2/2]

```
template<typename Network = UNetwork<>, typename EdgeRndIt = typename std::vector<typename
Network::Edge>::const_iterator, typename ScoreRndIt = typename std::vector<double>::iterator,
typename EdgeRndOutIt = typename std::vector<typename Network::Edge>::iterator>
UADAPredictor& LinkPred::UADAPredictor< Network, EdgeRndIt, ScoreRndIt, EdgeRndOutIt >::operator=
(
            UADAPredictor< Network, EdgeRndIt, ScoreRndIt, EdgeRndOutIt > const & that )
[default]
```

Copy assignment operator.

Parameters

that	The object to copy.

9.113.3.5 predict()

```
template<typename Network = UNetwork<>, typename EdgeRndIt = typename std::vector<typename
Network::Edge>::const_iterator, typename ScoreRndIt = typename std::vector<double>::iterator,
typename EdgeRndOutIt = typename std::vector<typename Network::Edge>::iterator>
virtual void LinkPred::UADAPredictor< Network, EdgeRndIt, ScoreRndIt, EdgeRndOutIt >::predict
(
            EdgeRndIt begin,
            EdgeRndIt end,
            ScoreRndIt scores ) [virtual]
```

Predict the links.

Parameters

begin	Beginning of the links to be predicted.
end	end of the links to be predicted.
scores	Beginning of scores.

Reimplemented from LinkPred::ULPredictor< UNetwork<>, typename std::vector< typename UNetwork<> ::Edge >::const_iterat

9.113.3.6 score()

```
template<typename Network = UNetwork<>, typename EdgeRndIt = typename std::vector<typename
Network::Edge>::const_iterator, typename ScoreRndIt = typename std::vector<double>::iterator,
```

```
typename EdgeRndOutIt = typename std::vector<typename Network::Edge>::iterator>
virtual double LinkPred::UADAPredictor< Network, EdgeRndIt, ScoreRndIt, EdgeRndOutIt >::score
(
            Edge const & e )  [inline], [virtual]
```

Compute the score of a single edge.

Parameters

e	The edge.

Returns

The score of e.

9.113.3.7 top()

```
template<typename Network = UNetwork<>, typename EdgeRndIt = typename std::vector<typename
Network::Edge>::const_iterator, typename ScoreRndIt = typename std::vector<double>::iterator,
typename EdgeRndOutIt = typename std::vector<typename Network::Edge>::iterator>
virtual std::size_t LinkPred::UADAPredictor< Network, EdgeRndIt, ScoreRndIt, EdgeRndOutIt >↩
::top (
            std::size_t k,
            EdgeRndOutIt eit,
            ScoreRndIt sit )  [virtual]
```

Finds the k negative edges with the top score. Ties are broken randomly.

Parameters

k	The number of edges to find.
eit	An output iterator where the edges are written.
sit	An output iterator where the scores are written. The scores are written in the same order as the edges.

Returns

The number of negative edges inserted. It is the minimum between k and the number of negative edges in the network.

Reimplemented from LinkPred::ULPredictor< UNetwork<>, typename std::vector< typename UNetwork<> ::Edge >::const_iterat

The documentation for this class was generated from the following file:

- include/linkpred/predictors/undirected/uadapredictor.hpp

9.114 LinkPred::UCNEPredictor< Network, EdgeRndIt, ScoreRndIt, EdgeRndOutIt > Class Template Reference

Common neighbor link predictor.

`#include <ucnepredictor.hpp>`

Inheritance diagram for LinkPred::UCNEPredictor< Network, EdgeRndIt, ScoreRndIt, EdgeRndOutIt >:

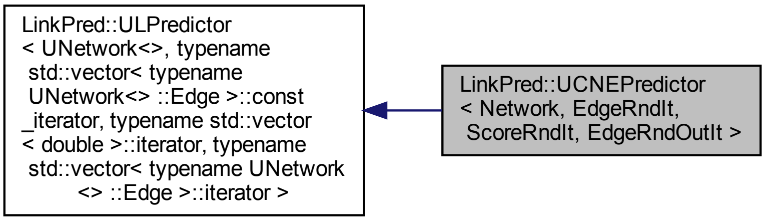

Collaboration diagram for LinkPred::UCNEPredictor< Network, EdgeRndIt, ScoreRndIt, EdgeRndOutIt >:

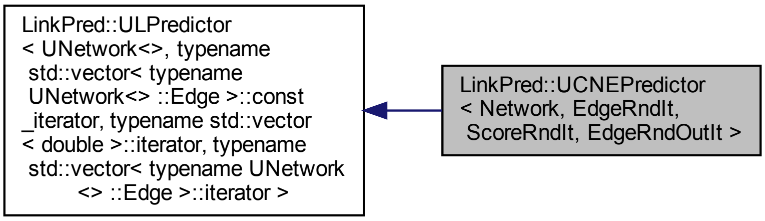

Public Member Functions

- UCNEPredictor (std::shared_ptr< Network const > net)
- UCNEPredictor (UCNEPredictor const &that)=default
- UCNEPredictor & operator= (UCNEPredictor const &that)=default
- UCNEPredictor (UCNEPredictor &&that)=default
- UCNEPredictor & operator= (UCNEPredictor &&that)=default
- virtual void init ()
- virtual void learn ()
- virtual void predict (EdgeRndIt begin, EdgeRndIt end, ScoreRndIt scores)
- virtual double score (Edge const &e)
- virtual std::size_t top (std::size_t k, EdgeRndOutIt eit, ScoreRndIt sit)
- virtual ~UCNEPredictor ()=default

Additional Inherited Members

9.114.1 Detailed Description

template<typename Network = UNetwork<>, typename EdgeRndIt = typename std::vector<typename Network::Edge>::const_iterator, typename ScoreRndIt = typename std::vector<double>::iterator, typename EdgeRndOutIt = typename std::vector<typename Network::Edge>::iterator>
class LinkPred::UCNEPredictor< Network, EdgeRndIt, ScoreRndIt, EdgeRndOutIt >

Common neighbor link predictor.

Template Parameters

Network	The network type.
EdgeRndIt	A random iterator type used to iterate on edges.
ScoreRndIt	A random iterator type used to iterate on scores.

9.114.2 Constructor & Destructor Documentation

9.114.2.1 UCNEPredictor() [1/3]

```
template<typename Network = UNetwork<>, typename EdgeRndIt = typename std::vector<typename
Network::Edge>::const_iterator, typename ScoreRndIt = typename std::vector<double>::iterator,
typename EdgeRndOutIt = typename std::vector<typename Network::Edge>::iterator>
LinkPred::UCNEPredictor< Network, EdgeRndIt, ScoreRndIt, EdgeRndOutIt >::UCNEPredictor (
            std::shared_ptr< Network const > net )  [inline]
```

Parameters

net	The network.

9.114.2.2 UCNEPredictor() [2/3]

```
template<typename Network = UNetwork<>, typename EdgeRndIt = typename std::vector<typename
Network::Edge>::const_iterator, typename ScoreRndIt = typename std::vector<double>::iterator,
typename EdgeRndOutIt = typename std::vector<typename Network::Edge>::iterator>
LinkPred::UCNEPredictor< Network, EdgeRndIt, ScoreRndIt, EdgeRndOutIt >::UCNEPredictor (
            UCNEPredictor< Network, EdgeRndIt, ScoreRndIt, EdgeRndOutIt > const & that )
[default]
```

Copy constructor.

Parameters

that	The object to copy.

9.114.2.3 UCNEPredictor() [3/3]

```
template<typename Network = UNetwork<>, typename EdgeRndIt = typename std::vector<typename
Network::Edge>::const_iterator, typename ScoreRndIt = typename std::vector<double>::iterator,
typename EdgeRndOutIt = typename std::vector<typename Network::Edge>::iterator>
LinkPred::UCNEPredictor< Network, EdgeRndIt, ScoreRndIt, EdgeRndOutIt >::UCNEPredictor (
            UCNEPredictor< Network, EdgeRndIt, ScoreRndIt, EdgeRndOutIt > && that )  [default]
```

Move constructor.

Parameters

that	The object to move.

9.114.2.4 ∼UCNEPredictor()

```
template<typename Network = UNetwork<>, typename EdgeRndIt = typename std::vector<typename
Network::Edge>::const_iterator, typename ScoreRndIt = typename std::vector<double>::iterator,
typename EdgeRndOutIt = typename std::vector<typename Network::Edge>::iterator>
virtual LinkPred::UCNEPredictor< Network, EdgeRndIt, ScoreRndIt, EdgeRndOutIt >::~UCNEPredictor
( )  [virtual], [default]
```

Destructor.

9.114.3 Member Function Documentation

9.114.3.1 init()

```
template<typename Network = UNetwork<>, typename EdgeRndIt = typename std::vector<typename
Network::Edge>::const_iterator, typename ScoreRndIt = typename std::vector<double>::iterator,
typename EdgeRndOutIt = typename std::vector<typename Network::Edge>::iterator>
virtual void LinkPred::UCNEPredictor< Network, EdgeRndIt, ScoreRndIt, EdgeRndOutIt >::init ( )
[inline], [virtual]
```

Initialize the solver.

Implements LinkPred::ULPredictor< UNetwork<>, typename std::vector< typename UNetwork<> ::Edge >::const_iterator, typena

9.114.3.2 learn()

```
template<typename Network = UNetwork<>, typename EdgeRndIt = typename std::vector<typename
Network::Edge>::const_iterator, typename ScoreRndIt = typename std::vector<double>::iterator,
typename EdgeRndOutIt = typename std::vector<typename Network::Edge>::iterator>
virtual void LinkPred::UCNEPredictor< Network, EdgeRndIt, ScoreRndIt, EdgeRndOutIt >::learn (
) [inline], [virtual]
```

Learn.

Implements LinkPred::ULPredictor< UNetwork<>, typename std::vector< typename UNetwork<> ::Edge >::const_iterator, typena

9.114.3.3 operator=() [1/2]

```
template<typename Network = UNetwork<>, typename EdgeRndIt = typename std::vector<typename
Network::Edge>::const_iterator, typename ScoreRndIt = typename std::vector<double>::iterator,
typename EdgeRndOutIt = typename std::vector<typename Network::Edge>::iterator>
UCNEPredictor& LinkPred::UCNEPredictor< Network, EdgeRndIt, ScoreRndIt, EdgeRndOutIt >::operator=
(
            UCNEPredictor< Network, EdgeRndIt, ScoreRndIt, EdgeRndOutIt > && that )  [default]
```

Move assignment operator.

Parameters

that	The object to move.

9.114.3.4 operator=() [2/2]

```
template<typename Network = UNetwork<>, typename EdgeRndIt = typename std::vector<typename
Network::Edge>::const_iterator, typename ScoreRndIt = typename std::vector<double>::iterator,
typename EdgeRndOutIt = typename std::vector<typename Network::Edge>::iterator>
UCNEPredictor& LinkPred::UCNEPredictor< Network, EdgeRndIt, ScoreRndIt, EdgeRndOutIt >::operator=
(
            UCNEPredictor< Network, EdgeRndIt, ScoreRndIt, EdgeRndOutIt > const & that )
[default]
```

Copy assignment operator.

Parameters

that	The object to copy.

9.114.3.5 predict()

```
template<typename Network = UNetwork<>, typename EdgeRndIt = typename std::vector<typename
Network::Edge>::const_iterator, typename ScoreRndIt = typename std::vector<double>::iterator,
typename EdgeRndOutIt = typename std::vector<typename Network::Edge>::iterator>
virtual void LinkPred::UCNEPredictor< Network, EdgeRndIt, ScoreRndIt, EdgeRndOutIt >::predict
(
            EdgeRndIt begin,
            EdgeRndIt end,
            ScoreRndIt scores )  [virtual]
```

Predict the links.

Parameters

begin	Beginning of the links to be predicted.
end	end of the links to be predicted.
scores	Beginning of scores.

Reimplemented from LinkPred::ULPredictor< UNetwork<>, typename std::vector< typename UNetwork<> ::Edge >::const_iterat

9.114.3.6 score()

```
template<typename Network = UNetwork<>, typename EdgeRndIt = typename std::vector<typename
Network::Edge>::const_iterator, typename ScoreRndIt = typename std::vector<double>::iterator,
typename EdgeRndOutIt = typename std::vector<typename Network::Edge>::iterator>
virtual double LinkPred::UCNEPredictor< Network, EdgeRndIt, ScoreRndIt, EdgeRndOutIt >::score
(
            Edge const & e )  [virtual]
```

Compute the score of a single edge.

Parameters

e	The edge.

Returns

The score of e.

9.114.3.7 top()

```
template<typename Network = UNetwork<>, typename EdgeRndIt = typename std::vector<typename
Network::Edge>::const_iterator, typename ScoreRndIt = typename std::vector<double>::iterator,
typename EdgeRndOutIt = typename std::vector<typename Network::Edge>::iterator>
```

```
virtual std::size_t LinkPred::UCNEPredictor< Network, EdgeRndIt, ScoreRndIt, EdgeRndOutIt >↩
::top (
            std::size_t k,
            EdgeRndOutIt eit,
            ScoreRndIt sit )  [virtual]
```

Finds the k negative edges with the top score. Ties are broken randomly.

Parameters

k	The number of edges to find.
eit	An output iterator where the edges are written.
sit	An output iterator where the scores are written. The scores are written in the same order as the edges.

Returns

The number of negative edges inserted. It is the minimum between k and the number of negative edges in the network.

Reimplemented from LinkPred::ULPredictor< UNetwork<>, typename std::vector< typename UNetwork<> ::Edge >::const_iterato

The documentation for this class was generated from the following file:

- include/linkpred/predictors/undirected/ucnepredictor.hpp

9.115 LinkPred::UCRAPredictor< Network, EdgeRndIt, ScoreRndIt, EdgeRndOutIt > Class Template Reference

Local path link predictor.

`#include <ucrapredictor.hpp>`

Inheritance diagram for LinkPred::UCRAPredictor< Network, EdgeRndIt, ScoreRndIt, EdgeRndOutIt >:

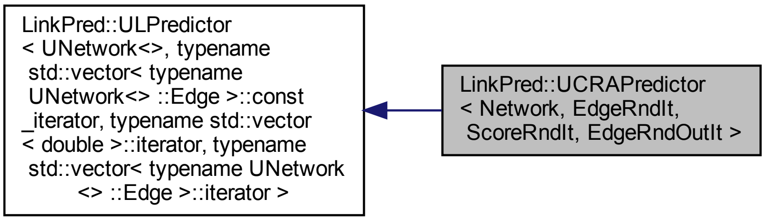

Collaboration diagram for LinkPred::UCRAPredictor< Network, EdgeRndIt, ScoreRndIt, EdgeRndOutIt >:

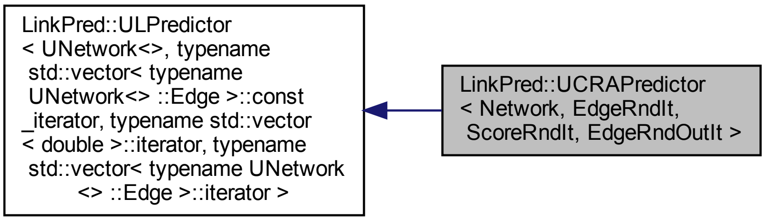

Public Member Functions

- UCRAPredictor (std::shared_ptr< Network const > net)
- UCRAPredictor (UCRAPredictor const &that)=default
- UCRAPredictor & operator= (UCRAPredictor const &that)=default
- UCRAPredictor (UCRAPredictor &&that)=default
- UCRAPredictor & operator= (UCRAPredictor &&that)=default
- virtual void init ()
- virtual void learn ()
- virtual double score (Edge const &e)
- virtual void predict (EdgeRndIt begin, EdgeRndIt end, ScoreRndIt scores)
- virtual std::size_t top (std::size_t k, EdgeRndOutIt eit, ScoreRndIt sit)
- virtual ~UCRAPredictor ()=default

Additional Inherited Members

9.115.1 Detailed Description

template<typename Network = UNetwork<>, typename EdgeRndIt = typename std::vector<typename Network::Edge>↩ ::const_iterator, typename ScoreRndIt = typename std::vector<double>::iterator, typename EdgeRndOutIt = typename std↩ ::vector<typename Network::Edge>::iterator>
class LinkPred::UCRAPredictor< Network, EdgeRndIt, ScoreRndIt, EdgeRndOutIt >

Local path link predictor.

Template Parameters

Network	The network type.
Edge↩ RndIt	A random iterator type used to iterate on edges.
Score↩ RndIt	A random iterator type used to iterate on scores.

9.115.2 Constructor & Destructor Documentation

9.115.2.1 UCRAPredictor() [1/3]

```
template<typename Network = UNetwork<>, typename EdgeRndIt = typename std::vector<typename
Network::Edge>::const_iterator, typename ScoreRndIt = typename std::vector<double>::iterator,
typename EdgeRndOutIt = typename std::vector<typename Network::Edge>::iterator>
LinkPred::UCRAPredictor< Network, EdgeRndIt, ScoreRndIt, EdgeRndOutIt >::UCRAPredictor (
            std::shared_ptr< Network const > net ) [inline]
```

Parameters

net	The network.

9.115.2.2 UCRAPredictor() [2/3]

```
template<typename Network = UNetwork<>, typename EdgeRndIt = typename std::vector<typename
Network::Edge>::const_iterator, typename ScoreRndIt = typename std::vector<double>::iterator,
typename EdgeRndOutIt = typename std::vector<typename Network::Edge>::iterator>
LinkPred::UCRAPredictor< Network, EdgeRndIt, ScoreRndIt, EdgeRndOutIt >::UCRAPredictor (
            UCRAPredictor< Network, EdgeRndIt, ScoreRndIt, EdgeRndOutIt > const & that )
[default]
```

Copy constructor.

Parameters

that	The object to copy.

9.115.2.3 UCRAPredictor() [3/3]

```
template<typename Network = UNetwork<>, typename EdgeRndIt = typename std::vector<typename
Network::Edge>::const_iterator, typename ScoreRndIt = typename std::vector<double>::iterator,
typename EdgeRndOutIt = typename std::vector<typename Network::Edge>::iterator>
LinkPred::UCRAPredictor< Network, EdgeRndIt, ScoreRndIt, EdgeRndOutIt >::UCRAPredictor (
            UCRAPredictor< Network, EdgeRndIt, ScoreRndIt, EdgeRndOutIt > && that ) [default]
```

Move constructor.

Parameters

that	The object to move.

9.115.2.4 ∼UCRAPredictor()

```
template<typename Network = UNetwork<>, typename EdgeRndIt = typename std::vector<typename
Network::Edge>::const_iterator, typename ScoreRndIt = typename std::vector<double>::iterator,
typename EdgeRndOutIt = typename std::vector<typename Network::Edge>::iterator>
virtual LinkPred::UCRAPredictor< Network, EdgeRndIt, ScoreRndIt, EdgeRndOutIt >::~UCRAPredictor
( )  [virtual], [default]
```

Destructor.

9.115.3 Member Function Documentation

9.115.3.1 init()

```
template<typename Network = UNetwork<>, typename EdgeRndIt = typename std::vector<typename
Network::Edge>::const_iterator, typename ScoreRndIt = typename std::vector<double>::iterator,
typename EdgeRndOutIt = typename std::vector<typename Network::Edge>::iterator>
virtual void LinkPred::UCRAPredictor< Network, EdgeRndIt, ScoreRndIt, EdgeRndOutIt >::init ( )
[inline], [virtual]
```

Initialize the predictor.

Implements LinkPred::ULPredictor< UNetwork<>, typename std::vector< typename UNetwork<> ::Edge >::const_iterator, typena

9.115.3.2 learn()

```
template<typename Network = UNetwork<>, typename EdgeRndIt = typename std::vector<typename
Network::Edge>::const_iterator, typename ScoreRndIt = typename std::vector<double>::iterator,
typename EdgeRndOutIt = typename std::vector<typename Network::Edge>::iterator>
virtual void LinkPred::UCRAPredictor< Network, EdgeRndIt, ScoreRndIt, EdgeRndOutIt >::learn (
)  [inline], [virtual]
```

Learn.

Implements LinkPred::ULPredictor< UNetwork<>, typename std::vector< typename UNetwork<> ::Edge >::const_iterator, typena

9.115.3.3 operator=() [1/2]

```
template<typename Network = UNetwork<>, typename EdgeRndIt = typename std::vector<typename
Network::Edge>::const_iterator, typename ScoreRndIt = typename std::vector<double>::iterator,
typename EdgeRndOutIt = typename std::vector<typename Network::Edge>::iterator>
UCRAPredictor& LinkPred::UCRAPredictor< Network, EdgeRndIt, ScoreRndIt, EdgeRndOutIt >::operator=
(
            UCRAPredictor< Network, EdgeRndIt, ScoreRndIt, EdgeRndOutIt > && that )  [default]
```

Move assignment operator.

Parameters

that	The object to move.

9.115.3.4 operator=() [2/2]

```
template<typename Network = UNetwork<>, typename EdgeRndIt = typename std::vector<typename
Network::Edge>::const_iterator, typename ScoreRndIt = typename std::vector<double>::iterator,
typename EdgeRndOutIt = typename std::vector<typename Network::Edge>::iterator>
UCRAPredictor& LinkPred::UCRAPredictor< Network, EdgeRndIt, ScoreRndIt, EdgeRndOutIt >::operator=
(
            UCRAPredictor< Network, EdgeRndIt, ScoreRndIt, EdgeRndOutIt > const & that )
[default]
```

Copy assignment operator.

Parameters

that	The object to copy.

9.115.3.5 predict()

```
template<typename Network = UNetwork<>, typename EdgeRndIt = typename std::vector<typename
Network::Edge>::const_iterator, typename ScoreRndIt = typename std::vector<double>::iterator,
typename EdgeRndOutIt = typename std::vector<typename Network::Edge>::iterator>
virtual void LinkPred::UCRAPredictor< Network, EdgeRndIt, ScoreRndIt, EdgeRndOutIt >::predict
(
            EdgeRndIt begin,
            EdgeRndIt end,
            ScoreRndIt scores )  [virtual]
```

Predict the links.

Parameters

begin	Beginning of the links to be predicted.
end	end of the links to be predicted.
scores	Beginning of scores.

Reimplemented from LinkPred::ULPredictor< UNetwork<>, typename std::vector< typename UNetwork<> ::Edge >::const_iterat

9.115.3.6 score()

```
template<typename Network = UNetwork<>, typename EdgeRndIt = typename std::vector<typename
Network::Edge>::const_iterator, typename ScoreRndIt = typename std::vector<double>::iterator,
```

```
typename EdgeRndOutIt = typename std::vector<typename Network::Edge>::iterator>
virtual double LinkPred::UCRAPredictor< Network, EdgeRndIt, ScoreRndIt, EdgeRndOutIt >::score
(
            Edge const & e )  [inline], [virtual]
```

Compute the score of a single edge.

Parameters

e	The edge.

Returns

The score of e.

9.115.3.7 top()

```
template<typename Network = UNetwork<>, typename EdgeRndIt = typename std::vector<typename
Network::Edge>::const_iterator, typename ScoreRndIt = typename std::vector<double>::iterator,
typename EdgeRndOutIt = typename std::vector<typename Network::Edge>::iterator>
virtual std::size_t LinkPred::UCRAPredictor< Network, EdgeRndIt, ScoreRndIt, EdgeRndOutIt >↩
::top (
            std::size_t k,
            EdgeRndOutIt eit,
            ScoreRndIt sit )  [virtual]
```

Finds the k negative edges with the top score. Ties are broken randmly.

Parameters

k	The number of edges to find.
eit	An output iterator where the edges are written.
sit	An output iterator where the scores are written. The scores are written in the same order as the edges.

Returns

The number of negative edges inserted. It is the minimum between k and the number of negative edges in the network.

Reimplemented from LinkPred::ULPredictor< UNetwork<>, typename std::vector< typename UNetwork<> ::Edge >::const_iterat

The documentation for this class was generated from the following file:

- include/linkpred/predictors/undirected/ucrapredictor.hpp

9.116 LinkPred::UCSTPredictor< Network, EdgeRndIt, ScoreRndIt, EdgeRndOutIt > Class Template Reference

Constant link predictor.

```
#include <ucstpredictor.hpp>
```

Inheritance diagram for LinkPred::UCSTPredictor< Network, EdgeRndIt, ScoreRndIt, EdgeRndOutIt >:

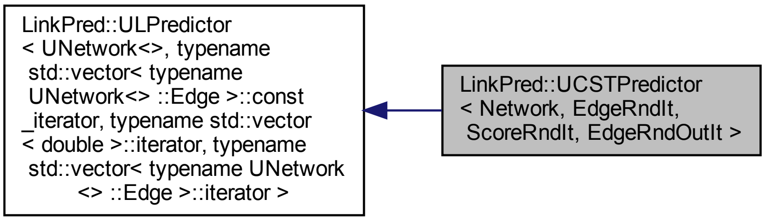

Collaboration diagram for LinkPred::UCSTPredictor< Network, EdgeRndIt, ScoreRndIt, EdgeRndOutIt >:

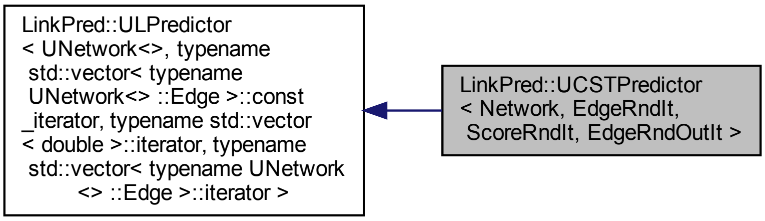

Public Member Functions

- UCSTPredictor (std::shared_ptr< Network const > net, long int seed)
- UCSTPredictor (UCSTPredictor const &that)=default
- UCSTPredictor & operator= (UCSTPredictor const &that)=default
- UCSTPredictor (UCSTPredictor &&that)=default
- UCSTPredictor & operator= (UCSTPredictor &&that)=default
- virtual void init ()
- virtual void learn ()
- virtual double score (Edge const &e)
- virtual void predict (EdgeRndIt begin, EdgeRndIt end, ScoreRndIt scores)
- virtual std::size_t top (std::size_t k, EdgeRndOutIt eit, ScoreRndIt sit)
- virtual ~UCSTPredictor ()=default

Additional Inherited Members

9.116.1 Detailed Description

template<typename Network = UNetwork<>, typename EdgeRndIt = typename std::vector<typename Network::Edge>↩ ::const_iterator, typename ScoreRndIt = typename std::vector<double>::iterator, typename EdgeRndOutIt = typename std↩ ::vector<typename Network::Edge>::iterator>
class LinkPred::UCSTPredictor< Network, EdgeRndIt, ScoreRndIt, EdgeRndOutIt >

Constant link predictor.

Template Parameters

Network	The network type.
Edge↩ RndIt	A random iterator type used to iterate on edges.
Score↩ RndIt	A random iterator type used to iterate on scores.

9.116.2 Constructor & Destructor Documentation

9.116.2.1 UCSTPredictor() [1/3]

```
template<typename Network = UNetwork<>, typename EdgeRndIt = typename std::vector<typename
Network::Edge>::const_iterator, typename ScoreRndIt = typename std::vector<double>::iterator,
typename EdgeRndOutIt = typename std::vector<typename Network::Edge>::iterator>
LinkPred::UCSTPredictor< Network, EdgeRndIt, ScoreRndIt, EdgeRndOutIt >::UCSTPredictor (
            std::shared_ptr< Network const > net,
            long int seed )  [inline]
```

Parameters

net	The network.
seed	Seed for the random number generator. This is used to select randomly k edges in the method top. Different calls to top return different set of edges, which the correct behavior.

9.116.2.2 UCSTPredictor() [2/3]

```
template<typename Network = UNetwork<>, typename EdgeRndIt = typename std::vector<typename
Network::Edge>::const_iterator, typename ScoreRndIt = typename std::vector<double>::iterator,
typename EdgeRndOutIt = typename std::vector<typename Network::Edge>::iterator>
LinkPred::UCSTPredictor< Network, EdgeRndIt, ScoreRndIt, EdgeRndOutIt >::UCSTPredictor (
            UCSTPredictor< Network, EdgeRndIt, ScoreRndIt, EdgeRndOutIt > const & that )
[default]
```

Copy constructor.

Parameters

that	The object to copy.

9.116.2.3 UCSTPredictor() [3/3]

```
template<typename Network = UNetwork<>, typename EdgeRndIt = typename std::vector<typename
Network::Edge>::const_iterator, typename ScoreRndIt = typename std::vector<double>::iterator,
typename EdgeRndOutIt = typename std::vector<typename Network::Edge>::iterator>
LinkPred::UCSTPredictor< Network, EdgeRndIt, ScoreRndIt, EdgeRndOutIt >::UCSTPredictor (
            UCSTPredictor< Network, EdgeRndIt, ScoreRndIt, EdgeRndOutIt > && that )  [default]
```

Move constructor.

Parameters

that	The object to move.

9.116.2.4 ∼UCSTPredictor()

```
template<typename Network = UNetwork<>, typename EdgeRndIt = typename std::vector<typename
Network::Edge>::const_iterator, typename ScoreRndIt = typename std::vector<double>::iterator,
typename EdgeRndOutIt = typename std::vector<typename Network::Edge>::iterator>
virtual LinkPred::UCSTPredictor< Network, EdgeRndIt, ScoreRndIt, EdgeRndOutIt >::~UCSTPredictor
( )  [virtual], [default]
```

Destructor.

9.116.3 Member Function Documentation

9.116.3.1 init()

```
template<typename Network = UNetwork<>, typename EdgeRndIt = typename std::vector<typename
Network::Edge>::const_iterator, typename ScoreRndIt = typename std::vector<double>::iterator,
typename EdgeRndOutIt = typename std::vector<typename Network::Edge>::iterator>
virtual void LinkPred::UCSTPredictor< Network, EdgeRndIt, ScoreRndIt, EdgeRndOutIt >::init ( )
[inline], [virtual]
```

Initialize the solver.

Implements LinkPred::ULPredictor< UNetwork<>, typename std::vector< typename UNetwork<> ::Edge >::const_iterator, typena

9.116.3.2 learn()

```
template<typename Network = UNetwork<>, typename EdgeRndIt = typename std::vector<typename
Network::Edge>::const_iterator, typename ScoreRndIt = typename std::vector<double>::iterator,
typename EdgeRndOutIt = typename std::vector<typename Network::Edge>::iterator>
virtual void LinkPred::UCSTPredictor< Network, EdgeRndIt, ScoreRndIt, EdgeRndOutIt >::learn (
) [inline], [virtual]
```

Learn.

Implements LinkPred::ULPredictor< UNetwork<>, typename std::vector< typename UNetwork<> ::Edge >::const_iterator, typena

9.116.3.3 operator=() [1/2]

```
template<typename Network = UNetwork<>, typename EdgeRndIt = typename std::vector<typename
Network::Edge>::const_iterator, typename ScoreRndIt = typename std::vector<double>::iterator,
typename EdgeRndOutIt = typename std::vector<typename Network::Edge>::iterator>
UCSTPredictor& LinkPred::UCSTPredictor< Network, EdgeRndIt, ScoreRndIt, EdgeRndOutIt >::operator=
(
            UCSTPredictor< Network, EdgeRndIt, ScoreRndIt, EdgeRndOutIt > && that )  [default]
```

Move assignment operator.

Parameters

that	The object to move.

9.116.3.4 operator=() [2/2]

```
template<typename Network = UNetwork<>, typename EdgeRndIt = typename std::vector<typename
Network::Edge>::const_iterator, typename ScoreRndIt = typename std::vector<double>::iterator,
typename EdgeRndOutIt = typename std::vector<typename Network::Edge>::iterator>
UCSTPredictor& LinkPred::UCSTPredictor< Network, EdgeRndIt, ScoreRndIt, EdgeRndOutIt >::operator=
(
            UCSTPredictor< Network, EdgeRndIt, ScoreRndIt, EdgeRndOutIt > const & that )
[default]
```

Copy assignment operator.

Parameters

that	The object to copy.

9.116.3.5 predict()

```
template<typename Network = UNetwork<>, typename EdgeRndIt = typename std::vector<typename
Network::Edge>::const_iterator, typename ScoreRndIt = typename std::vector<double>::iterator,
typename EdgeRndOutIt = typename std::vector<typename Network::Edge>::iterator>
virtual void LinkPred::UCSTPredictor< Network, EdgeRndIt, ScoreRndIt, EdgeRndOutIt >::predict
(
            EdgeRndIt begin,
            EdgeRndIt end,
            ScoreRndIt scores )  [virtual]
```

Predict the links.

Parameters

begin	Beginning of the links to be predicted.
end	end of the links to be predicted.
scores	Beginning of scores.

Reimplemented from LinkPred::ULPredictor< UNetwork<>, typename std::vector< typename UNetwork<> ::Edge >::const_iterat

9.116.3.6 score()

```
template<typename Network = UNetwork<>, typename EdgeRndIt = typename std::vector<typename
Network::Edge>::const_iterator, typename ScoreRndIt = typename std::vector<double>::iterator,
typename EdgeRndOutIt = typename std::vector<typename Network::Edge>::iterator>
virtual double LinkPred::UCSTPredictor< Network, EdgeRndIt, ScoreRndIt, EdgeRndOutIt >::score
(
            Edge const & e )  [inline], [virtual]
```

Compute the score of a single edge.

Parameters

e	The edge.

Returns

The score of e.

9.116.3.7 top()

```
template<typename Network = UNetwork<>, typename EdgeRndIt = typename std::vector<typename
Network::Edge>::const_iterator, typename ScoreRndIt = typename std::vector<double>::iterator,
typename EdgeRndOutIt = typename std::vector<typename Network::Edge>::iterator>
```

```
virtual std::size_t LinkPred::UCSTPredictor< Network, EdgeRndIt, ScoreRndIt, EdgeRndOutIt >↩
::top (
            std::size_t k,
            EdgeRndOutIt eit,
            ScoreRndIt sit ) [virtual]
```

Finds the k negative edges with the top score. Ties are broken randomly.

Parameters

k	The number of edges to find.
eit	An output iterator where the edges are written.
sit	An output iterator where the scores are written. The scores are written in the same order as the edges.

Returns

The number of negative edges inserted. It is the minimum between k and the number of negative edges in the network.

Reimplemented from LinkPred::ULPredictor< UNetwork<>, typename std::vector< typename UNetwork<> ::Edge >::const_iterat

The documentation for this class was generated from the following file:

- include/linkpred/predictors/undirected/ucstpredictor.hpp

9.117 LinkPred::UECLPredictor< Network, EdgeRndIt, ScoreRndIt, EdgeRndOutIt > Class Template Reference

Encoder-classifier link predictor.

`#include <ueclpredictor.hpp>`

Inheritance diagram for LinkPred::UECLPredictor< Network, EdgeRndIt, ScoreRndIt, EdgeRndOutIt >:

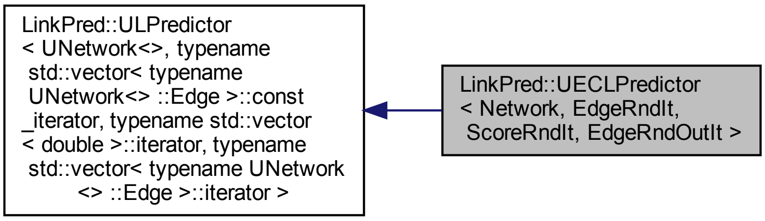

Collaboration diagram for LinkPred::UECLPredictor< Network, EdgeRndIt, ScoreRndIt, EdgeRndOutIt >:

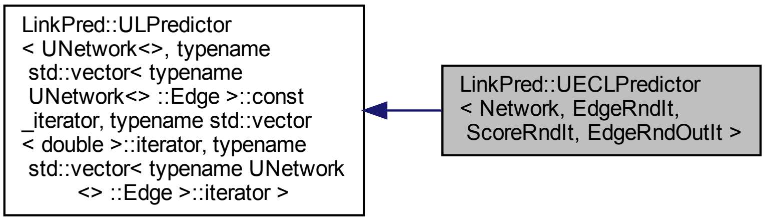

Public Member Functions

- UECLPredictor (std::shared_ptr< Network const > net, std::shared_ptr< Encoder< Network > > encoder, std::shared_ptr< Classifier<> > classifier, long int seed)
- UECLPredictor (UECLPredictor const &that)=default
- UECLPredictor & operator= (UECLPredictor const &that)=default
- UECLPredictor (UECLPredictor &&that)=default
- UECLPredictor & operator= (UECLPredictor &&that)=default
- virtual void init ()
- virtual void learn ()
- virtual double score (Edge const &e)
- const std::shared_ptr< Classifier<> > & getClassifier () const
- const std::shared_ptr< Encoder< Network > > & getEncoder () const
- void setClassifier (const std::shared_ptr< Classifier<> > &classifier)
- void setEncoder (const std::shared_ptr< Encoder< Network > > &encoder)
- double getNegRatio () const
- void setNegRatio (double negRatio)
- double getPosRatio () const
- void setPosRatio (double posRatio)
- virtual ~UECLPredictor ()=default

Additional Inherited Members

9.117.1 Detailed Description

template<typename Network = UNetwork<>, typename EdgeRndIt = typename std::vector<typename Network::Edge>::const_iterator, typename ScoreRndIt = typename std::vector<double>::iterator, typename EdgeRndOutIt = typename std::vector<typename Network::Edge>::iterator>
class LinkPred::UECLPredictor< Network, EdgeRndIt, ScoreRndIt, EdgeRndOutIt >

Encoder-classifier link predictor.

Template Parameters

Network	The network type.
EdgeRndIt	A random iterator type used to iterate on edges.
ScoreRndIt	A random iterator type used to iterate on scores.

9.117.2 Constructor & Destructor Documentation

9.117.2.1 UECLPredictor() [1/3]

```
template<typename Network = UNetwork<>, typename EdgeRndIt = typename std::vector<typename
Network::Edge>::const_iterator, typename ScoreRndIt = typename std::vector<double>::iterator,
typename EdgeRndOutIt = typename std::vector<typename Network::Edge>::iterator>
LinkPred::UECLPredictor< Network, EdgeRndIt, ScoreRndIt, EdgeRndOutIt >::UECLPredictor (
            std::shared_ptr< Network const > net,
            std::shared_ptr< Encoder< Network > > encoder,
            std::shared_ptr< Classifier<> > classifier,
            long int seed ) [inline]
```

Parameters

net	The network.
encoder	The encoder used to embed the network.
classifier	The classifier used to discrminate between positive and negative links.
seed	Seed for the random number generator.

9.117.2.2 UECLPredictor() [2/3]

```
template<typename Network = UNetwork<>, typename EdgeRndIt = typename std::vector<typename
Network::Edge>::const_iterator, typename ScoreRndIt = typename std::vector<double>::iterator,
typename EdgeRndOutIt = typename std::vector<typename Network::Edge>::iterator>
LinkPred::UECLPredictor< Network, EdgeRndIt, ScoreRndIt, EdgeRndOutIt >::UECLPredictor (
            UECLPredictor< Network, EdgeRndIt, ScoreRndIt, EdgeRndOutIt > const & that )
[default]
```

Copy constructor.

Parameters

that	The object to copy.

9.117.2.3 UECLPredictor() [3/3]

```
template<typename Network = UNetwork<>, typename EdgeRndIt = typename std::vector<typename
Network::Edge>::const_iterator, typename ScoreRndIt = typename std::vector<double>::iterator,
typename EdgeRndOutIt = typename std::vector<typename Network::Edge>::iterator>
LinkPred::UECLPredictor< Network, EdgeRndIt, ScoreRndIt, EdgeRndOutIt >::UECLPredictor (
            UECLPredictor< Network, EdgeRndIt, ScoreRndIt, EdgeRndOutIt > && that ) [default]
```

Move constructor.

Parameters

that	The object to move.

9.117.2.4 ∼UECLPredictor()

```
template<typename Network = UNetwork<>, typename EdgeRndIt = typename std::vector<typename
Network::Edge>::const_iterator, typename ScoreRndIt = typename std::vector<double>::iterator,
typename EdgeRndOutIt = typename std::vector<typename Network::Edge>::iterator>
virtual LinkPred::UECLPredictor< Network, EdgeRndIt, ScoreRndIt, EdgeRndOutIt >::∼UECLPredictor
( ) [virtual], [default]
```

Destructor.

9.117.3 Member Function Documentation

9.117.3.1 getClassifier()

```
template<typename Network = UNetwork<>, typename EdgeRndIt = typename std::vector<typename
Network::Edge>::const_iterator, typename ScoreRndIt = typename std::vector<double>::iterator,
typename EdgeRndOutIt = typename std::vector<typename Network::Edge>::iterator>
const std::shared_ptr<Classifier<> >& LinkPred::UECLPredictor< Network, EdgeRndIt, ScoreRndIt,
EdgeRndOutIt >::getClassifier ( ) const [inline]
```

Returns

The classifier.

9.117.3.2 getEncoder()

```
template<typename Network = UNetwork<>, typename EdgeRndIt = typename std::vector<typename
Network::Edge>::const_iterator, typename ScoreRndIt = typename std::vector<double>::iterator,
typename EdgeRndOutIt = typename std::vector<typename Network::Edge>::iterator>
const std::shared_ptr<Encoder<Network> >& LinkPred::UECLPredictor< Network, EdgeRndIt, ScoreRndIt,
EdgeRndOutIt >::getEncoder ( ) const [inline]
```

Returns

The encoder.

9.117.3.3 getNegRatio()

template<typename Network = UNetwork<>, typename EdgeRndIt = typename std::vector<typename Network::Edge>::const_iterator, typename ScoreRndIt = typename std::vector<double>::iterator, typename EdgeRndOutIt = typename std::vector<typename Network::Edge>::iterator>
double LinkPred::UECLPredictor< Network, EdgeRndIt, ScoreRndIt, EdgeRndOutIt >::getNegRatio ( ) const [inline]

Returns

Ratio of negative edges used in the training of the classifier.

9.117.3.4 getPosRatio()

template<typename Network = UNetwork<>, typename EdgeRndIt = typename std::vector<typename Network::Edge>::const_iterator, typename ScoreRndIt = typename std::vector<double>::iterator, typename EdgeRndOutIt = typename std::vector<typename Network::Edge>::iterator>
double LinkPred::UECLPredictor< Network, EdgeRndIt, ScoreRndIt, EdgeRndOutIt >::getPosRatio ( ) const [inline]

Returns

Ratio of positive edges used in the training of the classifier.

9.117.3.5 init()

template<typename Network = UNetwork<>, typename EdgeRndIt = typename std::vector<typename Network::Edge>::const_iterator, typename ScoreRndIt = typename std::vector<double>::iterator, typename EdgeRndOutIt = typename std::vector<typename Network::Edge>::iterator>
virtual void LinkPred::UECLPredictor< Network, EdgeRndIt, ScoreRndIt, EdgeRndOutIt >::init ( ) [virtual]

Initialize the predictor.

Implements LinkPred::ULPredictor< UNetwork<>, typename std::vector< typename UNetwork<> ::Edge >::const_iterator, typena

9.117.3.6 learn()

template<typename Network = UNetwork<>, typename EdgeRndIt = typename std::vector<typename Network::Edge>::const_iterator, typename ScoreRndIt = typename std::vector<double>::iterator, typename EdgeRndOutIt = typename std::vector<typename Network::Edge>::iterator>
virtual void LinkPred::UECLPredictor< Network, EdgeRndIt, ScoreRndIt, EdgeRndOutIt >::learn ( ) [virtual]

Learning.

Implements LinkPred::ULPredictor< UNetwork<>, typename std::vector< typename UNetwork<> ::Edge >::const_iterator, typena

9.117.3.7 operator=() [1/2]

```
template<typename Network = UNetwork<>, typename EdgeRndIt = typename std::vector<typename
Network::Edge>::const_iterator, typename ScoreRndIt = typename std::vector<double>::iterator,
typename EdgeRndOutIt = typename std::vector<typename Network::Edge>::iterator>
UECLPredictor& LinkPred::UECLPredictor< Network, EdgeRndIt, ScoreRndIt, EdgeRndOutIt >::operator=
(
            UECLPredictor< Network, EdgeRndIt, ScoreRndIt, EdgeRndOutIt > && that )  [default]
```

Move assignment operator.

Parameters

that	The object to move.

9.117.3.8 operator=() [2/2]

```
template<typename Network = UNetwork<>, typename EdgeRndIt = typename std::vector<typename
Network::Edge>::const_iterator, typename ScoreRndIt = typename std::vector<double>::iterator,
typename EdgeRndOutIt = typename std::vector<typename Network::Edge>::iterator>
UECLPredictor& LinkPred::UECLPredictor< Network, EdgeRndIt, ScoreRndIt, EdgeRndOutIt >::operator=
(
            UECLPredictor< Network, EdgeRndIt, ScoreRndIt, EdgeRndOutIt > const & that )
[default]
```

Copy assignment operator.

Parameters

that	The object to copy.

9.117.3.9 score()

```
template<typename Network = UNetwork<>, typename EdgeRndIt = typename std::vector<typename
Network::Edge>::const_iterator, typename ScoreRndIt = typename std::vector<double>::iterator,
typename EdgeRndOutIt = typename std::vector<typename Network::Edge>::iterator>
virtual double LinkPred::UECLPredictor< Network, EdgeRndIt, ScoreRndIt, EdgeRndOutIt >::score
(
            Edge const & e )  [virtual]
```

Compute the score of a single edge.

Parameters

e	The edge.

Returns

The score of e.

9.117.3.10 setClassifier()

```
template<typename Network = UNetwork<>, typename EdgeRndIt = typename std::vector<typename
Network::Edge>::const_iterator, typename ScoreRndIt = typename std::vector<double>::iterator,
typename EdgeRndOutIt = typename std::vector<typename Network::Edge>::iterator>
void LinkPred::UECLPredictor< Network, EdgeRndIt, ScoreRndIt, EdgeRndOutIt >::setClassifier (
            const std::shared_ptr< Classifier<> > & classifier ) [inline]
```

Set the classifier.

Parameters

classifier	The new classifier.

9.117.3.11 setEncoder()

```
template<typename Network = UNetwork<>, typename EdgeRndIt = typename std::vector<typename
Network::Edge>::const_iterator, typename ScoreRndIt = typename std::vector<double>::iterator,
typename EdgeRndOutIt = typename std::vector<typename Network::Edge>::iterator>
void LinkPred::UECLPredictor< Network, EdgeRndIt, ScoreRndIt, EdgeRndOutIt >::setEncoder (
            const std::shared_ptr< Encoder< Network > > & encoder ) [inline]
```

Set the encoder.

Parameters

encoder	The new encoder.

9.117.3.12 setNegRatio()

```
template<typename Network = UNetwork<>, typename EdgeRndIt = typename std::vector<typename
Network::Edge>::const_iterator, typename ScoreRndIt = typename std::vector<double>::iterator,
typename EdgeRndOutIt = typename std::vector<typename Network::Edge>::iterator>
void LinkPred::UECLPredictor< Network, EdgeRndIt, ScoreRndIt, EdgeRndOutIt >::setNegRatio (
            double negRatio ) [inline]
```

Set the ratio of negative edges used in the training of the classifier.

Parameters

negRatio	Ratio of negative edges used in the training of the classifier.

9.117.3.13 setPosRatio()

```
template<typename Network = UNetwork<>, typename EdgeRndIt = typename std::vector<typename
Network::Edge>::const_iterator, typename ScoreRndIt = typename std::vector<double>::iterator,
typename EdgeRndOutIt = typename std::vector<typename Network::Edge>::iterator>
void LinkPred::UECLPredictor< Network, EdgeRndIt, ScoreRndIt, EdgeRndOutIt >::setPosRatio (
            double posRatio ) [inline]
```

Set the ratio of positive edges used in the training of the classifier.

Parameters

posRatio	Ratio of positive edges used in the training of the classifier.

The documentation for this class was generated from the following file:

- include/linkpred/predictors/undirected/ueclpredictor.hpp

9.118 LinkPred::UESMPredictor< Network, EdgeRndIt, ScoreRndIt, EdgeRndOutIt > Class Template Reference

Encoder-Similarity measure link predictor.

```
#include <uesmpredictor.hpp>
```

Inheritance diagram for LinkPred::UESMPredictor< Network, EdgeRndIt, ScoreRndIt, EdgeRndOutIt >:

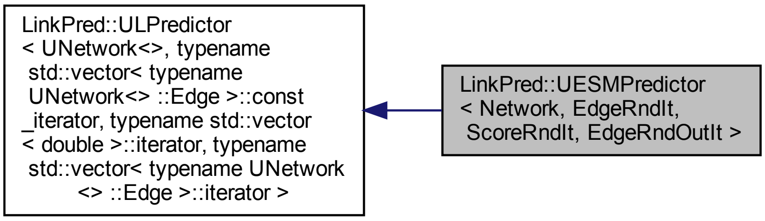

Collaboration diagram for LinkPred::UESMPredictor< Network, EdgeRndIt, ScoreRndIt, EdgeRndOutIt >:

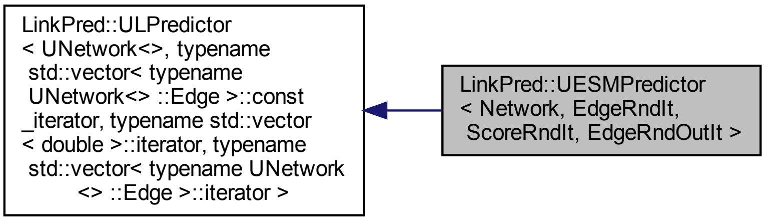

Public Member Functions

- UESMPredictor (std::shared_ptr< Network const > net, std::shared_ptr< Encoder< Network > > encoder, std::shared_ptr< SimMeasure > simMeasure)
- UESMPredictor (UESMPredictor const &that)=default
- UESMPredictor & operator= (UESMPredictor const &that)=default
- UESMPredictor (UESMPredictor &&that)=default
- UESMPredictor & operator= (UESMPredictor &&that)=default
- virtual void init ()
- virtual void learn ()
- virtual double score (Edge const &e)
- const std::shared_ptr< Encoder< Network > > & getEncoder () const
- void setEncoder (const std::shared_ptr< Encoder< Network > > &encoder)
- const std::shared_ptr< SimMeasure > & getSimMeasure () const
- void setSimMeasure (const std::shared_ptr< SimMeasure > &simMeasure)
- virtual ∼UESMPredictor ()=default

Additional Inherited Members

9.118.1 Detailed Description

template<typename Network = UNetwork<>, typename EdgeRndIt = typename std::vector<typename Network::Edge>↩::const_iterator, typename ScoreRndIt = typename std::vector<double>::iterator, typename EdgeRndOutIt = typename std↩::vector<typename Network::Edge>::iterator>
class LinkPred::UESMPredictor< Network, EdgeRndIt, ScoreRndIt, EdgeRndOutIt >

Encoder-Similarity measure link predictor.

Template Parameters

Network	The network type.
Edge↩RndIt	A random iterator type used to iterate on edges.
Score↩RndIt	A random iterator type used to iterate on scores.

9.118.2 Constructor & Destructor Documentation

9.118.2.1 UESMPredictor() [1/3]

```
template<typename Network = UNetwork<>, typename EdgeRndIt = typename std::vector<typename
Network::Edge>::const_iterator, typename ScoreRndIt = typename std::vector<double>::iterator,
typename EdgeRndOutIt = typename std::vector<typename Network::Edge>::iterator>
LinkPred::UESMPredictor< Network, EdgeRndIt, ScoreRndIt, EdgeRndOutIt >::UESMPredictor (
            std::shared_ptr< Network const > net,
            std::shared_ptr< Encoder< Network > > encoder,
            std::shared_ptr< SimMeasure > simMeasure )  [inline]
```

Parameters

net	The network.
encoder	The encoder used to embed the network.
simMeasure	The similarity measure.

9.118.2.2 UESMPredictor() [2/3]

```
template<typename Network = UNetwork<>, typename EdgeRndIt = typename std::vector<typename
Network::Edge>::const_iterator, typename ScoreRndIt = typename std::vector<double>::iterator,
typename EdgeRndOutIt = typename std::vector<typename Network::Edge>::iterator>
LinkPred::UESMPredictor< Network, EdgeRndIt, ScoreRndIt, EdgeRndOutIt >::UESMPredictor (
            UESMPredictor< Network, EdgeRndIt, ScoreRndIt, EdgeRndOutIt > const & that )
[default]
```

Copy constructor.

Parameters

that	The object to copy.

9.118.2.3 UESMPredictor() [3/3]

```
template<typename Network = UNetwork<>, typename EdgeRndIt = typename std::vector<typename
Network::Edge>::const_iterator, typename ScoreRndIt = typename std::vector<double>::iterator,
typename EdgeRndOutIt = typename std::vector<typename Network::Edge>::iterator>
LinkPred::UESMPredictor< Network, EdgeRndIt, ScoreRndIt, EdgeRndOutIt >::UESMPredictor (
            UESMPredictor< Network, EdgeRndIt, ScoreRndIt, EdgeRndOutIt > && that )  [default]
```

Move constructor.

Parameters

that	The object to move.

9.118.2.4 ∼UESMPredictor()

```
template<typename Network = UNetwork<>, typename EdgeRndIt = typename std::vector<typename
Network::Edge>::const_iterator, typename ScoreRndIt = typename std::vector<double>::iterator,
typename EdgeRndOutIt = typename std::vector<typename Network::Edge>::iterator>
virtual LinkPred::UESMPredictor< Network, EdgeRndIt, ScoreRndIt, EdgeRndOutIt >::∼UESMPredictor
( ) [virtual], [default]
```

Destructor.

9.118.3 Member Function Documentation

9.118.3.1 getEncoder()

```
template<typename Network = UNetwork<>, typename EdgeRndIt = typename std::vector<typename
Network::Edge>::const_iterator, typename ScoreRndIt = typename std::vector<double>::iterator,
typename EdgeRndOutIt = typename std::vector<typename Network::Edge>::iterator>
const std::shared_ptr<Encoder<Network> >& LinkPred::UESMPredictor< Network, EdgeRndIt, ScoreRndIt,
EdgeRndOutIt >::getEncoder ( ) const [inline]
```

Returns

The encoder.

9.118.3.2 getSimMeasure()

```
template<typename Network = UNetwork<>, typename EdgeRndIt = typename std::vector<typename
Network::Edge>::const_iterator, typename ScoreRndIt = typename std::vector<double>::iterator,
typename EdgeRndOutIt = typename std::vector<typename Network::Edge>::iterator>
const std::shared_ptr<SimMeasure>& LinkPred::UESMPredictor< Network, EdgeRndIt, ScoreRndIt,
EdgeRndOutIt >::getSimMeasure ( ) const [inline]
```

Returns

The similarity measure.

9.118.3.3 init()

```
template<typename Network = UNetwork<>, typename EdgeRndIt = typename std::vector<typename
Network::Edge>::const_iterator, typename ScoreRndIt = typename std::vector<double>::iterator,
typename EdgeRndOutIt = typename std::vector<typename Network::Edge>::iterator>
virtual void LinkPred::UESMPredictor< Network, EdgeRndIt, ScoreRndIt, EdgeRndOutIt >::init ( )
[virtual]
```

Initialize the predictor.

Implements LinkPred::ULPredictor< UNetwork<>, typename std::vector< typename UNetwork<> ::Edge >::const_iterator, typena

9.118.3.4 learn()

```
template<typename Network = UNetwork<>, typename EdgeRndIt = typename std::vector<typename
Network::Edge>::const_iterator, typename ScoreRndIt = typename std::vector<double>::iterator,
typename EdgeRndOutIt = typename std::vector<typename Network::Edge>::iterator>
virtual void LinkPred::UESMPredictor< Network, EdgeRndIt, ScoreRndIt, EdgeRndOutIt >::learn (
) [virtual]
```

Learning.

Implements LinkPred::ULPredictor< UNetwork<>, typename std::vector< typename UNetwork<> ::Edge >::const_iterator, typena

9.118.3.5 operator=() [1/2]

```
template<typename Network = UNetwork<>, typename EdgeRndIt = typename std::vector<typename
Network::Edge>::const_iterator, typename ScoreRndIt = typename std::vector<double>::iterator,
typename EdgeRndOutIt = typename std::vector<typename Network::Edge>::iterator>
UESMPredictor& LinkPred::UESMPredictor< Network, EdgeRndIt, ScoreRndIt, EdgeRndOutIt >::operator=
(
            UESMPredictor< Network, EdgeRndIt, ScoreRndIt, EdgeRndOutIt > && that )  [default]
```

Move assignment operator.

Parameters

that	The object to move.

9.118.3.6 operator=() [2/2]

```
template<typename Network = UNetwork<>, typename EdgeRndIt = typename std::vector<typename
Network::Edge>::const_iterator, typename ScoreRndIt = typename std::vector<double>::iterator,
typename EdgeRndOutIt = typename std::vector<typename Network::Edge>::iterator>
```

```
UESMPredictor& LinkPred::UESMPredictor< Network, EdgeRndIt, ScoreRndIt, EdgeRndOutIt >::operator=
(
            UESMPredictor< Network, EdgeRndIt, ScoreRndIt, EdgeRndOutIt > const & that )
[default]
```

Copy assignment operator.

Parameters

that	The object to copy.

9.118.3.7 score()

```
template<typename Network = UNetwork<>, typename EdgeRndIt = typename std::vector<typename
Network::Edge>::const_iterator, typename ScoreRndIt = typename std::vector<double>::iterator,
typename EdgeRndOutIt = typename std::vector<typename Network::Edge>::iterator>
virtual double LinkPred::UESMPredictor< Network, EdgeRndIt, ScoreRndIt, EdgeRndOutIt >::score
(
            Edge const & e )  [virtual]
```

Compute the score of a single edge.

Parameters

e	The edge.

Returns

The score of e.

9.118.3.8 setEncoder()

```
template<typename Network = UNetwork<>, typename EdgeRndIt = typename std::vector<typename
Network::Edge>::const_iterator, typename ScoreRndIt = typename std::vector<double>::iterator,
typename EdgeRndOutIt = typename std::vector<typename Network::Edge>::iterator>
void LinkPred::UESMPredictor< Network, EdgeRndIt, ScoreRndIt, EdgeRndOutIt >::setEncoder (
            const std::shared_ptr< Encoder< Network > > & encoder )  [inline]
```

Set the encoder.

Parameters

encoder	The new encoder.

9.118.3.9 setSimMeasure()

```
template<typename Network = UNetwork<>, typename EdgeRndIt = typename std::vector<typename
Network::Edge>::const_iterator, typename ScoreRndIt = typename std::vector<double>::iterator,
typename EdgeRndOutIt = typename std::vector<typename Network::Edge>::iterator>
void LinkPred::UESMPredictor< Network, EdgeRndIt, ScoreRndIt, EdgeRndOutIt >::setSimMeasure (
            const std::shared_ptr< SimMeasure > & simMeasure )  [inline]
```

Set the similarity measure.

Parameters

simMeasure	The new similarity measure.

The documentation for this class was generated from the following file:

- include/linkpred/predictors/undirected/uesmpredictor.hpp

9.119 LinkPred::UFBMPredictor< Network, EdgeRndIt, ScoreRndIt, EdgeRndOutIt > Class Template Reference

Fast blocking model link predictor.

```
#include <ufbmpredictor.hpp>
```

Inheritance diagram for LinkPred::UFBMPredictor< Network, EdgeRndIt, ScoreRndIt, EdgeRndOutIt >:

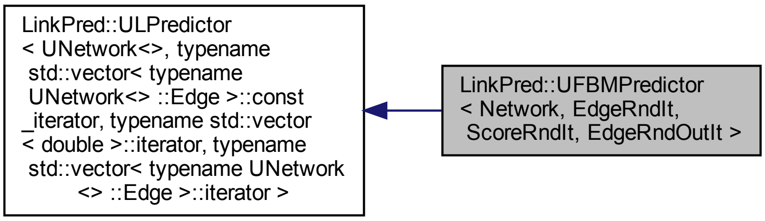

Collaboration diagram for LinkPred::UFBMPredictor< Network, EdgeRndIt, ScoreRndIt, EdgeRndOutIt >:

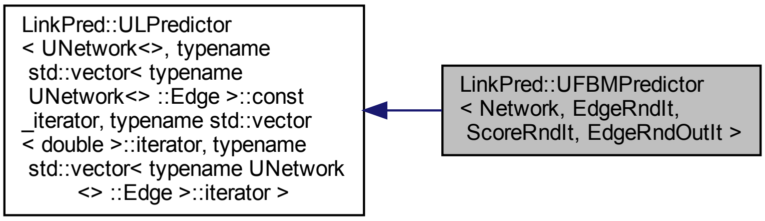

Public Member Functions

- UFBMPredictor (std::shared_ptr< Network const > net, long int seed)
- UFBMPredictor (UFBMPredictor const &that)=default
- UFBMPredictor & operator= (UFBMPredictor const &that)=default
- UFBMPredictor (UFBMPredictor &&that)=default
- UFBMPredictor & operator= (UFBMPredictor &&that)=default
- virtual void init ()
- virtual void learn ()
- virtual double score (Edge const &e)
- virtual void predict (EdgeRndIt begin, EdgeRndIt end, ScoreRndIt scores)
- std::size_t getMaxIter () const
- void setMaxIter (std::size_t maxIter)
- virtual ∼UFBMPredictor ()=default

Additional Inherited Members

9.119.1 Detailed Description

template<typename Network = UNetwork<>, typename EdgeRndIt = typename std::vector<typename Network::Edge>↩ ::const_iterator, typename ScoreRndIt = typename std::vector<double>::iterator, typename EdgeRndOutIt = typename std↩ ::vector<typename Network::Edge>::iterator>
class LinkPred::UFBMPredictor< Network, EdgeRndIt, ScoreRndIt, EdgeRndOutIt >

Fast blocking model link predictor.

This is a C++ translation of the Matlab code provided by the authors.

Template Parameters

Network	The network type.
Edge↩ RndIt	A random iterator type used to iterate on edges.
Score↩ RndIt	A random iterator type used to iterate on scores.

9.119.2 Constructor & Destructor Documentation

9.119.2.1 UFBMPredictor() [1/3]

```
template<typename Network = UNetwork<>, typename EdgeRndIt = typename std::vector<typename
Network::Edge>::const_iterator, typename ScoreRndIt = typename std::vector<double>::iterator,
typename EdgeRndOutIt = typename std::vector<typename Network::Edge>::iterator>
LinkPred::UFBMPredictor< Network, EdgeRndIt, ScoreRndIt, EdgeRndOutIt >::UFBMPredictor (
            std::shared_ptr< Network const > net,
            long int seed ) [inline]
```

Parameters

net	The network.
seed	Random number generator seed.

9.119.2.2 UFBMPredictor() [2/3]

```
template<typename Network = UNetwork<>, typename EdgeRndIt = typename std::vector<typename
Network::Edge>::const_iterator, typename ScoreRndIt = typename std::vector<double>::iterator,
typename EdgeRndOutIt = typename std::vector<typename Network::Edge>::iterator>
LinkPred::UFBMPredictor< Network, EdgeRndIt, ScoreRndIt, EdgeRndOutIt >::UFBMPredictor (
            UFBMPredictor< Network, EdgeRndIt, ScoreRndIt, EdgeRndOutIt > const & that )
[default]
```

Copy constructor.

Parameters

that	The object to copy.

9.119.2.3 UFBMPredictor() [3/3]

```
template<typename Network = UNetwork<>, typename EdgeRndIt = typename std::vector<typename
Network::Edge>::const_iterator, typename ScoreRndIt = typename std::vector<double>::iterator,
typename EdgeRndOutIt = typename std::vector<typename Network::Edge>::iterator>
LinkPred::UFBMPredictor< Network, EdgeRndIt, ScoreRndIt, EdgeRndOutIt >::UFBMPredictor (
            UFBMPredictor< Network, EdgeRndIt, ScoreRndIt, EdgeRndOutIt > && that )  [default]
```

Move constructor.

Parameters

that	The object to move.

9.119.2.4 ∼UFBMPredictor()

```
template<typename Network = UNetwork<>, typename EdgeRndIt = typename std::vector<typename
Network::Edge>::const_iterator, typename ScoreRndIt = typename std::vector<double>::iterator,
typename EdgeRndOutIt = typename std::vector<typename Network::Edge>::iterator>
virtual LinkPred::UFBMPredictor< Network, EdgeRndIt, ScoreRndIt, EdgeRndOutIt >::~UFBMPredictor
( )  [virtual], [default]
```

Destructor.

9.119.3 Member Function Documentation

9.119.3.1 getMaxIter()

```
template<typename Network = UNetwork<>, typename EdgeRndIt = typename std::vector<typename
Network::Edge>::const_iterator, typename ScoreRndIt = typename std::vector<double>::iterator,
typename EdgeRndOutIt = typename std::vector<typename Network::Edge>::iterator>
std::size_t LinkPred::UFBMPredictor< Network, EdgeRndIt, ScoreRndIt, EdgeRndOutIt >::getMax↩
Iter ( ) const [inline]
```

Returns

The maximum number of iterations.

9.119.3.2 init()

```
template<typename Network = UNetwork<>, typename EdgeRndIt = typename std::vector<typename
Network::Edge>::const_iterator, typename ScoreRndIt = typename std::vector<double>::iterator,
typename EdgeRndOutIt = typename std::vector<typename Network::Edge>::iterator>
virtual void LinkPred::UFBMPredictor< Network, EdgeRndIt, ScoreRndIt, EdgeRndOutIt >::init ( )
[virtual]
```

Initialize the solver.

Implements LinkPred::ULPredictor< UNetwork<>, typename std::vector< typename UNetwork<> ::Edge >::const_iterator, typena

9.119.3.3 learn()

```
template<typename Network = UNetwork<>, typename EdgeRndIt = typename std::vector<typename
Network::Edge>::const_iterator, typename ScoreRndIt = typename std::vector<double>::iterator,
typename EdgeRndOutIt = typename std::vector<typename Network::Edge>::iterator>
virtual void LinkPred::UFBMPredictor< Network, EdgeRndIt, ScoreRndIt, EdgeRndOutIt >::learn (
) [virtual]
```

Learn.

Implements LinkPred::ULPredictor< UNetwork<>, typename std::vector< typename UNetwork<> ::Edge >::const_iterator, typena

9.119.3.4 operator=() [1/2]

```
template<typename Network = UNetwork<>, typename EdgeRndIt = typename std::vector<typename
Network::Edge>::const_iterator, typename ScoreRndIt = typename std::vector<double>::iterator,
typename EdgeRndOutIt = typename std::vector<typename Network::Edge>::iterator>
UFBMPredictor& LinkPred::UFBMPredictor< Network, EdgeRndIt, ScoreRndIt, EdgeRndOutIt >::operator=
(
            UFBMPredictor< Network, EdgeRndIt, ScoreRndIt, EdgeRndOutIt > && that ) [default]
```

Move assignment operator.

Parameters

that	The object to move.

9.119.3.5 operator=() [2/2]

```
template<typename Network = UNetwork<>, typename EdgeRndIt = typename std::vector<typename
Network::Edge>::const_iterator, typename ScoreRndIt = typename std::vector<double>::iterator,
typename EdgeRndOutIt = typename std::vector<typename Network::Edge>::iterator>
UFBMPredictor& LinkPred::UFBMPredictor< Network, EdgeRndIt, ScoreRndIt, EdgeRndOutIt >::operator=
(
            UFBMPredictor< Network, EdgeRndIt, ScoreRndIt, EdgeRndOutIt > const & that )
[default]
```

Copy assignment operator.

Parameters

that	The object to copy.

9.119.3.6 predict()

```
template<typename Network = UNetwork<>, typename EdgeRndIt = typename std::vector<typename
Network::Edge>::const_iterator, typename ScoreRndIt = typename std::vector<double>::iterator,
typename EdgeRndOutIt = typename std::vector<typename Network::Edge>::iterator>
virtual void LinkPred::UFBMPredictor< Network, EdgeRndIt, ScoreRndIt, EdgeRndOutIt >::predict
(
            EdgeRndIt begin,
            EdgeRndIt end,
            ScoreRndIt scores )  [virtual]
```

Predict the links.

Parameters

begin	Beginning of the links to be predicted.
end	end of the links to be predicted.
scores	Beginning of scores.

Reimplemented from LinkPred::ULPredictor< UNetwork<>, typename std::vector< typename UNetwork<> ::Edge >::const_iterat

9.119.3.7 score()

```
template<typename Network = UNetwork<>, typename EdgeRndIt = typename std::vector<typename
Network::Edge>::const_iterator, typename ScoreRndIt = typename std::vector<double>::iterator,
```

```
typename EdgeRndOutIt = typename std::vector<typename Network::Edge>::iterator>
virtual double LinkPred::UFBMPredictor< Network, EdgeRndIt, ScoreRndIt, EdgeRndOutIt >::score
(
            Edge const & e )  [inline], [virtual]
```

Compute the score of a single edge.

Parameters

e	The edge.

Returns

The score of e.

9.119.3.8 setMaxIter()

```
template<typename Network = UNetwork<>, typename EdgeRndIt = typename std::vector<typename
Network::Edge>::const_iterator, typename ScoreRndIt = typename std::vector<double>::iterator,
typename EdgeRndOutIt = typename std::vector<typename Network::Edge>::iterator>
void LinkPred::UFBMPredictor< Network, EdgeRndIt, ScoreRndIt, EdgeRndOutIt >::setMaxIter (
            std::size_t maxIter )  [inline]
```

Set the maximum number of iterations.

Parameters

maxIter	The new maximum number of iterations.

The documentation for this class was generated from the following file:

- include/linkpred/predictors/undirected/ufbmpredictor.hpp

9.120 LinkPred::UHDIPredictor< Network, EdgeRndIt, ScoreRndIt, EdgeRndOutIt > Class Template Reference

Hub depromoted index link predictor.

```
#include <uhdipredictor.hpp>
```

Inheritance diagram for LinkPred::UHDIPredictor< Network, EdgeRndIt, ScoreRndIt, EdgeRndOutIt >:

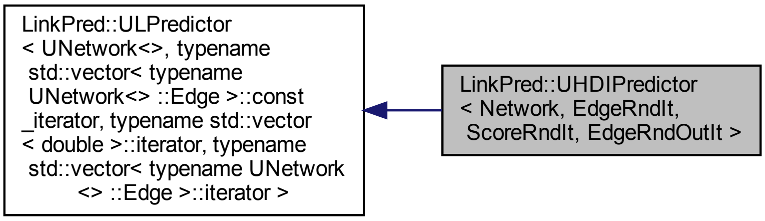

Collaboration diagram for LinkPred::UHDIPredictor< Network, EdgeRndIt, ScoreRndIt, EdgeRndOutIt >:

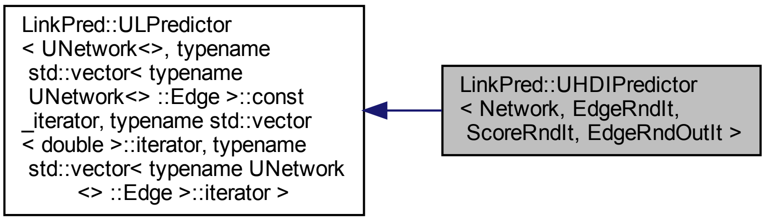

Public Member Functions

- UHDIPredictor (std::shared_ptr< Network const > net)
- UHDIPredictor (UHDIPredictor const &that)=default
- UHDIPredictor & operator= (UHDIPredictor const &that)=default
- UHDIPredictor (UHDIPredictor &&that)=default
- UHDIPredictor & operator= (UHDIPredictor &&that)=default
- virtual void init ()
- virtual void learn ()
- virtual double score (Edge const &e)
- virtual void predict (EdgeRndIt begin, EdgeRndIt end, ScoreRndIt scores)
- virtual std::size_t top (std::size_t k, EdgeRndOutIt eit, ScoreRndIt sit)
- virtual ~UHDIPredictor ()=default

Additional Inherited Members

9.120.1 Detailed Description

template<typename Network = UNetwork<>, typename EdgeRndIt = typename std::vector<typename Network::Edge>↩ ::const_iterator, typename ScoreRndIt = typename std::vector<double>::iterator, typename EdgeRndOutIt = typename std↩ ::vector<typename Network::Edge>::iterator>
class LinkPred::UHDIPredictor< Network, EdgeRndIt, ScoreRndIt, EdgeRndOutIt >

Hub depromoted index link predictor.

Template Parameters

Network	The network type.
Edge↩ RndIt	A random iterator type used to iterate on edges.
Score↩ RndIt	A random iterator type used to iterate on scores.

9.120.2 Constructor & Destructor Documentation

9.120.2.1 UHDIPredictor() [1/3]

```
template<typename Network = UNetwork<>, typename EdgeRndIt = typename std::vector<typename
Network::Edge>::const_iterator, typename ScoreRndIt = typename std::vector<double>::iterator,
typename EdgeRndOutIt = typename std::vector<typename Network::Edge>::iterator>
LinkPred::UHDIPredictor< Network, EdgeRndIt, ScoreRndIt, EdgeRndOutIt >::UHDIPredictor (
            std::shared_ptr< Network const > net ) [inline]
```

Parameters

net	The network.

9.120.2.2 UHDIPredictor() [2/3]

```
template<typename Network = UNetwork<>, typename EdgeRndIt = typename std::vector<typename
Network::Edge>::const_iterator, typename ScoreRndIt = typename std::vector<double>::iterator,
typename EdgeRndOutIt = typename std::vector<typename Network::Edge>::iterator>
LinkPred::UHDIPredictor< Network, EdgeRndIt, ScoreRndIt, EdgeRndOutIt >::UHDIPredictor (
            UHDIPredictor< Network, EdgeRndIt, ScoreRndIt, EdgeRndOutIt > const & that )
[default]
```

Copy constructor.

Parameters

that	The object to copy.

9.120.2.3 UHDIPredictor() [3/3]

```
template<typename Network = UNetwork<>, typename EdgeRndIt = typename std::vector<typename
Network::Edge>::const_iterator, typename ScoreRndIt = typename std::vector<double>::iterator,
```

```
typename EdgeRndOutIt = typename std::vector<typename Network::Edge>::iterator>
LinkPred::UHDIPredictor< Network, EdgeRndIt, ScoreRndIt, EdgeRndOutIt >::UHDIPredictor (
            UHDIPredictor< Network, EdgeRndIt, ScoreRndIt, EdgeRndOutIt > && that )  [default]
```

Move constructor.

Parameters

that	The object to move.

9.120.2.4 ∼UHDIPredictor()

```
template<typename Network = UNetwork<>, typename EdgeRndIt = typename std::vector<typename
Network::Edge>::const_iterator, typename ScoreRndIt = typename std::vector<double>::iterator,
typename EdgeRndOutIt = typename std::vector<typename Network::Edge>::iterator>
virtual LinkPred::UHDIPredictor< Network, EdgeRndIt, ScoreRndIt, EdgeRndOutIt >::~UHDIPredictor
( )  [virtual], [default]
```

Destructor.

9.120.3 Member Function Documentation

9.120.3.1 init()

```
template<typename Network = UNetwork<>, typename EdgeRndIt = typename std::vector<typename
Network::Edge>::const_iterator, typename ScoreRndIt = typename std::vector<double>::iterator,
typename EdgeRndOutIt = typename std::vector<typename Network::Edge>::iterator>
virtual void LinkPred::UHDIPredictor< Network, EdgeRndIt, ScoreRndIt, EdgeRndOutIt >::init ( )
[inline], [virtual]
```

Initialize the predictor.

Implements LinkPred::ULPredictor< UNetwork<>, typename std::vector< typename UNetwork<> ::Edge >::const_iterator, typena

9.120.3.2 learn()

```
template<typename Network = UNetwork<>, typename EdgeRndIt = typename std::vector<typename
Network::Edge>::const_iterator, typename ScoreRndIt = typename std::vector<double>::iterator,
typename EdgeRndOutIt = typename std::vector<typename Network::Edge>::iterator>
virtual void LinkPred::UHDIPredictor< Network, EdgeRndIt, ScoreRndIt, EdgeRndOutIt >::learn (
)  [inline], [virtual]
```

Learning.

Implements LinkPred::ULPredictor< UNetwork<>, typename std::vector< typename UNetwork<> ::Edge >::const_iterator, typena

9.120.3.3 operator=() [1/2]

```
template<typename Network = UNetwork<>, typename EdgeRndIt = typename std::vector<typename
Network::Edge>::const_iterator, typename ScoreRndIt = typename std::vector<double>::iterator,
typename EdgeRndOutIt = typename std::vector<typename Network::Edge>::iterator>
UHDIPredictor& LinkPred::UHDIPredictor< Network, EdgeRndIt, ScoreRndIt, EdgeRndOutIt >::operator=
(
            UHDIPredictor< Network, EdgeRndIt, ScoreRndIt, EdgeRndOutIt > && that )  [default]
```

Move assignment operator.

Parameters

that	The object to move.

9.120.3.4 operator=() [2/2]

```
template<typename Network = UNetwork<>, typename EdgeRndIt = typename std::vector<typename
Network::Edge>::const_iterator, typename ScoreRndIt = typename std::vector<double>::iterator,
typename EdgeRndOutIt = typename std::vector<typename Network::Edge>::iterator>
UHDIPredictor& LinkPred::UHDIPredictor< Network, EdgeRndIt, ScoreRndIt, EdgeRndOutIt >::operator=
(
            UHDIPredictor< Network, EdgeRndIt, ScoreRndIt, EdgeRndOutIt > const & that )
[default]
```

Copy assignment operator.

Parameters

that	The object to copy.

9.120.3.5 predict()

```
template<typename Network = UNetwork<>, typename EdgeRndIt = typename std::vector<typename
Network::Edge>::const_iterator, typename ScoreRndIt = typename std::vector<double>::iterator,
typename EdgeRndOutIt = typename std::vector<typename Network::Edge>::iterator>
virtual void LinkPred::UHDIPredictor< Network, EdgeRndIt, ScoreRndIt, EdgeRndOutIt >::predict
(
            EdgeRndIt begin,
            EdgeRndIt end,
            ScoreRndIt scores )  [virtual]
```

Predict the links.

Parameters

begin	Beginning of the links to be predicted.
end	end of the links to be predicted.
scores	Beginning of scores.

Reimplemented from LinkPred::ULPredictor< UNetwork<>, typename std::vector< typename UNetwork<> ::Edge >::const_iterato

9.120.3.6 score()

```
template<typename Network = UNetwork<>, typename EdgeRndIt = typename std::vector<typename
Network::Edge>::const_iterator, typename ScoreRndIt = typename std::vector<double>::iterator,
typename EdgeRndOutIt = typename std::vector<typename Network::Edge>::iterator>
virtual double LinkPred::UHDIPredictor< Network, EdgeRndIt, ScoreRndIt, EdgeRndOutIt >::score
(
            Edge const & e )  [inline], [virtual]
```

Compute the score of a single edge.

Parameters

e	The edge.

Returns

The score of e.

9.120.3.7 top()

```
template<typename Network = UNetwork<>, typename EdgeRndIt = typename std::vector<typename
Network::Edge>::const_iterator, typename ScoreRndIt = typename std::vector<double>::iterator,
typename EdgeRndOutIt = typename std::vector<typename Network::Edge>::iterator>
virtual std::size_t LinkPred::UHDIPredictor< Network, EdgeRndIt, ScoreRndIt, EdgeRndOutIt >↩
::top (
            std::size_t k,
            EdgeRndOutIt eit,
            ScoreRndIt sit )  [virtual]
```

Finds the k negative edges with the top score. Ties are broken randmly.

Parameters

k	The number of edges to find.
eit	An output iterator where the edges are written.
sit	An output iterator where the scores are written. The scores are written in the same order as the edges.

Returns

The number of negative edges inserted. It is the minimum between k and the number of negative edges in the network.

Reimplemented from LinkPred::ULPredictor< UNetwork<>, typename std::vector< typename UNetwork<> ::Edge >::const_iterato

The documentation for this class was generated from the following file:

- include/linkpred/predictors/undirected/uhdipredictor.hpp

9.121 LinkPred::UHPIPredictor< Network, EdgeRndIt, ScoreRndIt, EdgeRndOutIt > Class Template Reference

Hub promoted index link predictor.

```
#include <uhpipredictor.hpp>
```

Inheritance diagram for LinkPred::UHPIPredictor< Network, EdgeRndIt, ScoreRndIt, EdgeRndOutIt >:

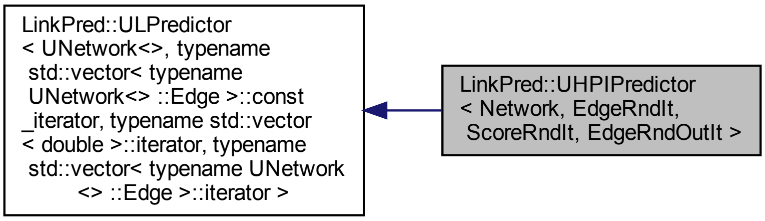

Collaboration diagram for LinkPred::UHPIPredictor< Network, EdgeRndIt, ScoreRndIt, EdgeRndOutIt >:

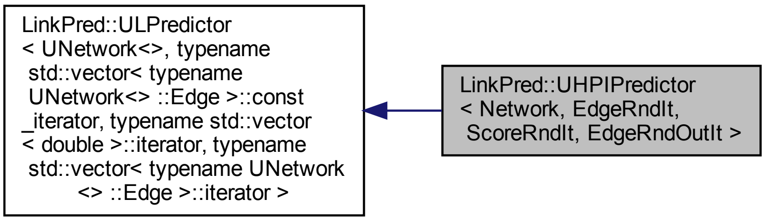

Public Member Functions

- UHPIPredictor (std::shared_ptr< Network const > net)
- UHPIPredictor (UHPIPredictor const &that)=default
- UHPIPredictor & operator= (UHPIPredictor const &that)=default
- UHPIPredictor (UHPIPredictor &&that)=default
- UHPIPredictor & operator= (UHPIPredictor &&that)=default
- virtual void init ()
- virtual void learn ()
- virtual double score (Edge const &e)
- virtual void predict (EdgeRndIt begin, EdgeRndIt end, ScoreRndIt scores)
- virtual std::size_t top (std::size_t k, EdgeRndOutIt eit, ScoreRndIt sit)
- virtual ~UHPIPredictor ()=default

Additional Inherited Members

9.121.1 Detailed Description

template<typename Network = UNetwork<>, typename EdgeRndIt = typename std::vector<typename Network::Edge>::const_iterator, typename ScoreRndIt = typename std::vector<double>::iterator, typename EdgeRndOutIt = typename std::vector<typename Network::Edge>::iterator>
class LinkPred::UHPIPredictor< Network, EdgeRndIt, ScoreRndIt, EdgeRndOutIt >

Hub promoted index link predictor.

Template Parameters

Network	The network type.
EdgeRndIt	A random iterator type used to iterate on edges.
ScoreRndIt	A random iterator type used to iterate on scores.

9.121.2 Constructor & Destructor Documentation

9.121.2.1 UHPIPredictor() [1/3]

```
template<typename Network = UNetwork<>, typename EdgeRndIt = typename std::vector<typename
Network::Edge>::const_iterator, typename ScoreRndIt = typename std::vector<double>::iterator,
typename EdgeRndOutIt = typename std::vector<typename Network::Edge>::iterator>
LinkPred::UHPIPredictor< Network, EdgeRndIt, ScoreRndIt, EdgeRndOutIt >::UHPIPredictor (
            std::shared_ptr< Network const > net )  [inline]
```

Parameters

net	The network.

9.121.2.2 UHPIPredictor() [2/3]

```
template<typename Network = UNetwork<>, typename EdgeRndIt = typename std::vector<typename
Network::Edge>::const_iterator, typename ScoreRndIt = typename std::vector<double>::iterator,
typename EdgeRndOutIt = typename std::vector<typename Network::Edge>::iterator>
LinkPred::UHPIPredictor< Network, EdgeRndIt, ScoreRndIt, EdgeRndOutIt >::UHPIPredictor (
            UHPIPredictor< Network, EdgeRndIt, ScoreRndIt, EdgeRndOutIt > const & that )
[default]
```

Copy constructor.

Parameters

that	The object to copy.

9.121.2.3 UHPIPredictor() [3/3]

```
template<typename Network = UNetwork<>, typename EdgeRndIt = typename std::vector<typename
Network::Edge>::const_iterator, typename ScoreRndIt = typename std::vector<double>::iterator,
typename EdgeRndOutIt = typename std::vector<typename Network::Edge>::iterator>
LinkPred::UHPIPredictor< Network, EdgeRndIt, ScoreRndIt, EdgeRndOutIt >::UHPIPredictor (
            UHPIPredictor< Network, EdgeRndIt, ScoreRndIt, EdgeRndOutIt > && that )  [default]
```

Move constructor.

Parameters

that	The object to move.

9.121.2.4 ∼UHPIPredictor()

```
template<typename Network = UNetwork<>, typename EdgeRndIt = typename std::vector<typename
Network::Edge>::const_iterator, typename ScoreRndIt = typename std::vector<double>::iterator,
typename EdgeRndOutIt = typename std::vector<typename Network::Edge>::iterator>
virtual LinkPred::UHPIPredictor< Network, EdgeRndIt, ScoreRndIt, EdgeRndOutIt >::~UHPIPredictor
( )  [virtual], [default]
```

Destructor.

9.121.3 Member Function Documentation

9.121.3.1 init()

```
template<typename Network = UNetwork<>, typename EdgeRndIt = typename std::vector<typename
Network::Edge>::const_iterator, typename ScoreRndIt = typename std::vector<double>::iterator,
typename EdgeRndOutIt = typename std::vector<typename Network::Edge>::iterator>
virtual void LinkPred::UHPIPredictor< Network, EdgeRndIt, ScoreRndIt, EdgeRndOutIt >::init ( )
[inline], [virtual]
```

Initialize the predictor.

Implements LinkPred::ULPredictor< UNetwork<>, typename std::vector< typename UNetwork<> ::Edge >::const_iterator, typena

9.121.3.2 learn()

```
template<typename Network = UNetwork<>, typename EdgeRndIt = typename std::vector<typename
Network::Edge>::const_iterator, typename ScoreRndIt = typename std::vector<double>::iterator,
typename EdgeRndOutIt = typename std::vector<typename Network::Edge>::iterator>
virtual void LinkPred::UHPIPredictor< Network, EdgeRndIt, ScoreRndIt, EdgeRndOutIt >::learn (
) [inline], [virtual]
```

Learning.

Implements LinkPred::ULPredictor< UNetwork<>, typename std::vector< typename UNetwork<> ::Edge >::const_iterator, typena

9.121.3.3 operator=() [1/2]

```
template<typename Network = UNetwork<>, typename EdgeRndIt = typename std::vector<typename
Network::Edge>::const_iterator, typename ScoreRndIt = typename std::vector<double>::iterator,
typename EdgeRndOutIt = typename std::vector<typename Network::Edge>::iterator>
UHPIPredictor& LinkPred::UHPIPredictor< Network, EdgeRndIt, ScoreRndIt, EdgeRndOutIt >::operator=
(
            UHPIPredictor< Network, EdgeRndIt, ScoreRndIt, EdgeRndOutIt > && that ) [default]
```

Move assignment operator.

Parameters

that	The object to move.

9.121.3.4 operator=() [2/2]

```
template<typename Network = UNetwork<>, typename EdgeRndIt = typename std::vector<typename
Network::Edge>::const_iterator, typename ScoreRndIt = typename std::vector<double>::iterator,
typename EdgeRndOutIt = typename std::vector<typename Network::Edge>::iterator>
UHPIPredictor& LinkPred::UHPIPredictor< Network, EdgeRndIt, ScoreRndIt, EdgeRndOutIt >::operator=
(
            UHPIPredictor< Network, EdgeRndIt, ScoreRndIt, EdgeRndOutIt > const & that )
[default]
```

Copy assignment operator.

Parameters

that	The object to copy.

9.121.3.5 predict()

```
template<typename Network = UNetwork<>, typename EdgeRndIt = typename std::vector<typename
Network::Edge>::const_iterator, typename ScoreRndIt = typename std::vector<double>::iterator,
typename EdgeRndOutIt = typename std::vector<typename Network::Edge>::iterator>
virtual void LinkPred::UHPIPredictor< Network, EdgeRndIt, ScoreRndIt, EdgeRndOutIt >::predict
(
            EdgeRndIt begin,
            EdgeRndIt end,
            ScoreRndIt scores )  [virtual]
```

Predict the links.

Parameters

begin	Beginning of the links to be predicted.
end	end of the links to be predicted.
scores	Beginning of scores.

Reimplemented from LinkPred::ULPredictor< UNetwork<>, typename std::vector< typename UNetwork<> ::Edge >::const_iterat

9.121.3.6 score()

```
template<typename Network = UNetwork<>, typename EdgeRndIt = typename std::vector<typename
Network::Edge>::const_iterator, typename ScoreRndIt = typename std::vector<double>::iterator,
typename EdgeRndOutIt = typename std::vector<typename Network::Edge>::iterator>
virtual double LinkPred::UHPIPredictor< Network, EdgeRndIt, ScoreRndIt, EdgeRndOutIt >::score
(
            Edge const & e )  [inline], [virtual]
```

Compute the score of a single edge.

Parameters

e	The edge.

Returns

The score of e.

9.121.3.7 top()

```
template<typename Network = UNetwork<>, typename EdgeRndIt = typename std::vector<typename
Network::Edge>::const_iterator, typename ScoreRndIt = typename std::vector<double>::iterator,
typename EdgeRndOutIt = typename std::vector<typename Network::Edge>::iterator>
```

```
virtual std::size_t LinkPred::UHPIPredictor< Network, EdgeRndIt, ScoreRndIt, EdgeRndOutIt >↩
::top (
            std::size_t k,
            EdgeRndOutIt eit,
            ScoreRndIt sit )  [virtual]
```

Finds the k negative edges with the top score. Ties are broken randmly.

Parameters

k	The number of edges to find.
eit	An output iterator where the edges are written.
sit	An output iterator where the scores are written. The scores are written in the same order as the edges.

Returns

The number of negative edges inserted. It is the minimum between k and the number of negative edges in the network.

Reimplemented from LinkPred::ULPredictor< UNetwork<>, typename std::vector< typename UNetwork<> ::Edge >::const_iterat

The documentation for this class was generated from the following file:

- include/linkpred/predictors/undirected/uhpipredictor.hpp

9.122 LinkPred::UHRGPredictor< Network, EdgeRndIt, ScoreRndIt, EdgeRndOutIt > Class Template Reference

HRG predictor.

```
#include <uhrgpredictor.hpp>
```

Inheritance diagram for LinkPred::UHRGPredictor< Network, EdgeRndIt, ScoreRndIt, EdgeRndOutIt >:

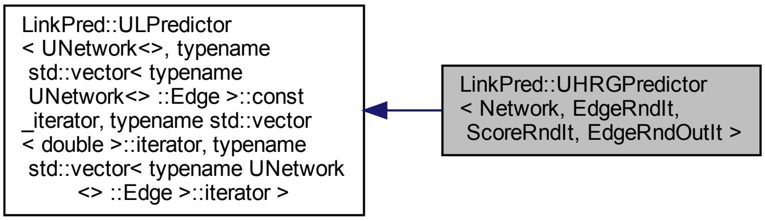

Collaboration diagram for LinkPred::UHRGPredictor< Network, EdgeRndIt, ScoreRndIt, EdgeRndOutIt >:

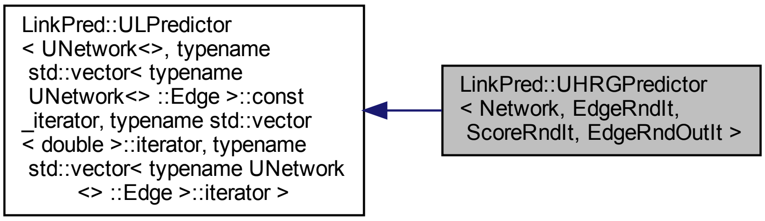

Public Member Functions

- UHRGPredictor (std::shared_ptr< Network const > net, long int seed)
- UHRGPredictor (UHRGPredictor const &that)=delete
- UHRGPredictor & operator= (UHRGPredictor const &that)=delete
- UHRGPredictor (UHRGPredictor &&that)=delete
- UHRGPredictor & operator= (UHRGPredictor &&that)=delete
- virtual void init ()
- virtual void learn ()
- virtual double score (Edge const &e)
- virtual void predict (EdgeRndIt begin, EdgeRndIt end, ScoreRndIt scores)
- int getNbBeans () const
- void setNbBeans (int nbBeans)
- int getNbSamples () const
- void setNbSamples (int nbSamples)
- virtual ~UHRGPredictor ()

Additional Inherited Members

9.122.1 Detailed Description

template<typename Network = UNetwork<>, typename EdgeRndIt = typename std::vector<typename Network::Edge>↩ ::const_iterator, typename ScoreRndIt = typename std::vector<double>::iterator, typename EdgeRndOutIt = typename std↩ ::vector<typename Network::Edge>::iterator>
class LinkPred::UHRGPredictor< Network, EdgeRndIt, ScoreRndIt, EdgeRndOutIt >

HRG predictor.

This is actually a modified and wrapped version the code provided by the authors.

Template Parameters

Network	The network type.
Edge↩ RndIt	A random iterator type used to iterate on edges.
Score↩ RndIt	A random iterator type used to iterate on scores.

9.122.2 Constructor & Destructor Documentation

9.122.2.1 UHRGPredictor() [1/3]

```
template<typename Network = UNetwork<>, typename EdgeRndIt = typename std::vector<typename
Network::Edge>::const_iterator, typename ScoreRndIt = typename std::vector<double>::iterator,
typename EdgeRndOutIt = typename std::vector<typename Network::Edge>::iterator>
LinkPred::UHRGPredictor< Network, EdgeRndIt, ScoreRndIt, EdgeRndOutIt >::UHRGPredictor (
            std::shared_ptr< Network const > net,
            long int seed ) [inline]
```

Parameters

net	The network.
seed	The random number generator's seed.

9.122.2.2 UHRGPredictor() [2/3]

```
template<typename Network = UNetwork<>, typename EdgeRndIt = typename std::vector<typename
Network::Edge>::const_iterator, typename ScoreRndIt = typename std::vector<double>::iterator,
typename EdgeRndOutIt = typename std::vector<typename Network::Edge>::iterator>
LinkPred::UHRGPredictor< Network, EdgeRndIt, ScoreRndIt, EdgeRndOutIt >::UHRGPredictor (
            UHRGPredictor< Network, EdgeRndIt, ScoreRndIt, EdgeRndOutIt > const & that )
[delete]
```

Copy constructor.

9.122.2.3 UHRGPredictor() [3/3]

```
template<typename Network = UNetwork<>, typename EdgeRndIt = typename std::vector<typename
Network::Edge>::const_iterator, typename ScoreRndIt = typename std::vector<double>::iterator,
typename EdgeRndOutIt = typename std::vector<typename Network::Edge>::iterator>
LinkPred::UHRGPredictor< Network, EdgeRndIt, ScoreRndIt, EdgeRndOutIt >::UHRGPredictor (
            UHRGPredictor< Network, EdgeRndIt, ScoreRndIt, EdgeRndOutIt > && that ) [delete]
```

Move constructor.

9.122.2.4 ∼UHRGPredictor()

```
template<typename Network = UNetwork<>, typename EdgeRndIt = typename std::vector<typename
Network::Edge>::const_iterator, typename ScoreRndIt = typename std::vector<double>::iterator,
typename EdgeRndOutIt = typename std::vector<typename Network::Edge>::iterator>
virtual LinkPred::UHRGPredictor< Network, EdgeRndIt, ScoreRndIt, EdgeRndOutIt >::~UHRGPredictor
( ) [virtual]
```

Destructor.

9.122.3 Member Function Documentation

9.122.3.1 getNbBeans()

```
template<typename Network = UNetwork<>, typename EdgeRndIt = typename std::vector<typename
Network::Edge>::const_iterator, typename ScoreRndIt = typename std::vector<double>::iterator,
typename EdgeRndOutIt = typename std::vector<typename Network::Edge>::iterator>
int LinkPred::UHRGPredictor< Network, EdgeRndIt, ScoreRndIt, EdgeRndOutIt >::getNbBeans ( )
const [inline]
```

Returns

The number of bins.

9.122.3.2 getNbSamples()

```
template<typename Network = UNetwork<>, typename EdgeRndIt = typename std::vector<typename
Network::Edge>::const_iterator, typename ScoreRndIt = typename std::vector<double>::iterator,
typename EdgeRndOutIt = typename std::vector<typename Network::Edge>::iterator>
int LinkPred::UHRGPredictor< Network, EdgeRndIt, ScoreRndIt, EdgeRndOutIt >::getNbSamples ( )
const [inline]
```

Returns

The number of samples.

9.122.3.3 init()

```
template<typename Network = UNetwork<>, typename EdgeRndIt = typename std::vector<typename
Network::Edge>::const_iterator, typename ScoreRndIt = typename std::vector<double>::iterator,
typename EdgeRndOutIt = typename std::vector<typename Network::Edge>::iterator>
virtual void LinkPred::UHRGPredictor< Network, EdgeRndIt, ScoreRndIt, EdgeRndOutIt >::init ( )
[virtual]
```

Initialize the solver.

Implements LinkPred::ULPredictor< UNetwork<>, typename std::vector< typename UNetwork<> ::Edge >::const_iterator, typena

9.122.3.4 learn()

```
template<typename Network = UNetwork<>, typename EdgeRndIt = typename std::vector<typename
Network::Edge>::const_iterator, typename ScoreRndIt = typename std::vector<double>::iterator,
typename EdgeRndOutIt = typename std::vector<typename Network::Edge>::iterator>
virtual void LinkPred::UHRGPredictor< Network, EdgeRndIt, ScoreRndIt, EdgeRndOutIt >::learn (
) [virtual]
```

Learning.

Implements LinkPred::ULPredictor< UNetwork<>, typename std::vector< typename UNetwork<> ::Edge >::const_iterator, typena

9.122.3.5 operator=() [1/2]

```
template<typename Network = UNetwork<>, typename EdgeRndIt = typename std::vector<typename
Network::Edge>::const_iterator, typename ScoreRndIt = typename std::vector<double>::iterator,
typename EdgeRndOutIt = typename std::vector<typename Network::Edge>::iterator>
UHRGPredictor& LinkPred::UHRGPredictor< Network, EdgeRndIt, ScoreRndIt, EdgeRndOutIt >::operator=
(
            UHRGPredictor< Network, EdgeRndIt, ScoreRndIt, EdgeRndOutIt > && that )  [delete]
```

Move assignment operator.

9.122.3.6 operator=() [2/2]

```
template<typename Network = UNetwork<>, typename EdgeRndIt = typename std::vector<typename
Network::Edge>::const_iterator, typename ScoreRndIt = typename std::vector<double>::iterator,
typename EdgeRndOutIt = typename std::vector<typename Network::Edge>::iterator>
UHRGPredictor& LinkPred::UHRGPredictor< Network, EdgeRndIt, ScoreRndIt, EdgeRndOutIt >::operator=
(
            UHRGPredictor< Network, EdgeRndIt, ScoreRndIt, EdgeRndOutIt > const & that )
[delete]
```

Copy assignment operator.

9.122.3.7 predict()

```
template<typename Network = UNetwork<>, typename EdgeRndIt = typename std::vector<typename
Network::Edge>::const_iterator, typename ScoreRndIt = typename std::vector<double>::iterator,
typename EdgeRndOutIt = typename std::vector<typename Network::Edge>::iterator>
virtual void LinkPred::UHRGPredictor< Network, EdgeRndIt, ScoreRndIt, EdgeRndOutIt >::predict
(
            EdgeRndIt begin,
            EdgeRndIt end,
            ScoreRndIt scores )  [virtual]
```

Predict the links.

Parameters

begin	Beginning of the links to be predicted.
end	end of the links to be predicted.
scores	Beginning of scores.

Reimplemented from LinkPred::ULPredictor< UNetwork<>, typename std::vector< typename UNetwork<> ::Edge >::const_iterat

9.122.3.8 score()

```
template<typename Network = UNetwork<>, typename EdgeRndIt = typename std::vector<typename
Network::Edge>::const_iterator, typename ScoreRndIt = typename std::vector<double>::iterator,
typename EdgeRndOutIt = typename std::vector<typename Network::Edge>::iterator>
virtual double LinkPred::UHRGPredictor< Network, EdgeRndIt, ScoreRndIt, EdgeRndOutIt >::score
(
            Edge const & e )  [inline], [virtual]
```

Compute the score of a single edge.

Parameters

e	The edge.

Returns

The score of e.

9.122.3.9 setNbBeans()

```
template<typename Network = UNetwork<>, typename EdgeRndIt = typename std::vector<typename
Network::Edge>::const_iterator, typename ScoreRndIt = typename std::vector<double>::iterator,
typename EdgeRndOutIt = typename std::vector<typename Network::Edge>::iterator>
void LinkPred::UHRGPredictor< Network, EdgeRndIt, ScoreRndIt, EdgeRndOutIt >::setNbBeans (
            int nbBeans )  [inline]
```

Set the number of bins.

Parameters

nbBeans	The new number of bins.

9.122.3.10 setNbSamples()

```
template<typename Network = UNetwork<>, typename EdgeRndIt = typename std::vector<typename
Network::Edge>::const_iterator, typename ScoreRndIt = typename std::vector<double>::iterator,
typename EdgeRndOutIt = typename std::vector<typename Network::Edge>::iterator>
void LinkPred::UHRGPredictor< Network, EdgeRndIt, ScoreRndIt, EdgeRndOutIt >::setNbSamples (
            int nbSamples )  [inline]
```

Set the number of samples.

Parameters

nbSamples	The new number of samples.

The documentation for this class was generated from the following file:

- include/linkpred/predictors/undirected/uhrgpredictor.hpp

9.123 LinkPred::UHYPPredictor< Network, EdgeRndIt, ScoreRndIt, EdgeRndOutIt > Class Template Reference

Hypermap predictor.

```
#include <uhyppredictor.hpp>
```

Inheritance diagram for LinkPred::UHYPPredictor< Network, EdgeRndIt, ScoreRndIt, EdgeRndOutIt >:

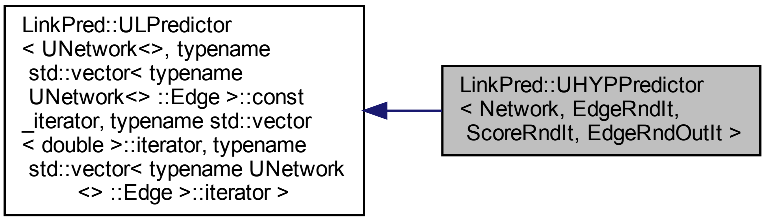

Collaboration diagram for LinkPred::UHYPPredictor< Network, EdgeRndIt, ScoreRndIt, EdgeRndOutIt >:

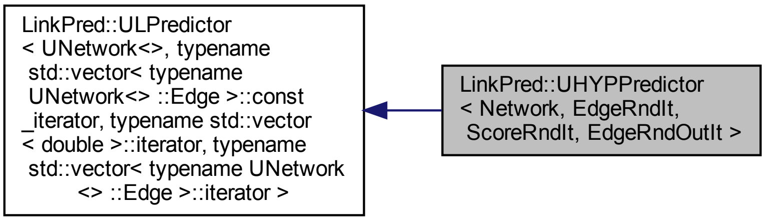

Public Member Functions

- UHYPPredictor (std::shared_ptr< Network const > net, long int seed)
- UHYPPredictor (UHYPPredictor const &that)=delete
- UHYPPredictor & operator= (UHYPPredictor const &that)=delete
- UHYPPredictor (UHYPPredictor &&that)=delete
- UHYPPredictor & operator= (UHYPPredictor &&that)=delete
- virtual void init ()
- virtual void learn ()
- virtual double score (Edge const &e)
- virtual void predict (EdgeRndIt begin, EdgeRndIt end, ScoreRndIt scores)
- long int getSeed () const
- double getGamma () const
- void setGamma (double gamma)
- double getL () const
- void setL (double L)
- double getM () const
- void setM (double m)
- double getT () const
- void setT (double T)
- double getZeta () const
- void setZeta (double zeta)
- virtual ~UHYPPredictor ()=default

Additional Inherited Members

9.123.1 Detailed Description

template<typename Network = UNetwork<>, typename EdgeRndIt = typename std::vector<typename Network::Edge>::const_iterator, typename ScoreRndIt = typename std::vector<double>::iterator, typename EdgeRndOutIt = typename std::vector<typename Network::Edge>::iterator>
class LinkPred::UHYPPredictor< Network, EdgeRndIt, ScoreRndIt, EdgeRndOutIt >

Hypermap predictor.

This is a modified and wrapped version of the code provided by the authors.

Template Parameters

Network	The network type.
EdgeRndIt	A random iterator type used to iterate on edges.
ScoreRndIt	A random iterator type used to iterate on scores.

9.123.2 Constructor & Destructor Documentation

9.123.2.1 UHYPPredictor() [1/3]

```
template<typename Network = UNetwork<>, typename EdgeRndIt = typename std::vector<typename
Network::Edge>::const_iterator, typename ScoreRndIt = typename std::vector<double>::iterator,
typename EdgeRndOutIt = typename std::vector<typename Network::Edge>::iterator>
LinkPred::UHYPPredictor< Network, EdgeRndIt, ScoreRndIt, EdgeRndOutIt >::UHYPPredictor (
            std::shared_ptr< Network const > net,
            long int seed ) [inline]
```

Parameters

net	The network.
seed	The random number generator's seed.

9.123.2.2 UHYPPredictor() [2/3]

```
template<typename Network = UNetwork<>, typename EdgeRndIt = typename std::vector<typename
Network::Edge>::const_iterator, typename ScoreRndIt = typename std::vector<double>::iterator,
typename EdgeRndOutIt = typename std::vector<typename Network::Edge>::iterator>
LinkPred::UHYPPredictor< Network, EdgeRndIt, ScoreRndIt, EdgeRndOutIt >::UHYPPredictor (
            UHYPPredictor< Network, EdgeRndIt, ScoreRndIt, EdgeRndOutIt > const & that )
[delete]
```

Copy constructor.

Parameters

that	The object to copy.

9.123.2.3 UHYPPredictor() [3/3]

```
template<typename Network = UNetwork<>, typename EdgeRndIt = typename std::vector<typename
Network::Edge>::const_iterator, typename ScoreRndIt = typename std::vector<double>::iterator,
typename EdgeRndOutIt = typename std::vector<typename Network::Edge>::iterator>
LinkPred::UHYPPredictor< Network, EdgeRndIt, ScoreRndIt, EdgeRndOutIt >::UHYPPredictor (
            UHYPPredictor< Network, EdgeRndIt, ScoreRndIt, EdgeRndOutIt > && that ) [delete]
```

Move constructor.

Parameters

that	The object to move.

9.123.2.4 ∼UHYPPredictor()

template<typename Network = UNetwork<>, typename EdgeRndIt = typename std::vector<typename Network::Edge>::const_iterator, typename ScoreRndIt = typename std::vector<double>::iterator, typename EdgeRndOutIt = typename std::vector<typename Network::Edge>::iterator>
virtual LinkPred::UHYPPredictor< Network, EdgeRndIt, ScoreRndIt, EdgeRndOutIt >::∼UHYPPredictor ( ) [virtual], [default]

Destructor.

9.123.3 Member Function Documentation

9.123.3.1 getGamma()

template<typename Network = UNetwork<>, typename EdgeRndIt = typename std::vector<typename Network::Edge>::const_iterator, typename ScoreRndIt = typename std::vector<double>::iterator, typename EdgeRndOutIt = typename std::vector<typename Network::Edge>::iterator>
double LinkPred::UHYPPredictor< Network, EdgeRndIt, ScoreRndIt, EdgeRndOutIt >::getGamma ( ) const [inline]

Returns

The power law exponent gamma (see the algorithm description).

9.123.3.2 getL()

template<typename Network = UNetwork<>, typename EdgeRndIt = typename std::vector<typename Network::Edge>::const_iterator, typename ScoreRndIt = typename std::vector<double>::iterator, typename EdgeRndOutIt = typename std::vector<typename Network::Edge>::iterator>
double LinkPred::UHYPPredictor< Network, EdgeRndIt, ScoreRndIt, EdgeRndOutIt >::getL ( ) const [inline]

Returns

The parameter L (see the algorithm description).

9.123.3.3 getM()

template<typename Network = UNetwork<>, typename EdgeRndIt = typename std::vector<typename Network::Edge>::const_iterator, typename ScoreRndIt = typename std::vector<double>::iterator, typename EdgeRndOutIt = typename std::vector<typename Network::Edge>::iterator>
double LinkPred::UHYPPredictor< Network, EdgeRndIt, ScoreRndIt, EdgeRndOutIt >::getM ( ) const [inline]

Returns

The parameter m (see the algorithm description).

9.123.3.4 getSeed()

```
template<typename Network = UNetwork<>, typename EdgeRndIt = typename std::vector<typename
Network::Edge>::const_iterator, typename ScoreRndIt = typename std::vector<double>::iterator,
typename EdgeRndOutIt = typename std::vector<typename Network::Edge>::iterator>
long int LinkPred::UHYPPredictor< Network, EdgeRndIt, ScoreRndIt, EdgeRndOutIt >::getSeed ( )
const [inline]
```

Returns

The random number generator seed.

9.123.3.5 getT()

```
template<typename Network = UNetwork<>, typename EdgeRndIt = typename std::vector<typename
Network::Edge>::const_iterator, typename ScoreRndIt = typename std::vector<double>::iterator,
typename EdgeRndOutIt = typename std::vector<typename Network::Edge>::iterator>
double LinkPred::UHYPPredictor< Network, EdgeRndIt, ScoreRndIt, EdgeRndOutIt >::getT ( ) const
[inline]
```

Returns

The parameter L (see the algorithm description).

9.123.3.6 getZeta()

```
template<typename Network = UNetwork<>, typename EdgeRndIt = typename std::vector<typename
Network::Edge>::const_iterator, typename ScoreRndIt = typename std::vector<double>::iterator,
typename EdgeRndOutIt = typename std::vector<typename Network::Edge>::iterator>
double LinkPred::UHYPPredictor< Network, EdgeRndIt, ScoreRndIt, EdgeRndOutIt >::getZeta ( )
const [inline]
```

Returns

The parameter zeta (see the algorithm description).

9.123.3.7 init()

```
template<typename Network = UNetwork<>, typename EdgeRndIt = typename std::vector<typename
Network::Edge>::const_iterator, typename ScoreRndIt = typename std::vector<double>::iterator,
typename EdgeRndOutIt = typename std::vector<typename Network::Edge>::iterator>
virtual void LinkPred::UHYPPredictor< Network, EdgeRndIt, ScoreRndIt, EdgeRndOutIt >::init ( )
[virtual]
```

Initialize the solver.

Implements LinkPred::ULPredictor< UNetwork<>, typename std::vector< typename UNetwork<> ::Edge >::const_iterator, typena

9.123.3.8 learn()

```
template<typename Network = UNetwork<>, typename EdgeRndIt = typename std::vector<typename
Network::Edge>::const_iterator, typename ScoreRndIt = typename std::vector<double>::iterator,
typename EdgeRndOutIt = typename std::vector<typename Network::Edge>::iterator>
virtual void LinkPred::UHYPPredictor< Network, EdgeRndIt, ScoreRndIt, EdgeRndOutIt >::learn (
) [virtual]
```

Learning.

Implements LinkPred::ULPredictor< UNetwork<>, typename std::vector< typename UNetwork<> ::Edge >::const_iterator, typena

9.123.3.9 operator=() [1/2]

```
template<typename Network = UNetwork<>, typename EdgeRndIt = typename std::vector<typename
Network::Edge>::const_iterator, typename ScoreRndIt = typename std::vector<double>::iterator,
typename EdgeRndOutIt = typename std::vector<typename Network::Edge>::iterator>
UHYPPredictor& LinkPred::UHYPPredictor< Network, EdgeRndIt, ScoreRndIt, EdgeRndOutIt >::operator=
(
            UHYPPredictor< Network, EdgeRndIt, ScoreRndIt, EdgeRndOutIt > && that )  [delete]
```

Move assignment operator.

Parameters

that	The object to move.

9.123.3.10 operator=() [2/2]

```
template<typename Network = UNetwork<>, typename EdgeRndIt = typename std::vector<typename
Network::Edge>::const_iterator, typename ScoreRndIt = typename std::vector<double>::iterator,
typename EdgeRndOutIt = typename std::vector<typename Network::Edge>::iterator>
UHYPPredictor& LinkPred::UHYPPredictor< Network, EdgeRndIt, ScoreRndIt, EdgeRndOutIt >::operator=
(
            UHYPPredictor< Network, EdgeRndIt, ScoreRndIt, EdgeRndOutIt > const & that )
[delete]
```

Copy assignment operator.

Parameters

that	The object to copy.

9.123.3.11 predict()

```
template<typename Network = UNetwork<>, typename EdgeRndIt = typename std::vector<typename
Network::Edge>::const_iterator, typename ScoreRndIt = typename std::vector<double>::iterator,
typename EdgeRndOutIt = typename std::vector<typename Network::Edge>::iterator>
virtual void LinkPred::UHYPPredictor< Network, EdgeRndIt, ScoreRndIt, EdgeRndOutIt >::predict
(
            EdgeRndIt begin,
            EdgeRndIt end,
            ScoreRndIt scores )  [virtual]
```

Predict the links.

Parameters

begin	Beginning of the links to be predicted.
end	end of the links to be predicted.
scores	Beginning of scores.

Reimplemented from LinkPred::ULPredictor< UNetwork<>, typename std::vector< typename UNetwork<> ::Edge >::const_iterat

9.123.3.12 score()

```
template<typename Network = UNetwork<>, typename EdgeRndIt = typename std::vector<typename
Network::Edge>::const_iterator, typename ScoreRndIt = typename std::vector<double>::iterator,
typename EdgeRndOutIt = typename std::vector<typename Network::Edge>::iterator>
virtual double LinkPred::UHYPPredictor< Network, EdgeRndIt, ScoreRndIt, EdgeRndOutIt >::score
(
            Edge const & e )  [inline], [virtual]
```

Compute the score of a single edge.

Parameters

e	The edge.

Returns

The score of e.

9.123.3.13 setGamma()

```
template<typename Network = UNetwork<>, typename EdgeRndIt = typename std::vector<typename
Network::Edge>::const_iterator, typename ScoreRndIt = typename std::vector<double>::iterator,
typename EdgeRndOutIt = typename std::vector<typename Network::Edge>::iterator>
void LinkPred::UHYPPredictor< Network, EdgeRndIt, ScoreRndIt, EdgeRndOutIt >::setGamma (
            double gamma )  [inline]
```

Set the power law exponent gamma (see the algorithm description).

Parameters

gamma	The new power law exponent gamma (see the algorithm description).

9.123.3.14 setL()

```
template<typename Network = UNetwork<>, typename EdgeRndIt = typename std::vector<typename
Network::Edge>::const_iterator, typename ScoreRndIt = typename std::vector<double>::iterator,
typename EdgeRndOutIt = typename std::vector<typename Network::Edge>::iterator>
void LinkPred::UHYPPredictor< Network, EdgeRndIt, ScoreRndIt, EdgeRndOutIt >::setL (
            double L ) [inline]
```

Set the parameter L (see the algorithm description).

Parameters

L	The new value of the parameter L (see the algorithm description).

9.123.3.15 setM()

```
template<typename Network = UNetwork<>, typename EdgeRndIt = typename std::vector<typename
Network::Edge>::const_iterator, typename ScoreRndIt = typename std::vector<double>::iterator,
typename EdgeRndOutIt = typename std::vector<typename Network::Edge>::iterator>
void LinkPred::UHYPPredictor< Network, EdgeRndIt, ScoreRndIt, EdgeRndOutIt >::setM (
            double m ) [inline]
```

Set the parameter m (see the algorithm description).

Parameters

m	The new value of the parameter m (see the algorithm description).

9.123.3.16 setT()

```
template<typename Network = UNetwork<>, typename EdgeRndIt = typename std::vector<typename
Network::Edge>::const_iterator, typename ScoreRndIt = typename std::vector<double>::iterator,
typename EdgeRndOutIt = typename std::vector<typename Network::Edge>::iterator>
void LinkPred::UHYPPredictor< Network, EdgeRndIt, ScoreRndIt, EdgeRndOutIt >::setT (
            double T ) [inline]
```

Set the parameter L (see the algorithm description).

Parameters

T	The new value of the parameter L (see the algorithm description).

9.123.3.17 setZeta()

```
template<typename Network = UNetwork<>, typename EdgeRndIt = typename std::vector<typename
Network::Edge>::const_iterator, typename ScoreRndIt = typename std::vector<double>::iterator,
typename EdgeRndOutIt = typename std::vector<typename Network::Edge>::iterator>
void LinkPred::UHYPPredictor< Network, EdgeRndIt, ScoreRndIt, EdgeRndOutIt >::setZeta (
            double zeta )  [inline]
```

Set the parameter zeta (see the algorithm description).

Parameters

zeta	The new value of the parameter zeta (see the algorithm description).

The documentation for this class was generated from the following file:

- include/linkpred/predictors/undirected/uhyppredictor.hpp

9.124 LinkPred::UJIDPredictor< Network, EdgeRndIt, ScoreRndIt, EdgeRndOutIt > Class Template Reference

Jackard index link predictor.

```
#include <ujidpredictor.hpp>
```

Inheritance diagram for LinkPred::UJIDPredictor< Network, EdgeRndIt, ScoreRndIt, EdgeRndOutIt >:

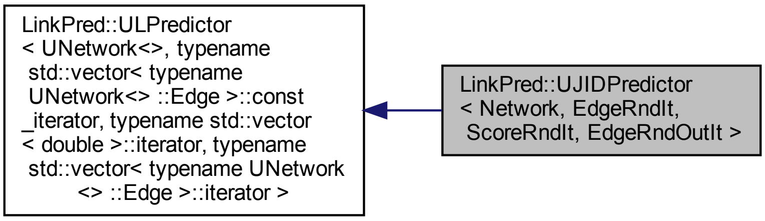

Collaboration diagram for LinkPred::UJIDPredictor< Network, EdgeRndIt, ScoreRndIt, EdgeRndOutIt >:

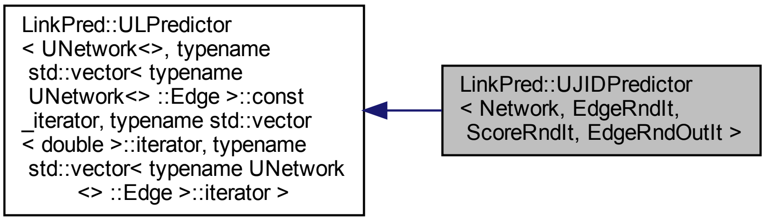

Public Member Functions

- UJIDPredictor (std::shared_ptr< Network const > net)
- UJIDPredictor (UJIDPredictor const &that)
- UJIDPredictor & operator= (UJIDPredictor const &that)
- UJIDPredictor (UJIDPredictor &&that)
- UJIDPredictor & operator= (UJIDPredictor &&that)
- virtual void init ()
- virtual void learn ()
- virtual double score (Edge const &e)
- virtual void predict (EdgeRndIt begin, EdgeRndIt end, ScoreRndIt scores)
- virtual std::size_t top (std::size_t k, EdgeRndOutIt eit, ScoreRndIt sit)
- virtual ~UJIDPredictor ()=default

Additional Inherited Members

9.124.1 Detailed Description

template<typename Network = UNetwork<>, typename EdgeRndIt = typename std::vector<typename Network::Edge>↩ ::const_iterator, typename ScoreRndIt = typename std::vector<double>::iterator, typename EdgeRndOutIt = typename std↩ ::vector<typename Network::Edge>::iterator>
class LinkPred::UJIDPredictor< Network, EdgeRndIt, ScoreRndIt, EdgeRndOutIt >

Jackard index link predictor.

Template Parameters

Network	The network type.
Edge↩ RndIt	A random iterator type used to iterate on edges.
Score↩ RndIt	A random iterator type used to iterate on scores.

9.124.2 Constructor & Destructor Documentation

9.124.2.1 UJIDPredictor() [1/3]

```
template<typename Network = UNetwork<>, typename EdgeRndIt = typename std::vector<typename
Network::Edge>::const_iterator, typename ScoreRndIt = typename std::vector<double>::iterator,
typename EdgeRndOutIt = typename std::vector<typename Network::Edge>::iterator>
LinkPred::UJIDPredictor< Network, EdgeRndIt, ScoreRndIt, EdgeRndOutIt >::UJIDPredictor (
            std::shared_ptr< Network const > net ) [inline]
```

Parameters

net	The network.

9.124.2.2 UJIDPredictor() [2/3]

```
template<typename Network = UNetwork<>, typename EdgeRndIt = typename std::vector<typename
Network::Edge>::const_iterator, typename ScoreRndIt = typename std::vector<double>::iterator,
typename EdgeRndOutIt = typename std::vector<typename Network::Edge>::iterator>
LinkPred::UJIDPredictor< Network, EdgeRndIt, ScoreRndIt, EdgeRndOutIt >::UJIDPredictor (
            UJIDPredictor< Network, EdgeRndIt, ScoreRndIt, EdgeRndOutIt > const & that )
```

Copy constructor.

Parameters

that	The object to copy.

9.124.2.3 UJIDPredictor() [3/3]

```
template<typename Network = UNetwork<>, typename EdgeRndIt = typename std::vector<typename
Network::Edge>::const_iterator, typename ScoreRndIt = typename std::vector<double>::iterator,
typename EdgeRndOutIt = typename std::vector<typename Network::Edge>::iterator>
LinkPred::UJIDPredictor< Network, EdgeRndIt, ScoreRndIt, EdgeRndOutIt >::UJIDPredictor (
            UJIDPredictor< Network, EdgeRndIt, ScoreRndIt, EdgeRndOutIt > && that )
```

Move constructor.

Parameters

that	The object to move.

9.124.2.4 ∼UJIDPredictor()

```
template<typename Network = UNetwork<>, typename EdgeRndIt = typename std::vector<typename
Network::Edge>::const_iterator, typename ScoreRndIt = typename std::vector<double>::iterator,
typename EdgeRndOutIt = typename std::vector<typename Network::Edge>::iterator>
virtual LinkPred::UJIDPredictor< Network, EdgeRndIt, ScoreRndIt, EdgeRndOutIt >::∼UJIDPredictor
( ) [virtual], [default]
```

Destructor.

9.124.3 Member Function Documentation

9.124.3.1 init()

```
template<typename Network = UNetwork<>, typename EdgeRndIt = typename std::vector<typename
Network::Edge>::const_iterator, typename ScoreRndIt = typename std::vector<double>::iterator,
typename EdgeRndOutIt = typename std::vector<typename Network::Edge>::iterator>
virtual void LinkPred::UJIDPredictor< Network, EdgeRndIt, ScoreRndIt, EdgeRndOutIt >::init ( )
[inline], [virtual]
```

Initialize the solver.

Implements LinkPred::ULPredictor< UNetwork<>, typename std::vector< typename UNetwork<> ::Edge >::const_iterator, typena

9.124.3.2 learn()

```
template<typename Network = UNetwork<>, typename EdgeRndIt = typename std::vector<typename
Network::Edge>::const_iterator, typename ScoreRndIt = typename std::vector<double>::iterator,
typename EdgeRndOutIt = typename std::vector<typename Network::Edge>::iterator>
virtual void LinkPred::UJIDPredictor< Network, EdgeRndIt, ScoreRndIt, EdgeRndOutIt >::learn (
) [inline], [virtual]
```

Learning.

Implements LinkPred::ULPredictor< UNetwork<>, typename std::vector< typename UNetwork<> ::Edge >::const_iterator, typena

9.124.3.3 operator=() [1/2]

```
template<typename Network = UNetwork<>, typename EdgeRndIt = typename std::vector<typename
Network::Edge>::const_iterator, typename ScoreRndIt = typename std::vector<double>::iterator,
typename EdgeRndOutIt = typename std::vector<typename Network::Edge>::iterator>
UJIDPredictor& LinkPred::UJIDPredictor< Network, EdgeRndIt, ScoreRndIt, EdgeRndOutIt >::operator=
(
            UJIDPredictor< Network, EdgeRndIt, ScoreRndIt, EdgeRndOutIt > && that )
```

Move assignment operator.

Parameters

that	The object to move.

9.124.3.4 operator=() [2/2]

```
template<typename Network = UNetwork<>, typename EdgeRndIt = typename std::vector<typename
Network::Edge>::const_iterator, typename ScoreRndIt = typename std::vector<double>::iterator,
typename EdgeRndOutIt = typename std::vector<typename Network::Edge>::iterator>
UJIDPredictor& LinkPred::UJIDPredictor< Network, EdgeRndIt, ScoreRndIt, EdgeRndOutIt >::operator=
(
            UJIDPredictor< Network, EdgeRndIt, ScoreRndIt, EdgeRndOutIt > const & that )
```

Copy assignment operator.

Parameters

that	The object to copy.

9.124.3.5 predict()

```
template<typename Network = UNetwork<>, typename EdgeRndIt = typename std::vector<typename
Network::Edge>::const_iterator, typename ScoreRndIt = typename std::vector<double>::iterator,
typename EdgeRndOutIt = typename std::vector<typename Network::Edge>::iterator>
virtual void LinkPred::UJIDPredictor< Network, EdgeRndIt, ScoreRndIt, EdgeRndOutIt >::predict
(
            EdgeRndIt begin,
            EdgeRndIt end,
            ScoreRndIt scores )  [virtual]
```

Predict the links.

Parameters

begin	Beginning of the links to be predicted.
end	end of the links to be predicted.
scores	Beginning of scores.

Reimplemented from LinkPred::ULPredictor< UNetwork<>, typename std::vector< typename UNetwork<> ::Edge >::const_iterat

9.124.3.6 score()

```
template<typename Network = UNetwork<>, typename EdgeRndIt = typename std::vector<typename
Network::Edge>::const_iterator, typename ScoreRndIt = typename std::vector<double>::iterator,
```

```
typename EdgeRndOutIt = typename std::vector<typename Network::Edge>::iterator>
virtual double LinkPred::UJIDPredictor< Network, EdgeRndIt, ScoreRndIt, EdgeRndOutIt >::score
(
            Edge const & e )  [inline], [virtual]
```

Compute the score of a single edge.

Parameters

e	The edge.

Returns

The score of e.

9.124.3.7 top()

```
template<typename Network = UNetwork<>, typename EdgeRndIt = typename std::vector<typename
Network::Edge>::const_iterator, typename ScoreRndIt = typename std::vector<double>::iterator,
typename EdgeRndOutIt = typename std::vector<typename Network::Edge>::iterator>
virtual std::size_t LinkPred::UJIDPredictor< Network, EdgeRndIt, ScoreRndIt, EdgeRndOutIt >↩
::top (
            std::size_t k,
            EdgeRndOutIt eit,
            ScoreRndIt sit )  [virtual]
```

Finds the k negative edges with the top score. Ties are broken randmly.

Parameters

k	The number of edges to find.
eit	An output iterator where the edges are written.
sit	An output iterator where the scores are written. The scores are written in the same order as the edges.

Returns

The number of negative edges inserted. It is the minimum between k and the number of negative edges in the network.

Reimplemented from LinkPred::ULPredictor< UNetwork<>, typename std::vector< typename UNetwork<> ::Edge >::const_iterat

The documentation for this class was generated from the following file:

- include/linkpred/predictors/undirected/ujidpredictor.hpp

9.125 LinkPred::UKABPredictor< Network, EdgeRndIt, ScoreRndIt, EdgeRndOutIt > Class Template Reference

A scalable popularity similarity link predictor proposed in: "Kerrache, S., Alharbi, R. & Benhidour, H. A Scalable Similarity-Popularity Link Prediction Method. Sci Rep 10, 6394 (2020)". https://doi.org/10.1038/s41598-020-62636-1.

`#include <ukabpredictor.hpp>`

Inheritance diagram for LinkPred::UKABPredictor< Network, EdgeRndIt, ScoreRndIt, EdgeRndOutIt >:

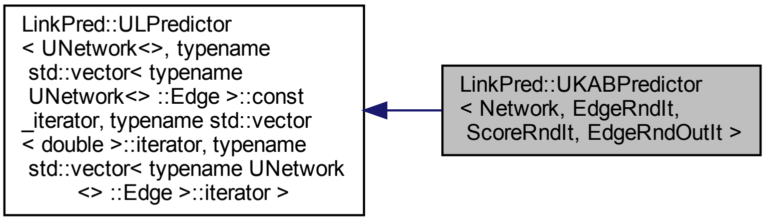

Collaboration diagram for LinkPred::UKABPredictor< Network, EdgeRndIt, ScoreRndIt, EdgeRndOutIt >:

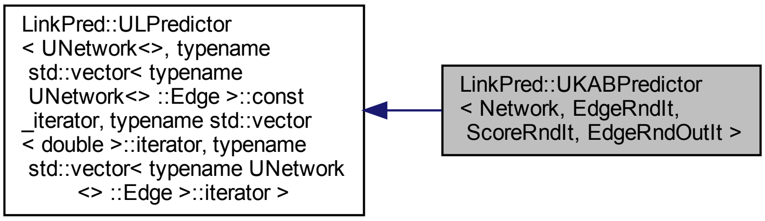

Public Types

- using NodeID = typename ULPredictor< Network, EdgeRndIt, ScoreRndIt, EdgeRndOutIt >::NodeID
- using Edge = typename ULPredictor< Network, EdgeRndIt, ScoreRndIt, EdgeRndOutIt >::Edge

Public Member Functions

- UKABPredictor (std::shared_ptr< Network const > net)
- UKABPredictor (UKABPredictor const &that)=default
- UKABPredictor & operator= (UKABPredictor const &that)=default
- UKABPredictor (UKABPredictor &&that)=default
- UKABPredictor & operator= (UKABPredictor &&that)=default

- virtual void init ()
- virtual void learn ()
- virtual double score (Edge const &e)
- virtual void predict (EdgeRndIt begin, EdgeRndIt end, ScoreRndIt scores)
- virtual std::size_t top (std::size_t k, EdgeRndOutIt eit, ScoreRndIt sit)
- CacheLevel getCacheLevel () const
- void setCacheLevel (CacheLevel cacheLevel)
- std::size_t getHorizLim () const
- void setHorizLim (std::size_t horizLim)
- virtual ~UKABPredictor ()=default

9.125.1 Detailed Description

template<typename Network = UNetwork<>, typename EdgeRndIt = typename std::vector<typename Network::Edge>↩ ::const_iterator, typename ScoreRndIt = typename std::vector<double>::iterator, typename EdgeRndOutIt = typename std↩ ::vector<typename Network::Edge>::iterator>
class LinkPred::UKABPredictor< Network, EdgeRndIt, ScoreRndIt, EdgeRndOutIt >

A scalable popularity similarity link predictor proposed in: "Kerrache, S., Alharbi, R. & Benhidour, H. A Scalable Similarity-Popularity Link Prediction Method. Sci Rep 10, 6394 (2020)". https://doi.org/10.↩ 1038/s41598-020-62636-1.

Template Parameters

Network	The network type.
Edge↩ RndIt	A random iterator type used to iterate on edges.
Score↩ RndIt	A random iterator type used to iterate on scores.

9.125.2 Member Typedef Documentation

9.125.2.1 Edge

```
template<typename Network = UNetwork<>, typename EdgeRndIt = typename std::vector<typename
Network::Edge>::const_iterator, typename ScoreRndIt = typename std::vector<double>::iterator,
typename EdgeRndOutIt = typename std::vector<typename Network::Edge>::iterator>
using LinkPred::UKABPredictor< Network, EdgeRndIt, ScoreRndIt, EdgeRndOutIt >::Edge = typename
ULPredictor<Network, EdgeRndIt,ScoreRndIt, EdgeRndOutIt>::Edge
```

The edges type.

9.125.2.2 NodeID

```
template<typename Network = UNetwork<>, typename EdgeRndIt = typename std::vector<typename
Network::Edge>::const_iterator, typename ScoreRndIt = typename std::vector<double>::iterator,
typename EdgeRndOutIt = typename std::vector<typename Network::Edge>::iterator>
using LinkPred::UKABPredictor< Network, EdgeRndIt, ScoreRndIt, EdgeRndOutIt >::NodeID = typename
ULPredictor<Network, EdgeRndIt,ScoreRndIt, EdgeRndOutIt>::NodeID
```

The node IDs type.

9.125.3 Constructor & Destructor Documentation

9.125.3.1 UKABPredictor() [1/3]

```
template<typename Network = UNetwork<>, typename EdgeRndIt = typename std::vector<typename
Network::Edge>::const_iterator, typename ScoreRndIt = typename std::vector<double>::iterator,
typename EdgeRndOutIt = typename std::vector<typename Network::Edge>::iterator>
LinkPred::UKABPredictor< Network, EdgeRndIt, ScoreRndIt, EdgeRndOutIt >::UKABPredictor (
            std::shared_ptr< Network const > net ) [inline]
```

Parameters

net	The network.

9.125.3.2 UKABPredictor() [2/3]

```
template<typename Network = UNetwork<>, typename EdgeRndIt = typename std::vector<typename
Network::Edge>::const_iterator, typename ScoreRndIt = typename std::vector<double>::iterator,
typename EdgeRndOutIt = typename std::vector<typename Network::Edge>::iterator>
LinkPred::UKABPredictor< Network, EdgeRndIt, ScoreRndIt, EdgeRndOutIt >::UKABPredictor (
            UKABPredictor< Network, EdgeRndIt, ScoreRndIt, EdgeRndOutIt > const & that )
[default]
```

Copy constructor.

Parameters

that	The object to copy.

9.125.3.3 UKABPredictor() [3/3]

```
template<typename Network = UNetwork<>, typename EdgeRndIt = typename std::vector<typename
Network::Edge>::const_iterator, typename ScoreRndIt = typename std::vector<double>::iterator,
typename EdgeRndOutIt = typename std::vector<typename Network::Edge>::iterator>
LinkPred::UKABPredictor< Network, EdgeRndIt, ScoreRndIt, EdgeRndOutIt >::UKABPredictor (
            UKABPredictor< Network, EdgeRndIt, ScoreRndIt, EdgeRndOutIt > && that ) [default]
```

Move constructor.

Parameters

that	The object to move.

9.125.3.4 ∼UKABPredictor()

```
template<typename Network = UNetwork<>, typename EdgeRndIt = typename std::vector<typename
Network::Edge>::const_iterator, typename ScoreRndIt = typename std::vector<double>::iterator,
typename EdgeRndOutIt = typename std::vector<typename Network::Edge>::iterator>
virtual LinkPred::UKABPredictor< Network, EdgeRndIt, ScoreRndIt, EdgeRndOutIt >::∼UKABPredictor
( ) [virtual], [default]
```

Destructor.

9.125.4 Member Function Documentation

9.125.4.1 getCacheLevel()

```
template<typename Network = UNetwork<>, typename EdgeRndIt = typename std::vector<typename
Network::Edge>::const_iterator, typename ScoreRndIt = typename std::vector<double>::iterator,
typename EdgeRndOutIt = typename std::vector<typename Network::Edge>::iterator>
CacheLevel LinkPred::UKABPredictor< Network, EdgeRndIt, ScoreRndIt, EdgeRndOutIt >::getCache←-
Level ( ) const [inline]
```

Returns

The distances cache level.

9.125.4.2 getHorizLim()

```
template<typename Network = UNetwork<>, typename EdgeRndIt = typename std::vector<typename
Network::Edge>::const_iterator, typename ScoreRndIt = typename std::vector<double>::iterator,
typename EdgeRndOutIt = typename std::vector<typename Network::Edge>::iterator>
std::size_t LinkPred::UKABPredictor< Network, EdgeRndIt, ScoreRndIt, EdgeRndOutIt >::get←-
HorizLim ( ) const [inline]
```

Returns

The horizon limit.

9.125.4.3 init()

```
template<typename Network = UNetwork<>, typename EdgeRndIt = typename std::vector<typename
Network::Edge>::const_iterator, typename ScoreRndIt = typename std::vector<double>::iterator,
typename EdgeRndOutIt = typename std::vector<typename Network::Edge>::iterator>
virtual void LinkPred::UKABPredictor< Network, EdgeRndIt, ScoreRndIt, EdgeRndOutIt >::init ( )
[virtual]
```

Initialize the predictor.

Implements LinkPred::ULPredictor< UNetwork<>, typename std::vector< typename UNetwork<> ::Edge >::const_iterator, typena

9.125.4.4 learn()

```
template<typename Network = UNetwork<>, typename EdgeRndIt = typename std::vector<typename
Network::Edge>::const_iterator, typename ScoreRndIt = typename std::vector<double>::iterator,
typename EdgeRndOutIt = typename std::vector<typename Network::Edge>::iterator>
virtual void LinkPred::UKABPredictor< Network, EdgeRndIt, ScoreRndIt, EdgeRndOutIt >::learn (
) [virtual]
```

Learn.

Implements LinkPred::ULPredictor< UNetwork<>, typename std::vector< typename UNetwork<> ::Edge >::const_iterator, typena

9.125.4.5 operator=() [1/2]

```
template<typename Network = UNetwork<>, typename EdgeRndIt = typename std::vector<typename
Network::Edge>::const_iterator, typename ScoreRndIt = typename std::vector<double>::iterator,
typename EdgeRndOutIt = typename std::vector<typename Network::Edge>::iterator>
UKABPredictor& LinkPred::UKABPredictor< Network, EdgeRndIt, ScoreRndIt, EdgeRndOutIt >::operator=
(
            UKABPredictor< Network, EdgeRndIt, ScoreRndIt, EdgeRndOutIt > && that )  [default]
```

Move assignment operator.

Parameters

that	The object to move.

9.125.4.6 operator=() [2/2]

```
template<typename Network = UNetwork<>, typename EdgeRndIt = typename std::vector<typename
Network::Edge>::const_iterator, typename ScoreRndIt = typename std::vector<double>::iterator,
typename EdgeRndOutIt = typename std::vector<typename Network::Edge>::iterator>
```

```
UKABPredictor& LinkPred::UKABPredictor< Network, EdgeRndIt, ScoreRndIt, EdgeRndOutIt >::operator=
(
            UKABPredictor< Network, EdgeRndIt, ScoreRndIt, EdgeRndOutIt > const & that )
[default]
```

Copy assignment operator.

Parameters

that	The object to copy.

9.125.4.7 predict()

```
template<typename Network = UNetwork<>, typename EdgeRndIt = typename std::vector<typename
Network::Edge>::const_iterator, typename ScoreRndIt = typename std::vector<double>::iterator,
typename EdgeRndOutIt = typename std::vector<typename Network::Edge>::iterator>
virtual void LinkPred::UKABPredictor< Network, EdgeRndIt, ScoreRndIt, EdgeRndOutIt >::predict
(
            EdgeRndIt begin,
            EdgeRndIt end,
            ScoreRndIt scores )  [virtual]
```

Predict the links.

Parameters

begin	Beginning of the links to be predicted.
end	end of the links to be predicted.
scores	Beginning of scores.

Reimplemented from LinkPred::ULPredictor< UNetwork<>, typename std::vector< typename UNetwork<> ::Edge >::const_iterat

9.125.4.8 score()

```
template<typename Network = UNetwork<>, typename EdgeRndIt = typename std::vector<typename
Network::Edge>::const_iterator, typename ScoreRndIt = typename std::vector<double>::iterator,
typename EdgeRndOutIt = typename std::vector<typename Network::Edge>::iterator>
virtual double LinkPred::UKABPredictor< Network, EdgeRndIt, ScoreRndIt, EdgeRndOutIt >::score
(
            Edge const & e )  [virtual]
```

Compute the score of a single edge.

Parameters

e	The edge.

Returns

The score of e.

9.125.4.9 setCacheLevel()

```
template<typename Network = UNetwork<>, typename EdgeRndIt = typename std::vector<typename
Network::Edge>::const_iterator, typename ScoreRndIt = typename std::vector<double>::iterator,
typename EdgeRndOutIt = typename std::vector<typename Network::Edge>::iterator>
void LinkPred::UKABPredictor< Network, EdgeRndIt, ScoreRndIt, EdgeRndOutIt >::setCacheLevel (
            CacheLevel cacheLevel ) [inline]
```

Set the distances cache level.

Parameters

cacheLevel	The new distances cache level.

9.125.4.10 setHorizLim()

```
template<typename Network = UNetwork<>, typename EdgeRndIt = typename std::vector<typename
Network::Edge>::const_iterator, typename ScoreRndIt = typename std::vector<double>::iterator,
typename EdgeRndOutIt = typename std::vector<typename Network::Edge>::iterator>
void LinkPred::UKABPredictor< Network, EdgeRndIt, ScoreRndIt, EdgeRndOutIt >::setHorizLim (
            std::size_t horizLim ) [inline]
```

Parameters

horizLim	New horizon limit.

9.125.4.11 top()

```
template<typename Network = UNetwork<>, typename EdgeRndIt = typename std::vector<typename
Network::Edge>::const_iterator, typename ScoreRndIt = typename std::vector<double>::iterator,
typename EdgeRndOutIt = typename std::vector<typename Network::Edge>::iterator>
virtual std::size_t LinkPred::UKABPredictor< Network, EdgeRndIt, ScoreRndIt, EdgeRndOutIt >↩
::top (
            std::size_t k,
            EdgeRndOutIt eit,
            ScoreRndIt sit ) [virtual]
```

Finds the k negative edges with the top score. Ties are broken randomly.

Parameters

k	The number of edges to find.
eit	An output iterator where the edges are written.
sit	An output iterator where the scores are written. The scores are written in the same order as the edges.

Returns

The number of negative edges inserted. It is the minimum between k and the number of negative edges in the network.

Reimplemented from LinkPred::ULPredictor< UNetwork<>, typename std::vector< typename UNetwork<> ::Edge >::const_iterat

The documentation for this class was generated from the following file:

- include/linkpred/predictors/undirected/ukabpredictor.hpp

9.126 LinkPred::ULCPPredictor< Network, EdgeRndIt, ScoreRndIt, EdgeRndOutIt > Class Template Reference

Local path link predictor.

`#include <ulcppredictor.hpp>`

Inheritance diagram for LinkPred::ULCPPredictor< Network, EdgeRndIt, ScoreRndIt, EdgeRndOutIt >:

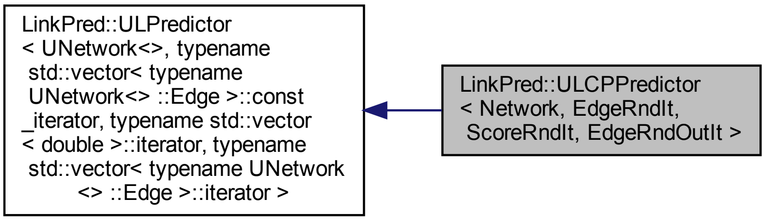

Collaboration diagram for LinkPred::ULCPPredictor< Network, EdgeRndIt, ScoreRndIt, EdgeRndOutIt >:

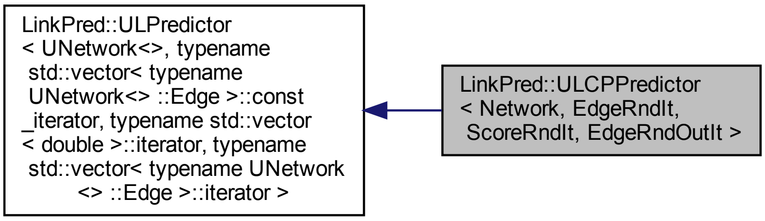

Public Member Functions

- ULCPPredictor (std::shared_ptr< Network const > net)
- ULCPPredictor (ULCPPredictor const &that)=default
- ULCPPredictor & operator= (ULCPPredictor const &that)=default
- ULCPPredictor (ULCPPredictor &&that)=default
- ULCPPredictor & operator= (ULCPPredictor &&that)=default
- virtual void init ()
- virtual void learn ()
- virtual void predict (EdgeRndIt begin, EdgeRndIt end, ScoreRndIt scores)
- virtual double score (Edge const &e)
- double getEpsilon () const
- void setEpsilon (double epsilon)
- virtual ~ULCPPredictor ()=default

Additional Inherited Members

9.126.1 Detailed Description

template<typename Network = UNetwork<>, typename EdgeRndIt = typename std::vector<typename Network::Edge>↩ ::const_iterator, typename ScoreRndIt = typename std::vector<double>::iterator, typename EdgeRndOutIt = typename std↩ ::vector<typename Network::Edge>::iterator>
class LinkPred::ULCPPredictor< Network, EdgeRndIt, ScoreRndIt, EdgeRndOutIt >

Local path link predictor.

Template Parameters

Network	The network type.
Edge↩ RndIt	A random iterator type used to iterate on edges.
Score↩ RndIt	A random iterator type used to iterate on scores.

9.126.2 Constructor & Destructor Documentation

9.126.2.1 ULCPPredictor() [1/3]

```
template<typename Network = UNetwork<>, typename EdgeRndIt = typename std::vector<typename
Network::Edge>::const_iterator, typename ScoreRndIt = typename std::vector<double>::iterator,
typename EdgeRndOutIt = typename std::vector<typename Network::Edge>::iterator>
LinkPred::ULCPPredictor< Network, EdgeRndIt, ScoreRndIt, EdgeRndOutIt >::ULCPPredictor (
            std::shared_ptr< Network const > net )  [inline]
```

Parameters

net	The network.

9.126.2.2 ULCPPredictor() [2/3]

```
template<typename Network = UNetwork<>, typename EdgeRndIt = typename std::vector<typename
Network::Edge>::const_iterator, typename ScoreRndIt = typename std::vector<double>::iterator,
typename EdgeRndOutIt = typename std::vector<typename Network::Edge>::iterator>
LinkPred::ULCPPredictor< Network, EdgeRndIt, ScoreRndIt, EdgeRndOutIt >::ULCPPredictor (
            ULCPPredictor< Network, EdgeRndIt, ScoreRndIt, EdgeRndOutIt > const & that )
[default]
```

Copy constructor.

Parameters

that	The object to copy.

9.126.2.3 ULCPPredictor() [3/3]

```
template<typename Network = UNetwork<>, typename EdgeRndIt = typename std::vector<typename
Network::Edge>::const_iterator, typename ScoreRndIt = typename std::vector<double>::iterator,
typename EdgeRndOutIt = typename std::vector<typename Network::Edge>::iterator>
LinkPred::ULCPPredictor< Network, EdgeRndIt, ScoreRndIt, EdgeRndOutIt >::ULCPPredictor (
            ULCPPredictor< Network, EdgeRndIt, ScoreRndIt, EdgeRndOutIt > && that )  [default]
```

Move constructor.

Parameters

that	The object to move.

9.126.2.4 ∼ULCPPredictor()

```
template<typename Network = UNetwork<>, typename EdgeRndIt = typename std::vector<typename
Network::Edge>::const_iterator, typename ScoreRndIt = typename std::vector<double>::iterator,
typename EdgeRndOutIt = typename std::vector<typename Network::Edge>::iterator>
virtual LinkPred::ULCPPredictor< Network, EdgeRndIt, ScoreRndIt, EdgeRndOutIt >::~ULCPPredictor
( )  [virtual], [default]
```

Destructor.

9.126.3 Member Function Documentation

9.126.3.1 getEpsilon()

```
template<typename Network = UNetwork<>, typename EdgeRndIt = typename std::vector<typename
Network::Edge>::const_iterator, typename ScoreRndIt = typename std::vector<double>::iterator,
typename EdgeRndOutIt = typename std::vector<typename Network::Edge>::iterator>
double LinkPred::ULCPPredictor< Network, EdgeRndIt, ScoreRndIt, EdgeRndOutIt >::getEpsilon ( )
const [inline]
```

Returns

epsilon, the weight of paths of length 3.

9.126.3.2 init()

```
template<typename Network = UNetwork<>, typename EdgeRndIt = typename std::vector<typename
Network::Edge>::const_iterator, typename ScoreRndIt = typename std::vector<double>::iterator,
typename EdgeRndOutIt = typename std::vector<typename Network::Edge>::iterator>
virtual void LinkPred::ULCPPredictor< Network, EdgeRndIt, ScoreRndIt, EdgeRndOutIt >::init ( )
[inline], [virtual]
```

Initialize the predictor.

Implements LinkPred::ULPredictor< UNetwork<>, typename std::vector< typename UNetwork<> ::Edge >::const_iterator, typena

9.126.3.3 learn()

```
template<typename Network = UNetwork<>, typename EdgeRndIt = typename std::vector<typename
Network::Edge>::const_iterator, typename ScoreRndIt = typename std::vector<double>::iterator,
typename EdgeRndOutIt = typename std::vector<typename Network::Edge>::iterator>
virtual void LinkPred::ULCPPredictor< Network, EdgeRndIt, ScoreRndIt, EdgeRndOutIt >::learn (
) [inline], [virtual]
```

Learn.

Implements LinkPred::ULPredictor< UNetwork<>, typename std::vector< typename UNetwork<> ::Edge >::const_iterator, typena

9.126.3.4 operator=() [1/2]

```
template<typename Network = UNetwork<>, typename EdgeRndIt = typename std::vector<typename
Network::Edge>::const_iterator, typename ScoreRndIt = typename std::vector<double>::iterator,
typename EdgeRndOutIt = typename std::vector<typename Network::Edge>::iterator>
ULCPPredictor& LinkPred::ULCPPredictor< Network, EdgeRndIt, ScoreRndIt, EdgeRndOutIt >::operator=
(
            ULCPPredictor< Network, EdgeRndIt, ScoreRndIt, EdgeRndOutIt > && that ) [default]
```

Move assignment operator.

Parameters

that	The object to move.

9.126.3.5 operator=() [2/2]

```
template<typename Network = UNetwork<>, typename EdgeRndIt = typename std::vector<typename
Network::Edge>::const_iterator, typename ScoreRndIt = typename std::vector<double>::iterator,
typename EdgeRndOutIt = typename std::vector<typename Network::Edge>::iterator>
ULCPPredictor& LinkPred::ULCPPredictor< Network, EdgeRndIt, ScoreRndIt, EdgeRndOutIt >::operator=
(
            ULCPPredictor< Network, EdgeRndIt, ScoreRndIt, EdgeRndOutIt > const & that )
[default]
```

Copy assignment operator.

Parameters

that	The object to copy.

9.126.3.6 predict()

```
template<typename Network = UNetwork<>, typename EdgeRndIt = typename std::vector<typename
Network::Edge>::const_iterator, typename ScoreRndIt = typename std::vector<double>::iterator,
typename EdgeRndOutIt = typename std::vector<typename Network::Edge>::iterator>
virtual void LinkPred::ULCPPredictor< Network, EdgeRndIt, ScoreRndIt, EdgeRndOutIt >::predict
(
            EdgeRndIt begin,
            EdgeRndIt end,
            ScoreRndIt scores )  [virtual]
```

Predict the links.

Parameters

begin	Beginning of the links to be predicted.
end	end of the links to be predicted.
scores	Beginning of scores.

Reimplemented from LinkPred::ULPredictor< UNetwork<>, typename std::vector< typename UNetwork<> ::Edge >::const_iterat

9.126.3.7 score()

```
template<typename Network = UNetwork<>, typename EdgeRndIt = typename std::vector<typename
Network::Edge>::const_iterator, typename ScoreRndIt = typename std::vector<double>::iterator,
```

```
typename EdgeRndOutIt = typename std::vector<typename Network::Edge>::iterator>
virtual double LinkPred::ULCPPredictor< Network, EdgeRndIt, ScoreRndIt, EdgeRndOutIt >::score
(
            Edge const & e )  [virtual]
```

Compute the score of a single edge.

Parameters

e	The edge.

Returns

The score of e.

9.126.3.8 setEpsilon()

```
template<typename Network = UNetwork<>, typename EdgeRndIt = typename std::vector<typename
Network::Edge>::const_iterator, typename ScoreRndIt = typename std::vector<double>::iterator,
typename EdgeRndOutIt = typename std::vector<typename Network::Edge>::iterator>
void LinkPred::ULCPPredictor< Network, EdgeRndIt, ScoreRndIt, EdgeRndOutIt >::setEpsilon (
            double epsilon )  [inline]
```

Set epsilon, the weight of paths of length 3.

Parameters

epsilon	The new weight of paths of length 3.

The documentation for this class was generated from the following file:

- include/linkpred/predictors/undirected/ulcppredictor.hpp

9.127 LinkPred::ULHNPredictor< Network, EdgeRndIt, ScoreRndIt, EdgeRndOutIt > Class Template Reference

Leicht-Holme-Newman index link predictor.

```
#include <ulhnpredictor.hpp>
```

Inheritance diagram for LinkPred::ULHNPredictor< Network, EdgeRndIt, ScoreRndIt, EdgeRndOutIt >:

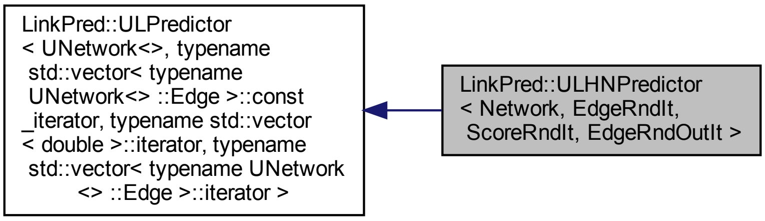

Collaboration diagram for LinkPred::ULHNPredictor< Network, EdgeRndIt, ScoreRndIt, EdgeRndOutIt >:

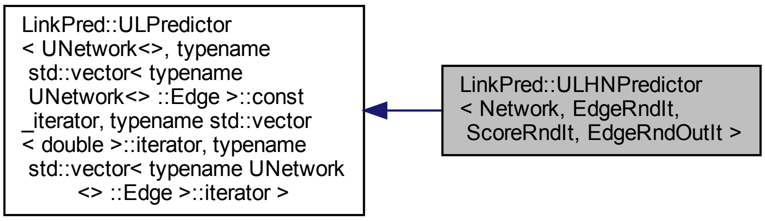

Public Member Functions

- ULHNPredictor (std::shared_ptr< Network const > net)
- ULHNPredictor (ULHNPredictor const &that)=default
- ULHNPredictor & operator= (ULHNPredictor const &that)=default
- ULHNPredictor (ULHNPredictor &&that)=default
- ULHNPredictor & operator= (ULHNPredictor &&that)=default
- virtual void init ()
- virtual void learn ()
- virtual double score (Edge const &e)
- virtual void predict (EdgeRndIt begin, EdgeRndIt end, ScoreRndIt scores)
- virtual std::size_t top (std::size_t k, EdgeRndOutIt eit, ScoreRndIt sit)
- virtual ~ULHNPredictor ()=default

Additional Inherited Members

9.127.1 Detailed Description

template<typename Network = UNetwork<>, typename EdgeRndIt = typename std::vector<typename Network::Edge>↩ ::const_iterator, typename ScoreRndIt = typename std::vector<double>::iterator, typename EdgeRndOutIt = typename std↩ ::vector<typename Network::Edge>::iterator>
class LinkPred::ULHNPredictor< Network, EdgeRndIt, ScoreRndIt, EdgeRndOutIt >

Leicht-Holme-Newman index link predictor.

Template Parameters

Network	The network type.
Edge↵ RndIt	A random iterator type used to iterate on edges.
Score↵ RndIt	A random iterator type used to iterate on scores.

9.127.2 Constructor & Destructor Documentation

9.127.2.1 ULHNPredictor() [1/3]

```
template<typename Network = UNetwork<>, typename EdgeRndIt = typename std::vector<typename
Network::Edge>::const_iterator, typename ScoreRndIt = typename std::vector<double>::iterator,
typename EdgeRndOutIt = typename std::vector<typename Network::Edge>::iterator>
LinkPred::ULHNPredictor< Network, EdgeRndIt, ScoreRndIt, EdgeRndOutIt >::ULHNPredictor (
            std::shared_ptr< Network const > net )  [inline]
```

Parameters

net	The network.

9.127.2.2 ULHNPredictor() [2/3]

```
template<typename Network = UNetwork<>, typename EdgeRndIt = typename std::vector<typename
Network::Edge>::const_iterator, typename ScoreRndIt = typename std::vector<double>::iterator,
typename EdgeRndOutIt = typename std::vector<typename Network::Edge>::iterator>
LinkPred::ULHNPredictor< Network, EdgeRndIt, ScoreRndIt, EdgeRndOutIt >::ULHNPredictor (
            ULHNPredictor< Network, EdgeRndIt, ScoreRndIt, EdgeRndOutIt > const & that )
[default]
```

Copy constructor.

Parameters

that	The object to copy.

9.127.2.3 ULHNPredictor() [3/3]

```
template<typename Network = UNetwork<>, typename EdgeRndIt = typename std::vector<typename
Network::Edge>::const_iterator, typename ScoreRndIt = typename std::vector<double>::iterator,
```

```
typename EdgeRndOutIt = typename std::vector<typename Network::Edge>::iterator>
LinkPred::ULHNPredictor< Network, EdgeRndIt, ScoreRndIt, EdgeRndOutIt >::ULHNPredictor (
            ULHNPredictor< Network, EdgeRndIt, ScoreRndIt, EdgeRndOutIt > && that )  [default]
```

Move constructor.

Parameters

that	The object to move.

9.127.2.4 ∼ULHNPredictor()

```
template<typename Network = UNetwork<>, typename EdgeRndIt = typename std::vector<typename
Network::Edge>::const_iterator, typename ScoreRndIt = typename std::vector<double>::iterator,
typename EdgeRndOutIt = typename std::vector<typename Network::Edge>::iterator>
virtual LinkPred::ULHNPredictor< Network, EdgeRndIt, ScoreRndIt, EdgeRndOutIt >::∼ULHNPredictor
( )  [virtual], [default]
```

Destructor.

9.127.3 Member Function Documentation

9.127.3.1 init()

```
template<typename Network = UNetwork<>, typename EdgeRndIt = typename std::vector<typename
Network::Edge>::const_iterator, typename ScoreRndIt = typename std::vector<double>::iterator,
typename EdgeRndOutIt = typename std::vector<typename Network::Edge>::iterator>
virtual void LinkPred::ULHNPredictor< Network, EdgeRndIt, ScoreRndIt, EdgeRndOutIt >::init ( )
[inline], [virtual]
```

Initialize the predictor.

Implements LinkPred::ULPredictor< UNetwork<>, typename std::vector< typename UNetwork<> ::Edge >::const_iterator, typena

9.127.3.2 learn()

```
template<typename Network = UNetwork<>, typename EdgeRndIt = typename std::vector<typename
Network::Edge>::const_iterator, typename ScoreRndIt = typename std::vector<double>::iterator,
typename EdgeRndOutIt = typename std::vector<typename Network::Edge>::iterator>
virtual void LinkPred::ULHNPredictor< Network, EdgeRndIt, ScoreRndIt, EdgeRndOutIt >::learn (
)  [inline], [virtual]
```

Learning.

Implements LinkPred::ULPredictor< UNetwork<>, typename std::vector< typename UNetwork<> ::Edge >::const_iterator, typena

9.127.3.3 operator=() [1/2]

```
template<typename Network = UNetwork<>, typename EdgeRndIt = typename std::vector<typename
Network::Edge>::const_iterator, typename ScoreRndIt = typename std::vector<double>::iterator,
typename EdgeRndOutIt = typename std::vector<typename Network::Edge>::iterator>
ULHNPredictor& LinkPred::ULHNPredictor< Network, EdgeRndIt, ScoreRndIt, EdgeRndOutIt >::operator=
(
            ULHNPredictor< Network, EdgeRndIt, ScoreRndIt, EdgeRndOutIt > && that )  [default]
```

Move assignment operator.

Parameters

that	The object to move.

9.127.3.4 operator=() [2/2]

```
template<typename Network = UNetwork<>, typename EdgeRndIt = typename std::vector<typename
Network::Edge>::const_iterator, typename ScoreRndIt = typename std::vector<double>::iterator,
typename EdgeRndOutIt = typename std::vector<typename Network::Edge>::iterator>
ULHNPredictor& LinkPred::ULHNPredictor< Network, EdgeRndIt, ScoreRndIt, EdgeRndOutIt >::operator=
(
            ULHNPredictor< Network, EdgeRndIt, ScoreRndIt, EdgeRndOutIt > const & that )
[default]
```

Copy assignment operator.

Parameters

that	The object to copy.

9.127.3.5 predict()

```
template<typename Network = UNetwork<>, typename EdgeRndIt = typename std::vector<typename
Network::Edge>::const_iterator, typename ScoreRndIt = typename std::vector<double>::iterator,
typename EdgeRndOutIt = typename std::vector<typename Network::Edge>::iterator>
virtual void LinkPred::ULHNPredictor< Network, EdgeRndIt, ScoreRndIt, EdgeRndOutIt >::predict
(
            EdgeRndIt begin,
            EdgeRndIt end,
            ScoreRndIt scores )  [virtual]
```

Predict the links.

Parameters

begin	Beginning of the links to be predicted.
end	end of the links to be predicted.
scores	Beginning of scores.

Reimplemented from LinkPred::ULPredictor< UNetwork<>, typename std::vector< typename UNetwork<> ::Edge >::const_iterat

9.127.3.6 score()

```
template<typename Network = UNetwork<>, typename EdgeRndIt = typename std::vector<typename
Network::Edge>::const_iterator, typename ScoreRndIt = typename std::vector<double>::iterator,
typename EdgeRndOutIt = typename std::vector<typename Network::Edge>::iterator>
virtual double LinkPred::ULHNPredictor< Network, EdgeRndIt, ScoreRndIt, EdgeRndOutIt >::score
(
            Edge const & e ) [inline], [virtual]
```

Compute the score of a single edge.

Parameters

e	The edge.

Returns

The score of e.

9.127.3.7 top()

```
template<typename Network = UNetwork<>, typename EdgeRndIt = typename std::vector<typename
Network::Edge>::const_iterator, typename ScoreRndIt = typename std::vector<double>::iterator,
typename EdgeRndOutIt = typename std::vector<typename Network::Edge>::iterator>
virtual std::size_t LinkPred::ULHNPredictor< Network, EdgeRndIt, ScoreRndIt, EdgeRndOutIt >←
::top (
            std::size_t k,
            EdgeRndOutIt eit,
            ScoreRndIt sit ) [virtual]
```

Finds the k negative edges with the top score. Ties are broken randmly.

Parameters

k	The number of edges to find.
eit	An output iterator where the edges are written.
sit	An output iterator where the scores are written. The scores are written in the same order as the edges.

Returns

The number of negative edges inserted. It is the minimum between k and the number of negative edges in the network.

Reimplemented from LinkPred::ULPredictor< UNetwork<>, typename std::vector< typename UNetwork<> ::Edge >::const_iterat

The documentation for this class was generated from the following file:

- include/linkpred/predictors/undirected/ulhnpredictor.hpp

9.128 LinkPred::ULPredictor< NetworkT, EdgeRndItT, ScoreRndItT, EdgeRndOutItT > Class Template Reference

The interface of a link predictor in an undirected network.

```
#include <ulpredictor.hpp>
```

Public Types

- using Network = NetworkT
- using EdgeRndIt = EdgeRndItT
- using ScoreRndIt = ScoreRndItT
- using EdgeRndOutIt = EdgeRndOutItT
- using NodeID = typename Network::NodeID
- using Edge = typename Network::Edge

Public Member Functions

- ULPredictor (std::shared_ptr< Network const > net)
- ULPredictor (ULPredictor const &that)=default
- ULPredictor & operator= (ULPredictor const &that)=default
- ULPredictor (ULPredictor &&that)=default
- ULPredictor & operator= (ULPredictor &&that)=default
- virtual void init ()=0
- virtual void learn ()=0
- virtual void predict (EdgeRndIt begin, EdgeRndIt end, ScoreRndIt scores)
- virtual std::pair< typename Network::NonEdgeIt, typename Network::NonEdgeIt > predictNeg (ScoreRndIt scores)
- virtual double score (Edge const &e)=0
- virtual std::size_t top (std::size_t k, EdgeRndOutIt eit, ScoreRndIt sit)
- auto getNet () const
- const std::string & getName () const
- void setName (const std::string &name)
- virtual ~ULPredictor ()=default

9.128.1 Detailed Description

template<typename NetworkT = UNetwork<>, typename EdgeRndItT = typename std::vector<typename NetworkT::Edge>↩ ::const_iterator, typename ScoreRndItT = typename std::vector<double>::iterator, typename EdgeRndOutItT = typename std↩ ::vector<typename NetworkT::Edge>::iterator>
class LinkPred::ULPredictor< NetworkT, EdgeRndItT, ScoreRndItT, EdgeRndOutItT >

The interface of a link predictor in an undirected network.

Template Parameters

NetworkT	The network type.
EdgeRndItT	A random iterator type used to iterate on edges.
ScoreRndItT	A random iterator type used to iterate on scores.

Parameters

EdgeRndOutItT	A random output iterator to write edges.

9.128.2 Member Typedef Documentation

9.128.2.1 Edge

```
template<typename NetworkT = UNetwork<>, typename EdgeRndItT = typename std::vector<typename
NetworkT::Edge>::const_iterator, typename ScoreRndItT = typename std::vector<double>::iterator,
typename EdgeRndOutItT = typename std::vector<typename NetworkT::Edge>::iterator>
using LinkPred::ULPredictor< NetworkT, EdgeRndItT, ScoreRndItT, EdgeRndOutItT >::Edge = typename
Network::Edge
```

The edges type.

9.128.2.2 EdgeRndIt

```
template<typename NetworkT = UNetwork<>, typename EdgeRndItT = typename std::vector<typename
NetworkT::Edge>::const_iterator, typename ScoreRndItT = typename std::vector<double>::iterator,
typename EdgeRndOutItT = typename std::vector<typename NetworkT::Edge>::iterator>
using LinkPred::ULPredictor< NetworkT, EdgeRndItT, ScoreRndItT, EdgeRndOutItT >::EdgeRndIt =
EdgeRndItT
```

A random iterator type used to iterate on edges.

9.128.2.3 EdgeRndOutIt

```
template<typename NetworkT = UNetwork<>, typename EdgeRndItT = typename std::vector<typename
NetworkT::Edge>::const_iterator, typename ScoreRndItT = typename std::vector<double>::iterator,
typename EdgeRndOutItT = typename std::vector<typename NetworkT::Edge>::iterator>
using LinkPred::ULPredictor< NetworkT, EdgeRndItT, ScoreRndItT, EdgeRndOutItT >::EdgeRndOutIt
= EdgeRndOutItT
```

A random output iterator to write edges.

9.128.2.4 Network

```
template<typename NetworkT = UNetwork<>, typename EdgeRndItT = typename std::vector<typename
NetworkT::Edge>::const_iterator, typename ScoreRndItT = typename std::vector<double>::iterator,
typename EdgeRndOutItT = typename std::vector<typename NetworkT::Edge>::iterator>
using LinkPred::ULPredictor< NetworkT, EdgeRndItT, ScoreRndItT, EdgeRndOutItT >::Network =
NetworkT
```

The network type.

9.128.2.5 NodeID

```
template<typename NetworkT = UNetwork<>, typename EdgeRndItT = typename std::vector<typename
NetworkT::Edge>::const_iterator, typename ScoreRndItT = typename std::vector<double>::iterator,
typename EdgeRndOutItT = typename std::vector<typename NetworkT::Edge>::iterator>
using LinkPred::ULPredictor< NetworkT, EdgeRndItT, ScoreRndItT, EdgeRndOutItT >::NodeID =
typename Network::NodeID
```

The node IDs type.

9.128.2.6 ScoreRndIt

```
template<typename NetworkT = UNetwork<>, typename EdgeRndItT = typename std::vector<typename
NetworkT::Edge>::const_iterator, typename ScoreRndItT = typename std::vector<double>::iterator,
typename EdgeRndOutItT = typename std::vector<typename NetworkT::Edge>::iterator>
using LinkPred::ULPredictor< NetworkT, EdgeRndItT, ScoreRndItT, EdgeRndOutItT >::ScoreRndIt =
ScoreRndItT
```

A random iterator type used to iterate on scores.

9.128.3 Constructor & Destructor Documentation

9.128.3.1 ULPredictor() [1/3]

```
template<typename NetworkT = UNetwork<>, typename EdgeRndItT = typename std::vector<typename
NetworkT::Edge>::const_iterator, typename ScoreRndItT = typename std::vector<double>::iterator,
typename EdgeRndOutItT = typename std::vector<typename NetworkT::Edge>::iterator>
LinkPred::ULPredictor< NetworkT, EdgeRndItT, ScoreRndItT, EdgeRndOutItT >::ULPredictor (
            std::shared_ptr< Network const > net )  [inline]
```

Constructor.

Parameters

net	The network.

9.128.3.2 ULPredictor() [2/3]

```
template<typename NetworkT = UNetwork<>, typename EdgeRndItT = typename std::vector<typename
NetworkT::Edge>::const_iterator, typename ScoreRndItT = typename std::vector<double>::iterator,
typename EdgeRndOutItT = typename std::vector<typename NetworkT::Edge>::iterator>
LinkPred::ULPredictor< NetworkT, EdgeRndItT, ScoreRndItT, EdgeRndOutItT >::ULPredictor (
            ULPredictor< NetworkT, EdgeRndItT, ScoreRndItT, EdgeRndOutItT > const & that )
[default]
```

Copy constructor.

Parameters

that	The object to copy.

9.128.3.3 ULPredictor() [3/3]

```
template<typename NetworkT = UNetwork<>, typename EdgeRndItT = typename std::vector<typename
NetworkT::Edge>::const_iterator, typename ScoreRndItT = typename std::vector<double>::iterator,
typename EdgeRndOutItT = typename std::vector<typename NetworkT::Edge>::iterator>
LinkPred::ULPredictor< NetworkT, EdgeRndItT, ScoreRndItT, EdgeRndOutItT >::ULPredictor (
            ULPredictor< NetworkT, EdgeRndItT, ScoreRndItT, EdgeRndOutItT > && that )  [default]
```

Move constructor.

Parameters

that	The object to move.

9.128.3.4 ∼ULPredictor()

```
template<typename NetworkT = UNetwork<>, typename EdgeRndItT = typename std::vector<typename
NetworkT::Edge>::const_iterator, typename ScoreRndItT = typename std::vector<double>::iterator,
typename EdgeRndOutItT = typename std::vector<typename NetworkT::Edge>::iterator>
virtual LinkPred::ULPredictor< NetworkT, EdgeRndItT, ScoreRndItT, EdgeRndOutItT >::~ULPredictor
( )  [virtual], [default]
```

Destructor.

9.128.4 Member Function Documentation

9.128.4.1 getName()

```
template<typename NetworkT = UNetwork<>, typename EdgeRndItT = typename std::vector<typename
NetworkT::Edge>::const_iterator, typename ScoreRndItT = typename std::vector<double>::iterator,
typename EdgeRndOutItT = typename std::vector<typename NetworkT::Edge>::iterator>
const std::string& LinkPred::ULPredictor< NetworkT, EdgeRndItT, ScoreRndItT, EdgeRndOutItT >↩
::getName ( ) const [inline]
```

Returns

The name of the predictor.

9.128.4.2 getNet()

```
template<typename NetworkT = UNetwork<>, typename EdgeRndItT = typename std::vector<typename
NetworkT::Edge>::const_iterator, typename ScoreRndItT = typename std::vector<double>::iterator,
typename EdgeRndOutItT = typename std::vector<typename NetworkT::Edge>::iterator>
auto LinkPred::ULPredictor< NetworkT, EdgeRndItT, ScoreRndItT, EdgeRndOutItT >::getNet ( )
const [inline]
```

Returns

The network.

9.128.4.3 init()

```
template<typename NetworkT = UNetwork<>, typename EdgeRndItT = typename std::vector<typename
NetworkT::Edge>::const_iterator, typename ScoreRndItT = typename std::vector<double>::iterator,
typename EdgeRndOutItT = typename std::vector<typename NetworkT::Edge>::iterator>
virtual void LinkPred::ULPredictor< NetworkT, EdgeRndItT, ScoreRndItT, EdgeRndOutItT >::init (
) [pure virtual]
```

Initialize the solver.

Implemented in LinkPred::UHYPPredictor< Network, EdgeRndIt, ScoreRndIt, EdgeRndOutIt >, LinkPred::UMPSPredictor< Network [illegible]
LinkPred::UKABPredictor< Network, EdgeRndIt, ScoreRndIt, EdgeRndOutIt >, LinkPred::UHRGPredictor< Network, EdgeRndIt, Sc [illegible]
LinkPred::UNEDPredictor< Network, EdgeRndIt, ScoreRndIt, EdgeRndOutIt >, LinkPred::UFBMPredictor< Network, EdgeRndIt, Sc [illegible]
LinkPred::USHPPredictor< Network, EdgeRndIt, ScoreRndIt, EdgeRndOutIt >, LinkPred::UPSTPredictor< Network, EdgeRndIt, Sc [illegible]
LinkPred::UECLPredictor< Network, EdgeRndIt, ScoreRndIt, EdgeRndOutIt >, LinkPred::UESMPredictor< Network, EdgeRndIt, Sc [illegible]
LinkPred::USBMPredictor< Network, EdgeRndIt, ScoreRndIt, EdgeRndOutIt >, LinkPred::UCSTPredictor< Network, EdgeRndIt, Sc [illegible]
LinkPred::UADAPredictor< Network, EdgeRndIt, ScoreRndIt, EdgeRndOutIt >, LinkPred::UCNEPredictor< Network, EdgeRndIt, Sc [illegible]
LinkPred::UCRAPredictor< Network, EdgeRndIt, ScoreRndIt, EdgeRndOutIt >, LinkPred::URNDPredictor< Network, EdgeRndIt, Sc [illegible]
LinkPred::USUMPredictor< Network, EdgeRndIt, ScoreRndIt, EdgeRndOutIt >, LinkPred::URALPredictor< Network, EdgeRndIt, Sc [illegible]
LinkPred::USOIPredictor< Network, EdgeRndIt, ScoreRndIt, EdgeRndOutIt >, LinkPred::UHDIPredictor< Network, EdgeRndIt, Sco [illegible]
LinkPred::UHPIPredictor< Network, EdgeRndIt, ScoreRndIt, EdgeRndOutIt >, LinkPred::UJIDPredictor< Network, EdgeRndIt, Scor [illegible]
LinkPred::ULHNPredictor< Network, EdgeRndIt, ScoreRndIt, EdgeRndOutIt >, LinkPred::UPATPredictor< Network, EdgeRndIt, Sc [illegible]
LinkPred::USAIPredictor< Network, EdgeRndIt, ScoreRndIt, EdgeRndOutIt >, and LinkPred::ULCPPredictor< Network, EdgeRndIt, [illegible]

9.128.4.4 learn()

```
template<typename NetworkT = UNetwork<>, typename EdgeRndItT = typename std::vector<typename
NetworkT::Edge>::const_iterator, typename ScoreRndItT = typename std::vector<double>::iterator,
typename EdgeRndOutItT = typename std::vector<typename NetworkT::Edge>::iterator>
virtual void LinkPred::ULPredictor< NetworkT, EdgeRndItT, ScoreRndItT, EdgeRndOutItT >::learn
( ) [pure virtual]
```

Learning.

Implemented in LinkPred::UHYPPredictor< Network, EdgeRndIt, ScoreRndIt, EdgeRndOutIt >, LinkPred::UMPSPredictor< Network
LinkPred::UKABPredictor< Network, EdgeRndIt, ScoreRndIt, EdgeRndOutIt >, LinkPred::UHRGPredictor< Network, EdgeRndIt, Sc
LinkPred::UNEDPredictor< Network, EdgeRndIt, ScoreRndIt, EdgeRndOutIt >, LinkPred::UFBMPredictor< Network, EdgeRndIt, Sc
LinkPred::USHPPredictor< Network, EdgeRndIt, ScoreRndIt, EdgeRndOutIt >, LinkPred::UPSTPredictor< Network, EdgeRndIt, Sc
LinkPred::UECLPredictor< Network, EdgeRndIt, ScoreRndIt, EdgeRndOutIt >, LinkPred::UESMPredictor< Network, EdgeRndIt, Sc
LinkPred::USBMPredictor< Network, EdgeRndIt, ScoreRndIt, EdgeRndOutIt >, LinkPred::UCSTPredictor< Network, EdgeRndIt, Sc
LinkPred::UADAPredictor< Network, EdgeRndIt, ScoreRndIt, EdgeRndOutIt >, LinkPred::UCNEPredictor< Network, EdgeRndIt, Sc
LinkPred::UCRAPredictor< Network, EdgeRndIt, ScoreRndIt, EdgeRndOutIt >, LinkPred::URNDPredictor< Network, EdgeRndIt, Sc
LinkPred::USUMPredictor< Network, EdgeRndIt, ScoreRndIt, EdgeRndOutIt >, LinkPred::URALPredictor< Network, EdgeRndIt, Sc
LinkPred::USOIPredictor< Network, EdgeRndIt, ScoreRndIt, EdgeRndOutIt >, LinkPred::UHDIPredictor< Network, EdgeRndIt, Sco
LinkPred::UHPIPredictor< Network, EdgeRndIt, ScoreRndIt, EdgeRndOutIt >, LinkPred::UJIDPredictor< Network, EdgeRndIt, Scor
LinkPred::ULHNPredictor< Network, EdgeRndIt, ScoreRndIt, EdgeRndOutIt >, LinkPred::UPATPredictor< Network, EdgeRndIt, Sc
LinkPred::USAIPredictor< Network, EdgeRndIt, ScoreRndIt, EdgeRndOutIt >, and LinkPred::ULCPPredictor< Network, EdgeRndIt,

9.128.4.5 operator=() [1/2]

```
template<typename NetworkT = UNetwork<>, typename EdgeRndItT = typename std::vector<typename
NetworkT::Edge>::const_iterator, typename ScoreRndItT = typename std::vector<double>::iterator,
typename EdgeRndOutItT = typename std::vector<typename NetworkT::Edge>::iterator>
ULPredictor& LinkPred::ULPredictor< NetworkT, EdgeRndItT, ScoreRndItT, EdgeRndOutItT >::operator=
(
            ULPredictor< NetworkT, EdgeRndItT, ScoreRndItT, EdgeRndOutItT > && that )  [default]
```

Move assignment operator.

Parameters

that	The object to move.

9.128.4.6 operator=() [2/2]

```
template<typename NetworkT = UNetwork<>, typename EdgeRndItT = typename std::vector<typename
NetworkT::Edge>::const_iterator, typename ScoreRndItT = typename std::vector<double>::iterator,
typename EdgeRndOutItT = typename std::vector<typename NetworkT::Edge>::iterator>
ULPredictor& LinkPred::ULPredictor< NetworkT, EdgeRndItT, ScoreRndItT, EdgeRndOutItT >::operator=
(
            ULPredictor< NetworkT, EdgeRndItT, ScoreRndItT, EdgeRndOutItT > const & that )
[default]
```

Copy assignment operator.

Parameters

that	The object to copy.

9.128.4.7 predict()

```
template<typename NetworkT = UNetwork<>, typename EdgeRndItT = typename std::vector<typename
NetworkT::Edge>::const_iterator, typename ScoreRndItT = typename std::vector<double>::iterator,
typename EdgeRndOutItT = typename std::vector<typename NetworkT::Edge>::iterator>
virtual void LinkPred::ULPredictor< NetworkT, EdgeRndItT, ScoreRndItT, EdgeRndOutItT >::predict
(
            EdgeRndIt begin,
            EdgeRndIt end,
            ScoreRndIt scores )  [inline], [virtual]
```

Predict links.

Parameters

begin	Iterator to the first edge to be predicted.
end	end Iterator to one past the last edge to be predicted.
scores	Random output iterator to store the scores.

Reimplemented in LinkPred::UHYPPredictor< Network, EdgeRndIt, ScoreRndIt, EdgeRndOutIt >, LinkPred::UMPSPredictor< Netw[illegible] LinkPred::UKABPredictor< Network, EdgeRndIt, ScoreRndIt, EdgeRndOutIt >, LinkPred::UHRGPredictor< Network, EdgeRndIt, Sc[illegible] LinkPred::UNEDPredictor< Network, EdgeRndIt, ScoreRndIt, EdgeRndOutIt >, LinkPred::UFBMPredictor< Network, EdgeRndIt, Sc[illegible] LinkPred::UCRAPredictor< Network, EdgeRndIt, ScoreRndIt, EdgeRndOutIt >, LinkPred::USHPPredictor< Network, EdgeRndIt, Sc[illegible] LinkPred::USBMPredictor< Network, EdgeRndIt, ScoreRndIt, EdgeRndOutIt >, LinkPred::UADAPredictor< Network, EdgeRndIt, Sc[illegible] LinkPred::URALPredictor< Network, EdgeRndIt, ScoreRndIt, EdgeRndOutIt >, LinkPred::UJIDPredictor< Network, EdgeRndIt, Sco[illegible] LinkPred::UCSTPredictor< Network, EdgeRndIt, ScoreRndIt, EdgeRndOutIt >, LinkPred::USAIPredictor< Network, EdgeRndIt, Sco[illegible] LinkPred::USOIPredictor< Network, EdgeRndIt, ScoreRndIt, EdgeRndOutIt >, LinkPred::UHDIPredictor< Network, EdgeRndIt, Sco[illegible] LinkPred::UHPIPredictor< Network, EdgeRndIt, ScoreRndIt, EdgeRndOutIt >, LinkPred::ULHNPredictor< Network, EdgeRndIt, Sco[illegible] LinkPred::UPATPredictor< Network, EdgeRndIt, ScoreRndIt, EdgeRndOutIt >, LinkPred::UCNEPredictor< Network, EdgeRndIt, Sc[illegible] LinkPred::URNDPredictor< Network, EdgeRndIt, ScoreRndIt, EdgeRndOutIt >, LinkPred::USUMPredictor< Network, EdgeRndIt, S[illegible] and LinkPred::ULCPPredictor< Network, EdgeRndIt, ScoreRndIt, EdgeRndOutIt >.

9.128.4.8 predictNeg()

```
template<typename NetworkT = UNetwork<>, typename EdgeRndItT = typename std::vector<typename
NetworkT::Edge>::const_iterator, typename ScoreRndItT = typename std::vector<double>::iterator,
typename EdgeRndOutItT = typename std::vector<typename NetworkT::Edge>::iterator>
virtual std::pair<typename Network::NonEdgeIt, typename Network::NonEdgeIt> LinkPred::ULPredictor<
NetworkT, EdgeRndItT, ScoreRndItT, EdgeRndOutItT >::predictNeg (
            ScoreRndIt scores )  [inline], [virtual]
```

Predict score for all negative (non-existing) links in the network.

Parameters

scores	Random output iterator to store the scores.

Returns

A pair of iterators begin and end to the range of non-existing links predicted by the method.

9.128.4.9 score()

```
template<typename NetworkT = UNetwork<>, typename EdgeRndItT = typename std::vector<typename
NetworkT::Edge>::const_iterator, typename ScoreRndItT = typename std::vector<double>::iterator,
typename EdgeRndOutItT = typename std::vector<typename NetworkT::Edge>::iterator>
virtual double LinkPred::ULPredictor< NetworkT, EdgeRndItT, ScoreRndItT, EdgeRndOutItT >↩
::score (
            Edge const & e )  [pure virtual]
```

Compute the score of a single edge.

Parameters

e	The edge.

Returns

The score of e.

9.128.4.10 setName()

```
template<typename NetworkT = UNetwork<>, typename EdgeRndItT = typename std::vector<typename
NetworkT::Edge>::const_iterator, typename ScoreRndItT = typename std::vector<double>::iterator,
typename EdgeRndOutItT = typename std::vector<typename NetworkT::Edge>::iterator>
void LinkPred::ULPredictor< NetworkT, EdgeRndItT, ScoreRndItT, EdgeRndOutItT >::setName (
            const std::string & name )  [inline]
```

Set the name of the predictor.

Parameters

name	The new name of the predictor.

9.128.4.11 top()

```
template<typename NetworkT = UNetwork<>, typename EdgeRndItT = typename std::vector<typename
NetworkT::Edge>::const_iterator, typename ScoreRndItT = typename std::vector<double>::iterator,
typename EdgeRndOutItT = typename std::vector<typename NetworkT::Edge>::iterator>
virtual std::size_t LinkPred::ULPredictor< NetworkT, EdgeRndItT, ScoreRndItT, EdgeRndOutItT
>::top (
            std::size_t k,
            EdgeRndOutIt eit,
            ScoreRndIt sit )  [inline], [virtual]
```

Finds the k negative edges with the top score. Ties are broken randomly.

Parameters

k	The number of edges to find.
eit	An output iterator where the edges are written.
sit	An output iterator where the scores are written. The scores are written in the same order as the edges.

Returns

The number of negative edges inserted. It is the minimum between k and the number of negative edges in the network.

Reimplemented in LinkPred::UKABPredictor< Network, EdgeRndIt, ScoreRndIt, EdgeRndOutIt >, LinkPred::UNEDPredictor< Netw[illegible] LinkPred::UCRAPredictor< Network, EdgeRndIt, ScoreRndIt, EdgeRndOutIt >, LinkPred::UADAPredictor< Network, EdgeRndIt, Sc[illegible] LinkPred::URALPredictor< Network, EdgeRndIt, ScoreRndIt, EdgeRndOutIt >, LinkPred::UJIDPredictor< Network, EdgeRndIt, Sco[illegible] LinkPred::UCSTPredictor< Network, EdgeRndIt, ScoreRndIt, EdgeRndOutIt >, LinkPred::USAIPredictor< Network, EdgeRndIt, Sco[illegible] LinkPred::USOIPredictor< Network, EdgeRndIt, ScoreRndIt, EdgeRndOutIt >, LinkPred::UHDIPredictor< Network, EdgeRndIt, Sco[illegible] LinkPred::UHPIPredictor< Network, EdgeRndIt, ScoreRndIt, EdgeRndOutIt >, LinkPred::ULHNPredictor< Network, EdgeRndIt, Sco[illegible] LinkPred::UPATPredictor< Network, EdgeRndIt, ScoreRndIt, EdgeRndOutIt >, LinkPred::UCNEPredictor< Network, EdgeRndIt, Sc[illegible] and LinkPred::USUMPredictor< Network, EdgeRndIt, ScoreRndIt, EdgeRndOutIt >.

The documentation for this class was generated from the following file:

- include/linkpred/predictors/undirected/ulpredictor.hpp

9.129 LinkPred::UMPSPredictor< Network, EdgeRndIt, ScoreRndIt, EdgeRndOutIt > Class Template Reference

A scalable popularity similarity link predictor.

```
#include <umpspredictor.hpp>
```

Inheritance diagram for LinkPred::UMPSPredictor< Network, EdgeRndIt, ScoreRndIt, EdgeRndOutIt >:

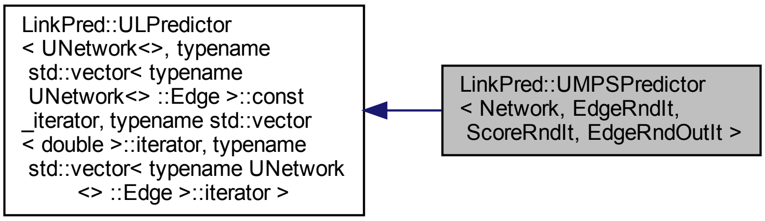

Collaboration diagram for LinkPred::UMPSPredictor< Network, EdgeRndIt, ScoreRndIt, EdgeRndOutIt >:

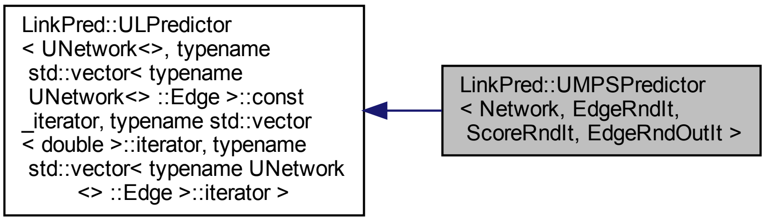

Public Types

- enum LandmarkStrategy { Random, Hub, IHub }

  An enumeration of the different landmark positioning strategies.
- enum LambdaMethod { User, MeanApp, Scan, Opt }

  An enumeration of different methods to find lambda.
- enum EdgeLengthMethod { PAT, RA1, RA2 }

  An enumeration of edge length methods.

Public Member Functions

- UMPSPredictor (std::shared_ptr< Network const > net, double remRatio, long int seed)
- UMPSPredictor (UMPSPredictor const &that)=default
- UMPSPredictor & operator= (UMPSPredictor const &that)=default
- UMPSPredictor (UMPSPredictor &&that)=default
- UMPSPredictor & operator= (UMPSPredictor &&that)=default
- virtual void init ()
- virtual void learn ()
- virtual double score (Edge const &e)
- virtual void predict (EdgeRndIt begin, EdgeRndIt end, ScoreRndIt scores)
- auto getLength () const

- bool getAsp () const
- void setAsp (bool asp)
- double getLandmarkRatio () const
- void setLandmarkRatio (double landmarkRatio)
- LandmarkStrategy getLandmarkStrategy () const
- void setLandmarkStrategy (LandmarkStrategy landmarkStrategy)
- double getLambda () const
- void setLambda (double lambda)
- CacheLevel getCacheLevel () const
- void setCacheLevel (CacheLevel cacheLevel)
- double getNegScore () const
- void setNegScore (double negScore)
- double getPosScore () const
- void setPosScore (double posScore)
- LambdaMethod getLambdaMethod () const
- void setLambdaMethod (LambdaMethod lambdaMethod)
- double getLambdaStep () const
- void setLambdaStep (double lambdaStep)
- double getMaxLambda () const
- void setMaxLambda (double maxLambda)
- double getMinLambda () const
- void setMinLambda (double minLambda)
- auto getPerfMeasure () const
- void setPerfMeasure (std::shared_ptr< PerfMeasure<>> perfMeasure)
- double getTol () const
- void setTol (double tol)
- double getLambdaNegEstRatio () const
- void setLambdaNegEstRatio (double lambdaNegEstRatio)
- double getLambdaPosEstRatio () const
- void setLambdaPosEstRatio (double lambdaPosEstRatio)
- EdgeLengthMethod getEdgeLengthMethod () const
- void setEdgeLengthMethod (EdgeLengthMethod edgeLengthMethod)
- virtual ~UMPSPredictor ()=default

9.129.1 Detailed Description

template<typename Network = UNetwork<>, typename EdgeRndIt = typename std::vector<typename Network::Edge>↩ ::const_iterator, typename ScoreRndIt = typename std::vector<double>::iterator, typename EdgeRndOutIt = typename std↩ ::vector<typename Network::Edge>::iterator>
class LinkPred::UMPSPredictor< Network, EdgeRndIt, ScoreRndIt, EdgeRndOutIt >

A scalable popularity similarity link predictor.

Template Parameters

Network	The network type.
Edge↩ RndIt	A random iterator type used to iterate on edges.
Score↩ RndIt	A random iterator type used to iterate on scores.

9.129.2 Member Enumeration Documentation

9.129.2.1 EdgeLengthMethod

```
template<typename Network = UNetwork<>, typename EdgeRndIt = typename std::vector<typename
Network::Edge>::const_iterator, typename ScoreRndIt = typename std::vector<double>::iterator,
typename EdgeRndOutIt = typename std::vector<typename Network::Edge>::iterator>
enum LinkPred::UMPSPredictor::EdgeLengthMethod
```

An enumeration of edge length methods.

Enumerator

PAT	Degree products.
RA1	RA1 method.
RA2	RA2 method.

9.129.2.2 LambdaMethod

```
template<typename Network = UNetwork<>, typename EdgeRndIt = typename std::vector<typename
Network::Edge>::const_iterator, typename ScoreRndIt = typename std::vector<double>::iterator,
typename EdgeRndOutIt = typename std::vector<typename Network::Edge>::iterator>
enum LinkPred::UMPSPredictor::LambdaMethod
```

An enumeration of different methods to find lambda.

Enumerator

User	Use the value fixed by the user.
MeanApp	Lambda is approximated by an average.
Scan	Scan specified values and choose the best.
Opt	Find lambda by optimization.

9.129.2.3 LandmarkStrategy

```
template<typename Network = UNetwork<>, typename EdgeRndIt = typename std::vector<typename
Network::Edge>::const_iterator, typename ScoreRndIt = typename std::vector<double>::iterator,
typename EdgeRndOutIt = typename std::vector<typename Network::Edge>::iterator>
enum LinkPred::UMPSPredictor::LandmarkStrategy
```

An enumeration of the different landmark positioning strategies.

Enumerator

Random	Landmarks are chosen randomly.
Hub	The nodes with the highest degree are chosen.
lHub	The nodes with the lowest degree are chosen.

9.129.3 Constructor & Destructor Documentation

9.129.3.1 UMPSPredictor() [1/3]

```
template<typename Network = UNetwork<>, typename EdgeRndIt = typename std::vector<typename
Network::Edge>::const_iterator, typename ScoreRndIt = typename std::vector<double>::iterator,
typename EdgeRndOutIt = typename std::vector<typename Network::Edge>::iterator>
LinkPred::UMPSPredictor< Network, EdgeRndIt, ScoreRndIt, EdgeRndOutIt >::UMPSPredictor (
            std::shared_ptr< Network const > net,
            double remRatio,
            long int seed ) [inline]
```

Parameters

net	The network.
seed	The random number generator's seed.
remRatio	Ratio of removed links.

9.129.3.2 UMPSPredictor() [2/3]

```
template<typename Network = UNetwork<>, typename EdgeRndIt = typename std::vector<typename
Network::Edge>::const_iterator, typename ScoreRndIt = typename std::vector<double>::iterator,
typename EdgeRndOutIt = typename std::vector<typename Network::Edge>::iterator>
LinkPred::UMPSPredictor< Network, EdgeRndIt, ScoreRndIt, EdgeRndOutIt >::UMPSPredictor (
            UMPSPredictor< Network, EdgeRndIt, ScoreRndIt, EdgeRndOutIt > const & that )
[default]
```

Copy constructor.

Parameters

that	The object to copy.

9.129.3.3 UMPSPredictor() [3/3]

```
template<typename Network = UNetwork<>, typename EdgeRndIt = typename std::vector<typename
```

```
Network::Edge>::const_iterator, typename ScoreRndIt = typename std::vector<double>::iterator,
typename EdgeRndOutIt = typename std::vector<typename Network::Edge>::iterator>
LinkPred::UMPSPredictor< Network, EdgeRndIt, ScoreRndIt, EdgeRndOutIt >::UMPSPredictor (
            UMPSPredictor< Network, EdgeRndIt, ScoreRndIt, EdgeRndOutIt > && that )  [default]
```

Move constructor.

Parameters

that	The object to move.

9.129.3.4 ∼UMPSPredictor()

```
template<typename Network = UNetwork<>, typename EdgeRndIt = typename std::vector<typename
Network::Edge>::const_iterator, typename ScoreRndIt = typename std::vector<double>::iterator,
typename EdgeRndOutIt = typename std::vector<typename Network::Edge>::iterator>
virtual LinkPred::UMPSPredictor< Network, EdgeRndIt, ScoreRndIt, EdgeRndOutIt >::~UMPSPredictor
( )  [virtual], [default]
```

Destructor.

9.129.4 Member Function Documentation

9.129.4.1 getAsp()

```
template<typename Network = UNetwork<>, typename EdgeRndIt = typename std::vector<typename
Network::Edge>::const_iterator, typename ScoreRndIt = typename std::vector<double>::iterator,
typename EdgeRndOutIt = typename std::vector<typename Network::Edge>::iterator>
bool LinkPred::UMPSPredictor< Network, EdgeRndIt, ScoreRndIt, EdgeRndOutIt >::getAsp ( ) const
[inline]
```

Returns

Whether approximate shortest path distances are used.

9.129.4.2 getCacheLevel()

```
template<typename Network = UNetwork<>, typename EdgeRndIt = typename std::vector<typename
Network::Edge>::const_iterator, typename ScoreRndIt = typename std::vector<double>::iterator,
typename EdgeRndOutIt = typename std::vector<typename Network::Edge>::iterator>
CacheLevel LinkPred::UMPSPredictor< Network, EdgeRndIt, ScoreRndIt, EdgeRndOutIt >::getCache↩
Level ( ) const  [inline]
```

Returns

The distances cache level.

9.129.4.3 getEdgeLengthMethod()

```
template<typename Network = UNetwork<>, typename EdgeRndIt = typename std::vector<typename
Network::Edge>::const_iterator, typename ScoreRndIt = typename std::vector<double>::iterator,
typename EdgeRndOutIt = typename std::vector<typename Network::Edge>::iterator>
EdgeLengthMethod LinkPred::UMPSPredictor< Network, EdgeRndIt, ScoreRndIt, EdgeRndOutIt >↩
::getEdgeLengthMethod ( ) const [inline]
```

Returns

The method used to compute edge lengths.

9.129.4.4 getLambda()

```
template<typename Network = UNetwork<>, typename EdgeRndIt = typename std::vector<typename
Network::Edge>::const_iterator, typename ScoreRndIt = typename std::vector<double>::iterator,
typename EdgeRndOutIt = typename std::vector<typename Network::Edge>::iterator>
double LinkPred::UMPSPredictor< Network, EdgeRndIt, ScoreRndIt, EdgeRndOutIt >::getLambda ( )
const [inline]
```

Returns

The parameter lambda.

9.129.4.5 getLambdaMethod()

```
template<typename Network = UNetwork<>, typename EdgeRndIt = typename std::vector<typename
Network::Edge>::const_iterator, typename ScoreRndIt = typename std::vector<double>::iterator,
typename EdgeRndOutIt = typename std::vector<typename Network::Edge>::iterator>
LambdaMethod LinkPred::UMPSPredictor< Network, EdgeRndIt, ScoreRndIt, EdgeRndOutIt >::get↩
LambdaMethod ( ) const [inline]
```

Returns

The lambda estimation method.

9.129.4.6 getLambdaNegEstRatio()

```
template<typename Network = UNetwork<>, typename EdgeRndIt = typename std::vector<typename
Network::Edge>::const_iterator, typename ScoreRndIt = typename std::vector<double>::iterator,
typename EdgeRndOutIt = typename std::vector<typename Network::Edge>::iterator>
double LinkPred::UMPSPredictor< Network, EdgeRndIt, ScoreRndIt, EdgeRndOutIt >::getLambdaNeg↩
EstRatio ( ) const [inline]
```

Returns

Ratio of negative links used to estimate lambda (used only if lambdaMethod != User).

9.129.4.7 getLambdaPosEstRatio()

template<typename Network = UNetwork<>, typename EdgeRndIt = typename std::vector<typename Network::Edge>::const_iterator, typename ScoreRndIt = typename std::vector<double>::iterator, typename EdgeRndOutIt = typename std::vector<typename Network::Edge>::iterator>
double LinkPred::UMPSPredictor< Network, EdgeRndIt, ScoreRndIt, EdgeRndOutIt >::getLambdaPos↩
EstRatio ( ) const [inline]

Returns

Ratio of positive links used to estimate lambda (used only if lambdaMethod != User).

9.129.4.8 getLambdaStep()

template<typename Network = UNetwork<>, typename EdgeRndIt = typename std::vector<typename Network::Edge>::const_iterator, typename ScoreRndIt = typename std::vector<double>::iterator, typename EdgeRndOutIt = typename std::vector<typename Network::Edge>::iterator>
double LinkPred::UMPSPredictor< Network, EdgeRndIt, ScoreRndIt, EdgeRndOutIt >::getLambdaStep ( ) const [inline]

Returns

The step size when scanning for lambda. Used when betMethod is set to Scan.

9.129.4.9 getLandmarkRatio()

template<typename Network = UNetwork<>, typename EdgeRndIt = typename std::vector<typename Network::Edge>::const_iterator, typename ScoreRndIt = typename std::vector<double>::iterator, typename EdgeRndOutIt = typename std::vector<typename Network::Edge>::iterator>
double LinkPred::UMPSPredictor< Network, EdgeRndIt, ScoreRndIt, EdgeRndOutIt >::getLandmark↩
Ratio ( ) const [inline]

Returns

The landmark ratio.

9.129.4.10 getLandmarkStrategy()

template<typename Network = UNetwork<>, typename EdgeRndIt = typename std::vector<typename Network::Edge>::const_iterator, typename ScoreRndIt = typename std::vector<double>::iterator, typename EdgeRndOutIt = typename std::vector<typename Network::Edge>::iterator>
LandmarkStrategy LinkPred::UMPSPredictor< Network, EdgeRndIt, ScoreRndIt, EdgeRndOutIt >↩
::getLandmarkStrategy ( ) const [inline]

Returns

The landmark strategy.

9.129.4.11 getLength()

```
template<typename Network = UNetwork<>, typename EdgeRndIt = typename std::vector<typename
Network::Edge>::const_iterator, typename ScoreRndIt = typename std::vector<double>::iterator,
typename EdgeRndOutIt = typename std::vector<typename Network::Edge>::iterator>
auto LinkPred::UMPSPredictor< Network, EdgeRndIt, ScoreRndIt, EdgeRndOutIt >::getLength ( )
const [inline]
```

Returns

The length map.

9.129.4.12 getMaxLambda()

```
template<typename Network = UNetwork<>, typename EdgeRndIt = typename std::vector<typename
Network::Edge>::const_iterator, typename ScoreRndIt = typename std::vector<double>::iterator,
typename EdgeRndOutIt = typename std::vector<typename Network::Edge>::iterator>
double LinkPred::UMPSPredictor< Network, EdgeRndIt, ScoreRndIt, EdgeRndOutIt >::getMaxLambda (
) const [inline]
```

Returns

The maximum lambda value to try. Used when betMethod is set to Scan.

9.129.4.13 getMinLambda()

```
template<typename Network = UNetwork<>, typename EdgeRndIt = typename std::vector<typename
Network::Edge>::const_iterator, typename ScoreRndIt = typename std::vector<double>::iterator,
typename EdgeRndOutIt = typename std::vector<typename Network::Edge>::iterator>
double LinkPred::UMPSPredictor< Network, EdgeRndIt, ScoreRndIt, EdgeRndOutIt >::getMinLambda (
) const [inline]
```

Returns

The minimum lambda value to try. Used when betMethod is set to Scan.

9.129.4.14 getNegScore()

```
template<typename Network = UNetwork<>, typename EdgeRndIt = typename std::vector<typename
Network::Edge>::const_iterator, typename ScoreRndIt = typename std::vector<double>::iterator,
typename EdgeRndOutIt = typename std::vector<typename Network::Edge>::iterator>
double LinkPred::UMPSPredictor< Network, EdgeRndIt, ScoreRndIt, EdgeRndOutIt >::getNegScore (
) const [inline]
```

Returns

Score given to a negative edge.

9.129.4.15 getPerfMeasure()

```
template<typename Network = UNetwork<>, typename EdgeRndIt = typename std::vector<typename
Network::Edge>::const_iterator, typename ScoreRndIt = typename std::vector<double>::iterator,
typename EdgeRndOutIt = typename std::vector<typename Network::Edge>::iterator>
auto LinkPred::UMPSPredictor< Network, EdgeRndIt, ScoreRndIt, EdgeRndOutIt >::getPerfMeasure (
) const [inline]
```

Returns

The performance measure used to determine the best edge score method.

9.129.4.16 getPosScore()

```
template<typename Network = UNetwork<>, typename EdgeRndIt = typename std::vector<typename
Network::Edge>::const_iterator, typename ScoreRndIt = typename std::vector<double>::iterator,
typename EdgeRndOutIt = typename std::vector<typename Network::Edge>::iterator>
double LinkPred::UMPSPredictor< Network, EdgeRndIt, ScoreRndIt, EdgeRndOutIt >::getPosScore (
) const [inline]
```

Returns

Score given to a positive edge.

9.129.4.17 getTol()

```
template<typename Network = UNetwork<>, typename EdgeRndIt = typename std::vector<typename
Network::Edge>::const_iterator, typename ScoreRndIt = typename std::vector<double>::iterator,
typename EdgeRndOutIt = typename std::vector<typename Network::Edge>::iterator>
double LinkPred::UMPSPredictor< Network, EdgeRndIt, ScoreRndIt, EdgeRndOutIt >::getTol ( )
const [inline]
```

Returns

The tolerance when optimizing for lambda. Used only when lambdaMethod == Opt.

9.129.4.18 init()

```
template<typename Network = UNetwork<>, typename EdgeRndIt = typename std::vector<typename
Network::Edge>::const_iterator, typename ScoreRndIt = typename std::vector<double>::iterator,
typename EdgeRndOutIt = typename std::vector<typename Network::Edge>::iterator>
virtual void LinkPred::UMPSPredictor< Network, EdgeRndIt, ScoreRndIt, EdgeRndOutIt >::init ( )
[virtual]
```

Initialize the predictor.

Implements LinkPred::ULPredictor< UNetwork<>, typename std::vector< typename UNetwork<> ::Edge >::const_iterator, typena

9.129.4.19 learn()

```
template<typename Network = UNetwork<>, typename EdgeRndIt = typename std::vector<typename
Network::Edge>::const_iterator, typename ScoreRndIt = typename std::vector<double>::iterator,
typename EdgeRndOutIt = typename std::vector<typename Network::Edge>::iterator>
virtual void LinkPred::UMPSPredictor< Network, EdgeRndIt, ScoreRndIt, EdgeRndOutIt >::learn (
) [virtual]
```

Learn.

Implements LinkPred::ULPredictor< UNetwork<>, typename std::vector< typename UNetwork<> ::Edge >::const_iterator, typena

9.129.4.20 operator=() [1/2]

```
template<typename Network = UNetwork<>, typename EdgeRndIt = typename std::vector<typename
Network::Edge>::const_iterator, typename ScoreRndIt = typename std::vector<double>::iterator,
typename EdgeRndOutIt = typename std::vector<typename Network::Edge>::iterator>
UMPSPredictor& LinkPred::UMPSPredictor< Network, EdgeRndIt, ScoreRndIt, EdgeRndOutIt >::operator=
(
            UMPSPredictor< Network, EdgeRndIt, ScoreRndIt, EdgeRndOutIt > && that )  [default]
```

Move assignment operator.

Parameters

that	The object to move.

9.129.4.21 operator=() [2/2]

```
template<typename Network = UNetwork<>, typename EdgeRndIt = typename std::vector<typename
Network::Edge>::const_iterator, typename ScoreRndIt = typename std::vector<double>::iterator,
typename EdgeRndOutIt = typename std::vector<typename Network::Edge>::iterator>
UMPSPredictor& LinkPred::UMPSPredictor< Network, EdgeRndIt, ScoreRndIt, EdgeRndOutIt >::operator=
(
            UMPSPredictor< Network, EdgeRndIt, ScoreRndIt, EdgeRndOutIt > const & that )
[default]
```

Copy assignment operator.

Parameters

that	The object to copy.

9.129.4.22 predict()

```
template<typename Network = UNetwork<>, typename EdgeRndIt = typename std::vector<typename
Network::Edge>::const_iterator, typename ScoreRndIt = typename std::vector<double>::iterator,
typename EdgeRndOutIt = typename std::vector<typename Network::Edge>::iterator>
virtual void LinkPred::UMPSPredictor< Network, EdgeRndIt, ScoreRndIt, EdgeRndOutIt >::predict
(
            EdgeRndIt begin,
            EdgeRndIt end,
            ScoreRndIt scores )  [virtual]
```

Predict the links.

Parameters

begin	Beginning of the links to be predicted.
end	end of the links to be predicted.
scores	Beginning of scores.

Reimplemented from LinkPred::ULPredictor< UNetwork<>, typename std::vector< typename UNetwork<> ::Edge >::const_iterat

9.129.4.23 score()

```
template<typename Network = UNetwork<>, typename EdgeRndIt = typename std::vector<typename
Network::Edge>::const_iterator, typename ScoreRndIt = typename std::vector<double>::iterator,
typename EdgeRndOutIt = typename std::vector<typename Network::Edge>::iterator>
virtual double LinkPred::UMPSPredictor< Network, EdgeRndIt, ScoreRndIt, EdgeRndOutIt >::score
(
            Edge const & e )  [inline], [virtual]
```

Compute the score of a single edge.

Parameters

e	The edge.

Returns

The score of e.

9.129.4.24 setAsp()

```
template<typename Network = UNetwork<>, typename EdgeRndIt = typename std::vector<typename
Network::Edge>::const_iterator, typename ScoreRndIt = typename std::vector<double>::iterator,
typename EdgeRndOutIt = typename std::vector<typename Network::Edge>::iterator>
void LinkPred::UMPSPredictor< Network, EdgeRndIt, ScoreRndIt, EdgeRndOutIt >::setAsp (
            bool asp )  [inline]
```

Set Whether approximate shortest path distances are used.

Parameters

asp	Whether approximate shortest path distances should be used.

9.129.4.25 setCacheLevel()

```
template<typename Network = UNetwork<>, typename EdgeRndIt = typename std::vector<typename
Network::Edge>::const_iterator, typename ScoreRndIt = typename std::vector<double>::iterator,
typename EdgeRndOutIt = typename std::vector<typename Network::Edge>::iterator>
void LinkPred::UMPSPredictor< Network, EdgeRndIt, ScoreRndIt, EdgeRndOutIt >::setCacheLevel (
            CacheLevel cacheLevel ) [inline]
```

Set the distances cache level.

Parameters

cacheLevel	The new distances cache level.

9.129.4.26 setEdgeLengthMethod()

```
template<typename Network = UNetwork<>, typename EdgeRndIt = typename std::vector<typename
Network::Edge>::const_iterator, typename ScoreRndIt = typename std::vector<double>::iterator,
typename EdgeRndOutIt = typename std::vector<typename Network::Edge>::iterator>
void LinkPred::UMPSPredictor< Network, EdgeRndIt, ScoreRndIt, EdgeRndOutIt >::setEdgeLength↩
Method (
            EdgeLengthMethod edgeLengthMethod ) [inline]
```

Set the edge length method.

Parameters

edgeLengthMethod	The new edge length method.

9.129.4.27 setLambda()

```
template<typename Network = UNetwork<>, typename EdgeRndIt = typename std::vector<typename
Network::Edge>::const_iterator, typename ScoreRndIt = typename std::vector<double>::iterator,
typename EdgeRndOutIt = typename std::vector<typename Network::Edge>::iterator>
void LinkPred::UMPSPredictor< Network, EdgeRndIt, ScoreRndIt, EdgeRndOutIt >::setLambda (
            double lambda ) [inline]
```

Set the parameter lambda.

Parameters

lambda	The new value of the parameter lambda.

9.129.4.28 setLambdaMethod()

```
template<typename Network = UNetwork<>, typename EdgeRndIt = typename std::vector<typename
Network::Edge>::const_iterator, typename ScoreRndIt = typename std::vector<double>::iterator,
typename EdgeRndOutIt = typename std::vector<typename Network::Edge>::iterator>
void LinkPred::UMPSPredictor< Network, EdgeRndIt, ScoreRndIt, EdgeRndOutIt >::setLambdaMethod
(
            LambdaMethod lambdaMethod ) [inline]
```

Set the lambda estimation method.

Parameters

lambdaMethod	The new lambda estimation method.

9.129.4.29 setLambdaNegEstRatio()

```
template<typename Network = UNetwork<>, typename EdgeRndIt = typename std::vector<typename
Network::Edge>::const_iterator, typename ScoreRndIt = typename std::vector<double>::iterator,
typename EdgeRndOutIt = typename std::vector<typename Network::Edge>::iterator>
void LinkPred::UMPSPredictor< Network, EdgeRndIt, ScoreRndIt, EdgeRndOutIt >::setLambdaNeg←
EstRatio (
            double lambdaNegEstRatio ) [inline]
```

Set the ratio of negative links used to estimate lambda (used only if lambdaMethod != User).

Parameters

lambdaNegEstRatio	The new ratio of negative links used to estimate lambda (used only if lambdaMethod != User).

9.129.4.30 setLambdaPosEstRatio()

```
template<typename Network = UNetwork<>, typename EdgeRndIt = typename std::vector<typename
Network::Edge>::const_iterator, typename ScoreRndIt = typename std::vector<double>::iterator,
typename EdgeRndOutIt = typename std::vector<typename Network::Edge>::iterator>
void LinkPred::UMPSPredictor< Network, EdgeRndIt, ScoreRndIt, EdgeRndOutIt >::setLambdaPos←
EstRatio (
            double lambdaPosEstRatio ) [inline]
```

Set the ratio of positive links used to estimate lambda (used only if lambdaMethod != User).

Parameters

lambdaPosEstRatio	The new ratio of positive links used to estimate lambda (used only if lambdaMethod != User).

9.129.4.31 setLambdaStep()

```
template<typename Network = UNetwork<>, typename EdgeRndIt = typename std::vector<typename
Network::Edge>::const_iterator, typename ScoreRndIt = typename std::vector<double>::iterator,
typename EdgeRndOutIt = typename std::vector<typename Network::Edge>::iterator>
void LinkPred::UMPSPredictor< Network, EdgeRndIt, ScoreRndIt, EdgeRndOutIt >::setLambdaStep (
            double lambdaStep )  [inline]
```

Set the step size when scanning for lambda. Used when betMethod is set to Scan.

Parameters

lambdaStep	The new step size when scanning for lambda. Used when betMethod is set to Scan.

9.129.4.32 setLandmarkRatio()

```
template<typename Network = UNetwork<>, typename EdgeRndIt = typename std::vector<typename
Network::Edge>::const_iterator, typename ScoreRndIt = typename std::vector<double>::iterator,
typename EdgeRndOutIt = typename std::vector<typename Network::Edge>::iterator>
void LinkPred::UMPSPredictor< Network, EdgeRndIt, ScoreRndIt, EdgeRndOutIt >::setLandmarkRatio
(
            double landmarkRatio )  [inline]
```

Set the landmark ratio.

Parameters

landmarkRatio	The new landmark ratio.

9.129.4.33 setLandmarkStrategy()

```
template<typename Network = UNetwork<>, typename EdgeRndIt = typename std::vector<typename
Network::Edge>::const_iterator, typename ScoreRndIt = typename std::vector<double>::iterator,
typename EdgeRndOutIt = typename std::vector<typename Network::Edge>::iterator>
void LinkPred::UMPSPredictor< Network, EdgeRndIt, ScoreRndIt, EdgeRndOutIt >::setLandmark←
Strategy (
            LandmarkStrategy landmarkStrategy )  [inline]
```

Set the landmark strategy.

Parameters

landmarkStrategy	The new landmark strategy.

9.129.4.34 setMaxLambda()

```
template<typename Network = UNetwork<>, typename EdgeRndIt = typename std::vector<typename
Network::Edge>::const_iterator, typename ScoreRndIt = typename std::vector<double>::iterator,
typename EdgeRndOutIt = typename std::vector<typename Network::Edge>::iterator>
void LinkPred::UMPSPredictor< Network, EdgeRndIt, ScoreRndIt, EdgeRndOutIt >::setMaxLambda (
            double maxLambda )  [inline]
```

Set the maximum lambda value to try. Used when betMethod is set to Scan.

Parameters

maxLambda	The new maximum lambda value to try. Used when betMethod is set to Scan.

9.129.4.35 setMinLambda()

```
template<typename Network = UNetwork<>, typename EdgeRndIt = typename std::vector<typename
Network::Edge>::const_iterator, typename ScoreRndIt = typename std::vector<double>::iterator,
typename EdgeRndOutIt = typename std::vector<typename Network::Edge>::iterator>
void LinkPred::UMPSPredictor< Network, EdgeRndIt, ScoreRndIt, EdgeRndOutIt >::setMinLambda (
            double minLambda )  [inline]
```

Set the minimum lambda value to try. Used when betMethod is set to Scan.

Parameters

minLambda	The new minimum lambda value to try. Used when betMethod is set to Scan.

9.129.4.36 setNegScore()

```
template<typename Network = UNetwork<>, typename EdgeRndIt = typename std::vector<typename
Network::Edge>::const_iterator, typename ScoreRndIt = typename std::vector<double>::iterator,
typename EdgeRndOutIt = typename std::vector<typename Network::Edge>::iterator>
void LinkPred::UMPSPredictor< Network, EdgeRndIt, ScoreRndIt, EdgeRndOutIt >::setNegScore (
            double negScore )  [inline]
```

Set the score given to a negative edge.

Parameters

negScore	The new score given to a negative edge.

9.129.4.37 setPerfMeasure()

```
template<typename Network = UNetwork<>, typename EdgeRndIt = typename std::vector<typename
Network::Edge>::const_iterator, typename ScoreRndIt = typename std::vector<double>::iterator,
typename EdgeRndOutIt = typename std::vector<typename Network::Edge>::iterator>
void LinkPred::UMPSPredictor< Network, EdgeRndIt, ScoreRndIt, EdgeRndOutIt >::setPerfMeasure (
            std::shared_ptr< PerfMeasure<>> perfMeasure ) [inline]
```

Set the performance measure used to determine the best edge score method.

Parameters

perfMeasure	The new performance measure used to determine the best edge score method.

9.129.4.38 setPosScore()

```
template<typename Network = UNetwork<>, typename EdgeRndIt = typename std::vector<typename
Network::Edge>::const_iterator, typename ScoreRndIt = typename std::vector<double>::iterator,
typename EdgeRndOutIt = typename std::vector<typename Network::Edge>::iterator>
void LinkPred::UMPSPredictor< Network, EdgeRndIt, ScoreRndIt, EdgeRndOutIt >::setPosScore (
            double posScore ) [inline]
```

Set the score given to a positive edge.

Parameters

posScore	The new score given to a positive edge.

9.129.4.39 setTol()

```
template<typename Network = UNetwork<>, typename EdgeRndIt = typename std::vector<typename
Network::Edge>::const_iterator, typename ScoreRndIt = typename std::vector<double>::iterator,
typename EdgeRndOutIt = typename std::vector<typename Network::Edge>::iterator>
void LinkPred::UMPSPredictor< Network, EdgeRndIt, ScoreRndIt, EdgeRndOutIt >::setTol (
            double tol ) [inline]
```

Set the tolerance when optimizing for lambda. Used only when lambdaMethod == Opt.

Parameters

tol	The new tolerance when optimizing for lambda. Used only when lambdaMethod == Opt.

The documentation for this class was generated from the following file:

- include/linkpred/predictors/undirected/umpspredictor.hpp

9.130 LinkPred::UNEDPredictor< Network, EdgeRndIt, ScoreRndIt, EdgeRndOutIt > Class Template Reference

A neighbors degree link predictor.

```
#include <unedpredictor.hpp>
```

Inheritance diagram for LinkPred::UNEDPredictor< Network, EdgeRndIt, ScoreRndIt, EdgeRndOutIt >:

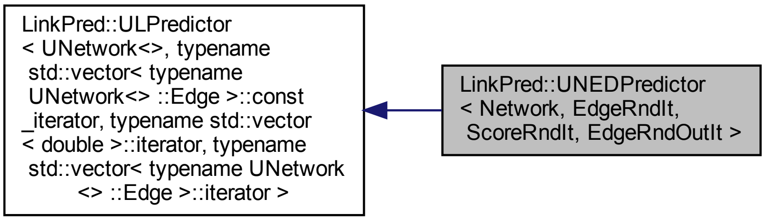

Collaboration diagram for LinkPred::UNEDPredictor< Network, EdgeRndIt, ScoreRndIt, EdgeRndOutIt >:

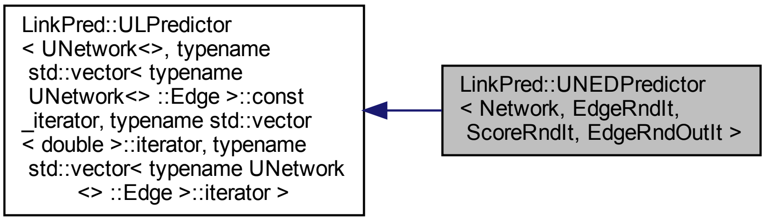

Public Types

- using NodeID = typename ULPredictor< Network, EdgeRndIt, ScoreRndIt, EdgeRndOutIt >::NodeID
- using Edge = typename ULPredictor< Network, EdgeRndIt, ScoreRndIt, EdgeRndOutIt >::Edge

Public Member Functions

- UNEDPredictor (std::shared_ptr< Network const > net)
- UNEDPredictor (UNEDPredictor const &that)=default
- UNEDPredictor & operator= (UNEDPredictor const &that)=default
- UNEDPredictor (UNEDPredictor &&that)=default
- UNEDPredictor & operator= (UNEDPredictor &&that)=default
- virtual void init ()
- virtual void learn ()
- virtual double score (Edge const &e)
- virtual void predict (EdgeRndIt begin, EdgeRndIt end, ScoreRndIt scores)
- virtual std::size_t top (std::size_t k, EdgeRndOutIt eit, ScoreRndIt sit)
- virtual ~UNEDPredictor ()=default

9.130.1 Detailed Description

template<typename Network = UNetwork<>, typename EdgeRndIt = typename std::vector<typename Network::Edge>↩ ::const_iterator, typename ScoreRndIt = typename std::vector<double>::iterator, typename EdgeRndOutIt = typename std↩ ::vector<typename Network::Edge>::iterator>
class LinkPred::UNEDPredictor< Network, EdgeRndIt, ScoreRndIt, EdgeRndOutIt >

A neighbors degree link predictor.

Template Parameters

Network	The network type.
Edge↩ RndIt	A random iterator type used to iterate on edges.
Score↩ RndIt	A random iterator type used to iterate on scores.

9.130.2 Member Typedef Documentation

9.130.2.1 Edge

```
template<typename Network = UNetwork<>, typename EdgeRndIt = typename std::vector<typename
Network::Edge>::const_iterator, typename ScoreRndIt = typename std::vector<double>::iterator,
typename EdgeRndOutIt = typename std::vector<typename Network::Edge>::iterator>
using LinkPred::UNEDPredictor< Network, EdgeRndIt, ScoreRndIt, EdgeRndOutIt >::Edge = typename
ULPredictor<Network, EdgeRndIt,ScoreRndIt, EdgeRndOutIt>::Edge
```

The edges type.

9.130.2.2 NodeID

```
template<typename Network = UNetwork<>, typename EdgeRndIt = typename std::vector<typename
Network::Edge>::const_iterator, typename ScoreRndIt = typename std::vector<double>::iterator,
typename EdgeRndOutIt = typename std::vector<typename Network::Edge>::iterator>
using LinkPred::UNEDPredictor< Network, EdgeRndIt, ScoreRndIt, EdgeRndOutIt >::NodeID = typename
ULPredictor<Network, EdgeRndIt,ScoreRndIt, EdgeRndOutIt>::NodeID
```

The node IDs type.

9.130.3 Constructor & Destructor Documentation

9.130.3.1 UNEDPredictor() [1/3]

```
template<typename Network = UNetwork<>, typename EdgeRndIt = typename std::vector<typename
Network::Edge>::const_iterator, typename ScoreRndIt = typename std::vector<double>::iterator,
typename EdgeRndOutIt = typename std::vector<typename Network::Edge>::iterator>
LinkPred::UNEDPredictor< Network, EdgeRndIt, ScoreRndIt, EdgeRndOutIt >::UNEDPredictor (
            std::shared_ptr< Network const > net )  [inline]
```

Parameters

net	The network.

9.130.3.2 UNEDPredictor() [2/3]

```
template<typename Network = UNetwork<>, typename EdgeRndIt = typename std::vector<typename
Network::Edge>::const_iterator, typename ScoreRndIt = typename std::vector<double>::iterator,
typename EdgeRndOutIt = typename std::vector<typename Network::Edge>::iterator>
LinkPred::UNEDPredictor< Network, EdgeRndIt, ScoreRndIt, EdgeRndOutIt >::UNEDPredictor (
            UNEDPredictor< Network, EdgeRndIt, ScoreRndIt, EdgeRndOutIt > const & that )
[default]
```

Copy constructor.

Parameters

that	The object to copy.

9.130.3.3 UNEDPredictor() [3/3]

```
template<typename Network = UNetwork<>, typename EdgeRndIt = typename std::vector<typename
Network::Edge>::const_iterator, typename ScoreRndIt = typename std::vector<double>::iterator,
```

```
typename EdgeRndOutIt = typename std::vector<typename Network::Edge>::iterator>
LinkPred::UNEDPredictor< Network, EdgeRndIt, ScoreRndIt, EdgeRndOutIt >::UNEDPredictor (
            UNEDPredictor< Network, EdgeRndIt, ScoreRndIt, EdgeRndOutIt > && that )  [default]
```

Move constructor.

Parameters

that	The object to move.

9.130.3.4 ∼UNEDPredictor()

```
template<typename Network = UNetwork<>, typename EdgeRndIt = typename std::vector<typename
Network::Edge>::const_iterator, typename ScoreRndIt = typename std::vector<double>::iterator,
typename EdgeRndOutIt = typename std::vector<typename Network::Edge>::iterator>
virtual LinkPred::UNEDPredictor< Network, EdgeRndIt, ScoreRndIt, EdgeRndOutIt >::~UNEDPredictor
( )  [virtual], [default]
```

Destructor.

9.130.4 Member Function Documentation

9.130.4.1 init()

```
template<typename Network = UNetwork<>, typename EdgeRndIt = typename std::vector<typename
Network::Edge>::const_iterator, typename ScoreRndIt = typename std::vector<double>::iterator,
typename EdgeRndOutIt = typename std::vector<typename Network::Edge>::iterator>
virtual void LinkPred::UNEDPredictor< Network, EdgeRndIt, ScoreRndIt, EdgeRndOutIt >::init ( )
[inline], [virtual]
```

Initialize the predictor.

Implements LinkPred::ULPredictor< UNetwork<>, typename std::vector< typename UNetwork<> ::Edge >::const_iterator, typena

9.130.4.2 learn()

```
template<typename Network = UNetwork<>, typename EdgeRndIt = typename std::vector<typename
Network::Edge>::const_iterator, typename ScoreRndIt = typename std::vector<double>::iterator,
typename EdgeRndOutIt = typename std::vector<typename Network::Edge>::iterator>
virtual void LinkPred::UNEDPredictor< Network, EdgeRndIt, ScoreRndIt, EdgeRndOutIt >::learn (
)  [inline], [virtual]
```

Learn.

Implements LinkPred::ULPredictor< UNetwork<>, typename std::vector< typename UNetwork<> ::Edge >::const_iterator, typena

9.130.4.3 operator=() [1/2]

```
template<typename Network = UNetwork<>, typename EdgeRndIt = typename std::vector<typename
Network::Edge>::const_iterator, typename ScoreRndIt = typename std::vector<double>::iterator,
typename EdgeRndOutIt = typename std::vector<typename Network::Edge>::iterator>
UNEDPredictor& LinkPred::UNEDPredictor< Network, EdgeRndIt, ScoreRndIt, EdgeRndOutIt >::operator=
(
            UNEDPredictor< Network, EdgeRndIt, ScoreRndIt, EdgeRndOutIt > && that )  [default]
```

Move assignment operator.

Parameters

that	The object to move.

9.130.4.4 operator=() [2/2]

```
template<typename Network = UNetwork<>, typename EdgeRndIt = typename std::vector<typename
Network::Edge>::const_iterator, typename ScoreRndIt = typename std::vector<double>::iterator,
typename EdgeRndOutIt = typename std::vector<typename Network::Edge>::iterator>
UNEDPredictor& LinkPred::UNEDPredictor< Network, EdgeRndIt, ScoreRndIt, EdgeRndOutIt >::operator=
(
            UNEDPredictor< Network, EdgeRndIt, ScoreRndIt, EdgeRndOutIt > const & that )
[default]
```

Copy assignment operator.

Parameters

that	The object to copy.

9.130.4.5 predict()

```
template<typename Network = UNetwork<>, typename EdgeRndIt = typename std::vector<typename
Network::Edge>::const_iterator, typename ScoreRndIt = typename std::vector<double>::iterator,
typename EdgeRndOutIt = typename std::vector<typename Network::Edge>::iterator>
virtual void LinkPred::UNEDPredictor< Network, EdgeRndIt, ScoreRndIt, EdgeRndOutIt >::predict
(
            EdgeRndIt begin,
            EdgeRndIt end,
            ScoreRndIt scores )  [virtual]
```

Predict the links.

Parameters

begin	Beginning of the links to be predicted.
end	end of the links to be predicted.
scores	Beginning of scores.

Reimplemented from LinkPred::ULPredictor< UNetwork<>, typename std::vector< typename UNetwork<> ::Edge >::const_iterat

9.130.4.6 score()

```
template<typename Network = UNetwork<>, typename EdgeRndIt = typename std::vector<typename
Network::Edge>::const_iterator, typename ScoreRndIt = typename std::vector<double>::iterator,
typename EdgeRndOutIt = typename std::vector<typename Network::Edge>::iterator>
virtual double LinkPred::UNEDPredictor< Network, EdgeRndIt, ScoreRndIt, EdgeRndOutIt >::score
(
            Edge const & e )  [virtual]
```

Compute the score of a single edge.

Parameters

e	The edge.

Returns

The score of e.

9.130.4.7 top()

```
template<typename Network = UNetwork<>, typename EdgeRndIt = typename std::vector<typename
Network::Edge>::const_iterator, typename ScoreRndIt = typename std::vector<double>::iterator,
typename EdgeRndOutIt = typename std::vector<typename Network::Edge>::iterator>
virtual std::size_t LinkPred::UNEDPredictor< Network, EdgeRndIt, ScoreRndIt, EdgeRndOutIt >↩
::top (
            std::size_t k,
            EdgeRndOutIt eit,
            ScoreRndIt sit )  [virtual]
```

Finds the k negative edges with the top score. Ties are broken randmly.

Parameters

k	The number of edges to find.
eit	An output iterator where the edges are written.
sit	An output iterator where the scores are written. The scores are written in the same order as the edges.

Returns

The number of negative edges inserted. It is the minimum between k and the number of negative edges in the network.

Reimplemented from LinkPred::ULPredictor< UNetwork<>, typename std::vector< typename UNetwork<> ::Edge >::const_iterat

The documentation for this class was generated from the following file:

- include/linkpred/predictors/undirected/unedpredictor.hpp

9.131 LinkPred::UNetwork< LabelT, NodeIDT, EdgeT > Class Template Reference

This class represents an undirected network in the sense of graph theory.

```
#include <unetwork.hpp>
```

Classes

- class EdgeMap

  An edge map.
- class NodeDegIt

  Node-degree iterator. This class can be used to iterate over pairs of node IDs and degrees.
- class NodeMap

  A node map.
- class NodeSMap

  A sparse node map.
- class NonEdgeIt

  Nonedges iterator.
- class RndEdgeIt

  Randomized edges iterator.
- class RndNodeIt

  Randomized Nodes iterator.
- class RndNonEdgeIt

  Randomized nonedges iterator.

Public Types

- using Label = LabelT
- using NodeID = NodeIDT
- using Edge = EdgeT
- using LabelIt = typename Bhmap< Label, NodeID >::k_const_iterator
- using NodeIt = typename Bhmap< Label, NodeID >::p_const_iterator
- using EdgeIt = typename std::vector< Edge >::const_iterator
- template<typename ValueT >
  using NodeMapSP = std::shared_ptr< NodeMap< ValueT > >
- template<typename ValueT >
  using NodeSMapSP = std::shared_ptr< NodeSMap< ValueT > >
- template<typename ValueT >
  using EdgeMapSP = std::shared_ptr< EdgeMap< ValueT > >

Public Member Functions

- UNetwork ()=default
- UNetwork (std::vector< std::pair< Label, Label >> const &edges)
- UNetwork (UNetwork const &that)=default
- UNetwork & operator= (UNetwork const &that)=default
- UNetwork (UNetwork &&that)=default
- UNetwork & operator= (UNetwork &&that)=default
- std::pair< NodeID, bool > addNode (Label const &nodeId)
- NodeID getID (Label const &label) const
- Label getLabel (NodeID const &iid) const
- LabelIt findLabel (Label const &label) const
- NodeIt findNode (NodeID const &iid) const
- void addEdge (NodeID const &i, NodeID const &j)
- EdgeIt neighbBegin (NodeID const &iid) const
- std::size_t coupleOrd (Edge const &e) const
- std::size_t coupleAtOrd (std::size_t ord) const
- EdgeIt neighbEnd (NodeID const &iid) const
- std::size_t getDeg (NodeID const &iid) const
- bool isEdge (NodeID const &ii, NodeID const &ij) const
- bool isEdge (Edge const &edge) const
- std::size_t getNbNodes () const
- std::size_t getNbCouples () const
- std::size_t getNbEdges () const
- std::size_t getNbNonEdges () const
- double getAvgDeg () const
- std::size_t getMaxDeg () const
- std::size_t getMinDeg () const
- void assemble ()
- std::pair< std::vector< std::size_t >, std::vector< std::size_t > > getCSR () const
- void shuffle (long int seed)
- LabelIt labelsBegin () const
- LabelIt labelsEnd () const
- NodeIt nodesBegin () const
- NodeIt nodesEnd () const
- NodeDegIt nodesDegBegin () const
- NodeDegIt nodesDegEnd () const
- EdgeIt edgesBegin () const
- EdgeIt edgesEnd () const
- NonEdgeIt nonEdgesBegin () const
- NonEdgeIt nonEdgesEnd () const
- RndNodeIt rndNodesBegin (double ratio, long int seed) const
- RndNodeIt rndNodesEnd () const
- RndNonEdgeIt rndNonEdgesBegin (double ratio, long int seed) const
- RndNonEdgeIt rndNonEdgesEnd () const
- RndEdgeIt rndEdgesBegin (double ratio, long int seed) const
- RndEdgeIt rndEdgesEnd () const
- void getDegStat (std::size_t &minDeg, std::size_t &maxDeg, double &avgDeg) const
- template<typename ValueT >
  NodeMap< ValueT > createNodeMap () const
- template<typename ValueT >
  NodeMapSP< ValueT > createNodeMapSP () const
- template<typename ValueT >
  NodeSMap< ValueT > createNodeSMap (ValueT const &defVal) const

- template<typename ValueT >
  NodeSMapSP< ValueT > createNodeSMapSP (ValueT const &defVal) const
- template<typename ValueT >
  EdgeMap< ValueT > createEdgeMap () const
- template<typename ValueT >
  EdgeMapSP< ValueT > createEdgeMapSP () const
- std::size_t getNbCommonNeighbors (NodeID const &i, NodeID const &j) const
- template<typename InserterIt >
  void getCommonNeighbors (NodeID const &i, NodeID const &j, InserterIt inserter) const
- std::set< NodeID > getCommonNeighbors (NodeID const &i, NodeID const &j) const
- double getCC (NodeID const &i) const
- double getCC () const
- std::size_t getNbPaths (NodeID const &srcId, NodeID const &endId, std::size_t length) const
- std::size_t getNbInEdges (std::set< NodeID > const &ns) const
- std::shared_ptr< std::vector< Edge > > readEdges (std::string fileName) const
- void write (std::string fileName) const
- void print () const
- template<typename ForwardIterator >
  void printEdges (ForwardIterator edgesBegin, ForwardIterator edgesEnd) const
- virtual ~UNetwork ()=default

Static Public Member Functions

- static Edge makeEdge (NodeID const &i, NodeID const &j)
- static Edge reverseEdge (Edge const &e)
- static const NodeID start (Edge const &edge)
- static const NodeID end (Edge const &edge)
- static bool compareEdgeEnd (Edge const &e1, Edge const &e2)
- static std::shared_ptr< UNetwork< Label, NodeID, Edge > > read (std::string fileName, bool ignoreRepetitions=false, bool ignoreLoops=false)
- static std::shared_ptr< UNetwork< unsigned int, NodeID, Edge > > generateERN (std::size_t nbNodes, double pr, long int seed)
- static std::shared_ptr< UNetwork< unsigned int, NodeID, Edge > > generateRNC (std::size_t nbNodes, double pr, long int seed)
- static std::shared_ptr< UNetwork< unsigned int, NodeID, Edge > > generateREG (std::size_t h, std::size_t w)

9.131.1 Detailed Description

**template<typename LabelT = std::string, typename NodeIDT = unsigned int, typename EdgeT = unsigned long long int>
class LinkPred::UNetwork< LabelT, NodeIDT, EdgeT >**

This class represents an undirected network in the sense of graph theory.

Template Parameters

LabelT	Type of external labels.
NodeIDT	Type of internal node IDs. This must be an unsigned integral type.
EdgeT	Type of edges. This must be an unsigned integral type having at least double the size of `NodeID` UNetwork.

9.131.2 Member Typedef Documentation

9.131.2.1 Edge

```
template<typename LabelT = std::string, typename NodeIDT = unsigned int, typename EdgeT =
unsigned long long int>
using LinkPred::UNetwork< LabelT, NodeIDT, EdgeT >::Edge = EdgeT
```

Internal edge type.

9.131.2.2 EdgeIt

```
template<typename LabelT = std::string, typename NodeIDT = unsigned int, typename EdgeT =
unsigned long long int>
using LinkPred::UNetwork< LabelT, NodeIDT, EdgeT >::EdgeIt = typename std::vector<Edge>↩
::const_iterator
```

Edge iterator.

9.131.2.3 EdgeMapSP

```
template<typename LabelT = std::string, typename NodeIDT = unsigned int, typename EdgeT =
unsigned long long int>
template<typename ValueT >
using LinkPred::UNetwork< LabelT, NodeIDT, EdgeT >::EdgeMapSP = std::shared_ptr<EdgeMap<ValueT>
>
```

Shared pointer to an edge map.

9.131.2.4 Label

```
template<typename LabelT = std::string, typename NodeIDT = unsigned int, typename EdgeT =
unsigned long long int>
using LinkPred::UNetwork< LabelT, NodeIDT, EdgeT >::Label = LabelT
```

External label type.

9.131.2.5 LabelIt

```
template<typename LabelT = std::string, typename NodeIDT = unsigned int, typename EdgeT =
unsigned long long int>
using LinkPred::UNetwork< LabelT, NodeIDT, EdgeT >::LabelIt = typename Bhmap<Label, NodeID>↩
::k_const_iterator
```

External node iterator that offers the mapping to internal IDs.

9.131.2.6 NodeID

```
template<typename LabelT = std::string, typename NodeIDT = unsigned int, typename EdgeT =
unsigned long long int>
using LinkPred::UNetwork< LabelT, NodeIDT, EdgeT >::NodeID = NodeIDT
```

Internal node ID type.

9.131.2.7 NodeIt

```
template<typename LabelT = std::string, typename NodeIDT = unsigned int, typename EdgeT =
unsigned long long int>
using LinkPred::UNetwork< LabelT, NodeIDT, EdgeT >::NodeIt = typename Bhmap<Label, NodeID>↩
::p_const_iterator
```

Internal node iterator (random access iterator).

9.131.2.8 NodeMapSP

```
template<typename LabelT = std::string, typename NodeIDT = unsigned int, typename EdgeT =
unsigned long long int>
template<typename ValueT >
using LinkPred::UNetwork< LabelT, NodeIDT, EdgeT >::NodeMapSP = std::shared_ptr<NodeMap<ValueT>
>
```

Shared pointer to a node map.

9.131.2.9 NodeSMapSP

```
template<typename LabelT = std::string, typename NodeIDT = unsigned int, typename EdgeT =
unsigned long long int>
template<typename ValueT >
using LinkPred::UNetwork< LabelT, NodeIDT, EdgeT >::NodeSMapSP = std::shared_ptr<NodeSMap<ValueT>
>
```

Shared pointer to a node map.

9.131.3 Constructor & Destructor Documentation

9.131.3.1 UNetwork() [1/4]

```
template<typename LabelT = std::string, typename NodeIDT = unsigned int, typename EdgeT =
unsigned long long int>
LinkPred::UNetwork< LabelT, NodeIDT, EdgeT >::UNetwork ( )  [default]
```

Default constructor.

9.131.3.2 UNetwork() [2/4]

```
template<typename LabelT = std::string, typename NodeIDT = unsigned int, typename EdgeT =
unsigned long long int>
LinkPred::UNetwork< LabelT, NodeIDT, EdgeT >::UNetwork (
            std::vector< std::pair< Label, Label >> const & edges )
```

Build the network form a list of edges. The network is assembled within this constructor.

Parameters

edges	List of edges.

9.131.3.3 UNetwork() [3/4]

```
template<typename LabelT = std::string, typename NodeIDT = unsigned int, typename EdgeT =
unsigned long long int>
LinkPred::UNetwork< LabelT, NodeIDT, EdgeT >::UNetwork (
            UNetwork< LabelT, NodeIDT, EdgeT > const & that )  [default]
```

Copy constructor.

Parameters

that	The object to copy.

9.131.3.4 UNetwork() [4/4]

```
template<typename LabelT = std::string, typename NodeIDT = unsigned int, typename EdgeT =
unsigned long long int>
LinkPred::UNetwork< LabelT, NodeIDT, EdgeT >::UNetwork (
            UNetwork< LabelT, NodeIDT, EdgeT > && that )  [default]
```

Move constructor.

Parameters

that	The object to move.

9.131.3.5 ∼UNetwork()

```
template<typename LabelT = std::string, typename NodeIDT = unsigned int, typename EdgeT =
unsigned long long int>
virtual LinkPred::UNetwork< LabelT, NodeIDT, EdgeT >::~UNetwork ( )  [virtual], [default]
```

Destructor.

9.131.4 Member Function Documentation

9.131.4.1 addEdge()

```
template<typename LabelT = std::string, typename NodeIDT = unsigned int, typename EdgeT =
unsigned long long int>
void LinkPred::UNetwork< LabelT, NodeIDT, EdgeT >::addEdge (
            NodeID const & i,
            NodeID const & j )
```

Add an edge.

Parameters

i	The starting node.
j	The end node.

9.131.4.2 addNode()

```
template<typename LabelT = std::string, typename NodeIDT = unsigned int, typename EdgeT =
unsigned long long int>
std::pair<NodeID, bool> LinkPred::UNetwork< LabelT, NodeIDT, EdgeT >::addNode (
            Label const & nodeId )
```

Add a node.

Parameters

node←↩ Id	The ID of the node.

Returns

An std::pair, where first is the internal ID, and second is a boolean which is true if the node is actually added.

9.131.4.3 assemble()

```
template<typename LabelT = std::string, typename NodeIDT = unsigned int, typename EdgeT =
unsigned long long int>
void LinkPred::UNetwork< LabelT, NodeIDT, EdgeT >::assemble ( )
```

Assemble the network. No changes to the network are allowed after calling this method.

9.131.4.4 compareEdgeEnd()

```
template<typename LabelT = std::string, typename NodeIDT = unsigned int, typename EdgeT =
unsigned long long int>
static bool LinkPred::UNetwork< LabelT, NodeIDT, EdgeT >::compareEdgeEnd (
            Edge const & e1,
            Edge const & e2 ) [inline], [static]
```

Compare edge ends.

Parameters

e1	First edge.
e2	Second edge.

Returns

True if the end of e1 is smaller than that of e2 (comparison is based on node IDs).

9.131.4.5 coupleAtOrd()

```
template<typename LabelT = std::string, typename NodeIDT = unsigned int, typename EdgeT =
unsigned long long int>
std::size_t LinkPred::UNetwork< LabelT, NodeIDT, EdgeT >::coupleAtOrd (
            std::size_t ord ) const [inline]
```

Parameters

ord	The order of an edge.

Returns

The edge given its order.

9.131.4.6 coupleOrd()

```
template<typename LabelT = std::string, typename NodeIDT = unsigned int, typename EdgeT =
unsigned long long int>
std::size_t LinkPred::UNetwork< LabelT, NodeIDT, EdgeT >::coupleOrd (
            Edge const & e ) const [inline]
```

Parameters

e	An edge.

Returns

The order of the edge

9.131.4.7 createEdgeMap()

```
template<typename LabelT = std::string, typename NodeIDT = unsigned int, typename EdgeT =
unsigned long long int>
```

```
template<typename ValueT >
EdgeMap<ValueT> LinkPred::UNetwork< LabelT, NodeIDT, EdgeT >::createEdgeMap ( ) const  [inline]
```

Template Parameters

ValueT	Value type.

Returns

An edge map.

9.131.4.8 createEdgeMapSP()

```
template<typename LabelT = std::string, typename NodeIDT = unsigned int, typename EdgeT =
unsigned long long int>
template<typename ValueT >
EdgeMapSP<ValueT> LinkPred::UNetwork< LabelT, NodeIDT, EdgeT >::createEdgeMapSP ( ) const
[inline]
```

Template Parameters

ValueT	Value type.

Returns

A pointer to an edge map.

9.131.4.9 createNodeMap()

```
template<typename LabelT = std::string, typename NodeIDT = unsigned int, typename EdgeT =
unsigned long long int>
template<typename ValueT >
NodeMap<ValueT> LinkPred::UNetwork< LabelT, NodeIDT, EdgeT >::createNodeMap ( ) const  [inline]
```

Template Parameters

ValueT	Value type.

Returns

A node map.

9.131.4.10 createNodeMapSP()

```
template<typename LabelT = std::string, typename NodeIDT = unsigned int, typename EdgeT =
unsigned long long int>
template<typename ValueT >
NodeMapSP<ValueT> LinkPred::UNetwork< LabelT, NodeIDT, EdgeT >::createNodeMapSP ( ) const
[inline]
```

Template Parameters

ValueT	Value type.

Returns

A pointer to a node map.

9.131.4.11 createNodeSMap()

```
template<typename LabelT = std::string, typename NodeIDT = unsigned int, typename EdgeT =
unsigned long long int>
template<typename ValueT >
NodeSMap<ValueT> LinkPred::UNetwork< LabelT, NodeIDT, EdgeT >::createNodeSMap (
            ValueT const & defVal ) const  [inline]
```

Template Parameters

ValueT	Value type.

Returns

A sparse node map.

9.131.4.12 createNodeSMapSP()

```
template<typename LabelT = std::string, typename NodeIDT = unsigned int, typename EdgeT =
unsigned long long int>
template<typename ValueT >
NodeSMapSP<ValueT> LinkPred::UNetwork< LabelT, NodeIDT, EdgeT >::createNodeSMapSP (
            ValueT const & defVal ) const  [inline]
```

Template Parameters

ValueT	Value type.

Returns

A pointer to a sparse node map.

9.131.4.13 edgesBegin()

```
template<typename LabelT = std::string, typename NodeIDT = unsigned int, typename EdgeT =
unsigned long long int>
EdgeIt LinkPred::UNetwork< LabelT, NodeIDT, EdgeT >::edgesBegin ( ) const [inline]
```

Returns

A read-only (constant) iterator that points to the first edge (with internal ID).

9.131.4.14 edgesEnd()

```
template<typename LabelT = std::string, typename NodeIDT = unsigned int, typename EdgeT =
unsigned long long int>
EdgeIt LinkPred::UNetwork< LabelT, NodeIDT, EdgeT >::edgesEnd ( ) const [inline]
```

Returns

A read-only (constant) iterator that points one past the last edge (with internal ID).

9.131.4.15 end()

```
template<typename LabelT = std::string, typename NodeIDT = unsigned int, typename EdgeT =
unsigned long long int>
static const NodeID LinkPred::UNetwork< LabelT, NodeIDT, EdgeT >::end (
            Edge const & edge ) [inline], [static]
```

Parameters

edge	An edge.

Returns

The end node of edge.

9.131.4.16 findLabel()

```
template<typename LabelT = std::string, typename NodeIDT = unsigned int, typename EdgeT =
unsigned long long int>
LabelIt LinkPred::UNetwork< LabelT, NodeIDT, EdgeT >::findLabel (
            Label const & label ) const  [inline]
```

Parameters

label	An external node ID.

Returns

Iterator to the external node ID.

9.131.4.17 findNode()

```
template<typename LabelT = std::string, typename NodeIDT = unsigned int, typename EdgeT =
unsigned long long int>
NodeIt LinkPred::UNetwork< LabelT, NodeIDT, EdgeT >::findNode (
            NodeID const & iid ) const  [inline]
```

Parameters

iid	An internal node ID.

Returns

Iterator to the internal node ID.

9.131.4.18 generateERN()

```
template<typename LabelT = std::string, typename NodeIDT = unsigned int, typename EdgeT =
unsigned long long int>
static std::shared_ptr<UNetwork<unsigned int, NodeID, Edge> > LinkPred::UNetwork< LabelT,
NodeIDT, EdgeT >::generateERN (
            std::size_t nbNodes,
            double pr,
            long int seed )  [static]
```

Generate an Erdos-Renyi (random) network.

Parameters

nbNodes	The number of nodes.
pr	The probability of connecting any two nodes.
seed	Number generator seed.

9.131.4.19 generateREG()

```
template<typename LabelT = std::string, typename NodeIDT = unsigned int, typename EdgeT =
unsigned long long int>
static std::shared_ptr<UNetwork<unsigned int, NodeID, Edge> > LinkPred::UNetwork< LabelT,
NodeIDT, EdgeT >::generateREG (
            std::size_t h,
            std::size_t w ) [static]
```

Generate a regular two-dimensional grid of size $h \times w$, where h is the height of grid and w its width. The nodes are connected to their four neighbors right, left, up and down, except of course for boundary nodes. The nodes are assigned coordinates in $[0, 1]^2$ using equi-distant spacing in each dimension. The step size in the first dimension is $1/(h - 1)$, in the second dimension, it is $1/(w - 1)$.

Parameters

h	Height of the grid.
w	Width of the grid.

9.131.4.20 generateRNC()

```
template<typename LabelT = std::string, typename NodeIDT = unsigned int, typename EdgeT =
unsigned long long int>
static std::shared_ptr<UNetwork<unsigned int, NodeID, Edge> > LinkPred::UNetwork< LabelT,
NodeIDT, EdgeT >::generateRNC (
            std::size_t nbNodes,
            double pr,
            long int seed ) [static]
```

Generate a random connected network.

Parameters

nbNodes	The number of nodes.
pr	The probability of connecting any two nodes.
seed	Number generator seed.

9.131.4.21 getAvgDeg()

```
template<typename LabelT = std::string, typename NodeIDT = unsigned int, typename EdgeT =
unsigned long long int>
double LinkPred::UNetwork< LabelT, NodeIDT, EdgeT >::getAvgDeg ( ) const [inline]
```

Returns

Average degree. Can only be called after the network is assembled.

9.131.4.22 getCC() [1/2]

```
template<typename LabelT = std::string, typename NodeIDT = unsigned int, typename EdgeT =
unsigned long long int>
double LinkPred::UNetwork< LabelT, NodeIDT, EdgeT >::getCC ( ) const  [inline]
```

Returns

The average clustering coefficient of the network.

9.131.4.23 getCC() [2/2]

```
template<typename LabelT = std::string, typename NodeIDT = unsigned int, typename EdgeT =
unsigned long long int>
double LinkPred::UNetwork< LabelT, NodeIDT, EdgeT >::getCC (
            NodeID const & i ) const  [inline]
```

Parameters

i	A node ID.

Returns

The clustering coefficient of i.

9.131.4.24 getCommonNeighbors() [1/2]

```
template<typename LabelT = std::string, typename NodeIDT = unsigned int, typename EdgeT =
unsigned long long int>
std::set<NodeID> LinkPred::UNetwork< LabelT, NodeIDT, EdgeT >::getCommonNeighbors (
            NodeID const & i,
            NodeID const & j ) const  [inline]
```

Parameters

i	A node ID.
j	A node ID.

Returns

The set of common neighbors of i and j as an std::set.

9.131.4.25 getCommonNeighbors() [2/2]

```
template<typename LabelT = std::string, typename NodeIDT = unsigned int, typename EdgeT =
unsigned long long int>
template<typename InserterIt >
void LinkPred::UNetwork< LabelT, NodeIDT, EdgeT >::getCommonNeighbors (
            NodeID const & i,
            NodeID const & j,
            InserterIt inserter ) const  [inline]
```

Parameters

i	A node ID.
j	A node ID.
inserter	An inserter iterator to insert the common neighbors of i and j.

9.131.4.26 getCSR()

```
template<typename LabelT = std::string, typename NodeIDT = unsigned int, typename EdgeT =
unsigned long long int>
std::pair<std::vector<std::size_t>, std::vector<std::size_t> > LinkPred::UNetwork< LabelT,
NodeIDT, EdgeT >::getCSR ( ) const  [inline]
```

Returns

The CSR representation of the network. The first element contains the row index, whereas the second contains the column index.

9.131.4.27 getDeg()

```
template<typename LabelT = std::string, typename NodeIDT = unsigned int, typename EdgeT =
unsigned long long int>
std::size_t LinkPred::UNetwork< LabelT, NodeIDT, EdgeT >::getDeg (
            NodeID const & iid ) const  [inline]
```

Parameters

iid	The node internal ID.

Returns

The degree of node iid.

9.131.4.28 getDegStat()

```
template<typename LabelT = std::string, typename NodeIDT = unsigned int, typename EdgeT =
unsigned long long int>
void LinkPred::UNetwork< LabelT, NodeIDT, EdgeT >::getDegStat (
            std::size_t & minDeg,
            std::size_t & maxDeg,
            double & avgDeg ) const [inline]
```

Compute some degree statistics.

Parameters

minDeg	(output parameter) minimum degree.
maxDeg	(output parameter) maximum degree.
avgDeg	(output parameter) average degree.

9.131.4.29 getID()

```
template<typename LabelT = std::string, typename NodeIDT = unsigned int, typename EdgeT =
unsigned long long int>
NodeID LinkPred::UNetwork< LabelT, NodeIDT, EdgeT >::getID (
            Label const & label ) const [inline]
```

Translates from external label to internal IDs. This method is O(log n), where n is the number of nodes.

Parameters

label	An external node label.

Returns

The internal ID of label;

9.131.4.30 getLabel()

```
template<typename LabelT = std::string, typename NodeIDT = unsigned int, typename EdgeT =
unsigned long long int>
Label LinkPred::UNetwork< LabelT, NodeIDT, EdgeT >::getLabel (
            NodeID const & iid ) const [inline]
```

Parameters

iid	An internal node ID.

Returns

The external label of the node iid.

9.131.4.31 getMaxDeg()

```
template<typename LabelT = std::string, typename NodeIDT = unsigned int, typename EdgeT =
unsigned long long int>
std::size_t LinkPred::UNetwork< LabelT, NodeIDT, EdgeT >::getMaxDeg ( ) const  [inline]
```

Returns

Maximum degree. Can only be called after the network is assembled.

9.131.4.32 getMinDeg()

```
template<typename LabelT = std::string, typename NodeIDT = unsigned int, typename EdgeT =
unsigned long long int>
std::size_t LinkPred::UNetwork< LabelT, NodeIDT, EdgeT >::getMinDeg ( ) const  [inline]
```

Returns

Minimum degree. Can only be called after the network is assembled.

9.131.4.33 getNbCommonNeighbors()

```
template<typename LabelT = std::string, typename NodeIDT = unsigned int, typename EdgeT =
unsigned long long int>
std::size_t LinkPred::UNetwork< LabelT, NodeIDT, EdgeT >::getNbCommonNeighbors (
            NodeID const & i,
            NodeID const & j ) const  [inline]
```

Parameters

i	A node ID.
j	A node ID.

Returns

The number of common neighbors between i and j.

9.131.4.34 getNbCouples()

```
template<typename LabelT = std::string, typename NodeIDT = unsigned int, typename EdgeT =
unsigned long long int>
std::size_t LinkPred::UNetwork< LabelT, NodeIDT, EdgeT >::getNbCouples ( ) const [inline]
```

Returns

The number of couples in the network.

9.131.4.35 getNbEdges()

```
template<typename LabelT = std::string, typename NodeIDT = unsigned int, typename EdgeT =
unsigned long long int>
std::size_t LinkPred::UNetwork< LabelT, NodeIDT, EdgeT >::getNbEdges ( ) const [inline]
```

Returns

The number of edges in the network.

9.131.4.36 getNbInEdges()

```
template<typename LabelT = std::string, typename NodeIDT = unsigned int, typename EdgeT =
unsigned long long int>
std::size_t LinkPred::UNetwork< LabelT, NodeIDT, EdgeT >::getNbInEdges (
            std::set< NodeID > const & ns ) const
```

Parameters

ns	A set of nodes.

Returns

The number of edges connecting nodes in the set ns.

9.131.4.37 getNbNodes()

```
template<typename LabelT = std::string, typename NodeIDT = unsigned int, typename EdgeT =
unsigned long long int>
std::size_t LinkPred::UNetwork< LabelT, NodeIDT, EdgeT >::getNbNodes ( ) const  [inline]
```

Returns

The number of nodes in the network.

9.131.4.38 getNbNonEdges()

```
template<typename LabelT = std::string, typename NodeIDT = unsigned int, typename EdgeT =
unsigned long long int>
std::size_t LinkPred::UNetwork< LabelT, NodeIDT, EdgeT >::getNbNonEdges ( ) const  [inline]
```

Returns

The number of non-edges in the network.

9.131.4.39 getNbPaths()

```
template<typename LabelT = std::string, typename NodeIDT = unsigned int, typename EdgeT =
unsigned long long int>
std::size_t LinkPred::UNetwork< LabelT, NodeIDT, EdgeT >::getNbPaths (
            NodeID const & srcId,
            NodeID const & endId,
            std::size_t length ) const
```

Parameters

srcId	The source node ID.
endId	The end node ID.
length	Specified length.

Returns

The number of paths of length exactly length joining srcNode and endNode.

9.131.4.40 isEdge() [1/2]

```
template<typename LabelT = std::string, typename NodeIDT = unsigned int, typename EdgeT =
unsigned long long int>
```

```
bool LinkPred::UNetwork< LabelT, NodeIDT, EdgeT >::isEdge (
            Edge const & edge ) const
```

Check if an edge exists in O(k_max).

Parameters

edge	An edge.

Returns

True if edge exists in the network, false otherwise.

9.131.4.41 isEdge() [2/2]

```
template<typename LabelT = std::string, typename NodeIDT = unsigned int, typename EdgeT =
unsigned long long int>
bool LinkPred::UNetwork< LabelT, NodeIDT, EdgeT >::isEdge (
            NodeID const & ii,
            NodeID const & ij ) const  [inline]
```

Check if an edge exists in O(k_max).

Parameters

ii	An internal node ID.
ij	An internal node ID.

Returns

true if the edge (ii, ij) exists, false otherwise.

9.131.4.42 labelsBegin()

```
template<typename LabelT = std::string, typename NodeIDT = unsigned int, typename EdgeT =
unsigned long long int>
LabelIt LinkPred::UNetwork< LabelT, NodeIDT, EdgeT >::labelsBegin ( ) const  [inline]
```

Returns

A read-only (constant) external node ID iterator that points to the first node.

9.131.4.43 labelsEnd()

```
template<typename LabelT = std::string, typename NodeIDT = unsigned int, typename EdgeT =
unsigned long long int>
LabelIt LinkPred::UNetwork< LabelT, NodeIDT, EdgeT >::labelsEnd ( ) const  [inline]
```

Returns

A read-only (constant) external node ID iterator that points one past the last node.

9.131.4.44 makeEdge()

```
template<typename LabelT = std::string, typename NodeIDT = unsigned int, typename EdgeT =
unsigned long long int>
static Edge LinkPred::UNetwork< LabelT, NodeIDT, EdgeT >::makeEdge (
            NodeID const & i,
            NodeID const & j )  [inline], [static]
```

Make an edge in internal representation out of two nodes' internal IDs.

Parameters

i	The starting node.
j	The end node.

Returns

The edge (i, j).

9.131.4.45 neighbBegin()

```
template<typename LabelT = std::string, typename NodeIDT = unsigned int, typename EdgeT =
unsigned long long int>
EdgeIt LinkPred::UNetwork< LabelT, NodeIDT, EdgeT >::neighbBegin (
            NodeID const & iid ) const  [inline]
```

Parameters

iid	An internal node ID.

Returns

An iterator to the first neighbor of iid.

9.131.4.46 neighbEnd()

```
template<typename LabelT = std::string, typename NodeIDT = unsigned int, typename EdgeT =
unsigned long long int>
EdgeIt LinkPred::UNetwork< LabelT, NodeIDT, EdgeT >::neighbEnd (
            NodeID const & iid ) const [inline]
```

Parameters

iid	An internal node ID.

Returns

An iterator to one past the last neighbor of iid.

9.131.4.47 nodesBegin()

```
template<typename LabelT = std::string, typename NodeIDT = unsigned int, typename EdgeT =
unsigned long long int>
NodeIt LinkPred::UNetwork< LabelT, NodeIDT, EdgeT >::nodesBegin ( ) const [inline]
```

Returns

A read-only (constant) external node ID iterator that points to the first node.

9.131.4.48 nodesDegBegin()

```
template<typename LabelT = std::string, typename NodeIDT = unsigned int, typename EdgeT =
unsigned long long int>
NodeDegIt LinkPred::UNetwork< LabelT, NodeIDT, EdgeT >::nodesDegBegin ( ) const [inline]
```

Returns

A read-only (constant) external node ID iterator that points to the first node-degree couple.

9.131.4.49 nodesDegEnd()

```
template<typename LabelT = std::string, typename NodeIDT = unsigned int, typename EdgeT =
unsigned long long int>
NodeDegIt LinkPred::UNetwork< LabelT, NodeIDT, EdgeT >::nodesDegEnd ( ) const [inline]
```

Returns

A read-only (constant) external node ID iterator that points one past the last node-degree couple.

9.131.4.50 nodesEnd()

```
template<typename LabelT = std::string, typename NodeIDT = unsigned int, typename EdgeT =
unsigned long long int>
NodeIt LinkPred::UNetwork< LabelT, NodeIDT, EdgeT >::nodesEnd ( ) const  [inline]
```

Returns

A read-only (constant) external node ID iterator that points one past the last node.

9.131.4.51 nonEdgesBegin()

```
template<typename LabelT = std::string, typename NodeIDT = unsigned int, typename EdgeT =
unsigned long long int>
NonEdgeIt LinkPred::UNetwork< LabelT, NodeIDT, EdgeT >::nonEdgesBegin ( ) const  [inline]
```

Returns

A read-only (constant) iterator that points to the first non-edge (with internal ID).

9.131.4.52 nonEdgesEnd()

```
template<typename LabelT = std::string, typename NodeIDT = unsigned int, typename EdgeT =
unsigned long long int>
NonEdgeIt LinkPred::UNetwork< LabelT, NodeIDT, EdgeT >::nonEdgesEnd ( ) const  [inline]
```

Returns

A read-only (constant) iterator that points one past the last non-edge (with internal ID).

9.131.4.53 operator=() [1/2]

```
template<typename LabelT = std::string, typename NodeIDT = unsigned int, typename EdgeT =
unsigned long long int>
UNetwork& LinkPred::UNetwork< LabelT, NodeIDT, EdgeT >::operator= (
            UNetwork< LabelT, NodeIDT, EdgeT > && that )  [default]
```

Move assignment operator.

Parameters

that	The object to move.

9.131.4.54 operator=() [2/2]

```
template<typename LabelT = std::string, typename NodeIDT = unsigned int, typename EdgeT =
unsigned long long int>
UNetwork& LinkPred::UNetwork< LabelT, NodeIDT, EdgeT >::operator= (
            UNetwork< LabelT, NodeIDT, EdgeT > const & that )  [default]
```

Copy assignment operator.

Parameters

that	The object to copy.

9.131.4.55 print()

```
template<typename LabelT = std::string, typename NodeIDT = unsigned int, typename EdgeT =
unsigned long long int>
void LinkPred::UNetwork< LabelT, NodeIDT, EdgeT >::print ( ) const
```

Print edges to std::cout.

9.131.4.56 printEdges()

```
template<typename LabelT = std::string, typename NodeIDT = unsigned int, typename EdgeT =
unsigned long long int>
template<typename ForwardIterator >
void LinkPred::UNetwork< LabelT, NodeIDT, EdgeT >::printEdges (
            ForwardIterator edgesBegin,
            ForwardIterator edgesEnd ) const  [inline]
```

Print edges to std::cout.

Parameters

edgesBegin	Iterator to the beginning of the edges.
edgesEnd	Iterator to one past the the end of the edges.

9.131.4.57 read()

```
template<typename LabelT = std::string, typename NodeIDT = unsigned int, typename EdgeT =
unsigned long long int>
```

```
static std::shared_ptr<UNetwork<Label, NodeID, Edge> > LinkPred::UNetwork< LabelT, NodeIDT,
EdgeT >::read (
            std::string fileName,
            bool ignoreRepetitions = false,
            bool ignoreLoops = false )  [static]
```

Read network from file.

Parameters

fileName	The file name.
ignoreRepetitions	Whether to ignore repeated edges.
ignoreLoops	Whether to ignore loops.

Returns

The read network.

9.131.4.58 readEdges()

```
template<typename LabelT = std::string, typename NodeIDT = unsigned int, typename EdgeT =
unsigned long long int>
std::shared_ptr<std::vector<Edge> > LinkPred::UNetwork< LabelT, NodeIDT, EdgeT >::readEdges
(
            std::string fileName ) const
```

Read couples from file.

Parameters

fileName	The file name.

Returns

The edges.

9.131.4.59 reverseEdge()

```
template<typename LabelT = std::string, typename NodeIDT = unsigned int, typename EdgeT =
unsigned long long int>
static Edge LinkPred::UNetwork< LabelT, NodeIDT, EdgeT >::reverseEdge (
            Edge const & e )  [inline], [static]
```

Parameters

e	An edge (i,j).

Returns

The edge (j, i).

9.131.4.60 rndEdgesBegin()

```
template<typename LabelT = std::string, typename NodeIDT = unsigned int, typename EdgeT =
unsigned long long int>
RndEdgeIt LinkPred::UNetwork< LabelT, NodeIDT, EdgeT >::rndEdgesBegin (
            double ratio,
            long int seed ) const  [inline]
```

Parameters

ratio	Ratio of edges that are selected.
seed	The random number gnerator's seed.

Returns

a read-only (constant) randomized iterator that points to the first edge (with internal ID).

9.131.4.61 rndEdgesEnd()

```
template<typename LabelT = std::string, typename NodeIDT = unsigned int, typename EdgeT =
unsigned long long int>
RndEdgeIt LinkPred::UNetwork< LabelT, NodeIDT, EdgeT >::rndEdgesEnd ( ) const  [inline]
```

Returns

A read-only (constant) randomized iterator that points one past the last edge (with internal ID).

9.131.4.62 rndNodesBegin()

```
template<typename LabelT = std::string, typename NodeIDT = unsigned int, typename EdgeT =
unsigned long long int>
RndNodeIt LinkPred::UNetwork< LabelT, NodeIDT, EdgeT >::rndNodesBegin (
            double ratio,
            long int seed ) const  [inline]
```

Parameters

ratio	Ratio of nodes that are selected.
seed	The random number gnerator's seed.

Returns

a read-only (constant) randomized iterator that points to the first node (with internal ID).

9.131.4.63 rndNodesEnd()

```
template<typename LabelT = std::string, typename NodeIDT = unsigned int, typename EdgeT =
unsigned long long int>
RndNodeIt LinkPred::UNetwork< LabelT, NodeIDT, EdgeT >::rndNodesEnd ( ) const [inline]
```

Returns

A read-only (constant) randomized iterator that points one past the last node (with internal ID).

9.131.4.64 rndNonEdgesBegin()

```
template<typename LabelT = std::string, typename NodeIDT = unsigned int, typename EdgeT =
unsigned long long int>
RndNonEdgeIt LinkPred::UNetwork< LabelT, NodeIDT, EdgeT >::rndNonEdgesBegin (
            double ratio,
            long int seed ) const [inline]
```

Parameters

ratio	Ratio of nonedges that are selected.
seed	The random number gnerator's seed.

Returns

a read-only (constant) randomized iterator that points to the first non-edge (with internal ID).

9.131.4.65 rndNonEdgesEnd()

```
template<typename LabelT = std::string, typename NodeIDT = unsigned int, typename EdgeT =
unsigned long long int>
RndNonEdgeIt LinkPred::UNetwork< LabelT, NodeIDT, EdgeT >::rndNonEdgesEnd ( ) const [inline]
```

Returns

A read-only (constant) randomized iterator that points one past the last non-edge (with internal ID).

9.131.4.66 shuffle()

```
template<typename LabelT = std::string, typename NodeIDT = unsigned int, typename EdgeT =
unsigned long long int>
void LinkPred::UNetwork< LabelT, NodeIDT, EdgeT >::shuffle (
            long int seed )
```

Shuffles the nodes' internal IDs. This is useful to eliminate bias in methods that depend on node/edge order. Upon calling this method, all iterators and maps associated with the network are invalidated.

Parameters

seed	Random number generator's seed.

9.131.4.67 start()

```
template<typename LabelT = std::string, typename NodeIDT = unsigned int, typename EdgeT =
unsigned long long int>
static const NodeID LinkPred::UNetwork< LabelT, NodeIDT, EdgeT >::start (
            Edge const & edge ) [inline], [static]
```

Parameters

edge	An edge.

Returns

The starting node of edge.

9.131.4.68 write()

```
template<typename LabelT = std::string, typename NodeIDT = unsigned int, typename EdgeT =
unsigned long long int>
void LinkPred::UNetwork< LabelT, NodeIDT, EdgeT >::write (
            std::string fileName ) const
```

Write adjacency matrix in sparse form to file.

Parameters

fileName	the file name.

The documentation for this class was generated from the following file:

- include/linkpred/core/unetwork/unetwork.hpp

9.132 LinkPred::UPATPredictor< Network, EdgeRndIt, ScoreRndIt, EdgeRndOutIt > Class Template Reference

A preferential attachment link predictor.

```
#include <upatpredictor.hpp>
```

Inheritance diagram for LinkPred::UPATPredictor< Network, EdgeRndIt, ScoreRndIt, EdgeRndOutIt >:

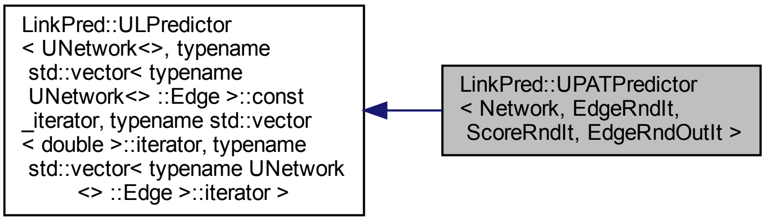

Collaboration diagram for LinkPred::UPATPredictor< Network, EdgeRndIt, ScoreRndIt, EdgeRndOutIt >:

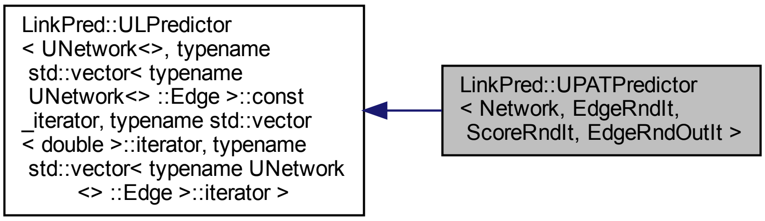

Public Member Functions

- UPATPredictor (std::shared_ptr< Network const > net)
- UPATPredictor (UPATPredictor const &that)=default
- UPATPredictor & operator= (UPATPredictor const &that)=default
- UPATPredictor (UPATPredictor &&that)=default
- UPATPredictor & operator= (UPATPredictor &&that)=default
- virtual void init ()
- virtual void learn ()
- virtual double score (Edge const &e)
- virtual void predict (EdgeRndIt begin, EdgeRndIt end, ScoreRndIt scores)
- virtual std::size_t top (std::size_t k, EdgeRndOutIt eit, ScoreRndIt sit)
- virtual ~UPATPredictor ()=default

Additional Inherited Members

9.132.1 Detailed Description

template<typename Network = UNetwork<>, typename EdgeRndIt = typename std::vector<typename Network::Edge>↩ ::const_iterator, typename ScoreRndIt = typename std::vector<double>::iterator, typename EdgeRndOutIt = typename std↩ ::vector<typename Network::Edge>::iterator>
class LinkPred::UPATPredictor< Network, EdgeRndIt, ScoreRndIt, EdgeRndOutIt >

A preferential attachment link predictor.

Template Parameters

Network	The network type.
Edge↩ RndIt	A random iterator type used to iterate on edges.
Score↩ RndIt	A random iterator type used to iterate on scores.

9.132.2 Constructor & Destructor Documentation

9.132.2.1 UPATPredictor() [1/3]

```
template<typename Network = UNetwork<>, typename EdgeRndIt = typename std::vector<typename
Network::Edge>::const_iterator, typename ScoreRndIt = typename std::vector<double>::iterator,
typename EdgeRndOutIt = typename std::vector<typename Network::Edge>::iterator>
LinkPred::UPATPredictor< Network, EdgeRndIt, ScoreRndIt, EdgeRndOutIt >::UPATPredictor (
            std::shared_ptr< Network const > net )  [inline]
```

Parameters

net	The network.

9.132.2.2 UPATPredictor() [2/3]

```
template<typename Network = UNetwork<>, typename EdgeRndIt = typename std::vector<typename
Network::Edge>::const_iterator, typename ScoreRndIt = typename std::vector<double>::iterator,
typename EdgeRndOutIt = typename std::vector<typename Network::Edge>::iterator>
LinkPred::UPATPredictor< Network, EdgeRndIt, ScoreRndIt, EdgeRndOutIt >::UPATPredictor (
            UPATPredictor< Network, EdgeRndIt, ScoreRndIt, EdgeRndOutIt > const & that )
[default]
```

Copy constructor.

Parameters

that	The object to copy.

9.132.2.3 UPATPredictor() [3/3]

```
template<typename Network = UNetwork<>, typename EdgeRndIt = typename std::vector<typename
Network::Edge>::const_iterator, typename ScoreRndIt = typename std::vector<double>::iterator,
typename EdgeRndOutIt = typename std::vector<typename Network::Edge>::iterator>
LinkPred::UPATPredictor< Network, EdgeRndIt, ScoreRndIt, EdgeRndOutIt >::UPATPredictor (
            UPATPredictor< Network, EdgeRndIt, ScoreRndIt, EdgeRndOutIt > && that ) [default]
```

Move constructor.

Parameters

that	The object to move.

9.132.2.4 ∼UPATPredictor()

```
template<typename Network = UNetwork<>, typename EdgeRndIt = typename std::vector<typename
Network::Edge>::const_iterator, typename ScoreRndIt = typename std::vector<double>::iterator,
typename EdgeRndOutIt = typename std::vector<typename Network::Edge>::iterator>
virtual LinkPred::UPATPredictor< Network, EdgeRndIt, ScoreRndIt, EdgeRndOutIt >::~UPATPredictor
( ) [virtual], [default]
```

Destructor.

9.132.3 Member Function Documentation

9.132.3.1 init()

```
template<typename Network = UNetwork<>, typename EdgeRndIt = typename std::vector<typename
Network::Edge>::const_iterator, typename ScoreRndIt = typename std::vector<double>::iterator,
typename EdgeRndOutIt = typename std::vector<typename Network::Edge>::iterator>
virtual void LinkPred::UPATPredictor< Network, EdgeRndIt, ScoreRndIt, EdgeRndOutIt >::init ( )
[inline], [virtual]
```

Initialize the predictor.

Implements LinkPred::ULPredictor< UNetwork<>, typename std::vector< typename UNetwork<> ::Edge >::const_iterator, typena

9.132.3.2 learn()

```
template<typename Network = UNetwork<>, typename EdgeRndIt = typename std::vector<typename
Network::Edge>::const_iterator, typename ScoreRndIt = typename std::vector<double>::iterator,
typename EdgeRndOutIt = typename std::vector<typename Network::Edge>::iterator>
virtual void LinkPred::UPATPredictor< Network, EdgeRndIt, ScoreRndIt, EdgeRndOutIt >::learn (
) [inline], [virtual]
```

Learning.

Implements LinkPred::ULPredictor< UNetwork<>, typename std::vector< typename UNetwork<> ::Edge >::const_iterator, typena [illegible]

9.132.3.3 operator=() [1/2]

```
template<typename Network = UNetwork<>, typename EdgeRndIt = typename std::vector<typename
Network::Edge>::const_iterator, typename ScoreRndIt = typename std::vector<double>::iterator,
typename EdgeRndOutIt = typename std::vector<typename Network::Edge>::iterator>
UPATPredictor& LinkPred::UPATPredictor< Network, EdgeRndIt, ScoreRndIt, EdgeRndOutIt >::operator=
(
            UPATPredictor< Network, EdgeRndIt, ScoreRndIt, EdgeRndOutIt > && that )  [default]
```

Move assignment operator.

Parameters

that	The object to move.

9.132.3.4 operator=() [2/2]

```
template<typename Network = UNetwork<>, typename EdgeRndIt = typename std::vector<typename
Network::Edge>::const_iterator, typename ScoreRndIt = typename std::vector<double>::iterator,
typename EdgeRndOutIt = typename std::vector<typename Network::Edge>::iterator>
UPATPredictor& LinkPred::UPATPredictor< Network, EdgeRndIt, ScoreRndIt, EdgeRndOutIt >::operator=
(
            UPATPredictor< Network, EdgeRndIt, ScoreRndIt, EdgeRndOutIt > const & that )
[default]
```

Copy assignment operator.

Parameters

that	The object to copy.

9.132.3.5 predict()

```
template<typename Network = UNetwork<>, typename EdgeRndIt = typename std::vector<typename
Network::Edge>::const_iterator, typename ScoreRndIt = typename std::vector<double>::iterator,
typename EdgeRndOutIt = typename std::vector<typename Network::Edge>::iterator>
virtual void LinkPred::UPATPredictor< Network, EdgeRndIt, ScoreRndIt, EdgeRndOutIt >::predict
(
            EdgeRndIt begin,
            EdgeRndIt end,
            ScoreRndIt scores )  [virtual]
```

Predict the links.

Parameters

begin	Beginning of the links to be predicted.
end	end of the links to be predicted.
scores	Beginning of scores.

Reimplemented from LinkPred::ULPredictor< UNetwork<>, typename std::vector< typename UNetwork<> ::Edge >::const_iterat

9.132.3.6 score()

```
template<typename Network = UNetwork<>, typename EdgeRndIt = typename std::vector<typename
Network::Edge>::const_iterator, typename ScoreRndIt = typename std::vector<double>::iterator,
typename EdgeRndOutIt = typename std::vector<typename Network::Edge>::iterator>
virtual double LinkPred::UPATPredictor< Network, EdgeRndIt, ScoreRndIt, EdgeRndOutIt >::score
(
            Edge const & e )  [inline], [virtual]
```

Compute the score of a single edge.

Parameters

e	The edge.

Returns

The score of e.

9.132.3.7 top()

```
template<typename Network = UNetwork<>, typename EdgeRndIt = typename std::vector<typename
Network::Edge>::const_iterator, typename ScoreRndIt = typename std::vector<double>::iterator,
typename EdgeRndOutIt = typename std::vector<typename Network::Edge>::iterator>
```

```
virtual std::size_t LinkPred::UPATPredictor< Network, EdgeRndIt, ScoreRndIt, EdgeRndOutIt >↩
::top (
            std::size_t k,
            EdgeRndOutIt eit,
            ScoreRndIt sit )  [inline], [virtual]
```

Finds the k negative edges with the top score. Ties are broken randomly.

Parameters

k	The number of edges to find.
eit	An output iterator where the edges are written.
sit	An output iterator where the scores are written. The scores are written in the same order as the edges.

Returns

The number of negative edges inserted. It is the minimum between k and the number of negative edges in the network.

Reimplemented from LinkPred::ULPredictor< UNetwork<>, typename std::vector< typename UNetwork<> ::Edge >::const_iterat

The documentation for this class was generated from the following file:

- include/linkpred/predictors/undirected/upatpredictor.hpp

9.133 LinkPred::UPSTPredictor< Network, EdgeRndIt, ScoreRndIt, EdgeRndOutIt > Class Template Reference

A link predictor that prestores edge scores. This allows to seemingly integrate results from external link prediction algorithms to LinkPred (for example, users may implement their own link prediction algorithm and then use this link predictor to use compare their results to algorithms available in LinkPred).

```
#include <upstpredictor.hpp>
```

Inheritance diagram for LinkPred::UPSTPredictor< Network, EdgeRndIt, ScoreRndIt, EdgeRndOutIt >:

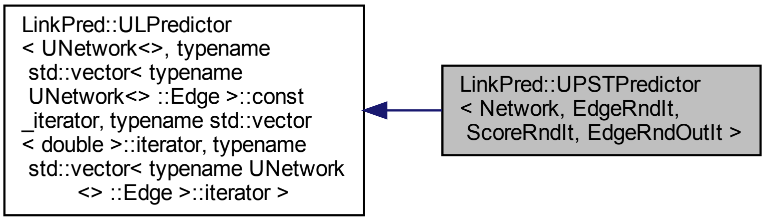

Collaboration diagram for LinkPred::UPSTPredictor< Network, EdgeRndIt, ScoreRndIt, EdgeRndOutIt >:

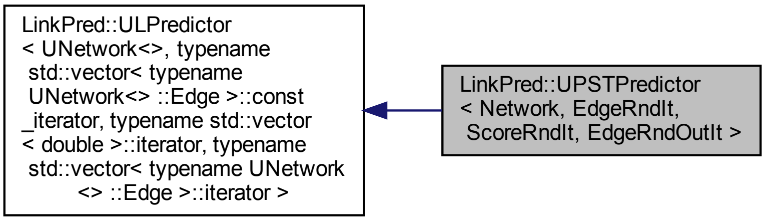

Public Member Functions

- UPSTPredictor (std::shared_ptr< Network const > net)
- UPSTPredictor (UPSTPredictor const &that)=default
- UPSTPredictor & operator= (UPSTPredictor const &that)=default
- UPSTPredictor (UPSTPredictor &&that)=default
- UPSTPredictor & operator= (UPSTPredictor &&that)=default
- void setEdgeScores (typename Network::template EdgeMapSP< double > edgeScores)
- void loadEdgeScores (std::string fileName)
- virtual void init ()
- virtual void learn ()
- virtual double score (Edge const &e)
- virtual ~UPSTPredictor ()=default

Additional Inherited Members

9.133.1 Detailed Description

template<typename Network = UNetwork<>, typename EdgeRndIt = typename std::vector<typename Network::Edge>↩ ::const_iterator, typename ScoreRndIt = typename std::vector<double>::iterator, typename EdgeRndOutIt = typename std↩ ::vector<typename Network::Edge>::iterator>
class LinkPred::UPSTPredictor< Network, EdgeRndIt, ScoreRndIt, EdgeRndOutIt >

A link predictor that prestores edge scores. This allows to seemingly integrate results from external link prediction algorithms to LinkPred (for example, users may implement their own link prediction algorithm and then use this link predictor to use compare their results to algorithms available in LinkPred).

Template Parameters

Network	The network type.
Edge↩ RndIt	A random iterator type used to iterate on edges.
Score↩ RndIt	A random iterator type used to iterate on scores.

9.133.2 Constructor & Destructor Documentation

9.133.2.1 UPSTPredictor() [1/3]

```
template<typename Network = UNetwork<>, typename EdgeRndIt = typename std::vector<typename
Network::Edge>::const_iterator, typename ScoreRndIt = typename std::vector<double>::iterator,
typename EdgeRndOutIt = typename std::vector<typename Network::Edge>::iterator>
LinkPred::UPSTPredictor< Network, EdgeRndIt, ScoreRndIt, EdgeRndOutIt >::UPSTPredictor (
            std::shared_ptr< Network const > net ) [inline]
```

Parameters

net	The network.

9.133.2.2 UPSTPredictor() [2/3]

```
template<typename Network = UNetwork<>, typename EdgeRndIt = typename std::vector<typename
Network::Edge>::const_iterator, typename ScoreRndIt = typename std::vector<double>::iterator,
typename EdgeRndOutIt = typename std::vector<typename Network::Edge>::iterator>
LinkPred::UPSTPredictor< Network, EdgeRndIt, ScoreRndIt, EdgeRndOutIt >::UPSTPredictor (
            UPSTPredictor< Network, EdgeRndIt, ScoreRndIt, EdgeRndOutIt > const & that )
[default]
```

Copy constructor.

Parameters

that	The object to copy.

9.133.2.3 UPSTPredictor() [3/3]

```
template<typename Network = UNetwork<>, typename EdgeRndIt = typename std::vector<typename
Network::Edge>::const_iterator, typename ScoreRndIt = typename std::vector<double>::iterator,
typename EdgeRndOutIt = typename std::vector<typename Network::Edge>::iterator>
LinkPred::UPSTPredictor< Network, EdgeRndIt, ScoreRndIt, EdgeRndOutIt >::UPSTPredictor (
            UPSTPredictor< Network, EdgeRndIt, ScoreRndIt, EdgeRndOutIt > && that ) [default]
```

Move constructor.

Parameters

that	The object to move.

9.133.2.4 ∼UPSTPredictor()

```
template<typename Network = UNetwork<>, typename EdgeRndIt = typename std::vector<typename
Network::Edge>::const_iterator, typename ScoreRndIt = typename std::vector<double>::iterator,
typename EdgeRndOutIt = typename std::vector<typename Network::Edge>::iterator>
virtual LinkPred::UPSTPredictor< Network, EdgeRndIt, ScoreRndIt, EdgeRndOutIt >::~UPSTPredictor
( ) [virtual], [default]
```

Destructor.

9.133.3 Member Function Documentation

9.133.3.1 init()

```
template<typename Network = UNetwork<>, typename EdgeRndIt = typename std::vector<typename
Network::Edge>::const_iterator, typename ScoreRndIt = typename std::vector<double>::iterator,
typename EdgeRndOutIt = typename std::vector<typename Network::Edge>::iterator>
virtual void LinkPred::UPSTPredictor< Network, EdgeRndIt, ScoreRndIt, EdgeRndOutIt >::init ( )
[inline], [virtual]
```

Initialize the solver.

Implements LinkPred::ULPredictor< UNetwork<>, typename std::vector< typename UNetwork<> ::Edge >::const_iterator, typena

9.133.3.2 learn()

```
template<typename Network = UNetwork<>, typename EdgeRndIt = typename std::vector<typename
Network::Edge>::const_iterator, typename ScoreRndIt = typename std::vector<double>::iterator,
typename EdgeRndOutIt = typename std::vector<typename Network::Edge>::iterator>
virtual void LinkPred::UPSTPredictor< Network, EdgeRndIt, ScoreRndIt, EdgeRndOutIt >::learn (
) [inline], [virtual]
```

Learn.

Implements LinkPred::ULPredictor< UNetwork<>, typename std::vector< typename UNetwork<> ::Edge >::const_iterator, typena

9.133.3.3 loadEdgeScores()

```
template<typename Network = UNetwork<>, typename EdgeRndIt = typename std::vector<typename
Network::Edge>::const_iterator, typename ScoreRndIt = typename std::vector<double>::iterator,
typename EdgeRndOutIt = typename std::vector<typename Network::Edge>::iterator>
void LinkPred::UPSTPredictor< Network, EdgeRndIt, ScoreRndIt, EdgeRndOutIt >::loadEdgeScores (
            std::string fileName )
```

Load edge scores from file. This file should contain the scores of all non-exisitng links of net.

Parameters

fileName	The name of the file containing edge scores. This should be a text file in which each line contains three columns separate by a space character (one or multiple spaces or tabs). The first two columns contain the the labels (not the internal IDs) of two nodes composing the edge. The third column contains the score. For example: A B 0.35 or: 5 8 2.6 All node labels that appear in this file must already exist in the network.

9.133.3.4 operator=() [1/2]

```
template<typename Network = UNetwork<>, typename EdgeRndIt = typename std::vector<typename
Network::Edge>::const_iterator, typename ScoreRndIt = typename std::vector<double>::iterator,
typename EdgeRndOutIt = typename std::vector<typename Network::Edge>::iterator>
UPSTPredictor& LinkPred::UPSTPredictor< Network, EdgeRndIt, ScoreRndIt, EdgeRndOutIt >::operator=
(
            UPSTPredictor< Network, EdgeRndIt, ScoreRndIt, EdgeRndOutIt > && that )  [default]
```

Move assignment operator.

Parameters

that	The object to move.

9.133.3.5 operator=() [2/2]

```
template<typename Network = UNetwork<>, typename EdgeRndIt = typename std::vector<typename
Network::Edge>::const_iterator, typename ScoreRndIt = typename std::vector<double>::iterator,
typename EdgeRndOutIt = typename std::vector<typename Network::Edge>::iterator>
UPSTPredictor& LinkPred::UPSTPredictor< Network, EdgeRndIt, ScoreRndIt, EdgeRndOutIt >::operator=
(
            UPSTPredictor< Network, EdgeRndIt, ScoreRndIt, EdgeRndOutIt > const & that )
[default]
```

Copy assignment operator.

Parameters

that	The object to copy.

9.133.3.6 score()

```
template<typename Network = UNetwork<>, typename EdgeRndIt = typename std::vector<typename
Network::Edge>::const_iterator, typename ScoreRndIt = typename std::vector<double>::iterator,
```

```
typename EdgeRndOutIt = typename std::vector<typename Network::Edge>::iterator>
virtual double LinkPred::UPSTPredictor< Network, EdgeRndIt, ScoreRndIt, EdgeRndOutIt >::score
(
            Edge const & e )  [virtual]
```

Compute the score of a single edge.

Parameters

e	The edge.

Returns

The score of e.

9.133.3.7 setEdgeScores()

```
template<typename Network = UNetwork<>, typename EdgeRndIt = typename std::vector<typename
Network::Edge>::const_iterator, typename ScoreRndIt = typename std::vector<double>::iterator,
typename EdgeRndOutIt = typename std::vector<typename Network::Edge>::iterator>
void LinkPred::UPSTPredictor< Network, EdgeRndIt, ScoreRndIt, EdgeRndOutIt >::setEdgeScores (
            typename Network::template EdgeMapSP< double > edgeScores )  [inline]
```

Set the edge scores. This should contain the scores of all non-exisitng links of net.

Parameters

edgeScores	A map contatining the scores of non-exisitng links of net.

The documentation for this class was generated from the following file:

- include/linkpred/predictors/undirected/upstpredictor.hpp

9.134 LinkPred::URALPredictor< Network, EdgeRndIt, ScoreRndIt, EdgeRndOutIt > Class Template Reference

A resource allocation link predictor.

```
#include <uralpredictor.hpp>
```

Inheritance diagram for LinkPred::URALPredictor< Network, EdgeRndIt, ScoreRndIt, EdgeRndOutIt >:

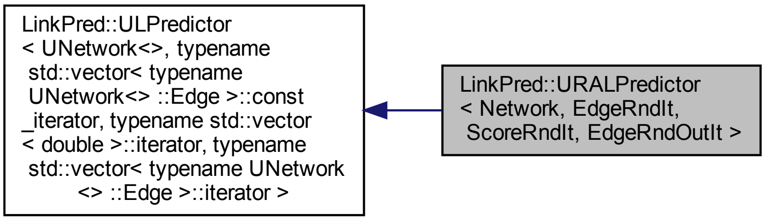

Collaboration diagram for LinkPred::URALPredictor< Network, EdgeRndIt, ScoreRndIt, EdgeRndOutIt >:

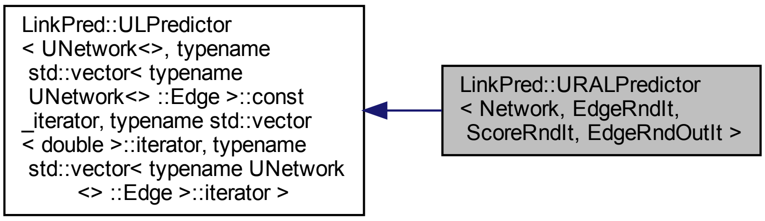

Public Member Functions

- URALPredictor (std::shared_ptr< Network const > net)
- URALPredictor (URALPredictor const &that)=default
- URALPredictor & operator= (URALPredictor const &that)=default
- URALPredictor (URALPredictor &&that)=default
- URALPredictor & operator= (URALPredictor &&that)=default
- virtual void init ()
- virtual void learn ()
- virtual double score (Edge const &e)
- virtual void predict (EdgeRndIt begin, EdgeRndIt end, ScoreRndIt scores)
- virtual std::size_t top (std::size_t k, EdgeRndOutIt eit, ScoreRndIt sit)
- virtual ~URALPredictor ()=default

Additional Inherited Members

9.134.1 Detailed Description

template<typename Network = UNetwork<>, typename EdgeRndIt = typename std::vector<typename Network::Edge>::const_iterator, typename ScoreRndIt = typename std::vector<double>::iterator, typename EdgeRndOutIt = typename std::vector<typename Network::Edge>::iterator>
class LinkPred::URALPredictor< Network, EdgeRndIt, ScoreRndIt, EdgeRndOutIt >

A resource allocation link predictor.

Template Parameters

Network	The network type.
Edge↩ RndIt	A random iterator type used to iterate on edges.
Score↩ RndIt	A random iterator type used to iterate on scores.

9.134.2 Constructor & Destructor Documentation

9.134.2.1 URALPredictor() [1/3]

```
template<typename Network = UNetwork<>, typename EdgeRndIt = typename std::vector<typename
Network::Edge>::const_iterator, typename ScoreRndIt = typename std::vector<double>::iterator,
typename EdgeRndOutIt = typename std::vector<typename Network::Edge>::iterator>
LinkPred::URALPredictor< Network, EdgeRndIt, ScoreRndIt, EdgeRndOutIt >::URALPredictor (
            std::shared_ptr< Network const > net )  [inline]
```

Parameters

net	The network.

9.134.2.2 URALPredictor() [2/3]

```
template<typename Network = UNetwork<>, typename EdgeRndIt = typename std::vector<typename
Network::Edge>::const_iterator, typename ScoreRndIt = typename std::vector<double>::iterator,
typename EdgeRndOutIt = typename std::vector<typename Network::Edge>::iterator>
LinkPred::URALPredictor< Network, EdgeRndIt, ScoreRndIt, EdgeRndOutIt >::URALPredictor (
            URALPredictor< Network, EdgeRndIt, ScoreRndIt, EdgeRndOutIt > const & that )
[default]
```

Copy constructor.

Parameters

that	The object to copy.

9.134.2.3 URALPredictor() [3/3]

```
template<typename Network = UNetwork<>, typename EdgeRndIt = typename std::vector<typename
Network::Edge>::const_iterator, typename ScoreRndIt = typename std::vector<double>::iterator,
```

```
typename EdgeRndOutIt = typename std::vector<typename Network::Edge>::iterator>
LinkPred::URALPredictor< Network, EdgeRndIt, ScoreRndIt, EdgeRndOutIt >::URALPredictor (
            URALPredictor< Network, EdgeRndIt, ScoreRndIt, EdgeRndOutIt > && that )  [default]
```

Move constructor.

Parameters

that	The object to move.

9.134.2.4 ∼URALPredictor()

```
template<typename Network = UNetwork<>, typename EdgeRndIt = typename std::vector<typename
Network::Edge>::const_iterator, typename ScoreRndIt = typename std::vector<double>::iterator,
typename EdgeRndOutIt = typename std::vector<typename Network::Edge>::iterator>
virtual LinkPred::URALPredictor< Network, EdgeRndIt, ScoreRndIt, EdgeRndOutIt >::~URALPredictor
( )  [virtual], [default]
```

Destructor.

9.134.3 Member Function Documentation

9.134.3.1 init()

```
template<typename Network = UNetwork<>, typename EdgeRndIt = typename std::vector<typename
Network::Edge>::const_iterator, typename ScoreRndIt = typename std::vector<double>::iterator,
typename EdgeRndOutIt = typename std::vector<typename Network::Edge>::iterator>
virtual void LinkPred::URALPredictor< Network, EdgeRndIt, ScoreRndIt, EdgeRndOutIt >::init ( )
[inline], [virtual]
```

Initialize the predictor.

Implements LinkPred::ULPredictor< UNetwork<>, typename std::vector< typename UNetwork<> ::Edge >::const_iterator, typena

9.134.3.2 learn()

```
template<typename Network = UNetwork<>, typename EdgeRndIt = typename std::vector<typename
Network::Edge>::const_iterator, typename ScoreRndIt = typename std::vector<double>::iterator,
typename EdgeRndOutIt = typename std::vector<typename Network::Edge>::iterator>
virtual void LinkPred::URALPredictor< Network, EdgeRndIt, ScoreRndIt, EdgeRndOutIt >::learn (
)  [inline], [virtual]
```

Learning.

Implements LinkPred::ULPredictor< UNetwork<>, typename std::vector< typename UNetwork<> ::Edge >::const_iterator, typena

9.134.3.3 operator=() [1/2]

```
template<typename Network = UNetwork<>, typename EdgeRndIt = typename std::vector<typename
Network::Edge>::const_iterator, typename ScoreRndIt = typename std::vector<double>::iterator,
typename EdgeRndOutIt = typename std::vector<typename Network::Edge>::iterator>
URALPredictor& LinkPred::URALPredictor< Network, EdgeRndIt, ScoreRndIt, EdgeRndOutIt >::operator=
(
            URALPredictor< Network, EdgeRndIt, ScoreRndIt, EdgeRndOutIt > && that )  [default]
```

Move assignment operator.

Parameters

that	The object to move.

9.134.3.4 operator=() [2/2]

```
template<typename Network = UNetwork<>, typename EdgeRndIt = typename std::vector<typename
Network::Edge>::const_iterator, typename ScoreRndIt = typename std::vector<double>::iterator,
typename EdgeRndOutIt = typename std::vector<typename Network::Edge>::iterator>
URALPredictor& LinkPred::URALPredictor< Network, EdgeRndIt, ScoreRndIt, EdgeRndOutIt >::operator=
(
            URALPredictor< Network, EdgeRndIt, ScoreRndIt, EdgeRndOutIt > const & that )
[default]
```

Copy assignment operator.

Parameters

that	The object to copy.

9.134.3.5 predict()

```
template<typename Network = UNetwork<>, typename EdgeRndIt = typename std::vector<typename
Network::Edge>::const_iterator, typename ScoreRndIt = typename std::vector<double>::iterator,
typename EdgeRndOutIt = typename std::vector<typename Network::Edge>::iterator>
virtual void LinkPred::URALPredictor< Network, EdgeRndIt, ScoreRndIt, EdgeRndOutIt >::predict
(
            EdgeRndIt begin,
            EdgeRndIt end,
            ScoreRndIt scores )  [virtual]
```

Predict the links.

Parameters

begin	Beginning of the links to be predicted.
end	end of the links to be predicted.
scores	Beginning of scores.

Reimplemented from LinkPred::ULPredictor< UNetwork<>, typename std::vector< typename UNetwork<> ::Edge >::const_iterato

9.134.3.6 score()

```
template<typename Network = UNetwork<>, typename EdgeRndIt = typename std::vector<typename
Network::Edge>::const_iterator, typename ScoreRndIt = typename std::vector<double>::iterator,
typename EdgeRndOutIt = typename std::vector<typename Network::Edge>::iterator>
virtual double LinkPred::URALPredictor< Network, EdgeRndIt, ScoreRndIt, EdgeRndOutIt >::score
(
            Edge const & e )  [inline], [virtual]
```

Compute the score of a single edge.

Parameters

e	The edge.

Returns

The score of e.

9.134.3.7 top()

```
template<typename Network = UNetwork<>, typename EdgeRndIt = typename std::vector<typename
Network::Edge>::const_iterator, typename ScoreRndIt = typename std::vector<double>::iterator,
typename EdgeRndOutIt = typename std::vector<typename Network::Edge>::iterator>
virtual std::size_t LinkPred::URALPredictor< Network, EdgeRndIt, ScoreRndIt, EdgeRndOutIt >←
::top (
            std::size_t k,
            EdgeRndOutIt eit,
            ScoreRndIt sit )  [virtual]
```

Finds the k negative edges with the top score. Ties are broken randmly.

Parameters

k	The number of edges to find.
eit	An output iterator where the edges are written.
sit	An output iterator where the scores are written. The scores are written in the same order as the edges.

Returns

The number of negative edges inserted. It is the minimum between k and the number of negative edges in the network.

Reimplemented from LinkPred::ULPredictor< UNetwork<>, typename std::vector< typename UNetwork<> ::Edge >::const_iterato

The documentation for this class was generated from the following file:

- include/linkpred/predictors/undirected/uralpredictor.hpp

9.135 LinkPred::URNDPredictor< Network, EdgeRndIt, ScoreRndIt, EdgeRndOutIt > Class Template Reference

A random link predictor.

```
#include <urndpredictor.hpp>
```

Inheritance diagram for LinkPred::URNDPredictor< Network, EdgeRndIt, ScoreRndIt, EdgeRndOutIt >:

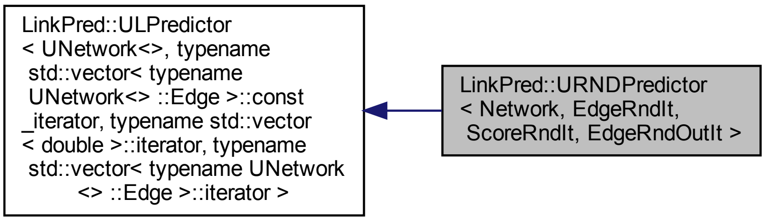

Collaboration diagram for LinkPred::URNDPredictor< Network, EdgeRndIt, ScoreRndIt, EdgeRndOutIt >:

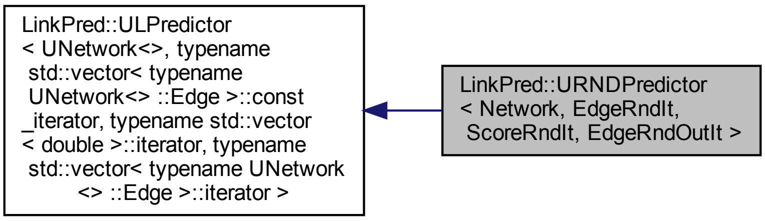

Public Member Functions

- URNDPredictor (std::shared_ptr< Network const > net, long int seed)
- URNDPredictor (URNDPredictor const &that)=default
- URNDPredictor & operator= (URNDPredictor const &that)=default
- URNDPredictor (URNDPredictor &&that)=default
- URNDPredictor & operator= (URNDPredictor &&that)=default
- virtual void init ()
- virtual void learn ()
- virtual void predict (EdgeRndIt begin, EdgeRndIt end, ScoreRndIt scores)
- virtual double score (Edge const &e)
- virtual ~URNDPredictor ()=default

Additional Inherited Members

9.135.1 Detailed Description

template< typename Network = UNetwork<>, typename EdgeRndIt = typename std::vector< typename Network::Edge >↩ ::const_iterator, typename ScoreRndIt = typename std::vector< double >::iterator, typename EdgeRndOutIt = typename std↩ ::vector< typename Network::Edge >::iterator>
class LinkPred::URNDPredictor< Network, EdgeRndIt, ScoreRndIt, EdgeRndOutIt >

A random link predictor.

Template Parameters

Network	The network type.
Edge↩ RndIt	A random iterator type used to iterate on edges.
Score↩ RndIt	A random iterator type used to iterate on scores.

9.135.2 Constructor & Destructor Documentation

9.135.2.1 URNDPredictor() [1/3]

```
template<typename Network = UNetwork<>, typename EdgeRndIt = typename std::vector<typename
Network::Edge>::const_iterator, typename ScoreRndIt = typename std::vector<double>::iterator,
typename EdgeRndOutIt = typename std::vector<typename Network::Edge>::iterator>
LinkPred::URNDPredictor< Network, EdgeRndIt, ScoreRndIt, EdgeRndOutIt >::URNDPredictor (
            std::shared_ptr< Network const > net,
            long int seed )  [inline]
```

Parameters

net	The network.
seed	The random number generator's seed.

9.135.2.2 URNDPredictor() [2/3]

```
template<typename Network = UNetwork<>, typename EdgeRndIt = typename std::vector<typename
Network::Edge>::const_iterator, typename ScoreRndIt = typename std::vector<double>::iterator,
typename EdgeRndOutIt = typename std::vector<typename Network::Edge>::iterator>
LinkPred::URNDPredictor< Network, EdgeRndIt, ScoreRndIt, EdgeRndOutIt >::URNDPredictor (
            URNDPredictor< Network, EdgeRndIt, ScoreRndIt, EdgeRndOutIt > const & that )
[default]
```

Copy constructor.

Parameters

that	The object to copy.

9.135.2.3 URNDPredictor() [3/3]

```
template<typename Network = UNetwork<>, typename EdgeRndIt = typename std::vector<typename
Network::Edge>::const_iterator, typename ScoreRndIt = typename std::vector<double>::iterator,
typename EdgeRndOutIt = typename std::vector<typename Network::Edge>::iterator>
LinkPred::URNDPredictor< Network, EdgeRndIt, ScoreRndIt, EdgeRndOutIt >::URNDPredictor (
            URNDPredictor< Network, EdgeRndIt, ScoreRndIt, EdgeRndOutIt > && that )  [default]
```

Move constructor.

Parameters

that	The object to move.

9.135.2.4 ~URNDPredictor()

```
template<typename Network = UNetwork<>, typename EdgeRndIt = typename std::vector<typename
Network::Edge>::const_iterator, typename ScoreRndIt = typename std::vector<double>::iterator,
typename EdgeRndOutIt = typename std::vector<typename Network::Edge>::iterator>
virtual LinkPred::URNDPredictor< Network, EdgeRndIt, ScoreRndIt, EdgeRndOutIt >::~URNDPredictor
( )  [virtual], [default]
```

Destructor.

9.135.3 Member Function Documentation

9.135.3.1 init()

```
template<typename Network = UNetwork<>, typename EdgeRndIt = typename std::vector<typename
Network::Edge>::const_iterator, typename ScoreRndIt = typename std::vector<double>::iterator,
typename EdgeRndOutIt = typename std::vector<typename Network::Edge>::iterator>
virtual void LinkPred::URNDPredictor< Network, EdgeRndIt, ScoreRndIt, EdgeRndOutIt >::init ( )
[inline], [virtual]
```

Initialize the solver.

Implements LinkPred::ULPredictor< UNetwork<>, typename std::vector< typename UNetwork<> ::Edge >::const_iterator, typena

9.135.3.2 learn()

```
template<typename Network = UNetwork<>, typename EdgeRndIt = typename std::vector<typename
Network::Edge>::const_iterator, typename ScoreRndIt = typename std::vector<double>::iterator,
typename EdgeRndOutIt = typename std::vector<typename Network::Edge>::iterator>
virtual void LinkPred::URNDPredictor< Network, EdgeRndIt, ScoreRndIt, EdgeRndOutIt >::learn (
) [inline], [virtual]
```

Learn.

Implements LinkPred::ULPredictor< UNetwork<>, typename std::vector< typename UNetwork<> ::Edge >::const_iterator, typena

9.135.3.3 operator=() [1/2]

```
template<typename Network = UNetwork<>, typename EdgeRndIt = typename std::vector<typename
Network::Edge>::const_iterator, typename ScoreRndIt = typename std::vector<double>::iterator,
typename EdgeRndOutIt = typename std::vector<typename Network::Edge>::iterator>
URNDPredictor& LinkPred::URNDPredictor< Network, EdgeRndIt, ScoreRndIt, EdgeRndOutIt >::operator=
(
            URNDPredictor< Network, EdgeRndIt, ScoreRndIt, EdgeRndOutIt > && that )  [default]
```

Move assignment operator.

Parameters

that	The object to move.

9.135.3.4 operator=() [2/2]

```
template<typename Network = UNetwork<>, typename EdgeRndIt = typename std::vector<typename
Network::Edge>::const_iterator, typename ScoreRndIt = typename std::vector<double>::iterator,
typename EdgeRndOutIt = typename std::vector<typename Network::Edge>::iterator>
URNDPredictor& LinkPred::URNDPredictor< Network, EdgeRndIt, ScoreRndIt, EdgeRndOutIt >::operator=
(
            URNDPredictor< Network, EdgeRndIt, ScoreRndIt, EdgeRndOutIt > const & that )
[default]
```

Copy assignment operator.

Parameters

that	The object to copy.

9.135.3.5 predict()

```
template<typename Network = UNetwork<>, typename EdgeRndIt = typename std::vector<typename
Network::Edge>::const_iterator, typename ScoreRndIt = typename std::vector<double>::iterator,
typename EdgeRndOutIt = typename std::vector<typename Network::Edge>::iterator>
virtual void LinkPred::URNDPredictor< Network, EdgeRndIt, ScoreRndIt, EdgeRndOutIt >::predict
(
            EdgeRndIt begin,
            EdgeRndIt end,
            ScoreRndIt scores )  [virtual]
```

Predict the links.

Parameters

begin	Beginning of the links to be predicted.
end	end of the links to be predicted.
scores	Beginning of scores.

Reimplemented from LinkPred::ULPredictor< UNetwork<>, typename std::vector< typename UNetwork<> ::Edge >::const_iterat[illegible]

9.135.3.6 score()

```
template<typename Network = UNetwork<>, typename EdgeRndIt = typename std::vector<typename
Network::Edge>::const_iterator, typename ScoreRndIt = typename std::vector<double>::iterator,
typename EdgeRndOutIt = typename std::vector<typename Network::Edge>::iterator>
virtual double LinkPred::URNDPredictor< Network, EdgeRndIt, ScoreRndIt, EdgeRndOutIt >::score
(
            Edge const & e )  [virtual]
```

Compute the score of a single edge.

Parameters

e	The edge.

Returns

The score of e.

The documentation for this class was generated from the following file:

- include/linkpred/predictors/undirected/urndpredictor.hpp

9.136 LinkPred::USAIPredictor< Network, EdgeRndIt, ScoreRndIt, EdgeRndOutIt > Class Template Reference

A Salton index link predictor.

```
#include <usaipredictor.hpp>
```

Inheritance diagram for LinkPred::USAIPredictor< Network, EdgeRndIt, ScoreRndIt, EdgeRndOutIt >:

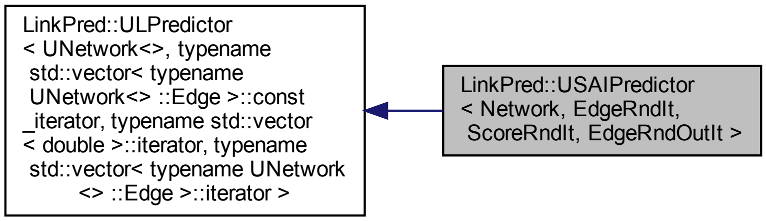

Collaboration diagram for LinkPred::USAIPredictor< Network, EdgeRndIt, ScoreRndIt, EdgeRndOutIt >:

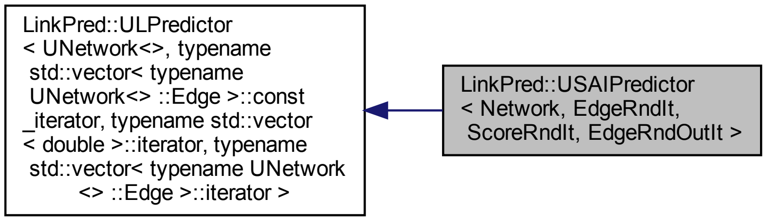

Public Member Functions

- USAIPredictor (std::shared_ptr< Network const > net)
- USAIPredictor (USAIPredictor const &that)=default
- USAIPredictor & operator= (USAIPredictor const &that)=default
- USAIPredictor (USAIPredictor &&that)=default
- USAIPredictor & operator= (USAIPredictor &&that)=default
- virtual void init ()
- virtual void learn ()
- virtual double score (Edge const &e)
- virtual void predict (EdgeRndIt begin, EdgeRndIt end, ScoreRndIt scores)
- virtual std::size_t top (std::size_t k, EdgeRndOutIt eit, ScoreRndIt sit)
- virtual ~USAIPredictor ()=default

Additional Inherited Members

9.136.1 Detailed Description

template<typename Network = UNetwork<>, typename EdgeRndIt = typename std::vector<typename Network::Edge>↩ ::const_iterator, typename ScoreRndIt = typename std::vector<double>::iterator, typename EdgeRndOutIt = typename std↩ ::vector<typename Network::Edge>::iterator>
class LinkPred::USAIPredictor< Network, EdgeRndIt, ScoreRndIt, EdgeRndOutIt >

A Salton index link predictor.

Template Parameters

Network	The network type.
Edge↩ RndIt	A random iterator type used to iterate on edges.
Score↩ RndIt	A random iterator type used to iterate on scores.

9.136.2 Constructor & Destructor Documentation

9.136.2.1 USAIPredictor() [1/3]

```
template<typename Network = UNetwork<>, typename EdgeRndIt = typename std::vector<typename
Network::Edge>::const_iterator, typename ScoreRndIt = typename std::vector<double>::iterator,
typename EdgeRndOutIt = typename std::vector<typename Network::Edge>::iterator>
LinkPred::USAIPredictor< Network, EdgeRndIt, ScoreRndIt, EdgeRndOutIt >::USAIPredictor (
            std::shared_ptr< Network const > net ) [inline]
```

Parameters

net	The network.

9.136.2.2 USAIPredictor() [2/3]

```
template<typename Network = UNetwork<>, typename EdgeRndIt = typename std::vector<typename
Network::Edge>::const_iterator, typename ScoreRndIt = typename std::vector<double>::iterator,
typename EdgeRndOutIt = typename std::vector<typename Network::Edge>::iterator>
LinkPred::USAIPredictor< Network, EdgeRndIt, ScoreRndIt, EdgeRndOutIt >::USAIPredictor (
            USAIPredictor< Network, EdgeRndIt, ScoreRndIt, EdgeRndOutIt > const & that )
[default]
```

Copy constructor.

Parameters

that	The object to copy.

9.136.2.3 USAIPredictor() [3/3]

```
template<typename Network = UNetwork<>, typename EdgeRndIt = typename std::vector<typename
Network::Edge>::const_iterator, typename ScoreRndIt = typename std::vector<double>::iterator,
```

```
typename EdgeRndOutIt = typename std::vector<typename Network::Edge>::iterator>
LinkPred::USAIPredictor< Network, EdgeRndIt, ScoreRndIt, EdgeRndOutIt >::USAIPredictor (
            USAIPredictor< Network, EdgeRndIt, ScoreRndIt, EdgeRndOutIt > && that )  [default]
```

Move constructor.

Parameters

that	The object to move.

9.136.2.4 ∼USAIPredictor()

```
template<typename Network = UNetwork<>, typename EdgeRndIt = typename std::vector<typename
Network::Edge>::const_iterator, typename ScoreRndIt = typename std::vector<double>::iterator,
typename EdgeRndOutIt = typename std::vector<typename Network::Edge>::iterator>
virtual LinkPred::USAIPredictor< Network, EdgeRndIt, ScoreRndIt, EdgeRndOutIt >::~USAIPredictor
( )  [virtual], [default]
```

Destructor.

9.136.3 Member Function Documentation

9.136.3.1 init()

```
template<typename Network = UNetwork<>, typename EdgeRndIt = typename std::vector<typename
Network::Edge>::const_iterator, typename ScoreRndIt = typename std::vector<double>::iterator,
typename EdgeRndOutIt = typename std::vector<typename Network::Edge>::iterator>
virtual void LinkPred::USAIPredictor< Network, EdgeRndIt, ScoreRndIt, EdgeRndOutIt >::init ( )
[inline], [virtual]
```

Initialize the predictor.

Implements LinkPred::ULPredictor< UNetwork<>, typename std::vector< typename UNetwork<> ::Edge >::const_iterator, typena

9.136.3.2 learn()

```
template<typename Network = UNetwork<>, typename EdgeRndIt = typename std::vector<typename
Network::Edge>::const_iterator, typename ScoreRndIt = typename std::vector<double>::iterator,
typename EdgeRndOutIt = typename std::vector<typename Network::Edge>::iterator>
virtual void LinkPred::USAIPredictor< Network, EdgeRndIt, ScoreRndIt, EdgeRndOutIt >::learn (
)  [inline], [virtual]
```

Learning.

Implements LinkPred::ULPredictor< UNetwork<>, typename std::vector< typename UNetwork<> ::Edge >::const_iterator, typena

9.136.3.3 operator=() [1/2]

```
template<typename Network = UNetwork<>, typename EdgeRndIt = typename std::vector<typename
Network::Edge>::const_iterator, typename ScoreRndIt = typename std::vector<double>::iterator,
typename EdgeRndOutIt = typename std::vector<typename Network::Edge>::iterator>
USAIPredictor& LinkPred::USAIPredictor< Network, EdgeRndIt, ScoreRndIt, EdgeRndOutIt >::operator=
(
            USAIPredictor< Network, EdgeRndIt, ScoreRndIt, EdgeRndOutIt > && that )  [default]
```

Move assignment operator.

Parameters

that	The object to move.

9.136.3.4 operator=() [2/2]

```
template<typename Network = UNetwork<>, typename EdgeRndIt = typename std::vector<typename
Network::Edge>::const_iterator, typename ScoreRndIt = typename std::vector<double>::iterator,
typename EdgeRndOutIt = typename std::vector<typename Network::Edge>::iterator>
USAIPredictor& LinkPred::USAIPredictor< Network, EdgeRndIt, ScoreRndIt, EdgeRndOutIt >::operator=
(
            USAIPredictor< Network, EdgeRndIt, ScoreRndIt, EdgeRndOutIt > const & that )
[default]
```

Copy assignment operator.

Parameters

that	The object to copy.

9.136.3.5 predict()

```
template<typename Network = UNetwork<>, typename EdgeRndIt = typename std::vector<typename
Network::Edge>::const_iterator, typename ScoreRndIt = typename std::vector<double>::iterator,
typename EdgeRndOutIt = typename std::vector<typename Network::Edge>::iterator>
virtual void LinkPred::USAIPredictor< Network, EdgeRndIt, ScoreRndIt, EdgeRndOutIt >::predict
(
            EdgeRndIt begin,
            EdgeRndIt end,
            ScoreRndIt scores )  [virtual]
```

Predict the links.

Parameters

begin	Beginning of the links to be predicted.
end	end of the links to be predicted.
scores	Beginning of scores.

Reimplemented from LinkPred::ULPredictor< UNetwork<>, typename std::vector< typename UNetwork<> ::Edge >::const_iterato

9.136.3.6 score()

```
template<typename Network = UNetwork<>, typename EdgeRndIt = typename std::vector<typename
Network::Edge>::const_iterator, typename ScoreRndIt = typename std::vector<double>::iterator,
typename EdgeRndOutIt = typename std::vector<typename Network::Edge>::iterator>
virtual double LinkPred::USAIPredictor< Network, EdgeRndIt, ScoreRndIt, EdgeRndOutIt >::score
(
            Edge const & e )  [inline], [virtual]
```

Compute the score of a single edge.

Parameters

e	The edge.

Returns

The score of e.

9.136.3.7 top()

```
template<typename Network = UNetwork<>, typename EdgeRndIt = typename std::vector<typename
Network::Edge>::const_iterator, typename ScoreRndIt = typename std::vector<double>::iterator,
typename EdgeRndOutIt = typename std::vector<typename Network::Edge>::iterator>
virtual std::size_t LinkPred::USAIPredictor< Network, EdgeRndIt, ScoreRndIt, EdgeRndOutIt >↩
::top (
            std::size_t k,
            EdgeRndOutIt eit,
            ScoreRndIt sit )  [virtual]
```

Finds the k negative edges with the top score. Ties are broken randmly.

Parameters

k	The number of edges to find.
eit	An output iterator where the edges are written.
sit	An output iterator where the scores are written. The scores are written in the same order as the edges.

Returns

The number of negative edges inserted. It is the minimum between k and the number of negative edges in the network.

Reimplemented from LinkPred::ULPredictor< UNetwork<>, typename std::vector< typename UNetwork<> ::Edge >::const_iterato

The documentation for this class was generated from the following file:

- include/linkpred/predictors/undirected/usaipredictor.hpp

9.137 LinkPred::USBMPredictor< Network, EdgeRndIt, ScoreRndIt, EdgeRndOutIt > Class Template Reference

The stochastic block model link predictor.

```
#include <usbmpredictor.hpp>
```

Inheritance diagram for LinkPred::USBMPredictor< Network, EdgeRndIt, ScoreRndIt, EdgeRndOutIt >:

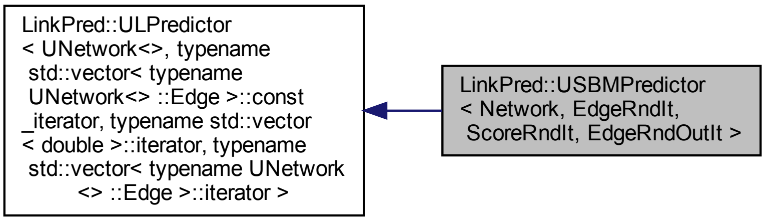

Collaboration diagram for LinkPred::USBMPredictor< Network, EdgeRndIt, ScoreRndIt, EdgeRndOutIt >:

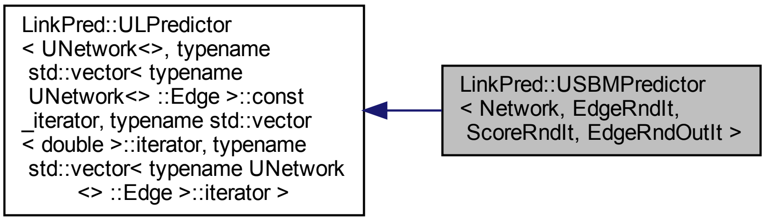

Public Member Functions

- USBMPredictor (std::shared_ptr< Network const > net, long int seed)
- USBMPredictor (USBMPredictor const &that)=delete
- USBMPredictor & operator= (USBMPredictor const &that)=delete
- USBMPredictor (USBMPredictor &&that)=delete
- USBMPredictor & operator= (USBMPredictor &&that)=delete
- virtual void init ()
- virtual void learn ()
- virtual double score (Edge const &e)
- virtual void predict (EdgeRndIt begin, EdgeRndIt end, ScoreRndIt scores)
- std::size_t getMaxIter () const
- void setMaxIter (std::size_t maxIter)
- virtual ∼USBMPredictor ()

Additional Inherited Members

9.137.1 Detailed Description

template<typename Network = UNetwork<>, typename EdgeRndIt = typename std::vector<typename Network::Edge>↩ ::const_iterator, typename ScoreRndIt = typename std::vector<double>::iterator, typename EdgeRndOutIt = typename std↩ ::vector<typename Network::Edge>::iterator>
class LinkPred::USBMPredictor< Network, EdgeRndIt, ScoreRndIt, EdgeRndOutIt >

The stochastic block model link predictor.

Template Parameters

Network	The network type.
Edge↩ RndIt	A random iterator type used to iterate on edges.
Score↩ RndIt	A random iterator type used to iterate on scores.

9.137.2 Constructor & Destructor Documentation

9.137.2.1 USBMPredictor() [1/3]

```
template<typename Network = UNetwork<>, typename EdgeRndIt = typename std::vector<typename
Network::Edge>::const_iterator, typename ScoreRndIt = typename std::vector<double>::iterator,
typename EdgeRndOutIt = typename std::vector<typename Network::Edge>::iterator>
LinkPred::USBMPredictor< Network, EdgeRndIt, ScoreRndIt, EdgeRndOutIt >::USBMPredictor (
            std::shared_ptr< Network const > net,
            long int seed )  [inline]
```

Parameters

net	The network.
seed	The random number generator's seed.

9.137.2.2 USBMPredictor() [2/3]

```
template<typename Network = UNetwork<>, typename EdgeRndIt = typename std::vector<typename
Network::Edge>::const_iterator, typename ScoreRndIt = typename std::vector<double>::iterator,
typename EdgeRndOutIt = typename std::vector<typename Network::Edge>::iterator>
LinkPred::USBMPredictor< Network, EdgeRndIt, ScoreRndIt, EdgeRndOutIt >::USBMPredictor (
            USBMPredictor< Network, EdgeRndIt, ScoreRndIt, EdgeRndOutIt > const & that )
[delete]
```

Copy constructor.

Parameters

that	The object to copy.

9.137.2.3 USBMPredictor() [3/3]

```
template<typename Network = UNetwork<>, typename EdgeRndIt = typename std::vector<typename
Network::Edge>::const_iterator, typename ScoreRndIt = typename std::vector<double>::iterator,
typename EdgeRndOutIt = typename std::vector<typename Network::Edge>::iterator>
LinkPred::USBMPredictor< Network, EdgeRndIt, ScoreRndIt, EdgeRndOutIt >::USBMPredictor (
            USBMPredictor< Network, EdgeRndIt, ScoreRndIt, EdgeRndOutIt > && that ) [delete]
```

Move constructor.

Parameters

that	The object to move.

9.137.2.4 ∼USBMPredictor()

```
template<typename Network = UNetwork<>, typename EdgeRndIt = typename std::vector<typename
Network::Edge>::const_iterator, typename ScoreRndIt = typename std::vector<double>::iterator,
typename EdgeRndOutIt = typename std::vector<typename Network::Edge>::iterator>
virtual LinkPred::USBMPredictor< Network, EdgeRndIt, ScoreRndIt, EdgeRndOutIt >::∼USBMPredictor
( ) [virtual]
```

Destructor.

9.137.3 Member Function Documentation

9.137.3.1 getMaxIter()

```
template<typename Network = UNetwork<>, typename EdgeRndIt = typename std::vector<typename
Network::Edge>::const_iterator, typename ScoreRndIt = typename std::vector<double>::iterator,
typename EdgeRndOutIt = typename std::vector<typename Network::Edge>::iterator>
std::size_t LinkPred::USBMPredictor< Network, EdgeRndIt, ScoreRndIt, EdgeRndOutIt >::getMax←
Iter ( ) const [inline]
```

Returns

The maximum number of iterations.

9.137.3.2 init()

```
template<typename Network = UNetwork<>, typename EdgeRndIt = typename std::vector<typename
Network::Edge>::const_iterator, typename ScoreRndIt = typename std::vector<double>::iterator,
typename EdgeRndOutIt = typename std::vector<typename Network::Edge>::iterator>
virtual void LinkPred::USBMPredictor< Network, EdgeRndIt, ScoreRndIt, EdgeRndOutIt >::init ( )
[virtual]
```

Initialize the solver.

Implements LinkPred::ULPredictor< UNetwork<>, typename std::vector< typename UNetwork<> ::Edge >::const_iterator, typena

9.137.3.3 learn()

```
template<typename Network = UNetwork<>, typename EdgeRndIt = typename std::vector<typename
Network::Edge>::const_iterator, typename ScoreRndIt = typename std::vector<double>::iterator,
typename EdgeRndOutIt = typename std::vector<typename Network::Edge>::iterator>
virtual void LinkPred::USBMPredictor< Network, EdgeRndIt, ScoreRndIt, EdgeRndOutIt >::learn (
) [virtual]
```

Learning.

Implements LinkPred::ULPredictor< UNetwork<>, typename std::vector< typename UNetwork<> ::Edge >::const_iterator, typena

9.137.3.4 operator=() [1/2]

```
template<typename Network = UNetwork<>, typename EdgeRndIt = typename std::vector<typename
Network::Edge>::const_iterator, typename ScoreRndIt = typename std::vector<double>::iterator,
typename EdgeRndOutIt = typename std::vector<typename Network::Edge>::iterator>
USBMPredictor& LinkPred::USBMPredictor< Network, EdgeRndIt, ScoreRndIt, EdgeRndOutIt >::operator=
(
            USBMPredictor< Network, EdgeRndIt, ScoreRndIt, EdgeRndOutIt > && that )  [delete]
```

Move assignment operator.

Parameters

that	The object to move.

9.137.3.5 operator=() [2/2]

```
template<typename Network = UNetwork<>, typename EdgeRndIt = typename std::vector<typename
Network::Edge>::const_iterator, typename ScoreRndIt = typename std::vector<double>::iterator,
typename EdgeRndOutIt = typename std::vector<typename Network::Edge>::iterator>
```

```
USBMPredictor& LinkPred::USBMPredictor< Network, EdgeRndIt, ScoreRndIt, EdgeRndOutIt >::operator=
(
            USBMPredictor< Network, EdgeRndIt, ScoreRndIt, EdgeRndOutIt > const & that )
[delete]
```

Copy assignment operator.

Parameters

that	The object to copy.

9.137.3.6 predict()

```
template<typename Network = UNetwork<>, typename EdgeRndIt = typename std::vector<typename
Network::Edge>::const_iterator, typename ScoreRndIt = typename std::vector<double>::iterator,
typename EdgeRndOutIt = typename std::vector<typename Network::Edge>::iterator>
virtual void LinkPred::USBMPredictor< Network, EdgeRndIt, ScoreRndIt, EdgeRndOutIt >::predict
(
            EdgeRndIt begin,
            EdgeRndIt end,
            ScoreRndIt scores )  [virtual]
```

Predict the links.

Parameters

begin	Beginning of the links to be predicted.
end	end of the links to be predicted.
scores	Beginning of scores.

Reimplemented from LinkPred::ULPredictor< UNetwork<>, typename std::vector< typename UNetwork<> ::Edge >::const_iterat

9.137.3.7 score()

```
template<typename Network = UNetwork<>, typename EdgeRndIt = typename std::vector<typename
Network::Edge>::const_iterator, typename ScoreRndIt = typename std::vector<double>::iterator,
typename EdgeRndOutIt = typename std::vector<typename Network::Edge>::iterator>
virtual double LinkPred::USBMPredictor< Network, EdgeRndIt, ScoreRndIt, EdgeRndOutIt >::score
(
            Edge const & e )  [inline], [virtual]
```

Compute the score of a single edge.

Parameters

e	The edge.

Returns

The score of e.

9.137.3.8 setMaxIter()

```
template<typename Network = UNetwork<>, typename EdgeRndIt = typename std::vector<typename
Network::Edge>::const_iterator, typename ScoreRndIt = typename std::vector<double>::iterator,
typename EdgeRndOutIt = typename std::vector<typename Network::Edge>::iterator>
void LinkPred::USBMPredictor< Network, EdgeRndIt, ScoreRndIt, EdgeRndOutIt >::setMaxIter (
            std::size_t maxIter )  [inline]
```

Set the maximum number of iterations.

Parameters

maxIter	The new maximum number of iterations.

The documentation for this class was generated from the following file:

- include/linkpred/predictors/undirected/usbmpredictor.hpp

9.138 LinkPred::USHPPredictor< Network, EdgeRndIt, ScoreRndIt, EdgeRndOutIt > Class Template Reference

A shortest path link predictor link predictor.

```
#include <ushppredictor.hpp>
```

Inheritance diagram for LinkPred::USHPPredictor< Network, EdgeRndIt, ScoreRndIt, EdgeRndOutIt >:

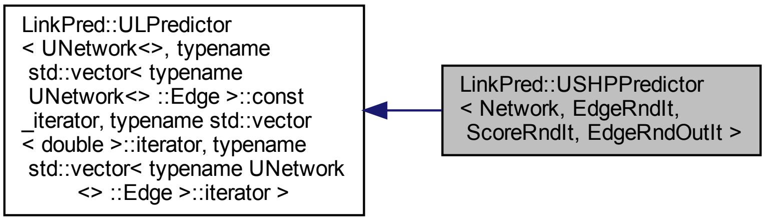

Collaboration diagram for LinkPred::USHPPredictor< Network, EdgeRndIt, ScoreRndIt, EdgeRndOutIt >:

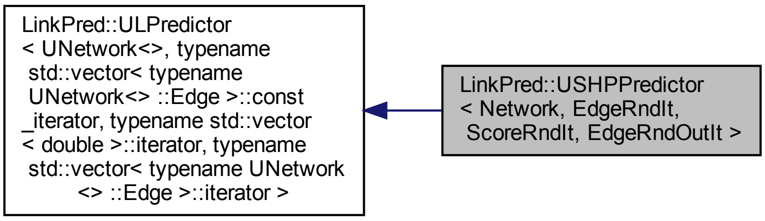

Public Types

- enum LandmarkStrategy { Random, Hub, IHub }

  An enumeration of the different landmark positioning strategies.

Public Member Functions

- USHPPredictor (std::shared_ptr< Network const > net, long int seed)
- USHPPredictor (USHPPredictor const &that)=default
- USHPPredictor & operator= (USHPPredictor const &that)=default
- USHPPredictor (USHPPredictor &&that)=default
- USHPPredictor & operator= (USHPPredictor &&that)=default
- virtual void init ()
- virtual void learn ()
- virtual void predict (EdgeRndIt begin, EdgeRndIt end, ScoreRndIt scores)
- virtual double score (Edge const &e)
- bool getAsp () const
- void setAsp (bool asp)
- double getLandmarkRatio () const
- void setLandmarkRatio (double landmarkRatio)
- LandmarkStrategy getLandmarkStrategy () const
- void setLandmarkStrategy (LandmarkStrategy landmarkStrategy)
- CacheLevel getCacheLevel () const
- void setCacheLevel (CacheLevel cacheLevel)
- virtual ∼USHPPredictor ()=default

9.138.1 Detailed Description

template<typename Network = UNetwork<>, typename EdgeRndIt = typename std::vector<typename Network::Edge>↵ ::const_iterator, typename ScoreRndIt = typename std::vector<double>::iterator, typename EdgeRndOutIt = typename std↵ ::vector<typename Network::Edge>::iterator>
class LinkPred::USHPPredictor< Network, EdgeRndIt, ScoreRndIt, EdgeRndOutIt >

A shortest path link predictor link predictor.

Template Parameters

Network	The network type.
Edge↩ RndIt	A random iterator type used to iterate on edges.
Score↩ RndIt	A random iterator type used to iterate on scores.

9.138.2 Member Enumeration Documentation

9.138.2.1 LandmarkStrategy

```
template<typename Network = UNetwork<>, typename EdgeRndIt = typename std::vector<typename
Network::Edge>::const_iterator, typename ScoreRndIt = typename std::vector<double>::iterator,
typename EdgeRndOutIt = typename std::vector<typename Network::Edge>::iterator>
enum LinkPred::USHPPredictor::LandmarkStrategy
```

An enumeration of the different landmark positioning strategies.

Enumerator

Random	Landmarks are chosen randomly.
Hub	The nodes with the highest degree are chosen.
IHub	The nodes with the lowest degree are chosen.

9.138.3 Constructor & Destructor Documentation

9.138.3.1 USHPPredictor() [1/3]

```
template<typename Network = UNetwork<>, typename EdgeRndIt = typename std::vector<typename
Network::Edge>::const_iterator, typename ScoreRndIt = typename std::vector<double>::iterator,
typename EdgeRndOutIt = typename std::vector<typename Network::Edge>::iterator>
LinkPred::USHPPredictor< Network, EdgeRndIt, ScoreRndIt, EdgeRndOutIt >::USHPPredictor (
            std::shared_ptr< Network const > net,
            long int seed )  [inline]
```

Parameters

net	The network.
seed	The random number generator's seed.

9.138.3.2 USHPPredictor() [2/3]

```
template<typename Network = UNetwork<>, typename EdgeRndIt = typename std::vector<typename
Network::Edge>::const_iterator, typename ScoreRndIt = typename std::vector<double>::iterator,
typename EdgeRndOutIt = typename std::vector<typename Network::Edge>::iterator>
LinkPred::USHPPredictor< Network, EdgeRndIt, ScoreRndIt, EdgeRndOutIt >::USHPPredictor (
            USHPPredictor< Network, EdgeRndIt, ScoreRndIt, EdgeRndOutIt > const & that )
[default]
```

Copy constructor.

Parameters

that	The object to copy.

9.138.3.3 USHPPredictor() [3/3]

```
template<typename Network = UNetwork<>, typename EdgeRndIt = typename std::vector<typename
Network::Edge>::const_iterator, typename ScoreRndIt = typename std::vector<double>::iterator,
typename EdgeRndOutIt = typename std::vector<typename Network::Edge>::iterator>
LinkPred::USHPPredictor< Network, EdgeRndIt, ScoreRndIt, EdgeRndOutIt >::USHPPredictor (
            USHPPredictor< Network, EdgeRndIt, ScoreRndIt, EdgeRndOutIt > && that )  [default]
```

Move constructor.

Parameters

that	The object to move.

9.138.3.4 ∼USHPPredictor()

```
template<typename Network = UNetwork<>, typename EdgeRndIt = typename std::vector<typename
Network::Edge>::const_iterator, typename ScoreRndIt = typename std::vector<double>::iterator,
typename EdgeRndOutIt = typename std::vector<typename Network::Edge>::iterator>
virtual LinkPred::USHPPredictor< Network, EdgeRndIt, ScoreRndIt, EdgeRndOutIt >::~USHPPredictor
( )  [virtual], [default]
```

Destructor.

9.138.4 Member Function Documentation

9.138.4.1 getAsp()

```
template<typename Network = UNetwork<>, typename EdgeRndIt = typename std::vector<typename
Network::Edge>::const_iterator, typename ScoreRndIt = typename std::vector<double>::iterator,
typename EdgeRndOutIt = typename std::vector<typename Network::Edge>::iterator>
bool LinkPred::USHPPredictor< Network, EdgeRndIt, ScoreRndIt, EdgeRndOutIt >::getAsp ( ) const
[inline]
```

Returns

Whether approximate shortest path distances are used.

9.138.4.2 getCacheLevel()

```
template<typename Network = UNetwork<>, typename EdgeRndIt = typename std::vector<typename
Network::Edge>::const_iterator, typename ScoreRndIt = typename std::vector<double>::iterator,
typename EdgeRndOutIt = typename std::vector<typename Network::Edge>::iterator>
CacheLevel LinkPred::USHPPredictor< Network, EdgeRndIt, ScoreRndIt, EdgeRndOutIt >::getCache←
Level ( ) const [inline]
```

Returns

The distances cache level.

9.138.4.3 getLandmarkRatio()

```
template<typename Network = UNetwork<>, typename EdgeRndIt = typename std::vector<typename
Network::Edge>::const_iterator, typename ScoreRndIt = typename std::vector<double>::iterator,
typename EdgeRndOutIt = typename std::vector<typename Network::Edge>::iterator>
double LinkPred::USHPPredictor< Network, EdgeRndIt, ScoreRndIt, EdgeRndOutIt >::getLandmark←
Ratio ( ) const [inline]
```

Returns

The landmark ratio.

9.138.4.4 getLandmarkStrategy()

```
template<typename Network = UNetwork<>, typename EdgeRndIt = typename std::vector<typename
Network::Edge>::const_iterator, typename ScoreRndIt = typename std::vector<double>::iterator,
typename EdgeRndOutIt = typename std::vector<typename Network::Edge>::iterator>
LandmarkStrategy LinkPred::USHPPredictor< Network, EdgeRndIt, ScoreRndIt, EdgeRndOutIt >←
::getLandmarkStrategy ( ) const [inline]
```

Returns

The landmark strategy.

9.138.4.5 init()

```
template<typename Network = UNetwork<>, typename EdgeRndIt = typename std::vector<typename
Network::Edge>::const_iterator, typename ScoreRndIt = typename std::vector<double>::iterator,
typename EdgeRndOutIt = typename std::vector<typename Network::Edge>::iterator>
virtual void LinkPred::USHPPredictor< Network, EdgeRndIt, ScoreRndIt, EdgeRndOutIt >::init ( )
[virtual]
```

Initialize the solver.

Implements LinkPred::ULPredictor< UNetwork<>, typename std::vector< typename UNetwork<> ::Edge >::const_iterator, typena

9.138.4.6 learn()

```
template<typename Network = UNetwork<>, typename EdgeRndIt = typename std::vector<typename
Network::Edge>::const_iterator, typename ScoreRndIt = typename std::vector<double>::iterator,
typename EdgeRndOutIt = typename std::vector<typename Network::Edge>::iterator>
virtual void LinkPred::USHPPredictor< Network, EdgeRndIt, ScoreRndIt, EdgeRndOutIt >::learn (
) [virtual]
```

Learn.

Implements LinkPred::ULPredictor< UNetwork<>, typename std::vector< typename UNetwork<> ::Edge >::const_iterator, typena

9.138.4.7 operator=() [1/2]

```
template<typename Network = UNetwork<>, typename EdgeRndIt = typename std::vector<typename
Network::Edge>::const_iterator, typename ScoreRndIt = typename std::vector<double>::iterator,
typename EdgeRndOutIt = typename std::vector<typename Network::Edge>::iterator>
USHPPredictor& LinkPred::USHPPredictor< Network, EdgeRndIt, ScoreRndIt, EdgeRndOutIt >::operator=
(
            USHPPredictor< Network, EdgeRndIt, ScoreRndIt, EdgeRndOutIt > && that ) [default]
```

Move assignment operator.

Parameters

that	The object to move.

9.138.4.8 operator=() [2/2]

```
template<typename Network = UNetwork<>, typename EdgeRndIt = typename std::vector<typename
Network::Edge>::const_iterator, typename ScoreRndIt = typename std::vector<double>::iterator,
typename EdgeRndOutIt = typename std::vector<typename Network::Edge>::iterator>
```

```
USHPPredictor& LinkPred::USHPPredictor< Network, EdgeRndIt, ScoreRndIt, EdgeRndOutIt >::operator=
(
            USHPPredictor< Network, EdgeRndIt, ScoreRndIt, EdgeRndOutIt > const & that )
[default]
```

Copy assignment operator.

Parameters

that	The object to copy.

9.138.4.9 predict()

```
template<typename Network = UNetwork<>, typename EdgeRndIt = typename std::vector<typename
Network::Edge>::const_iterator, typename ScoreRndIt = typename std::vector<double>::iterator,
typename EdgeRndOutIt = typename std::vector<typename Network::Edge>::iterator>
virtual void LinkPred::USHPPredictor< Network, EdgeRndIt, ScoreRndIt, EdgeRndOutIt >::predict
(
            EdgeRndIt begin,
            EdgeRndIt end,
            ScoreRndIt scores )  [virtual]
```

Predict the links.

Parameters

begin	Beginning of the links to be predicted.
end	end of the links to be predicted.
scores	Beginning of scores.

Reimplemented from LinkPred::ULPredictor< UNetwork<>, typename std::vector< typename UNetwork<> ::Edge >::const_iterat[illegible]

9.138.4.10 score()

```
template<typename Network = UNetwork<>, typename EdgeRndIt = typename std::vector<typename
Network::Edge>::const_iterator, typename ScoreRndIt = typename std::vector<double>::iterator,
typename EdgeRndOutIt = typename std::vector<typename Network::Edge>::iterator>
virtual double LinkPred::USHPPredictor< Network, EdgeRndIt, ScoreRndIt, EdgeRndOutIt >::score
(
            Edge const & e )  [virtual]
```

Compute the score of a single edge.

Parameters

e	The edge.

Returns

The score of e.

9.138.4.11 setAsp()

```
template<typename Network = UNetwork<>, typename EdgeRndIt = typename std::vector<typename
Network::Edge>::const_iterator, typename ScoreRndIt = typename std::vector<double>::iterator,
typename EdgeRndOutIt = typename std::vector<typename Network::Edge>::iterator>
void LinkPred::USHPPredictor< Network, EdgeRndIt, ScoreRndIt, EdgeRndOutIt >::setAsp (
            bool asp ) [inline]
```

Set Whether approximate shortest path distances are used.

Parameters

asp	Whether approximate shortest path distances should be used.

9.138.4.12 setCacheLevel()

```
template<typename Network = UNetwork<>, typename EdgeRndIt = typename std::vector<typename
Network::Edge>::const_iterator, typename ScoreRndIt = typename std::vector<double>::iterator,
typename EdgeRndOutIt = typename std::vector<typename Network::Edge>::iterator>
void LinkPred::USHPPredictor< Network, EdgeRndIt, ScoreRndIt, EdgeRndOutIt >::setCacheLevel (
            CacheLevel cacheLevel ) [inline]
```

Set the distances cache level.

Parameters

cacheLevel	The new distances cache level.

9.138.4.13 setLandmarkRatio()

```
template<typename Network = UNetwork<>, typename EdgeRndIt = typename std::vector<typename
Network::Edge>::const_iterator, typename ScoreRndIt = typename std::vector<double>::iterator,
typename EdgeRndOutIt = typename std::vector<typename Network::Edge>::iterator>
void LinkPred::USHPPredictor< Network, EdgeRndIt, ScoreRndIt, EdgeRndOutIt >::setLandmarkRatio
(
            double landmarkRatio ) [inline]
```

Set the landmark ratio.

Parameters

landmarkRatio	The new landmark ratio.

9.138.4.14 setLandmarkStrategy()

```
template<typename Network = UNetwork<>, typename EdgeRndIt = typename std::vector<typename
Network::Edge>::const_iterator, typename ScoreRndIt = typename std::vector<double>::iterator,
typename EdgeRndOutIt = typename std::vector<typename Network::Edge>::iterator>
void LinkPred::USHPPredictor< Network, EdgeRndIt, ScoreRndIt, EdgeRndOutIt >::setLandmark↵
Strategy (
            LandmarkStrategy landmarkStrategy )  [inline]
```

Set the landmark strategy.

Parameters

landmarkStrategy	The new landmark strategy.

The documentation for this class was generated from the following file:

- include/linkpred/predictors/undirected/ushppredictor.hpp

9.139 LinkPred::USOIPredictor< Network, EdgeRndIt, ScoreRndIt, EdgeRndOutIt > Class Template Reference

A Sorensen index link predictor.

```
#include <usoipredictor.hpp>
```

Inheritance diagram for LinkPred::USOIPredictor< Network, EdgeRndIt, ScoreRndIt, EdgeRndOutIt >:

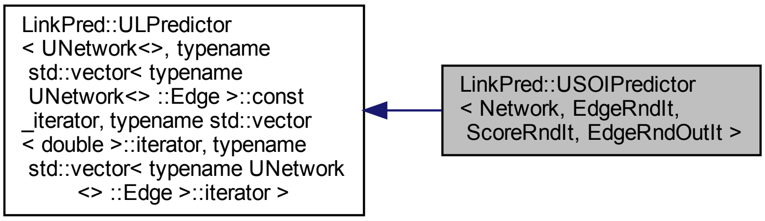

Collaboration diagram for LinkPred::USOIPredictor< Network, EdgeRndIt, ScoreRndIt, EdgeRndOutIt >:

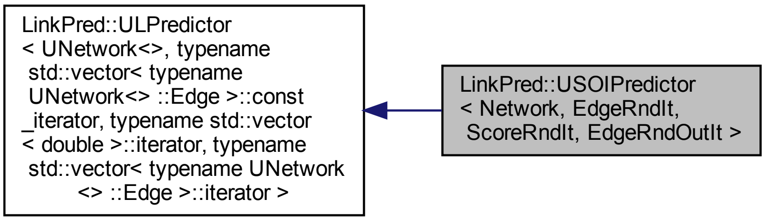

Public Member Functions

- USOIPredictor (std::shared_ptr< Network const > net)
- USOIPredictor (USOIPredictor const &that)=default
- USOIPredictor & operator= (USOIPredictor const &that)=default
- USOIPredictor (USOIPredictor &&that)=default
- USOIPredictor & operator= (USOIPredictor &&that)=default
- virtual void init ()
- virtual void learn ()
- virtual double score (Edge const &e)
- virtual void predict (EdgeRndIt begin, EdgeRndIt end, ScoreRndIt scores)
- virtual std::size_t top (std::size_t k, EdgeRndOutIt eit, ScoreRndIt sit)
- virtual ~USOIPredictor ()=default

Additional Inherited Members

9.139.1 Detailed Description

template<typename Network = UNetwork<>, typename EdgeRndIt = typename std::vector<typename Network::Edge>↩ ::const_iterator, typename ScoreRndIt = typename std::vector<double>::iterator, typename EdgeRndOutIt = typename std↩ ::vector<typename Network::Edge>::iterator>
class LinkPred::USOIPredictor< Network, EdgeRndIt, ScoreRndIt, EdgeRndOutIt >

A Sorensen index link predictor.

Template Parameters

Network	The network type.
Edge↩ RndIt	A random iterator type used to iterate on edges.
Score↩ RndIt	A random iterator type used to iterate on scores.

9.139.2 Constructor & Destructor Documentation

9.139.2.1 USOIPredictor() [1/3]

```
template<typename Network = UNetwork<>, typename EdgeRndIt = typename std::vector<typename
Network::Edge>::const_iterator, typename ScoreRndIt = typename std::vector<double>::iterator,
typename EdgeRndOutIt = typename std::vector<typename Network::Edge>::iterator>
LinkPred::USOIPredictor< Network, EdgeRndIt, ScoreRndIt, EdgeRndOutIt >::USOIPredictor (
            std::shared_ptr< Network const > net ) [inline]
```

Parameters

net	The network.

9.139.2.2 USOIPredictor() [2/3]

```
template<typename Network = UNetwork<>, typename EdgeRndIt = typename std::vector<typename
Network::Edge>::const_iterator, typename ScoreRndIt = typename std::vector<double>::iterator,
typename EdgeRndOutIt = typename std::vector<typename Network::Edge>::iterator>
LinkPred::USOIPredictor< Network, EdgeRndIt, ScoreRndIt, EdgeRndOutIt >::USOIPredictor (
            USOIPredictor< Network, EdgeRndIt, ScoreRndIt, EdgeRndOutIt > const & that )
[default]
```

Copy constructor.

Parameters

that	The object to copy.

9.139.2.3 USOIPredictor() [3/3]

```
template<typename Network = UNetwork<>, typename EdgeRndIt = typename std::vector<typename
Network::Edge>::const_iterator, typename ScoreRndIt = typename std::vector<double>::iterator,
typename EdgeRndOutIt = typename std::vector<typename Network::Edge>::iterator>
LinkPred::USOIPredictor< Network, EdgeRndIt, ScoreRndIt, EdgeRndOutIt >::USOIPredictor (
            USOIPredictor< Network, EdgeRndIt, ScoreRndIt, EdgeRndOutIt > && that ) [default]
```

Move constructor.

Parameters

that	The object to move.

9.139.2.4 ∼USOIPredictor()

```
template<typename Network = UNetwork<>, typename EdgeRndIt = typename std::vector<typename
Network::Edge>::const_iterator, typename ScoreRndIt = typename std::vector<double>::iterator,
typename EdgeRndOutIt = typename std::vector<typename Network::Edge>::iterator>
virtual LinkPred::USOIPredictor< Network, EdgeRndIt, ScoreRndIt, EdgeRndOutIt >::∼USOIPredictor
( ) [virtual], [default]
```

Destructor.

9.139.3 Member Function Documentation

9.139.3.1 init()

```
template<typename Network = UNetwork<>, typename EdgeRndIt = typename std::vector<typename
Network::Edge>::const_iterator, typename ScoreRndIt = typename std::vector<double>::iterator,
typename EdgeRndOutIt = typename std::vector<typename Network::Edge>::iterator>
virtual void LinkPred::USOIPredictor< Network, EdgeRndIt, ScoreRndIt, EdgeRndOutIt >::init ( )
[inline], [virtual]
```

Initialize the solver.

Implements LinkPred::ULPredictor< UNetwork<>, typename std::vector< typename UNetwork<> ::Edge >::const_iterator, typena

9.139.3.2 learn()

```
template<typename Network = UNetwork<>, typename EdgeRndIt = typename std::vector<typename
Network::Edge>::const_iterator, typename ScoreRndIt = typename std::vector<double>::iterator,
typename EdgeRndOutIt = typename std::vector<typename Network::Edge>::iterator>
virtual void LinkPred::USOIPredictor< Network, EdgeRndIt, ScoreRndIt, EdgeRndOutIt >::learn (
) [inline], [virtual]
```

Solve the link prediction problem.

Implements LinkPred::ULPredictor< UNetwork<>, typename std::vector< typename UNetwork<> ::Edge >::const_iterator, typena

9.139.3.3 operator=() [1/2]

```
template<typename Network = UNetwork<>, typename EdgeRndIt = typename std::vector<typename
Network::Edge>::const_iterator, typename ScoreRndIt = typename std::vector<double>::iterator,
typename EdgeRndOutIt = typename std::vector<typename Network::Edge>::iterator>
USOIPredictor& LinkPred::USOIPredictor< Network, EdgeRndIt, ScoreRndIt, EdgeRndOutIt >::operator=
(
            USOIPredictor< Network, EdgeRndIt, ScoreRndIt, EdgeRndOutIt > && that ) [default]
```

Move assignment operator.

Parameters

that	The object to move.

9.139.3.4 operator=() [2/2]

```
template<typename Network = UNetwork<>, typename EdgeRndIt = typename std::vector<typename
Network::Edge>::const_iterator, typename ScoreRndIt = typename std::vector<double>::iterator,
typename EdgeRndOutIt = typename std::vector<typename Network::Edge>::iterator>
USOIPredictor& LinkPred::USOIPredictor< Network, EdgeRndIt, ScoreRndIt, EdgeRndOutIt >::operator=
(
            USOIPredictor< Network, EdgeRndIt, ScoreRndIt, EdgeRndOutIt > const & that )
[default]
```

Copy assignment operator.

Parameters

that	The object to copy.

9.139.3.5 predict()

```
template<typename Network = UNetwork<>, typename EdgeRndIt = typename std::vector<typename
Network::Edge>::const_iterator, typename ScoreRndIt = typename std::vector<double>::iterator,
typename EdgeRndOutIt = typename std::vector<typename Network::Edge>::iterator>
virtual void LinkPred::USOIPredictor< Network, EdgeRndIt, ScoreRndIt, EdgeRndOutIt >::predict
(
            EdgeRndIt begin,
            EdgeRndIt end,
            ScoreRndIt scores )  [virtual]
```

Predict the links.

Parameters

begin	Beginning of the links to be predicted.
end	end of the links to be predicted.
scores	Beginning of scores.

Reimplemented from LinkPred::ULPredictor< UNetwork<>, typename std::vector< typename UNetwork<> ::Edge >::const_iterat

9.139.3.6 score()

```
template<typename Network = UNetwork<>, typename EdgeRndIt = typename std::vector<typename
Network::Edge>::const_iterator, typename ScoreRndIt = typename std::vector<double>::iterator,
```

```
typename EdgeRndOutIt = typename std::vector<typename Network::Edge>::iterator>
virtual double LinkPred::USOIPredictor< Network, EdgeRndIt, ScoreRndIt, EdgeRndOutIt >::score
(
            Edge const & e )  [inline], [virtual]
```

Compute the score of a single edge.

Parameters

e	The edge.

Returns

The score of e.

9.139.3.7 top()

```
template<typename Network = UNetwork<>, typename EdgeRndIt = typename std::vector<typename
Network::Edge>::const_iterator, typename ScoreRndIt = typename std::vector<double>::iterator,
typename EdgeRndOutIt = typename std::vector<typename Network::Edge>::iterator>
virtual std::size_t LinkPred::USOIPredictor< Network, EdgeRndIt, ScoreRndIt, EdgeRndOutIt >↩
::top (
            std::size_t k,
            EdgeRndOutIt eit,
            ScoreRndIt sit )  [virtual]
```

Finds the k negative edges with the top score. Ties are broken randmly.

Parameters

k	The number of edges to find.
eit	An output iterator where the edges are written.
sit	An output iterator where the scores are written. The scores are written in the same order as the edges.

Returns

The number of negative edges inserted. It is the minimum between k and the number of negative edges in the network.

Reimplemented from LinkPred::ULPredictor< UNetwork<>, typename std::vector< typename UNetwork<> ::Edge >::const_iterato

The documentation for this class was generated from the following file:

- include/linkpred/predictors/undirected/usoipredictor.hpp

9.140 LinkPred::USUMPredictor< Network, EdgeRndIt, ScoreRndIt, EdgeRndOutIt > Class Template Reference

A sum-of-degrees link predictor.

```
#include <usumpredictor.hpp>
```

Inheritance diagram for LinkPred::USUMPredictor< Network, EdgeRndIt, ScoreRndIt, EdgeRndOutIt >:

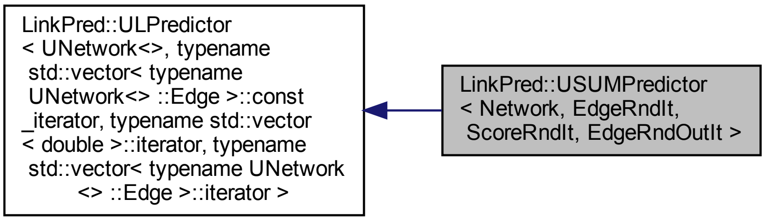

Collaboration diagram for LinkPred::USUMPredictor< Network, EdgeRndIt, ScoreRndIt, EdgeRndOutIt >:

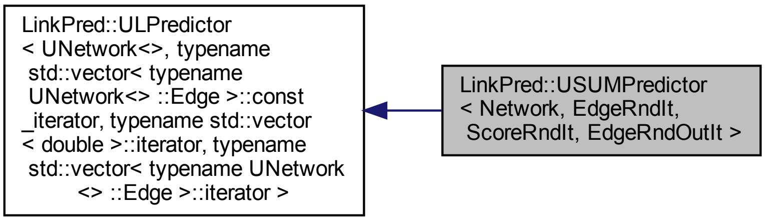

Public Member Functions

- USUMPredictor (std::shared_ptr< Network const > net)
- USUMPredictor (USUMPredictor const &that)=default
- USUMPredictor & operator= (USUMPredictor const &that)=default
- USUMPredictor (USUMPredictor &&that)=default
- USUMPredictor & operator= (USUMPredictor &&that)=default
- virtual void init ()
- virtual void learn ()
- virtual void predict (EdgeRndIt begin, EdgeRndIt end, ScoreRndIt scores)
- virtual double score (Edge const &e)
- virtual std::size_t top (std::size_t k, EdgeRndOutIt eit, ScoreRndIt sit)
- virtual ~USUMPredictor ()=default

Additional Inherited Members

9.140.1 Detailed Description

template<typename Network = UNetwork<>, typename EdgeRndIt = typename std::vector<typename Network::Edge>↩ ::const_iterator, typename ScoreRndIt = typename std::vector<double>::iterator, typename EdgeRndOutIt = typename std↩ ::vector<typename Network::Edge>::iterator>
class LinkPred::USUMPredictor< Network, EdgeRndIt, ScoreRndIt, EdgeRndOutIt >

A sum-of-degrees link predictor.

Template Parameters

Network	The network type.
Edge↩ RndIt	A random iterator type used to iterate on edges.
Score↩ RndIt	A random iterator type used to iterate on scores.

9.140.2 Constructor & Destructor Documentation

9.140.2.1 USUMPredictor() [1/3]

```
template<typename Network = UNetwork<>, typename EdgeRndIt = typename std::vector<typename
Network::Edge>::const_iterator, typename ScoreRndIt = typename std::vector<double>::iterator,
typename EdgeRndOutIt = typename std::vector<typename Network::Edge>::iterator>
LinkPred::USUMPredictor< Network, EdgeRndIt, ScoreRndIt, EdgeRndOutIt >::USUMPredictor (
            std::shared_ptr< Network const > net )  [inline]
```

Parameters

net	The network.

9.140.2.2 USUMPredictor() [2/3]

```
template<typename Network = UNetwork<>, typename EdgeRndIt = typename std::vector<typename
Network::Edge>::const_iterator, typename ScoreRndIt = typename std::vector<double>::iterator,
typename EdgeRndOutIt = typename std::vector<typename Network::Edge>::iterator>
LinkPred::USUMPredictor< Network, EdgeRndIt, ScoreRndIt, EdgeRndOutIt >::USUMPredictor (
            USUMPredictor< Network, EdgeRndIt, ScoreRndIt, EdgeRndOutIt > const & that )
[default]
```

Copy constructor.

Parameters

that	The object to copy.

9.140.2.3 USUMPredictor() [3/3]

```
template<typename Network = UNetwork<>, typename EdgeRndIt = typename std::vector<typename
Network::Edge>::const_iterator, typename ScoreRndIt = typename std::vector<double>::iterator,
typename EdgeRndOutIt = typename std::vector<typename Network::Edge>::iterator>
LinkPred::USUMPredictor< Network, EdgeRndIt, ScoreRndIt, EdgeRndOutIt >::USUMPredictor (
            USUMPredictor< Network, EdgeRndIt, ScoreRndIt, EdgeRndOutIt > && that )  [default]
```

Move constructor.

Parameters

that	The object to move.

9.140.2.4 ∼USUMPredictor()

```
template<typename Network = UNetwork<>, typename EdgeRndIt = typename std::vector<typename
Network::Edge>::const_iterator, typename ScoreRndIt = typename std::vector<double>::iterator,
typename EdgeRndOutIt = typename std::vector<typename Network::Edge>::iterator>
virtual LinkPred::USUMPredictor< Network, EdgeRndIt, ScoreRndIt, EdgeRndOutIt >::~USUMPredictor
( )  [virtual], [default]
```

Destructor.

9.140.3 Member Function Documentation

9.140.3.1 init()

```
template<typename Network = UNetwork<>, typename EdgeRndIt = typename std::vector<typename
Network::Edge>::const_iterator, typename ScoreRndIt = typename std::vector<double>::iterator,
typename EdgeRndOutIt = typename std::vector<typename Network::Edge>::iterator>
virtual void LinkPred::USUMPredictor< Network, EdgeRndIt, ScoreRndIt, EdgeRndOutIt >::init ( )
[inline], [virtual]
```

Initialize the predictor.

Implements LinkPred::ULPredictor< UNetwork<>, typename std::vector< typename UNetwork<> ::Edge >::const_iterator, typena

9.140.3.2 learn()

```
template<typename Network = UNetwork<>, typename EdgeRndIt = typename std::vector<typename
Network::Edge>::const_iterator, typename ScoreRndIt = typename std::vector<double>::iterator,
typename EdgeRndOutIt = typename std::vector<typename Network::Edge>::iterator>
virtual void LinkPred::USUMPredictor< Network, EdgeRndIt, ScoreRndIt, EdgeRndOutIt >::learn (
) [inline], [virtual]
```

Learning.

Implements LinkPred::ULPredictor< UNetwork<>, typename std::vector< typename UNetwork<> ::Edge >::const_iterator, typena

9.140.3.3 operator=() [1/2]

```
template<typename Network = UNetwork<>, typename EdgeRndIt = typename std::vector<typename
Network::Edge>::const_iterator, typename ScoreRndIt = typename std::vector<double>::iterator,
typename EdgeRndOutIt = typename std::vector<typename Network::Edge>::iterator>
USUMPredictor& LinkPred::USUMPredictor< Network, EdgeRndIt, ScoreRndIt, EdgeRndOutIt >::operator=
(
            USUMPredictor< Network, EdgeRndIt, ScoreRndIt, EdgeRndOutIt > && that ) [default]
```

Move assignment operator.

Parameters

that	The object to move.

9.140.3.4 operator=() [2/2]

```
template<typename Network = UNetwork<>, typename EdgeRndIt = typename std::vector<typename
Network::Edge>::const_iterator, typename ScoreRndIt = typename std::vector<double>::iterator,
typename EdgeRndOutIt = typename std::vector<typename Network::Edge>::iterator>
USUMPredictor& LinkPred::USUMPredictor< Network, EdgeRndIt, ScoreRndIt, EdgeRndOutIt >::operator=
(
            USUMPredictor< Network, EdgeRndIt, ScoreRndIt, EdgeRndOutIt > const & that )
[default]
```

Copy assignment operator.

Parameters

that	The object to copy.

9.140.3.5 predict()

```
template<typename Network = UNetwork<>, typename EdgeRndIt = typename std::vector<typename
Network::Edge>::const_iterator, typename ScoreRndIt = typename std::vector<double>::iterator,
typename EdgeRndOutIt = typename std::vector<typename Network::Edge>::iterator>
virtual void LinkPred::USUMPredictor< Network, EdgeRndIt, ScoreRndIt, EdgeRndOutIt >::predict
(
            EdgeRndIt begin,
            EdgeRndIt end,
            ScoreRndIt scores )  [virtual]
```

Predict the links.

Parameters

begin	Beginning of the links to be predicted.
end	end of the links to be predicted.
scores	Beginning of scores.

Reimplemented from LinkPred::ULPredictor< UNetwork<>, typename std::vector< typename UNetwork<> ::Edge >::const_iterat

9.140.3.6 score()

```
template<typename Network = UNetwork<>, typename EdgeRndIt = typename std::vector<typename
Network::Edge>::const_iterator, typename ScoreRndIt = typename std::vector<double>::iterator,
typename EdgeRndOutIt = typename std::vector<typename Network::Edge>::iterator>
virtual double LinkPred::USUMPredictor< Network, EdgeRndIt, ScoreRndIt, EdgeRndOutIt >::score
(
            Edge const & e )  [virtual]
```

Compute the score of a single edge.

Parameters

e	The edge.

Returns

The score of e.

9.140.3.7 top()

```
template<typename Network = UNetwork<>, typename EdgeRndIt = typename std::vector<typename
Network::Edge>::const_iterator, typename ScoreRndIt = typename std::vector<double>::iterator,
typename EdgeRndOutIt = typename std::vector<typename Network::Edge>::iterator>
```

```
virtual std::size_t LinkPred::USUMPredictor< Network, EdgeRndIt, ScoreRndIt, EdgeRndOutIt >↩
::top (
            std::size_t k,
            EdgeRndOutIt eit,
            ScoreRndIt sit )  [inline], [virtual]
```

Finds the k negative edges with the top score. Ties are broken randomly.

Parameters

k	The number of edges to find.
eit	An output iterator where the edges are written.
sit	An output iterator where the scores are written. The scores are written in the same order as the edges.

Returns

The number of negative edges inserted. It is the minimum between k and the number of negative edges in the network.

Reimplemented from LinkPred::ULPredictor< UNetwork<>, typename std::vector< typename UNetwork<> ::Edge >::const_iterat[illegible]

The documentation for this class was generated from the following file:

- include/linkpred/predictors/undirected/usumpredictor.hpp

9.141 LinkPred::Vec Class Reference

This class represent a vector (in the sense of linear algebra).

```
#include <vec.hpp>
```

Public Member Functions

- Vec ()
- Vec (int n)
- Vec (std::initializer_list< double > l)
- Vec (int n, double ∗vals)
- Vec (Vec const &v, std::shared_ptr< std::vector< int >> ind)
- Vec (Vec const &v1, Vec const &v2)
- Vec (Vec const &that)
- Vec & operator= (Vec const &that)
- Vec (Vec &&that)
- Vec & operator= (Vec &&that)
- int size () const
- double ∗ getValues () const
- bool isConstant () const
- void setConstant (bool constant)
- bool makeConstant (double tol)
- void resize (int newSize)
- void resize (int newSize, double val)

- bool operator== (const Vec &other) const
- bool operator!= (const Vec &other) const
- double & operator[ ] (const int k)
- double operator[ ] (const int k) const
- std::vector< int > findNz () const
- std::vector< int > find (double val) const
- void reset ()
- void reset (double val)
- void read (std::ifstream ∗in)
- void write (std::ofstream ∗out) const
- void read (std::string fileName)
- void write (std::string fileName) const
- double norm () const
- double mean () const
- double amin () const
- double amax () const
- double min () const
- double max () const
- int imin () const
- int imax () const
- int ifmin () const
- int ifmax () const
- int ilmin () const
- int ilmax () const
- void center ()
- double norm (int lim) const
- double mean (int lim) const
- double amin (int lim) const
- double amax (int lim) const
- double min (int lim) const
- double max (int lim) const
- void center (int lim)
- void axpy (double a, Vec const &x)
- void scale (double a)
- void partScale (int start, int nb, double a)
- void scatter (int start, Vec const &v, int const ∗ind)
- double sum () const
- void print () const
- void print (std::string header) const
- void print (std::ostream &out) const
- void print (std::ostream &out, std::string header) const
- virtual ∼Vec ()

Friends

- class **GFMatrix**
- Vec operator+ (Vec const &v1, Vec const &v2)
- Vec operator- (Vec const &v1, Vec const &v2)
- Vec operator∗ (double a, Vec const &v)
- Vec operator∗ (Vec const &v1, Vec const &v2)
- Vec operator/ (Vec const &v1, Vec const &v2)
- Vec operator+ (double a, Vec const &v)
- Vec operator- (double a, Vec const &v)
- Vec operator∗ (double a, Vec const &v)

- Vec operator/ (double a, Vec const &v)
- Vec operator+ (Vec const &v, double a)
- Vec operator- (Vec const &v, double a)
- Vec operator* (Vec const &v, double a)
- Vec operator/ (Vec const &v, double a)
- double operator^ (Vec const &v1, Vec const &v2)
- GFMatrix **operator+** (const GFMatrix &mat1, const Vec &mat2)
- GFMatrix **operator+** (const Vec &mat1, const GFMatrix &mat2)
- GFMatrix **operator-** (const GFMatrix &mat1, const Vec &mat2)
- GFMatrix **operator-** (const Vec &mat1, const GFMatrix &mat2)
- Vec operator* (const GFMatrix &mat, const Vec &vec)

9.141.1 Detailed Description

This class represent a vector (in the sense of linear algebra).

9.141.2 Constructor & Destructor Documentation

9.141.2.1 Vec() [1/8]

```
LinkPred::Vec::Vec ( )
```

Default constructor.

9.141.2.2 Vec() [2/8]

```
LinkPred::Vec::Vec (
             int n )
```

Constructor.

Parameters

n	The dimension.

9.141.2.3 Vec() [3/8]

```
LinkPred::Vec::Vec (
             std::initializer_list< double > l )
```

Constructor.

Parameters

l	Initilizer list.

9.141.2.4 Vec() [4/8]

```
LinkPred::Vec::Vec (
            int n,
            double * vals )
```

Constructor.

Parameters

n	The dimension.
vals	The values.

9.141.2.5 Vec() [5/8]

```
LinkPred::Vec::Vec (
            Vec const & v,
            std::shared_ptr< std::vector< int >> ind )
```

Create a new vector from an existing one by copying specified elements.

Parameters

v	The vector from which the data is taken.
ind	Index of the elements to be copied.

9.141.2.6 Vec() [6/8]

```
LinkPred::Vec::Vec (
            Vec const & v1,
            Vec const & v2 )
```

Create a new vector from two existing ones by concatenation.

Parameters

v1	The first vector.
v2	The second vector.

9.141.2.7 Vec() [7/8]

```
LinkPred::Vec::Vec (
            Vec const & that )
```

Copy constructor.

Parameters

that	The object to copy.

9.141.2.8 Vec() [8/8]

```
LinkPred::Vec::Vec (
            Vec && that )
```

Move constructor.

Parameters

that	The object to move.

9.141.2.9 ∼Vec()

```
virtual LinkPred::Vec::∼Vec ( ) [virtual]
```

Destructor.

9.141.3 Member Function Documentation

9.141.3.1 amax() [1/2]

```
double LinkPred::Vec::amax ( ) const
```

Returns

The maximum absolute value in the vector.

9.141.3.2 amax() [2/2]

```
double LinkPred::Vec::amax (
             int lim ) const
```

Returns

The maximum absolute value in the vector up to lim number of elements.

Parameters

lim	The limit.

9.141.3.3 amin() [1/2]

```
double LinkPred::Vec::amin ( ) const
```

Returns

The minimum absolute value in the vector.

9.141.3.4 amin() [2/2]

```
double LinkPred::Vec::amin (
             int lim ) const
```

Returns

The minimum absolute value in the vector up to lim number of elements.

Parameters

lim	The limit.

9.141.3.5 axpy()

```
void LinkPred::Vec::axpy (
             double a,
             Vec const & x )
```

Computes y = a ∗ x + y, where y is the current object (this) and x is a full vector.

Parameters

a	The value of a.
x	The other vector.

9.141.3.6 center() [1/2]

```
void LinkPred::Vec::center ( )
```

Center vector.

9.141.3.7 center() [2/2]

```
void LinkPred::Vec::center (
            int lim )
```

Center vector up to lim.

Parameters

lim	The limit.

9.141.3.8 find()

```
std::vector<int> LinkPred::Vec::find (
            double val ) const
```

Parameters

val	The avlue we are lookign for.

Returns

Indices of elements equal to val.

9.141.3.9 findNz()

```
std::vector<int> LinkPred::Vec::findNz ( ) const
```

Returns

Indices of non-zero elements.

9.141.3.10 getValues()

```
double* LinkPred::Vec::getValues ( ) const [inline]
```

Returns

Array of values.

9.141.3.11 ifmax()

```
int LinkPred::Vec::ifmax ( ) const
```

Returns

The index of the first maximum value in the vector.

9.141.3.12 ifmin()

```
int LinkPred::Vec::ifmin ( ) const
```

Returns

The index of the first minimum value in the vector.

9.141.3.13 ilmax()

```
int LinkPred::Vec::ilmax ( ) const
```

Returns

The index of the last maximum value in the vector.

9.141.3.14 ilmin()

```
int LinkPred::Vec::ilmin ( ) const
```

Returns

The index of the last minimum value in the vector.

9.141.3.15 imax()

```
int LinkPred::Vec::imax ( ) const
```

Returns

The index of the maximum value in the vector.

9.141.3.16 imin()

```
int LinkPred::Vec::imin ( ) const
```

Returns

The index of the minimum value in the vector.

9.141.3.17 isConstant()

```
bool LinkPred::Vec::isConstant ( ) const  [inline]
```

Returns

True if all entries of the vector are equal.

9.141.3.18 makeConstant()

```
bool LinkPred::Vec::makeConstant (
             double tol )
```

Check if the vector is constant (all entries are equal) up to a specific tolerance.

Parameters

tol	The tolerance.

Returns

True if the vector is constant.

9.141.3.19 max() [1/2]

```
double LinkPred::Vec::max ( ) const
```

Returns

The maximum value in the vector.

9.141.3.20 max() [2/2]

```
double LinkPred::Vec::max (
            int lim ) const
```

Returns

The maximum value in the vector up to lim number of elements.

Parameters

lim	The limit.

9.141.3.21 mean() [1/2]

```
double LinkPred::Vec::mean ( ) const
```

Computes the mean of the vector.

Returns

The mean of the vector.

9.141.3.22 mean() [2/2]

```
double LinkPred::Vec::mean (
            int lim ) const
```

Computes the mean of the vector.

Returns

The mean of the vector.

9.141.3.23 min() [1/2]

```
double LinkPred::Vec::min ( ) const
```

Returns

The minimum value in the vector.

9.141.3.24 min() [2/2]

```
double LinkPred::Vec::min (
            int lim ) const
```

Returns

The minimum value in the vector up to lim number of elements.

Parameters

lim	The limit.

9.141.3.25 norm() [1/2]

```
double LinkPred::Vec::norm ( ) const
```

Computes the norm of the vector.

Returns

The norm of the vector.

9.141.3.26 norm() [2/2]

```
double LinkPred::Vec::norm (
            int lim ) const
```

Computes the norm of the vector.

Parameters

lim	The limit.

Returns

The norm of the vector.

9.141.3.27 operator"!=()

```
bool LinkPred::Vec::operator!= (
            const Vec & other ) const
```

Overloads operator !=.

Parameters

other	The other vector.

Returns

True if the the two vectors are not equal.

9.141.3.28 operator=() [1/2]

```
Vec& LinkPred::Vec::operator= (
            Vec && that )
```

Move assignment operator.

Parameters

that	The object to move.

9.141.3.29 operator=() [2/2]

```
Vec& LinkPred::Vec::operator= (
            Vec const & that )
```

Copy assignment operator.

Parameters

that	The object to copy.

9.141.3.30 operator==()

```
bool LinkPred::Vec::operator== (
            const Vec & other ) const
```

Overloads operator ==.

Parameters

other	The other vector.

Returns

True if the the two vectors are equal.

9.141.3.31 operator[]() [1/2]

```
double& LinkPred::Vec::operator[ ] (
            const int k ) [inline]
```

Overloaded subscript.

Parameters

k	index.

Returns

reference to the k'th entry.

9.141.3.32 operator[]() [2/2]

```
double LinkPred::Vec::operator[ ] (
            const int k ) const [inline]
```

Overloaded subscript.

Parameters

k	index.

Returns

the k'th entry.

9.141.3.33 partScale()

```
void LinkPred::Vec::partScale (
            int start,
            int nb,
            double a )
```

Scale part of the vector.

Parameters

start	The starting index.
nb	Number of elements to be scaled.
a	The scale factor.

9.141.3.34 print() [1/4]

```
void LinkPred::Vec::print ( ) const
```

Print vector to standard output.

9.141.3.35 print() [2/4]

```
void LinkPred::Vec::print (
            std::ostream & out ) const
```

Print vector to output stream.

Parameters

out	The output stream.

9.141.3.36 print() [3/4]

```
void LinkPred::Vec::print (
            std::ostream & out,
            std::string header ) const
```

Print vector to output stream.

Parameters

out	The output stream.
header	Header.

9.141.3.37 print() [4/4]

```
void LinkPred::Vec::print (
            std::string header ) const
```

Print vector to standard output.

Parameters

header	Header.

9.141.3.38 read() [1/2]

```
void LinkPred::Vec::read (
            std::ifstream * in )
```

Read the values from a text file.

Parameters

in	Input file.

9.141.3.39 read() [2/2]

```
void LinkPred::Vec::read (
            std::string fileName )
```

Read the values from a text file.

Parameters

fileName	Input file name.

9.141.3.40 reset() [1/2]

```
void LinkPred::Vec::reset ( )
```

Reset vector to 0.

9.141.3.41 reset() [2/2]

```
void LinkPred::Vec::reset (
            double val )
```

Reset vector to a specified value.

Parameters

val	All vector elements are set to val.

9.141.3.42 resize() [1/2]

```
void LinkPred::Vec::resize (
            int newSize )
```

Resize the vector.

Parameters

newSize	The new size. If this is smaller than the current size, nothing happens.

9.141.3.43 resize() [2/2]

```
void LinkPred::Vec::resize (
            int newSize,
            double val )
```

Resize the vector.

Parameters

newSize	The new size. If this is smaller than the current size, nothing happens.
val	Initialization value for new entries.

9.141.3.44 scale()

```
void LinkPred::Vec::scale (
            double a )
```

Scale the vector by a scalar.

Parameters

a	The scale.

9.141.3.45 scatter()

```
void LinkPred::Vec::scatter (
            int start,
            Vec const & v,
            int const * ind )
```

Scatter a vector into the current vector starting from given position.

Parameters

start	The starting position.
v	The vector to be scattered.
ind	The index. Only entries in this array are affected by this operation.

9.141.3.46 setConstant()

```
void LinkPred::Vec::setConstant (
            bool constant ) [inline]
```

Parameters

constant	New value for constant.

9.141.3.47 size()

```
int LinkPred::Vec::size ( ) const [inline]
```

Returns

The size of the vector.

9.141.3.48 sum()

```
double LinkPred::Vec::sum ( ) const
```

Returns

The sum of the elements of the vector.

9.141.3.49 write() [1/2]

```
void LinkPred::Vec::write (
            std::ofstream * out ) const
```

Write values to file.

Parameters

out	Output file.

9.141.3.50 write() [2/2]

```
void LinkPred::Vec::write (
            std::string fileName ) const
```

Write values to file.

Parameters

fileName	Output file name.

9.141.4 Friends And Related Function Documentation

9.141.4.1 operator* [1/5]

```
Vec operator* (
            const GFMatrix & mat,
            const Vec & vec )  [friend]
```

GFMatrix-vector multiplication.

Parameters

mat	The matrix.
vec	The vector that will be multiplied by the matrix.

Returns

The resulting vector.

9.141.4.2 operator* [2/5]

```
Vec operator* (
            double a,
            Vec const & v )  [friend]
```

Scalar-vector multiplication.

Parameters

a	The scalar.
v	The vector.

Returns

a * v.

9.141.4.3 operator* [3/5]

```
Vec operator* (
            double a,
            Vec const & v )  [friend]
```

Scalar-vector multiplication.

Parameters

a	The scalar.
v	The vector.

Returns

a * v.

9.141.4.4 operator* [4/5]

```
Vec operator* (
            Vec const & v,
            double a )  [friend]
```

Vector-scalar multiplication.

Parameters

v	The vector.
a	The scalar.

Returns

v * a.

9.141.4.5 operator* [5/5]

```
Vec operator* (
            Vec const & v1,
            Vec const & v2 )  [friend]
```

Vector element-wise multiplication.

Parameters

v1	The first vector.
v2	The second vector.

Returns

v1 .* v2.

9.141.4.6 operator+ [1/3]

```
Vec operator+ (
            double a,
            Vec const & v )  [friend]
```

Scalar-vector addition.

Parameters

a	The scalar.
v	The vector.

Returns

a + v.

9.141.4.7 operator+ [2/3]

```
Vec operator+ (
            Vec const & v,
            double a )  [friend]
```

Vector-scalar addition.

Parameters

v	The vector.
a	The scalar.

Returns

v + a.

9.141.4.8 operator+ [3/3]

```
Vec operator+ (
            Vec const & v1,
            Vec const & v2 )  [friend]
```

Vector addition.

Parameters

v1	The first vector.
v2	The second vector.

Returns

v1 + v2.

9.141.4.9 operator- [1/3]

```
Vec operator- (
            double a,
            Vec const & v )  [friend]
```

Scalar-vector subtraction.

Parameters

a	The scalar.
v	The vector.

Returns

a - v.

9.141.4.10 operator- [2/3]

```
Vec operator- (
            Vec const & v,
            double a )  [friend]
```

Vector-scalar subtraction.

Parameters

v	The vector.
a	The scalar.

Returns

v - a.

9.141.4.11 operator- [3/3]

```
Vec operator- (
            Vec const & v1,
            Vec const & v2 )  [friend]
```

Vector subtraction.

Parameters

v1	The first vector.
v2	The second vector.

Returns

v1 - v2.

9.141.4.12 operator/ [1/3]

```
Vec operator/ (
            double a,
            Vec const & v )  [friend]
```

Scalar-vector division.

Parameters

a	The scalar.
v	The vector.

Returns

a / v.

9.141.4.13 operator/ [2/3]

```
Vec operator/ (
            Vec const & v,
            double a )  [friend]
```

Vector-scalar division.

Parameters

v	The vector.
a	The scalar.

Returns

v / a.

9.141.4.14 operator/ [3/3]

```
Vec operator/ (
            Vec const & v1,
            Vec const & v2 )  [friend]
```

Vector element-wise division.

Parameters

v1	The first vector.
v2	The second vector.

Returns

v1 ./ v2.

9.141.4.15 operator^

```
double operator^ (
            Vec const & v1,
            Vec const & v2 )  [friend]
```

Dot product.

Parameters

v1	The first vector.
v2	The second vector.

Returns

v1' ∗ v2.

The documentation for this class was generated from the following file:

- include/linkpred/numerical/linear/vec.hpp

Chapter 10

File Documentation

10.1 bindings/Java/include/LinkPredJavaConfig.hpp File Reference

Contains configuration options.

Macros

- #define **LinkPredJava_VERSION_MAJOR** 1
- #define **LinkPredJava_VERSION_MINOR** 0
- #define **LinkPredJava_VERSION_PATCH** 0

10.1.1 Detailed Description

Contains configuration options.

10.2 bindings/Python/include/LinkPredPythonConfig.hpp File Reference

Contains configuration options.

Macros

- #define **LinkPredPython_VERSION_MAJOR** 1
- #define **LinkPredPython_VERSION_MINOR** 0
- #define **LinkPredPython_VERSION_PATCH** 0
- #define **LINKPRED_WITH_OPENMP**
- #define **LINKPRED_WITH_MPI**
- #define **LINKPRED_WITH_MLPACK**

10.2.1 Detailed Description

Contains configuration options.

10.3 include/LinkPredConfig.hpp File Reference

Contains configuration options.

This graph shows which files directly or indirectly include this file:

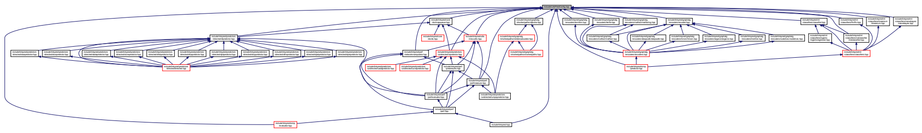

Macros

- #define **LinkPred_VERSION_MAJOR** 1
- #define **LinkPred_VERSION_MINOR** 0
- #define **LinkPred_VERSION_PATCH** 0

10.3.1 Detailed Description

Contains configuration options.

10.4 include/linkpred.hpp File Reference

Includes all headers of the library.

```
#include "LinkPredConfig.hpp"
#include "linkpred/core/core.hpp"
#include "linkpred/graphalg/graphalg.hpp"
#include "linkpred/predictors/predictors.hpp"
#include "linkpred/perf/perf.hpp"
#include "linkpred/utils/utils.hpp"
#include "linkpred/ml/ml.hpp"
#include "linkpred/numerical/numerical.hpp"
#include "linkpred/simp/simp.hpp"
```

Include dependency graph for linkpred.hpp:

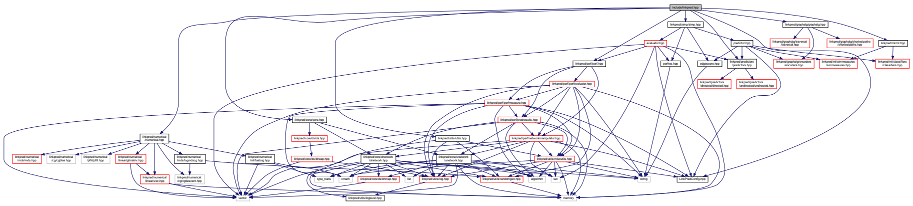

Namespaces

- LinkPred

  Main namespace.

10.4.1 Detailed Description

Includes all headers of the library.

10.5 include/linkpred/core/core.hpp File Reference

Includes the headers related to core classes.

```
#include "linkpred/core/unetwork/unetwork.hpp"
#include "linkpred/core/dnetwork/dnetwork.hpp"
#include "linkpred/core/ds/ds.hpp"
```

Include dependency graph for core.hpp:

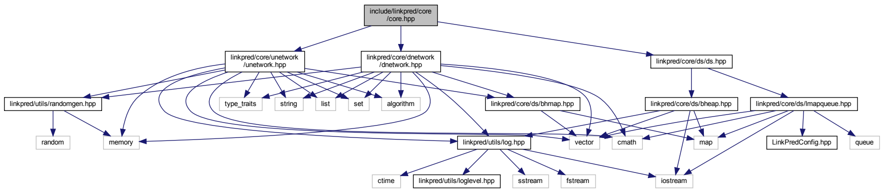

This graph shows which files directly or indirectly include this file:

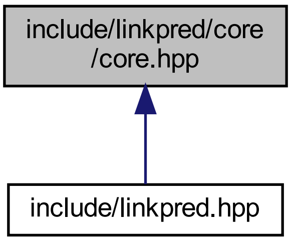

10.5.1 Detailed Description

Includes the headers related to core classes.

10.6 include/linkpred/core/dnetwork/dnetwork.hpp File Reference

Contains the implementation of a directed network data structure.

```
#include "linkpred/utils/log.hpp"
#include "linkpred/core/ds/bhmap.hpp"
#include "linkpred/utils/randomgen.hpp"
#include <cmath>
#include <memory>
#include <vector>
#include <list>
#include <set>
#include <algorithm>
#include <type_traits>
#include <string>
```

Include dependency graph for dnetwork.hpp:

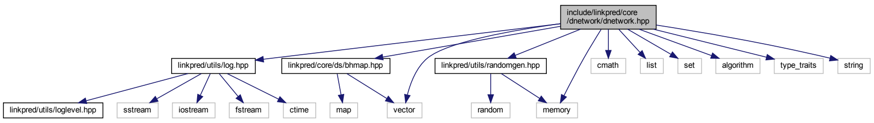

This graph shows which files directly or indirectly include this file:

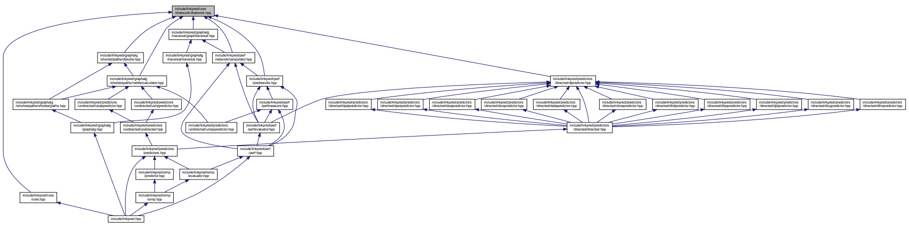

Classes

- class LinkPred::DNetwork< LabelT, NodeIDT, EdgeT >

  This class represents a directed network in the sense of graph theory.
- class LinkPred::DNetwork< LabelT, NodeIDT, EdgeT >::NodeDegIt

  Node-degree iterator. This class can be used to iterate over pairs of node IDs and in and out degrees.
- class LinkPred::DNetwork< LabelT, NodeIDT, EdgeT >::NonEdgeIt

  Nonedges iterator.
- class LinkPred::DNetwork< LabelT, NodeIDT, EdgeT >::RndNodeIt

  Randomized Nodes iterator.
- class LinkPred::DNetwork< LabelT, NodeIDT, EdgeT >::RndEdgeIt

  Randomized edges iterator.
- class LinkPred::DNetwork< LabelT, NodeIDT, EdgeT >::RndNonEdgeIt

  Randomized nonedges iterator.
- class LinkPred::DNetwork< LabelT, NodeIDT, EdgeT >::NodeMap< ValueType >

  A node map.
- class LinkPred::DNetwork< LabelT, NodeIDT, EdgeT >::NodeSMap< ValueType >

  A sparse node map.
- class LinkPred::DNetwork< LabelT, NodeIDT, EdgeT >::EdgeMap< ValueType >

  An edge map.

Namespaces

- LinkPred

  Main namespace.

10.6.1 Detailed Description

Contains the implementation of a directed network data structure.

10.7 include/linkpred/core/ds/bheap.hpp File Reference

Contains the implementation of a templated binary heap.

```
#include "linkpred/utils/log.hpp"
#include <vector>
#include <map>
#include <iostream>
```

Include dependency graph for bheap.hpp:

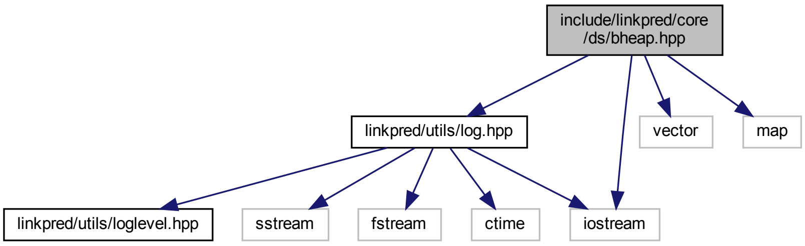

This graph shows which files directly or indirectly include this file:

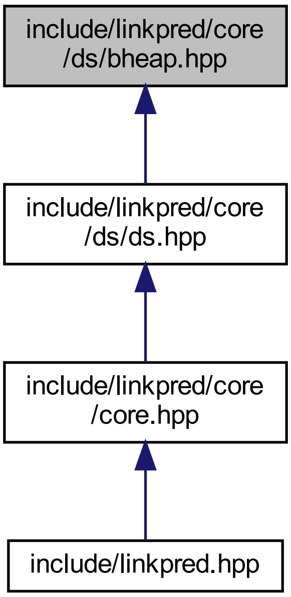

Classes

- class LinkPred::BHeap< T, P, ComparatorT, ComparatorP >

    A binary heap.

Namespaces

- LinkPred

    Main namespace.

10.7.1 Detailed Description

Contains the implementation of a templated binary heap.

10.8 include/linkpred/core/ds/bhmap.hpp File Reference

Contains the implementation of a templated bidirectional half map.

```
#include <map>
#include <vector>
```

Include dependency graph for bhmap.hpp:

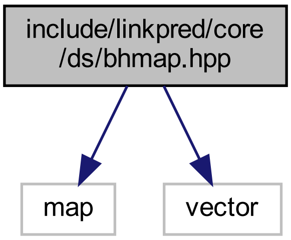

This graph shows which files directly or indirectly include this file:

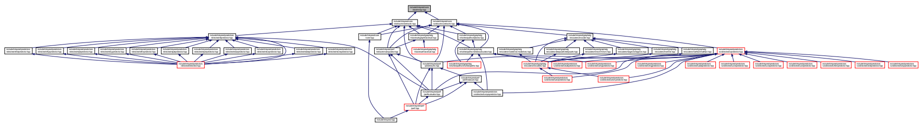

Classes

- class LinkPred::Bhmap< K, P, Comparator >

  A bidirectional half map.

Namespaces

- LinkPred

  Main namespace.

10.8.1 Detailed Description

Contains the implementation of a templated bidirectional half map.

10.9 include/linkpred/core/ds/ds.hpp File Reference

Includes the headers related to data structures.

```
#include "linkpred/core/ds/bheap.hpp"
#include "linkpred/core/ds/lmapqueue.hpp"
```

Include dependency graph for ds.hpp:

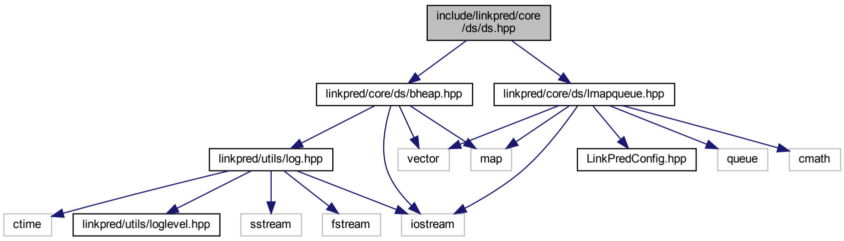

This graph shows which files directly or indirectly include this file:

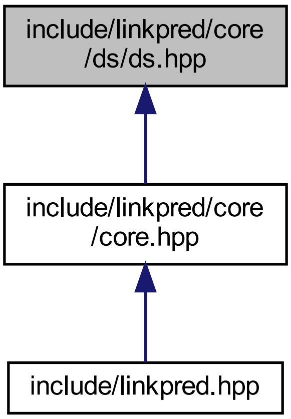

10.9.1 Detailed Description

Includes the headers related to data structures.

10.10 include/linkpred/core/ds/lmapqueue.hpp File Reference

Contains the implementation of a templated map-priority queue with limit on the capacity.

```
#include "LinkPredConfig.hpp"
#include <map>
#include <queue>
#include <vector>
#include <iostream>
```

```
#include <cmath>
```

Include dependency graph for lmapqueue.hpp:

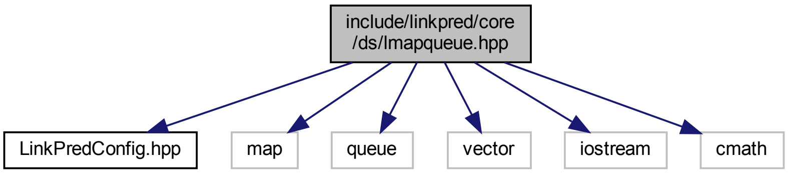

This graph shows which files directly or indirectly include this file:

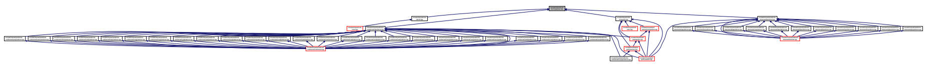

Classes

- class LinkPred::LMapQueue< K, P, KComparator, PComparator >

    A map-priority queue with limit on the capacity.

Namespaces

- LinkPred

    Main namespace.

10.10.1 Detailed Description

Contains the implementation of a templated map-priority queue with limit on the capacity.

10.11 include/linkpred/core/unetwork/unetwork.hpp File Reference

Contains the implementation of an undirected network data structure.

```
#include "linkpred/utils/log.hpp"
#include "linkpred/core/ds/bhmap.hpp"
#include "linkpred/utils/randomgen.hpp"
#include <cmath>
#include <memory>
#include <vector>
#include <list>
#include <set>
#include <algorithm>
#include <type_traits>
```

```
#include <string>
```

Include dependency graph for unetwork.hpp:

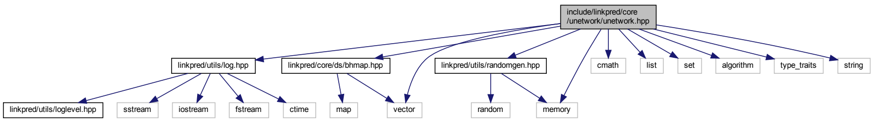

This graph shows which files directly or indirectly include this file:

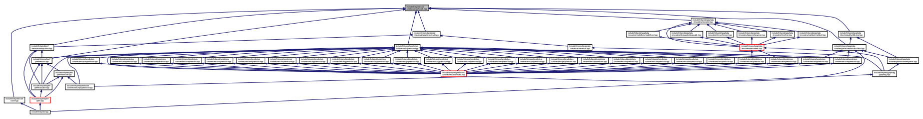

Classes

- class LinkPred::UNetwork< LabelT, NodeIDT, EdgeT >

  This class represents an undirected network in the sense of graph theory.
- class LinkPred::UNetwork< LabelT, NodeIDT, EdgeT >::NodeDegIt

  Node-degree iterator. This class can be used to iterate over pairs of node IDs and degrees.
- class LinkPred::UNetwork< LabelT, NodeIDT, EdgeT >::NonEdgeIt

  Nonedges iterator.
- class LinkPred::UNetwork< LabelT, NodeIDT, EdgeT >::RndNodeIt

  Randomized Nodes iterator.
- class LinkPred::UNetwork< LabelT, NodeIDT, EdgeT >::RndEdgeIt

  Randomized edges iterator.
- class LinkPred::UNetwork< LabelT, NodeIDT, EdgeT >::RndNonEdgeIt

  Randomized nonedges iterator.
- class LinkPred::UNetwork< LabelT, NodeIDT, EdgeT >::NodeMap< ValueType >

  A node map.
- class LinkPred::UNetwork< LabelT, NodeIDT, EdgeT >::NodeSMap< ValueType >

  A sparse node map.
- class LinkPred::UNetwork< LabelT, NodeIDT, EdgeT >::EdgeMap< ValueType >

  An edge map.

Namespaces

- LinkPred

  Main namespace.

10.11.1 Detailed Description

Contains the implementation of an undirected network data structure.

10.12 include/linkpred/graphalg/encoders/deepwalk/deepwalk.hpp File Reference

DeepWalk encoder.

```
#include "linkpred/graphalg/encoders/encoder.hpp"
#include "linkpred/utils/randomgen.hpp"
#include "linkpred/numerical/mf/fastsig.hpp"
#include <cmath>
#include <stdexcept>
#include <cstddef>
```

Include dependency graph for deepwalk.hpp:

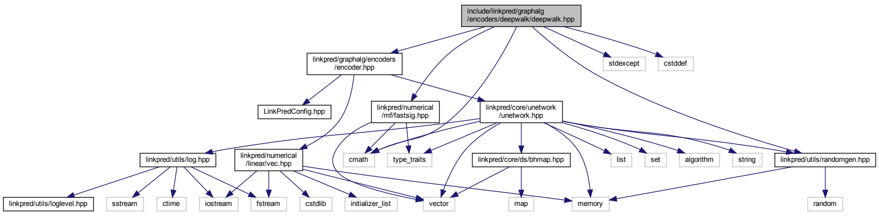

This graph shows which files directly or indirectly include this file:

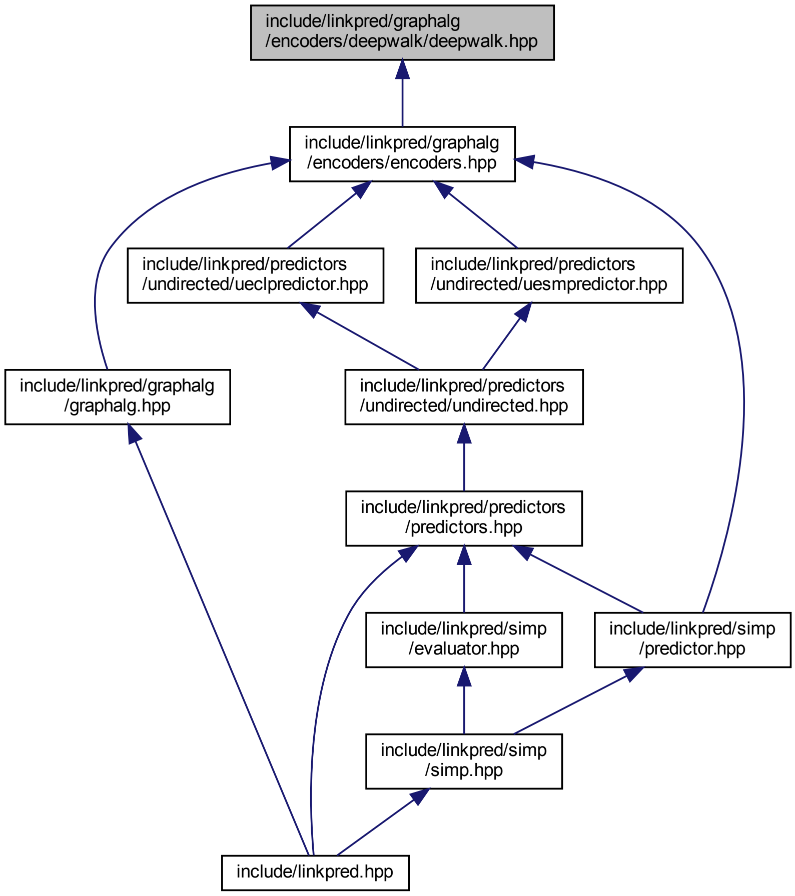

Classes

- class LinkPred::DeepWalk< Network >

  DeepWalk encoder. Reference: Perozzi, B., Al-Rfou, R., and Skiena, S. (2014). Deepwalk: Online learning of social representations. In Proceedings of the 20th ACM SIGKDD International Conference on Knowledge Discovery and Data Mining, KDD '14, pages 701–710, New York, NY, USA. Association for Computing Machinery. This implementation is based on the code `https://github.com/xgfs/deepwalk-c`.

Namespaces

- LinkPred

  Main namespace.

10.12.1 Detailed Description

DeepWalk encoder.

10.13 include/linkpred/graphalg/encoders/encoder.hpp File Reference

Contains the interface of a network encoder.

```
#include "LinkPredConfig.hpp"
#include "linkpred/core/unetwork/unetwork.hpp"
#include "linkpred/numerical/linear/vec.hpp"
```

Include dependency graph for encoder.hpp:

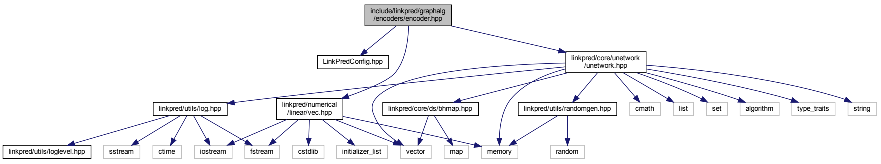

This graph shows which files directly or indirectly include this file:

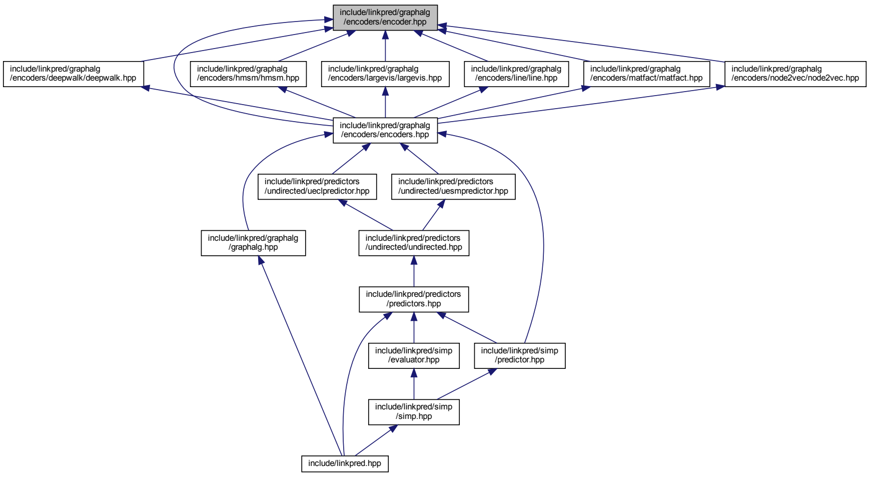

Classes

- class LinkPred::Encoder< Network >

    The interface of a network encoder.

Namespaces

- LinkPred

    Main namespace.

10.13.1 Detailed Description

Contains the interface of a network encoder.

10.14 include/linkpred/graphalg/encoders/encoders.hpp File Reference

Includes the headers related to network encoders.

```
#include "linkpred/graphalg/encoders/encoder.hpp"
#include "linkpred/graphalg/encoders/node2vec/node2vec.hpp"
#include "linkpred/graphalg/encoders/hmsm/hmsm.hpp"
#include "linkpred/graphalg/encoders/largevis/largevis.hpp"
#include "linkpred/graphalg/encoders/lem/lem.hpp"
#include "linkpred/graphalg/encoders/line/line.hpp"
#include "linkpred/graphalg/encoders/lle/lle.hpp"
#include "linkpred/graphalg/encoders/matfact/matfact.hpp"
#include "linkpred/graphalg/encoders/matfact/matfactcg.hpp"
#include "linkpred/graphalg/encoders/deepwalk/deepwalk.hpp"
```

Include dependency graph for encoders.hpp:

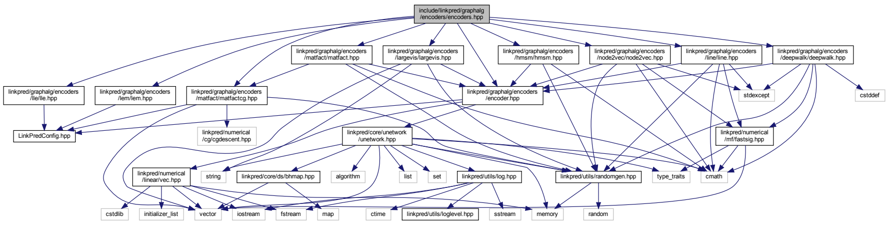

This graph shows which files directly or indirectly include this file:

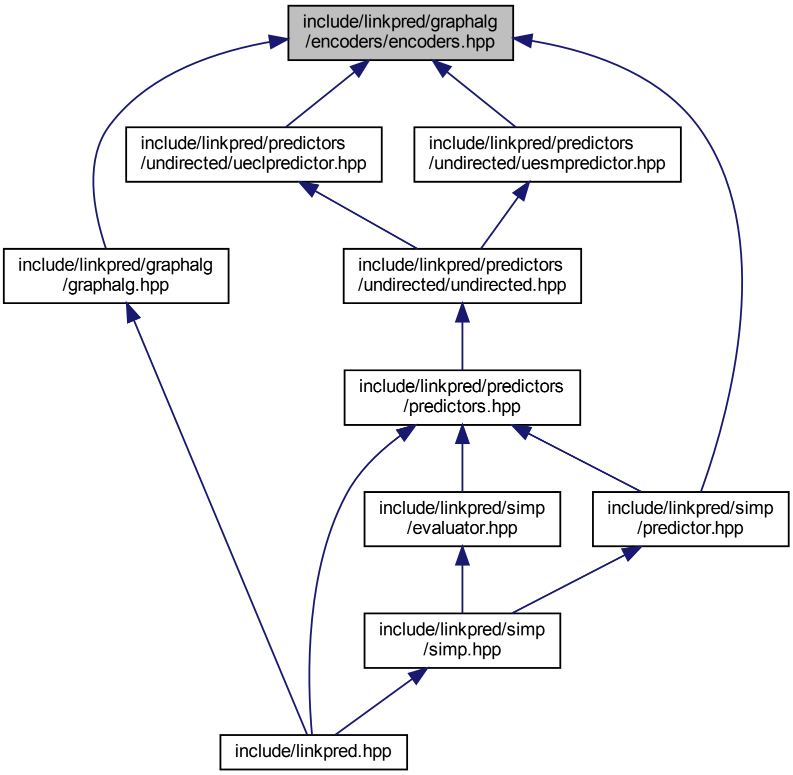

10.14.1 Detailed Description

Includes the headers related to network encoders.

10.15 include/linkpred/graphalg/encoders/hmsm/hmsm.hpp File Reference

Contains the implementation of an algorithm for embedding a network using a a hidden metric space model.

```
#include "linkpred/graphalg/encoders/encoder.hpp"
#include "linkpred/utils/randomgen.hpp"
#include <cmath>
```

Include dependency graph for hmsm.hpp:

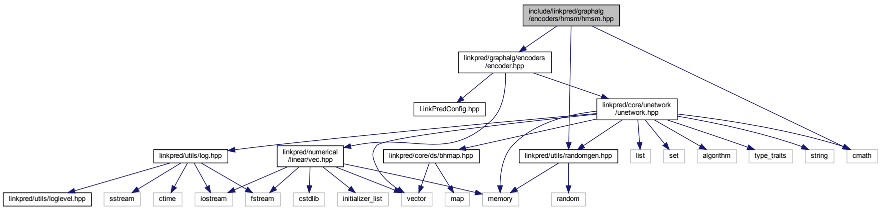

This graph shows which files directly or indirectly include this file:

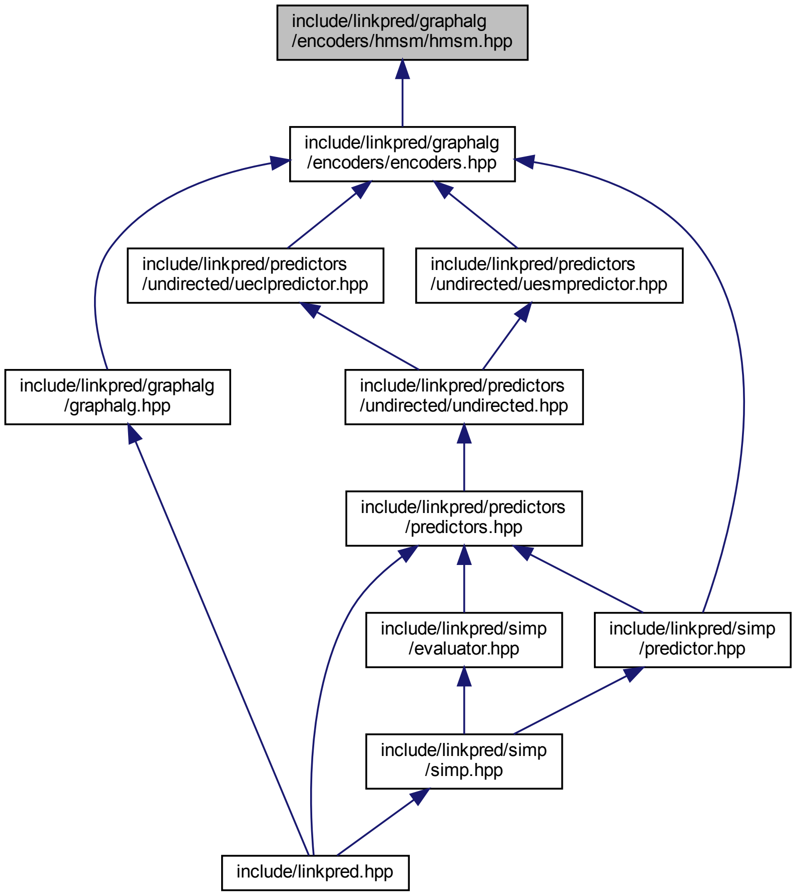

Classes

- class LinkPred::HMSM< Network >

  Contains the implementation of an algorithm for embedding a network using a a hidden metric space model. Reference: R. Alharbi, H. Benhidour, and S. Kerrache. "Link Prediction in Complex Net-works Based on a Hidden Variables Model". In: 2016 UKSim-AMSS 18th Inter-national Conference on Computer Modelling and Simulation (UKSim). 2016,pages 119–124.

Namespaces

- LinkPred

  Main namespace.

10.15.1 Detailed Description

Contains the implementation of an algorithm for embedding a network using a a hidden metric space model.

10.16 include/linkpred/graphalg/encoders/largevis/largevis.hpp File Reference

LargeVis encoder.

```
#include "linkpred/graphalg/encoders/encoder.hpp"
#include "linkpred/utils/randomgen.hpp"
#include <string>
#include <vector>
```

Include dependency graph for largevis.hpp:

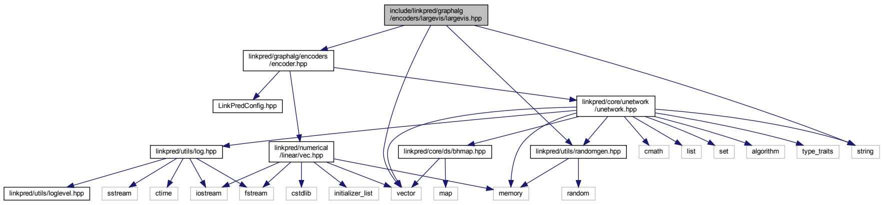

This graph shows which files directly or indirectly include this file:

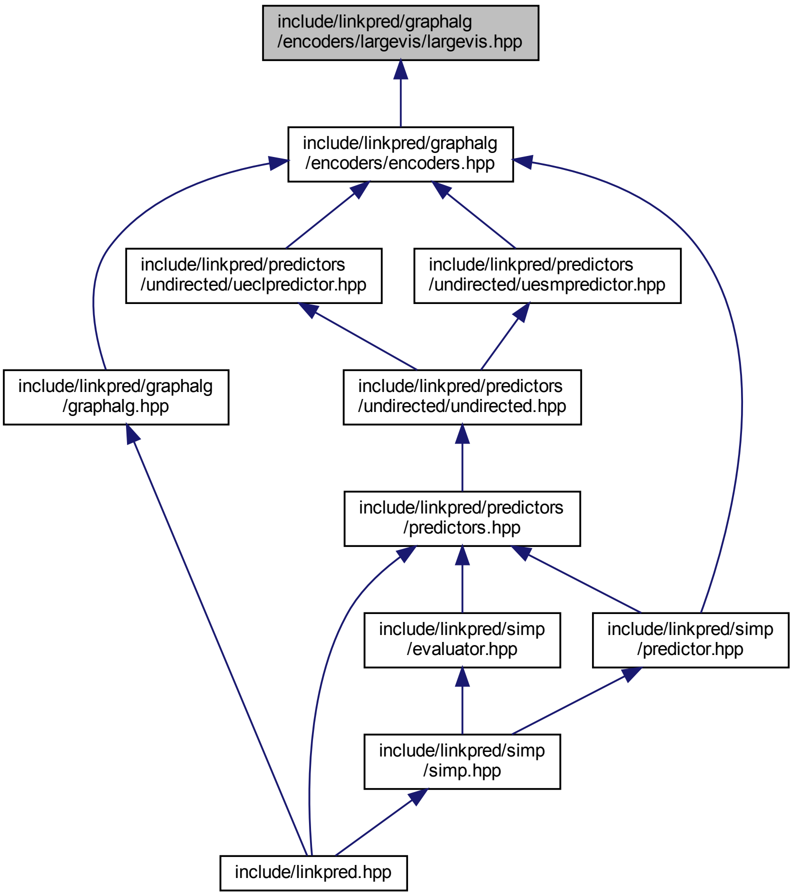

Classes

- class LinkPred::LargeVis< Network >

  LargeVis encoder. Reference: Tang, J., Liu, J., Zhang, M., and Mei, Q. (2016b). Visualizing large-scale and high-dimensional data. In Bourdeau, J., Hendler, J., Nkambou, R., Horrocks, I., and Zhao, B. Y., editors, WWW, pages 287–297. ACM. This implementation is based on the code `https://github.com/lferry007/LargeVis`.

Namespaces

- LinkPred

  Main namespace.

10.16.1 Detailed Description

LargeVis encoder.

10.17 include/linkpred/graphalg/encoders/lem/lem.hpp File Reference

Contains the implementation of Laplacian eigenmaps embedding (LEM).

`#include "LinkPredConfig.hpp"`
Include dependency graph for lem.hpp:

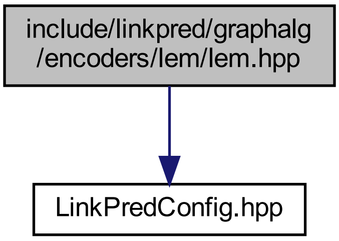

This graph shows which files directly or indirectly include this file:

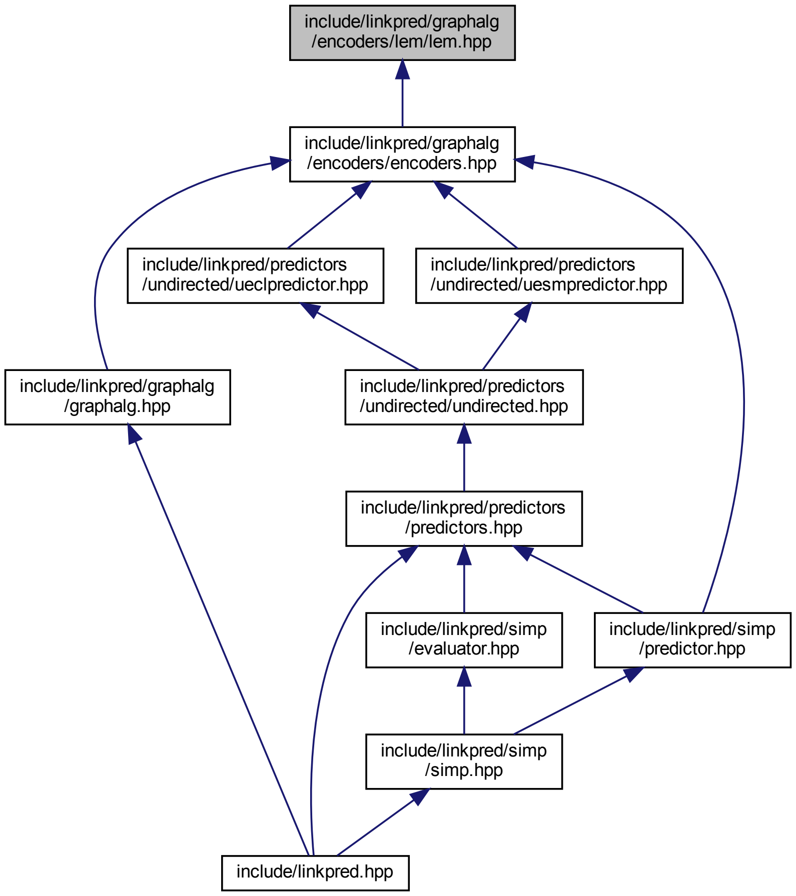

10.17.1 Detailed Description

Contains the implementation of Laplacian eigenmaps embedding (LEM).

10.18 include/linkpred/graphalg/encoders/line/line.hpp File Reference

The LINE (Large-scale Information Network Embedding) encoder.

```
#include "linkpred/graphalg/encoders/encoder.hpp"
#include "linkpred/utils/randomgen.hpp"
#include "linkpred/numerical/mf/fastsig.hpp"
#include <cmath>
```

`#include <stdexcept>`
Include dependency graph for line.hpp:

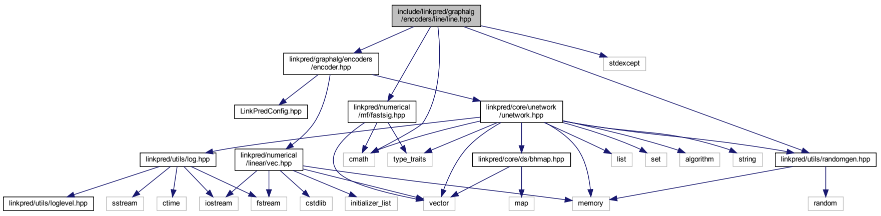

This graph shows which files directly or indirectly include this file:

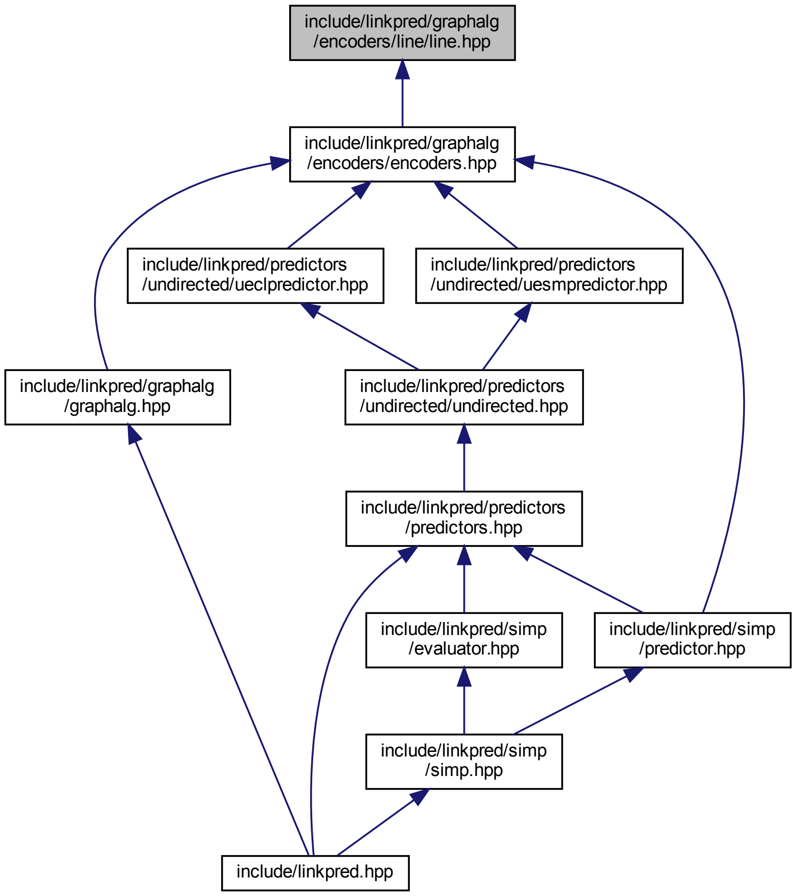

Classes

- class LinkPred::LINE< Network >

    LINE encoder.

Namespaces

- LinkPred

    Main namespace.

10.18.1 Detailed Description

The LINE (Large-scale Information Network Embedding) encoder.

10.19 include/linkpred/graphalg/encoders/lle/lle.hpp File Reference

Contains the implementation of locally linear graph embedding (LLE).

```
#include "LinkPredConfig.hpp"
```

Include dependency graph for lle.hpp:

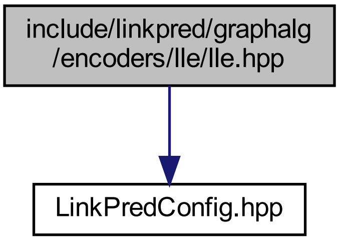

This graph shows which files directly or indirectly include this file:

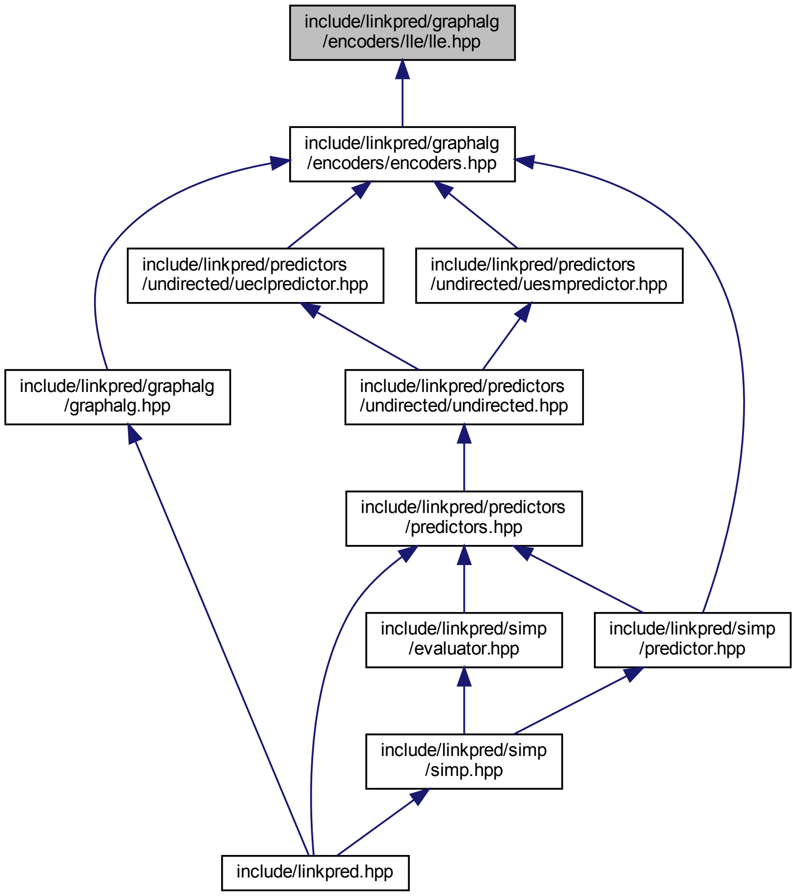

10.19.1 Detailed Description

Contains the implementation of locally linear graph embedding (LLE).

10.20 include/linkpred/graphalg/encoders/matfact/matfact.hpp File Reference

Contains the implementation of an algorithm for embedding a network using matrix factorization. Reference: Koren, Y., Bell, R., and Volinsky, C. (2009). Matrix factorization techniques for recommender systems. Computer, 42(8)↩:30–37 Ahmed, A., Shervashidze, N., Narayanamurthy, S., Josifovski, V., and Smola, A. J. (2013). Distributed large-scale natural graph factorization. In Proceedings of the 22nd International Conference on World Wide Web, WWW '13, pages 37–48, New York, NY, USA. Association for Computing Machinery.

```
#include "linkpred/graphalg/encoders/encoder.hpp"
#include "linkpred/graphalg/encoders/matfact/matfactcg.hpp"
#include "linkpred/utils/randomgen.hpp"
```

`#include <cmath>`

Include dependency graph for matfact.hpp:

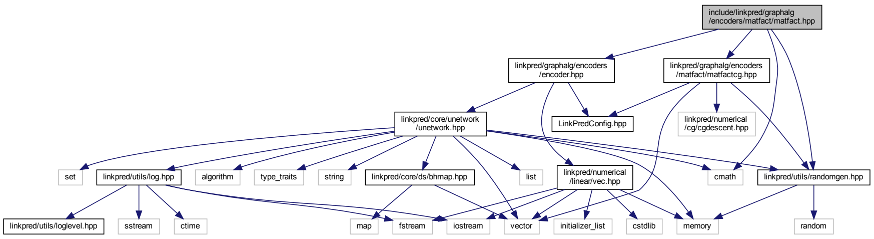

This graph shows which files directly or indirectly include this file:

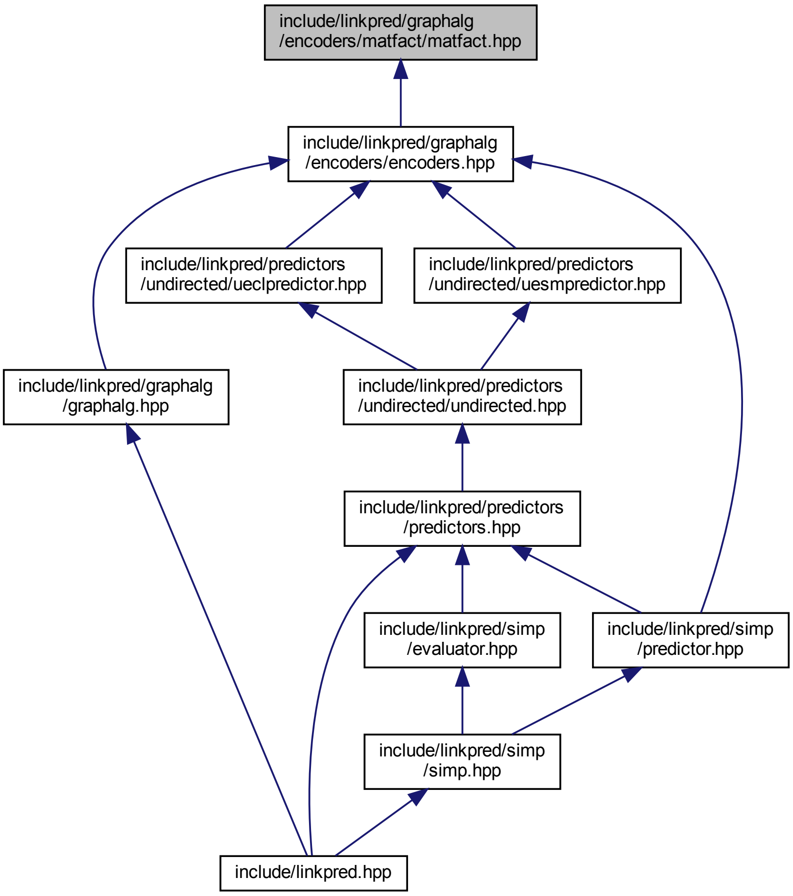

Classes

- class LinkPred::MatFact< Network >

  Contains the implementation of an algorithm for embedding a network using matrix factorization. Reference: Koren, Y., Bell, R., and Volinsky, C. (2009). Matrix factorization techniques for recommender systems. Computer, 42(8)↩ :30–37 Ahmed, A., Shervashidze, N., Narayanamurthy, S., Josifovski, V., and Smola, A. J. (2013). Distributed large-scale natural graph factorization. In Proceedings of the 22nd International Conference on World Wide Web, WWW '13, pages 37–48, New York, NY, USA. Association for Computing Machinery.

Namespaces

- LinkPred

  Main namespace.

10.20.1 Detailed Description

Contains the implementation of an algorithm for embedding a network using matrix factorization. Reference: Koren, Y., Bell, R., and Volinsky, C. (2009). Matrix factorization techniques for recommender systems. Computer, 42(8)↩ :30–37 Ahmed, A., Shervashidze, N., Narayanamurthy, S., Josifovski, V., and Smola, A. J. (2013). Distributed large-scale natural graph factorization. In Proceedings of the 22nd International Conference on World Wide Web, WWW ’13, pages 37–48, New York, NY, USA. Association for Computing Machinery.

10.21 include/linkpred/graphalg/encoders/matfact/matfactcg.hpp File Reference

Contains the implementation of the optimization problem associated with matrix factorization.

```
#include "LinkPredConfig.hpp"
#include "linkpred/numerical/cg/cgdescent.hpp"
#include "linkpred/utils/randomgen.hpp"
#include <vector>
```

Include dependency graph for matfactcg.hpp:

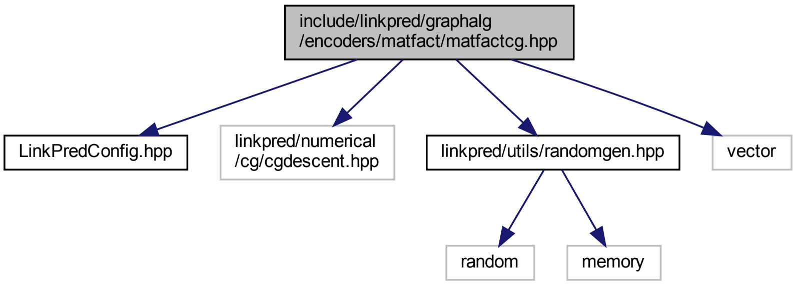

This graph shows which files directly or indirectly include this file:

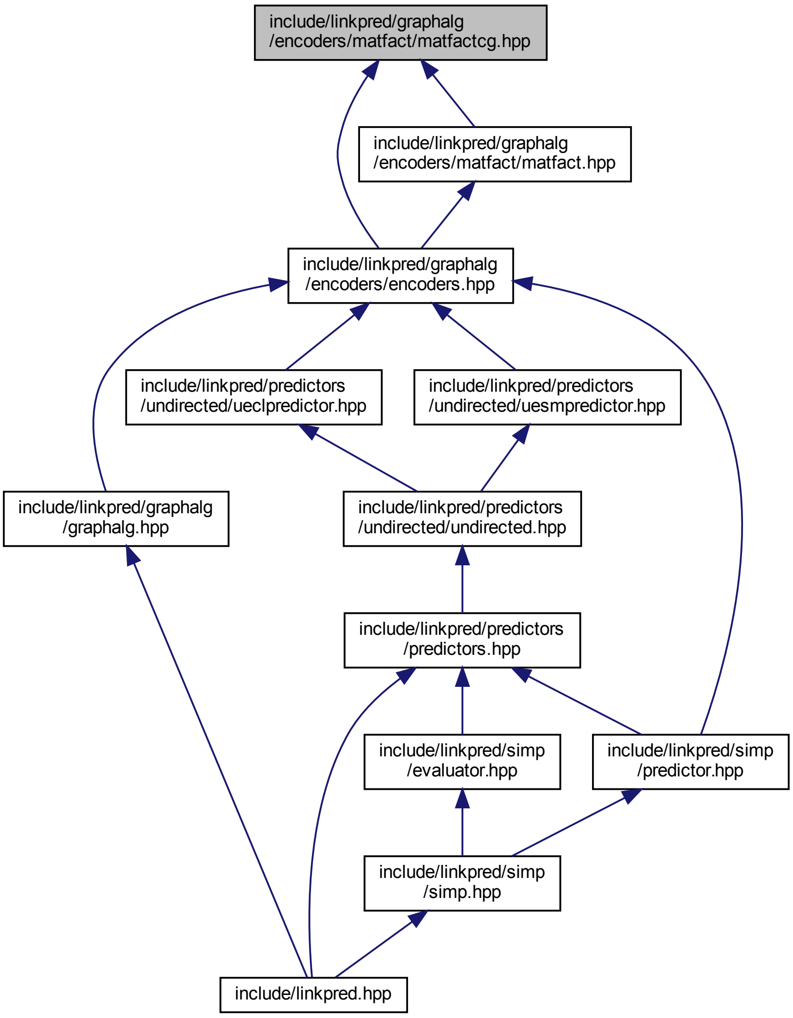

Classes

- struct LinkPred::MatFactPbData

  A simple structure to store matrix factoization problem data.
- class LinkPred::MatFactCG

  Optimization problem associated with matrix factorization.

Namespaces

- LinkPred

  Main namespace.

10.21.1 Detailed Description

Contains the implementation of the optimization problem associated with matrix factorization.

10.22 include/linkpred/graphalg/encoders/node2vec/node2vec.hpp File Reference

A node2vec encoder.

```
#include "linkpred/graphalg/encoders/encoder.hpp"
#include "linkpred/utils/randomgen.hpp"
#include "linkpred/numerical/mf/fastsig.hpp"
#include <cmath>
#include <stdexcept>
```

Include dependency graph for node2vec.hpp:

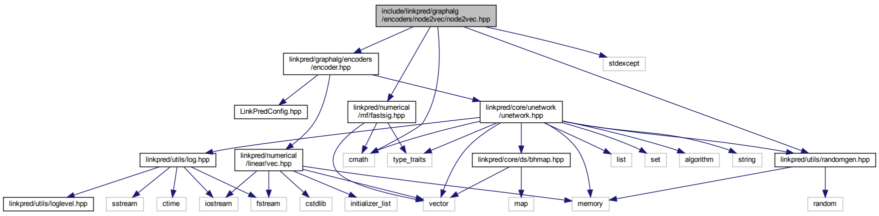

This graph shows which files directly or indirectly include this file:

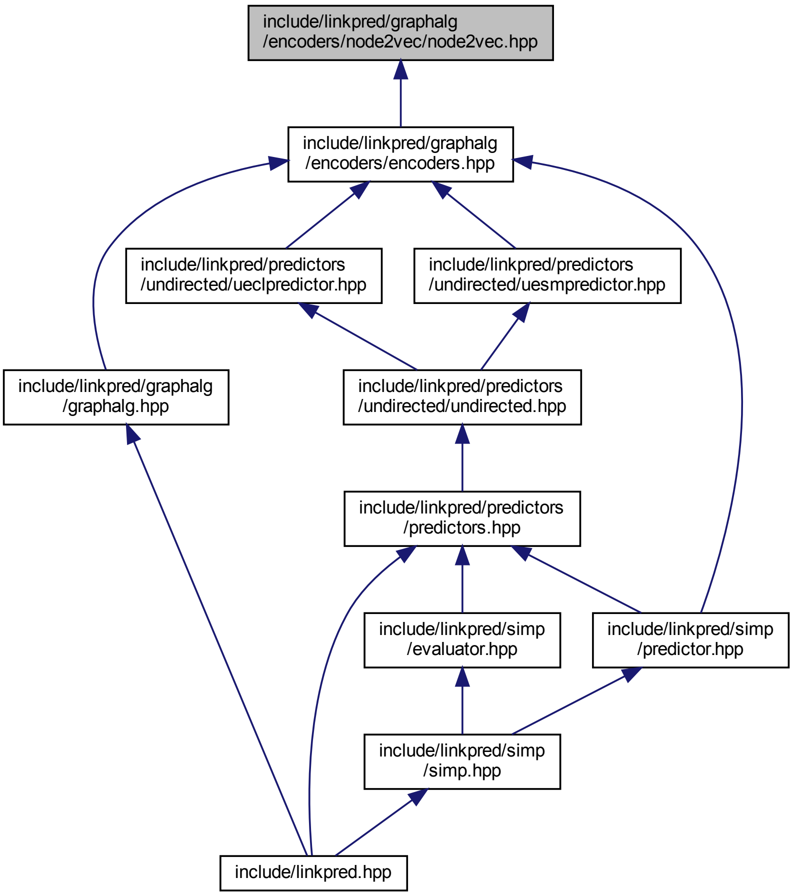

Classes

- class LinkPred::Node2Vec< Network >

  Node2Vec encoder. References: Grover, A. and Leskovec, J. (2016). Node2vec: Scalable feature learning for networks. In Proceedings of the 22nd ACM SIGKDD International Conference on Knowledge Discovery and Data Mining, KDD'16, pages 855–864, New York, NY, USA. Association for Computing Machinery. This implementation is based on the code `https://github.com/xgfs/node2vec-c`.

Namespaces

- LinkPred

  Main namespace.

10.22.1 Detailed Description

A node2vec encoder.

10.23 include/linkpred/graphalg/graphalg.hpp File Reference

Includes the headers related to graph algorithms.

```
#include "linkpred/graphalg/encoders/encoders.hpp"
#include "linkpred/graphalg/shortestpaths/shortestpaths.hpp"
#include "linkpred/graphalg/traversal/traversal.hpp"
```

Include dependency graph for graphalg.hpp:

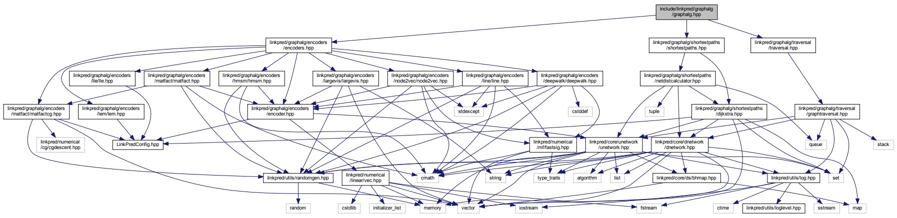

This graph shows which files directly or indirectly include this file:

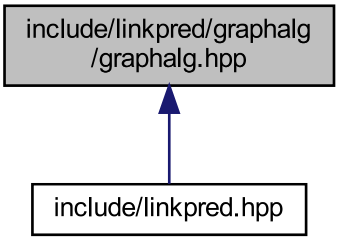

10.23.1 Detailed Description

Includes the headers related to graph algorithms.

10.24 include/linkpred/graphalg/shortestpaths/dijkstra.hpp File Reference

Contains an implementation of Dijkstra's algorithm.

```
#include "LinkPredConfig.hpp"
#include "linkpred/core/unetwork/unetwork.hpp"
#include "linkpred/core/dnetwork/dnetwork.hpp"
#include <map>
#include <set>
#include <vector>
```

Include dependency graph for dijkstra.hpp:

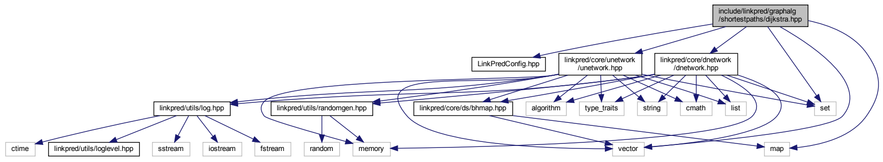

This graph shows which files directly or indirectly include this file:

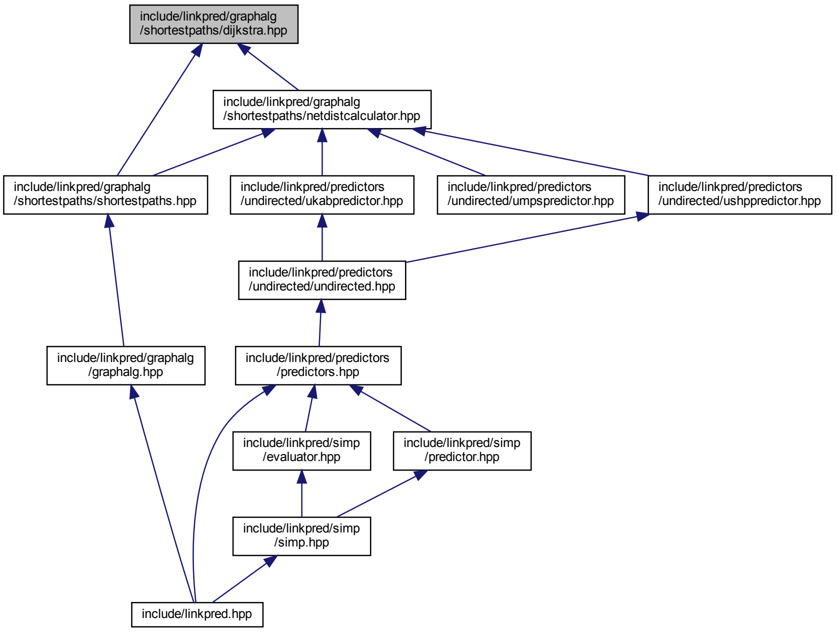

Classes

- class LinkPred::Dijkstra< Network, DistType, NbHopsType >

  An implementation of Dijkstra's algorithm.

Namespaces

- LinkPred

  Main namespace.

10.24.1 Detailed Description

Contains an implementation of Dijkstra's algorithm.

10.25 include/linkpred/graphalg/shortestpaths/netdistcalculator.hpp File Reference

Contains the implementation of classes for computing distances in network.

```
#include "linkpred/core/unetwork/unetwork.hpp"
#include "linkpred/core/dnetwork/dnetwork.hpp"
#include "linkpred/graphalg/shortestpaths/dijkstra.hpp"
#include <tuple>
#include <queue>
```

Include dependency graph for netdistcalculator.hpp:

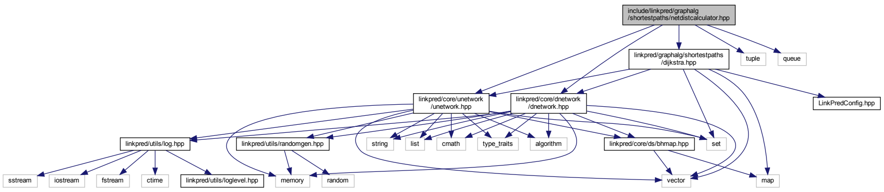

This graph shows which files directly or indirectly include this file:

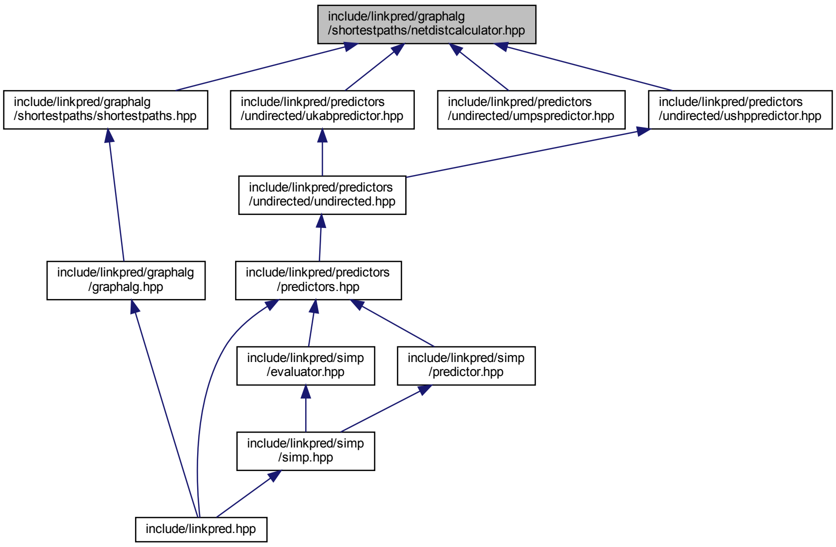

Classes

- class LinkPred::NetDistCalculator< Network, DistType, NbHopsType >

  Interface for calculating the distance between nodes in a network.
- class LinkPred::NetSimlCalculator< Network, DistType, NbHopsType >

  Interface for calculating the similarity between nodes in a network.
- class LinkPred::NetIndSimlCalculator< Network, DistType, NbHopsType >

  Interface for calculating the indirect similarity between nodes in a network.
- class LinkPred::ESPDistCalculator< Network, DistType, NbHopsType >

  Exact shortest path distance calculator.
- class LinkPred::ESPLDistCalculator< Network, DistType, NbHopsType >

  Exact shortest path distance calculator with limits on the number of hops.
- class LinkPred::ASPDistCalculator< Network, DistType, NbHopsType >

  Approximate shortest path distance calculator.
- class LinkPred::ESPDsimCalculator< Network, DsimType, NbHopsType >

  Exact shortest path dissimilarity calculator.
- class LinkPred::ASPDsimCalculator< Network, DsimType, NbHopsType >

  Approximate shortest path dissimilarity calculator.
- class LinkPred::ESPSimlCalculator< Network, DistType, NbHopsType >

  Exact shortest path distance calculator.
- class LinkPred::ESPLSimlCalculator< Network, DistType, NbHopsType >

  Exact shortest path distance calculator.
- class LinkPred::ESPIndSimlCalculator< Network, DistType, NbHopsType >

  Exact shortest path distance calculator.
- class LinkPred::DESPLDistCalculator< Network, DistType, NbHopsType >

  Exact shortest path distance calculator on a directed network with limits on the number of hops.

Namespaces

- LinkPred

  Main namespace.

Enumerations

- enum LinkPred::CacheLevel { LinkPred::NoCache, LinkPred::NodeCache, LinkPred::NetworkCache }

  Cache levels.

10.25.1 Detailed Description

Contains the implementation of classes for computing distances in network.

10.26 include/linkpred/graphalg/shortestpaths/shortestpaths.hpp File Reference

Includes the headers related to shortest paths algorithms.

```
#include "linkpred/graphalg/shortestpaths/dijkstra.hpp"
#include "linkpred/graphalg/shortestpaths/netdistcalculator.hpp"
```

Include dependency graph for shortestpaths.hpp:

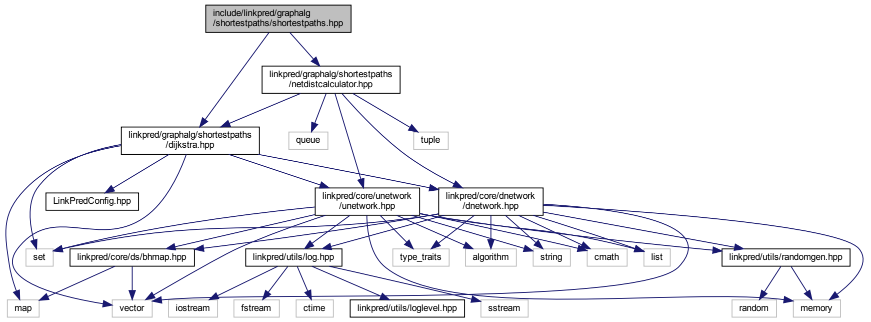

This graph shows which files directly or indirectly include this file:

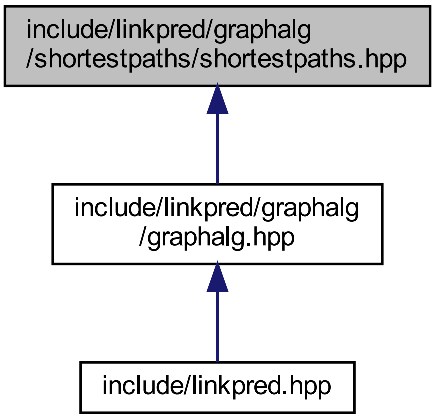

10.26.1 Detailed Description

Includes the headers related to shortest paths algorithms.

10.27 include/linkpred/graphalg/traversal/graphtraversal.hpp File Reference

Contains the implementation of graph traversal algorithms.

```
#include "linkpred/core/unetwork/unetwork.hpp"
#include "linkpred/core/dnetwork/dnetwork.hpp"
#include "linkpred/utils/log.hpp"
#include <queue>
#include <stack>
#include <set>
```

Include dependency graph for graphtraversal.hpp:

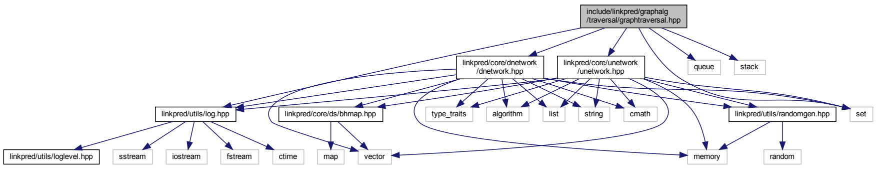

This graph shows which files directly or indirectly include this file:

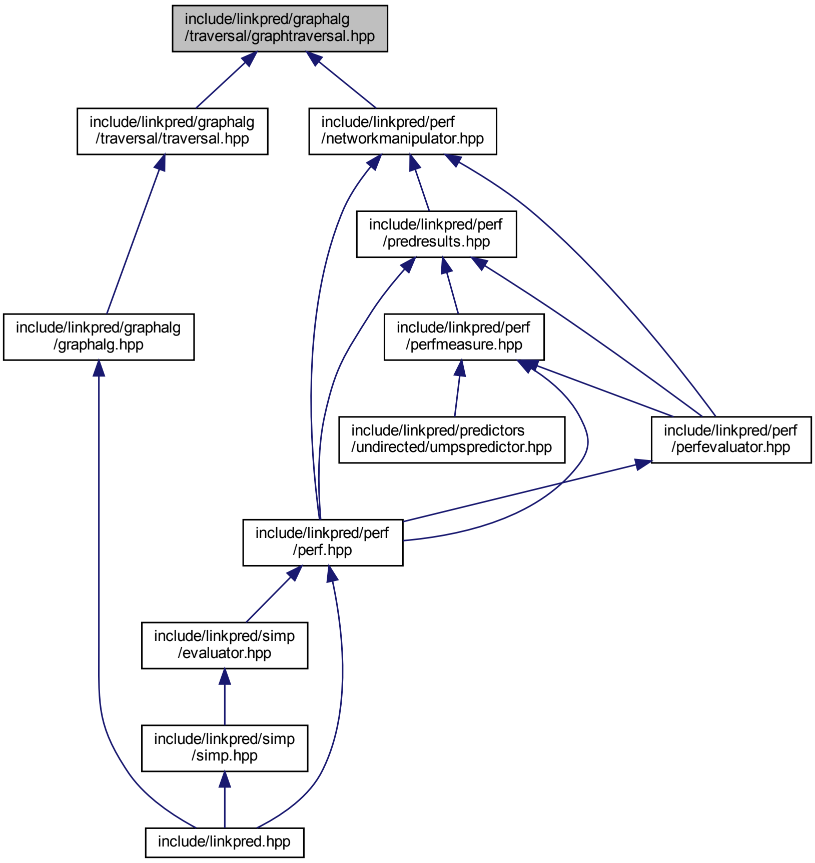

Classes

- class LinkPred::Counter< Network >

  A class that counts nodes during traversal.
- class LinkPred::Collector< Network >

  A class that collects nodes during traversal.
- class LinkPred::GraphTraversal< Network, NodeProcessor >

  Graph traversal interface.
- class LinkPred::BFS< Network, NodeProcessor >

  BFS graph traversal.
- class LinkPred::DFS< Network, NodeProcessor >

  DFS graph traversal.

Namespaces

- LinkPred

  Main namespace.

10.27.1 Detailed Description

Contains the implementation of graph traversal algorithms.

10.28 include/linkpred/graphalg/traversal/traversal.hpp File Reference

Includes the headers related to graph traversal algorithms.

`#include "linkpred/graphalg/traversal/graphtraversal.hpp"`
Include dependency graph for traversal.hpp:

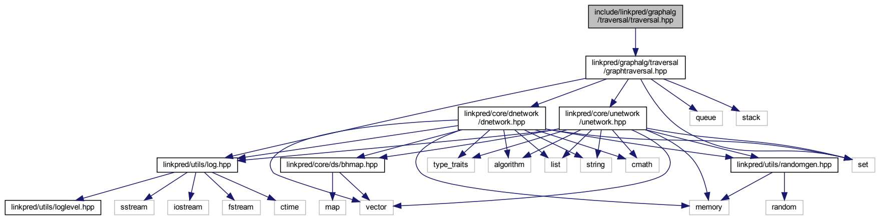

This graph shows which files directly or indirectly include this file:

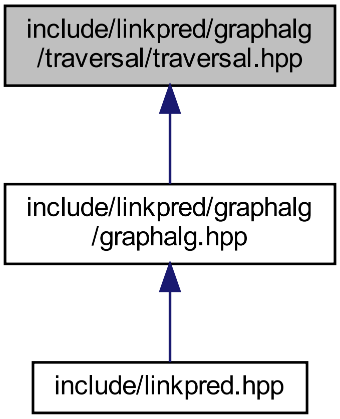

10.28.1 Detailed Description

Includes the headers related to graph traversal algorithms.

10.29 include/linkpred/instantiations.hpp File Reference

Contains explicit instantiations of template classes.

10.29.1 Detailed Description

Contains explicit instantiations of template classes.

10.30 include/linkpred/ml/classifiers/classifier.hpp File Reference

Contains the interface of a classifier.

```
#include "LinkPredConfig.hpp"
#include "linkpred/numerical/linear/vec.hpp"
#include <vector>
```

Include dependency graph for classifier.hpp:

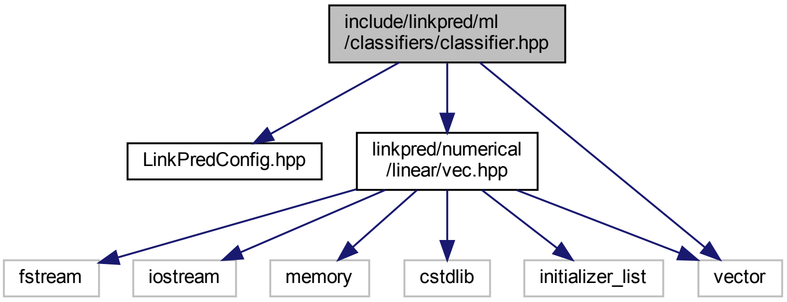

This graph shows which files directly or indirectly include this file:

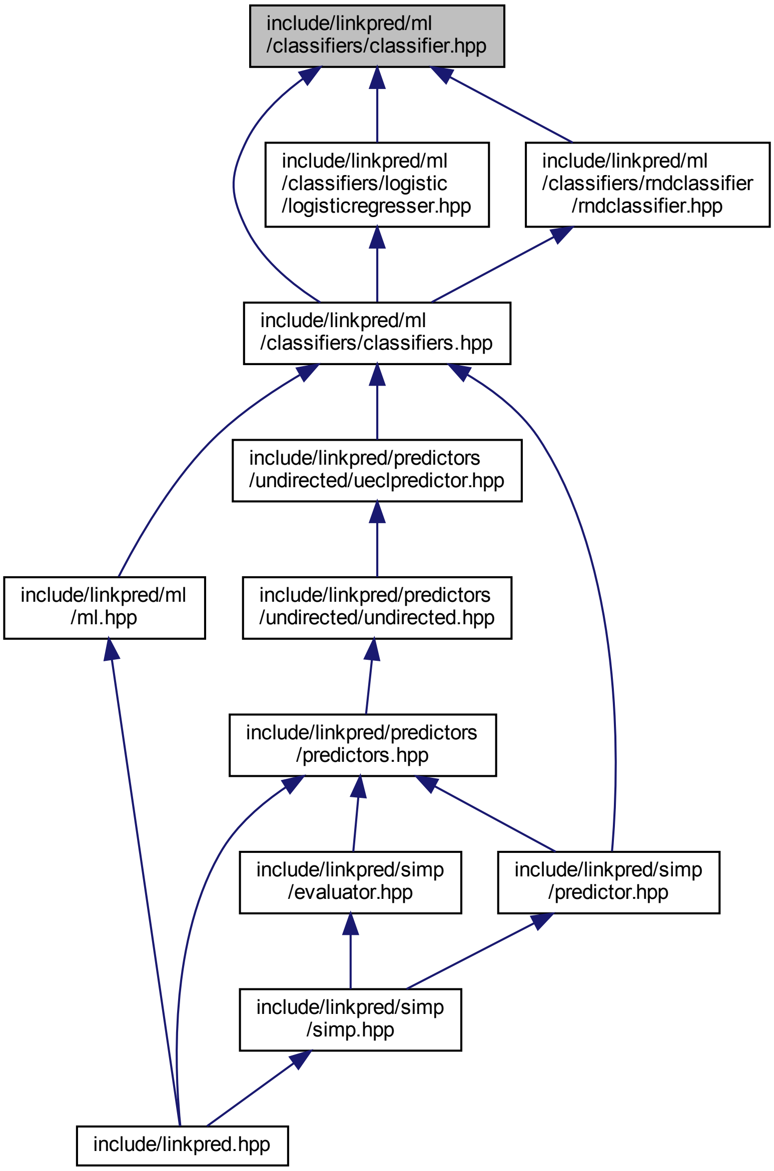

Classes

- class LinkPred::Classifier< InRndIt, OutRndIt, ScoreRndIt >

  Interface of a binary classifier.

Namespaces

- LinkPred

  Main namespace.

10.30.1 Detailed Description

Contains the interface of a classifier.

10.31 include/linkpred/ml/classifiers/classifiers.hpp File Reference

Includes the headers related to classifiers.

```
#include "linkpred/ml/classifiers/classifier.hpp"
#include "linkpred/ml/classifiers/ffn/ffn.hpp"
#include "linkpred/ml/classifiers/logistic/logisticregresser.hpp"
#include "linkpred/ml/classifiers/linearsvm/linearsvm.hpp"
#include "linkpred/ml/classifiers/naivebayes/naivebayes.hpp"
#include "linkpred/ml/classifiers/rndclassifier/rndclassifier.hpp"
```

Include dependency graph for classifiers.hpp:

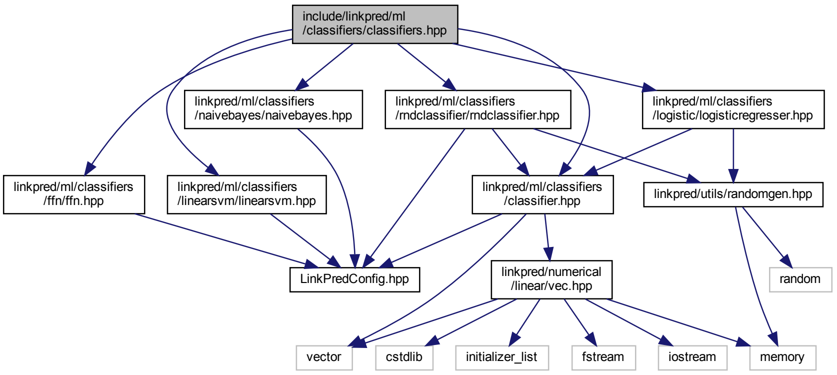

This graph shows which files directly or indirectly include this file:

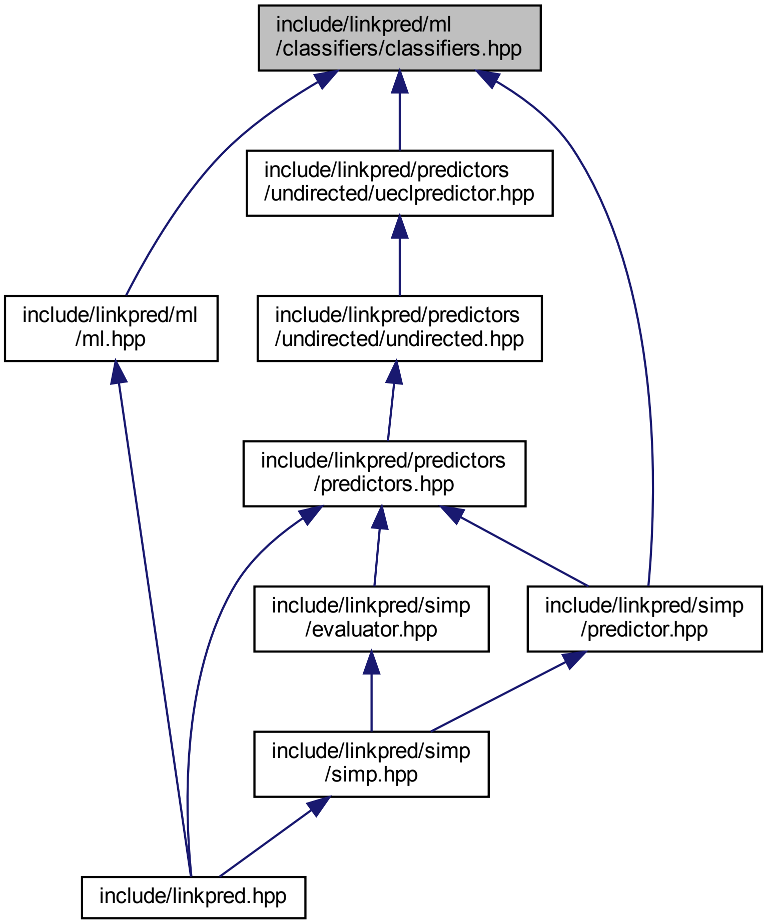

10.31.1 Detailed Description

Includes the headers related to classifiers.

10.32 include/linkpred/ml/classifiers/ffn/ffn.hpp File Reference

Contains a wrapper of mlpack::ann::FFN (feed-forward neural network).

```
#include "LinkPredConfig.hpp"
```

Include dependency graph for ffn.hpp:

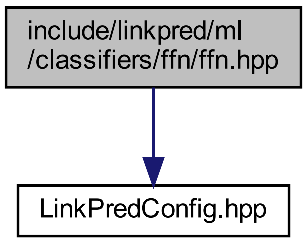

This graph shows which files directly or indirectly include this file:

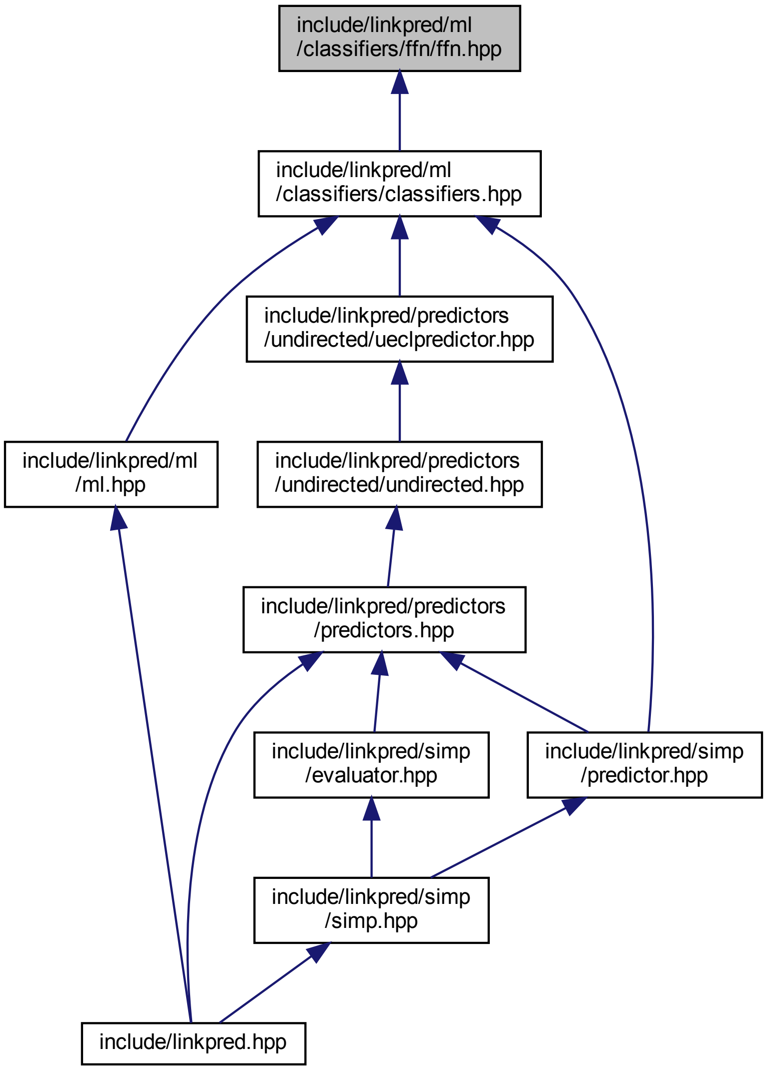

10.32.1 Detailed Description

Contains a wrapper of mlpack::ann::FFN (feed-forward neural network).

10.33 include/linkpred/ml/classifiers/linearsvm/linearsvm.hpp File Reference

Contains a wrapper of mlpack::smv::LinearSVM.

```
#include "LinkPredConfig.hpp"
```

Include dependency graph for linearsvm.hpp:

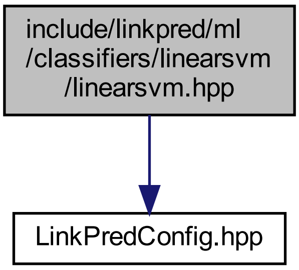

This graph shows which files directly or indirectly include this file:

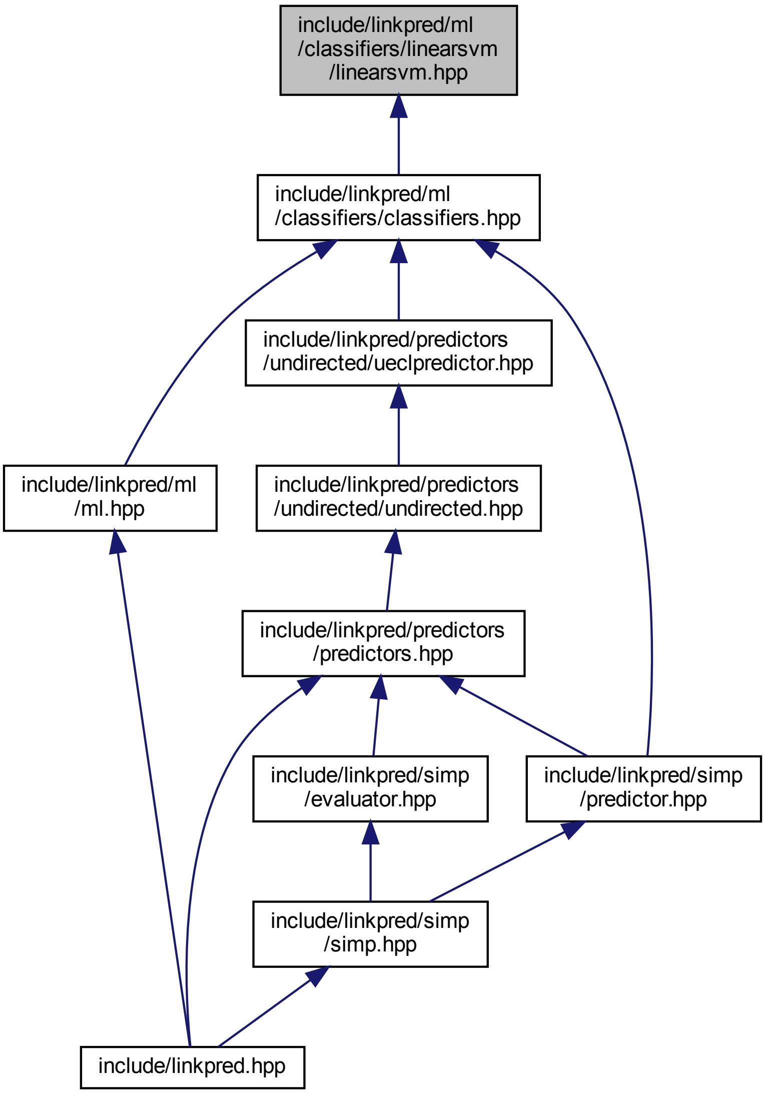

10.33.1 Detailed Description

Contains a wrapper of mlpack::smv::LinearSVM.

10.34 include/linkpred/ml/classifiers/logistic/logisticregresser.hpp File Reference

Contains the implementation of a logistic regression algorithm.

```
#include "linkpred/ml/classifiers/classifier.hpp"
#include "linkpred/utils/randomgen.hpp"
```

Include dependency graph for logisticregresser.hpp:

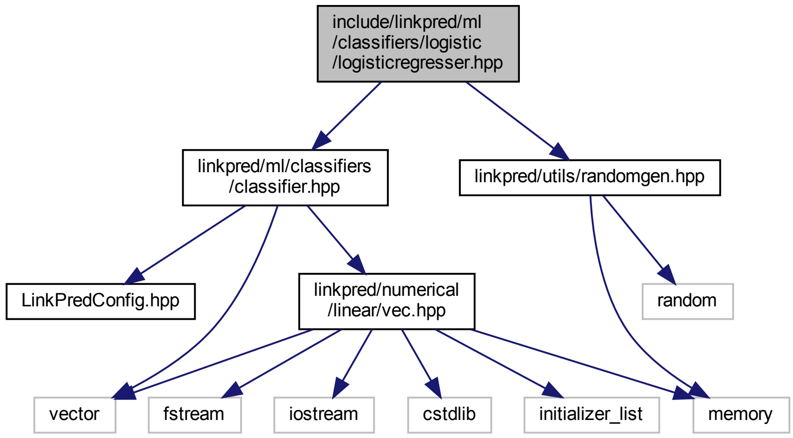

This graph shows which files directly or indirectly include this file:

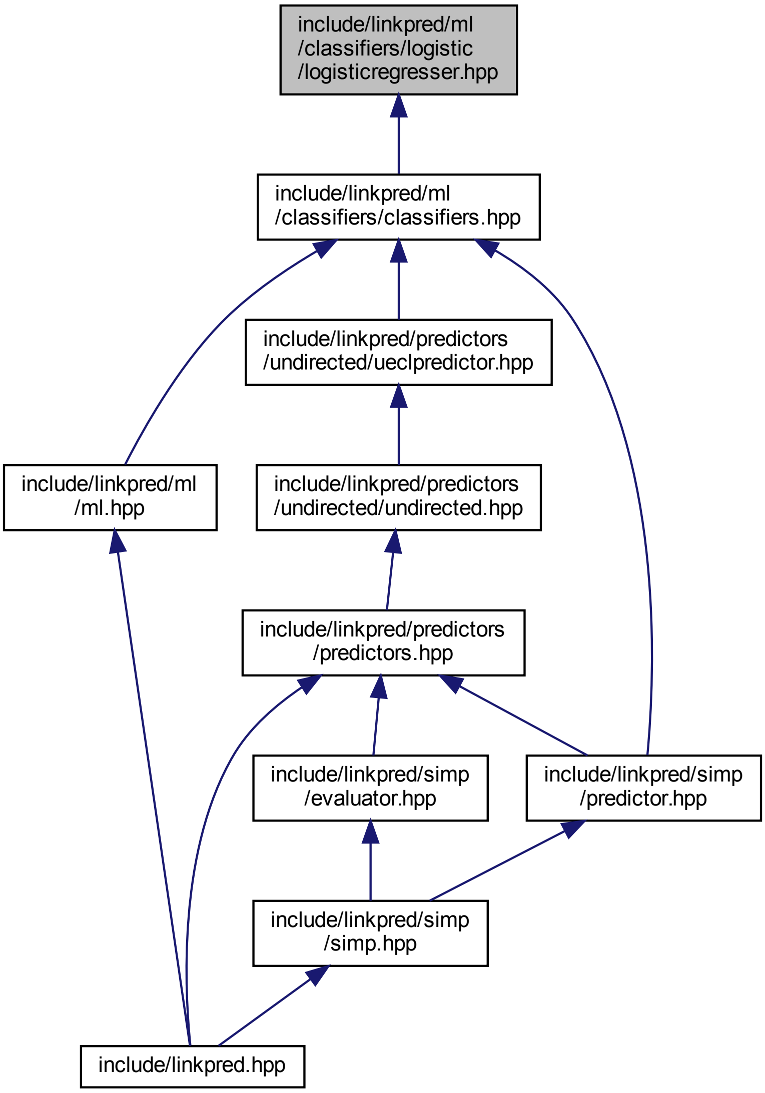

Classes

- class LinkPred::LogisticRegresser< InRndIt, OutRndIt, ScoreRndIt >

  Logistic regression algorithm.

Namespaces

- LinkPred

  Main namespace.

10.34.1 Detailed Description

Contains the implementation of a logistic regression algorithm.

10.35 include/linkpred/ml/classifiers/logistic/logregcg.hpp File Reference

Contains the implementation of a logistic regression optimization problem.

```
#include "linkpred/numerical/cg/cgdescent.hpp"
#include "linkpred/utils/randomgen.hpp"
#include "linkpred/numerical/linear/vec.hpp"
#include <vector>
```

Include dependency graph for logregcg.hpp:

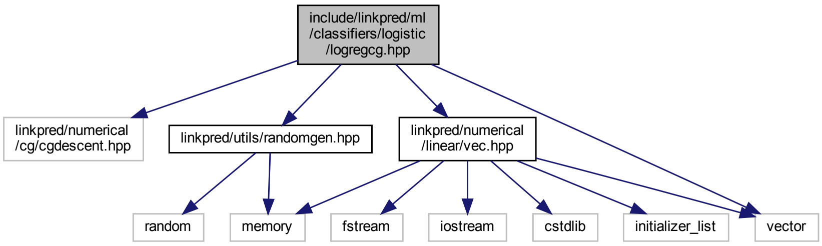

Classes

- class LinkPred::LogRegCG< InRndIt, OutRndIt >

  Logistic regression optimization problem.

Namespaces

- LinkPred

  Main namespace.

10.35.1 Detailed Description

Contains the implementation of a logistic regression optimization problem.

10.36 include/linkpred/ml/classifiers/naivebayes/naivebayes.hpp File Reference

Contains a wrapper of mlpack::maive_bayes::NaiveBayesClassifier.

```
#include "LinkPredConfig.hpp"
```

Include dependency graph for naivebayes.hpp:

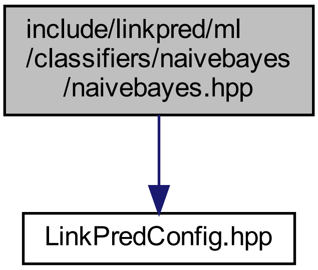

This graph shows which files directly or indirectly include this file:

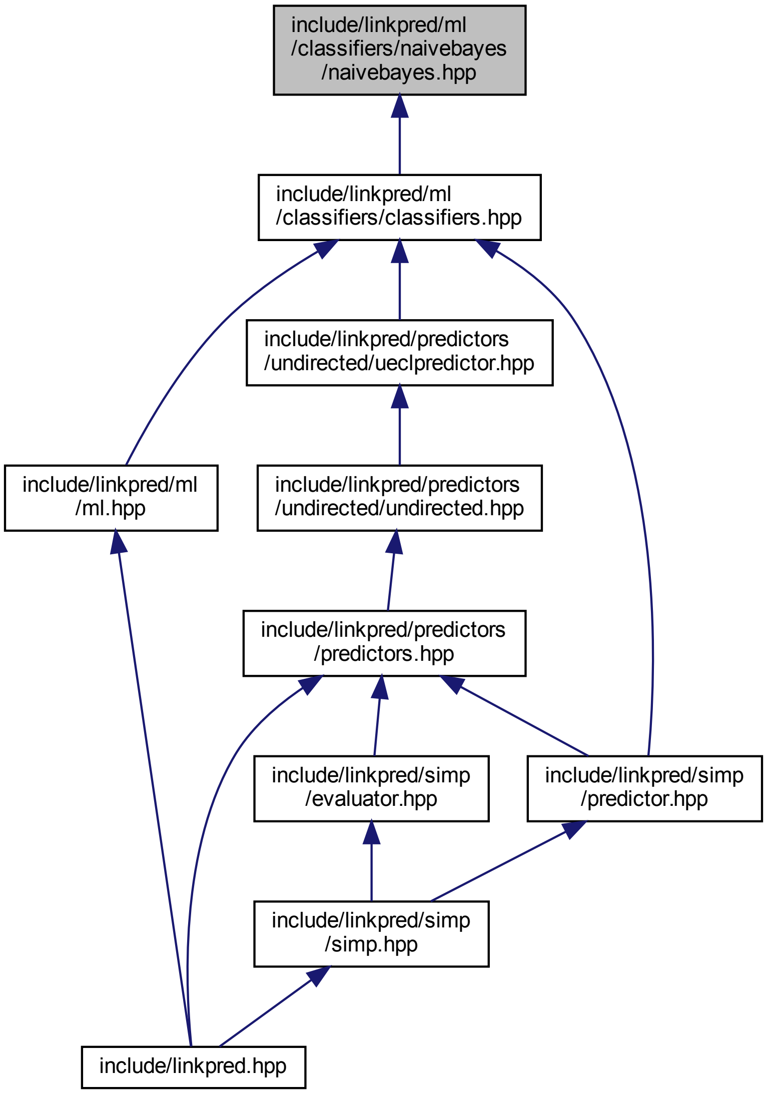

10.36.1 Detailed Description

Contains a wrapper of mlpack::maive_bayes::NaiveBayesClassifier.

10.37 include/linkpred/ml/classifiers/rndclassifier/rndclassifier.hpp File Reference

Contains a random classiffier (for debugging purposes).

```
#include "LinkPredConfig.hpp"
#include "linkpred/ml/classifiers/classifier.hpp"
```

`#include "linkpred/utils/randomgen.hpp"`

Include dependency graph for rndclassifier.hpp:

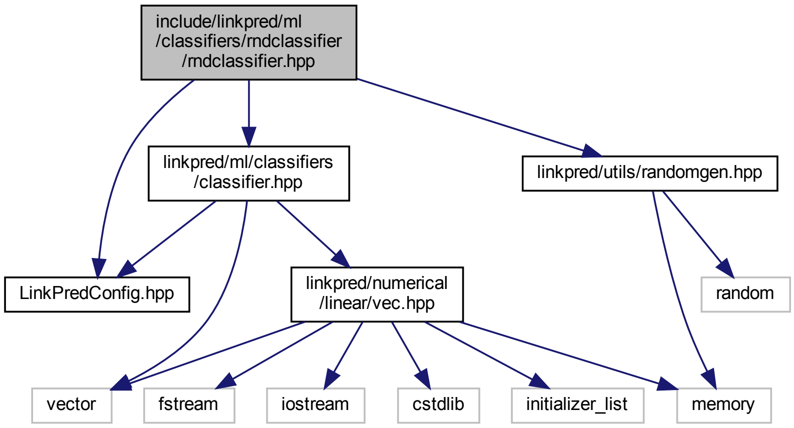

This graph shows which files directly or indirectly include this file:

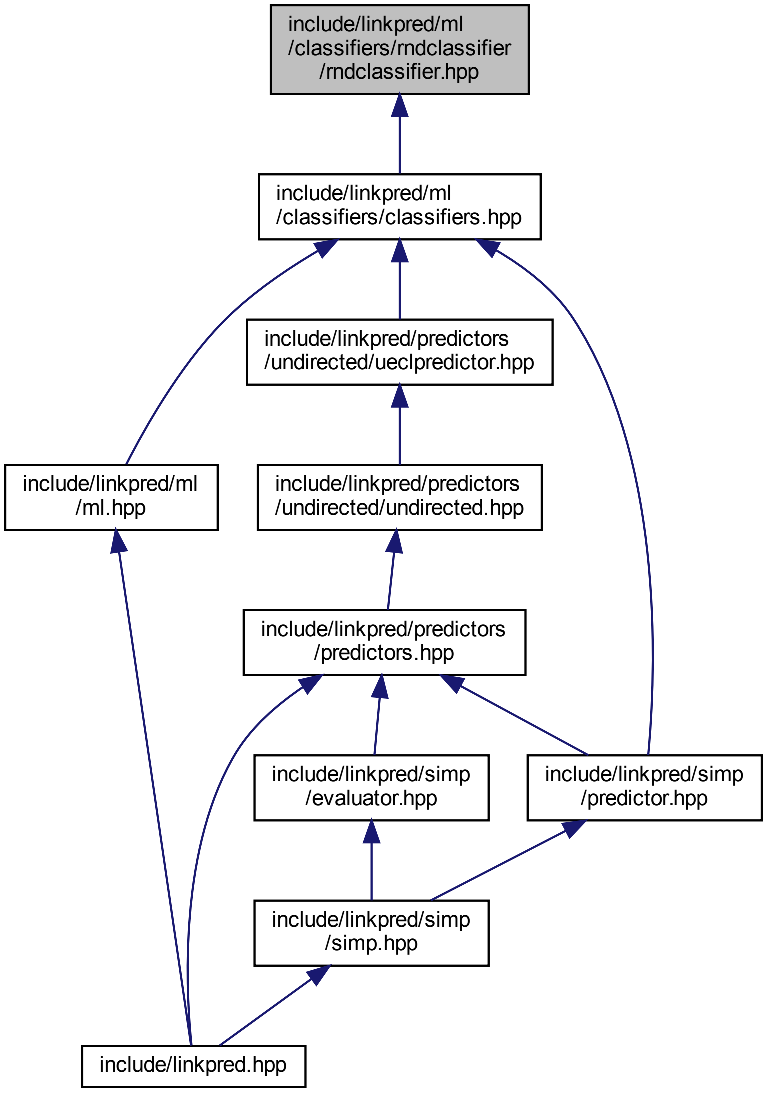

Classes

- class LinkPred::RndClassifier< InRndIt, OutRndIt, ScoreRndIt >

    Random classifier.

Namespaces

- LinkPred

    Main namespace.

10.37.1 Detailed Description

Contains a random classiffier (for debugging purposes).

10.38 include/linkpred/ml/ml.hpp File Reference

Includes the headers related to learning-based predictors.

```
#include "linkpred/ml/classifiers/classifiers.hpp"
#include "linkpred/ml/simmeasures/simmeasures.hpp"
```

Include dependency graph for ml.hpp:

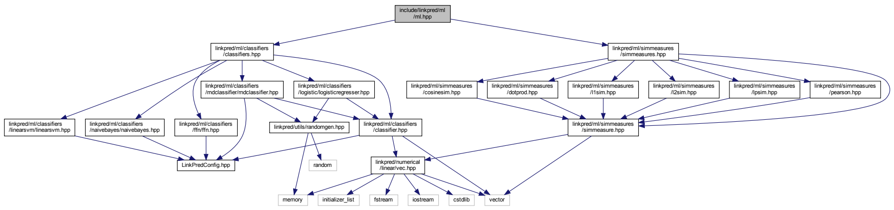

This graph shows which files directly or indirectly include this file:

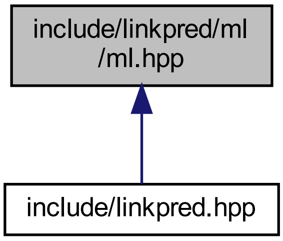

10.38.1 Detailed Description

Includes the headers related to learning-based predictors.

10.39 include/linkpred/ml/simmeasures/cosinesim.hpp File Reference

Contains the implementation of cosine similarity.

`#include "linkpred/ml/simmeasures/simmeasure.hpp"`

Include dependency graph for cosinesim.hpp:

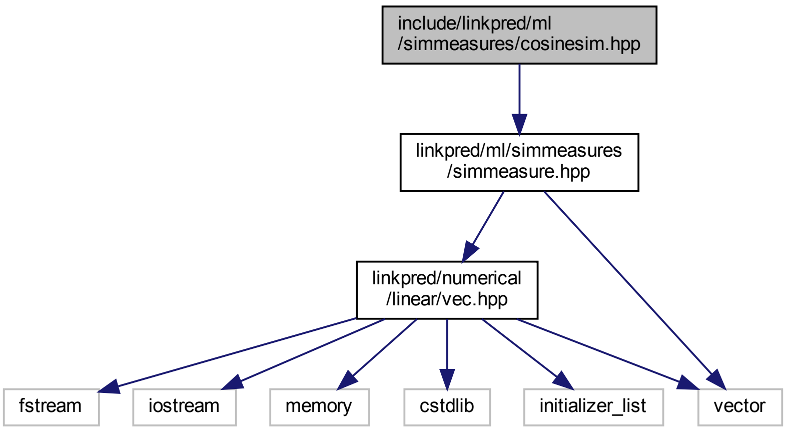

This graph shows which files directly or indirectly include this file:

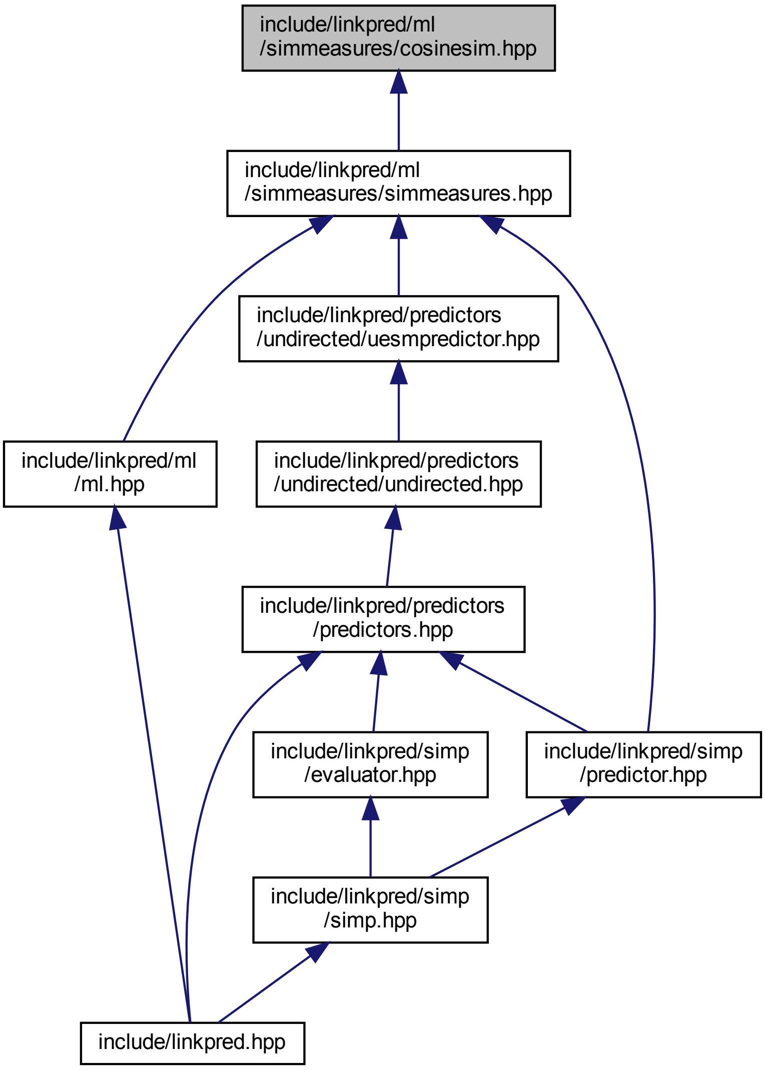

Classes

- class LinkPred::CosineSim

  Cosine similarity.

Namespaces

- LinkPred

  Main namespace.

10.39.1 Detailed Description

Contains the implementation of cosine similarity.

10.40 include/linkpred/ml/simmeasures/dotprod.hpp File Reference

Contains the implementation of the dot product similarity measure.

```
#include "linkpred/ml/simmeasures/simmeasure.hpp"
```

Include dependency graph for dotprod.hpp:

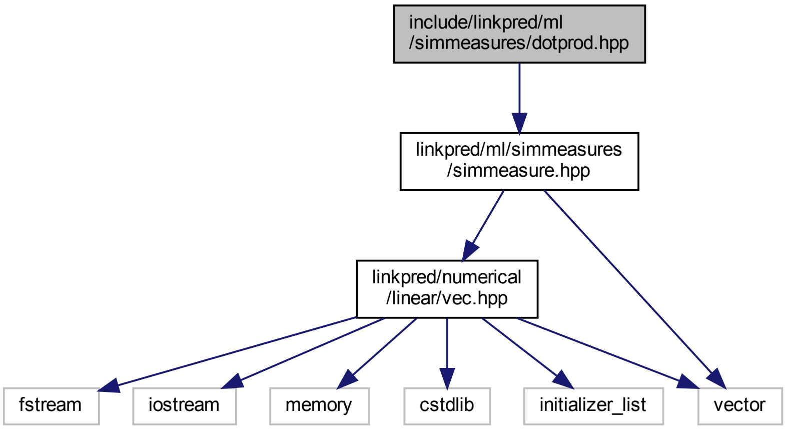

This graph shows which files directly or indirectly include this file:

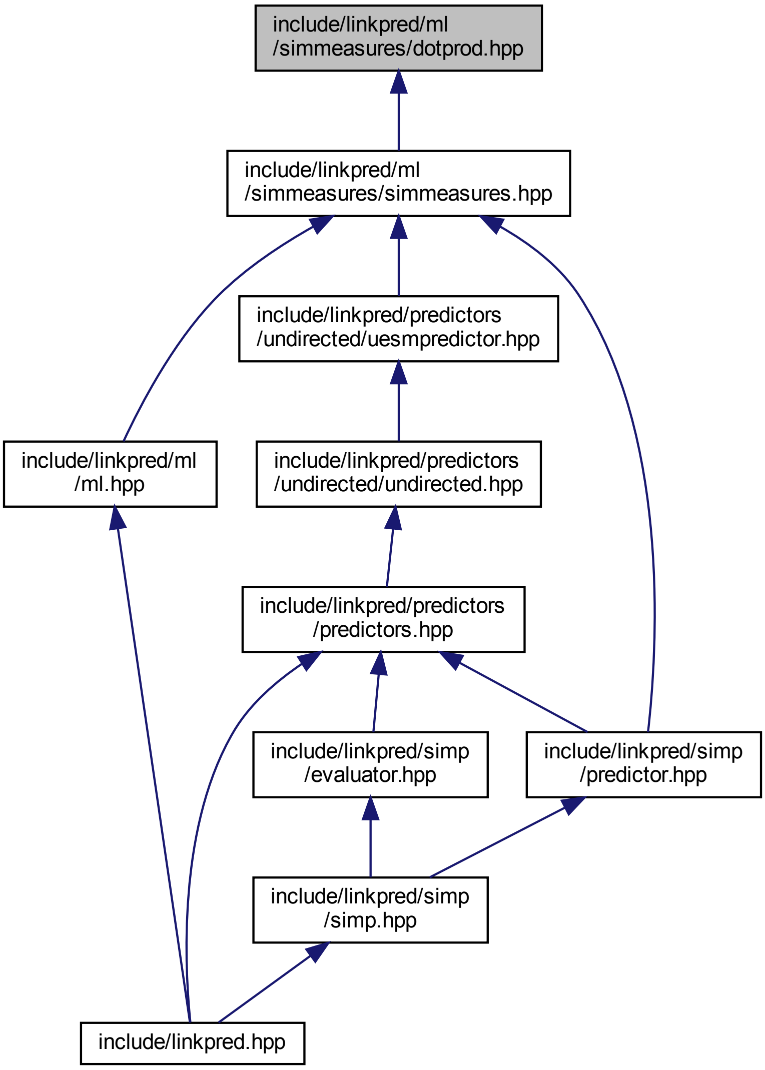

Classes

- class LinkPred::DotProd

  A simple dot product similarity measure.

Namespaces

- LinkPred

  Main namespace.

10.40.1 Detailed Description

Contains the implementation of the dot product similarity measure.

10.41 include/linkpred/ml/simmeasures/l1sim.hpp File Reference

Contains the implementation of L1 similarity.

```
#include "linkpred/ml/simmeasures/simmeasure.hpp"
```

Include dependency graph for l1sim.hpp:

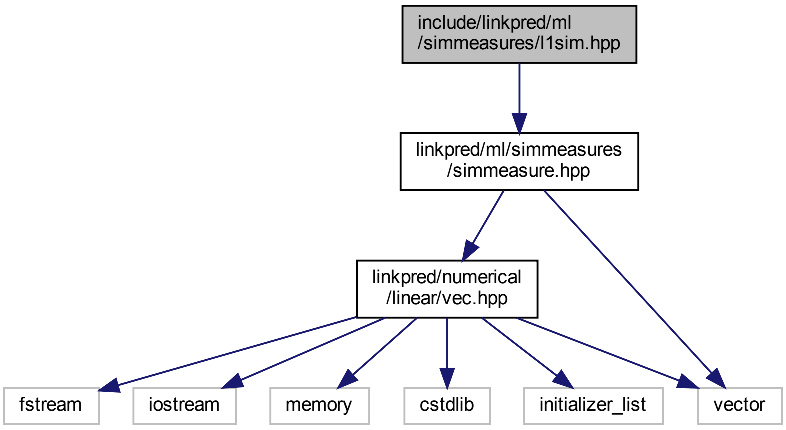

This graph shows which files directly or indirectly include this file:

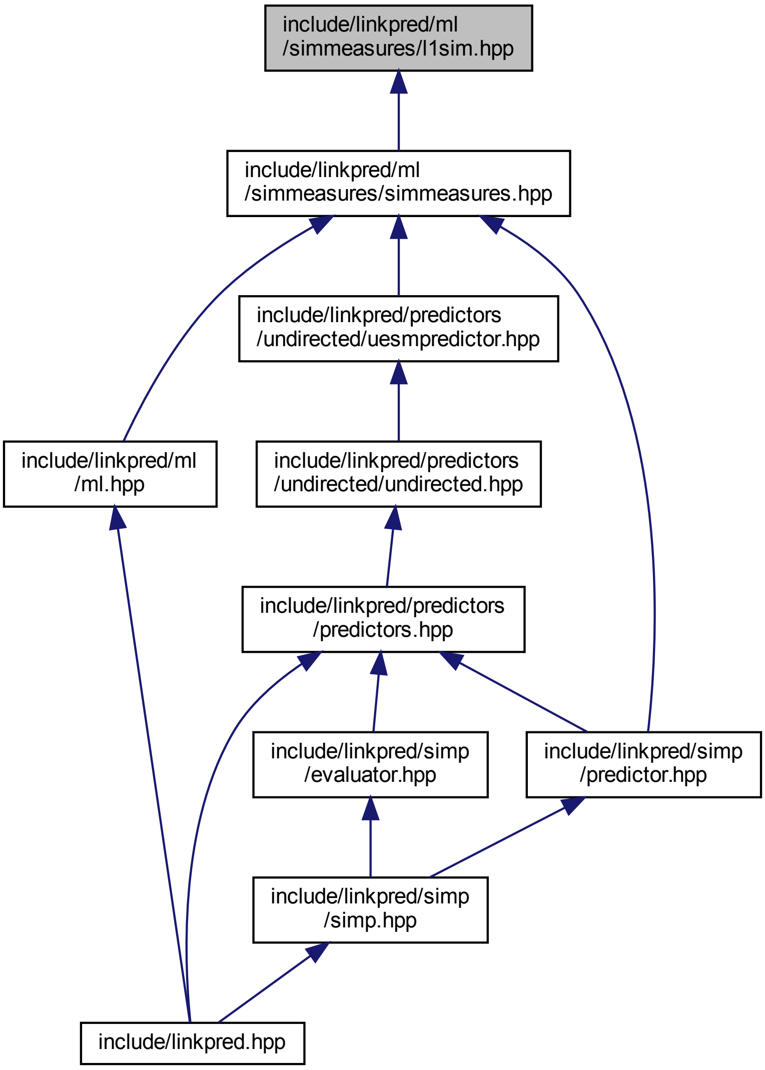

Classes

- class LinkPred::L1Sim

  L1 similarity (negative the L1 norm or Manhattan distance).

Namespaces

- LinkPred

  Main namespace.

10.41.1 Detailed Description

Contains the implementation of L1 similarity.

10.42 include/linkpred/ml/simmeasures/l2sim.hpp File Reference

Contains the implementation of L2 similarity.

```
#include "linkpred/ml/simmeasures/simmeasure.hpp"
```

Include dependency graph for l2sim.hpp:

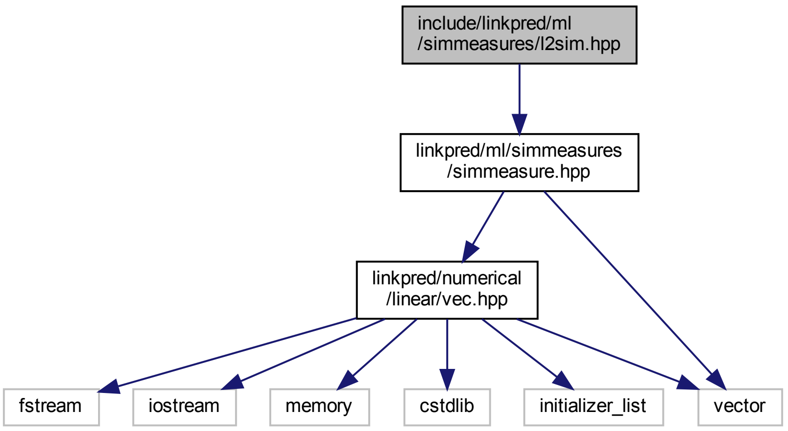

This graph shows which files directly or indirectly include this file:

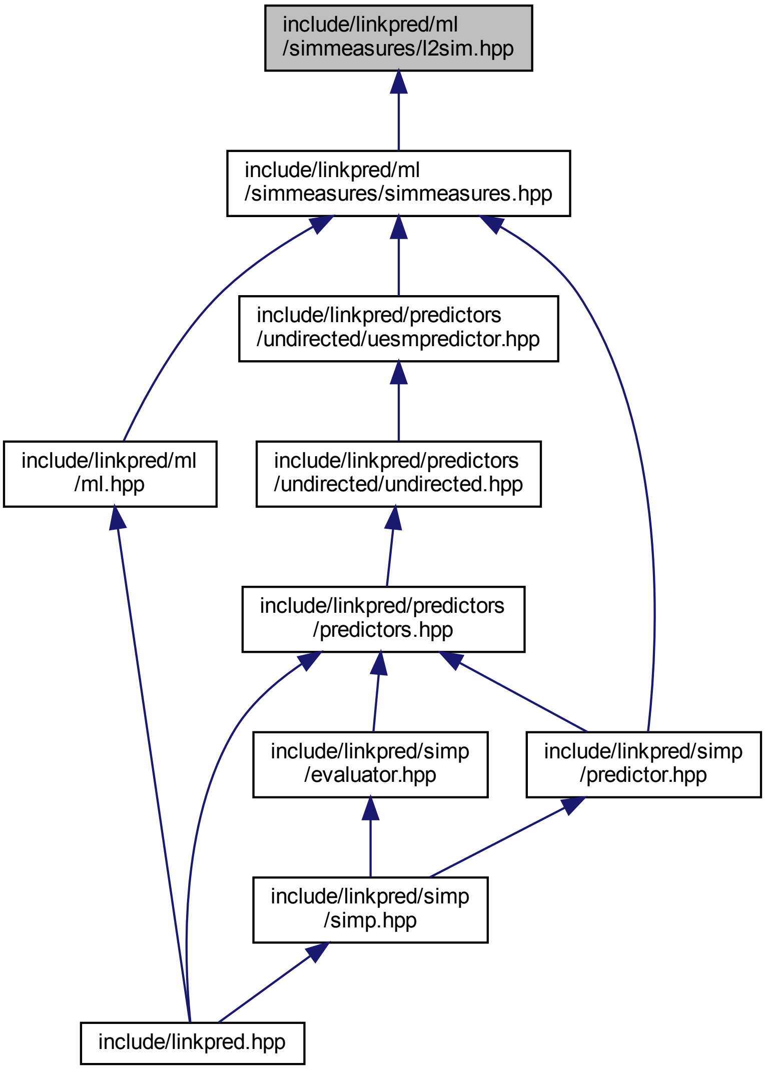

Classes

- class LinkPred::L2Sim

  L2 similarity (negative the Euclidean distance).

Namespaces

- LinkPred

  Main namespace.

10.42.1 Detailed Description

Contains the implementation of L2 similarity.

10.43 include/linkpred/ml/simmeasures/lpsim.hpp File Reference

Contains the implementation of LP similarity.

```
#include "linkpred/ml/simmeasures/simmeasure.hpp"
```

Include dependency graph for lpsim.hpp:

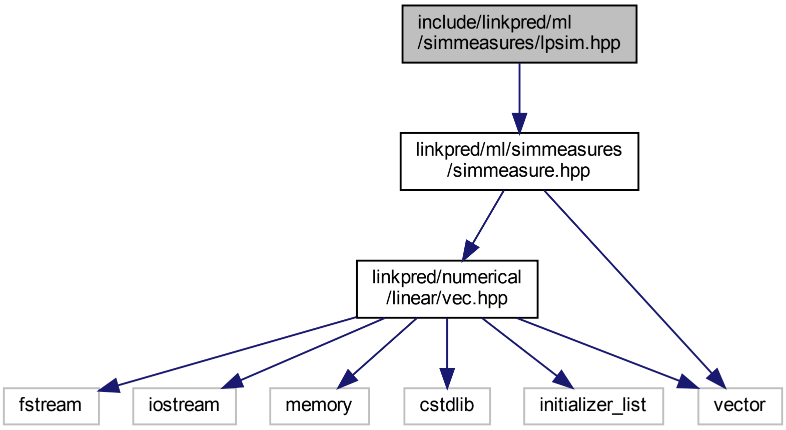

This graph shows which files directly or indirectly include this file:

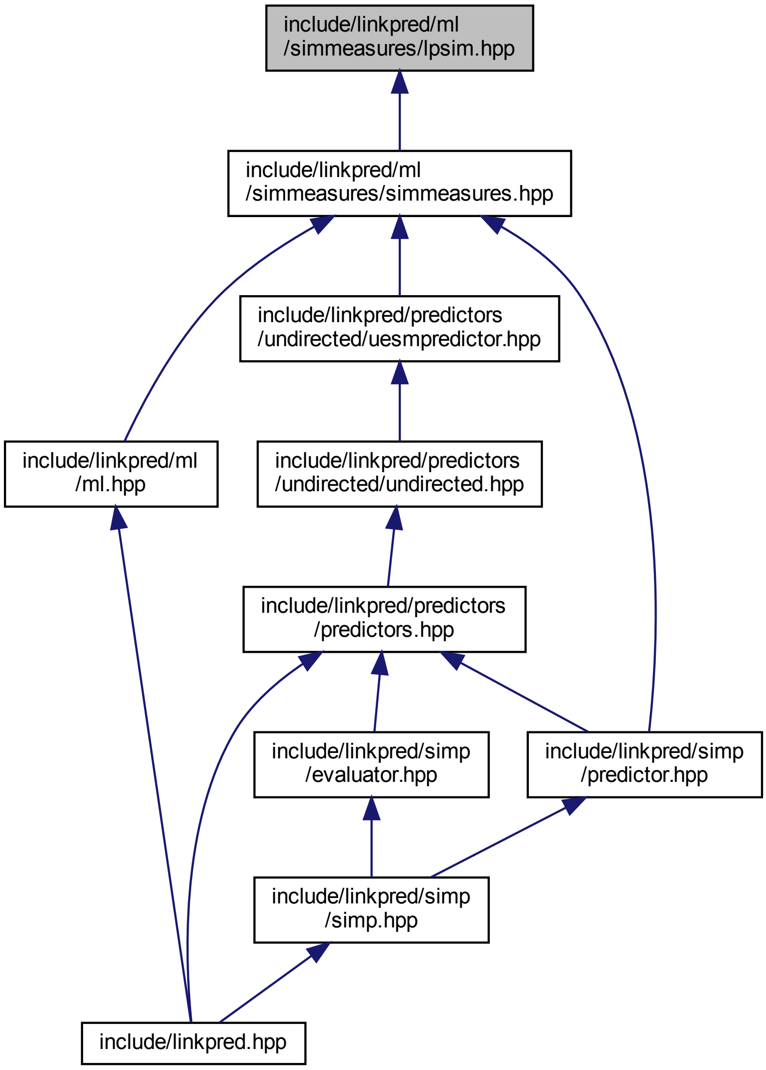

Classes

- class LinkPred::LPSim

    LP similarity (negative the Lp norm).

Namespaces

- LinkPred

    Main namespace.

10.43.1 Detailed Description

Contains the implementation of LP similarity.

10.44 include/linkpred/ml/simmeasures/pearson.hpp File Reference

Contains the implementation of Perason similarity.

```
#include "linkpred/ml/simmeasures/simmeasure.hpp"
```

Include dependency graph for pearson.hpp:

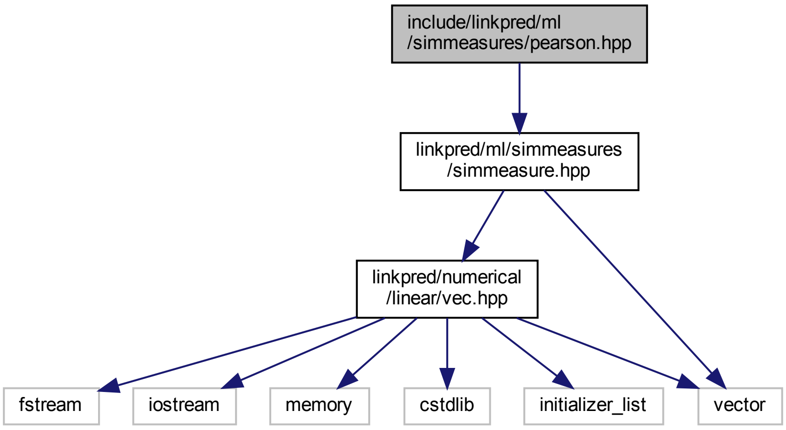

This graph shows which files directly or indirectly include this file:

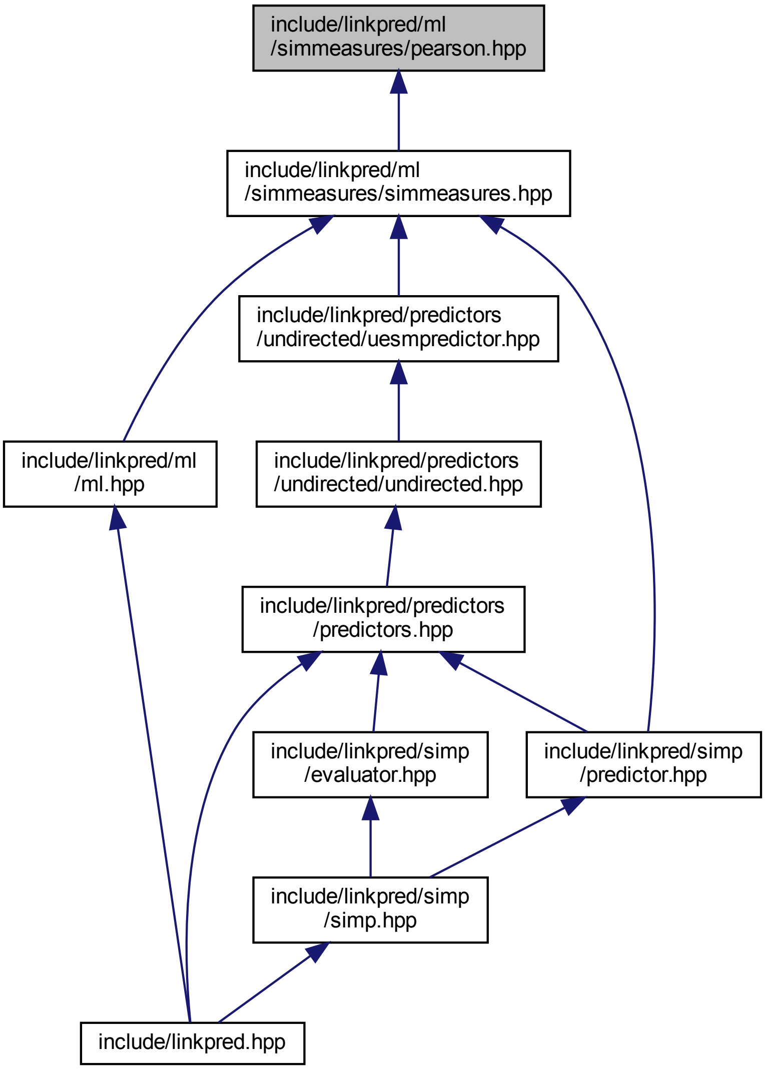

Classes

- class LinkPred::Pearson

    Pearson similarity (Pearson correlation coefficient).

Namespaces

- LinkPred

    Main namespace.

10.44.1 Detailed Description

Contains the implementation of Perason similarity.

10.45 include/linkpred/ml/simmeasures/simmeasure.hpp File Reference

Contains the interface of a similarity measure.

```
#include "linkpred/numerical/linear/vec.hpp"
#include <vector>
```

Include dependency graph for simmeasure.hpp:

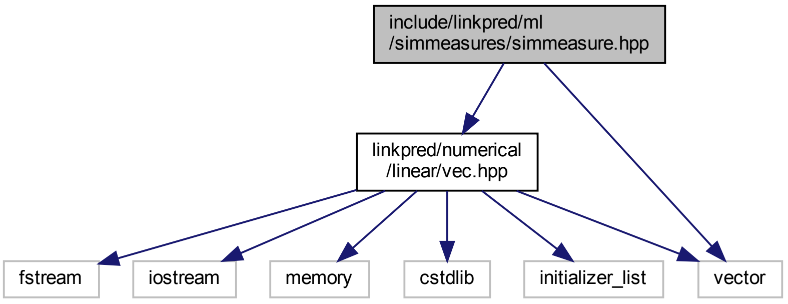

This graph shows which files directly or indirectly include this file:

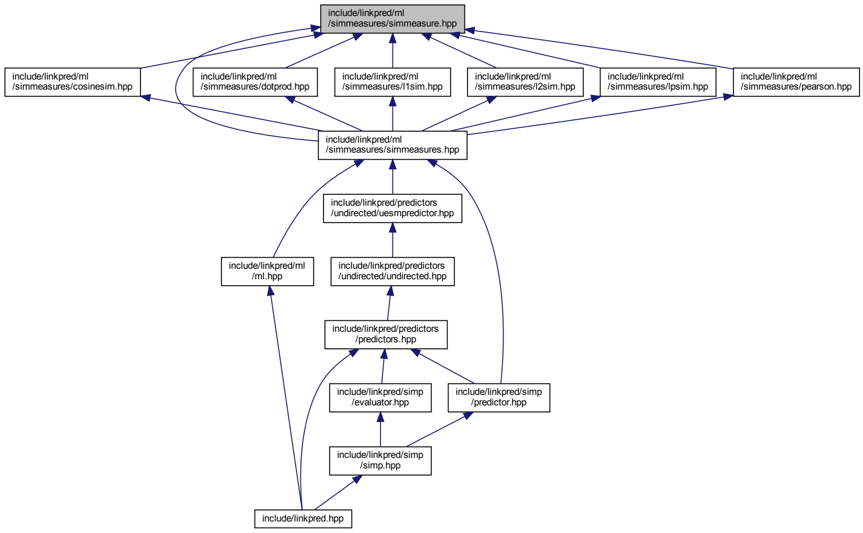

Classes

- class LinkPred::SimMeasure

  Interface of a similarity measure.

Namespaces

- LinkPred

  Main namespace.

10.45.1 Detailed Description

Contains the interface of a similarity measure.

10.46 include/linkpred/ml/simmeasures/simmeasures.hpp File Reference

Includes the headers related to similarity measures.

```
#include "linkpred/ml/simmeasures/simmeasure.hpp"
#include "linkpred/ml/simmeasures/cosinesim.hpp"
#include "linkpred/ml/simmeasures/dotprod.hpp"
#include "linkpred/ml/simmeasures/l1sim.hpp"
#include "linkpred/ml/simmeasures/l2sim.hpp"
#include "linkpred/ml/simmeasures/lpsim.hpp"
#include "linkpred/ml/simmeasures/pearson.hpp"
```

Include dependency graph for simmeasures.hpp:

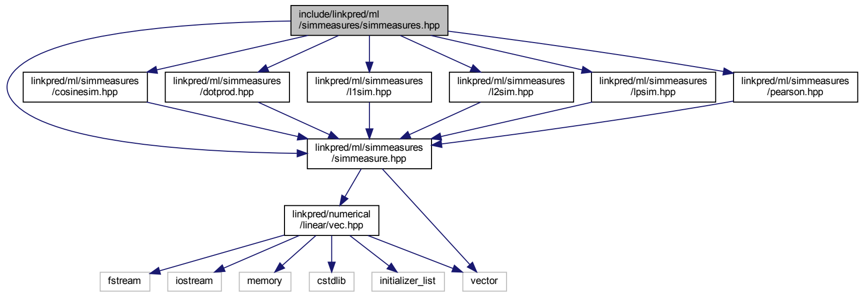

This graph shows which files directly or indirectly include this file:

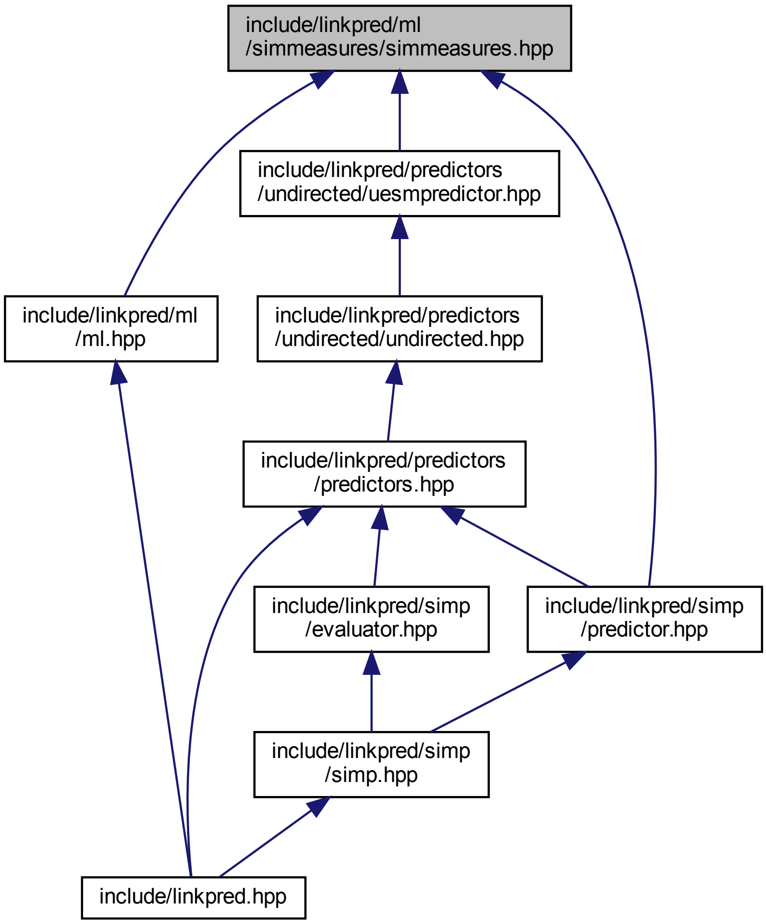

10.46.1 Detailed Description

Includes the headers related to similarity measures.

10.47 include/linkpred/numerical/linear/gfmatrix.hpp File Reference

Contains the implementation of a full matrix.

```
#include <cstdlib>
#include <vector>
```

`#include "linkpred/numerical/linear/vec.hpp"`

Include dependency graph for gfmatrix.hpp:

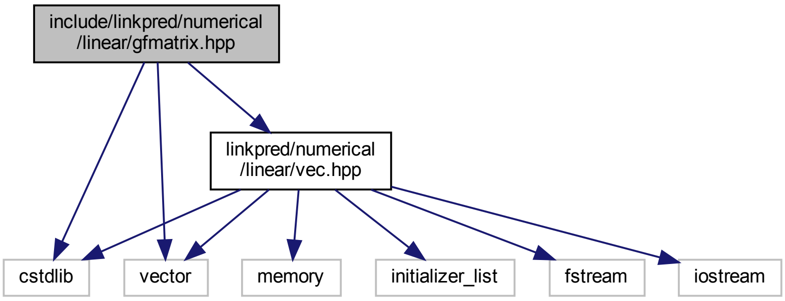

This graph shows which files directly or indirectly include this file:

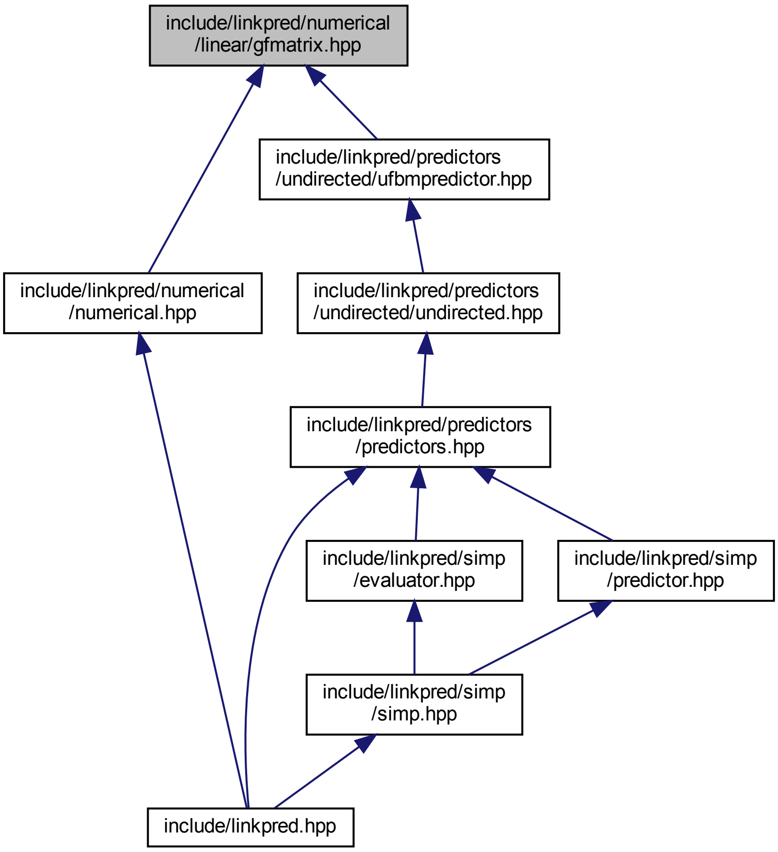

Classes

- class LinkPred::GFMatrix

Generalized full matrix. The storage scheme used is column-major.

Namespaces

- LinkPred

  Main namespace.

10.47.1 Detailed Description

Contains the implementation of a full matrix.

10.48 include/linkpred/numerical/linear/vec.hpp File Reference

Contains the implementation of a vector (in the sense of linear algebra).

```
#include <fstream>
#include <iostream>
#include <memory>
#include <vector>
#include <cstdlib>
#include <initializer_list>
```

Include dependency graph for vec.hpp:

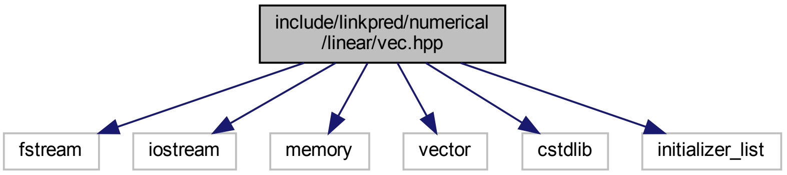

This graph shows which files directly or indirectly include this file:

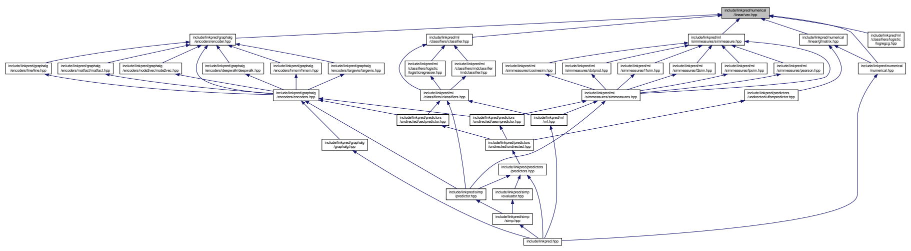

Classes

- class LinkPred::Vec

  This class represent a vector (in the sense of linear algebra).

Namespaces

- LinkPred

  Main namespace.

Functions

- Vec LinkPred::operator+ (Vec const &v1, Vec const &v2)
- Vec LinkPred::operator- (Vec const &v1, Vec const &v2)
- Vec LinkPred::operator* (Vec const &v1, Vec const &v2)
- Vec LinkPred::operator/ (Vec const &v1, Vec const &v2)
- Vec LinkPred::operator+ (double a, Vec const &v)
- Vec LinkPred::operator- (double a, Vec const &v)
- Vec LinkPred::operator* (double a, Vec const &v)
- Vec LinkPred::operator/ (double a, Vec const &v)
- Vec LinkPred::operator+ (Vec const &v, double a)
- Vec LinkPred::operator- (Vec const &v, double a)
- Vec LinkPred::operator* (Vec const &v, double a)
- Vec LinkPred::operator/ (Vec const &v, double a)
- double LinkPred::operator^ (Vec const &v1, Vec const &v2)

10.48.1 Detailed Description

Contains the implementation of a vector (in the sense of linear algebra).

10.49 include/linkpred/numerical/mds/logmdscg.hpp File Reference

Contains the implementation of the optimization problem associated with multidimensional scaling using the logarithmic (MULTISCALE) loss function.

```
#include "linkpred/numerical/cg/cgdescent.hpp"
#include "linkpred/utils/randomgen.hpp"
```

Include dependency graph for logmdscg.hpp:

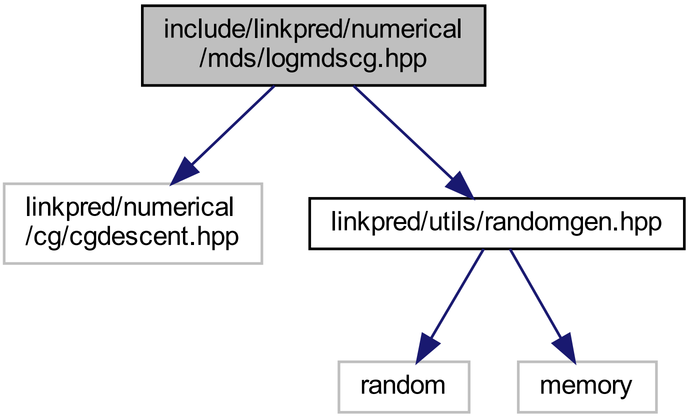

This graph shows which files directly or indirectly include this file:

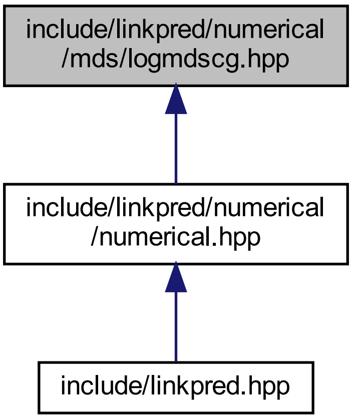

Classes

- class LinkPred::LogMDSCG

  Optimization problem associated with multidimensional scaling using the logarithmic (MULTISCALE) loss function.

Namespaces

- LinkPred

  Main namespace.

10.49.1 Detailed Description

Contains the implementation of the optimization problem associated with multidimensional scaling using the logarithmic (MULTISCALE) loss function.

10.50 include/linkpred/numerical/mds/mds.hpp File Reference

Contains the implementation of a solver of the multidimensional scaling problem using the logarithmic (MULTISC↩ ALE) loss function.

`#include <cstdlib>`

Include dependency graph for mds.hpp:

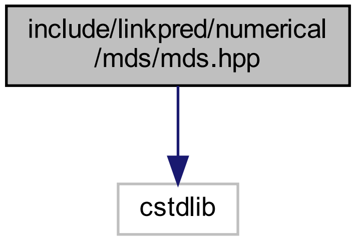

This graph shows which files directly or indirectly include this file:

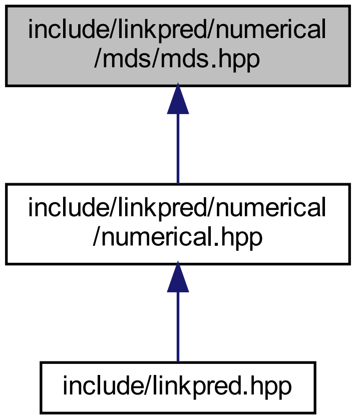

Classes

- class LinkPred::MDS

    Solve the MDS problem.

Namespaces

- LinkPred

    Main namespace.

Enumerations

- enum LinkPred::MDSAlg { LinkPred::IpoptMDS, LinkPred::CGMDS }

    MDS solution methods.

10.50.1 Detailed Description

Contains the implementation of a solver of the multidimensional scaling problem using the logarithmic (MULTISC←ALE) loss function.

10.51 include/linkpred/numerical/mf/fastsig.hpp File Reference

Contains the implementation of a fast sigmoid.

```
#include <type_traits>
#include <vector>
#include <cmath>
```

Include dependency graph for fastsig.hpp:

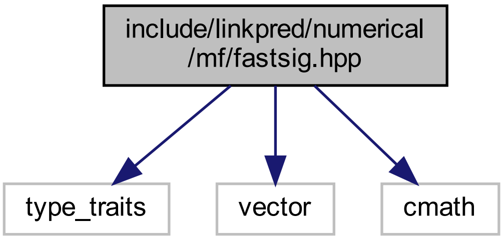

This graph shows which files directly or indirectly include this file:

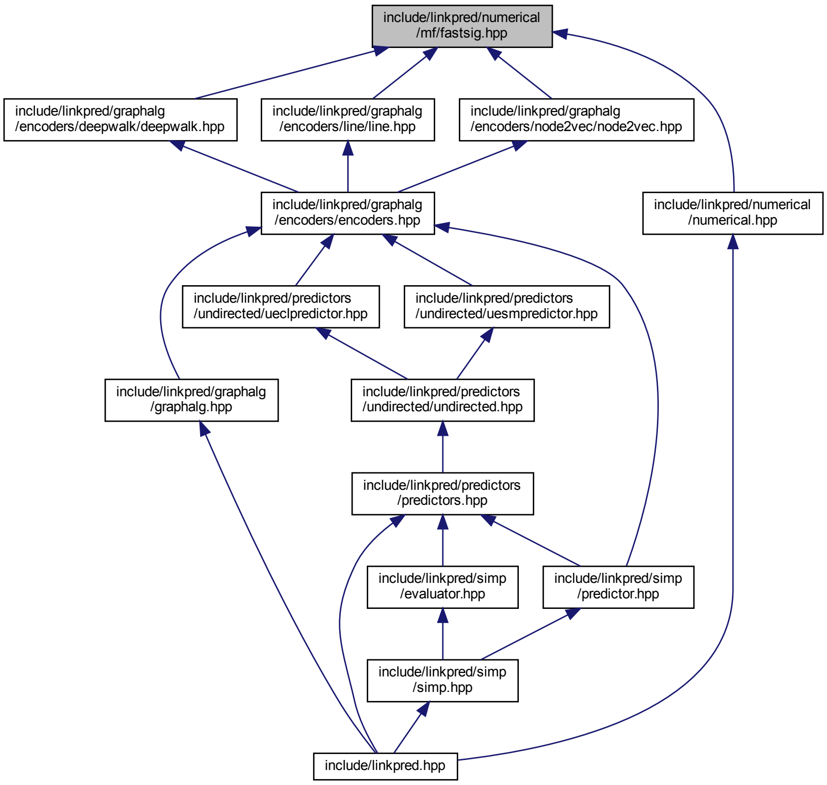

Classes

- class LinkPred::FastSig< T >

  A fast sigmoid.

Namespaces

- LinkPred

  Main namespace.

10.51.1 Detailed Description

Contains the implementation of a fast sigmoid.

10.52 include/linkpred/numerical/numerical.hpp File Reference

Includes the headers related to numerical algorithms.

```
#include "linkpred/numerical/mf/fastsig.hpp"
#include "linkpred/numerical/mds/logmdscg.hpp"
#include "linkpred/numerical/mds/mds.hpp"
#include "linkpred/numerical/linear/gfmatrix.hpp"
#include "linkpred/numerical/linear/vec.hpp"
#include "linkpred/numerical/cg/cgblas.hpp"
#include "linkpred/numerical/cg/cgdescent.hpp"
#include "linkpred/numerical/plfit/plfit.hpp"
```

Include dependency graph for numerical.hpp:

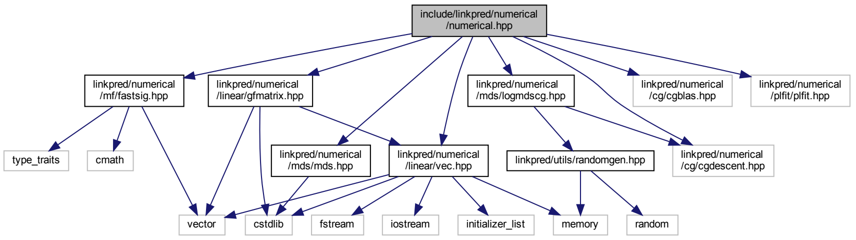

This graph shows which files directly or indirectly include this file:

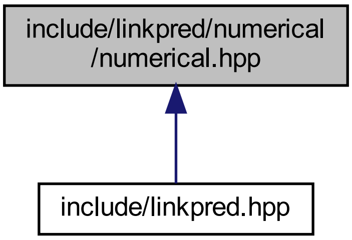

10.52.1 Detailed Description

Includes the headers related to numerical algorithms.

10.53 include/linkpred/perf/networkmanipulator.hpp File Reference

Contains the implementation of test data related classes.

```
#include "LinkPredConfig.hpp"
#include <linkpred/utils/miscutils.hpp>
#include "linkpred/core/unetwork/unetwork.hpp"
#include "linkpred/core/dnetwork/dnetwork.hpp"
#include "linkpred/graphalg/traversal/graphtraversal.hpp"
#include "linkpred/utils/log.hpp"
#include <memory>
#include <vector>
#include <set>
#include <iterator>
```

Include dependency graph for networkmanipulator.hpp:

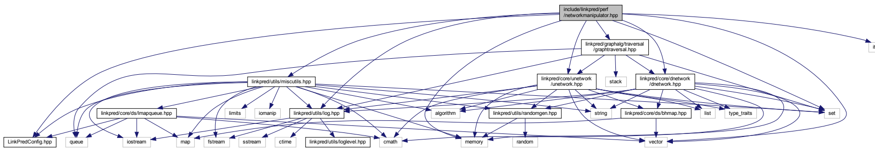

This graph shows which files directly or indirectly include this file:

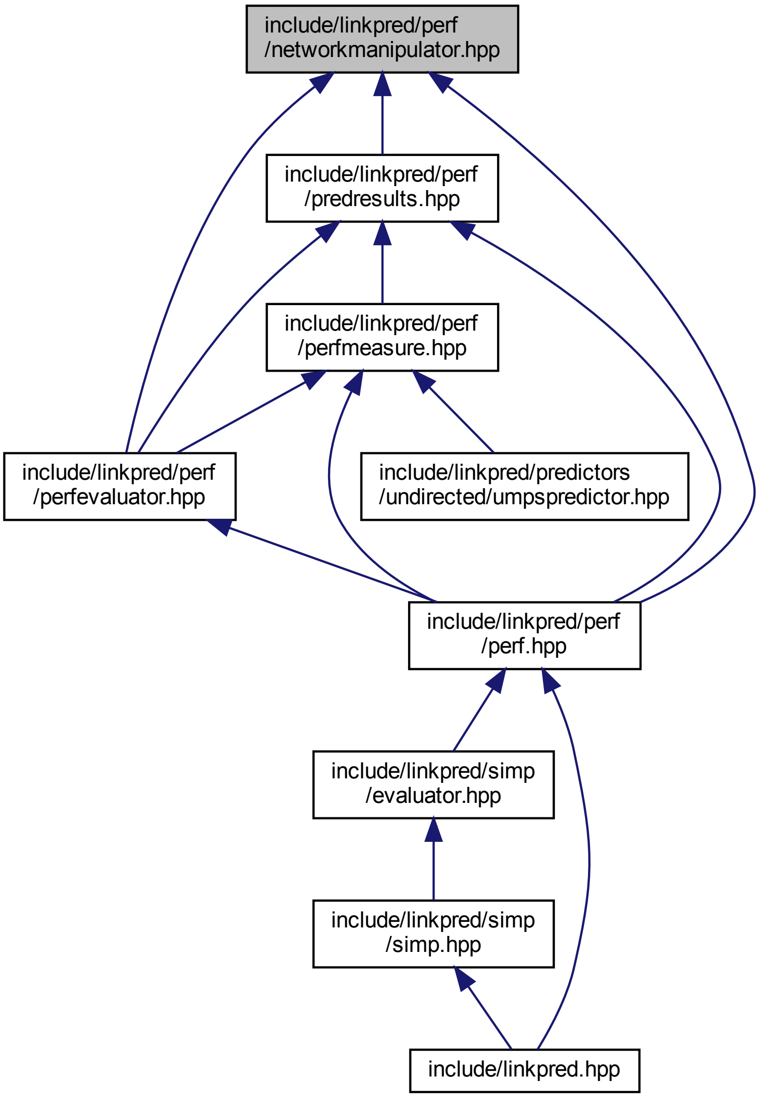

Classes

- class LinkPred::TestEdgeGen< Network, EdgeContT >
  Generate true positives and true negatives.
- class LinkPred::TestEdgeGen< Network, EdgeContT >::TPEdgeIt
  TP edges iterator.
- class LinkPred::TestEdgeGen< Network, EdgeContT >::TNEdgeIt
  TN edges iterator.
- class LinkPred::TestData< Network, EdgeContT >
  Test data.
- class LinkPred::TestData< Network, EdgeContT >::TestEdgeIt
  Test edges iterator.

- class LinkPred::NetworkManipulator< Network >

    Class to manipulate network by removing or adding edges.

Namespaces

- LinkPred

    Main namespace.

Enumerations

- enum LinkPred::LinkClass { LinkPred::TP, LinkPred::FN, LinkPred::FP, LinkPred::TN }

    Enumeration of all classes of links.

10.53.1 Detailed Description

Contains the implementation of test data related classes.

10.54 include/linkpred/perf/perf.hpp File Reference

Includes the headers related to performance evaluation classes.

```
#include "linkpred/perf/predresults.hpp"
#include "linkpred/perf/perfmeasure.hpp"
#include "linkpred/perf/networkmanipulator.hpp"
#include "linkpred/perf/perfevaluator.hpp"
```

Include dependency graph for perf.hpp:

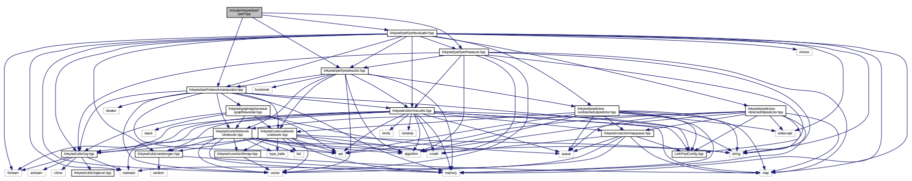

This graph shows which files directly or indirectly include this file:

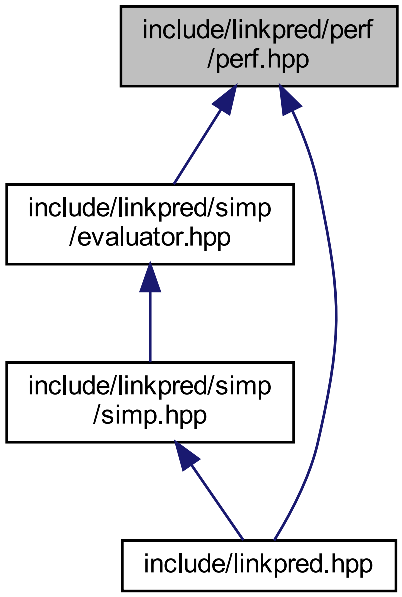

10.54.1 Detailed Description

Includes the headers related to performance evaluation classes.

10.55 include/linkpred/perf/perfevaluator.hpp File Reference

Includes the headers related to core classes.

```
#include <linkpred/predictors/undirected/ulpredictor.hpp>
#include <linkpred/utils/miscutils.hpp>
#include "linkpred/predictors/directed/dlpredictor.hpp"
#include "linkpred/perf/predresults.hpp"
#include "linkpred/perf/perfmeasure.hpp"
#include "linkpred/perf/networkmanipulator.hpp"
#include "linkpred/utils/randomgen.hpp"
#include "linkpred/utils/log.hpp"
#include <string>
#include <memory>
#include <vector>
#include <chrono>
#include <map>
#include <stdexcept>
#include <iostream>
```

`#include <fstream>`

Include dependency graph for perfevaluator.hpp:

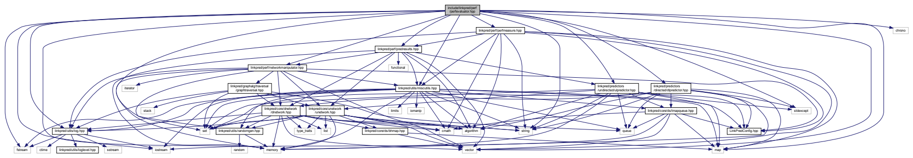

This graph shows which files directly or indirectly include this file:

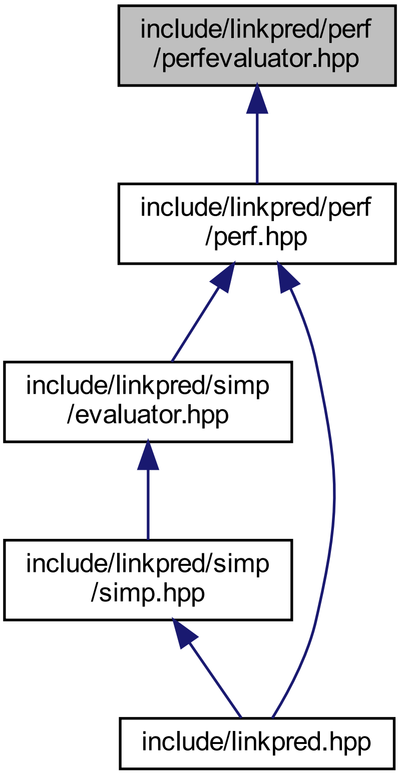

Classes

- class LinkPred::PerfEvaluator< TestDataT, LPredictorT, PredResultsT, PerfMeasureT >

  Performance evaluator.
- struct LinkPred::PerfeEvalExpDescp< Network >

  Structure storing experiment description.
- class LinkPred::PEFactory< Network, LPredictorT, TestDataT, PerfMeasureT >

  Factory class to create link predictors and performance measures.
- class LinkPred::PerfEvalExp< Network, TestDataT, LPredictorT, PredResultsT, PerfMeasureT >

  Performance evaluation experiment.

Namespaces

- LinkPred

  Main namespace.

10.55.1 Detailed Description

Includes the headers related to core classes.

10.56 include/linkpred/perf/perfmeasure.hpp File Reference

Contains the implementation of several performance measures.

```
#include "LinkPredConfig.hpp"
#include <linkpred/utils/miscutils.hpp>
#include "linkpred/perf/predresults.hpp"
#include "linkpred/utils/log.hpp"
#include <vector>
#include <algorithm>
#include <map>
#include <string>
#include <cmath>
```

Include dependency graph for perfmeasure.hpp:

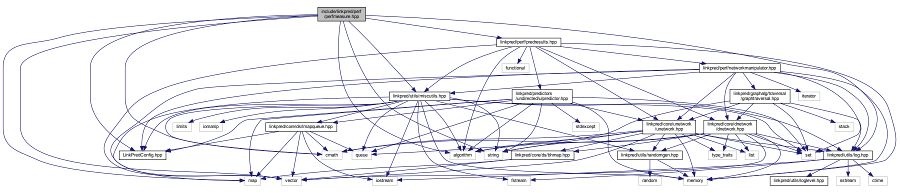

This graph shows which files directly or indirectly include this file:

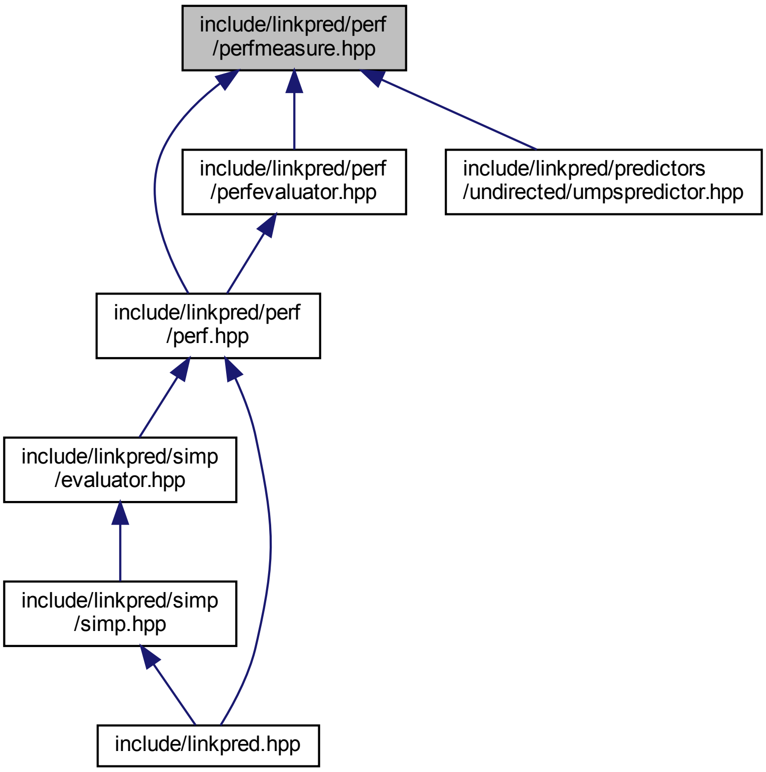

Classes

- class LinkPred::PerfMeasure< PredResultsT >

  Abstract performance measure.
- class LinkPred::PerfCurve< PredResultsT >

  Abstract performance curve.
- class LinkPred::ROC< PredResultsT >

  Receiver Operating Characteristic curve.
- class LinkPred::PR< PredResultsT >

  The precision recall curve.
- class LinkPred::TPR< PredResultsT >

  Compute top precision.
- class LinkPred::GCurve< PredResultsT >

  General performance curve.

Namespaces

- LinkPred

  Main namespace.

Typedefs

- using LinkPred::PerfResults = std::map< std::string, double >
- using LinkPred::PerfLambda::PerfLambdaT = std::function< double(std::size_t tp, std::size_t fn, std::size_t tn, std::size_t fp, std::size_t P, std::size_t N)>

Variables

- auto LinkPred::PerfLambda::rec
- auto LinkPred::PerfLambda::fpr
- auto LinkPred::PerfLambda::pre
- auto LinkPred::PerfLambda::fnr
- auto LinkPred::PerfLambda::tnr
- auto LinkPred::PerfLambda::fmr
- auto LinkPred::PerfLambda::acc
- auto LinkPred::PerfLambda::fdr
- auto LinkPred::PerfLambda::npv

10.56.1 Detailed Description

Contains the implementation of several performance measures.

10.56.2 Typedef Documentation

10.56.2.1 PerfLambdaT

```
using LinkPred::PerfLambda::PerfLambdaT = typedef std::function<double(std::size_t tp, std↩
::size_t fn, std::size_t tn, std::size_t fp, std::size_t P, std::size_t N)>
```

Signature of performance lambdas.

10.56.3 Variable Documentation

10.56.3.1 acc

```
auto LinkPred::PerfLambda::acc
```

Initial value:

```
= [](std::size_t tp, std::size_t fn, std::size_t tn, std::size_t fp,
        std::size_t P, std::size_t N) {
    return (double) (tp + tn) / (P + N);
}
```

Accuracy.

Parameters

tp	The number of true positives.
fn	The number of false negatives.
tn	The number of true negatives.
fp	The number of false positives.
P	The number of positives.
N	The number of negatives.

10.56.3.2 fdr

```
auto LinkPred::PerfLambda::fdr
```

Initial value:

```
= [](std::size_t tp, std::size_t fn, std::size_t tn, std::size_t fp,
        std::size_t P, std::size_t N) {
    return (double) fp / (tp + fp);
}
```

False discovery rate.

Parameters

tp	The number of true positives.
fn	The number of false negatives.
tn	The number of true negatives.
fp	The number of false positives.
P	The number of positives.
N	The number of negatives.

10.56.3.3 fmr

```
auto LinkPred::PerfLambda::fmr
```

Initial value:

```
= [](std::size_t tp, std::size_t fn, std::size_t tn, std::size_t fp,
        std::size_t P, std::size_t N) {
    return (double) fn / (tn + fn);
}
```

False omission rate.

Parameters

tp	The number of true positives.
fn	The number of false negatives.
tn	The number of true negatives.
fp	The number of false positives.
P	The number of positives.
N	The number of negatives.

10.56.3.4 fnr

`auto LinkPred::PerfLambda::fnr`

Initial value:
```
= [](std::size_t tp, std::size_t fn, std::size_t tn, std::size_t fp,
        std::size_t P, std::size_t N) {
    return (double) fn / P;
}
```

False negative rate.

Parameters

tp	The number of true positives.
fn	The number of false negatives.
tn	The number of true negatives.
fp	The number of false positives.
P	The number of positives.
N	The number of negatives.

10.56.3.5 fpr

`auto LinkPred::PerfLambda::fpr`

Initial value:
```
= [](std::size_t tp, std::size_t fn, std::size_t tn, std::size_t fp,
        std::size_t P, std::size_t N) {
    return (double) fp / N;
}
```

False positive rate.

Parameters

tp	The number of true positives.
fn	The number of false negatives.
tn	The number of true negatives.
fp	The number of false positives.
P	The number of positives.
N	The number of negatives.

10.56.3.6 npv

`auto LinkPred::PerfLambda::npv`

Initial value:
```
= [](std::size_t tp, std::size_t fn, std::size_t tn, std::size_t fp,
        std::size_t P, std::size_t N) {
    return (double) tn / (tn + fn);
}
```

Negative predictive value.

Parameters

tp	The number of true positives.
fn	The number of false negatives.
tn	The number of true negatives.
fp	The number of false positives.
P	The number of positives.
N	The number of negatives.

10.56.3.7 pre

`auto LinkPred::PerfLambda::pre`

Initial value:
```
= [](std::size_t tp, std::size_t fn, std::size_t tn, std::size_t fp,
        std::size_t P, std::size_t N) {
    return (double) tp / (tp + fp);
}
```

Precision.

Parameters

tp	The number of true positives.
fn	The number of false negatives.
tn	The number of true negatives.
fp	The number of false positives.
P	The number of positives.
N	The number of negatives.

10.56.3.8 rec

`auto LinkPred::PerfLambda::rec`

Initial value:
```
= [](std::size_t tp, std::size_t fn, std::size_t tn, std::size_t fp,
        std::size_t P, std::size_t N) {
    return (double) tp / P;
}
```

Recall.

Parameters

tp	The number of true positives.
fn	The number of false negatives.
tn	The number of true negatives.
fp	The number of false positives.
P	The number of positives.
N	The number of negatives.

10.56.3.9 tnr

```
auto LinkPred::PerfLambda::tnr
```

Initial value:
```
= [](std::size_t tp, std::size_t fn, std::size_t tn, std::size_t fp,
        std::size_t P, std::size_t N) {
    return (double) tn / N;
}
```

True negative rate.

Parameters

tp	The number of true positives.
fn	The number of false negatives.
tn	The number of true negatives.
fp	The number of false positives.
P	The number of positives.
N	The number of negatives.

10.57 include/linkpred/perf/predresults.hpp File Reference

Contains the implementation of a class to store and manage prediction results.

```
#include <linkpred/predictors/undirected/ulpredictor.hpp>
#include <linkpred/utils/miscutils.hpp>
#include "linkpred/core/unetwork/unetwork.hpp"
#include "linkpred/core/dnetwork/dnetwork.hpp"
#include "linkpred/perf/networkmanipulator.hpp"
#include <vector>
#include <memory>
#include <algorithm>
#include <functional>
```

Include dependency graph for predresults.hpp:

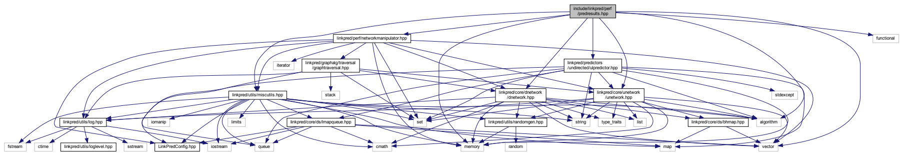

This graph shows which files directly or indirectly include this file:

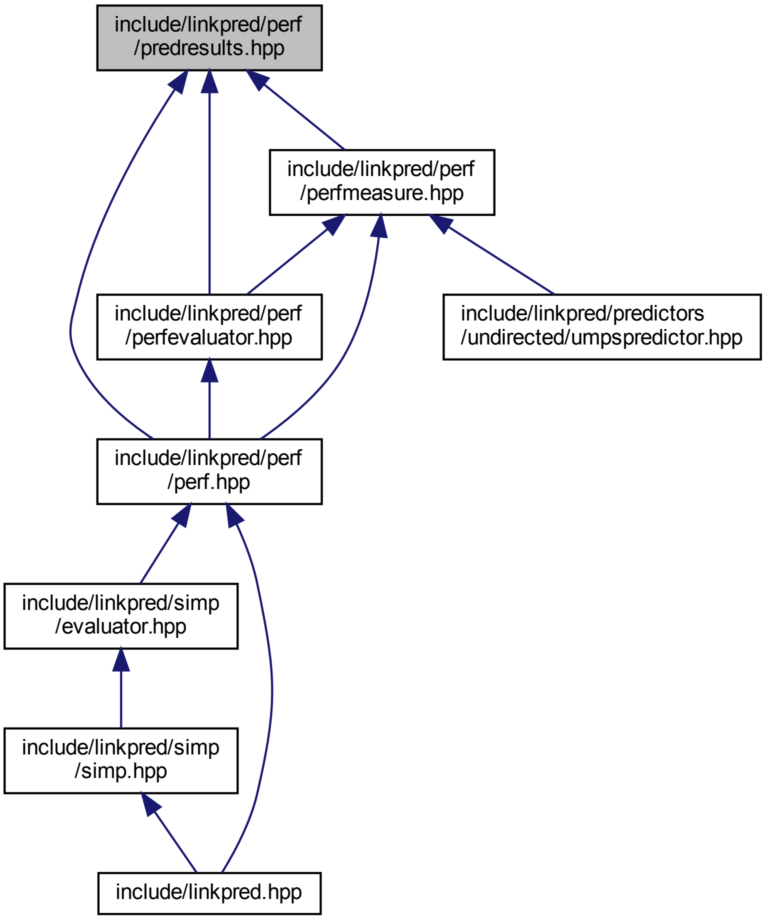

Classes

- class LinkPred::PredResults< TestDataT, LPredictorT, ScoresContainerT >

  A class to store and manage prediction results.
- class LinkPred::PredResults< TestDataT, LPredictorT, ScoresContainerT >::ScoreIterator

  Score iterator.

Namespaces

- [LinkPred](#)

  Main namespace.

10.57.1 Detailed Description

Contains the implementation of a class to store and manage prediction results.

10.58 include/linkpred/predictors/directed/dadapredictor.hpp File Reference

Contains the implementation of a common neighbor link predictor.

```
#include "linkpred/predictors/directed/dlpredictor.hpp"
#include <memory>
```

Include dependency graph for dadapredictor.hpp:

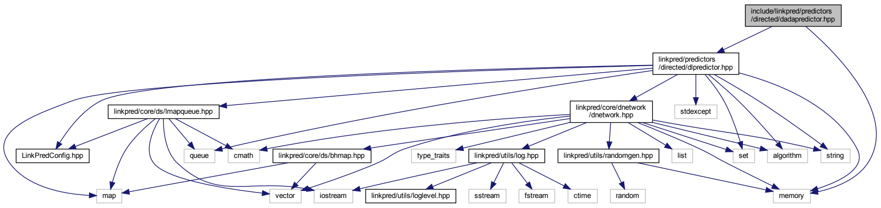

This graph shows which files directly or indirectly include this file:

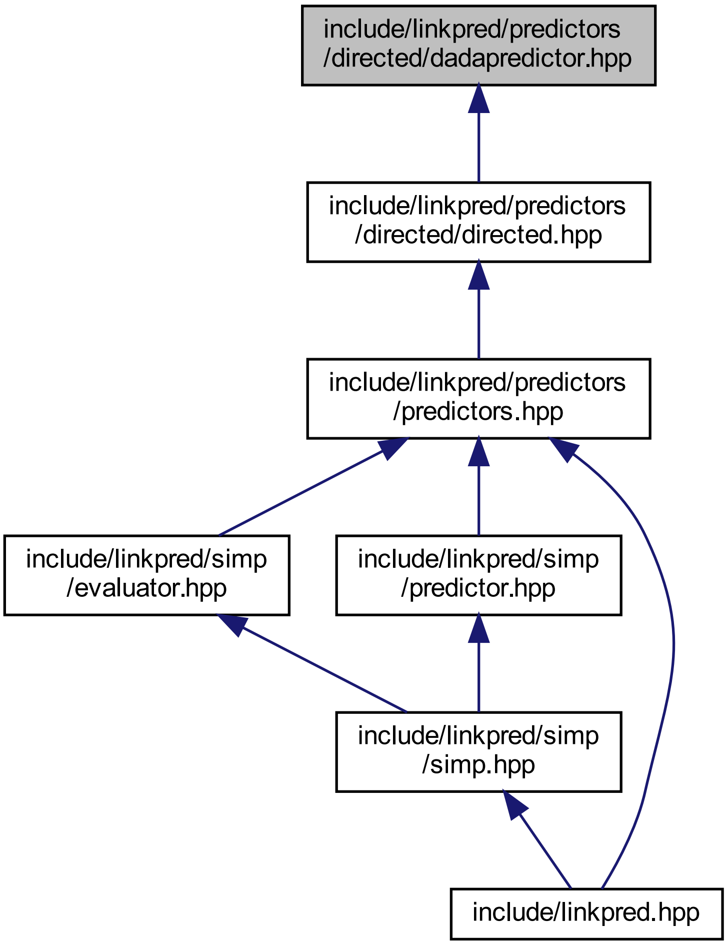

Classes

- class LinkPred::DADAPredictor< Network, EdgeRndIt, ScoreRndIt, EdgeRndOutIt >

  Common neighbor link predictor adapted to directed networks.

Namespaces

- LinkPred

  Main namespace.

10.58.1 Detailed Description

Contains the implementation of a common neighbor link predictor.

10.59 include/linkpred/predictors/directed/dcnepredictor.hpp File Reference

Contains the implementation of a common neighbor link predictor.

```
#include "linkpred/predictors/directed/dlpredictor.hpp"
#include <memory>
```

Include dependency graph for dcnepredictor.hpp:

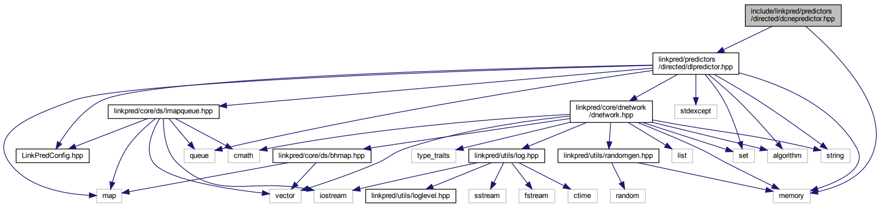

This graph shows which files directly or indirectly include this file:

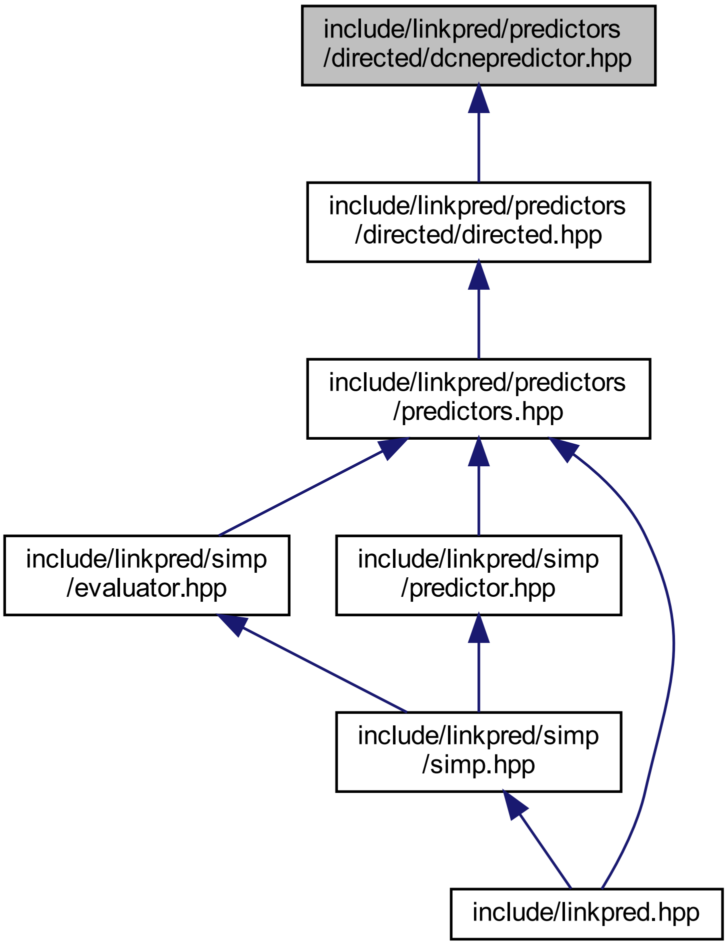

Classes

- class LinkPred::DCNEPredictor< Network, EdgeRndIt, ScoreRndIt, EdgeRndOutIt >

    Common neighbor link predictor adapted to directed networks.

Namespaces

- LinkPred

    Main namespace.

10.59.1 Detailed Description

Contains the implementation of a common neighbor link predictor.

10.60 include/linkpred/predictors/directed/dhdipredictor.hpp File Reference

Contains the implementation of a common neighbor link predictor.

```
#include "linkpred/predictors/directed/dlpredictor.hpp"
#include <memory>
```

Include dependency graph for dhdipredictor.hpp:

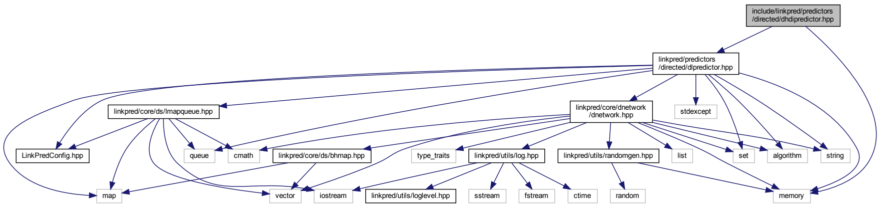

This graph shows which files directly or indirectly include this file:

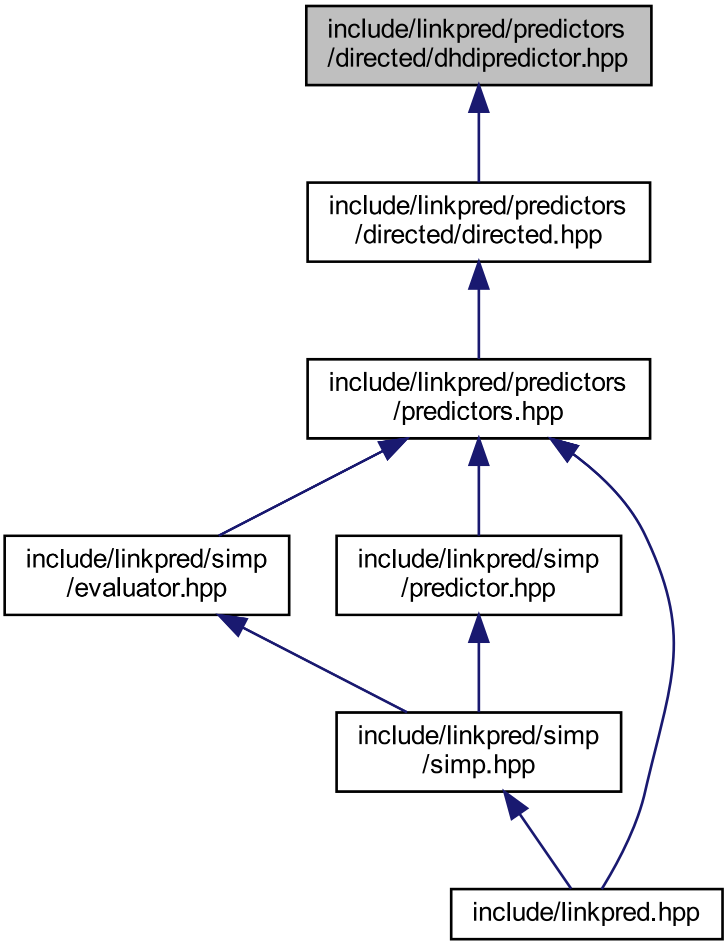

Classes

- class LinkPred::DHDIPredictor< Network, EdgeRndIt, ScoreRndIt, EdgeRndOutIt >

  Common neighbor link predictor adapted to directed networks.

Namespaces

- LinkPred

  Main namespace.

10.60.1 Detailed Description

Contains the implementation of a common neighbor link predictor.

10.61 include/linkpred/predictors/directed/dhpipredictor.hpp File Reference

Contains the implementation of a common neighbor link predictor.

```
#include "linkpred/predictors/directed/dlpredictor.hpp"
#include <memory>
```

Include dependency graph for dhpipredictor.hpp:

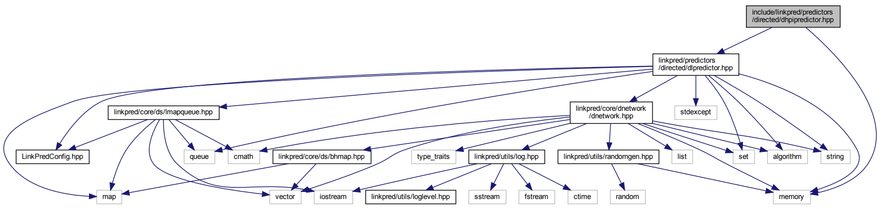

This graph shows which files directly or indirectly include this file:

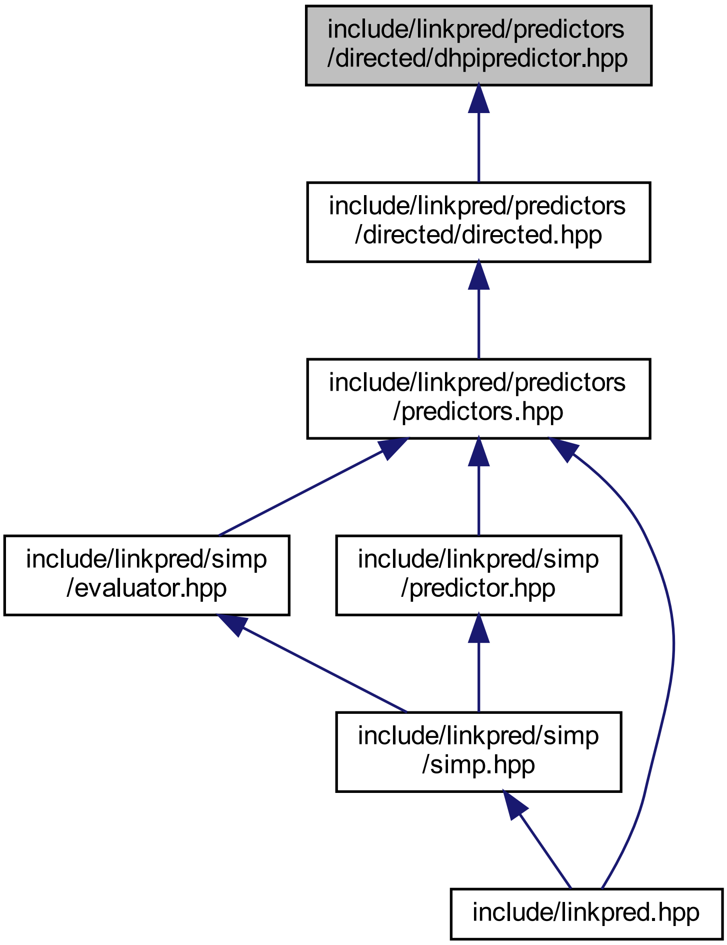

Classes

- class LinkPred::DHPIPredictor< Network, EdgeRndIt, ScoreRndIt, EdgeRndOutIt >

  Common neighbor link predictor adapted to directed networks.

Namespaces

- LinkPred

  Main namespace.

10.61.1 Detailed Description

Contains the implementation of a common neighbor link predictor.

10.62 include/linkpred/predictors/directed/directed.hpp File Reference

Includes the headers of link predictors for directed networks.

```
#include "linkpred/predictors/directed/dadapredictor.hpp"
#include "linkpred/predictors/directed/dcnepredictor.hpp"
#include "linkpred/predictors/directed/dhdipredictor.hpp"
#include "linkpred/predictors/directed/dhpipredictor.hpp"
#include "linkpred/predictors/directed/djidpredictor.hpp"
#include "linkpred/predictors/directed/dlcppredictor.hpp"
#include "linkpred/predictors/directed/dlhnpredictor.hpp"
#include "linkpred/predictors/directed/dlpredictor.hpp"
#include "linkpred/predictors/directed/dpatpredictor.hpp"
#include "linkpred/predictors/directed/dpstpredictor.hpp"
#include "linkpred/predictors/directed/dsaipredictor.hpp"
#include "linkpred/predictors/directed/dsoipredictor.hpp"
```

Include dependency graph for directed.hpp:

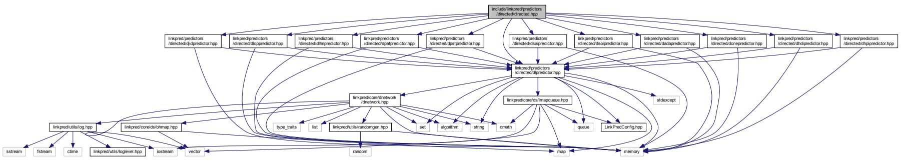

This graph shows which files directly or indirectly include this file:

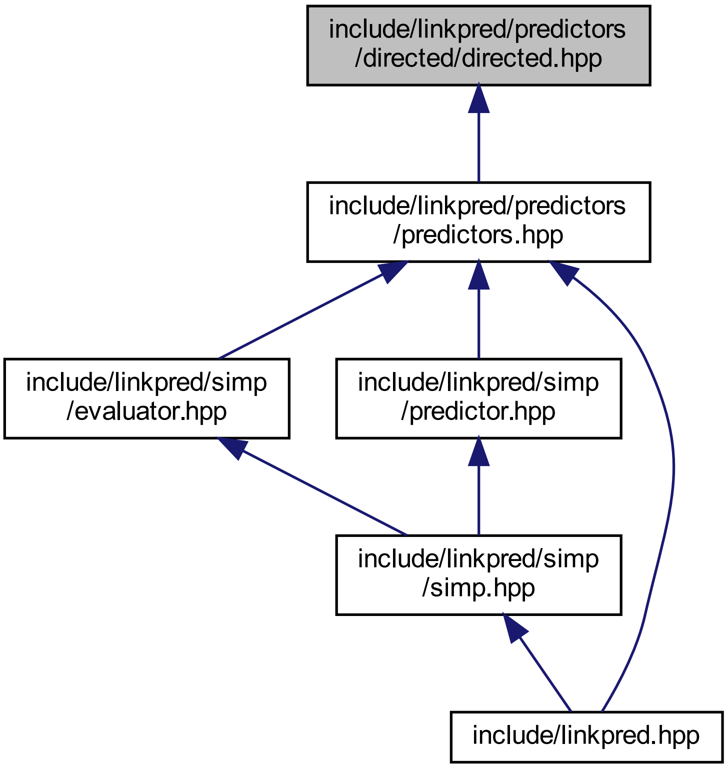

10.62.1 Detailed Description

Includes the headers of link predictors for directed networks.

10.63 include/linkpred/predictors/directed/djidpredictor.hpp File Reference

Contains the implementation of a common neighbor link predictor.

```
#include "linkpred/predictors/directed/dlpredictor.hpp"
#include <memory>
```

Include dependency graph for djidpredictor.hpp:

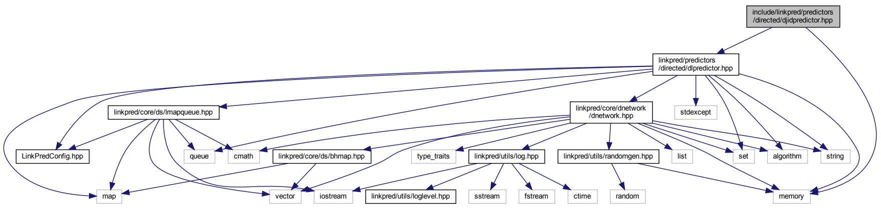

This graph shows which files directly or indirectly include this file:

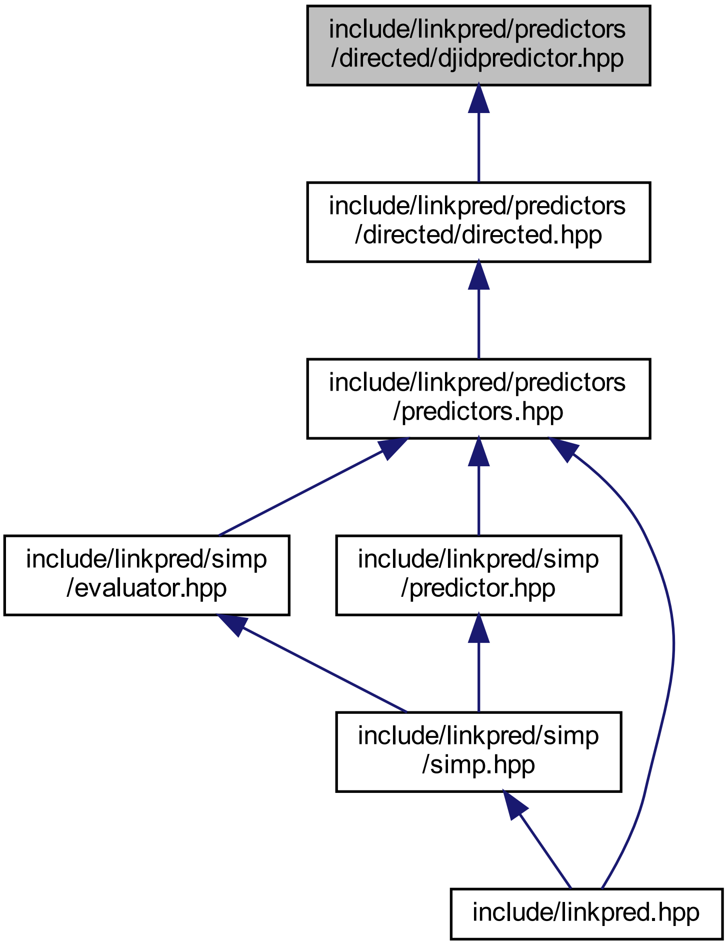

Classes

- class LinkPred::DJIDPredictor< Network, EdgeRndIt, ScoreRndIt, EdgeRndOutIt >

    Common neighbor link predictor adapted to directed networks.

Namespaces

- LinkPred

    Main namespace.

10.63.1 Detailed Description

Contains the implementation of a common neighbor link predictor.

10.64 include/linkpred/predictors/directed/dlcppredictor.hpp File Reference

Contains the implementation of a directed LCP link predictor.

```
#include "linkpred/predictors/directed/dlpredictor.hpp"
#include <memory>
```

Include dependency graph for dlcppredictor.hpp:

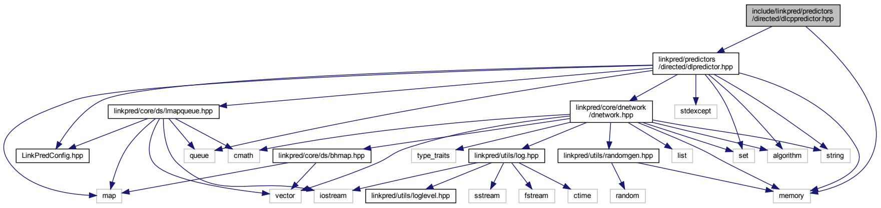

This graph shows which files directly or indirectly include this file:

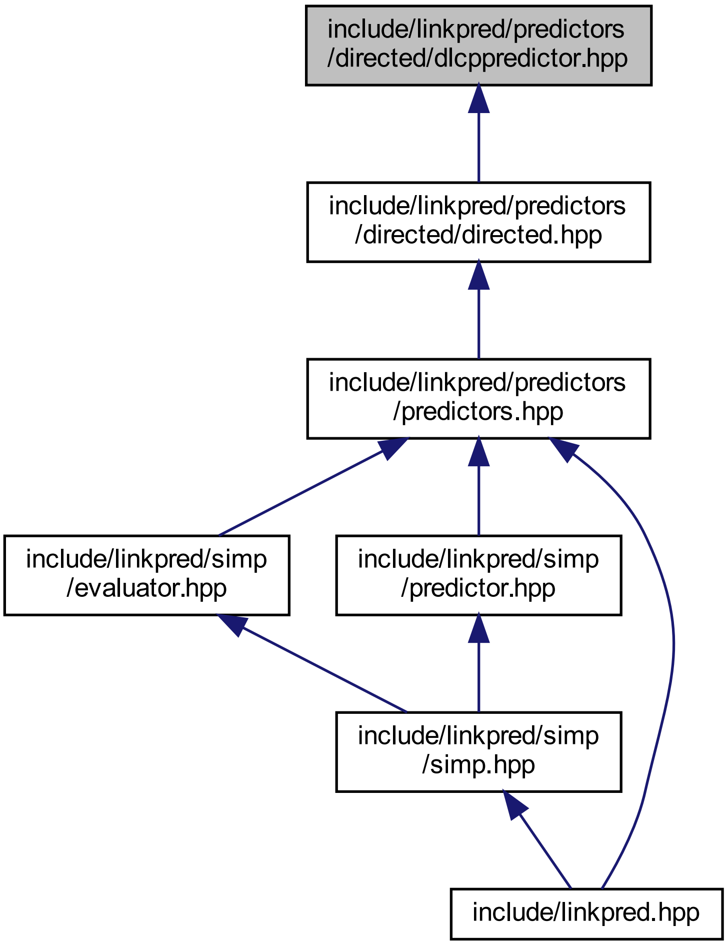

Classes

- class LinkPred::DLCPPredictor< Network, EdgeRndIt, ScoreRndIt, EdgeRndOutIt >

  Common neighbor link predictor adapted to directed networks.

Namespaces

- LinkPred

  Main namespace.

10.64.1 Detailed Description

Contains the implementation of a directed LCP link predictor.

10.65 include/linkpred/predictors/directed/dlhnpredictor.hpp File Reference

Contains the implementation of a common neighbor link predictor.

```
#include "linkpred/predictors/directed/dlpredictor.hpp"
#include <memory>
```

Include dependency graph for dlhnpredictor.hpp:

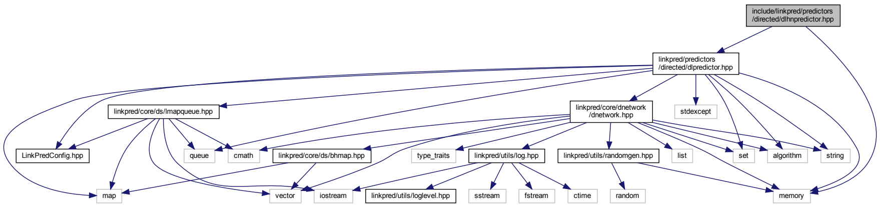

This graph shows which files directly or indirectly include this file:

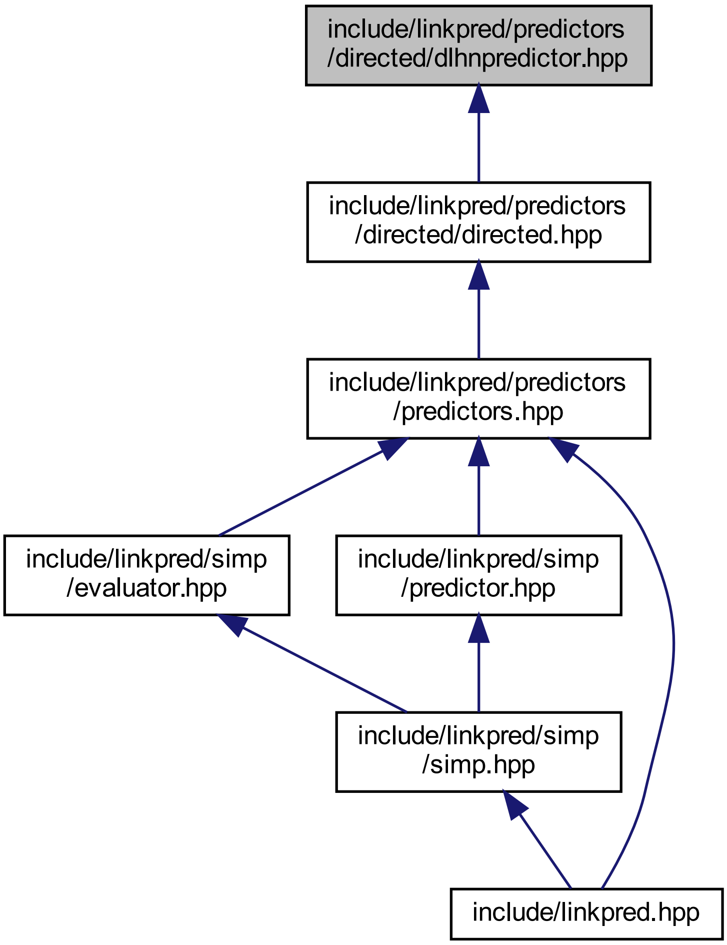

Classes

- class LinkPred::DLHNPredictor< Network, EdgeRndIt, ScoreRndIt, EdgeRndOutIt >

  Common neighbor link predictor adapted to directed networks.

Namespaces

- LinkPred

  Main namespace.

10.65.1 Detailed Description

Contains the implementation of a common neighbor link predictor.

10.66 include/linkpred/predictors/directed/dlpredictor.hpp File Reference

Contains the interface of a link predictor.

```
#include "LinkPredConfig.hpp"
#include "linkpred/core/dnetwork/dnetwork.hpp"
#include "linkpred/core/ds/lmapqueue.hpp"
#include <memory>
#include <map>
#include <string>
#include <stdexcept>
#include <algorithm>
#include <queue>
#include <set>
```

Include dependency graph for dlpredictor.hpp:

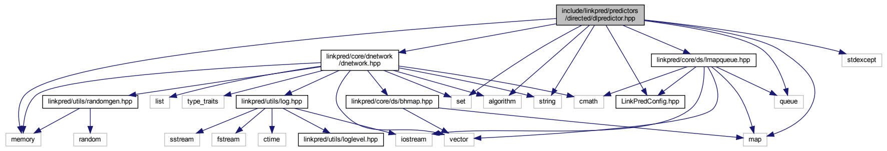

This graph shows which files directly or indirectly include this file:

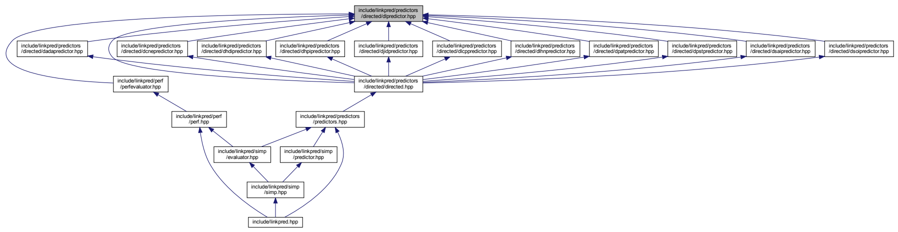

Classes

- class LinkPred::DLPredictor< NetworkT, EdgeRndItT, ScoreRndItT, EdgeRndOutItT >

  The interface of a link predictor in a directed network.

Namespaces

- LinkPred

  Main namespace.

10.66.1 Detailed Description

Contains the interface of a link predictor.

10.67 include/linkpred/predictors/directed/dpatpredictor.hpp File Reference

Contains the implementation of a common neighbor link predictor.

```
#include "linkpred/predictors/directed/dlpredictor.hpp"
#include <memory>
```

Include dependency graph for dpatpredictor.hpp:

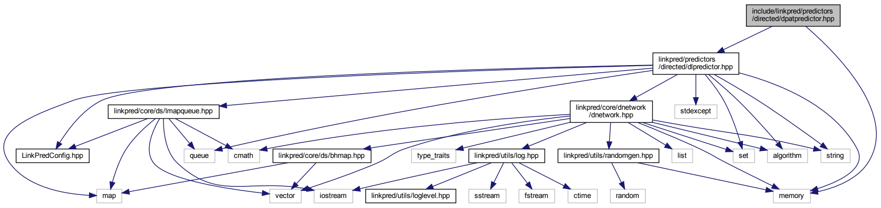

This graph shows which files directly or indirectly include this file:

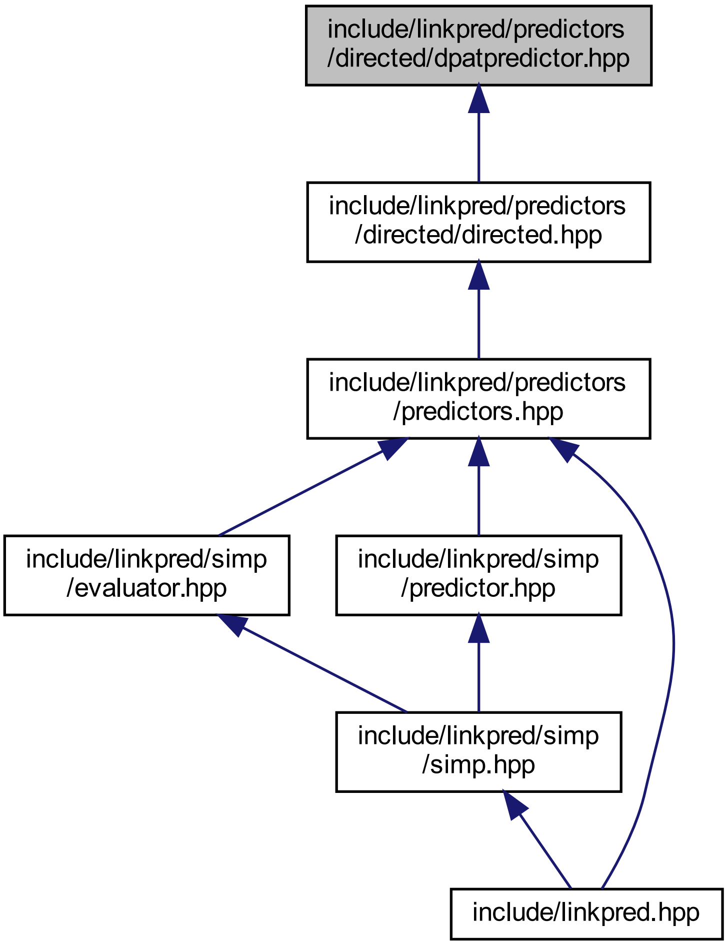

Classes

- class LinkPred::DPATPredictor< Network, EdgeRndIt, ScoreRndIt, EdgeRndOutIt >

    Common neighbor link predictor adapted to directed networks.

Namespaces

- LinkPred

    Main namespace.

10.67.1 Detailed Description

Contains the implementation of a common neighbor link predictor.

10.68 include/linkpred/predictors/directed/dpstpredictor.hpp File Reference

Contains the implementation of a link predictor that prestores the scores of edges.

```
#include <linkpred/predictors/directed/dlpredictor.hpp>
#include <memory>
```

Include dependency graph for dpstpredictor.hpp:

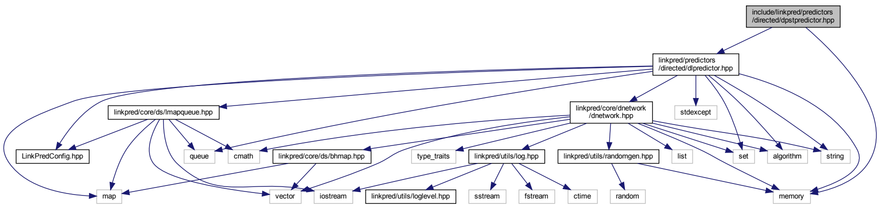

This graph shows which files directly or indirectly include this file:

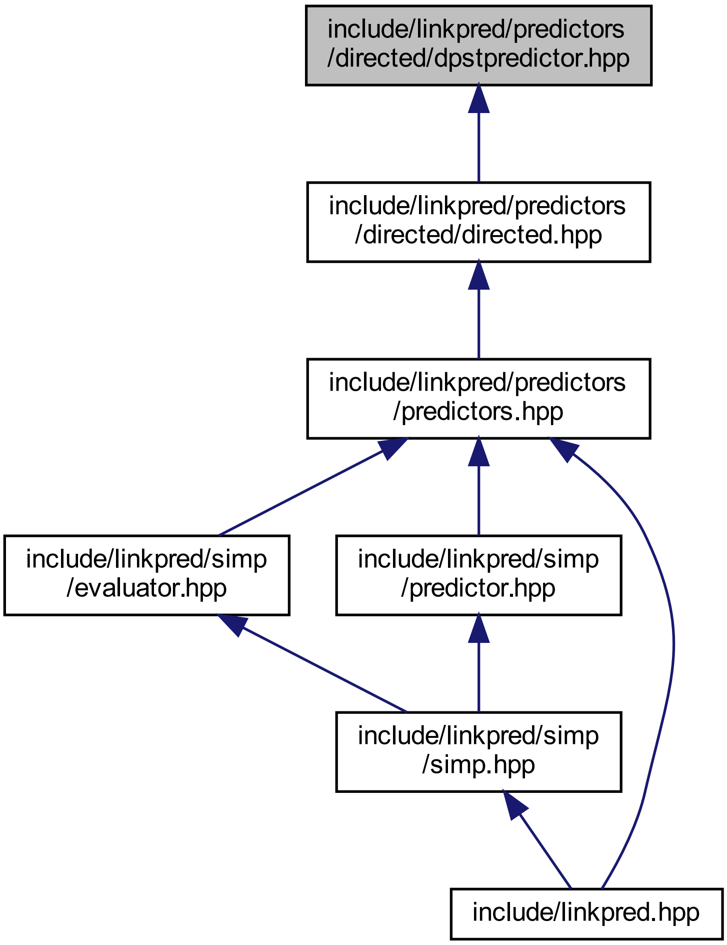

Classes

- class LinkPred::DPSTPredictor< Network, EdgeRndIt, ScoreRndIt, EdgeRndOutIt >

  A link predictor that prestores edge scores. This allows to seemingly integrate results from external link prediction algorithms to LinkPred (for example, users may implement their own link prediction algorithm and then use this link predictor to use compare their results to algorithms available in LinkPred).

Namespaces

- LinkPred

  Main namespace.

10.68.1 Detailed Description

Contains the implementation of a link predictor that prestores the scores of edges.

10.69 include/linkpred/predictors/directed/dsaipredictor.hpp File Reference

Contains the implementation of a common neighbor link predictor.

```
#include "linkpred/predictors/directed/dlpredictor.hpp"
#include <memory>
```

Include dependency graph for dsaipredictor.hpp:

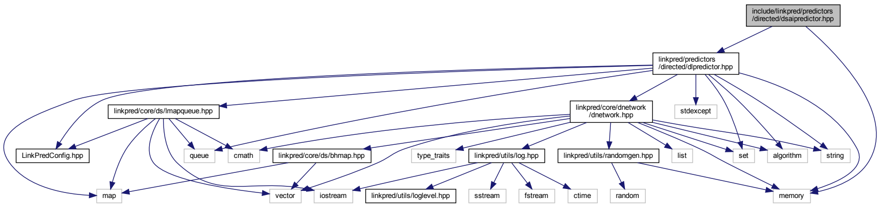

This graph shows which files directly or indirectly include this file:

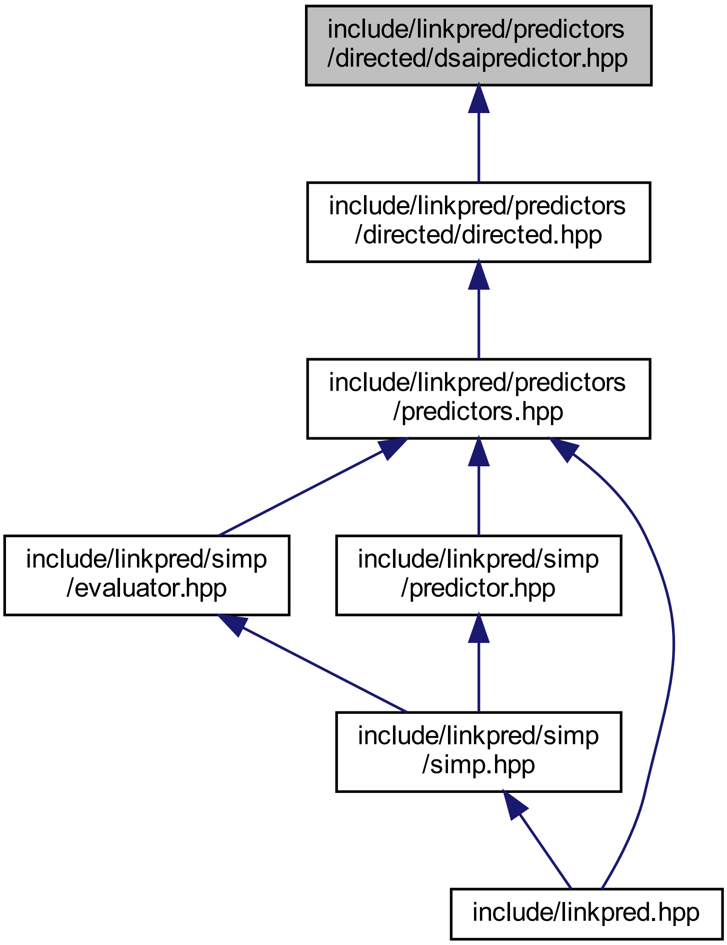

Classes

- class LinkPred::DSAIPredictor< Network, EdgeRndIt, ScoreRndIt, EdgeRndOutIt >

    Common neighbor link predictor adapted to directed networks.

Namespaces

- LinkPred

    Main namespace.

10.69.1 Detailed Description

Contains the implementation of a common neighbor link predictor.

10.70 include/linkpred/predictors/directed/dsoipredictor.hpp File Reference

Contains the implementation of a common neighbor link predictor.

```
#include "linkpred/predictors/directed/dlpredictor.hpp"
#include <memory>
```

Include dependency graph for dsoipredictor.hpp:

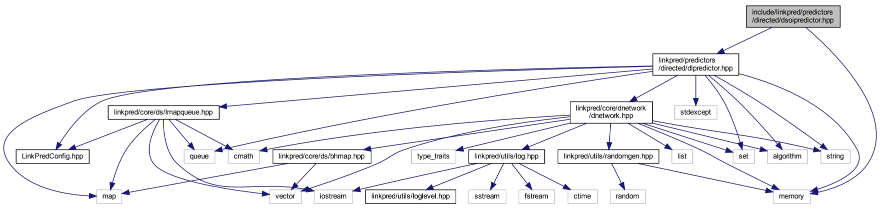

This graph shows which files directly or indirectly include this file:

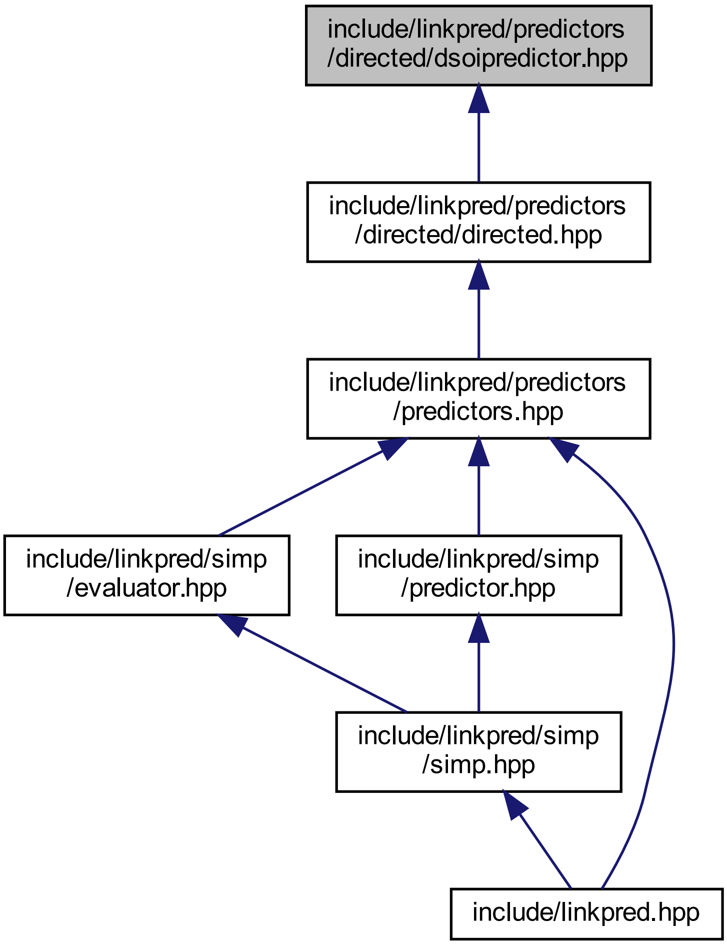

Classes

- class LinkPred::DSOIPredictor< Network, EdgeRndIt, ScoreRndIt, EdgeRndOutIt >

    Common neighbor link predictor adapted to directed networks.

Namespaces

- LinkPred

    Main namespace.

10.70.1 Detailed Description

Contains the implementation of a common neighbor link predictor.

10.71 include/linkpred/predictors/predictors.hpp File Reference

Includes the headers of link predictors.

```
#include "linkpred/predictors/directed/directed.hpp"
#include "linkpred/predictors/undirected/undirected.hpp"
```

Include dependency graph for predictors.hpp:

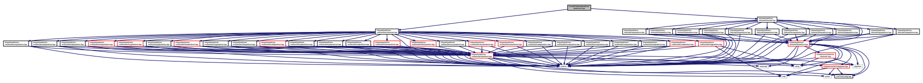

This graph shows which files directly or indirectly include this file:

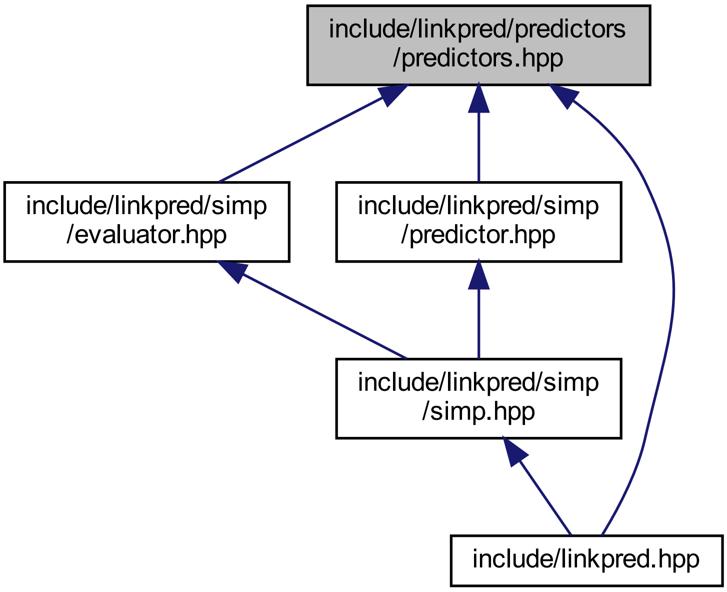

10.71.1 Detailed Description

Includes the headers of link predictors.

10.72 include/linkpred/predictors/undirected/uadapredictor.hpp File Reference

Contains the implementation of an Adamic Adar index link predictor.

```
#include <linkpred/predictors/undirected/ulpredictor.hpp>
#include <memory>
```

Include dependency graph for uadapredictor.hpp:

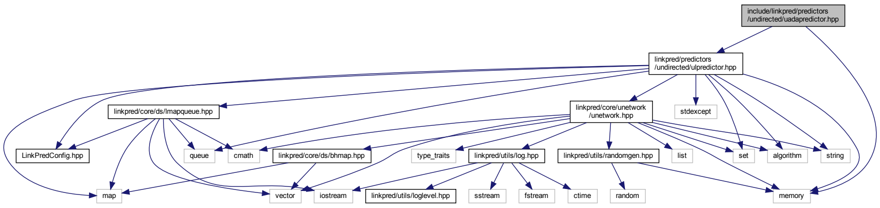

This graph shows which files directly or indirectly include this file:

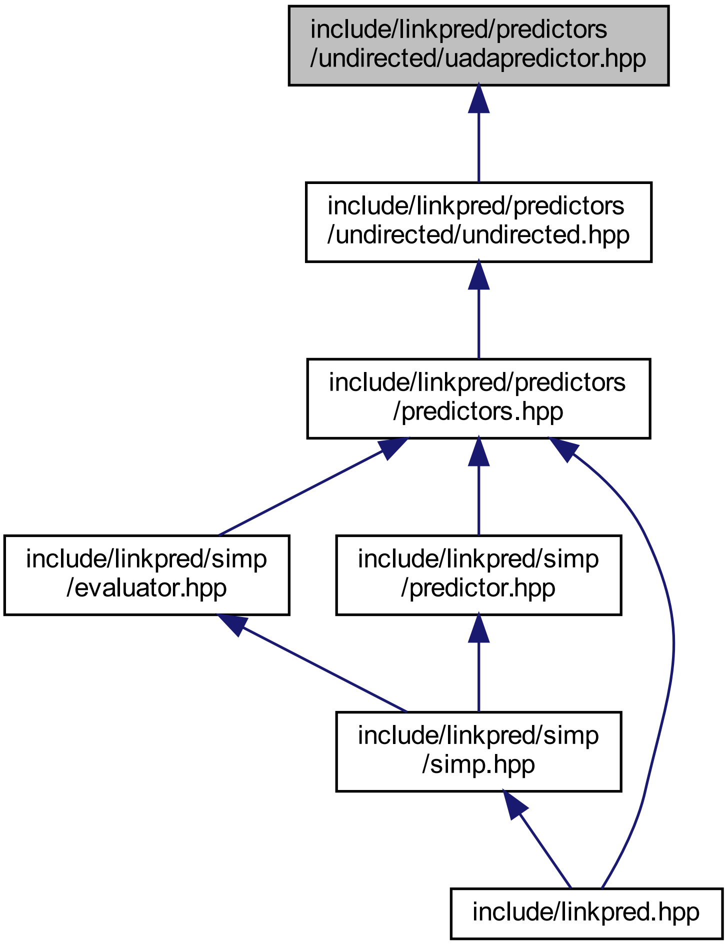

Classes

- class LinkPred::UADAPredictor< Network, EdgeRndIt, ScoreRndIt, EdgeRndOutIt >

    Adamic Adar index link predictor.

Namespaces

- LinkPred

    Main namespace.

10.72.1 Detailed Description

Contains the implementation of an Adamic Adar index link predictor.

10.73 include/linkpred/predictors/undirected/ucnepredictor.hpp File Reference

Contains the implementation of a common neighbor link predictor.

```
#include <linkpred/predictors/undirected/ulpredictor.hpp>
#include <memory>
```

Include dependency graph for ucnepredictor.hpp:

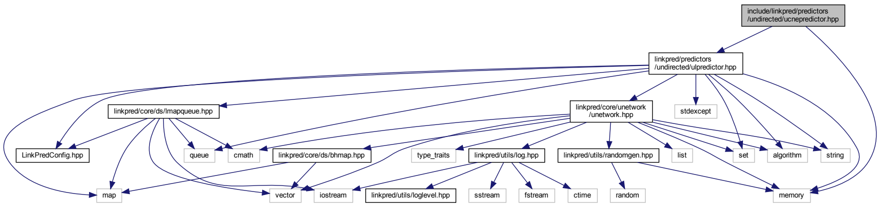

This graph shows which files directly or indirectly include this file:

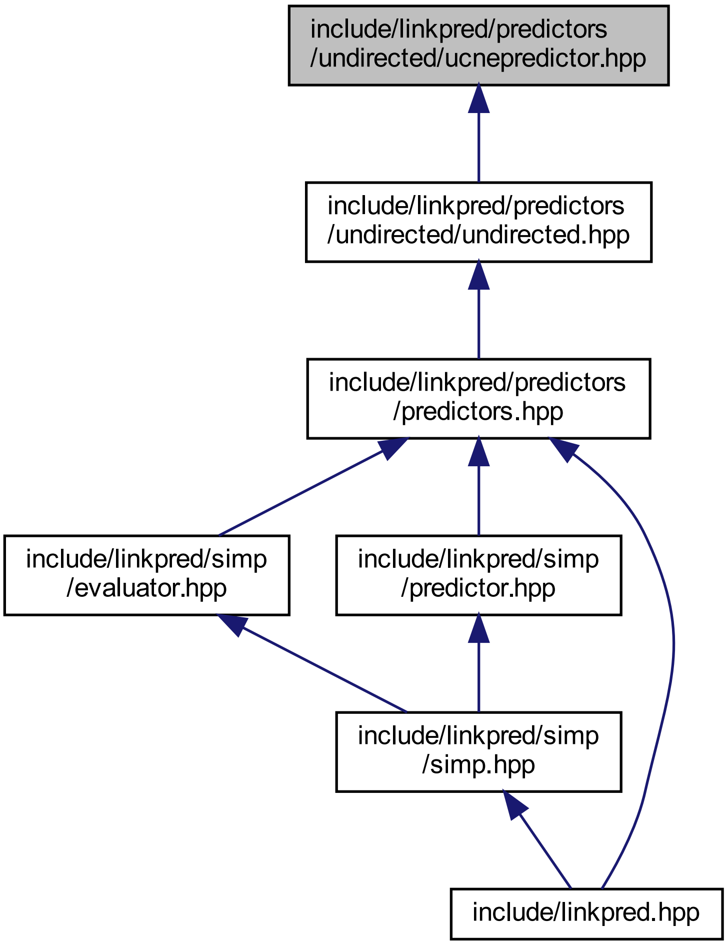

Classes

- class LinkPred::UCNEPredictor< Network, EdgeRndIt, ScoreRndIt, EdgeRndOutIt >

    Common neighbor link predictor.

Namespaces

- LinkPred

    Main namespace.

10.73.1 Detailed Description

Contains the implementation of a common neighbor link predictor.

10.74 include/linkpred/predictors/undirected/ucrapredictor.hpp File Reference

Contains the implementation of the Cannistraci Resource Allocation link predictor.

```
#include <linkpred/predictors/undirected/ulpredictor.hpp>
#include <memory>
```

Include dependency graph for ucrapredictor.hpp:

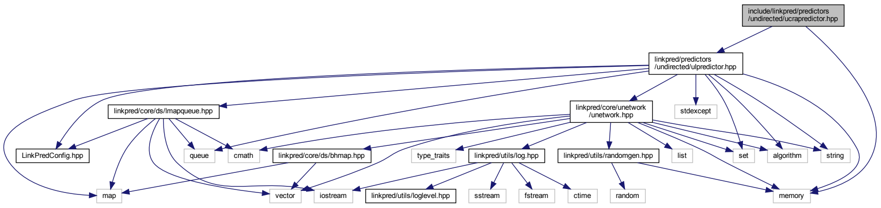

This graph shows which files directly or indirectly include this file:

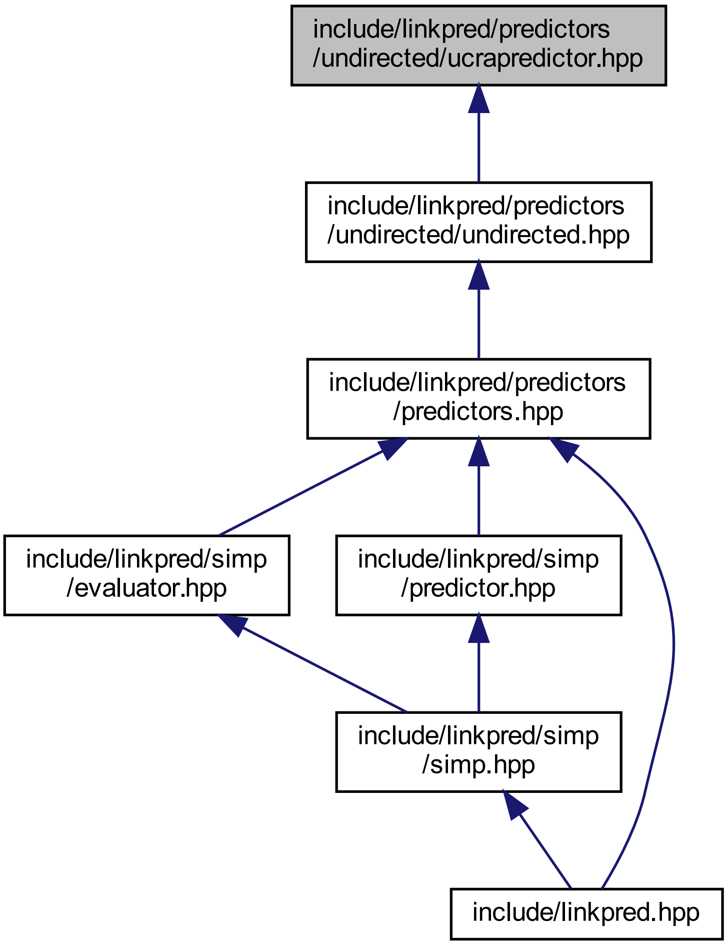

Classes

- class LinkPred::UCRAPredictor< Network, EdgeRndIt, ScoreRndIt, EdgeRndOutIt >

  Local path link predictor.

Namespaces

- LinkPred

  Main namespace.

10.74.1 Detailed Description

Contains the implementation of the Cannistraci Resource Allocation link predictor.

10.75 include/linkpred/predictors/undirected/ucstpredictor.hpp File Reference

Contains the implementation of a constant link predictor.

```
#include <linkpred/predictors/undirected/ulpredictor.hpp>
#include <memory>
```

Include dependency graph for ucstpredictor.hpp:

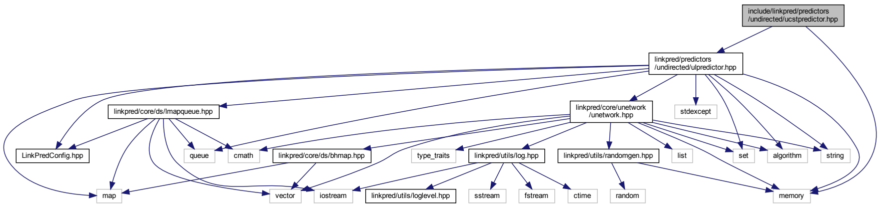

This graph shows which files directly or indirectly include this file:

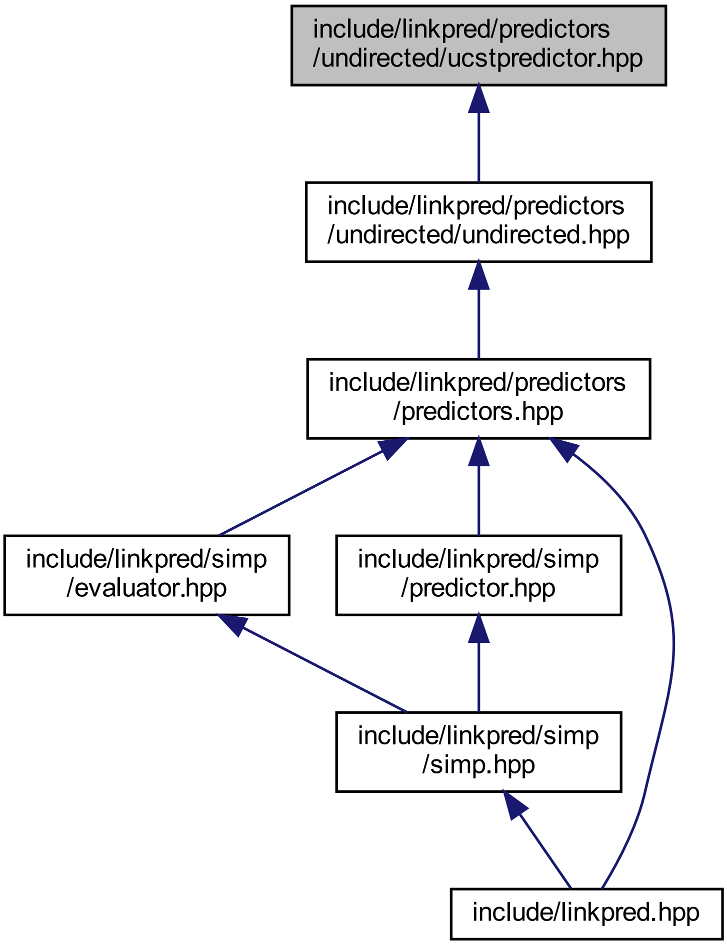

Classes

- class LinkPred::UCSTPredictor< Network, EdgeRndIt, ScoreRndIt, EdgeRndOutIt >

    Constant link predictor.

Namespaces

- LinkPred

    Main namespace.

10.75.1 Detailed Description

Contains the implementation of a constant link predictor.

10.76 include/linkpred/predictors/undirected/ueclpredictor.hpp File Reference

Contains the implementation of an encoder-classifier link predictor.

```
#include <linkpred/predictors/undirected/ulpredictor.hpp>
#include <linkpred/graphalg/encoders/encoders.hpp>
#include <linkpred/ml/classifiers/classifiers.hpp>
#include <memory>
```

Include dependency graph for ueclpredictor.hpp:

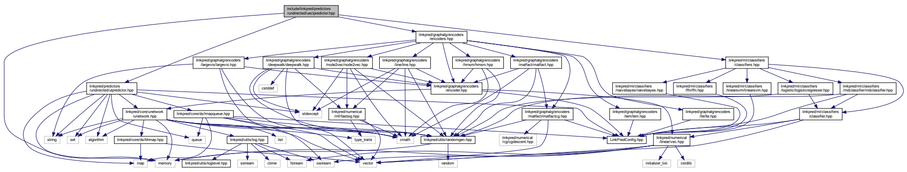

This graph shows which files directly or indirectly include this file:

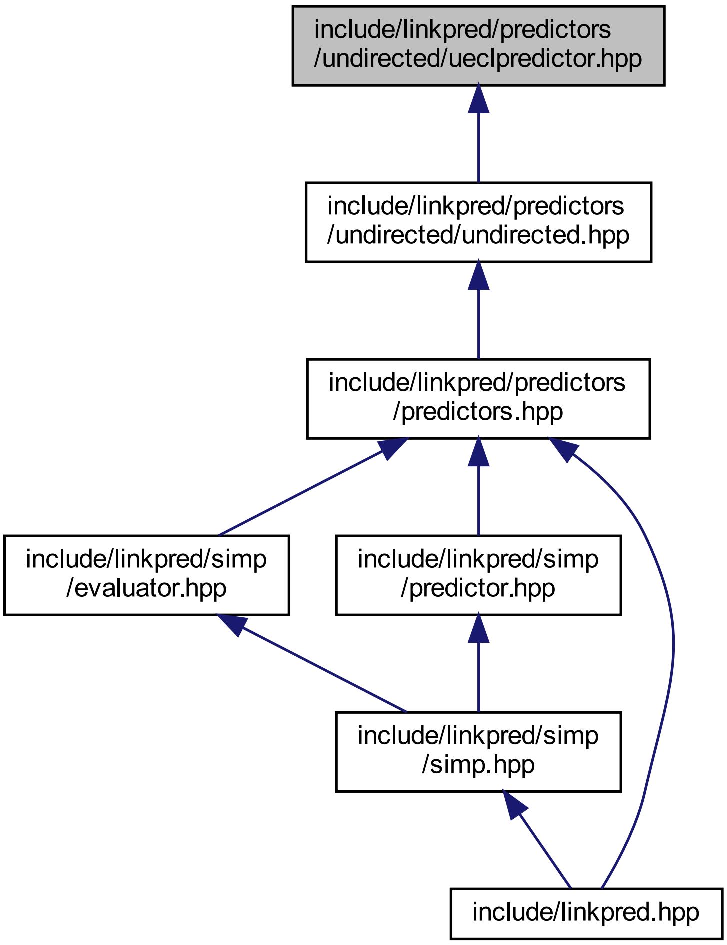

Classes

- class LinkPred::UECLPredictor< Network, EdgeRndIt, ScoreRndIt, EdgeRndOutIt >

  Encoder-classifier link predictor.

Namespaces

- LinkPred

  Main namespace.

10.76.1 Detailed Description

Contains the implementation of an encoder-classifier link predictor.

10.77 include/linkpred/predictors/undirected/uesmpredictor.hpp File Reference

Contains the implementation of an encoder-similarity measure link predictor.

```
#include <linkpred/predictors/undirected/ulpredictor.hpp>
#include <linkpred/graphalg/encoders/encoders.hpp>
#include <linkpred/ml/simmeasures/simmeasures.hpp>
#include <memory>
```

Include dependency graph for uesmpredictor.hpp:

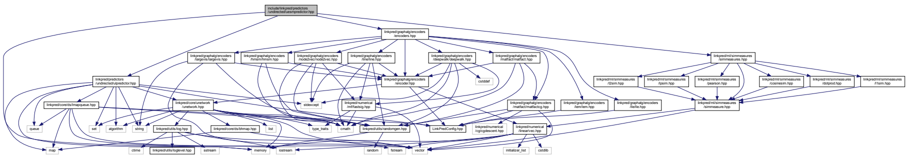

This graph shows which files directly or indirectly include this file:

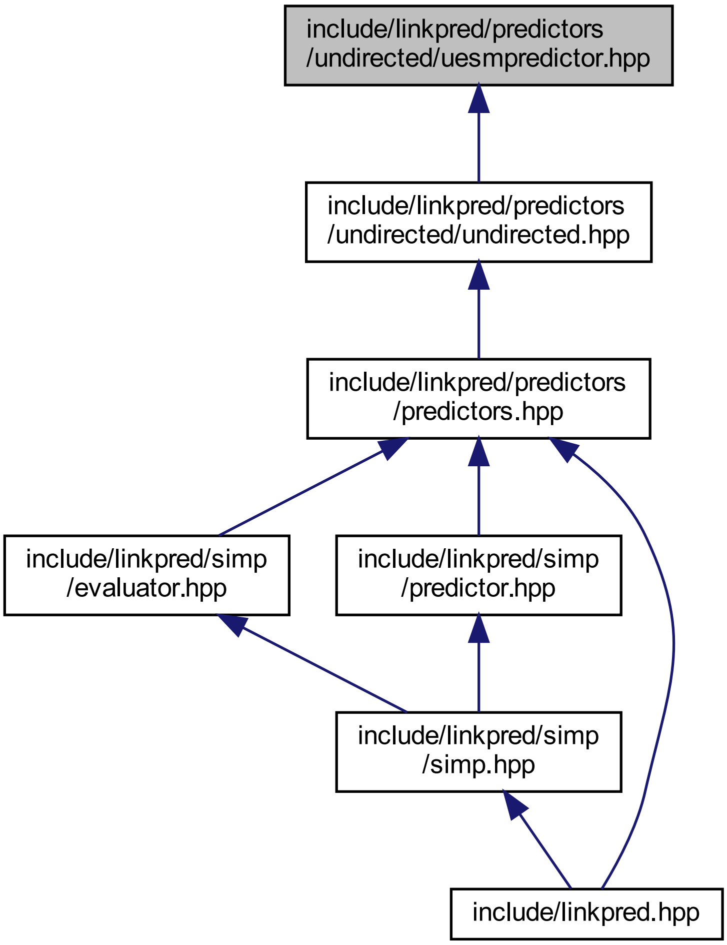

Classes

- class LinkPred::UESMPredictor< Network, EdgeRndIt, ScoreRndIt, EdgeRndOutIt >

    Encoder-Similarity measure link predictor.

Namespaces

- LinkPred

    Main namespace.

10.77.1 Detailed Description

Contains the implementation of an encoder-similarity measure link predictor.

10.78 include/linkpred/predictors/undirected/ufbmpredictor.hpp File Reference

Contains the implementation of the fast blocking model link predictor.

```
#include <linkpred/predictors/undirected/ulpredictor.hpp>
#include "linkpred/numerical/linear/gfmatrix.hpp"
#include "linkpred/utils/randomgen.hpp"
```

Include dependency graph for ufbmpredictor.hpp:

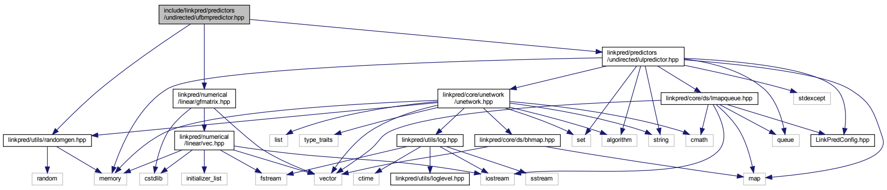

This graph shows which files directly or indirectly include this file:

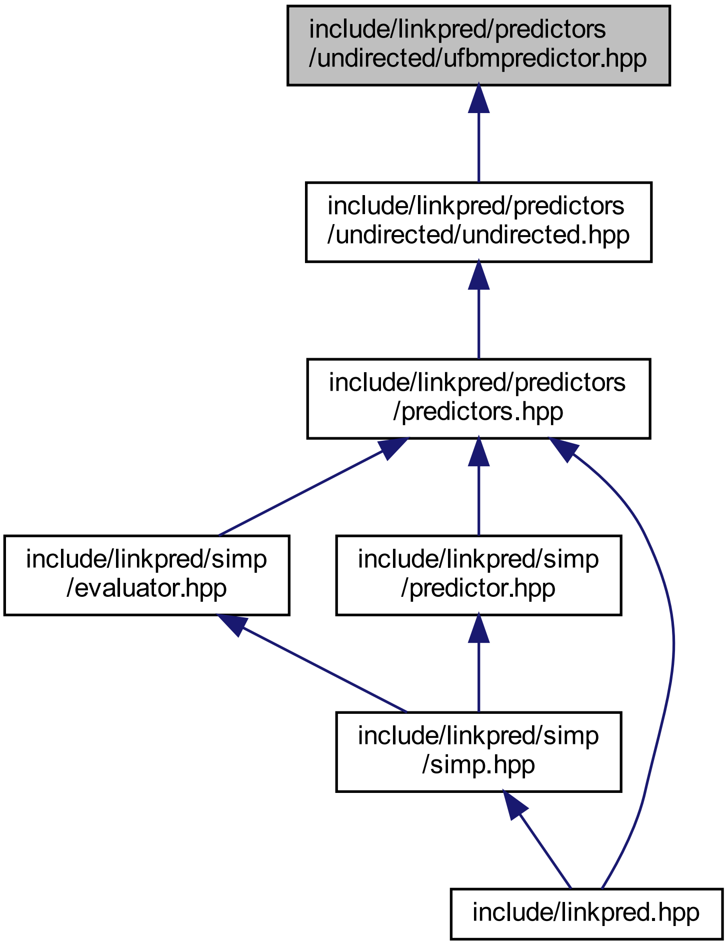

Classes

- class LinkPred::UFBMPredictor< Network, EdgeRndIt, ScoreRndIt, EdgeRndOutIt >

    Fast blocking model link predictor.

Namespaces

- LinkPred

    Main namespace.

10.78.1 Detailed Description

Contains the implementation of the fast blocking model link predictor.

10.79 include/linkpred/predictors/undirected/uhdipredictor.hpp File Reference

Contains the implementation of a hub depromoted index link predictor.

```
#include <linkpred/predictors/undirected/ulpredictor.hpp>
#include <memory>
```

Include dependency graph for uhdipredictor.hpp:

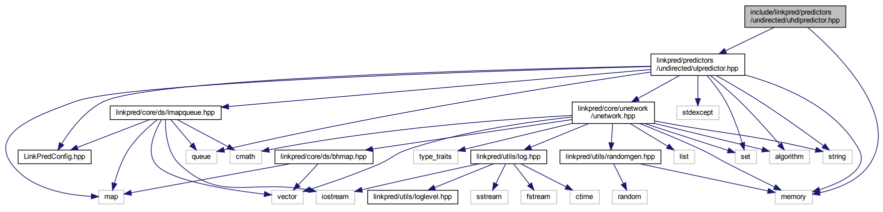

This graph shows which files directly or indirectly include this file:

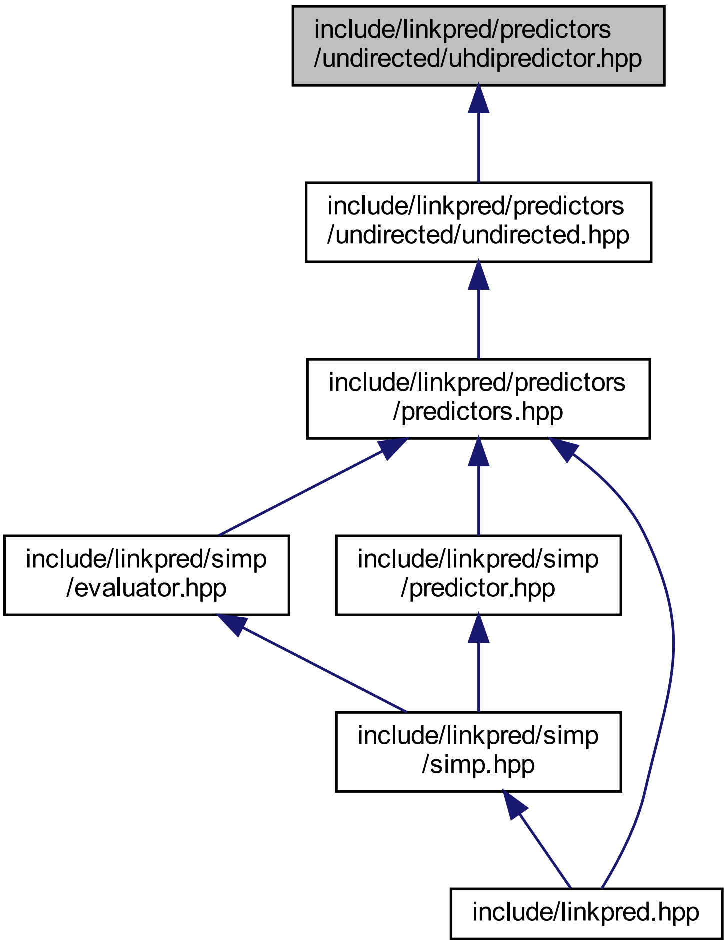

Classes

- class LinkPred::UHDIPredictor< Network, EdgeRndIt, ScoreRndIt, EdgeRndOutIt >

  Hub depromoted index link predictor.

Namespaces

- LinkPred

  Main namespace.

10.79.1 Detailed Description

Contains the implementation of a hub depromoted index link predictor.

10.80 include/linkpred/predictors/undirected/uhpipredictor.hpp File Reference

Contains the implementation of a hub promoted index link predictor.

```
#include <linkpred/predictors/undirected/ulpredictor.hpp>
#include <memory>
```

Include dependency graph for uhpipredictor.hpp:

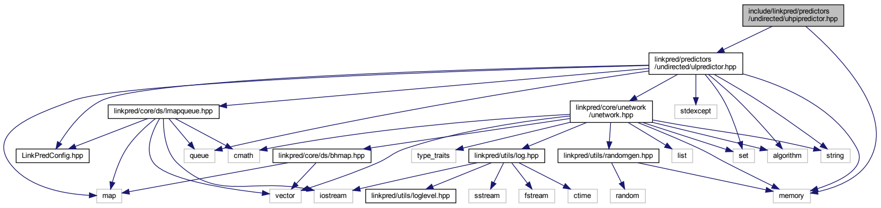

This graph shows which files directly or indirectly include this file:

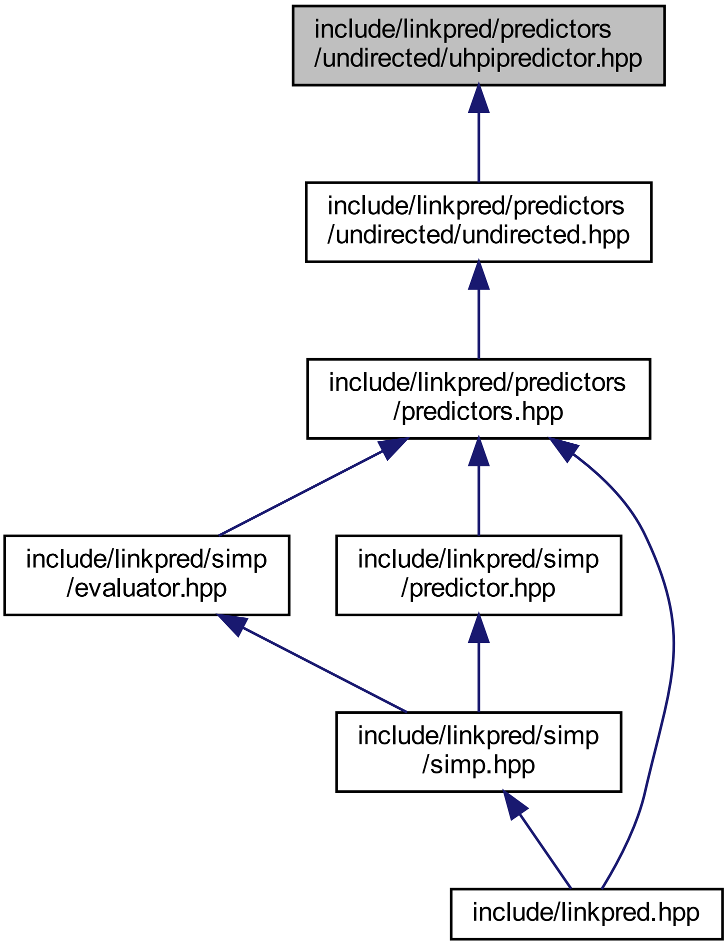

Classes

- class LinkPred::UHPIPredictor< Network, EdgeRndIt, ScoreRndIt, EdgeRndOutIt >

  Hub promoted index link predictor.

Namespaces

- LinkPred

  Main namespace.

10.80.1 Detailed Description

Contains the implementation of a hub promoted index link predictor.

10.81 include/linkpred/predictors/undirected/uhrgpredictor.hpp File Reference

Contains the implementation of the HRG link predictor.

```
#include <linkpred/predictors/undirected/uhrgpredictor/dendro_pr.hpp>
#include <linkpred/predictors/undirected/uhrgpredictor/graph_pr.h>
#include <linkpred/predictors/undirected/uhrgpredictor/graph_simp.h>
#include <linkpred/predictors/undirected/uhrgpredictor/rbtree.h>
#include <linkpred/predictors/undirected/ulpredictor.hpp>
#include "linkpred/utils/randomgen.hpp"
#include <string>
#include <map>
```

Include dependency graph for uhrgpredictor.hpp:

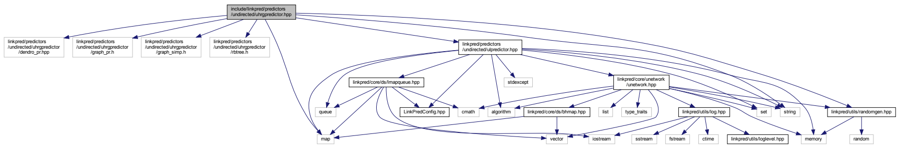

This graph shows which files directly or indirectly include this file:

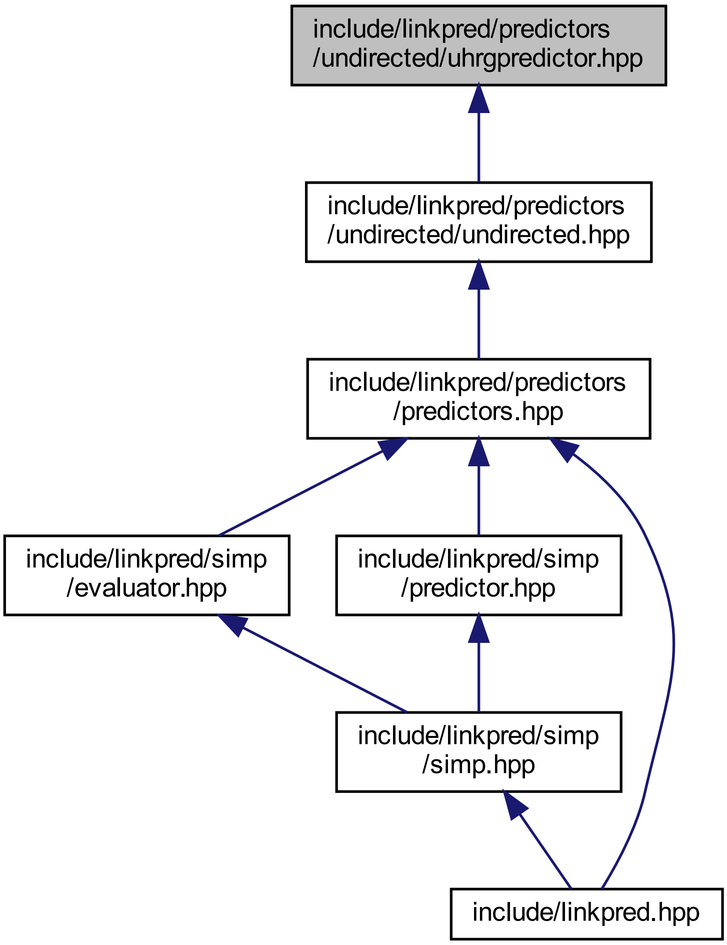

Classes

- class LinkPred::UHRGPredictor< Network, EdgeRndIt, ScoreRndIt, EdgeRndOutIt >

  HRG predictor.

Namespaces

- LinkPred

  Main namespace.

10.81.1 Detailed Description

Contains the implementation of the HRG link predictor.

10.82 include/linkpred/predictors/undirected/uhyppredictor.hpp File Reference

Contains the implementation of the hypermap link predictor.

```
#include <linkpred/predictors/undirected/ulpredictor.hpp>
#include "linkpred/utils/randomgen.hpp"
#include <vector>
#include <memory>
```

Include dependency graph for uhyppredictor.hpp:

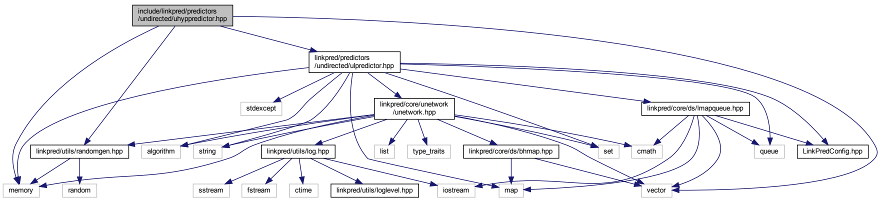

This graph shows which files directly or indirectly include this file:

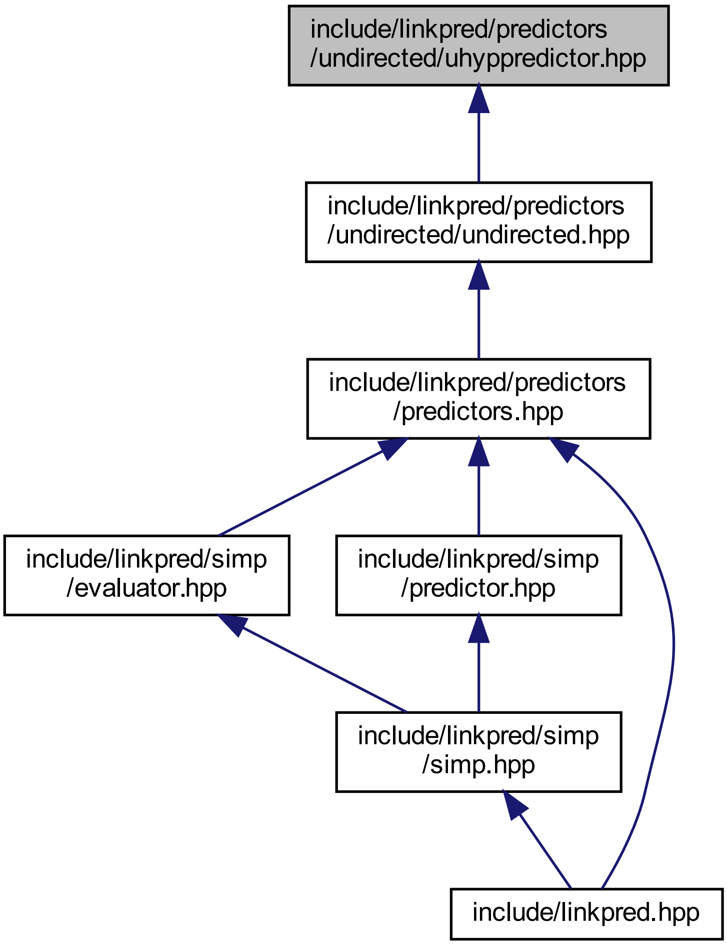

Classes

- class LinkPred::UHYPPredictor< Network, EdgeRndIt, ScoreRndIt, EdgeRndOutIt >

  Hypermap predictor.

Namespaces

- LinkPred

  Main namespace.

Variables

- constexpr double LinkPred::MathPI = 3.14159265358979323846264338327950288 4L

10.82.1 Detailed Description

Contains the implementation of the hypermap link predictor.

10.83 include/linkpred/predictors/undirected/ujidpredictor.hpp File Reference

Contains the implementation of a Jackard index link predictor.

```
#include <linkpred/predictors/undirected/ulpredictor.hpp>
#include <memory>
```

Include dependency graph for ujidpredictor.hpp:

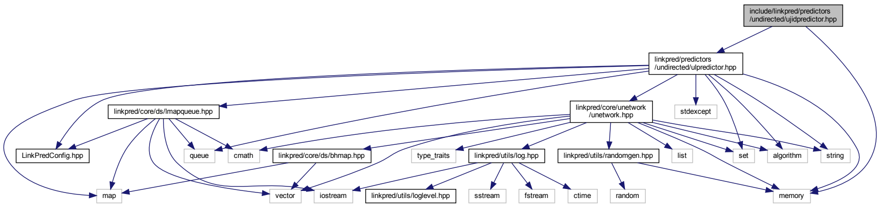

This graph shows which files directly or indirectly include this file:

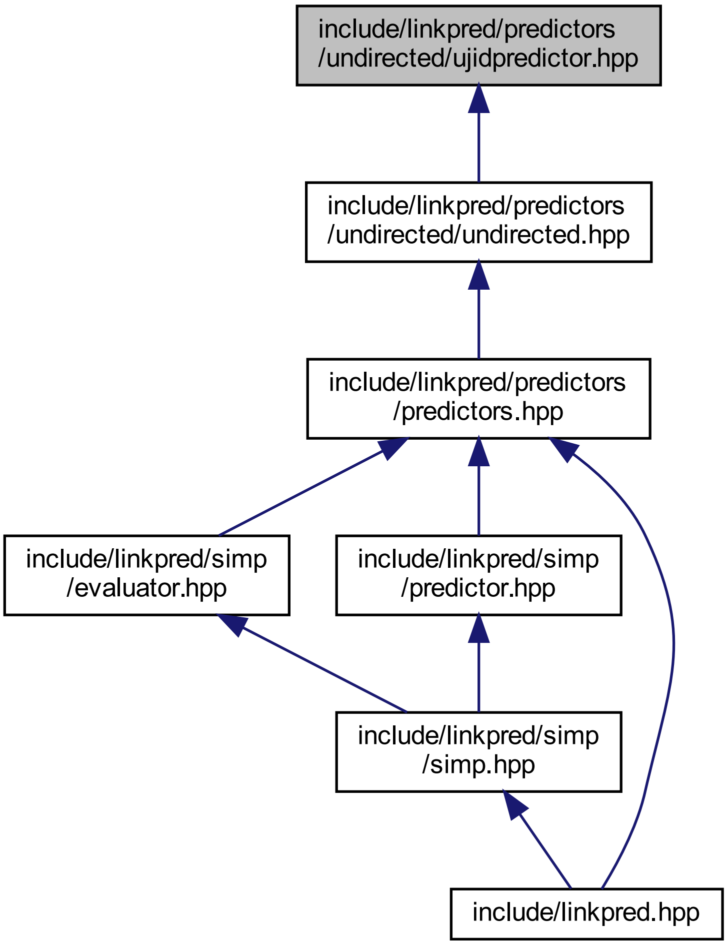

Classes

- class LinkPred::UJIDPredictor< Network, EdgeRndIt, ScoreRndIt, EdgeRndOutIt >

    Jackard index link predictor.

Namespaces

- LinkPred

    Main namespace.

10.83.1 Detailed Description

Contains the implementation of a Jackard index link predictor.

10.84 include/linkpred/predictors/undirected/ukabpredictor.hpp File Reference

Contains the implementation of a scalable popularity-similarity link predictor.

```
#include "linkpred/predictors/undirected/ulpredictor.hpp"
#include "linkpred/graphalg/shortestpaths/netdistcalculator.hpp"
#include "linkpred/utils/log.hpp"
#include <memory>
#include <cmath>
#include <limits>
#include <iostream>
```

Include dependency graph for ukabpredictor.hpp:

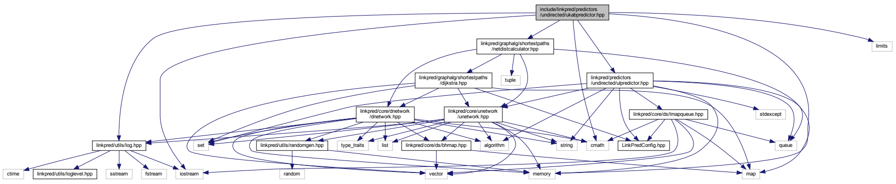

This graph shows which files directly or indirectly include this file:

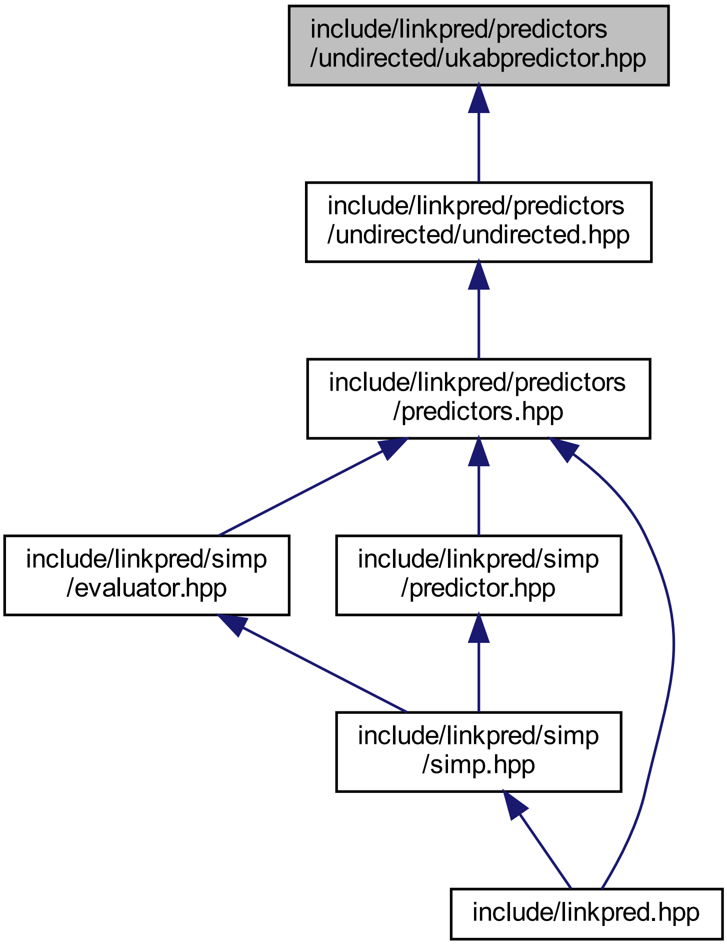

Classes

- class LinkPred::UKABPredictor< Network, EdgeRndIt, ScoreRndIt, EdgeRndOutIt >

  A scalable popularity similarity link predictor proposed in: "Kerrache, S., Alharbi, R. & Benhidour, H. A Scalable Similarity-Popularity Link Prediction Method. Sci Rep 10, 6394 (2020)". https://doi.org/10.1038/s41598-020-62636-1.

Namespaces

- LinkPred

  Main namespace.

10.84.1 Detailed Description

Contains the implementation of a scalable popularity-similarity link predictor.

10.85 include/linkpred/predictors/undirected/ulcppredictor.hpp File Reference

Contains the implementation of a local path link predictor.

```
#include <linkpred/predictors/undirected/ulpredictor.hpp>
#include <memory>
```

Include dependency graph for ulcppredictor.hpp:

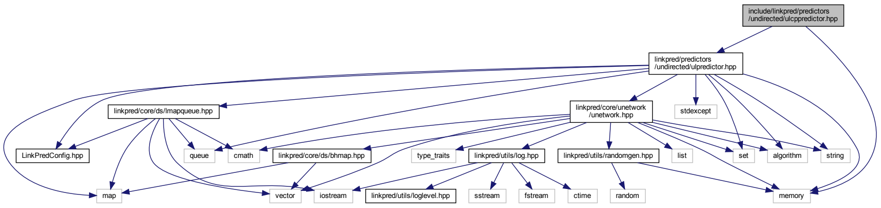

This graph shows which files directly or indirectly include this file:

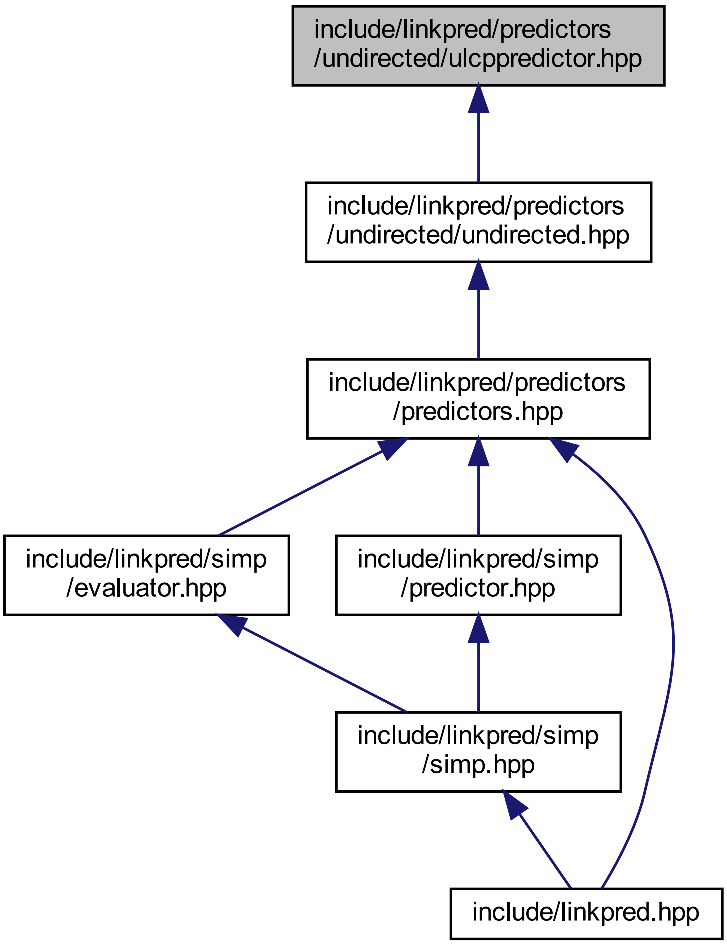

Classes

- class LinkPred::ULCPPredictor< Network, EdgeRndIt, ScoreRndIt, EdgeRndOutIt >

    Local path link predictor.

Namespaces

- LinkPred

    Main namespace.

10.85.1 Detailed Description

Contains the implementation of a local path link predictor.

10.86 include/linkpred/predictors/undirected/ulhnpredictor.hpp File Reference

Contains the implementation of a Leicht-Holme-Newman index link predictor.

```
#include <linkpred/predictors/undirected/ulpredictor.hpp>
#include <memory>
```

Include dependency graph for ulhnpredictor.hpp:

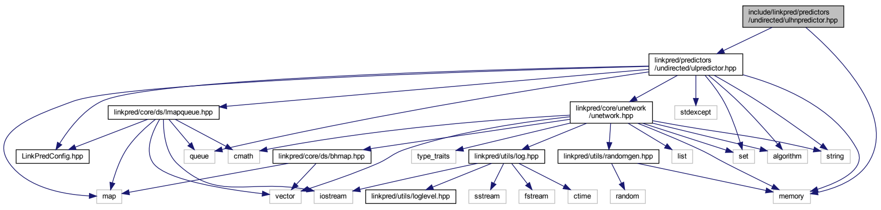

This graph shows which files directly or indirectly include this file:

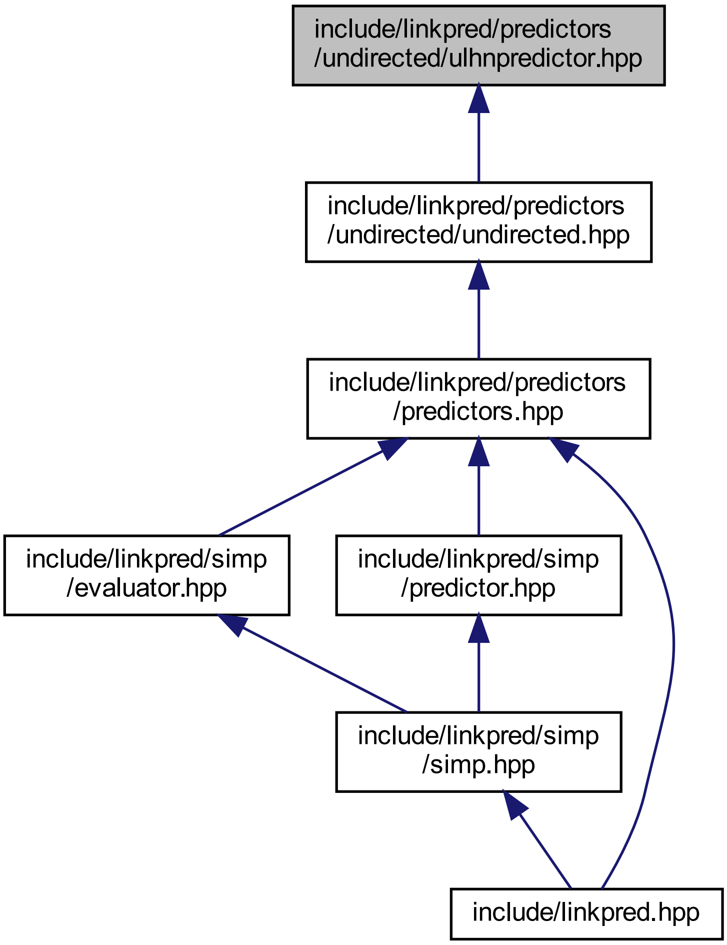

Classes

- class LinkPred::ULHNPredictor< Network, EdgeRndIt, ScoreRndIt, EdgeRndOutIt >

  Leicht-Holme-Newman index link predictor.

Namespaces

- LinkPred

  Main namespace.

10.86.1 Detailed Description

Contains the implementation of a Leicht-Holme-Newman index link predictor.

10.87 include/linkpred/predictors/undirected/ulpredictor.hpp File Reference

Contains the interface of a link predictor.

```
#include "LinkPredConfig.hpp"
#include "linkpred/core/unetwork/unetwork.hpp"
#include "linkpred/core/ds/lmapqueue.hpp"
#include <memory>
#include <map>
#include <string>
#include <stdexcept>
#include <algorithm>
#include <queue>
#include <set>
```

Include dependency graph for ulpredictor.hpp:

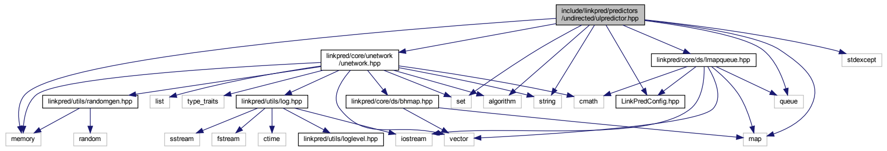

This graph shows which files directly or indirectly include this file:

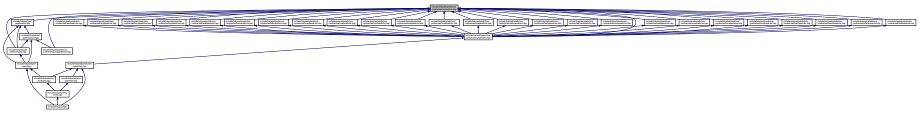

Classes

- class LinkPred::ULPredictor< NetworkT, EdgeRndItT, ScoreRndItT, EdgeRndOutItT >

  The interface of a link predictor in an undirected network.

Namespaces

- LinkPred

  Main namespace.

10.87.1 Detailed Description

Contains the interface of a link predictor.

10.88 include/linkpred/predictors/undirected/umpspredictor.hpp File Reference

Contains the implementation of a scalable popularity similarity link predictor.

```
#include <linkpred/predictors/undirected/ulpredictor.hpp>
#include "linkpred/graphalg/shortestpaths/netdistcalculator.hpp"
#include "linkpred/perf/perfmeasure.hpp"
#include "linkpred/utils/log.hpp"
#include <memory>
#include <cmath>
#include <limits>
```

Include dependency graph for umpspredictor.hpp:

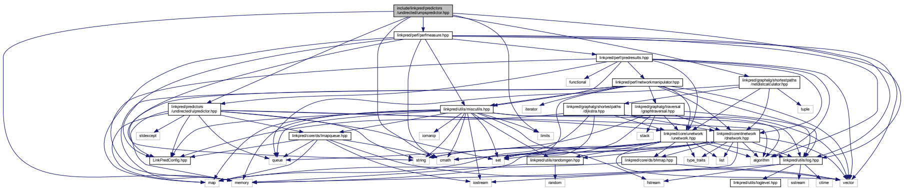

Classes

- class LinkPred::UMPSPredictor< Network, EdgeRndIt, ScoreRndIt, EdgeRndOutIt >

  A scalable popularity similarity link predictor.

Namespaces

- LinkPred

  Main namespace.

10.88.1 Detailed Description

Contains the implementation of a scalable popularity similarity link predictor.

10.89 include/linkpred/predictors/undirected/undirected.hpp File Reference

Includes the headers of link predictors for undirected networks.

```
#include "linkpred/predictors/undirected/uesmpredictor.hpp"
#include "linkpred/predictors/undirected/ueclpredictor.hpp"
#include "linkpred/predictors/undirected/uadapredictor.hpp"
#include "linkpred/predictors/undirected/ucnepredictor.hpp"
#include "linkpred/predictors/undirected/ucrapredictor.hpp"
#include "linkpred/predictors/undirected/ucstpredictor.hpp"
#include "linkpred/predictors/undirected/ufbmpredictor.hpp"
```

```
#include "linkpred/predictors/undirected/uhdipredictor.hpp"
#include "linkpred/predictors/undirected/uhpipredictor.hpp"
#include "linkpred/predictors/undirected/uhrgpredictor.hpp"
#include "linkpred/predictors/undirected/uhyppredictor.hpp"
#include "linkpred/predictors/undirected/ujidpredictor.hpp"
#include "linkpred/predictors/undirected/ukabpredictor.hpp"
#include "linkpred/predictors/undirected/ulcppredictor.hpp"
#include "linkpred/predictors/undirected/ulhnpredictor.hpp"
#include "linkpred/predictors/undirected/ulpredictor.hpp"
#include "linkpred/predictors/undirected/unedpredictor.hpp"
#include "linkpred/predictors/undirected/upatpredictor.hpp"
#include "linkpred/predictors/undirected/upstpredictor.hpp"
#include "linkpred/predictors/undirected/uralpredictor.hpp"
#include "linkpred/predictors/undirected/urndpredictor.hpp"
#include "linkpred/predictors/undirected/usaipredictor.hpp"
#include "linkpred/predictors/undirected/usbmpredictor.hpp"
#include "linkpred/predictors/undirected/ushppredictor.hpp"
#include "linkpred/predictors/undirected/usoipredictor.hpp"
#include "linkpred/predictors/undirected/usumpredictor.hpp"
```

Include dependency graph for undirected.hpp:

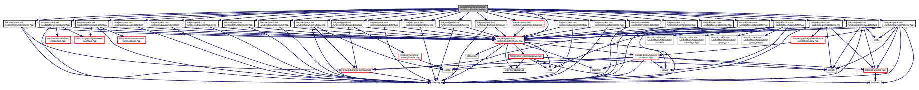

This graph shows which files directly or indirectly include this file:

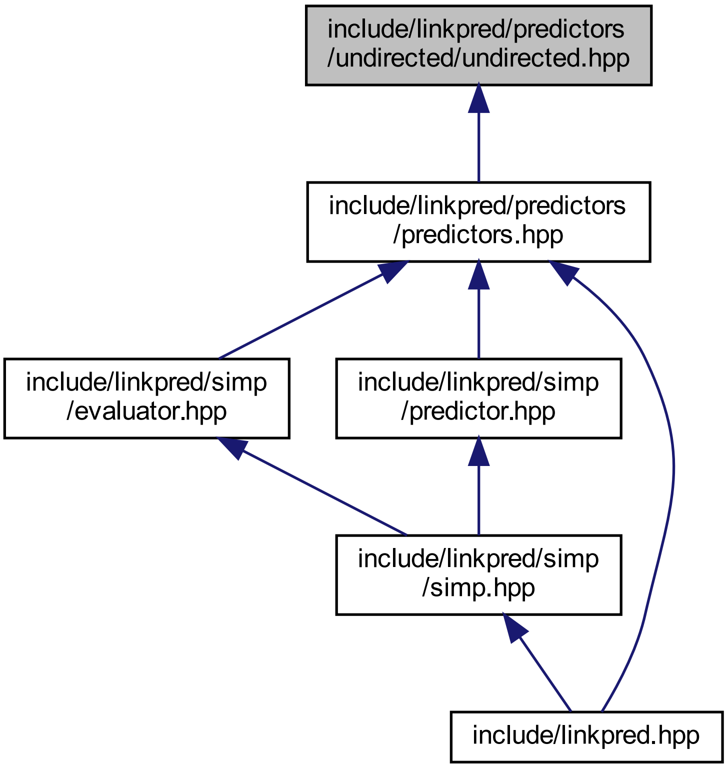

10.89.1 Detailed Description

Includes the headers of link predictors for undirected networks.

10.90 include/linkpred/predictors/undirected/unedpredictor.hpp File Reference

Contains the implementation of a link prediction algorithm based on the degrees of neighbors.

```
#include <linkpred/predictors/undirected/ulpredictor.hpp>
#include "linkpred/utils/log.hpp"
#include <memory>
#include <cmath>
```

`#include <limits>`

Include dependency graph for unedpredictor.hpp:

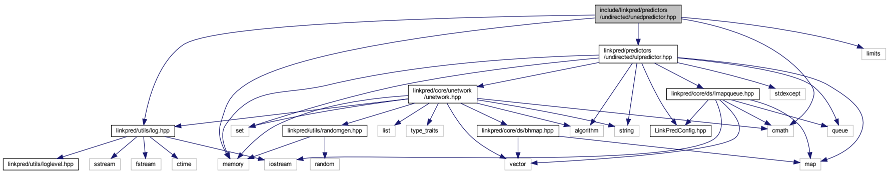

This graph shows which files directly or indirectly include this file:

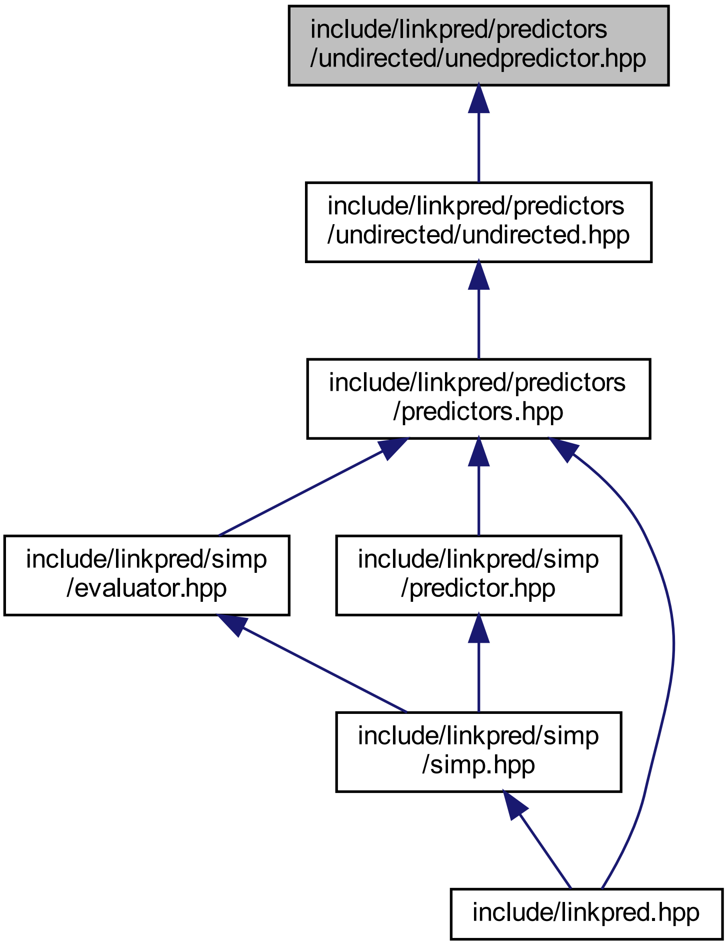

Classes

- class LinkPred::UNEDPredictor< Network, EdgeRndIt, ScoreRndIt, EdgeRndOutIt >

    A neighbors degree link predictor.

Namespaces

- [LinkPred]

  Main namespace.

10.90.1 Detailed Description

Contains the implementation of a link prediction algorithm based on the degrees of neighbors.

10.91 include/linkpred/predictors/undirected/upatpredictor.hpp File Reference

Contains the implementation of a preferential attachment link predictor.

#include <linkpred/predictors/undirected/ulpredictor.hpp>
#include <memory>
Include dependency graph for upatpredictor.hpp:

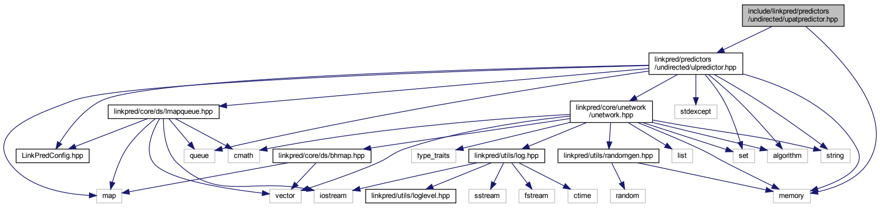

This graph shows which files directly or indirectly include this file:

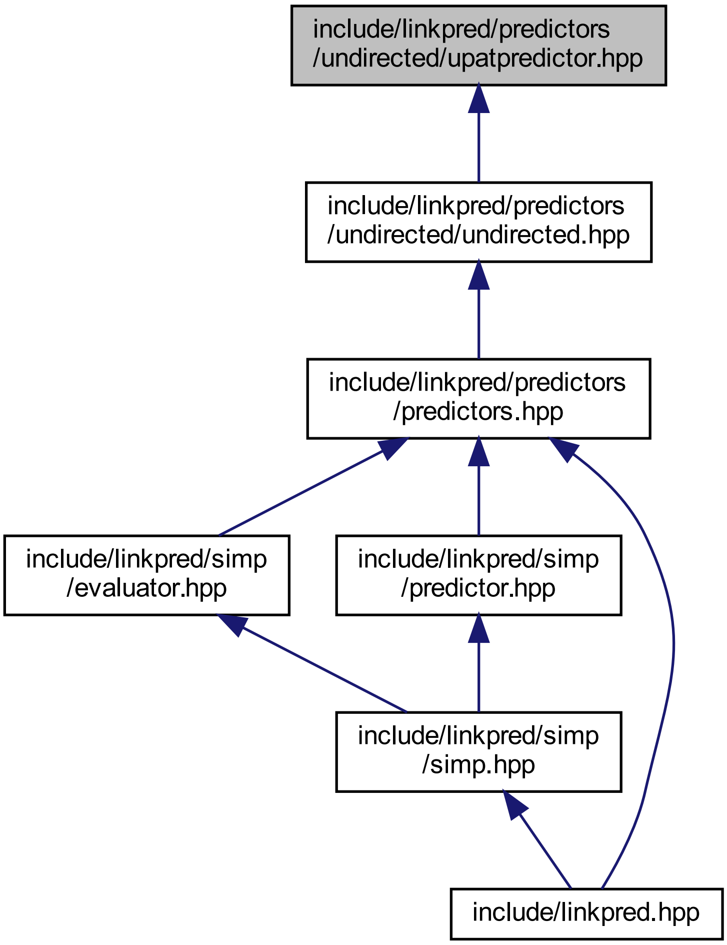

Classes

- class LinkPred::UPATPredictor< Network, EdgeRndIt, ScoreRndIt, EdgeRndOutIt >

  A preferential attachment link predictor.

Namespaces

- LinkPred

  Main namespace.

10.91.1 Detailed Description

Contains the implementation of a preferential attachment link predictor.

10.92 include/linkpred/predictors/undirected/upstpredictor.hpp File Reference

Contains the implementation of a link predictor that prestores the scores of edges.

```
#include <linkpred/predictors/undirected/ulpredictor.hpp>
#include <memory>
```

Include dependency graph for upstpredictor.hpp:

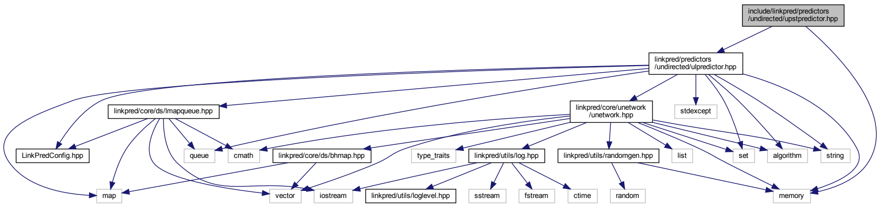

This graph shows which files directly or indirectly include this file:

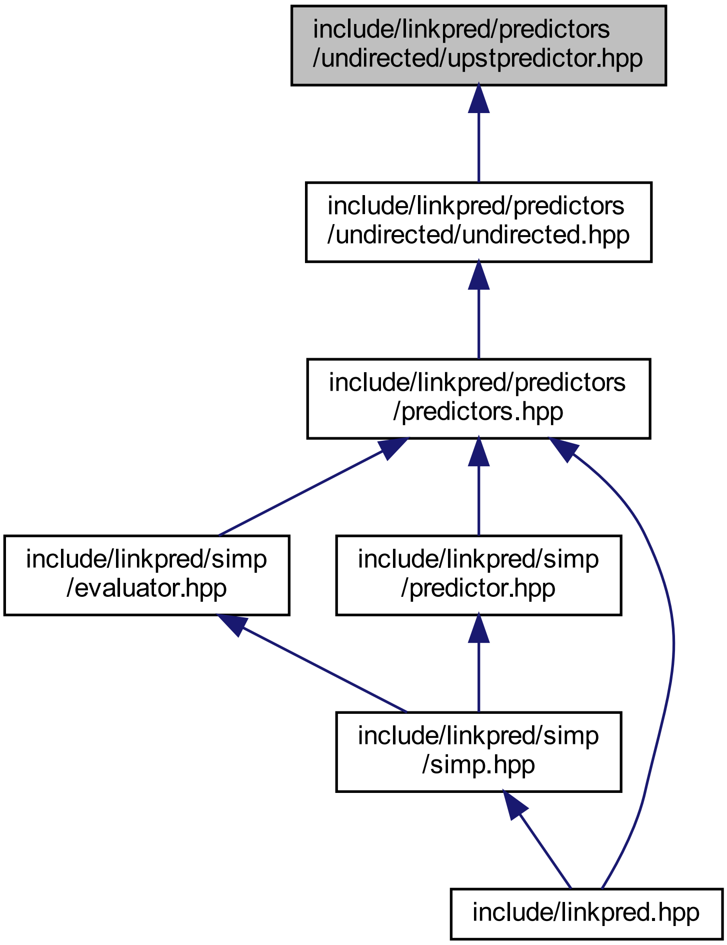

Classes

- class LinkPred::UPSTPredictor< Network, EdgeRndIt, ScoreRndIt, EdgeRndOutIt >

  A link predictor that prestores edge scores. This allows to seemingly integrate results from external link prediction algorithms to LinkPred (for example, users may implement their own link prediction algorithm and then use this link predictor to use compare their results to algorithms available in LinkPred).

Namespaces

- LinkPred

  Main namespace.

10.92.1 Detailed Description

Contains the implementation of a link predictor that prestores the scores of edges.

10.93 include/linkpred/predictors/undirected/uralpredictor.hpp File Reference

Contains the implementation of a resource allocation link predictor.

```
#include <linkpred/predictors/undirected/ulpredictor.hpp>
#include <memory>
```

Include dependency graph for uralpredictor.hpp:

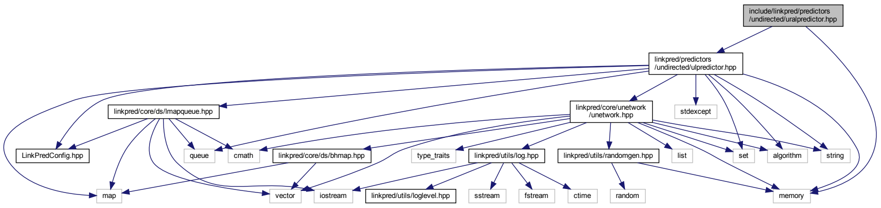

This graph shows which files directly or indirectly include this file:

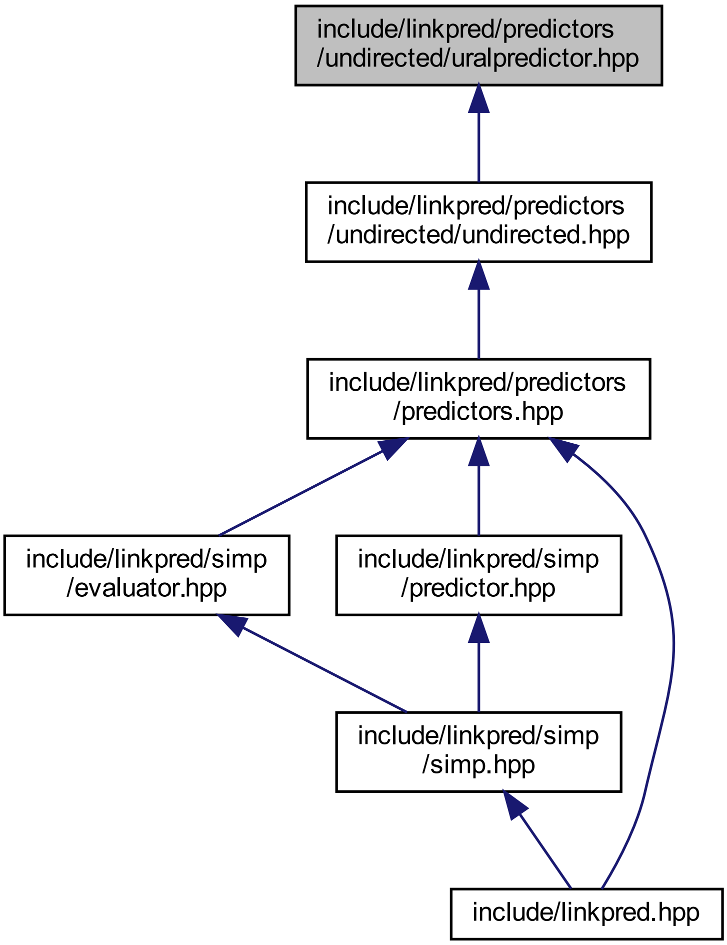

Classes

- class LinkPred::URALPredictor< Network, EdgeRndIt, ScoreRndIt, EdgeRndOutIt >

    A resource allocation link predictor.

Namespaces

- LinkPred

    Main namespace.

10.93.1 Detailed Description

Contains the implementation of a resource allocation link predictor.

10.94 include/linkpred/predictors/undirected/urndpredictor.hpp File Reference

Contains the implementation of a random link predictor.

```
#include <linkpred/predictors/undirected/ulpredictor.hpp>
#include "linkpred/utils/randomgen.hpp"
#include <memory>
```

Include dependency graph for urndpredictor.hpp:

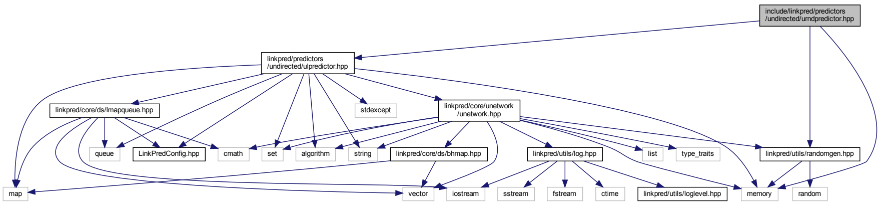

This graph shows which files directly or indirectly include this file:

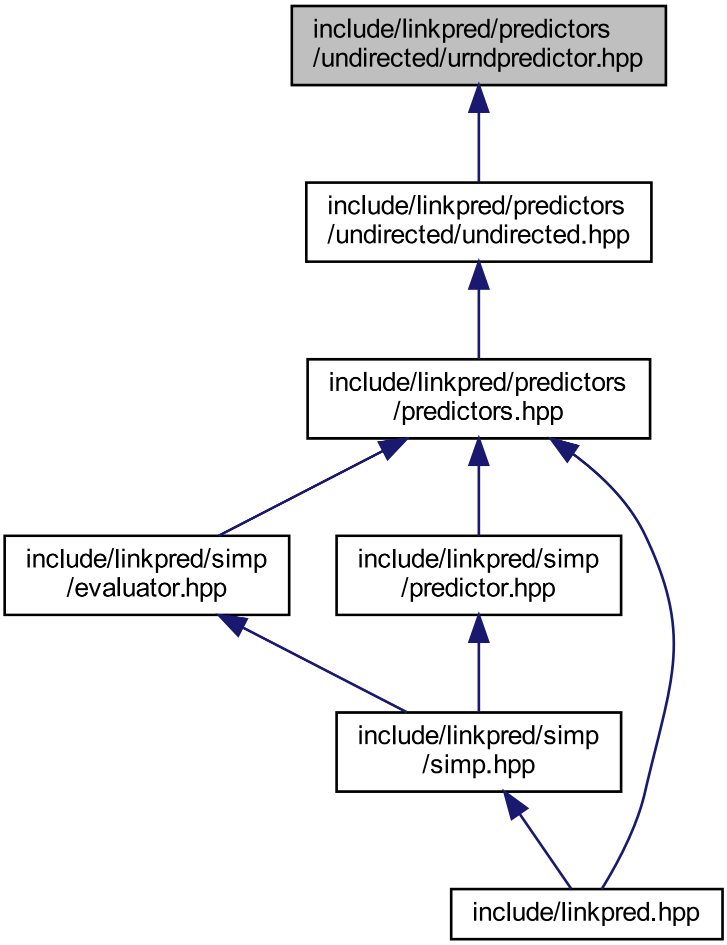

Classes

- class LinkPred::URNDPredictor< Network, EdgeRndIt, ScoreRndIt, EdgeRndOutIt >

    A random link predictor.

Namespaces

- LinkPred

    Main namespace.

10.94.1 Detailed Description

Contains the implementation of a random link predictor.

10.95 include/linkpred/predictors/undirected/usaipredictor.hpp File Reference

Contains the implementation of a Salton index link predictor.

```
#include <linkpred/predictors/undirected/ulpredictor.hpp>
#include <memory>
```

Include dependency graph for usaipredictor.hpp:

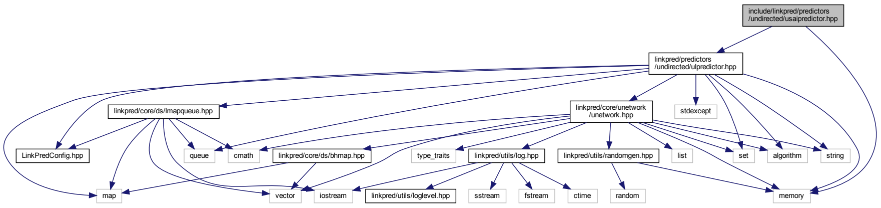

This graph shows which files directly or indirectly include this file:

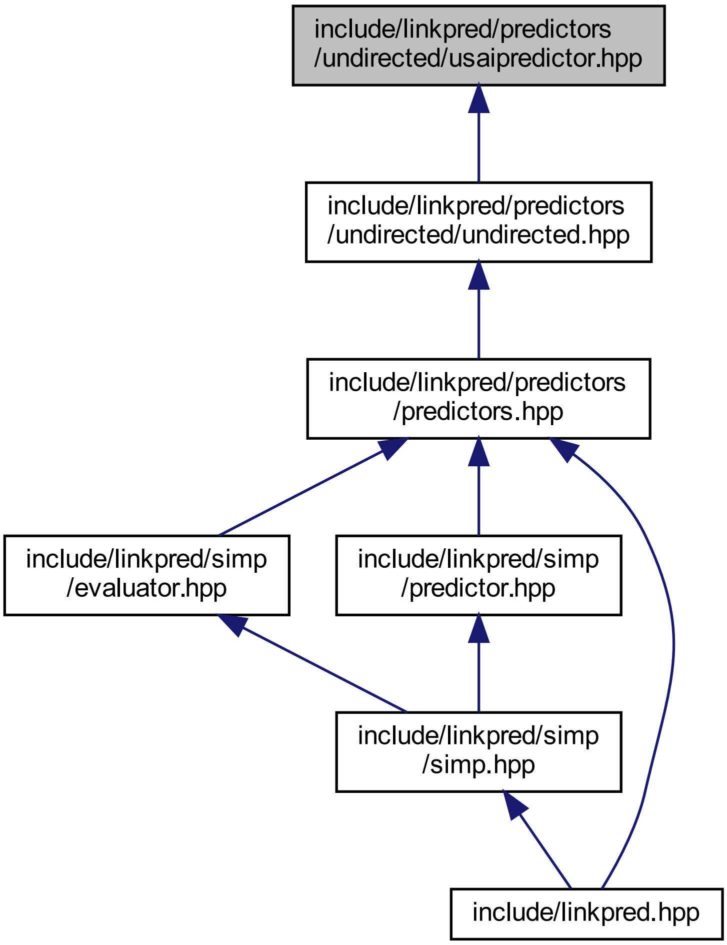

Classes

- class LinkPred::USAIPredictor< Network, EdgeRndIt, ScoreRndIt, EdgeRndOutIt >

  A Salton index link predictor.

Namespaces

- LinkPred

  Main namespace.

10.95.1 Detailed Description

Contains the implementation of a Salton index link predictor.

10.96 include/linkpred/predictors/undirected/usbmpredictor.hpp File Reference

Contains the implementation of the stochastic block model link predictor.

`#include <linkpred/predictors/undirected/ulpredictor.hpp>`
`#include <linkpred/predictors/undirected/usbmpredictor/sbm.hpp>`

Include dependency graph for usbmpredictor.hpp:

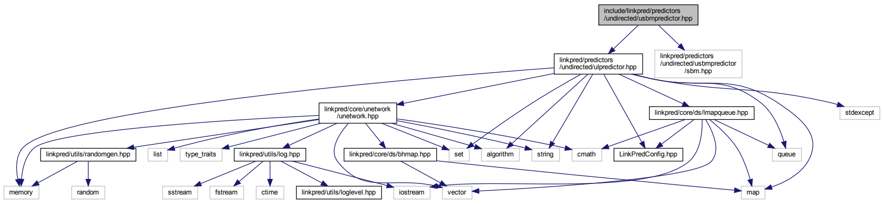

This graph shows which files directly or indirectly include this file:

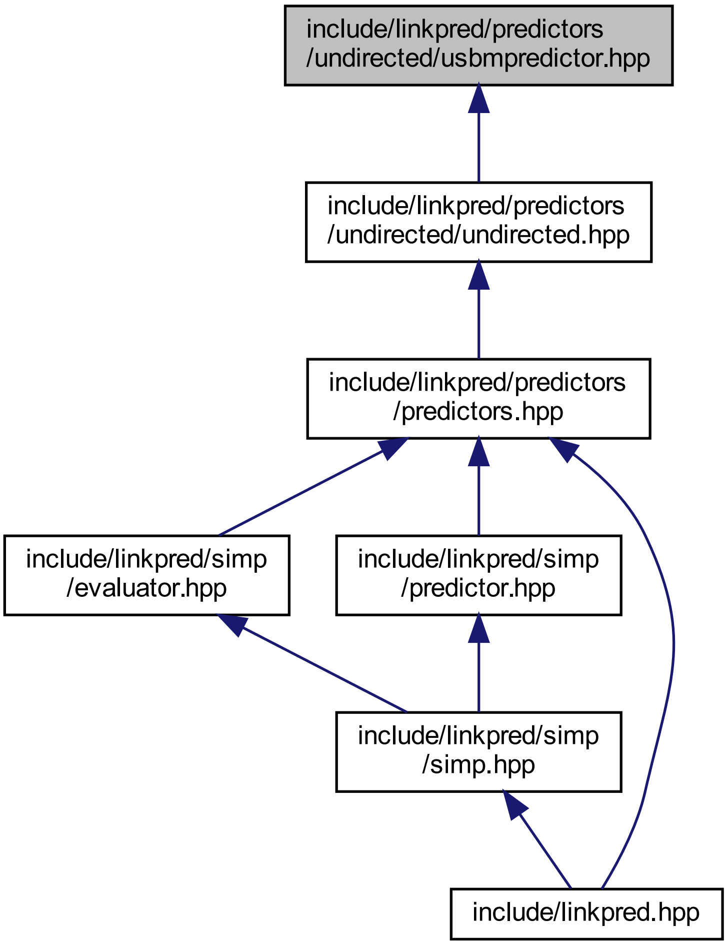

Classes

- class LinkPred::USBMPredictor< Network, EdgeRndIt, ScoreRndIt, EdgeRndOutIt >

    The stochastic block model link predictor.

Namespaces

- LinkPred

    Main namespace.

10.96.1 Detailed Description

Contains the implementation of the stochastic block model link predictor.

10.97 include/linkpred/predictors/undirected/ushppredictor.hpp File Reference

Contains the implementation of a shortest path link predictor.

```
#include <linkpred/predictors/undirected/ulpredictor.hpp>
#include "linkpred/graphalg/shortestpaths/netdistcalculator.hpp"
#include "linkpred/utils/log.hpp"
#include <memory>
#include <cmath>
```

Include dependency graph for ushppredictor.hpp:

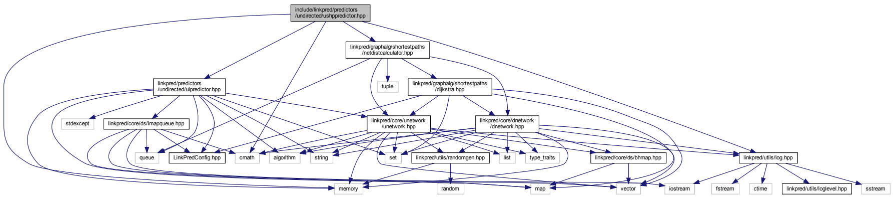

This graph shows which files directly or indirectly include this file:

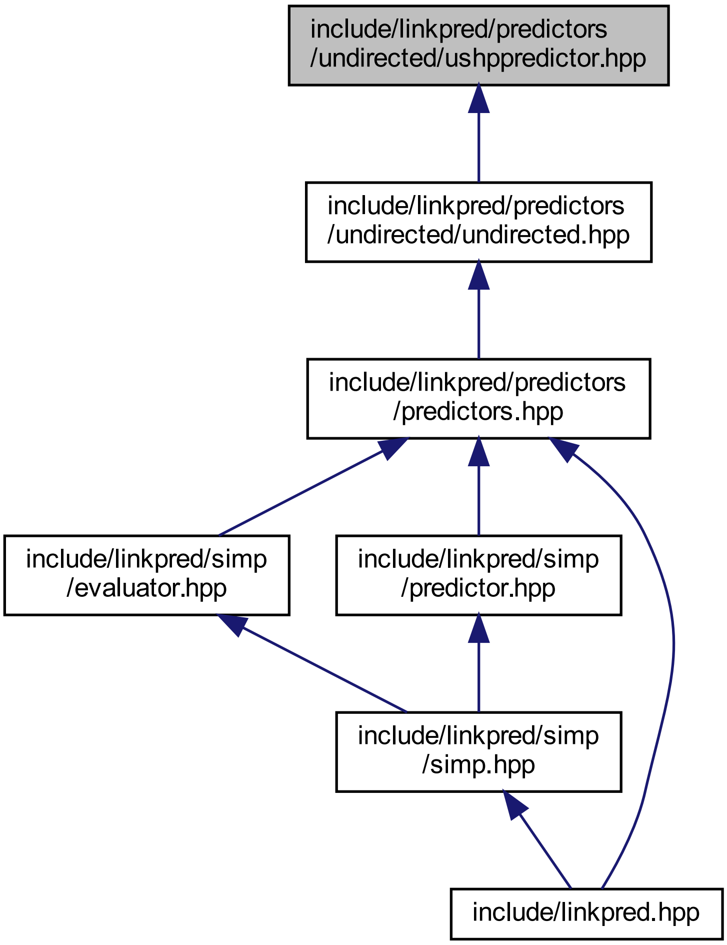

Classes

- class LinkPred::USHPPredictor< Network, EdgeRndIt, ScoreRndIt, EdgeRndOutIt >

  A shortest path link predictor link predictor.

Namespaces

- LinkPred

  Main namespace.

10.97.1 Detailed Description

Contains the implementation of a shortest path link predictor.

10.98 include/linkpred/predictors/undirected/usoipredictor.hpp File Reference

Contains the implementation of a Sorensen index link predictor.

```
#include <linkpred/predictors/undirected/ulpredictor.hpp>
#include <memory>
```

Include dependency graph for usoipredictor.hpp:

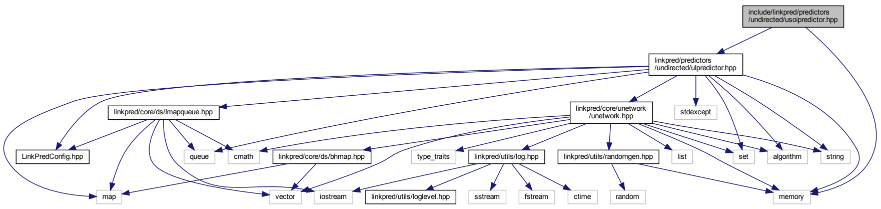

This graph shows which files directly or indirectly include this file:

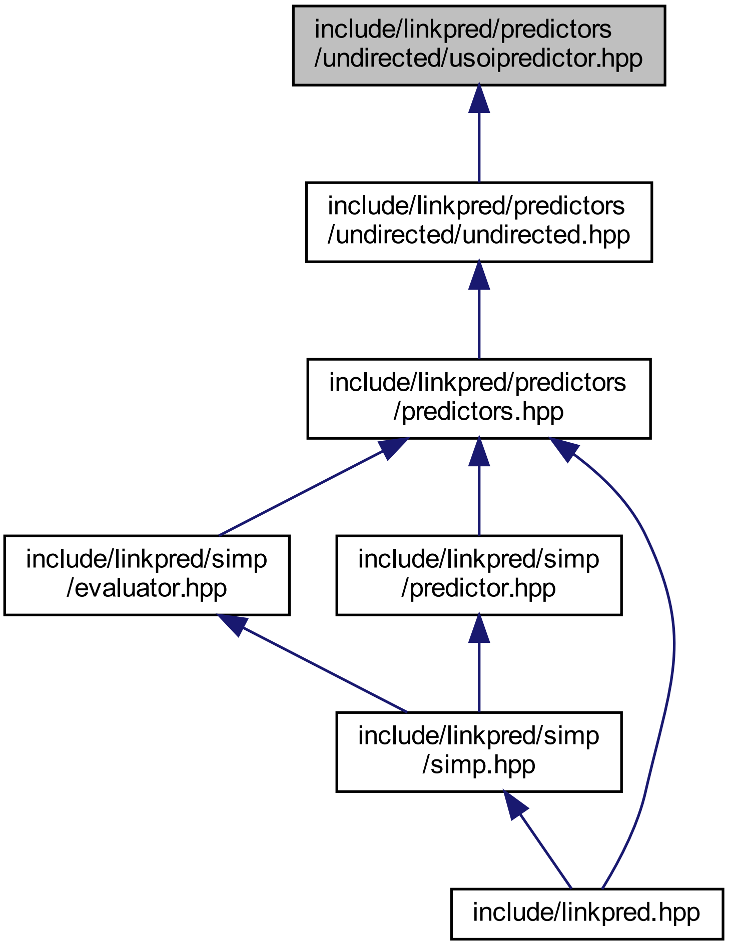

Classes

- class LinkPred::USOIPredictor< Network, EdgeRndIt, ScoreRndIt, EdgeRndOutIt >

  A Sorensen index link predictor.

Namespaces

- LinkPred

  Main namespace.

10.98.1 Detailed Description

Contains the implementation of a Sorensen index link predictor.

10.99 include/linkpred/predictors/undirected/usumpredictor.hpp File Reference

Contains the implementation of a sum-of-degrees link predictor.

```
#include <linkpred/predictors/undirected/ulpredictor.hpp>
#include <memory>
```

Include dependency graph for usumpredictor.hpp:

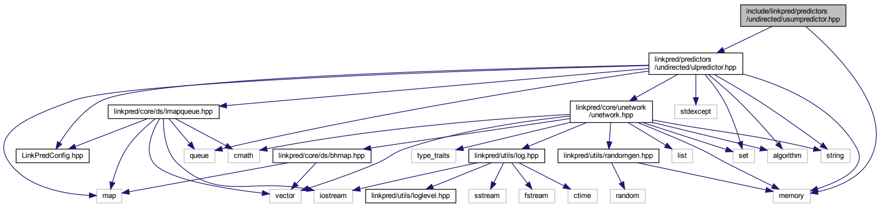

This graph shows which files directly or indirectly include this file:

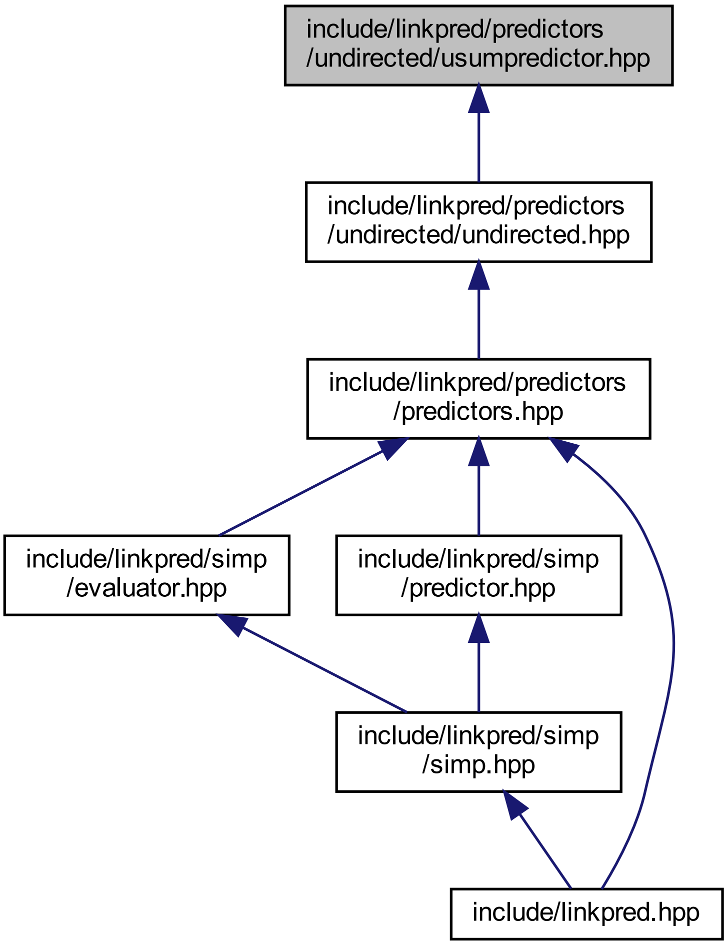

Classes

- class LinkPred::USUMPredictor< Network, EdgeRndIt, ScoreRndIt, EdgeRndOutIt >

  A sum-of-degrees link predictor.

Namespaces

- LinkPred

  Main namespace.

10.99.1 Detailed Description

Contains the implementation of a sum-of-degrees link predictor.

10.100 include/linkpred/simp/edgescore.hpp File Reference

Contains the definition of a structure to store the score of an edge.

`#include <string>`
Include dependency graph for edgescore.hpp:

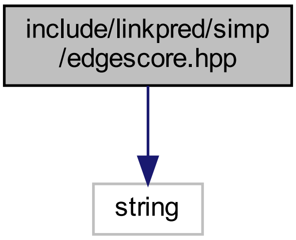

This graph shows which files directly or indirectly include this file:

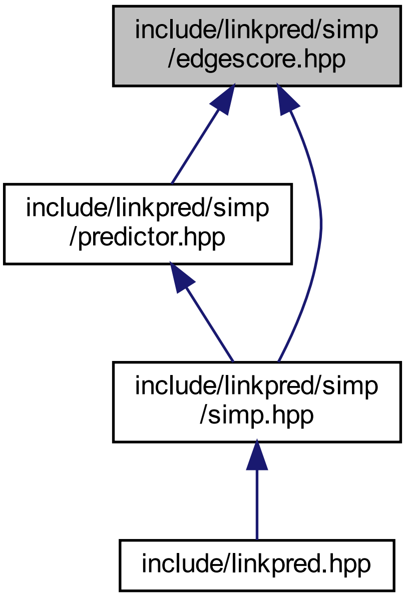

Classes

- struct LinkPred::Simp::EdgeScore

  A structure to store the score of an edge.
- struct LinkPred::Simp::EdgeScoreByID

  A structure to store the score of an edge. The node IDs are used instead of labels.

Namespaces

- LinkPred

    Main namespace.
- LinkPred::Simp

    Simplified interface. Contains a simplified interface for LinkPred that includes the essential functionalities.

10.100.1 Detailed Description

Contains the definition of a structure to store the score of an edge.

10.101 include/linkpred/simp/evaluator.hpp File Reference

Contains the definition of a class that simplifies the evaluation of link prediction algorithms.

```
#include "LinkPredConfig.hpp"
#include "linkpred/simp/perfres.hpp"
#include "linkpred/predictors/predictors.hpp"
#include "linkpred/perf/perf.hpp"
#include <memory>
#include <vector>
#include <map>
#include <set>
```
Include dependency graph for evaluator.hpp:

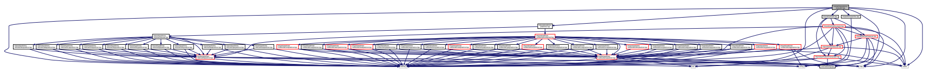

This graph shows which files directly or indirectly include this file:

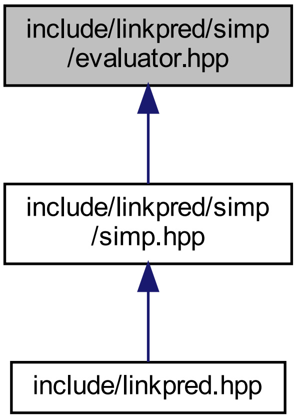

Classes

- class LinkPred::Simp::Evaluator

    A class that simplifies the evaluation of link prediction algorithms.
- struct LinkPred::Simp::Evaluator::Factory::ECLParams

    Parameters of ECL.
- struct LinkPred::Simp::Evaluator::Factory::ESMParams

    Parameters of ESM.
- struct LinkPred::Simp::Evaluator::Factory::FBMParams

    Parameters of FBM.
- struct LinkPred::Simp::Evaluator::Factory::HRGParams

    Parameters of HRG.
- struct LinkPred::Simp::Evaluator::Factory::HYPParams

    Parameters of HYP.
- struct LinkPred::Simp::Evaluator::Factory::KABParams

    Parameters of KAB.
- struct LinkPred::Simp::Evaluator::Factory::LCPParams

    Parameters of LCP.
- struct LinkPred::Simp::Evaluator::Factory::PSTParams

    Parameters of PST.
- struct LinkPred::Simp::Evaluator::Factory::RNDParams

    Parameters of RND.
- struct LinkPred::Simp::Evaluator::Factory::SBMParams

    Parameters of SBM.
- struct LinkPred::Simp::Evaluator::Factory::SHPParams

    Parameters of SHP.

Namespaces

- LinkPred

    Main namespace.
- LinkPred::Simp

    Simplified interface. Contains a simplified interface for LinkPred that includes the essential functionalities.

10.101.1 Detailed Description

Contains the definition of a class that simplifies the evaluation of link prediction algorithms.

10.102 include/linkpred/simp/perfres.hpp File Reference

Contains the definition of a structure to store performance results.

`#include <string>`

Include dependency graph for perfres.hpp:

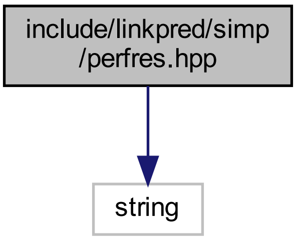

This graph shows which files directly or indirectly include this file:

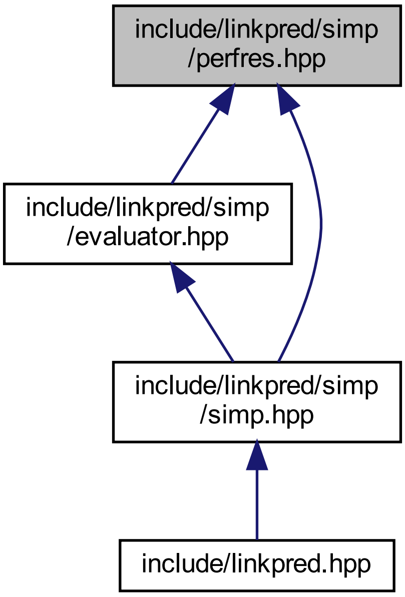

Classes

- struct LinkPred::Simp::PerfRes

    A structure to store performance results.

Namespaces

- LinkPred

  Main namespace.
- LinkPred::Simp

  Simplified interface. Contains a simplified interface for LinkPred that includes the essential functionalities.

10.102.1 Detailed Description

Contains the definition of a structure to store performance results.

10.103 include/linkpred/simp/predictor.hpp File Reference

Contains the definition of a class that simplifies the use of link prediction algorithms.

```
#include "LinkPredConfig.hpp"
#include "linkpred/simp/edgescore.hpp"
#include "linkpred/predictors/predictors.hpp"
#include "linkpred/graphalg/encoders/encoders.hpp"
#include "linkpred/ml/classifiers/classifiers.hpp"
#include "linkpred/ml/simmeasures/simmeasures.hpp"
```

Include dependency graph for predictor.hpp:

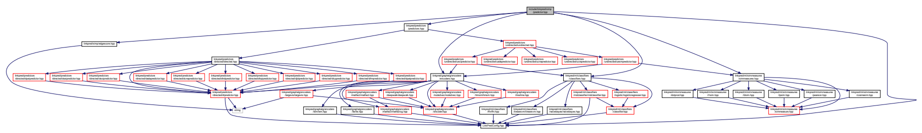

This graph shows which files directly or indirectly include this file:

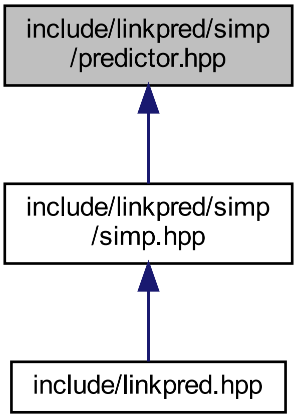

Classes

- class LinkPred::Simp::Predictor

    A class that simplifies the use of link prediction algorithms.

Namespaces

- LinkPred

    Main namespace.
- LinkPred::Simp

    Simplified interface. Contains a simplified interface for LinkPred that includes the essential functionalities.

10.103.1 Detailed Description

Contains the definition of a class that simplifies the use of link prediction algorithms.

10.104 include/linkpred/simp/simp.hpp File Reference

Includes all headers of the essential interface.

```
#include "edgescore.hpp"
#include "predictor.hpp"
#include "perfres.hpp"
#include "evaluator.hpp"
```

Include dependency graph for simp.hpp:

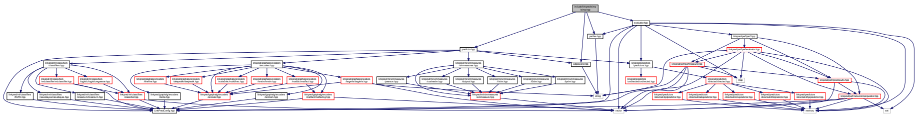

This graph shows which files directly or indirectly include this file:

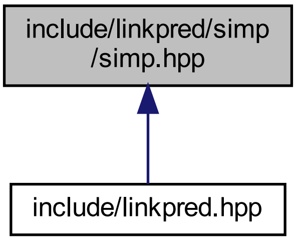

Namespaces

- LinkPred

    Main namespace.
- LinkPred::Simp

    Simplified interface. Contains a simplified interface for LinkPred that includes the essential functionalities.

10.104.1 Detailed Description

Includes all headers of the essential interface.

10.105 include/linkpred/utils/log.hpp File Reference

Contains the implementation of a log class.

```
#include "linkpred/utils/loglevel.hpp"
#include <sstream>
#include <iostream>
#include <fstream>
#include <ctime>
```

Include dependency graph for log.hpp:

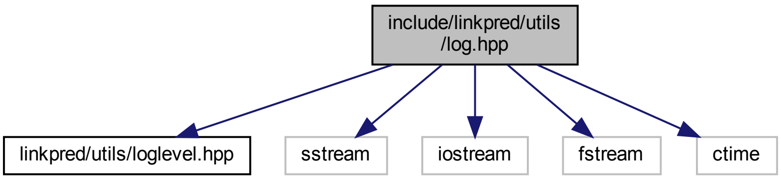

This graph shows which files directly or indirectly include this file:

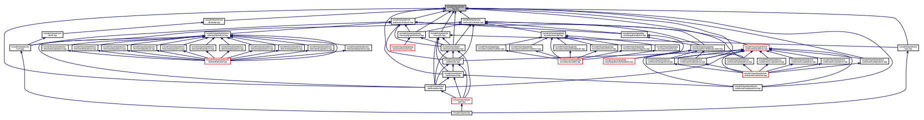

Classes

- class LinkPred::Log

    A log class.

Namespaces

- LinkPred

    Main namespace.

Macros

- #define logger(level, message)

10.105.1 Detailed Description

Contains the implementation of a log class.

10.105.2 Macro Definition Documentation

10.105.2.1 logger

```
#define logger(
              level,
              message )
```

Value:
```
if (level > Log::logLevel) ; \
else Log(level) « std::string(__FILE__)« " in "« std::string(__FUNCTION__) « ":" « __LINE__ « " : " «
       message;
```

Macro for writing to log.

Parameters

level	The message log level.
message	The text that is added to the log.

10.106 include/linkpred/utils/loglevel.hpp File Reference

Contains the definition of log levels.

This graph shows which files directly or indirectly include this file:

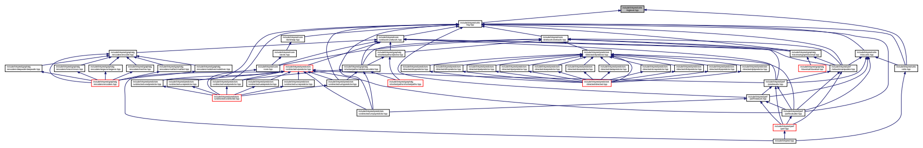

Namespaces

- LinkPred

  Main namespace.

Enumerations

- enum LinkPred::LogLevel {
  LinkPred::logError, LinkPred::logWarning, LinkPred::logInfo, LinkPred::logDebug,
  LinkPred::logDebug1, LinkPred::logDebug2, LinkPred::logDebug3 }

  Enumeration of log levels.

10.106.1 Detailed Description

Contains the definition of log levels.

10.107 include/linkpred/utils/miscutils.hpp File Reference

Contains the implementation of miscellaneous useful methods.

```
#include "LinkPredConfig.hpp"
#include "linkpred/utils/randomgen.hpp"
#include "linkpred/utils/log.hpp"
#include "linkpred/core/ds/lmapqueue.hpp"
#include <string>
#include <cmath>
#include <map>
#include <memory>
#include <set>
#include <queue>
#include <fstream>
#include <iostream>
#include <limits>
#include <algorithm>
#include <iomanip>
```

Include dependency graph for miscutils.hpp:

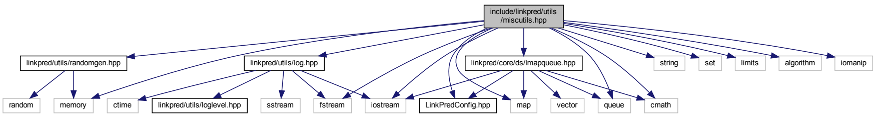

This graph shows which files directly or indirectly include this file:

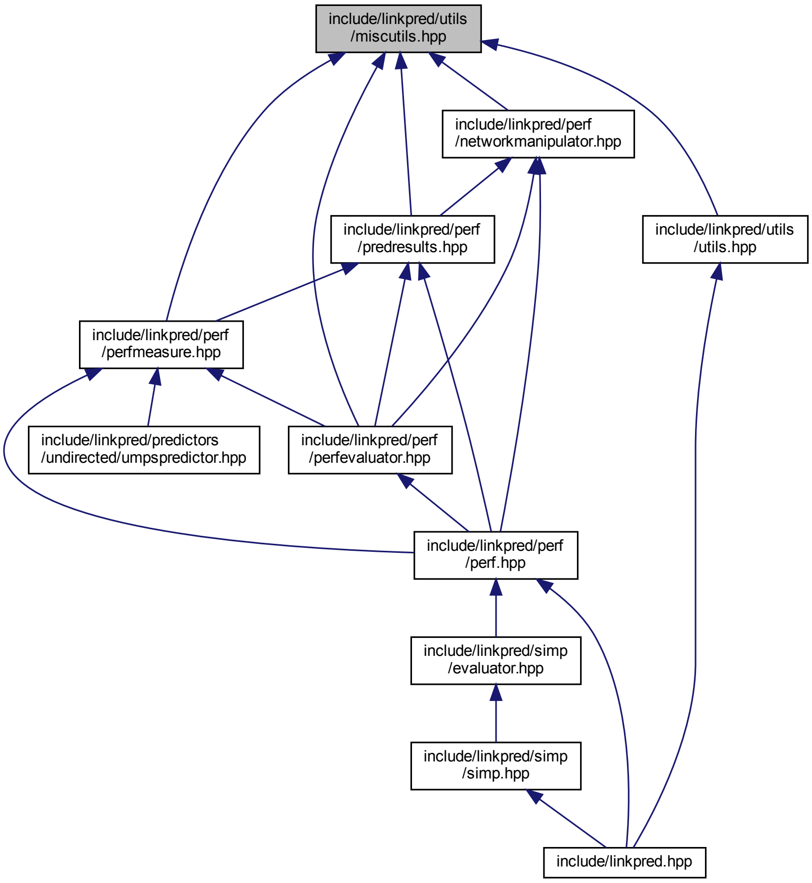

Classes

- struct LinkPred::Utils::PairCompRight< FirstT, SecondT, CompareT >

    Class for comparing pairs based on second elements only.
- struct LinkPred::Utils::EdgeScore< Label >

    A structure to store the score of an edge.

Namespaces

- LinkPred

    Main namespace.
- LinkPred::Utils

    Some utility functions.

Enumerations

- enum LinkPred::SortOrder { LinkPred::None, LinkPred::Inc, LinkPred::Dec }

    Enumeration of different sorting orders.

Functions

- std::string & LinkPred::Utils::ltrim (std::string &str, const char *spaces=" \t\n\r\f\v")
- std::string & LinkPred::Utils::rtrim (std::string &str, const char *spaces=" \t\n\r\f\v")
- std::string & LinkPred::Utils::trim (std::string &str, const char *spaces=" \t\n\r\f\v")
- template< typename T >
  void LinkPred::Utils::clear (std::queue< T > &q)
- template< typename IteratorT >
  void LinkPred::Utils::sort (IteratorT begin, IteratorT end, SortOrder sortOrder)
- std::vector< std::size_t > LinkPred::Utils::getRndPerm (std::size_t n)
- std::vector< std::size_t > LinkPred::Utils::getRndPerm (std::size_t n, long int seed)
- std::pair< double, double > LinkPred::Utils::plFit (std::vector< std::size_t > const &data)
- std::pair< double, double > LinkPred::Utils::plFit (std::vector< double > const &data)
- template< typename T >
  void LinkPred::Utils::print (std::vector< T > const &v, std::string name)
- template< typename U , typename V >
  std::pair< V, U > LinkPred::Utils::flip (const std::pair< U, V > &p)
- template< typename U , typename V >
  std::multimap< V, U > LinkPred::Utils::flipMap (const std::map< U, V > &map)
- template< typename RandomIterator >
  std::set< typename std::iterator_traits< RandomIterator >::value_type > LinkPred::Utils::selectRandom (RandomIterator begin, RandomIterator end, std::size_t k, long int seed)
- template< typename RandomIterator >
  void LinkPred::Utils::selectRandomInPlace (RandomIterator begin, RandomIterator end, std::size_t k, long int seed)
- template< typename T , typename InputIterator , typename InserterIt , typename Compare >
  void LinkPred::Utils::selectTopK (InputIterator begin, InputIterator end, InserterIt inserter, std::size_t k)
- template< typename RandomIterator >
  RandomIterator LinkPred::Utils::getRandom (RandomIterator begin, RandomIterator end, long int seed)
- template< typename T , typename InputIterator , typename InserterIt >
  void LinkPred::Utils::filter (InputIterator begin, InputIterator end, std::set< T > const &excepts, Inserter↩
  It inserter)
- template< typename InputIterator >
  void LinkPred::Utils::print (InputIterator begin, InputIterator end, std::string const &title, std::ostream &out)
- template< typename InputIterator >
  void LinkPred::Utils::print (InputIterator begin, InputIterator end, std::string const &title)
- template< typename InputIterator , typename Network >
  void LinkPred::Utils::printEdges (InputIterator begin, InputIterator end, Network const &net, std::string const &title, std::ostream &out)
- template< typename InputIterator , typename Network >
  void LinkPred::Utils::printEdges (InputIterator begin, InputIterator end, Network const &net, std::string const &title)
- template< typename InputIterator >
  double LinkPred::Utils::norm (InputIterator begin, InputIterator end)
- int LinkPred::Utils::int_cast (std::size_t source)
- template< typename InputIterator >
  void LinkPred::Utils::assertNoNaN (InputIterator begin, InputIterator end)
- std::pair< std::size_t, std::size_t > LinkPred::Utils::localRange (std::size_t n, int nbProcs, int procID)
- template< typename RandomIterator >
  void LinkPred::Utils::shuffle (RandomIterator begin, RandomIterator end, long int seed)
- template< typename Label = std::string>
  std::vector< std::pair< Label, Label > > LinkPred::Utils::readEdges (std::string fileName, bool ignore↩
  Loops=true)
- template< typename Label = std::string>
  std::vector< EdgeScore< Label > > LinkPred::Utils::readEdgeScores (std::string fileName)
- template< typename Label = std::string>
  void LinkPred::Utils::writeEdgeScores (std::string fileName, std::vector< EdgeScore< Label >> const &esv)

10.107.1 Detailed Description

Contains the implementation of miscellaneous useful methods.

10.108 include/linkpred/utils/randomgen.hpp File Reference

Contains the implementation of a random number generator.

```
#include <random>
#include <memory>
```

Include dependency graph for randomgen.hpp:

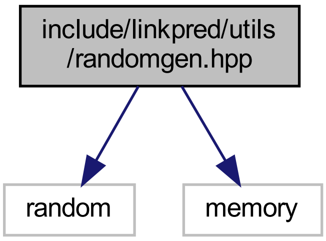

This graph shows which files directly or indirectly include this file:

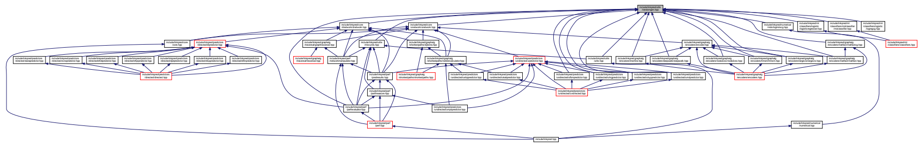

Classes

- class LinkPred::RandomGen

    A random number generator.

Namespaces

- LinkPred

    Main namespace.

10.108.1 Detailed Description

Contains the implementation of a random number generator.

10.109 include/linkpred/utils/utils.hpp File Reference

Includes the headers related to utility classes.

```
#include <linkpred/utils/miscutils.hpp>
#include "linkpred/utils/log.hpp"
#include "linkpred/utils/loglevel.hpp"
#include "linkpred/utils/randomgen.hpp"
```

Include dependency graph for utils.hpp:

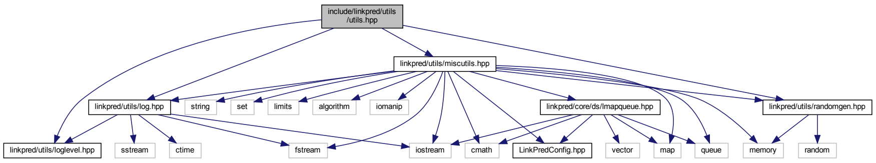

This graph shows which files directly or indirectly include this file:

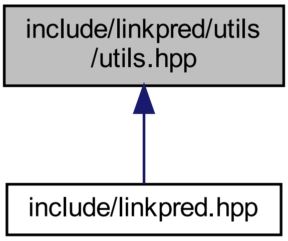

10.109.1 Detailed Description

Includes the headers related to utility classes.

Index

~ DESPLDistCalculator
LinkPred::DESPLDistCalculator< Network, DistType, NbHopsType >, 126
~ ESPLDistCalculator
LinkPred::ESPLDistCalculator< Network, DistType, NbHopsType >, 288
~ ESPLSimlCalculator
LinkPred::ESPLSimlCalculator< Network, DistType, NbHopsType >, 297
~ASPDistCalculator
LinkPred::ASPDistCalculator< Network, DistType, NbHopsType >, 63
~ASPDsimCalculator
LinkPred::ASPDsimCalculator< Network, DsimType, NbHopsType >, 70
~BFS
LinkPred::BFS< Network, NodeProcessor >, 75
~BHeap
LinkPred::BHeap< T, P, ComparatorT, ComparatorP >, 79
~Bhmap
LinkPred::Bhmap< K, P, Comparator >, 85
~Classifier
LinkPred::Classifier< InRndIt, OutRndIt, ScoreRndIt >, 90
~Collector
LinkPred::Collector< Network >, 94
~CosineSim
LinkPred::CosineSim, 97
~Counter
LinkPred::Counter< Network >, 100
~DADAPredictor
LinkPred::DADAPredictor< Network, EdgeRndIt, ScoreRndIt, EdgeRndOutIt >, 104
~DCNEPredictor
LinkPred::DCNEPredictor< Network, EdgeRndIt, ScoreRndIt, EdgeRndOutIt >, 110
~DFS
LinkPred::DFS< Network, NodeProcessor >, 133
~DHDIPredictor
LinkPred::DHDIPredictor< Network, EdgeRndIt, ScoreRndIt, EdgeRndOutIt >, 136
~DHPIPredictor
LinkPred::DHPIPredictor< Network, EdgeRndIt, ScoreRndIt, EdgeRndOutIt >, 142
~DJIDPredictor
LinkPred::DJIDPredictor< Network, EdgeRndIt, ScoreRndIt, EdgeRndOutIt >, 161
~DLCPPredictor
LinkPred::DLCPPredictor< Network, EdgeRndIt, ScoreRndIt, EdgeRndOutIt >, 166
~DLHNPredictor
LinkPred::DLHNPredictor< Network, EdgeRndIt, ScoreRndIt, EdgeRndOutIt >, 171
~DLPredictor
LinkPred::DLPredictor< NetworkT, EdgeRndItT, ScoreRndItT, EdgeRndOutItT >, 178
~DNetwork
LinkPred::DNetwork< LabelT, NodeIDT, EdgeT >, 188
~DPATPredictor
LinkPred::DPATPredictor< Network, EdgeRndIt, ScoreRndIt, EdgeRndOutIt >, 218
~DPSTPredictor
LinkPred::DPSTPredictor< Network, EdgeRndIt, ScoreRndIt, EdgeRndOutIt >, 223
~DSAIPredictor
LinkPred::DSAIPredictor< Network, EdgeRndIt, ScoreRndIt, EdgeRndOutIt >, 228
~DSOIPredictor
LinkPred::DSOIPredictor< Network, EdgeRndIt, ScoreRndIt, EdgeRndOutIt >, 234
~DeepWalk
LinkPred::DeepWalk< Network >, 115
~Dijkstra
LinkPred::Dijkstra< Network, DistType, NbHopsType >, 149
~DotProd
LinkPred::DotProd, 214
~ESPDistCalculator
LinkPred::ESPDistCalculator< Network, DistType, NbHopsType >, 267
~ESPDsimCalculator
LinkPred::ESPDsimCalculator< Network, DsimType, NbHopsType >, 275
~ESPIndSimlCalculator
LinkPred::ESPIndSimlCalculator< Network, DistType, NbHopsType >, 282
~ESPSimlCalculator
LinkPred::ESPSimlCalculator< Network, DistType, NbHopsType >, 307
~EdgeMap
LinkPred::DNetwork< LabelT, NodeIDT, EdgeT >::EdgeMap< ValueType >, 246
LinkPred::UNetwork< LabelT, NodeIDT, EdgeT >::EdgeMap< ValueType >, 240
~Encoder
LinkPred::Encoder< Network >, 257

~Evaluator
LinkPred::Simp::Evaluator, 311
~FastSig
LinkPred::FastSig< T >, 322
~GCurve
LinkPred::GCurve< PredResultsT >, 328
~GFMatrix
LinkPred::GFMatrix, 334
~GraphTraversal
LinkPred::GraphTraversal< Network, NodeProcessor >, 349
~HMSM
LinkPred::HMSM< Network >, 353
~L1Sim
LinkPred::L1Sim, 363
~L2Sim
LinkPred::L2Sim, 366
~LINE
LinkPred::LINE< Network >, 379
~LMapQueue
LinkPred::LMapQueue< K, P, KComparator, PComparator >, 386
~LPSim
LinkPred::LPSim, 414
~LargeVis
LinkPred::LargeVis< Network >, 370
~Log
LinkPred::Log, 394
~LogMDSCG
LinkPred::LogMDSCG, 403
~LogRegCG
LinkPred::LogRegCG< InRndIt, OutRndIt >, 409
~LogisticRegresser
LinkPred::LogisticRegresser< InRndIt, OutRndIt, ScoreRndIt >, 398
~MDS
LinkPred::MDS, 429
~MatFact
LinkPred::MatFact< Network >, 419
~MatFactCG
LinkPred::MatFactCG, 425
~NetDistCalculator
LinkPred::NetDistCalculator< Network, DistType, NbHopsType >, 435
~NetIndSimlCalculator
LinkPred::NetIndSimlCalculator< Network, DistType, NbHopsType >, 440
~NetSimlCalculator
LinkPred::NetSimlCalculator< Network, DistType, NbHopsType >, 446
~NetworkManipulator
LinkPred::NetworkManipulator< Network >, 453
~Node2Vec
LinkPred::Node2Vec< Network >, 467
~NodeMap
LinkPred::DNetwork< LabelT, NodeIDT, EdgeT >::NodeMap< ValueType >, 501
LinkPred::UNetwork< LabelT, NodeIDT, EdgeT >::NodeMap< ValueType >, 496
~NodeSMap
LinkPred::DNetwork< LabelT, NodeIDT, EdgeT >::NodeSMap< ValueType >, 507
LinkPred::UNetwork< LabelT, NodeIDT, EdgeT >::NodeSMap< ValueType >, 513
~PEFactory
LinkPred::PEFactory< Network, LPredictorT, TestDataT, PerfMeasureT >, 540
~PR
LinkPred::PR< PredResultsT >, 572
~Pearson
LinkPred::Pearson, 538
~PerfCurve
LinkPred::PerfCurve< PredResultsT >, 544
~PerfEvalExp
LinkPred::PerfEvalExp< Network, TestDataT, LPredictorT, PredResultsT, PerfMeasureT >, 551
~PerfEvaluator
LinkPred::PerfEvaluator< TestDataT, LPredictorT, PredResultsT, PerfMeasureT >, 557
~PerfMeasure
LinkPred::PerfMeasure< PredResultsT >, 564
~PredResults
LinkPred::PredResults< TestDataT, LPredictorT, ScoresContainerT >, 616
~Predictor
LinkPred::Simp::Predictor, 581
~ROC
LinkPred::ROC< PredResultsT >, 670
~RandomGen
LinkPred::RandomGen, 628
~RndClassifier
LinkPred::RndClassifier< InRndIt, OutRndIt, ScoreRndIt >, 635
~SimMeasure
LinkPred::SimMeasure, 691
~TPR
LinkPred::TPR< PredResultsT >, 741
~TestData
LinkPred::TestData< Network, EdgeContT >, 697
~TestEdgeGen
LinkPred::TestEdgeGen< Network, EdgeContT >, 706
~UADAPredictor
LinkPred::UADAPredictor< Network, EdgeRndIt, ScoreRndIt, EdgeRndOutIt >, 748
~UCNEPredictor
LinkPred::UCNEPredictor< Network, EdgeRndIt, ScoreRndIt, EdgeRndOutIt >, 753
~UCRAPredictor
LinkPred::UCRAPredictor< Network, EdgeRndIt, ScoreRndIt, EdgeRndOutIt >, 759
~UCSTPredictor
LinkPred::UCSTPredictor< Network, EdgeRndIt, ScoreRndIt, EdgeRndOutIt >, 765

~UECLPredictor
LinkPred::UECLPredictor< Network, EdgeRndIt, ScoreRndIt, EdgeRndOutIt >, 771
~UESMPredictor
LinkPred::UESMPredictor< Network, EdgeRndIt, ScoreRndIt, EdgeRndOutIt >, 778
~UFBMPredictor
LinkPred::UFBMPredictor< Network, EdgeRndIt, ScoreRndIt, EdgeRndOutIt >, 783
~UHDIPredictor
LinkPred::UHDIPredictor< Network, EdgeRndIt, ScoreRndIt, EdgeRndOutIt >, 789
~UHPIPredictor
LinkPred::UHPIPredictor< Network, EdgeRndIt, ScoreRndIt, EdgeRndOutIt >, 794
~UHRGPredictor
LinkPred::UHRGPredictor< Network, EdgeRndIt, ScoreRndIt, EdgeRndOutIt >, 799
~UHYPPredictor
LinkPred::UHYPPredictor< Network, EdgeRndIt, ScoreRndIt, EdgeRndOutIt >, 805
~UJIDPredictor
LinkPred::UJIDPredictor< Network, EdgeRndIt, ScoreRndIt, EdgeRndOutIt >, 814
~UKABPredictor
LinkPred::UKABPredictor< Network, EdgeRndIt, ScoreRndIt, EdgeRndOutIt >, 820
~ULCPPredictor
LinkPred::ULCPPredictor< Network, EdgeRndIt, ScoreRndIt, EdgeRndOutIt >, 826
~ULHNPredictor
LinkPred::ULHNPredictor< Network, EdgeRndIt, ScoreRndIt, EdgeRndOutIt >, 832
~ULPredictor
LinkPred::ULPredictor< NetworkT, EdgeRndItT, ScoreRndItT, EdgeRndOutItT >, 838
~UMPSPredictor
LinkPred::UMPSPredictor< Network, EdgeRndIt, ScoreRndIt, EdgeRndOutIt >, 848
~UNEDPredictor
LinkPred::UNEDPredictor< Network, EdgeRndIt, ScoreRndIt, EdgeRndOutIt >, 864
~UNetwork
LinkPred::UNetwork< LabelT, NodeIDT, EdgeT >, 872
~UPATPredictor
LinkPred::UPATPredictor< Network, EdgeRndIt, ScoreRndIt, EdgeRndOutIt >, 897
~UPSTPredictor
LinkPred::UPSTPredictor< Network, EdgeRndIt, ScoreRndIt, EdgeRndOutIt >, 903
~URALPredictor
LinkPred::URALPredictor< Network, EdgeRndIt, ScoreRndIt, EdgeRndOutIt >, 908
~URNDPredictor
LinkPred::URNDPredictor< Network, EdgeRndIt, ScoreRndIt, EdgeRndOutIt >, 913
~USAIPredictor
LinkPred::USAIPredictor< Network, EdgeRndIt, ScoreRndIt, EdgeRndOutIt >, 919
~USBMPredictor
LinkPred::USBMPredictor< Network, EdgeRndIt, ScoreRndIt, EdgeRndOutIt >, 924
~USHPPredictor
LinkPred::USHPPredictor< Network, EdgeRndIt, ScoreRndIt, EdgeRndOutIt >, 930
~USOIPredictor
LinkPred::USOIPredictor< Network, EdgeRndIt, ScoreRndIt, EdgeRndOutIt >, 938
~USUMPredictor
LinkPred::USUMPredictor< Network, EdgeRndIt, ScoreRndIt, EdgeRndOutIt >, 943
~Vec
LinkPred::Vec, 950

acc
perfmeasure.hpp, 1051
addADA
LinkPred::Simp::Evaluator, 311
addCNE
LinkPred::Simp::Evaluator, 312
addCRA
LinkPred::Simp::Evaluator, 312
addECL
LinkPred::Simp::Evaluator, 312
addEdge
LinkPred::DNetwork< LabelT, NodeIDT, EdgeT >, 188
LinkPred::UNetwork< LabelT, NodeIDT, EdgeT >, 872
addESM
LinkPred::Simp::Evaluator, 313
addFBM
LinkPred::Simp::Evaluator, 313
addHDI
LinkPred::Simp::Evaluator, 314
addHPI
LinkPred::Simp::Evaluator, 314
addHRG
LinkPred::Simp::Evaluator, 314
addHYP
LinkPred::Simp::Evaluator, 315
addJID
LinkPred::Simp::Evaluator, 315
addKAB
LinkPred::Simp::Evaluator, 315
addLCP
LinkPred::Simp::Evaluator, 316
addLHN
LinkPred::Simp::Evaluator, 316
addNode
LinkPred::DNetwork< LabelT, NodeIDT, EdgeT >, 189
LinkPred::UNetwork< LabelT, NodeIDT, EdgeT >, 873
addPAT
LinkPred::Simp::Evaluator, 316

addPerfMeasure
LinkPred::PerfEvaluator< TestDataT, LPredictorT, PredResultsT, PerfMeasureT >, 557
addPR
LinkPred::Simp::Evaluator, 316
addPredictor
LinkPred::PerfEvaluator< TestDataT, LPredictorT, PredResultsT, PerfMeasureT >, 557
addPST
LinkPred::Simp::Evaluator, 317
addRAL
LinkPred::Simp::Evaluator, 317
addRND
LinkPred::Simp::Evaluator, 317
addROC
LinkPred::Simp::Evaluator, 318
addSAI
LinkPred::Simp::Evaluator, 318
addSBM
LinkPred::Simp::Evaluator, 318
addSHP
LinkPred::Simp::Evaluator, 318
addSOI
LinkPred::Simp::Evaluator, 319
addTPR
LinkPred::Simp::Evaluator, 319
amax
LinkPred::Vec, 950
amin
LinkPred::Vec, 951
ASPDistCalculator
LinkPred::ASPDistCalculator< Network, DistType, NbHopsType >, 62, 63
ASPDsimCalculator
LinkPred::ASPDsimCalculator< Network, DsimType, NbHopsType >, 69, 70
assemble
LinkPred::DNetwork< LabelT, NodeIDT, EdgeT >, 189
LinkPred::UNetwork< LabelT, NodeIDT, EdgeT >, 873
assertNoNaN
LinkPred::Utils, 48
at
LinkPred::DNetwork< LabelT, NodeIDT, EdgeT >::EdgeMap< ValueType >, 247
LinkPred::DNetwork< LabelT, NodeIDT, EdgeT >::NodeMap< ValueType >, 502
LinkPred::DNetwork< LabelT, NodeIDT, EdgeT >::NodeSMap< ValueType >, 507
LinkPred::LMapQueue< K, P, KComparator, PComparator >, 387
LinkPred::UNetwork< LabelT, NodeIDT, EdgeT >::EdgeMap< ValueType >, 240
LinkPred::UNetwork< LabelT, NodeIDT, EdgeT >::NodeMap< ValueType >, 496
LinkPred::UNetwork< LabelT, NodeIDT, EdgeT >::NodeSMap< ValueType >, 513
axpy
LinkPred::Vec, 951

begin
LinkPred::DNetwork< LabelT, NodeIDT, EdgeT >::EdgeMap< ValueType >, 247
LinkPred::DNetwork< LabelT, NodeIDT, EdgeT >::NodeMap< ValueType >, 502
LinkPred::DNetwork< LabelT, NodeIDT, EdgeT >::NodeSMap< ValueType >, 507
LinkPred::LMapQueue< K, P, KComparator, PComparator >, 387, 388
LinkPred::UNetwork< LabelT, NodeIDT, EdgeT >::EdgeMap< ValueType >, 241
LinkPred::UNetwork< LabelT, NodeIDT, EdgeT >::NodeMap< ValueType >, 497
LinkPred::UNetwork< LabelT, NodeIDT, EdgeT >::NodeSMap< ValueType >, 513
BFS
LinkPred::BFS< Network, NodeProcessor >, 74
BHeap
LinkPred::BHeap< T, P, ComparatorT, ComparatorP >, 78
Bhmap
LinkPred::Bhmap< K, P, Comparator >, 84, 85
bindings/Java/include/LinkPredJavaConfig.hpp, 971
bindings/Python/include/LinkPredPythonConfig.hpp, 971

CacheLevel
LinkPred, 39
cbegin
LinkPred::DNetwork< LabelT, NodeIDT, EdgeT >::EdgeMap< ValueType >, 247
LinkPred::DNetwork< LabelT, NodeIDT, EdgeT >::NodeMap< ValueType >, 502
LinkPred::DNetwork< LabelT, NodeIDT, EdgeT >::NodeSMap< ValueType >, 508
LinkPred::LMapQueue< K, P, KComparator, PComparator >, 388
LinkPred::UNetwork< LabelT, NodeIDT, EdgeT >::EdgeMap< ValueType >, 241
LinkPred::UNetwork< LabelT, NodeIDT, EdgeT >::NodeMap< ValueType >, 497
LinkPred::UNetwork< LabelT, NodeIDT, EdgeT >::NodeSMap< ValueType >, 513
cend
LinkPred::DNetwork< LabelT, NodeIDT, EdgeT >::EdgeMap< ValueType >, 247
LinkPred::DNetwork< LabelT, NodeIDT, EdgeT >::NodeMap< ValueType >, 502
LinkPred::DNetwork< LabelT, NodeIDT, EdgeT >::NodeSMap< ValueType >, 508
LinkPred::LMapQueue< K, P, KComparator, PComparator >, 388
LinkPred::UNetwork< LabelT, NodeIDT, EdgeT >::EdgeMap< ValueType >, 241
LinkPred::UNetwork< LabelT, NodeIDT, EdgeT >::NodeMap< ValueType >, 497

LinkPred::UNetwork< LabelT, NodeIDT, EdgeT >::NodeSMap< ValueType >, 514
center
LinkPred::Vec, 952
CGMDS
LinkPred, 40
cgMDS
LinkPred::MDS, 430
Classifier
LinkPred::Classifier< InRndIt, OutRndIt, ScoreRndIt >, 89, 90
classifierName
LinkPred::Simp::Evaluator::Factory::ECLParams, 237
clear
LinkPred::LMapQueue< K, P, KComparator, PComparator >, 388
LinkPred::Utils, 49
CodeMap
LinkPred::Encoder< Network >, 255
CodeMapSP
LinkPred::Encoder< Network >, 255
Collector
LinkPred::Collector< Network >, 93, 94
compareEdgeEnd
LinkPred::DNetwork< LabelT, NodeIDT, EdgeT >, 189
LinkPred::UNetwork< LabelT, NodeIDT, EdgeT >, 873
compareTop
LinkPred::LMapQueue< K, P, KComparator, PComparator >, 388
compNegScores
LinkPred::PredResults< TestDataT, LPredictorT, ScoresContainerT >, 617
compPosScores
LinkPred::PredResults< TestDataT, LPredictorT, ScoresContainerT >, 617
compTopScores
LinkPred::PredResults< TestDataT, LPredictorT, ScoresContainerT >, 617
contains
LinkPred::BHeap< T, P, ComparatorT, ComparatorP >, 79
Core classes, 23
CosineSim
LinkPred::CosineSim, 96, 97
count
LinkPred::LMapQueue< K, P, KComparator, PComparator >, 389
Counter
LinkPred::Counter< Network >, 99, 100
coupleAtOrd
LinkPred::DNetwork< LabelT, NodeIDT, EdgeT >, 190
LinkPred::UNetwork< LabelT, NodeIDT, EdgeT >, 874
coupleOrd
LinkPred::DNetwork< LabelT, NodeIDT, EdgeT >, 190
LinkPred::UNetwork< LabelT, NodeIDT, EdgeT >, 874
createEdgeMap
LinkPred::DNetwork< LabelT, NodeIDT, EdgeT >, 190
LinkPred::UNetwork< LabelT, NodeIDT, EdgeT >, 874
createEdgeMapSP
LinkPred::DNetwork< LabelT, NodeIDT, EdgeT >, 191
LinkPred::UNetwork< LabelT, NodeIDT, EdgeT >, 875
createNodeMap
LinkPred::DNetwork< LabelT, NodeIDT, EdgeT >, 191
LinkPred::UNetwork< LabelT, NodeIDT, EdgeT >, 875
createNodeMapSP
LinkPred::DNetwork< LabelT, NodeIDT, EdgeT >, 191
LinkPred::UNetwork< LabelT, NodeIDT, EdgeT >, 875
createNodeSMap
LinkPred::DNetwork< LabelT, NodeIDT, EdgeT >, 192
LinkPred::UNetwork< LabelT, NodeIDT, EdgeT >, 876
createNodeSMapSP
LinkPred::DNetwork< LabelT, NodeIDT, EdgeT >, 192
LinkPred::UNetwork< LabelT, NodeIDT, EdgeT >, 876
createTestData
LinkPred::NetworkManipulator< Network >, 453, 454
createTestDataAdd
LinkPred::NetworkManipulator< Network >, 455
createTestDataRem
LinkPred::NetworkManipulator< Network >, 456
createTestDataSeq
LinkPred::NetworkManipulator< Network >, 457
createTestDataSeqInter
LinkPred::NetworkManipulator< Network >, 458

DADAPredictor
LinkPred::DADAPredictor< Network, EdgeRndIt, ScoreRndIt, EdgeRndOutIt >, 103, 104
DCNEPredictor
LinkPred::DCNEPredictor< Network, EdgeRndIt, ScoreRndIt, EdgeRndOutIt >, 109
Dec
LinkPred, 40
decrease
LinkPred::BHeap< T, P, ComparatorT, ComparatorP >, 79
DeepWalk
LinkPred::DeepWalk< Network >, 114, 115

DefaultLogLevel
LinkPred::Log, 395
DefaultOutputStream
LinkPred::Log, 395
DESPLDistCalculator
LinkPred::DESPLDistCalculator< Network, DistType, NbHopsType >, 125, 126
DFS
LinkPred::DFS< Network, NodeProcessor >, 132
DGI
LinkPred::PR< PredResultsT >, 571
DHDIPredictor
LinkPred::DHDIPredictor< Network, EdgeRndIt, ScoreRndIt, EdgeRndOutIt >, 135, 136
DHPIPredictor
LinkPred::DHPIPredictor< Network, EdgeRndIt, ScoreRndIt, EdgeRndOutIt >, 141
diag
LinkPred::GFMatrix, 334
difference_type
LinkPred::DNetwork< LabelT, NodeIDT, EdgeT >::NodeDegIt, 475
LinkPred::DNetwork< LabelT, NodeIDT, EdgeT >::NonEdgeIt, 528
LinkPred::PredResults< TestDataT, LPredictorT, ScoresContainerT >::ScoreIterator, 679
LinkPred::TestData< Network, EdgeContT >::TestEdgeIt, 711
LinkPred::TestEdgeGen< Network, EdgeContT >::TNEdgeIt, 721
LinkPred::TestEdgeGen< Network, EdgeContT >::TPEdgeIt, 730
LinkPred::UNetwork< LabelT, NodeIDT, EdgeT >::NodeDegIt, 485
LinkPred::UNetwork< LabelT, NodeIDT, EdgeT >::NonEdgeIt, 518
Dijkstra
LinkPred::Dijkstra< Network, DistType, NbHopsType >, 148, 149
dim
LinkPred::Simp::Evaluator::Factory::ECLParams, 237
LinkPred::Simp::Evaluator::Factory::ESMParams, 261
DJIDPredictor
LinkPred::DJIDPredictor< Network, EdgeRndIt, ScoreRndIt, EdgeRndOutIt >, 160
DLCPPredictor
LinkPred::DLCPPredictor< Network, EdgeRndIt, ScoreRndIt, EdgeRndOutIt >, 165, 166
DLHNPredictor
LinkPred::DLHNPredictor< Network, EdgeRndIt, ScoreRndIt, EdgeRndOutIt >, 170, 171
DLPredictor
LinkPred::DLPredictor< NetworkT, EdgeRndItT, ScoreRndItT, EdgeRndOutItT >, 177
DNetwork
LinkPred::DNetwork< LabelT, NodeIDT, EdgeT >, 187, 188
LinkPred::DNetwork< LabelT, NodeIDT, EdgeT >::NodeDegIt, 483
LinkPred::DNetwork< LabelT, NodeIDT, EdgeT >::NonEdgeIt, 535
DotProd
LinkPred::DotProd, 213, 214
DPATPredictor
LinkPred::DPATPredictor< Network, EdgeRndIt, ScoreRndIt, EdgeRndOutIt >, 217
DPSTPredictor
LinkPred::DPSTPredictor< Network, EdgeRndIt, ScoreRndIt, EdgeRndOutIt >, 222, 223
DSAIPredictor
LinkPred::DSAIPredictor< Network, EdgeRndIt, ScoreRndIt, EdgeRndOutIt >, 227, 228
DSOIPredictor
LinkPred::DSOIPredictor< Network, EdgeRndIt, ScoreRndIt, EdgeRndOutIt >, 233

ECEIT
LinkPred::TestData< Network, EdgeContT >, 696
Edge
LinkPred::ASPDistCalculator< Network, DistType, NbHopsType >, 61
LinkPred::ASPDsimCalculator< Network, DsimType, NbHopsType >, 67
LinkPred::DESPLDistCalculator< Network, DistType, NbHopsType >, 123
LinkPred::Dijkstra< Network, DistType, NbHopsType >, 146
LinkPred::DLPredictor< NetworkT, EdgeRndItT, ScoreRndItT, EdgeRndOutItT >, 175
LinkPred::DNetwork< LabelT, NodeIDT, EdgeT >, 185
LinkPred::Encoder< Network >, 255
LinkPred::ESPDistCalculator< Network, DistType, NbHopsType >, 264
LinkPred::ESPDsimCalculator< Network, DsimType, NbHopsType >, 272
LinkPred::ESPIndSimlCalculator< Network, DistType, NbHopsType >, 279
LinkPred::ESPLDistCalculator< Network, DistType, NbHopsType >, 285
LinkPred::ESPLSimlCalculator< Network, DistType, NbHopsType >, 295
LinkPred::ESPSimlCalculator< Network, DistType, NbHopsType >, 304
LinkPred::NetDistCalculator< Network, DistType, NbHopsType >, 433
LinkPred::NetIndSimlCalculator< Network, DistType, NbHopsType >, 439
LinkPred::NetSimlCalculator< Network, DistType, NbHopsType >, 444
LinkPred::NetworkManipulator< Network >, 452
LinkPred::TestData< Network, EdgeContT >, 694
LinkPred::UKABPredictor< Network, EdgeRndIt, ScoreRndIt, EdgeRndOutIt >, 818

LinkPred::ULPredictor< NetworkT, EdgeRndItT, ScoreRndItT, EdgeRndOutItT >, 836
LinkPred::UNEDPredictor< Network, EdgeRndIt, ScoreRndIt, EdgeRndOutIt >, 862
LinkPred::UNetwork< LabelT, NodeIDT, EdgeT >, 870
EdgeIt
LinkPred::DNetwork< LabelT, NodeIDT, EdgeT >, 185
LinkPred::TestData< Network, EdgeContT >, 695
LinkPred::UNetwork< LabelT, NodeIDT, EdgeT >, 870
EdgeLengthMap
LinkPred::ASPDistCalculator< Network, DistType, NbHopsType >, 61
LinkPred::ASPDsimCalculator< Network, DsimType, NbHopsType >, 68
LinkPred::DESPLDistCalculator< Network, DistType, NbHopsType >, 123
LinkPred::Dijkstra< Network, DistType, NbHopsType >, 146
LinkPred::ESPDistCalculator< Network, DistType, NbHopsType >, 264
LinkPred::ESPDsimCalculator< Network, DsimType, NbHopsType >, 272
LinkPred::ESPIndSimlCalculator< Network, DistType, NbHopsType >, 279
LinkPred::ESPLDistCalculator< Network, DistType, NbHopsType >, 286
LinkPred::ESPLSimlCalculator< Network, DistType, NbHopsType >, 295
LinkPred::ESPSimlCalculator< Network, DistType, NbHopsType >, 304
LinkPred::NetDistCalculator< Network, DistType, NbHopsType >, 434
LinkPred::NetIndSimlCalculator< Network, DistType, NbHopsType >, 439
LinkPred::NetSimlCalculator< Network, DistType, NbHopsType >, 445
EdgeLengthMapSP
LinkPred::ASPDistCalculator< Network, DistType, NbHopsType >, 61
LinkPred::ASPDsimCalculator< Network, DsimType, NbHopsType >, 68
LinkPred::DESPLDistCalculator< Network, DistType, NbHopsType >, 124
LinkPred::Dijkstra< Network, DistType, NbHopsType >, 147
LinkPred::ESPDistCalculator< Network, DistType, NbHopsType >, 264
LinkPred::ESPDsimCalculator< Network, DsimType, NbHopsType >, 272
LinkPred::ESPIndSimlCalculator< Network, DistType, NbHopsType >, 279
LinkPred::ESPLDistCalculator< Network, DistType, NbHopsType >, 286
LinkPred::ESPLSimlCalculator< Network, DistType, NbHopsType >, 295
LinkPred::ESPSimlCalculator< Network, DistType, NbHopsType >, 304
LinkPred::NetDistCalculator< Network, DistType, NbHopsType >, 434
LinkPred::NetIndSimlCalculator< Network, DistType, NbHopsType >, 439
LinkPred::NetSimlCalculator< Network, DistType, NbHopsType >, 445
EdgeLengthMethod
LinkPred::UMPSPredictor< Network, EdgeRndIt, ScoreRndIt, EdgeRndOutIt >, 846
EdgeMap
LinkPred::DNetwork< LabelT, NodeIDT, EdgeT >::EdgeMap< ValueType >, 246
LinkPred::NetworkManipulator< Network >, 452
LinkPred::UNetwork< LabelT, NodeIDT, EdgeT >::EdgeMap< ValueType >, 239, 240
EdgeMapConstIt
LinkPred::DNetwork< LabelT, NodeIDT, EdgeT >::EdgeMap< ValueType >, 245
LinkPred::UNetwork< LabelT, NodeIDT, EdgeT >::EdgeMap< ValueType >, 239
EdgeMapIt
LinkPred::DNetwork< LabelT, NodeIDT, EdgeT >::EdgeMap< ValueType >, 245
LinkPred::UNetwork< LabelT, NodeIDT, EdgeT >::EdgeMap< ValueType >, 239
EdgeMapSP
LinkPred::DNetwork< LabelT, NodeIDT, EdgeT >, 186
LinkPred::UNetwork< LabelT, NodeIDT, EdgeT >, 870
EdgeRndIt
LinkPred::DLPredictor< NetworkT, EdgeRndItT, ScoreRndItT, EdgeRndOutItT >, 175
LinkPred::ULPredictor< NetworkT, EdgeRndItT, ScoreRndItT, EdgeRndOutItT >, 836
EdgeRndOutIt
LinkPred::DLPredictor< NetworkT, EdgeRndItT, ScoreRndItT, EdgeRndOutItT >, 176
LinkPred::ULPredictor< NetworkT, EdgeRndItT, ScoreRndItT, EdgeRndOutItT >, 836
edgesBegin
LinkPred::DNetwork< LabelT, NodeIDT, EdgeT >, 192
LinkPred::UNetwork< LabelT, NodeIDT, EdgeT >, 877
edgesEnd
LinkPred::DNetwork< LabelT, NodeIDT, EdgeT >, 193
LinkPred::UNetwork< LabelT, NodeIDT, EdgeT >, 877
elemMult
LinkPred::GFMatrix, 335
empty
LinkPred::LMapQueue< K, P, KComparator, PComparator >, 389
encode

LinkPred::DeepWalk< Network >, 115
LinkPred::Encoder< Network >, 257
LinkPred::HMSM< Network >, 353
LinkPred::LargeVis< Network >, 370
LinkPred::LINE< Network >, 379
LinkPred::MatFact< Network >, 419
LinkPred::Node2Vec< Network >, 467
Encoder
LinkPred::Encoder< Network >, 256
encoderName
LinkPred::Simp::Evaluator::Factory::ECLParams, 237
LinkPred::Simp::Evaluator::Factory::ESMParams, 261
end
LinkPred::DNetwork< LabelT, NodeIDT, EdgeT >, 193
LinkPred::DNetwork< LabelT, NodeIDT, EdgeT >::EdgeMap< ValueType >, 248
LinkPred::DNetwork< LabelT, NodeIDT, EdgeT >::NodeMap< ValueType >, 503
LinkPred::DNetwork< LabelT, NodeIDT, EdgeT >::NodeSMap< ValueType >, 508
LinkPred::LMapQueue< K, P, KComparator, PComparator >, 389
LinkPred::UNetwork< LabelT, NodeIDT, EdgeT >, 877
LinkPred::UNetwork< LabelT, NodeIDT, EdgeT >::EdgeMap< ValueType >, 241
LinkPred::UNetwork< LabelT, NodeIDT, EdgeT >::NodeMap< ValueType >, 497
LinkPred::UNetwork< LabelT, NodeIDT, EdgeT >::NodeSMap< ValueType >, 514
epsilon
LinkPred::Simp::Evaluator::Factory::LCPParams, 376
ESPDistCalculator
LinkPred::ESPDistCalculator< Network, DistType, NbHopsType >, 265, 267
ESPDsimCalculator
LinkPred::ESPDsimCalculator< Network, DsimType, NbHopsType >, 273, 275
ESPIndSimlCalculator
LinkPred::ESPIndSimlCalculator< Network, DistType, NbHopsType >, 281
ESPLDistCalculator
LinkPred::ESPLDistCalculator< Network, DistType, NbHopsType >, 287, 288
ESPLSimlCalculator
LinkPred::ESPLSimlCalculator< Network, DistType, NbHopsType >, 296, 297
ESPSimlCalculator
LinkPred::ESPSimlCalculator< Network, DistType, NbHopsType >, 306
eval
LinkPred::GCurve< PredResultsT >, 328, 329
LinkPred::PerfCurve< PredResultsT >, 544, 545
LinkPred::PerfEvaluator< TestDataT, LPredictorT, PredResultsT, PerfMeasureT >, 559
LinkPred::PerfMeasure< PredResultsT >, 565
LinkPred::PR< PredResultsT >, 572, 573
LinkPred::ROC< PredResultsT >, 670, 671
LinkPred::TPR< PredResultsT >, 741, 742
evalNoStream
LinkPred::ROC< PredResultsT >, 671
evalNoTiming
LinkPred::PerfEvaluator< TestDataT, LPredictorT, PredResultsT, PerfMeasureT >, 559
evalStream
LinkPred::ROC< PredResultsT >, 672
evalTiming
LinkPred::PerfEvaluator< TestDataT, LPredictorT, PredResultsT, PerfMeasureT >, 559
Evaluator
LinkPred::Simp::Evaluator, 311
evalUsingPredict
LinkPred::TPR< PredResultsT >, 742
evalUsingTop
LinkPred::TPR< PredResultsT >, 743

f
LinkPred::LogMDSCG, 404
LinkPred::LogRegCG< InRndIt, OutRndIt >, 409
LinkPred::MatFactCG, 425
FastSig
LinkPred::FastSig< T >, 321, 322
fdr
perfmeasure.hpp, 1052
fgrad
LinkPred::LogMDSCG, 404
LinkPred::LogRegCG< InRndIt, OutRndIt >, 409
LinkPred::MatFactCG, 425
fileName
LinkPred::Simp::Evaluator::Factory::PSTParams, 626
filter
LinkPred::Utils, 49
finalize
LinkPred::LogMDSCG, 404
LinkPred::LogRegCG< InRndIt, OutRndIt >, 410
LinkPred::MatFactCG, 426
find
LinkPred::DNetwork< LabelT, NodeIDT, EdgeT >::EdgeMap< ValueType >, 248
LinkPred::DNetwork< LabelT, NodeIDT, EdgeT >::NodeSMap< ValueType >, 508
LinkPred::LMapQueue< K, P, KComparator, PComparator >, 390
LinkPred::UNetwork< LabelT, NodeIDT, EdgeT >::EdgeMap< ValueType >, 242
LinkPred::UNetwork< LabelT, NodeIDT, EdgeT >::NodeSMap< ValueType >, 514
LinkPred::Vec, 952
findLabel
LinkPred::DNetwork< LabelT, NodeIDT, EdgeT >, 193

LinkPred::UNetwork< LabelT, NodeIDT, EdgeT >, 877
findNode
LinkPred::DNetwork< LabelT, NodeIDT, EdgeT >, 194
LinkPred::UNetwork< LabelT, NodeIDT, EdgeT >, 878
findNz
LinkPred::Vec, 952
flip
LinkPred::Utils, 49
flipMap
LinkPred::Utils, 50
fmr
perfmeasure.hpp, 1052
FN
LinkPred, 39
fnr
perfmeasure.hpp, 1053
fnRatio
LinkPred::PerfeEvalExpDescp< Network >, 548
FP
LinkPred, 39
fpr
perfmeasure.hpp, 1053

gamma
LinkPred::Simp::Evaluator::Factory::HYPParams, 359
GCurve
LinkPred::GCurve< PredResultsT >, 327, 328
generateERN
LinkPred::UNetwork< LabelT, NodeIDT, EdgeT >, 878
generateREG
LinkPred::UNetwork< LabelT, NodeIDT, EdgeT >, 879
generateRNC
LinkPred::UNetwork< LabelT, NodeIDT, EdgeT >, 879
generateTN
LinkPred::TestEdgeGen< Network, EdgeContT >, 706
generateTP
LinkPred::TestEdgeGen< Network, EdgeContT >, 707
genNeg
LinkPred::TestData< Network, EdgeContT >, 698
genPos
LinkPred::TestData< Network, EdgeContT >, 698
genTestData
LinkPred::Simp::Evaluator, 319
get
LinkPred::GFMatrix, 335
getAlg
LinkPred::MDS, 430
getAllNegLinksExcept
LinkPred::NetworkManipulator< Network >, 459
getAlpha
LinkPred::DeepWalk< Network >, 116
LinkPred::HMSM< Network >, 354
getAsp
LinkPred::UMPSPredictor< Network, EdgeRndIt, ScoreRndIt, EdgeRndOutIt >, 848
LinkPred::USHPPredictor< Network, EdgeRndIt, ScoreRndIt, EdgeRndOutIt >, 930
getAvgDeg
LinkPred::DNetwork< LabelT, NodeIDT, EdgeT >, 194
LinkPred::UNetwork< LabelT, NodeIDT, EdgeT >, 879
getAvgInDeg
LinkPred::DNetwork< LabelT, NodeIDT, EdgeT >, 194
getAvgOutDeg
LinkPred::DNetwork< LabelT, NodeIDT, EdgeT >, 194
getBool
LinkPred::RandomGen, 628
getCacheHits
LinkPred::ESPDistCalculator< Network, DistType, NbHopsType >, 268
getCacheLevel
LinkPred::UKABPredictor< Network, EdgeRndIt, ScoreRndIt, EdgeRndOutIt >, 820
LinkPred::UMPSPredictor< Network, EdgeRndIt, ScoreRndIt, EdgeRndOutIt >, 848
LinkPred::USHPPredictor< Network, EdgeRndIt, ScoreRndIt, EdgeRndOutIt >, 931
getCacheMiss
LinkPred::ESPDistCalculator< Network, DistType, NbHopsType >, 268
getCC
LinkPred::UNetwork< LabelT, NodeIDT, EdgeT >, 880
getClassifier
LinkPred::UECLPredictor< Network, EdgeRndIt, ScoreRndIt, EdgeRndOutIt >, 771
getCol
LinkPred::GFMatrix, 335
getCols
LinkPred::GFMatrix, 336
getCommonNeighbors
LinkPred::UNetwork< LabelT, NodeIDT, EdgeT >, 880, 881
getCount
LinkPred::Counter< Network >, 100
getCSR
LinkPred::UNetwork< LabelT, NodeIDT, EdgeT >, 881
getCurve
LinkPred::GCurve< PredResultsT >, 329, 330
LinkPred::PerfCurve< PredResultsT >, 545, 546
LinkPred::PR< PredResultsT >, 573, 574
LinkPred::ROC< PredResultsT >, 672
getDefaultDim
LinkPred::DeepWalk< Network >, 116

LinkPred::HMSM< Network >, 354
LinkPred::LargeVis< Network >, 370
LinkPred::LINE< Network >, 379
LinkPred::MatFact< Network >, 419
LinkPred::Node2Vec< Network >, 468

getDeg
LinkPred::DNetwork< LabelT, NodeIDT, EdgeT >, 195
LinkPred::UNetwork< LabelT, NodeIDT, EdgeT >, 881

getDegStat
LinkPred::DNetwork< LabelT, NodeIDT, EdgeT >, 195
LinkPred::UNetwork< LabelT, NodeIDT, EdgeT >, 882

getDim
LinkPred::Encoder< Network >, 257

getDirIndSiml
LinkPred::ESPIndSimlCalculator< Network, DistType, NbHopsType >, 282
LinkPred::NetIndSimlCalculator< Network, DistType, NbHopsType >, 440

getDirSiml
LinkPred::ESPLSimlCalculator< Network, DistType, NbHopsType >, 298
LinkPred::ESPSimlCalculator< Network, DistType, NbHopsType >, 307
LinkPred::NetSimlCalculator< Network, DistType, NbHopsType >, 446

getDiscDist
LinkPred::NetDistCalculator< Network, DistType, NbHopsType >, 435
LinkPred::NetIndSimlCalculator< Network, DistType, NbHopsType >, 441
LinkPred::NetSimlCalculator< Network, DistType, NbHopsType >, 447

getDiscNbHops
LinkPred::NetDistCalculator< Network, DistType, NbHopsType >, 435
LinkPred::NetIndSimlCalculator< Network, DistType, NbHopsType >, 441
LinkPred::NetSimlCalculator< Network, DistType, NbHopsType >, 447

getDist
LinkPred::ASPDistCalculator< Network, DistType, NbHopsType >, 63, 64
LinkPred::ASPDsimCalculator< Network, DsimType, NbHopsType >, 70, 71
LinkPred::DESPLDistCalculator< Network, DistType, NbHopsType >, 127
LinkPred::Dijkstra< Network, DistType, NbHopsType >, 150
LinkPred::ESPDistCalculator< Network, DistType, NbHopsType >, 268
LinkPred::ESPDsimCalculator< Network, DsimType, NbHopsType >, 276
LinkPred::ESPLDistCalculator< Network, DistType, NbHopsType >, 289
LinkPred::NetDistCalculator< Network, DistType, NbHopsType >, 436

getDistL
LinkPred::Dijkstra< Network, DistType, NbHopsType >, 151

getDouble
LinkPred::RandomGen, 628

getDsim
LinkPred::Dijkstra< Network, DistType, NbHopsType >, 151, 153

getEdgeCode
LinkPred::Encoder< Network >, 257, 258

getEdgeCodeDim
LinkPred::Encoder< Network >, 258

getEdgeLengthMethod
LinkPred::UMPSPredictor< Network, EdgeRndIt, ScoreRndIt, EdgeRndOutIt >, 848

getEg
LinkPred::TestData< Network, EdgeContT >, 698

getEncoder
LinkPred::UECLPredictor< Network, EdgeRndIt, ScoreRndIt, EdgeRndOutIt >, 771
LinkPred::UESMPredictor< Network, EdgeRndIt, ScoreRndIt, EdgeRndOutIt >, 778

getEps
LinkPred::HMSM< Network >, 354

getEpsilon
LinkPred::ULCPPredictor< Network, EdgeRndIt, ScoreRndIt, EdgeRndOutIt >, 826

getFactory
LinkPred::PerfEvalExp< Network, TestDataT, LPredictorT, PredResultsT, PerfMeasureT >, 552

getFinDistMapNoNeighb
LinkPred::DESPLDistCalculator< Network, DistType, NbHopsType >, 128
LinkPred::ESPLDistCalculator< Network, DistType, NbHopsType >, 290

getGamma
LinkPred::LargeVis< Network >, 371
LinkPred::UHYPPredictor< Network, EdgeRndIt, ScoreRndIt, EdgeRndOutIt >, 806

getGCurve
LinkPred::GCurve< PredResultsT >, 330

getGCurveAuc
LinkPred::GCurve< PredResultsT >, 330

getGeo
LinkPred::RandomGen, 629

getHorizLim
LinkPred::UKABPredictor< Network, EdgeRndIt, ScoreRndIt, EdgeRndOutIt >, 820

getHProb
LinkPred::HMSM< Network >, 354

getID
LinkPred::DNetwork< LabelT, NodeIDT, EdgeT >, 195
LinkPred::Simp::Predictor, 581
LinkPred::UNetwork< LabelT, NodeIDT, EdgeT >,

882
getIndDist
LinkPred::ASPDistCalculator< Network, DistType, NbHopsType >, 64
LinkPred::ASPDsimCalculator< Network, DsimType, NbHopsType >, 71
LinkPred::DESPLDistCalculator< Network, DistType, NbHopsType >, 128
LinkPred::Dijkstra< Network, DistType, NbHopsType >, 153
LinkPred::ESPDistCalculator< Network, DistType, NbHopsType >, 269
LinkPred::ESPDsimCalculator< Network, DsimType, NbHopsType >, 276
LinkPred::ESPLDistCalculator< Network, DistType, NbHopsType >, 290
LinkPred::NetDistCalculator< Network, DistType, NbHopsType >, 436
getIndDistToAll
LinkPred::Dijkstra< Network, DistType, NbHopsType >, 154
getIndDsim
LinkPred::Dijkstra< Network, DistType, NbHopsType >, 154
getInDeg
LinkPred::DNetwork< LabelT, NodeIDT, EdgeT >, 196
getInDegStat
LinkPred::DNetwork< LabelT, NodeIDT, EdgeT >, 196
getIndSiml
LinkPred::ESPIndSimlCalculator< Network, DistType, NbHopsType >, 282
LinkPred::NetIndSimlCalculator< Network, DistType, NbHopsType >, 441
getInitLR
LinkPred::DeepWalk< Network >, 116
LinkPred::LargeVis< Network >, 371
LinkPred::Node2Vec< Network >, 468
getInitLr
LinkPred::LINE< Network >, 380
getInOutDeg
LinkPred::DNetwork< LabelT, NodeIDT, EdgeT >, 197
getInt
LinkPred::RandomGen, 629
getInterpolMethod
LinkPred::PR< PredResultsT >, 574
getL
LinkPred::LMapQueue< K, P, KComparator, PComparator >, 390
LinkPred::UHYPPredictor< Network, EdgeRndIt, ScoreRndIt, EdgeRndOutIt >, 806
getLabel
LinkPred::DNetwork< LabelT, NodeIDT, EdgeT >, 197
LinkPred::Simp::Predictor, 581
LinkPred::UNetwork< LabelT, NodeIDT, EdgeT >, 882
getLambda
LinkPred::LogisticRegresser< InRndIt, OutRndIt, ScoreRndIt >, 398
LinkPred::LogRegCG< InRndIt, OutRndIt >, 410
LinkPred::MatFact< Network >, 419
LinkPred::NetIndSimlCalculator< Network, DistType, NbHopsType >, 442
LinkPred::NetSimlCalculator< Network, DistType, NbHopsType >, 447
LinkPred::UMPSPredictor< Network, EdgeRndIt, ScoreRndIt, EdgeRndOutIt >, 849
getLambdaMethod
LinkPred::UMPSPredictor< Network, EdgeRndIt, ScoreRndIt, EdgeRndOutIt >, 849
getLambdaNegEstRatio
LinkPred::UMPSPredictor< Network, EdgeRndIt, ScoreRndIt, EdgeRndOutIt >, 849
getLambdaPosEstRatio
LinkPred::UMPSPredictor< Network, EdgeRndIt, ScoreRndIt, EdgeRndOutIt >, 849
getLambdaStep
LinkPred::UMPSPredictor< Network, EdgeRndIt, ScoreRndIt, EdgeRndOutIt >, 850
getLandmarkRatio
LinkPred::UMPSPredictor< Network, EdgeRndIt, ScoreRndIt, EdgeRndOutIt >, 850
LinkPred::USHPPredictor< Network, EdgeRndIt, ScoreRndIt, EdgeRndOutIt >, 931
getLandmarkStrategy
LinkPred::UMPSPredictor< Network, EdgeRndIt, ScoreRndIt, EdgeRndOutIt >, 850
LinkPred::USHPPredictor< Network, EdgeRndIt, ScoreRndIt, EdgeRndOutIt >, 931
getLength
LinkPred::UMPSPredictor< Network, EdgeRndIt, ScoreRndIt, EdgeRndOutIt >, 850
getLim
LinkPred::DESPLDistCalculator< Network, DistType, NbHopsType >, 128
LinkPred::ESPLDistCalculator< Network, DistType, NbHopsType >, 291
LinkPred::ESPLSimlCalculator< Network, DistType, NbHopsType >, 298
getLProb
LinkPred::HMSM< Network >, 354
getM
LinkPred::GFMatrix, 336
LinkPred::UHYPPredictor< Network, EdgeRndIt, ScoreRndIt, EdgeRndOutIt >, 806
getMaxCodeLength
LinkPred::DeepWalk< Network >, 116
getMaxDeg
LinkPred::DNetwork< LabelT, NodeIDT, EdgeT >, 197
LinkPred::UNetwork< LabelT, NodeIDT, EdgeT >, 883
getMaxDist

LinkPred::NetDistCalculator< Network, DistType, NbHopsType >, 437
getMaxInDeg
LinkPred::DNetwork< LabelT, NodeIDT, EdgeT >, 197
getMaxIter
LinkPred::UFBMPredictor< Network, EdgeRndIt, ScoreRndIt, EdgeRndOutIt >, 784
LinkPred::USBMPredictor< Network, EdgeRndIt, ScoreRndIt, EdgeRndOutIt >, 924
getMaxLambda
LinkPred::UMPSPredictor< Network, EdgeRndIt, ScoreRndIt, EdgeRndOutIt >, 851
getMaxNbNodesInCache
LinkPred::DESPLDistCalculator< Network, DistType, NbHopsType >, 129
LinkPred::ESPLDistCalculator< Network, DistType, NbHopsType >, 291
LinkPred::ESPLSimlCalculator< Network, DistType, NbHopsType >, 299
LinkPred::ESPSimlCalculator< Network, DistType, NbHopsType >, 308
getMaxOutDeg
LinkPred::DNetwork< LabelT, NodeIDT, EdgeT >, 198
getMinDeg
LinkPred::DNetwork< LabelT, NodeIDT, EdgeT >, 198
LinkPred::UNetwork< LabelT, NodeIDT, EdgeT >, 883
getMinInDeg
LinkPred::DNetwork< LabelT, NodeIDT, EdgeT >, 198
getMinLambda
LinkPred::UMPSPredictor< Network, EdgeRndIt, ScoreRndIt, EdgeRndOutIt >, 851
getMinOutDeg
LinkPred::DNetwork< LabelT, NodeIDT, EdgeT >, 198
getN
LinkPred::GFMatrix, 336
getName
LinkPred::Classifier< InRndIt, OutRndIt, ScoreRndIt >, 90
LinkPred::DLPredictor< NetworkT, EdgeRndItT, ScoreRndItT, EdgeRndOutItT >, 178
LinkPred::Encoder< Network >, 258
LinkPred::GFMatrix, 336
LinkPred::PerfMeasure< PredResultsT >, 566
LinkPred::SimMeasure, 691
LinkPred::ULPredictor< NetworkT, EdgeRndItT, ScoreRndItT, EdgeRndOutItT >, 838
getNbBeans
LinkPred::UHRGPredictor< Network, EdgeRndIt, ScoreRndIt, EdgeRndOutIt >, 800
getNbCommonNeighbors
LinkPred::UNetwork< LabelT, NodeIDT, EdgeT >, 883
getNbCouples
LinkPred::DNetwork< LabelT, NodeIDT, EdgeT >, 199
LinkPred::UNetwork< LabelT, NodeIDT, EdgeT >, 884
getNbEdges
LinkPred::DNetwork< LabelT, NodeIDT, EdgeT >, 199
LinkPred::UNetwork< LabelT, NodeIDT, EdgeT >, 884
getNbInEdges
LinkPred::UNetwork< LabelT, NodeIDT, EdgeT >, 884
getNbMdsRuns
LinkPred::HMSM< Network >, 355
getNbNeg
LinkPred::TestData< Network, EdgeContT >, 698
getNbNegSamples
LinkPred::LargeVis< Network >, 371
LinkPred::LINE< Network >, 380
LinkPred::Node2Vec< Network >, 468
getNbNodes
LinkPred::DNetwork< LabelT, NodeIDT, EdgeT >, 199
LinkPred::Simp::Predictor, 582
LinkPred::UNetwork< LabelT, NodeIDT, EdgeT >, 884
getNbNodeWalks
LinkPred::DeepWalk< Network >, 117
getNbNonEdges
LinkPred::DNetwork< LabelT, NodeIDT, EdgeT >, 199
LinkPred::UNetwork< LabelT, NodeIDT, EdgeT >, 885
getNbPaths
LinkPred::DNetwork< LabelT, NodeIDT, EdgeT >, 200
LinkPred::UNetwork< LabelT, NodeIDT, EdgeT >, 885
getNbPerfMeasures
LinkPred::PerfEvaluator< TestDataT, LPredictorT, PredResultsT, PerfMeasureT >, 559
getNbPos
LinkPred::TestData< Network, EdgeContT >, 698
getNbPredictors
LinkPred::PerfEvaluator< TestDataT, LPredictorT, PredResultsT, PerfMeasureT >, 559
getNbRuns
LinkPred::MDS, 430
getNbSamples
LinkPred::LargeVis< Network >, 371
LinkPred::UHRGPredictor< Network, EdgeRndIt, ScoreRndIt, EdgeRndOutIt >, 800
getNbTop
LinkPred::PredResults< TestDataT, LPredictorT, ScoresContainerT >, 617
getNbWalks
LinkPred::DeepWalk< Network >, 117

LinkPred::Node2Vec< Network >, 468
getNegClass
LinkPred::TestData< Network, EdgeContT >, 699
getNegRatio
LinkPred::MatFact< Network >, 419
LinkPred::UECLPredictor< Network, EdgeRndIt, ScoreRndIt, EdgeRndOutIt >, 771
getNegSamplingPower
LinkPred::LargeVis< Network >, 371
LinkPred::LINE< Network >, 380
getNegScore
LinkPred::UMPSPredictor< Network, EdgeRndIt, ScoreRndIt, EdgeRndOutIt >, 851
getNegSize
LinkPred::LargeVis< Network >, 372
LinkPred::LINE< Network >, 380
getNegSortOrder
LinkPred::PredResults< TestDataT, LPredictorT, ScoresContainerT >, 618
getNet
LinkPred::Dijkstra< Network, DistType, NbHopsType >, 155
LinkPred::DLPredictor< NetworkT, EdgeRndItT, ScoreRndItT, EdgeRndOutItT >, 178
LinkPred::ULPredictor< NetworkT, EdgeRndItT, ScoreRndItT, EdgeRndOutItT >, 839
getNnzSimlMap
LinkPred::ESPLSimlCalculator< Network, DistType, NbHopsType >, 299
LinkPred::NetSimlCalculator< Network, DistType, NbHopsType >, 447
getNnzSimlMapNoNeighb
LinkPred::ESPLSimlCalculator< Network, DistType, NbHopsType >, 299
LinkPred::NetSimlCalculator< Network, DistType, NbHopsType >, 448
getNodeCode
LinkPred::Encoder< Network >, 258
getNodeCodeMap
LinkPred::Encoder< Network >, 259
getObsNet
LinkPred::TestData< Network, EdgeContT >, 699
getOrder
LinkPred::LINE< Network >, 381
getOutDeg
LinkPred::DNetwork< LabelT, NodeIDT, EdgeT >, 200
getOutDegStat
LinkPred::DNetwork< LabelT, NodeIDT, EdgeT >, 200
getOutPrec
LinkPred::PerfEvalExp< Network, TestDataT, LPredictorT, PredResultsT, PerfMeasureT >, 552
getP
LinkPred::LPSim, 414
LinkPred::Node2Vec< Network >, 469
getPed
LinkPred::PerfEvalExp< Network, TestDataT, LPredictorT, PredResultsT, PerfMeasureT >, 552
getPerfMeasure
LinkPred::UMPSPredictor< Network, EdgeRndIt, ScoreRndIt, EdgeRndOutIt >, 851
getPerfMeasures
LinkPred::PEFactory< Network, LPredictorT, TestDataT, PerfMeasureT >, 541
getPerfRes
LinkPred::Simp::Evaluator, 320
getPL
LinkPred::RandomGen, 629
getPosClass
LinkPred::TestData< Network, EdgeContT >, 699
getPosRatio
LinkPred::MatFact< Network >, 420
LinkPred::UECLPredictor< Network, EdgeRndIt, ScoreRndIt, EdgeRndOutIt >, 772
getPosScore
LinkPred::UMPSPredictor< Network, EdgeRndIt, ScoreRndIt, EdgeRndOutIt >, 852
getPosSortOrder
LinkPred::PredResults< TestDataT, LPredictorT, ScoresContainerT >, 618
getPRAuc
LinkPred::PR< PredResultsT >, 574
getPRAucDGI
LinkPred::PR< PredResultsT >, 575
getPRAucLIN
LinkPred::PR< PredResultsT >, 576
getPRCurve
LinkPred::PR< PredResultsT >, 576
getPredictor
LinkPred::PerfEvaluator< TestDataT, LPredictorT, PredResultsT, PerfMeasureT >, 560
LinkPred::PredResults< TestDataT, LPredictorT, ScoresContainerT >, 618
getPredictors
LinkPred::PEFactory< Network, LPredictorT, TestDataT, PerfMeasureT >, 541
getQ
LinkPred::Node2Vec< Network >, 469
getRandom
LinkPred::Utils, 50
getRecNet
LinkPred::LINE< Network >, 381
getRecWeightMap
LinkPred::LINE< Network >, 381
getRefNet
LinkPred::TestData< Network, EdgeContT >, 699
getRemLinksMap
LinkPred::TestData< Network, EdgeContT >, 700
getRndNegLinksExcept
LinkPred::NetworkManipulator< Network >, 459
getRndPerm
LinkPred::Utils, 50
getRndPosLinksExcept

LinkPred::NetworkManipulator< Network >, 459
getROCAuc
LinkPred::ROC< PredResultsT >, 673
getROCAucStrm
LinkPred::ROC< PredResultsT >, 673
getROCCurve
LinkPred::ROC< PredResultsT >, 674
getRow
LinkPred::GFMatrix, 337
getRows
LinkPred::GFMatrix, 337
getSDist
LinkPred::Dijkstra< Network, DistType, NbHopsType >, 155
getSDistMap
LinkPred::DESPLDistCalculator< Network, DistType, NbHopsType >, 129
LinkPred::ESPLDistCalculator< Network, DistType, NbHopsType >, 291
getSeed
LinkPred::RandomGen, 630
LinkPred::UHYPPredictor< Network, EdgeRndIt, ScoreRndIt, EdgeRndOutIt >, 806
getSelfSiml
LinkPred::NetIndSimlCalculator< Network, DistType, NbHopsType >, 442
LinkPred::NetSimlCalculator< Network, DistType, NbHopsType >, 448
getShortestPath
LinkPred::Dijkstra< Network, DistType, NbHopsType >, 156
getSiml
LinkPred::ESPLSimlCalculator< Network, DistType, NbHopsType >, 300
LinkPred::ESPSimlCalculator< Network, DistType, NbHopsType >, 308
LinkPred::NetSimlCalculator< Network, DistType, NbHopsType >, 448
getSimMeasure
LinkPred::UESMPredictor< Network, EdgeRndIt, ScoreRndIt, EdgeRndOutIt >, 778
getSInt
LinkPred::RandomGen, 630
getStepInterval
LinkPred::DeepWalk< Network >, 117
LinkPred::Node2Vec< Network >, 469
getSubSample
LinkPred::Node2Vec< Network >, 469
getT
LinkPred::UHYPPredictor< Network, EdgeRndIt, ScoreRndIt, EdgeRndOutIt >, 807
getTestData
LinkPred::PredResults< TestDataT, LPredictorT, ScoresContainerT >, 618
getTheta
LinkPred::LogisticRegresser< InRndIt, OutRndIt, ScoreRndIt >, 398
getThresholds
LinkPred::GCurve< PredResultsT >, 331
LinkPred::PR< PredResultsT >, 577
LinkPred::ROC< PredResultsT >, 674
getTol
LinkPred::HMSM< Network >, 355
LinkPred::LogisticRegresser< InRndIt, OutRndIt, ScoreRndIt >, 399
LinkPred::MatFact< Network >, 420
LinkPred::MDS, 431
LinkPred::UMPSPredictor< Network, EdgeRndIt, ScoreRndIt, EdgeRndOutIt >, 852
getTotalSteps
LinkPred::DeepWalk< Network >, 117
LinkPred::Node2Vec< Network >, 470
getTPR
LinkPred::TPR< PredResultsT >, 743
getUInt
LinkPred::RandomGen, 630
getValues
LinkPred::Vec, 952
getVisited
LinkPred::Collector< Network >, 94
getWalkLength
LinkPred::DeepWalk< Network >, 118
LinkPred::Node2Vec< Network >, 470
getWeightMap
LinkPred::Encoder< Network >, 259
getWindowSize
LinkPred::DeepWalk< Network >, 118
LinkPred::Node2Vec< Network >, 470
getZeta
LinkPred::UHYPPredictor< Network, EdgeRndIt, ScoreRndIt, EdgeRndOutIt >, 807
GFMatrix
LinkPred::GFMatrix, 333, 334
grad
LinkPred::LogMDSCG, 405
LinkPred::LogRegCG< InRndIt, OutRndIt >, 410
LinkPred::MatFactCG, 426
Graph algorithms., 24
GraphTraversal
LinkPred::GraphTraversal< Network, NodeProcessor >, 348, 349

HMSM
LinkPred::HMSM< Network >, 352, 353
horizLim
LinkPred::Simp::Evaluator::Factory::KABParams, 361
Hub
LinkPred::UMPSPredictor< Network, EdgeRndIt, ScoreRndIt, EdgeRndOutIt >, 847
LinkPred::USHPPredictor< Network, EdgeRndIt, ScoreRndIt, EdgeRndOutIt >, 929

i
LinkPred::MatFactPbData, 428
LinkPred::Simp::EdgeScore, 252
LinkPred::Simp::EdgeScoreByID, 253

LinkPred::Utils::EdgeScore< Label >, 251
ifmax
LinkPred::Vec, 953
ifmin
LinkPred::Vec, 953
IHub
LinkPred::UMPSPredictor< Network, EdgeRndIt, ScoreRndIt, EdgeRndOutIt >, 847
LinkPred::USHPPredictor< Network, EdgeRndIt, ScoreRndIt, EdgeRndOutIt >, 929
ilmax
LinkPred::Vec, 953
ilmin
LinkPred::Vec, 953
imax
LinkPred::Vec, 953
imin
LinkPred::Vec, 954
Inc
LinkPred, 40
include/linkpred.hpp, 972
include/linkpred/core/core.hpp, 973
include/linkpred/core/dnetwork/dnetwork.hpp, 974
include/linkpred/core/ds/bheap.hpp, 975
include/linkpred/core/ds/bhmap.hpp, 976
include/linkpred/core/ds/ds.hpp, 977
include/linkpred/core/ds/lmapqueue.hpp, 978
include/linkpred/core/unetwork/unetwork.hpp, 979
include/linkpred/graphalg/encoders/deepwalk/deepwalk.hpp, 981
include/linkpred/graphalg/encoders/encoder.hpp, 983
include/linkpred/graphalg/encoders/encoders.hpp, 984
include/linkpred/graphalg/encoders/hmsm/hmsm.hpp, 985
include/linkpred/graphalg/encoders/largevis/largevis.hpp, 987
include/linkpred/graphalg/encoders/lem/lem.hpp, 988
include/linkpred/graphalg/encoders/line/line.hpp, 989
include/linkpred/graphalg/encoders/lle/lle.hpp, 991
include/linkpred/graphalg/encoders/matfact/matfact.hpp, 992
include/linkpred/graphalg/encoders/matfact/matfactcg.hpp, 994
include/linkpred/graphalg/encoders/node2vec/node2vec.hpp, 996
include/linkpred/graphalg/graphalg.hpp, 997
include/linkpred/graphalg/shortestpaths/dijkstra.hpp, 998
include/linkpred/graphalg/shortestpaths/netdistcalculator.hpp, 999
include/linkpred/graphalg/shortestpaths/shortestpaths.hpp, 1001
include/linkpred/graphalg/traversal/graphtraversal.hpp, 1002
include/linkpred/graphalg/traversal/traversal.hpp, 1004
include/linkpred/instantiations.hpp, 1004
include/linkpred/ml/classifiers/classifier.hpp, 1005
include/linkpred/ml/classifiers/classifiers.hpp, 1007
include/linkpred/ml/classifiers/ffn/ffn.hpp, 1008
include/linkpred/ml/classifiers/linearsvm/linearsvm.hpp, 1010
include/linkpred/ml/classifiers/logistic/logisticregresser.hpp, 1012
include/linkpred/ml/classifiers/logistic/logregcg.hpp, 1015
include/linkpred/ml/classifiers/naivebayes/naivebayes.hpp, 1016
include/linkpred/ml/classifiers/rndclassifier/rndclassifier.hpp, 1017
include/linkpred/ml/ml.hpp, 1020
include/linkpred/ml/simmeasures/cosinesim.hpp, 1021
include/linkpred/ml/simmeasures/dotprod.hpp, 1023
include/linkpred/ml/simmeasures/l1sim.hpp, 1025
include/linkpred/ml/simmeasures/l2sim.hpp, 1027
include/linkpred/ml/simmeasures/lpsim.hpp, 1029
include/linkpred/ml/simmeasures/pearson.hpp, 1031
include/linkpred/ml/simmeasures/simmeasure.hpp, 1033
include/linkpred/ml/simmeasures/simmeasures.hpp, 1034
include/linkpred/numerical/linear/gfmatrix.hpp, 1035
include/linkpred/numerical/linear/vec.hpp, 1037
include/linkpred/numerical/mds/logmdscg.hpp, 1038
include/linkpred/numerical/mds/mds.hpp, 1039
include/linkpred/numerical/mf/fastsig.hpp, 1041
include/linkpred/numerical/numerical.hpp, 1043
include/linkpred/perf/networkmanipulator.hpp, 1043
include/linkpred/perf/perf.hpp, 1046
include/linkpred/perf/perfevaluator.hpp, 1047
include/linkpred/perf/perfmeasure.hpp, 1049
include/linkpred/perf/predresults.hpp, 1055
include/linkpred/predictors/directed/dadapredictor.hpp, 1057
include/linkpred/predictors/directed/dcnepredictor.hpp, 1059
include/linkpred/predictors/directed/dhdipredictor.hpp, 1060
include/linkpred/predictors/directed/dhpipredictor.hpp, 1062
include/linkpred/predictors/directed/directed.hpp, 1063
include/linkpred/predictors/directed/djidpredictor.hpp, 1064
include/linkpred/predictors/directed/dlcppredictor.hpp, 1066
include/linkpred/predictors/directed/dlhnpredictor.hpp, 1067
include/linkpred/predictors/directed/dlpredictor.hpp, 1069
include/linkpred/predictors/directed/dpatpredictor.hpp, 1070
include/linkpred/predictors/directed/dpstpredictor.hpp, 1071
include/linkpred/predictors/directed/dsaipredictor.hpp, 1073
include/linkpred/predictors/directed/dsoipredictor.hpp, 1074

include/linkpred/predictors/predictors.hpp, 1076
include/linkpred/predictors/undirected/uadapredictor.hpp, 1076
include/linkpred/predictors/undirected/ucnepredictor.hpp, 1078
include/linkpred/predictors/undirected/ucrapredictor.hpp, 1080
include/linkpred/predictors/undirected/ucstpredictor.hpp, 1081
include/linkpred/predictors/undirected/ueclpredictor.hpp, 1083
include/linkpred/predictors/undirected/uesmpredictor.hpp, 1084
include/linkpred/predictors/undirected/ufbmpredictor.hpp, 1086
include/linkpred/predictors/undirected/uhdipredictor.hpp, 1087
include/linkpred/predictors/undirected/uhpipredictor.hpp, 1089
include/linkpred/predictors/undirected/uhrgpredictor.hpp, 1090
include/linkpred/predictors/undirected/uhyppredictor.hpp, 1092
include/linkpred/predictors/undirected/ujidpredictor.hpp, 1094
include/linkpred/predictors/undirected/ukabpredictor.hpp, 1096
include/linkpred/predictors/undirected/ulcppredictor.hpp, 1098
include/linkpred/predictors/undirected/ulhnpredictor.hpp, 1099
include/linkpred/predictors/undirected/ulpredictor.hpp, 1101
include/linkpred/predictors/undirected/umpspredictor.hpp, 1102
include/linkpred/predictors/undirected/undirected.hpp, 1102
include/linkpred/predictors/undirected/unedpredictor.hpp, 1104
include/linkpred/predictors/undirected/upatpredictor.hpp, 1106
include/linkpred/predictors/undirected/upstpredictor.hpp, 1108
include/linkpred/predictors/undirected/uralpredictor.hpp, 1109
include/linkpred/predictors/undirected/urndpredictor.hpp, 1111
include/linkpred/predictors/undirected/usaipredictor.hpp, 1113
include/linkpred/predictors/undirected/usbmpredictor.hpp, 1114
include/linkpred/predictors/undirected/ushppredictor.hpp, 1116
include/linkpred/predictors/undirected/usoipredictor.hpp, 1118
include/linkpred/predictors/undirected/usumpredictor.hpp, 1119
include/linkpred/simp/edgescore.hpp, 1121
include/linkpred/simp/evaluator.hpp, 1122
include/linkpred/simp/perfres.hpp, 1123
include/linkpred/simp/predictor.hpp, 1125
include/linkpred/simp/simp.hpp, 1126
include/linkpred/utils/log.hpp, 1127
include/linkpred/utils/loglevel.hpp, 1128
include/linkpred/utils/miscutils.hpp, 1129
include/linkpred/utils/randomgen.hpp, 1132
include/linkpred/utils/utils.hpp, 1133
include/LinkPredConfig.hpp, 972
increase
LinkPred::BHeap< T, P, ComparatorT, ComparatorP >, 80
inEdgesBegin
LinkPred::DNetwork< LabelT, NodeIDT, EdgeT >, 201
inEdgesEnd
LinkPred::DNetwork< LabelT, NodeIDT, EdgeT >, 201
init
LinkPred::DADAPredictor< Network, EdgeRndIt, ScoreRndIt, EdgeRndOutIt >, 104
LinkPred::DCNEPredictor< Network, EdgeRndIt, ScoreRndIt, EdgeRndOutIt >, 110
LinkPred::DeepWalk< Network >, 118
LinkPred::DHDIPredictor< Network, EdgeRndIt, ScoreRndIt, EdgeRndOutIt >, 136
LinkPred::DHPIPredictor< Network, EdgeRndIt, ScoreRndIt, EdgeRndOutIt >, 142
LinkPred::DJIDPredictor< Network, EdgeRndIt, ScoreRndIt, EdgeRndOutIt >, 161
LinkPred::DLCPPredictor< Network, EdgeRndIt, ScoreRndIt, EdgeRndOutIt >, 166
LinkPred::DLHNPredictor< Network, EdgeRndIt, ScoreRndIt, EdgeRndOutIt >, 171
LinkPred::DLPredictor< NetworkT, EdgeRndItT, ScoreRndItT, EdgeRndOutItT >, 178
LinkPred::DPATPredictor< Network, EdgeRndIt, ScoreRndIt, EdgeRndOutIt >, 218
LinkPred::DPSTPredictor< Network, EdgeRndIt, ScoreRndIt, EdgeRndOutIt >, 223
LinkPred::DSAIPredictor< Network, EdgeRndIt, ScoreRndIt, EdgeRndOutIt >, 228
LinkPred::DSOIPredictor< Network, EdgeRndIt, ScoreRndIt, EdgeRndOutIt >, 234
LinkPred::Encoder< Network >, 259
LinkPred::HMSM< Network >, 355
LinkPred::LargeVis< Network >, 372
LinkPred::LINE< Network >, 381
LinkPred::LogMDSCG, 405
LinkPred::LogRegCG< InRndIt, OutRndIt >, 411
LinkPred::MatFact< Network >, 420
LinkPred::MatFactCG, 426
LinkPred::Node2Vec< Network >, 470
LinkPred::UADAPredictor< Network, EdgeRndIt, ScoreRndIt, EdgeRndOutIt >, 748
LinkPred::UCNEPredictor< Network, EdgeRndIt, ScoreRndIt, EdgeRndOutIt >, 753

LinkPred::UCRAPredictor< Network, EdgeRndIt, ScoreRndIt, EdgeRndOutIt >, 759
LinkPred::UCSTPredictor< Network, EdgeRndIt, ScoreRndIt, EdgeRndOutIt >, 765
LinkPred::UECLPredictor< Network, EdgeRndIt, ScoreRndIt, EdgeRndOutIt >, 772
LinkPred::UESMPredictor< Network, EdgeRndIt, ScoreRndIt, EdgeRndOutIt >, 778
LinkPred::UFBMPredictor< Network, EdgeRndIt, ScoreRndIt, EdgeRndOutIt >, 784
LinkPred::UHDIPredictor< Network, EdgeRndIt, ScoreRndIt, EdgeRndOutIt >, 789
LinkPred::UHPIPredictor< Network, EdgeRndIt, ScoreRndIt, EdgeRndOutIt >, 794
LinkPred::UHRGPredictor< Network, EdgeRndIt, ScoreRndIt, EdgeRndOutIt >, 800
LinkPred::UHYPPredictor< Network, EdgeRndIt, ScoreRndIt, EdgeRndOutIt >, 807
LinkPred::UJIDPredictor< Network, EdgeRndIt, ScoreRndIt, EdgeRndOutIt >, 814
LinkPred::UKABPredictor< Network, EdgeRndIt, ScoreRndIt, EdgeRndOutIt >, 820
LinkPred::ULCPPredictor< Network, EdgeRndIt, ScoreRndIt, EdgeRndOutIt >, 827
LinkPred::ULHNPredictor< Network, EdgeRndIt, ScoreRndIt, EdgeRndOutIt >, 832
LinkPred::ULPredictor< NetworkT, EdgeRndItT, ScoreRndItT, EdgeRndOutItT >, 839
LinkPred::UMPSPredictor< Network, EdgeRndIt, ScoreRndIt, EdgeRndOutIt >, 852
LinkPred::UNEDPredictor< Network, EdgeRndIt, ScoreRndIt, EdgeRndOutIt >, 864
LinkPred::UPATPredictor< Network, EdgeRndIt, ScoreRndIt, EdgeRndOutIt >, 897
LinkPred::UPSTPredictor< Network, EdgeRndIt, ScoreRndIt, EdgeRndOutIt >, 903
LinkPred::URALPredictor< Network, EdgeRndIt, ScoreRndIt, EdgeRndOutIt >, 908
LinkPred::URNDPredictor< Network, EdgeRndIt, ScoreRndIt, EdgeRndOutIt >, 913
LinkPred::USAIPredictor< Network, EdgeRndIt, ScoreRndIt, EdgeRndOutIt >, 919
LinkPred::USBMPredictor< Network, EdgeRndIt, ScoreRndIt, EdgeRndOutIt >, 924
LinkPred::USHPPredictor< Network, EdgeRndIt, ScoreRndIt, EdgeRndOutIt >, 931
LinkPred::USOIPredictor< Network, EdgeRndIt, ScoreRndIt, EdgeRndOutIt >, 938
LinkPred::USUMPredictor< Network, EdgeRndIt, ScoreRndIt, EdgeRndOutIt >, 943

inNeighborsBegin
LinkPred::DNetwork< LabelT, NodeIDT, EdgeT >, 201

inNeighborsEnd
LinkPred::DNetwork< LabelT, NodeIDT, EdgeT >, 202

insert
LinkPred::Bhmap< K, P, Comparator >, 85

int_cast
LinkPred::Utils, 51

InterpolMethod
LinkPred::PR< PredResultsT >, 571

IpoptMDS
LinkPred, 40

isConnected
LinkPred::NetworkManipulator< Network >, 460

isConstant
LinkPred::Vec, 954

isEdge
LinkPred::DNetwork< LabelT, NodeIDT, EdgeT >, 202
LinkPred::UNetwork< LabelT, NodeIDT, EdgeT >, 885, 886

isEdgeByID
LinkPred::Simp::Predictor, 582

isEdgeByLabel
LinkPred::Simp::Predictor, 582

isEnableReconstruct
LinkPred::LINE< Network >, 381

isLocked
LinkPred::TestData< Network, EdgeContT >, 700

isNegComputed
LinkPred::PredResults< TestDataT, LPredictorT, ScoresContainerT >, 619

isPosComputed
LinkPred::PredResults< TestDataT, LPredictorT, ScoresContainerT >, 619

isStrmEnabled
LinkPred::ROC< PredResultsT >, 675

isStrmNeg
LinkPred::ROC< PredResultsT >, 675

isTimingEnabled
LinkPred::PerfEvaluator< TestDataT, LPredictorT, PredResultsT, PerfMeasureT >, 560

isTnGenerated
LinkPred::TestData< Network, EdgeContT >, 700

isTopComputed
LinkPred::PredResults< TestDataT, LPredictorT, ScoresContainerT >, 619

isTpGenerated
LinkPred::TestData< Network, EdgeContT >, 700

isUseHops
LinkPred::ESPLSimlCalculator< Network, DistType, NbHopsType >, 300

isUseTopMethod
LinkPred::TPR< PredResultsT >, 743

IteratorType
LinkPred::TestData< Network, EdgeContT >, 695

j
LinkPred::MatFactPbData, 428
LinkPred::Simp::EdgeScore, 252
LinkPred::Simp::EdgeScoreByID, 253
LinkPred::Utils::EdgeScore< Label >, 251

k_const_iterator
LinkPred::Bhmap< K, P, Comparator >, 84

kbegin
LinkPred::Bhmap< K, P, Comparator >, 86
keepConnected
LinkPred::PerfeEvalExpDescp< Network >, 548
kend
LinkPred::Bhmap< K, P, Comparator >, 86
key
LinkPred::Bhmap< K, P, Comparator >, 86
kfind
LinkPred::Bhmap< K, P, Comparator >, 86

L
LinkPred::Simp::Evaluator::Factory::HYPParams, 360
L1Sim
LinkPred::L1Sim, 362, 363
L2Sim
LinkPred::L2Sim, 365, 366
Label
LinkPred::ASPDistCalculator< Network, DistType, NbHopsType >, 61
LinkPred::ASPDsimCalculator< Network, DsimType, NbHopsType >, 68
LinkPred::DESPLDistCalculator< Network, DistType, NbHopsType >, 124
LinkPred::Dijkstra< Network, DistType, NbHopsType >, 147
LinkPred::DNetwork< LabelT, NodeIDT, EdgeT >, 186
LinkPred::ESPDistCalculator< Network, DistType, NbHopsType >, 264
LinkPred::ESPDsimCalculator< Network, DsimType, NbHopsType >, 272
LinkPred::ESPIndSimlCalculator< Network, DistType, NbHopsType >, 280
LinkPred::ESPLDistCalculator< Network, DistType, NbHopsType >, 286
LinkPred::ESPLSimlCalculator< Network, DistType, NbHopsType >, 295
LinkPred::ESPSimlCalculator< Network, DistType, NbHopsType >, 304
LinkPred::NetDistCalculator< Network, DistType, NbHopsType >, 434
LinkPred::NetIndSimlCalculator< Network, DistType, NbHopsType >, 439
LinkPred::NetSimlCalculator< Network, DistType, NbHopsType >, 445
LinkPred::NetworkManipulator< Network >, 452
LinkPred::UNetwork< LabelT, NodeIDT, EdgeT >, 870
LabelIt
LinkPred::DNetwork< LabelT, NodeIDT, EdgeT >, 186
LinkPred::UNetwork< LabelT, NodeIDT, EdgeT >, 870
labelsBegin
LinkPred::DNetwork< LabelT, NodeIDT, EdgeT >, 203
LinkPred::UNetwork< LabelT, NodeIDT, EdgeT >, 886
labelsEnd
LinkPred::DNetwork< LabelT, NodeIDT, EdgeT >, 203
LinkPred::UNetwork< LabelT, NodeIDT, EdgeT >, 886
LambdaMethod
LinkPred::UMPSPredictor< Network, EdgeRndIt, ScoreRndIt, EdgeRndOutIt >, 846
LandmarkStrategy
LinkPred::UMPSPredictor< Network, EdgeRndIt, ScoreRndIt, EdgeRndOutIt >, 846
LinkPred::USHPPredictor< Network, EdgeRndIt, ScoreRndIt, EdgeRndOutIt >, 929
LargeVis
LinkPred::LargeVis< Network >, 369, 370
learn
LinkPred::Classifier< InRndIt, OutRndIt, ScoreRndIt >, 91
LinkPred::DADAPredictor< Network, EdgeRndIt, ScoreRndIt, EdgeRndOutIt >, 104
LinkPred::DCNEPredictor< Network, EdgeRndIt, ScoreRndIt, EdgeRndOutIt >, 110
LinkPred::DHDIPredictor< Network, EdgeRndIt, ScoreRndIt, EdgeRndOutIt >, 137
LinkPred::DHPIPredictor< Network, EdgeRndIt, ScoreRndIt, EdgeRndOutIt >, 142
LinkPred::DJIDPredictor< Network, EdgeRndIt, ScoreRndIt, EdgeRndOutIt >, 161
LinkPred::DLCPPredictor< Network, EdgeRndIt, ScoreRndIt, EdgeRndOutIt >, 167
LinkPred::DLHNPredictor< Network, EdgeRndIt, ScoreRndIt, EdgeRndOutIt >, 172
LinkPred::DLPredictor< NetworkT, EdgeRndItT, ScoreRndItT, EdgeRndOutItT >, 179
LinkPred::DPATPredictor< Network, EdgeRndIt, ScoreRndIt, EdgeRndOutIt >, 218
LinkPred::DPSTPredictor< Network, EdgeRndIt, ScoreRndIt, EdgeRndOutIt >, 223
LinkPred::DSAIPredictor< Network, EdgeRndIt, ScoreRndIt, EdgeRndOutIt >, 229
LinkPred::DSOIPredictor< Network, EdgeRndIt, ScoreRndIt, EdgeRndOutIt >, 234
LinkPred::LogisticRegresser< InRndIt, OutRndIt, ScoreRndIt >, 399
LinkPred::RndClassifier< InRndIt, OutRndIt, ScoreRndIt >, 635
LinkPred::UADAPredictor< Network, EdgeRndIt, ScoreRndIt, EdgeRndOutIt >, 748
LinkPred::UCNEPredictor< Network, EdgeRndIt, ScoreRndIt, EdgeRndOutIt >, 753
LinkPred::UCRAPredictor< Network, EdgeRndIt, ScoreRndIt, EdgeRndOutIt >, 759
LinkPred::UCSTPredictor< Network, EdgeRndIt, ScoreRndIt, EdgeRndOutIt >, 765
LinkPred::UECLPredictor< Network, EdgeRndIt, ScoreRndIt, EdgeRndOutIt >, 772

LinkPred::UESMPredictor< Network, EdgeRndIt, ScoreRndIt, EdgeRndOutIt >, 779
LinkPred::UFBMPredictor< Network, EdgeRndIt, ScoreRndIt, EdgeRndOutIt >, 784
LinkPred::UHDIPredictor< Network, EdgeRndIt, ScoreRndIt, EdgeRndOutIt >, 789
LinkPred::UHPIPredictor< Network, EdgeRndIt, ScoreRndIt, EdgeRndOutIt >, 794
LinkPred::UHRGPredictor< Network, EdgeRndIt, ScoreRndIt, EdgeRndOutIt >, 800
LinkPred::UHYPPredictor< Network, EdgeRndIt, ScoreRndIt, EdgeRndOutIt >, 807
LinkPred::UJIDPredictor< Network, EdgeRndIt, ScoreRndIt, EdgeRndOutIt >, 814
LinkPred::UKABPredictor< Network, EdgeRndIt, ScoreRndIt, EdgeRndOutIt >, 821
LinkPred::ULCPPredictor< Network, EdgeRndIt, ScoreRndIt, EdgeRndOutIt >, 827
LinkPred::ULHNPredictor< Network, EdgeRndIt, ScoreRndIt, EdgeRndOutIt >, 832
LinkPred::ULPredictor< NetworkT, EdgeRndItT, ScoreRndItT, EdgeRndOutItT >, 839
LinkPred::UMPSPredictor< Network, EdgeRndIt, ScoreRndIt, EdgeRndOutIt >, 852
LinkPred::UNEDPredictor< Network, EdgeRndIt, ScoreRndIt, EdgeRndOutIt >, 864
LinkPred::UPATPredictor< Network, EdgeRndIt, ScoreRndIt, EdgeRndOutIt >, 897
LinkPred::UPSTPredictor< Network, EdgeRndIt, ScoreRndIt, EdgeRndOutIt >, 903
LinkPred::URALPredictor< Network, EdgeRndIt, ScoreRndIt, EdgeRndOutIt >, 908
LinkPred::URNDPredictor< Network, EdgeRndIt, ScoreRndIt, EdgeRndOutIt >, 913
LinkPred::USAIPredictor< Network, EdgeRndIt, ScoreRndIt, EdgeRndOutIt >, 919
LinkPred::USBMPredictor< Network, EdgeRndIt, ScoreRndIt, EdgeRndOutIt >, 925
LinkPred::USHPPredictor< Network, EdgeRndIt, ScoreRndIt, EdgeRndOutIt >, 932
LinkPred::USOIPredictor< Network, EdgeRndIt, ScoreRndIt, EdgeRndOutIt >, 938
LinkPred::USUMPredictor< Network, EdgeRndIt, ScoreRndIt, EdgeRndOutIt >, 943
LengthMapIdType
LinkPred::ASPDistCalculator< Network, DistType, NbHopsType >, 61
LinkPred::ASPDsimCalculator< Network, DsimType, NbHopsType >, 68
LinkPred::DESPLDistCalculator< Network, DistType, NbHopsType >, 124
LinkPred::Dijkstra< Network, DistType, NbHopsType >, 147
LinkPred::ESPDistCalculator< Network, DistType, NbHopsType >, 264
LinkPred::ESPDsimCalculator< Network, DsimType, NbHopsType >, 272
LinkPred::ESPIndSimlCalculator< Network, DistType, NbHopsType >, 280
LinkPred::ESPLDistCalculator< Network, DistType, NbHopsType >, 286
LinkPred::ESPLSimlCalculator< Network, DistType, NbHopsType >, 295
LinkPred::ESPSimlCalculator< Network, DistType, NbHopsType >, 305
LinkPred::NetDistCalculator< Network, DistType, NbHopsType >, 434
LinkPred::NetIndSimlCalculator< Network, DistType, NbHopsType >, 439
LinkPred::NetSimlCalculator< Network, DistType, NbHopsType >, 445
LIN
LinkPred::PR< PredResultsT >, 571
LINE
LinkPred::LINE< Network >, 378, 379
Link prediction algorithms, 28
LinkClass
LinkPred, 39
LinkPred, 33
CacheLevel, 39
CGMDS, 40
Dec, 40
FN, 39
FP, 39
Inc, 40
IpoptMDS, 40
LinkClass, 39
logDebug, 40
logDebug1, 40
logDebug2, 40
logDebug3, 40
logError, 40
logInfo, 40
LogLevel, 39
logWarning, 40
MathPI, 46
MDSAlg, 40
NetworkCache, 39
NoCache, 39
NodeCache, 39
None, 40
operator∗, 40, 42
operator^, 46
operator+, 42, 43
operator-, 44
operator/, 45
PerfResults, 38
SortOrder, 40
TN, 39
TP, 39
LinkPred::ASPDistCalculator< Network, DistType, NbHopsType >, 59
∼ASPDistCalculator, 63
ASPDistCalculator, 62, 63
Edge, 61
EdgeLengthMap, 61

EdgeLengthMapSP, 61
getDist, 63, 64
getIndDist, 64
Label, 61
LengthMapIdType, 61
NetworkSP, 62
NodeDistMap, 62
NodeDistMapSP, 62
NodeID, 62
operator=, 65
setLandmarks, 65
LinkPred::ASPDsimCalculator< Network, DsimType, NbHopsType >, 66
~ASPDsimCalculator, 70
ASPDsimCalculator, 69, 70
Edge, 67
EdgeLengthMap, 68
EdgeLengthMapSP, 68
getDist, 70, 71
getIndDist, 71
Label, 68
LengthMapIdType, 68
NetworkSP, 68
NodeDsimMap, 68
NodeDsimMapSP, 69
NodeID, 69
operator=, 71, 72
setLandmarks, 72
LinkPred::BFS< Network, NodeProcessor >, 73
~BFS, 75
BFS, 74
operator=, 75
traverse, 76
LinkPred::BHeap< T, P, ComparatorT, ComparatorP >, 76
~BHeap, 79
BHeap, 78
contains, 79
decrease, 79
increase, 80
operator=, 80
pop, 80
print, 81
push, 81
set, 81
size, 82
top, 82
tryDecrease, 82
tryIncrease, 82
LinkPred::Bhmap< K, P, Comparator >, 83
~Bhmap, 85
Bhmap, 84, 85
insert, 85
k_const_iterator, 84
kbegin, 86
kend, 86
key, 86
kfind, 86
operator=, 87
p_const_iterator, 84
pbegin, 87
pend, 87
pfind, 88
pos, 88
size, 88
LinkPred::Classifier< InRndIt, OutRndIt, ScoreRndIt >, 89
~Classifier, 90
Classifier, 89, 90
getName, 90
learn, 91
operator=, 91
predict, 92
setName, 92
LinkPred::Collector< Network >, 93
~Collector, 94
Collector, 93, 94
getVisited, 94
operator=, 94, 95
process, 95
LinkPred::CosineSim, 95
~CosineSim, 97
CosineSim, 96, 97
operator=, 97, 98
sim, 98
LinkPred::Counter< Network >, 98
~Counter, 100
Counter, 99, 100
getCount, 100
operator=, 100, 101
process, 101
resetCount, 101
LinkPred::DADAPredictor< Network, EdgeRndIt, ScoreRndIt, EdgeRndOutIt >, 102
~DADAPredictor, 104
DADAPredictor, 103, 104
init, 104
learn, 104
operator=, 105
predict, 105
score, 106
top, 106
LinkPred::DCNEPredictor< Network, EdgeRndIt, ScoreRndIt, EdgeRndOutIt >, 107
~DCNEPredictor, 110
DCNEPredictor, 109
init, 110
learn, 110
operator=, 110, 111
predict, 111
score, 111
top, 112
LinkPred::DeepWalk< Network >, 113
~DeepWalk, 115
DeepWalk, 114, 115
encode, 115

getAlpha, 116
getDefaultDim, 116
getInitLR, 116
getMaxCodeLength, 116
getNbNodeWalks, 117
getNbWalks, 117
getStepInterval, 117
getTotalSteps, 117
getWalkLength, 118
getWindowSize, 118
init, 118
operator=, 118, 119
setAlpha, 119
setInitLR, 119
setNbNodeWalks, 119
setNbWalks, 120
setStepInterval, 120
setWalkLength, 120
setWeightMap, 121
setWindowSize, 121
LinkPred::DESPLDistCalculator< Network, DistType, NbHopsType >, 121
∼ DESPLDistCalculator, 126
DESPLDistCalculator, 125, 126
Edge, 123
EdgeLengthMap, 123
EdgeLengthMapSP, 124
getDist, 127
getFinDistMapNoNeighb, 128
getIndDist, 128
getLim, 128
getMaxNbNodesInCache, 129
getSDistMap, 129
Label, 124
LengthMapIdType, 124
NetworkSP, 124
NodeDistMap, 124
NodeDistMapSP, 124
NodeID, 125
NodeSDistMap, 125
NodeSDistMapSP, 125
operator=, 129, 130
setMaxNbNodesInCache, 130
LinkPred::DFS< Network, NodeProcessor >, 130
∼DFS, 133
DFS, 132
operator=, 133
traverse, 133
LinkPred::DHDIPredictor< Network, EdgeRndIt, ScoreRndIt, EdgeRndOutIt >, 134
∼DHDIPredictor, 136
DHDIPredictor, 135, 136
init, 136
learn, 137
operator=, 137
predict, 138
score, 138
top, 139
LinkPred::DHPIPredictor< Network, EdgeRndIt, ScoreRndIt, EdgeRndOutIt >, 139
∼DHPIPredictor, 142
DHPIPredictor, 141
init, 142
learn, 142
operator=, 142, 143
predict, 143
score, 144
top, 144
LinkPred::Dijkstra< Network, DistType, NbHopsType >, 145
∼Dijkstra, 149
Dijkstra, 148, 149
Edge, 146
EdgeLengthMap, 146
EdgeLengthMapSP, 147
getDist, 150
getDistL, 151
getDsim, 151, 153
getIndDist, 153
getIndDistToAll, 154
getIndDsim, 154
getNet, 155
getSDist, 155
getShortestPath, 156
Label, 147
LengthMapIdType, 147
NetworkSP, 147
NodeDistMap, 147
NodeDistMapSP, 147
NodeID, 148
NodeSDistMapSP, 148
operator=, 156, 157
PathType, 148
PathTypeSP, 148
registerLengthMap, 157
setNet, 157
unregisterLengthMap, 158
LinkPred::DJIDPredictor< Network, EdgeRndIt, ScoreRndIt, EdgeRndOutIt >, 158
∼DJIDPredictor, 161
DJIDPredictor, 160
init, 161
learn, 161
operator=, 161, 162
predict, 162
score, 162
top, 163
LinkPred::DLCPPredictor< Network, EdgeRndIt, ScoreRndIt, EdgeRndOutIt >, 164
∼DLCPPredictor, 166
DLCPPredictor, 165, 166
init, 166
learn, 167
operator=, 167
predict, 168
score, 168

LinkPred::DLHNPredictor< Network, EdgeRndIt, ScoreRndIt, EdgeRndOutIt >, 169
~DLHNPredictor, 171
DLHNPredictor, 170, 171
init, 171
learn, 172
operator=, 172
predict, 173
score, 173
top, 174
LinkPred::DLPredictor< NetworkT, EdgeRndItT, ScoreRndItT, EdgeRndOutItT >, 174
~DLPredictor, 178
DLPredictor, 177
Edge, 175
EdgeRndIt, 175
EdgeRndOutIt, 176
getName, 178
getNet, 178
init, 178
learn, 179
Network, 176
NodeID, 176
operator=, 179, 180
predict, 180
predictNeg, 180
score, 181
ScoreRndIt, 176
setName, 181
top, 182
LinkPred::DNetwork< LabelT, NodeIDT, EdgeT >, 182
~DNetwork, 188
addEdge, 188
addNode, 189
assemble, 189
compareEdgeEnd, 189
coupleAtOrd, 190
coupleOrd, 190
createEdgeMap, 190
createEdgeMapSP, 191
createNodeMap, 191
createNodeMapSP, 191
createNodeSMap, 192
createNodeSMapSP, 192
DNetwork, 187, 188
Edge, 185
EdgeIt, 185
EdgeMapSP, 186
edgesBegin, 192
edgesEnd, 193
end, 193
findLabel, 193
findNode, 194
getAvgDeg, 194
getAvgInDeg, 194
getAvgOutDeg, 194
getDeg, 195
getDegStat, 195
getID, 195
getInDeg, 196
getInDegStat, 196
getInOutDeg, 197
getLabel, 197
getMaxDeg, 197
getMaxInDeg, 197
getMaxOutDeg, 198
getMinDeg, 198
getMinInDeg, 198
getMinOutDeg, 198
getNbCouples, 199
getNbEdges, 199
getNbNodes, 199
getNbNonEdges, 199
getNbPaths, 200
getOutDeg, 200
getOutDegStat, 200
inEdgesBegin, 201
inEdgesEnd, 201
inNeighborsBegin, 201
inNeighborsEnd, 202
isEdge, 202
Label, 186
LabelIt, 186
labelsBegin, 203
labelsEnd, 203
makeEdge, 203
neighbBegin, 204
neighbEnd, 204
NodeID, 186
NodeIt, 186
NodeMapSP, 186
nodesBegin, 204
nodesDegBegin, 205
nodesDegEnd, 205
nodesEnd, 205
NodeSMapSP, 187
nonEdgesBegin, 205
nonEdgesEnd, 205
operator=, 206
outEdgesBegin, 206
outEdgesEnd, 207
outNeighborsBegin, 207
outNeighborsEnd, 207
print, 208
printEdges, 208
read, 208
readEdges, 209
reverseEdge, 209
rndEdgesBegin, 209
rndEdgesEnd, 210
rndNodesBegin, 210
rndNodesEnd, 210
rndNonEdgesBegin, 211
rndNonEdgesEnd, 211
shuffle, 211
start, 212

write, 212
LinkPred::DNetwork< LabelT, NodeIDT, EdgeT >::EdgeMap< ValueType >, 244
∼EdgeMap, 246
at, 247
begin, 247
cbegin, 247
cend, 247
EdgeMap, 246
EdgeMapConstIt, 245
EdgeMapIt, 245
end, 248
find, 248
operator=, 249
operator[], 249, 250
size, 250
LinkPred::DNetwork< LabelT, NodeIDT, EdgeT >::NodeDegIt, 474
difference_type, 475
DNetwork, 483
NodeDegIt, 476
operator!=, 477
operator<, 481
operator<=, 481
operator>, 482
operator>=, 483
operator∗, 477
operator+, 477
operator++, 478
operator+=, 478
operator-, 479
operator->, 480
operator--, 479, 480
operator-=, 480
operator=, 481, 482
operator==, 482
pointer, 476
reference, 476
LinkPred::DNetwork< LabelT, NodeIDT, EdgeT >::NodeMap< ValueType >, 499
∼NodeMap, 501
at, 502
begin, 502
cbegin, 502
cend, 502
end, 503
NodeMap, 501
NodeMapConstIt, 500
NodeMapIt, 500
operator=, 503
operator[], 504
LinkPred::DNetwork< LabelT, NodeIDT, EdgeT >::NodeSMap< ValueType >, 505
∼NodeSMap, 507
at, 507
begin, 507
cbegin, 508
cend, 508
end, 508
find, 508
NodeSMap, 506
NodeSMapConstIterator, 506
NodeSMapIterator, 506
operator=, 509
operator[], 509, 510
size, 510
LinkPred::DNetwork< LabelT, NodeIDT, EdgeT >::NonEdgeIt, 526
difference_type, 528
DNetwork, 535
NonEdgeIt, 528, 529
operator!=, 529
operator<, 533
operator<=, 533
operator>, 535
operator>=, 535
operator∗, 529
operator+, 530
operator++, 530
operator+=, 530
operator-, 531
operator->, 533
operator--, 532
operator-=, 532
operator=, 534
operator==, 534
pointer, 528
reference, 528
LinkPred::DNetwork< LabelT, NodeIDT, EdgeT >::RndEdgeIt, 641
operator!=, 644
operator∗, 644
operator++, 645
operator->, 645
operator=, 645, 646
operator==, 646
pointer, 643
reference, 643
RndEdgeIt, 643, 644
LinkPred::DNetwork< LabelT, NodeIDT, EdgeT >::RndNodeIt, 646
operator!=, 649
operator∗, 649
operator++, 649, 650
operator->, 650
operator=, 650, 651
operator==, 651
pointer, 648
reference, 648
RndNodeIt, 648, 649
LinkPred::DNetwork< LabelT, NodeIDT, EdgeT >::RndNonEdgeIt, 661
operator!=, 664
operator∗, 664
operator++, 665
operator->, 665

operator=, 665, 666
operator==, 666
pointer, 663
reference, 663
RndNonEdgeIt, 663, 664
LinkPred::DotProd, 212
∼DotProd, 214
DotProd, 213, 214
operator=, 214, 215
sim, 215
LinkPred::DPATPredictor< Network, EdgeRndIt, ScoreRndIt, EdgeRndOutIt >, 215
∼DPATPredictor, 218
DPATPredictor, 217
init, 218
learn, 218
operator=, 219
predict, 219
score, 220
LinkPred::DPSTPredictor< Network, EdgeRndIt, ScoreRndIt, EdgeRndOutIt >, 220
∼DPSTPredictor, 223
DPSTPredictor, 222, 223
init, 223
learn, 223
loadEdgeScores, 224
operator=, 224
score, 225
setEdgeScores, 225
LinkPred::DSAIPredictor< Network, EdgeRndIt, ScoreRndIt, EdgeRndOutIt >, 226
∼DSAIPredictor, 228
DSAIPredictor, 227, 228
init, 228
learn, 229
operator=, 229
predict, 230
score, 230
top, 231
LinkPred::DSOIPredictor< Network, EdgeRndIt, ScoreRndIt, EdgeRndOutIt >, 231
∼DSOIPredictor, 234
DSOIPredictor, 233
init, 234
learn, 234
operator=, 234, 235
predict, 235
score, 236
top, 236
LinkPred::Encoder< Network >, 254
∼Encoder, 257
CodeMap, 255
CodeMapSP, 255
Edge, 255
encode, 257
Encoder, 256
getDim, 257
getEdgeCode, 257, 258
getEdgeCodeDim, 258
getName, 258
getNodeCode, 258
getNodeCodeMap, 259
getWeightMap, 259
init, 259
NodeID, 255
operator=, 259, 260
setDim, 260
setName, 260
setWeightMap, 260
WeightMap, 255
WeightMapSP, 255
LinkPred::ESPDistCalculator< Network, DistType, NbHopsType >, 262
∼ESPDistCalculator, 267
Edge, 264
EdgeLengthMap, 264
EdgeLengthMapSP, 264
ESPDistCalculator, 265, 267
getCacheHits, 268
getCacheMiss, 268
getDist, 268
getIndDist, 269
Label, 264
LengthMapIdType, 264
NetworkSP, 265
NodeDistMap, 265
NodeDistMapSP, 265
NodeID, 265
operator=, 269, 270
LinkPred::ESPDsimCalculator< Network, DsimType, NbHopsType >, 270
∼ESPDsimCalculator, 275
Edge, 272
EdgeLengthMap, 272
EdgeLengthMapSP, 272
ESPDsimCalculator, 273, 275
getDist, 276
getIndDist, 276
Label, 272
LengthMapIdType, 272
NetworkSP, 273
NodeDsimMap, 273
NodeDsimMapSP, 273
NodeID, 273
operator=, 277
LinkPred::ESPIndSimlCalculator< Network, DistType, NbHopsType >, 277
∼ESPIndSimlCalculator, 282
Edge, 279
EdgeLengthMap, 279
EdgeLengthMapSP, 279
ESPIndSimlCalculator, 281
getDirIndSiml, 282
getIndSiml, 282
Label, 280
LengthMapIdType, 280

NetworkSP, 280
NodeDistMap, 280
NodeDistMapSP, 280
NodeID, 280
operator=, 283
LinkPred::ESPLDistCalculator< Network, DistType, NbHopsType >, 284
∼ ESPLDistCalculator, 288
Edge, 285
EdgeLengthMap, 286
EdgeLengthMapSP, 286
ESPLDistCalculator, 287, 288
getDist, 289
getFinDistMapNoNeighb, 290
getIndDist, 290
getLim, 291
getMaxNbNodesInCache, 291
getSDistMap, 291
Label, 286
LengthMapIdType, 286
NetworkSP, 286
NodeDistMap, 286
NodeDistMapSP, 287
NodeID, 287
NodeSDistMap, 287
NodeSDistMapSP, 287
operator=, 292
setMaxNbNodesInCache, 292
LinkPred::ESPLSimlCalculator< Network, DistType, NbHopsType >, 293
∼ ESPLSimlCalculator, 297
Edge, 295
EdgeLengthMap, 295
EdgeLengthMapSP, 295
ESPLSimlCalculator, 296, 297
getDirSiml, 298
getLim, 298
getMaxNbNodesInCache, 299
getNnzSimlMap, 299
getNnzSimlMapNoNeighb, 299
getSiml, 300
isUseHops, 300
Label, 295
LengthMapIdType, 295
NetworkSP, 295
NodeID, 296
NodeSDistMap, 296
NodeSDistMapSP, 296
operator=, 301
setMaxNbNodesInCache, 301
setUseHops, 302
LinkPred::ESPSimlCalculator< Network, DistType, NbHopsType >, 302
∼ESPSimlCalculator, 307
Edge, 304
EdgeLengthMap, 304
EdgeLengthMapSP, 304
ESPSimlCalculator, 306
getDirSiml, 307
getMaxNbNodesInCache, 308
getSiml, 308
Label, 304
LengthMapIdType, 305
NetworkSP, 305
NodeDistMap, 305
NodeDistMapSP, 305
NodeID, 305
operator=, 309
setMaxNbNodesInCache, 309
LinkPred::FastSig< T >, 321
∼FastSig, 322
FastSig, 321, 322
operator(), 323
operator=, 323
LinkPred::GCurve< PredResultsT >, 325
∼GCurve, 328
eval, 328, 329
GCurve, 327, 328
getCurve, 329, 330
getGCurve, 330
getGCurveAuc, 330
getThresholds, 331
operator=, 331, 332
ScoresItT, 326
LinkPred::GFMatrix, 332
∼GFMatrix, 334
diag, 334
elemMult, 335
get, 335
getCol, 335
getCols, 336
getM, 336
getN, 336
getName, 336
getRow, 337
getRows, 337
GFMatrix, 333, 334
mult, 337, 338
operator!=, 338
operator∗, 342, 343
operator+, 344, 345
operator-, 345–347
operator/, 347
operator=, 338, 339
operator==, 339
print, 339
removeNaN, 340
set, 340
setCol, 340
setDiag, 341
setName, 341
setRow, 341
sum, 341
sumCols, 342
sumRows, 342

LinkPred::GraphTraversal< Network, NodeProcessor >, 348
~GraphTraversal, 349
GraphTraversal, 348, 349
operator=, 350
traverse, 350
LinkPred::HMSM< Network >, 351
~HMSM, 353
encode, 353
getAlpha, 354
getDefaultDim, 354
getEps, 354
getHProb, 354
getLProb, 354
getNbMdsRuns, 355
getTol, 355
HMSM, 352, 353
init, 355
operator=, 355, 356
setAlpha, 356
setEps, 356
setHProb, 356
setNbMdsRuns, 357
setProb, 357
setTol, 357
setWeightMap, 358
LinkPred::L1Sim, 361
~L1Sim, 363
L1Sim, 362, 363
operator=, 363
sim, 364
LinkPred::L2Sim, 364
~L2Sim, 366
L2Sim, 365, 366
operator=, 366
sim, 367
LinkPred::LargeVis< Network >, 367
~LargeVis, 370
encode, 370
getDefaultDim, 370
getGamma, 371
getInitLR, 371
getNbNegSamples, 371
getNbSamples, 371
getNegSamplingPower, 371
getNegSize, 372
init, 372
LargeVis, 369, 370
operator=, 372
setGamma, 374
setInitLR, 374
setNbNegSamples, 374
setNbSamples, 374, 375
setNegSamplingPower, 375
setNegSize, 375
LinkPred::LINE< Network >, 377
~LINE, 379
encode, 379
getDefaultDim, 379
getInitLr, 380
getNbNegSamples, 380
getNegSamplingPower, 380
getNegSize, 380
getOrder, 381
getRecNet, 381
getRecWeightMap, 381
init, 381
isEnableReconstruct, 381
LINE, 378, 379
operator=, 382
setEnableReconstruct, 382
setInitLr, 383
setNbNegSamples, 383
setNegSamplingPower, 383
setNegSize, 383
setOrder, 384
LinkPred::LMapQueue< K, P, KComparator, PComparator >, 384
~LMapQueue, 386
at, 387
begin, 387, 388
cbegin, 388
cend, 388
clear, 388
compareTop, 388
count, 389
empty, 389
end, 389
find, 390
getL, 390
LMapQueue, 385, 386
merge, 390
operator=, 390, 391
pop, 391
printPQ, 392
push, 392
size, 393
top, 393
LinkPred::Log, 393
~Log, 394
DefaultLogLevel, 395
DefaultOutputStream, 395
Log, 394
logLevel, 395
operator<<, 394
out, 395
LinkPred::LogisticRegresser< InRndIt, OutRndIt, ScoreRndIt >, 396
~LogisticRegresser, 398
getLambda, 398
getTheta, 398
getTol, 399
learn, 399
LogisticRegresser, 397, 398
operator=, 399, 400
predict, 400

setLambda, 400
setTol, 401
LinkPred::LogMDSCG, 401
~LogMDSCG, 403
f, 404
fgrad, 404
finalize, 404
grad, 405
init, 405
LogMDSCG, 402, 403
operator=, 405, 406
LinkPred::LogRegCG< InRndIt, OutRndIt >, 406
~LogRegCG, 409
f, 409
fgrad, 409
finalize, 410
getLambda, 410
grad, 410
init, 411
LogRegCG, 408
operator=, 411, 412
setLambda, 412
LinkPred::LPSim, 412
~LPSim, 414
getP, 414
LPSim, 413, 414
operator=, 415
setP, 415
sim, 416
LinkPred::MatFact< Network >, 416
~MatFact, 419
encode, 419
getDefaultDim, 419
getLambda, 419
getNegRatio, 419
getPosRatio, 420
getTol, 420
init, 420
MatFact, 418
operator=, 420, 421
setLambda, 421
setNegRatio, 421
setPosRatio, 421
setTol, 422
setWeightMap, 422
LinkPred::MatFactCG, 423
~MatFactCG, 425
f, 425
fgrad, 425
finalize, 426
grad, 426
init, 426
MatFactCG, 424
operator=, 427
LinkPred::MatFactPbData, 427
i, 428
j, 428
target, 428
LinkPred::MDS, 428
~MDS, 429
cgMDS, 430
getAlg, 430
getNbRuns, 430
getTol, 431
MDS, 429
setAlg, 431
setNbRuns, 431
setTol, 431
solve, 432
LinkPred::NetDistCalculator< Network, DistType, NbHopsType >, 433
~NetDistCalculator, 435
Edge, 433
EdgeLengthMap, 434
EdgeLengthMapSP, 434
getDiscDist, 435
getDiscNbHops, 435
getDist, 436
getIndDist, 436
getMaxDist, 437
Label, 434
LengthMapIdType, 434
NetworkSP, 434
NodeDistMap, 434
NodeDistMapSP, 435
NodeID, 435
setDiscDist, 437
setDiscNbHops, 438
LinkPred::NetIndSimlCalculator< Network, DistType, NbHopsType >, 438
~NetIndSimlCalculator, 440
Edge, 439
EdgeLengthMap, 439
EdgeLengthMapSP, 439
getDirIndSiml, 440
getDiscDist, 441
getDiscNbHops, 441
getIndSiml, 441
getLambda, 442
getSelfSiml, 442
Label, 439
LengthMapIdType, 439
NetworkSP, 440
NodeID, 440
setDiscDist, 442
setDiscNbHops, 442
setLambda, 443
setSelfSiml, 443
LinkPred::NetSimlCalculator< Network, DistType, NbHopsType >, 443
~NetSimlCalculator, 446
Edge, 444
EdgeLengthMap, 445
EdgeLengthMapSP, 445
getDirSiml, 446
getDiscDist, 447

getDiscNbHops, 447
getLambda, 447
getNnzSimlMap, 447
getNnzSimlMapNoNeighb, 448
getSelfSiml, 448
getSiml, 448
Label, 445
LengthMapIdType, 445
NetworkSP, 445
NodeID, 445
NodeSDistMap, 446
NodeSDistMapSP, 446
setDiscDist, 449
setDiscNbHops, 449
setLambda, 449
setSelfSiml, 449
LinkPred::NetworkManipulator< Network >, 450
∼NetworkManipulator, 453
createTestData, 453, 454
createTestDataAdd, 455
createTestDataRem, 456
createTestDataSeq, 457
createTestDataSeqInter, 458
Edge, 452
EdgeMap, 452
getAllNegLinksExcept, 459
getRndNegLinksExcept, 459
getRndPosLinksExcept, 459
isConnected, 460
Label, 452
loadTestData, 460
loadTestDataAdd, 462
loadTestDataRem, 462
NetworkCSP, 452
NetworkManipulator, 453
NetworkSP, 452
NodeID, 452
NodeMap, 453
rndConExtract, 463
rndExtract, 463
rst, 464
LinkPred::Node2Vec< Network >, 464
∼Node2Vec, 467
encode, 467
getDefaultDim, 468
getInitLR, 468
getNbNegSamples, 468
getNbWalks, 468
getP, 469
getQ, 469
getStepInterval, 469
getSubSample, 469
getTotalSteps, 470
getWalkLength, 470
getWindowSize, 470
init, 470
Node2Vec, 466, 467
operator=, 470, 471
setInitLR, 471
setNbNegSamples, 471
setNbWalks, 471
setP, 472
setQ, 472
setStepInterval, 472
setSubSample, 473
setWalkLength, 473
setWeightMap, 473
setWindowSize, 473
LinkPred::Pearson, 537
∼Pearson, 538
operator=, 539
Pearson, 538
sim, 539
LinkPred::PEFactory< Network, LPredictorT, TestDataT, PerfMeasureT >, 540
∼PEFactory, 540
getPerfMeasures, 541
getPredictors, 541
LinkPred::PerfCurve< PredResultsT >, 541
∼PerfCurve, 544
eval, 544, 545
getCurve, 545, 546
operator=, 546, 547
PerfCurve, 543, 544
ScoresItT, 543
LinkPred::PerfeEvalExpDescp< Network >, 547
fnRatio, 548
keepConnected, 548
nbTestRuns, 548
out, 548
ratioEnd, 548
ratioStart, 548
ratioStep, 548
refNet, 549
seed, 549
timingEnabled, 549
tnRatio, 549
LinkPred::PerfEvalExp< Network, TestDataT, LPredictorT, PredResultsT, PerfMeasureT >, 549
∼PerfEvalExp, 551
getFactory, 552
getOutPrec, 552
getPed, 552
operator=, 552, 553
PerfEvalExp, 550, 551
resultsBegin, 553
resultsEnd, 553
run, 554
runNoTiming, 554
runTiming, 554
setOutPrec, 554
LinkPred::PerfEvaluator< TestDataT, LPredictorT, PredResultsT, PerfMeasureT >, 555
∼PerfEvaluator, 557
addPerfMeasure, 557
addPredictor, 557

eval, 559
evalNoTiming, 559
evalTiming, 559
getNbPerfMeasures, 559
getNbPredictors, 559
getPredictor, 560
isTimingEnabled, 560
operator=, 560, 561
PerfEvaluator, 556
resultsBegin, 561
resultsEnd, 561
setTimingEnabled, 562
LinkPred::PerfMeasure< PredResultsT >, 562
∼PerfMeasure, 564
eval, 565
getName, 566
operator=, 566
PerfMeasure, 563, 564
requiresNeg, 566
requiresPos, 567
requiresShuffling, 567
ScoresItT, 563
setName, 567
LinkPred::PR< PredResultsT >, 569
∼PR, 572
DGI, 571
eval, 572, 573
getCurve, 573, 574
getInterpolMethod, 574
getPRAuc, 574
getPRAucDGI, 575
getPRAucLIN, 576
getPRCurve, 576
getThresholds, 577
InterpolMethod, 571
LIN, 571
operator=, 577
PR, 571, 572
ScoresItT, 571
setInterpolMethod, 578
LinkPred::PredResults< TestDataT, LPredictorT, ScoresContainerT >, 614
∼PredResults, 616
compNegScores, 617
compPosScores, 617
compTopScores, 617
getNbTop, 617
getNegSortOrder, 618
getPosSortOrder, 618
getPredictor, 618
getTestData, 618
isNegComputed, 619
isPosComputed, 619
isTopComputed, 619
negBegin, 619
negEnd, 620
negStrmBegin, 620
negStrmEnd, 620
operator=, 620, 622
posBegin, 622
posEnd, 622
posStrmBegin, 622
posStrmEnd, 623
PredResults, 615, 616
ScoresItT, 615
sortNeg, 623
sortPos, 623
topBegin, 625
topEdgesBegin, 625
topEdgesEnd, 625
topEnd, 625
LinkPred::PredResults< TestDataT, LPredictorT, ScoresContainerT >::ScoreIterator, 678
difference_type, 679
operator!=, 682
operator<, 686
operator<=, 686
operator>, 688
operator>=, 688
operator∗, 682
operator+, 683
operator++, 683
operator+=, 683
operator-, 684
operator->, 686
operator--, 685
operator-=, 685
operator=, 687
operator==, 687
pointer, 679
PredResults, 688
reference, 680
ScoreIterator, 680, 682
LinkPred::RandomGen, 627
∼RandomGen, 628
getBool, 628
getDouble, 628
getGeo, 629
getInt, 629
getPL, 629
getSeed, 630
getSInt, 630
getUInt, 630
operator=, 631
RandomGen, 627, 628
LinkPred::RndClassifier< InRndIt, OutRndIt, ScoreRndIt >, 632
∼RndClassifier, 635
learn, 635
operator=, 635, 636
predict, 636
RndClassifier, 633
LinkPred::ROC< PredResultsT >, 667
∼ROC, 670
eval, 670, 671
evalNoStream, 671

evalStream, 672
getCurve, 672
getROCAuc, 673
getROCAucStrm, 673
getROCCurve, 674
getThresholds, 674
isStrmEnabled, 675
isStrmNeg, 675
operator=, 675, 676
ROC, 669, 670
ScoresItT, 669
setStrmEnabled, 676
setStrmNeg, 676
LinkPred::SimMeasure, 690
∼SimMeasure, 691
getName, 691
operator=, 691, 692
setName, 692
sim, 692
SimMeasure, 690, 691
LinkPred::Simp, 47
LinkPred::Simp::EdgeScore, 252
i, 252
j, 252
score, 252
LinkPred::Simp::EdgeScoreByID, 253
i, 253
j, 253
score, 253
LinkPred::Simp::Evaluator, 310
∼Evaluator, 311
addADA, 311
addCNE, 312
addCRA, 312
addECL, 312
addESM, 313
addFBM, 313
addHDI, 314
addHPI, 314
addHRG, 314
addHYP, 315
addJID, 315
addKAB, 315
addLCP, 316
addLHN, 316
addPAT, 316
addPR, 316
addPST, 317
addRAL, 317
addRND, 317
addROC, 318
addSAI, 318
addSBM, 318
addSHP, 318
addSOI, 319
addTPR, 319
Evaluator, 311
genTestData, 319
getPerfRes, 320
run, 320
LinkPred::Simp::Evaluator::Factory::ECLParams, 237
classifierName, 237
dim, 237
encoderName, 237
negRatio, 237
posRatio, 238
seed, 238
LinkPred::Simp::Evaluator::Factory::ESMParams, 261
dim, 261
encoderName, 261
seed, 262
simMeasureName, 262
LinkPred::Simp::Evaluator::Factory::FBMParams, 324
maxIter, 324
seed, 324
LinkPred::Simp::Evaluator::Factory::HRGParams, 358
nbBeans, 358
nbSamples, 359
seed, 359
LinkPred::Simp::Evaluator::Factory::HYPParams, 359
gamma, 359
L, 360
m, 360
seed, 360
T, 360
zeta, 360
LinkPred::Simp::Evaluator::Factory::KABParams, 360
horizLim, 361
LinkPred::Simp::Evaluator::Factory::LCPParams, 376
epsilon, 376
LinkPred::Simp::Evaluator::Factory::PSTParams, 626
fileName, 626
LinkPred::Simp::Evaluator::Factory::RNDParams, 666
seed, 667
LinkPred::Simp::Evaluator::Factory::SBMParams, 677
maxIter, 677
seed, 677
LinkPred::Simp::Evaluator::Factory::SHPParams, 689
seed, 689
LinkPred::Simp::PerfRes, 568
name, 568
res, 568
LinkPred::Simp::Predictor, 578
∼Predictor, 581
getID, 581
getLabel, 581
getNbNodes, 582
isEdgeByID, 582
isEdgeByLabel, 582
loadnet, 583
predADA, 583
predAllADA, 583
predAllADAByID, 583
predAllCNE, 584
predAllCNEByID, 584
predAllCRA, 584

predAllCRAByID, 584
predAllECL, 585
predAllECLByID, 585
predAllESM, 586
predAllESMByID, 586
predAllFBM, 587
predAllFBMByID, 587
predAllHDI, 589
predAllHDIByID, 589
predAllHPI, 589
predAllHPIByID, 589
predAllHRG, 590
predAllHRGByID, 590
predAllHYP, 591
predAllHYPByID, 591
predAllJID, 592
predAllJIDByID, 592
predAllKAB, 592
predAllKABByID, 592
predAllLCP, 593
predAllLCPByID, 593
predAllLHN, 594
predAllLHNByID, 594
predAllPAT, 594
predAllPATByID, 594
predAllRAL, 594
predAllRALByID, 595
predAllRND, 595
predAllRNDByID, 595
predAllSAI, 596
predAllSAIByID, 596
predAllSBM, 596
predAllSBMByID, 597
predAllSHP, 597
predAllSHPByID, 597
predAllSOI, 598
predAllSOIByID, 598
predCNE, 598
predCRA, 598
predECL, 599
predESM, 599
predFBM, 600
predHDI, 600
predHPI, 600
predHRG, 601
predHYP, 601
Predictor, 580
predJID, 602
predKAB, 602
predLCP, 602
predLHN, 603
predPAT, 603
predRAL, 603
predRND, 603
predSAI, 604
predSBM, 604
predSHP, 604
predSOI, 605
predTopADA, 605
predTopCNE, 605
predTopCRA, 606
predTopECL, 606
predTopESM, 607
predTopFBM, 607
predTopHDI, 608
predTopHPI, 608
predTopHRG, 608
predTopHYP, 609
predTopJID, 609
predTopKAB, 610
predTopLCP, 610
predTopLHN, 611
predTopPAT, 611
predTopRAL, 611
predTopRND, 612
predTopSAI, 612
predTopSBM, 612
predTopSHP, 613
predTopSOI, 613
LinkPred::TestData< Network, EdgeContT >, 693
~TestData, 697
ECEIT, 696
Edge, 694
EdgeIt, 695
genNeg, 698
genPos, 698
getEg, 698
getNbNeg, 698
getNbPos, 698
getNegClass, 699
getObsNet, 699
getPosClass, 699
getRefNet, 699
getRemLinksMap, 700
isLocked, 700
isTnGenerated, 700
isTpGenerated, 700
IteratorType, 695
lock, 701
negBegin, 701
negEnd, 701
negStrmBegin, 701
negStrmEnd, 702
NetworkCSP, 695
NetworkSP, 695
NonEdgeIt, 695
operator=, 702
posBegin, 703
posEnd, 703
posStrmBegin, 703
posStrmEnd, 703
TestData, 696, 697
TNEIT, 696
TPEIT, 696
LinkPred::TestData< Network, EdgeContT >::TestEdgeIt, 709

difference_type, 711
operator!=, 712
operator<, 716
operator<=, 716
operator>, 718
operator>=, 718
operator*, 713
operator+, 713
operator++, 713, 714
operator+=, 714
operator-, 714, 715
operator->, 716
operator--, 715
operator-=, 715
operator=, 717
operator==, 717
pointer, 711
reference, 711
TestData, 719
TestEdgeIt, 711, 712
LinkPred::TestEdgeGen< Network, EdgeContT >, 704
~TestEdgeGen, 706
generateTN, 706
generateTP, 707
operator=, 707
TestEdgeGen, 705, 706
tnBegin, 708
tnEnd, 708
tpBegin, 708
tpEnd, 708
LinkPred::TestEdgeGen< Network, EdgeContT >::TNEdgeIt, 719
difference_type, 721
operator!=, 722
operator<, 726
operator<=, 726
operator>, 728
operator>=, 728
operator*, 722
operator+, 723
operator++, 723
operator+=, 723
operator-, 724
operator->, 726
operator--, 725
operator-=, 725
operator=, 727
operator==, 727
pointer, 721
reference, 721
TestEdgeGen, 728
TNEdgeIt, 721, 722
LinkPred::TestEdgeGen< Network, EdgeContT >::TPEdgeIt, 729
difference_type, 730
operator!=, 732
operator<, 735
operator<=, 736
operator>, 737
operator>=, 737
operator*, 732
operator+, 732
operator++, 733
operator+=, 733
operator-, 734
operator->, 735
operator--, 734, 735
operator-=, 735
operator=, 736
operator==, 737
pointer, 730
reference, 731
TestEdgeGen, 738
TPEdgeIt, 731
LinkPred::TPR< PredResultsT >, 738
~TPR, 741
eval, 741, 742
evalUsingPredict, 742
evalUsingTop, 743
getTPR, 743
isUseTopMethod, 743
operator=, 744
requiresNeg, 744
requiresShuffling, 744
ScoresItT, 740
setUseTopMethod, 745
TPR, 740, 741
LinkPred::UADAPredictor< Network, EdgeRndIt, ScoreRndIt, EdgeRndOutIt >, 745
~UADAPredictor, 748
init, 748
learn, 748
operator=, 748, 749
predict, 749
score, 749
top, 750
UADAPredictor, 747
LinkPred::UCNEPredictor< Network, EdgeRndIt, ScoreRndIt, EdgeRndOutIt >, 751
~UCNEPredictor, 753
init, 753
learn, 753
operator=, 754
predict, 754
score, 755
top, 755
UCNEPredictor, 752, 753
LinkPred::UCRAPredictor< Network, EdgeRndIt, ScoreRndIt, EdgeRndOutIt >, 756
~UCRAPredictor, 759
init, 759
learn, 759
operator=, 759, 760
predict, 760
score, 760
top, 761

UCRAPredictor, 758
LinkPred::UCSTPredictor< Network, EdgeRndIt, ScoreRndIt, EdgeRndOutIt >, 762
~UCSTPredictor, 765
init, 765
learn, 765
operator=, 766
predict, 766
score, 767
top, 767
UCSTPredictor, 763, 765
LinkPred::UECLPredictor< Network, EdgeRndIt, ScoreRndIt, EdgeRndOutIt >, 768
~UECLPredictor, 771
getClassifier, 771
getEncoder, 771
getNegRatio, 771
getPosRatio, 772
init, 772
learn, 772
operator=, 772, 773
score, 773
setClassifier, 774
setEncoder, 774
setNegRatio, 774
setPosRatio, 775
UECLPredictor, 770
LinkPred::UESMPredictor< Network, EdgeRndIt, ScoreRndIt, EdgeRndOutIt >, 775
~UESMPredictor, 778
getEncoder, 778
getSimMeasure, 778
init, 778
learn, 779
operator=, 779
score, 780
setEncoder, 780
setSimMeasure, 780
UESMPredictor, 777
LinkPred::UFBMPredictor< Network, EdgeRndIt, ScoreRndIt, EdgeRndOutIt >, 781
~UFBMPredictor, 783
getMaxIter, 784
init, 784
learn, 784
operator=, 784, 785
predict, 785
score, 785
setMaxIter, 786
UFBMPredictor, 782, 783
LinkPred::UHDIPredictor< Network, EdgeRndIt, ScoreRndIt, EdgeRndOutIt >, 786
~UHDIPredictor, 789
init, 789
learn, 789
operator=, 789, 790
predict, 790
score, 791
top, 791
UHDIPredictor, 788
LinkPred::UHPIPredictor< Network, EdgeRndIt, ScoreRndIt, EdgeRndOutIt >, 792
~UHPIPredictor, 794
init, 794
learn, 794
operator=, 795
predict, 795
score, 796
top, 796
UHPIPredictor, 793, 794
LinkPred::UHRGPredictor< Network, EdgeRndIt, ScoreRndIt, EdgeRndOutIt >, 797
~UHRGPredictor, 799
getNbBeans, 800
getNbSamples, 800
init, 800
learn, 800
operator=, 801
predict, 801
score, 802
setNbBeans, 802
setNbSamples, 802
UHRGPredictor, 799
LinkPred::UHYPPredictor< Network, EdgeRndIt, ScoreRndIt, EdgeRndOutIt >, 803
~UHYPPredictor, 805
getGamma, 806
getL, 806
getM, 806
getSeed, 806
getT, 807
getZeta, 807
init, 807
learn, 807
operator=, 808
predict, 808
score, 809
setGamma, 809
setL, 810
setM, 810
setT, 810
setZeta, 811
UHYPPredictor, 804, 805
LinkPred::UJIDPredictor< Network, EdgeRndIt, ScoreRndIt, EdgeRndOutIt >, 811
~UJIDPredictor, 814
init, 814
learn, 814
operator=, 814, 815
predict, 815
score, 815
top, 816
UJIDPredictor, 813
LinkPred::UKABPredictor< Network, EdgeRndIt, ScoreRndIt, EdgeRndOutIt >, 817
~UKABPredictor, 820

Edge, 818
getCacheLevel, 820
getHorizLim, 820
init, 820
learn, 821
NodeID, 818
operator=, 821
predict, 822
score, 822
setCacheLevel, 823
setHorizLim, 823
top, 823
UKABPredictor, 819
LinkPred::ULCPPredictor< Network, EdgeRndIt, ScoreRndIt, EdgeRndOutIt >, 824
~ULCPPredictor, 826
getEpsilon, 826
init, 827
learn, 827
operator=, 827, 828
predict, 828
score, 828
setEpsilon, 829
ULCPPredictor, 825, 826
LinkPred::ULHNPredictor< Network, EdgeRndIt, ScoreRndIt, EdgeRndOutIt >, 829
~ULHNPredictor, 832
init, 832
learn, 832
operator=, 832, 833
predict, 833
score, 834
top, 834
ULHNPredictor, 831
LinkPred::ULPredictor< NetworkT, EdgeRndItT, ScoreRndItT, EdgeRndOutItT >, 835
~ULPredictor, 838
Edge, 836
EdgeRndIt, 836
EdgeRndOutIt, 836
getName, 838
getNet, 839
init, 839
learn, 839
Network, 836
NodeID, 837
operator=, 840
predict, 841
predictNeg, 841
score, 842
ScoreRndIt, 837
setName, 842
top, 842
ULPredictor, 837, 838
LinkPred::UMPSPredictor< Network, EdgeRndIt, ScoreRndIt, EdgeRndOutIt >, 843
~UMPSPredictor, 848
EdgeLengthMethod, 846
getAsp, 848
getCacheLevel, 848
getEdgeLengthMethod, 848
getLambda, 849
getLambdaMethod, 849
getLambdaNegEstRatio, 849
getLambdaPosEstRatio, 849
getLambdaStep, 850
getLandmarkRatio, 850
getLandmarkStrategy, 850
getLength, 850
getMaxLambda, 851
getMinLambda, 851
getNegScore, 851
getPerfMeasure, 851
getPosScore, 852
getTol, 852
Hub, 847
IHub, 847
init, 852
LambdaMethod, 846
LandmarkStrategy, 846
learn, 852
MeanApp, 846
operator=, 853
Opt, 846
PAT, 846
predict, 853
RA1, 846
RA2, 846
Random, 847
Scan, 846
score, 854
setAsp, 854
setCacheLevel, 855
setEdgeLengthMethod, 855
setLambda, 855
setLambdaMethod, 856
setLambdaNegEstRatio, 856
setLambdaPosEstRatio, 856
setLambdaStep, 858
setLandmarkRatio, 858
setLandmarkStrategy, 858
setMaxLambda, 859
setMinLambda, 859
setNegScore, 859
setPerfMeasure, 860
setPosScore, 860
setTol, 860
UMPSPredictor, 847
User, 846
LinkPred::UNEDPredictor< Network, EdgeRndIt, ScoreRndIt, EdgeRndOutIt >, 861
~UNEDPredictor, 864
Edge, 862
init, 864
learn, 864
NodeID, 862

operator=, 864, 865
predict, 865
score, 866
top, 866
UNEDPredictor, 863
LinkPred::UNetwork< LabelT, NodeIDT, EdgeT >, 867
~UNetwork, 872
addEdge, 872
addNode, 873
assemble, 873
compareEdgeEnd, 873
coupleAtOrd, 874
coupleOrd, 874
createEdgeMap, 874
createEdgeMapSP, 875
createNodeMap, 875
createNodeMapSP, 875
createNodeSMap, 876
createNodeSMapSP, 876
Edge, 870
EdgeIt, 870
EdgeMapSP, 870
edgesBegin, 877
edgesEnd, 877
end, 877
findLabel, 877
findNode, 878
generateERN, 878
generateREG, 879
generateRNC, 879
getAvgDeg, 879
getCC, 880
getCommonNeighbors, 880, 881
getCSR, 881
getDeg, 881
getDegStat, 882
getID, 882
getLabel, 882
getMaxDeg, 883
getMinDeg, 883
getNbCommonNeighbors, 883
getNbCouples, 884
getNbEdges, 884
getNbInEdges, 884
getNbNodes, 884
getNbNonEdges, 885
getNbPaths, 885
isEdge, 885, 886
Label, 870
LabelIt, 870
labelsBegin, 886
labelsEnd, 886
makeEdge, 887
neighbBegin, 887
neighbEnd, 887
NodeID, 870
NodeIt, 871
NodeMapSP, 871
nodesBegin, 888
nodesDegBegin, 888
nodesDegEnd, 888
nodesEnd, 888
NodeSMapSP, 871
nonEdgesBegin, 889
nonEdgesEnd, 889
operator=, 889, 890
print, 890
printEdges, 890
read, 890
readEdges, 891
reverseEdge, 891
rndEdgesBegin, 892
rndEdgesEnd, 892
rndNodesBegin, 892
rndNodesEnd, 893
rndNonEdgesBegin, 893
rndNonEdgesEnd, 893
shuffle, 893
start, 894
UNetwork, 871, 872
write, 894
LinkPred::UNetwork< LabelT, NodeIDT, EdgeT >::EdgeMap< ValueType >, 238
~EdgeMap, 240
at, 240
begin, 241
cbegin, 241
cend, 241
EdgeMap, 239, 240
EdgeMapConstIt, 239
EdgeMapIt, 239
end, 241
find, 242
operator=, 242, 243
operator[], 243
size, 244
LinkPred::UNetwork< LabelT, NodeIDT, EdgeT >::NodeDegIt, 484
difference_type, 485
NodeDegIt, 486
operator!=, 486
operator<, 490
operator<=, 491
operator>, 493
operator>=, 493
operator*, 487
operator+, 487
operator++, 487, 488
operator+=, 488
operator-, 488, 489
operator->, 490
operator--, 489
operator-=, 490
operator=, 491
operator==, 493
pointer, 485

reference, 485
UNetwork, 494
LinkPred::UNetwork< LabelT, NodeIDT, EdgeT >::NodeMap< ValueType >, 494
~NodeMap, 496
at, 496
begin, 497
cbegin, 497
cend, 497
end, 497
NodeMap, 495, 496
NodeMapConstIt, 495
NodeMapIt, 495
operator=, 498
operator[], 498, 499
LinkPred::UNetwork< LabelT, NodeIDT, EdgeT >::NodeSMap< ValueType >, 510
~NodeSMap, 513
at, 513
begin, 513
cbegin, 513
cend, 514
end, 514
find, 514
NodeSMap, 512
NodeSMapConstIterator, 511
NodeSMapIterator, 512
operator=, 515
operator[], 515, 516
size, 516
LinkPred::UNetwork< LabelT, NodeIDT, EdgeT >::NonEdgeIt, 517
difference_type, 518
NonEdgeIt, 519
operator!=, 520
operator<, 523
operator<=, 524
operator>, 525
operator>=, 525
operator*, 520
operator+, 520
operator++, 521
operator+=, 521
operator-, 522
operator->, 523
operator--, 522, 523
operator-=, 523
operator=, 524
operator==, 525
pointer, 518
reference, 519
UNetwork, 526
LinkPred::UNetwork< LabelT, NodeIDT, EdgeT >::RndEdgeIt, 636
operator!=, 639
operator*, 639
operator++, 640
operator->, 640
operator=, 640, 641
operator==, 641
pointer, 638
reference, 638
RndEdgeIt, 638, 639
LinkPred::UNetwork< LabelT, NodeIDT, EdgeT >::RndNodeIt, 651
operator!=, 654
operator*, 654
operator++, 654, 655
operator->, 655
operator=, 655, 656
operator==, 656
pointer, 653
reference, 653
RndNodeIt, 653, 654
LinkPred::UNetwork< LabelT, NodeIDT, EdgeT >::RndNonEdgeIt, 656
operator!=, 659
operator*, 659
operator++, 660
operator->, 660
operator=, 660, 661
operator==, 661
pointer, 658
reference, 658
RndNonEdgeIt, 658, 659
LinkPred::UPATPredictor< Network, EdgeRndIt, ScoreRndIt, EdgeRndOutIt >, 895
~UPATPredictor, 897
init, 897
learn, 897
operator=, 898
predict, 898
score, 899
top, 899
UPATPredictor, 896, 897
LinkPred::UPSTPredictor< Network, EdgeRndIt, ScoreRndIt, EdgeRndOutIt >, 900
~UPSTPredictor, 903
init, 903
learn, 903
loadEdgeScores, 903
operator=, 904
score, 904
setEdgeScores, 905
UPSTPredictor, 902
LinkPred::URALPredictor< Network, EdgeRndIt, ScoreRndIt, EdgeRndOutIt >, 905
~URALPredictor, 908
init, 908
learn, 908
operator=, 908, 909
predict, 909
score, 910
top, 910
URALPredictor, 907

LinkPred::URNDPredictor< Network, EdgeRndIt, ScoreRndIt, EdgeRndOutIt >, 911
- ~URNDPredictor, 913
- init, 913
- learn, 913
- operator=, 914
- predict, 914
- score, 915
- URNDPredictor, 912, 913

LinkPred::USAIPredictor< Network, EdgeRndIt, ScoreRndIt, EdgeRndOutIt >, 915
- ~USAIPredictor, 919
- init, 919
- learn, 919
- operator=, 919, 920
- predict, 920
- score, 921
- top, 921
- USAIPredictor, 918

LinkPred::USBMPredictor< Network, EdgeRndIt, ScoreRndIt, EdgeRndOutIt >, 922
- ~USBMPredictor, 924
- getMaxIter, 924
- init, 924
- learn, 925
- operator=, 925
- predict, 926
- score, 926
- setMaxIter, 927
- USBMPredictor, 923, 924

LinkPred::USHPPredictor< Network, EdgeRndIt, ScoreRndIt, EdgeRndOutIt >, 927
- ~USHPPredictor, 930
- getAsp, 930
- getCacheLevel, 931
- getLandmarkRatio, 931
- getLandmarkStrategy, 931
- Hub, 929
- IHub, 929
- init, 931
- LandmarkStrategy, 929
- learn, 932
- operator=, 932
- predict, 933
- Random, 929
- score, 933
- setAsp, 934
- setCacheLevel, 934
- setLandmarkRatio, 934
- setLandmarkStrategy, 935
- USHPPredictor, 929, 930

LinkPred::USOIPredictor< Network, EdgeRndIt, ScoreRndIt, EdgeRndOutIt >, 935
- ~USOIPredictor, 938
- init, 938
- learn, 938
- operator=, 938, 939
- predict, 939
- score, 939
- top, 940
- USOIPredictor, 937

LinkPred::USUMPredictor< Network, EdgeRndIt, ScoreRndIt, EdgeRndOutIt >, 941
- ~USUMPredictor, 943
- init, 943
- learn, 943
- operator=, 944
- predict, 944
- score, 945
- top, 945
- USUMPredictor, 942, 943

LinkPred::Utils, 47
- assertNoNaN, 48
- clear, 49
- filter, 49
- flip, 49
- flipMap, 50
- getRandom, 50
- getRndPerm, 50
- int_cast, 51
- localRange, 51
- ltrim, 51
- norm, 52
- plFit, 52
- print, 53
- printEdges, 53, 54
- readEdges, 54
- readEdgeScores, 54
- rtrim, 55
- selectRandom, 55
- selectRandomInPlace, 55
- selectTopK, 56
- shuffle, 56
- sort, 56
- trim, 57
- writeEdgeScores, 57

LinkPred::Utils::EdgeScore< Label >, 251
- i, 251
- j, 251
- score, 251

LinkPred::Utils::PairCompRight< FirstT, SecondT, CompareT >, 536
- operator(), 536

LinkPred::Vec, 946
- ~Vec, 950
- amax, 950
- amin, 951
- axpy, 951
- center, 952
- find, 952
- findNz, 952
- getValues, 952
- ifmax, 953
- ifmin, 953
- ilmax, 953
- ilmin, 953

imax, 953
imin, 954
isConstant, 954
makeConstant, 954
max, 954, 955
mean, 955
min, 955, 956
norm, 956
operator!=, 957
operator∗, 963–965
operator^, 969
operator+, 965, 966
operator-, 966, 967
operator/, 967, 968
operator=, 957
operator==, 957
operator[], 958
partScale, 959
print, 959, 960
read, 960
reset, 960, 961
resize, 961
scale, 961
scatter, 962
setConstant, 962
size, 962
sum, 962
Vec, 948–950
write, 963
LMapQueue
LinkPred::LMapQueue< K, P, KComparator, PComparator >, 385, 386
loadEdgeScores
LinkPred::DPSTPredictor< Network, EdgeRndIt, ScoreRndIt, EdgeRndOutIt >, 224
LinkPred::UPSTPredictor< Network, EdgeRndIt, ScoreRndIt, EdgeRndOutIt >, 903
loadnet
LinkPred::Simp::Predictor, 583
loadTestData
LinkPred::NetworkManipulator< Network >, 460
loadTestDataAdd
LinkPred::NetworkManipulator< Network >, 462
loadTestDataRem
LinkPred::NetworkManipulator< Network >, 462
localRange
LinkPred::Utils, 51
lock
LinkPred::TestData< Network, EdgeContT >, 701
Log
LinkPred::Log, 394
log.hpp
logger, 1128
logDebug
LinkPred, 40
logDebug1
LinkPred, 40
logDebug2
LinkPred, 40
logDebug3
LinkPred, 40
logError
LinkPred, 40
logger
log.hpp, 1128
logInfo
LinkPred, 40
LogisticRegresser
LinkPred::LogisticRegresser< InRndIt, OutRndIt, ScoreRndIt >, 397, 398
LogLevel
LinkPred, 39
logLevel
LinkPred::Log, 395
LogMDSCG
LinkPred::LogMDSCG, 402, 403
LogRegCG
LinkPred::LogRegCG< InRndIt, OutRndIt >, 408
logWarning
LinkPred, 40
LPSim
LinkPred::LPSim, 413, 414
ltrim
LinkPred::Utils, 51

m
LinkPred::Simp::Evaluator::Factory::HYPParams, 360
Machine learning classes, 25
makeConstant
LinkPred::Vec, 954
makeEdge
LinkPred::DNetwork< LabelT, NodeIDT, EdgeT >, 203
LinkPred::UNetwork< LabelT, NodeIDT, EdgeT >, 887
MatFact
LinkPred::MatFact< Network >, 418
MatFactCG
LinkPred::MatFactCG, 424
MathPI
LinkPred, 46
max
LinkPred::Vec, 954, 955
maxIter
LinkPred::Simp::Evaluator::Factory::FBMParams, 324
LinkPred::Simp::Evaluator::Factory::SBMParams, 677
MDS
LinkPred::MDS, 429
MDSAlg
LinkPred, 40
mean
LinkPred::Vec, 955
MeanApp

LinkPred::UMPSPredictor< Network, EdgeRndIt, ScoreRndIt, EdgeRndOutIt >, 846
merge
LinkPred::LMapQueue< K, P, KComparator, PComparator >, 390
min
LinkPred::Vec, 955, 956
mult
LinkPred::GFMatrix, 337, 338

name
LinkPred::Simp::PerfRes, 568
nbBeans
LinkPred::Simp::Evaluator::Factory::HRGParams, 358
nbSamples
LinkPred::Simp::Evaluator::Factory::HRGParams, 359
nbTestRuns
LinkPred::PerfeEvalExpDescp< Network >, 548
negBegin
LinkPred::PredResults< TestDataT, LPredictorT, ScoresContainerT >, 619
LinkPred::TestData< Network, EdgeContT >, 701
negEnd
LinkPred::PredResults< TestDataT, LPredictorT, ScoresContainerT >, 620
LinkPred::TestData< Network, EdgeContT >, 701
negRatio
LinkPred::Simp::Evaluator::Factory::ECLParams, 237
negStrmBegin
LinkPred::PredResults< TestDataT, LPredictorT, ScoresContainerT >, 620
LinkPred::TestData< Network, EdgeContT >, 701
negStrmEnd
LinkPred::PredResults< TestDataT, LPredictorT, ScoresContainerT >, 620
LinkPred::TestData< Network, EdgeContT >, 702
neighbBegin
LinkPred::DNetwork< LabelT, NodeIDT, EdgeT >, 204
LinkPred::UNetwork< LabelT, NodeIDT, EdgeT >, 887
neighbEnd
LinkPred::DNetwork< LabelT, NodeIDT, EdgeT >, 204
LinkPred::UNetwork< LabelT, NodeIDT, EdgeT >, 887
Network
LinkPred::DLPredictor< NetworkT, EdgeRndItT, ScoreRndItT, EdgeRndOutItT >, 176
LinkPred::ULPredictor< NetworkT, EdgeRndItT, ScoreRndItT, EdgeRndOutItT >, 836
NetworkCache
LinkPred, 39
NetworkCSP
LinkPred::NetworkManipulator< Network >, 452
LinkPred::TestData< Network, EdgeContT >, 695
NetworkManipulator
LinkPred::NetworkManipulator< Network >, 453
NetworkSP
LinkPred::ASPDistCalculator< Network, DistType, NbHopsType >, 62
LinkPred::ASPDsimCalculator< Network, DsimType, NbHopsType >, 68
LinkPred::DESPLDistCalculator< Network, DistType, NbHopsType >, 124
LinkPred::Dijkstra< Network, DistType, NbHopsType >, 147
LinkPred::ESPDistCalculator< Network, DistType, NbHopsType >, 265
LinkPred::ESPDsimCalculator< Network, DsimType, NbHopsType >, 273
LinkPred::ESPIndSimlCalculator< Network, DistType, NbHopsType >, 280
LinkPred::ESPLDistCalculator< Network, DistType, NbHopsType >, 286
LinkPred::ESPLSimlCalculator< Network, DistType, NbHopsType >, 295
LinkPred::ESPSimlCalculator< Network, DistType, NbHopsType >, 305
LinkPred::NetDistCalculator< Network, DistType, NbHopsType >, 434
LinkPred::NetIndSimlCalculator< Network, DistType, NbHopsType >, 440
LinkPred::NetSimlCalculator< Network, DistType, NbHopsType >, 445
LinkPred::NetworkManipulator< Network >, 452
LinkPred::TestData< Network, EdgeContT >, 695
NoCache
LinkPred, 39
Node2Vec
LinkPred::Node2Vec< Network >, 466, 467
NodeCache
LinkPred, 39
NodeDegIt
LinkPred::DNetwork< LabelT, NodeIDT, EdgeT >::NodeDegIt, 476
LinkPred::UNetwork< LabelT, NodeIDT, EdgeT >::NodeDegIt, 486
NodeDistMap
LinkPred::ASPDistCalculator< Network, DistType, NbHopsType >, 62
LinkPred::DESPLDistCalculator< Network, DistType, NbHopsType >, 124
LinkPred::Dijkstra< Network, DistType, NbHopsType >, 147
LinkPred::ESPDistCalculator< Network, DistType, NbHopsType >, 265
LinkPred::ESPIndSimlCalculator< Network, DistType, NbHopsType >, 280
LinkPred::ESPLDistCalculator< Network, DistType, NbHopsType >, 286
LinkPred::ESPSimlCalculator< Network, DistType, NbHopsType >, 305
LinkPred::NetDistCalculator< Network, DistType,

NbHopsType >, 434
NodeDistMapSP
LinkPred::ASPDistCalculator< Network, DistType, NbHopsType >, 62
LinkPred::DESPLDistCalculator< Network, DistType, NbHopsType >, 124
LinkPred::Dijkstra< Network, DistType, NbHopsType >, 147
LinkPred::ESPDistCalculator< Network, DistType, NbHopsType >, 265
LinkPred::ESPIndSimlCalculator< Network, DistType, NbHopsType >, 280
LinkPred::ESPLDistCalculator< Network, DistType, NbHopsType >, 287
LinkPred::ESPSimlCalculator< Network, DistType, NbHopsType >, 305
LinkPred::NetDistCalculator< Network, DistType, NbHopsType >, 435
NodeDsimMap
LinkPred::ASPDsimCalculator< Network, DsimType, NbHopsType >, 68
LinkPred::ESPDsimCalculator< Network, DsimType, NbHopsType >, 273
NodeDsimMapSP
LinkPred::ASPDsimCalculator< Network, DsimType, NbHopsType >, 69
LinkPred::ESPDsimCalculator< Network, DsimType, NbHopsType >, 273
NodeID
LinkPred::ASPDistCalculator< Network, DistType, NbHopsType >, 62
LinkPred::ASPDsimCalculator< Network, DsimType, NbHopsType >, 69
LinkPred::DESPLDistCalculator< Network, DistType, NbHopsType >, 125
LinkPred::Dijkstra< Network, DistType, NbHopsType >, 148
LinkPred::DLPredictor< NetworkT, EdgeRndItT, ScoreRndItT, EdgeRndOutItT >, 176
LinkPred::DNetwork< LabelT, NodeIDT, EdgeT >, 186
LinkPred::Encoder< Network >, 255
LinkPred::ESPDistCalculator< Network, DistType, NbHopsType >, 265
LinkPred::ESPDsimCalculator< Network, DsimType, NbHopsType >, 273
LinkPred::ESPIndSimlCalculator< Network, DistType, NbHopsType >, 280
LinkPred::ESPLDistCalculator< Network, DistType, NbHopsType >, 287
LinkPred::ESPLSimlCalculator< Network, DistType, NbHopsType >, 296
LinkPred::ESPSimlCalculator< Network, DistType, NbHopsType >, 305
LinkPred::NetDistCalculator< Network, DistType, NbHopsType >, 435
LinkPred::NetIndSimlCalculator< Network, DistType, NbHopsType >, 440
LinkPred::NetSimlCalculator< Network, DistType, NbHopsType >, 445
LinkPred::NetworkManipulator< Network >, 452
LinkPred::UKABPredictor< Network, EdgeRndIt, ScoreRndIt, EdgeRndOutIt >, 818
LinkPred::ULPredictor< NetworkT, EdgeRndItT, ScoreRndItT, EdgeRndOutItT >, 837
LinkPred::UNEDPredictor< Network, EdgeRndIt, ScoreRndIt, EdgeRndOutIt >, 862
LinkPred::UNetwork< LabelT, NodeIDT, EdgeT >, 870
NodeIt
LinkPred::DNetwork< LabelT, NodeIDT, EdgeT >, 186
LinkPred::UNetwork< LabelT, NodeIDT, EdgeT >, 871
NodeMap
LinkPred::DNetwork< LabelT, NodeIDT, EdgeT >::NodeMap< ValueType >, 501
LinkPred::NetworkManipulator< Network >, 453
LinkPred::UNetwork< LabelT, NodeIDT, EdgeT >::NodeMap< ValueType >, 495, 496
NodeMapConstIt
LinkPred::DNetwork< LabelT, NodeIDT, EdgeT >::NodeMap< ValueType >, 500
LinkPred::UNetwork< LabelT, NodeIDT, EdgeT >::NodeMap< ValueType >, 495
NodeMapIt
LinkPred::DNetwork< LabelT, NodeIDT, EdgeT >::NodeMap< ValueType >, 500
LinkPred::UNetwork< LabelT, NodeIDT, EdgeT >::NodeMap< ValueType >, 495
NodeMapSP
LinkPred::DNetwork< LabelT, NodeIDT, EdgeT >, 186
LinkPred::UNetwork< LabelT, NodeIDT, EdgeT >, 871
nodesBegin
LinkPred::DNetwork< LabelT, NodeIDT, EdgeT >, 204
LinkPred::UNetwork< LabelT, NodeIDT, EdgeT >, 888
nodesDegBegin
LinkPred::DNetwork< LabelT, NodeIDT, EdgeT >, 205
LinkPred::UNetwork< LabelT, NodeIDT, EdgeT >, 888
nodesDegEnd
LinkPred::DNetwork< LabelT, NodeIDT, EdgeT >, 205
LinkPred::UNetwork< LabelT, NodeIDT, EdgeT >, 888
NodeSDistMap
LinkPred::DESPLDistCalculator< Network, DistType, NbHopsType >, 125
LinkPred::ESPLDistCalculator< Network, DistType, NbHopsType >, 287
LinkPred::ESPLSimlCalculator< Network, Dist-

Type, NbHopsType >, 296
LinkPred::NetSimlCalculator< Network, DistType, NbHopsType >, 446

NodeSDistMapSP
LinkPred::DESPLDistCalculator< Network, DistType, NbHopsType >, 125
LinkPred::Dijkstra< Network, DistType, NbHopsType >, 148
LinkPred::ESPLDistCalculator< Network, DistType, NbHopsType >, 287
LinkPred::ESPLSimlCalculator< Network, DistType, NbHopsType >, 296
LinkPred::NetSimlCalculator< Network, DistType, NbHopsType >, 446

nodesEnd
LinkPred::DNetwork< LabelT, NodeIDT, EdgeT >, 205
LinkPred::UNetwork< LabelT, NodeIDT, EdgeT >, 888

NodeSMap
LinkPred::DNetwork< LabelT, NodeIDT, EdgeT >::NodeSMap< ValueType >, 506
LinkPred::UNetwork< LabelT, NodeIDT, EdgeT >::NodeSMap< ValueType >, 512

NodeSMapConstIterator
LinkPred::DNetwork< LabelT, NodeIDT, EdgeT >::NodeSMap< ValueType >, 506
LinkPred::UNetwork< LabelT, NodeIDT, EdgeT >::NodeSMap< ValueType >, 511

NodeSMapIterator
LinkPred::DNetwork< LabelT, NodeIDT, EdgeT >::NodeSMap< ValueType >, 506
LinkPred::UNetwork< LabelT, NodeIDT, EdgeT >::NodeSMap< ValueType >, 512

NodeSMapSP
LinkPred::DNetwork< LabelT, NodeIDT, EdgeT >, 187
LinkPred::UNetwork< LabelT, NodeIDT, EdgeT >, 871

None
LinkPred, 40

NonEdgeIt
LinkPred::DNetwork< LabelT, NodeIDT, EdgeT >::NonEdgeIt, 528, 529
LinkPred::TestData< Network, EdgeContT >, 695
LinkPred::UNetwork< LabelT, NodeIDT, EdgeT >::NonEdgeIt, 519

nonEdgesBegin
LinkPred::DNetwork< LabelT, NodeIDT, EdgeT >, 205
LinkPred::UNetwork< LabelT, NodeIDT, EdgeT >, 889

nonEdgesEnd
LinkPred::DNetwork< LabelT, NodeIDT, EdgeT >, 205
LinkPred::UNetwork< LabelT, NodeIDT, EdgeT >, 889

norm
LinkPred::Utils, 52
LinkPred::Vec, 956

npv
perfmeasure.hpp, 1053

Numerical classes and algorithms, 26

operator!=
LinkPred::DNetwork< LabelT, NodeIDT, EdgeT >::NodeDegIt, 477
LinkPred::DNetwork< LabelT, NodeIDT, EdgeT >::NonEdgeIt, 529
LinkPred::DNetwork< LabelT, NodeIDT, EdgeT >::RndEdgeIt, 644
LinkPred::DNetwork< LabelT, NodeIDT, EdgeT >::RndNodeIt, 649
LinkPred::DNetwork< LabelT, NodeIDT, EdgeT >::RndNonEdgeIt, 664
LinkPred::GFMatrix, 338
LinkPred::PredResults< TestDataT, LPredictorT, ScoresContainerT >::ScoreIterator, 682
LinkPred::TestData< Network, EdgeContT >::TestEdgeIt, 712
LinkPred::TestEdgeGen< Network, EdgeContT >::TNEdgeIt, 722
LinkPred::TestEdgeGen< Network, EdgeContT >::TPEdgeIt, 732
LinkPred::UNetwork< LabelT, NodeIDT, EdgeT >::NodeDegIt, 486
LinkPred::UNetwork< LabelT, NodeIDT, EdgeT >::NonEdgeIt, 520
LinkPred::UNetwork< LabelT, NodeIDT, EdgeT >::RndEdgeIt, 639
LinkPred::UNetwork< LabelT, NodeIDT, EdgeT >::RndNodeIt, 654
LinkPred::UNetwork< LabelT, NodeIDT, EdgeT >::RndNonEdgeIt, 659
LinkPred::Vec, 957

operator<
LinkPred::DNetwork< LabelT, NodeIDT, EdgeT >::NodeDegIt, 481
LinkPred::DNetwork< LabelT, NodeIDT, EdgeT >::NonEdgeIt, 533
LinkPred::PredResults< TestDataT, LPredictorT, ScoresContainerT >::ScoreIterator, 686
LinkPred::TestData< Network, EdgeContT >::TestEdgeIt, 716
LinkPred::TestEdgeGen< Network, EdgeContT >::TNEdgeIt, 726
LinkPred::TestEdgeGen< Network, EdgeContT >::TPEdgeIt, 735
LinkPred::UNetwork< LabelT, NodeIDT, EdgeT >::NodeDegIt, 490
LinkPred::UNetwork< LabelT, NodeIDT, EdgeT >::NonEdgeIt, 523

operator<<
LinkPred::Log, 394

operator<=
LinkPred::DNetwork< LabelT, NodeIDT, EdgeT >::NodeDegIt, 481

LinkPred::DNetwork< LabelT, NodeIDT, EdgeT >::NonEdgeIt, 533
LinkPred::PredResults< TestDataT, LPredictorT, ScoresContainerT >::ScoreIterator, 686
LinkPred::TestData< Network, EdgeContT >::TestEdgeIt, 716
LinkPred::TestEdgeGen< Network, EdgeContT >::TNEdgeIt, 726
LinkPred::TestEdgeGen< Network, EdgeContT >::TPEdgeIt, 736
LinkPred::UNetwork< LabelT, NodeIDT, EdgeT >::NodeDegIt, 491
LinkPred::UNetwork< LabelT, NodeIDT, EdgeT >::NonEdgeIt, 524
operator>
LinkPred::DNetwork< LabelT, NodeIDT, EdgeT >::NodeDegIt, 482
LinkPred::DNetwork< LabelT, NodeIDT, EdgeT >::NonEdgeIt, 535
LinkPred::PredResults< TestDataT, LPredictorT, ScoresContainerT >::ScoreIterator, 688
LinkPred::TestData< Network, EdgeContT >::TestEdgeIt, 718
LinkPred::TestEdgeGen< Network, EdgeContT >::TNEdgeIt, 728
LinkPred::TestEdgeGen< Network, EdgeContT >::TPEdgeIt, 737
LinkPred::UNetwork< LabelT, NodeIDT, EdgeT >::NodeDegIt, 493
LinkPred::UNetwork< LabelT, NodeIDT, EdgeT >::NonEdgeIt, 525
operator>=
LinkPred::DNetwork< LabelT, NodeIDT, EdgeT >::NodeDegIt, 483
LinkPred::DNetwork< LabelT, NodeIDT, EdgeT >::NonEdgeIt, 535
LinkPred::PredResults< TestDataT, LPredictorT, ScoresContainerT >::ScoreIterator, 688
LinkPred::TestData< Network, EdgeContT >::TestEdgeIt, 718
LinkPred::TestEdgeGen< Network, EdgeContT >::TNEdgeIt, 728
LinkPred::TestEdgeGen< Network, EdgeContT >::TPEdgeIt, 737
LinkPred::UNetwork< LabelT, NodeIDT, EdgeT >::NodeDegIt, 493
LinkPred::UNetwork< LabelT, NodeIDT, EdgeT >::NonEdgeIt, 525
operator*
LinkPred, 40, 42
LinkPred::DNetwork< LabelT, NodeIDT, EdgeT >::NodeDegIt, 477
LinkPred::DNetwork< LabelT, NodeIDT, EdgeT >::NonEdgeIt, 529
LinkPred::DNetwork< LabelT, NodeIDT, EdgeT >::RndEdgeIt, 644
LinkPred::DNetwork< LabelT, NodeIDT, EdgeT >::RndNodeIt, 649
LinkPred::DNetwork< LabelT, NodeIDT, EdgeT >::RndNonEdgeIt, 664
LinkPred::GFMatrix, 342, 343
LinkPred::PredResults< TestDataT, LPredictorT, ScoresContainerT >::ScoreIterator, 682
LinkPred::TestData< Network, EdgeContT >::TestEdgeIt, 713
LinkPred::TestEdgeGen< Network, EdgeContT >::TNEdgeIt, 722
LinkPred::TestEdgeGen< Network, EdgeContT >::TPEdgeIt, 732
LinkPred::UNetwork< LabelT, NodeIDT, EdgeT >::NodeDegIt, 487
LinkPred::UNetwork< LabelT, NodeIDT, EdgeT >::NonEdgeIt, 520
LinkPred::UNetwork< LabelT, NodeIDT, EdgeT >::RndEdgeIt, 639
LinkPred::UNetwork< LabelT, NodeIDT, EdgeT >::RndNodeIt, 654
LinkPred::UNetwork< LabelT, NodeIDT, EdgeT >::RndNonEdgeIt, 659
LinkPred::Vec, 963–965
operator^
LinkPred, 46
LinkPred::Vec, 969
operator()
LinkPred::FastSig< T >, 323
LinkPred::Utils::PairCompRight< FirstT, SecondT, CompareT >, 536
operator+
LinkPred, 42, 43
LinkPred::DNetwork< LabelT, NodeIDT, EdgeT >::NodeDegIt, 477
LinkPred::DNetwork< LabelT, NodeIDT, EdgeT >::NonEdgeIt, 530
LinkPred::GFMatrix, 344, 345
LinkPred::PredResults< TestDataT, LPredictorT, ScoresContainerT >::ScoreIterator, 683
LinkPred::TestData< Network, EdgeContT >::TestEdgeIt, 713
LinkPred::TestEdgeGen< Network, EdgeContT >::TNEdgeIt, 723
LinkPred::TestEdgeGen< Network, EdgeContT >::TPEdgeIt, 732
LinkPred::UNetwork< LabelT, NodeIDT, EdgeT >::NodeDegIt, 487
LinkPred::UNetwork< LabelT, NodeIDT, EdgeT >::NonEdgeIt, 520
LinkPred::Vec, 965, 966
operator++
LinkPred::DNetwork< LabelT, NodeIDT, EdgeT >::NodeDegIt, 478
LinkPred::DNetwork< LabelT, NodeIDT, EdgeT >::NonEdgeIt, 530
LinkPred::DNetwork< LabelT, NodeIDT, EdgeT >::RndEdgeIt, 645
LinkPred::DNetwork< LabelT, NodeIDT, EdgeT >::RndNodeIt, 649, 650

LinkPred::DNetwork< LabelT, NodeIDT, EdgeT >::RndNonEdgeIt, 665
LinkPred::PredResults< TestDataT, LPredictorT, ScoresContainerT >::ScoreIterator, 683
LinkPred::TestData< Network, EdgeContT >::TestEdgeIt, 713, 714
LinkPred::TestEdgeGen< Network, EdgeContT >::TNEdgeIt, 723
LinkPred::TestEdgeGen< Network, EdgeContT >::TPEdgeIt, 733
LinkPred::UNetwork< LabelT, NodeIDT, EdgeT >::NodeDegIt, 487, 488
LinkPred::UNetwork< LabelT, NodeIDT, EdgeT >::NonEdgeIt, 521
LinkPred::UNetwork< LabelT, NodeIDT, EdgeT >::RndEdgeIt, 640
LinkPred::UNetwork< LabelT, NodeIDT, EdgeT >::RndNodeIt, 654, 655
LinkPred::UNetwork< LabelT, NodeIDT, EdgeT >::RndNonEdgeIt, 660

operator+=
LinkPred::DNetwork< LabelT, NodeIDT, EdgeT >::NodeDegIt, 478
LinkPred::DNetwork< LabelT, NodeIDT, EdgeT >::NonEdgeIt, 530
LinkPred::PredResults< TestDataT, LPredictorT, ScoresContainerT >::ScoreIterator, 683
LinkPred::TestData< Network, EdgeContT >::TestEdgeIt, 714
LinkPred::TestEdgeGen< Network, EdgeContT >::TNEdgeIt, 723
LinkPred::TestEdgeGen< Network, EdgeContT >::TPEdgeIt, 733
LinkPred::UNetwork< LabelT, NodeIDT, EdgeT >::NodeDegIt, 488
LinkPred::UNetwork< LabelT, NodeIDT, EdgeT >::NonEdgeIt, 521

operator-
LinkPred, 44
LinkPred::DNetwork< LabelT, NodeIDT, EdgeT >::NodeDegIt, 479
LinkPred::DNetwork< LabelT, NodeIDT, EdgeT >::NonEdgeIt, 531
LinkPred::GFMatrix, 345–347
LinkPred::PredResults< TestDataT, LPredictorT, ScoresContainerT >::ScoreIterator, 684
LinkPred::TestData< Network, EdgeContT >::TestEdgeIt, 714, 715
LinkPred::TestEdgeGen< Network, EdgeContT >::TNEdgeIt, 724
LinkPred::TestEdgeGen< Network, EdgeContT >::TPEdgeIt, 734
LinkPred::UNetwork< LabelT, NodeIDT, EdgeT >::NodeDegIt, 488, 489
LinkPred::UNetwork< LabelT, NodeIDT, EdgeT >::NonEdgeIt, 522
LinkPred::Vec, 966, 967

operator->
LinkPred::DNetwork< LabelT, NodeIDT, EdgeT >::NodeDegIt, 480
LinkPred::DNetwork< LabelT, NodeIDT, EdgeT >::NonEdgeIt, 533
LinkPred::DNetwork< LabelT, NodeIDT, EdgeT >::RndEdgeIt, 645
LinkPred::DNetwork< LabelT, NodeIDT, EdgeT >::RndNodeIt, 650
LinkPred::DNetwork< LabelT, NodeIDT, EdgeT >::RndNonEdgeIt, 665
LinkPred::PredResults< TestDataT, LPredictorT, ScoresContainerT >::ScoreIterator, 686
LinkPred::TestData< Network, EdgeContT >::TestEdgeIt, 716
LinkPred::TestEdgeGen< Network, EdgeContT >::TNEdgeIt, 726
LinkPred::TestEdgeGen< Network, EdgeContT >::TPEdgeIt, 735
LinkPred::UNetwork< LabelT, NodeIDT, EdgeT >::NodeDegIt, 490
LinkPred::UNetwork< LabelT, NodeIDT, EdgeT >::NonEdgeIt, 523
LinkPred::UNetwork< LabelT, NodeIDT, EdgeT >::RndEdgeIt, 640
LinkPred::UNetwork< LabelT, NodeIDT, EdgeT >::RndNodeIt, 655
LinkPred::UNetwork< LabelT, NodeIDT, EdgeT >::RndNonEdgeIt, 660

operator--
LinkPred::DNetwork< LabelT, NodeIDT, EdgeT >::NodeDegIt, 479, 480
LinkPred::DNetwork< LabelT, NodeIDT, EdgeT >::NonEdgeIt, 532
LinkPred::PredResults< TestDataT, LPredictorT, ScoresContainerT >::ScoreIterator, 685
LinkPred::TestData< Network, EdgeContT >::TestEdgeIt, 715
LinkPred::TestEdgeGen< Network, EdgeContT >::TNEdgeIt, 725
LinkPred::TestEdgeGen< Network, EdgeContT >::TPEdgeIt, 734, 735
LinkPred::UNetwork< LabelT, NodeIDT, EdgeT >::NodeDegIt, 489
LinkPred::UNetwork< LabelT, NodeIDT, EdgeT >::NonEdgeIt, 522, 523

operator-=
LinkPred::DNetwork< LabelT, NodeIDT, EdgeT >::NodeDegIt, 480
LinkPred::DNetwork< LabelT, NodeIDT, EdgeT >::NonEdgeIt, 532
LinkPred::PredResults< TestDataT, LPredictorT, ScoresContainerT >::ScoreIterator, 685
LinkPred::TestData< Network, EdgeContT >::TestEdgeIt, 715
LinkPred::TestEdgeGen< Network, EdgeContT >::TNEdgeIt, 725
LinkPred::TestEdgeGen< Network, EdgeContT >::TPEdgeIt, 735

LinkPred::UNetwork< LabelT, NodeIDT, EdgeT >::NodeDegIt, 490
LinkPred::UNetwork< LabelT, NodeIDT, EdgeT >::NonEdgeIt, 523
operator/
LinkPred, 45
LinkPred::GFMatrix, 347
LinkPred::Vec, 967, 968
operator=
LinkPred::ASPDistCalculator< Network, DistType, NbHopsType >, 65
LinkPred::ASPDsimCalculator< Network, DsimType, NbHopsType >, 71, 72
LinkPred::BFS< Network, NodeProcessor >, 75
LinkPred::BHeap< T, P, ComparatorT, ComparatorP >, 80
LinkPred::Bhmap< K, P, Comparator >, 87
LinkPred::Classifier< InRndIt, OutRndIt, ScoreRndIt >, 91
LinkPred::Collector< Network >, 94, 95
LinkPred::CosineSim, 97, 98
LinkPred::Counter< Network >, 100, 101
LinkPred::DADAPredictor< Network, EdgeRndIt, ScoreRndIt, EdgeRndOutIt >, 105
LinkPred::DCNEPredictor< Network, EdgeRndIt, ScoreRndIt, EdgeRndOutIt >, 110, 111
LinkPred::DeepWalk< Network >, 118, 119
LinkPred::DESPLDistCalculator< Network, DistType, NbHopsType >, 129, 130
LinkPred::DFS< Network, NodeProcessor >, 133
LinkPred::DHDIPredictor< Network, EdgeRndIt, ScoreRndIt, EdgeRndOutIt >, 137
LinkPred::DHPIPredictor< Network, EdgeRndIt, ScoreRndIt, EdgeRndOutIt >, 142, 143
LinkPred::Dijkstra< Network, DistType, NbHopsType >, 156, 157
LinkPred::DJIDPredictor< Network, EdgeRndIt, ScoreRndIt, EdgeRndOutIt >, 161, 162
LinkPred::DLCPPredictor< Network, EdgeRndIt, ScoreRndIt, EdgeRndOutIt >, 167
LinkPred::DLHNPredictor< Network, EdgeRndIt, ScoreRndIt, EdgeRndOutIt >, 172
LinkPred::DLPredictor< NetworkT, EdgeRndItT, ScoreRndItT, EdgeRndOutItT >, 179, 180
LinkPred::DNetwork< LabelT, NodeIDT, EdgeT >, 206
LinkPred::DNetwork< LabelT, NodeIDT, EdgeT >::EdgeMap< ValueType >, 249
LinkPred::DNetwork< LabelT, NodeIDT, EdgeT >::NodeDegIt, 481, 482
LinkPred::DNetwork< LabelT, NodeIDT, EdgeT >::NodeMap< ValueType >, 503
LinkPred::DNetwork< LabelT, NodeIDT, EdgeT >::NodeSMap< ValueType >, 509
LinkPred::DNetwork< LabelT, NodeIDT, EdgeT >::NonEdgeIt, 534
LinkPred::DNetwork< LabelT, NodeIDT, EdgeT >::RndEdgeIt, 645, 646
LinkPred::DNetwork< LabelT, NodeIDT, EdgeT >::RndNodeIt, 650, 651
LinkPred::DNetwork< LabelT, NodeIDT, EdgeT >::RndNonEdgeIt, 665, 666
LinkPred::DotProd, 214, 215
LinkPred::DPATPredictor< Network, EdgeRndIt, ScoreRndIt, EdgeRndOutIt >, 219
LinkPred::DPSTPredictor< Network, EdgeRndIt, ScoreRndIt, EdgeRndOutIt >, 224
LinkPred::DSAIPredictor< Network, EdgeRndIt, ScoreRndIt, EdgeRndOutIt >, 229
LinkPred::DSOIPredictor< Network, EdgeRndIt, ScoreRndIt, EdgeRndOutIt >, 234, 235
LinkPred::Encoder< Network >, 259, 260
LinkPred::ESPDistCalculator< Network, DistType, NbHopsType >, 269, 270
LinkPred::ESPDsimCalculator< Network, DsimType, NbHopsType >, 277
LinkPred::ESPIndSimlCalculator< Network, DistType, NbHopsType >, 283
LinkPred::ESPLDistCalculator< Network, DistType, NbHopsType >, 292
LinkPred::ESPLSimlCalculator< Network, DistType, NbHopsType >, 301
LinkPred::ESPSimlCalculator< Network, DistType, NbHopsType >, 309
LinkPred::FastSig< T >, 323
LinkPred::GCurve< PredResultsT >, 331, 332
LinkPred::GFMatrix, 338, 339
LinkPred::GraphTraversal< Network, NodeProcessor >, 350
LinkPred::HMSM< Network >, 355, 356
LinkPred::L1Sim, 363
LinkPred::L2Sim, 366
LinkPred::LargeVis< Network >, 372
LinkPred::LINE< Network >, 382
LinkPred::LMapQueue< K, P, KComparator, PComparator >, 390, 391
LinkPred::LogisticRegresser< InRndIt, OutRndIt, ScoreRndIt >, 399, 400
LinkPred::LogMDSCG, 405, 406
LinkPred::LogRegCG< InRndIt, OutRndIt >, 411, 412
LinkPred::LPSim, 415
LinkPred::MatFact< Network >, 420, 421
LinkPred::MatFactCG, 427
LinkPred::Node2Vec< Network >, 470, 471
LinkPred::Pearson, 539
LinkPred::PerfCurve< PredResultsT >, 546, 547
LinkPred::PerfEvalExp< Network, TestDataT, LPredictorT, PredResultsT, PerfMeasureT >, 552, 553
LinkPred::PerfEvaluator< TestDataT, LPredictorT, PredResultsT, PerfMeasureT >, 560, 561
LinkPred::PerfMeasure< PredResultsT >, 566
LinkPred::PR< PredResultsT >, 577
LinkPred::PredResults< TestDataT, LPredictorT, ScoresContainerT >, 620, 622

LinkPred::PredResults< TestDataT, LPredictorT, ScoresContainerT >::ScoreIterator, 687
LinkPred::RandomGen, 631
LinkPred::RndClassifier< InRndIt, OutRndIt, ScoreRndIt >, 635, 636
LinkPred::ROC< PredResultsT >, 675, 676
LinkPred::SimMeasure, 691, 692
LinkPred::TestData< Network, EdgeContT >, 702
LinkPred::TestData< Network, EdgeContT >::TestEdgeIt, 717
LinkPred::TestEdgeGen< Network, EdgeContT >, 707
LinkPred::TestEdgeGen< Network, EdgeContT >::TNEdgeIt, 727
LinkPred::TestEdgeGen< Network, EdgeContT >::TPEdgeIt, 736
LinkPred::TPR< PredResultsT >, 744
LinkPred::UADAPredictor< Network, EdgeRndIt, ScoreRndIt, EdgeRndOutIt >, 748, 749
LinkPred::UCNEPredictor< Network, EdgeRndIt, ScoreRndIt, EdgeRndOutIt >, 754
LinkPred::UCRAPredictor< Network, EdgeRndIt, ScoreRndIt, EdgeRndOutIt >, 759, 760
LinkPred::UCSTPredictor< Network, EdgeRndIt, ScoreRndIt, EdgeRndOutIt >, 766
LinkPred::UECLPredictor< Network, EdgeRndIt, ScoreRndIt, EdgeRndOutIt >, 772, 773
LinkPred::UESMPredictor< Network, EdgeRndIt, ScoreRndIt, EdgeRndOutIt >, 779
LinkPred::UFBMPredictor< Network, EdgeRndIt, ScoreRndIt, EdgeRndOutIt >, 784, 785
LinkPred::UHDIPredictor< Network, EdgeRndIt, ScoreRndIt, EdgeRndOutIt >, 789, 790
LinkPred::UHPIPredictor< Network, EdgeRndIt, ScoreRndIt, EdgeRndOutIt >, 795
LinkPred::UHRGPredictor< Network, EdgeRndIt, ScoreRndIt, EdgeRndOutIt >, 801
LinkPred::UHYPPredictor< Network, EdgeRndIt, ScoreRndIt, EdgeRndOutIt >, 808
LinkPred::UJIDPredictor< Network, EdgeRndIt, ScoreRndIt, EdgeRndOutIt >, 814, 815
LinkPred::UKABPredictor< Network, EdgeRndIt, ScoreRndIt, EdgeRndOutIt >, 821
LinkPred::ULCPPredictor< Network, EdgeRndIt, ScoreRndIt, EdgeRndOutIt >, 827, 828
LinkPred::ULHNPredictor< Network, EdgeRndIt, ScoreRndIt, EdgeRndOutIt >, 832, 833
LinkPred::ULPredictor< NetworkT, EdgeRndItT, ScoreRndItT, EdgeRndOutItT >, 840
LinkPred::UMPSPredictor< Network, EdgeRndIt, ScoreRndIt, EdgeRndOutIt >, 853
LinkPred::UNEDPredictor< Network, EdgeRndIt, ScoreRndIt, EdgeRndOutIt >, 864, 865
LinkPred::UNetwork< LabelT, NodeIDT, EdgeT >, 889, 890
LinkPred::UNetwork< LabelT, NodeIDT, EdgeT >::EdgeMap< ValueType >, 242, 243
LinkPred::UNetwork< LabelT, NodeIDT, EdgeT >::NodeDegIt, 491
LinkPred::UNetwork< LabelT, NodeIDT, EdgeT >::NodeMap< ValueType >, 498
LinkPred::UNetwork< LabelT, NodeIDT, EdgeT >::NodeSMap< ValueType >, 515
LinkPred::UNetwork< LabelT, NodeIDT, EdgeT >::NonEdgeIt, 524
LinkPred::UNetwork< LabelT, NodeIDT, EdgeT >::RndEdgeIt, 640, 641
LinkPred::UNetwork< LabelT, NodeIDT, EdgeT >::RndNodeIt, 655, 656
LinkPred::UNetwork< LabelT, NodeIDT, EdgeT >::RndNonEdgeIt, 660, 661
LinkPred::UPATPredictor< Network, EdgeRndIt, ScoreRndIt, EdgeRndOutIt >, 898
LinkPred::UPSTPredictor< Network, EdgeRndIt, ScoreRndIt, EdgeRndOutIt >, 904
LinkPred::URALPredictor< Network, EdgeRndIt, ScoreRndIt, EdgeRndOutIt >, 908, 909
LinkPred::URNDPredictor< Network, EdgeRndIt, ScoreRndIt, EdgeRndOutIt >, 914
LinkPred::USAIPredictor< Network, EdgeRndIt, ScoreRndIt, EdgeRndOutIt >, 919, 920
LinkPred::USBMPredictor< Network, EdgeRndIt, ScoreRndIt, EdgeRndOutIt >, 925
LinkPred::USHPPredictor< Network, EdgeRndIt, ScoreRndIt, EdgeRndOutIt >, 932
LinkPred::USOIPredictor< Network, EdgeRndIt, ScoreRndIt, EdgeRndOutIt >, 938, 939
LinkPred::USUMPredictor< Network, EdgeRndIt, ScoreRndIt, EdgeRndOutIt >, 944
LinkPred::Vec, 957
operator==
LinkPred::DNetwork< LabelT, NodeIDT, EdgeT >::NodeDegIt, 482
LinkPred::DNetwork< LabelT, NodeIDT, EdgeT >::NonEdgeIt, 534
LinkPred::DNetwork< LabelT, NodeIDT, EdgeT >::RndEdgeIt, 646
LinkPred::DNetwork< LabelT, NodeIDT, EdgeT >::RndNodeIt, 651
LinkPred::DNetwork< LabelT, NodeIDT, EdgeT >::RndNonEdgeIt, 666
LinkPred::GFMatrix, 339
LinkPred::PredResults< TestDataT, LPredictorT, ScoresContainerT >::ScoreIterator, 687
LinkPred::TestData< Network, EdgeContT >::TestEdgeIt, 717
LinkPred::TestEdgeGen< Network, EdgeContT >::TNEdgeIt, 727
LinkPred::TestEdgeGen< Network, EdgeContT >::TPEdgeIt, 737
LinkPred::UNetwork< LabelT, NodeIDT, EdgeT >::NodeDegIt, 493
LinkPred::UNetwork< LabelT, NodeIDT, EdgeT >::NonEdgeIt, 525
LinkPred::UNetwork< LabelT, NodeIDT, EdgeT >::RndEdgeIt, 641

LinkPred::UNetwork< LabelT, NodeIDT, EdgeT >::RndNodeIt, 656
LinkPred::UNetwork< LabelT, NodeIDT, EdgeT >::RndNonEdgeIt, 661
LinkPred::Vec, 957
operator[]
LinkPred::DNetwork< LabelT, NodeIDT, EdgeT >::EdgeMap< ValueType >, 249, 250
LinkPred::DNetwork< LabelT, NodeIDT, EdgeT >::NodeMap< ValueType >, 504
LinkPred::DNetwork< LabelT, NodeIDT, EdgeT >::NodeSMap< ValueType >, 509, 510
LinkPred::UNetwork< LabelT, NodeIDT, EdgeT >::EdgeMap< ValueType >, 243
LinkPred::UNetwork< LabelT, NodeIDT, EdgeT >::NodeMap< ValueType >, 498, 499
LinkPred::UNetwork< LabelT, NodeIDT, EdgeT >::NodeSMap< ValueType >, 515, 516
LinkPred::Vec, 958
Opt
LinkPred::UMPSPredictor< Network, EdgeRndIt, ScoreRndIt, EdgeRndOutIt >, 846
out
LinkPred::Log, 395
LinkPred::PerfeEvalExpDescp< Network >, 548
outEdgesBegin
LinkPred::DNetwork< LabelT, NodeIDT, EdgeT >, 206
outEdgesEnd
LinkPred::DNetwork< LabelT, NodeIDT, EdgeT >, 207
outNeighborsBegin
LinkPred::DNetwork< LabelT, NodeIDT, EdgeT >, 207
outNeighborsEnd
LinkPred::DNetwork< LabelT, NodeIDT, EdgeT >, 207

p_const_iterator
LinkPred::Bhmap< K, P, Comparator >, 84
partScale
LinkPred::Vec, 959
PAT
LinkPred::UMPSPredictor< Network, EdgeRndIt, ScoreRndIt, EdgeRndOutIt >, 846
PathType
LinkPred::Dijkstra< Network, DistType, NbHopsType >, 148
PathTypeSP
LinkPred::Dijkstra< Network, DistType, NbHopsType >, 148
pbegin
LinkPred::Bhmap< K, P, Comparator >, 87
Pearson
LinkPred::Pearson, 538
pend
LinkPred::Bhmap< K, P, Comparator >, 87
PerfCurve
LinkPred::PerfCurve< PredResultsT >, 543, 544
PerfEvalExp
LinkPred::PerfEvalExp< Network, TestDataT, LPredictorT, PredResultsT, PerfMeasureT >, 550, 551
PerfEvaluator
LinkPred::PerfEvaluator< TestDataT, LPredictorT, PredResultsT, PerfMeasureT >, 556
PerfLambdaT
perfmeasure.hpp, 1051
PerfMeasure
LinkPred::PerfMeasure< PredResultsT >, 563, 564
perfmeasure.hpp
acc, 1051
fdr, 1052
fmr, 1052
fnr, 1053
fpr, 1053
npv, 1053
PerfLambdaT, 1051
pre, 1054
rec, 1054
tnr, 1055
Performance evaluation classes, 27
PerfResults
LinkPred, 38
pfind
LinkPred::Bhmap< K, P, Comparator >, 88
plFit
LinkPred::Utils, 52
pointer
LinkPred::DNetwork< LabelT, NodeIDT, EdgeT >::NodeDegIt, 476
LinkPred::DNetwork< LabelT, NodeIDT, EdgeT >::NonEdgeIt, 528
LinkPred::DNetwork< LabelT, NodeIDT, EdgeT >::RndEdgeIt, 643
LinkPred::DNetwork< LabelT, NodeIDT, EdgeT >::RndNodeIt, 648
LinkPred::DNetwork< LabelT, NodeIDT, EdgeT >::RndNonEdgeIt, 663
LinkPred::PredResults< TestDataT, LPredictorT, ScoresContainerT >::ScoreIterator, 679
LinkPred::TestData< Network, EdgeContT >::TestEdgeIt, 711
LinkPred::TestEdgeGen< Network, EdgeContT >::TNEdgeIt, 721
LinkPred::TestEdgeGen< Network, EdgeContT >::TPEdgeIt, 730
LinkPred::UNetwork< LabelT, NodeIDT, EdgeT >::NodeDegIt, 485
LinkPred::UNetwork< LabelT, NodeIDT, EdgeT >::NonEdgeIt, 518
LinkPred::UNetwork< LabelT, NodeIDT, EdgeT >::RndEdgeIt, 638
LinkPred::UNetwork< LabelT, NodeIDT, EdgeT >::RndNodeIt, 653
LinkPred::UNetwork< LabelT, NodeIDT, EdgeT

>::RndNonEdgeIt, 658
pop
LinkPred::BHeap< T, P, ComparatorT, ComparatorP >, 80
LinkPred::LMapQueue< K, P, KComparator, PComparator >, 391
pos
LinkPred::Bhmap< K, P, Comparator >, 88
posBegin
LinkPred::PredResults< TestDataT, LPredictorT, ScoresContainerT >, 622
LinkPred::TestData< Network, EdgeContT >, 703
posEnd
LinkPred::PredResults< TestDataT, LPredictorT, ScoresContainerT >, 622
LinkPred::TestData< Network, EdgeContT >, 703
posRatio
LinkPred::Simp::Evaluator::Factory::ECLParams, 238
posStrmBegin
LinkPred::PredResults< TestDataT, LPredictorT, ScoresContainerT >, 622
LinkPred::TestData< Network, EdgeContT >, 703
posStrmEnd
LinkPred::PredResults< TestDataT, LPredictorT, ScoresContainerT >, 623
LinkPred::TestData< Network, EdgeContT >, 703
PR
LinkPred::PR< PredResultsT >, 571, 572
pre
perfmeasure.hpp, 1054
predADA
LinkPred::Simp::Predictor, 583
predAllADA
LinkPred::Simp::Predictor, 583
predAllADAByID
LinkPred::Simp::Predictor, 583
predAllCNE
LinkPred::Simp::Predictor, 584
predAllCNEByID
LinkPred::Simp::Predictor, 584
predAllCRA
LinkPred::Simp::Predictor, 584
predAllCRAByID
LinkPred::Simp::Predictor, 584
predAllECL
LinkPred::Simp::Predictor, 585
predAllECLByID
LinkPred::Simp::Predictor, 585
predAllESM
LinkPred::Simp::Predictor, 586
predAllESMByID
LinkPred::Simp::Predictor, 586
predAllFBM
LinkPred::Simp::Predictor, 587
predAllFBMByID
LinkPred::Simp::Predictor, 587
predAllHDI
LinkPred::Simp::Predictor, 589
predAllHDIByID
LinkPred::Simp::Predictor, 589
predAllHPI
LinkPred::Simp::Predictor, 589
predAllHPIByID
LinkPred::Simp::Predictor, 589
predAllHRG
LinkPred::Simp::Predictor, 590
predAllHRGByID
LinkPred::Simp::Predictor, 590
predAllHYP
LinkPred::Simp::Predictor, 591
predAllHYPByID
LinkPred::Simp::Predictor, 591
predAllJID
LinkPred::Simp::Predictor, 592
predAllJIDByID
LinkPred::Simp::Predictor, 592
predAllKAB
LinkPred::Simp::Predictor, 592
predAllKABByID
LinkPred::Simp::Predictor, 592
predAllLCP
LinkPred::Simp::Predictor, 593
predAllLCPByID
LinkPred::Simp::Predictor, 593
predAllLHN
LinkPred::Simp::Predictor, 594
predAllLHNByID
LinkPred::Simp::Predictor, 594
predAllPAT
LinkPred::Simp::Predictor, 594
predAllPATByID
LinkPred::Simp::Predictor, 594
predAllRAL
LinkPred::Simp::Predictor, 594
predAllRALByID
LinkPred::Simp::Predictor, 595
predAllRND
LinkPred::Simp::Predictor, 595
predAllRNDByID
LinkPred::Simp::Predictor, 595
predAllSAI
LinkPred::Simp::Predictor, 596
predAllSAIByID
LinkPred::Simp::Predictor, 596
predAllSBM
LinkPred::Simp::Predictor, 596
predAllSBMByID
LinkPred::Simp::Predictor, 597
predAllSHP
LinkPred::Simp::Predictor, 597
predAllSHPByID
LinkPred::Simp::Predictor, 597
predAllSOI
LinkPred::Simp::Predictor, 598
predAllSOIByID

LinkPred::Simp::Predictor, 598
predCNE
LinkPred::Simp::Predictor, 598
predCRA
LinkPred::Simp::Predictor, 598
predECL
LinkPred::Simp::Predictor, 599
predESM
LinkPred::Simp::Predictor, 599
predFBM
LinkPred::Simp::Predictor, 600
predHDI
LinkPred::Simp::Predictor, 600
predHPI
LinkPred::Simp::Predictor, 600
predHRG
LinkPred::Simp::Predictor, 601
predHYP
LinkPred::Simp::Predictor, 601
predict
LinkPred::Classifier< InRndIt, OutRndIt, ScoreRndIt >, 92
LinkPred::DADAPredictor< Network, EdgeRndIt, ScoreRndIt, EdgeRndOutIt >, 105
LinkPred::DCNEPredictor< Network, EdgeRndIt, ScoreRndIt, EdgeRndOutIt >, 111
LinkPred::DHDIPredictor< Network, EdgeRndIt, ScoreRndIt, EdgeRndOutIt >, 138
LinkPred::DHPIPredictor< Network, EdgeRndIt, ScoreRndIt, EdgeRndOutIt >, 143
LinkPred::DJIDPredictor< Network, EdgeRndIt, ScoreRndIt, EdgeRndOutIt >, 162
LinkPred::DLCPPredictor< Network, EdgeRndIt, ScoreRndIt, EdgeRndOutIt >, 168
LinkPred::DLHNPredictor< Network, EdgeRndIt, ScoreRndIt, EdgeRndOutIt >, 173
LinkPred::DLPredictor< NetworkT, EdgeRndItT, ScoreRndItT, EdgeRndOutItT >, 180
LinkPred::DPATPredictor< Network, EdgeRndIt, ScoreRndIt, EdgeRndOutIt >, 219
LinkPred::DSAIPredictor< Network, EdgeRndIt, ScoreRndIt, EdgeRndOutIt >, 230
LinkPred::DSOIPredictor< Network, EdgeRndIt, ScoreRndIt, EdgeRndOutIt >, 235
LinkPred::LogisticRegresser< InRndIt, OutRndIt, ScoreRndIt >, 400
LinkPred::RndClassifier< InRndIt, OutRndIt, ScoreRndIt >, 636
LinkPred::UADAPredictor< Network, EdgeRndIt, ScoreRndIt, EdgeRndOutIt >, 749
LinkPred::UCNEPredictor< Network, EdgeRndIt, ScoreRndIt, EdgeRndOutIt >, 754
LinkPred::UCRAPredictor< Network, EdgeRndIt, ScoreRndIt, EdgeRndOutIt >, 760
LinkPred::UCSTPredictor< Network, EdgeRndIt, ScoreRndIt, EdgeRndOutIt >, 766
LinkPred::UFBMPredictor< Network, EdgeRndIt, ScoreRndIt, EdgeRndOutIt >, 785
LinkPred::UHDIPredictor< Network, EdgeRndIt, ScoreRndIt, EdgeRndOutIt >, 790
LinkPred::UHPIPredictor< Network, EdgeRndIt, ScoreRndIt, EdgeRndOutIt >, 795
LinkPred::UHRGPredictor< Network, EdgeRndIt, ScoreRndIt, EdgeRndOutIt >, 801
LinkPred::UHYPPredictor< Network, EdgeRndIt, ScoreRndIt, EdgeRndOutIt >, 808
LinkPred::UJIDPredictor< Network, EdgeRndIt, ScoreRndIt, EdgeRndOutIt >, 815
LinkPred::UKABPredictor< Network, EdgeRndIt, ScoreRndIt, EdgeRndOutIt >, 822
LinkPred::ULCPPredictor< Network, EdgeRndIt, ScoreRndIt, EdgeRndOutIt >, 828
LinkPred::ULHNPredictor< Network, EdgeRndIt, ScoreRndIt, EdgeRndOutIt >, 833
LinkPred::ULPredictor< NetworkT, EdgeRndItT, ScoreRndItT, EdgeRndOutItT >, 841
LinkPred::UMPSPredictor< Network, EdgeRndIt, ScoreRndIt, EdgeRndOutIt >, 853
LinkPred::UNEDPredictor< Network, EdgeRndIt, ScoreRndIt, EdgeRndOutIt >, 865
LinkPred::UPATPredictor< Network, EdgeRndIt, ScoreRndIt, EdgeRndOutIt >, 898
LinkPred::URALPredictor< Network, EdgeRndIt, ScoreRndIt, EdgeRndOutIt >, 909
LinkPred::URNDPredictor< Network, EdgeRndIt, ScoreRndIt, EdgeRndOutIt >, 914
LinkPred::USAIPredictor< Network, EdgeRndIt, ScoreRndIt, EdgeRndOutIt >, 920
LinkPred::USBMPredictor< Network, EdgeRndIt, ScoreRndIt, EdgeRndOutIt >, 926
LinkPred::USHPPredictor< Network, EdgeRndIt, ScoreRndIt, EdgeRndOutIt >, 933
LinkPred::USOIPredictor< Network, EdgeRndIt, ScoreRndIt, EdgeRndOutIt >, 939
LinkPred::USUMPredictor< Network, EdgeRndIt, ScoreRndIt, EdgeRndOutIt >, 944
predictNeg
LinkPred::DLPredictor< NetworkT, EdgeRndItT, ScoreRndItT, EdgeRndOutItT >, 180
LinkPred::ULPredictor< NetworkT, EdgeRndItT, ScoreRndItT, EdgeRndOutItT >, 841
Predictor
LinkPred::Simp::Predictor, 580
predJID
LinkPred::Simp::Predictor, 602
predKAB
LinkPred::Simp::Predictor, 602
predLCP
LinkPred::Simp::Predictor, 602
predLHN
LinkPred::Simp::Predictor, 603
predPAT
LinkPred::Simp::Predictor, 603
predRAL
LinkPred::Simp::Predictor, 603
PredResults

LinkPred::PredResults< TestDataT, LPredictorT, ScoresContainerT >, 615, 616
LinkPred::PredResults< TestDataT, LPredictorT, ScoresContainerT >::ScoreIterator, 688
predRND
LinkPred::Simp::Predictor, 603
predSAI
LinkPred::Simp::Predictor, 604
predSBM
LinkPred::Simp::Predictor, 604
predSHP
LinkPred::Simp::Predictor, 604
predSOI
LinkPred::Simp::Predictor, 605
predTopADA
LinkPred::Simp::Predictor, 605
predTopCNE
LinkPred::Simp::Predictor, 605
predTopCRA
LinkPred::Simp::Predictor, 606
predTopECL
LinkPred::Simp::Predictor, 606
predTopESM
LinkPred::Simp::Predictor, 607
predTopFBM
LinkPred::Simp::Predictor, 607
predTopHDI
LinkPred::Simp::Predictor, 608
predTopHPI
LinkPred::Simp::Predictor, 608
predTopHRG
LinkPred::Simp::Predictor, 608
predTopHYP
LinkPred::Simp::Predictor, 609
predTopJID
LinkPred::Simp::Predictor, 609
predTopKAB
LinkPred::Simp::Predictor, 610
predTopLCP
LinkPred::Simp::Predictor, 610
predTopLHN
LinkPred::Simp::Predictor, 611
predTopPAT
LinkPred::Simp::Predictor, 611
predTopRAL
LinkPred::Simp::Predictor, 611
predTopRND
LinkPred::Simp::Predictor, 612
predTopSAI
LinkPred::Simp::Predictor, 612
predTopSBM
LinkPred::Simp::Predictor, 612
predTopSHP
LinkPred::Simp::Predictor, 613
predTopSOI
LinkPred::Simp::Predictor, 613
print
LinkPred::BHeap< T, P, ComparatorT, ComparatorP >, 81
LinkPred::DNetwork< LabelT, NodeIDT, EdgeT >, 208
LinkPred::GFMatrix, 339
LinkPred::UNetwork< LabelT, NodeIDT, EdgeT >, 890
LinkPred::Utils, 53
LinkPred::Vec, 959, 960
printEdges
LinkPred::DNetwork< LabelT, NodeIDT, EdgeT >, 208
LinkPred::UNetwork< LabelT, NodeIDT, EdgeT >, 890
LinkPred::Utils, 53, 54
printPQ
LinkPred::LMapQueue< K, P, KComparator, PComparator >, 392
process
LinkPred::Collector< Network >, 95
LinkPred::Counter< Network >, 101
push
LinkPred::BHeap< T, P, ComparatorT, ComparatorP >, 81
LinkPred::LMapQueue< K, P, KComparator, PComparator >, 392

RA1
LinkPred::UMPSPredictor< Network, EdgeRndIt, ScoreRndIt, EdgeRndOutIt >, 846
RA2
LinkPred::UMPSPredictor< Network, EdgeRndIt, ScoreRndIt, EdgeRndOutIt >, 846
Random
LinkPred::UMPSPredictor< Network, EdgeRndIt, ScoreRndIt, EdgeRndOutIt >, 847
LinkPred::USHPPredictor< Network, EdgeRndIt, ScoreRndIt, EdgeRndOutIt >, 929
RandomGen
LinkPred::RandomGen, 627, 628
ratioEnd
LinkPred::PerfeEvalExpDescp< Network >, 548
ratioStart
LinkPred::PerfeEvalExpDescp< Network >, 548
ratioStep
LinkPred::PerfeEvalExpDescp< Network >, 548
read
LinkPred::DNetwork< LabelT, NodeIDT, EdgeT >, 208
LinkPred::UNetwork< LabelT, NodeIDT, EdgeT >, 890
LinkPred::Vec, 960
readEdges
LinkPred::DNetwork< LabelT, NodeIDT, EdgeT >, 209
LinkPred::UNetwork< LabelT, NodeIDT, EdgeT >, 891
LinkPred::Utils, 54
readEdgeScores

LinkPred::Utils, 54
rec
perfmeasure.hpp, 1054
reference
LinkPred::DNetwork< LabelT, NodeIDT, EdgeT >::NodeDegIt, 476
LinkPred::DNetwork< LabelT, NodeIDT, EdgeT >::NonEdgeIt, 528
LinkPred::DNetwork< LabelT, NodeIDT, EdgeT >::RndEdgeIt, 643
LinkPred::DNetwork< LabelT, NodeIDT, EdgeT >::RndNodeIt, 648
LinkPred::DNetwork< LabelT, NodeIDT, EdgeT >::RndNonEdgeIt, 663
LinkPred::PredResults< TestDataT, LPredictorT, ScoresContainerT >::ScoreIterator, 680
LinkPred::TestData< Network, EdgeContT >::TestEdgeIt, 711
LinkPred::TestEdgeGen< Network, EdgeContT >::TNEdgeIt, 721
LinkPred::TestEdgeGen< Network, EdgeContT >::TPEdgeIt, 731
LinkPred::UNetwork< LabelT, NodeIDT, EdgeT >::NodeDegIt, 485
LinkPred::UNetwork< LabelT, NodeIDT, EdgeT >::NonEdgeIt, 519
LinkPred::UNetwork< LabelT, NodeIDT, EdgeT >::RndEdgeIt, 638
LinkPred::UNetwork< LabelT, NodeIDT, EdgeT >::RndNodeIt, 653
LinkPred::UNetwork< LabelT, NodeIDT, EdgeT >::RndNonEdgeIt, 658
refNet
LinkPred::PerfeEvalExpDescp< Network >, 549
registerLengthMap
LinkPred::Dijkstra< Network, DistType, NbHopsType >, 157
removeNaN
LinkPred::GFMatrix, 340
requiresNeg
LinkPred::PerfMeasure< PredResultsT >, 566
LinkPred::TPR< PredResultsT >, 744
requiresPos
LinkPred::PerfMeasure< PredResultsT >, 567
requiresShuffling
LinkPred::PerfMeasure< PredResultsT >, 567
LinkPred::TPR< PredResultsT >, 744
res
LinkPred::Simp::PerfRes, 568
reset
LinkPred::Vec, 960, 961
resetCount
LinkPred::Counter< Network >, 101
resize
LinkPred::Vec, 961
resultsBegin
LinkPred::PerfEvalExp< Network, TestDataT, LPredictorT, PredResultsT, PerfMeasureT >, 553
LinkPred::PerfEvaluator< TestDataT, LPredictorT, PredResultsT, PerfMeasureT >, 561
resultsEnd
LinkPred::PerfEvalExp< Network, TestDataT, LPredictorT, PredResultsT, PerfMeasureT >, 553
LinkPred::PerfEvaluator< TestDataT, LPredictorT, PredResultsT, PerfMeasureT >, 561
reverseEdge
LinkPred::DNetwork< LabelT, NodeIDT, EdgeT >, 209
LinkPred::UNetwork< LabelT, NodeIDT, EdgeT >, 891
RndClassifier
LinkPred::RndClassifier< InRndIt, OutRndIt, ScoreRndIt >, 633
rndConExtract
LinkPred::NetworkManipulator< Network >, 463
RndEdgeIt
LinkPred::DNetwork< LabelT, NodeIDT, EdgeT >::RndEdgeIt, 643, 644
LinkPred::UNetwork< LabelT, NodeIDT, EdgeT >::RndEdgeIt, 638, 639
rndEdgesBegin
LinkPred::DNetwork< LabelT, NodeIDT, EdgeT >, 209
LinkPred::UNetwork< LabelT, NodeIDT, EdgeT >, 892
rndEdgesEnd
LinkPred::DNetwork< LabelT, NodeIDT, EdgeT >, 210
LinkPred::UNetwork< LabelT, NodeIDT, EdgeT >, 892
rndExtract
LinkPred::NetworkManipulator< Network >, 463
RndNodeIt
LinkPred::DNetwork< LabelT, NodeIDT, EdgeT >::RndNodeIt, 648, 649
LinkPred::UNetwork< LabelT, NodeIDT, EdgeT >::RndNodeIt, 653, 654
rndNodesBegin
LinkPred::DNetwork< LabelT, NodeIDT, EdgeT >, 210
LinkPred::UNetwork< LabelT, NodeIDT, EdgeT >, 892
rndNodesEnd
LinkPred::DNetwork< LabelT, NodeIDT, EdgeT >, 210
LinkPred::UNetwork< LabelT, NodeIDT, EdgeT >, 893
RndNonEdgeIt
LinkPred::DNetwork< LabelT, NodeIDT, EdgeT >::RndNonEdgeIt, 663, 664
LinkPred::UNetwork< LabelT, NodeIDT, EdgeT >::RndNonEdgeIt, 658, 659
rndNonEdgesBegin
LinkPred::DNetwork< LabelT, NodeIDT, EdgeT >,

211
LinkPred::UNetwork< LabelT, NodeIDT, EdgeT >, 893
rndNonEdgesEnd
LinkPred::DNetwork< LabelT, NodeIDT, EdgeT >, 211
LinkPred::UNetwork< LabelT, NodeIDT, EdgeT >, 893
ROC
LinkPred::ROC< PredResultsT >, 669, 670
rst
LinkPred::NetworkManipulator< Network >, 464
rtrim
LinkPred::Utils, 55
run
LinkPred::PerfEvalExp< Network, TestDataT, LPredictorT, PredResultsT, PerfMeasureT >, 554
LinkPred::Simp::Evaluator, 320
runNoTiming
LinkPred::PerfEvalExp< Network, TestDataT, LPredictorT, PredResultsT, PerfMeasureT >, 554
runTiming
LinkPred::PerfEvalExp< Network, TestDataT, LPredictorT, PredResultsT, PerfMeasureT >, 554

scale
LinkPred::Vec, 961
Scan
LinkPred::UMPSPredictor< Network, EdgeRndIt, ScoreRndIt, EdgeRndOutIt >, 846
scatter
LinkPred::Vec, 962
score
LinkPred::DADAPredictor< Network, EdgeRndIt, ScoreRndIt, EdgeRndOutIt >, 106
LinkPred::DCNEPredictor< Network, EdgeRndIt, ScoreRndIt, EdgeRndOutIt >, 111
LinkPred::DHDIPredictor< Network, EdgeRndIt, ScoreRndIt, EdgeRndOutIt >, 138
LinkPred::DHPIPredictor< Network, EdgeRndIt, ScoreRndIt, EdgeRndOutIt >, 144
LinkPred::DJIDPredictor< Network, EdgeRndIt, ScoreRndIt, EdgeRndOutIt >, 162
LinkPred::DLCPPredictor< Network, EdgeRndIt, ScoreRndIt, EdgeRndOutIt >, 168
LinkPred::DLHNPredictor< Network, EdgeRndIt, ScoreRndIt, EdgeRndOutIt >, 173
LinkPred::DLPredictor< NetworkT, EdgeRndItT, ScoreRndItT, EdgeRndOutItT >, 181
LinkPred::DPATPredictor< Network, EdgeRndIt, ScoreRndIt, EdgeRndOutIt >, 220
LinkPred::DPSTPredictor< Network, EdgeRndIt, ScoreRndIt, EdgeRndOutIt >, 225
LinkPred::DSAIPredictor< Network, EdgeRndIt, ScoreRndIt, EdgeRndOutIt >, 230
LinkPred::DSOIPredictor< Network, EdgeRndIt, ScoreRndIt, EdgeRndOutIt >, 236
LinkPred::Simp::EdgeScore, 252
LinkPred::Simp::EdgeScoreByID, 253
LinkPred::UADAPredictor< Network, EdgeRndIt, ScoreRndIt, EdgeRndOutIt >, 749
LinkPred::UCNEPredictor< Network, EdgeRndIt, ScoreRndIt, EdgeRndOutIt >, 755
LinkPred::UCRAPredictor< Network, EdgeRndIt, ScoreRndIt, EdgeRndOutIt >, 760
LinkPred::UCSTPredictor< Network, EdgeRndIt, ScoreRndIt, EdgeRndOutIt >, 767
LinkPred::UECLPredictor< Network, EdgeRndIt, ScoreRndIt, EdgeRndOutIt >, 773
LinkPred::UESMPredictor< Network, EdgeRndIt, ScoreRndIt, EdgeRndOutIt >, 780
LinkPred::UFBMPredictor< Network, EdgeRndIt, ScoreRndIt, EdgeRndOutIt >, 785
LinkPred::UHDIPredictor< Network, EdgeRndIt, ScoreRndIt, EdgeRndOutIt >, 791
LinkPred::UHPIPredictor< Network, EdgeRndIt, ScoreRndIt, EdgeRndOutIt >, 796
LinkPred::UHRGPredictor< Network, EdgeRndIt, ScoreRndIt, EdgeRndOutIt >, 802
LinkPred::UHYPPredictor< Network, EdgeRndIt, ScoreRndIt, EdgeRndOutIt >, 809
LinkPred::UJIDPredictor< Network, EdgeRndIt, ScoreRndIt, EdgeRndOutIt >, 815
LinkPred::UKABPredictor< Network, EdgeRndIt, ScoreRndIt, EdgeRndOutIt >, 822
LinkPred::ULCPPredictor< Network, EdgeRndIt, ScoreRndIt, EdgeRndOutIt >, 828
LinkPred::ULHNPredictor< Network, EdgeRndIt, ScoreRndIt, EdgeRndOutIt >, 834
LinkPred::ULPredictor< NetworkT, EdgeRndItT, ScoreRndItT, EdgeRndOutItT >, 842
LinkPred::UMPSPredictor< Network, EdgeRndIt, ScoreRndIt, EdgeRndOutIt >, 854
LinkPred::UNEDPredictor< Network, EdgeRndIt, ScoreRndIt, EdgeRndOutIt >, 866
LinkPred::UPATPredictor< Network, EdgeRndIt, ScoreRndIt, EdgeRndOutIt >, 899
LinkPred::UPSTPredictor< Network, EdgeRndIt, ScoreRndIt, EdgeRndOutIt >, 904
LinkPred::URALPredictor< Network, EdgeRndIt, ScoreRndIt, EdgeRndOutIt >, 910
LinkPred::URNDPredictor< Network, EdgeRndIt, ScoreRndIt, EdgeRndOutIt >, 915
LinkPred::USAIPredictor< Network, EdgeRndIt, ScoreRndIt, EdgeRndOutIt >, 921
LinkPred::USBMPredictor< Network, EdgeRndIt, ScoreRndIt, EdgeRndOutIt >, 926
LinkPred::USHPPredictor< Network, EdgeRndIt, ScoreRndIt, EdgeRndOutIt >, 933
LinkPred::USOIPredictor< Network, EdgeRndIt, ScoreRndIt, EdgeRndOutIt >, 939
LinkPred::USUMPredictor< Network, EdgeRndIt, ScoreRndIt, EdgeRndOutIt >, 945

LinkPred::Utils::EdgeScore< Label >, 251
ScoreIterator
LinkPred::PredResults< TestDataT, LPredictorT, ScoresContainerT >::ScoreIterator, 680, 682
ScoreRndIt
LinkPred::DLPredictor< NetworkT, EdgeRndItT, ScoreRndItT, EdgeRndOutItT >, 176
LinkPred::ULPredictor< NetworkT, EdgeRndItT, ScoreRndItT, EdgeRndOutItT >, 837
ScoresItT
LinkPred::GCurve< PredResultsT >, 326
LinkPred::PerfCurve< PredResultsT >, 543
LinkPred::PerfMeasure< PredResultsT >, 563
LinkPred::PR< PredResultsT >, 571
LinkPred::PredResults< TestDataT, LPredictorT, ScoresContainerT >, 615
LinkPred::ROC< PredResultsT >, 669
LinkPred::TPR< PredResultsT >, 740
seed
LinkPred::PerfeEvalExpDescp< Network >, 549
LinkPred::Simp::Evaluator::Factory::ECLParams, 238
LinkPred::Simp::Evaluator::Factory::ESMParams, 262
LinkPred::Simp::Evaluator::Factory::FBMParams, 324
LinkPred::Simp::Evaluator::Factory::HRGParams, 359
LinkPred::Simp::Evaluator::Factory::HYPParams, 360
LinkPred::Simp::Evaluator::Factory::RNDParams, 667
LinkPred::Simp::Evaluator::Factory::SBMParams, 677
LinkPred::Simp::Evaluator::Factory::SHPParams, 689
selectRandom
LinkPred::Utils, 55
selectRandomInPlace
LinkPred::Utils, 55
selectTopK
LinkPred::Utils, 56
set
LinkPred::BHeap< T, P, ComparatorT, ComparatorP >, 81
LinkPred::GFMatrix, 340
setAlg
LinkPred::MDS, 431
setAlpha
LinkPred::DeepWalk< Network >, 119
LinkPred::HMSM< Network >, 356
setAsp
LinkPred::UMPSPredictor< Network, EdgeRndIt, ScoreRndIt, EdgeRndOutIt >, 854
LinkPred::USHPPredictor< Network, EdgeRndIt, ScoreRndIt, EdgeRndOutIt >, 934
setCacheLevel
LinkPred::UKABPredictor< Network, EdgeRndIt, ScoreRndIt, EdgeRndOutIt >, 823
LinkPred::UMPSPredictor< Network, EdgeRndIt, ScoreRndIt, EdgeRndOutIt >, 855
LinkPred::USHPPredictor< Network, EdgeRndIt, ScoreRndIt, EdgeRndOutIt >, 934
setClassifier
LinkPred::UECLPredictor< Network, EdgeRndIt, ScoreRndIt, EdgeRndOutIt >, 774
setCol
LinkPred::GFMatrix, 340
setConstant
LinkPred::Vec, 962
setDiag
LinkPred::GFMatrix, 341
setDim
LinkPred::Encoder< Network >, 260
setDiscDist
LinkPred::NetDistCalculator< Network, DistType, NbHopsType >, 437
LinkPred::NetIndSimlCalculator< Network, DistType, NbHopsType >, 442
LinkPred::NetSimlCalculator< Network, DistType, NbHopsType >, 449
setDiscNbHops
LinkPred::NetDistCalculator< Network, DistType, NbHopsType >, 438
LinkPred::NetIndSimlCalculator< Network, DistType, NbHopsType >, 442
LinkPred::NetSimlCalculator< Network, DistType, NbHopsType >, 449
setEdgeLengthMethod
LinkPred::UMPSPredictor< Network, EdgeRndIt, ScoreRndIt, EdgeRndOutIt >, 855
setEdgeScores
LinkPred::DPSTPredictor< Network, EdgeRndIt, ScoreRndIt, EdgeRndOutIt >, 225
LinkPred::UPSTPredictor< Network, EdgeRndIt, ScoreRndIt, EdgeRndOutIt >, 905
setEnableReconstruct
LinkPred::LINE< Network >, 382
setEncoder
LinkPred::UECLPredictor< Network, EdgeRndIt, ScoreRndIt, EdgeRndOutIt >, 774
LinkPred::UESMPredictor< Network, EdgeRndIt, ScoreRndIt, EdgeRndOutIt >, 780
setEps
LinkPred::HMSM< Network >, 356
setEpsilon
LinkPred::ULCPPredictor< Network, EdgeRndIt, ScoreRndIt, EdgeRndOutIt >, 829
setGamma
LinkPred::LargeVis< Network >, 374
LinkPred::UHYPPredictor< Network, EdgeRndIt, ScoreRndIt, EdgeRndOutIt >, 809
setHorizLim
LinkPred::UKABPredictor< Network, EdgeRndIt, ScoreRndIt, EdgeRndOutIt >, 823
setHProb

LinkPred::HMSM< Network >, 356
setInitLR
LinkPred::DeepWalk< Network >, 119
LinkPred::LargeVis< Network >, 374
LinkPred::Node2Vec< Network >, 471
setInitLr
LinkPred::LINE< Network >, 383
setInterpolMethod
LinkPred::PR< PredResultsT >, 578
setL
LinkPred::UHYPPredictor< Network, EdgeRndIt, ScoreRndIt, EdgeRndOutIt >, 810
setLambda
LinkPred::LogisticRegresser< InRndIt, OutRndIt, ScoreRndIt >, 400
LinkPred::LogRegCG< InRndIt, OutRndIt >, 412
LinkPred::MatFact< Network >, 421
LinkPred::NetIndSimlCalculator< Network, DistType, NbHopsType >, 443
LinkPred::NetSimlCalculator< Network, DistType, NbHopsType >, 449
LinkPred::UMPSPredictor< Network, EdgeRndIt, ScoreRndIt, EdgeRndOutIt >, 855
setLambdaMethod
LinkPred::UMPSPredictor< Network, EdgeRndIt, ScoreRndIt, EdgeRndOutIt >, 856
setLambdaNegEstRatio
LinkPred::UMPSPredictor< Network, EdgeRndIt, ScoreRndIt, EdgeRndOutIt >, 856
setLambdaPosEstRatio
LinkPred::UMPSPredictor< Network, EdgeRndIt, ScoreRndIt, EdgeRndOutIt >, 856
setLambdaStep
LinkPred::UMPSPredictor< Network, EdgeRndIt, ScoreRndIt, EdgeRndOutIt >, 858
setLandmarkRatio
LinkPred::UMPSPredictor< Network, EdgeRndIt, ScoreRndIt, EdgeRndOutIt >, 858
LinkPred::USHPPredictor< Network, EdgeRndIt, ScoreRndIt, EdgeRndOutIt >, 934
setLandmarks
LinkPred::ASPDistCalculator< Network, DistType, NbHopsType >, 65
LinkPred::ASPDsimCalculator< Network, DsimType, NbHopsType >, 72
setLandmarkStrategy
LinkPred::UMPSPredictor< Network, EdgeRndIt, ScoreRndIt, EdgeRndOutIt >, 858
LinkPred::USHPPredictor< Network, EdgeRndIt, ScoreRndIt, EdgeRndOutIt >, 935
setM
LinkPred::UHYPPredictor< Network, EdgeRndIt, ScoreRndIt, EdgeRndOutIt >, 810
setMaxIter
LinkPred::UFBMPredictor< Network, EdgeRndIt, ScoreRndIt, EdgeRndOutIt >, 786
LinkPred::USBMPredictor< Network, EdgeRndIt, ScoreRndIt, EdgeRndOutIt >, 927
setMaxLambda
LinkPred::UMPSPredictor< Network, EdgeRndIt, ScoreRndIt, EdgeRndOutIt >, 859
setMaxNbNodesInCache
LinkPred::DESPLDistCalculator< Network, DistType, NbHopsType >, 130
LinkPred::ESPLDistCalculator< Network, DistType, NbHopsType >, 292
LinkPred::ESPLSimlCalculator< Network, DistType, NbHopsType >, 301
LinkPred::ESPSimlCalculator< Network, DistType, NbHopsType >, 309
setMinLambda
LinkPred::UMPSPredictor< Network, EdgeRndIt, ScoreRndIt, EdgeRndOutIt >, 859
setName
LinkPred::Classifier< InRndIt, OutRndIt, ScoreRndIt >, 92
LinkPred::DLPredictor< NetworkT, EdgeRndItT, ScoreRndItT, EdgeRndOutItT >, 181
LinkPred::Encoder< Network >, 260
LinkPred::GFMatrix, 341
LinkPred::PerfMeasure< PredResultsT >, 567
LinkPred::SimMeasure, 692
LinkPred::ULPredictor< NetworkT, EdgeRndItT, ScoreRndItT, EdgeRndOutItT >, 842
setNbBeans
LinkPred::UHRGPredictor< Network, EdgeRndIt, ScoreRndIt, EdgeRndOutIt >, 802
setNbMdsRuns
LinkPred::HMSM< Network >, 357
setNbNegSamples
LinkPred::LargeVis< Network >, 374
LinkPred::LINE< Network >, 383
LinkPred::Node2Vec< Network >, 471
setNbNodeWalks
LinkPred::DeepWalk< Network >, 119
setNbRuns
LinkPred::MDS, 431
setNbSamples
LinkPred::LargeVis< Network >, 374, 375
LinkPred::UHRGPredictor< Network, EdgeRndIt, ScoreRndIt, EdgeRndOutIt >, 802
setNbWalks
LinkPred::DeepWalk< Network >, 120
LinkPred::Node2Vec< Network >, 471
setNegRatio
LinkPred::MatFact< Network >, 421
LinkPred::UECLPredictor< Network, EdgeRndIt, ScoreRndIt, EdgeRndOutIt >, 774
setNegSamplingPower
LinkPred::LargeVis< Network >, 375
LinkPred::LINE< Network >, 383
setNegScore
LinkPred::UMPSPredictor< Network, EdgeRndIt, ScoreRndIt, EdgeRndOutIt >, 859
setNegSize
LinkPred::LargeVis< Network >, 375

LinkPred::LINE< Network >, 383
setNet
LinkPred::Dijkstra< Network, DistType, NbHopsType >, 157
setOrder
LinkPred::LINE< Network >, 384
setOutPrec
LinkPred::PerfEvalExp< Network, TestDataT, LPredictorT, PredResultsT, PerfMeasureT >, 554
setP
LinkPred::LPSim, 415
LinkPred::Node2Vec< Network >, 472
setPerfMeasure
LinkPred::UMPSPredictor< Network, EdgeRndIt, ScoreRndIt, EdgeRndOutIt >, 860
setPosRatio
LinkPred::MatFact< Network >, 421
LinkPred::UECLPredictor< Network, EdgeRndIt, ScoreRndIt, EdgeRndOutIt >, 775
setPosScore
LinkPred::UMPSPredictor< Network, EdgeRndIt, ScoreRndIt, EdgeRndOutIt >, 860
setProb
LinkPred::HMSM< Network >, 357
setQ
LinkPred::Node2Vec< Network >, 472
setRow
LinkPred::GFMatrix, 341
setSelfSiml
LinkPred::NetIndSimlCalculator< Network, DistType, NbHopsType >, 443
LinkPred::NetSimlCalculator< Network, DistType, NbHopsType >, 449
setSimMeasure
LinkPred::UESMPredictor< Network, EdgeRndIt, ScoreRndIt, EdgeRndOutIt >, 780
setStepInterval
LinkPred::DeepWalk< Network >, 120
LinkPred::Node2Vec< Network >, 472
setStrmEnabled
LinkPred::ROC< PredResultsT >, 676
setStrmNeg
LinkPred::ROC< PredResultsT >, 676
setSubSample
LinkPred::Node2Vec< Network >, 473
setT
LinkPred::UHYPPredictor< Network, EdgeRndIt, ScoreRndIt, EdgeRndOutIt >, 810
setTimingEnabled
LinkPred::PerfEvaluator< TestDataT, LPredictorT, PredResultsT, PerfMeasureT >, 562
setTol
LinkPred::HMSM< Network >, 357
LinkPred::LogisticRegresser< InRndIt, OutRndIt, ScoreRndIt >, 401
LinkPred::MatFact< Network >, 422
LinkPred::MDS, 431
LinkPred::UMPSPredictor< Network, EdgeRndIt, ScoreRndIt, EdgeRndOutIt >, 860
setUseHops
LinkPred::ESPLSimlCalculator< Network, DistType, NbHopsType >, 302
setUseTopMethod
LinkPred::TPR< PredResultsT >, 745
setWalkLength
LinkPred::DeepWalk< Network >, 120
LinkPred::Node2Vec< Network >, 473
setWeightMap
LinkPred::DeepWalk< Network >, 121
LinkPred::Encoder< Network >, 260
LinkPred::HMSM< Network >, 358
LinkPred::MatFact< Network >, 422
LinkPred::Node2Vec< Network >, 473
setWindowSize
LinkPred::DeepWalk< Network >, 121
LinkPred::Node2Vec< Network >, 473
setZeta
LinkPred::UHYPPredictor< Network, EdgeRndIt, ScoreRndIt, EdgeRndOutIt >, 811
shuffle
LinkPred::DNetwork< LabelT, NodeIDT, EdgeT >, 211
LinkPred::UNetwork< LabelT, NodeIDT, EdgeT >, 893
LinkPred::Utils, 56
sim
LinkPred::CosineSim, 98
LinkPred::DotProd, 215
LinkPred::L1Sim, 364
LinkPred::L2Sim, 367
LinkPred::LPSim, 416
LinkPred::Pearson, 539
LinkPred::SimMeasure, 692
SimMeasure
LinkPred::SimMeasure, 690, 691
simMeasureName
LinkPred::Simp::Evaluator::Factory::ESMParams, 262
Simplified interface, 30
size
LinkPred::BHeap< T, P, ComparatorT, ComparatorP >, 82
LinkPred::Bhmap< K, P, Comparator >, 88
LinkPred::DNetwork< LabelT, NodeIDT, EdgeT >::EdgeMap< ValueType >, 250
LinkPred::DNetwork< LabelT, NodeIDT, EdgeT >::NodeSMap< ValueType >, 510
LinkPred::LMapQueue< K, P, KComparator, PComparator >, 393
LinkPred::UNetwork< LabelT, NodeIDT, EdgeT >::EdgeMap< ValueType >, 244
LinkPred::UNetwork< LabelT, NodeIDT, EdgeT >::NodeSMap< ValueType >, 516
LinkPred::Vec, 962
solve

LinkPred::MDS, 432
sort
LinkPred::Utils, 56
sortNeg
LinkPred::PredResults< TestDataT, LPredictorT, ScoresContainerT >, 623
SortOrder
LinkPred, 40
sortPos
LinkPred::PredResults< TestDataT, LPredictorT, ScoresContainerT >, 623
start
LinkPred::DNetwork< LabelT, NodeIDT, EdgeT >, 212
LinkPred::UNetwork< LabelT, NodeIDT, EdgeT >, 894
sum
LinkPred::GFMatrix, 341
LinkPred::Vec, 962
sumCols
LinkPred::GFMatrix, 342
sumRows
LinkPred::GFMatrix, 342

T
LinkPred::Simp::Evaluator::Factory::HYPParams, 360
target
LinkPred::MatFactPbData, 428
TestData
LinkPred::TestData< Network, EdgeContT >, 696, 697
LinkPred::TestData< Network, EdgeContT >::TestEdgeIt, 719
TestEdgeGen
LinkPred::TestEdgeGen< Network, EdgeContT >, 705, 706
LinkPred::TestEdgeGen< Network, EdgeContT >::TNEdgeIt, 728
LinkPred::TestEdgeGen< Network, EdgeContT >::TPEdgeIt, 738
TestEdgeIt
LinkPred::TestData< Network, EdgeContT >::TestEdgeIt, 711, 712
timingEnabled
LinkPred::PerfeEvalExpDescp< Network >, 549
TN
LinkPred, 39
tnBegin
LinkPred::TestEdgeGen< Network, EdgeContT >, 708
TNEdgeIt
LinkPred::TestEdgeGen< Network, EdgeContT >::TNEdgeIt, 721, 722
TNEIT
LinkPred::TestData< Network, EdgeContT >, 696
tnEnd
LinkPred::TestEdgeGen< Network, EdgeContT >, 708
tnr
perfmeasure.hpp, 1055
tnRatio
LinkPred::PerfeEvalExpDescp< Network >, 549
top
LinkPred::BHeap< T, P, ComparatorT, ComparatorP >, 82
LinkPred::DADAPredictor< Network, EdgeRndIt, ScoreRndIt, EdgeRndOutIt >, 106
LinkPred::DCNEPredictor< Network, EdgeRndIt, ScoreRndIt, EdgeRndOutIt >, 112
LinkPred::DHDIPredictor< Network, EdgeRndIt, ScoreRndIt, EdgeRndOutIt >, 139
LinkPred::DHPIPredictor< Network, EdgeRndIt, ScoreRndIt, EdgeRndOutIt >, 144
LinkPred::DJIDPredictor< Network, EdgeRndIt, ScoreRndIt, EdgeRndOutIt >, 163
LinkPred::DLHNPredictor< Network, EdgeRndIt, ScoreRndIt, EdgeRndOutIt >, 174
LinkPred::DLPredictor< NetworkT, EdgeRndItT, ScoreRndItT, EdgeRndOutItT >, 182
LinkPred::DSAIPredictor< Network, EdgeRndIt, ScoreRndIt, EdgeRndOutIt >, 231
LinkPred::DSOIPredictor< Network, EdgeRndIt, ScoreRndIt, EdgeRndOutIt >, 236
LinkPred::LMapQueue< K, P, KComparator, PComparator >, 393
LinkPred::UADAPredictor< Network, EdgeRndIt, ScoreRndIt, EdgeRndOutIt >, 750
LinkPred::UCNEPredictor< Network, EdgeRndIt, ScoreRndIt, EdgeRndOutIt >, 755
LinkPred::UCRAPredictor< Network, EdgeRndIt, ScoreRndIt, EdgeRndOutIt >, 761
LinkPred::UCSTPredictor< Network, EdgeRndIt, ScoreRndIt, EdgeRndOutIt >, 767
LinkPred::UHDIPredictor< Network, EdgeRndIt, ScoreRndIt, EdgeRndOutIt >, 791
LinkPred::UHPIPredictor< Network, EdgeRndIt, ScoreRndIt, EdgeRndOutIt >, 796
LinkPred::UJIDPredictor< Network, EdgeRndIt, ScoreRndIt, EdgeRndOutIt >, 816
LinkPred::UKABPredictor< Network, EdgeRndIt, ScoreRndIt, EdgeRndOutIt >, 823
LinkPred::ULHNPredictor< Network, EdgeRndIt, ScoreRndIt, EdgeRndOutIt >, 834
LinkPred::ULPredictor< NetworkT, EdgeRndItT, ScoreRndItT, EdgeRndOutItT >, 842
LinkPred::UNEDPredictor< Network, EdgeRndIt, ScoreRndIt, EdgeRndOutIt >, 866
LinkPred::UPATPredictor< Network, EdgeRndIt, ScoreRndIt, EdgeRndOutIt >, 899
LinkPred::URALPredictor< Network, EdgeRndIt, ScoreRndIt, EdgeRndOutIt >, 910
LinkPred::USAIPredictor< Network, EdgeRndIt, ScoreRndIt, EdgeRndOutIt >, 921
LinkPred::USOIPredictor< Network, EdgeRndIt, ScoreRndIt, EdgeRndOutIt >, 940
LinkPred::USUMPredictor< Network, EdgeRndIt,

ScoreRndIt, EdgeRndOutIt >, 945
topBegin
LinkPred::PredResults< TestDataT, LPredictorT, ScoresContainerT >, 625
topEdgesBegin
LinkPred::PredResults< TestDataT, LPredictorT, ScoresContainerT >, 625
topEdgesEnd
LinkPred::PredResults< TestDataT, LPredictorT, ScoresContainerT >, 625
topEnd
LinkPred::PredResults< TestDataT, LPredictorT, ScoresContainerT >, 625
TP
LinkPred, 39
tpBegin
LinkPred::TestEdgeGen< Network, EdgeContT >, 708
TPEdgeIt
LinkPred::TestEdgeGen< Network, EdgeContT >::TPEdgeIt, 731
TPEIT
LinkPred::TestData< Network, EdgeContT >, 696
tpEnd
LinkPred::TestEdgeGen< Network, EdgeContT >, 708
TPR
LinkPred::TPR< PredResultsT >, 740, 741
traverse
LinkPred::BFS< Network, NodeProcessor >, 76
LinkPred::DFS< Network, NodeProcessor >, 133
LinkPred::GraphTraversal< Network, NodeProcessor >, 350
trim
LinkPred::Utils, 57
tryDecrease
LinkPred::BHeap< T, P, ComparatorT, ComparatorP >, 82
tryIncrease
LinkPred::BHeap< T, P, ComparatorT, ComparatorP >, 82

UADAPredictor
LinkPred::UADAPredictor< Network, EdgeRndIt, ScoreRndIt, EdgeRndOutIt >, 747
UCNEPredictor
LinkPred::UCNEPredictor< Network, EdgeRndIt, ScoreRndIt, EdgeRndOutIt >, 752, 753
UCRAPredictor
LinkPred::UCRAPredictor< Network, EdgeRndIt, ScoreRndIt, EdgeRndOutIt >, 758
UCSTPredictor
LinkPred::UCSTPredictor< Network, EdgeRndIt, ScoreRndIt, EdgeRndOutIt >, 763, 765
UECLPredictor
LinkPred::UECLPredictor< Network, EdgeRndIt, ScoreRndIt, EdgeRndOutIt >, 770
UESMPredictor
LinkPred::UESMPredictor< Network, EdgeRndIt, ScoreRndIt, EdgeRndOutIt >, 777
UFBMPredictor
LinkPred::UFBMPredictor< Network, EdgeRndIt, ScoreRndIt, EdgeRndOutIt >, 782, 783
UHDIPredictor
LinkPred::UHDIPredictor< Network, EdgeRndIt, ScoreRndIt, EdgeRndOutIt >, 788
UHPIPredictor
LinkPred::UHPIPredictor< Network, EdgeRndIt, ScoreRndIt, EdgeRndOutIt >, 793, 794
UHRGPredictor
LinkPred::UHRGPredictor< Network, EdgeRndIt, ScoreRndIt, EdgeRndOutIt >, 799
UHYPPredictor
LinkPred::UHYPPredictor< Network, EdgeRndIt, ScoreRndIt, EdgeRndOutIt >, 804, 805
UJIDPredictor
LinkPred::UJIDPredictor< Network, EdgeRndIt, ScoreRndIt, EdgeRndOutIt >, 813
UKABPredictor
LinkPred::UKABPredictor< Network, EdgeRndIt, ScoreRndIt, EdgeRndOutIt >, 819
ULCPPredictor
LinkPred::ULCPPredictor< Network, EdgeRndIt, ScoreRndIt, EdgeRndOutIt >, 825, 826
ULHNPredictor
LinkPred::ULHNPredictor< Network, EdgeRndIt, ScoreRndIt, EdgeRndOutIt >, 831
ULPredictor
LinkPred::ULPredictor< NetworkT, EdgeRndItT, ScoreRndItT, EdgeRndOutItT >, 837, 838
UMPSPredictor
LinkPred::UMPSPredictor< Network, EdgeRndIt, ScoreRndIt, EdgeRndOutIt >, 847
UNEDPredictor
LinkPred::UNEDPredictor< Network, EdgeRndIt, ScoreRndIt, EdgeRndOutIt >, 863
UNetwork
LinkPred::UNetwork< LabelT, NodeIDT, EdgeT >, 871, 872
LinkPred::UNetwork< LabelT, NodeIDT, EdgeT >::NodeDegIt, 494
LinkPred::UNetwork< LabelT, NodeIDT, EdgeT >::NonEdgeIt, 526
unregisterLengthMap
LinkPred::Dijkstra< Network, DistType, NbHopsType >, 158
UPATPredictor
LinkPred::UPATPredictor< Network, EdgeRndIt, ScoreRndIt, EdgeRndOutIt >, 896, 897
UPSTPredictor
LinkPred::UPSTPredictor< Network, EdgeRndIt, ScoreRndIt, EdgeRndOutIt >, 902
URALPredictor
LinkPred::URALPredictor< Network, EdgeRndIt, ScoreRndIt, EdgeRndOutIt >, 907
URNDPredictor

LinkPred::URNDPredictor< Network, EdgeRndIt, ScoreRndIt, EdgeRndOutIt >, 912, 913
USAIPredictor
LinkPred::USAIPredictor< Network, EdgeRndIt, ScoreRndIt, EdgeRndOutIt >, 918
USBMPredictor
LinkPred::USBMPredictor< Network, EdgeRndIt, ScoreRndIt, EdgeRndOutIt >, 923, 924
User
LinkPred::UMPSPredictor< Network, EdgeRndIt, ScoreRndIt, EdgeRndOutIt >, 846
USHPPredictor
LinkPred::USHPPredictor< Network, EdgeRndIt, ScoreRndIt, EdgeRndOutIt >, 929, 930
USOIPredictor
LinkPred::USOIPredictor< Network, EdgeRndIt, ScoreRndIt, EdgeRndOutIt >, 937
USUMPredictor
LinkPred::USUMPredictor< Network, EdgeRndIt, ScoreRndIt, EdgeRndOutIt >, 942, 943
Utility functions and classes, 31

Vec
LinkPred::Vec, 948–950

WeightMap
LinkPred::Encoder< Network >, 255
WeightMapSP
LinkPred::Encoder< Network >, 255
write
LinkPred::DNetwork< LabelT, NodeIDT, EdgeT >, 212
LinkPred::UNetwork< LabelT, NodeIDT, EdgeT >, 894
LinkPred::Vec, 963
writeEdgeScores
LinkPred::Utils, 57

zeta
LinkPred::Simp::Evaluator::Factory::HYPParams, 360
