# Supplementary material for: LinkPred: a high performance library for link prediction in complex networks
Source: PeerJ Comput Sci. 2021 May 21;7:e521. doi: 10.7717/peerj-cs.521 (PMC8157017; doi:10.7717/peerj-cs.521)
Supplement: Supplemental Information 1 [file peerj-cs-07-521-s001.zip › LinkPred/tutorials/Tutorials.pdf]

# LinkPred Tutorials

## A High Performance Library for Link Prediction in Complex Networks

Said Kerrache

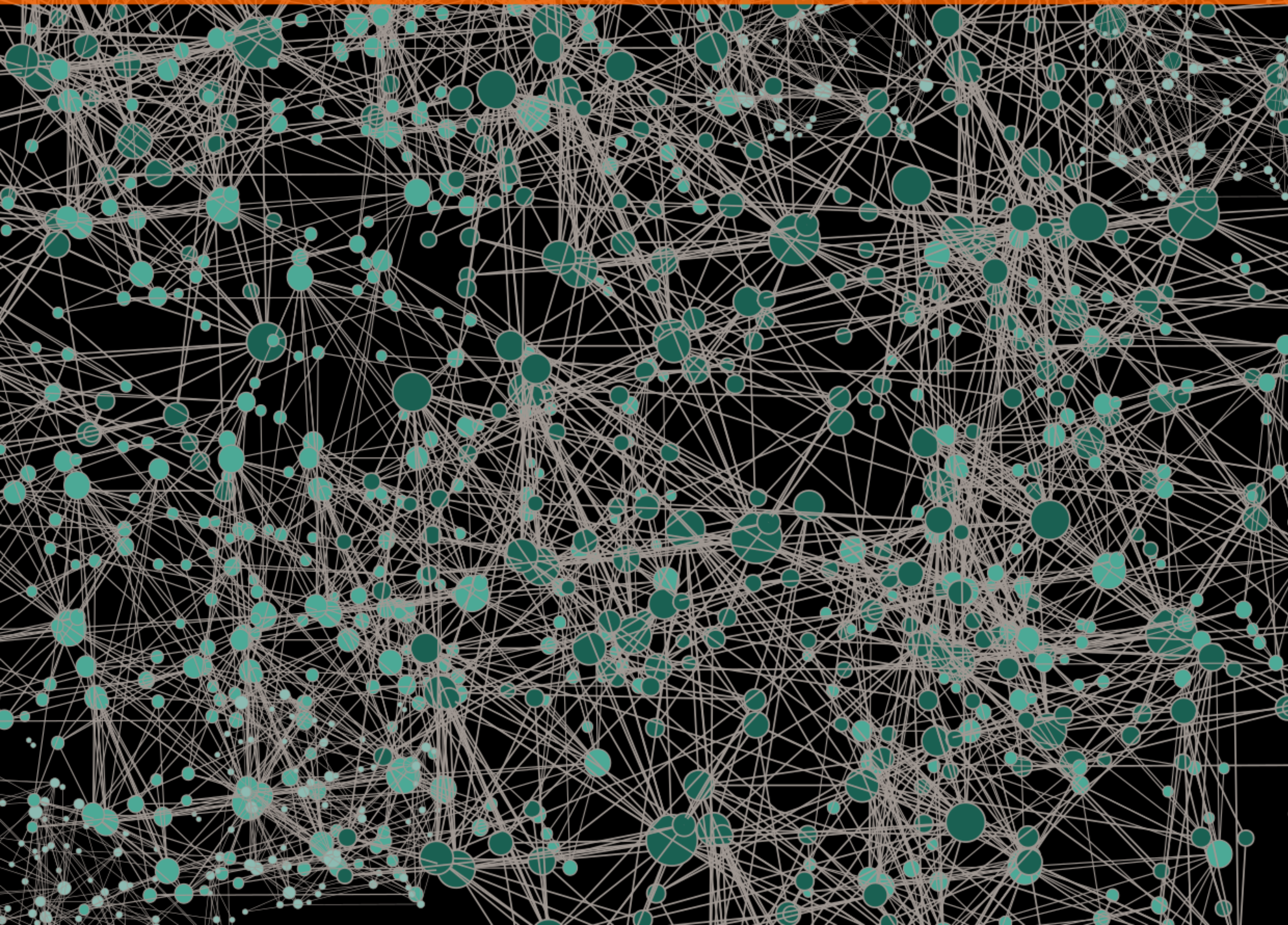

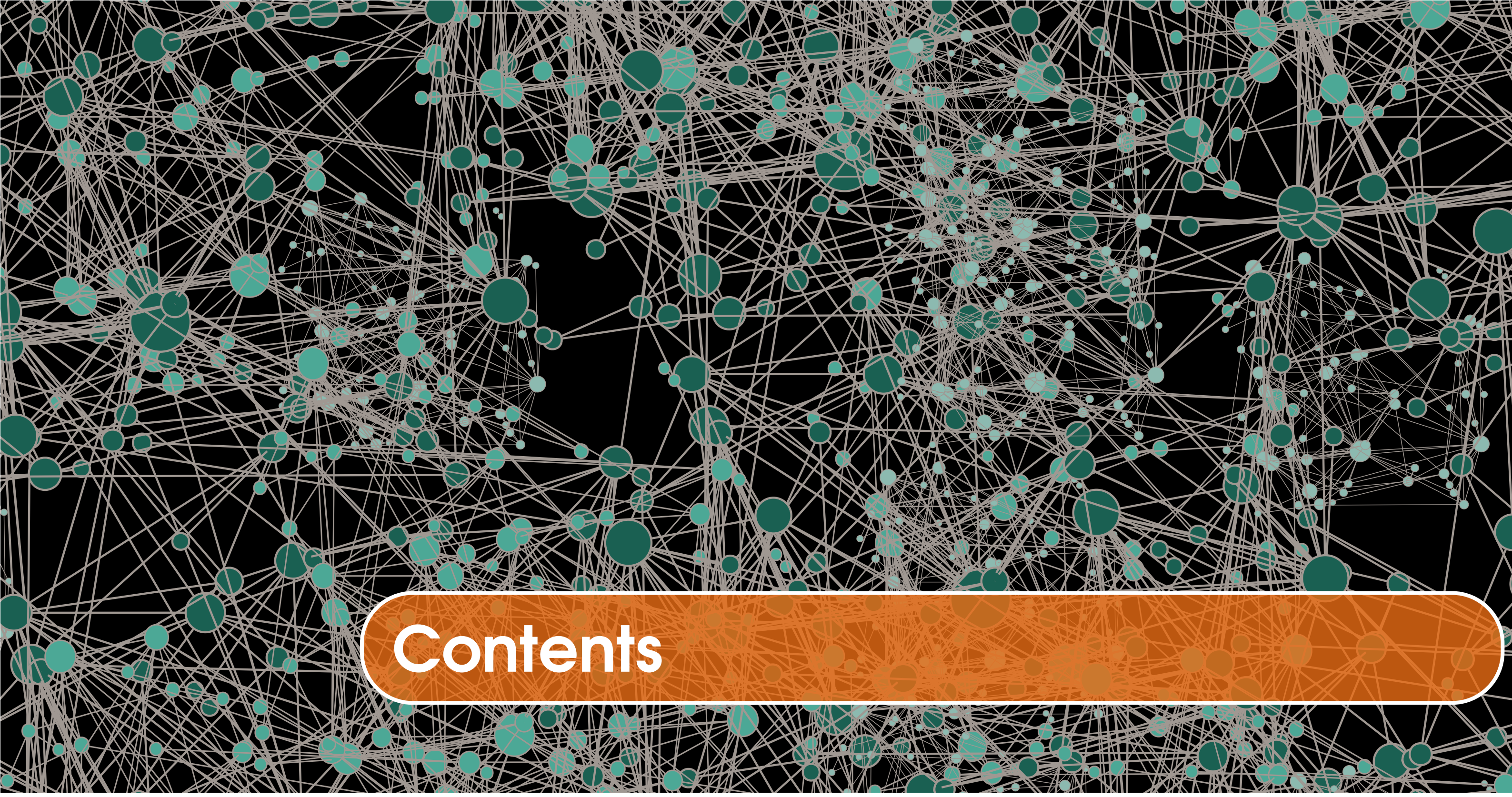

# Contents


1 Setup . . . . . . . . . . 7

1.1 Tutorial: Installing LinkPred 8

1.2 Tutorial: Compilation 9

2 Using the Simplified Interface . . . . . . . . . . 11

2.1 C++ 12

2.1.1 Tutorial: Computing all scores using the class `Simp::Predictor` . . . . . . 13

2.1.2 Tutorial: Computing scores of specific edges using the class `Simp::Predictor` 14

2.1.3 Tutorial: Computing top scores using the class `Simp::Predictor` . . . . . 15

2.1.4 Tutorial: Encoder-classifier prediction using the class `Simp::Predictor` 16

2.1.5 Tutorial: Encoder-similarity prediction using the class `Simp::Predictor` 18

2.1.6 Tutorial: Performance evaluation with automatically generated test data using the class `Simp::Evaluator` . . . . . . . . . . 20

2.1.7 Tutorial: Performance evaluation with pre-generated test data using the class `Simp::Evaluator` . . . . . . . . . . 23

2.1.8 Tutorial: Performance evaluation of external prediction results using the class `Simp::Evaluator` . . . . . . . . . . 25

2.2 Java 28

2.2.1 Tutorial: Computing all scores using the class `Predictor` . . . . . . . . . . 29

2.2.2 Tutorial: Computing scores of specific edges using the class `Predictor` 30

2.2.3 Tutorial: Computing top scores using the class `Predictor` . . . . . . . . . . 31

2.2.4 Tutorial: Encoder-classifier prediction using the class `Predictor` . . . . . 32

2.2.5 Tutorial: Encoder-similarity prediction using the class `Predictor` . . . . . 34

2.2.6 Tutorial: Performance evaluation with automatically generated test data using the class `Evaluator` . . . 36
2.2.7 Tutorial: Performance evaluation with pre-generated test data using the class `Evaluator` . . . 39
2.2.8 Tutorial: Performance evaluation of external prediction results using the class `Evaluator` . . . 41
**2.3 Python** **44**
2.3.1 Tutorial: Computing all scores using the class `Predictor` . . . 45
2.3.2 Tutorial: Computing scores of specific edges using the class `Predictor` 46
2.3.3 Tutorial: Computing top scores using the class `Predictor` . . . 47
2.3.4 Tutorial: Encoder-classifier prediction using the class `Predictor` . . . 48
2.3.5 Tutorial: Encoder-similarity prediction using the class `Predictor` . . . 50
2.3.6 Tutorial: Performance evaluation with automatically generated test data using the class `Evaluator` . . . 52
2.3.7 Tutorial: Performance evaluation with pre-generated test data using the class `Evaluator` . . . 54
2.3.8 Tutorial: Performance evaluation of external prediction results using the class `Evaluator` . . . 56

**3 Core Components . . . 59**
**3.1 Representing undirected networks using the class `UNetwork`** **60**
3.1.1 Tutorial: Reading a network from file and printing it . . . 61
3.1.2 Tutorial: Building a network . . . 62
3.1.3 Tutorial: Accessing network information . . . 63
**3.2 Representing directed networks using the class `DNetwork`** **68**
3.2.1 Tutorial: Reading a network from file and printing it . . . 69
3.2.2 Tutorial: Building a network . . . 70
3.2.3 Tutorial: Accessing network information . . . 71
**3.3 Maps** **76**
3.3.1 Tutorial: Node maps . . . 77
3.3.2 Tutorial: Edge maps . . . 78

**4 Graph Algorithms . . . 81**
**4.1 Traversing a network** **82**
4.1.1 Tutorial: Traverse a network in BFS . . . 83
4.1.2 Tutorial: Traverse a network in DFS . . . 85
**4.2 Shortest paths** **86**
4.2.1 Tutorial: Finding the shortest path between two nodes . . . 87
4.2.2 Tutorial: Computing the distance from one node to all nodes . . . 88
4.2.3 Tutorial: Memory management when computing distances . . . 90
**4.3 Network embedding** **92**
4.3.1 Tutorial: Embed a network using the HMSM encoder . . . 93
4.3.2 Tutorial: Embed a network using the LINE encoder . . . 95

4.3.3 Tutorial: Embed a network using the Node2Vec encoder . . . . . . . . . . 97

5 Predictors . . . . . . . . . . . . . . . . . . . . . . . . . . . . . . . . . . . . . . . . . . . . 99

**5.1 Link prediction algorithms in undirected networks 100**

5.1.1 Tutorial: Computing all scores . . . . . . . . . . . . . . . . . . . . . . . . . . . . . . . . 101
5.1.2 Tutorial: Computing scores of specific edges . . . . . . . . . . . . . . . . . . . . 103
5.1.3 Tutorial: Computing top scores . . . . . . . . . . . . . . . . . . . . . . . . . . . . . . . 105
5.1.4 Tutorial: Encoder-classifier prediction . . . . . . . . . . . . . . . . . . . . . . . . . 107
5.1.5 Tutorial: Encoder-similarity prediction . . . . . . . . . . . . . . . . . . . . . . . . . 109

**5.2 Link prediction algorithms in directed networks 111**

5.2.1 Tutorial: Computing all scores . . . . . . . . . . . . . . . . . . . . . . . . . . . . . . . . 112
5.2.2 Tutorial: Computing scores of specific edges . . . . . . . . . . . . . . . . . . . . 114
5.2.3 Tutorial: Computing top scores . . . . . . . . . . . . . . . . . . . . . . . . . . . . . . . 116

6 Performance Evaluation . . . . . . . . . . . . . . . . . . . . . . . . . . . . . . 119

**6.1 Data setup 120**

6.1.1 Tutorial: Creating test data by removing edges . . . . . . . . . . . . . . . . . 121
6.1.2 Tutorial: Creating test data by adding edges . . . . . . . . . . . . . . . . . . . 124
6.1.3 Tutorial: Loading test data obtained by removing edges . . . . . . . . . 127
6.1.4 Tutorial: Loading test data obtained by adding edges . . . . . . . . . . . 130

**6.2 Performance evaluation 133**

6.2.1 Tutorial: Using performance measures . . . . . . . . . . . . . . . . . . . . . . . . 134
6.2.2 Tutorial: Using the class `PerfEvaluator` for performance evaluation . 136
6.2.3 Tutorial: Using the class `PerfEvalExp` for performance evaluation . . . 138

7 Parallelism . . . . . . . . . . . . . . . . . . . . . . . . . . . . . . . . . . . . . . . . . . . 141

**7.1 Shared memory parallelism 142**

7.1.1 Tutorial: Computing the score of all negative links in parallel . . . . . . 143
7.1.2 Tutorial: Computing the top edge scores in parallel . . . . . . . . . . . . . . 145

**7.2 Distributed memory parallelism 147**

7.2.1 Tutorial: Computing the top edge scores distributively . . . . . . . . . . . . 148
7.2.2 Tutorial: Computing the area under the ROC curve distributively . . 150

# 1. Setup

This chapter contains two tutorials, the first on how to install LinkPred, and the second explains the compilation process of programs that use LinkPred.

## 1.1 Tutorial: Installing LinkPred

This tutorial shows the installation steps under Linux:

1. Get the latest version of the library from Github: https://github.com/kerrache/linkpred
2. Make sure you have all required softwares. See the user guide Section 1.3.
3. Create a build directory in the root of the LinkPred source directory:

```
$ mkdir build
```

4. Configure the library:

```
$ cd build
$ cmake ../
```

> **R** Build options can be set by editing the file `CMakeLists.txt` or through the user interface if GUI CMake is used.

5. If you do not need Java and Python bindings, you can disable the option in `CMakeLists.txt` (search for `LINKPRED_WITH_BINDINGS`). Note that finding Python requires a recent version of cmake. However, even if cmake fails to configure the bindings, the library can still be built successfully.
6. Build the library:

```
$ make
```

7. Build documentation (optional): this step requires Doxygen and generates documentation in HTML and Latex:

```
$ make doc
```

8. If you want to install the library:

```
$ make install
```

To install the library system-wide, you may need root privilege:

```
$ sudo make install
```

If you prefer a local install instead (which is usually the case when working on institution-wide HPC clusters/supercomputers), you need to set the install directory in the configuration step:

```
$ cmake -DCMAKE_INSTALL_PREFIX=YOUR_PATH ../
```

9. You may need to refresh ld cache by running:

```
$ sudo ldconfig
```

10. If you install the library in a non-default path, you will need to add that path to the environment variable `LD_LIBRARY_PATH`.

## 1.2 Tutorial: Compilation

This tutorial shows how to compile your code and link it with LinkPred. We assume that the library was successfully installed in the default directory.

1. In a file named `cne.cpp` copy the following code:

```
#include <linkpred.hpp>
#include <iostream>
using namespace LinkPred;
int main() {
  auto net = UNetwork<>::read("Infectious.edges");
  UCNEPredictor<> predictor(net);
  predictor.init();
  predictor.learn();
  std::cout << "#Start\tEnd\tScore\n";
  for (auto it=net->nonEdgesBegin();it!=net->nonEdgesEnd();++
     it){
    auto i = net->getLabel(net->start(*it));
    auto j = net->getLabel(net->end(*it));
    double sc = predictor.score(*it);
    std::cout << i << "\t" << j << "\t" << sc << std::endl;
  }
  return 0;
}
```

   R This code is available in: `tutorials/code/setup/compilation`

2. Compile your code. For example, if you compiled LinkPred with MPI an OpenMP enabled:

```
$ mpiCC cne.cpp -o cne -fopenmp -lLinkPred
```

   R This assumes using a recent compiler. If you face any dialect-related complaints from the compiler, you may need to add the option: `-std=c++14`. Also, depending on the LinkPred functionalities used in your code, you may need to additionally link against the MKL library (using `-lmkl_rt`) and/or gsl (using `-lgsl -lgslcblas`).

   If you built LinkPred without MPI and OpenMP, compile as follows:

```
$ g++ cne.cpp -o cne -lLinkPred
```

3. If you face errors in compilation, such as linkPred headers not found, you can specify the path to the headers using the `-I` option.
4. If the linker cannot find the library, you may specify its path using `-L` option or by using the environment variable `LIBRARY_PATH`.
5. Run your code:

```
$ ./cne
```

   The first few lines of this programs' output are as follows:

```
#Start  End  Score
100  10   0
100  11   0
100  113  7
```

```
100 12  0
100 13  0
100 14  0
100 15  0
100 16  0
100 107 10
...
```

R Make sure that the library is located in the load path. Under Linux, you may need to set the environment variable `LD_LIBRARY_PATH`. In the case of a default system-wide install, LinkPred will be installed to the default directory, which is already in the load path. You may, however, need to refresh the ld cache by running:

```
$ sudo ldconfig
```

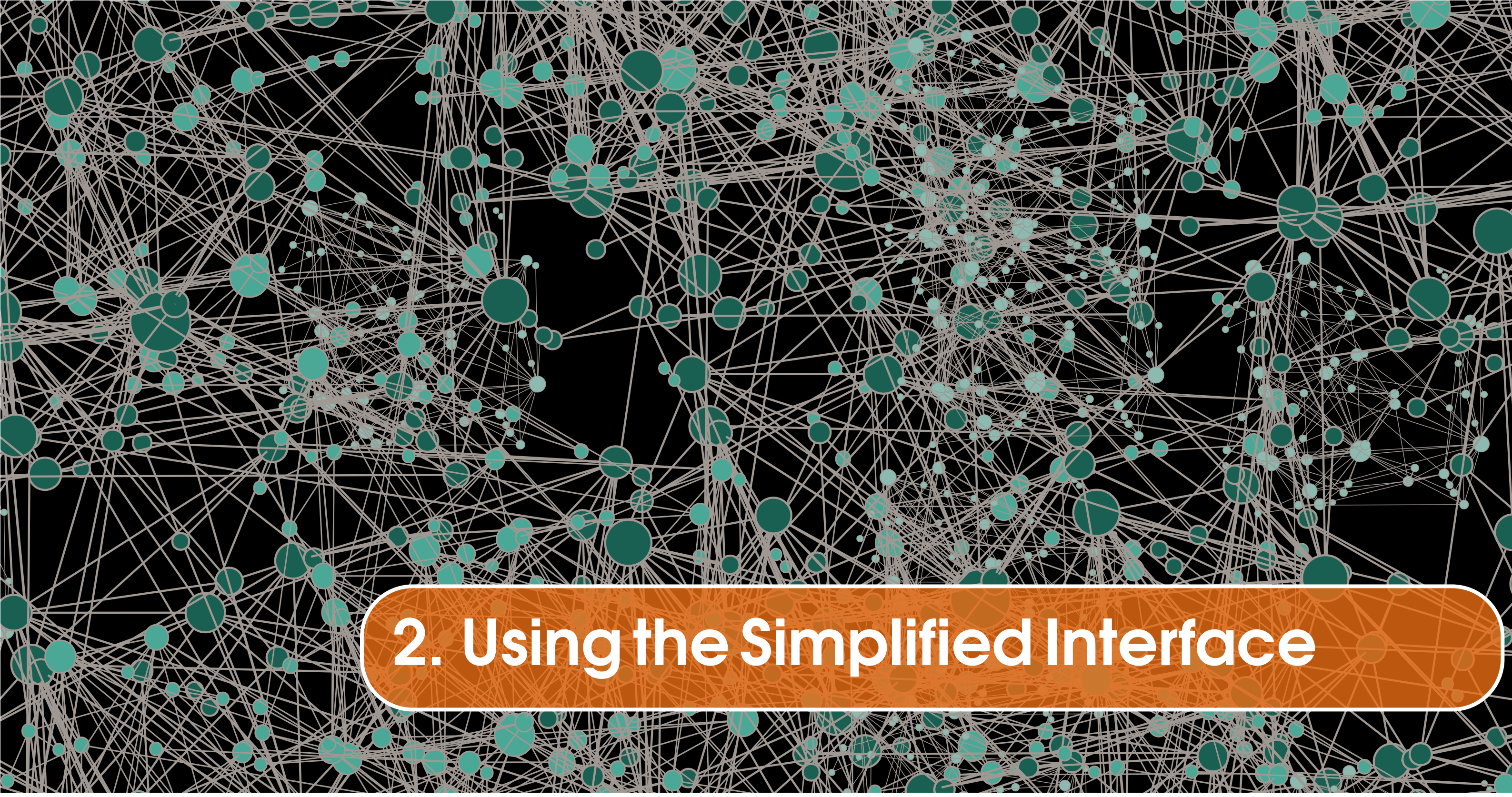

# 2. Using the Simplified Interface

The easiest and fastest way to start using `linkPred` is by using its simplified interface, that is, the classes available under the namespace `LinkPred::Simp`. This chapter contains tutorials on using this simplified interface in C++, Java, and Python.

## 2.1 C++

The two main classes in the namepsace are `Simp::Predictor` and `Simp::Evaluator`. This sections contains several tutorials showing how to uses these classes for predicting links and evaluating performance.

### 2.1.1 Tutorial: Computing all scores using the class `Simp::Predictor`

This tutorial shows how to use the simplified interface, more precisely `Simp::Predictor` to compute the scores of all non-existing edges in a network. For more information on the simplified interface, consult Chapter 2 of the user guide.

1. In a file named `pred-all.cpp` write the following code:

```
#include <linkpred.hpp>
#include <iostream>
using namespace LinkPred::Simp;
int main() {
  // Create a prtedictor object
  Predictor p;
  // Load network from file
  p.loadnet("Zakarays_Karate_Club.edges");
  // Predict the score of all non-existing edges using Adamic
      Adar index
  std::vector<EdgeScore> esv = p.predAllADA();
  // Print the scores
  for (auto it = esv.begin(); it != esv.end(); ++it) {
    std::cout << it->i << "\t" << it->j << "\t" << it->score
        << std::endl;
  }
  return 0;
}
```

> **R** This code is available in: `tutorials/code/simp/cpp/predAll`

2. Compile your code:

```
$ mpiCC predAll.cpp -o predAll -fopenmp -lLinkPred
```

> **R** Check Tutorial 1.2 if you face any compilation issues.

3. Run your code:

```
$ ./predAll
```

The first few lines of this programs' output are as follows:

```
1       31      1.07645
1       10      0.434294
1       28      0.434294
1       29      0.992405
1       33      1.61374
1       17      1.4427
1       34      2.71102
1       15      0
1       16      0
1       19      0
...
```

### 2.1.2 Tutorial: Computing scores of specific edges using the class `Simp::Predictor`

This tutorial shows how to use the simplified interface, more precisely `Simp::Predictor` to compute the scores of specific edges. For more information on the simplified interface, consult Chapter 2 of the user guide.

1. In a file named `pred.cpp` write the following code:

```
#include <linkpred.hpp>
#include <iostream>
using namespace LinkPred::Simp;
int main() {
  // Create a prtedictor object
  Predictor p;
  // Load network from file
  p.loadnet("Zakarays_Karate_Club.edges");
  // Compute the score for the two edges (1, 34) and (26,34)
  std::vector<EdgeScore> esv = {{"1","34"},{"26","34"}};
  p.predADA(esv);
  // Print the scores
  for (auto it = esv.begin(); it != esv.end(); ++it) {
    std::cout << it->i << "\t" << it->j << "\t" << it->score
        << std::endl;
  }
  return 0;
}
```

   R This code is available in: `tutorials/code/simp/cpp/pred`

   R The edges to be predicted are passed using the nodes' labels (as they appear in the network file) and are of type std::string.

2. Compile your code:

```
$ mpiCC pred.cpp -o pred -fopenmp -lLinkPred
```

   R Check Tutorial 1.2 if you face any compilation issues.

3. Run your code:

```
$ ./pred
```

   The output of this program is as follows:

```
1       34      2.71102
26      34      1.17945
```

### 2.1.3 Tutorial: Computing top scores using the class `Simp::Predictor`

This tutorial shows how to use the simplified interface, more precisely `Simp::Predictor` to compute the top edge scores. For more information on the simplified interface, consult Chapter 2 of the user guide.

1. In a file named `predTop.cpp` write the following code:

```
#include <linkpred.hpp>
#include <iostream>
using namespace LinkPred::Simp;
int main() {
  int k = 10; // Find top 10
  // Create a prtedictor object
  Predictor p;
  // Load network from file
  p.loadnet("Zakarays_Karate_Club.edges");
  // Predict the top k edges using Adamic Adar index
  std::vector<EdgeScore> esv = p.predTopADA(k);
  // Print the scores
  for (auto it = esv.begin(); it != esv.end(); ++it) {
    std::cout << it->i << "\t" << it->j << "\t" << it->score
        << std::endl;
  }
  return 0;
}
```

R This code is available in: `tutorials/code/simp/cpp/predTop`

2. Compile your code:

```
        $ mpiCC predTop.cpp -o predTop -fopenmp -lLinkPred
```

R Check Tutorial 1.2 if you face any compilation issues.

3. Run your code:

```
$ ./predTop
```

The output of this program is as follows:

```
1       33      1.61374
1       34      2.71102
2       34      2.25292
3       32      1.67334
3       34      4.71938
5       6       1.99226
7       11      1.99226
8       14      1.8082
32      24      1.66562
24      25      1.63159
```

### 2.1.4 Tutorial: Encoder-classifier prediction using the class `Simp::Predictor`

This tutorial shows how to predict links using an encoder-classifier using `Simp::Predictor`. For more information on the simplified interface, consult Chapter 2 of the user guide. For information on link prediction using graph-embedding methods consult Section 5.2.3.

1. In a file named `ecl.cpp` write the following code:

```
#include <linkpred.hpp>
#include <iostream>
using namespace LinkPred::Simp;
int main() {
  int k = 10;
  // Create a prtedictor object
  Predictor p;
  // Load network from file
  p.loadnet("Zakarays_Karate_Club.edges");
  // Predict top k scores using an encoder-classifier
     predictor with Node2Vec as encoder and logistic
     regression as classifier
  auto esv = p.predTopECL(k, "N2V", "LGR");
  // Print scores
  std::cout << "N2V-LGR\n";
  for (auto it = esv.begin(); it != esv.end(); ++it) {
    std::cout << it->i << "\t" << it->j << "\t" << it->score
        << std::endl;
  }
  // Predict top k scores using an encoder-classifier
     predictor with LINE as encoder and feed-forward neural
     network as classifier
  esv = p.predTopECL(k, "LIN", "FFN"); // FFN requires mlpack
  // Print scores
  std::cout << "LIN-FFN\n";
  for (auto it = esv.begin(); it != esv.end(); ++it) {
    std::cout << it->i << "\t" << it->j << "\t" << it->score
        << std::endl;
  }
  return 0;
}
```

   R This code is available in: `tutorials/code/simp/cpp/ecl`

   R For the names of available encoders and classifiers, consult the library documentation.

2. Compile your code:

```
$ mpiCC ecl.cpp -o ecl -fopenmp -lLinkPred
```

   R Check Tutorial 1.2 if you face any compilation issues.

3. Run your code:

```
$ ./ecl
```

The output of this program is as follows:

```
N2V-LGR
1       34      0.638526
9       11      0.637297
9       13      0.543905
9       14      0.55602
9       18      0.534107
32      31      0.615972
32      10      0.666815
32      28      0.506243
32      17      0.641181
33      17      0.667946
LIN-FFN
3       7       0.416399
3       13      0.402937
3       34      0.400492
11      13      0.430696
11      34      0.42946
11      24      0.380592
10      19      0.394202
10      24      0.437404
10      30      0.393994
15      24      0.396383
```

### 2.1.5 Tutorial: Encoder-similarity prediction using the class `Simp::Predictor`

This tutorial shows how to predict links using an encoder-similarity using `Simp::Predictor`. For more information on the simplified interface, consult Chapter 2 of the user guide. For information on link prediction using graph-embedding methods consult Section 5.2.3.

1. In a file named `esm.cpp` write the following code:

```
#include <linkpred.hpp>
#include <iostream>
using namespace LinkPred::Simp;
int main() {
  int k = 10;
  // Create a prtedictor object
  Predictor p;
  // Load network from file
  p.loadnet("Zakarays_Karate_Club.edges");
  // Predict top k scores using an encoder-classifier
     predictor with Node2Vec as encoder and L2 similarity
  auto esv = p.predTopESM(k, "N2V", "L2");
  // Print scores
  std::cout << "N2V-L2\n";
  for (auto it = esv.begin(); it != esv.end(); ++it) {
    std::cout << it->i << "\t" << it->j << "\t" << it->score
       << std::endl;
  }
  // Predict top k scores using an encoder-similarity measure
      predictor with LINE as encoder and cosine similarity
  esv = p.predTopESM(k, "LIN", "CSM");
  // Print scores
  std::cout << "LIN-CSM\n";
  for (auto it = esv.begin(); it != esv.end(); ++it) {
    std::cout << it->i << "\t" << it->j << "\t" << it->score
       << std::endl;
  }
  return 0;
}
```

   R This code is available in: `tutorials/code/simp/cpp/esm`

   R For the names of available encoders and similarity measures, consult the library documentation.

2. Compile your code:

```
$ mpiCC esm.cpp -o esm -fopenmp -lLinkPred
```

   R Check Tutorial 1.2 if you face any compilation issues.

3. Run your code:

```
$ ./esm
```

   The output of this program is as follows:

```
N2V-L2
13      14      -0.552179
13      18      -0.537708
18      22      -0.414516
15      16      -0.394946
15      21      -0.431844
15      23      -0.533414
16      21      -0.388185
16      23      -0.374324
19      21      -0.471841
21      23      -0.385193
LIN-CSM
1       21      0.620443
2       5       0.693219
9       13      0.702609
12      19      0.621401
14      30      0.688929
20      21      0.708784
22      21      0.609064
31      30      0.905744
10      15      0.617589
16      25      0.674067
```

### 2.1.6 Tutorial: Performance evaluation with automatically generated test data using the class `Simp::Evaluator`

This tutorial shows how to evaluate the performance of link prediction algorithm with test data automatically generated from a ground-truth network using `Simp::Evaluator`. For more information on the simplified interface, consult Chapter 2 of the user guide.

1. In a file named `eval-auto.cpp` write the following code:

```cpp
#include <linkpred.hpp>
#include <iostream>
using namespace LinkPred::Simp;
int main() {
  int nbRuns = 10;
  double edgeRemRatio = 0.1;
  // Create an evaluator object
  Evaluator eval;
  // Add predictors to be evaluated
  eval.addCNE();
  eval.addADA();
  eval.addKAB();
  // Add performance measures
  eval.addROC();
  eval.addTPR();
  // Run experiment on the specified network
  eval.run("Zakarays_Karate_Club.edges", nbRuns, edgeRemRatio
     );
  return 0;
}
```

   R This code is available in: `tutorials/code/simp/cpp/eval-auto`

2. Compile your code:

```
        $ mpiCC eval-auto.cpp -o eval-auto -fopenmp -
          lLinkPred
```

   R Check Tutorial 1.2 if you face any compilation issues.

3. Run your code:

```
        $ ./eval-auto
```

   The output of this program is as follows:

```
# n: 34 m: 78
#ratio  ROCADA  ROCCNE  ROCKAB  TPRADA  TPRCNE  TPRKAB
0.10    0.7737  0.7149  0.8280  0.1250  0.1932  0.1250
0.10    0.6593  0.6333  0.7030  0.1250  0.0000  0.1250
0.10    0.5967  0.5762  0.6095  0.1875  0.1818  0.2500
0.10    0.8464  0.7913  0.9343  0.1875  0.1290  0.3750
0.10    0.8324  0.7785  0.8967  0.1250  0.1750  0.1250
0.10    0.7240  0.6953  0.7547  0.0000  0.2222  0.0000
0.10    0.6753  0.6610  0.7262  0.0000  0.1591  0.1250
0.10    0.6048  0.5792  0.6672  0.0000  0.0000  0.0000
0.10    0.7627  0.7547  0.7808  0.2917  0.3194  0.3750
0.10    0.6442  0.5835  0.6727  0.1250  0.1250  0.1250
#Time: 97.2945 ms
```

4. The output above is a trace generated from within the evaluation method. You may also access the results as follows. n a file name `eval-auto-print.cpp` write the following code:

```
#include <linkpred.hpp>
#include <iostream>
using namespace LinkPred::Simp;
int main() {
  int nbRuns = 10;
  double edgeRemRatio = 0.1;
  // Create an evaluator object
  Evaluator eval;
  // Add predictors to be evaluated
  eval.addCNE();
  eval.addADA();
  eval.addKAB();
  // Add performance measures
  eval.addROC();
  eval.addTPR();
  // Run experiment on the specified network
  eval.run("Zakarays_Karate_Club.edges", nbRuns, edgeRemRatio
     );
  // Print the header row
  auto res = eval.getPerfRes(0);
  for (auto it =  res.begin(); it != res.end(); ++it) {
    std::cout << it->name << "\t" ;
  }
  std::cout << "\n";
  // Print the results of each iteration
  for(int i = 0; i < nbRuns; i++) {
    auto res = eval.getPerfRes(i);
    for (auto it =  res.begin(); it != res.end(); ++it) {
      std::cout << it->res << "\t";
    }
    std::cout << "\n";
  }
  return 0;
}
```

R This code is available in: `tutorials/code/simp/cpp/eval-auto`

5. Compile your code:

```
        $ mpiCC eval-auto-print.cpp -o eval-auto-print -
           fopenmp -lLinkPred
```

6. Run your code:

```
        $ ./eval-auto-print
```

The output of this program is as follows:

```
# n: 34 m: 78
#ratio  ROCADA  ROCCNE  ROCKAB  TPRADA  TPRCNE  TPRKAB
0.10    0.7737  0.7149  0.8280  0.1250  0.1932  0.1250
0.10    0.6593  0.6333  0.7030  0.1250  0.0000  0.1250
0.10    0.5967  0.5762  0.6095  0.1875  0.1818  0.2500
```

```
0.10    0.8464  0.7913  0.9343  0.1875  0.1290  0.3750
0.10    0.8324  0.7785  0.8967  0.1250  0.1750  0.1250
0.10    0.7240  0.6953  0.7547  0.0000  0.2222  0.0000
0.10    0.6753  0.6610  0.7262  0.0000  0.1591  0.1250
0.10    0.6048  0.5792  0.6672  0.0000  0.0000  0.0000
0.10    0.7627  0.7547  0.7808  0.2917  0.3194  0.3750
0.10    0.6442  0.5835  0.6727  0.1250  0.1250  0.1250
#Time: 91.2499 ms
ROCADA  ROCCNE  ROCKAB  TPRADA  TPRCNE  TPRKAB
0.7737  0.7149  0.8280  0.1250  0.1932  0.1250
0.6593  0.6333  0.7030  0.1250  0.0000  0.1250
0.5967  0.5762  0.6095  0.1875  0.1818  0.2500
0.8464  0.7913  0.9343  0.1875  0.1290  0.3750
0.8324  0.7785  0.8967  0.1250  0.1750  0.1250
0.7240  0.6953  0.7547  0.0000  0.2222  0.0000
0.6753  0.6610  0.7262  0.0000  0.1591  0.1250
0.6048  0.5792  0.6672  0.0000  0.0000  0.0000
0.7627  0.7547  0.7808  0.2917  0.3194  0.3750
0.6442  0.5835  0.6727  0.1250  0.1250  0.1250
```

### 2.1.7 Tutorial: Performance evaluation with pre-generated test data using the class `Simp::Evaluator`

This tutorial shows how to evaluate the performance of link prediction algorithm with pre-generated test data using `Simp::Evaluator`. For more information on the simplified interface, consult Chapter 2 of the user guide.

1. In a file named `eval-pregenerated.cpp` write the following code:

```
#include <linkpred.hpp>
#include <iostream>
using namespace LinkPred::Simp;
int main() {
  // Create an evaluator object
  Evaluator eval;
  // Add predictors to be evaluated
  eval.addADA();
  eval.addRAL();
  // Add performance measures
  eval.addPR();
  eval.addTPR();
  // Run experiment on the specified network
  eval.run("Zakarays_Karate_Club_Train.edges", "
     Zakarays_Karate_Club_Test.edges");
  return 0;
}
```

   The file `Zakarays_Karate_Club_Train.edges` contains the set of observed edges, whereas `Zakarays_Karate_Club_Test.edges` contains the set of edges that have been removed, i.e. the test set.

   R This code is available in: `tutorials/code/simp/cpp/eval-pregenerated`

2. Compile your code:

```
$ mpiCC eval-pregenerated.cpp -o eval-pregenerated -fopenmp -
   lLinkPred
```

   R Check Tutorial 1.2 if you face any compilation issues.

3. Run your code:

```
$ ./eval-pregenerated
```

   The output of this program is as follows:

```
PRADA   PRRAL   TPRADA  TPRRAL
0.1561  0.1568  0.1250  0.1250
```

4. The output above is a trace generated from within the evaluation method. You may also access the results as follows. In a file named `eval-pregenerated-print.cpp` write the following code:

```
#include <linkpred.hpp>
#include <iostream>
using namespace LinkPred::Simp;
int main() {
  // Create an evaluator object
```

```
  Evaluator eval;
  // Add predictors to be evaluated
  eval.addADA();
  eval.addRAL();
  // Add performance measures
  eval.addPR();
  eval.addTPR();
  // Run experiment on the specified network
  eval.run("Zakarays_Karate_Club_Train.edges", "
     Zakarays_Karate_Club_Test.edges");
  auto res = eval.getPerfRes(0);
  for (auto it =  res.begin(); it != res.end(); ++it) {
    std::cout << it->name << "\t" ;
  }
  std::cout << "\n";
  for (auto it =  res.begin(); it != res.end(); ++it) {
    std::cout << it->res << "\t" ;
  }
  std::cout << "\n";
  return 0;
}
```

R This code is available in: `tutorials/code/simp/cpp/eval-pregenerated`

5. Compile your code:

```
$ mpiCC eval-pregenerated-print.cpp -o eval-pregenerated-
    print -fopenmp -lLinkPred
```

6. Run your code:

```
$ ./eval-pregenerated-print
```

The output of this program is as follows:

```
PRADA   PRRAL   TPRADA  TPRRAL
0.1561  0.1568  0.1250  0.1250
PRADA   PRRAL   TPRADA  TPRRAL
0.1561  0.1568  0.1250  0.1250
```

### 2.1.8 Tutorial: Performance evaluation of external prediction results using the class `Simp::Evaluator`

This tutorial shows how to evaluate external link prediction results obtained by a user link prediction algorithm. For more information on the simplified interface, consult Chapter 2 of the user guide.

> **R** It is possible to implement new link prediction algorithms and integrate them into LinkPred, which allows for better use of the library's performance evaluation routines. See Section 5.4 of the user guide for more details.

1. The first step consists in generating test data. If you already have a ready test data, split into training and test sets, you can skip this part (go directly to Step 5 of this tutorial).
2. In a file name `gen-data.cpp` write the following code:

```
#include <linkpred.hpp>
#include <iostream>
using namespace LinkPred::Simp;
int main() {
  // We remove 10% of the edges
  double edgeRemRatio = 0.1;
  // We will not keep the network connected when removing
     edges
  bool keepConnected = false;
  // Seed of the random number generator
  long int seed = 0;
  // Create an Evaluator object
  Evaluator eval;
  // The ground truth network "Zakarays_Karate_Club.edges" is
      split into an observed network stored in "
     Zakarays_Train.edges" and a list of removed edges stored
      in "Zakarays_Test.edges"
  eval.genTestData("Zakarays_Karate_Club.edges", "
     Zakarays_Train.edges", "Zakarays_Test.edges",
     edgeRemRatio, keepConnected, seed);
  return 0;
}
```

   The file `Zakarays_Train.edges` will contain the set of observed edges, whereas `Zakarays_Test.edges` will contain the set of edges that have been removed, i.e. the test set.
3. Compile your code:

```
$ mpiCC gen-data.cpp -o gen-data -fopenmp -lLinkPred
```

   > **R** Check Tutorial 1.2 if you face any compilation issues.

4. Run your code:

```
$ ./gen-data
```

**R** The method `addPST` is used to load the pre-stored results by internally creating a `PST` predictor. For more details about this predictor consult Section 5.2.4 of the user guide.

The first few lines of `Zakarays_Train.edges` and the file `Zakarays_Test.edges` generated by this program are as follows:

```
# First few lines of the file Zakarays_Train.edges
1       2
1       3
1       4
1       5
1       6
1       7
1       8
1       9
1       11
...
# The file Zakarays_Test.edges
2       22
3       4
24      26
33      24
9       34
32      33
33      30
28      25
```

5. At this stage, we have two files: `Zakarays_Train.edges` which contains the set of observed edges and `Zakarays_Test.edges` which contains the set of removed edges. Train your algorithm on the file `Zakarays_Train.edges` and computed the scores of all non-existing links in that network. This includes the edges that have been removed (contained in `Zakarays_Test.edges`) and those that are absent from the ground truth network (true negative links). Store the scores in a file name "pst.txt" in the following format (the first line is a comment and can be safely omitted):

```
#Start  End     Score
1       31      0.41374
1       10      0.276687
1       28      0.283587
1       29      0.374494
1       33      0.463135
1       17      0.531863
1       34      0.49409
1       26      0.325087
1       25      0.325087
...
```

6. In a file name `eval-pst.cpp` write the following code:

```
#include <linkpred.hpp>
#include <iostream>
using namespace LinkPred::Simp;
int main() {
  // Create an evaluator object
  Evaluator eval;
  // Add predictors to be evaluated
```

```
    eval.addADA();
    // Use the method addPST to create a predictor that loads
       scores from pst.txt
    eval.addPST("PST", "pst.txt");
    eval.addRAL();
    // Add performance measures
    eval.addPR();
    eval.addTPR();
    // Run experiment on the specified network
    eval.run("Zakarays_Train.edges", "Zakarays_Test.edges");
    return 0;
}
```

7. Compile your code:

```
$ mpiCC eval-pst.cpp -o eval-pst -fopenmp -lLinkPred
```

> R Check Tutorial 1.2 if you face any compilation issues.

8. Run your code:

```
$ ./eval-pst
```

The output of this program is as follows:

```
PRADA   PRPST   PRRAL   TPRADA  TPRPST  TPRRAL
0.0510  0.0510  0.0391  0.1250  0.1250  0.1250
```

## 2.2 Java

LinkPred bindings are optional (by default on). To change this setting, edit the file `CMakeLists.txt` in the root of the library source directory (search for the option `LINKPRED_WITH_BINDINGS`). Upon successful building, the library `LinkPredJava` will be generated (named `libLinkPredJava.so` in Linux). This library will be loaded when running your program, and for that, it must be accessible to the Java virtual machine.

> **R** In Linux, you can make the library accessible to the JVM by including its path in the environment variable `LD_LIBRARY_PATH`. If LinkPred is installed in the default location, this can be accomplished using the following command:
>
> ```
> $ export LD_LIBRARY_PATH = $LD_LIBRARY_PATH:/usr/local/
>    lib
> ```

The Java proxy classes needed to interface with the library can be found in source form and JAR compiled form (`LinkPredJava.jar`) in the source directory of LinkPred in `/bindings/Java`. These classes (either in source or as JAR) must be included in the class path during compilation and at run-time.

### 2.2.1 Tutorial: Computing all scores using the class `Predictor`

This tutorial shows how to use the Java bindings of the simplified interface, more precisely the class `Predictor` to compute the scores of all non-existing edges in a network. For more information on the simplified interface, consult Chapter 2 of the user guide.

1. In a file named `PredAll.java` write the following code:

```
public class PredAll {
  static {
    // Load the library
    System.loadLibrary("LinkPredJava");
  }
  public static void main(String[] args) {
    // Create a prtedictor object
    Predictor p = new Predictor();
    // Load network from file
    p.loadnet("Zakarays_Karate_Club.edges");
    // Predict the score of all non-exisitng edges using
       Adamic Adar index
    EdgeScoreVec esv = p.predAllADA();
    // Print the scores
    for (int i = 0; i < esv.size(); i++) {
      EdgeScore es = esv.get(i);
      System.out.println(es.getI() + "\t" + es.getJ() + "\t"
          + es.getScore());
    }
  }
}
```

R This code is available in: `tutorials/code/simp/java/predAll`

2. Compile your code:

```
$ javac -cp .:./LinkPredJava.jar PredAll.java
```

3. Run your code:

```
$ java -cp .:./LinkPredJava.jar PredAll
```

The first few lines of this programs' output are as follows:

```
1       31      1.0764545478730305
1       10      0.43429448190325176
1       28      0.43429448190325176
1       29      0.9924051084544989
1       33      1.613740043014111
1       17      1.4426950408889634
1       34      2.7110197222973085
1       15      0.0
1       16      0.0
1       19      0.0
...
```

### 2.2.2 Tutorial: Computing scores of specific edges using the class `Predictor`

This tutorial shows how to use the Java bindings of the simplified interface, more precisely the class `Predictor` to compute the scores of specific edges. For more information on the simplified interface, consult Chapter 2 of the user guide.

1. In a file name `Pred.java` write the following code:

```
public class Pred {
  static {
    // Load the library
    System.loadLibrary("LinkPredJava");
  }
  public static void main(String[] args) {
    // Create a prtedictor object
    Predictor p = new Predictor();
    // Load network from file
    p.loadnet("Zakarays_Karate_Club.edges");
    // Compute the score for the two edges (1, 34) and
       (26,34)
    EdgeScoreVec esv = new EdgeScoreVec();
    EdgeScore es;
    es = new EdgeScore();
    es.setI("1");
    es.setJ("34");
    esv.add(es);
    es = new EdgeScore();
    es.setI("26");
    es.setJ("34");
    esv.add(es);
    p.predADA(esv);
    // Print the scores
    for (int i = 0; i < esv.size(); i++) {
      es = esv.get(i);
      System.out.println(es.getI() + "\t" + es.getJ() + "\t"
          + es.getScore());
    }
  }
}
```

R This code is available in: `tutorials/code/simp/java/pred`

2. Compile your code:

```
$ javac -cp .:./LinkPredJava.jar Pred.java
```

3. Run your code:

```
$ java -cp .:./LinkPredJava.jar Pred
```

The first few lines of this programs' output are as follows:

```
1       34      2.7110197222973085
26      34      1.179445561110859
```

### 2.2.3 Tutorial: Computing top scores using the class `Predictor`

This tutorial shows how to use the simplified interface, more precisely `Predictor` to compute the top edge scores. For more information on the simplified interface, consult Chapter 2 of the user guide.

1. In a file name `PredTop.java` write the following code:

```java
public class PredTop {
  static {
    // Load the library
    System.loadLibrary("LinkPredJava");
  }
  public static void main(String [] args) {
    int k = 10; // Find top 10
    // Create a prtedictor object
    Predictor p = new Predictor();
    // Load network from file
    p.loadnet("Zakarays_Karate_Club.edges");
    // Predict the top k edges using Adamic Adar index
    EdgeScoreVec esv = p.predTopADA(k);
    // Print the scores
    for (int i = 0; i < esv.size(); i++) {
      EdgeScore es = esv.get(i);
      System.out.println(es.getI() + "\t" + es.getJ() + "\t"
          + es.getScore());
    }
  }
}
```

R This code is available in: `tutorials/code/simp/java/predTop`

2. Compile your code:

```
$ javac -cp .:./LinkPredJava.jar PredTop.java
```

3. Run your code:

```
$ java -cp .:./LinkPredJava.jar PredTop
```

The first few lines of this programs' output are as follows:

```
1       33      1.613740043014111
1       34      2.7110197222973085
2       34      2.252921681630931
3       32      1.6733425912309228
3       34      4.719381261461351
5       6       1.9922605072935597
7       11      1.9922605072935597
8       14      1.8081984819901584
32      24      1.6656249548734432
24      25      1.631586747071319
```

### 2.2.4 Tutorial: Encoder-classifier prediction using the class `Predictor`

This tutorial shows how to predict links using an encoder-classifier using `Predictor`. For more information on the simplified interface, consult Chapter 2 of the user guide. For information on link prediction using graph-embedding methods consult Section 5.2.3.

1. In a file name `ECL.java` write the following code:

```java
public class ECL {
  static {
    // Load the library
    System.loadLibrary("LinkPredJava");
  }
  public static void main(String[] args) {
    int k = 10;
    // Create a prtedictor object
    Predictor p = new Predictor();
    // Load network from file
    p.loadnet("Zakarays_Karate_Club.edges");
    // Predict top k scores using an encoder-classifier
       predictor with Node2Vec as encoder and logistic
       regression as classifier
    EdgeScoreVec esv = p.predTopECL(k, "N2V", "LGR");
    // Print scores
    System.out.println("N2V-LGR");
    for (int i = 0; i < esv.size(); i++) {
      EdgeScore es = esv.get(i);
      System.out.println(es.getI() + "\t" + es.getJ() + "\t"
         + es.getScore());
    }
    // Predict top k scores using an encoder-classifier
       predictor with LINE as encoder and feed-forward neural
        network as classifier
    esv = p.predTopECL(k, "LIN", "FFN"); // FFN requires
       mlpack
    // Print scores
    System.out.println("LIN-FFN");
    for (int i = 0; i < esv.size(); i++) {
      EdgeScore es = esv.get(i);
      System.out.println(es.getI() + "\t" + es.getJ() + "\t"
         + es.getScore());
    }
  }
}
```

R This code is available in: `tutorials/code/simp/java/ecl`

R For the names of available encoders and classifiers, consult the library reference manual.

2. Compile your code:

```
$ javac -cp .:./LinkPredJava.jar ECL.java
```

3. Run your code:

```
$ java -cp .:./LinkPredJava.jar ECL
```

The output of this program is as follows:

```
N2V-LGR
1       34      0.6385261305821565
9       11      0.6372967584173628
9       13      0.5439045029916033
9       14      0.5560201995865949
9       18      0.5341066259961504
32      31      0.6159717145419226
32      10      0.6668148165127163
32      28      0.5062429834583236
32      17      0.6411806541242171
33      17      0.6679461676338299
LIN-FFN
3       7       0.4163985044250176
3       13      0.40293727072886226
3       34      0.40049178211918146
11      13      0.4306964396488587
11      34      0.42945998491801923
11      24      0.380591821298675
10      19      0.3942022523784909
10      24      0.43740409522018153
10      30      0.3939938517366693
15      24      0.3963832972307969
```

### 2.2.5 Tutorial: Encoder-similarity prediction using the class `Predictor`

This tutorial shows how to predict links using an encoder-similarity using `Predictor`. For more information on the simplified interface, consult Chapter 2 of the user guide. For information on link prediction using graph-embedding methods consult Section 5.2.3.

1. In a file name `ESM.java` write the following code:

```
public class ESM {
  static {
    // Load the library
    System.loadLibrary("LinkPredJava");
  }
  public static void main(String[] args) {
    int k = 10;
    // Create a prtedictor object
    Predictor p = new Predictor();
    // Load network from file
    p.loadnet("Zakarays_Karate_Club.edges");
    // Predict top k scores using an encoder-classifier
       predictor with Node2Vec as encoder and L2 similarity
    EdgeScoreVec esv = p.predTopESM(k, "N2V", "L2");
    // Print scores
    System.out.println("N2V-L2");
    for (int i = 0; i < esv.size(); i++) {
      EdgeScore es = esv.get(i);
      System.out.println(es.getI() + "\t" + es.getJ() + "\t"
         + es.getScore());
    }
    // Predict top k scores using an encoder-similarity
       measure predictor with LINE as encoder and cosine
       similarity
    esv = p.predTopESM(k, "LIN", "CSM");
    // Print scores
    System.out.println("LIN-CSM");
    for (int i = 0; i < esv.size(); i++) {
      EdgeScore es = esv.get(i);
      System.out.println(es.getI() + "\t" + es.getJ() + "\t"
         + es.getScore());
    }
  }
}
```

R This code is available in: `tutorials/code/simp/java/esm`

R For the names of available encoders and similarity measures, consult the library reference manual.

2. Compile your code:

```
$ javac -cp .:./LinkPredJava.jar ESM.java
```

3. Run your code:

```
$ java -cp .:./LinkPredJava.jar ESM
```

The output of this program is as follows:

```
N2V-L2
13      14      -0.5521786311666944
13      18      -0.5377083804555659
18      22      -0.41451576606008234
15      16      -0.39494585351335626
15      21      -0.43184372730810316
15      23      -0.533414287409406
16      21      -0.388184764773398
16      23      -0.3743242327498225
19      21      -0.4718410793861365
21      23      -0.3851929020614546
LIN-CSM
1       21      0.6204433804816786
2       5       0.6932189050437948
9       13      0.7026086708697828
12      19      0.6214013483476032
14      30      0.6889288481459729
20      21      0.708784319919729
22      21      0.6090643830619246
31      30      0.9057438074014044
10      15      0.6175888844497922
16      25      0.674066958588203
```

### 2.2.6 Tutorial: Performance evaluation with automatically generated test data using the class `Evaluator`

This tutorial shows how to evaluate the performance of link prediction algorithm with test data automatically generated from a ground-truth network using `Evaluator`. For more information on the simplified interface, consult Chapter 2 of the user guide.

1. In a file name `EvalAuto.java` write the following code:

```
public class EvalAuto {
  static {
    // Load the library
    System.loadLibrary("LinkPredJava");
  }
  public static void main(String[] args) {
    int nbRuns = 10;
    double edgeRemRatio = 0.1;
    // Create an evaluator object
    Evaluator eval = new Evaluator();
    // Add predictors to be evaluated
    eval.addCNE();
    eval.addADA();
    eval.addKAB();
    // Add performance measures
    eval.addROC();
    eval.addTPR();
    // Run experiment on the specified network
    eval.run("Zakarays_Karate_Club.edges", nbRuns,
        edgeRemRatio);
  }
}
```

R This code is available in: `tutorials/code/simp/java/eval-auto`

R For the names of available predictors and performance measures, consult the library reference manual.

2. Compile your code:

```
$ javac -cp .:./LinkPredJava.jar EvalAuto.java
```

3. Run your code:

```
$ java -cp .:./LinkPredJava.jar EvalAuto
```

The output of this program is as follows:

```
# n: 34 m: 78
#ratio  ROCADA  ROCCNE  ROCKAB  TPRADA  TPRCNE  TPRKAB
0.10    0.7737  0.7149  0.8280  0.1250  0.1932  0.1250
0.10    0.6593  0.6333  0.7030  0.1250  0.0000  0.1250
0.10    0.5967  0.5762  0.6095  0.1875  0.1818  0.2500
0.10    0.8464  0.7913  0.9343  0.1875  0.1290  0.3750
0.10    0.8324  0.7785  0.8967  0.1250  0.1750  0.1250
0.10    0.7240  0.6953  0.7547  0.0000  0.2222  0.0000
0.10    0.6753  0.6610  0.7262  0.0000  0.1591  0.1250
0.10    0.6048  0.5792  0.6672  0.0000  0.0000  0.0000
```

```
0.10    0.7627  0.7547  0.7808  0.2917  0.3194  0.3750
0.10    0.6442  0.5835  0.6727  0.1250  0.1250  0.1250
#Time: 93.0054 ms
```

4. The output above is a trace generated from within the evaluation method. You may also access the results as follows. In a file name `EvalAutoPrint.java` write the following code:

```java
public class EvalAutoPrint {
  static {
    // Load the library
    System.loadLibrary("LinkPredJava");
  }
  public static void main(String[] args) {
    int nbRuns = 10;
    double edgeRemRatio = 0.1;
    // Create an evaluator object
    Evaluator eval = new Evaluator();
    // Add predictors to be evaluated
    eval.addCNE();
    eval.addADA();
    eval.addKAB();
    // Add performance measures
    eval.addROC();
    eval.addTPR();
    // Run experiment on the specified network
    eval.run("Zakarays_Karate_Club.edges", nbRuns,
        edgeRemRatio);
    // Print the header row
    PerfResVec res = eval.getPerfRes(0);
    for (int j = 0; j < res.size(); j++) {
      System.out.print(res.get(j).getName() + "\t") ;
    }
    System.out.println();
    // Print the results of each iteration
    for(int i = 0; i < nbRuns; i++) {
      res = eval.getPerfRes(i);
      for (int j = 0; j < res.size(); j++) {
        System.out.printf("%.4f\t", res.get(j).getRes()) ;
      }
      System.out.println();
    }
  }
}
```

R This code is available in: `tutorials/code/simp/java/eval-auto`

5. Compile your code:

```
$ javac -cp .:./LinkPredJava.jar EvalAutoPrint.java
```

6. Run your code:

```
$ java -cp .:./LinkPredJava.jar EvalAutoPrint
```

The output of this program is as follows:

```
# n: 34 m: 78
```

```
#ratio  ROCADA  ROCCNE  ROCKAB  TPRADA  TPRCNE  TPRKAB
0.10    0.7737  0.7149  0.8280  0.1250  0.1932  0.1250
0.10    0.6593  0.6333  0.7030  0.1250  0.0000  0.1250
0.10    0.5967  0.5762  0.6095  0.1875  0.1818  0.2500
0.10    0.8464  0.7913  0.9343  0.1875  0.1290  0.3750
0.10    0.8324  0.7785  0.8967  0.1250  0.1750  0.1250
0.10    0.7240  0.6953  0.7547  0.0000  0.2222  0.0000
0.10    0.6753  0.6610  0.7262  0.0000  0.1591  0.1250
0.10    0.6048  0.5792  0.6672  0.0000  0.0000  0.0000
0.10    0.7627  0.7547  0.7808  0.2917  0.3194  0.3750
0.10    0.6442  0.5835  0.6727  0.1250  0.1250  0.1250
#Time: 86.0519 ms
ROCADA  ROCCNE  ROCKAB  TPRADA  TPRCNE  TPRKAB
0.7737  0.7149  0.8280  0.1250  0.1932  0.1250
0.6593  0.6333  0.7030  0.1250  0.0000  0.1250
0.5967  0.5762  0.6095  0.1875  0.1818  0.2500
0.8464  0.7913  0.9343  0.1875  0.1290  0.3750
0.8324  0.7785  0.8967  0.1250  0.1750  0.1250
0.7240  0.6953  0.7547  0.0000  0.2222  0.0000
0.6753  0.6610  0.7262  0.0000  0.1591  0.1250
0.6048  0.5792  0.6672  0.0000  0.0000  0.0000
0.7627  0.7547  0.7808  0.2917  0.3194  0.3750
0.6442  0.5835  0.6727  0.1250  0.1250  0.1250
```

### 2.2.7 Tutorial: Performance evaluation with pre-generated test data using the class `Evaluator`

This tutorial shows how to evaluate the performance of link prediction algorithm with pre-generated test data using `Evaluator`. For more information on the simplified interface, consult Chapter 2 of the user guide.

1. In a file name `EvalPregenerated.java` write the following code:

```
public class EvalPregenerated {
  static {
    // Load the library
    System.loadLibrary("LinkPredJava");
  }
  public static void main(String[] args) {
    // Create an evaluator object
    Evaluator eval = new Evaluator();
    // Add predictors to be evaluated
    eval.addADA();
    eval.addRAL();
    // Add performance measures
    eval.addPR();
    eval.addTPR();
    // Run experiment on the specified train and test set
    eval.run("Zakarays_Karate_Club_Train.edges", "
       Zakarays_Karate_Club_Test.edges");
  }
}
```

   The file `Zakarays_Karate_Club_Train.edges` contains the set of observed edges, whereas `Zakarays_Karate_Club_Test.edges` contains the set of edges that have been removed, i.e. the test set.

   R This code is available in: `tutorials/code/simp/java/eval-pregenerated`

2. Compile your code:

```
$ javac -cp .:./LinkPredJava.jar EvalPregenerated.java
```

3. Run your code:

```
$ java -cp .:./LinkPredJava.jar EvalPregenerated
```

   The output of this program is as follows:

```
PRADA   PRRAL   TPRADA  TPRRAL
0.1561  0.1568  0.1250  0.1250
```

4. The output above is a trace generated from within the evaluation method. You may also access the results as follows. In a file name `EvalPregeneratedPrint.java` write the following code:

```
public class EvalPregeneratedPrint {
  static {
    // Load the library
    System.loadLibrary("LinkPredJava");
  }
  public static void main(String[] args) {
```

```
    // Create an evaluator object
    Evaluator eval = new Evaluator();
    // Add predictors to be evaluated
    eval.addADA();
    eval.addRAL();
    // Add performance measures
    eval.addPR();
    eval.addTPR();
    // Run experiment on the specified train and test set
    eval.run("Zakarays_Karate_Club_Train.edges", "
       Zakarays_Karate_Club_Test.edges");
    // Print the header row
    PerfResVec res = eval.getPerfRes(0);
    for (int j = 0; j < res.size(); j++) {
      System.out.print(res.get(j).getName() + "\t") ;
    }
    System.out.println();
    // Print the results
    for (int j = 0; j < res.size(); j++) {
      System.out.printf("%.4f\t", res.get(j).getRes()) ;
    }
    System.out.println();
  }
}
```

R This code is available in: `tutorials/code/simp/java/eval-pregenerated`

5. Compile your code:

```
$ javac -cp .:./LinkPredJava.jar EvalPregeneratedPrint.java
```

6. Run your code:

```
$ java -cp .:./LinkPredJava.jar EvalPregeneratedPrint
```

The output of this program is as follows:

```
PRADA   PRRAL   TPRADA  TPRRAL
0.1561  0.1568  0.1250  0.1250
PRADA   PRRAL   TPRADA  TPRRAL
0.1561  0.1568  0.1250  0.1250
```

### 2.2.8 Tutorial: Performance evaluation of external prediction results using the class `Evaluator`

This tutorial shows how to evaluate external link prediction results obtained by a user link prediction algorithm. For more information on the simplified interface, consult Chapter 2 of the user guide.

> **R** It is possible to implement new link prediction algorithms and integrate them into LinkPred, which allows for better use of the library's performance evaluation routines. See Section 5.4 of the user guide for more details.

1. The first step consists in generating test data. If you already have a ready test data, split into training and test sets, you can skip this part (go directly to Step 5 of this tutorial).
2. In a file name `GenData.java` write the following code:

```
public class GenData {
  static {
    // Load the library
    System.loadLibrary("LinkPredJava");
  }
  public static void main(String[] args) {
    // We remove 10% of the edges
    double edgeRemRatio = 0.1;
    // We will not keep the network connected when removing
       edges
    boolean keepConnected = false;
    // Seed of the random number generator
    int seed = 0;
    // Create an Evaluator object
    Evaluator eval = new Evaluator();
    // The ground truth network "Zakarays_Karate_Club.edges"
       is split into an observed network stored in "
       Zakarays_Train.edges" and a list of removed edges
       stored in "Zakarays_Test.edges"
    eval.genTestData("Zakarays_Karate_Club.edges", "
       Zakarays_Train.edges", "Zakarays_Test.edges",
       edgeRemRatio, keepConnected, seed);
  }
}
```

   The file `Zakarays_Train.edges` will contain the set of observed edges, whereas `Zakarays_Test.edges` will contain the set of edges that have been removed, i.e. the test set.
3. Compile your code:

```
$ javac -cp .:./LinkPredJava.jar GenData.java
```

4. Run your code:

```
$ java -cp .:./LinkPredJava.jar GenData
```

> **R** The method `addPST` is used to load the pre-stored results by internally creating a `PST` predictor. For more details about this predictor consult Section 5.2.4 of the user guide.

The first few lines of `Zakarays_Train.edges` and the file `Zakarays_Test.edges` generated by this program are as follows:

```
# First few lines of the file Zakarays_Train.edges
1       2
1       3
1       4
1       5
1       6
1       7
1       8
1       9
1       11
...
# The file Zakarays_Test.edges
2       22
3       4
24      26
33      24
9       34
32      33
33      30
28      25
```

5. At this stage, we have two files: `Zakarays_Train.edges` which contains the set of observed edges and `Zakarays_Test.edges` which contains the set of removed edges. Train your algorithm on the file `Zakarays_Train.edges` and computed the scores of all non-existing links in that network. This includes the edges that have been removed (contained in `Zakarays_Test.edges`) and those that are absent from the ground truth network (true negative links). Store the scores in a file name "pst.txt" in the following format (the first line is a comment and can be safely omitted):

```
#Start  End     Score
1       31      0.41374
1       10      0.276687
1       28      0.283587
1       29      0.374494
1       33      0.463135
1       17      0.531863
1       34      0.49409
1       26      0.325087
1       25      0.325087
...
```

6. In a file name `EvalPST.java` write the following code:

```
public class EvalPST {
  static {
    // Load the library
    System.loadLibrary("LinkPredJava");
  }
  public static void main(String[] args) {
    // Create an evaluator object
    Evaluator eval = new Evaluator();
    // Add predictors to be evaluated
    eval.addADA();
```

```
    // Use the method addPST to create a predictor that loads
        scores from pst.txt
    eval.addPST("PST", "pst.txt");
    eval.addRAL();
    // Add performance measures
    eval.addPR();
    eval.addTPR();
    // Run experiment on the specified network
    eval.run("Zakarays_Train.edges", "Zakarays_Test.edges");
  }
}
```

7. Compile your code:

```
$ javac -cp .:./LinkPredJava.jar EvalPST.java
```

8. Run your code:

```
$ java -cp .:./LinkPredJava.jar EvalPST
```

The output of this program is as follows:

```
PRADA   PRPST   PRRAL   TPRADA  TPRPST  TPRRAL
0.0510  0.0510  0.0391  0.1250  0.1250  0.1250
```

## 2.3 Python

LinkPred bindings are optional (by default on). To change this setting, edit the file `CMakeLists.txt` in the root of the library source directory (search for the option `LINKPRED_WITH_BINDINGS`). Upon successful building, the library `_LinkPredPython` will be generated (named `_LinkPredPython.so` in Linux). This library will be loaded when running your program, and for that, it must be accessible to Python.

> **R** In Linux, you can make the library accessible to Python by including its path in the environment variable `PYTHONPATH`. If LinkPred is installed in the default location, this can be accomplished using the following command:
>
> ```
> $ export PYTHONPATH=$PYTHONPATH:/usr/local/lib
> ```

The Python module `LinkPredPython` containing the proxy classes needed to interface with `LinkPred` is located in `/bindings/Python`. Python programs that use `LinkPred` must import this module, which must therefore be in the Python module search path (for instance, in the same directory as your code).

### 2.3.1 Tutorial: Computing all scores using the class `Predictor`

This tutorial shows how to use the Java bindings of the simplified interface, more precisely the class `Predictor` to compute the scores of all non-existing edges in a network. For more information on the simplified interface, consult Chapter 2 of the user guide.

1. In a file name `predAll.py` write the following code:

```
# Import the module
import LinkPredPython as lpp
# Create a predictor object
p = lpp.Predictor();
# Load network from file
p.loadnet("Zakarays_Karate_Club.edges");
# Predict the score of all non-exisitng edges using Adamic
   Adar index
esv = p.predAllADA();
# Print the scores
for es in esv:
  print(es.i + "\t" + es.j + "\t" + "{:.4f}".format(es.score)
     );
```

R This code is available in: `tutorials/code/simp/python/predAll`

2. Run your code:

```
$ python predAll.py
```

The first few lines of this programs' output are as follows:

```
1       31      1.0765
1       10      0.4343
1       28      0.4343
1       29      0.9924
1       33      1.6137
1       17      1.4427
1       34      2.7110
1       15      0.0000
1       16      0.0000
1       19      0.0000
...
```

### 2.3.2 Tutorial: Computing scores of specific edges using the class `Predictor`

This tutorial shows how to use the Java bindings of the simplified interface, more precisely the class `Predictor` to compute the scores of specific edges. For more information on the simplified interface, consult Chapter 2 of the user guide.

1. In a file name `pred.py` write the following code:

```
# Import the module
import LinkPredPython as lpp
# Create a prtedictor object
p = lpp.Predictor();
# Load network from file
p.loadnet("Zakarays_Karate_Club.edges");
# Compute the score for the two edges (1, 34) and (26,34)
esv = lpp.EdgeScoreVec();
es = lpp.EdgeScore();
es.i = "1";
es.j = "34";
esv.push_back(es);
es.i = "26";
es.j = "34";
esv.push_back(es);
p.predKAB(esv);
# Print the scores
for es in esv:
  print(es.i + "\t" + es.j + "\t" + "{:.4f}".format(es.score)
    );
```

R This code is available in: `tutorials/code/simp/python/pred`

2. Run your code:

```
$ python pred.py
```

The first few lines of this programs' output are as follows:

```
1       34      0.5420
26      34      0.4688
```

### 2.3.3 Tutorial: Computing top scores using the class `Predictor`

This tutorial shows how to use the simplified interface, more precisely `Predictor` to compute the top edge scores. For more information on the simplified interface, consult Chapter 2 of the user guide.

1. In a file name `predTop.py` write the following code:

```
# Import the module
import LinkPredPython as lpp
k = 10;
# Create a predictor object
p = lpp.Predictor();
# Load network from file
p.loadnet("Zakarays_Karate_Club.edges");
# Predict the top k edges using Adamic Adar index
esv = p.predTopADA(k);
# Print the scores
for es in esv:
  print(es.i + "\t" + es.j + "\t" + "{:.4f}".format(es.score)
     );
```

R This code is available in: `tutorials/code/simp/python/predTop`

2. Run your code:

```
$ python predTop.py
```

The first few lines of this programs' output are as follows:

```
1       33      1.6137
1       34      2.7110
2       34      2.2529
3       32      1.6733
3       34      4.7194
5       6       1.9923
7       11      1.9923
8       14      1.8082
32      24      1.6656
24      25      1.6316
```

### 2.3.4 Tutorial: Encoder-classifier prediction using the class `Predictor`

This tutorial shows how to predict links using an encoder-classifier using `Predictor`. For more information on the simplified interface, consult Chapter 2 of the user guide. For information on link prediction using graph-embedding methods consult Section 5.2.3.

1. In a file name `ecl.py` write the following code:

```
# Import the module
import LinkPredPython as lpp
k = 10;
# Create a predictor object
p = lpp.Predictor();
# Load network from file
p.loadnet("Zakarays_Karate_Club.edges");
# Predict top k scores using an encoder-classifier predictor
    with Node2Vec as encoder and logistic regression as
    classifier
esv = p.predTopECL(k, "N2V", "LGR");
# Print the scores
print("N2V-LGR");
for es in esv:
  print(es.i + "\t" + es.j + "\t" + "{:.4f}".format(es.score)
     );
# Predict top k scores using an encoder-classifier predictor
    with LINE as encoder and feed-forward neural network as
    classifier
esv = p.predTopECL(k, "LIN", "FFN"); # FFN requires mlpack
# Print the scores
print("LIN-FFN");
for es in esv:
  print(es.i + "\t" + es.j + "\t" + "{:.4f}".format(es.score)
     );
```

R This code is available in: `tutorials/code/simp/python/ecl`

R For the names of available encoders and classifiers, consult the library reference manual.

2. Run your code:

```
$ python ecl.py
```

The output of this program is as follows:

```
N2V-LGR
1       34      0.6385
9       11      0.6373
9       13      0.5439
9       14      0.5560
9       18      0.5341
32      31      0.6160
32      10      0.6668
32      28      0.5062
32      17      0.6412
33      17      0.6679
```

```
LIN-FFN
3       7       0.4164
3       13      0.4029
3       34      0.4005
11      13      0.4307
11      34      0.4295
11      24      0.3806
10      19      0.3942
10      24      0.4374
10      30      0.3940
15      24      0.3964
```

### 2.3.5 Tutorial: Encoder-similarity prediction using the class `Predictor`

This tutorial shows how to predict links using an encoder-similarity using `Predictor`. For more information on the simplified interface, consult Chapter 2 of the user guide. For information on link prediction using graph-embedding methods consult Section 5.2.3.

1. In a file name `esm.py` write the following code:

```
# Import the module
import LinkPredPython as lpp
k = 10;
# Create a predictor object
p = lpp.Predictor();
# Load network from file
p.loadnet("Zakarays_Karate_Club.edges");
# Predict top k scores using an encoder-classifier predictor
   with Node2Vec as encoder and L2 similarity
esv = p.predTopESM(k, "N2V", "L2");
# Print the scores
print("N2V-L2");
for es in esv:
  print(es.i + "\t" + es.j + "\t" + "{:.4f}".format(es.score)
     );
# Predict top k scores using an encoder-similarity measure
   predictor with LINE as encoder and cosine similarity
esv = p.predTopESM(k, "LIN", "CSM");
# Print the scores
print("LIN-CSM");
for es in esv:
  print(es.i + "\t" + es.j + "\t" + "{:.4f}".format(es.score)
     );
```

R This code is available in: `tutorials/code/simp/python/esm`

R For the names of available encoders and similarity measures, consult the library reference manual.

2. Run your code:

```
$ python esm.py
```

The output of this program is as follows:

```
N2V-L2
13      14      -0.5522
13      18      -0.5377
18      22      -0.4145
15      16      -0.3949
15      21      -0.4318
15      23      -0.5334
16      21      -0.3882
16      23      -0.3743
19      21      -0.4718
21      23      -0.3852
LIN-CSM
1       21      0.6204
```

```
2       5       0.6932
9       13      0.7026
12      19      0.6214
14      30      0.6889
20      21      0.7088
22      21      0.6091
31      30      0.9057
10      15      0.6176
16      25      0.6741
```

### 2.3.6 Tutorial: Performance evaluation with automatically generated test data using the class `Evaluator`

This tutorial shows how to evaluate the performance of link prediction algorithm with test data automatically generated from a ground-truth network using `Evaluator`. For more information on the simplified interface, consult Chapter 2 of the user guide.

1. In a file name `eval-auto.py` write the following code:

```
# Import the module
import LinkPredPython as lpp
nbRuns = 10;
edgeRemRatio = 0.1;
# Create an evuator object
ev = lpp.Evaluator();
# Add predictors to be evuated
ev.addCNE();
ev.addADA();
ev.addKAB();
# Add performance measures
ev.addROC();
ev.addTPR();
# Run experiment on the specified network
ev.run("Zakarays_Karate_Club.edges", nbRuns, edgeRemRatio);
```

R This code is available in: `tutorials/code/simp/python/eval-auto`

R For the names of available predictors and performance measures, consult the library reference manual.

2. Run your code:

```
$ python eval-auto.py
```

The output of this program is as follows:

```
# n: 34 m: 78
#ratio  ROCADA  ROCCNE  ROCKAB  TPRADA  TPRCNE  TPRKAB
0.10    0.7737  0.7149  0.8280  0.1250  0.1932  0.1250
0.10    0.6593  0.6333  0.7030  0.1250  0.0000  0.1250
0.10    0.5967  0.5762  0.6095  0.1875  0.1818  0.2500
0.10    0.8464  0.7913  0.9343  0.1875  0.1290  0.3750
0.10    0.8324  0.7785  0.8967  0.1250  0.1750  0.1250
0.10    0.7240  0.6953  0.7547  0.0000  0.2222  0.0000
0.10    0.6753  0.6610  0.7262  0.0000  0.1591  0.1250
0.10    0.6048  0.5792  0.6672  0.0000  0.0000  0.0000
0.10    0.7627  0.7547  0.7808  0.2917  0.3194  0.3750
0.10    0.6442  0.5835  0.6727  0.1250  0.1250  0.1250
#Time: 88.3233 ms
```

3. The output above is a trace generated from within the evaluation method. You may also access the results as follows. n a file name `eval-auto-print.py` write the following code:

```
# Import the module
import LinkPredPython as lpp
nbRuns = 10;
```

```
edgeRemRatio = 0.1;
# Create an evuator object
ev = lpp.Evaluator();
# Add predictors to be evuated
ev.addCNE();
ev.addADA();
ev.addKAB();
# Add performance measures
ev.addROC();
ev.addTPR();
# Run experiment on the specified network
ev.run("Zakarays_Karate_Club.edges", nbRuns, edgeRemRatio);
import sys # For printing
# Print the header row
res = ev.getPerfRes(0);
for r in res:
  sys.stdout.write(r.name + "\t");
sys.stdout.write("\n");
# Print the results of each iteration
for i in range(nbRuns):
  res = ev.getPerfRes(i);
  for r in res:
    sys.stdout.write("{:.4f}".format(r.res) + "\t");
  sys.stdout.write("\n");
```

R This code is available in: `tutorials/code/simp/python/eval-auto`

4. Run your code:

```
$ python eval-auto-print.py
```

The output of this program is as follows:

```
# n: 34 m: 78
#ratio  ROCADA  ROCCNE  ROCKAB  TPRADA  TPRCNE  TPRKAB
0.10    0.7737  0.7149  0.8280  0.1250  0.1932  0.1250
0.10    0.6593  0.6333  0.7030  0.1250  0.0000  0.1250
0.10    0.5967  0.5762  0.6095  0.1875  0.1818  0.2500
0.10    0.8464  0.7913  0.9343  0.1875  0.1290  0.3750
0.10    0.8324  0.7785  0.8967  0.1250  0.1750  0.1250
0.10    0.7240  0.6953  0.7547  0.0000  0.2222  0.0000
0.10    0.6753  0.6610  0.7262  0.0000  0.1591  0.1250
0.10    0.6048  0.5792  0.6672  0.0000  0.0000  0.0000
0.10    0.7627  0.7547  0.7808  0.2917  0.3194  0.3750
0.10    0.6442  0.5835  0.6727  0.1250  0.1250  0.1250
#Time: 91.3887 ms
ROCADA  ROCCNE  ROCKAB  TPRADA  TPRCNE  TPRKAB
0.7737  0.7149  0.8280  0.1250  0.1932  0.1250
0.6593  0.6333  0.7030  0.1250  0.0000  0.1250
0.5967  0.5762  0.6095  0.1875  0.1818  0.2500
0.8464  0.7913  0.9343  0.1875  0.1290  0.3750
0.8324  0.7785  0.8967  0.1250  0.1750  0.1250
0.7240  0.6953  0.7547  0.0000  0.2222  0.0000
0.6753  0.6610  0.7262  0.0000  0.1591  0.1250
0.6048  0.5792  0.6672  0.0000  0.0000  0.0000
0.7627  0.7547  0.7808  0.2917  0.3194  0.3750
0.6442  0.5835  0.6727  0.1250  0.1250  0.1250
```

### 2.3.7 Tutorial: Performance evaluation with pre-generated test data using the class `Evaluator`

This tutorial shows how to evaluate the performance of link prediction algorithm with pre-generated test data using `Evaluator`. For more information on the simplified interface, consult Chapter 2 of the user guide.

1. In a file name `eval-pregenerated.py` write the following code:

```
# Import the module
import LinkPredPython as lpp
# Create an evuator object
ev = lpp.Evaluator();
# Add predictors to be evuated
ev.addADA();
ev.addRAL();
# Add performance measures
ev.addPR();
ev.addTPR();
# Run experiment on the specified network
ev.run("Zakarays_Karate_Club_Train.edges", "
   Zakarays_Karate_Club_Test.edges");
```

   The file `Zakarays_Karate_Club_Train.edges` contains the set of observed edges, whereas `Zakarays_Karate_Club_Test.edges` contains the set of edges that have been removed, i.e. the test set.

   R This code is available in: `tutorials/code/simp/python/eval-pregenerated`

2. Run your code:

```
$ python eval-pregenerated.py
```

   The output of this program is as follows:

```
PRADA   PRRAL   TPRADA  TPRRAL
0.1561  0.1568  0.1250  0.1250
```

3. The output above is a trace generated from within the evaluation method. You may also access the results as follows. In a file name `eval-pregenerated-print.py` write the following code:

```
# Import the module
import LinkPredPython as lpp
# Create an evuator object
ev = lpp.Evaluator();
# Add predictors to be evuated
ev.addADA();
ev.addRAL();
# Add performance measures
ev.addPR();
ev.addTPR();
# Run experiment on the specified network
ev.run("Zakarays_Karate_Club_Train.edges", "
   Zakarays_Karate_Club_Test.edges");
import sys # For printing
res = ev.getPerfRes(0);
for r in res:
```

```
  sys.stdout.write(r.name + "\t");
sys.stdout.write("\n");
for r in res:
  sys.stdout.write("{:.4f}".format(r.res) + "\t");
sys.stdout.write("\n");
```

This code is available in: `tutorials/code/simp/python/eval-pregenerated`

4. Run your code:

```
$ python eval-pregenerated-print.py
```

The output of this program is as follows:

```
PRADA   PRRAL   TPRADA  TPRRAL
0.1561  0.1568  0.1250  0.1250
PRADA   PRRAL   TPRADA  TPRRAL
0.1561  0.1568  0.1250  0.1250
```

### 2.3.8 Tutorial: Performance evaluation of external prediction results using the class `Evaluator`

This tutorial shows how to evaluate external link prediction results obtained by a user link prediction algorithm. For more information on the simplified interface, consult Chapter 2 of the user guide.

> R It is possible to implement new link prediction algorithms and integrate them into LinkPred, which allows for better use of the library's performance evaluation routines. See Section 5.4 of the user guide for more details.

1. The first step consists in generating test data. If you already have a ready test data, split into training and test sets, you can skip this part (go directly to Step 4 of this tutorial).
2. In a file name `gen-data.py` write the following code:

```
# Import the module
import LinkPredPython as lpp
# We remove 10% of the edges
edgeRemRatio = 0.1;
# We will not keep the network connected when removing edges
keepConnected = False;
# Seed of the random number generator
seed = 0;
# Create an Evaluator object
ev = lpp.Evaluator();
# The ground truth network "Zakarays_Karate_Club.edges" is
    split into an observed network stored in "Zakarays_Train.
    edges" and a list of removed edges stored in "
    Zakarays_Test.edges"
ev.genTestData("Zakarays_Karate_Club.edges", "Zakarays_Train.
    edges", "Zakarays_Test.edges", edgeRemRatio, keepConnected
    , seed);
```

   The file `Zakarays_Train.edges` will contain the set of observed edges, whereas `Zakarays_Test.edges` will contain the set of edges that have been removed, i.e. the test set.
3. Run your code:

```
$ python gen-data.py
```

> R The method `addPST` is used to load the pre-stored results by internally creating a `PST` predictor. For more details about this predictor consult Section 5.2.4 of the user guide.

The first few lines of `Zakarays_Train.edges` and the file `Zakarays_Test.edges` generated by this program are as follows:

```
# First few lines of the file Zakarays_Train.edges
1       2
1       3
1       4
1       5
1       6
```

```
1       7
1       8
1       9
1       11
...
# The file Zakarays_Test.edges
2       22
3       4
24      26
33      24
9       34
32      33
33      30
28      25
```

4. At this stage, we have two files: `Zakarays_Train.edges` which contains the set of observed edges and `Zakarays_Test.edges` which contains the set of removed edges. Train your algorithm on the file `Zakarays_Train.edges` and computed the scores of all non-existing links in that network. This includes the edges that have been removed (contained in `Zakarays_Test.edges`) and those that are absent from the ground truth network (true negative links). Store the scores in a file name "pst.txt" in the following format (the first line is a comment and can be safely omitted):

```
#Start  End     Score
1       31      0.41374
1       10      0.276687
1       28      0.283587
1       29      0.374494
1       33      0.463135
1       17      0.531863
1       34      0.49409
1       26      0.325087
1       25      0.325087
...
```

5. In a file name `eval-pst.py` write the following code:

```python
# Import the module
import LinkPredPython as lpp
# Create an evuator object
ev = lpp.Evaluator();
# Add predictors to be evuated
ev.addADA();
# Use the method addPST to create a predictor that loads
   scores from pst.txt
ev.addPST("PST", "pst.txt");
ev.addRAL();
# Add performance measures
ev.addPR();
ev.addTPR();
# Run experiment on the specified network
ev.run("Zakarays_Train.edges", "Zakarays_Test.edges");
```

6. Run your code:

```
$ python eval-pst.py
```

The output of this program is as follows:

```
PRADA   PRPST   PRRAL   TPRADA  TPRPST  TPRRAL
0.0510  0.0510  0.0391  0.1250  0.1250  0.1250
```

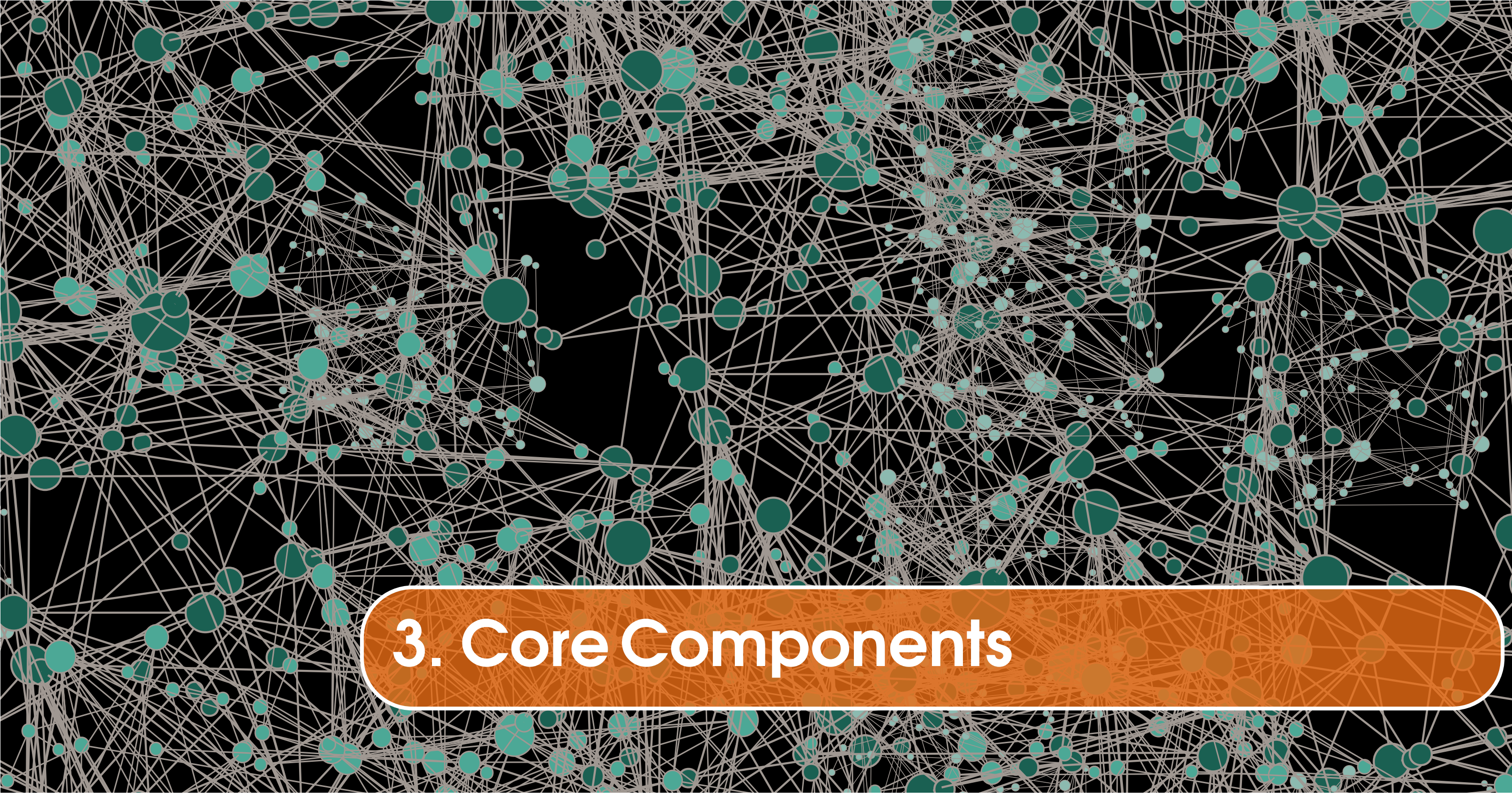

# 3. Core Components

This chapter contains tutorials on core components of LinkPred, namely, network data structures and maps.

## 3.1 Representing undirected networks using the class `UNetwork`

The class `UNetwork` is used to efficiently represent undirected networks. This section contains a number of tutorials that demonstrate how to build and access network information using this class.

### 3.1.1 Tutorial: Reading a network from file and printing it

This tutorial shows how to read a network from file and print it. For more information on the core components of LinkPred, consult Chapter 3 of the user guide.

1. In a file name `read-print.cpp` write the following code:

```
#include <linkpred.hpp>
using namespace LinkPred;
int main() {
  // Read network frome file
  auto net = UNetwork<>::read("Zakarays_Karate_Club.edges");
  // Print to standard output
  net->print();
  return 0;
}
```

   R This code is available in: `tutorials/code/core/unetwork/read-print`

   R The method `read` returns a shared pointer to an object of type `UNetwork`.

2. Compile your code:

```
$ mpiCC read-print.cpp -o read-print -fopenmp -lLinkPred
```

   R Check Tutorial 1.2 if you face any compilation issues.

3. Run your code:

```
$ ./read-print
```

   The following is a partial output of this program:

```
1       2
1       3
1       4
1       5
1       6
1       7
1       8
1       9
1       11
1       12
...
```

### 3.1.2 Tutorial: Building a network

This tutorial shows how to build a network by adding nodes and connecting them by edges. For more information on the core components of LinkPred, consult Chapter 3 of the user guide.

1. In a file name `net-build.cpp` write the following code:

```
#include <linkpred.hpp>
#include <iostream>
using namespace LinkPred;
int main() {
  // Create a network object
  UNetwork<> net;
  // Add nodes
  net.addNode("A");
  net.addNode("B");
  net.addNode("C");
  net.addNode("D");
  // Add edges
  net.addEdge(net.getID("A"), net.getID("B"));
  net.addEdge(net.getID("B"), net.getID("C"));
  net.addEdge(net.getID("C"), net.getID("D"));
  net.addEdge(net.getID("D"), net.getID("A"));
  // Assemble the network
  net.assemble();
  // Print the network
  net.print();
  return 0;
}
```

R This code is available in: `tutorials/code/core/unetwork/net-build`

R The method `addEdge` uses node IDs (**and not labels**) to identify nodes. This is why the method `getID` is used.

R It is important to assemble the network before using it.

2. Compile your code:

```
$ mpiCC net-build.cpp -o net-build -fopenmp -lLinkPred
```

R Check Tutorial 1.2 if you face any compilation issues.

3. Run your code:

```
$ ./net-build
```

The following is the output of this program:

```
A       B
A       D
B       C
C       D
```

### 3.1.3 Tutorial: Accessing network information

This tutorial shows how to access network information including:

- Listing all nodes in the network.
- Translating node labels to IDs.
- Translating node IDs to labels.
- List all edges in the network.
- List the neighbors of every node.

For more information on the core components of LinkPred, consult Chapter 3 of the user guide.

1. In a file name `net-access-nodes.cpp` write the following code:

```
#include <linkpred.hpp>
#include <iostream>
using namespace LinkPred;
int main() {
  // Create a network object
  UNetwork<> net;
  // Add nodes
  net.addNode("A");
  net.addNode("B");
  net.addNode("C");
  net.addNode("D");
  // Add edges
  net.addEdge(net.getID("A"), net.getID("B"));
  net.addEdge(net.getID("B"), net.getID("C"));
  net.addEdge(net.getID("C"), net.getID("D"));
  net.addEdge(net.getID("D"), net.getID("A"));
  // Assemble the network
  net.assemble();
  // Accessing nodes
  std::cout << "Nodes:\n";
  std::cout << "ID\tLabel\n";
  for (auto it = net.nodesBegin(); it != net.nodesEnd(); ++it
     ) {
    std::cout << it->first << "\t" << it->second << std::endl
       ;
  }
  std::cout << "Translating␣labels␣to␣IDs\n";
  std::cout << "A␣->␣" << net.getID("A") << std::endl;
  std::cout << "B␣->␣" << net.getID("B") << std::endl;
  std::cout << "C␣->␣" << net.getID("C") << std::endl;
  std::cout << "D␣->␣" << net.getID("D") << std::endl;
  std::cout << "Translating␣IDs␣to␣labels\n";
  for (std::size_t i = 0; i < net.getNbNodes(); i++) {
    std::cout << i << "␣->␣" << net.getLabel(i) << std::endl;
  }
  return 0;
}
```

R This code is available in: `tutorials/code/core/unetwork/net-access`

2. Compile your code:

```
$ mpiCC net-access-nodes.cpp -o net-access-nodes -fopenmp -
   lLinkPred
```

**R** Check Tutorial 1.2 if you face any compilation issues.

3. Run your code:

```
$ ./net-access-nodes
```

The following is the output of this program:

```
Nodes:
ID      Label
0       A
1       B
2       C
3       D
Translating labels to IDs
A -> 0
B -> 1
C -> 2
D -> 3
Translating IDs to labels
0 -> A
1 -> B
2 -> C
3 -> D
```

4. In a file name `net-access-nodes.cpp` write the following code:

```
#include <linkpred.hpp>
#include <iostream>
using namespace LinkPred;
int main() {
  // Create a network object
  UNetwork<> net;
  // Add nodes
  net.addNode("A");
  net.addNode("B");
  net.addNode("C");
  net.addNode("D");
  // Add edges
  net.addEdge(net.getID("A"), net.getID("B"));
  net.addEdge(net.getID("B"), net.getID("C"));
  net.addEdge(net.getID("C"), net.getID("D"));
  net.addEdge(net.getID("D"), net.getID("A"));
  // Assemble the network
  net.assemble();
  // Accessing nodes
  std::cout << "Nodes:\n";
  std::cout << "ID\tLabel\n";
  for (auto it = net.nodesBegin(); it != net.nodesEnd(); ++it
     ) {
    std::cout << it->first << "\t" << it->second << std::endl
       ;
  }
  std::cout << "Translating␣labels␣to␣IDs\n";
```

```
    std::cout << "A␣->␣" << net.getID("A") << std::endl;
    std::cout << "B␣->␣" << net.getID("B") << std::endl;
    std::cout << "C␣->␣" << net.getID("C") << std::endl;
    std::cout << "D␣->␣" << net.getID("D") << std::endl;
    std::cout << "Translating␣IDs␣to␣labels\n";
    for (std::size_t i = 0; i < net.getNbNodes(); i++) {
      std::cout << i << "␣->␣" << net.getLabel(i) << std::endl;
    }
    return 0;
}
```

R This code is available in: `tutorials/code/core/unetwork/net-access`

5. Compile your code:

```
$ mpiCC net-access-nodes.cpp -o net-access-nodes -fopenmp -
   lLinkPred
```

R Check Tutorial 1.2 if you face any compilation issues.

6. Run your code:

```
$ ./net-access-nodes
```

The following is the output of this program:

```
Nodes:
ID      Label
0       A
1       B
2       C
3       D
Translating labels to IDs
A -> 0
B -> 1
C -> 2
D -> 3
Translating IDs to labels
0 -> A
1 -> B
2 -> C
3 -> D
```

7. In a file name `net-access-edges.cpp` write the following code:

```
#include <linkpred.hpp>
#include <iostream>
using namespace LinkPred;
int main() {
  // Create a network object
  UNetwork<> net;
  // Add nodes
  net.addNode("A");
  net.addNode("B");
  net.addNode("C");
  net.addNode("D");
  // Add edges
  net.addEdge(net.getID("A"), net.getID("B"));
  net.addEdge(net.getID("B"), net.getID("C"));
  net.addEdge(net.getID("C"), net.getID("D"));
  net.addEdge(net.getID("D"), net.getID("A"));
  // Assemble the network
  net.assemble();
  // Accessing edges
  std::cout << "Edges:\n";
  std::cout << "Start\tEnd\n";
  for (auto it = net.edgesBegin(); it != net.edgesEnd(); ++it
     ) {
    std::cout << net.start(*it) << "\t" << net.end(*it) <<
       std::endl;
  }
  // Neighbors
  for (std::size_t i = 0; i < net.getNbNodes(); i++) {
    std::cout << "Neighbors␣of␣" << i << std::endl;
    for (auto it = net.neighbBegin(i); it != net.neighbEnd(i)
       ; ++it) {
      std::cout << net.end(*it) << std::endl;
    }
  }
  return 0;
}
```

This code is available in: `tutorials/code/core/unetwork/net-access`

8. Compile your code:

```
$ mpiCC net-access-edges.cpp -o net-access-edges -fopenmp -
   lLinkPred
```

9. Run your code:

```
$ ./net-access-edges
```

The following is the output of this program:

```
Edges:
Start   End
0       1
0       3
1       2
2       3
Neighbors of 0
```

```
1
3
Neighbors of 1
0
2
Neighbors of 2
1
3
Neighbors of 3
0
2
```

## 3.2 Representing directed networks using the class `DNetwork`

The class `DNetwork` is used to efficiently represent directed networks. This section contains a number of tutorials that demonstrate how to build and access network information using this class.

### 3.2.1 Tutorial: Reading a network from file and printing it

This tutorial shows how to read a network from file and print it. For more information on the core components of LinkPred, consult Chapter 3 of the user guide.

1. In a file name `read-print.cpp` write the following code:

```
#include <linkpred.hpp>
using namespace LinkPred;
int main() {
  // Read network frome file
  auto net = DNetwork<>::read("Zakarays_Karate_Club.edges");
  // Print to standard output
  net->print();
  return 0;
}
```

   R This code is available in: `tutorials/code/core/dnetwork/read-print`

   R The method `read` returns a shared pointer to an object of type `DNetwork`.

2. Compile your code:

```
$ mpiCC read-print.cpp -o read-print -fopenmp -lLinkPred
```

   R Check Tutorial 1.2 if you face any compilation issues.

3. Run your code:

```
$ ./read-print
```

The following is a partial output of this program:

```
1       2
1       3
1       4
1       5
1       6
1       7
1       8
1       9
1       11
1       12
...
```

### 3.2.2 Tutorial: Building a network

This tutorial shows how to build a network by adding nodes and connecting them by edges. For more information on the core components of LinkPred, consult Chapter 3 of the user guide.

1. In a file name `net-build.cpp` write the following code:

```
#include <linkpred.hpp>
#include <iostream>
using namespace LinkPred;
int main() {
  // Create a network object
  DNetwork<> net;
  // Add nodes
  net.addNode("A");
  net.addNode("B");
  net.addNode("C");
  net.addNode("D");
  // Add edges
  net.addEdge(net.getID("A"), net.getID("B"));
  net.addEdge(net.getID("B"), net.getID("C"));
  net.addEdge(net.getID("C"), net.getID("D"));
  net.addEdge(net.getID("D"), net.getID("A"));
  // Assemble the network
  net.assemble();
  // Print the network
  net.print();
  return 0;
}
```

   R This code is available in: `tutorials/code/core/dnetwork/net-build`

   R The method `addEdge` uses node IDs (**and not labels**) to identify nodes. This is why the method `getID` is used.

   R It is important to assemble the network before using it.

2. Compile your code:

```
$ mpiCC net-build.cpp -o net-build -fopenmp -lLinkPred
```

   R Check Tutorial 1.2 if you face any compilation issues.

3. Run your code:

```
$ ./net-build
```

   The following is the output of this program:

```
A       B
B       C
C       D
D       A
```

### 3.2.3 Tutorial: Accessing network information

This tutorial shows how to access network information including:

- Listing all nodes in the network.
- Translating node labels to IDs.
- Translating node IDs to labels.
- List all edges in the network.
- List the neighbors of every node.

For more information on the core components of LinkPred, consult Chapter 3 of the user guide.

1. In a file name `net-access-nodes.cpp` write the following code:

```
#include <linkpred.hpp>
#include <iostream>
using namespace LinkPred;
int main() {
  // Create a network object
  DNetwork<> net;
  // Add nodes
  net.addNode("A");
  net.addNode("B");
  net.addNode("C");
  net.addNode("D");
  // Add edges
  net.addEdge(net.getID("A"), net.getID("B"));
  net.addEdge(net.getID("B"), net.getID("C"));
  net.addEdge(net.getID("C"), net.getID("D"));
  net.addEdge(net.getID("D"), net.getID("A"));
  // Assemble the network
  net.assemble();
  // Accessing nodes
  std::cout << "Nodes:\n";
  std::cout << "ID\tLabel\n";
  for (auto it = net.nodesBegin(); it != net.nodesEnd(); ++it
     ) {
    std::cout << it->first << "\t" << it->second << std::endl
       ;
  }
  std::cout << "Translating␣labels␣to␣IDs\n";
  std::cout << "A␣->␣" << net.getID("A") << std::endl;
  std::cout << "B␣->␣" << net.getID("B") << std::endl;
  std::cout << "C␣->␣" << net.getID("C") << std::endl;
  std::cout << "D␣->␣" << net.getID("D") << std::endl;
  std::cout << "Translating␣IDs␣to␣labels\n";
  for (std::size_t i = 0; i < net.getNbNodes(); i++) {
    std::cout << i << "␣->␣" << net.getLabel(i) << std::endl;
  }
  return 0;
}
```

R This code is available in: `tutorials/code/core/dnetwork/net-access`

2. Compile your code:

```
$ mpiCC net-access-nodes.cpp -o net-access-nodes -fopenmp -
   lLinkPred
```

Ⓡ Check Tutorial 1.2 if you face any compilation issues.

3. Run your code:

```
$ ./net-access-nodes
```

The following is the output of this program:

```
Nodes:
ID      Label
0       A
1       B
2       C
3       D
Translating labels to IDs
A -> 0
B -> 1
C -> 2
D -> 3
Translating IDs to labels
0 -> A
1 -> B
2 -> C
3 -> D
```

4. In a file name `net-access-nodes.cpp` write the following code:

```
#include <linkpred.hpp>
#include <iostream>
using namespace LinkPred;
int main() {
  // Create a network object
  DNetwork<> net;
  // Add nodes
  net.addNode("A");
  net.addNode("B");
  net.addNode("C");
  net.addNode("D");
  // Add edges
  net.addEdge(net.getID("A"), net.getID("B"));
  net.addEdge(net.getID("B"), net.getID("C"));
  net.addEdge(net.getID("C"), net.getID("D"));
  net.addEdge(net.getID("D"), net.getID("A"));
  // Assemble the network
  net.assemble();
  // Accessing nodes
  std::cout << "Nodes:\n";
  std::cout << "ID\tLabel\n";
  for (auto it = net.nodesBegin(); it != net.nodesEnd(); ++it
     ) {
    std::cout << it->first << "\t" << it->second << std::endl
       ;
  }
  std::cout << "Translating␣labels␣to␣IDs\n";
```

```
  std::cout << "A␣->␣" << net.getID("A") << std::endl;
  std::cout << "B␣->␣" << net.getID("B") << std::endl;
  std::cout << "C␣->␣" << net.getID("C") << std::endl;
  std::cout << "D␣->␣" << net.getID("D") << std::endl;
  std::cout << "Translating␣IDs␣to␣labels\n";
  for (std::size_t i = 0; i < net.getNbNodes(); i++) {
    std::cout << i << "␣->␣" << net.getLabel(i) << std::endl;
  }
  return 0;
}
```

R This code is available in: `tutorials/code/core/dnetwork/net-access`

5. Compile your code:

```
$ mpiCC net-access-nodes.cpp -o net-access-nodes -fopenmp -
   lLinkPred
```

R Check Tutorial 1.2 if you face any compilation issues.

6. Run your code:

```
$ ./net-access-nodes
```

The following is the output of this program:

```
Nodes:
ID      Label
0       A
1       B
2       C
3       D
Translating labels to IDs
A -> 0
B -> 1
C -> 2
D -> 3
Translating IDs to labels
0 -> A
1 -> B
2 -> C
3 -> D
```

7. In a file name `net-access-edges.cpp` write the following code:

```
#include <linkpred.hpp>
#include <iostream>
using namespace LinkPred;
int main() {
  // Create a network object
  DNetwork<> net;
  // Add nodes
  net.addNode("A");
  net.addNode("B");
  net.addNode("C");
  net.addNode("D");
  // Add edges
  net.addEdge(net.getID("A"), net.getID("B"));
  net.addEdge(net.getID("B"), net.getID("C"));
  net.addEdge(net.getID("C"), net.getID("D"));
  net.addEdge(net.getID("D"), net.getID("A"));
  // Assemble the network
  net.assemble();
  // Accessing edges
  std::cout << "Edges:\n";
  std::cout << "Start\tEnd\n";
  for (auto it = net.edgesBegin(); it != net.edgesEnd(); ++it
     ) {
    std::cout << net.start(*it) << "\t" << net.end(*it) <<
        std::endl;
  }
  // Neighbors
  for (std::size_t i = 0; i < net.getNbNodes(); i++) {
    std::cout << "Neighbors␣of␣" << i << std::endl;
    for (auto it = net.neighbBegin(i); it != net.neighbEnd(i)
        ; ++it) {
      std::cout << net.end(*it) << std::endl;
    }
  }
  return 0;
}
```

R This code is available in: `tutorials/code/core/dnetwork/net-access`

8. Compile your code:

```
$ mpiCC net-access-edges.cpp -o net-access-edges -fopenmp -
   lLinkPred
```

9. Run your code:

```
$ ./net-access-edges
```

The following is the output of this program:

```
Edges:
Start   End
0       1
1       2
2       3
3       0
Neighbors of 0
```

```
1
Neighbors of 1
2
Neighbors of 2
3
Neighbors of 3
0
```

## 3.3 Maps

Maps are a useful way to associate data to nodes or edges. Two types of maps are available in LinkPred: *node maps* (class `NodeMap`) and *edge maps* (class `EdgeMap`), both member of `UNetwork` and `DNetwork`. The first assigns data to the nodes of the network, whereas the latter maps data to edges. This section contains tutorials on how to use maps to associate data to nodes and edges.

### 3.3.1 Tutorial: Node maps

This tutorial shows how to associate data to nodes using node maps. For more information on the core components of LinkPred, consult Chapter 3 of the user guide.

1. In a file name `node-map.cpp` write the following code:

```
#include <linkpred.hpp>
#include <iostream>
using namespace LinkPred;
int main(int argc, char*argv[]) {
  // Create a network object
  UNetwork<> net;
  // Add nodes
  net.addNode("A");
  net.addNode("B");
  net.addNode("C");
  net.addNode("D");
  // Add edges
  net.addEdge(net.getID("A"), net.getID("B"));
  net.addEdge(net.getID("B"), net.getID("C"));
  net.addEdge(net.getID("C"), net.getID("D"));
  net.addEdge(net.getID("D"), net.getID("A"));
  // Assemble the network
  net.assemble();
  // Create a node map that associates a double to every node
  auto nodeMap = net.template createNodeMap<double>();
  // Fill the map
  for (std::size_t i = 0; i < net.getNbNodes(); i++) {
    nodeMap[i] = i / 2.0;
  }
  // Access the map
  std::cout << "Label\tValue" << std::endl;
  for (std::size_t i = 0; i < net.getNbNodes(); i++) {
    std::cout << net.getLabel(i) << "\t" << nodeMap[i] << std
        ::endl;
  }
  return 0;
}
```

   R This code is available in: `tutorials/code/core/maps/node-map`

2. Compile your code:

```
$ mpiCC node-map.cpp -o node-map -fopenmp -lLinkPred
```

   R Check Tutorial 1.2 if you face any compilation issues.

3. Run your code:

```
$ ./node-map
```

   The following is a partial output of this program:

```
Label   Value
A       0
B       0.5
C       1
D       1.5
```

### 3.3.2 Tutorial: Edge maps

This tutorial shows how to associate data to edges using edge maps. For more information on the core components of LinkPred, consult Chapter 3 of the user guide.

1. In a file name `edge-map.cpp` write the following code:

```
#include <linkpred.hpp>
#include <iostream>
using namespace LinkPred;
int main(int argc, char*argv[]) {
  // Create a network object
  UNetwork<> net;
  // Add nodes
  net.addNode("A");
  net.addNode("B");
  net.addNode("C");
  net.addNode("D");
  // Add edges
  net.addEdge(net.getID("A"), net.getID("B"));
  net.addEdge(net.getID("B"), net.getID("C"));
  net.addEdge(net.getID("C"), net.getID("D"));
  net.addEdge(net.getID("D"), net.getID("A"));
  // Assemble the network
  net.assemble();
  // Create a node map that associates an integer to every
     edge
  auto edgeMap = net.template createEdgeMap<int>();
  edgeMap[net.makeEdge(0, 1)] = 3;
  edgeMap[net.makeEdge(1, 2)] = 2;
  edgeMap[net.makeEdge(2, 3)] = 5;
  edgeMap[net.makeEdge(3, 0)] = 4;
  // Access the map
  std::cout << "Start\tEnd\tValue" << std::endl;
  for (auto it = net.edgesBegin(); it != net.edgesEnd(); ++it
     ) {
    auto i = net.start(*it);
    auto j = net.end(*it);
    std::cout << net.getLabel(i) << "\t" << net.getLabel(j)
        << "\t" << edgeMap.at(*it) << std::endl;
  }
  return 0;
}
```

R This code is available in: `tutorials/code/core/maps/edge-map`

2. Compile your code:

```
$ mpiCC edge-map.cpp -o edge-map -fopenmp -lLinkPred
```

R Check Tutorial 1.2 if you face any compilation issues.

3. Run your code:

```
$ ./edge-map
```

The following is a partial output of this program:

| Start | End | Value |
|---|---|---|
| A | B | 3 |
| A | D | 4 |
| B | C | 2 |
| C | D | 5 |

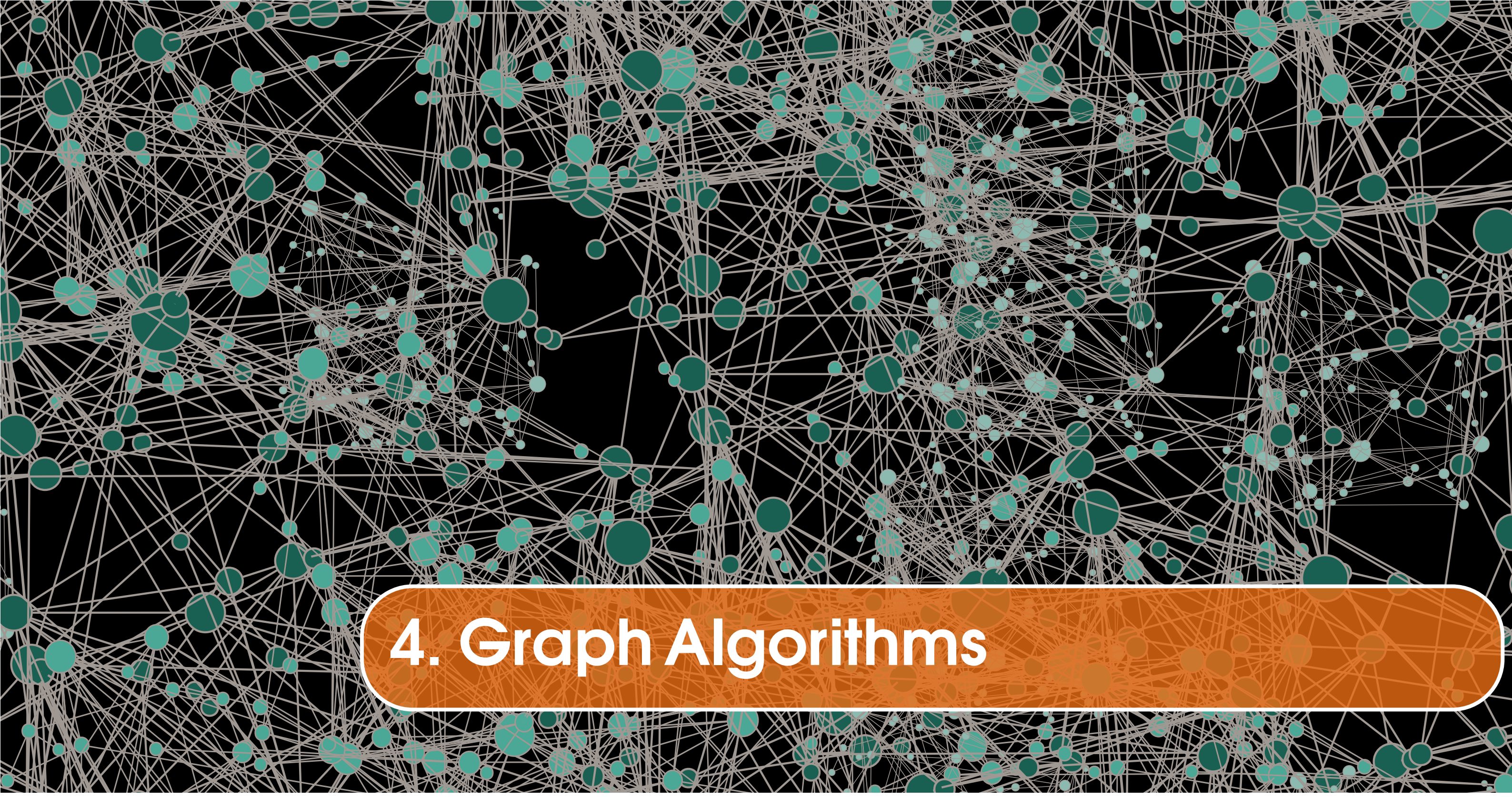

# 4. Graph Algorithms

This chapter contains tutorials on graph algorithms available in LinkPred, namely, traversal algorithms, shortest path algorithms, and graph embedding algorithms.

## 4.1 Traversing a network

LinkPred provides two classes for graph traversal: `BFS`, for Breadth First traversal, and `DFS` for Depth First traversal. They can be used to process nodes as they are being visited. The library offers two useful node processing classes: `Counter`, which simply counts the visited nodes, and `Collector`, which collects the visited nodes' IDs into a queue in the order of their visit.

### 4.1.1 Tutorial: Traverse a network in BFS

This tutorial shows how to traverse a network in BFS (Breadth-First Search). For more information on graph algorithms available in LinkPred, consult Chapter 4 of the user guide.

1. In a file name `bfs.cpp` write the following code:

```
#include <linkpred.hpp>
#include <iostream>
using namespace LinkPred;
int main() {
  // Read network from file
  auto net = UNetwork<>::read("net-traversal.edges");
  // Create a BFS objec
  BFS<> bfs(net);
  // We collect nodes during traversal
  Collector<> col;
  // We start traversal at node 1
  bfs.traverse(net->getID("1"), col);
  // Retrieve the set of visited nodes
  auto visited = col.getVisited();
  // Print visited nodes
  std::cout << "Visited␣nodes:" << std::endl;
  while (!visited.empty()) {
    auto i = visited.front();
    visited.pop();
    std::cout << net->getLabel(i) << std::endl;
  }
  return 0;
}
```

R This code is available in: `tutorials/code/graphalg/traversal/bfs`

R `BFS` and `DFS` are class templates with two template parameters: the network (with default value `UNetwork<>`) and the node processor (with default value `Collector`). In this example, `BFS` is instantiated with the default parameters.

2. Compile your code:

```
$ mpiCC bfs.cpp -o bfs -fopenmp -lLinkPred
```

R Check Tutorial 1.2 if you face any compilation issues.

3. Run your code:

```
$ ./bfs
```

The following is the output of this program:

```
Visited nodes:
1
2
3
4
```

```
8
5
6
7
```

### 4.1.2 Tutorial: Traverse a network in DFS

This tutorial shows how to traverse a network in DFS (Depth-First Search). For more information on graph algorithms available in LinkPred, consult Chapter 4 of the user guide.

1. In a file name `dfs.cpp` write the following code:

```
#include <linkpred.hpp>
#include <iostream>
using namespace LinkPred;
int main() {
  // Read network from file
  auto net = UNetwork<>::read("net-traversal.edges");
  // Create a DFS objec
  DFS<UNetwork<>, Counter<>> dfs(net);
  // We count nodes during traversal
  Counter<> counter;
  // We start traversal at node 1
  dfs.traverse(net->getID("1"), counter);
  // Print the number of visited nodes
  std::cout << "DFS␣visited␣" << counter.getCount() << "␣
     nodes" << std::endl;
  return 0;
}
```

   R This code is available in: `tutorials/code/graphalg/traversal/dfs`

   R `BFS` and `DFS` are class templates with two template parameters: the network (with default value `UNetwork<>`) and the node processor (with default value `Collector`). In this example, `DFS` is instantiated with `Counter` as the node processor instead of the default.

2. Compile your code:

```
$ mpiCC dfs.cpp -o dfs -fopenmp -lLinkPred
```

   R Check Tutorial 1.2 if you face any compilation issues.

3. Run your code:

```
$ ./dfs
```

The following is the output of this program:

```
DFS visited 8 nodes
```

## 4.2 Shortest paths

This section shows how to use LinkPred classes to solve the shortest path problems. The class `Dijkstra` allows to find shortest paths between two nodes and compute shot-path distances from one node to all other nodes. The class `ESPDistCalculator` (exact shortest path distance calculator), which inherits from the abstract class facilitates memory management when computing shortest paths, a task that is crucial when dealing with very large networks. This section contains tutorials explaining the basic use scenarios of these classes.More details are presented in Section 4.2 of the user guide.

### 4.2.1 Tutorial: Finding the shortest path between two nodes

This tutorial shows how to find the shortest path between two nodes using the class `Dijkstra`. For more information on shortest path algorithms available in LinkPred, consult Section 4.2 of the user guide.

1. In a file name `dijkstra-two-nodes.cpp` write the following code:

```
#include <linkpred.hpp>
#include <iostream>
using namespace LinkPred;
int main() {
  // Read network from file
  auto net = UNetwork<>::read("net-sp.edges");
  // Create an edge length (weight) map
  auto length = net->template createEdgeMapSP<double>();
  // Assign a length to every edge
  int i = 1;
  for (auto it = net->edgesBegin(); it != net->edgesEnd(); ++
      it, i++) {
    (*length)[*it] = (13 * i) % 3 + 1;
  }
  // Create a Dijkstra object
  Dijkstra<> dijkstra(net);
  // Register length map
  auto lengthMapId = dijkstra.registerLengthMap(length);
  // Find shortest path between node 1 and 6
  auto res = dijkstra.getShortestPath(net->getID("1"), net->
      getID("6"), lengthMapId);
  // Print path and distance
  auto path = res.first;
  auto dist = res.second;
  std::cout << "Path: ";
  for (auto it = path->begin(); it != path->end(); ++it) {
    std::cout << net->getLabel(*it) << " ";
  }
  std::cout << "\nDistance: " << dist << std::endl;
  return 0;
}
```

> R This code is available in: `tutorials/code/graphalg/sp/dijkstra-two-nodes`

2. Compile your code:

```
$ mpiCC dijkstra-two-nodes.cpp -o dijkstra-two-nodes -fopenmp
    -lLinkPred
```

> R Check Tutorial 1.2 if you face any compilation issues.

3. Run your code:

```
$ ./dijkstra-two-nodes
```

The following is the output of this program:

```
Path: 1 2 4 6
Distance: 5
```

### 4.2.2 Tutorial: Computing the distance from one node to all nodes

This tutorial shows how to find the distance from one node to all other nodes using the class `Dijkstra`. For more information on shortest path algorithms available in LinkPred, consult Section 4.2 of the user guide.

1. In a file name `dijkstra-one-to-all.cpp` write the following code:

```
#include <linkpred.hpp>
#include <iostream>
using namespace LinkPred;
int main() {
  // Read network from file
  auto net = UNetwork<>::read("net-sp.edges");
  // Create an edge length (weight) map
  auto length = net->template createEdgeMapSP<double>();
  // Assign a length to every edge
  int i = 1;
  for (auto it = net->edgesBegin(); it != net->edgesEnd(); ++
      it, i++) {
    (*length)[*it] = (13 * i) % 3 + 1;
  }
  // Create a Dijkstra object
  Dijkstra<> dijkstra(net);
  // Register length map
  auto lengthMapId = dijkstra.registerLengthMap(length);
  // Compute the distance from node 1 to all other nodes
  auto distMap = dijkstra.getDist(net->getID("1"),
      lengthMapId);
  // Print distances
  std::cout << "Target\tDist\tNumber␣of␣nodes␣in␣the␣path" <<
       std::endl;
  for (auto it = net->nodesBegin(); it != net->nodesEnd(); ++
      it) {
    auto res = distMap->at(it->first);
    std::cout << it->second << "\t" << res.first << "\t" <<
        res.second << std::endl;
  }
  return 0;
}
```

> **R** This code is available in: `tutorials/code/graphalg/sp/dijkstra-one-to-all`

2. Compile your code:

```
$ mpiCC dijkstra-one-to-all.cpp -o dijkstra-one-to-all -
    fopenmp -lLinkPred
```

> **R** Check Tutorial 1.2 if you face any compilation issues.

3. Run your code:

```
$ ./dijkstra-one-to-all
```

The following is the output of this program:

```
Target  Dist    Number of nodes in the path
1       0       0
2       2       1
3       3       1
4       4       2
5       4       2
6       5       3
7       inf     18446744073709551615
8       inf     18446744073709551615
```

### 4.2.3 Tutorial: Memory management when computing distances

This tutorial shows how to use the class `ESPDistCalculator` (exact shortest path distance calculator), to facilitates memory management when using the class `Dijkstra`. For more information on shortest path algorithms available in LinkPred, consult Section 4.2 of the user guide.

1. In a file name `netdistcalc.cpp` write the following code:

```
#include <linkpred.hpp>
#include <iostream>
using namespace LinkPred;
int main() {
  // Read network from file
  auto net = UNetwork<>::read("net-sp.edges");
  // Create an edge length (weight) map
  auto length = net->template createEdgeMapSP<double>();
  // Assign a length to every edge
  int i = 1;
  for (auto it = net->edgesBegin(); it != net->edgesEnd(); ++
     it, i++) {
    (*length)[*it] = (13 * i) % 3 + 1;
  }
  // Create a Dijkstra object
  Dijkstra<> dijkstra(net);
  // Create a distance calculator. Here, we pass the option
     NetworkCache, that is we cache all distances
  ESPDistCalculator<> calc(dijkstra, length, NetworkCache);
  // Print all distances
  std::cout << "i\tj\tDist" << std::endl;
  for (unsigned int i = 0; i < net->getNbNodes(); i++) {
    for (unsigned int j = 0; j < i; j++) {
      std::cout << net->getLabel(i) << "\t" << net->getLabel(
          j) << "\t" << calc.getDist(i, j).first << std::endl;
    }
  }
  return 0;
}
```

> **R** This code is available in: `tutorials/code/graphalg/sp/netdistcalc`

2. Compile your code:

```
$ mpiCC netdistcalc.cpp -o netdistcalc -fopenmp -lLinkPred
```

> **R** Check Tutorial 1.2 if you face any compilation issues.

3. Run your code:

```
$ ./netdistcalc
```

The following is the output of this program:

```
i       j       Dist
2       1       2
3       1       3
3       2       1
```

```
4       1       4
4       2       2
4       3       3
5       1       4
5       2       2
5       3       1
5       4       2
6       1       5
6       2       3
6       3       2
6       4       3
6       5       1
7       1       inf
7       2       inf
7       3       inf
7       4       inf
7       5       inf
7       6       inf
8       1       inf
8       2       inf
8       3       inf
8       4       inf
8       5       inf
8       6       inf
8       7       2
```

## 4.3 Network embedding

This section demonstrates the use of some of the graph embedding methods available in LinkPred. For more details, consult Section 4.3 of the user guide.

### 4.3.1 Tutorial: Embed a network using the HMSM encoder

This tutorial shows how to embed a network using the HMSM (Hidden Metric Space Model) encoder. For more information on graph embedding methods available in LinkPred, consult Section 4.3 of the user guide.

1. In a file name `hmsm.cpp` write the following code:

```
#include <linkpred.hpp>
#include <iostream>
using namespace LinkPred;
int main() {
  long int seed = 777;
  // Read network from file
  auto net = UNetwork<>::read("Zakarays_Karate_Club.edges");
  // Create a HMSM encoder
  HMSM<> encoder(net, seed);
  // Set encoding dimension
  encoder.setDim(3);
  // Initialize the encoder
  encoder.init();
  // Embed the network
  encoder.encode();
  // Print node codes
  for (std::size_t i = 0; i < net->getNbNodes(); i++) {
    auto v = encoder.getNodeCode(i);
    std::cout << net->getLabel(i) << "\t";
    for (int k = 0; k < v.size(); k++) {
      std::cout << std::fixed << std::setprecision(4) << v[k]
          << "\t";
    }
    std::cout << std::endl;
  }
  return 0;
}
```

R This code is available in: `tutorials/code/graphalg/encoder/hmsm`

2. Compile your code:

```
$ mpiCC hmsm.cpp -o hmsm -fopenmp -lLinkPred
```

R Check Tutorial 1.2 if you face any compilation issues.

3. Run your code:

```
$ ./hmsm
```

The following is a partial output of this program:

```
1       16.0000 9.5499  -8.1565
2       9.0000  19.0151 -7.9892
3       10.0000 20.9290 -7.7570
4       6.0000  19.9847 -8.3575
5       3.0000  0.3878  -13.0939
6       4.0000  -3.1152 -15.7564
7       4.0000  -3.1436 -16.0556
8       4.0000  19.4511 -8.8470
```

```
9       5.0000  -19.0161        -2.7566
11      3.0000  0.2081  -13.0402
...
```

### 4.3.2 Tutorial: Embed a network using the LINE encoder

This tutorial shows how to embed a network using the LINE encoder. For more information on graph embedding methods available in LinkPred, consult Section 4.3 of the user guide.

1. In a file name `line.cpp` write the following code:

```
#include <linkpred.hpp>
#include <iostream>
using namespace LinkPred;
int main() {
  long int seed = 777;
  // Read network from file
  auto net = UNetwork<>::read("Zakarays_Karate_Club.edges");
  // Create a LINE encoder
  LINE<> encoder(net, seed);
  // Set encoding dimension
  encoder.setDim(3);
  // Initialize the encoder
  encoder.init();
  // Embed the network
  encoder.encode();
  // Print node codes
  for (std::size_t i = 0; i < net->getNbNodes(); i++) {
    auto v = encoder.getNodeCode(i);
    std::cout << net->getLabel(i) << "\t";
    for (int k = 0; k < v.size(); k++) {
      std::cout << std::fixed << std::setprecision(4) << v[k]
          << "\t";
    }
    std::cout << std::endl;
  }
  return 0;
}
```

R This code is available in: `tutorials/code/graphalg/encoder/line`

2. Compile your code:

```
$ mpiCC line.cpp -o line -fopenmp -lLinkPred
```

R Check Tutorial 1.2 if you face any compilation issues.

3. Run your code:

```
$ ./line
```

The following is a partial output of this program:

```
1       -0.1389 0.0465  0.0879
2       -0.1594 -0.0152 0.0996
3       0.0456  0.1431  -0.1103
4       0.0607  0.1393  -0.1405
5       0.0962  -0.1040 -0.0480
6       -0.0421 -0.0347 -0.0358
7       -0.0409 0.1416  -0.0189
8       -0.0325 0.0381  -0.0339
```

```
9       -0.0325 -0.1009 0.0468
11      -0.1361 0.0734  0.0281
...
```

### 4.3.3 Tutorial: Embed a network using the Node2Vec encoder

This tutorial shows how to embed a network using the Node2Vec encoder. For more information on graph embedding methods available in LinkPred, consult Section 4.3 of the user guide.

1. In a file name `node2vec.cpp` write the following code:

```
#include <linkpred.hpp>
#include <iostream>
using namespace LinkPred;
int main() {
  long int seed = 777;
  // Read network from file
  auto net = UNetwork<>::read("Zakarays_Karate_Club.edges");
  // Create a Node2Vec encoder
  Node2Vec<> encoder(net, seed);
  // Set encoding dimension
  encoder.setDim(3);
  // Initialize the encoder
  encoder.init();
  // Embed the network
  encoder.encode();
  // Print node codes
  for (std::size_t i = 0; i < net->getNbNodes(); i++) {
    auto v = encoder.getNodeCode(i);
    std::cout << net->getLabel(i) << "\t";
    for (int k = 0; k < v.size(); k++) {
      std::cout << std::fixed << std::setprecision(4) << v[k]
          << "\t";
    }
    std::cout << std::endl;
  }
  return 0;
}
```

R This code is available in: `tutorials/code/graphalg/encoder/node2vec`

2. Compile your code:

```
$ mpiCC node2vec.cpp -o node2vec -fopenmp -lLinkPred
```

R Check Tutorial 1.2 if you face any compilation issues.

3. Run your code:

```
$ ./node2vec
```

The following is a partial output of this program:

```
1       -1.2866 0.8575  -0.0964
2       -1.6074 -0.1674 0.0551
3       -1.1292 -0.1409 -0.3301
4       -1.3907 0.3220  0.0459
5       -1.2650 1.7383  -0.3889
6       -1.5062 1.7597  -0.4098
7       -1.3218 2.0357  -0.3095
8       -1.2982 0.1699  -0.0778
```

```
9       -1.1241 -0.2370 -0.4324
11      -1.4957 1.8402  -0.2160
...
```

# 5. Predictors

This chapter contains tutorials on how to use the link prediction algorithms available in LinkPred.

## 5.1 Link prediction algorithms in undirected networks

This section describes how to use link prediction algorithms in undirected networks.

### 5.1.1 Tutorial: Computing all scores

This tutorial shows how to compute the scores of all non-existing edges in a network. For more information on link predictors in LinkPred, consult Chapter 5 of the user guide.

1. In a file name `pred-all.cpp` write the following code:

```
#include <linkpred.hpp>
#include <iostream>
using namespace LinkPred;
int main() {
  // Read network from file
  auto net = DNetwork<>::read("Zakarays_Karate_Club.edges");
  // Create an instance of the directed ADA predictor
  DADAPredictor<> p(net);
  // Initialize predictor
  p.init();
  // Train predictor
  p.learn();
  // Allocate memory for storing scores
  std::vector<double> scores(net->getNbNonEdges());
  // Predict the score of all non-existing edges
  auto its = p.predictNeg(scores.begin());
  // Print scores
  std::cout << "#Start\tEnd\tScore\n";
  int k = 0;
  for (auto it = its.first; it != its.second; ++it) {
    auto i = net->start(*it);
    auto j = net->end(*it);
    std::cout << net->getLabel(i) << "\t" << net->getLabel(j)
        << "\t" << scores[k++] << std::endl;
  }
  return 0;
}
```

R This code is available in: `tutorials/code/predictors/dnetwork/predAll`

2. Compile your code:

```
$ mpiCC predAll.cpp -o predAll -fopenmp -lLinkPred
```

R Check Tutorial 1.2 if you face any compilation issues.

3. Run your code:

```
$ ./predAll
```

The first few lines of this programs' output are as follows:

```
#Start  End     Score
1       31      0.780271
1       10      0.333808
1       28      0.333808
1       29      0.736238
1       33      1.17053
1       17      0.961797
1       34      1.82913
```

```
1       15      0
1       16      0
...
```

## 5.1.2 Tutorial: Computing scores of specific edges

This tutorial shows how to compute the scores of specific edges in a network. For more information on link predictors in LinkPred, consult Chapter 5 of the user guide.

There are two ways to predict the scores of specific edges: using the method `score`, which computes the score of a single edges, and the method `predict`, which computes the score for a range of edges. This tutorials give an example to each of these methods.

1. In a file name `pred-score.cpp` write the following code:

```
#include <linkpred.hpp>
#include <iostream>
using namespace LinkPred;
int main() {
  // Read network from file
  auto net = UNetwork<>::read("Zakarays_Karate_Club.edges");
  // Create an instance of the KAB predictor
  UKABPredictor<> p(net);
  // Initialize predictor
  p.init();
  // Train predictor
  p.learn();
  // Compute the score for the two edges (1, 34) and (26,34)
  double sc = p.score(net->makeEdge(net->getID("1"), net->
     getID("34")));
  std::cout << "1\t34\t" << sc << std::endl;
  sc = p.score(net->makeEdge(net->getID("26"), net->getID("34
     ")));
  std::cout << "26\t34\t" << sc << std::endl;
  return 0;
}
```

**R** This code is available in: `tutorials/code/predictors/unetwork/pred`

**R** For performance reasons, the edges to be predicted are passed using the nodes' IDs and not their labels.

2. Compile your code:

```
$ mpiCC pred-score.cpp -o pred-score -fopenmp -lLinkPred
```

**R** Check Tutorial 1.2 if you face any compilation issues.

3. Run your code:

```
$ ./pred-score
```

The output of this program is as follows:

```
1       34      0.54199
26      34      0.468782
```

4. In a file name `pred-predict.cpp` write the following code:

```
#include <linkpred.hpp>
#include <iostream>
using namespace LinkPred;
```

```
int main() {
  // Read network from file
  auto net = UNetwork<>::read("Zakarays_Karate_Club.edges");
  // Create an instance of the KAB predictor
  UKABPredictor<> p(net);
  // Initialize predictor
  p.init();
  // Train predictor
  p.learn();
  // Create a vector to store edges
  std::vector<typename UNetwork<>::Edge> ev;
  // Push the two edges (1, 34) and (26, 34)
  ev.push_back(net->makeEdge(net->getID("1"), net->getID("34"
     )));
  ev.push_back(net->makeEdge(net->getID("26"), net->getID("34
     ")));
  // Allocate memory for storing scores
  std::vector<double> scores(2);
  // Predict the scores
  p.predict(ev.begin(), ev.end(), scores.begin());
  // Print scores
  std::cout << "#Start\tEnd\tScore\n";
  int k = 0;
  for (auto it = ev.begin(); it != ev.end(); ++it) {
    auto i = net->start(*it);
    auto j = net->end(*it);
    std::cout << net->getLabel(i) << "\t" << net->getLabel(j)
        << "\t" << scores[k++] << std::endl;
  }

  return 0;
}
```

R This code is available in: `tutorials/code/predictors/unetwork/pred`

5. Compile your code:

```
$ mpiCC pred-predict.cpp -o pred-predict -fopenmp -lLinkPred
```

6. Run your code:

```
$ ./pred-predict
```

The output of this program is as follows:

```
#Start  End     Score
1       34      0.54199
26      34      0.468782
```

### 5.1.3 Tutorial: Computing top scores

This tutorial shows how to compute the top edge scores. For more information on link predictors in LinkPred, consult Chapter 5 of the user guide.

1. In a file name `predTop.cpp` write the following code:

```
#include <linkpred.hpp>
#include <iostream>
using namespace LinkPred;
int main() {
  int k = 10; // Find top 10
  // Read network from file
  auto net = UNetwork<>::read("Zakarays_Karate_Club.edges");
  // Create an instance of the KAB predictor
  UKABPredictor<> p(net);
  // Initialize predictor
  p.init();
  // Train predictor
  p.learn();
  // Allocate memory for storing scores
  std::vector<double> scores(k);
  // Create a vector to store edges
  std::vector<typename UNetwork<>::Edge> ev(k);
  // Predict top scores
  k = p.top(k, ev.begin(), scores.begin());
  for (int l = 0; l < k; l++) {
    auto i = net->start(ev[l]);
    auto j = net->end(ev[l]);
    std::cout << net->getLabel(i) << "\t" << net->getLabel(j)
        << "\t" << scores[l] << std::endl;
  }
  return 0;
}
```

R This code is available in: `tutorials/code/predictors/unetwork/predTop`

2. Compile your code:

```
$ mpiCC predTop.cpp -o predTop -fopenmp -lLinkPred
```

R Check Tutorial 1.2 if you face any compilation issues.

3. Run your code:

```
$ ./predTop
```

The output of this program is as follows:

```
1       33      0.495478
1       17      0.540224
1       34      0.54199
2       34      0.505588
3       34      0.589745
5       6       0.465234
7       11      0.465234
34      26      0.468782
```

```
34       25       0.483836
24       25       0.454449
```

### 5.1.4 Tutorial: Encoder-classifier prediction

This tutorial shows how to predict links using an encoder-classifier predictor. For more information on link predictors in LinkPred, consult Chapter 5 of the user guide.

1. In a file named `ecl.cpp` write the following code:

```
#include <linkpred.hpp>
#include <iostream>
using namespace LinkPred;
int main() {
  int  k = 10;
  // Read network from file
  auto net = UNetwork<>::read("Zakarays_Karate_Club.edges");
  // Create a N2V encoder
  auto encoder =  std::make_shared<Node2Vec<>>(net, 777);
  // Create a logistic regresser
  auto classifier =  std::make_shared<LogisticRegresser
     <>>(0.001, 888);
  // Create an instance of the ECL predictor
  UECLPredictor<> p(net, encoder, classifier, 999);
  // Initialize predictor
  p.init();
  // Train predictor
  p.learn();
  // Allocate memory for storing scores
  std::vector<double> scores(k);
  // Create a vector to store edges
  std::vector<typename UNetwork<>::Edge> ev(k);
  // Predict top scores
  k = p.top(k, ev.begin(), scores.begin());
  for (int l = 0; l < k; l++) {
    auto i = net->start(ev[l]);
    auto j = net->end(ev[l]);
    std::cout << net->getLabel(i) << "\t" << net->getLabel(j)
        << "\t" << scores[l] << std::endl;
  }
  return 0;
}
```

R This code is available in: `tutorials/code/predictors/unetwork/ecl`

R For the names of available encoders and classifiers, consult the library reference manual.

2. Compile your code:

```
$ mpiCC ecl.cpp -o ecl -fopenmp -lLinkPred
```

R Check Tutorial 1.2 if you face any compilation issues.

3. Run your code:

```
$ ./ecl
```

The output of this program is as follows:

```
1       10      0.506023
1       34      0.741211
2       5       0.521754
3       5       0.561345
3       13      0.501856
32      31      0.527168
32      10      0.616398
33      17      0.710924
34      26      0.584236
34      25      0.607985
```

### 5.1.5 Tutorial: Encoder-similarity prediction

This tutorial shows how to predict links using an encoder-similarity predictor. For more information on link predictors in LinkPred, consult Chapter 5 of the user guide.

1. In a file name `esm.cpp` write the following code:

```
#include <linkpred.hpp>
#include <iostream>
using namespace LinkPred;
int main() {
  int  k = 10;
  // Read network from file
  auto net = UNetwork<>::read("Zakarays_Karate_Club.edges");
  // Create a LIN encoder
  auto encoder =  std::make_shared<LINE<>>(net, 777);
  // Create an L2 similarity object
  auto simMeasure =  std::make_shared<L2Sim>();
  // Create an instance of the ESM predictor
  UESMPredictor<> p(net, encoder, simMeasure);
  // Initialize predictor
  p.init();
  // Train predictor
  p.learn();
  // Allocate memory for storing scores
  std::vector<double> scores(k);
  // Create a vector to store edges
  std::vector<typename UNetwork<>::Edge> ev(k);
  // Predict top scores
  k = p.top(k, ev.begin(), scores.begin());
  for (int l = 0; l < k; l++) {
    auto i = net->start(ev[l]);
    auto j = net->end(ev[l]);
    std::cout << net->getLabel(i) << "\t" << net->getLabel(j)
        << "\t" << scores[l] << std::endl;
  }
  return 0;
}
```

**R** This code is available in: `tutorials/code/predictors/unetwork/esm`

**R** For the names of available encoders and similarity measures, consult the library reference manual.

2. Compile your code:

```
$ mpiCC esm.cpp -o esm -fopenmp -lLinkPred
```

**R** Check Tutorial 1.2 if you face any compilation issues.

3. Run your code:

```
$ ./esm
```

The output of this program is as follows:

```
1       23      -0.0658803
4       19      -0.0623656
5       6       -0.0687798
6       29      -0.0682746
6       30      -0.0713388
11      31      -0.0725597
12      28      -0.0607567
12      16      -0.0715096
28      23      -0.0699596
29      30      -0.051615
```

## 5.2 Link prediction algorithms in directed networks

This section describes how to use link prediction algorithms in directed networks.

### 5.2.1 Tutorial: Computing all scores

This tutorial shows how to compute the scores of all non-existing edges in a network. For more information on link predictors in LinkPred, consult Chapter 5 of the user guide.

1. In a file name `pred-all.cpp` write the following code:

```
#include <linkpred.hpp>
#include <iostream>
using namespace LinkPred;
int main() {
  // Read network from file
  auto net = UNetwork<>::read("Zakarays_Karate_Club.edges");
  // Create an instance of the KAB predictor
  UKABPredictor<> p(net);
  // Initialize predictor
  p.init();
  // Train predictor
  p.learn();
  // Allocate memory for storing scores
  std::vector<double> scores(net->getNbNonEdges());
  // Predict the score of all non-existing edges
  auto its = p.predictNeg(scores.begin());
  // Print scores
  std::cout << "#Start\tEnd\tScore\n";
  int k = 0;
  for (auto it = its.first; it != its.second; ++it) {
    auto i = net->start(*it);
    auto j = net->end(*it);
    std::cout << net->getLabel(i) << "\t" << net->getLabel(j)
        << "\t" << scores[k++] << std::endl;
  }
  return 0;
}
```

> **R** This code is available in: `tutorials/code/predictors/unetwork/predAll`

2. Compile your code:

```
$ mpiCC predAll.cpp -o predAll -fopenmp -lLinkPred
```

> **R** Check Tutorial 1.2 if you face any compilation issues.

3. Run your code:

```
$ ./predAll
```

The first few lines of this programs' output are as follows:

```
#Start  End     Score
1       31      0.392467
1       10      0.268335
1       28      0.280569
1       29      0.358308
1       33      0.495478
1       17      0.540224
1       34      0.54199
```

```
1       15      0
1       16      0
...
```

### 5.2.2 Tutorial: Computing scores of specific edges

This tutorial shows how to compute the scores of specific edges in a network. For more information on link predictors in LinkPred, consult Chapter 5 of the user guide.

There are two ways to predict the scores of specific edges: using the method `score`, which computes the score of a single edges, and the method `predict`, which computes the score for a range of edges. This tutorials give an example to each of these methods.

1. In a file name `pred-score.cpp` write the following code:

```
#include <linkpred.hpp>
#include <iostream>
using namespace LinkPred;
int main() {
  // Read network from file
  auto net = DNetwork<>::read("Zakarays_Karate_Club.edges");
  // Create an instance of the directed ADA predictor
  DADAPredictor<> p(net);
  // Initialize predictor
  p.init();
  // Train predictor
  p.learn();
  // Compute the score for the two edges (1, 34) and (26,34)
  double sc = p.score(net->makeEdge(net->getID("1"), net->
      getID("34")));
  std::cout << "1\t34\t" << sc << std::endl;
  sc = p.score(net->makeEdge(net->getID("26"), net->getID("34
      ")));
  std::cout << "26\t34\t" << sc << std::endl;
  return 0;
}
```

R This code is available in: `tutorials/code/predictors/dnetwork/pred`

R For performance reasons, the edges to be predicted are passed using the nodes' IDs and not their labels.

2. Compile your code:

```
$ mpiCC pred-score.cpp -o pred-score -fopenmp -lLinkPred
```

R Check Tutorial 1.2 if you face any compilation issues.

3. Run your code:

```
$ ./pred-score
```

The output of this program is as follows:

```
1       34      1.82913
26      34      0.836724
```

4. In a file name `pred-predict.cpp` write the following code:

```
#include <linkpred.hpp>
#include <iostream>
using namespace LinkPred;
```

```
int main() {
  // Read network from file
  auto net = DNetwork<>::read("Zakarays_Karate_Club.edges");
  // Create an instance of the directed CNE predictor
  DCNEPredictor<> p(net);
  // Initialize predictor
  p.init();
  // Train predictor
  p.learn();
  // Create a vector to store edges
  std::vector<typename UNetwork<>::Edge> ev;
  // Push the two edges (1, 34) and (26, 34)
  ev.push_back(net->makeEdge(net->getID("1"), net->getID("34"
     )));
  ev.push_back(net->makeEdge(net->getID("26"), net->getID("34
     ")));
  // Allocate memory for storing scores
  std::vector<double> scores(2);
  // Predict the scores
  p.predict(ev.begin(), ev.end(), scores.begin());
  // Print scores
  std::cout << "#Start\tEnd\tScore\n";
  int k = 0;
  for (auto it = ev.begin(); it != ev.end(); ++it) {
    auto i = net->start(*it);
    auto j = net->end(*it);
    std::cout << net->getLabel(i) << "\t" << net->getLabel(j)
        << "\t" << scores[k++] << std::endl;
  }

  return 0;
}
```

This code is available in: `tutorials/code/predictors/dnetwork/pred`

5. Compile your code:

```
$ mpiCC pred-predict.cpp -o pred-predict -fopenmp -lLinkPred
```

6. Run your code:

```
$ ./pred-predict
```

The output of this program is as follows:

```
#Start  End     Score
1       34      4
26      34      2
```

### 5.2.3 Tutorial: Computing top scores

This tutorial shows how to compute the top edge scores. For more information on link predictors in LinkPred, consult Chapter 5 of the user guide.

1. In a file name `predTop.cpp` write the following code:

```
#include <linkpred.hpp>
#include <iostream>
using namespace LinkPred;
int main() {
  int k = 10; // Find top 10
  // Read network from file
  auto net = DNetwork<>::read("Zakarays_Karate_Club.edges");
  // Create an instance of the directed CNE predictor
  DCNEPredictor<> p(net);
  // Initialize predictor
  p.init();
  // Train predictor
  p.learn();
  // Allocate memory for storing scores
  std::vector<double> scores(k);
  // Create a vector to store edges
  std::vector<typename UNetwork<>::Edge> ev(k);
  // Predict top scores
  k = p.top(k, ev.begin(), scores.begin());
  for (int l = 0; l < k; l++) {
    auto i = net->start(ev[l]);
    auto j = net->end(ev[l]);
    std::cout << net->getLabel(i) << "\t" << net->getLabel(j)
        << "\t" << scores[l] << std::endl;
  }
  return 0;
}
```

R This code is available in: `tutorials/code/predictors/dnetwork/predTop`

2. Compile your code:

```
$ mpiCC predTop.cpp -o predTop -fopenmp -lLinkPred
```

R Check Tutorial 1.2 if you face any compilation issues.

3. Run your code:

```
$ ./predTop
```

The output of this program is as follows:

```
1       1       16
2       2       9
3       3       10
3       34      6
4       4       6
32      32      6
33      33      12
34      3       6
```

```
34        34        16
24        24        5
```

# 6. Performance Evaluation

This chapter covers the topic of test data setup and performance evaluation.

## 6.1 Data setup

This section describes the data setup process for performance evaluation, including, creating test data by removing and adding edges, and loading test data from file.

R LinkPred provides various methods to generate test data that offer flexibility and fit different use scenario, in particular with very large networks. This sections contains basic use scenarios, for more advanced options, the readers is invited to consult the user guide and reference manual.

### 6.1.1 Tutorial: Creating test data by removing edges

This tutorial shows how to create test data by removing edges from a ground-truth network. For more information on performance evaluation routines in LinkPred and the associated terminology, consult Chapter 6 of the user guide.

1. In a file named `create-rem.cpp` write the following code:

```
#include <linkpred.hpp>
#include <iostream>
using namespace LinkPred;
int main() {
  // Remove 20% of the edges
  double remRatio = 0.2;
  long int seed = 777;
  // Read network from file
  auto net = UNetwork<>::read("net.edges");
  // Create the test data
  auto testData = NetworkManipulator<>::createTestDataRem(net
      , remRatio, seed);
  std::cout << "Reference␣network:\n";
  testData.getRefNet()->print();
  std::cout << "Observed␣network:\n";
  testData.getObsNet()->print();
  std::cout << "Positive␣examples␣(removed␣edges):" << std::
      endl;
  for (auto it = testData.posBegin(); it != testData.posEnd()
      ; ++it) {
    auto i = net->start(*it);
    auto j = net->end(*it);
    std::cout << net->getLabel(i) << "\t" << net->getLabel(j)
        << std::endl;
  }
  std::cout << "Negative␣examples:" << std::endl;
  for (auto it = testData.negBegin(); it != testData.negEnd()
      ; ++it) {
    auto i = net->start(*it);
    auto j = net->end(*it);
    std::cout << net->getLabel(i) << "\t" << net->getLabel(j)
        << std::endl;
  }
  return 0;
}
```

R This code is available in: `tutorials/code/performance/data-setup/create-rem`

The input reference network contained in `net.edges` is:

```
1       2
2       3
3       4
4       5
5       6
6       7
7       8
8       1
```

```
1       3
3       5
5       7
7       1
```

2. Compile your code:

```
$ mpiCC create-rem.cpp -o create-rem -fopenmp -lLinkPred
```

Check Tutorial 1.2 if you face any compilation issues.

3. Run your code:

```
$ ./create-rem
```

The output of this program is as follows:

```
Reference network:
1       2
1       3
1       7
1       8
2       3
3       4
3       5
4       5
5       6
5       7
6       7
7       8
Observed network:
1       2
1       3
1       7
1       8
2       3
3       4
3       5
5       7
6       7
7       8
Positive examples (removed edges):
4       5
5       6
Negative examples:
1       4
1       5
1       6
2       4
2       5
2       6
2       7
2       8
3       6
3       7
3       8
4       6
```

```
4          7
4          8
5          8
6          8
```

### 6.1.2 Tutorial: Creating test data by adding edges

This tutorial shows how to create test data by adding edges to a ground-truth network. For more information on performance evaluation routines in LinkPred and the associated terminology, consult Chapter 6 of the user guide.

1. In a file named `create-add.cpp` write the following code:

```
#include <linkpred.hpp>
#include <iostream>
using namespace LinkPred;
int main() {
  // Add 20% of the edges
  double addRatio = 0.2;
  long int seed = 888;
  // Read network from file
  auto net = UNetwork<>::read("net.edges");
  // Create the test data
  auto testData = NetworkManipulator<>::createTestDataAdd(net
      , addRatio, seed);
  std::cout << "Reference␣network:\n";
  testData.getRefNet()->print();
  std::cout << "Observed␣network:\n";
  testData.getObsNet()->print();
  std::cout << "Positive␣examples:" << std::endl;
  for (auto it = testData.posBegin(); it != testData.posEnd()
      ; ++it) {
    auto i = net->start(*it);
    auto j = net->end(*it);
    std::cout << net->getLabel(i) << "\t" << net->getLabel(j)
        << std::endl;
  }
  std::cout << "Negative␣examples␣(added␣edges):" << std::
      endl;
  for (auto it = testData.negBegin(); it != testData.negEnd()
      ; ++it) {
    auto i = net->start(*it);
    auto j = net->end(*it);
    std::cout << net->getLabel(i) << "\t" << net->getLabel(j)
        << std::endl;
  }
  return 0;
}
```

R This code is available in: `tutorials/code/performance/data-setup/create-add`

The input reference network contained in `net.edges` is:

```
1       2
2       3
3       4
4       5
5       6
6       7
7       8
8       1
```

```
1       3
3       5
5       7
7       1
```

2. Compile your code:

```
$ mpiCC create-add.cpp -o create-add -fopenmp -lLinkPred
```

R Check Tutorial 1.2 if you face any compilation issues.

3. Run your code:

```
$ ./create-add
```

The output of this program is as follows:

```
Reference network:
1       2
1       3
1       7
1       8
2       3
3       4
3       5
4       5
5       6
5       7
6       7
7       8
Observed network:
1       2
1       3
1       6
1       7
1       8
2       3
2       4
3       4
3       5
4       5
5       6
5       7
6       7
7       8
Positive examples:
1       2
1       3
1       7
1       8
2       3
3       4
3       5
4       5
5       6
5       7
6       7
```

```
7       8
Negative examples (added edges):
1       6
2       4
```

### 6.1.3 Tutorial: Loading test data obtained by removing edges

This tutorial shows how to load from file test data obtained by removing edges from a ground-truth network. For more information on performance evaluation routines in LinkPred and the associated terminology, consult Chapter 6 of the user guide.

1. In a file named `load-rem.cpp` write the following code:

```
#include <linkpred.hpp>
#include <iostream>
using namespace LinkPred;
int main() {
  // Load test data
  auto testData = NetworkManipulator<>::loadTestDataRem("net-
     obs.edges", "net-rem.edges");
  std::cout << "Reference␣network:\n";
  auto refNet = testData.getRefNet();
  refNet->print();
  std::cout << "Observed␣network:\n";
  auto obsNet = testData.getObsNet();
  obsNet->print();
  std::cout << "Positive␣examples␣(removed␣edges):" << std::
     endl;
  for (auto it = testData.posBegin(); it != testData.posEnd()
     ; ++it) {
    auto i = refNet->start(*it);
    auto j = refNet->end(*it);
    std::cout << refNet->getLabel(i) << "\t" << refNet->
       getLabel(j) << std::endl;
  }
  std::cout << "Negative␣examples:" << std::endl;
  for (auto it = testData.negBegin(); it != testData.negEnd()
     ; ++it) {
    auto i = refNet->start(*it);
    auto j = refNet->end(*it);
    std::cout << refNet->getLabel(i) << "\t" << refNet->
       getLabel(j) << std::endl;
  }
  return 0;
}
```

R This code is available in: `tutorials/code/performance/data-setup/load-rem`

The file `net-obs.edges` contains the set of observed edges:

```
1       2
1       3
1       7
1       8
2       3
3       4
3       5
5       7
6       7
7       8
```

The file `net-rem.edges` contains the set of removed edges:

```
4       5
5       6
```

2. Compile your code:

```
$ mpiCC load-rem.cpp -o load-rem -fopenmp -lLinkPred
```

R Check Tutorial 1.2 if you face any compilation issues.

3. Run your code:

```
$ ./load-rem
```

The output of this program is as follows:

```
Reference network:
1       2
1       3
1       7
1       8
2       3
3       4
3       5
7       8
7       5
7       6
4       5
5       6
Observed network:
1       2
1       3
1       7
1       8
2       3
3       4
3       5
7       8
7       5
7       6
Positive examples (removed edges):
4       5
5       6
Negative examples:
1       4
1       5
1       6
2       7
2       8
2       4
2       5
2       6
3       7
3       8
3       6
7       4
8       4
8       5
```

```
8         6
4         6
```

### 6.1.4 Tutorial: Loading test data obtained by adding edges

This tutorial shows how to load from file test data obtained by adding edges to a ground-truth network. For more information on performance evaluation routines in LinkPred and the associated terminology, consult Chapter 6 of the user guide.

1. In a file named `load-add.cpp` write the following code:

```
#include <linkpred.hpp>
#include <iostream>
using namespace LinkPred;
int main() {
  // Load test data
  auto testData = NetworkManipulator<>::loadTestDataAdd("net-
     obs.edges", "net-add.edges");
  std::cout << "Reference␣network:\n";
  auto refNet = testData.getRefNet();
  refNet->print();
  std::cout << "Observed␣network:\n";
  auto obsNet = testData.getObsNet();
  obsNet->print();
  std::cout << "Positive␣examples:" << std::endl;
  for (auto it = testData.posBegin(); it != testData.posEnd()
     ; ++it) {
    auto i = refNet->start(*it);
    auto j = refNet->end(*it);
    std::cout << refNet->getLabel(i) << "\t" << refNet->
       getLabel(j) << std::endl;
  }
  std::cout << "Negative␣examples␣(added␣edges):" << std::
     endl;
  for (auto it = testData.negBegin(); it != testData.negEnd()
     ; ++it) {
    auto i = refNet->start(*it);
    auto j = refNet->end(*it);
    std::cout << refNet->getLabel(i) << "\t" << refNet->
       getLabel(j) << std::endl;
  }
  return 0;
}
```

> R This code is available in: `tutorials/code/performance/data-setup/load-add`

The file `net-obs.edges` contains the set of observed edges:

```
1	2
1	3
1	6
1	7
1	8
2	3
2	4
3	4
3	5
4	5
5	6
```

```
5       7
6       7
7       8
```

The file `net-add.edges` contains the set of added edges:

```
1       6
2       4
```

2. Compile your code:

```
$ mpiCC load-add.cpp -o load-add -fopenmp -lLinkPred
```

R Check Tutorial 1.2 if you face any compilation issues.

3. Run your code:

```
$ ./load-add
```

The output of this program is as follows:

```
Reference network:
1       2
1       3
1       7
1       8
2       3
3       4
3       5
6       7
6       5
7       8
7       5
4       5
Observed network:
1       2
1       3
1       6
1       7
1       8
2       3
2       4
3       4
3       5
6       7
6       5
7       8
7       5
4       5
Positive examples:
1       2
1       3
1       7
1       8
2       3
3       4
3       5
6       7
```

```
6       5
7       8
7       5
4       5
Negative examples (added edges):
1       6
2       4
```

## 6.2 Performance evaluation

This section describes the classes used for performance evaluation.

### 6.2.1 Tutorial: Using performance measures

This tutorial shows how to use performance measures to evaluate the performance of a link prediction algorithm. For more information on performance evaluation routines in LinkPred and the associated terminology, consult Chapter 6 of the user guide.

1. In a file named perfmeasures.cpp write the following code:

```
#include <linkpred.hpp>
#include <iostream>
using namespace LinkPred;
int main() {
  // Remove 10% of the edges
  double remRatio = 0.1;
  long int seed = 888;
  // Read network from file
  auto refNet = UNetwork<>::read("Zakarays_Karate_Club.edges"
     );
  // Create the test data
  auto testData = NetworkManipulator<>::createTestDataRem(
     refNet, remRatio, seed);
  // Lock test data
  testData.lock();
  // Create an instance of the KAB predictor
  auto p = std::make_shared<UKABPredictor<>>(testData.
     getObsNet());
  // Initialize predictor
  p->init();
  // Train predictor
  p->learn();
  // Create a prediction result object
  auto predResults = std::make_shared<PredResults<>>(testData
     , p);
  // A map to store prediction results
  PerfResults res;
  // Create a ROC object
  ROC<> roc;
  // Compute ROCAUC
  roc.eval(predResults, res);
  std::cout << "ROCAUC:␣" << res.at(roc.getName()) << std::
     endl;
  // Create a PR object
  PR<> pr;
  // Compute PRAUC
  pr.eval(predResults, res);
  std::cout << "PRAUC:␣" << res.at(pr.getName()) << std::endl
     ;
  // Create a TPR object
  TPR<> tpr(testData.getNbPos());
  // Compute TPR
  tpr.eval(predResults, res);
  std::cout << "TPR:␣" << res.at(tpr.getName()) << std::endl;
  return 0;
}
```

R This code is available in: tutorials/code/performance/perfeval/perfmeasures

2. Compile your code:

```
$ mpiCC perfmeasures.cpp -o perfmeasures -fopenmp -lLinkPred
```

   R Check Tutorial 1.2 if you face any compilation issues.

3. Run your code:

```
$ ./perfmeasures
```

   The output of this program is as follows:

```
ROCAUC: 0.712215
PRAUC: 0.224628
TPR: 0.25
```

### 6.2.2 Tutorial: Using the class `PerfEvaluator` for performance evaluation

This tutorial shows how to use the class `PerfEvaluator` to evaluate the performance of several link predictors based on several performance measures. For more information on performance evaluation routines in LinkPred and the associated terminology, consult Chapter 6 of the user guide.

1. In a file named `perfevaluator.cpp` write the following code:

```
#include <linkpred.hpp>
#include <iostream>
using namespace LinkPred;
int main() {
  // Remove 10% of the edges
  double remRatio = 0.1;
  long int seed = 888;
  // Read network from file
  auto refNet = UNetwork<>::read("Zakarays_Karate_Club.edges"
     );
  // Create the test data
  auto testData = NetworkManipulator<>::createTestDataRem(
     refNet, remRatio, seed);
  // Lock test data
  testData.lock();
  auto obsNet = testData.getObsNet();
  // Create an evaluator object
  PerfEvaluator<> perf(testData);
  // Create an ADA predictor
  auto ada = std::make_shared<UADAPredictor<>>(obsNet);
  // Add it to the evaluator
  perf.addPredictor(ada);
  // Create a CNE predictor
  auto cne = std::make_shared<UCNEPredictor<>>(obsNet);
  // Add it to the evaluator
  perf.addPredictor(cne);
  // Create a ROC object
  auto roc = std::make_shared<ROC<>>();
  // Add it to the evaluator
  perf.addPerfMeasure(roc);
  // Create a PR object
  auto pr = std::make_shared<PR<>>();
  // Add it to the evaluator
  perf.addPerfMeasure(pr);
  // Run evaluation
  perf.eval();
  // Print results
  for (auto it = perf.resultsBegin(); it != perf.resultsEnd()
     ; ++it) {
    std::cout << it->first << "\t" << it->second << std::endl
       ;
  }
  return 0;
}
```

R This code is available in: `tutorials/code/performance/perfeval/perfevaluator`

R This code executes one test run. To execute multiple test runs, it is possible to enclose the relevant parts in a for loop or use the class `PerfEvalExp` as described in the next tutorial.

2. Compile your code:

```
$ mpiCC perfevaluator.cpp -o perfevaluator -fopenmp -
   lLinkPred
```

R Check Tutorial 1.2 if you face any compilation issues.

3. Run your code:

```
$ ./perfevaluator
```

The output of this program is as follows:

```
PRADA   0.207284
PRCNE   0.183308
ROCADA  0.695135
ROCCNE  0.664467
```

### 6.2.3 Tutorial: Using the class `PerfEvalExp` for performance evaluation

This tutorial shows how to use the class `PerfEvalExp` to evaluate the performance of several link predictors based on several performance measures on multiple test runs. For more information on performance evaluation routines in LinkPred and the associated terminology, consult Chapter 6 of the user guide.

1. In a file named `perfevalexp.cpp` write the following code:

```
#include <linkpred.hpp>
#include <iostream>
using namespace LinkPred;
// This is a factory to create predictors and performance
   measures
class Factory: public PEFactory<> {
public:
  // This method creates predictors
  virtual std::vector<std::shared_ptr<ULPredictor<>>>
     getPredictors(std::shared_ptr<UNetwork<> const> obsNet)
     {
    std::vector<std::shared_ptr<ULPredictor<>>> prs;
    // Add ADA
    prs.push_back(std::make_shared<URALPredictor<>>(obsNet));
    // Add KAB
    prs.push_back(std::make_shared<UKABPredictor<>>(obsNet));
    return prs;
  }
  // This method creates performance measures
  virtual std::vector<std::shared_ptr<PerfMeasure<>>>
     getPerfMeasures(TestData<> const & testData) {
    std::vector<std::shared_ptr<PerfMeasure<>>> pms;
    // Add PR
    pms.push_back(std::make_shared<PR<>>());
    // Add ROC
    pms.push_back(std::make_shared<ROC<>>());
    return pms;
  }
  virtual ~Factory() = default;
};
int main() {
  // Remove 10% of the edges
  double remRatio = 0.1;
  long int seed = 888;
  // Read network from file
  auto refNet = UNetwork<>::read("Zakarays_Karate_Club.edges"
     );
  // The parameters of our experiment
  PerfeEvalExpDescp<> ped;
  // Set the reference network
  ped.refNet = refNet;
  // We run 10 tests
  ped.nbTestRuns = 10;
  ped.seed = 777;
  // Create the factory
  auto factory = std::make_shared<Factory>();
  // Create the experiment object
  PerfEvalExp<> exp(ped, factory);
```

```
  // Run the experiment
  exp.run();
  return 0;
}
```

R This code is available in: tutorials/code/performance/perfeval/perfevalexp

2. Compile your code:

```
$ mpiCC perfevalexp.cpp -o perfevalexp -fopenmp -lLinkPred
```

R Check Tutorial 1.2 if you face any compilation issues.

3. Run your code:

```
$ ./perfevalexp
```

The output of this program is as follows:

```
# n: 34 m: 78
#ratio  PRKAB   PRRAL   ROCKAB  ROCRAL
0.10    0.1159  0.0744  0.8615  0.8028
0.10    0.2231  0.1001  0.7943  0.7823
0.10    0.0398  0.0334  0.6945  0.6712
0.10    0.1787  0.1617  0.6417  0.6219
0.10    0.0196  0.0170  0.5817  0.5487
0.10    0.2072  0.1867  0.8527  0.8386
0.10    0.0198  0.0159  0.5705  0.5167
0.10    0.0901  0.0712  0.8834  0.8359
0.10    0.1207  0.0841  0.8962  0.8617
0.10    0.2244  0.1221  0.7650  0.7433
#Time: 64.3621 ms
```

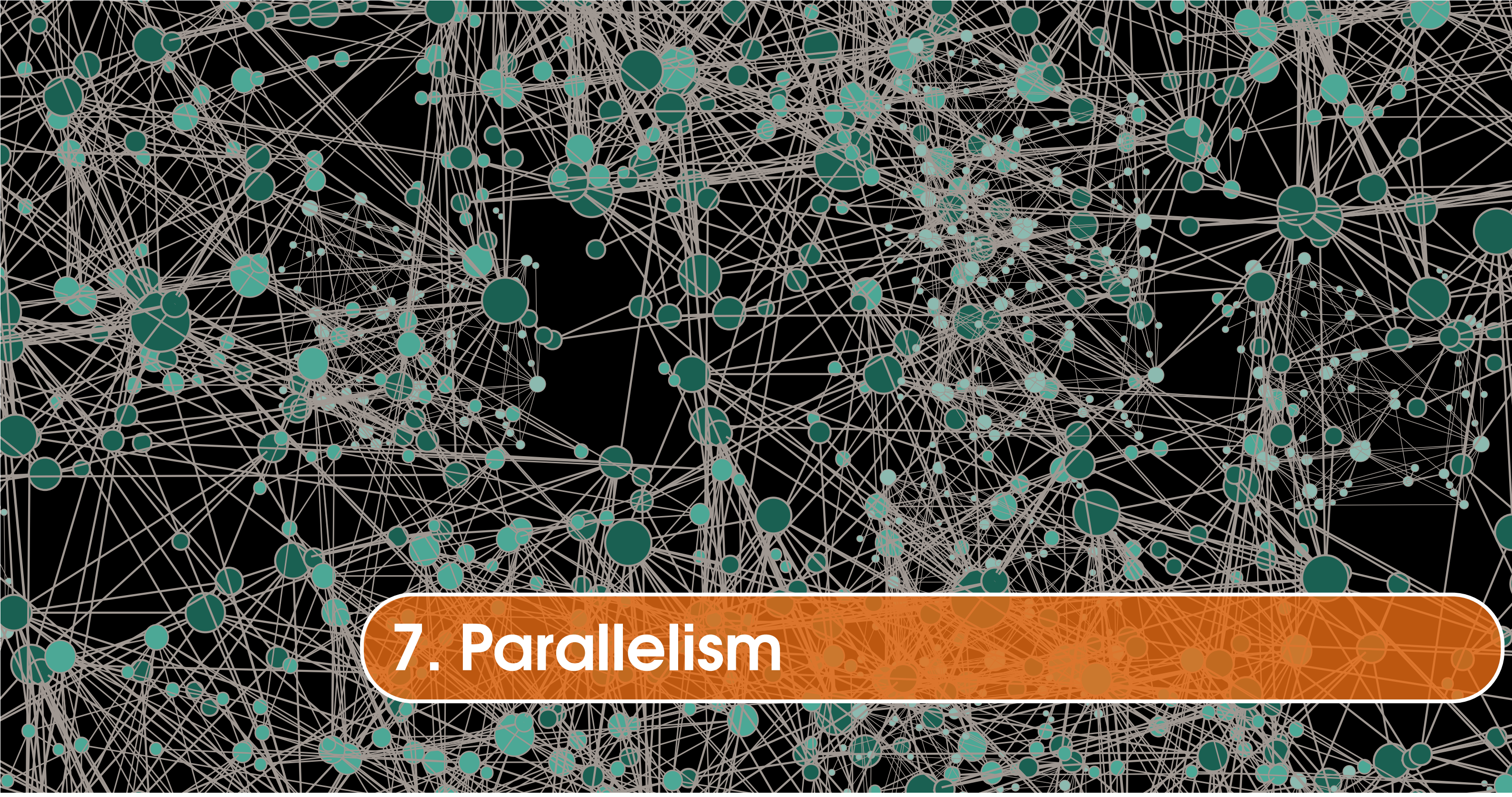

# 7. Parallelism

This chapter shows how to run LinkPred algorithms on shared and distributed memory architectures. Most link prediction algorithms included in LinkPred support shared memory parallelism, and many of them (especially local predictors) also support distributed memory parallelism, which allows the library to handle very large networks.

## 7.1 Shared memory parallelism

This section shows how to run LinkPred in parallel on shared memory architectures. Shared memory parallelism in LinkPred is implements using OpenMP. Parallelism is controlled at the object level by calling the method `setParallel`. For example, in a link predictor, this is achieved by:

```
predictor->setParallel(true);
```

The same applies for parallel measures:

```
measure->setParallel(true);
```

R When applicable, use the environment variable `OMP_NUM_THREADS` to control the number of threads, for instance:

```
$ export OMP_NUM_THREADS=4
```

### 7.1.1 Tutorial: Computing the score of all negative links in parallel

This tutorial shows how to compute the scores for all negative links of a network in parallel. For more information on parallelism in LinkPred, consult Chapter 7 of the user guide.

1. In a file named `cnepar.cpp` write the following code:

```
#include <linkpred.hpp>
#include <iostream>
using namespace LinkPred;
int main() {
  // Enable nested parallelism
  omp_set_nested(1);
  // Read network from file
  auto net = UNetwork<>::read("Infectious.edges");
  // Create a CNE predictor
  UCNEPredictor<> p(net);
  // Enable parllelism
  p.setParallel(true);
  // Initialize predictor
  p.init();
  // Train predictor
  p.learn();
  // Allocate memory for storing scores
  std::vector<double> scores(net->getNbNonEdges());
  // Predict the score of all non-existing edges
  auto its = p.predictNeg(scores.begin());
  // Print scores
  std::cout << "#Start\tEnd\tScore\n";
  int k = 0;
  for (auto it = its.first; it != its.second; ++it) {
    auto i = net->start(*it);
    auto j = net->end(*it);
    std::cout << net->getLabel(i) << "\t" << net->getLabel(j)
         << "\t" << scores[k++] << std::endl;
  }
  return 0;
}
```

R This code is available in: `tutorials/code/parallel/shared/cnepar`

2. Compile your code:

```
$ mpiCC cnepar.cpp -o cnepar -fopenmp -lLinkPred
```

R Check Tutorial 1.2 if you face any compilation issues.

3. Run your code:

```
$ ./cnepar
```

A partial output of this program is as follows:

```
#Start  End     Score
100     10      0
100     11      0
100     113     7
```

```
100     12      0
100     13      0
100     14      0
100     15      0
100     16      0
100     107     10
...
```

### 7.1.2 Tutorial: Computing the top edge scores in parallel

This tutorial shows how to compute the top edge scores in parallel. For more information on parallelism in LinkPred, consult Chapter 7 of the user guide.

1. In a file named `kabpar.cpp` write the following code:

```
#include <linkpred.hpp>
#include <iostream>
using namespace LinkPred;
int main() {
  int k = 10; // Find top 10
  // Read network from file
  auto net = UNetwork<>::read("Infectious.edges");
  // Create an instance of the KAB predictor
  UKABPredictor<> p(net);
  // Enable parllelism
  p.setParallel(true);
  // Initialize predictor
  p.init();
  // Train predictor
  p.learn();
  // Allocate memory for storing scores
  std::vector<double> scores(k);
  // Create a vector to store edges
  std::vector<typename UNetwork<>::Edge> ev(k);
  // Predict top scores
  k = p.top(k, ev.begin(), scores.begin());
  for (int l = 0; l < k; l++) {
    auto i = net->start(ev[l]);
    auto j = net->end(ev[l]);
    std::cout << net->getLabel(i) << "\t" << net->getLabel(j)
        << "\t" << scores[l] << std::endl;
  }
  return 0;
}
```

   R This code is available in: `tutorials/code/parallel/shared/kabpar`

2. Compile your code:

```
$ mpiCC kabpar.cpp -o kabpar -fopenmp -lLinkPred
```

   R Check Tutorial 1.2 if you face any compilation issues.

3. Run your code:

```
$ ./kabpar
```

   A partial output of this program is as follows:

```
102     109     0.698612
109     91      0.701562
12      19      0.726582
169     178     0.702122
164     155     0.698949
154     181     0.695083
51      39      0.708767
```

```
272     309     0.704433
30      44      0.713792
389     367     0.715296
```

## 7.2 Distributed memory parallelism

This section shows how to run LinkPred in parallel on distributed memory architectures. Distributed memory parallelism in LinkPred is implements using MPI and is controlled at the object level by calling the method `setDistributed`. For example, in a link predictor, this is achieved by:

```
predictor->setDistributed(true);
```

The same applies for parallel measures:

```
measure->setDistributed(true);
```

### 7.2.1 Tutorial: Computing the top edge scores distributively

This tutorial shows how to compute the scores for all negative links of a network distributively. For more information on parallelism in LinkPred, consult Chapter 7 of the user guide.

1. In a file named `raldist.cpp` write the following code:

```
#include <linkpred.hpp>
#include <iostream>
using namespace LinkPred;
int main(int argc, char*argv[]) {
  // Initialize MPI
  MPI_Init(&argc, &argv);
  std::size_t k = 10;
  // Read network from file
  auto net = UNetwork<>::read("Infectious.edges");
  // Create an RAL predictor
  URALPredictor<> p(net);
  // Enable distributed processing
  p.setDistributed(true);
  // Initialize predictor
  p.init();
  // Train predictor
  p.learn();
  // Allocate memory to store results
  std::vector<typename UNetwork<>::Edge> edges(k);
  std::vector<double> scores(k);
  // Find top k edges
  k = p.top(k, edges.begin(), scores.begin());
  int procID;
  // Get local process ID
  MPI_Comm_rank(MPI_COMM_WORLD, &procID);
  // Print tyhe results
  if (procID == 0) {
    std::cout << "#Start\tEnd\tScore\n";
  }
  for (std::size_t i = 0; i < k; i++) {
    std::cout << net->getLabel(net->start(edges[i])) << "\t"
        << net->getLabel(net->end(edges[i])) << "\t" << scores
        [i] << std::endl;
  }
  // Finalize MPI
  MPI_Finalize();
  return 0;
}
```

This code is available in: `tutorials/code/parallel/distributed/raldist`

2. Compile your code:

```
$ mpiCC raldist.cpp -o raldist -fopenmp -lLinkPred
```

Check Tutorial 1.2 if you face any compilation issues.

3. Run your code (here we are running the code on 4 nodes):

```
$ mpirun -n 4 ./raldist
```

A partial output of this program is as follows:

```
#Start  End     Score
169     178     0.912642
144     142     0.886008
51      39      0.985052
265     297     0.811915
300     295     0.806431
197     237     0.836456
257     299     0.864928
257     294     0.887479
261     292     0.973033
389     367     0.965622
```

### 7.2.2 Tutorial: Computing the area under the ROC curve distributively

This tutorial shows how to compute the area under the ROC curve distributively. For more information on parallelism in LinkPred, consult Chapter 7 of the user guide.

1. In a file named `rocstrmdist.cpp` write the following code:

```
#include <linkpred.hpp>
#include <iostream>
using namespace LinkPred;
int main(int argc, char*argv[]) {
  double remRatio = 0.1;
  long int seed = 777;
  // Initialize MPI
  MPI_Init(&argc, &argv);
  int procID = 0;
  // Get local process ID
  MPI_Comm_rank(MPI_COMM_WORLD, &procID);
  // Read network from file
  auto net = UNetwork<>::read("Infectious.edges");
  // Create the test data
  auto testData = NetworkManipulator<>::createTestDataRem(net
      , remRatio, seed, false);
  testData.lock();
  // Create an ADA predictor
  auto p = std::make_shared<UADAPredictor<>>(testData.
      getObsNet());
  // Initialize predictor
  p->init();
  // Train predictor
  p->learn();
  // Create object to store prediction results
  auto predResults = std::make_shared<PredResults<>>(testData
      , p);
  // Create a ROC object
  auto roc = std::make_shared<ROC<>>("ROC");
  // Set options
  roc->setParallel(true);
  roc->setDistributed(true);
  roc->setStrmEnabled(true);
  // Create object to store results
  PerfResults res;
  // Evaluate performance
  roc->eval(predResults, res);
  // Print results
  if (procID == 0) {
    std::cout << "#ROCAUC␣(streaming):␣" << res.at(roc->
        getName()) << std::endl;
  }
  // Finalize MPI
  MPI_Finalize();
  return 0;
}
```

This code is available in: `tutorials/code/parallel/distributed/rocstrmdist`

2. Compile your code:

   ```
   $ mpiCC rocstrmdist.cpp -o rocstrmdist -fopenmp -lLinkPred
   ```

   R Check Tutorial 1.2 if you face any compilation issues.

3. Run your code (here we are running the code on 4 nodes):

   ```
   $ mpirun -n 4 ./rocstrmdist
   ```

   The output of this program is as follows:

   ```
   #ROCAUC (streaming): 0.922352
   ```
